# Diseases, Injuries, and Risk Factors in Child and Adolescent Health, 1990 to 2017: Findings From the Global Burden of Diseases, Injuries, and Risk Factors 2017 Study

**DOI:** 10.1001/jamapediatrics.2019.0337

**Published:** 2019-04-29

**Authors:** GBD 2017 Child and Adolescent Health Collaborators, Robert C. Reiner, Helen Elizabeth Olsen, Chad Thomas Ikeda, Michelle M. Echko, Katherine E. Ballestreros, Helen Manguerra, Ira Martopullo, Anoushka Millear, Chloe Shields, Alison Smith, Bryan Strub, Molla Abebe, Zegeye Abebe, Beyene Meressa Adhena, Tara Ballav Adhikari, Mohammed Akibu, Rajaa M. Al-Raddadi, Nelson Alvis-Guzman, Carl Abelardo T. Antonio, Olatunde Aremu, Solomon Weldegebreal Asgedom, Netsanet Abera Asseffa, Leticia Avila-Burgos, Aleksandra Barac, Till W. Bärnighausen, Quique Bassat, Isabela M. Bensenor, Zulfiqar A. Bhutta, Ali Bijani, Nigus Bililign, Lucero Cahuana-Hurtado, Deborah Carvalho Malta, Jung-Chen Chang, Fiona J. Charlson, Samath Dhamminda Dharmaratne, David Teye Doku, Dumessa Edessa, Ziad El-Khatib, Holly E. Erskine, Alize J. Ferrari, Nancy Fullman, Rahul Gupta, Hamid Yimam Hassen, Simon I. Hay, Olayinka Stephen Ilesanmi, Kathryn H. Jacobsen, Amaha Kahsay, Amir Kasaeian, Tesfaye Dessale Kassa, Seifu Kebede, Yousef Saleh Khader, Ejaz Ahmad Khan, Mohammed Nuruzzaman Khan, Young-Ho Khang, Jagdish Khubchandani, Yohannes Kinfu, Sonali Kochhar, Yoshihiro Kokubo, Ai Koyanagi, Barthelemy Kuate Defo, Dharmesh Kumar Lal, Fekede Asefa Kumsa, Heidi J. Larson, Janni Leung, Abdullah A. Mamun, Suresh Mehata, Mulugeta Melku, Walter Mendoza, Haftay Berhane Mezgebe, Ted R. Miller, Nurilign Abebe Moges, Shafiu Mohammed, Ali H. Mokdad, Lorenzo Monasta, Subas Neupane, Huong Lan Thi Nguyen, Dina Nur Anggraini Ningrum, Yirga Legesse Nirayo, Vuong Minh Nong, Felix Akpojene Ogbo, Andrew T. Olagunju, Bolajoko Olubukunola Olusanya, Jacob Olusegun Olusanya, George C. Patton, David M. Pereira, Farshad Pourmalek, Mostafa Qorbani, Anwar Rafay, Rajesh Kumar Rai, Usha Ram, Chhabi Lal Ranabhat, Andre M. N. Renzaho, Mohammad Sadegh Rezai, Luca Ronfani, Gregory A. Roth, Saeid Safiri, Benn Sartorius, James G. Scott, Katya Anne Shackelford, Karen Sliwa, Chandrashekhar Sreeramareddy, Mu'awiyyah Bable Sufiyan, Abdullah Sulieman Terkawi, Roman Topor-Madry, Bach Xuan Tran, Kingsley Nnanna Ukwaja, Olalekan A. Uthman, Stein Emil Vollset, Kidu Gidey Weldegwergs, Andrea Werdecker, Harvey A. Whiteford, Tissa Wijeratne, Naohiro Yonemoto, Marcel Yotebieng, Liesl J. Zuhlke, Hmwe Hmwe Kyu, Mohsen Naghavi, Theo Vos, Christopher J. L. Murray, Nicholas J. Kassebaum

**Affiliations:** 1 Institute for Health Metrics and Evaluation, Seattle, Washington; 2 Department of Health Metrics Sciences, University of Washington, Seattle; 3 Department of Clinical Chemistry, University of Gondar, Gondar, Ethiopia; 4 Department of Human Nutrition, University of Gondar, Gondar, Ethiopia; 5 School of Public Health, Mekelle University, Tigray, Ethiopia; 6 Nepal Health Research Environment, Center for Social Science and Public Health Research Nepal, Lalitpur, Nepal; 7 Unit for Health Promotion Research, University of Southern Denmark, Esbjerg, Denmark; 8 Department of Midwifery, Debre Berhan University, Debre Berhan, Ethiopia; 9 Department of Family and Community Medicine, King Abdulaziz University, Jeddah, Saudi Arabia; 10 Research Group on Health Economics, University of Cartagena, Cartagena, Colombia; 11 Research Group on Hospital Management and Health Policies, University of the Coast, Barranquilla, Colombia; 12 Department of Health Policy and Administration, University of the Philippines Manila, Manila, Philippines; 13 Department of Applied Social Sciences, Hong Kong Polytechnic University, Hong Kong; 14 School of Health Sciences, Birmingham City University, Birmingham, United Kingdom; 15 School of Pharmacy, Mekelle University, Mekelle, Ethiopia; 16 School of Public Health, Wolaita Sodo University, Wolaita Dofo, Ethiopia; 17 Center for Health Systems Research, National Institute of Public Health, Cuernavaca, Mexico; 18 Clinic for Infectious and Tropical Diseases, Clinical Center of Serbia, Belgrade, Serbia; 19 Faculty of Medicine, University of Belgrade, Belgrade, Serbia; 20 Institute of Public Health, Heidelberg University, Heidelberg, Germany; 21 Harvard T.H. Chan School of Public Health, Harvard University, Boston, Massachusetts; 22 Barcelona Institute for Global Health, Barcelona, Spain; 23 Catalan Institution for Research and Advanced Studies, Manhiça Health Research Center, Manhiça, Mozambique; 24 Department of Internal Medicine, University of São Paulo, São Paulo, Brazil; 25 The Centre for Global Child Health, University of Toronto, Toronto, Canada; 26 Center of Excellence in Women and Child Health, Aga Khan University, Karachi, Pakistan; 27 Social Determinants of Health Research Center, Babol University of Medical Sciences, Babol, Iran; 28 Woldia University, Woldia, Ethiopia; 29 Department of Maternal and Child Nursing and Public Health, Federal University of Minas Gerais, Minas Gerais, Brazil; 30 College of Medicine, National Taiwan University, Taipei, Taiwan; 31 School of Public Health, The University of Queensland, Brisbane, Queensland, Australia; 32 Department of Global Health, University of Washington, Seattle; 33 Department of Community Medicine, University of Peradeniya, Peradeniya, Sri Lanka; 34 Department of Population and Health, University of Cape Coast, Cape Coast, Ghana; 35 Faculty of Social Sciences, Health Sciences, University of Tampere, Tampere, Finland; 36 School of Pharmacy, Haramaya University, Harar, Ethiopia; 37 Department of Public Health Sciences, Karolinska Institutet, Stockholm, Sweden; 38 Queensland Centre for Mental Health Research, Brisbane, Queensland, Australia; 39 West Virginia Bureau for Public Health, Charleston, West Virginia; 40 Department of Health Policy, Management, and Leadership, West Virginia University, Morgantown; 41 Public Health Department, Mizan-Tepi University, Teppi, Ethiopia; 42 Unit of Epidemiology and Social Medicine, University Hospital Antwerp, Antwerp, Belgium; 43 Department of Public Health and Community Medicine, University of Liberia, Monrovia, Liberia; 44 Department of Global and Community Health, George Mason University, Fairfax, Virginia; 45 Department of Nutrition and Dietetics, Mekelle University, Tigray, Ethiopia; 46 Hematology-Oncology and Stem Cell Transplantation Research Center, Tehran University of Medical Sciences, Tehran, Iran; 47 Hematologic Malignancies Research Center, Tehran University of Medical Sciences, Tehran, Iran; 48 Clinical Pharmacy Unit, Mekelle University, Mekelle, Ethiopia; 49 Midwifery Program, Salale University, Fiche, Ethiopia; 50 Department of Public Health and Community Medicine, Jordan University of Science and Technology, Ramtha, Jordan; 51 Department of Epidemiology and Biostatistics, Health Services Academy, Islamabad, Pakistan; 52 School of Medicine and Public Health, University of Newcastle, Newcastle, New South Wales, Australia; 53 Department of Population Sciences, Jatiya Kabi Kazi Nazrul Islam University, Mymensingh, Bangladesh; 54 Department of Health Policy and Management, Seoul National University, Seoul, South Korea; 55 Institute of Health Policy and Management, Seoul National University, Seoul, South Korea; 56 Department of Nutrition and Health Science, Ball State University, Muncie, Indiana; 57 Faculty of Health, University of Canberra, Canberra, Australian Capital Territory, Australia; 58 Murdoch Childrens Research Institute, Melbourne, Victoria, Australia; 59 Department of Public Health, Erasmus University Medical Center, Rotterdam, Netherlands; 60 Department of Preventive Cardiology, National Cerebral and Cardiovascular Center, Suita, Japan; 61 Research and Development Unit, San Juan de Dios Sanitary Park, Sant Boi de Llobregat, Barcelona; 62 Department of Demography, University of Montreal, Montreal, Quebec, Canada; 63 Department of Social and Preventive Medicine, University of Montreal, Montreal, Quebec, Canada; 64 Public Health Foundation of India, Gurugram, India; 65 Center for Midwifery, Child, and Family Health, University of Technology Sydney, Sydney, New South Wales, Australia; 66 School of Public Health, Haramaya University, Harar, Ethiopia; 67 Department of Infectious Disease Epidemiology, London School of Hygiene and Tropical Medicine, London, United Kingdom; 68 Center for Youth Substance Abuse Research, The University of Queensland, St Lucia, Queensland, Australia; 69 Institute for Social Science Research, The University of Queensland, Brisbane, Queensland, Australia; 70 Research, Monitoring, and Evaluation, Ipas Nepal, Kathmandu, Nepal; 71 Institute of Public Health, University of Gondar, Gondar, Ethiopia; 72 Peru Country Office, United Nations Population Fund, Lima, Peru; 73 Department of Pharmacy, Ethiopian Academy of Medical Science, Tigray, Ethiopia; 74 Pacific Institute for Research and Evaluation, Calverton, Maryland; 75 School of Public Health, Curtin University, Perth, Western Australia, Australia; 76 Department of Public Health, Debre Markos University, Debre Markos, Ethiopia; 77 Health Systems and Policy Research Unit, Ahmadu Bello University, Zaria, Nigeria; 78 Clinical Epidemiology and Public Health Research Unit, Burlo Garofolo Institute for Maternal and Child Health, Trieste, Italy; 79 Faculty of Health Sciences, University of Tampere, Tampere, Finland; 80 Institute for Global Health Innovations, Duy Tan University, Hanoi, Vietnam; 81 Department of Public Health Science, State University of Semarang, Kota Semarang, Indonesia; 82 Graduate Institute of Biomedical Informatics, Taipei Medical University, Taipei, Taiwan; 83 Translational Health Research Institute, Western Sydney University, Penrith, New South Wales, Australia; 84 Department of Psychiatry,University of Adelaide, Adelaide, South Australia, Australia; 85 Department of Psychiatry, University of Lagos, Lagos, Nigeria; 86 Centre for Healthy Start Initiative, Lagos, Nigeria; 87 Department of Paediatrics, University of Melbourne, Melbourne, Victoria, Australia; 88 Population Health Group, Murdoch Childrens Research Institute, Melbourne, Victoria, Australia; 89 Rede de Química e Tecnologia/Laboratório Asociado do Química Verde, University of Porto, Oporto, Portugal; 90 Cartagena University, Cartagena, Colombia; 91 School of Population and Public Health, University of British Columbia, Vancouver, British Columbia, Canada; 92 Noncommunicable Diseases Research Center, Alborz University of Medical Sciences, Karaj, Iran; 93 Department of Epidemiology and Biostatistics, Contech School of Public Health, Lahore, Pakistan; 94 Society for Health and Demographic Surveillance, Suri, India; 95 Department of Economics, University of Göttingen, Göttingen, Germany; 96 Department of Public Health and Mortality Studies, International Institute for Population Sciences, Mumbai, India; 97 Institute for Poverty Alleviation and International Development, Yonsei University, Wonju, South Korea; 98 School of Social Sciences and Psychology, Western Sydney University, Penrith, New South Wales, Australia; 99 Department of Pediatrics, Mazandaran University of Medical Sciences, Sari, Iran; 100 Division of Cardiology, University of Washington, Seattle; 101 Managerial Epidemiology Research Center, Maragheh University of Medical Sciences, Maragheh, Iran; 102 Department of Public Health Medicine, University of KwaZulu-Natal, Durban, South Africa; 103 Child and Youth Mental Health, Queensland Centre for Mental Health Research, Brisbane, Queensland, Australia; 104 Department of Medicine, University of Cape Town, Cape Town, Western Cape, South Africa; 105 Division of Community Medicine, International Medical University, Kuala Lumpur, Malaysia; 106 Department of Community Medicine, Ahmadu Bello University, Zaria, Nigeria; 107 Department of Anesthesiology, University of Virginia, Charlottesville; 108 Syrian Expatriate Medical Association, Charlottesville, Virginia; 109 Institute for Public Health, Jagiellonian University Medical College, Krakow, Poland; 110 Agency for Health Technology Assessment and Tariff System, Warszawa, Poland; 111 Department of Health Economics, Hanoi Medical University, Hanoi, Vietnam; 112 Department of Internal Medicine, Federal Teaching Hospital, Abakaliki, Nigeria; 113 Division of Health Sciences, University of Warwick, Coventry, United Kingdom; 114 Demographic Change and Ageing Research Area, Federal Institute for Population Research, Wiesbaden, Germany; 115 Independent Consultant, Staufenberg, Germany; 116 The University of Queensland, Brisbane, Queensland, Australia; 117 Department of Medicine, University of Melbourne, St Albans, Victoria, Australia; 118 Department of Psychology, La Trobe University, Melbourne, Victoria, Australia; 119 Department of Psychopharmacology, National Center of Neurology and Psychiatry, Tokyo, Japan; 120 College of Public Health, The Ohio State University, Columbus; 121 School of Public Health, University of Kinshasa, Kinshasa, Democratic Republic of the Congo; 122 Department of Paediatrics and Child Health, University of Cape Town, Cape Town, Western Cape, South Africa; 123 Department of Anesthesiology and Pain Medicine, University of Washington, Seattle

## Abstract

**Importance:**

Understanding causes and correlates of health loss among children and adolescents can identify areas of success, stagnation, and emerging threats and thereby facilitate effective improvement strategies.

**Objective:**

To estimate mortality and morbidity in children and adolescents from 1990 to 2017 by age and sex in 195 countries and territories.

**Design, Setting, and Participants:**

This study examined levels, trends, and spatiotemporal patterns of cause-specific mortality and nonfatal health outcomes using standardized approaches to data processing and statistical analysis. It also describes epidemiologic transitions by evaluating historical associations between disease indicators and the Socio-Demographic Index (SDI), a composite indicator of income, educational attainment, and fertility. Data collected from 1990 to 2017 on children and adolescents from birth through 19 years of age in 195 countries and territories were assessed. Data analysis occurred from January 2018 to August 2018.

**Exposures:**

Being under the age of 20 years between 1990 and 2017.

**Main Outcomes and Measures:**

Death and disability. All-cause and cause-specific deaths, disability-adjusted life years, years of life lost, and years of life lived with disability.

**Results:**

Child and adolescent deaths decreased 51.7% from 13.77 million (95% uncertainty interval [UI], 13.60-13.93 million) in 1990 to 6.64 million (95% UI, 6.44-6.87 million) in 2017, but in 2017, aggregate disability increased 4.7% to a total of 145 million (95% UI, 107-190 million) years lived with disability globally. Progress was uneven, and inequity increased, with low-SDI and low-middle–SDI locations experiencing 82.2% (95% UI, 81.6%-82.9%) of deaths, up from 70.9% (95% UI, 70.4%-71.4%) in 1990. The leading disaggregated causes of disability-adjusted life years in 2017 in the low-SDI quintile were neonatal disorders, lower respiratory infections, diarrhea, malaria, and congenital birth defects, whereas neonatal disorders, congenital birth defects, headache, dermatitis, and anxiety were highest-ranked in the high-SDI quintile.

**Conclusions and Relevance:**

Mortality reductions over this 27-year period mean that children are more likely than ever to reach their 20th birthdays. The concomitant expansion of nonfatal health loss and epidemiological transition in children and adolescents, especially in low-SDI and middle-SDI countries, has the potential to increase already overburdened health systems, will affect the human capital potential of societies, and may influence the trajectory of socioeconomic development. Continued monitoring of child and adolescent health loss is crucial to sustain the progress of the past 27 years.

## Key Points

**Question:**

How have the levels, trends, and leading causes of child and adolescent mortality and nonfatal health loss changed from 1990 to 2017?

**Findings:**

This study found that child and adolescent mortality decreased throughout the world from 1990 to 2017, but morbidity has increased as a proportion of total disease burden, because the major causes of nonfatal health loss during childhood and adolescence have not changed dramatically.

**Meaning:**

As the global health community continues to prioritize child and adolescent health during the Sustainable Development Goal era, careful attention should also be placed on examining and addressing nonfatal illness and disability across the development spectrum.

## Teaser

This analysis of the Global Disease Burden data estimates mortality and morbidity in children and adolescents from 1990 to 2017 by age and sex in 195 countries and territories worldwide.

## Introduction

Childhood and adolescence are vulnerable periods and a crucial window for adult health determination. While improvements in the mortality rate of children younger than 5 years (the population often called *under-5*) have been undeniably dramatic and positive,^1^ the full story of child and adolescent health is more nuanced and heterogeneous, with a notably broader range of characteristics than can be told with a single summary statistic.^2^ The effects of acute and chronic infectious diseases, nutrition, physical functioning, mental health, and intellectual development set the stage for both individual prosperity and the future human capital of all societies.^3^

Eleven of the 18 Sustainable Development Goals (SDGs) and 19 of the 53 health-associated SDG indicators are about child and adolescent health.^4,5^ These include ending all forms of malnutrition (SDG 2.2), reducing maternal mortality ratio to fewer than 70 per 100 000 live births (SDG 3.1), decreasing neonatal and under-5 mortality rate to fewer than 12 and 25 per 1000 live births, respectively (SDG 3.2), ensuring universal access to reproductive health care (SDG 3.7), and multiple objectives aimed at combating specific causes of health loss, such as malaria, tuberculosis, HIV, road traffic crashes, air pollution, substance abuse, and noncommunicable diseases (NCDs). However, many of the leading drivers of health loss among children and adolescents are notably absent from the SDG agenda.^6^

We have compiled this third annual global report to detail the levels, trends, causes, and correlates of health loss from birth through age 19 years. It reflects several notable improvements from Global Burden of Disease (GBD) 2017. First, we have generated a complete set of internally consistent demographics estimates, with uncertainty intervals (UIs), for age-specific fertility, population, and all-cause mortality.^7^ Second, 5 additional countries (Ethiopia, Iran, New Zealand, Norway, and Russia) were estimated at the subnational level. Third, in addition to adding many new sources of data, we have improved data-processing algorithms. Methods for redistributing deaths coded to nonspecific, implausible, or intermediate causes of death were updated to incorporate statistical uncertainty of cause reassignment. Clinical administrative data (hospital and claims) processing methods were updated to better account for hospital readmissions, multiple clinical visits, and ordering of discharge codes by age, sex, location, and time. Fourth, we have improved the epidemiological transition analysis through improved estimation of the SDI.

## Methods

Comprehensive descriptions of each analytic component of GBD 2017 are detailed elsewhere^1,7,8,9,10,11,12^ and compliant with the Guidelines for Accurate and Transparent Health Estimates Reporting.^13^ The GBD 2017 included 11 467 unique sources for cause of death estimation and 26 007 for estimation of nonfatal health loss. Data sources for each cause-level analysis are available online at the Global Health Data Exchange.^14^

The GBD 2017 used a geographic hierarchy of 7 superregions (high-income countries; Latin America and the Caribbean; North Africa and the Middle East; South Asia; sub-Saharan Africa [SSA]; Central Asia, Central Europe, and Eastern Europe; and Southeast Asia, East Asia, and Oceania) containing 21 regions and 195 countries and territories. Fifteen countries were estimated at the subnational level: Brazil, China, England, Ethiopia, India, Indonesia, Iran, Kenya, Mexico, New Zealand, Norway, the United States, Russia, Sweden, and South Africa. Estimates were produced for male individuals and female individuals separately in each of 23 standard age groups. We cover the first 7 of these age groups in this report: early neonatal (0-6 days’ postbirth age), late neonatal (7-27 days’ postbirth age), postneonatal (28-364 days’ postbirth age), 1 to 4 years, 5 to 9 years, 10 to 14 years, and 15 to 19 years.

Each of 359 diseases and injuries were arranged in a 4-level mutually exclusive and collectively exhaustive cause hierarchy; most were analyzed as causing both death and disability. The first level (level 1) of the cause list has 3 categories: communicable, maternal, neonatal, and nutritional conditions (CMNN); NCDs; and injuries. At level 2, there are 22 cause groups, and level 3 includes more disaggregated causes of burden (169 causes), as does level 4 (293 causes). The full GBD cause list, including corresponding *International Classification of Diseases, Ninth Revision *(*ICD-9*) and *Tenth Revision* (*ICD-10*) codes, is detailed in appendices to the GBD 2017 summary publications.^8,9^

All-cause mortality, cause-specific mortality, and years of life lost (YLLs) were estimated using standardized approaches of data identification, extraction, and processing to address data challenges such as incompleteness, variation in classification systems and coding practices, and inconsistent age group and sex reporting. Nonfatal estimates were generated using data from literature, hospital discharge and claims data systems, cross-sectional surveys, cohort studies, case notification systems, and disease-specific registries. Cause-specific years lived with disability (YLDs) were calculated by multiplying sequela-level prevalence with corresponding disability weights that were derived from population and internet surveys of more than 60 000 persons and adjusted for comorbidity through microsimulation.^15,16^ Disability-adjusted life years (DALYs) are the sum of YLDs and YLLs and used to measure the comprehensive health status of a population for a given location, sex, year, and age combination.

We sampled 1000 draws of the posterior distribution of quantity at the most granular level of each analysis, and 95% UIs represent the range of values between the ordinal 25th and 975th draws. Unlike confidence intervals, which only capture sampling error in a single statistical test, UIs also incorporate uncertainty from other associated steps. Aggregate estimates (eg, DALYs, combined age groups, geographical groups) were calculated by summing draw-level results assuming independence of each quantity. All draw-level results were summarized as mean values and 95% UIs.

We performed 3 secondary analyses for this report. First, we decomposed probability of death from birth to 19 years to illustrate how cause-specific trends are associated with overall survival improvements. Second, we explored the historical association between burden metrics and the SDI, a composite indicator of development based on per capita income, adult education, and total fertility rate for individuals younger than 25 years.^1^ Each GBD location’s SDI can vary by year, but for reporting purposes, each was assigned to an quintile based on its SDI in 2017. A map of SDI quintile assignments is shown in eFigure 1 in the Supplement and SDI values for each country by year are in eTable 1 in the Supplement. Observed values are the actual disease burden rates in each location-year, while expected values were determined by Gaussian process regression on the range of rates observed for each level of SDI. Third, given the intricate association between the health of women and their children, we examined the historical association between maternal mortality and DALY rates of children and adolescents.

We present a number of different formulations of results in the GBD 2017. Total number illustrates the cumulative size of burden, rates best compare between differently sized populations, and cause fraction (%) allows the comparison of relative importance of specific causes. We refer to those younger than 28 days as *neonates*, those younger than 1 year as *infants*, those younger than 10 years collectively as *children*, and those aged 10 to 19 years as *adolescents*. We focus on presenting aggregate results for the global level, SDI quintiles, and the GBD regions, either for birth to 19 years en bloc or for infants, children, and adolescents separately. Except when noted, results are for both sexes combined. More granular results are publicly available in an interactive online visualization tool called GBD Compare (https://vizhub.healthdata.org/gbd-compare/) and for download from the GBD Results Tool (http://ghdx.healthdata.org/gbd-results-tool).

## Results

### All-Cause Mortality and Decomposition of Causes of Death

Premature mortality is the dominant component of health loss in children and adolescents. The Table shows deaths by age group globally and by SDI quintile. eTable 2 in the Supplement shows the same for superregions, regions, countries, and territories. All-cause child and adolescent deaths decreased 51.7% from 13.77 million (95% UI, 13.60–13.93 million) in 1990 to 6.64 million (95% UI, 6.44-6.87 million) in 2017. More than half (60.1% [95% UI, 59.6%-60.5%]) occurred in infants younger than 1 year and, of those, 46.6% (95% UI, 46.0%-47.3%) occurred in the first week of life. The fastest decline was among children aged 1 to 4 years, in whom global deaths decreased by 61% from 3.62 million (95% UI, 3.52-3.72 million) in 1990 to 1.40 million (95% UI, 1.34-1.48 million) in 2017. Over the same period, mortality decreased 51% to 3.99 million (95% UI, 3.85-4.14 million) in infants younger than 1 year, by 52% to 0.41 million (95% UI, 0.40-0.42 million) in children aged 5 to 9 years, and by 27% to 0.84 million (95% UI, 0.82-0.85 million) in children aged 10 to 19 years. Improvements by age were similar across SDI quintiles.

**Table.** All-Cause Mortality in 1990, 2000, and 2017, With Mean Percentage Changes for Combined Sexes by Age

Metric	Individuals, No. (95% Uncertainty Intervals)										
	Early Neonatal^a^	Late Neonatal^b^	Postneonatal^c^	Aged 1 to 4 y	Aged 5 to 9 y	Aged 10 to 14 y	Aged 15 to 19 y	<1 y	<5 y	<20 y	Aged 10 to 19 y
Global											
Deaths, 1990, No.	3 037 044 (2 966 409- 3 104 943)	1 263 098 (1 220 453- 1 308 864)	3 854 632 (3 771 297- 3 942 560)	3 615 809 (3 519 278- 3 715 515)	853 941 (844 956- 862 845)	466 727 (462 844- 470 900)	674 033 (666 858- 681 883)	8 154 774 (8 024 357- 8 285 234)	11 770 583 (11 618 335- 11 926 603)	13 765 285 (13 601 506- 13 934 158)	1 140 760 (1 130 272- 1 151 953)
Deaths, 2000, No.	2 743 663 (2 683 053- 2 807 354)	918 145 (887 698- 954 872)	3 092 086 (3 030 247- 3 163 815)	2 926 972 (2 853 818- 3 007 898)	703 302 (696 132- 711 149)	483 735 (479 703- 487 837)	678 423 (670 973- 686 282)	6 753 895 (6 641 564- 6 883 034)	9 680 867 (9 545 127- 9 822 519)	11 546 328 (11 402 470- 11 696 143)	1 162 159 (1 151 054- 1 173 659)
Deaths, 2017, No.	1 859 529 (1 793 656- 1 933 838)	507 698 (489 055- 527 088)	1 621 135 (1 558 657- 1 693 323)	1 403 200 (1 340 221- 1 475 816)	412 113 (404 482- 420 010)	319 630 (314 713- 325 138)	516 509 (506 942- 527 177)	3 988 362 (3 847 837- 4 140 124)	5 391 562 (5 195 363- 5 612 906)	6 639 815 (6 437 215- 6 870 058)	836 139 (822 746- 851 197)
% Change, 1990-2017	−38.8 (−41.5 to −35.9)	−59.8 (−61.8 to −57.6)	−57.9 (−59.9 to −55.8)	−61.2 (−63.3 to −59.0)	−51.7 (−52.8 to −50.7)	−31.5 (−32.6 to −30.3)	−23.4 (−24.9 to −21.7)	−51.1 (−53.1 to −49.1)	−54.2 (−56.0 to −52.3)	−51.8 (−53.4 to −50.0)	−26.7 (−28.0 to −25.3)
% Change, 2000-2017	−32.2 (−35.1 to −29.1)	−44.7 (−47.5 to −41.7)	−47.6 (−49.9 to −45.0)	−52.1 (−54.5 to −49.4)	−41.4 (−42.7 to −40.1)	−33.9 (−35.0 to −32.9)	−23.9 (−25.2 to −22.5)	−41.0 (−43.2 to −38.3)	−44.3 (−46.4 to −41.9)	−42.5 (−44.4 to −40.3)	−28.1 (−29.2 to −26.9)
Low SDI											
Deaths, 1990, No.	1 048 756 (1 000 660- 1 099 967)	499 125 (462 152- 541 079)	1 407 561 (1 351 942- 1 473 057)	1 649 664 (1 571 877- 1 744 345)	294 379 (288 394- 300 299)	134 914 (132 405- 137 132)	173 480 (168 538- 178 248)	2 955 442 (2 859 649- 3 044 743)	4 605 107 (4 498 615- 4 697 610)	5 207 880 (5 094 452- 5 309 147)	308 395 (301 354- 315 308)
% of Total, 1990	34.5 (33.4-35.8)	39.5 (37.6-41.4)	36.5 (35.5-37.7)	45.6 (44.3-47.2)	34.5 (34.0-35.0)	28.9 (28.5-29.3)	25.7 (25.1-26.3)	36.2 (35.4-37.0)	39.1 (38.5-39.7)	37.8 (37.2-38.4)	27.0 (26.5-27.5)
Deaths, 2000, No.	1 047 243 (1 006 123- 1 091 383)	377 909 (352 083- 410 913)	1 312 429 (1 273 115- 1 357 139)	1 433 329 (1 378 745- 1 493 988)	269 317 (263 349- 274 995)	152 533 (150 081- 154 909)	192 073 (188 328- 195 751)	2 737 581 (2 658 104- 2 824 273)	4 170 910 (4 078 681- 4 267 250)	4 784 833 (4 684 232- 4 887 154)	344 605 (338 629- 350 377)
% of Total, 2000	38.2 (37.0-39.3)	41.2 (39.3-43.4)	42.4 (41.4-43.6)	49.0 (47.7-50.2)	38.3 (37.7-38.9)	31.5 (31.1-31.9)	28.3 (27.9-28.8)	40.5 (39.6-41.4)	43.1 (42.4-43.8)	41.4 (40.8-42.1)	29.6 (29.2-30.1)
Deaths, 2017, No.	799 558 (756 228- 844 647)	216 363 (204 706- 228 297)	764 529 (731 080- 800 527)	697 767 (666 056- 730 782)	170 474 (165 388- 175 721)	116 484 (113 419- 119 513)	164 477 (160 277- 168 964)	1 780 450 (1 700 258- 1 869 233)	2 478 217 (2 374 041- 2 592 363)	2 929 653 (2 817 698- 3 050 894)	280 962 (274 764- 287 360)
% of Total, 2017	43.0 (41.2-44.9)	42.6 (40.9-44.5)	47.2 (45.1-49.2)	49.7 (47.4-52.1)	41.4 (40.5-42.4)	36.4 (35.7-37.2)	31.8 (31.1-32.6)	44.6 (42.8-46.4)	46.0 (44.1-47.7)	44.1 (42.5-45.6)	33.6 (32.9-34.3)
% Change, 1990-2017	−23.8 (−29.0 to −17.6)	−56.6 (−60.4 to −52.0)	−45.7 (−49.1 to −42.2)	−57.7 (−60.8 to −54.4)	−42.1 (−44.1 to −39.9)	−13.7 (−16.3 to −10.7)	−5.2 (−8.6 to −1.6)	−39.8 (−43.0 to −36.1)	−46.2 (−48.6 to −43.4)	−43.8 (−46.1 to −41.2)	−8.9 (−11.8 to −6.0)
% Change, 2000-2017	−23.6 (−28.8 to −17.7)	−42.8 (−48.2 to −37.3)	−41.8 (−44.8 to −38.6)	−51.3 (−54.2 to −48.2)	−36.7 (−39.2 to −34.3)	−23.6 (−26.0 to −21.3)	−14.4 (−16.9 to −12.0)	−35.0 (−38.5 to −31.2)	−40.6 (−43.4 to −37.4)	−38.8 (−41.5 to −35.7)	−18.5 (−20.6 to −16.4)
Low-middle SDI											
Deaths, 1990, No.	1 050 591 (1 005 227- 1 101 086)	420 295 (402 095- 440 764)	1 231 685 (1 187 111- 1 281 266)	1 241 777 (1 188 516- 1 291 581)	252 941 (248 961- 256 718)	135 510 (133 125- 137 988)	191 154 (186 315- 196 539)	2 702 571 (2 635 701- 2 772 458)	3 944 348 (3 854 061- 4 037 247)	4 523 953 (4 433 296- 4 620 989)	326 664 (319 584- 334 446)
% of Total, 1990	34.6 (33.4-35.9)	33.3 (31.9-34.8)	31.9 (31.0-33.0)	34.3 (33.0-35.6)	29.6 (29.2-30.0)	29.0 (28.6-29.5)	28.4 (27.8-29.0)	33.1 (32.5-33.9)	33.5 (33.0-34.1)	32.9 (32.4-33.5)	28.6 (28.1-29.2)
Deaths, 2000, No.	1 027 894 (985 060- 1 072 304)	337 892 (322 334- 353 887)	1 103 094 (1 057 458- 1 152 018)	1 111 181 (1 061 360- 1 162 163)	226 250 (222 741- 229 883)	145 940 (143 053- 148 953)	207 612 (201 623- 214 285)	2 468 880 (2 386 134- 2 550 868)	3 580 061 (3 482 534- 3 681 176)	4 159 863 (4 056 664- 4 267 343)	353 552 (344 879- 363 321)
% of total, 2000	37.5 (36.2-38.7)	36.8 (35.0-38.4)	35.7 (34.6-36.8)	38.0 (36.6-39.3)	32.2 (31.7-32.6)	30.2 (29.7-30.6)	30.6 (30.0-31.3)	36.5 (35.6-37.5)	37.0 (36.2-37.7)	36.0 (35.4-36.7)	30.4 (29.9-31.0)
Deaths, 2017, No.	741 928 (687 548- 799 669)	201 579 (187 087- 217 116)	598 744 (546 451- 659 900)	556 133 (501 195- 617 348)	143 064 (137 522- 148 843)	109 895 (106 336- 114 277)	171 571 (163 916- 180 817)	1 542 251 (1 428 045- 1 667 161)	2 098 384 (1 931 802- 2 278 025)	2 522 915 (2 349 624- 2 716 084)	281 467 (270 507- 294 587)
% of Total, 2017	39.9 (37.9-42.0)	39.7 (37.7-41.8)	36.9 (34.6-39.4)	39.6 (36.9-42.2)	34.7 (33.7-35.7)	34.4 (33.6-35.3)	33.2 (32.1-34.4)	38.7 (36.7-40.7)	38.9 (36.9-41.0)	38.0 (36.2-39.8)	33.7 (32.7-34.7)
% Change, 1990-2017	−29.4 (−35.7 to −22.9)	−52.0 (−56.3 to −47.8)	−51.4 (−56.1 to −45.6)	−55.2 (−59.9 to −50.0)	−43.4 (−45.8 to −41.0)	−18.9 (−21.6 to −15.7)	−10.2 (−14.5 to −5.3)	−42.9 (−47.3 to −38.1)	−46.8 (−50.9 to −42.1)	−44.2 (−48.0 to −39.8)	−13.8 (−17.3 to −9.6)
% Change, 2000-2017	−27.8 (−34.1 to −21.1	−40.3 (−45.4 to −34.9)	−45.7 (−51.3 to −39.6)	−50.0 (−55.1 to −44.0)	−36.8 (−39.5 to −34.0)	−24.7 (−27.0 to −22.1)	−17.4 (−20.8 to −13.5)	−37.5 (−42.4 to −32.3)	−41.4 (−46.1 to −36.1)	−39.4 (−43.7 to −34.5)	−20.4 (−23.3 to −17.1)
Middle SDI											
Deaths, 1990, No.	628 643 (610 187- 646 947)	238 799 (230 555- 247 253)	868 238 (837 395- 901 662)	537 299 (517 549- 556 637)	202 488 (198 125- 206 891)	121 109 (119 519- 122 793)	173 289 (170 735- 175 772)	1 735 679 (1 683 546- 1 790 834)	2 272 978 (2 208 184- 2 340 940)	2 769 865 (2 698 798- 2 842 310)	294 398 (290 461- 298 249)
% of Total, 1990	20.7 (20.0-21.4)	18.9 (18.1-19.8)	22.5 (21.8-23.3)	14.9 (14.2-15.4)	23.7 (23.3-24.1)	25.9 (25.6-26.3)	25.7 (25.3-26.1)	21.3 (20.7-21.9)	19.3 (18.8-19.8)	20.1 (19.6-20.6)	25.8 (25.4-26.2)
Deaths, 2000, No.	462 225 (451 844- 472 926)	139 884 (135 990- 143 999)	493 464 (480 843- 506 778)	278 288 (269 324- 287 279)	141 272 (138 903- 143 669)	117 620 (116 463- 118 791)	157 626 (155 839- 159 372)	1 095 573 (1 073 483- 1 120 170)	1 373 862 (1 345 819- 1 401 556)	1 790 380 (1 759 629- 1 820 258)	275 246 (272 638- 277 738)
% of Total, 2000	16.9 (16.4-17.3)	15.2 (14.6-15.9)	16.0 (15.5-16.4)	9.5 (9.1-9.9)	20.1 (19.7-20.4)	24.3 (24.0-24.6)	23.2 (22.9-23.6)	16.2 (15.8-16.6)	14.2 (13.9-14.5)	15.5 (15.2-15.8)	23.7 (23.4-23.9)
Deaths, 2017, No.	222 226 (214 317- 230 498)	60 514 (58 427- 62 842)	183 100 (174 148- 193 196)	104 767 (100 420- 109 319)	68 791 (67 224- 70 555)	64 108 (63 168- 65 105)	116 098 (113 774- 118 242)	465 841 (449 615- 483 118)	570 608 (551 051- 591 996)	819 604 (797 304- 842 529)	180 206 (177 373- 182 959)
% of Total, 2017	11.9 (11.4-12.6)	11.9 (11.3-12.5)	11.3 (10.7-12.1)	7.5 (7.0-7.9)	16.7 (16.2-17.1)	20.1 (19.6-20.5)	22.5 (21.9-23.0)	11.7 (11.2-12.2)	10.6 (10.1-11.1)	12.3 (11.9-12.8)	21.6 (21.1-22.0)
% Change, 1990-2017	−64.7 (−66.2 to −62.9)	−74.7 (−75.8 to −73.3)	−78.9 (−80.3 to −77.4)	−80.5 (−81.5 to −79.3)	−66.0 (−67.2 to −64.7)	−47.1 (−48.2 to −45.9)	−33.0 (−34.6 to −31.4)	−73.2 (−74.4 to −71.8)	−74.9 (−76.0 to −73.7)	−70.4 (−71.5 to −69.2)	−38.8 (−40.1 to −37.5)
% Change, 2000-2017	−51.9 (−53.9 to −49.8)	−56.7 (−58.6 to −54.6)	−62.9 (−64.9 to −60.5)	−62.4 (−64.2 to −60.4)	−51.3 (−52.7 to −49.8)	−45.5 (−46.5 to −44.5	−26.4 (−27.9 to −24.7)	−57.5 (−59.0 to −55.6)	−58.5 (−60.0 to −56.7)	−54.2 (−55.6 to −52.7)	−34.5 (−35.7 to −33.3)
High-middle SDI											
Deaths, 1990, No.	251 380 (243 625- 259 229)	88 124 (85 426- 91 122)	294 385 (285 064- 304 510)	156 447 (149 526- 163 923)	85 156 (83 615- 86 820)	57 768 (57 072- 58 454)	86 847 (85 614- 88 149)	633 890 (616 360- 653 955)	790 337 (768 924- 813 197)	1 020 107 (996 550- 1 045 005)	144 615 (142 872- 146 484)
% of Total, 1990	8.3 (8.0-8.6)	7.0 (6.7-7.3)	7.6 (7.3-7.9)	4.3 (4.1-4.5)	10.0 (9.8-10.2)	12.4 (12.2-12.5)	12.9 (12.7-13.1)	7.8 (7.5-8.1)	6.7 (6.5-6.9)	7.4 (7.2-7.6)	12.7 (12.5-12.9)
Deaths, 2000, No.	168 748 (163 067- 174 696)	50 722 (48 938- 52 476)	152 970 (147 699- 158 189)	85 695 (81 187- 90 292)	54 832 (53 762- 55 920)	54 631 (53 990- 55 338)	84 218 (83 489- 85 016)	372 439 (361 974- 383 119)	458 135 (445 459- 471 978)	651 816 (637 814- 666 613)	138 849 (137 574- 140 224)
% of Total, 2000	6.2 (5.9-6.4)	5.5 (5.2-5.8)	5.0 (4.8-5.1)	2.9 (2.8-3.1)	7.8 (7.6-8.0)	11.3 (11.1-11.4)	12.4 (12.2-12.6)	5.5 (5.3-5.7)	4.7 (4.6-4.9)	5.7 (5.5-5.8)	11.9 (11.8-12.1)
Deaths, 2017, No.	69 837 (66 696- 73 005)	21 430 (20 560- 22 308)	56 477 (53 589- 59 480)	33 866 (32 037- 35 979)	23 303 (22 679- 23 920)	21 805 (21 438- 22 160)	42 409 (41 619- 43 208)	147 743 (141 663- 153 908)	181 609 (174 033- 188 764)	269 126 (260 627- 277 283)	64 214 (63 108- 65 265)
% of Total, 2017	3.8 (3.5-4.0)	4.2 (4.0-4.5)	3.5 (3.3-3.7)	2.4 (2.2-2.6)	5.7 (5.5-5.8)	6.8 (6.7-7.0)	8.2 (8.0-8.4)	3.7 (3.5-3.9)	3.4 (3.2-3.5)	4.0 (3.9-4.2)	7.7 (7.5-7.8)
% Change, 1990-2017	−72.2 (−73.7 to −70.7)	−75.7 (−76.9 to −74.4)	−80.8 (−82.0 to −79.6)	−78.3 (−79.8 to −76.8)	−72.6 (−73.4 to −71.9)	−62.2 (−63.0 to −61.5)	−51.2 (−52.3 to −50.0)	−76.7 (−77.8 to −75.5)	−77.0 (−78.1 to −76.0)	−73.6 (−74.6 to −72.7)	−55.6 (−56.5 to −54.7)
% Change, 2000-2017	−58.6 (−60.7 to −56.2)	−57.8 (−59.9 to −55.5)	−63.1 (−65.3 to −60.6	−60.5 (−63.4 to −57.4)	−57.5 (−58.8 to −56.2)	−60.1 (−60.9 to −59.3)	−49.6 (−50.8 to −48.6)	−60.3 (−62.2 to −58.4)	−60.4 (−62.2 to −58.5)	−58.7 (−60.2 to −57.2)	−53.8 (−54.7 to −52.8)
High SDI											
Deaths, 1990, No.	51 138 (50 076-52 156)	14 066 (13 636-14 520)	40 672 (39 568-41 787)	23 862 (23 087-24 712)	16 274 (15 946-16 603)	15 870 (15 745-15 997)	47 148 (47 037-47 267)	105 875 (104 730- 107 028)	129 737 (128 508- 131 047)	209 029 (207 361- 210 885)	63 018 (62 791- 63 243)
% of Total, 1990	1.7 (1.6-1.7)	1.1 (1.1-1.2)	1.1 (1.0-1.1)	0.7 (0.6-0.7)	1.9 (1.9-1.9)	3.4 (3.4-3.4)	7.0 (6.9-7.1)	1.3 (1.3-1.3)	1.1 (1.1-1.1)	1.5 (1.5-1.5)	5.5 (5.5-5.6)
Deaths, 2000, No.	33 111 (32 535-33 688)	10 224 (9938-10 536)	23 583 (22 957-24 177)	14 817 (14 389-15 295)	9693 (9641-9746)	11 387 (11 347-11 430)	35 359 (35 294-35 431)	66 918 (66 183-67 627)	81 735 (81 099-82 476)	138 175 (137 405-138 996)	46 747 (46 647-46 849)
% of Total, 2000	1.2 (1.2-1.2)	1.1 (1.1-1.2)	0.8 (0.7-0.8)	0.5 (0.5-0.5)	1.4 (1.4-1.4)	2.4 (2.3-2.4)	5.2 (5.2-5.3)	1.0 (1.0-1.0)	0.8 (0.8-0.9)	1.2 (1.2-1.2)	4.0 (4.0-4.1)
Deaths, 2017, No.	24 050 (23 335-24 802)	7181 (6937-7450)	16 108 (15 624-16 662)	9390 (9153-9642)	5757 (5681-5836)	6702 (6640-6765)	21 083 (20 783-21 392)	47 340 (45 953-48 790)	56 729 (55 145-58 397)	90 272 (88 442-92 159)	27 786 (27 447-28 137)
% of Total, 2017	1.3 (1.2-1.4)	1.4 (1.3-1.5)	1.0 (0.9-1.1)	0.7 (0.6-0.7)	1.4 (1.4-1.4)	2.1 (2.1-2.1)	4.1 (4.0-4.2)	1.2 (1.1-1.2)	1.1 (1.0-1.1)	1.4 (1.3-1.4)	3.3 (3.2-3.4)
% Change, 1990-2017	−53.0 (−54.6 to −51.1)	−48.9 (−51.3 to −46.3)	−60.4 (−62.1 to −58.8)	−60.6 (−62.1 to −58.9)	−64.6 (−65.4 to −63.8)	−57.8 (−58.3 to −57.3)	−55.3 (−55.9 to −54.6)	−55.3 (−56.6 to −53.9)	−56.3 (−57.5 to −54.9)	−56.8 (−57.7 to −55.9)	−55.9 (−56.5 to −55.3)
% Change, 2000-2017	−27.4 (−29.9 to −24.9	−29.8 (−33.1 to −26.3)	−31.7 (−34.3 to −28.8)	−36.6 (−39.1 to −34.0)	−40.6 (−41.5 to −39.8)	−41.1 (−41.8 to −40.5)	−40.4 (−41.2 to −39.5)	−29.3 (−31.5 to −27.0)	−30.6 (−32.6 to −28.5)	−34.7 (−36.0 to −33.3)	−40.6 (−41.3 to −39.8)

Abbreviation: SDI, Socio-Demographic Index.

^a^ Early neonatal includes individuals aged 0 to 6 days.

^b^ Late neonatal includes individuals aged 7 to 27 days.

^c^ Postneonatal includes individuals aged 28 to 364 days.

**Figure 1.**
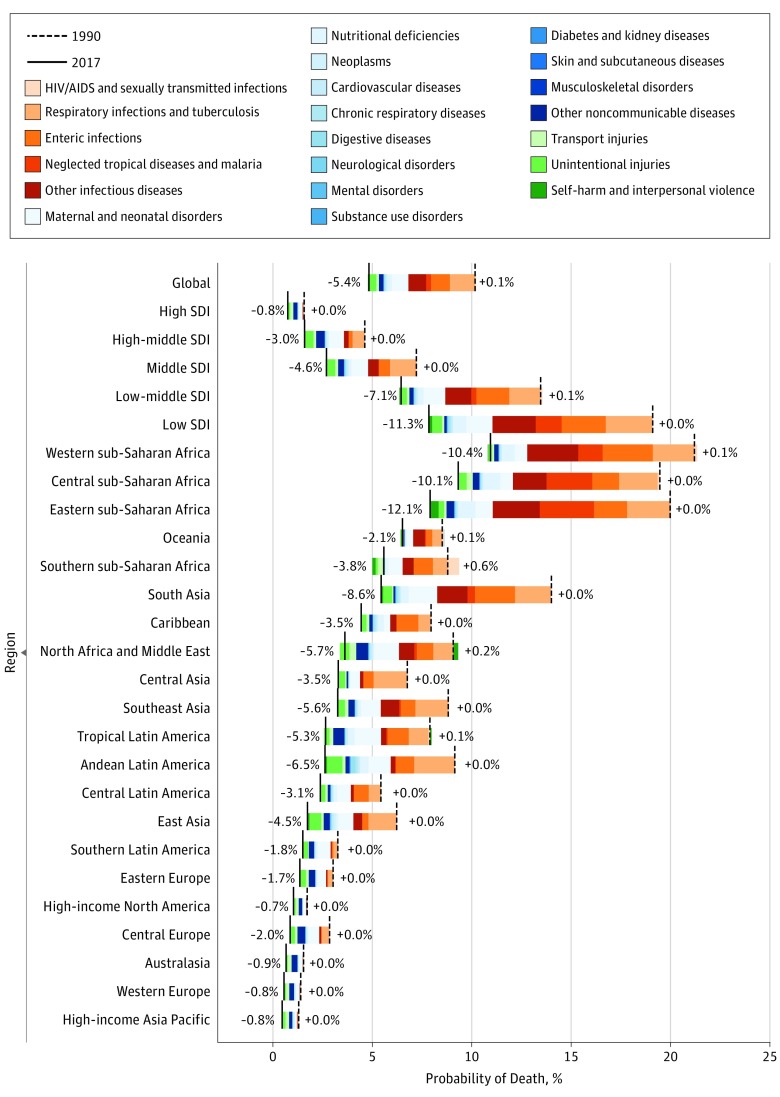
Decomposition of the Probability of Death Globally and in the Socio-Demographic Index (SDI) Quintiles and Global Burden of Disease Regions for Individuals Younger Than 20 Years of Both Sexes, From 1990 to 2017 Probability of death is plotted globally by SDI quintile and Global Disease Burden study region for 1990 (dashed vertical line) and 2017 (solid vertical line). The relative contribution of that change owing to different causes is indicated by different color bars. The SDI quintiles are sorted from lowest probability of death in 2017 to highest, and GBD regions are sorted from highest probability of death in 2017 to lowest.

Decomposition of changes in probability of death between birth and age 20 years from 1990 to 2017 revealed different level 2 cause-level drivers across GBD regions (Figure 1; country results are in eFigure 2 in the Supplement). Decreases in deaths owing to infectious diseases, neonatal disorders, and unintentional injuries drove improvements at the global level and for many less-developed regions (eg, CMNN deaths were virtually absent in high-SDI regions). In western, central, and eastern SSA, the probability of surviving to adulthood increased from 1990 to 2017 (western SSA: 1990, 78.8%; 2017, 89.1%; central SSA: 1990, 80.5%; 2017, 90.7%; eastern SSA: 1990, 80.0%; 2017, 92.1%), primarily as a result of decreased mortality owing to respiratory infections (percentage of decreased mortality owing to this change: western SSA, 20.4%; central SSA, 19.1%; eastern SSA, 17.9%), enteric infections (western SSA, 24.6%; central SSA, 13.3%; eastern SSA, 13.7%), neglected tropical diseases and malaria (western SSA, 11.9%; central SSA, 22.6%; eastern SSA, 22.6%), other infectious diseases (western SSA, 25.0%; central SSA, 16.7%; eastern SSA, 19.6%), and nutritional deficiencies (western SSA, 6.4%; central SSA, 8.5%; eastern SSA, 7.1%). The total decrease in mortality from these causes was 88.4% in western SSA, 80.3% in central SSA, and 81.0% in eastern SSA.

Decreased mortality from other NCDs (primarily congenital birth defects and hemoglobinopathies) and neonatal disorders contributed the most to survival improvements in most of high-middle–SDI and high-SDI regions (decrease in death rate, 1990-2017, for congenital birth defects: high-SDI countries, 52.6% [95% UI, 41.6%-55.8%]; high-middle–SDI countries, 59.4% [95% UI, 53.1%-64.8%]; hemoglobinopathies: high-SDI countries, 54.1% [95% UI, 38.8%-60.0%]; high-middle–SDI countries, 61.3% [95% UI, 49.1%-68.7%]; neonatal disorders: high-SDI countries, 45.5% [95% UI, 42.3%-50.4%]; high-middle–SDI countries, 65.0% [95% UI, 61.3%-68.4%]). Exceptions to broad survival improvements included a 0.6% increased probability of death owing to HIV/AIDS and sexually transmitted infections (1990, 0.6%; 2017, 1.2%; death rate per 100 000 population) in individuals younger than 20 years from HIV/AIDS and STI: 1990, 33.6 [95% UI, 22.4-46.7]; 2017, 57.2 [95% UI, 49.5-65.9]) in Southern sub-Saharan Africa.

There were a total of 50 countries where the probability of death by self-harm and interpersonal violence increased between 1990 and 2017. Nine of them had increase of more than 0.1% in the overall probability of death owing to self-harm and interpersonal violence between birth and age 20 years: Syria (1990, 0.04%; 2017, 3.45%), Iraq (1990, 0.26%; 2017, 1.22%), Yemen (1990, 0.07%; 2017, 0.89%), Central African Republic (1990, 0.1%; 2017, 0.64%), South Sudan (1990, 0.35%; 2017, 0.73%), Libya (1990, 0.05%; 2017, 0.41%), Venezuela (1990, 0.19%; 2017, 0.54%), Mexico (1990, 0.17%; 2017, 0.29%), and Lesotho (1990, 0.25%; 2017, 0.35%).

### Temporal and Sociodemographic Trends in DALYs

**Figure 2.**
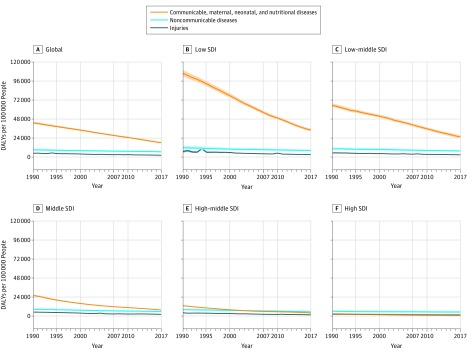
Trends of Disability-Adjusted Life Years From 1990 to 2017 for Global and Socio-Demographic Index (SDI) Quintiles for Children and Adolescents Younger Than 20 Years Temporal trends in disability adjusted life-years (DALYs) are plotted for children and adolescents younger than 20 years. Global trends are plotted in the top left subpanel, and the corresponding trends for each SDI quintile are plotted in the next 5 subpanels. Shaded areas show 95% uncertainty intervals. Communicable, maternal, neonatal, and nutritional disorders are shown in orange, noncommunicable disease causes in blue, and injuries in gray.

Total DALYs in children and adolescents decreased by 46% from 1.31 billion (95% UI, 1.27-1.36 billion) in 1990 to 709 million (95% UI, 665-757 million) in 2017 (Figure 2). Absolute DALYs decreases were greatest in the low-SDI quintile (1990, 476 million [95% UI, 464-490 million]; 2017, 292 million [95% UI, 277-309 million]), but slower relative gains led to increased inequity as the proportion of global DALYs rose from 36% in 1990 to 41% in 2017. The CMNN causes were by far the largest contributor to DALYs in the low-SDI quintile (75.3% of total DALYs [95% UI, 73.5%-77.0%]), low-middle–SDI quintile (71.6% of total DALYs [95% UI, 69.6%-73.6%]), and middle-SDI quintile (49.8% of total DALYs [95% UI, 47.6%-52.0%]) but also posted the biggest improvements, decreasing globally by 52.3% from 992 million (95% UI, 962 million-1.025 billion) to 473 million (95% UI, 452-498 million) in 2017, including a 72.3% reduction in the middle-SDI quintile from 179 million (95% UI, 172-187 million) to 49.5 million (95% UI, 46.5-52.9 million) in 2017. The NCD DALY rates were relatively similar across SDI quintiles and had the slowest global decrease (17.1%), from 210 million (95% UI, 186-238 million) to 174 million (95% UI, 151-201 million) in 2017. The NCDs were the bulk of DALYs in high-SDI locations (63.8% [95% UI, 60.1%-67.3%]), while the high-middle–SDI quintile transitioned in 2000 to also having the largest proportion of child and adolescent DALYs owing to NCDs (1990, 32.0% [95% UI, 29.9%-34.3%]; 2000, 39.2% [95% UI, 36.7%-41.8%]; 2017, 47.7% [95% UI, 44.7%-50.8%]). Global DALYs owing to injuries decreased by 46.0% from 113 million (95% UI, 101-122 million) to 61.0 million (95% UI, 58.0-64.1 million) in 2017 but also fluctuated widely by country and region, mainly because of war and (notably) the 1994 Rwandan genocide, 2004 tsunami in Southeast Asia, the 2010 earthquake in Haiti, and other recent conflicts and disasters.

Absolute difference in DALY rates between the lowest-SDI and highest-SDI locations were more apparent in children younger than 5 years than other age groups (eFigure 3 and eFigure 4 in the Supplement). The CMNN category was associated with the most DALYs in all SDI quintiles for children younger than 1 year (DALYs per 100 000 population, by SDI: low, 374 554 [95% UI, 357 5343-392 461]; low-middle, 301 824 [95% UI, 278 689-326 511]; middle, 96 368 [95% UI, 92 615-100 368]; high-middle, 48 272 [95% UI, 46 183-50 491]; high, 21 319 [95% UI, 19 936-22 237]). The NCDs increased in importance with age, causing the most DALYs in the middle-SDI quintile (3355 DALYs [95% UI, 2657-4169] per 100 000), high-middle–SDI quintile (3266 [95% UI, 2570-4102] per 100 000), and high-SDI quintile (2938 [95% UI, 2195-3800] per 100 000) in children aged 5 to 9 years, and in all but the low-SDI quintile in children aged 10 to 14 years (per SDI: low-middle quintile, 5116.1 [95% UI, 4061.4-6371.4]; middle quintile, 4503.3 [95% UI, 3519.9-5643.3]; high-middle quintile, 4446.0 [95% UI, 3453.1-5604.5]; high quintile, 4662.8 [95% UI, 3478.0-6089.5]) and 15 to 19 years (per SDI: low-middle quintile, 7266.1 [95% UI, 5839.7-8903.7]; middle quintile, 6370.9 [95% UI, 5039.9-7939.6]; high-middle quintile, 6420.5 [95% UI, 5018.1-8095.4]; high quintile, 7774.3 [95% UI, 5919.3-9924.6]). In the low-SDI quintile, NCDs and CMNN DALYs were close in value (NCDs, children aged 10-14 years, 4990.0 [95% UI, 3908.5-6217.1] DALYs per 100 000; those aged 15-19 years, 7051.07 [95% UI, 5632.0-8659.6] DALYs per 100 000; CMNNs: children aged 10-14 years, 5843.5 [95% UI, 5016.5-6828.1] DALYs per 100 000; those aged 15-19 years, 5943.6 [95% UI, 5284.8-6789.5] DALYs per 100 000). Despite decreasing rates in 179 of 195 countries between 1990 and 2017, injuries caused an increasing proportion of overall DALYs with advancing age of children and adolescents (global rates of injury DALYs per age group: 10-14 years, 1466.7 [95% UI, 1371.0-1570.3] DALYs per 100 000; 15-19 years, 2979.0 [95% UI, 2842.3-3127.0] DALYs per 100 000), to the point that total DALYs owing to injuries were only marginally lower than CMNN DALYs in those aged 10 to 19 years. The sociodemographic gradient for injuries was not as stark as for CMNN until the high-SDI quintile was reached, however. In this quintile, there was a dramatic decline in injury DALYs compared with other SDI settings from 1990 to 2017 (low, −58% [95% UI, −64% to −47%]; low-middle, −48%, [95% UI, −53% to −39%]; middle, −57% [95% UI, −59% to −57%]; high-middle, −58% [95% UI, −60% to −56%]; high, −52% [95% UI, −54% to −50%]), but this category rose in relative importance to cause nearly 20% of total DALYs by 2017 in all SDI settings (low, 6.5% [95% UI, 6.0%-7.1%]; low-middle, 7.5% [95% UI, 7.0%-7.9%]; middle, 14.0% [95% UI, 13.0%-15.0%]; high-middle, 15.9% [95% UI, 14.7%-17.2%]; high, 13.5% [95% UI, 12.2%-14.9%]).

Historical patterns in SDI and DALY rates illustrate the epidemiologic transition by age (eFigure 5 in the Supplement). The slope of the SDI gradient decreased with increasing age for all causes. For CMNN causes, DALY rates tracked closely with SDI differences in all regions, except Southern sub-Saharan Africa and the Caribbean. The association between SDI groups and NCDs was similar in all children younger than 10 years (range across all SDI levels: children younger than 1 year, 30 413.3-63 232.9; aged 1-4 years, 3504.3-12 070.2; aged 5-9 years, 3273.1-5017.1), but flattened somewhat in adolescents (range across all SDI levels: aged 10-14 years, 4757.6-5685.7; aged 15-19 years, 6835.6-8537.9). In this group, the DALY rate was also higher. In several regions, as evidenced by steep temporal slope of regional plots, especially in Andean Latin America and East Asia, improvements in NCD DALYs outpaced what would have been expected on the basis of SDI improvements alone (O:E DALY rates: Andean Latin America, 1990-2000, −24.1; 2000-2017, −17.0; East Asia, 1990-2000, 12.2; 2000-2017, −9.1). A trend toward increasing DALY rates owing to injury in children aged 5 to 9 years and 10 to 19 years was seen at the lower end of the development spectrum. Eastern Europe and Southern sub-Saharan Africa had consistently higher injury DALY rates than expected by SDI grouping.

### Identifying Exemplars

**Figure 3.**
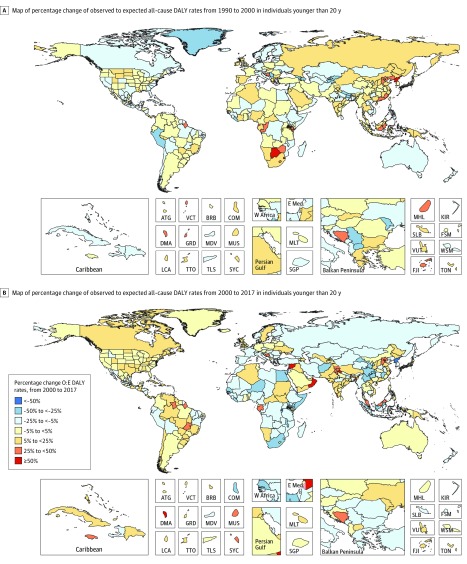
Percentage Change From 1990 to 2017 in Observed-to-Expected (O:E) Disability-Adjusted Life-Years Ratio in Children and Adolescents of Both Sexes, Aged 0 to 19 Years Percentage changes in observed-to-expected disability-adjusted life-years (DALYs) are plotted for 1990 to 2017 for all children and adolescents younger than 20 years. Both sexes are combined. Subnational differentiation occurs within each country’s Global Burden of Disease models at the subnational level. Inset plots provided for detailed inspection of small or clustered regions. ATG indicates Antigua and Barbuda; BRB, Barbados; COM, Comoros; DMA, Dominica; E Med, Eastern Mediterranean; FJI, Fiji; FSM, Federated States of Micronesia; GRD, Grenada; KIR, Kiribati; LCA, St Lucia; MDV, Maldives; MHL, Marshall Islands; MLT, Malta; MUS, Mauritius; SGP, Singapore; SLB, Solomon Islands; SYC, Seychelles; TLS, Timor-Leste; TON, Tonga; TTO, Trinidad and Tobago; W Africa, West Africa; WSM, Samoa; VCT, St Vincent and the Grenadines; and VUT, Vanuatu.

Changes in the ratio of observed-to-expected (O:E) DALY rates from 1990 to 2000 and 2000 to 2017 are mapped in Figure 3 (and by age group in eFigure 6 in the Supplement). Before 2000, 117 countries improved more than expected on the basis of SDI changes, while 116 countries did so after 2000. Seventy-six countries had faster than expected improvement in both periods; the O:E ratio of all-cause DALY rates were most notable in Liberia (1990-2000, −35.7; 2000-2017, −30.0), Niger (1990-2000, −14.7; 2000-2017, −43.3), Kyrgyzstan (1990-2000, −36.1; 2000-2017, −21.0), Peru (1990-2000, −32.8; 2000-2017, −19.0), and Georgia (1990-2000, −37.6; 2000-2017, −12.0). On the other end of the performance spectrum, 38 countries performed worse than expected in both periods. From 1990 to 2000, North Korea had O:E DALY rates increase 225% more than expected. However, many countries that underperformed expectations before 2000 reversed course post-2000. Notable exceptions include Dominica (all-cause O:E DALY rates: 1990-2000, 31.0; 2000-2017, 99.2), Equatorial Guinea (all-cause O:E DALY rates: 1990-2000, 41.8; 2000-2017, 23.1), Bosnia and Herzegovina (all-cause O:E DALY rates: 1990-2000, 29.8; 2000-2017, 34.2), and Lesotho (all-cause O:E DALY rates: 1990-2000, 46.9; 2000-2017, 14.9).

Syria was also an outlier in how much worse than expected observed DALY rates were in children and adolescents (O:E ratios in 1990: all-cause, 0.48; injuries, 0.49; O:E ratios in 2017: all-cause, 1.09; injuries, 4.73). This was primarily owing to increased injury rates (for all individuals younger than 20 years: 1990, 189 305 [95 UI, 154 987-224 769]; 2017, 1 253 214 [95% UI, 1 225 825-1 288 824]; percentage change, 562.0% [95% UI, 462.5%-705.8%]).

Corresponding maps depicting data for children younger than 1 year, 1 to 4 years, 5 to 9 years, and 10 to 19 years for CMNN, NCDs, and injuries separately are shown in eFigure 7 and eTable 3 in the Supplement. For most countries in sub-Saharan Africa, improvements were much faster than expected between 2000 and 2017 for children aged 1 to 4 years in particular, with several countries also having more rapid DALY improvement than expected in children younger than 1 year and aged 5 to 9 years. Among adolescents, on the other hand, there was little evidence of accelerated improvement after the turn of the century, with almost half of the countries in sub-Saharan Africa lagging behind expected improvements in DALY rates.

### Leading Causes of DALYs

The top 10 level 3 GBD causes of DALYs globally in 2017 for each region and country, along with their O:E DALY rates on the basis of SDI, are shown in eFigure 8 in the Supplement. Globally, for all children and adolescents, only 1 primarily nonfatal disease ranked in the top 10 of global DALYs: iron-deficient anemia (eighth; O:E ratio, 2.08). The rest of the top 10 are also leading causes of death, including neonatal disorders (O:E ratio, 1.38), lower respiratory infection (O:E ratio, 1.83), diarrhea (O:E ratio, 4.96), congenital birth defects (O:E ratio, 0.78), malaria (O:E ratio, 4596.33), meningitis (O:E ratio, 1.54), road injuries (O:E ratio, 0.69), protein-energy malnutrition O:E ratio, (9.73), and HIV/AIDS (O:E ratio, 10.49). Every country in sub-Saharan Africa had either neonatal disorders, malaria, or HIV/AIDS as the leading cause of DALYs, with either diarrhea or lower respiratory infection often ranked second. Neonatal disorders or congenital birth defects were ranked either first or second in most other countries. Important country-specific exceptions included natural disasters ranked first in Puerto Rico (O:E ratio, 5993.81), interpersonal violence ranked second or third in Brazil (O:E ratio, 5.36) and most of Central Latin America (example O:E ratios: Mexico, 3.59; Honduras, 3.11; Guatemala, 2.74; El Salvador, 5.89; Colombia, 3.36; Panama, 2.84; Venezuela, 7.18), and conflict and terror ranked first in Syria (O:E ratio, 11 497.8) and second in Iraq (3204.1) and Libya (O:E ratio, 3442.5).

Also notable was the burden of sudden infant death syndrome (SIDS) in infants; SIDS was ranked third cause of DALYs in children younger than 1 year in the high-SDI quintile and in the top 10 for all high-income countries, plus all of Eastern Europe and Central Europe, accounting for 0.71% of deaths in the late neonatal period (age range, 7-27 days) and 2.24% in the postneonatal period (age range, 28-364 days) in 2017 globally. Sudden infant death syndrome also accounted for 3.4% of postneonatal deaths and 17.0% of deaths in the late neonatal period in high-SDI locations, but only 0.67% and 2.15% of deaths, respectively, in low-SDI settings.

### The Growing Burden of Nonfatal Health Loss

Rates of YLDs decreased only slightly and nonsignificantly between 1990 and 2017. Amidst a backdrop of decreasing premature childhood death and population growth, global YLDs increased 4.7% to a total of 145 million (95% UI, 107-190 million) among children and adolescents. The YLD rates increased with age, from 4366 (3168-5797) per 100 000 population in children younger than 1 year to 4486 (3242-5956) per 100 000 population in children aged 1 to 4 years, 4981 (3560-6619) per 100 000 population in children aged 5 to 9 years, to 6542 (4845-8493) per 100 000 population in those aged 10 to 19 years. Temporal trends by region in YLL-to-YLD ratio and SDI (eFigure 9 in the Supplement) demonstrate an epidemiologic transition to nonfatal health loss even more pronounced than the transition in DALYs. The association of the YLL-to-YLD ratio and SDI categories was consistent for CMNN in all age groups (children younger than 1 year, 67.0 [95% UI, 48.8-90.4]; 1-4 years, 6.9 [95% UI, 4.9-9.0]; 5-9 years, 1.5 [95% UI, 0.8-1.6]; 10-19 years, 1.5 [95% UI, 0.8-1.4]) but was stark for NCDs in children younger than 1 year (children younger than 1 year, 34.0 [95% UI, 24.7-46.4]; 1-4 years, 2.1 [95% UI, 1.2-2.4]; 5-9 years, 0.9 [95% UI, 0.3-0.6]; 10-19 years, 0.8 [95% UI, 0.2-0.4]).

In this case, there was barely any association between SDI level and the YLL-to-YLD ratio until the highest SDI strata, which may have reflected a poor penetration of prevention and treatment services for congenital birth defects and neoplasms outside of high-income countries. In the case of NCDs, increasing SDI was associated with an increased YLL-to-YLD ratio in children aged 5 to 9 years and 10 to 19 years.

A similar association was found in the case of injuries (children younger than 1 year, 169.2 [95% UI, 117.2-245.8]; 1-4 years, 38.7 [95% UI, 27.2-52.7]; 5-9 years, 9.4 [95% UI, 6.4-11.7]; 10-19 years, 7.1 [95% UI, 4.8-8.3]). This could possibly reflect poorer prevention and treatment access for these causes and the effect of wars and natural disasters on disease burden.

In 2017, the top 10 level-3 causes of YLDs globally were iron-deficient anemia (O:E ratio, 2.08), vitamin A deficiency (O:E ratio, 1.88), headache (O:E ratio, 0.82), conduct disorder (O:E ratio, 0.87), neonatal disorders (O:E ratio, 1.13), anxiety disorder (O:E ratio, 0.86), skin diseases (O:E ratio, 0.79), lower back pain (O:E ratio, 0.79), congenital disorders (O:E ratio, 0.99), and depression (O:E ratio, 0.87) (eFigure 10 in the Supplement for results by age). Iron-deficient anemia or asthma sometimes lead most low and low-middle SDI countries, with neonatal disorders leading in most middle, high-middle, and high SDI countries.

Neonatal disorders was the only level 3 cause that ranked in the top 10 of both mortality and disability globally, ranking among the top 10 causes of YLDs in many countries in North Africa and the Middle East and sub-Saharan Africa. Musculoskeletal and mental health disorders (including anxiety disorders, conduct disorder, depression, autism spectrum disorders, and drug use disorders) were all highly ranked in high-income countries, in central and eastern Europe, and throughout Asia, Latin America, and the Caribbean. Hemoglobinopathies, such as sickle cell disorders and thalassemias, were also in the top 10 by O:E ratio in a number of countries, including Yemen (O:E ratio, 2.7), Burkina Faso (1.74), Côte d’Ivoire (2.03), Guinea (1.74), Liberia (1.85), Nigeria (3.21), and Sierra Leone (2.32). Among CMNN causes, HIV/AIDS was among the top 10 causes of YLDs in Malawi (O:E ratio, 55.32), Mozambique (O:E ratio, 54.35), Lesotho (O:E ratio, 150.1), Namibia (O:E ratio, 188.47), South Africa (O:E ratio, 339.23), Eswatini (also known as Swaziland; O:E ratio, 246.47), and Zimbabwe (O:E ratio, 58.79). Protein-energy malnutrition was in the top 10 causes in India (O:E ratio, 18.45), Mauritania (O:E ratio, 27.03), Djibouti (O:E ratio, 13.81), and South Sudan (O:E ratio, 1.82). Malaria was ranked in the top 5 causes in most countries of western and central sub-Saharan Africa, as well as Mozambique (O:E ratio, 2.48). Diarrhea, onchocerciasis, and intestinal nematode infections were the other CMNN causes among the top 10 causes of YLDs in certain countries. Injuries did not rank high in most countries, with notable exceptions of Iraq (where conflict ranked ninth; O:E ratio, 672.19) and Syria (where conflict ranked first; O:E ratio, 2962.5).

### Associating Maternal Health Outcomes With Those of Children and Adolescents

To evaluate the association between population-level trends in child and adolescent DALYs and those of their mothers, we compared percentage change from 1990 to 2017 in all-cause DALY rates for children younger than 1 year, 1 to 4 years, 5 to 9 years, and 10 to 19 years with percentage change in death rates owing to maternal disorders for women aged 10 to 54 years (eFigure 11 in the Supplement). There were strong correlations between trends in maternal death and all-cause DALY rates in all childhood age groups (<1 year, *r* = 0.589; 1-4 years, *r* = 0.452; 5-9 years, *r* = 0.507; 10-19 years, *r* = 0.379); those countries with the most improvement in maternal mortality also tended to have higher performance in reducing child and adolescent deaths. Statistical correlation was strongest for children younger than 1 year of age (*r* = 0.59), but continued even to health outcomes of older children and adolescents (*r* range = 0.38-0.45). The overall association between trends in maternal mortality and all-cause child and adolescent DALY rates became stronger after 2000 in all SDI quintiles other than high-middle SDI quintile (low: *r* = 0.539 in 1990-2000 vs *r* = 0.672 in 2000-2017; low-middle: *r* = 0.540 in 1990-2000 vs *r* = 0.576 in 2000-2017; middle: *r* = 0.419 in 1990-2000 vs *r* = 0.442 in 2000-2017; high-middle: *r* = 0.333 in 1990-2000 vs *r* = 0.296 in 2000-2017; high: *r* = 0.182 in 1990-2000 vs *r* = 0.379 in 2000-2017). A total of 25 countries (Afghanistan, American Samoa, Antigua, Burundi, Botswana, Canada, Switzerland, Costa Rica, Djibouti, Guam, Jamaica, Kuwait, Lesotho, Madagascar, Montenegro, Netherlands, Rwanda, Singapore, Sierra Leone, São Tomé and Principe, Eswatini, Chad, Thailand, United States, and St Vincent and the Grenadines) had divergent trends in maternal and child health (ie, 1 mortality rate increased while the other decreased) between 1990 and 2000, while that was only true for 21 countries (American Samoa, Antigua, Bahamas, Barbados, Brunei, Canada, Dominican Republic, Georgia, Greece, Grenada, Hungary, Ireland, Libya, Saint Lucia, Panama, Puerto Rico, Suriname, Syria, United States, St Vincent and the Grenadines, and US Virgin Islands) from 2000 to 2017. Only 8 countries had divergent trends throughout the entire time period, with all examples of divergence having increases in maternal mortality and decreases in all-cause child and adolescent DALY rates: American Samoa (21.5% and −33.1%, respectively), Canada (36.0% and −20.4%, respectively), Greece (33.8% and −28.5%, respectively), Guam (49.0% and −2.88%, respectively), Jamaica (4.14% and −28.0%, respectively), St Vincent and the Grenadines (7.29% and −24.2%, respectively), the United States (67.5% and −25.7%, respectively), and Zimbabwe (15.5% and −10.6%, respectively).

## Discussion

Children and adolescents in every country in the world were more likely to reach their 20th birthday in 2017 than ever before, but progress in improving health outcomes has been uneven. Mortality reductions were most rapid in children between the ages of 1 and 4 years, driven by global declines in deaths owing to diarrhea, lower respiratory infection, and other common infectious diseases. Improvements accelerated after 2000. The largest absolute declines were seen in Western, Eastern, and Central sub-Saharan Africa, while the fastest rates of decline were seen in East Asia, Andean Latin America, and South Asia. The pattern of change was closely associated with gains in sociodemographic development and temporally aligned with increased development assistance for health, which led to broad improvements in vaccination, early childhood nutrition, sanitation, clean water, and targeted interventions for HIV/AIDS and malaria.^3,17,18,19,20^

A vast unfinished agenda in child and adolescent health remains. While malaria has decreased dramatically across the African continent, there are many countries, especially in western sub-Saharan Africa, where parasite transmission, acute illness, and mortality from malaria remain high. Lower respiratory infection, diarrhea, and acute malnutrition also remain among the top killers of children and adolescents in the world in 2017. Investment in programs targeting prevention and effective syndromic treatment of CMNN disorders clearly pays dividends, and these investments must continue. In locations with higher SDIs, a continuing shift toward nonfatal health loss from NCDs, such as congenital birth defects, mental and behavioral disorders, injuries, and asthma are challenging health systems to adapt.^21^ The consistent burden of NCD-attributable DALYs in adolescents over the past 28 years illustrates a need for continued research and action on NCDs as communicable disease burden declines across the development spectrum. The burden of injuries in adolescents surpasses that of CMNN causes throughout the study period for middle-SDI through high-SDI countries, and with the relative faster decline of CMNN causes in low and low-middle countries, the relative ranking of injuries may switch in those locations in the near future.

Overall health improvements were slowest in adolescents. Few locations showed any evidence of improvements in health among adolescents that exceeded the trends expected with general societal development gains. Adolescence is a key phase of the life course and human development, including a phase of growth and maturation of the reproductive, musculoskeletal, neurodevelopmental, endocrine, metabolic, immune, and cardiometabolic systems into adulthood.^22^ Gains or lack thereof in adolescent health thus have the potential to influence individual and societal outcomes for periods substantially longer than the teenage years. In terms of family and home life, key issues include the improvement of sanitary and living conditions, stable food systems, quality education, and gainful employment.^23^ Also, HIV/AIDS remains an imminent threat to the health and well-being of older children and adolescents in many countries in sub-Saharan Africa, such as South Africa, Zimbabwe, Lesotho, Eswatini, Botswana, and Zambia. The large and growing burden of mental health and substance use disorders among older children and adolescents also is an emerging threat to the thrive component of the SDG survive and thrive agenda. While the psychological needs of children and adolescents show similarities across geographical settings,^24,25,26,27^ comparatively little is understood about modifiable risk factors or effective prevention programs for childhood mental illness, outside of ensuring that caregivers are attuned to the link between mental health disorders and self-harm.^28,29^ Injuries in general continue to be a major cause of early mortality and long-term disability among older children and adolescents in all countries. While many types of injuries, such as those arising from war and natural disasters, may not be preventable with health sector–based approaches, diligent preparedness planning can help mitigate the immediate health aftermath of them.^30,31,32^ Others are much more amenable to policies and programs that focus on prevention using what have come to be regarded as common-sense safety measures, such as speed limits, seat belts, and cycle helmets for road traffic accidents,^33,34^ fencing around water hazards and swimming-skills training for drowning,^35^ and policies to prevent self-harm via improving safety and limiting access to firearms and chemicals.^36,37^

At the other end of the age spectrum, neonatal disorders remain a major prevention and treatment challenge, especially for countries outside the high-SDI quintile that lack the same level of financial and human resources to dedicate to the intensive care needs of sick neonate. Investment is needed to develop and implement cost-effective interventions for neonatal disorders that take into account the dynamics of maternal health, risk-factor exposures during pregnancy, clinical care systems, supportive equipment needs, and the cultural differences around how families and communities care for newborns. It is important also to invest in the ongoing care of children who survive perinatal emergencies only to develop long-term complications, such as cerebral palsy. Congenital birth defects and hemoglobinopathies are 2 other groups of causes for which there is little evidence of improved outcomes outside the high-SDI quintile, perhaps reflecting the resource-intensive nature of averting deaths owing to such conditions and societal barriers to care^38^ but also likely because of a failure of recent clinical advances to be adopted in lower-resource settings.^39^

The close linkage between trends in maternal and child health reinforces the notion that the health of different population segments are closely interconnected.^40^ The simultaneous focus of the Millennium Development Goals on maternal and child mortality appears to have led to closer association between them since 2000 via alignment of funding streams, targeting of common risk factors between mothers and their children, an increased focus on delaying the age of parenthood by increasing education, contraception, and increased birth spacing, and catalyzing improved gender equity.^41,42,43,44,45,46,47^ There are strong ties between the physical health of women (eg, high body mass index, NCDs, nutrition) and neonatal outcomes (such as pregnancy complications, short gestational age, and low birth weight), which are in turn linked with poorer health outcomes and delayed development.^3,6^ This is to say nothing of the potential epigenetic connections between mothers and the health of their children that have the potential to extend beyond the neonatal period into childhood, adolescence, adulthood, and the next generation.^48^ The subset of countries that are outliers to this trend of concomitant improvement in maternal and child health warrant close examination to determine the underlying causes. Challenges are likely to arise whenever funding streams are decoupled, education or family planning programs are disrupted, or the health of young women is not prioritized.

The epidemiological transition has unique implications for the health of children and adolescents and the potential trajectory of socioeconomic development. In particular, as more children survive, the human capital potential societies will expand, but as more children with health problems are also surviving, there is potential for increased burden on health and education systems. The cost of sustaining progress on child and adolescent health and well-being is not insignificant. To achieve the goal of surviving and thriving and realized the human capital potential of children and adolescents, all countries must make strategic investments in education and health systems, including human resources for health, supply chains, infrastructure, governance, and increased support for children with developmental disabilities. Alignment of funding around interconnected drivers of human development and health loss is also required to achieve the SDGs.^49^

The SDGs are expansive, but they should not be considered a comprehensive rubric for achieving improved child and adolescent health. For example, outside of women’s reproductive health and experiences of sexual violence during adolescence, the SDG goals, targets, and indicators remain largely silent on the unique social, environmental, and biological determinants of health occurring in adolescence across the socioeconomic development spectrum. This blind spot in international health targets, planning, and prevention fails to capture the complex transitions occurring during adolescence in particular. Many additional nonhealth SDG indicators also focus on reducing poverty, expanding education, stabilizing environments, strengthening economies, and reducing overall socioeconomic inequality within each country and throughout the world, all of which are relevant to the health and well-being of young persons.

### Limitations

The GBD study is an iterative process and, despite continued methodological advancements and improvements in data, this study has a number of limitations. First, all limitations documented in the elements of the GBD estimation process that allow for YLL, YLD, and DALY estimation will contribute to uncertainty in these summary measures. Second, these summary measures of population health are influenced by data availability. Time lags in the reporting of health information by national authorities and thus subsequent incorporation into the GBD estimation mean that these estimates are based on data that are already out of date. Relatedly, data deficiencies from populations in conflict zones (eg, Syria, Iraq, Yemen, South Sudan, Afghanistan), autonomous subnational regions, and certain nongeographical subpopulations (ie, migrants, refugees, and some indigenous peoples) limit the precision of some of the estimated levels and trends of disease burden. Third, the association between YLLs, YLDs, DALYs, and SDIs, although explanatory, cannot be viewed as causal. Fourth, a nontrivial assumption of the analyses is the independence of the uncertainty calculated for YLLs and YLDs. Because of the link between death and prevalence, a positive correlation probably exists between these uncertainties that are not captured in this analysis. Study limitations specific to child and adolescent health include the comparatively poor quality of cause-of-death certification in neonates and infants vs older persons, the relatively broad age categorization of all 1-to-4-year-old children in 1 group, and the limited ability to quantify the magnitude of specific intergenerational, societal, and environmental factors that are ecologically suggested by this study.

## Conclusions

Globally, the aggregate health status of children and adolescents improved dramatically between 1990 and 2017, particularly owing to declines in death owing to infectious diseases, but nonfatal health loss has increased in both absolute and relative terms, and the gap between best and worst performers has widened. Continued monitoring of the drivers of child and adolescent health loss is crucial to sustain the progress of the past 26 years in the SDG era. The global community must commit to creating systematic accounting of drivers and consequences of long-lasting negative health outcomes beginning in childhood and the effects of long-term morbidity on health systems and human capital and ensuring that no populations are left behind. Only then will we be able to accelerate progress to 2030 and beyond.

## Notes

Supplement. **eFigure 1.** GBD 2017 Socio-demographic index (SDI) quintiles by GBD administrative level 1 geography **eFigure 2.** Decomposition of the probability of death in 195 countries for <20 years from 1990 to 2017, both sexes combined **eFigure 3. **Trends of DALYs from 1990 to 2017 for global and SDI quintiles for <1, 1-4, 5-9, 10-19 years **eFigure 4. **Trends of DALYs from 1990 to 2017 by age group (<1, 1-4, 5-9, 10-19 years) for global and SDI quintiles **eFigure 5.** Coevolution of disability-adjusted life years (DALYs) and socio-demographic index (SDI) for global disease burden (GDB) study regions and level 1 causes **eFigure 6. **Map of percent change for observed to expected (O:E) all-cause DALY rates from 1990 to 2000, 2000 to 2017 for <1, 1-4, 5-9, 10-19 years **eFigure 7. **Annual percent change of observed to expected (O:E) all-cause DALY rates in <20 years from 1990 to 2000 versus 2000 to 2017 **eFigure 8. **Leading ten causes of DALYs with the ratio of observed to expected on the basis of SDI alone in 2017 for a) <20 years, b) <1 years, c) 1-4 years, d) 5-9 years, and e) 10-19 years for both sexes combined **eFigure 9.** Co-evolution of YLL to YLD ratios and SDI for GBD regions and Level 1 causes, 1990 o 2017, both sexes combined for a) all causes, b) CMNN, c) NCDs, d) Injuries **eFigure 10.** Leading ten causes of YLDs with the ratio of observed to expected on the basis of SDI alone in 2017 for a) <20 years, b) <1 years, c) 1-4 years, d) 5-9 years, and e) 10-19 years for both sexes combined **eFigure 11. **Percent change in MMR vs. percent change in all-cause child DALYs rate for <1, 1-4, 5-9, and 10-19 years, 1990-2017 **eTable 1. **SDI groupings by location, based on GBD 2017 values **eTable 2. **ENN, LNN, PNN, 1-4, 5-9, 10-14, 15-19, <1, <5, 10-19, and <20 years mortality in 1990, 2000 and 2017 with mean percentage change for all GBD causes **eTable 3. **Percent change of observed to expected DALYs rate from 1990 to 2000 and 2000 to 2017 for each GBD location, for all causes combined and separately for communicable, maternal, neonatal, and nutritional (CMNN), non-communicable diseases (NCDs), and injuries or a) <20 years, b) <1 years, c) 1-4 years, d) 5-9 years, and e)10-19 years, both sexes combined
