# Supplementary material for: Diseases, Injuries, and Risk Factors in Child and Adolescent Health, 1990 to 2017: Findings From the Global Burden of Diseases, Injuries, and Risk Factors 2017 Study
Source: JAMA Pediatr. 2019 Apr 29;173(6):e190337. doi: 10.1001/jamapediatrics.2019.0337 (PMC6547084; doi:10.1001/jamapediatrics.2019.0337)
Supplement: Supplement. — eFigure 1. GBD 2017 Socio-demographic index (SDI) quintiles by GBD administrative level 1 geography eFigure 2. Decomposition of the probability of death in 195 countries for <20 years from 1990 to 2017, both sexes combined eFigure 3. Trends of DALYs from 1990 to 2017 for global and SDI quintiles for <1, 1-4, 5-9, 10-19 years eFigure 4. Trends of DALYs from 1990 to 2017 by age group (<1, 1-4, 5-9, 10-19 years) for global and SDI quintiles eFigure 5. Coevolution of disability-adjusted life years (DALYs) and socio-demographic index (SDI) for global disease burden (GDB) study regions and level 1 causes eFigure 6. Map of percent change for observed to expected (O:E) all-cause DALY rates from 1990 to 2000, 2000 to 2017 for <1, 1-4, 5-9, 10-19 years eFigure 7. Annual percent change of observed to expected (O:E) all-cause DALY rates in <20 years from 1990 to 2000 versus 2000 to 2017 eFigure 8. Leading ten causes of DALYs with the ratio of observed to expected on the basis of SDI alone in 2017 for a) <20 years, b) <1 years, c) 1-4 years, d) 5-9 years, and e) 10-19 years for both sexes combined eFigure 9. Co-evolution of YLL to YLD ratios and SDI for GBD regions and Level 1 causes, 1990 o 2017, both sexes combined for a) all causes, b) CMNN, c) NCDs, d) Injuries eFigure 10. Leading ten causes of YLDs with the ratio of observed to expected on the basis of SDI alone in 2017 for a) <20 years, b) <1 years, c) 1-4 years, d) 5-9 years, and e) 10-19 years for both sexes combined eFigure 11. Percent change in MMR vs. percent change in all-cause child DALYs rate for <1, 1-4, 5-9, and 10-19 years, 1990-2017 eTable 1. SDI groupings by location, based on GBD 2017 values eTable 2. ENN, LNN, PNN, 1-4, 5-9, 10-14, 15-19, <1, <5, 10-19, and <20 years mortality in 1990, 2000 and 2017 with mean percentage change for all GBD causes eTable 3. Percent change of observed to expected DALYs rate from 1990 to 2000 and 2000 to 2017 for each GBD location, for all causes combined and separately for comm [file jamapediatr-173-e190337-s001.pdf]

Supplementary Online Content

GBD 2017 Child and Adolescent Health Collaborators. Diseases, injuries, and risk factors in child and adolescent health, 1990 to 2017: findings from the Global Burden of Diseases, Injuries, and Risk Factors 2017 Study. *JAMA Pediatr.* Published online April 29, 2019. doi:10.1001/jamapediatrics.2019.0337

eFigure 1. GBD 2017 Socio-demographic index (SDI) quintiles by GBD administrative level 1 geography

eFigure 2. Decomposition of the probability of death in 195 countries for <20 years from 1990 to 2017, both sexes combined

eFigure 3. Trends of DALYs from 1990 to 2017 for global and SDI quintiles for <1, 1-4, 5-9, 10-19 years

eFigure 4. Trends of DALYs from 1990 to 2017 by age group (<1, 1-4, 5-9, 10-19 years) for global and SDI quintiles

eFigure 5. Map of percent change for observed to expected (O:E) all-cause DALY rates from 1990 to 2000, 2000 to 2017 for <1, 1-4, 5-9, 10-19 years

eFigure 6. Annual percent change of observed to expected (O:E) all-cause DALY rates in <20 years from 1990 to 2000 versus 2000 to 2017

eFigure 7. Leading ten causes of DALYs with the ratio of observed to expected on the basis of SDI alone in 2017 for a) <20 years, b) <1 years, c) 1-4 years, d) 5-9 years, and e) 10-19 years for both sexes combined

eFigure 8. Co-evolution of YLL to YLD ratios and SDI for GBD regions and Level 1 causes, 1990 o 2017, both sexes combined for a) all causes, b) CMNN, c) NCDs, d) Injuries

eFigure 9. Leading ten causes of YLDs with the ratio of observed to expected on the basis of SDI alone in 2017 for a) <20 years, b) <1 years, c) 1-4 years, d) 5-9 years, and e) 10-19 years for both sexes combined

eFigure 10. Percent change in MMR vs. percent change in all-cause child DALYs rate for <1, 1-4, 5-9, and 10-19 years, 1990-2017

eTable 1. SDI groupings by location, based on GBD 2017 values

eTable 2. ENN, LNN, PNN, 1-4, 5-9, 10-14, 15-19, <1, <5, 10-19, and <20 years mortality in 1990, 2000 and 2017 with mean percentage change for all GBD causes

eTable 3. Percent change of observed to expected DALYs rate from 1990 to 2000 and 2000 o 2017 for each GBD location, for all causes combined and separately for communicable, maternal, neonatal, and nutritional (CMNN), non-communicable diseases (NCDs), and injuries or a) <20 years, b) <1 years, c) 1-4 years, d) 5-9 years, and e)10-19 years, both sexes combined

© 2019 GBD 2017 Child and Adolescent Health Collaborators. JAMA Pediatrics.

eFigure 1. GBD 2017 Socio-demographic index quintiles by GBD geography

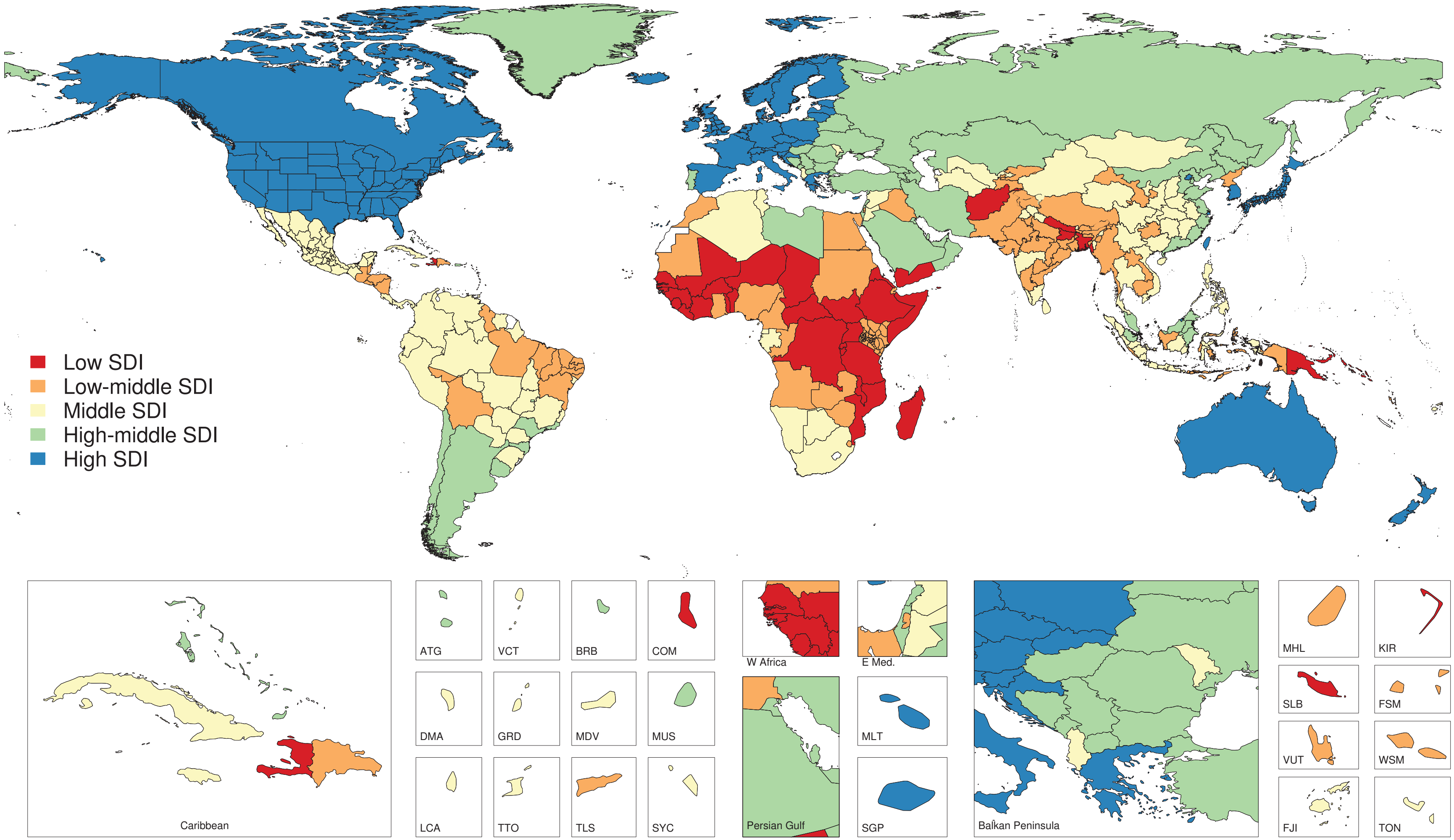

Socio-demographic index quintiles for each GBD analytic location are color-coded. SDI is calculated by principal components analysis of per capita income, years of education in persons 15 years and older, and total fertility rate under 25 years. Inset plots provided for detailed inspection of small or clustered regions. Abbreviations: GBD=Global Burden of Disease, ATG = Antigua and Barbuda, DMA = Dominica, LCA = Saint Lucia, VCT = Saint Vincent and the Grenadines, GRD = Grenada, TTO = Trinidad and Tobago, BRB = Barbados, MDV = Maldives, TLS = Timor-Leste, COM = Comoros, MUS = Mauritius, SYC = Seychelles, MLT = Malta, SGP = Singapore, MHL = Marshall Islands, SLB = Solomon Islands, VUT = Vanuatu, FJI = Fiji, KIR = Kiribati, FSM = Federated States of Micronesia, WSM = Samoa, TON = Tonga

eFigure 2. Decomposition of the Probability of Death in 195 countries for <20 years from 1990 to 2017, both sexes combined

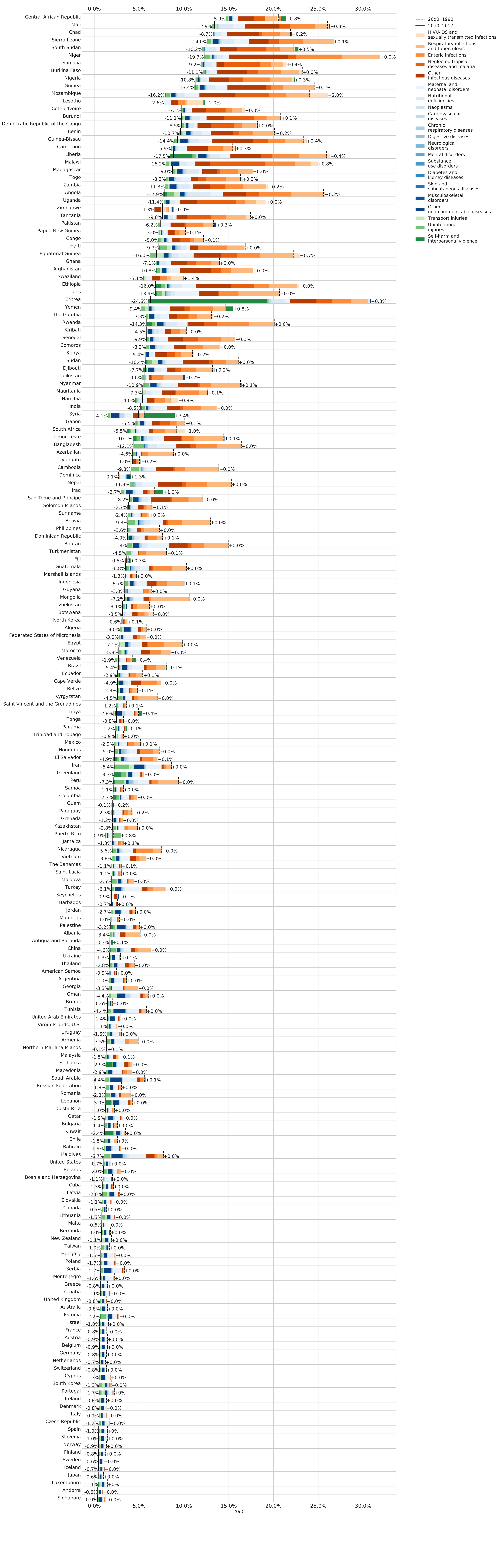

Probability of death between birth and age 20 is plotted by country for 1990 (dashed vertical line) and 2017 (solid vertical line). The relative contribution of that change due to the 22 different level 2 causes is indicated by different color bars. Any bar to the right of the dashed vertical line increased mortality over the period while all those to the left decreased. Countries are sorted from highest probability of death in 2017 to lowest. Country data are ordered according to probability of death in 2017, descending

eFigure 3. Trends of DALYs from 1990 to 2017 for global and SDI quintiles for <1, 1-4, 5-9, 10-19.

© 2019 GBD 2017 Child and Adolescent Health Collaborators. JAMA Pediatrics.

(a) <1 year

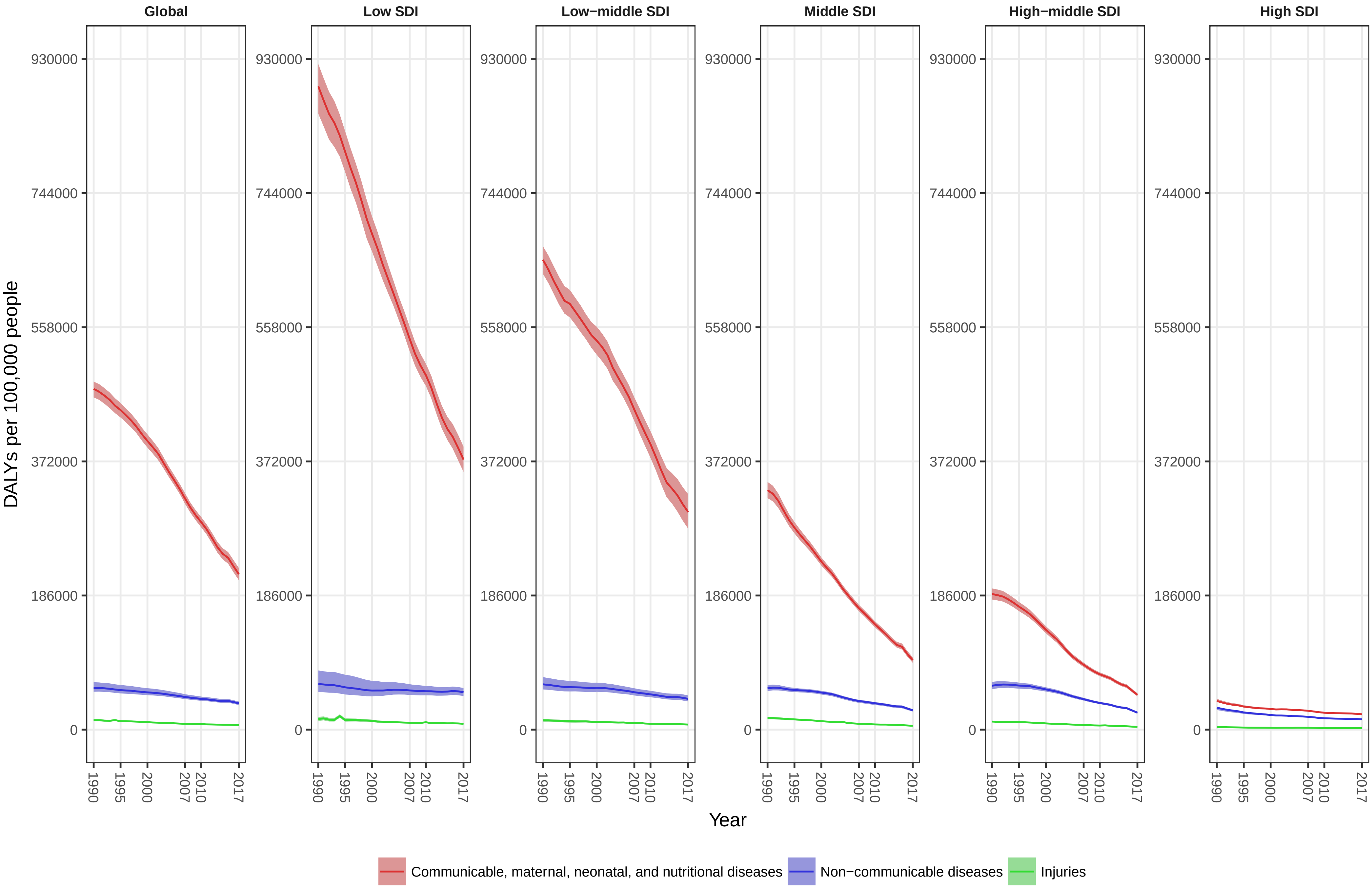

Temporal trends in disability adjusted life years (DALYs) are plotted for each adolescent age group. Panel a) less than 1 year old; panel b) between 1 and 4 years old; panel c) between 5 and 9 years old; panel d) between 10 and 19 years old. Within each panel, global trends are plotted in left sub-panel and the corresponding trends for each SDI quintile is plotted in the next 4 sub-panels. Shaded areas show 95% UIs. Communicable, maternal, neonatal, and nutritional disorders (CMNN) are shown in red, non−communicable disease (NCD) causes in blue, and injuries in green. Abbreviations: SDI=Socio−demographic Index.

eFigure 3. Trends of DALYs from 1990 to 2017 for global and SDI quintiles for <1, 1-4, 5-9, 10-19.

© 2019 GBD 2017 Child and Adolescent Health Collaborators. JAMA Pediatrics.

(b) 1 to 4 years

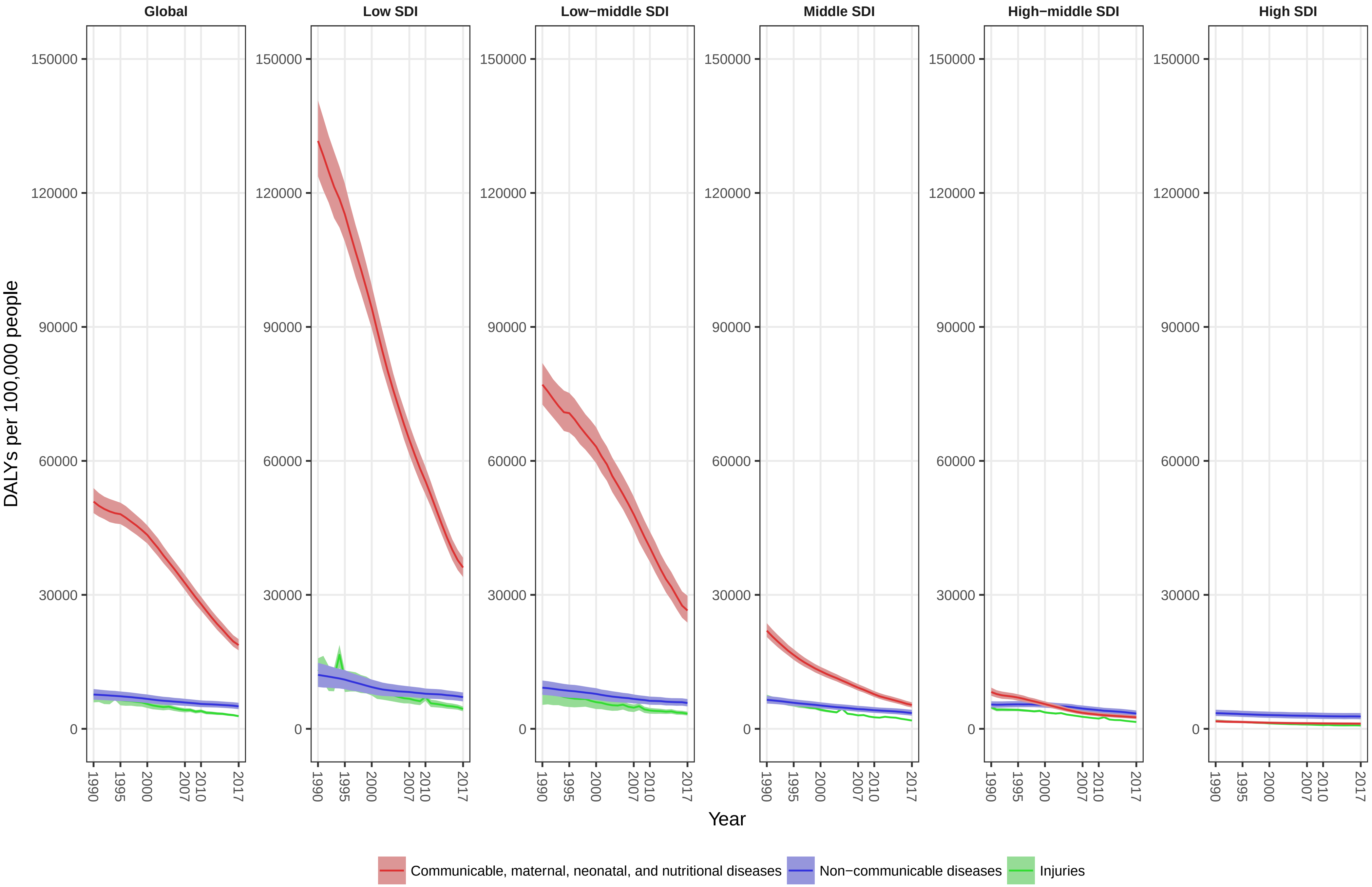

Temporal trends in disability adjusted life years (DALYs) are plotted for each adolescent age group. Panel a) less than 1 year old; panel b) between 1 and 4 years old; panel c) between 5 and 9 years old; panel d) between 10 and 19 years old. Within each panel, global trends are plotted in left sub-panel and the corresponding trends for each SDI quintile is plotted in the next 4 sub-panels. Shaded areas show 95% UIs. Communicable, maternal, neonatal, and nutritional disorders (CMNN) are shown in red, non−communicable disease (NCD) causes in blue, and injuries in green. Abbreviations: SDI=Socio−demographic Index.

eFigure 3. Trends of DALYs from 1990 to 2017 for global and SDI quintiles for <1, 1-4, 5-9, 10-19.

© 2019 GBD 2017 Child and Adolescent Health Collaborators. JAMA Pediatrics.

(c) 5 to 9 years

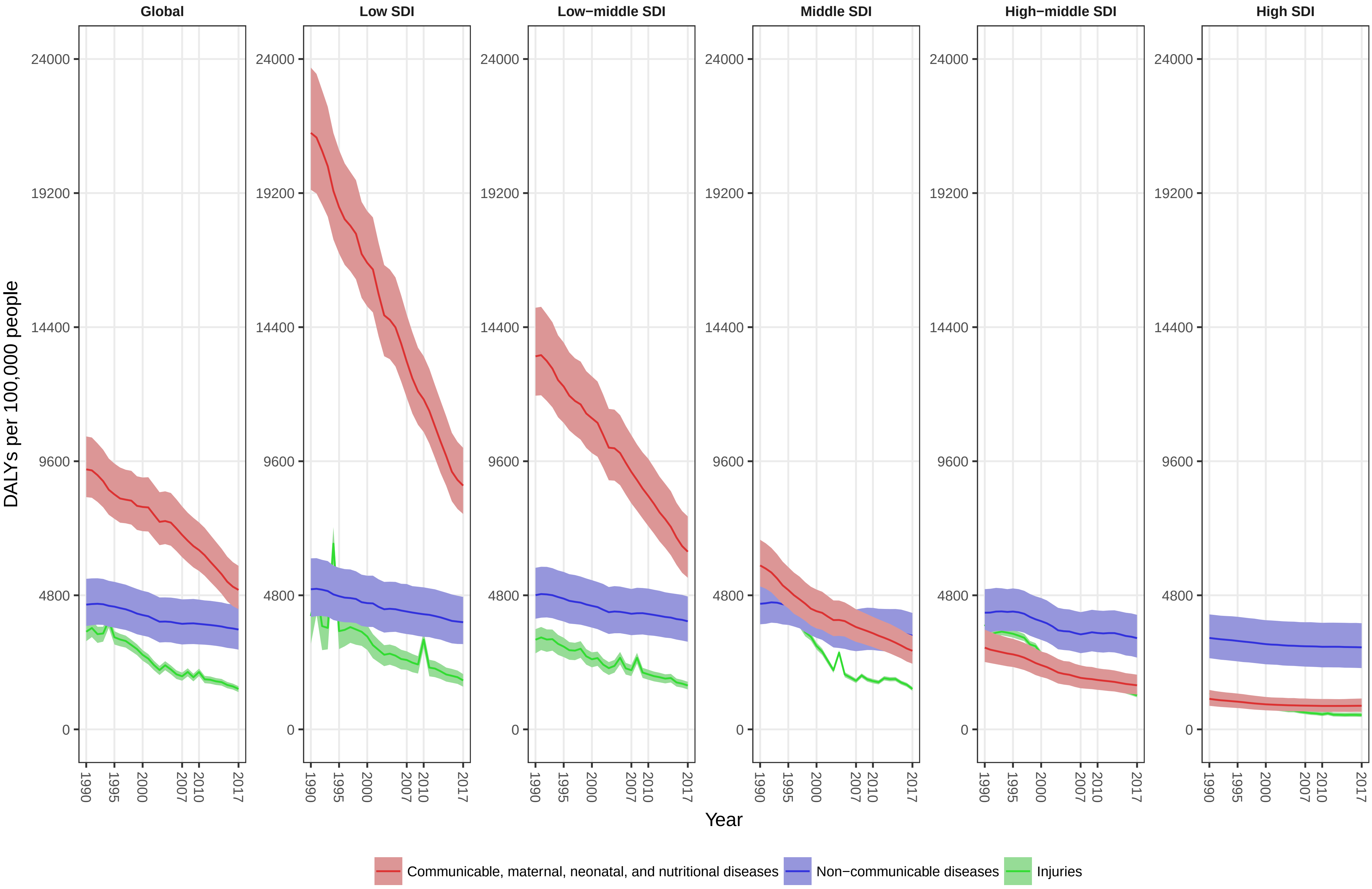

Temporal trends in disability adjusted life years (DALYs) are plotted for each adolescent age group. Panel a) less than 1 year old; panel b) between 1 and 4 years old; panel c) between 5 and 9 years old; panel d) between 10 and 19 years old. Within each panel, global trends are plotted in left sub-panel and the corresponding trends for each SDI quintile is plotted in the next 4 sub-panels. Shaded areas show 95% UIs. Communicable, maternal, neonatal, and nutritional disorders (CMNN) are shown in red, non−communicable disease (NCD) causes in blue, and injuries in green. Abbreviations: SDI=Socio−demographic Index.

eFigure 3. Trends of DALYs from 1990 to 2017 for global and SDI quintiles for <1, 1-4, 5-9, 10-19.

© 2019 GBD 2017 Child and Adolescent Health Collaborators. JAMA Pediatrics.

(d) 10 to 19 years

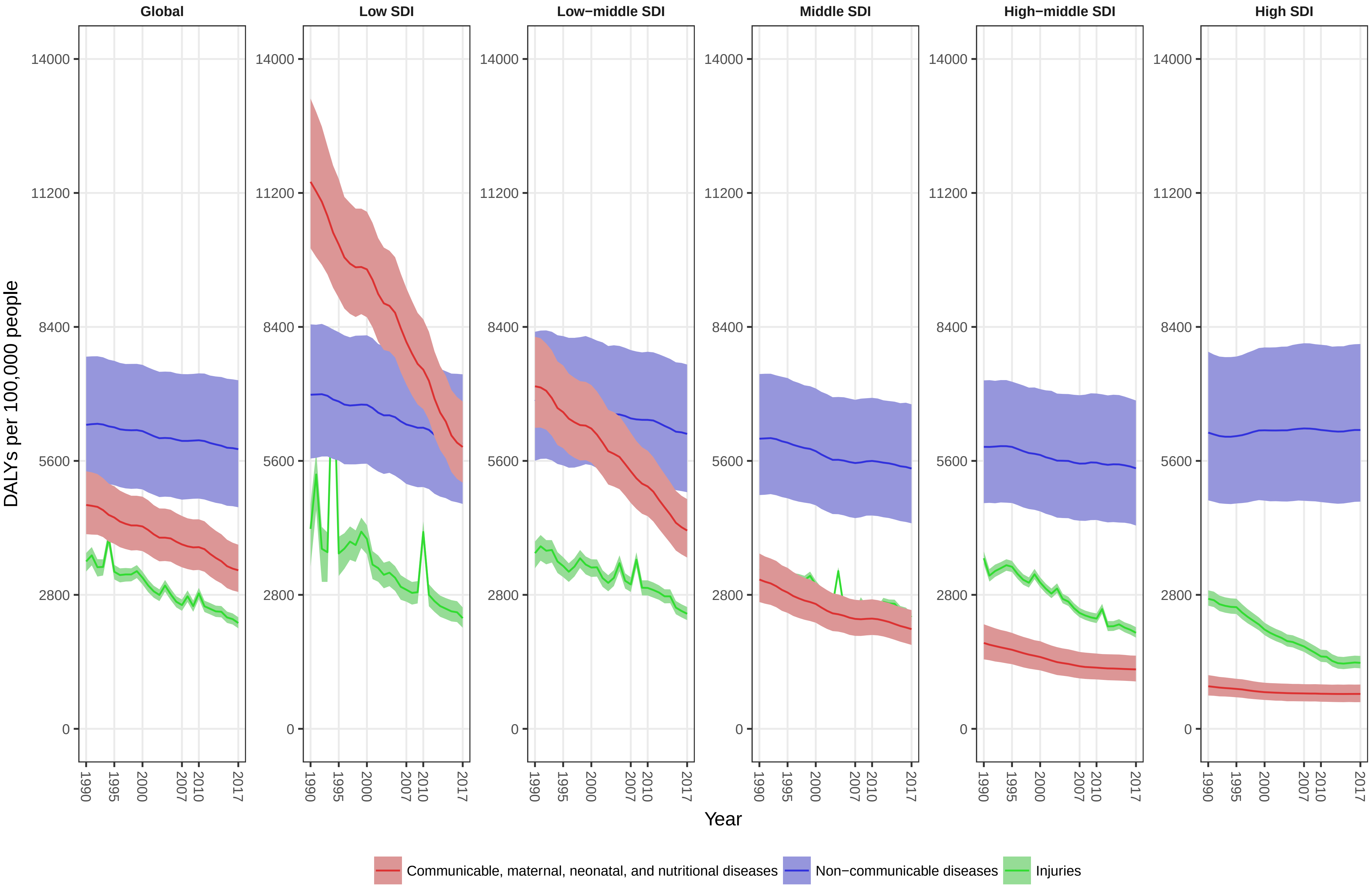

Temporal trends in disability adjusted life years (DALYs) are plotted for each adolescent age group. Panel a) less than 1 year old; panel b) between 1 and 4 years old; panel c) between 5 and 9 years old; panel d) between 10 and 19 years old. Within each panel, global trends are plotted in left sub-panel and the corresponding trends for each SDI quintile is plotted in the next 4 sub-panels. Shaded areas show 95% UIs. Communicable, maternal, neonatal, and nutritional disorders (CMNN) are shown in red, non−communicable disease (NCD) causes in blue, and injuries in green. Abbreviations: SDI=Socio−demographic Index.

eFigure 4. Trends of DALYs from 1990 to 2017 by age group (<1, 1-4, 5-9, 10-19 years) for global and SDI quintiles.

© 2019 GBD 2017 Child and Adolescent Health Collaborators. JAMA Pediatrics.

(a) Global

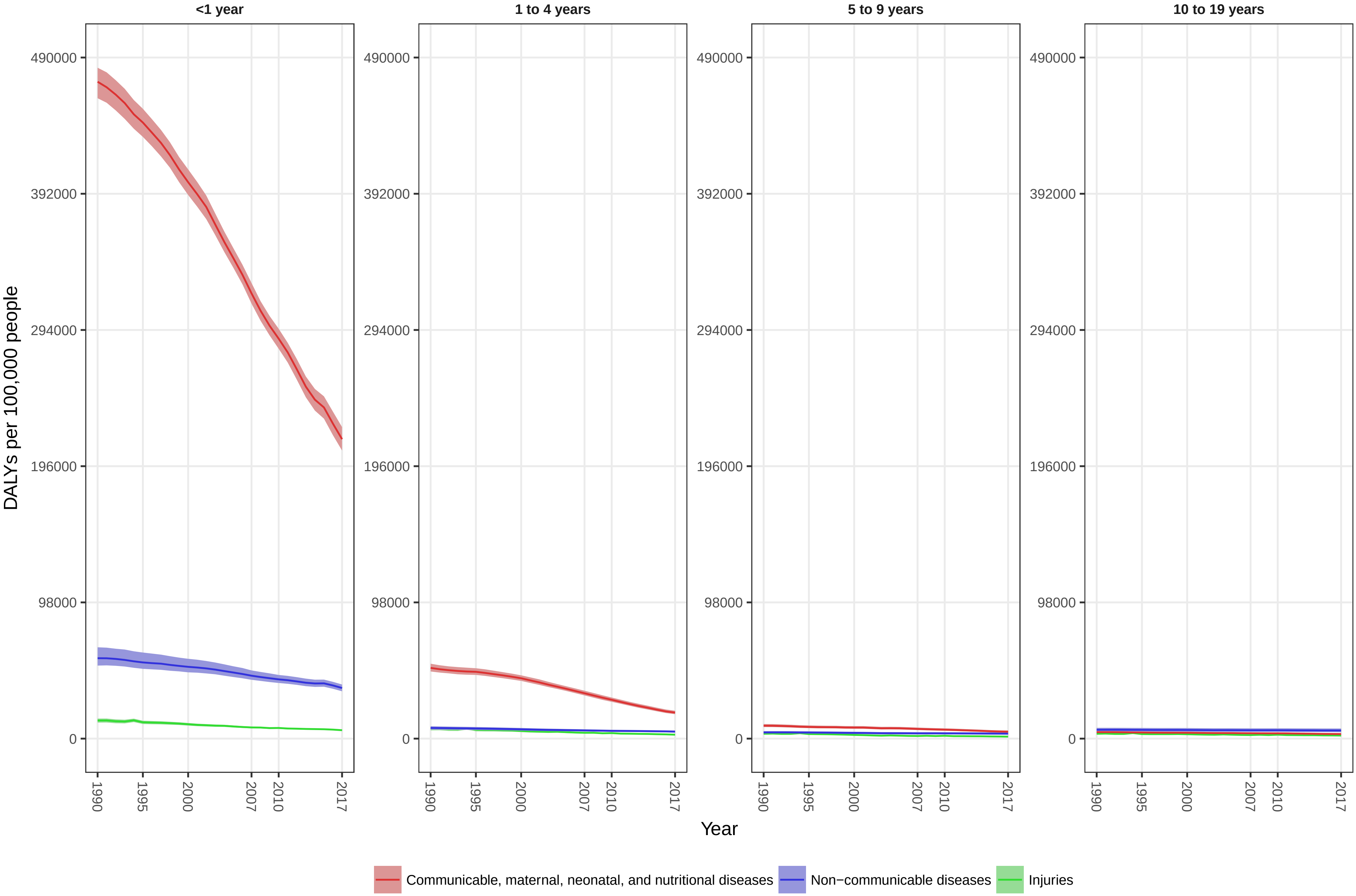

Temporal trends in disability adjusted life years (DALYs) are plotted across age groups globally and for each socio-demographic index (SDI) quintile. Panel a) global; panel b) low SDI; panel c) low-middle SDI; panel d) middle SDI; panel e) high-middle SDI; panel f) high SDI. Within each panel, trends for children less than 1 are plotted in left sub-panel, between 1 and 4 in the second sub-panel, between 5 and 9 in the third sub-panel, and between 10 and 19 in the right sub-panel. Shaded areas show 95% UIs. Communicable, maternal, neonatal, and nutritional disorders (CMNN) are shown in red, non−communicable disease (NCD) causes in blue, and injuries in green. Abbreviations: DALY=disability-adjusted life year, SDI=Socio-demographic Index.

eFigure 4. Trends of DALYs from 1990 to 2017 by age group (<1, 1-4, 5-9, 10-19 years) for global and SDI quintiles.

© 2019 GBD 2017 Child and Adolescent Health Collaborators. JAMA Pediatrics.

(b) Low SDI

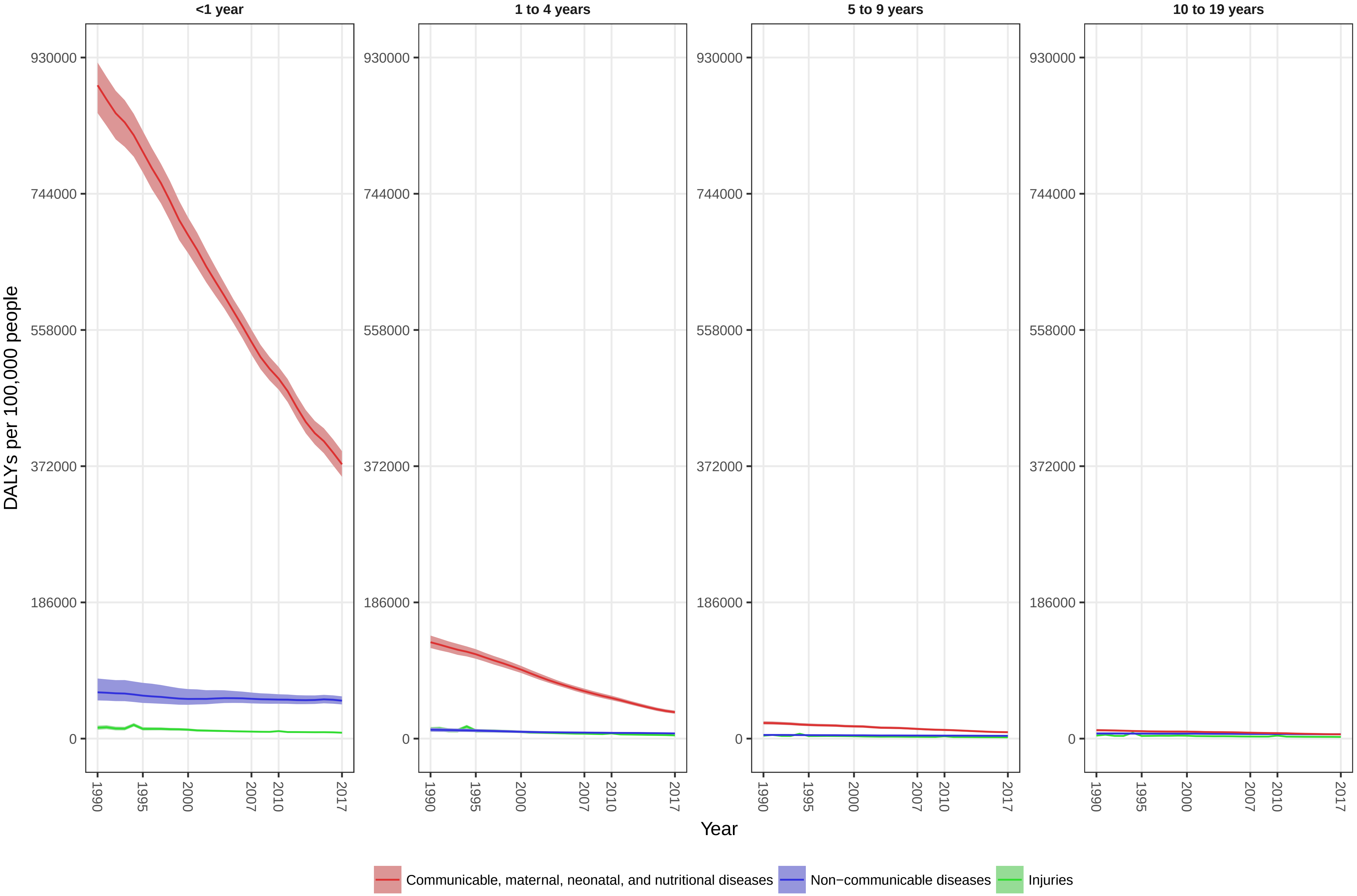

Temporal trends in disability adjusted life years (DALYs) are plotted across age groups globally and for each socio-demographic index (SDI) quintile. Panel a) global; panel b) low SDI; panel c) low-middle SDI; panel d) middle SDI; panel e) high-middle SDI; panel f) high SDI. Within each panel, trends for children less than 1 are plotted in left sub-panel, between 1 and 4 in the second sub-panel, between 5 and 9 in the third sub-panel, and between 10 and 19 in the right sub-panel. Shaded areas show 95% UIs. Communicable, maternal, neonatal, and nutritional disorders (CMNN) are shown in red, non−communicable disease (NCD) causes in blue, and injuries in green. Abbreviations: DALY=disability-adjusted life year, SDI=Socio-demographic Index.

eFigure 4. Trends of DALYs from 1990 to 2017 by age group (<1, 1-4, 5-9, 10-19 years) for global and SDI quintiles.

© 2019 GBD 2017 Child and Adolescent Health Collaborators. JAMA Pediatrics.

(c) Low−middle SDI

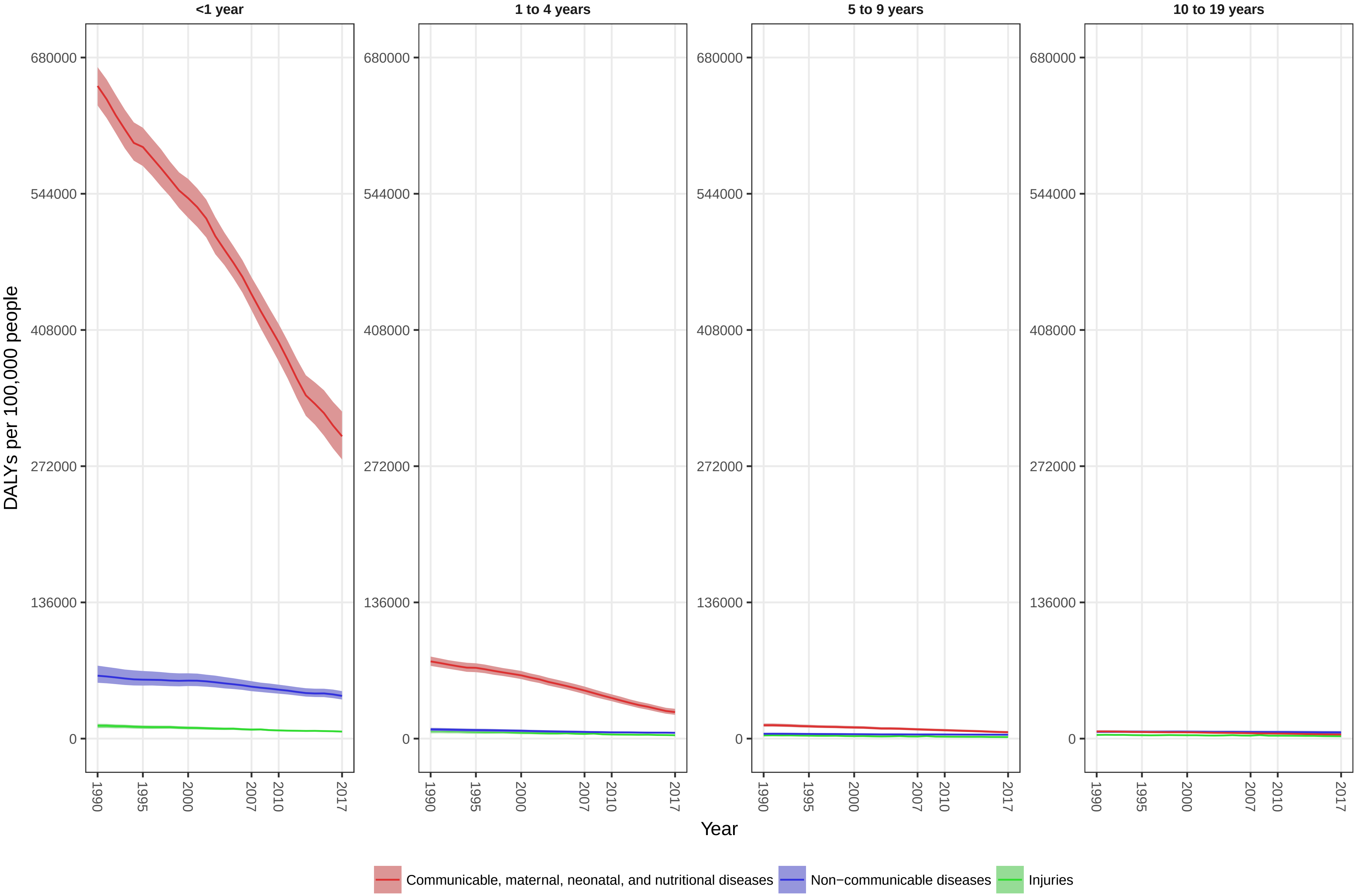

Temporal trends in disability adjusted life years (DALYs) are plotted across age groups globally and for each socio-demographic index (SDI) quintile. Panel a) global; panel b) low SDI; panel c) low-middle SDI; panel d) middle SDI; panel e) high-middle SDI; panel f) high SDI. Within each panel, trends for children less than 1 are plotted in left sub-panel, between 1 and 4 in the second sub-panel, between 5 and 9 in the third sub-panel, and between 10 and 19 in the right sub-panel. Shaded areas show 95% UIs. Communicable, maternal, neonatal, and nutritional disorders (CMNN) are shown in red, non−communicable disease (NCD) causes in blue, and injuries in green. Abbreviations: DALY=disability-adjusted life year, SDI=Socio-demographic Index.

eFigure 4. Trends of DALYs from 1990 to 2017 by age group (<1, 1-4, 5-9, 10-19 years) for global and SDI quintiles.

© 2019 GBD 2017 Child and Adolescent Health Collaborators. JAMA Pediatrics.

(d) Middle SDI

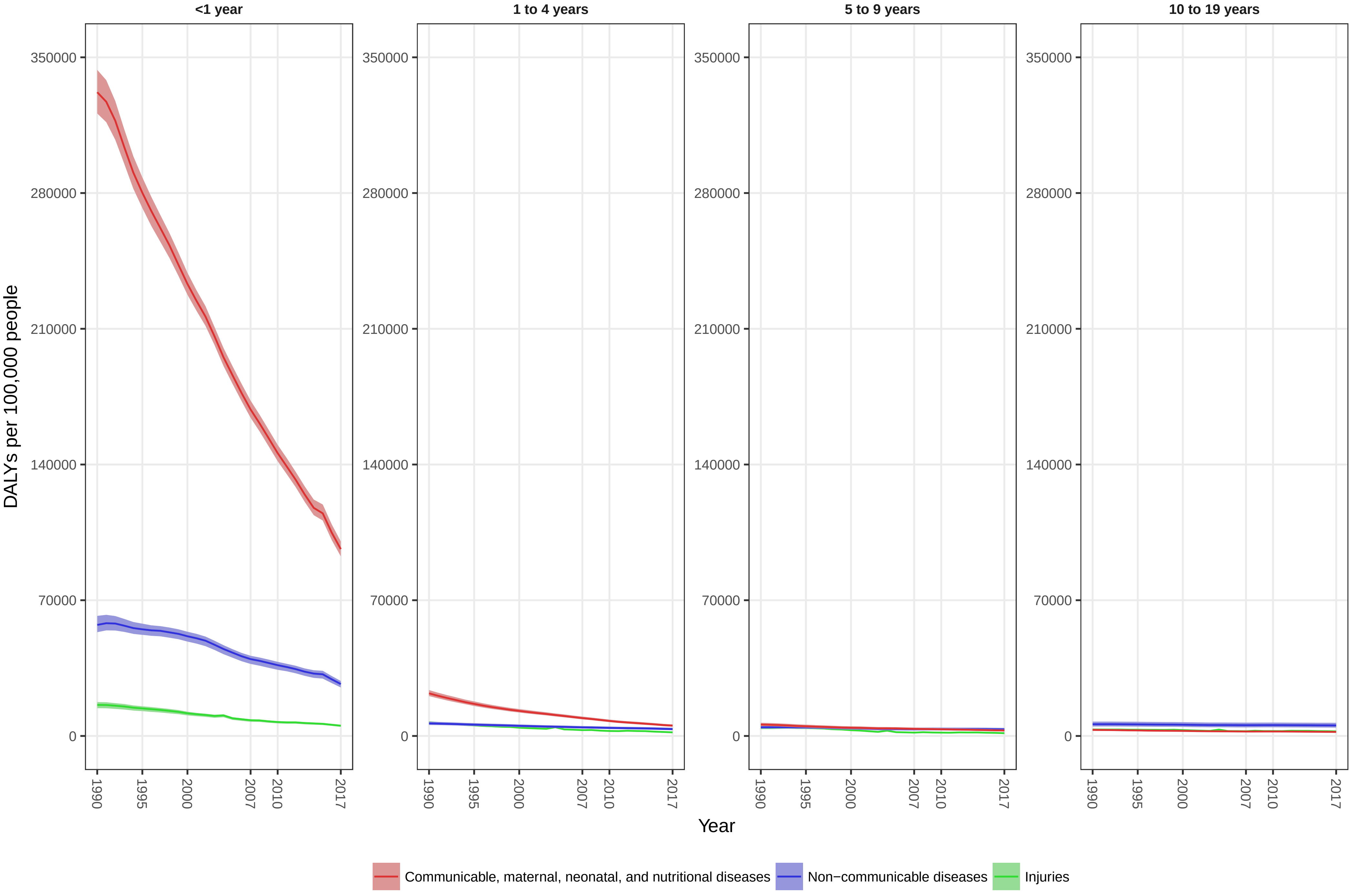

Temporal trends in disability adjusted life years (DALYs) are plotted across age groups globally and for each socio-demographic index (SDI) quintile. Panel a) global; panel b) low SDI; panel c) low-middle SDI; panel d) middle SDI; panel e) high-middle SDI; panel f) high SDI. Within each panel, trends for children less than 1 are plotted in left sub-panel, between 1 and 4 in the second sub-panel, between 5 and 9 in the third sub-panel, and between 10 and 19 in the right sub-panel. Shaded areas show 95% UIs. Communicable, maternal, neonatal, and nutritional disorders (CMNN) are shown in red, non−communicable disease (NCD) causes in blue, and injuries in green. Abbreviations: DALY=disability-adjusted life year, SDI=Socio-demographic Index.

eFigure 4. Trends of DALYs from 1990 to 2017 by age group (<1, 1-4, 5-9, 10-19 years) for global and SDI quintiles.

© 2019 GBD 2017 Child and Adolescent Health Collaborators. JAMA Pediatrics.

(e) High−middle SDI

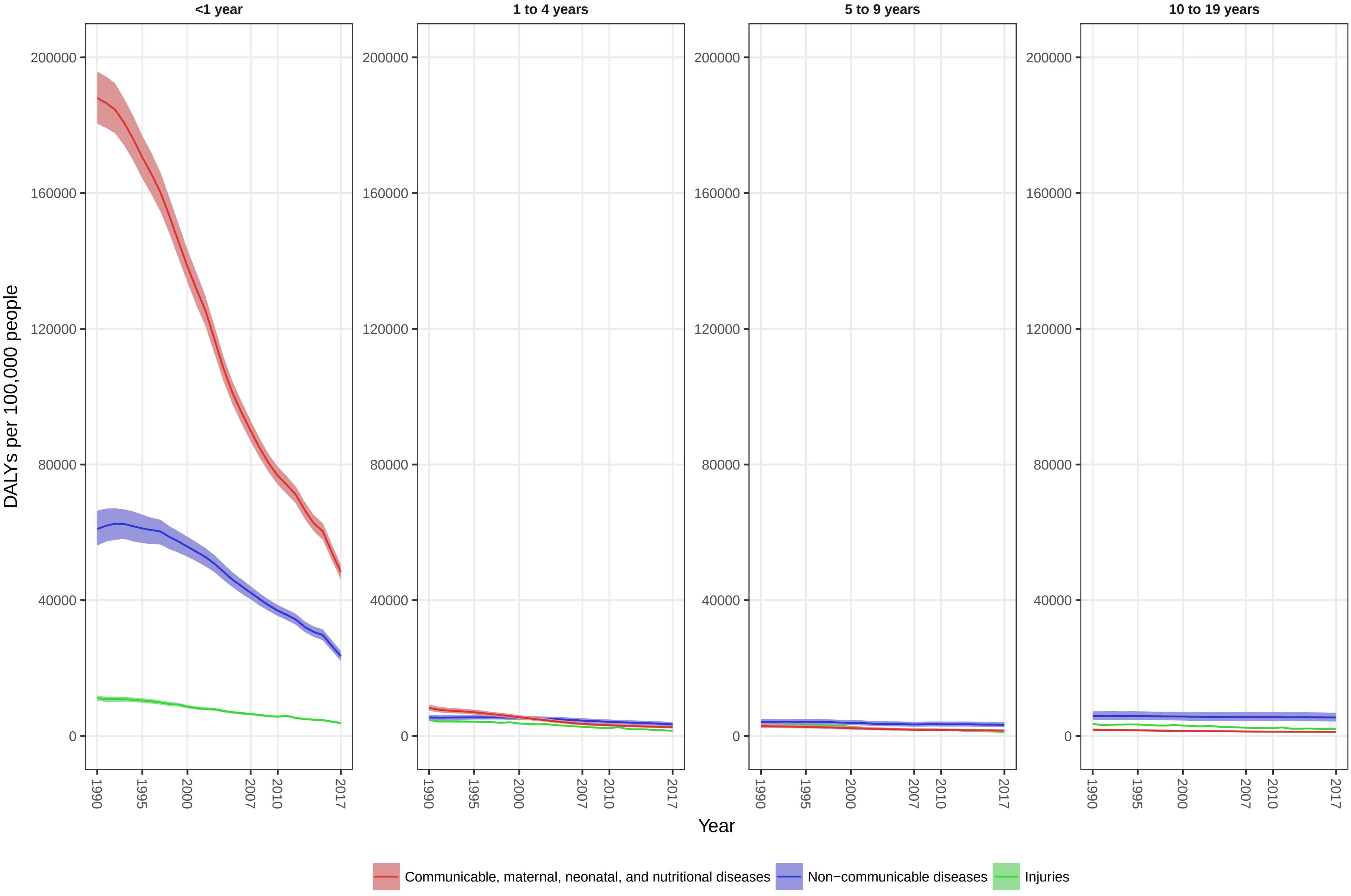

Temporal trends in disability adjusted life years (DALYs) are plotted across age groups globally and for each socio-demographic index (SDI) quintile. Panel a) global; panel b) low SDI; panel c) low-middle SDI; panel d) middle SDI; panel e) high-middle SDI; panel f) high SDI. Within each panel, trends for children less than 1 are plotted in left sub-panel, between 1 and 4 in the second sub-panel, between 5 and 9 in the third sub-panel, and between 10 and 19 in the right sub-panel. Shaded areas show 95% UIs. Communicable, maternal, neonatal, and nutritional disorders (CMNN) are shown in red, non−communicable disease (NCD) causes in blue, and injuries in green. Abbreviations: DALY=disability-adjusted life year, SDI=Socio-demographic Index.

eFigure 4. Trends of DALYs from 1990 to 2017 by age group (<1, 1-4, 5-9, 10-19 years) for global and SDI quintiles.

(f) High SDI

© 2019 GBD 2017 Child and Adolescent Health Collaborators. JAMA Pediatrics.

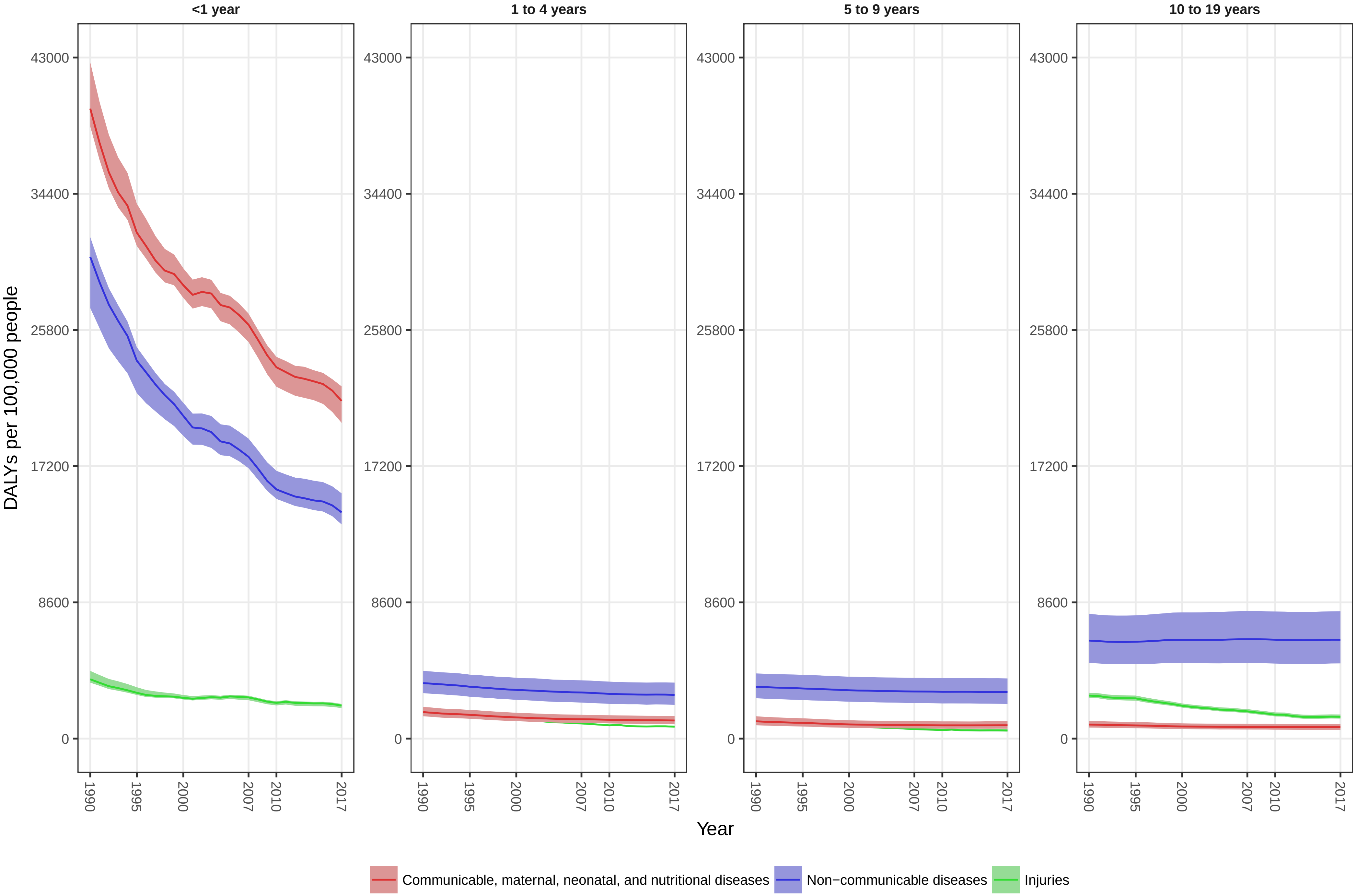

Temporal trends in disability adjusted life years (DALYs) are plotted across age groups globally and for each socio-demographic index (SDI) quintile. Panel a) global; panel b) low SDI; panel c) low-middle SDI; panel d) middle SDI; panel e) high-middle SDI; panel f) high SDI. Within each panel, trends for children less than 1 are plotted in left sub-panel, between 1 and 4 in the second sub-panel, between 5 and 9 in the third sub-panel, and between 10 and 19 in the right sub-panel. Shaded areas show 95% UIs. Communicable, maternal, neonatal, and nutritional disorders (CMNN) are shown in red, non−communicable disease (NCD) causes in blue, and injuries in green. Abbreviations: DALY=disability-adjusted life year, SDI=Socio-demographic Index.

eFigure 5a: Map of percent change for observed to expected (O:E) all−cause DALY rates from 1990 to 2000, 2000 to 2017 for <1, 1−4, 5−9, 10−19 years

© 2019 GBD 2017 Child and Adolescent Health Collaborators. JAMA Pediatrics.

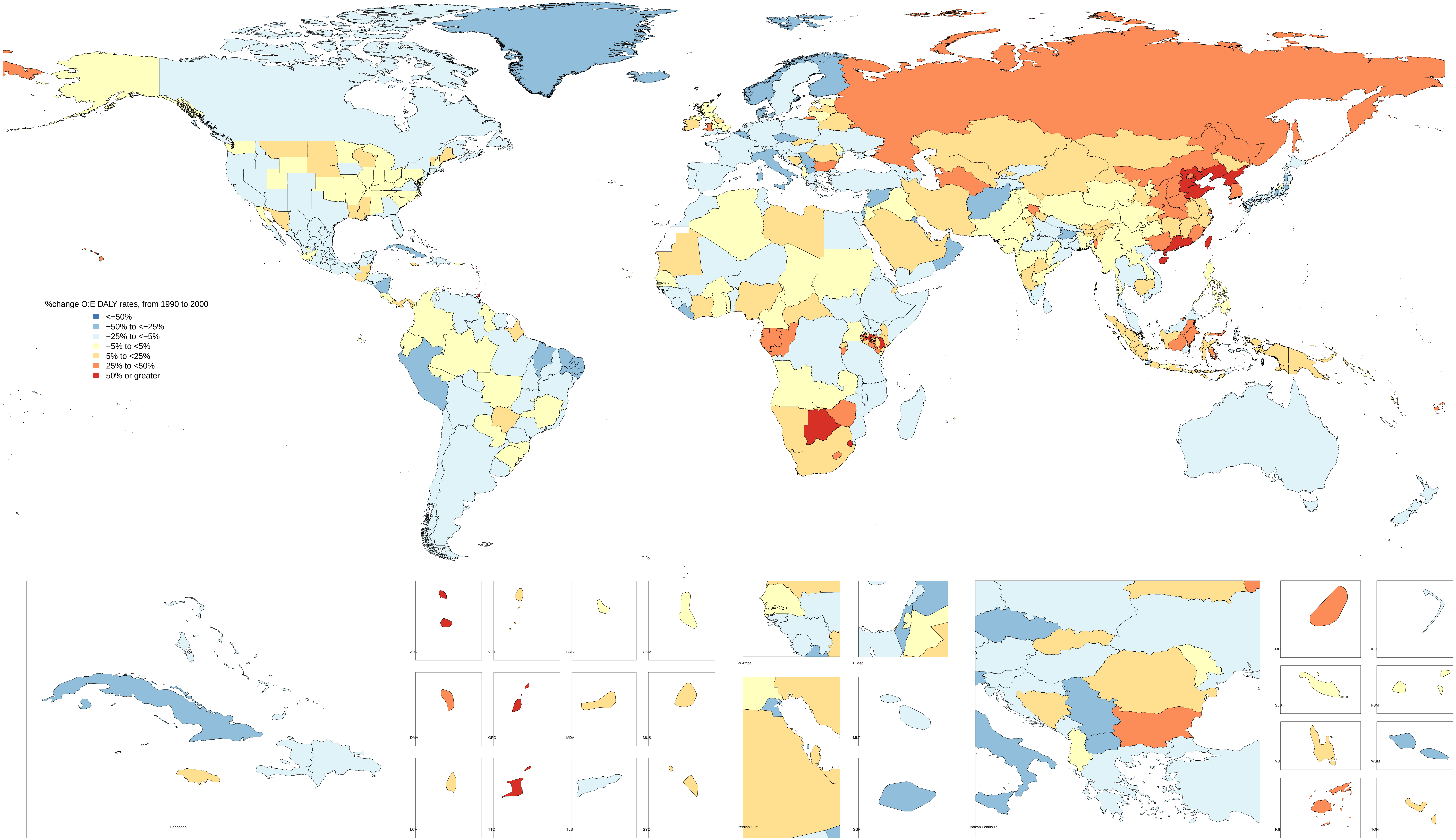

Percent changes in observed to expected disability-adjusted life years (DALYs) are plotted for different time periods and age groups. Panels A-D display changes from 1990 to 2000. Panels E-H display changes from 2000 to 2017. Panels A and E show changes for children less than 1; panels B and F show changes for children between 1 and 4; panels C and G show changes for children between 5 and 9; and panels D and H show changes for children between 10 and 19. For all plots, both sexes are combined. Subnational differentiation occurs within each country GBD models at the subnational level. Inset plots provided for detailed inspection of small or clustered regions. Abbreviations: DALY=disability-adjusted life year, GBD=Global Burden of Disease, ATG = Antigua and Barbuda, DMA = Dominica, LCA = Saint Lucia, VCT = Saint Vincent and the Grenadines, GRD = Grenada, TTO = Trinidad and Tobago, BRB = Barbados, MDV = Maldives, TLS = Timor-Leste, COM = Comoros, MUS = Mauritius, SYC = Seychelles, MLT = Malta, SGP = Singapore, MHL = Marshall Islands, SLB = Solomon Islands, VUT = Vanuatu, FJI = Fiji, KIR = Kiribati, FSM = Federated States of Micronesia, WSM = Samoa, TON = Tonga

eFigure 5b: Map of percent change for observed to expected (O:E) all−cause DALY rates from 1990 to 2000, 2000 to 2017 for <1, 1−4, 5−9, 10−19 years

© 2019 GBD 2017 Child and Adolescent Health Collaborators. JAMA Pediatrics.

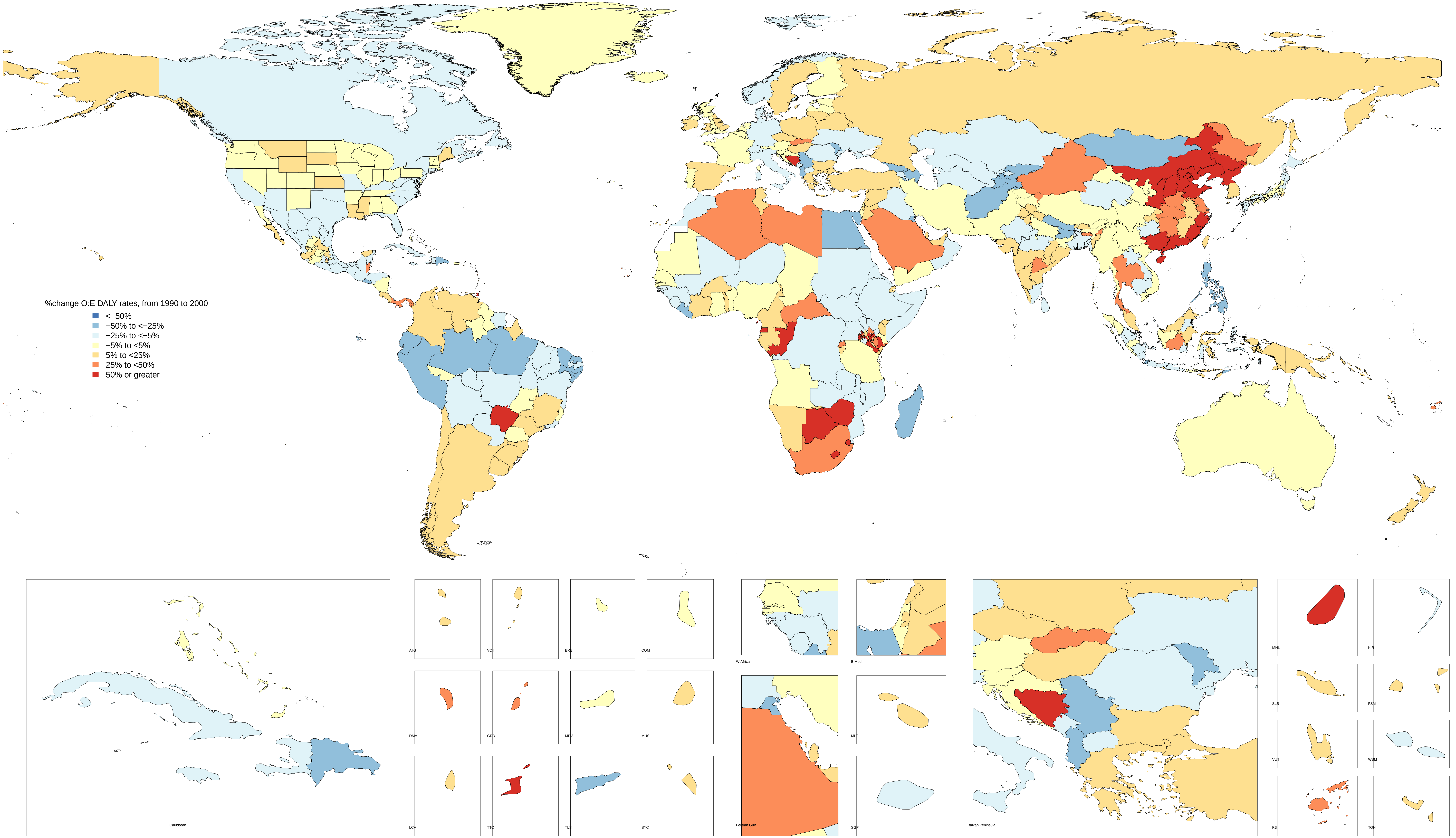

Percent changes in observed to expected disability-adjusted life years (DALYs) are plotted for different time periods and age groups. Panels A-D display changes from 1990 to 2000. Panels E-H display changes from 2000 to 2017. Panels A and E show changes for children less than 1; panels B and F show changes for children between 1 and 4; panels C and G show changes for children between 5 and 9; and panels D and H show changes for children between 10 and 19. For all plots, both sexes are combined. Subnational differentiation occurs within each country GBD models at the subnational level. Inset plots provided for detailed inspection of small or clustered regions. Abbreviations: DALY=disability-adjusted life year, GBD=Global Burden of Disease, ATG = Antigua and Barbuda, DMA = Dominica, LCA = Saint Lucia, VCT = Saint Vincent and the Grenadines, GRD = Grenada, TTO = Trinidad and Tobago, BRB = Barbados, MDV = Maldives, TLS = Timor-Leste, COM = Comoros, MUS = Mauritius, SYC = Seychelles, MLT = Malta, SGP = Singapore, MHL = Marshall Islands, SLB = Solomon Islands, VUT = Vanuatu, FJI = Fiji, KIR = Kiribati, FSM = Federated States of Micronesia, WSM = Samoa, TON = Tonga

eFigure 5c: Map of percent change for observed to expected (O:E) all−cause DALY rates from 1990 to 2000, 2000 to 2017 for <1, 1−4, 5−9, 10−19 years

© 2019 GBD 2017 Child and Adolescent Health Collaborators. JAMA Pediatrics.

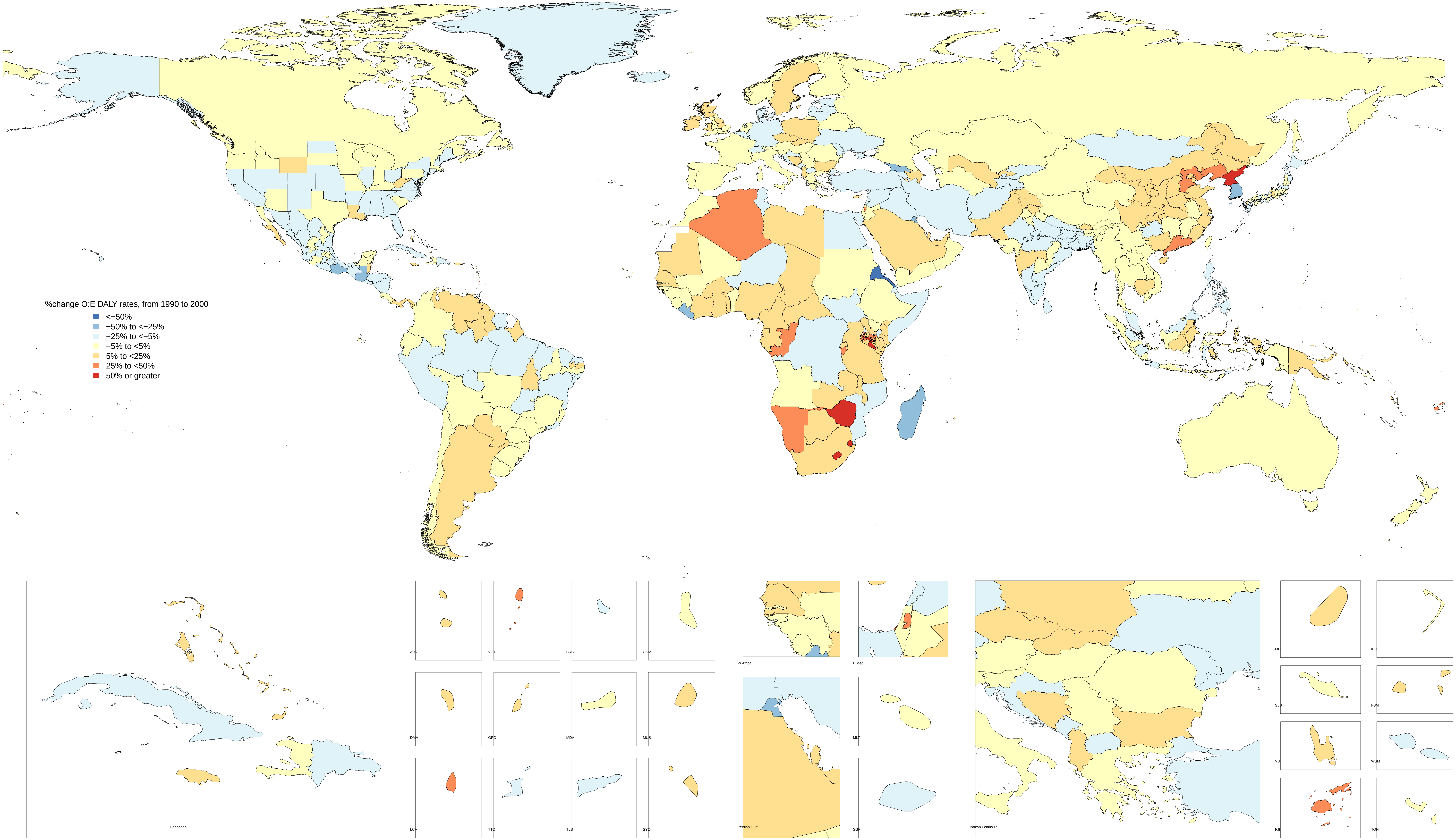

Percent changes in observed to expected disability-adjusted life years (DALYs) are plotted for different time periods and age groups. Panels A-D display changes from 1990 to 2000. Panels E-H display changes from 2000 to 2017. Panels A and E show changes for children less than 1; panels B and F show changes for children between 1 and 4; panels C and G show changes for children between 5 and 9; and panels D and H show changes for children between 10 and 19. For all plots, both sexes are combined. Subnational differentiation occurs within each country GBD models at the subnational level. Inset plots provided for detailed inspection of small or clustered regions. Abbreviations: DALY=disability-adjusted life year, GBD=Global Burden of Disease, ATG = Antigua and Barbuda, DMA = Dominica, LCA = Saint Lucia, VCT = Saint Vincent and the Grenadines, GRD = Grenada, TTO = Trinidad and Tobago, BRB = Barbados, MDV = Maldives, TLS = Timor-Leste, COM = Comoros, MUS = Mauritius, SYC = Seychelles, MLT = Malta, SGP = Singapore, MHL = Marshall Islands, SLB = Solomon Islands, VUT = Vanuatu, FJI = Fiji, KIR = Kiribati, FSM = Federated States of Micronesia, WSM = Samoa, TON = Tonga

eFigure 5d: Map of percent change for observed to expected (O:E) all−cause DALY rates from 1990 to 2000, 2000 to 2017 for <1, 1−4, 5−9, 10−19 years

© 2019 GBD 2017 Child and Adolescent Health Collaborators. JAMA Pediatrics.

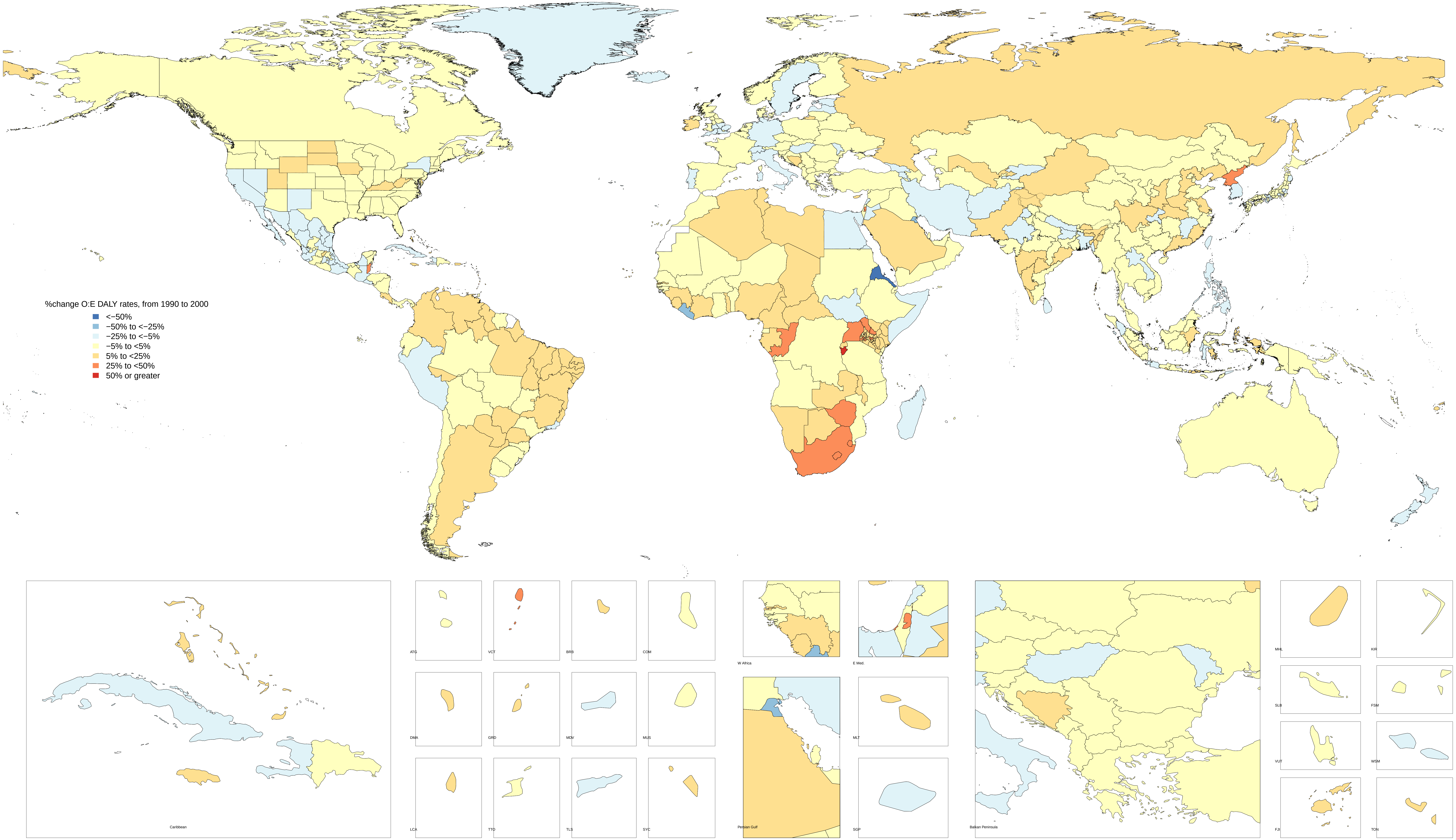

Percent changes in observed to expected disability-adjusted life years (DALYs) are plotted for different time periods and age groups. Panels A-D display changes from 1990 to 2000. Panels E-H display changes from 2000 to 2017. Panels A and E show changes for children less than 1; panels B and F show changes for children between 1 and 4; panels C and G show changes for children between 5 and 9; and panels D and H show changes for children between 10 and 19. For all plots, both sexes are combined. Subnational differentiation occurs within each country GBD models at the subnational level. Inset plots provided for detailed inspection of small or clustered regions. Abbreviations: DALY=disability-adjusted life year, GBD=Global Burden of Disease, ATG = Antigua and Barbuda, DMA = Dominica, LCA = Saint Lucia, VCT = Saint Vincent and the Grenadines, GRD = Grenada, TTO = Trinidad and Tobago, BRB = Barbados, MDV = Maldives, TLS = Timor-Leste, COM = Comoros, MUS = Mauritius, SYC = Seychelles, MLT = Malta, SGP = Singapore, MHL = Marshall Islands, SLB = Solomon Islands, VUT = Vanuatu, FJI = Fiji, KIR = Kiribati, FSM = Federated States of Micronesia, WSM = Samoa, TON = Tonga

eFigure 5e: Map of percent change for observed to expected (O:E) all−cause DALY rates from 1990 to 2000, 2000 to 2017 for <1, 1−4, 5−9, 10−19 years

© 2019 GBD 2017 Child and Adolescent Health Collaborators. JAMA Pediatrics.

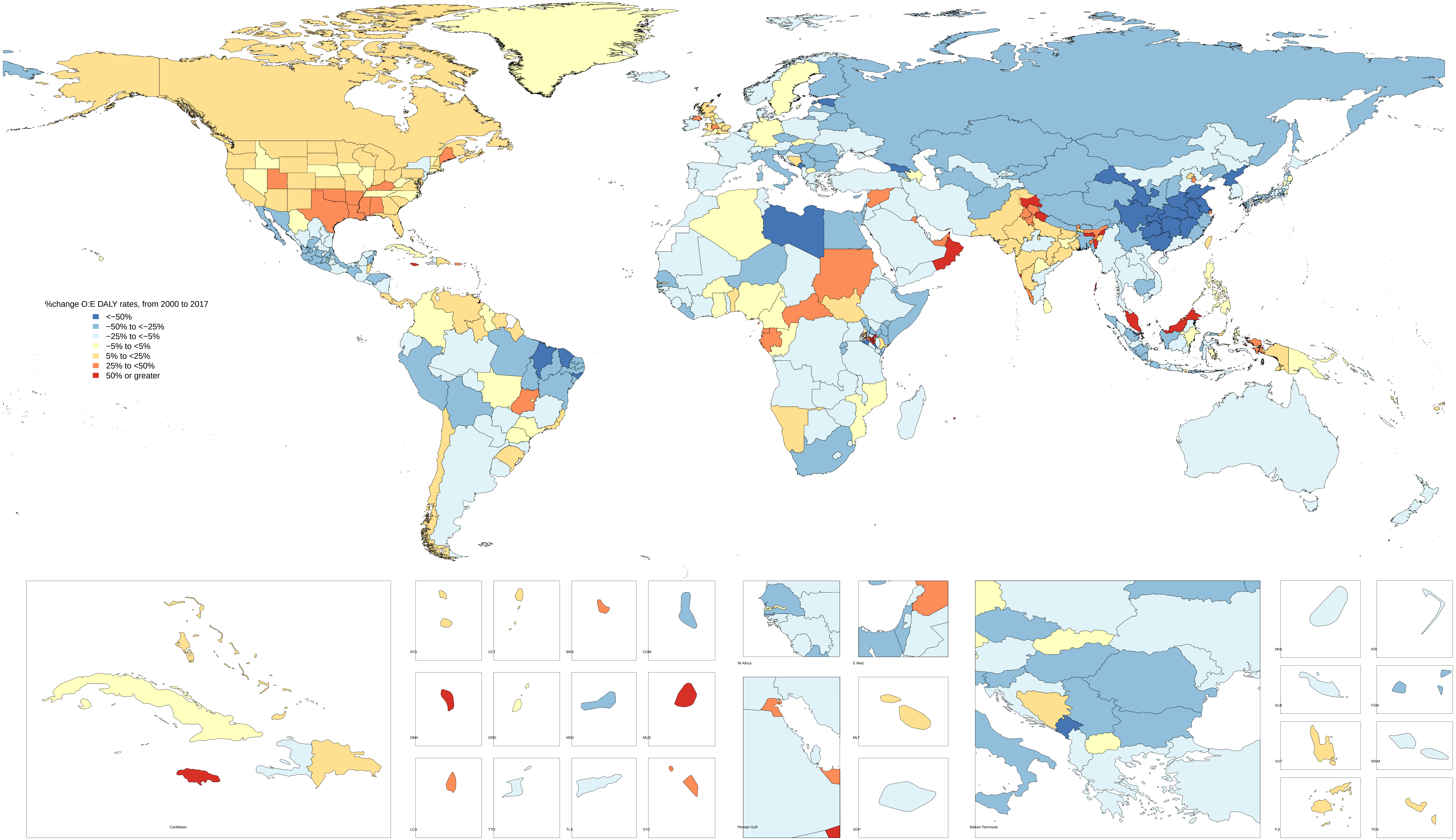

Percent changes in observed to expected disability-adjusted life years (DALYs) are plotted for different time periods and age groups. Panels A-D display changes from 1990 to 2000. Panels E-H display changes from 2000 to 2017. Panels A and E show changes for children less than 1; panels B and F show changes for children between 1 and 4; panels C and G show changes for children between 5 and 9; and panels D and H show changes for children between 10 and 19. For all plots, both sexes are combined. Subnational differentiation occurs within each country GBD models at the subnational level. Inset plots provided for detailed inspection of small or clustered regions. Abbreviations: DALY=disability-adjusted life year, GBD=Global Burden of Disease, ATG = Antigua and Barbuda, DMA = Dominica, LCA = Saint Lucia, VCT = Saint Vincent and the Grenadines, GRD = Grenada, TTO = Trinidad and Tobago, BRB = Barbados, MDV = Maldives, TLS = Timor-Leste, COM = Comoros, MUS = Mauritius, SYC = Seychelles, MLT = Malta, SGP = Singapore, MHL = Marshall Islands, SLB = Solomon Islands, VUT = Vanuatu, FJI = Fiji, KIR = Kiribati, FSM = Federated States of Micronesia, WSM = Samoa, TON = Tonga

eFigure 5f: Map of percent change for observed to expected (O:E) all−cause DALY rates from 1990 to 2000, 2000 to 2017 for <1, 1−4, 5−9, 10−19 years

© 2019 GBD 2017 Child and Adolescent Health Collaborators. JAMA Pediatrics.

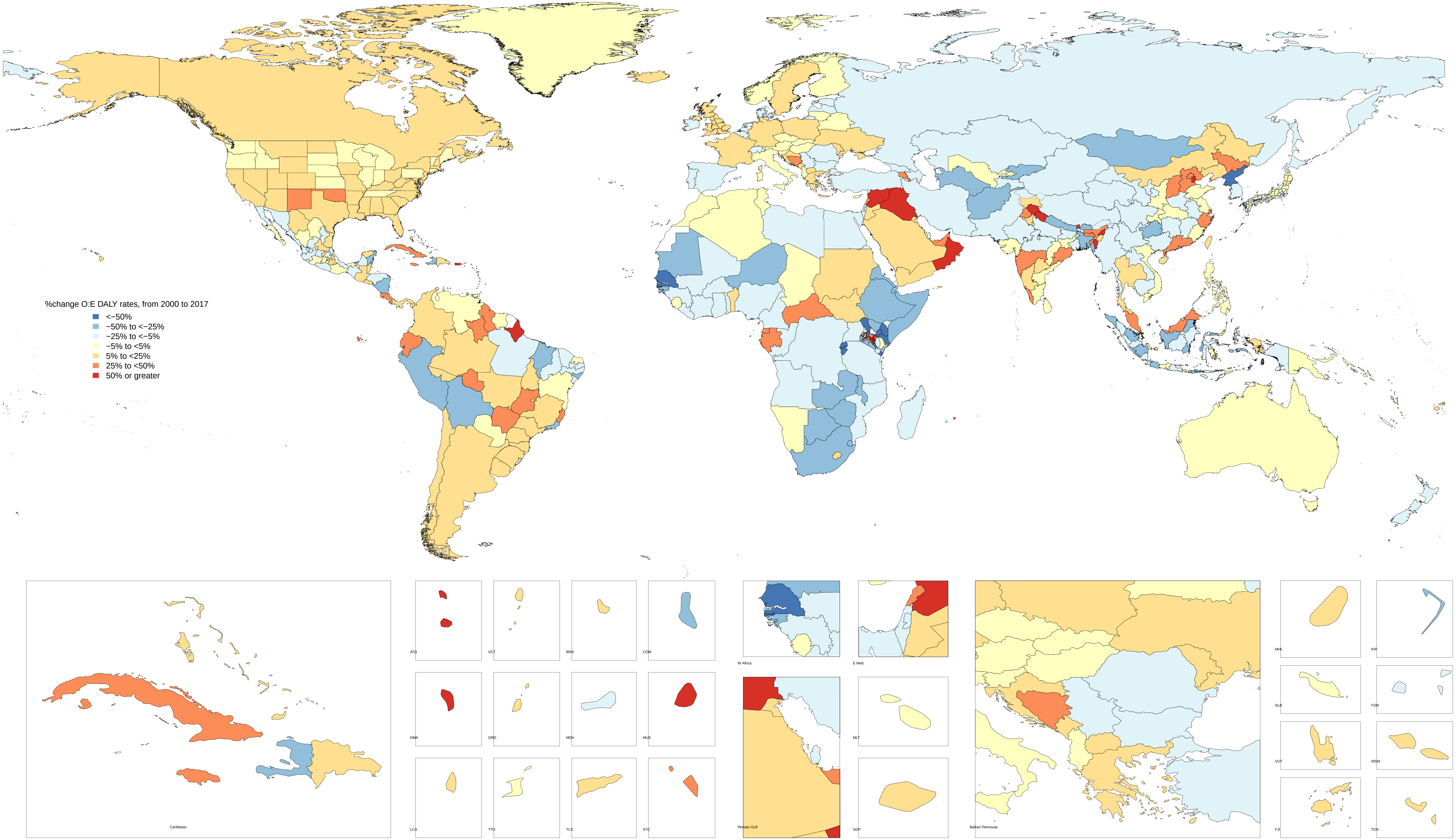

Percent changes in observed to expected disability-adjusted life years (DALYs) are plotted for different time periods and age groups. Panels A-D display changes from 1990 to 2000. Panels E-H display changes from 2000 to 2017. Panels A and E show changes for children less than 1; panels B and F show changes for children between 1 and 4; panels C and G show changes for children between 5 and 9; and panels D and H show changes for children between 10 and 19. For all plots, both sexes are combined. Subnational differentiation occurs within each country GBD models at the subnational level. Inset plots provided for detailed inspection of small or clustered regions. Abbreviations: DALY=disability-adjusted life year, GBD=Global Burden of Disease, ATG = Antigua and Barbuda, DMA = Dominica, LCA = Saint Lucia, VCT = Saint Vincent and the Grenadines, GRD = Grenada, TTO = Trinidad and Tobago, BRB = Barbados, MDV = Maldives, TLS = Timor-Leste, COM = Comoros, MUS = Mauritius, SYC = Seychelles, MLT = Malta, SGP = Singapore, MHL = Marshall Islands, SLB = Solomon Islands, VUT = Vanuatu, FJI = Fiji, KIR = Kiribati, FSM = Federated States of Micronesia, WSM = Samoa, TON = Tonga

eFigure 5g: Map of percent change for observed to expected (O:E) all−cause DALY rates from 1990 to 2000, 2000 to 2017 for <1, 1−4, 5−9, 10−19 years

© 2019 GBD 2017 Child and Adolescent Health Collaborators. JAMA Pediatrics.

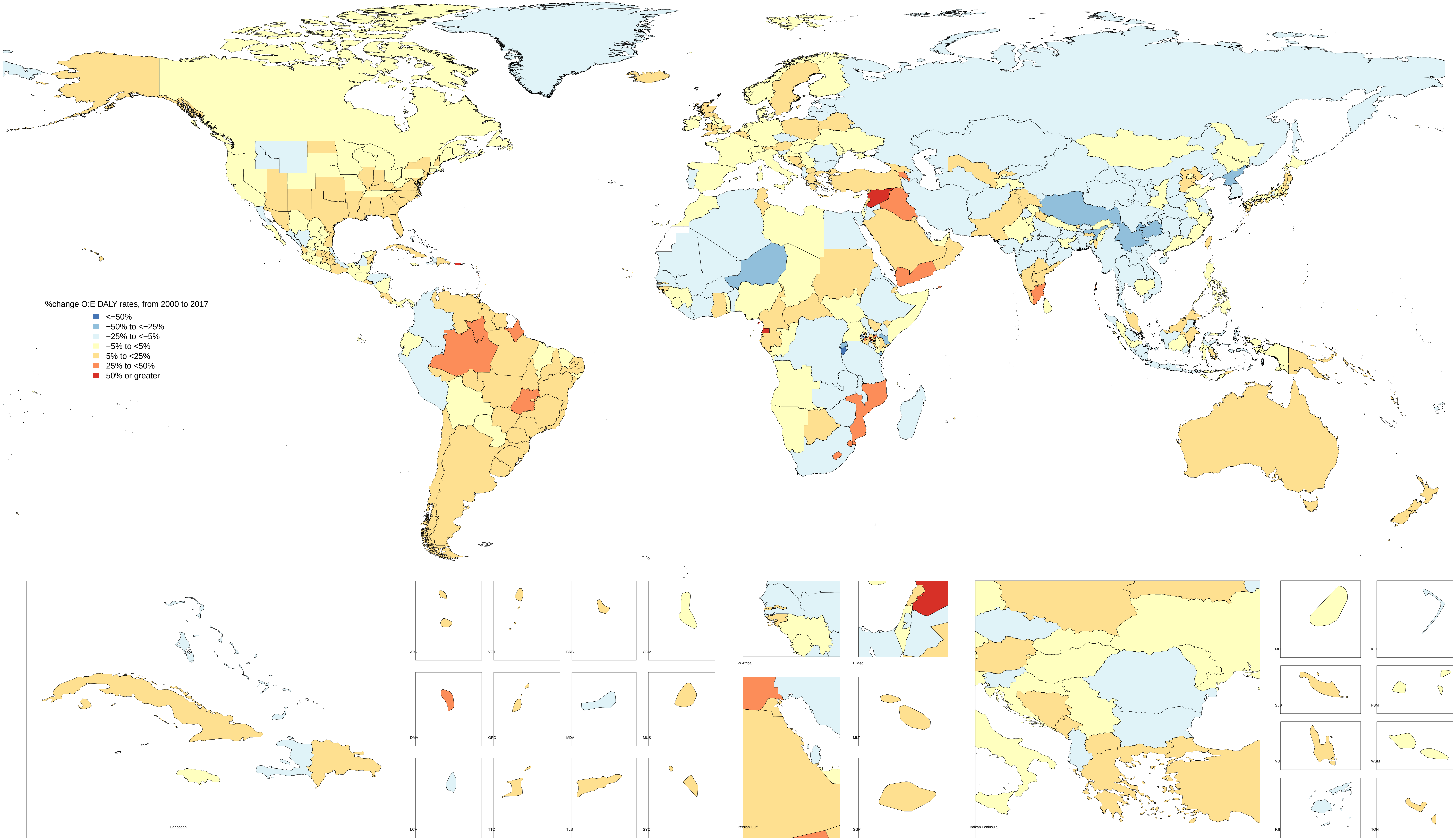

Percent changes in observed to expected disability-adjusted life years (DALYs) are plotted for different time periods and age groups. Panels A-D display changes from 1990 to 2000. Panels E-H display changes from 2000 to 2017. Panels A and E show changes for children less than 1; panels B and F show changes for children between 1 and 4; panels C and G show changes for children between 5 and 9; and panels D and H show changes for children between 10 and 19. For all plots, both sexes are combined. Subnational differentiation occurs within each country GBD models at the subnational level. Inset plots provided for detailed inspection of small or clustered regions. Abbreviations: DALY=disability-adjusted life year, GBD=Global Burden of Disease, ATG = Antigua and Barbuda, DMA = Dominica, LCA = Saint Lucia, VCT = Saint Vincent and the Grenadines, GRD = Grenada, TTO = Trinidad and Tobago, BRB = Barbados, MDV = Maldives, TLS = Timor-Leste, COM = Comoros, MUS = Mauritius, SYC = Seychelles, MLT = Malta, SGP = Singapore, MHL = Marshall Islands, SLB = Solomon Islands, VUT = Vanuatu, FJI = Fiji, KIR = Kiribati, FSM = Federated States of Micronesia, WSM = Samoa, TON = Tonga

eFigure 5h: Map of percent change for observed to expected (O:E) all−cause DALY rates from 1990 to 2000, 2000 to 2017 for <1, 1−4, 5−9, 10−19 years

© 2019 GBD 2017 Child and Adolescent Health Collaborators. JAMA Pediatrics.

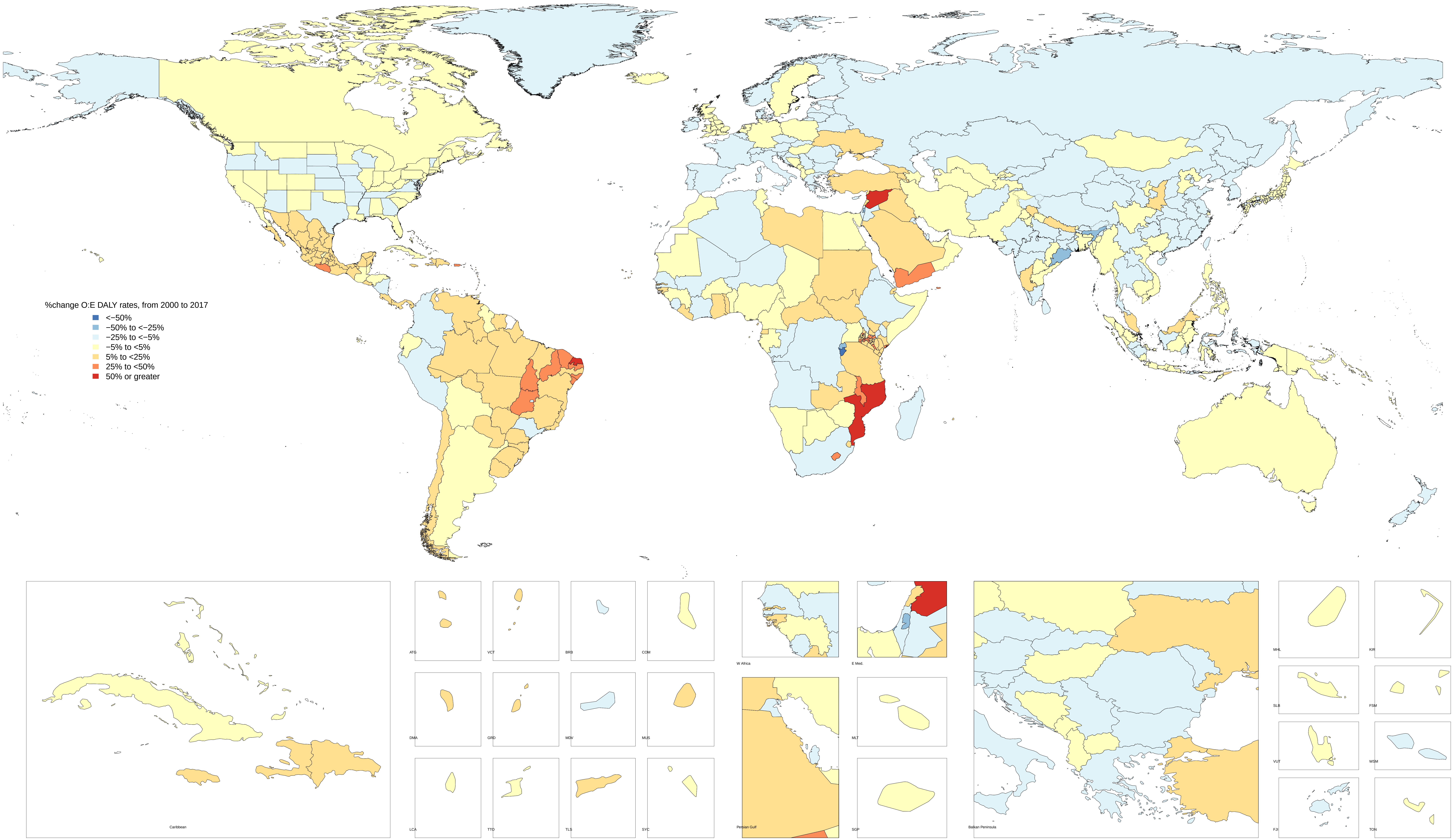

Percent changes in observed to expected disability-adjusted life years (DALYs) are plotted for different time periods and age groups. Panels A-D display changes from 1990 to 2000. Panels E-H display changes from 2000 to 2017. Panels A and E show changes for children less than 1; panels B and F show changes for children between 1 and 4; panels C and G show changes for children between 5 and 9; and panels D and H show changes for children between 10 and 19. For all plots, both sexes are combined. Subnational differentiation occurs within each country GBD models at the subnational level. Inset plots provided for detailed inspection of small or clustered regions. Abbreviations: DALY=disability-adjusted life year, GBD=Global Burden of Disease, ATG = Antigua and Barbuda, DMA = Dominica, LCA = Saint Lucia, VCT = Saint Vincent and the Grenadines, GRD = Grenada, TTO = Trinidad and Tobago, BRB = Barbados, MDV = Maldives, TLS = Timor-Leste, COM = Comoros, MUS = Mauritius, SYC = Seychelles, MLT = Malta, SGP = Singapore, MHL = Marshall Islands, SLB = Solomon Islands, VUT = Vanuatu, FJI = Fiji, KIR = Kiribati, FSM = Federated States of Micronesia, WSM = Samoa, TON = Tonga

© 2019 GBD 2017 Child and Adolescent Health Collaborators. JAMA Pediatrics.

eFigure 6a. Annual percent change of observed to expected (O:E) all−cause DALY rates in <20 years from 1990 to 2000 versus 2000 to 2017

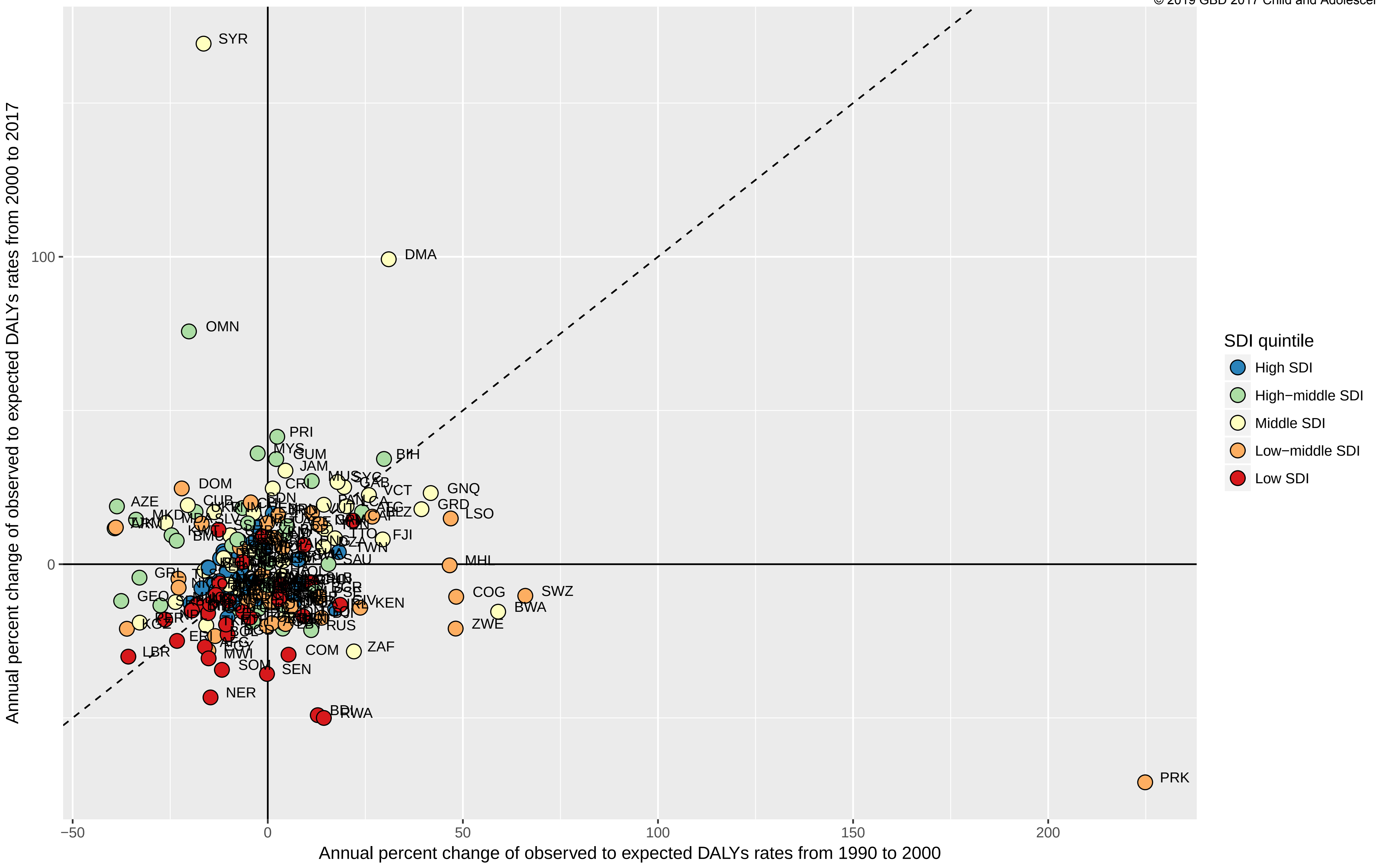

eFigure 6a. Annual percent change of observed to expected (O:E) all−cause DALY rates in <20 years from 1990 to 2000 versus 2000 to 2017 (graph subsetted to +/− 50%)

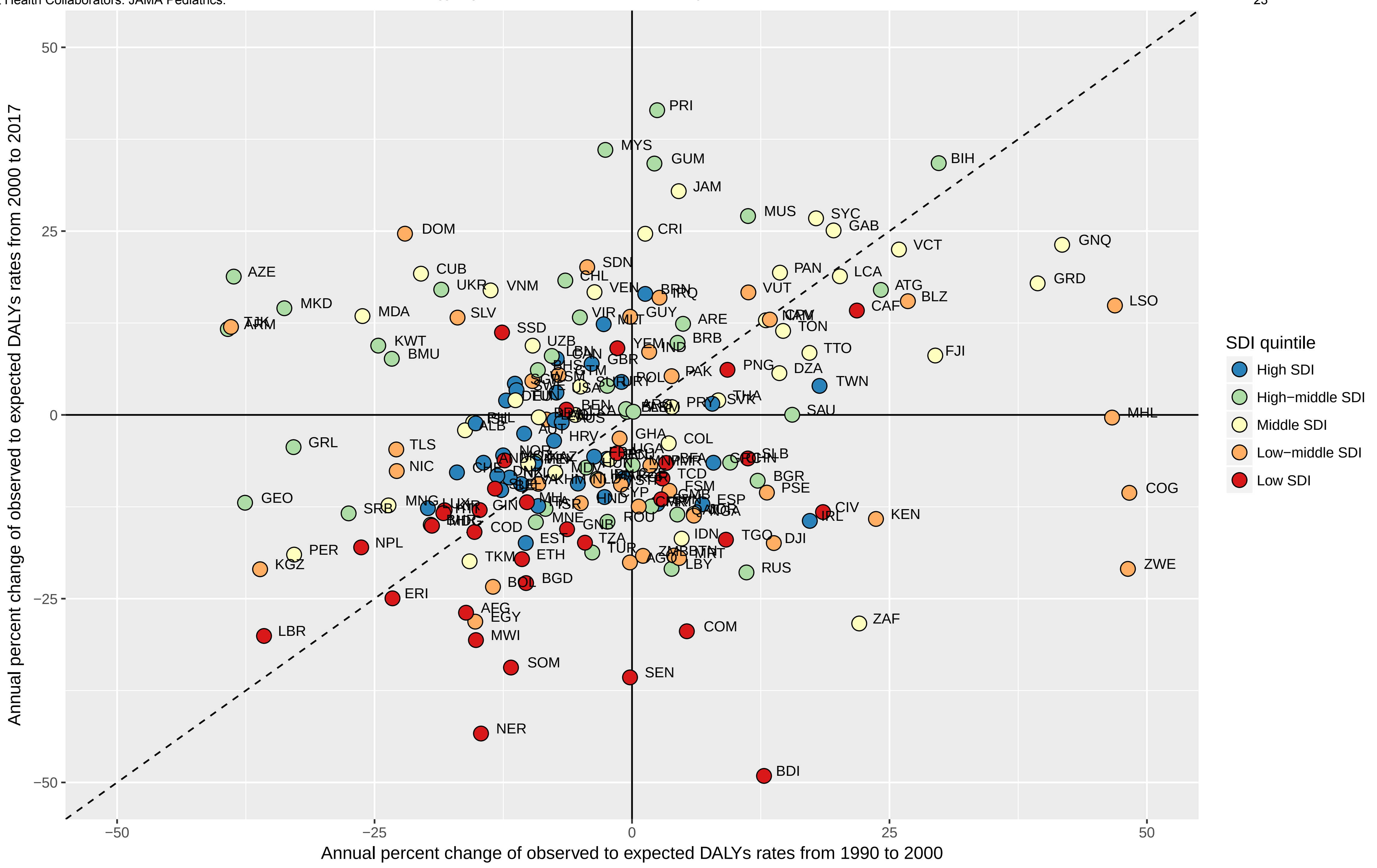

Annual percent change of observed versus expected (O:E) disability−adjusted life years (DALYs) rates by country for 1990 to 2000 versus 2000 to 2017 are plotted by country. In each pair of panels, the right panel focuses on changes within +/−50%. Panels a–d plot changes for all children and adolescents less than 20 years of age; panels e–h plot changes for children less than 1; panels i–l plot changes for children between 1 and 4; panels m–p plot changes for children between 5 and 9; and panels q–t plot changes for children between 10 and 19. For each age group, the first plot in the series is the all cause change in O:E rates (i.e., panels a, e, i, m, and q); the second plot in the series is the change in O:E rates associated with communicable, maternal, neonatal, and nutritional (CMNN) conditions (i.e., panels b, f, j, and r); the third plot in the series is the change in O:E rates associated with non−communicable diseases (NCDs – i.e., panels c, g, k, s); and the fourth plot in the series is the change in O:E rates associated with injuries (i.e., panels d, h, l, t). For each, countries in different socio−demographic index (SDI) quintiles are plotted with different countries. Abbreviations: DALY=disability−adjusted life year, AFG − Afghanistan, AGO − Angola, ALB − Albania, AND − Andorra, ARE − United Arab Emirates, ARG − Argentina, ARM − Armenia, ASM − American Samoa, ATG − Antigua and Barbuda, AUS − Australia, AUT − Austria, AZE − Azerbaijan, BDI − Burundi, BEL − Belgium, BEN − Benin, BFA − Burkina Faso, BGD − Bangladesh, BGR − Bulgaria, BHR − Bahrain, BHS − The Bahamas, BIH − Bosnia and Herzegovina, BLR − Belarus, BLZ − Belize, BMU − Bermuda, BOL − Bolivia, BRA − Brazil, BRB − Barbados, BRN − Brunei, BTN − Bhutan, BWA − Botswana, CAF − Central African Republic, CAN − Canada, CHE − Switzerland, CHL − Chile, CHN − China, CIV − Cote d'Ivoire, CMR − Cameroon, COD − Democratic Republic of the Congo, COG − Congo, COL − Colombia, COM − Comoros, CPV − Cape Verde, CRI − Costa Rica, CUB − Cuba, CYP − Cyprus, CZE − Czech Republic, DEU − Germany, DJI − Djibouti, DMA − Dominica, DNK − Denmark, DOM − Dominican Republic, DZA − Algeria, ECU − Ecuador, EGY − Egypt, ERI − Eritrea, ESP − Spain, EST − Estonia, ETH − Ethiopia, FIN − Finland, FJI − Fiji, FRA − France, FSM − Federated States of Micronesia, G − Global, GAB − Gabon, GBR − United Kingdom, GEO − Georgia, GHA − Ghana, GIN − Guinea, GMB − The Gambia, GNB − Guinea−Bissau, GNQ − Equatorial Guinea, GRC − Greece, GRD − Grenada, GRL − Greenland, GTM − Guatemala, GUM − Guam, GUY − Guyana, HND − Honduras, HRV − Croatia, HTI − Haiti, HUN − Hungary, IDN − Indonesia, IND − India, IRL − Ireland, IRN − Iran, IRQ − Iraq, ISL − Iceland, ISR − Israel, ITA − Italy, JAM − Jamaica, JOR − Jordan, JPN − Japan, KAZ − Kazakhstan, KEN − Kenya, KGZ − Kyrgyzstan, KHM − Cambodia, KIR − Kiribati, KOR − South Korea, KWT − Kuwait, LAO − Laos, LBN − Lebanon, LBR − Liberia, LBY − Libya, LCA − Saint Lucia, LKA − Sri Lanka, LSO − Lesotho, LTU − Lithuania, LUX − Luxembourg, LVA − Latvia, MAR − Morocco, MDA − Moldova, MDG − Madagascar, MDV − Maldives, MEX − Mexico, MHL − Marshall Islands, MKD − Macedonia, MLI − Mali, MLT − Malta, MMR − Myanmar, MNE − Montenegro, MNG − Mongolia, MNP − Northern Mariana Islands, MOZ − Mozambique, MRT − Mauritania, MUS − Mauritius, MWI − Malawi, MYS − Malaysia, NAM − Namibia, NER − Niger, NGA − Nigeria, NIC − Nicaragua, NLD − Netherlands, NOR − Norway, NPL − Nepal, NZL − New Zealand, OMN − Oman, PAK − Pakistan, PAN − Panama, PER − Peru, PHL − Philippines, PNG − Papua New Guinea, POL − Poland, PRI − Puerto Rico, PRK − North Korea, PRT − Portugal, PRY − Paraguay, PSE − Palestine, QAT − Qatar, ROU − Romania, RUS − Russian Federation, RWA − Rwanda, SAU − Saudi Arabia, SDN − Sudan, SEN − Senegal, SGP − Singapore, SLB − Solomon Islands, SLE − Sierra Leone, SLV − El Salvador, SOM − Somalia, SRB − Serbia, SSD − South Sudan, STP − Sao Tome and Principe, SUR − Suriname, SVK − Slovakia, SVN − Slovenia, SWE − Sweden, SWZ − Swaziland, SYC − Seychelles, SYR − Syria, TCD − Chad, TGO − Togo, THA − Thailand, TJK − Tajikistan, TKM − Turkmenistan, TLS − Timor−Leste, TON − Tonga, TTO − Trinidad and Tobago, TUN − Tunisia, TUR − Turkey, TWN − Taiwan, TZA − Tanzania, UGA − Uganda, UKR − Ukraine, URY − Uruguay, USA − United States, UZB − Uzbekistan, VCT − Saint Vincent and the Grenadines, VEN − Venezuela, VIR − Virgin Islands, U.S., VNM − Vietnam, VUT − Vanuatu, WSM − Samoa, YEM − Yemen, ZAF − South Africa, ZMB − Zambia, ZWE − Zimbabwe

eFigure 6b. Annual percent change of observed to expected (O:E) all−cause DALY rates in <20 years from 1990 to 2000 versus 2000 to 2017

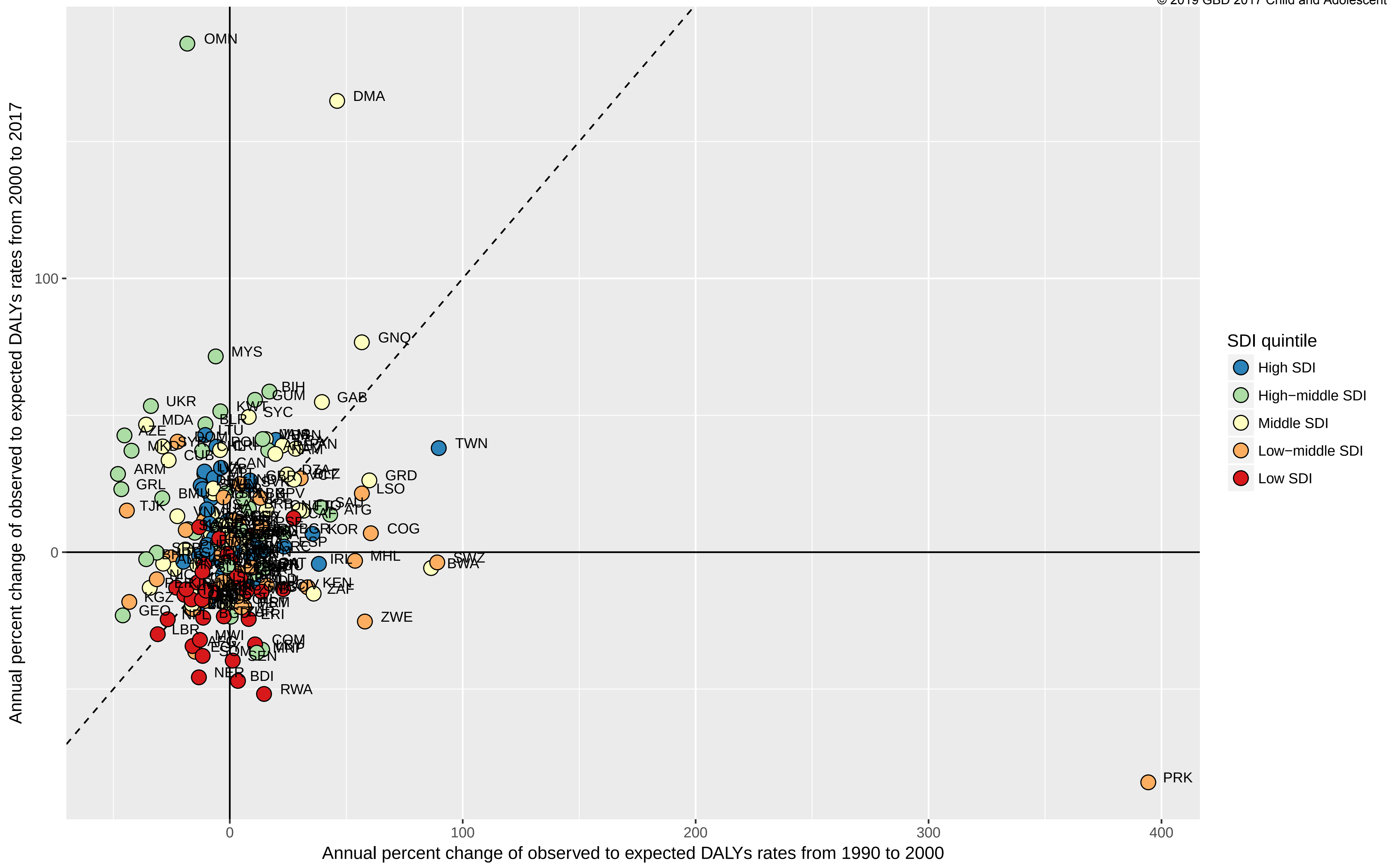

eFigure 6b. Annual percent change of observed to expected (O:E) all−cause DALY rates in <20 years from 1990 to 2000 versus 2000 to 2017 (graph subsetted to +/− 50%)

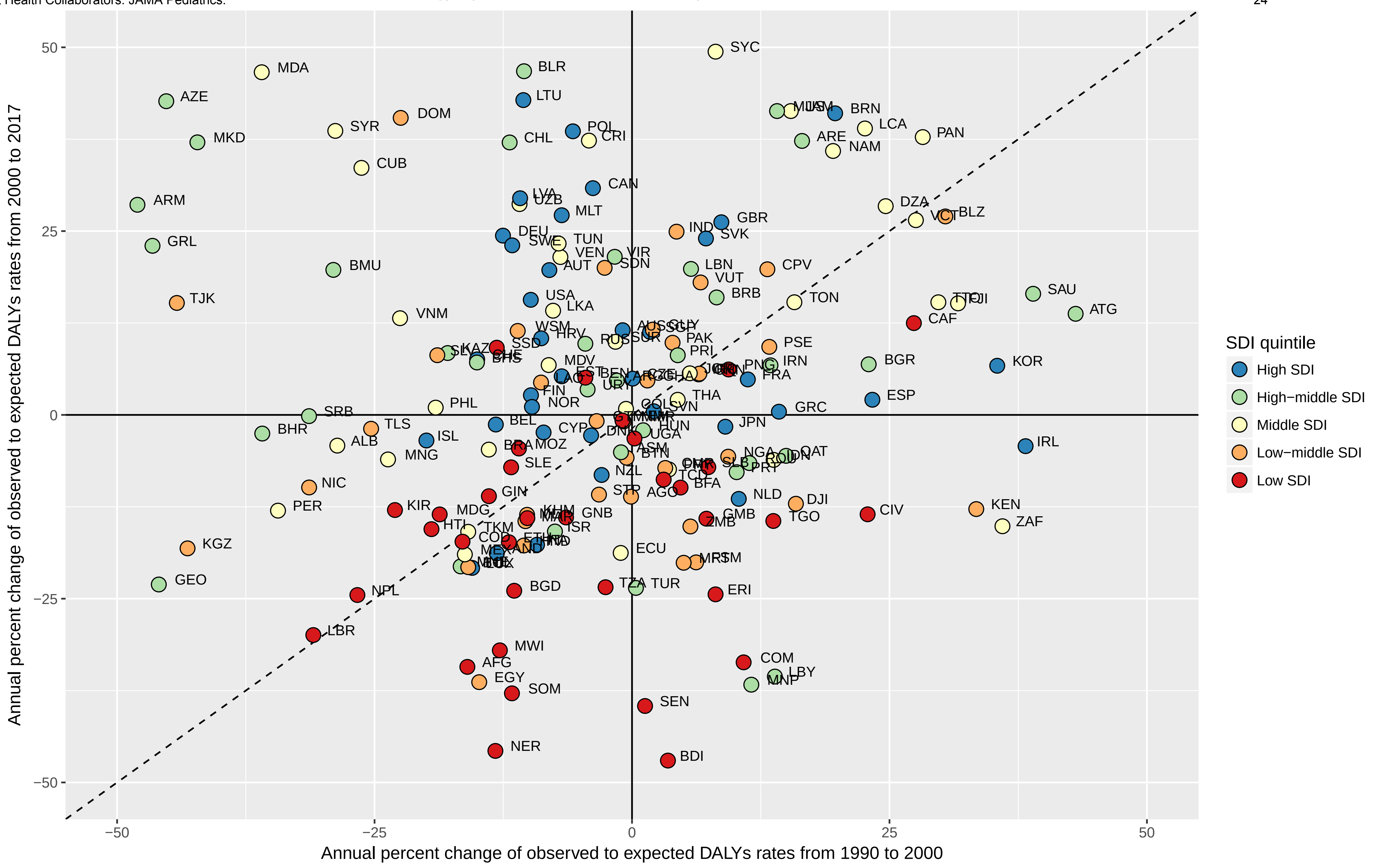

Annual percent change of observed versus expected (O:E) disability−adjusted life years (DALYs) rates by country for 1990 to 2000 versus 2000 to 2017 are plotted by country. In each pair of panels, the right panel focuses on changes within +/−50%. Panels a−d plot changes for all children and adolescents less than 20 years of age; panels e−h plot changes for children less than 1; panels i−l plot changes for children between 1 and 4; panels m−p plot changes for children between 5 and 9; and panels q−t plot changes for children between 10 and 19. For each age group, the first plot in the series is the all cause change in O:E rates (i.e., panels a, e, i, m, and q); the second plot in the series is the change in O:E rates associated with communicable, maternal, neonatal, and nutritional (CMNN) conditions (i.e., panels b, f, j, and r); the third plot in the series is the change in O:E rates associated with non−communicable diseases (NCDs – i.e., panels c, g, k, s); and the fourth plot in the series is the change in O:E rates associated with injuries (i.e., panels d, h, l, t). For each, countries in different socio−demographic index (SDI) quintiles are plotted with different countries. Abbreviations: DALY=disability−adjusted life year, AFG − Afghanistan, AGO − Angola, ALB − Albania, AND − Andorra, ARE − United Arab Emirates, ARG − Argentina, ARM − Armenia, ASM − American Samoa, ATG − Antigua and Barbuda, AUS − Australia, AUT − Austria, AZE − Azerbaijan, BDI − Burundi, BEL − Belgium, BEN − Benin, BFA − Burkina Faso, BGD − Bangladesh, BGR − Bulgaria, BHR − Bahrain, BHS − The Bahamas, BIH − Bosnia and Herzegovina, BLR − Belarus, BLZ − Belize, BMU − Bermuda, BOL − Bolivia, BRA − Brazil, BRB − Barbados, BRN − Brunei, BTN − Bhutan, BWA − Botswana, CAF − Central African Republic, CAN − Canada, CHE − Switzerland, CHL − Chile, CHN − China, CIV − Cote d'Ivoire, CMR − Cameroon, COD − Democratic Republic of the Congo, COG − Congo, COL − Colombia, COM − Comoros, CPV − Cape Verde, CRI − Costa Rica, CUB − Cuba, CYP − Cyprus, CZE − Czech Republic, DEU − Germany, DJI − Djibouti, DMA − Dominica, DNK − Denmark, DOM − Dominican Republic, DZA − Algeria, ECU − Ecuador, EGY − Egypt, ERI − Eritrea, ESP − Spain, EST − Estonia, ETH − Ethiopia, FIN − Finland, FJI − Fiji, FRA − France, FSM − Federated States of Micronesia, G − Global, GAB − Gabon, GBR − United Kingdom, GEO − Georgia, GHA − Ghana, GIN − Guinea, GMB − The Gambia, GNB − Guinea−Bissau, GNQ − Equatorial Guinea, GRC − Greece, GRD − Grenada, GRL − Greenland, GTM − Guatemala, GUM − Guam, GUY − Guyana, HND − Honduras, HRV − Croatia, HTI − Haiti, HUN − Hungary, IDN − Indonesia, IND − India, IRL − Ireland, IRN − Iran, IRQ − Iraq, ISL − Iceland, ISR − Israel, ITA − Italy, JAM − Jamaica, JOR − Jordan, JPN − Japan, KAZ − Kazakhstan, KEN − Kenya, KGZ − Kyrgyzstan, KHM − Cambodia, KIR − Kiribati, KOR − South Korea, KWT − Kuwait, LAO − Laos, LBN − Lebanon, LBR − Liberia, LBY − Libya, LCA − Saint Lucia, LKA − Sri Lanka, LSO − Lesotho, LTU − Lithuania, LUX − Luxembourg, LVA − Latvia, MAR − Morocco, MDA − Moldova, MDG − Madagascar, MDV − Maldives, MEX − Mexico, MHL − Marshall Islands, MKD − Macedonia, MLI − Mali, MLT − Malta, MMR − Myanmar, MNE − Montenegro, MNG − Mongolia, MNP − Northern Mariana Islands, MOZ − Mozambique, MRT − Mauritania, MUS − Mauritius, MWI − Malawi, MYS − Malaysia, NAM − Namibia, NER − Niger, NGA − Nigeria, NIC − Nicaragua, NLD − Netherlands, NOR − Norway, NPL − Nepal, NZL − New Zealand, OMN − Oman, PAK − Pakistan, PAN − Panama, PER − Peru, PHL − Philippines, PNG − Papua New Guinea, POL − Poland, PRI − Puerto Rico, PRK − North Korea, PRT − Portugal, PRY − Paraguay, PSE − Palestine, QAT − Qatar, ROU − Romania, RUS − Russian Federation, RWA − Rwanda, SAU − Saudi Arabia, SDN − Sudan, SEN − Senegal, SGP − Singapore, SLB − Solomon Islands, SLE − Sierra Leone, SLV − El Salvador, SOM − Somalia, SRB − Serbia, SSD − South Sudan, STP − Sao Tome and Principe, SUR − Suriname, SVK − Slovakia, SVN − Slovenia, SWE − Sweden, SWZ − Swaziland, SYC − Seychelles, SYR − Syria, TCD − Chad, TGO − Togo, THA − Thailand, TJK − Tajikistan, TKM − Turkmenistan, TLS − Timor−Leste, TON − Tonga, TTO − Trinidad and Tobago, TUN − Tunisia, TUR − Turkey, TWN − Taiwan, TZA − Tanzania, UGA − Uganda, UKR − Ukraine, URY − Uruguay, USA − United States, UZB − Uzbekistan, VCT − Saint Vincent and the Grenadines, VEN − Venezuela, VIR − Virgin Islands, U.S., VNM − Vietnam, VUT − Vanuatu, WSM − Samoa, YEM − Yemen, ZAF − South Africa, ZMB − Zambia, ZWE − Zimbabwe

eFigure 6c. Annual percent change of observed to expected (O:E) all−cause DALY rates in <20 years from 1990 to 2000 versus 2000 to 2017

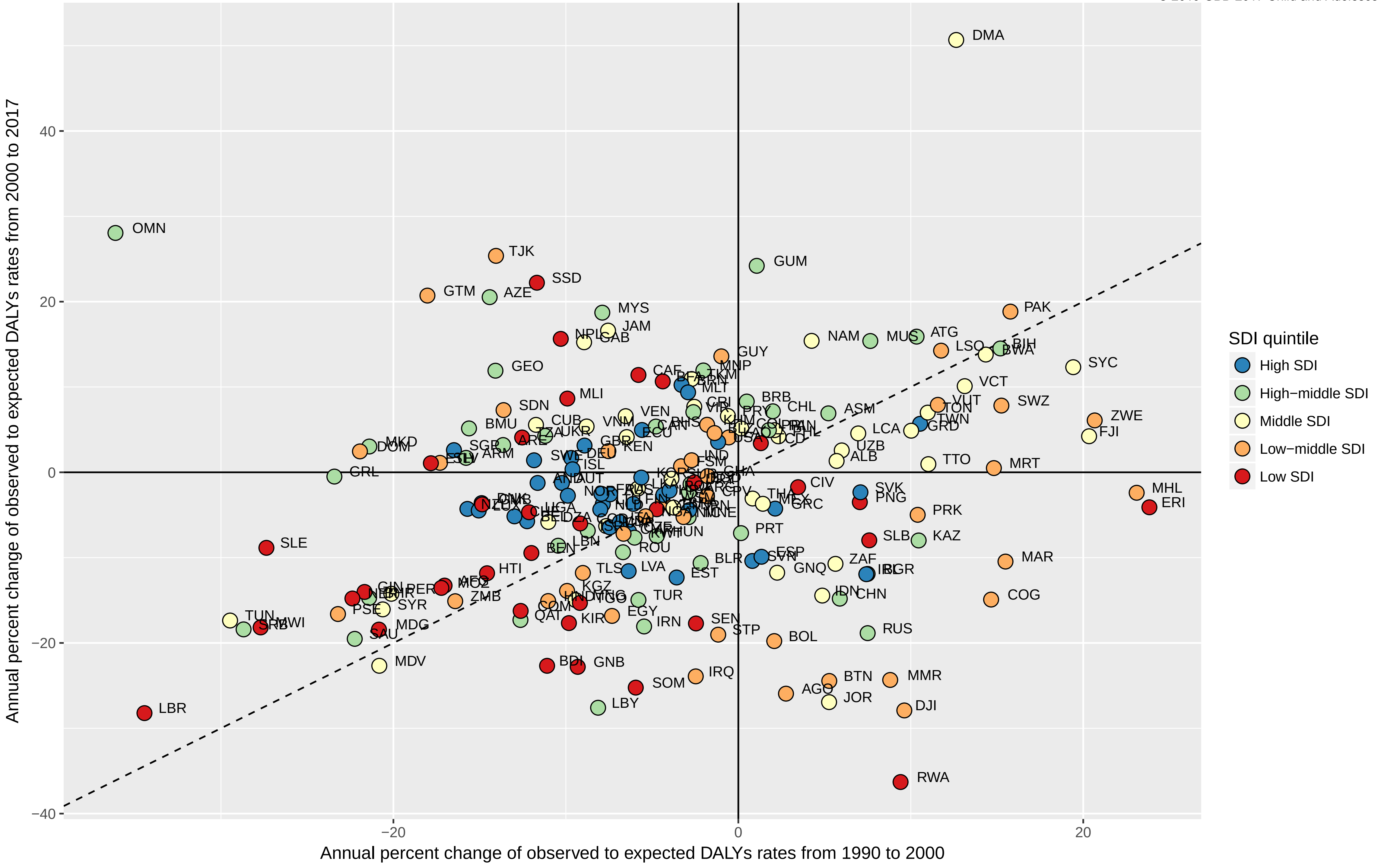

eFigure 6c. Annual percent change of observed to expected (O:E) all−cause DALY rates in <20 years from 1990 to 2000 versus 2000 to 2017 (graph subsetted to +/− 50%)

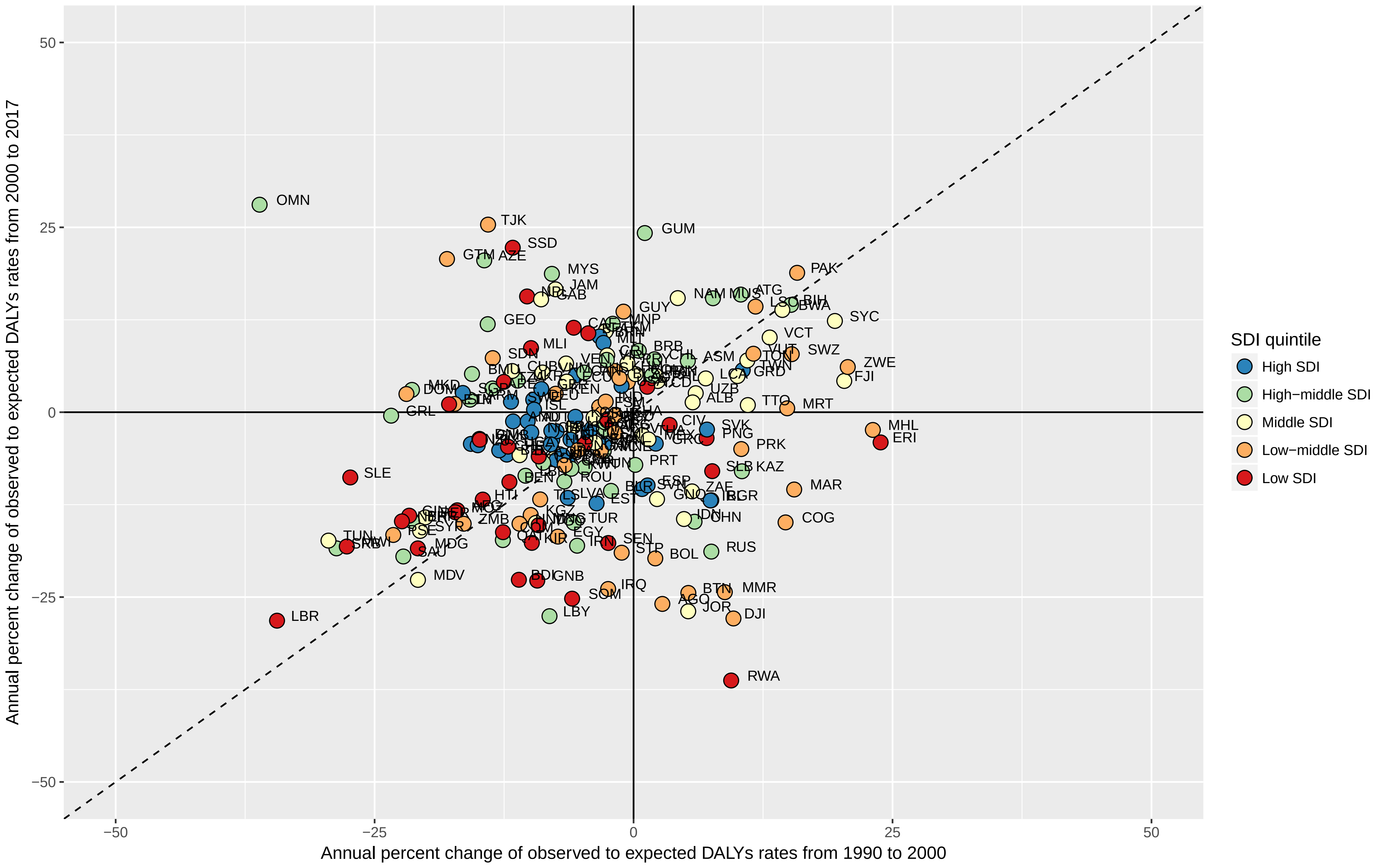

Annual percent change of observed versus expected (O:E) disability−adjusted life years (DALYs) rates by country for 1990 to 2000 versus 2000 to 2017 are plotted by country. In each pair of panels, the right panel focuses on changes within +/−50%. Panels a−d plot changes for all children and adolescents less than 20 years of age; panels e−h plot changes for children less than 1; panels i−l plot changes for children between 1 and 4; panels m−p plot changes for children between 5 and 9; and panels q−t plot changes for children between 10 and 19. For each age group, the first plot in the series is the all cause change in O:E rates (i.e., panels a, e, i, m, and q); the second plot in the series is the change in O:E rates associated with communicable, maternal, neonatal, and nutritional (CMNN) conditions (i.e., panels b, f, j, and r); the third plot in the series is the change in O:E rates associated with non−communicable diseases (NCDs – i.e., panels c, g, k, s); and the fourth plot in the series is the change in O:E rates associated with injuries (i.e., panels d, h, l, t). For each, countries in different socio−demographic index (SDI) quintiles are plotted with different countries. Abbreviations: DALY=disability−adjusted life year, AFG − Afghanistan, AGO − Angola, ALB − Albania, AND − Andorra, ARE − United Arab Emirates, ARG − Argentina, ARM − Armenia, ASM − American Samoa, ATG − Antigua and Barbuda, AUS − Australia, AUT − Austria, AZE − Azerbaijan, BDI − Burundi, BEL − Belgium, BEN − Benin, BFA − Burkina Faso, BGD − Bangladesh, BGR − Bulgaria, BHR − Bahrain, BHS − The Bahamas, BIH − Bosnia and Herzegovina, BLR − Belarus, BLZ − Belize, BMU − Bermuda, BOL − Bolivia, BRA − Brazil, BRB − Barbados, BRN − Brunei, BTN − Bhutan, BWA − Botswana, CAF − Central African Republic, CAN − Canada, CHE − Switzerland, CHL − Chile, CHN − China, CIV − Cote d'Ivoire, CMR − Cameroon, COD − Democratic Republic of the Congo, COG − Congo, COL − Colombia, COM − Comoros, CPV − Cape Verde, CRI − Costa Rica, CUB − Cuba, CYP − Cyprus, CZE − Czech Republic, DEU − Germany, DJI − Djibouti, DMA − Dominica, DNK − Denmark, DOM − Dominican Republic, DZA − Algeria, ECU − Ecuador, EGY − Egypt, ERI − Eritrea, ESP − Spain, EST − Estonia, ETH − Ethiopia, FIN − Finland, FJI − Fiji, FRA − France, FSM − Federated States of Micronesia, G − Global, GAB − Gabon, GBR − United Kingdom, GEO − Georgia, GHA − Ghana, GIN − Guinea, GMB − The Gambia, GNB − Guinea−Bissau, GNQ − Equatorial Guinea, GRC − Greece, GRD − Grenada, GRL − Greenland, GTM − Guatemala, GUM − Guam, GUY − Guyana, HND − Honduras, HRV − Croatia, HTI − Haiti, HUN − Hungary, IDN − Indonesia, IND − India, IRL − Ireland, IRN − Iran, IRQ − Iraq, ISL − Iceland, ISR − Israel, ITA − Italy, JAM − Jamaica, JOR − Jordan, JPN − Japan, KAZ − Kazakhstan, KEN − Kenya, KGZ − Kyrgyzstan, KHM − Cambodia, KIR − Kiribati, KOR − South Korea, KWT − Kuwait, LAO − Laos, LBN − Lebanon, LBR − Liberia, LBY − Libya, LCA − Saint Lucia, LKA − Sri Lanka, LSO − Lesotho, LTU − Lithuania, LUX − Luxembourg, LVA − Latvia, MAR − Morocco, MDA − Moldova, MDG − Madagascar, MDV − Maldives, MEX − Mexico, MHL − Marshall Islands, MKD − Macedonia, MLI − Mali, MLT − Malta, MMR − Myanmar, MNE − Montenegro, MNG − Mongolia, MNP − Northern Mariana Islands, MOZ − Mozambique, MRT − Mauritania, MUS − Mauritius, MWI − Malawi, MYS − Malaysia, NAM − Namibia, NER − Niger, NGA − Nigeria, NIC − Nicaragua, NLD − Netherlands, NOR − Norway, NPL − Nepal, NZL − New Zealand, OMN − Oman, PAK − Pakistan, PAN − Panama, PER − Peru, PHL − Philippines, PNG − Papua New Guinea, POL − Poland, PRI − Puerto Rico, PRK − North Korea, PRT − Portugal, PRY − Paraguay, PSE − Palestine, QAT − Qatar, ROU − Romania, RUS − Russian Federation, RWA − Rwanda, SAU − Saudi Arabia, SDN − Sudan, SEN − Senegal, SGP − Singapore, SLB − Solomon Islands, SLE − Sierra Leone, SLV − El Salvador, SOM − Somalia, SRB − Serbia, SSD − South Sudan, STP − Sao Tome and Principe, SUR − Suriname, SVK − Slovakia, SVN − Slovenia, SWE − Sweden, SWZ − Swaziland, SYC − Seychelles, SYR − Syria, TCD − Chad, TGO − Togo, THA − Thailand, TJK − Tajikistan, TKM − Turkmenistan, TLS − Timor−Leste, TON − Tonga, TTO − Trinidad and Tobago, TUN − Tunisia, TUR − Turkey, TWN − Taiwan, TZA − Tanzania, UGA − Uganda, UKR − Ukraine, URY − Uruguay, USA − United States, UZB − Uzbekistan, VCT − Saint Vincent and the Grenadines, VEN − Venezuela, VIR − Virgin Islands, U.S., VNM − Vietnam, VUT − Vanuatu, WSM − Samoa, YEM − Yemen, ZAF − South Africa, ZMB − Zambia, ZWE − Zimbabwe

© 2019 GBD 2017 Child and Adolescent Health Collaborators. JAMA Pediatrics.

eFigure 6d. Annual percent change of observed to expected (O:E) all−cause DALY rates in <20 years from 1990 to 2000 versus 2000 to 2017

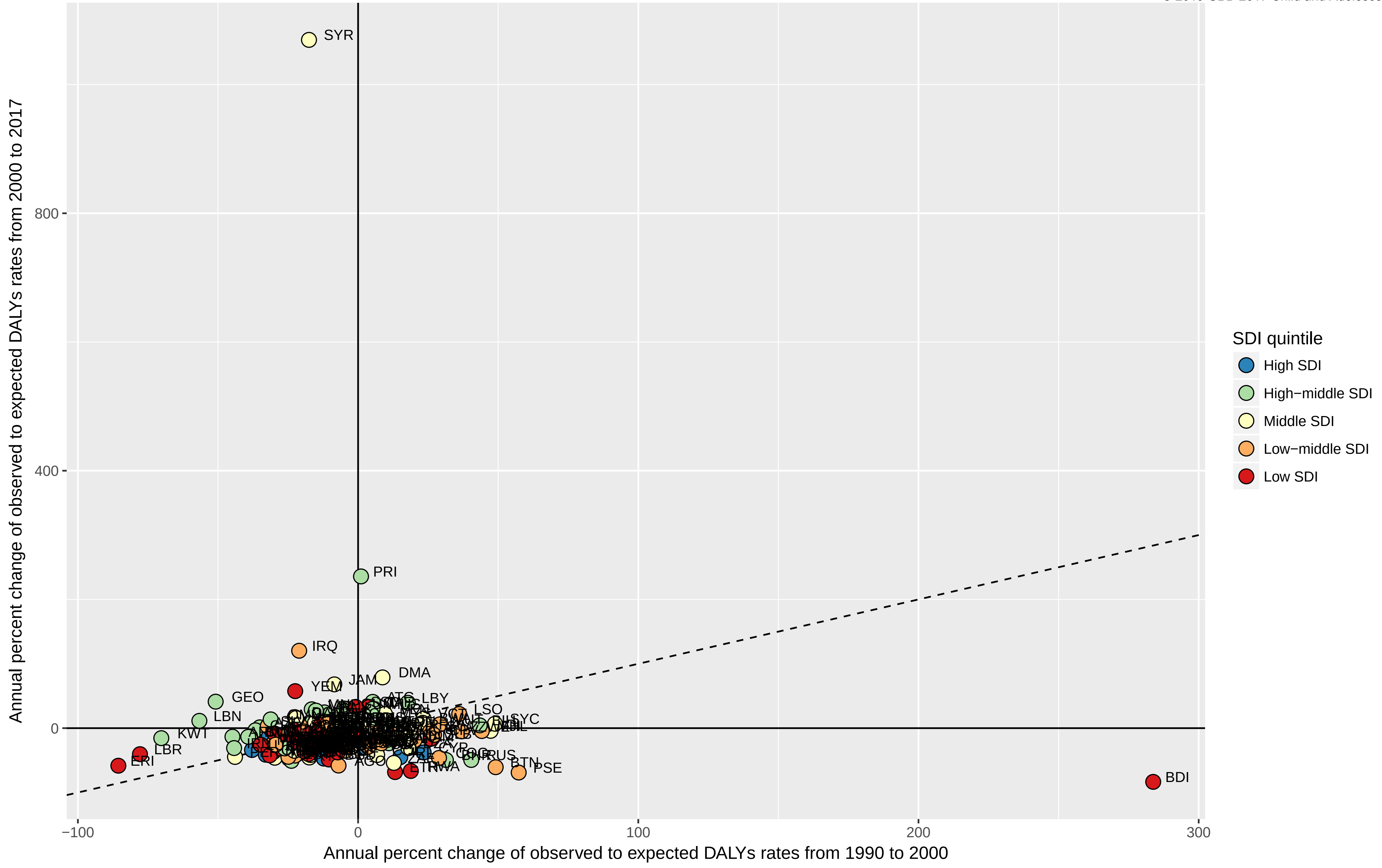

eFigure 6d. Annual percent change of observed to expected (O:E) all−cause DALY rates in <20 years from 1990 to 2000 versus 2000 to 2017 (graph subsetted to +/− 50%)

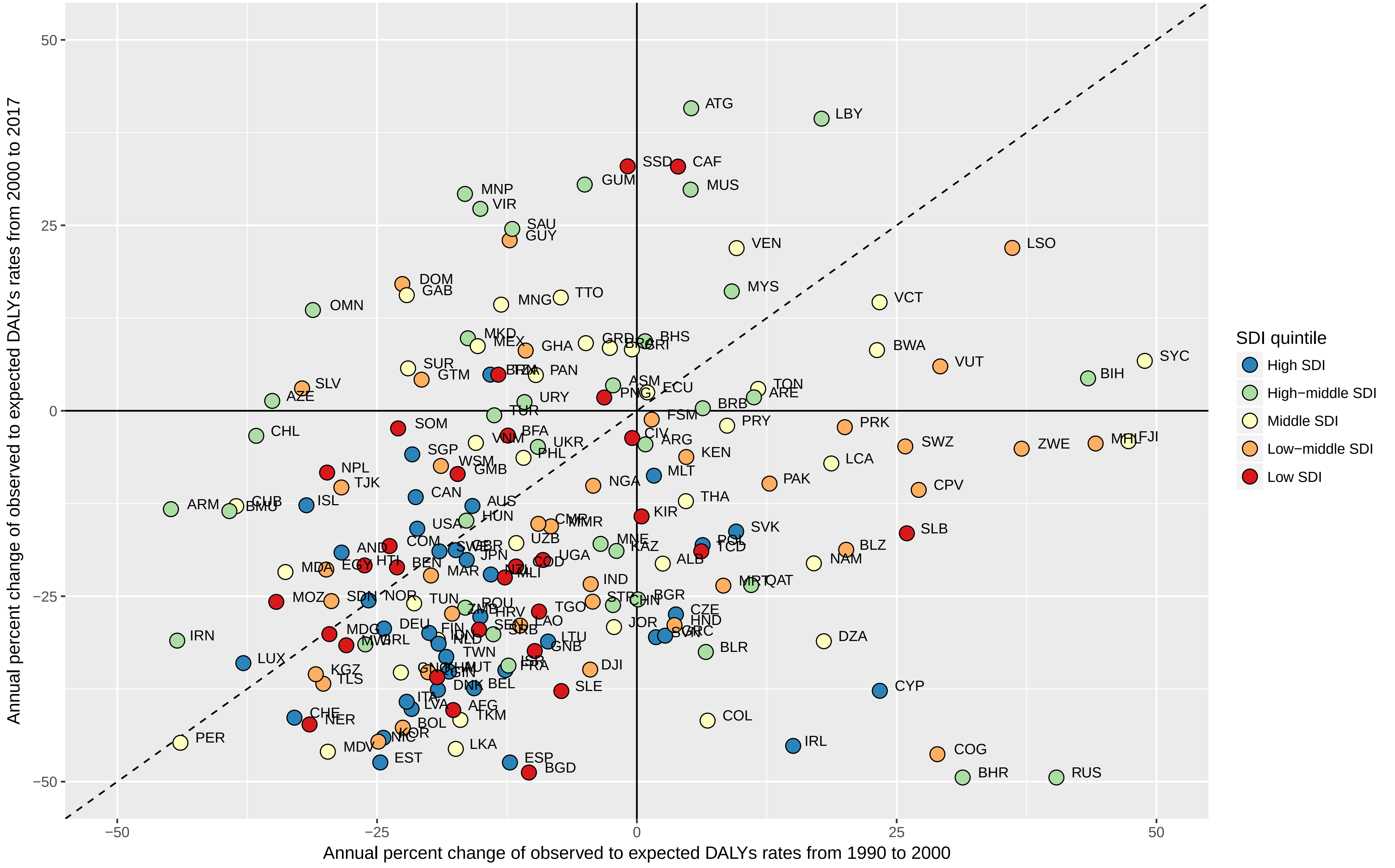

Annual percent change of observed versus expected (O:E) disability−adjusted life years (DALYs) rates by country for 1990 to 2000 versus 2000 to 2017 are plotted by country. In each pair of panels, the right panel focuses on changes within +/−50%. Panels a−d plot changes for all children and adolescents less than 20 years of age; panels e−h plot changes for children less than 1; panels i−l plot changes for children between 1 and 4; panels m−p plot changes for children between 5 and 9; and panels q−t plot changes for children between 10 and 19. For each age group, the first plot in the series is the all cause change in O:E rates (i.e., panels a, e, i, m, and q); the second plot in the series is the change in O:E rates associated with communicable, maternal, neonatal, and nutritional (CMNN) conditions (i.e., panels b, f, j, and r); the third plot in the series is the change in O:E rates associated with non−communicable diseases (NCDs – i.e., panels c, g, k, s); and the fourth plot in the series is the change in O:E rates associated with injuries (i.e., panels d, h, l, t). For each, countries in different socio−demographic index (SDI) quintiles are plotted with different colours. Abbreviations: DALY=disability−adjusted life year, AFG − Afghanistan, AGO − Angola, ALB − Albania, AND − Andorra, ARE − United Arab Emirates, ARG − Argentina, ARM − Armenia, ASM − American Samoa, ATG − Antigua and Barbuda, AUS − Australia, AUT − Austria, AZE − Azerbaijan, BDI − Burundi, BEL − Belgium, BEN − Benin, BFA − Burkina Faso, BGD − Bangladesh, BGR − Bulgaria, BHR − Bahrain, BHS − The Bahamas, BIH − Bosnia and Herzegovina, BLR − Belarus, BLZ − Belize, BMU − Bermuda, BOL − Bolivia, BRA − Brazil, BRB − Barbados, BRN − Brunei, BTN − Bhutan, BWA − Botswana, CAF − Central African Republic, CAN − Canada, CHE − Switzerland, CHL − Chile, CHN − China, CIV − Cote d'Ivoire, CMR − Cameroon, COD − Democratic Republic of the Congo, COG − Congo, COL − Colombia, COM − Comoros, CPV − Cape Verde, CRI − Costa Rica, CUB − Cuba, CYP − Cyprus, CZE − Czech Republic, DEU − Germany, DJI − Djibouti, DMA − Dominica, DNK − Denmark, DOM − Dominican Republic, DZA − Algeria, ECU − Ecuador, EGY − Egypt, ERI − Eritrea, ESP − Spain, EST − Estonia, ETH − Ethiopia, FIN − Finland, FJI − Fiji, FRA − France, FSM − Federated States of Micronesia, G − Global, GAB − Gabon, GBR − United Kingdom, GEO − Georgia, GHA − Ghana, GIN − Guinea, GMB − The Gambia, GNB − Guinea−Bissau, GNQ − Equatorial Guinea, GRC − Greece, GRD − Grenada, GRL − Greenland, GTM − Guatemala, GUM − Guam, GUY − Guyana, HND − Honduras, HRV − Croatia, HTI − Haiti, HUN − Hungary, IDN − Indonesia, IND − India, IRL − Ireland, IRN − Iran, IRQ − Iraq, ISL − Iceland, ISR − Israel, ITA − Italy, JAM − Jamaica, JOR − Jordan, JPN − Japan, KAZ − Kazakhstan, KEN − Kenya, KGZ − Kyrgyzstan, KHM − Cambodia, KIR − Kiribati, KOR − South Korea, KWT − Kuwait, LAO − Laos, LBN − Lebanon, LBR − Liberia, LBY − Libya, LCA − Saint Lucia, LKA − Sri Lanka, LSO − Lesotho, LTU − Lithuania, LUX − Luxembourg, LVA − Latvia, MAR − Morocco, MDA − Moldova, MDG − Madagascar, MDV − Maldives, MEX − Mexico, MHL − Marshall Islands, MKD − Macedonia, MLI − Mali, MLT − Malta, MMR − Myanmar, MNE − Montenegro, MNG − Mongolia, MNP − Northern Mariana Islands, MOZ − Mozambique, MRT − Mauritania, MUS − Mauritius, MWI − Malawi, MYS − Malaysia, NAM − Namibia, NER − Niger, NGA − Nigeria, NIC − Nicaragua, NLD − Netherlands, NOR − Norway, NPL − Nepal, NZL − New Zealand, OMN − Oman, PAK − Pakistan, PAN − Panama, PER − Peru, PHL − Philippines, PNG − Papua New Guinea, POL − Poland, PRI − Puerto Rico, PRK − North Korea, PRT − Portugal, PRY − Paraguay, PSE − Palestine, QAT − Qatar, ROU − Romania, RUS − Russian Federation, RWA − Rwanda, SAU − Saudi Arabia, SDN − Sudan, SEN − Senegal, SGP − Singapore, SLB − Solomon Islands, SLE − Sierra Leone, SLV − El Salvador, SOM − Somalia, SRB − Serbia, SSD − South Sudan, STP − Sao Tome and Principe, SUR − Suriname, SVK − Slovakia, SVN − Slovenia, SWE − Sweden, SWZ − Swaziland, SYC − Seychelles, SYR − Syria, TCD − Chad, TGO − Togo, THA − Thailand, TJK − Tajikistan, TKM − Turkmenistan, TLS − Timor−Leste, TON − Tonga, TTO − Trinidad and Tobago, TUN − Tunisia, TUR − Turkey, TWN − Taiwan, TZA − Tanzania, UGA − Uganda, UKR − Ukraine, URY − Uruguay, USA − United States, UZB − Uzbekistan, VCT − Saint Vincent and the Grenadines, VEN − Venezuela, VIR − Virgin Islands, U.S., VNM − Vietnam, VUT − Vanuatu, WSM − Samoa, YEM − Yemen, ZAF − South Africa, ZMB − Zambia, ZWE − Zimbabwe

© 2019 GBD 2017 Child and Adolescent Health Collaborators. JAMA Pediatrics.

eFigure 6e. Annual percent change of observed to expected (O:E) all−cause DALY rates in <20 years from 1990 to 2000 versus 2000 to 2017

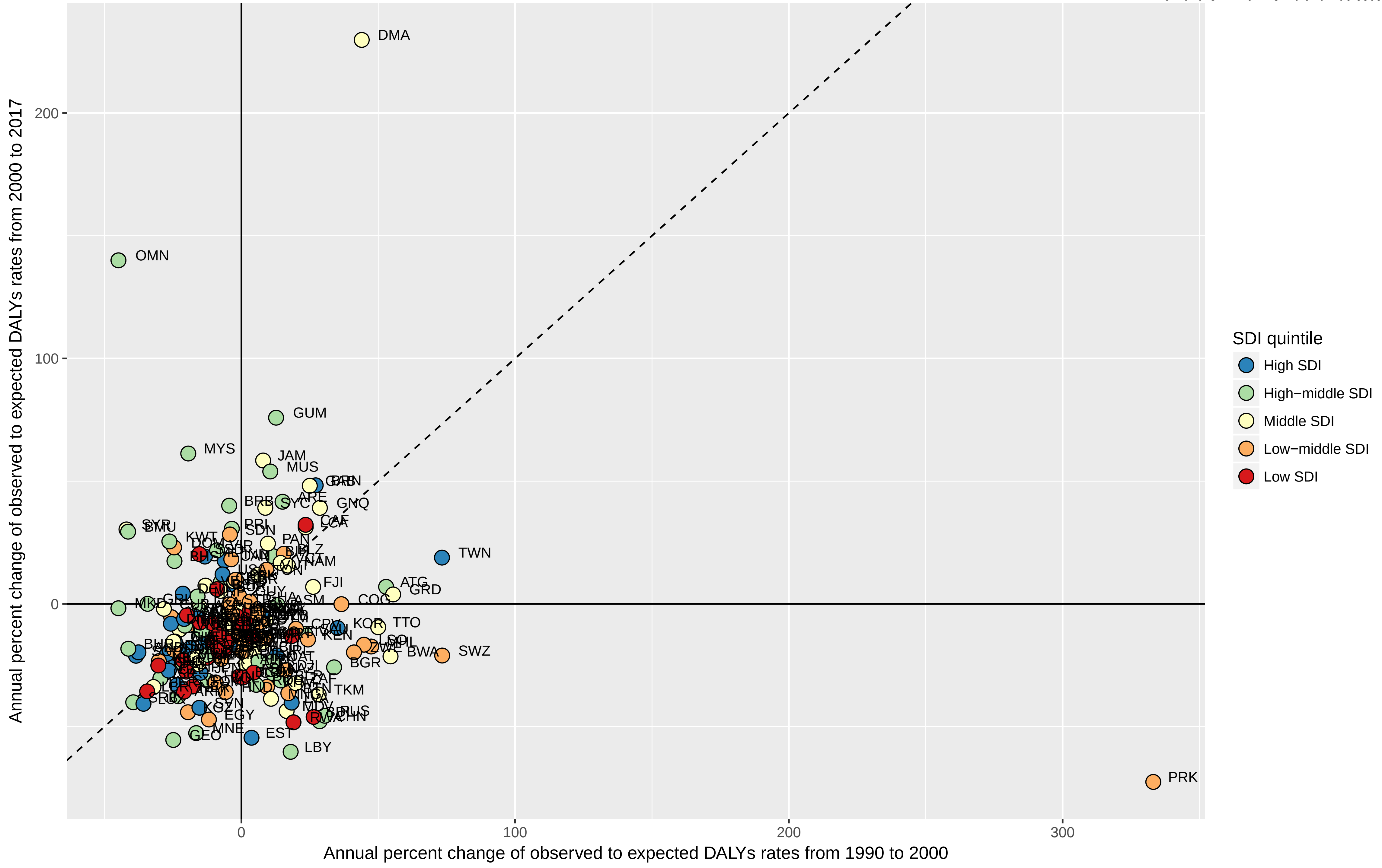

eFigure 6e. Annual percent change of observed to expected (O:E) all−cause DALY rates in <20 years from 1990 to 2000 versus 2000 to 2017 (graph subsetted to +/− 50%)

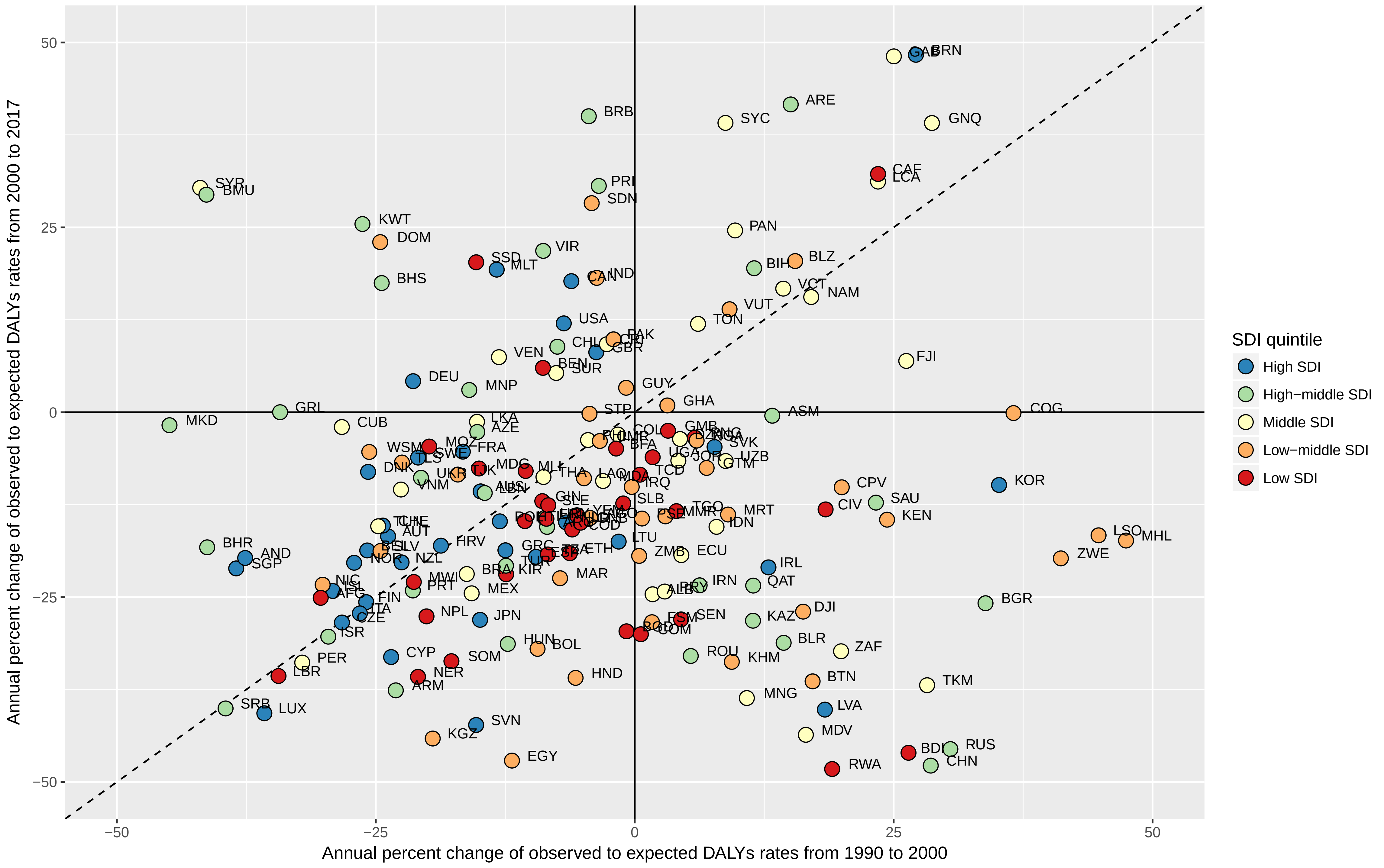

Annual percent change of observed versus expected (O:E) disability−adjusted life years (DALYs) rates by country for 1990 to 2000 versus 2000 to 2017 are plotted by country. In each pair of panels, the right panel focuses on changes within +/−50%. Panels a−d plot changes for all children and adolescents less than 20 years of age; panels e−h plot changes for children less than 1; panels i−l plot changes for children between 1 and 4; panels m−p plot changes for children between 5 and 9; and panels q−t plot changes for children between 10 and 19. For each age group, the first plot in the series is the all cause change in O:E rates (i.e., panels a, e, i, m, and q); the second plot in the series is the change in O:E rates associated with communicable, maternal, neonatal, and nutritional (CMNN) conditions (i.e., panels b, f, j, and r); the third plot in the series is the change in O:E rates associated with non−communicable diseases (NCDs – i.e., panels c, g, k, s); and the fourth plot in the series is the change in O:E rates associated with injuries (i.e., panels d, h, l, t). For each, countries in different socio−demographic index (SDI) quintiles are plotted with different countries. Abbreviations: DALY=disability−adjusted life year, AFG − Afghanistan, AGO − Angola, ALB − Albania, AND − Andorra, ARE − United Arab Emirates, ARG − Argentina, ARM − Armenia, ASM − American Samoa, ATG − Antigua and Barbuda, AUS − Australia, AUT − Austria, AZE − Azerbaijan, BDI − Burundi, BEL − Belgium, BEN − Benin, BFA − Burkina Faso, BGD − Bangladesh, BGR − Bulgaria, BHR − Bahrain, BHS − The Bahamas, BIH − Bosnia and Herzegovina, BLR − Belarus, BLZ − Belize, BMU − Bermuda, BOL − Bolivia, BRA − Brazil, BRB − Barbados, BRN − Brunei, BTN − Bhutan, BWA − Botswana, CAF − Central African Republic, CAN − Canada, CHE − Switzerland, CHL − Chile, CHN − China, CIV − Cote d'Ivoire, CMR − Cameroon, COD − Democratic Republic of the Congo, COG − Congo, COL − Colombia, COM − Comoros, CPV − Cape Verde, CRI − Costa Rica, CUB − Cuba, CYP − Cyprus, CZE − Czech Republic, DEU − Germany, DJI − Djibouti, DMA − Dominica, DNK − Denmark, DOM − Dominican Republic, DZA − Algeria, ECU − Ecuador, EGY − Egypt, ERI − Eritrea, ESP − Spain, EST − Estonia, ETH − Ethiopia, FIN − Finland, FJI − Fiji, FRA − France, FSM − Federated States of Micronesia, G − Global, GAB − Gabon, GBR − United Kingdom, GEO − Georgia, GHA − Ghana, GIN − Guinea, GMB − The Gambia, GNB − Guinea−Bissau, GNQ − Equatorial Guinea, GRC − Greece, GRD − Grenada, GRL − Greenland, GTM − Guatemala, GUM − Guam, GUY − Guyana, HND − Honduras, HRV − Croatia, HTI − Haiti, HUN − Hungary, IDN − Indonesia, IND − India, IRL − Ireland, IRN − Iran, IRQ − Iraq, ISL − Iceland, ISR − Israel, ITA − Italy, JAM − Jamaica, JOR − Jordan, JPN − Japan, KAZ − Kazakhstan, KEN − Kenya, KGZ − Kyrgyzstan, KHM − Cambodia, KIR − Kiribati, KOR − South Korea, KWT − Kuwait, LAO − Laos, LBN − Lebanon, LBR − Liberia, LBY − Libya, LCA − Saint Lucia, LKA − Sri Lanka, LSO − Lesotho, LTU − Lithuania, LUX − Luxembourg, LVA − Latvia, MAR − Morocco, MDA − Moldova, MDG − Madagascar, MDV − Maldives, MEX − Mexico, MHL − Marshall Islands, MKD − Macedonia, MLI − Mali, MLT − Malta, MMR − Myanmar, MNE − Montenegro, MNG − Mongolia, MNP − Northern Mariana Islands, MOZ − Mozambique, MRT − Mauritania, MUS − Mauritius, MWI − Malawi, MYS − Malaysia, NAM − Namibia, NER − Niger, NGA − Nigeria, NIC − Nicaragua, NLD − Netherlands, NOR − Norway, NPL − Nepal, NZL − New Zealand, OMN − Oman, PAK − Pakistan, PAN − Panama, PER − Peru, PHL − Philippines, PNG − Papua New Guinea, POL − Poland, PRI − Puerto Rico, PRK − North Korea, PRT − Portugal, PRY − Paraguay, PSE − Palestine, QAT − Qatar, ROU − Romania, RUS − Russian Federation, RWA − Rwanda, SAU − Saudi Arabia, SDN − Sudan, SEN − Senegal, SGP − Singapore, SLB − Solomon Islands, SLE − Sierra Leone, SLV − El Salvador, SOM − Somalia, SRB − Serbia, SSD − South Sudan, STP − Sao Tome and Principe, SUR − Suriname, SVK − Slovakia, SVN − Slovenia, SWE − Sweden, SWZ − Swaziland, SYC − Seychelles, SYR − Syria, TCD − Chad, TGO − Togo, THA − Thailand, TJK − Tajikistan, TKM − Turkmenistan, TLS − Timor−Leste, TON − Tonga, TTO − Trinidad and Tobago, TUN − Tunisia, TUR − Turkey, TWN − Taiwan, TZA − Tanzania, UGA − Uganda, UKR − Ukraine, URY − Uruguay, USA − United States, UZB − Uzbekistan, VCT − Saint Vincent and the Grenadines, VEN − Venezuela, VIR − Virgin Islands, U.S., VNM − Vietnam, VUT − Vanuatu, WSM − Samoa, YEM − Yemen, ZAF − South Africa, ZMB − Zambia, ZWE − Zimbabwe

© 2019 GBD 2017 Child and Adolescent Health Collaborators. JAMA Pediatrics.

eFigure 6f. Annual percent change of observed to expected (O:E) all−cause DALY rates in <20 years from 1990 to 2000 versus 2000 to 2017

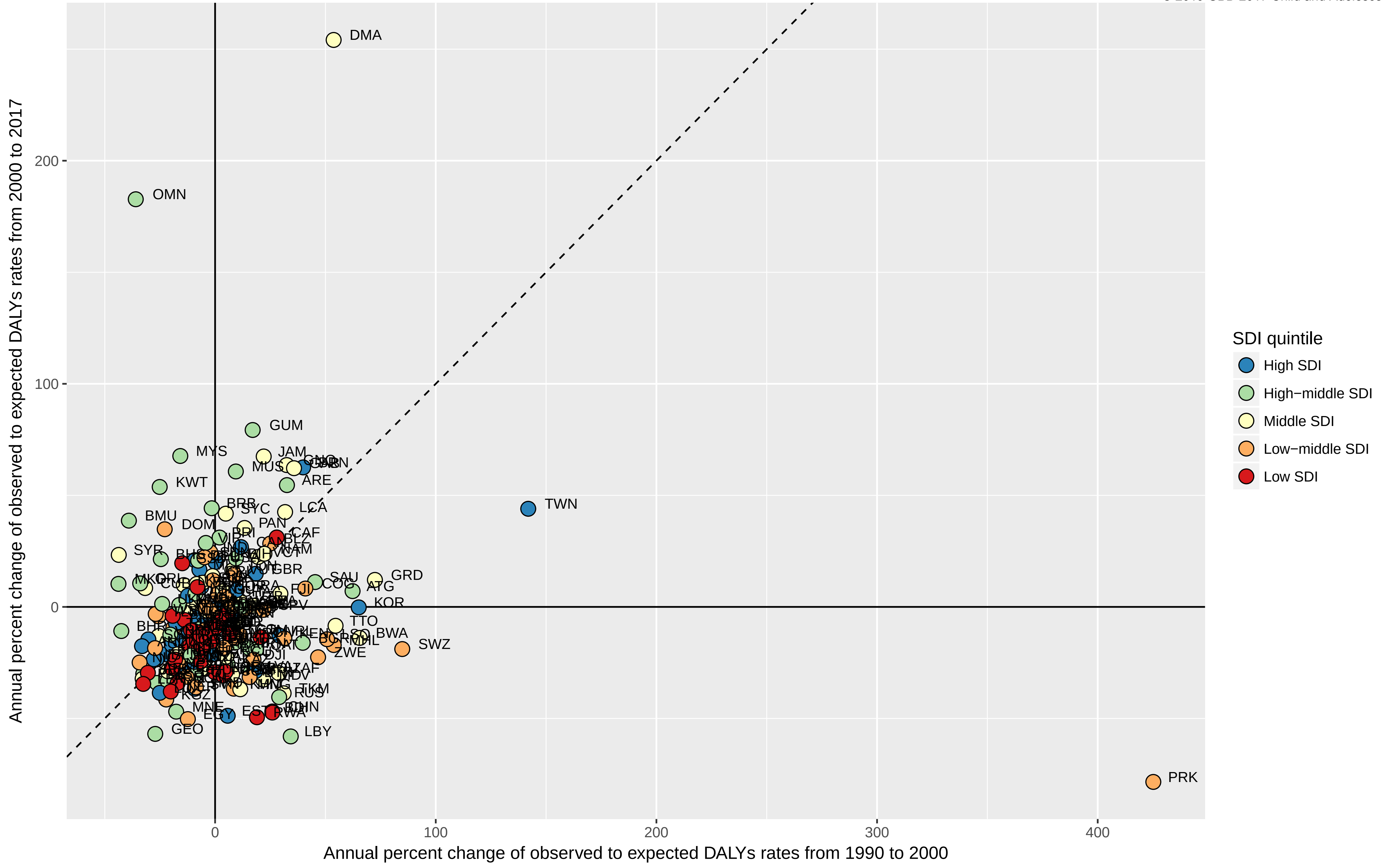

eFigure 6f. Annual percent change of observed to expected (O:E) all−cause DALY rates in <20 years from 1990 to 2000 versus 2000 to 2017 (graph subsetted to +/− 50%)

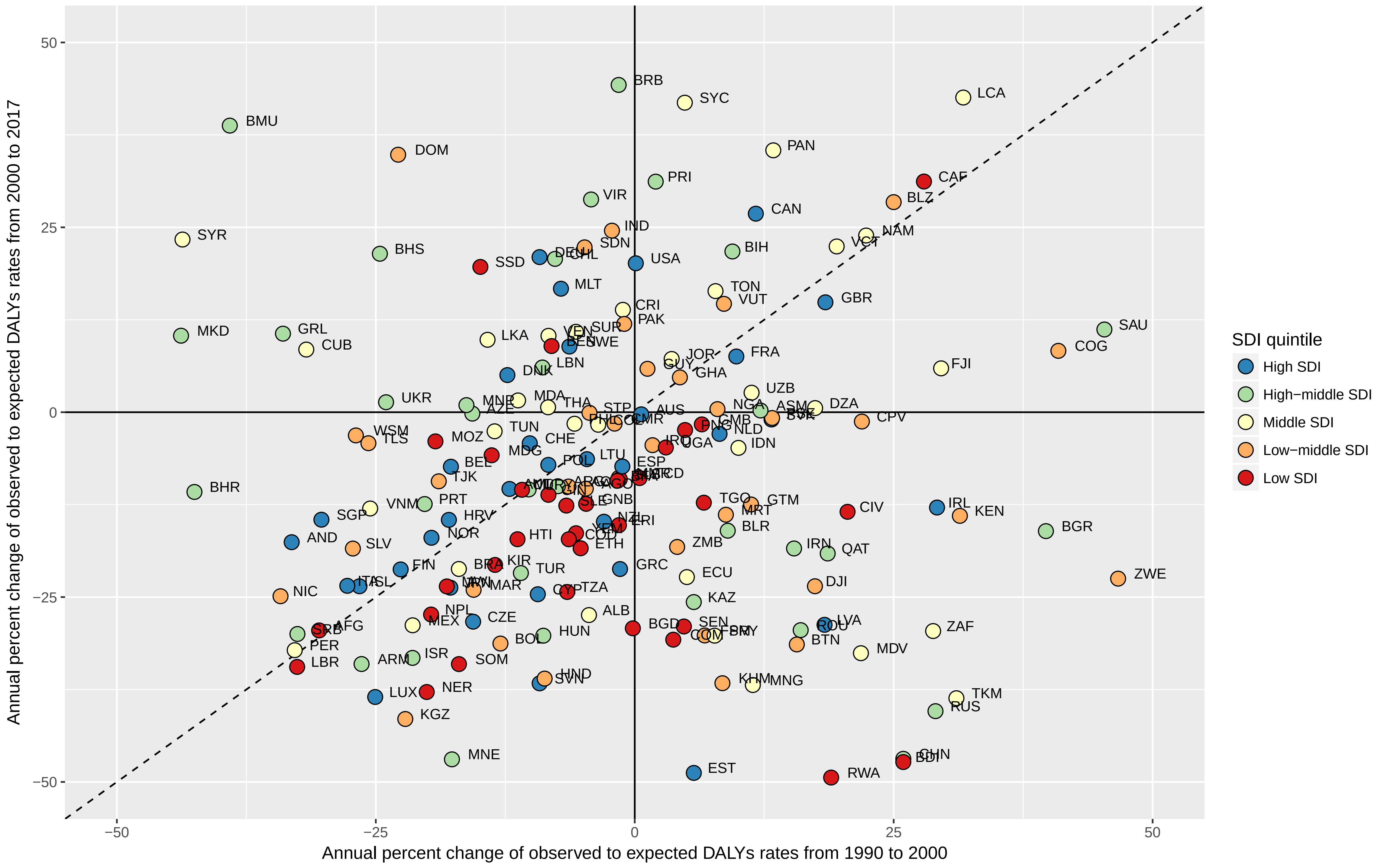

Annual percent change of observed versus expected (O:E) disability−adjusted life years (DALYs) rates by country for 1990 to 2000 versus 2000 to 2017 are plotted by country. In each pair of panels, the right panel focuses on changes within +/−50%. Panels a−d plot changes for all children and adolescents less than 20 years of age; panels e−h plot changes for children less than 1; panels i−l plot changes for children between 1 and 4; panels m−p plot changes for children between 5 and 9; and panels q−t plot changes for children between 10 and 19. For each age group, the first plot in the series is the all cause change in O:E rates (i.e., panels a, e, i, m, and q); the second plot in the series is the change in O:E rates associated with communicable, maternal, neonatal, and nutritional (CMNN) conditions (i.e., panels b, f, j, and r); the third plot in the series is the change in O:E rates associated with non−communicable diseases (NCDs – i.e., panels c, g, k, s); and the fourth plot in the series is the change in O:E rates associated with injuries (i.e., panels d, h, l, t). For each countries in different socio−demographic index (SDI) quintiles are plotted with different countries. Abbreviations: DALY=disability−adjusted life year, AFG − Afghanistan, AGO − Angola, ALB − Albania, AND − Andorra, ARE − United Arab Emirates, ARG − Argentina, ARM − Armenia, ASM − American Samoa, ATG − Antigua and Barbuda, AUS − Australia, AUT − Austria, AZE − Azerbaijan, BDI − Burundi, BEL − Belgium, BEN − Benin, BFA − Burkina Faso, BGD − Bangladesh, BGR − Bulgaria, BHR − Bahrain, BHS − The Bahamas, BIH − Bosnia and Herzegovina, BLR − Belarus, BLZ − Belize, BMU − Bermuda, BOL − Bolivia, BRA − Brazil, BRB − Barbados, BRN − Brunei, BTN − Bhutan, BWA − Botswana, CAF − Central African Republic, CAN − Canada, CHE − Switzerland, CHL − Chile, CHN − China, CIV − Cote d'Ivoire, CMR − Cameroon, COD − Democratic Republic of the Congo, COG − Congo, COL − Colombia, COM − Comoros, CPV − Cape Verde, CRI − Costa Rica, CUB − Cuba, CYP − Cyprus, CZE − Czech Republic, DEU − Germany, DJI − Djibouti, DMA − Dominica, DNK − Denmark, DOM − Dominican Republic, DZA − Algeria, ECU − Ecuador, EGY − Egypt, ERI − Eritrea, ESP − Spain, EST − Estonia, ETH − Ethiopia, FIN − Finland, FJI − Fiji, FRA − France, FSM − Federated States of Micronesia, G − Global, GAB − Gabon, GBR − United Kingdom, GEO − Georgia, GHA − Ghana, GIN − Guinea, GMB − The Gambia, GNB − Guinea−Bissau, GNQ − Equatorial Guinea, GRC − Greece, GRD − Grenada, GRL − Greenland, GTM − Guatemala, GUM − Guam, GUY − Guyana, HND − Honduras, HRV − Croatia, HTI − Haiti, HUN − Hungary, IDN − Indonesia, IND − India, IRL − Ireland, IRN − Iran, IRQ − Iraq, ISL − Iceland, ISR − Israel, ITA − Italy, JAM − Jamaica, JOR − Jordan, JPN − Japan, KAZ − Kazakhstan, KEN − Kenya, KGZ − Kyrgyzstan, KHM − Cambodia, KIR − Kiribati, KOR − South Korea, KWT − Kuwait, LAO − Laos, LBN − Lebanon, LBR − Liberia, LBY − Libya, LCA − Saint Lucia, LKA − Sri Lanka, LSO − Lesotho, LTU − Lithuania, LUX − Luxembourg, LVA − Latvia, MAR − Morocco, MDA − Moldova, MDG − Madagascar, MDV − Maldives, MEX − Mexico, MHL − Marshall Islands, MKD − Macedonia, MLI − Mali, MLT − Malta, MMR − Myanmar, MNE − Montenegro, MNG − Mongolia, MNP − Northern Mariana Islands, MOZ − Mozambique, MRT − Mauritania, MUS − Mauritius, MWI − Malawi, MYS − Malaysia, NAM − Namibia, NER − Niger, NGA − Nigeria, NIC − Nicaragua, NLD − Netherlands, NOR − Norway, NPL − Nepal, NZL − New Zealand, OMN − Oman, PAK − Pakistan, PAN − Panama, PER − Peru, PHL − Philippines, PNG − Papua New Guinea, POL − Poland, PRI − Puerto Rico, PRK − North Korea, PRT − Portugal, PRY − Paraguay, PSE − Palestine, QAT − Qatar, ROU − Romania, RUS − Russian Federation, RWA − Rwanda, SAU − Saudi Arabia, SDN − Sudan, SEN − Senegal, SGP − Singapore, SLB − Solomon Islands, SLE − Sierra Leone, SLV − El Salvador, SOM − Somalia, SRB − Serbia, SSD − South Sudan, STP − Sao Tome and Principe, SUR − Suriname, SVK − Slovakia, SVN − Slovenia, SWE − Sweden, SWZ − Swaziland, SYC − Seychelles, SYR − Syria, TCD − Chad, TGO − Togo, THA − Thailand, TJK − Tajikistan, TKM − Turkmenistan, TLS − Timor−Leste, TON − Tonga, TTO − Trinidad and Tobago, TUN − Tunisia, TUR − Turkey, TWN − Taiwan, TZA − Tanzania, UGA − Uganda, UKR − Ukraine, URY − Uruguay, USA − United States, UZB − Uzbekistan, VCT − Saint Vincent and the Grenadines, VEN − Venezuela, VIR − Virgin Islands, U.S., VNM − Vietnam, VUT − Vanuatu, WSM − Samoa, YEM − Yemen, ZAF − South Africa, ZMB − Zambia, ZWE − Zimbabwe

© 2019 GBD 2017 Child and Adolescent Health Collaborators. JAMA Pediatrics.

eFigure 6g. Annual percent change of observed to expected (O:E) all−cause DALY rates in <20 years from 1990 to 2000 versus 2000 to 2017

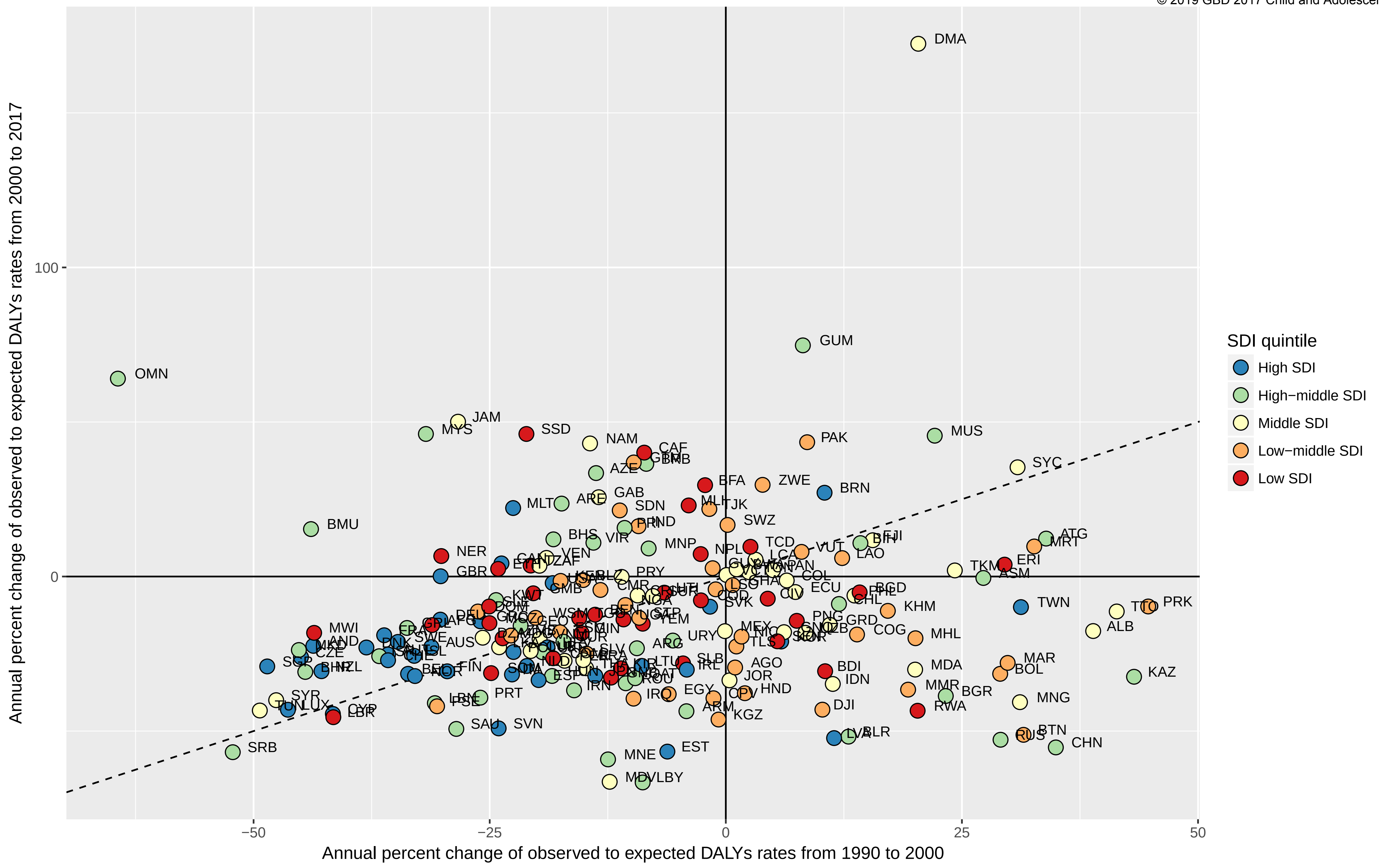

eFigure 6g. Annual percent change of observed to expected (O:E) all−cause DALY rates in <20 years from 1990 to 2000 versus 2000 to 2017 (graph subsetted to +/− 50%)

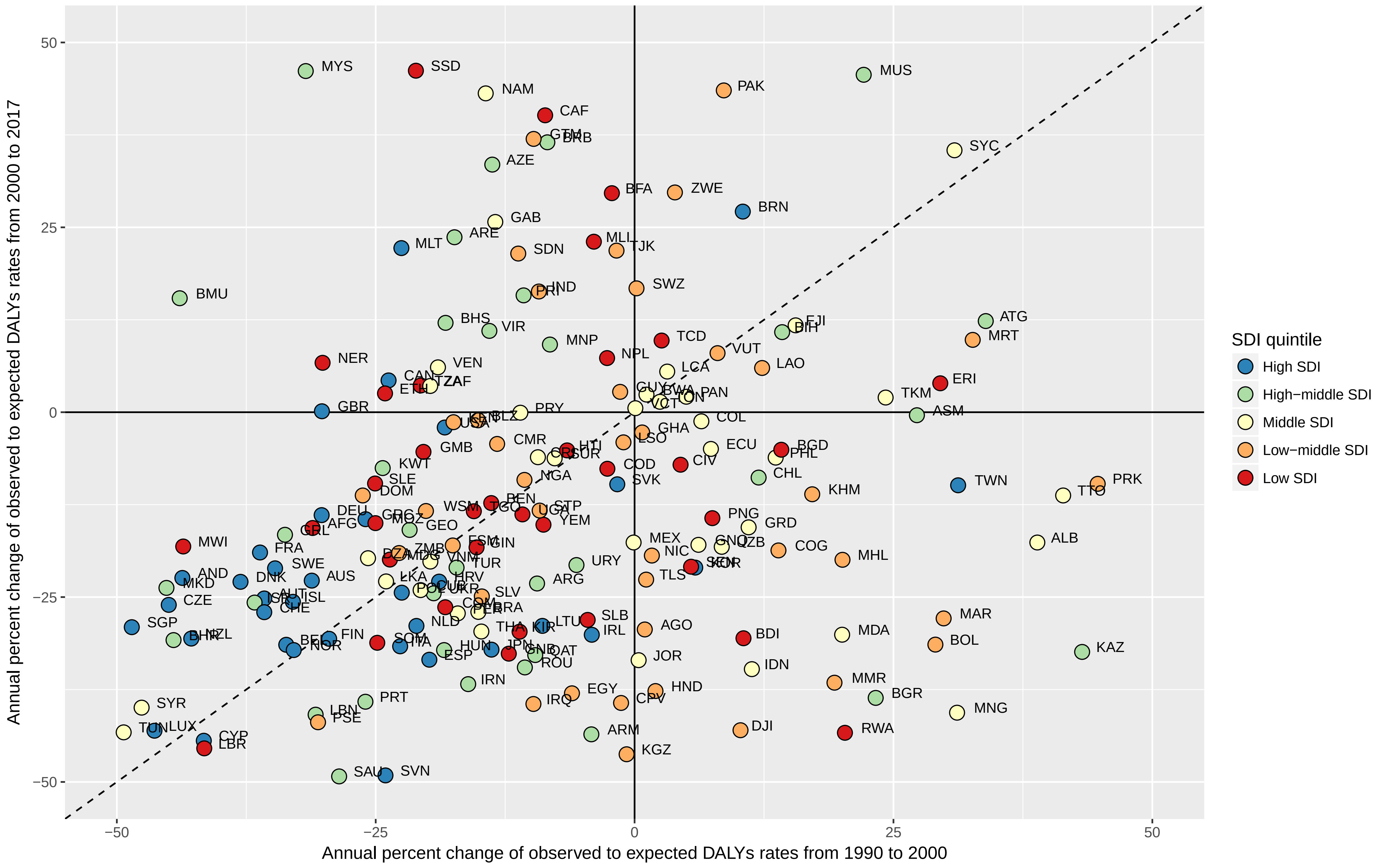

Annual percent change of observed versus expected (O:E) disability−adjusted life years (DALYs) rates by country for 1990 to 2000 versus 2000 to 2017 are plotted by country. In each pair of panels, the right panel focuses on changes within +/−50%. Panels a–d plot changes for all children and adolescents less than 20 years of age; panels e−h plot changes for children less than 1; panels i−l plot changes for children between 1 and 4; panels m−p plot changes for children between 5 and 9; and panels q−t plot changes for children between 10 and 19. For each age group, the first plot in the series is the all cause change in O:E rates (i.e., panels a, e, i, m, and q); the second plot in the series is the change in O:E rates associated with communicable, maternal, neonatal, and nutritional (CMNN) conditions (i.e., panels b, f, j, and r); the third plot in the series is the change in O:E rates associated with non−communicable diseases (NCDs – i.e., panels c, g, k, s); and the fourth plot in the series is the change in O:E rates associated with injuries (i.e., panels d, h, l, t). For each, countries in different socio−demographic index (SDI) quintiles are plotted with different countries. Abbreviations: DALY=disability−adjusted life year, AFG − Afghanistan, AGO − Angola, ALB − Albania, AND − Andorra, ARE − United Arab Emirates, ARG − Argentina, ARM − Armenia, ASM − American Samoa, ATG − Antigua and Barbuda, AUS − Australia, AUT − Austria, AZE − Azerbaijan, BDI − Burundi, BEL − Belgium, BEN − Benin, BFA − Burkina Faso, BGD − Bangladesh, BGR − Bulgaria, BHR − Bahrain, BHS − The Bahamas, BIH − Bosnia and Herzegovina, BLR − Belarus, BLZ − Belize, BMU − Bermuda, BOL − Bolivia, BRA − Brazil, BRB − Barbados, BRN − Brunei, BTN − Bhutan, BWA − Botswana, CAF − Central African Republic, CAN − Canada, CHE − Switzerland, CHL − Chile, CHN − China, CIV − Cote d'Ivoire, CMR − Cameroon, COD − Democratic Republic of the Congo, COG − Congo, COL − Colombia, COM − Comoros, CPV − Cape Verde, CRI − Costa Rica, CUB − Cuba, CYP − Cyprus, CZE − Czech Republic, DEU − Germany, DJI − Djibouti, DMA − Dominica, DNK − Denmark, DOM − Dominican Republic, DZA − Algeria, ECU − Ecuador, EGY − Egypt, ERI − Eritrea, ESP − Spain, EST − Estonia, ETH − Ethiopia, FIN − Finland, FJI − Fiji, FRA − France, FSM − Federated States of Micronesia, G − Global, GAB − Gabon, GBR − United Kingdom, GEO − Georgia, GHA − Ghana, GIN − Guinea, GMB − The Gambia, GNB − Guinea−Bissau, GNQ − Equatorial Guinea, GRC − Greece, GRD − Grenada, GRL − Greenland, GTM − Guatemala, GUM − Guam, GUY − Guyana, HND − Honduras, HRV − Croatia, HTI − Haiti, HUN − Hungary, IDN − Indonesia, IND − India, IRL − Ireland, IRN − Iran, IRQ − Iraq, ISL − Iceland, ISR − Israel, ITA − Italy, JAM − Jamaica, JOR − Jordan, JPN − Japan, KAZ − Kazakhstan, KEN − Kenya, KGZ − Kyrgyzstan, KHM − Cambodia, KIR − Kiribati, KOR − South Korea, KWT − Kuwait, LAO − Laos, LBN − Lebanon, LBR − Liberia, LBY − Libya, LCA − Saint Lucia, LKA − Sri Lanka, LSO − Lesotho, LTU − Lithuania, LUX − Luxembourg, LVA − Latvia, MAR − Morocco, MDA − Moldova, MDG − Madagascar, MDV − Maldives, MEX − Mexico, MHL − Marshall Islands, MKD − Macedonia, MLI − Mali, MLT − Malta, MMR − Myanmar, MNE − Montenegro, MNG − Mongolia, MNP − Northern Mariana Islands, MOZ − Mozambique, MRT − Mauritania, MUS − Mauritius, MWI − Malawi, MYS − Malaysia, NAM − Namibia, NER − Niger, NGA − Nigeria, NIC − Nicaragua, NLD − Netherlands, NOR − Norway, NPL − Nepal, NZL − New Zealand, OMN − Oman, PAK − Pakistan, PAN − Panama, PER − Peru, PHL − Philippines, PNG − Papua New Guinea, POL − Poland, PRI − Puerto Rico, PRK − North Korea, PRT − Portugal, PRY − Paraguay, PSE − Palestine, QAT − Qatar, ROU − Romania, RUS − Russian Federation, RWA − Rwanda, SAU − Saudi Arabia, SDN − Sudan, SEN − Senegal, SGP − Singapore, SLB − Solomon Islands, SLE − Sierra Leone, SLV − El Salvador, SOM − Somalia, SRB − Serbia, SSD − South Sudan, STP − Sao Tome and Principe, SUR − Suriname, SVK − Slovakia, SVN − Slovenia, SWE − Sweden, SWZ − Swaziland, SYC − Seychelles, SYR − Syria, TCD − Chad, TGO − Togo, THA − Thailand, TJK − Tajikistan, TKM − Turkmenistan, TLS − Timor−Leste, TON − Tonga, TTO − Trinidad and Tobago, TUN − Tunisia, TUR − Turkey, TWN − Taiwan, TZA − Tanzania, UGA − Uganda, UKR − Ukraine, URY − Uruguay, USA − United States, UZB − Uzbekistan, VCT − Saint Vincent and the Grenadines, VEN − Venezuela, VIR − Virgin Islands, U.S., VNM − Vietnam, VUT − Vanuatu, WSM − Samoa, YEM − Yemen, ZAF − South Africa, ZMB − Zambia, ZWE − Zimbabwe

© 2019 GBD 2017 Child and Adolescent Health Collaborators. JAMA Pediatrics.

eFigure 6h. Annual percent change of observed to expected (O:E) all−cause DALY rates in <20 years from 1990 to 2000 versus 2000 to 2017

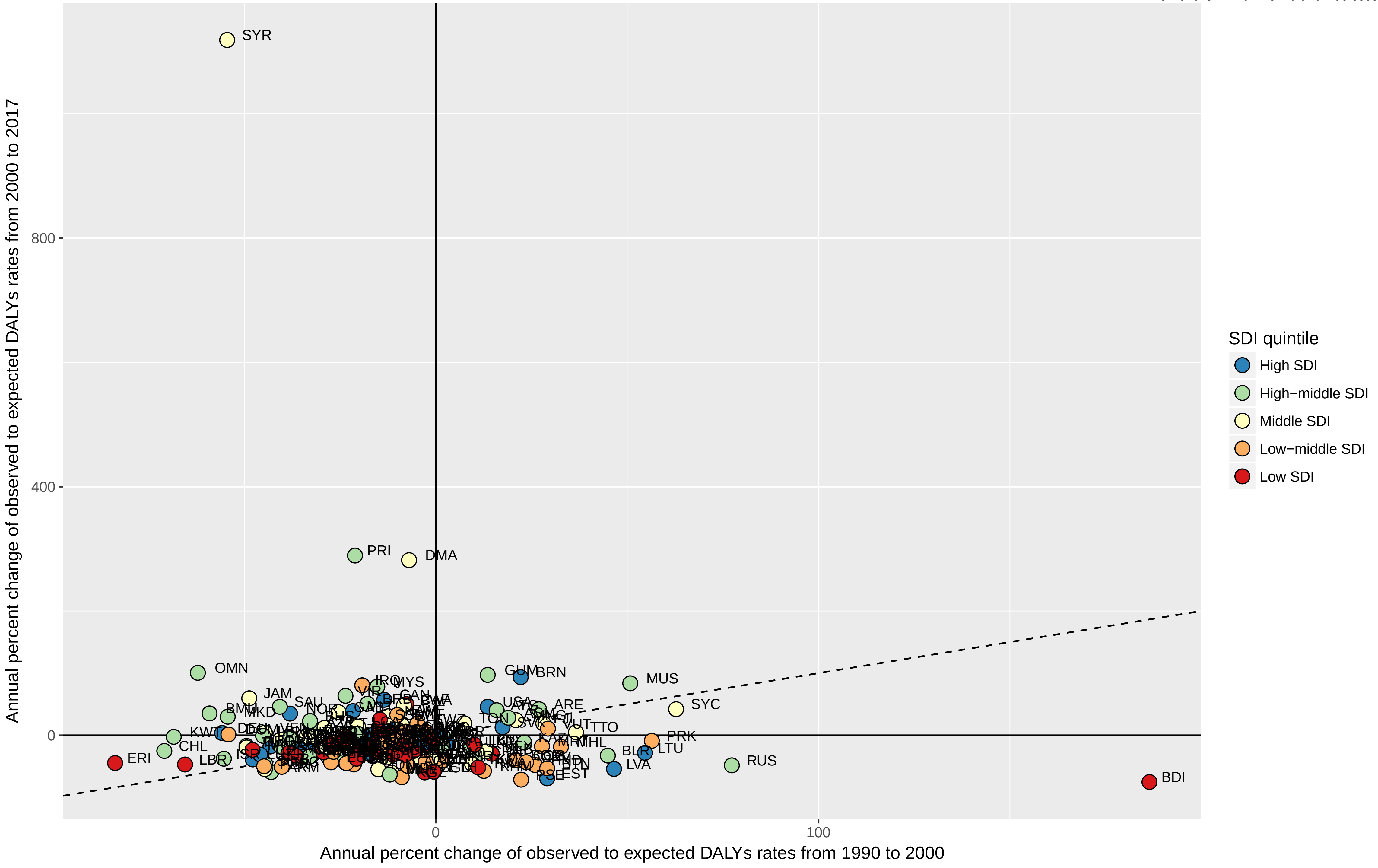

eFigure 6h. Annual percent change of observed to expected (O:E) all−cause DALY rates in <20 years from 1990 to 2000 versus 2000 to 2017 (graph subsetted to +/− 50%)

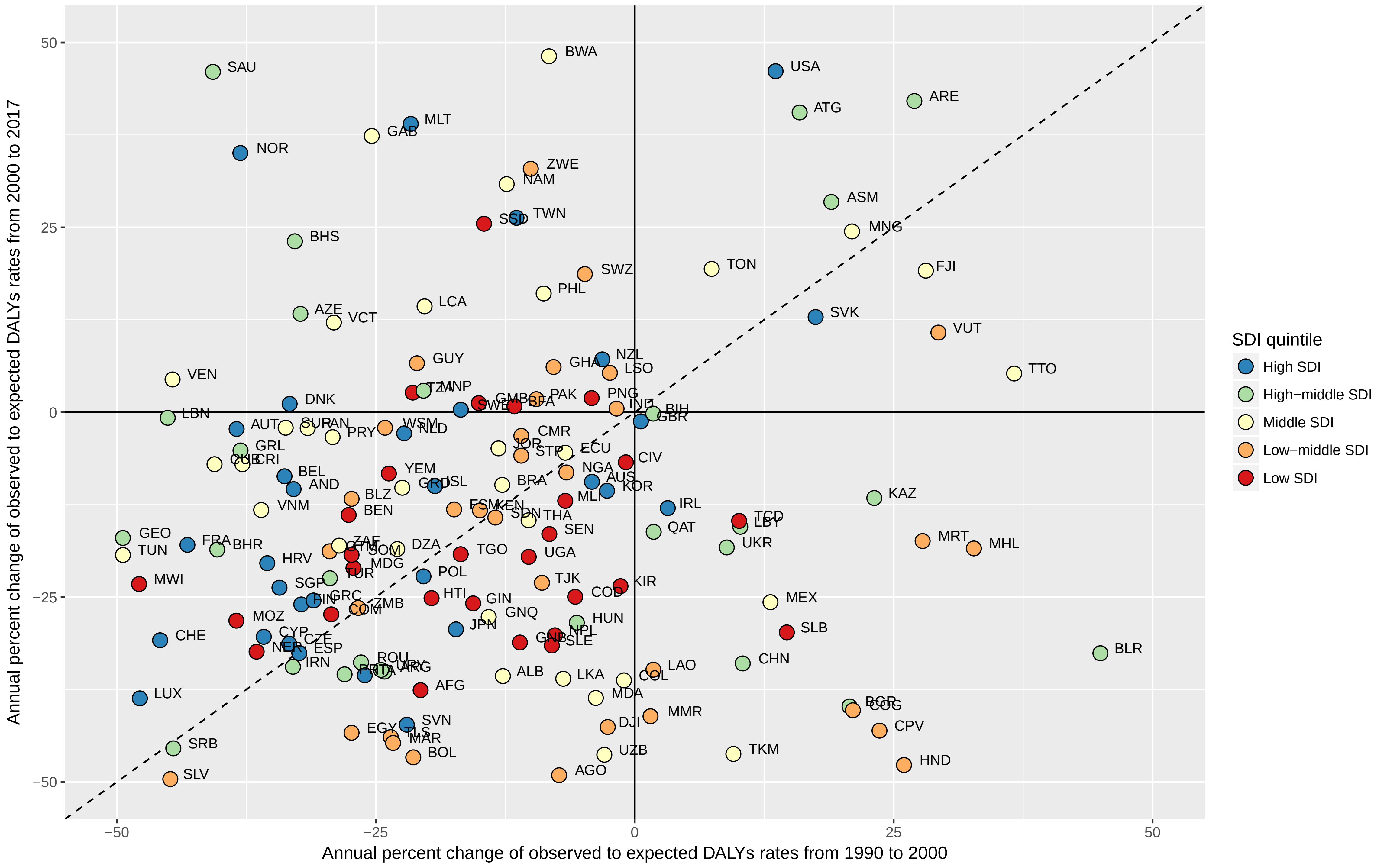

Annual percent change of observed versus expected (O:E) disability−adjusted life years (DALYs) rates by country for 1990 to 2000 versus 2000 to 2017 are plotted by country. In each pair of panels, the right panel focuses on changes within +/−50%. Panels a−d plot changes for all children and adolescents less than 20 years of age; panels e−h plot changes for children less than 1; panels i−l plot changes for children between 1 and 4; panels m−p plot changes for children between 5 and 9; and panels q−t plot changes for children between 10 and 19. For each age group, the first plot in the series is the all cause change in O:E rates (i.e., panels a, e, i, m, and q); the second plot in the series is the change in O:E rates associated with communicable, maternal, neonatal, and nutritional (CMNN) conditions (i.e., panels b, f, j, and r); the third plot in the series is the change in O:E rates associated with non−communicable diseases (NCDs – i.e., panels c, g, k, s); and the fourth plot in the series is the change in O:E rates associated with injuries (i.e., panels d, h, l, t). For each, countries in different socio−demographic index (SDI) quintiles are plotted with different colors. Abbreviations: DALY=disability−adjusted life year, AFG − Afghanistan, AGO − Angola, ALB − Albania, AND − Andorra, ARE − United Arab Emirates, ARG − Argentina, ARM − Armenia, ASM − American Samoa, ATG − Antigua and Barbuda, AUS − Australia, AUT − Austria, AZE − Azerbaijan, BDI − Burundi, BEL − Belgium, BEN − Benin, BFA − Burkina Faso, BGD − Bangladesh, BGR − Bulgaria, BHR − Bahrain, BHS − The Bahamas, BIH − Bosnia and Herzegovina, BLR − Belarus, BLZ − Belize, BMU − Bermuda, BOL − Bolivia, BRA − Brazil, BRB − Barbados, BRN − Brunei, BTN − Bhutan, BWA − Botswana, CAF − Central African Republic, CAN − Canada, CHE − Switzerland, CHL − Chile, CHN − China, CIV − Cote d'Ivoire, CMR − Cameroon, COD − Democratic Republic of the Congo, COG − Congo, COL − Colombia, COM − Comoros, CPV − Cape Verde, CRI − Costa Rica, CUB − Cuba, CYP − Cyprus, CZE − Czech Republic, DEU − Germany, DJI − Djibouti, DMA − Dominica, DNK − Denmark, DOM − Dominican Republic, DZA − Algeria, ECU − Ecuador, EGY − Egypt, ERI − Eritrea, ESP − Spain, EST − Estonia, ETH − Ethiopia, FIN − Finland, FJI − Fiji, FRA − France, FSM − Federated States of Micronesia, G − Global, GAB − Gabon, GBR − United Kingdom, GEO − Georgia, GHA − Ghana, GIN − Guinea, GMB − The Gambia, GNB − Guinea−Bissau, GNQ − Equatorial Guinea, GRC − Greece, GRD − Grenada, GRL − Greenland, GTM − Guatemala, GUM − Guam, GUY − Guyana, HND − Honduras, HRV − Croatia, HTI − Haiti, HUN − Hungary, IDN − Indonesia, IND − India, IRL − Ireland, IRN − Iran, IRQ − Iraq, ISL − Iceland, ISR − Israel, ITA − Italy, JAM − Jamaica, JOR − Jordan, JPN − Japan, KAZ − Kazakhstan, KEN − Kenya, KGZ − Kyrgyzstan, KHM − Cambodia, KIR − Kiribati, KOR − South Korea, KWT − Kuwait, LAO − Laos, LBN − Lebanon, LBR − Liberia, LBY − Libya, LCA − Saint Lucia, LKA − Sri Lanka, LSO − Lesotho, LTU − Lithuania, LUX − Luxembourg, LVA − Latvia, MAR − Morocco, MDA − Moldova, MDG − Madagascar, MDV − Maldives, MEX − Mexico, MHL − Marshall Islands, MKD − Macedonia, MLI − Mali, MLT − Malta, MMR − Myanmar, MNE − Montenegro, MNG − Mongolia, MNP − Northern Mariana Islands, MOZ − Mozambique, MRT − Mauritania, MUS − Mauritius, MWI − Malawi, MYS − Malaysia, NAM − Namibia, NER − Niger, NGA − Nigeria, NIC − Nicaragua, NLD − Netherlands, NOR − Norway, NPL − Nepal, NZL − New Zealand, OMN − Oman, PAK − Pakistan, PAN − Panama, PER − Peru, PHL − Philippines, PNG − Papua New Guinea, POL − Poland, PRI − Puerto Rico, PRK − North Korea, PRT − Portugal, PRY − Paraguay, PSE − Palestine, QAT − Qatar, ROU − Romania, RUS − Russian Federation, RWA − Rwanda, SAU − Saudi Arabia, SDN − Sudan, SEN − Senegal, SGP − Singapore, SLB − Solomon Islands, SLE − Sierra Leone, SLV − El Salvador, SOM − Somalia, SRB − Serbia, SSD − South Sudan, STP − Sao Tome and Principe, SUR − Suriname, SVK − Slovakia, SVN − Slovenia, SWE − Sweden, SWZ − Swaziland, SYC − Seychelles, SYR − Syria, TCD − Chad, TGO − Togo, THA − Thailand, TJK − Tajikistan, TKM − Turkmenistan, TLS − Timor−Leste, TON − Tonga, TTO − Trinidad and Tobago, TUN − Tunisia, TUR − Turkey, TWN − Taiwan, TZA − Tanzania, UGA − Uganda, UKR − Ukraine, URY − Uruguay, USA − United States, UZB − Uzbekistan, VCT − Saint Vincent and the Grenadines, VEN − Venezuela, VIR − Virgin Islands, U.S., VNM − Vietnam, VUT − Vanuatu, WSM − Samoa, YEM − Yemen, ZAF − South Africa, ZMB − Zambia, ZWE − Zimbabwe

© 2019 GBD 2017 Child and Adolescent Health Collaborators. JAMA Pediatrics.

eFigure 6i. Annual percent change of observed to expected (O:E) all−cause DALY rates in <20 years from 1990 to 2000 versus 2000 to 2017

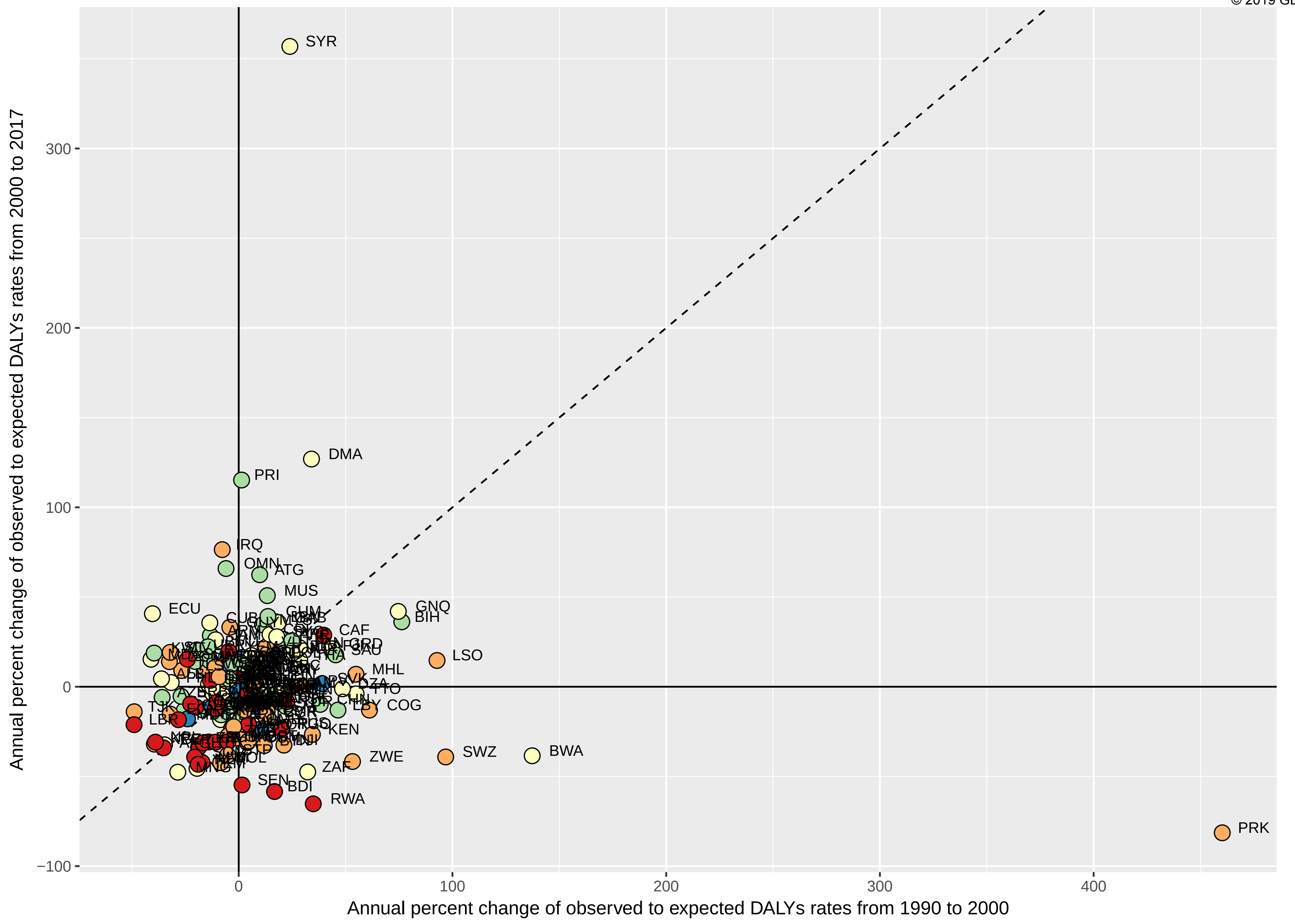

eFigure 6i. Annual percent change of observed to expected (O:E) all−cause DALY rates in <20 years from 1990 to 2000 versus 2000 to 2017 (graph subsetted to +/− 50%)

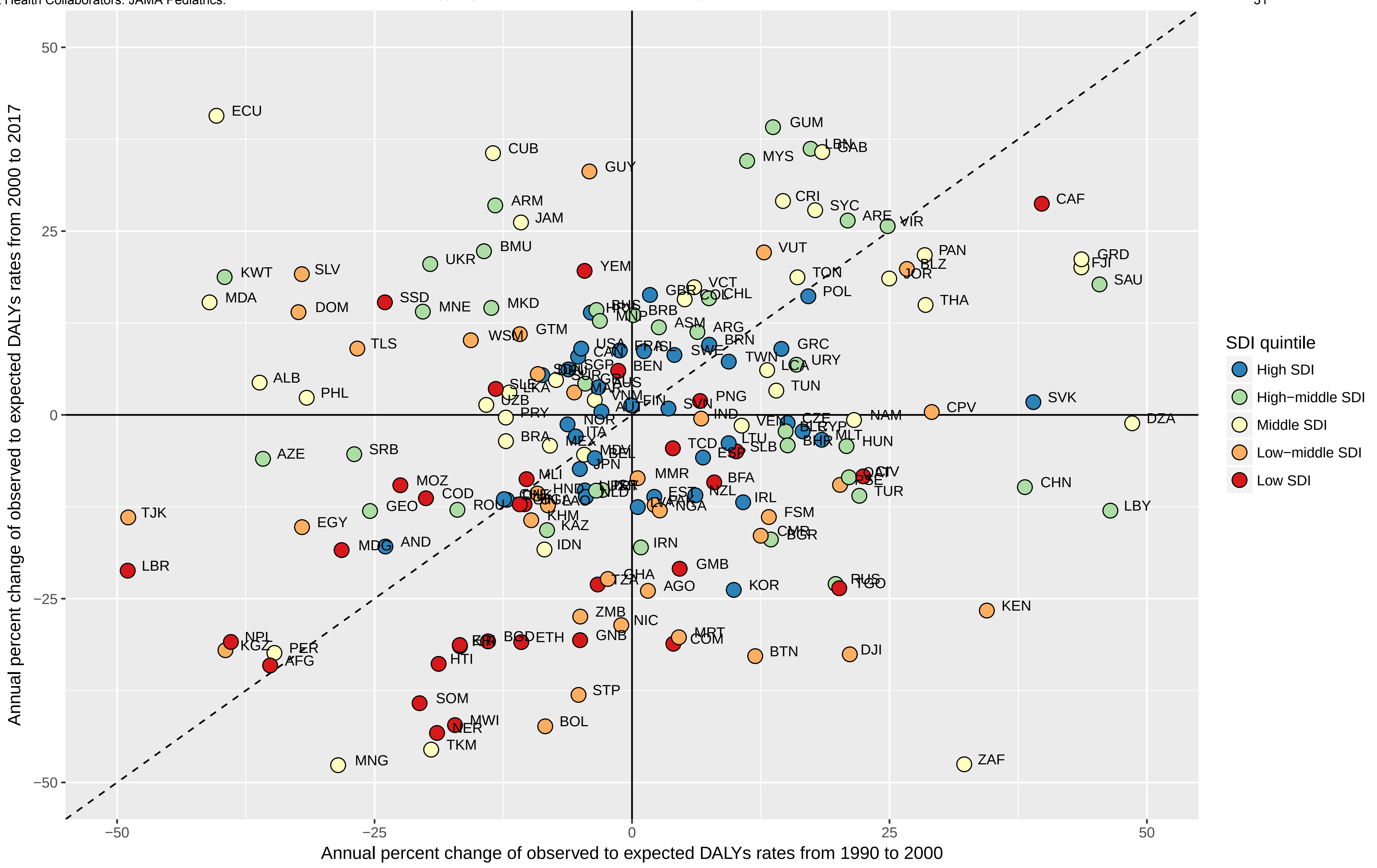

Annual percent change of observed versus expected (O:E) disability−adjusted life years (DALYs) rates by country for 1990 to 2000 versus 2000 to 2017 are plotted by country. In each pair of panels, the right panel focuses on changes within +/−50%. Panels a−d plot changes for all children and adolescents less than 20 years of age; panels e−h plot changes for children less than 1; panels i−l plot changes for children between 1 and 4; panels m−p plot changes for children between 5 and 9; and panels q−t plot changes for children between 10 and 19. For each age group, the first plot in the series is the all cause change in O:E rates (i.e., panels a, e, i, m, and q); the second plot in the series is the change in O:E rates associated with communicable, maternal, neonatal, and nutritional (CMNN) conditions (i.e., panels b, f, j, and r); the third plot in the series is the change in O:E rates associated with non−communicable diseases (NCDs – i.e., panels c, g, k, s); and the fourth plot in the series is the change in O:E rates associated with injuries (i.e., panels d, h, l, t). For each, countries in different socio−demographic index (SDI) quintiles are plotted with different countries. Abbreviations: DALY=disability−adjusted life year, AFG − Afghanistan, AGO − Angola, ALB − Albania, AND − Andorra, ARE − United Arab Emirates, ARG − Argentina, ARM − Armenia, ASM − American Samoa, ATG − Antigua and Barbuda, AUS − Australia, AUT − Austria, AZE − Azerbaijan, BDI − Burundi, BEL − Belgium, BEN − Benin, BFA − Burkina Faso, BGD − Bangladesh, BGR − Bulgaria, BHR − Bahrain, BHS − The Bahamas, BIH − Bosnia and Herzegovina, BLR − Belarus, BLZ − Belize, BMU − Bermuda, BOL − Bolivia, BRA − Brazil, BRB − Barbados, BRN − Brunei, BTN − Bhutan, BWA − Botswana, CAF − Central African Republic, CAN − Canada, CHE − Switzerland, CHL − Chile, CHN − China, CIV − Cote d'Ivoire, CMR − Cameroon, COD − Democratic Republic of the Congo, COG − Congo, COL − Colombia, COM − Comoros, CPV − Cape Verde, CRI − Costa Rica, CUB − Cuba, CYP − Cyprus, CZE − Czech Republic, DEU − Germany, DJI − Djibouti, DMA − Dominica, DNK − Denmark, DOM − Dominican Republic, DZA − Algeria, ECU − Ecuador, EGY − Egypt, ERI − Eritrea, ESP − Spain, EST − Estonia, ETH − Ethiopia, FIN − Finland, FJI − Fiji, FRA − France, FSM − Federated States of Micronesia, G − Global, GAB − Gabon, GBR − United Kingdom, GEO − Georgia, GHA − Ghana, GIN − Guinea, GMB − The Gambia, GNB − Guinea−Bissau, GNQ − Equatorial Guinea, GRC − Greece, GRD − Grenada, GRL − Greenland, GTM − Guatemala, GUM − Guam, GUY − Guyana, HND − Honduras, HRV − Croatia, HTI − Haiti, HUN − Hungary, IDN − Indonesia, IND − India, IRL − Ireland, IRN − Iran, IRQ − Iraq, ISL − Iceland, ISR − Israel, ITA − Italy, JAM − Jamaica, JOR − Jordan, JPN − Japan, KAZ − Kazakhstan, KEN − Kenya, KGZ − Kyrgyzstan, KHM − Cambodia, KIR − Kiribati, KOR − South Korea, KWT − Kuwait, LAO − Laos, LBN − Lebanon, LBR − Liberia, LBY − Libya, LCA − Saint Lucia, LKA − Sri Lanka, LSO − Lesotho, LTU − Lithuania, LUX − Luxembourg, LVA − Latvia, MAR − Morocco, MDA − Moldova, MDG − Madagascar, MDV − Maldives, MEX − Mexico, MHL − Marshall Islands, MKD − Macedonia, MLI − Mali, MLT − Malta, MMR − Myanmar, MNE − Montenegro, MNG − Mongolia, MNP − Northern Mariana Islands, MOZ − Mozambique, MRT − Mauritania, MUS − Mauritius, MWI − Malawi, MYS − Malaysia, NAM − Namibia, NER − Niger, NGA − Nigeria, NIC − Nicaragua, NLD − Netherlands, NOR − Norway, NPL − Nepal, NZL − New Zealand, OMN − Oman, PAK − Pakistan, PAN − Panama, PER − Peru, PHL − Philippines, PNG − Papua New Guinea, POL − Poland, PRI − Puerto Rico, PRK − North Korea, PRT − Portugal, PRY − Paraguay, PSE − Palestine, QAT − Qatar, ROU − Romania, RUS − Russian Federation, RWA − Rwanda, SAU − Saudi Arabia, SDN − Sudan, SEN − Senegal, SGP − Singapore, SLB − Solomon Islands, SLE − Sierra Leone, SLV − El Salvador, SOM − Somalia, SRB − Serbia, SSD − South Sudan, STP − Sao Tome and Principe, SUR − Suriname, SVK − Slovakia, SVN − Slovenia, SWE − Sweden, SWZ − Swaziland, SYC − Seychelles, SYR − Syria, TCD − Chad, TGO − Togo, THA − Thailand, TJK − Tajikistan, TKM − Turkmenistan, TLS − Timor−Leste, TON − Tonga, TTO − Trinidad and Tobago, TUN − Tunisia, TUR − Turkey, TWN − Taiwan, TZA − Tanzania, UGA − Uganda, UKR − Ukraine, URY − Uruguay, USA − United States, UZB − Uzbekistan, VCT − Saint Vincent and the Grenadines, VEN − Venezuela, VIR − Virgin Islands, U.S., VNM − Vietnam, VUT − Vanuatu, WSM − Samoa, YEM − Yemen, ZAF − South Africa, ZMB − Zambia, ZWE − Zimbabwe

© 2019 GBD 2017 Child and Adolescent Health Collaborators. JAMA Pediatrics.

eFigure 6j. Annual percent change of observed to expected (O:E) all−cause DALY rates in <20 years from 1990 to 2000 versus 2000 to 2017

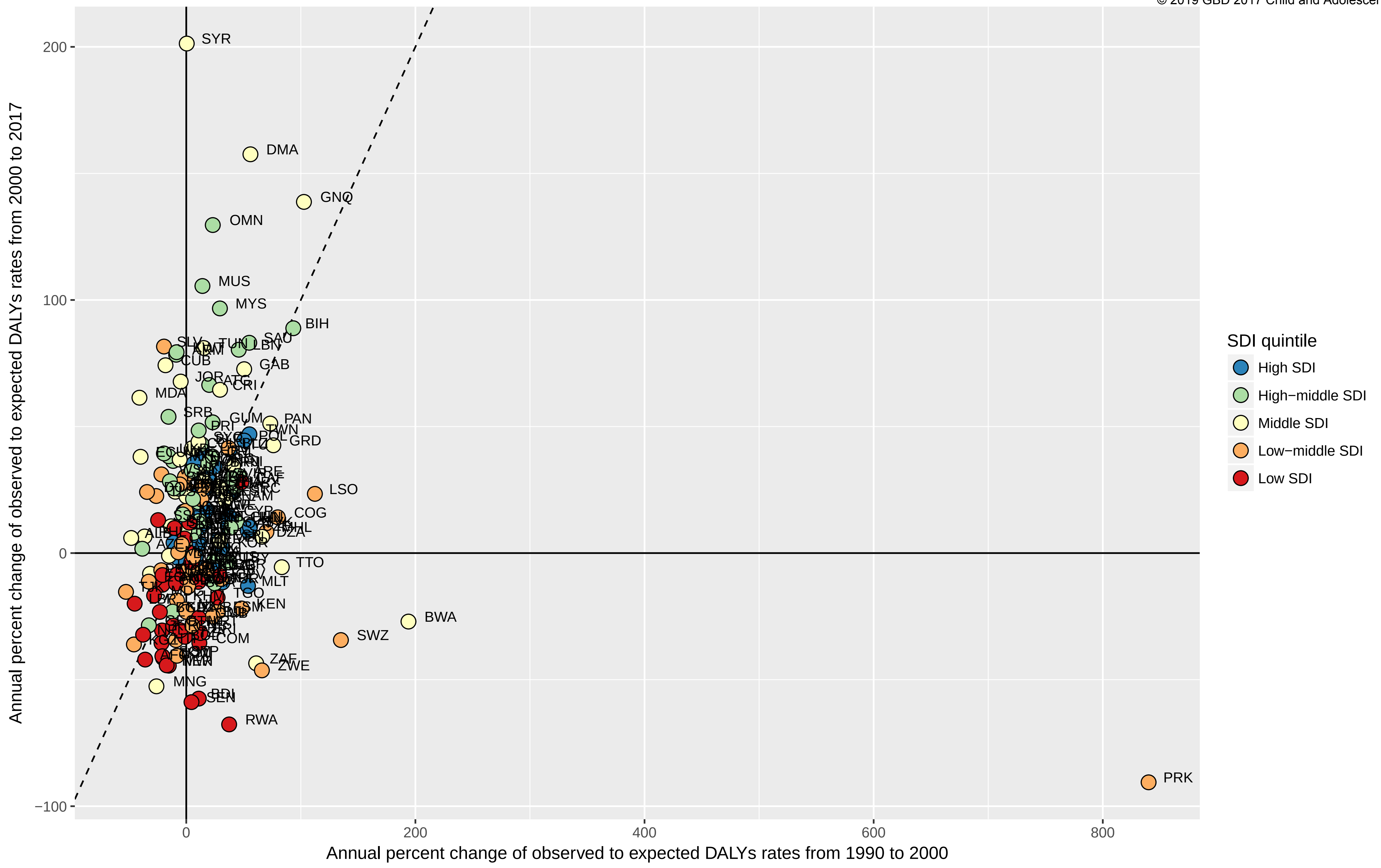

eFigure 6j. Annual percent change of observed to expected (O:E) all−cause DALY rates in <20 years from 1990 to 2000 versus 2000 to 2017 (graph subsetted to +/− 50%)

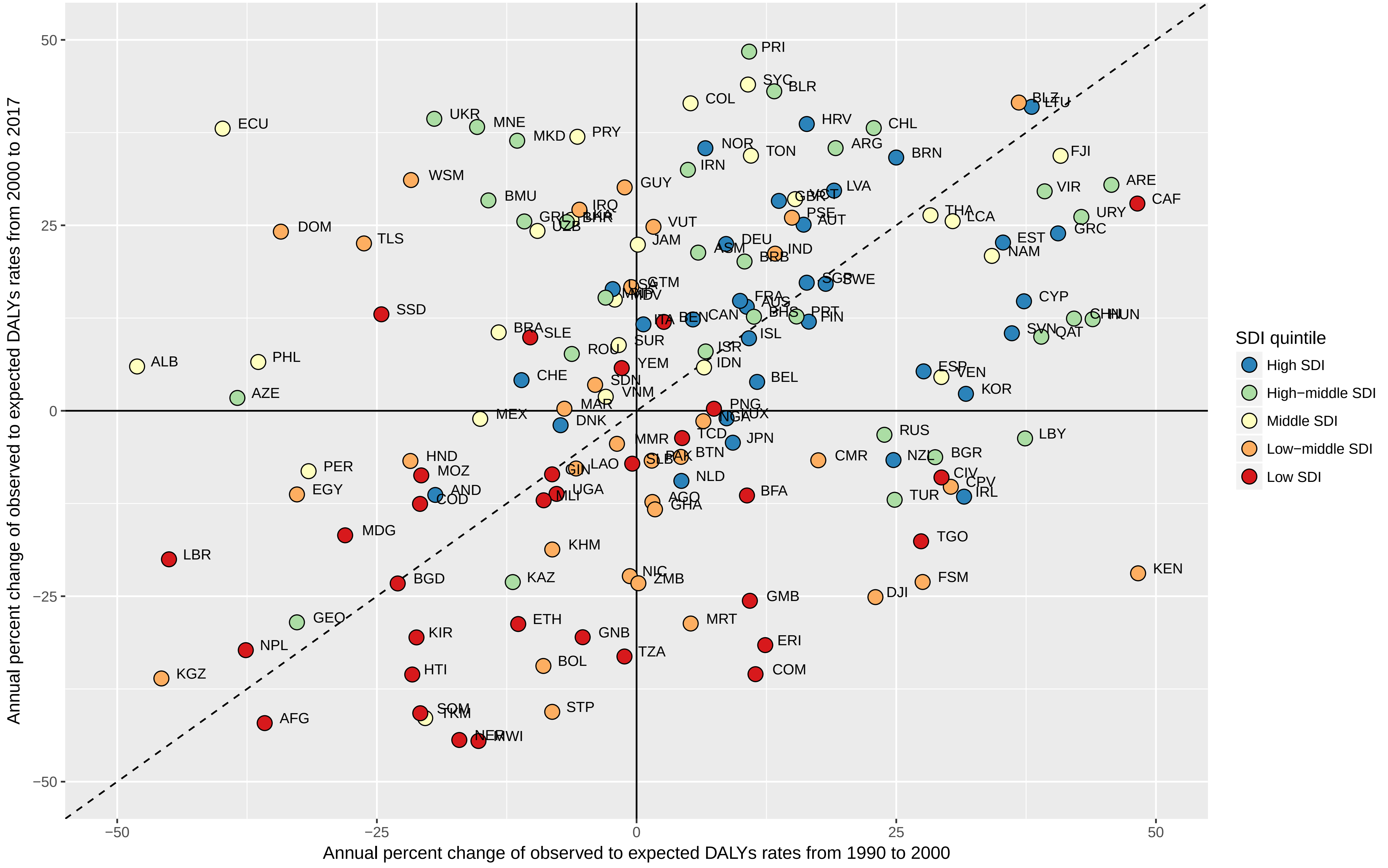

Annual percent change of observed versus expected (O:E) disability−adjusted life years (DALYs) rates by country for 1990 to 2000 versus 2000 to 2017 are plotted by country. In each pair of panels, the right panel focuses on changes within +/−50%. Panels a−d plot changes for all children and adolescents less than 20 years of age; panels e−h plot changes for children less than 1; panels i−l plot changes for children between 1 and 4; panels m−p plot changes for children between 5 and 9; and panels q−t plot changes for children between 10 and 19. For each age group, the first plot in the series is the all cause change in O:E rates (i.e., panels a, e, i, m, and q); the second plot in the series is the change in O:E rates associated with communicable, maternal, neonatal, and nutritional (CMNN) conditions (i.e., panels b, f, j, and r); the third plot in the series is the change in O:E rates associated with non−communicable diseases (NCDs – i.e., panels c, g, k, s); and the fourth plot in the series is the change in O:E rates associated with injuries (i.e., panels d, h, l, t). For each, countries in different socio−demographic index (SDI) quintiles are plotted with different colours. Abbreviations: DALY=disability−adjusted life year, AFG − Afghanistan, AGO − Angola, ALB − Albania, AND − Andorra, ARE − United Arab Emirates, ARG − Argentina, ARM − Armenia, ASM − American Samoa, ATG − Antigua and Barbuda, AUS − Australia, AUT − Austria, AZE − Azerbaijan, BDI − Burundi, BEL − Belgium, BEN − Benin, BFA − Burkina Faso, BGD − Bangladesh, BGR − Bulgaria, BHR − Bahrain, BHS − The Bahamas, BIH − Bosnia and Herzegovina, BLR − Belarus, BLZ − Belize, BMU − Bermuda, BOL − Bolivia, BRA − Brazil, BRB − Barbados, BRN − Brunei, BTN − Bhutan, BWA − Botswana, CAF − Central African Republic, CAN − Canada, CHE − Switzerland, CHL − Chile, CHN − China, CIV − Cote d'Ivoire, CMR − Cameroon, COD − Democratic Republic of the Congo, COG − Congo, COL − Colombia, COM − Comoros, CPV − Cape Verde, CRI − Costa Rica, CUB − Cuba, CYP − Cyprus, CZE − Czech Republic, DEU − Germany, DJI − Djibouti, DMA − Dominica, DNK − Denmark, DOM − Dominican Republic, DZA − Algeria, ECU − Ecuador, EGY − Egypt, ERI − Eritrea, ESP − Spain, EST − Estonia, ETH − Ethiopia, FIN − Finland, FJI − Fiji, FRA − France, FSM − Federated States of Micronesia, G − Global, GAB − Gabon, GBR − United Kingdom, GEO − Georgia, GHA − Ghana, GIN − Guinea, GMB − The Gambia, GNB − Guinea−Bissau, GNQ − Equatorial Guinea, GRC − Greece, GRD − Grenada, GRL − Greenland, GTM − Guatemala, GUM − Guam, GUY − Guyana, HND − Honduras, HRV − Croatia, HTI − Haiti, HUN − Hungary, IDN − Indonesia, IND − India, IRL − Ireland, IRN − Iran, IRQ − Iraq, ISL − Iceland, ISR − Israel, ITA − Italy, JAM − Jamaica, JOR − Jordan, JPN − Japan, KAZ − Kazakhstan, KEN − Kenya, KGZ − Kyrgyzstan, KHM − Cambodia, KIR − Kiribati, KOR − South Korea, KWT − Kuwait, LAO − Laos, LBN − Lebanon, LBR − Liberia, LBY − Libya, LCA − Saint Lucia, LKA − Sri Lanka, LSO − Lesotho, LTU − Lithuania, LUX − Luxembourg, LVA − Latvia, MAR − Morocco, MDA − Moldova, MDG − Madagascar, MDV − Maldives, MEX − Mexico, MHL − Marshall Islands, MKD − Macedonia, MLI − Mali, MLT − Malta, MMR − Myanmar, MNE − Montenegro, MNG − Mongolia, MNP − Northern Mariana Islands, MOZ − Mozambique, MRT − Mauritania, MUS − Mauritius, MWI − Malawi, MYS − Malaysia, NAM − Namibia, NER − Niger, NGA − Nigeria, NIC − Nicaragua, NLD − Netherlands, NOR − Norway, NPL − Nepal, NZL − New Zealand, OMN − Oman, PAK − Pakistan, PAN − Panama, PER − Peru, PHL − Philippines, PNG − Papua New Guinea, POL − Poland, PRI − Puerto Rico, PRK − North Korea, PRT − Portugal, PRY − Paraguay, PSE − Palestine, QAT − Qatar, ROU − Romania, RUS − Russian Federation, RWA − Rwanda, SAU − Saudi Arabia, SDN − Sudan, SEN − Senegal, SGP − Singapore, SLB − Solomon Islands, SLE − Sierra Leone, SLV − El Salvador, SOM − Somalia, SRB − Serbia, SSD − South Sudan, STP − Sao Tome and Principe, SUR − Suriname, SVK − Slovakia, SVN − Slovenia, SWE − Sweden, SWZ − Swaziland, SYC − Seychelles, SYR − Syria, TCD − Chad, TGO − Togo, THA − Thailand, TJK − Tajikistan, TKM − Turkmenistan, TLS − Timor−Leste, TON − Tonga, TTO − Trinidad and Tobago, TUN − Tunisia, TUR − Turkey, TWN − Taiwan, TZA − Tanzania, UGA − Uganda, UKR − Ukraine, URY − Uruguay, USA − United States, UZB − Uzbekistan, VCT − Saint Vincent and the Grenadines, VEN − Venezuela, VIR − Virgin Islands, U.S., VNM − Vietnam, VUT − Vanuatu, WSM − Samoa, YEM − Yemen, ZAF − South Africa, ZMB − Zambia, ZWE − Zimbabwe

© 2019 GBD 2017 Child and Adolescent Health Collaborators. JAMA Pediatrics.

eFigure 6k. Annual percent change of observed to expected (O:E) all−cause DALY rates in <20 years from 1990 to 2000 versus 2000 to 2017

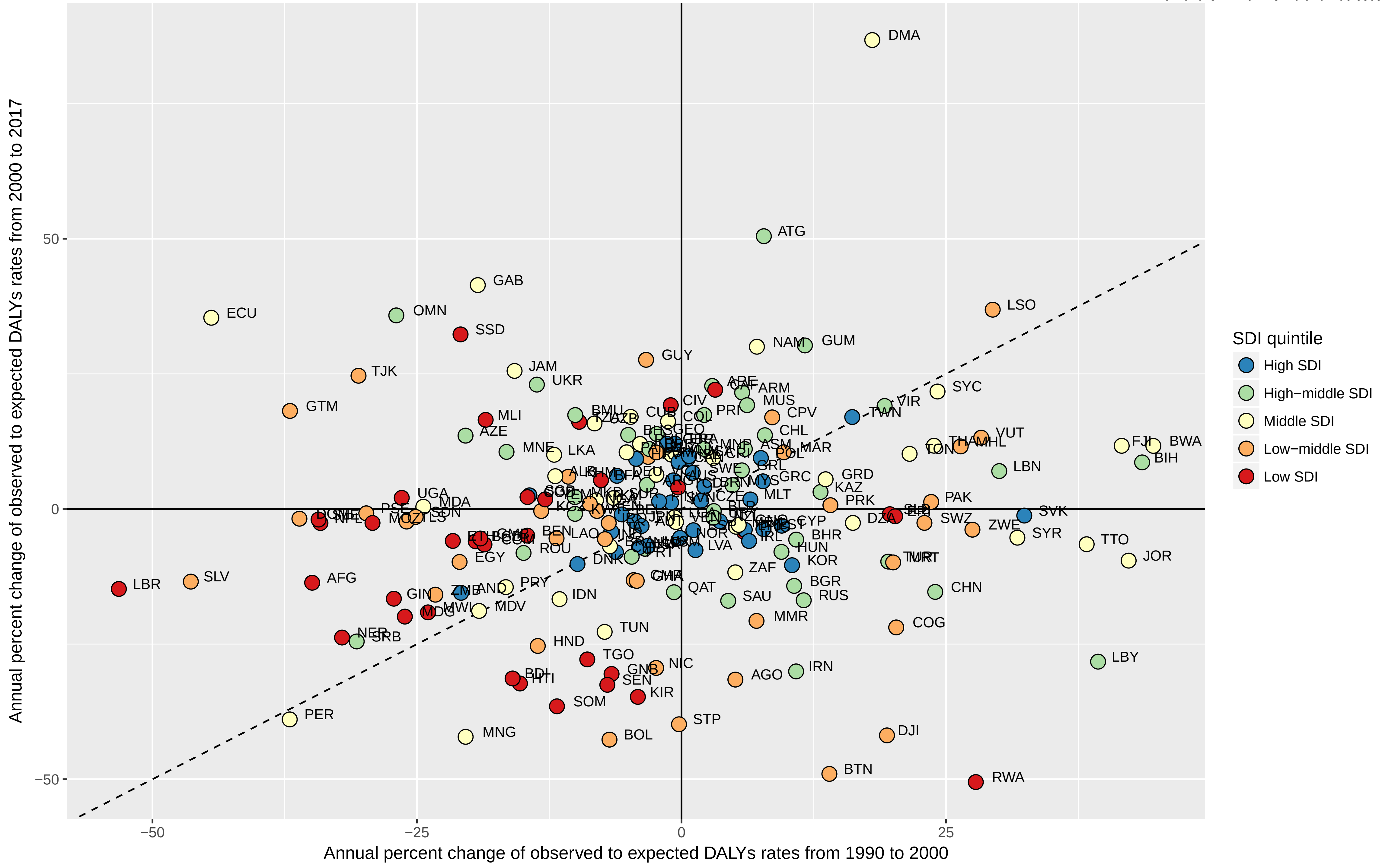

eFigure 6k. Annual percent change of observed to expected (O:E) all−cause DALY rates in <20 years from 1990 to 2000 versus 2000 to 2017 (graph subsetted to +/− 50%)

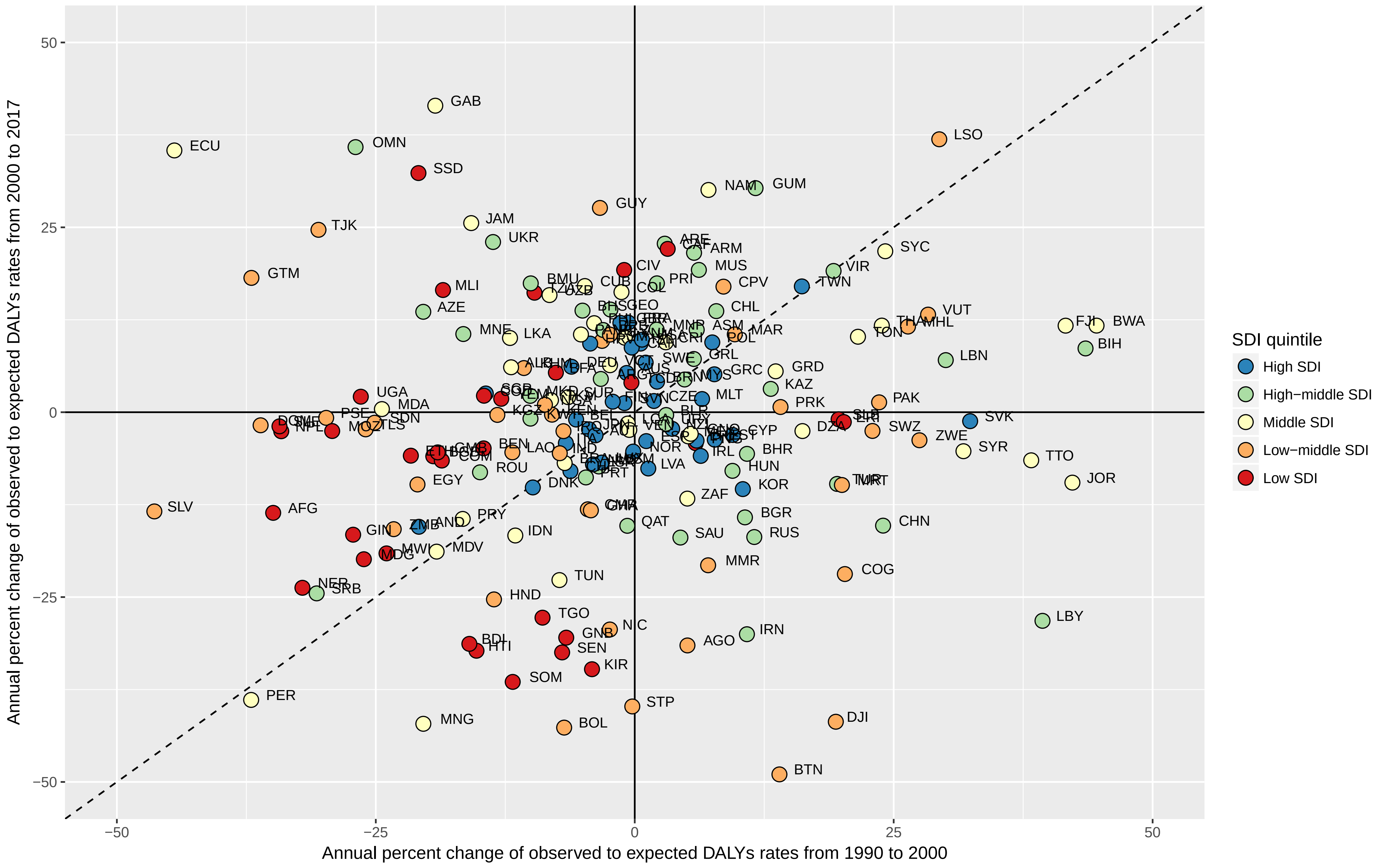

Annual percent change of observed versus expected (O:E) disability−adjusted life years (DALYs) rates by country for 1990 to 2000 versus 2000 to 2017 are plotted by country. In each pair of panels, the right panel focuses on changes within +/−50%. Panels a–d plot changes for all children and adolescents less than 20 years of age; panels e–h plot changes for children less than 1; panels i–l plot changes for children between 1 and 4; panels m–p plot changes for children between 5 and 9; and panels q–t plot changes for children between 10 and 19. For each age group, the first plot in the series is the all cause change in O:E rates (i.e., panels a, e, i, m, and q); the second plot in the series is the change in O:E rates associated with communicable, maternal, neonatal, and nutritional (CMNN) conditions (i.e., panels b, f, j, and r); the third plot in the series is the change in O:E rates associated with non−communicable diseases (NCDs – i.e., panels c, g, k, s); and the fourth plot in the series is the change in O:E rates associated with injuries (i.e., panels d, h, l, t). For each, countries in different socio−demographic index (SDI) quintiles are plotted with different countries. Abbreviations: DALY=disability−adjusted life year, AFG − Afghanistan, AGO − Angola, ALB − Albania, AND − Andorra, ARE − United Arab Emirates, ARG − Argentina, ARM − Armenia, ASM − American Samoa, ATG − Antigua and Barbuda, AUS − Australia, AUT − Austria, AZE − Azerbaijan, BDI − Burundi, BEL − Belgium, BEN − Benin, BFA − Burkina Faso, BGD − Bangladesh, BGR − Bulgaria, BHR − Bahrain, BHS − The Bahamas, BIH − Bosnia and Herzegovina, BLR − Belarus, BLZ − Belize, BMU − Bermuda, BOL − Bolivia, BRA − Brazil, BRB − Barbados, BRN − Brunei, BTN − Bhutan, BWA − Botswana, CAF − Central African Republic, CAN − Canada, CHE − Switzerland, CHL − Chile, CHN − China, CIV − Cote d'Ivoire, CMR − Cameroon, COD − Democratic Republic of the Congo, COG − Congo, COL − Colombia, COM − Comoros, CPV − Cape Verde, CRI − Costa Rica, CUB − Cuba, CYP − Cyprus, CZE − Czech Republic, DEU − Germany, DJI − Djibouti, DMA − Dominica, DNK − Denmark, DOM − Dominican Republic, DZA − Algeria, ECU − Ecuador, EGY − Egypt, ERI − Eritrea, ESP − Spain, EST − Estonia, ETH − Ethiopia, FIN − Finland, FJI − Fiji, FRA − France, FSM − Federated States of Micronesia, G − Global, GAB − Gabon, GBR − United Kingdom, GEO − Georgia, GHA − Ghana, GIN − Guinea, GMB − The Gambia, GNB − Guinea−Bissau, GNQ − Equatorial Guinea, GRC − Greece, GRD − Grenada, GRL − Greenland, GTM − Guatemala, GUM − Guam, GUY − Guyana, HND − Honduras, HRV − Croatia, HTI − Haiti, HUN − Hungary, IDN − Indonesia, IND − India, IRL − Ireland, IRN − Iran, IRQ − Iraq, ISL − Iceland, ISR − Israel, ITA − Italy, JAM − Jamaica, JOR − Jordan, JPN − Japan, KAZ − Kazakhstan, KEN − Kenya, KGZ − Kyrgyzstan, KHM − Cambodia, KIR − Kiribati, KOR − South Korea, KWT − Kuwait, LAO − Laos, LBN − Lebanon, LBR − Liberia, LBY − Libya, LCA − Saint Lucia, LKA − Sri Lanka, LSO − Lesotho, LTU − Lithuania, LUX − Luxembourg, LVA − Latvia, MAR − Morocco, MDA − Moldova, MDG − Madagascar, MDV − Maldives, MEX − Mexico, MHL − Marshall Islands, MKD − Macedonia, MLI − Mali, MLT − Malta, MMR − Myanmar, MNE − Montenegro, MNG − Mongolia, MNP − Northern Mariana Islands, MOZ − Mozambique, MRT − Mauritania, MUS − Mauritius, MWI − Malawi, MYS − Malaysia, NAM − Namibia, NER − Niger, NGA − Nigeria, NIC − Nicaragua, NLD − Netherlands, NOR − Norway, NPL − Nepal, NZL − New Zealand, OMN − Oman, PAK − Pakistan, PAN − Panama, PER − Peru, PHL − Philippines, PNG − Papua New Guinea, POL − Poland, PRI − Puerto Rico, PRK − North Korea, PRT − Portugal, PRY − Paraguay, PSE − Palestine, QAT − Qatar, ROU − Romania, RUS − Russian Federation, RWA − Rwanda, SAU − Saudi Arabia, SDN − Sudan, SEN − Senegal, SGP − Singapore, SLB − Solomon Islands, SLE − Sierra Leone, SLV − El Salvador, SOM − Somalia, SRB − Serbia, SSD − South Sudan, STP − Sao Tome and Principe, SUR − Suriname, SVK − Slovakia, SVN − Slovenia, SWE − Sweden, SWZ − Swaziland, SYC − Seychelles, SYR − Syria, TCD − Chad, TGO − Togo, THA − Thailand, TJK − Tajikistan, TKM − Turkmenistan, TLS − Timor−Leste, TON − Tonga, TTO − Trinidad and Tobago, TUN − Tunisia, TUR − Turkey, TWN − Taiwan, TZA − Tanzania, UGA − Uganda, UKR − Ukraine, URY − Uruguay, USA − United States, UZB − Uzbekistan, VCT − Saint Vincent and the Grenadines, VEN − Venezuela, VIR − Virgin Islands, U.S., VNM − Vietnam, VUT − Vanuatu, WSM − Samoa, YEM − Yemen, ZAF − South Africa, ZMB − Zambia, ZWE − Zimbabwe

© 2019 GBD 2017 Child and Adolescent Health Collaborators. JAMA Pediatrics.

eFigure 6l. Annual percent change of observed to expected (O:E) all−cause DALY rates in <20 years from 1990 to 2000 versus 2000 to 2017

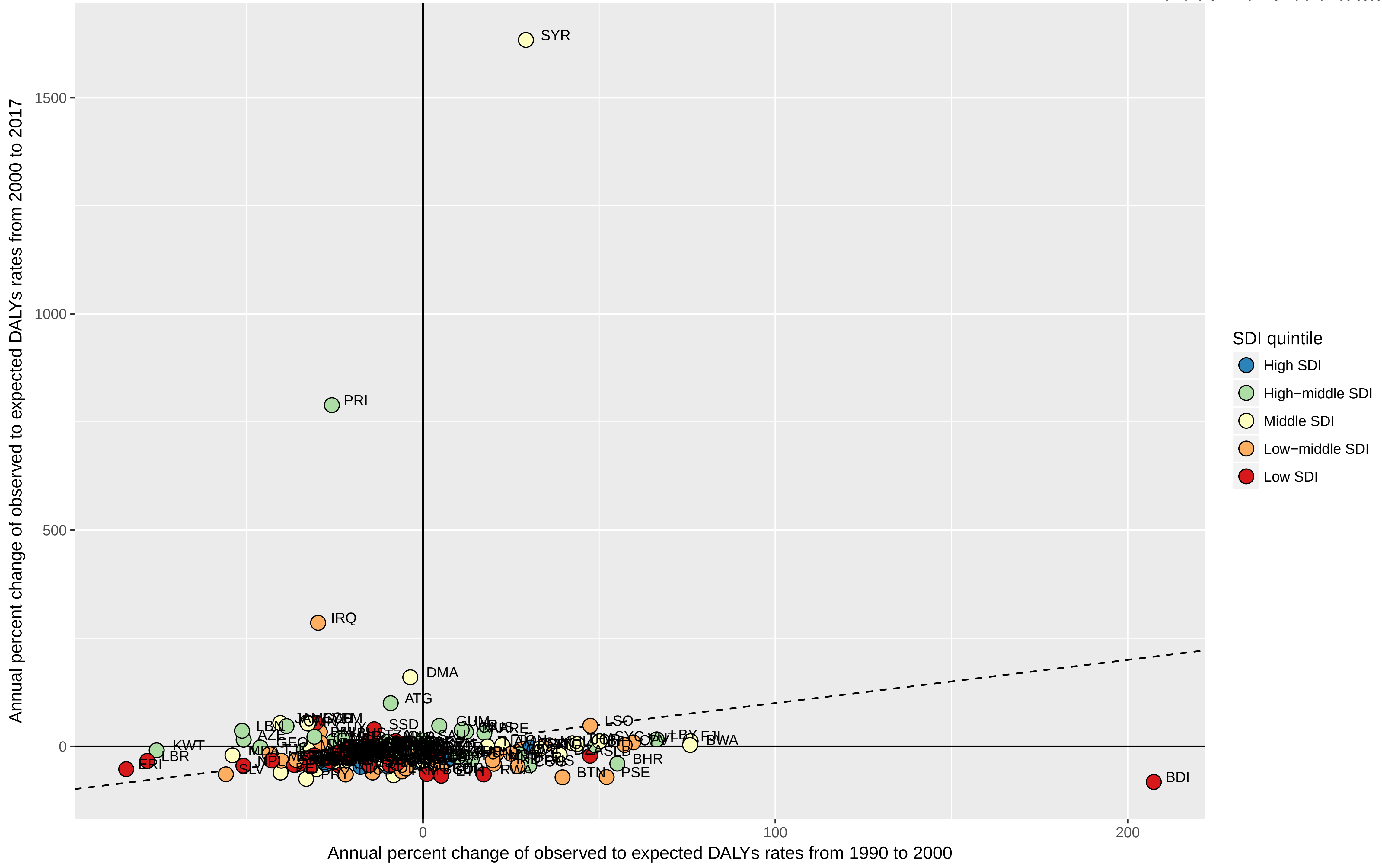

eFigure 6l. Annual percent change of observed to expected (O:E) all−cause DALY rates in <20 years from 1990 to 2000 versus 2000 to 2017 (graph subsetted to +/− 50%)

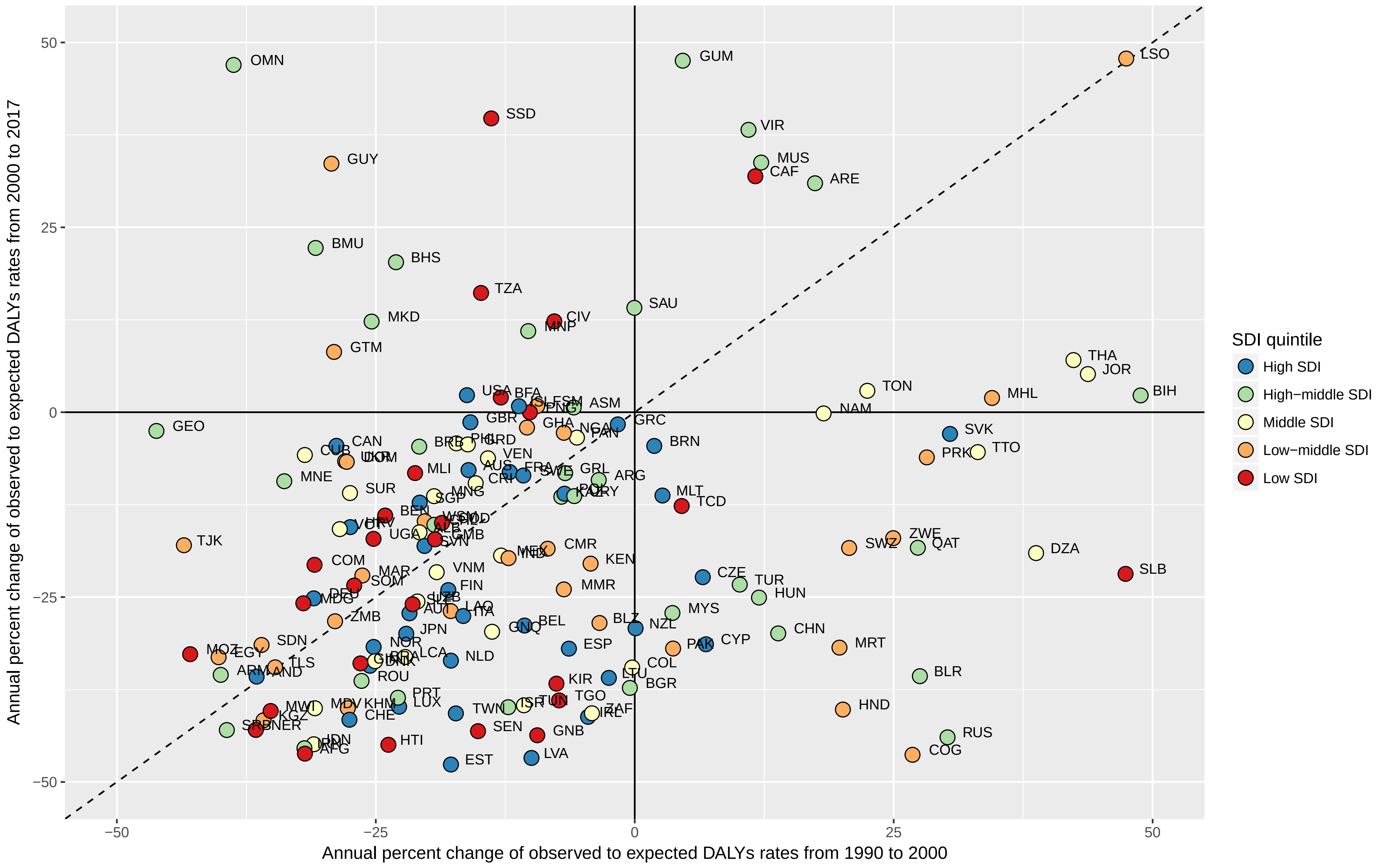

Annual percent change of observed versus expected (O:E) disability−adjusted life years (DALYs) rates by country for 1990 to 2000 versus 2000 to 2017 are plotted by country. In each pair of panels, the right panel focuses on changes within +/−50%. Panels a−d plot changes for all children and adolescents less than 20 years of age; panels e−h plot changes for children less than 1; panels i−l plot changes for children between 1 and 4; panels m−p plot changes for children between 5 and 9; and panels q−t plot changes for children between 10 and 19. For each age group, the first plot in the series is the all cause change in O:E rates (i.e., panels a, e, i, m, and q); the second plot in the series is the change in O:E rates associated with communicable, maternal, neonatal, and nutritional (CMNN) conditions (i.e., panels b, f, j, and r); the third plot in the series is the change in O:E rates associated with non−communicable diseases (NCDs – i.e., panels c, g, k, s); and the fourth plot in the series is the change in O:E rates associated with injuries (i.e., panels d, h, l, t). For each, countries in different socio−demographic index (SDI) quintiles are plotted with different countries. Abbreviations: DALY=disability−adjusted life year, AFG − Afghanistan, AGO − Angola, ALB − Albania, AND − Andorra, ARE − United Arab Emirates, ARG − Argentina, ARM − Armenia, ASM − American Samoa, ATG − Antigua and Barbuda, AUS − Australia, AUT − Austria, AZE − Azerbaijan, BDI − Burundi, BEL − Belgium, BEN − Benin, BFA − Burkina Faso, BGD − Bangladesh, BGR − Bulgaria, BHR − Bahrain, BHS − The Bahamas, BIH − Bosnia and Herzegovina, BLR − Belarus, BLZ − Belize, BMU − Bermuda, BOL − Bolivia, BRA − Brazil, BRB − Barbados, BRN − Brunei, BTN − Bhutan, BWA − Botswana, CAF − Central African Republic, CAN − Canada, CHE − Switzerland, CHL − Chile, CHN − China, CIV − Cote d'Ivoire, CMR − Cameroon, COD − Democratic Republic of the Congo, COG − Congo, COL − Colombia, COM − Comoros, CPV − Cape Verde, CRI − Costa Rica, CUB − Cuba, CYP − Cyprus, CZE − Czech Republic, DEU − Germany, DJI − Djibouti, DMA − Dominica, DNK − Denmark, DOM − Dominican Republic, DZA − Algeria, ECU − Ecuador, EGY − Egypt, ERI − Eritrea, ESP − Spain, EST − Estonia, ETH − Ethiopia, FIN − Finland, FJI − Fiji, FRA − France, FSM − Federated States of Micronesia, G − Global, GAB − Gabon, GBR − United Kingdom, GEO − Georgia, GHA − Ghana, GIN − Guinea, GMB − The Gambia, GNB − Guinea−Bissau, GNQ − Equatorial Guinea, GRC − Greece, GRD − Grenada, GRL − Greenland, GTM − Guatemala, GUM − Guam, GUY − Guyana, HND − Honduras, HRV − Croatia, HTI − Haiti, HUN − Hungary, IDN − Indonesia, IND − India, IRL − Ireland, IRN − Iran, IRQ − Iraq, ISL − Iceland, ISR − Israel, ITA − Italy, JAM − Jamaica, JOR − Jordan, JPN − Japan, KAZ − Kazakhstan, KEN − Kenya, KGZ − Kyrgyzstan, KHM − Cambodia, KIR − Kiribati, KOR − South Korea, KWT − Kuwait, LAO − Laos, LBN − Lebanon, LBR − Liberia, LBY − Libya, LCA − Saint Lucia, LKA − Sri Lanka, LSO − Lesotho, LTU − Lithuania, LUX − Luxembourg, LVA − Latvia, MAR − Morocco, MDA − Moldova, MDG − Madagascar, MDV − Maldives, MEX − Mexico, MHL − Marshall Islands, MKD − Macedonia, MLI − Mali, MLT − Malta, MMR − Myanmar, MNE − Montenegro, MNG − Mongolia, MNP − Northern Mariana Islands, MOZ − Mozambique, MRT − Mauritania, MUS − Mauritius, MWI − Malawi, MYS − Malaysia, NAM − Namibia, NER − Niger, NGA − Nigeria, NIC − Nicaragua, NLD − Netherlands, NOR − Norway, NPL − Nepal, NZL − New Zealand, OMN − Oman, PAK − Pakistan, PAN − Panama, PER − Peru, PHL − Philippines, PNG − Papua New Guinea, POL − Poland, PRI − Puerto Rico, PRK − North Korea, PRT − Portugal, PRY − Paraguay, PSE − Palestine, QAT − Qatar, ROU − Romania, RUS − Russian Federation, RWA − Rwanda, SAU − Saudi Arabia, SDN − Sudan, SEN − Senegal, SGP − Singapore, SLB − Solomon Islands, SLE − Sierra Leone, SLV − El Salvador, SOM − Somalia, SRB − Serbia, SSD − South Sudan, STP − Sao Tome and Principe, SUR − Suriname, SVK − Slovakia, SVN − Slovenia, SWE − Sweden, SWZ − Swaziland, SYC − Seychelles, SYR − Syria, TCD − Chad, TGO − Togo, THA − Thailand, TJK − Tajikistan, TKM − Turkmenistan, TLS − Timor−Leste, TON − Tonga, TTO − Trinidad and Tobago, TUN − Tunisia, TUR − Turkey, TWN − Taiwan, TZA − Tanzania, UGA − Uganda, UKR − Ukraine, URY − Uruguay, USA − United States, UZB − Uzbekistan, VCT − Saint Vincent and the Grenadines, VEN − Venezuela, VIR − Virgin Islands, U.S., VNM − Vietnam, VUT − Vanuatu, WSM − Samoa, YEM − Yemen, ZAF − South Africa, ZMB − Zambia, ZWE − Zimbabwe

© 2019 GBD 2017 Child and Adolescent Health Collaborators. JAMA Pediatrics.

eFigure 6m. Annual percent change of observed to expected (O:E) all−cause DALY rates in <20 years from 1990 to 2000 versus 2000 to 2017

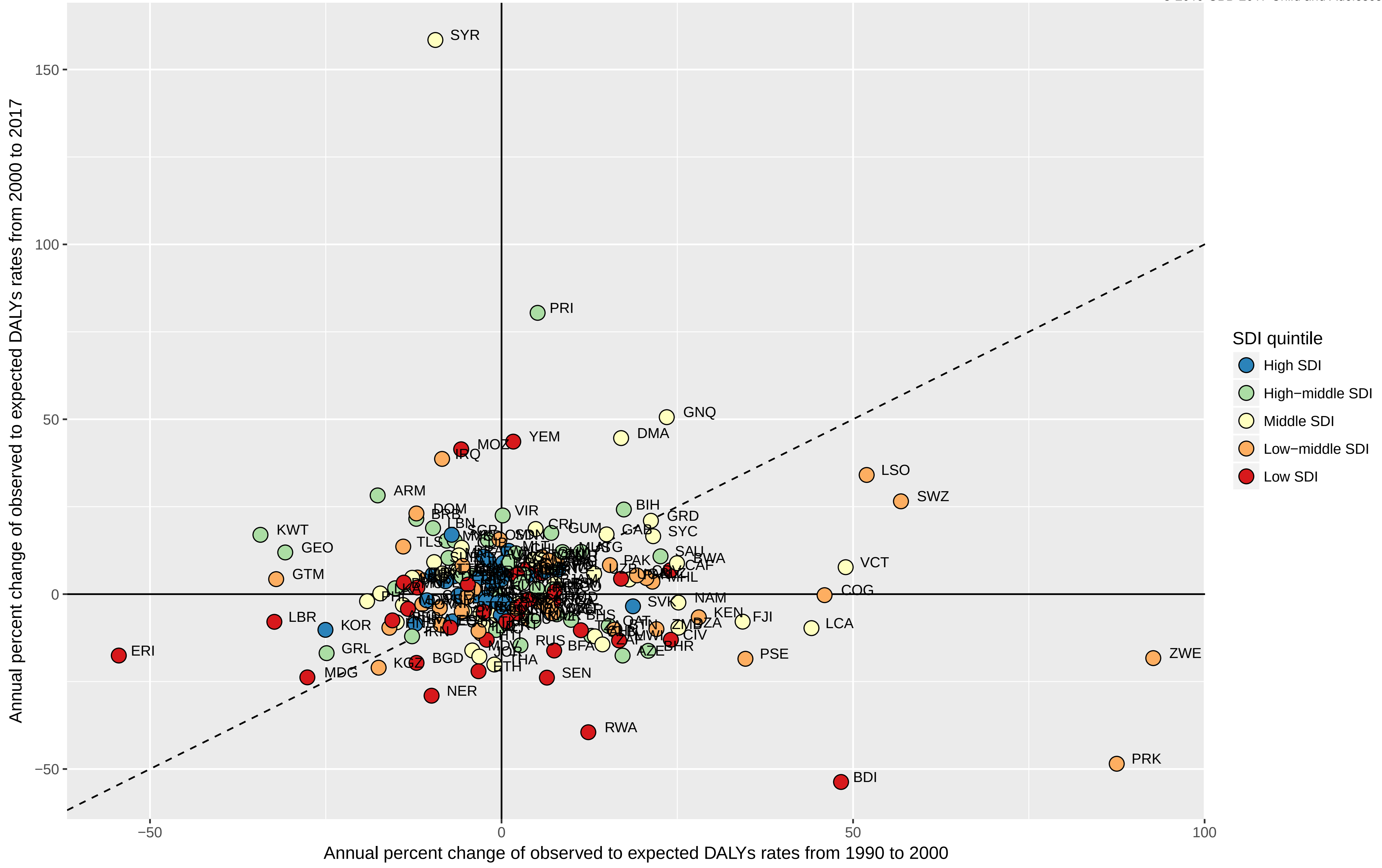

eFigure 6m. Annual percent change of observed to expected (O:E) all−cause DALY rates in <20 years from 1990 to 2000 versus 2000 to 2017 (graph subsetted to +/− 50%)

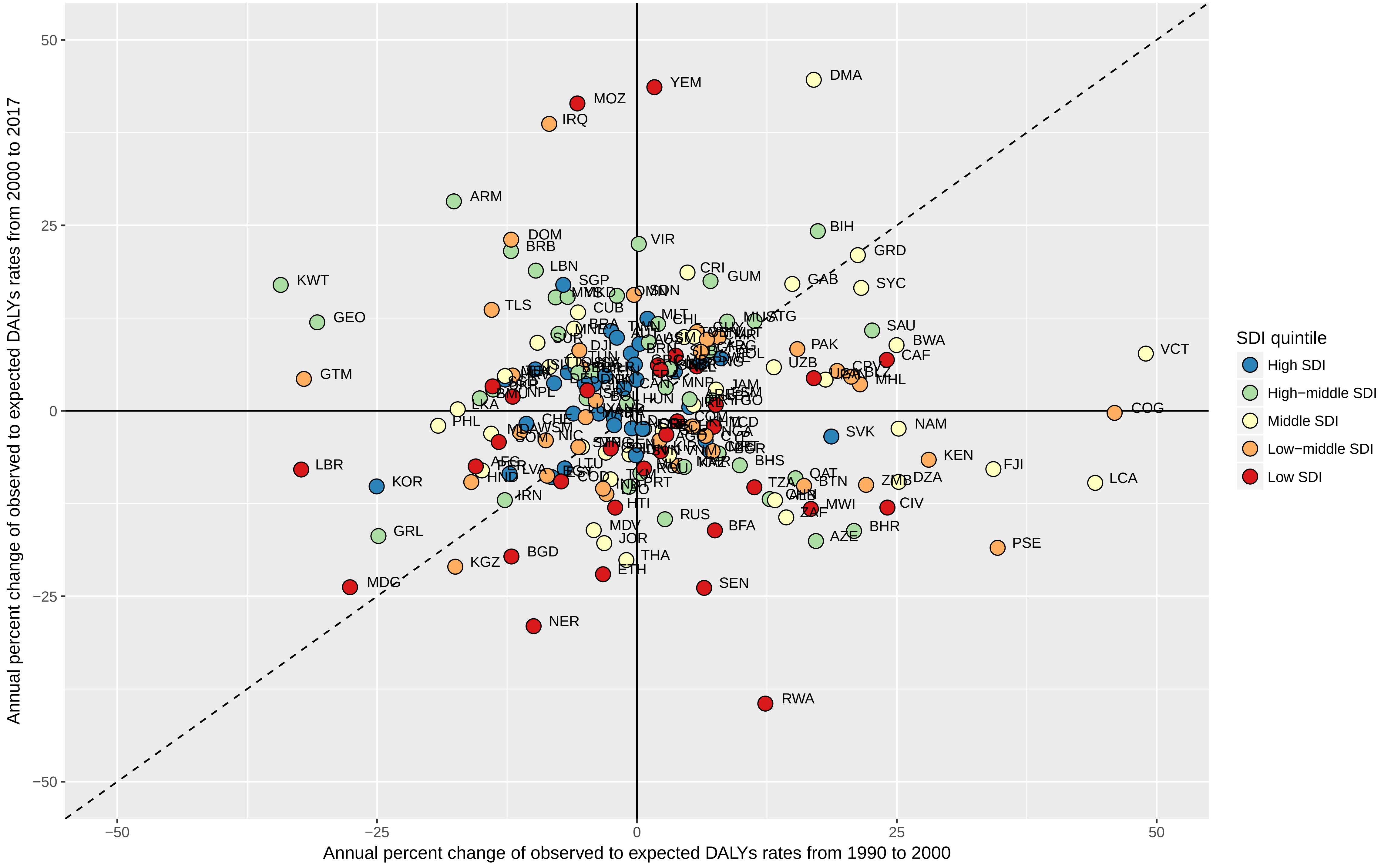

Annual percent change of observed versus expected (O:E) disability−adjusted life years (DALYs) rates by country for 1990 to 2000 versus 2000 to 2017 are plotted by country. In each pair of panels, the right panel focuses on changes within +/−50%. Panels a−d plot changes for all children and adolescents less than 20 years of age; panels e−h plot changes for children less than 1; panels i−l plot changes for children between 1 and 4; panels m−p plot changes for children between 5 and 9; and panels q−t plot changes for children between 10 and 19. For each age group, the first plot in the series is the all cause change in O:E rates (i.e., panels a, e, i, m, and q); the second plot in the series is the change in O:E rates associated with communicable, maternal, neonatal, and nutritional (CMNN) conditions (i.e., panels b, f, j, and r); the third plot in the series is the change in O:E rates associated with non−communicable diseases (NCDs – i.e., panels c, g, k, s); and the fourth plot in the series is the change in O:E rates associated with injuries (i.e., panels d, h, l, t). For each, countries in different socio−demographic index (SDI) quintiles are plotted with different countries. Abbreviations: DALY=disability−adjusted life year, AFG − Afghanistan, AGO − Angola, ALB − Albania, AND − Andorra, ARE − United Arab Emirates, ARG − Argentina, ARM − Armenia, ASM − American Samoa, ATG − Antigua and Barbuda, AUS − Australia, AUT − Austria, AZE − Azerbaijan, BDI − Burundi, BEL − Belgium, BEN − Benin, BFA − Burkina Faso, BGD − Bangladesh, BGR − Bulgaria, BHR − Bahrain, BHS − The Bahamas, BIH − Bosnia and Herzegovina, BLR − Belarus, BLZ − Belize, BMU − Bermuda, BOL − Bolivia, BRA − Brazil, BRB − Barbados, BRN − Brunei, BTN − Bhutan, BWA − Botswana, CAF − Central African Republic, CAN − Canada, CHE − Switzerland, CHL − Chile, CHN − China, CIV − Cote d'Ivoire, CMR − Cameroon, COD − Democratic Republic of the Congo, COG − Congo, COL − Colombia, COM − Comoros, CPV − Cape Verde, CRI − Costa Rica, CUB − Cuba, CYP − Cyprus, CZE − Czech Republic, DEU − Germany, DJI − Djibouti, DMA − Dominica, DNK − Denmark, DOM − Dominican Republic, DZA − Algeria, ECU − Ecuador, EGY − Egypt, ERI − Eritrea, ESP − Spain, EST − Estonia, ETH − Ethiopia, FIN − Finland, FJI − Fiji, FRA − France, FSM − Federated States of Micronesia, G − Global, GAB − Gabon, GBR − United Kingdom, GEO − Georgia, GHA − Ghana, GIN − Guinea, GMB − The Gambia, GNB − Guinea−Bissau, GNQ − Equatorial Guinea, GRC − Greece, GRD − Grenada, GRL − Greenland, GTM − Guatemala, GUM − Guam, GUY − Guyana, HND − Honduras, HRV − Croatia, HTI − Haiti, HUN − Hungary, IDN − Indonesia, IND − India, IRL − Ireland, IRN − Iran, IRQ − Iraq, ISL − Iceland, ISR − Israel, ITA − Italy, JAM − Jamaica, JOR − Jordan, JPN − Japan, KAZ − Kazakhstan, KEN − Kenya, KGZ − Kyrgyzstan, KHM − Cambodia, KIR − Kiribati, KOR − South Korea, KWT − Kuwait, LAO − Laos, LBN − Lebanon, LBR − Liberia, LBY − Libya, LCA − Saint Lucia, LKA − Sri Lanka, LSO − Lesotho, LTU − Lithuania, LUX − Luxembourg, LVA − Latvia, MAR − Morocco, MDA − Moldova, MDG − Madagascar, MDV − Maldives, MEX − Mexico, MHL − Marshall Islands, MKD − Macedonia, MLI − Mali, MLT − Malta, MMR − Myanmar, MNE − Montenegro, MNG − Mongolia, MNP − Northern Mariana Islands, MOZ − Mozambique, MRT − Mauritania, MUS − Mauritius, MWI − Malawi, MYS − Malaysia, NAM − Namibia, NER − Niger, NGA − Nigeria, NIC − Nicaragua, NLD − Netherlands, NOR − Norway, NPL − Nepal, NZL − New Zealand, OMN − Oman, PAK − Pakistan, PAN − Panama, PER − Peru, PHL − Philippines, PNG − Papua New Guinea, POL − Poland, PRI − Puerto Rico, PRK − North Korea, PRT − Portugal, PRY − Paraguay, PSE − Palestine, QAT − Qatar, ROU − Romania, RUS − Russian Federation, RWA − Rwanda, SAU − Saudi Arabia, SDN − Sudan, SEN − Senegal, SGP − Singapore, SLB − Solomon Islands, SLE − Sierra Leone, SLV − El Salvador, SOM − Somalia, SRB − Serbia, SSD − South Sudan, STP − Sao Tome and Principe, SUR − Suriname, SVK − Slovakia, SVN − Slovenia, SWE − Sweden, SWZ − Swaziland, SYC − Seychelles, SYR − Syria, TCD − Chad, TGO − Togo, THA − Thailand, TJK − Tajikistan, TKM − Turkmenistan, TLS − Timor−Leste, TON − Tonga, TTO − Trinidad and Tobago, TUN − Tunisia, TUR − Turkey, TWN − Taiwan, TZA − Tanzania, UGA − Uganda, UKR − Ukraine, URY − Uruguay, USA − United States, UZB − Uzbekistan, VCT − Saint Vincent and the Grenadines, VEN − Venezuela, VIR − Virgin Islands, U.S., VNM − Vietnam, VUT − Vanuatu, WSM − Samoa, YEM − Yemen, ZAF − South Africa, ZMB − Zambia, ZWE − Zimbabwe

© 2019 GBD 2017 Child and Adolescent Health Collaborators. JAMA Pediatrics.

eFigure 6n. Annual percent change of observed to expected (O:E) all−cause DALY rates in <20 years from 1990 to 2000 versus 2000 to 2017

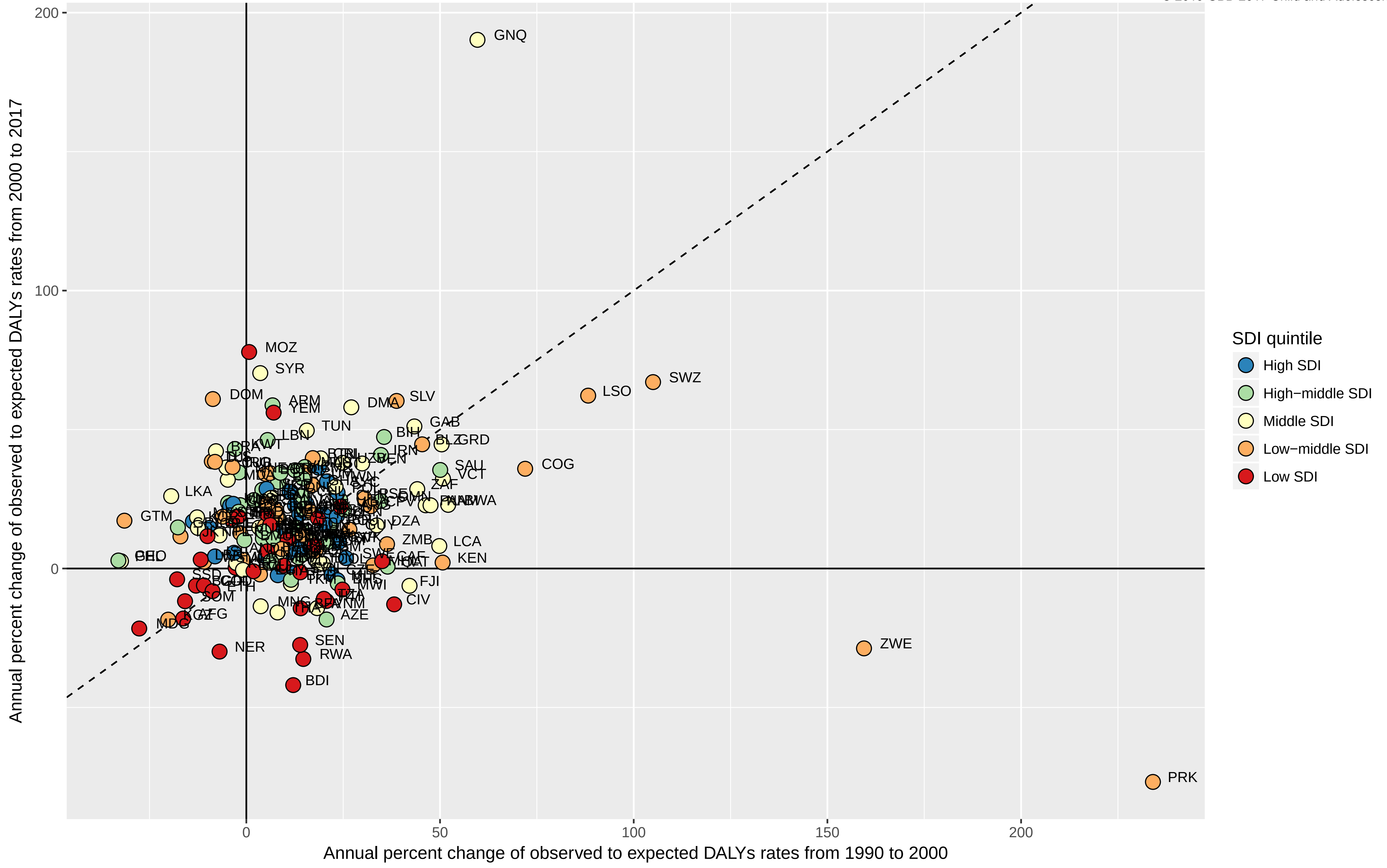

eFigure 6n. Annual percent change of observed to expected (O:E) all−cause DALY rates in <20 years from 1990 to 2000 versus 2000 to 2017 (graph subsetted to +/− 50%)

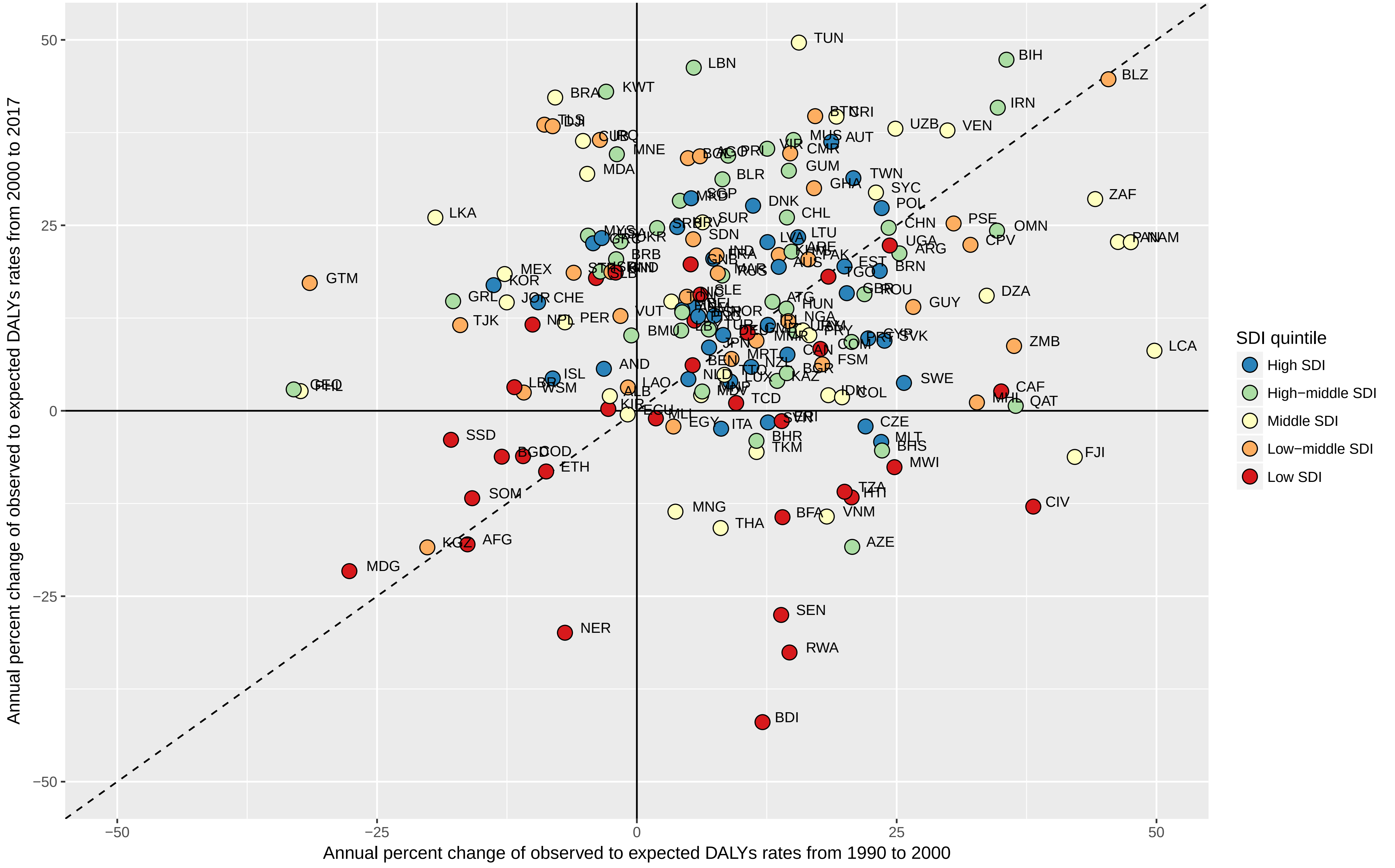

Annual percent change of observed versus expected (O:E) disability−adjusted life years (DALYs) rates by country for 1990 to 2000 versus 2000 to 2017 are plotted by country. In each pair of panels, the right panel focuses on changes within +/−50%. Panels a−d plot changes for all children and adolescents less than 20 years of age; panels e−h plot changes for children less than 1; panels i−l plot changes for children between 1 and 4; panels m−p plot changes for children between 5 and 9; and panels q−t plot changes for children between 10 and 19. For each age group, the first plot in the series is the all cause change in O:E rates (i.e., panels a, e, i, m, and q); the second plot in the series is the change in O:E rates associated with communicable, maternal, neonatal, and nutritional (CMNN) conditions (i.e., panels b, f, j, and r); the third plot in the series is the change in O:E rates associated with non−communicable diseases (NCDs – i.e., panels c, g, k, s); and the fourth plot in the series is the change in O:E rates associated with injuries (i.e., panels d, h, l, t). For each, countries in different socio−demographic index (SDI) quintiles are plotted with different countries. Abbreviations: DALY=disability−adjusted life year, AFG − Afghanistan, AGO − Angola, ALB − Albania, AND − Andorra, ARE − United Arab Emirates, ARG − Argentina, ARM − Armenia, ASM − American Samoa, ATG − Antigua and Barbuda, AUS − Australia, AUT − Austria, AZE − Azerbaijan, BDI − Burundi, BEL − Belgium, BEN − Benin, BFA − Burkina Faso, BGD − Bangladesh, BGR − Bulgaria, BHR − Bahrain, BHS − The Bahamas, BIH − Bosnia and Herzegovina, BLR − Belarus, BLZ − Belize, BMU − Bermuda, BOL − Bolivia, BRA − Brazil, BRB − Barbados, BRN − Brunei, BTN − Bhutan, BWA − Botswana, CAF − Central African Republic, CAN − Canada, CHE − Switzerland, CHL − Chile, CHN − China, CIV − Cote d'Ivoire, CMR − Cameroon, COD − Democratic Republic of the Congo, COG − Congo, COL − Colombia, COM − Comoros, CPV − Cape Verde, CRI − Costa Rica, CUB − Cuba, CYP − Cyprus, CZE − Czech Republic, DEU − Germany, DJI − Djibouti, DMA − Dominica, DNK − Denmark, DOM − Dominican Republic, DZA − Algeria, ECU − Ecuador, EGY − Egypt, ERI − Eritrea, ESP − Spain, EST − Estonia, ETH − Ethiopia, FIN − Finland, FJI − Fiji, FRA − France, FSM − Federated States of Micronesia, G − Global, GAB − Gabon, GBR − United Kingdom, GEO − Georgia, GHA − Ghana, GIN − Guinea, GMB − The Gambia, GNB − Guinea−Bissau, GNQ − Equatorial Guinea, GRC − Greece, GRD − Grenada, GRL − Greenland, GTM − Guatemala, GUM − Guam, GUY − Guyana, HND − Honduras, HRV − Croatia, HTI − Haiti, HUN − Hungary, IDN − Indonesia, IND − India, IRL − Ireland, IRN − Iran, IRQ − Iraq, ISL − Iceland, ISR − Israel, ITA − Italy, JAM − Jamaica, JOR − Jordan, JPN − Japan, KAZ − Kazakhstan, KEN − Kenya, KGZ − Kyrgyzstan, KHM − Cambodia, KIR − Kiribati, KOR − South Korea, KWT − Kuwait, LAO − Laos, LBN − Lebanon, LBR − Liberia, LBY − Libya, LCA − Saint Lucia, LKA − Sri Lanka, LSO − Lesotho, LTU − Lithuania, LUX − Luxembourg, LVA − Latvia, MAR − Morocco, MDA − Moldova, MDG − Madagascar, MDV − Maldives, MEX − Mexico, MHL − Marshall Islands, MKD − Macedonia, MLI − Mali, MLT − Malta, MMR − Myanmar, MNE − Montenegro, MNG − Mongolia, MNP − Northern Mariana Islands, MOZ − Mozambique, MRT − Mauritania, MUS − Mauritius, MWI − Malawi, MYS − Malaysia, NAM − Namibia, NER − Niger, NGA − Nigeria, NIC − Nicaragua, NLD − Netherlands, NOR − Norway, NPL − Nepal, NZL − New Zealand, OMN − Oman, PAK − Pakistan, PAN − Panama, PER − Peru, PHL − Philippines, PNG − Papua New Guinea, POL − Poland, PRI − Puerto Rico, PRK − North Korea, PRT − Portugal, PRY − Paraguay, PSE − Palestine, QAT − Qatar, ROU − Romania, RUS − Russian Federation, RWA − Rwanda, SAU − Saudi Arabia, SDN − Sudan, SEN − Senegal, SGP − Singapore, SLB − Solomon Islands, SLE − Sierra Leone, SLV − El Salvador, SOM − Somalia, SRB − Serbia, SSD − South Sudan, STP − Sao Tome and Principe, SUR − Suriname, SVK − Slovakia, SVN − Slovenia, SWE − Sweden, SWZ − Swaziland, SYC − Seychelles, SYR − Syria, TCD − Chad, TGO − Togo, THA − Thailand, TJK − Tajikistan, TKM − Turkmenistan, TLS − Timor−Leste, TON − Tonga, TTO − Trinidad and Tobago, TUN − Tunisia, TUR − Turkey, TWN − Taiwan, TZA − Tanzania, UGA − Uganda, UKR − Ukraine, URY − Uruguay, USA − United States, UZB − Uzbekistan, VCT − Saint Vincent and the Grenadines, VEN − Venezuela, VIR − Virgin Islands, U.S., VNM − Vietnam, VUT − Vanuatu, WSM − Samoa, YEM − Yemen, ZAF − South Africa, ZMB − Zambia, ZWE − Zimbabwe

© 2019 GBD 2017 Child and Adolescent Health Collaborators. JAMA Pediatrics.

eFigure 6o. Annual percent change of observed to expected (O:E) all−cause DALY rates in <20 years from 1990 to 2000 versus 2000 to 2017

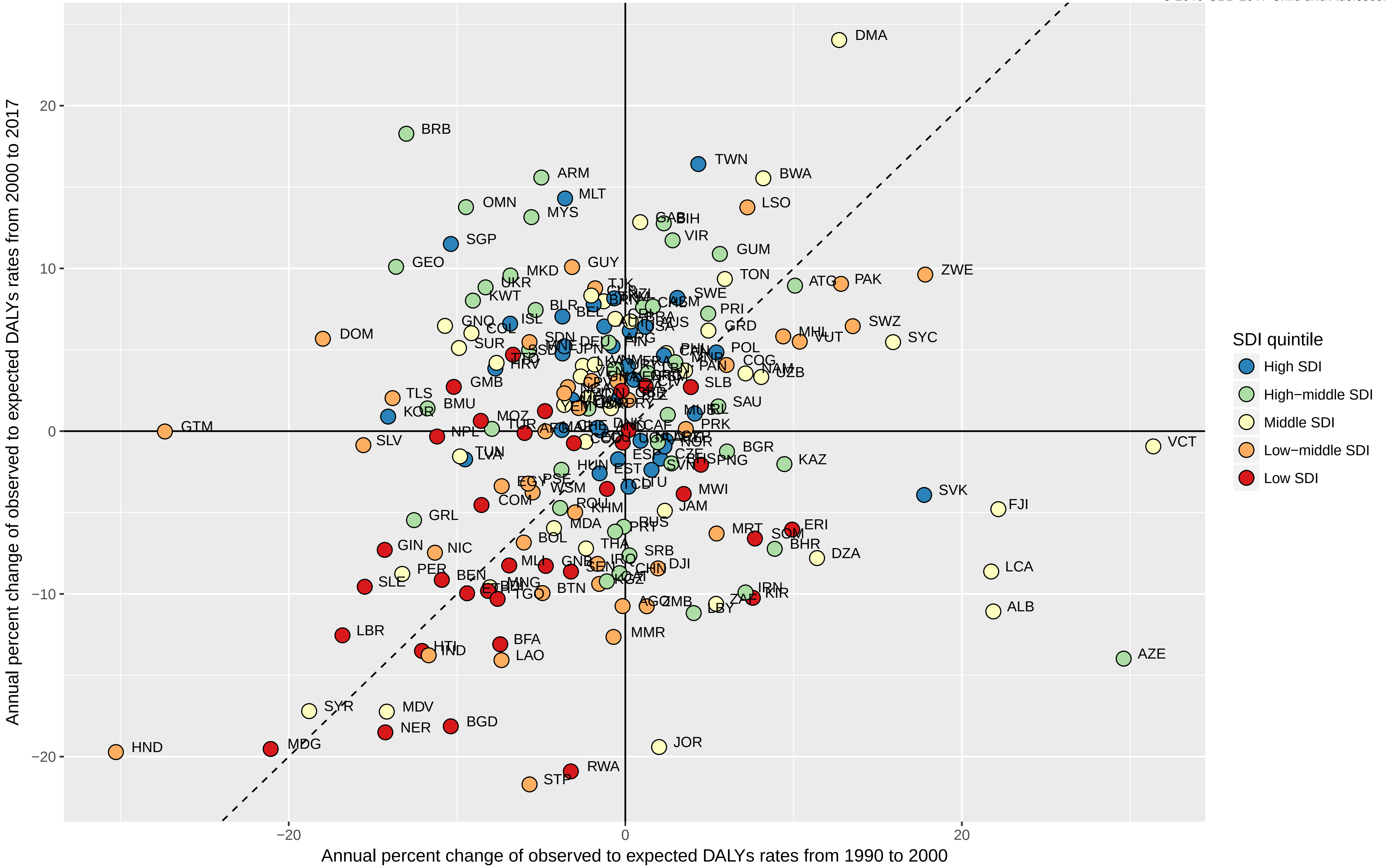

eFigure 6o. Annual percent change of observed to expected (O:E) all−cause DALY rates in <20 years from 1990 to 2000 versus 2000 to 2017 (graph subsetted to +/− 50%)

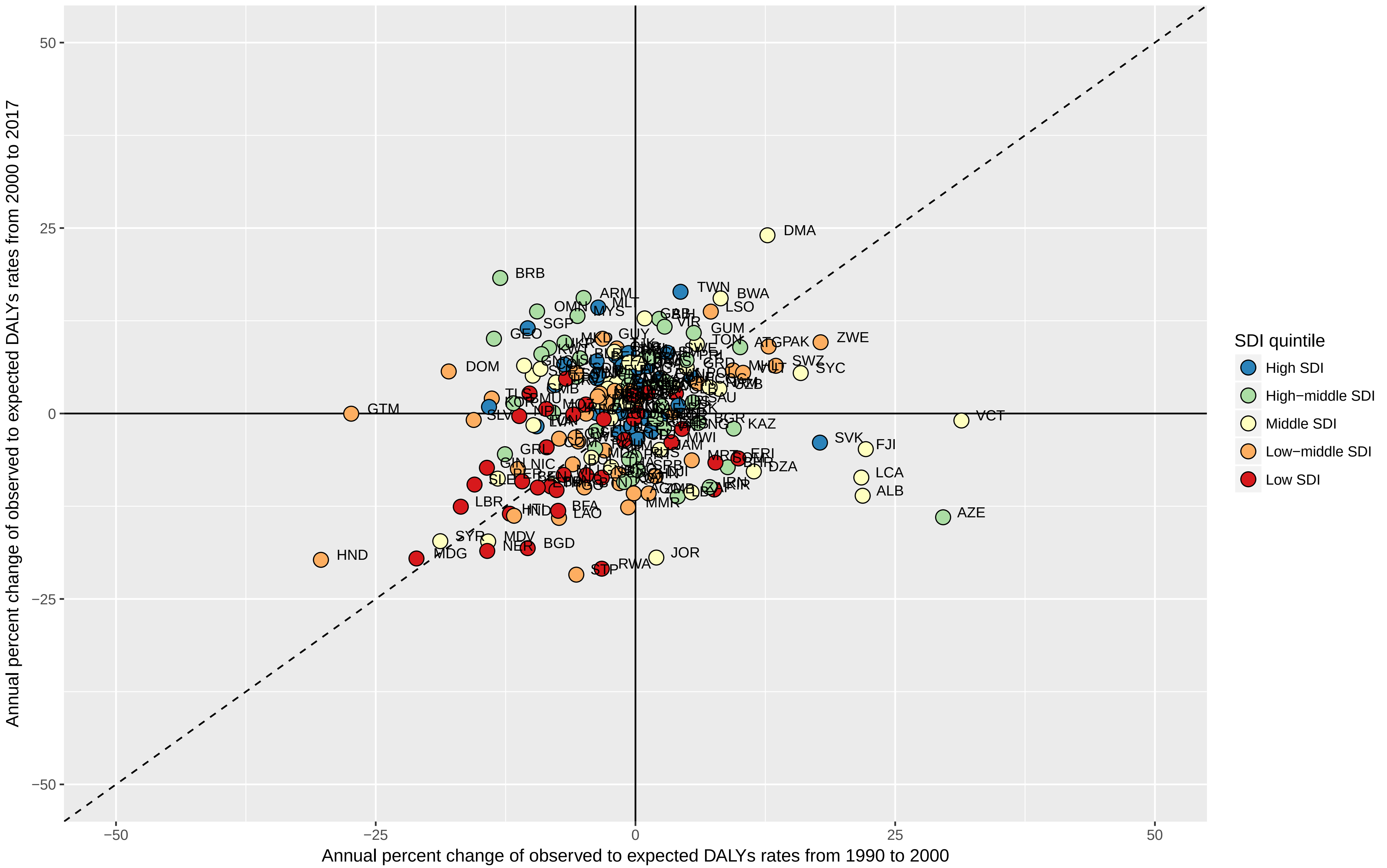

Annual percent change of observed versus expected (O:E) disability−adjusted life years (DALYs) rates by country for 1990 to 2000 versus 2000 to 2017 are plotted by country. In each pair of panels, the right panel focuses on changes within +/−50%. Panels a−d plot changes for all children and adolescents less than 20 years of age; panels e−h plot changes for children less than 1; panels i−l plot changes for children between 1 and 4; panels m−p plot changes for children between 5 and 9; and panels q−t plot changes for children between 10 and 19. For each age group, the first plot in the series is the all cause change in O:E rates (i.e., panels a, e, i, m, and q); the second plot in the series is the change in O:E rates associated with communicable, maternal, neonatal, and nutritional (CMNN) conditions (i.e., panels b, f, j, and r); the third plot in the series is the change in O:E rates associated with non−communicable diseases (NCDs – i.e., panels c, g, k, s); and the fourth plot in the series is the change in O:E rates associated with injuries (i.e., panels d, h, l, t). For each, countries in different socio−demographic index (SDI) quintiles are plotted with different countries. Abbreviations: DALY=disability−adjusted life year, AFG − Afghanistan, AGO − Angola, ALB − Albania, AND − Andorra, ARE − United Arab Emirates, ARG − Argentina, ARM − Armenia, ASM − American Samoa, ATG − Antigua and Barbuda, AUS − Australia, AUT − Austria, AZE − Azerbaijan, BDI − Burundi, BEL − Belgium, BEN − Benin, BFA − Burkina Faso, BGD − Bangladesh, BGR − Bulgaria, BHR − Bahrain, BHS − The Bahamas, BIH − Bosnia and Herzegovina, BLR − Belarus, BLZ − Belize, BMU − Bermuda, BOL − Bolivia, BRA − Brazil, BRB − Barbados, BRN − Brunei, BTN − Bhutan, BWA − Botswana, CAF − Central African Republic, CAN − Canada, CHE − Switzerland, CHL − Chile, CHN − China, CIV − Cote d'Ivoire, CMR − Cameroon, COD − Democratic Republic of the Congo, COG − Congo, COL − Colombia, COM − Comoros, CPV − Cape Verde, CRI − Costa Rica, CUB − Cuba, CYP − Cyprus, CZE − Czech Republic, DEU − Germany, DJI − Djibouti, DMA − Dominica, DNK − Denmark, DOM − Dominican Republic, DZA − Algeria, ECU − Ecuador, EGY − Egypt, ERI − Eritrea, ESP − Spain, EST − Estonia, ETH − Ethiopia, FIN − Finland, FJI − Fiji, FRA − France, FSM − Federated States of Micronesia, G − Global, GAB − Gabon, GBR − United Kingdom, GEO − Georgia, GHA − Ghana, GIN − Guinea, GMB − The Gambia, GNB − Guinea−Bissau, GNQ − Equatorial Guinea, GRC − Greece, GRD − Grenada, GRL − Greenland, GTM − Guatemala, GUM − Guam, GUY − Guyana, HND − Honduras, HRV − Croatia, HTI − Haiti, HUN − Hungary, IDN − Indonesia, IND − India, IRL − Ireland, IRN − Iran, IRQ − Iraq, ISL − Iceland, ISR − Israel, ITA − Italy, JAM − Jamaica, JOR − Jordan, JPN − Japan, KAZ − Kazakhstan, KEN − Kenya, KGZ − Kyrgyzstan, KHM − Cambodia, KIR − Kiribati, KOR − South Korea, KWT − Kuwait, LAO − Laos, LBN − Lebanon, LBR − Liberia, LBY − Libya, LCA − Saint Lucia, LKA − Sri Lanka, LSO − Lesotho, LTU − Lithuania, LUX − Luxembourg, LVA − Latvia, MAR − Morocco, MDA − Moldova, MDG − Madagascar, MDV − Maldives, MEX − Mexico, MHL − Marshall Islands, MKD − Macedonia, MLI − Mali, MLT − Malta, MMR − Myanmar, MNE − Montenegro, MNG − Mongolia, MNP − Northern Mariana Islands, MOZ − Mozambique, MRT − Mauritania, MUS − Mauritius, MWI − Malawi, MYS − Malaysia, NAM − Namibia, NER − Niger, NGA − Nigeria, NIC − Nicaragua, NLD − Netherlands, NOR − Norway, NPL − Nepal, NZL − New Zealand, OMN − Oman, PAK − Pakistan, PAN − Panama, PER − Peru, PHL − Philippines, PNG − Papua New Guinea, POL − Poland, PRI − Puerto Rico, PRK − North Korea, PRT − Portugal, PRY − Paraguay, PSE − Palestine, QAT − Qatar, ROU − Romania, RUS − Russian Federation, RWA − Rwanda, SAU − Saudi Arabia, SDN − Sudan, SEN − Senegal, SGP − Singapore, SLB − Solomon Islands, SLE − Sierra Leone, SLV − El Salvador, SOM − Somalia, SRB − Serbia, SSD − South Sudan, STP − Sao Tome and Principe, SUR − Suriname, SVK − Slovakia, SVN − Slovenia, SWE − Sweden, SWZ − Swaziland, SYC − Seychelles, SYR − Syria, TCD − Chad, TGO − Togo, THA − Thailand, TJK − Tajikistan, TKM − Turkmenistan, TLS − Timor−Leste, TON − Tonga, TTO − Trinidad and Tobago, TUN − Tunisia, TUR − Turkey, TWN − Taiwan, TZA − Tanzania, UGA − Uganda, UKR − Ukraine, URY − Uruguay, USA − United States, UZB − Uzbekistan, VCT − Saint Vincent and the Grenadines, VEN − Venezuela, VIR − Virgin Islands, U.S., VNM − Vietnam, VUT − Vanuatu, WSM − Samoa, YEM − Yemen, ZAF − South Africa, ZMB − Zambia, ZWE − Zimbabwe

© 2019 GBD 2017 Child and Adolescent Health Collaborators. JAMA Pediatrics.

eFigure 6p. Annual percent change of observed to expected (O:E) all−cause DALY rates in <20 years from 1990 to 2000 versus 2000 to 2017

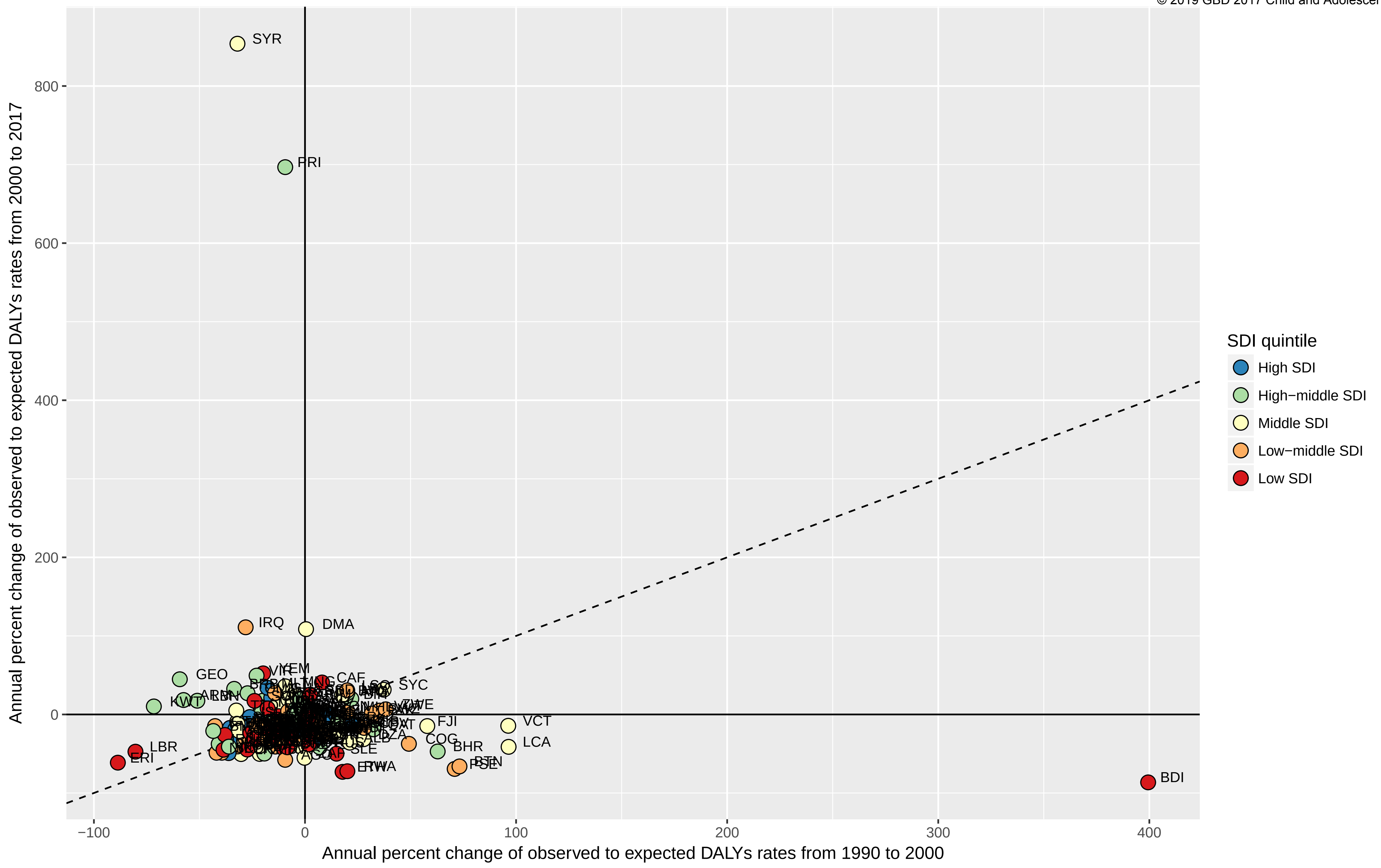

eFigure 6p. Annual percent change of observed to expected (O:E) all−cause DALY rates in <20 years from 1990 to 2000 versus 2000 to 2017 (graph subsetted to +/− 50%)

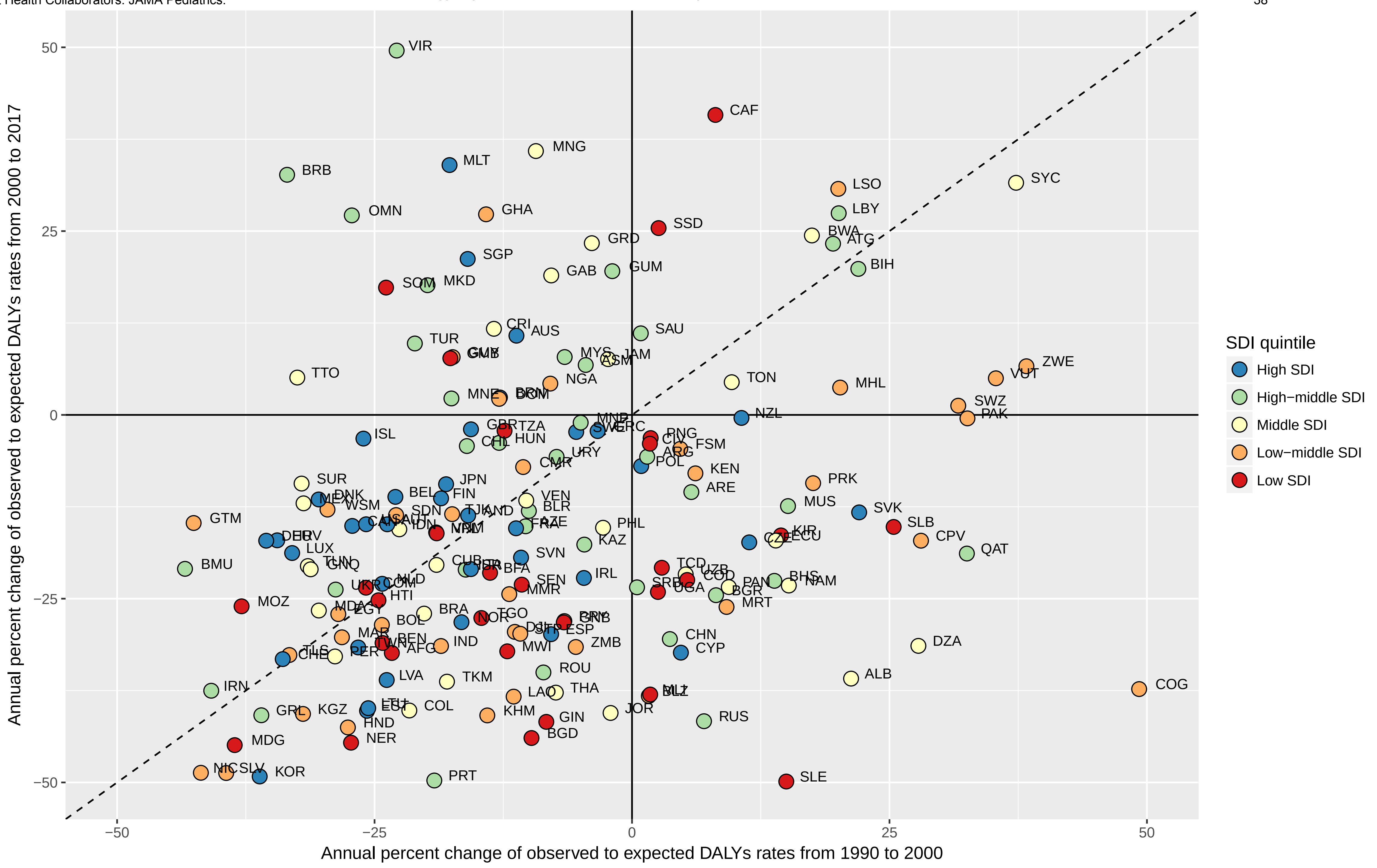

Annual percent change of observed versus expected (O:E) disability−adjusted life years (DALYs) rates by country for 1990 to 2000 versus 2000 to 2017 are plotted by country. In each pair of panels, the right panel focuses on changes within +/−50%. Panels a–d plot changes for all children and adolescents less than 20 years of age; panels e–h plot changes for children less than 1; panels i–l plot changes for children between 1 and 4; panels m–p plot changes for children between 5 and 9; and panels q–t plot changes for children between 10 and 19. For each age group, the first plot in the series is the all cause change in O:E rates (i.e., panels a, e, i, m, and q); the second plot in the series is the change in O:E rates associated with communicable, maternal, neonatal, and nutritional (CMNN) conditions (i.e., panels b, f, j, and r); the third plot in the series is the change in O:E rates associated with non−communicable diseases (NCDs – i.e., panels c, g, k, s); and the fourth plot in the series is the change in O:E rates associated with injuries (i.e., panels d, h, l, t). For each, countries in different socio−demographic index (SDI) quintiles are plotted with different countries. Abbreviations: DALY=disability−adjusted life year, AFG − Afghanistan, AGO − Angola, ALB − Albania, AND − Andorra, ARE − United Arab Emirates, ARG − Argentina, ARM − Armenia, ASM − American Samoa, ATG − Antigua and Barbuda, AUS − Australia, AUT − Austria, AZE − Azerbaijan, BDI − Burundi, BEL − Belgium, BEN − Benin, BFA − Burkina Faso, BGD − Bangladesh, BGR − Bulgaria, BHR − Bahrain, BHS − The Bahamas, BIH − Bosnia and Herzegovina, BLR − Belarus, BLZ − Belize, BMU − Bermuda, BOL − Bolivia, BRA − Brazil, BRB − Barbados, BRN − Brunei, BTN − Bhutan, BWA − Botswana, CAF − Central African Republic, CAN − Canada, CHE − Switzerland, CHL − Chile, CHN − China, CIV − Cote d'Ivoire, CMR − Cameroon, COD − Democratic Republic of the Congo, COG − Congo, COL − Colombia, COM − Comoros, CPV − Cape Verde, CRI − Costa Rica, CUB − Cuba, CYP − Cyprus, CZE − Czech Republic, DEU − Germany, DJI − Djibouti, DMA − Dominica, DNK − Denmark, DOM − Dominican Republic, DZA − Algeria, ECU − Ecuador, EGY − Egypt, ERI − Eritrea, ESP − Spain, EST − Estonia, ETH − Ethiopia, FIN − Finland, FJI − Fiji, FRA − France, FSM − Federated States of Micronesia, G − Global, GAB − Gabon, GBR − United Kingdom, GEO − Georgia, GHA − Ghana, GIN − Guinea, GMB − The Gambia, GNB − Guinea−Bissau, GNQ − Equatorial Guinea, GRC − Greece, GRD − Grenada, GRL − Greenland, GTM − Guatemala, GUM − Guam, GUY − Guyana, HND − Honduras, HRV − Croatia, HTI − Haiti, HUN − Hungary, IDN − Indonesia, IND − India, IRL − Ireland, IRN − Iran, IRQ − Iraq, ISL − Iceland, ISR − Israel, ITA − Italy, JAM − Jamaica, JOR − Jordan, JPN − Japan, KAZ − Kazakhstan, KEN − Kenya, KGZ − Kyrgyzstan, KHM − Cambodia, KIR − Kiribati, KOR − South Korea, KWT − Kuwait, LAO − Laos, LBN − Lebanon, LBR − Liberia, LBY − Libya, LCA − Saint Lucia, LKA − Sri Lanka, LSO − Lesotho, LTU − Lithuania, LUX − Luxembourg, LVA − Latvia, MAR − Morocco, MDA − Moldova, MDG − Madagascar, MDV − Maldives, MEX − Mexico, MHL − Marshall Islands, MKD − Macedonia, MLI − Mali, MLT − Malta, MMR − Myanmar, MNE − Montenegro, MNG − Mongolia, MNP − Northern Mariana Islands, MOZ − Mozambique, MRT − Mauritania, MUS − Mauritius, MWI − Malawi, MYS − Malaysia, NAM − Namibia, NER − Niger, NGA − Nigeria, NIC − Nicaragua, NLD − Netherlands, NOR − Norway, NPL − Nepal, NZL − New Zealand, OMN − Oman, PAK − Pakistan, PAN − Panama, PER − Peru, PHL − Philippines, PNG − Papua New Guinea, POL − Poland, PRI − Puerto Rico, PRK − North Korea, PRT − Portugal, PRY − Paraguay, PSE − Palestine, QAT − Qatar, ROU − Romania, RUS − Russian Federation, RWA − Rwanda, SAU − Saudi Arabia, SDN − Sudan, SEN − Senegal, SGP − Singapore, SLB − Solomon Islands, SLE − Sierra Leone, SLV − El Salvador, SOM − Somalia, SRB − Serbia, SSD − South Sudan, STP − Sao Tome and Principe, SUR − Suriname, SVK − Slovakia, SVN − Slovenia, SWE − Sweden, SWZ − Swaziland, SYC − Seychelles, SYR − Syria, TCD − Chad, TGO − Togo, THA − Thailand, TJK − Tajikistan, TKM − Turkmenistan, TLS − Timor−Leste, TON − Tonga, TTO − Trinidad and Tobago, TUN − Tunisia, TUR − Turkey, TWN − Taiwan, TZA − Tanzania, UGA − Uganda, UKR − Ukraine, URY − Uruguay, USA − United States, UZB − Uzbekistan, VCT − Saint Vincent and the Grenadines, VEN − Venezuela, VIR − Virgin Islands, U.S., VNM − Vietnam, VUT − Vanuatu, WSM − Samoa, YEM − Yemen, ZAF − South Africa, ZMB − Zambia, ZWE − Zimbabwe

© 2019 GBD 2017 Child and Adolescent Health Collaborators. JAMA Pediatrics.

eFigure 6q. Annual percent change of observed to expected (O:E) all−cause DALY rates in <20 years from 1990 to 2000 versus 2000 to 2017

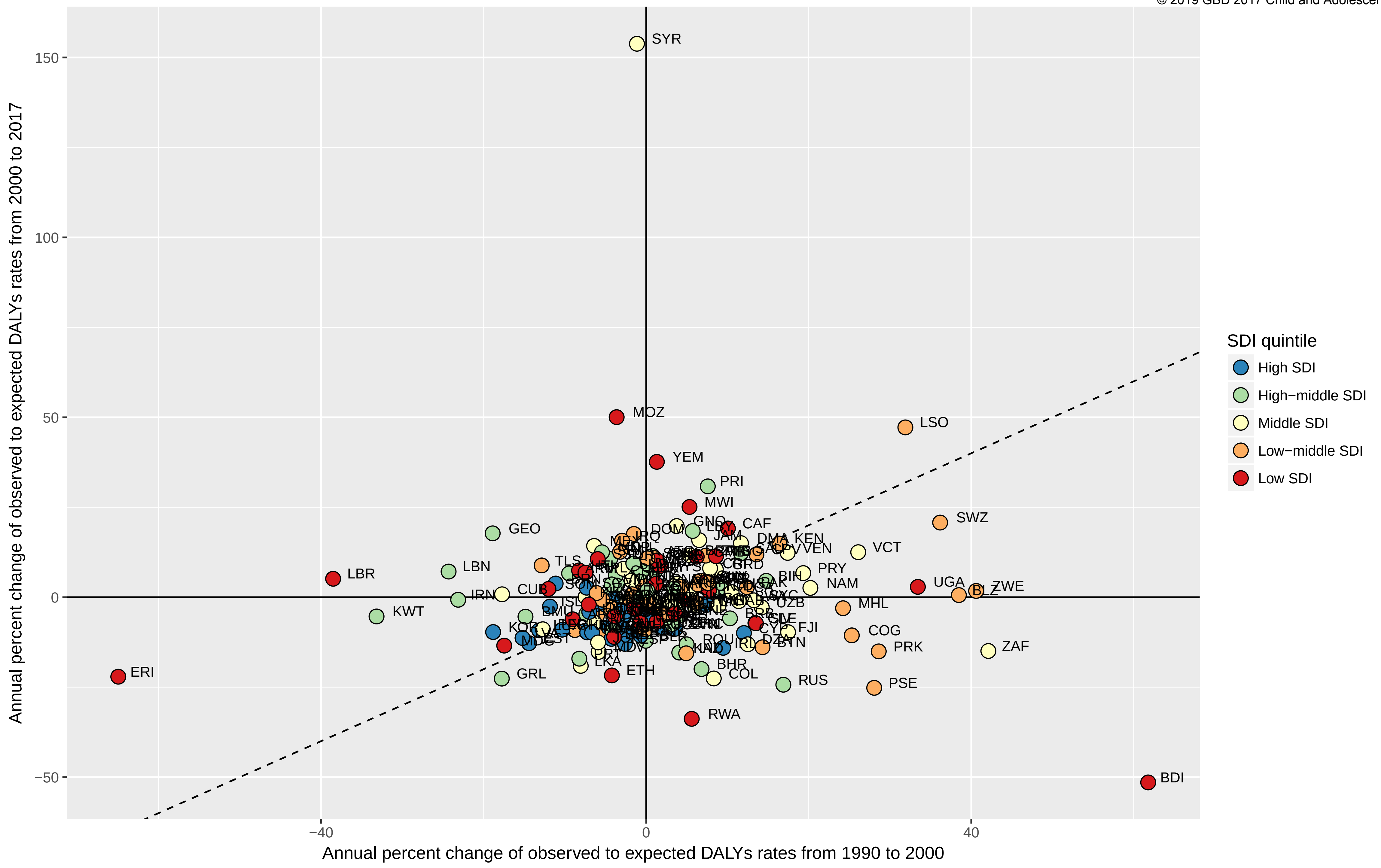

eFigure 6q. Annual percent change of observed to expected (O:E) all−cause DALY rates in <20 years from 1990 to 2000 versus 2000 to 2017 (graph subsetted to +/− 50%)

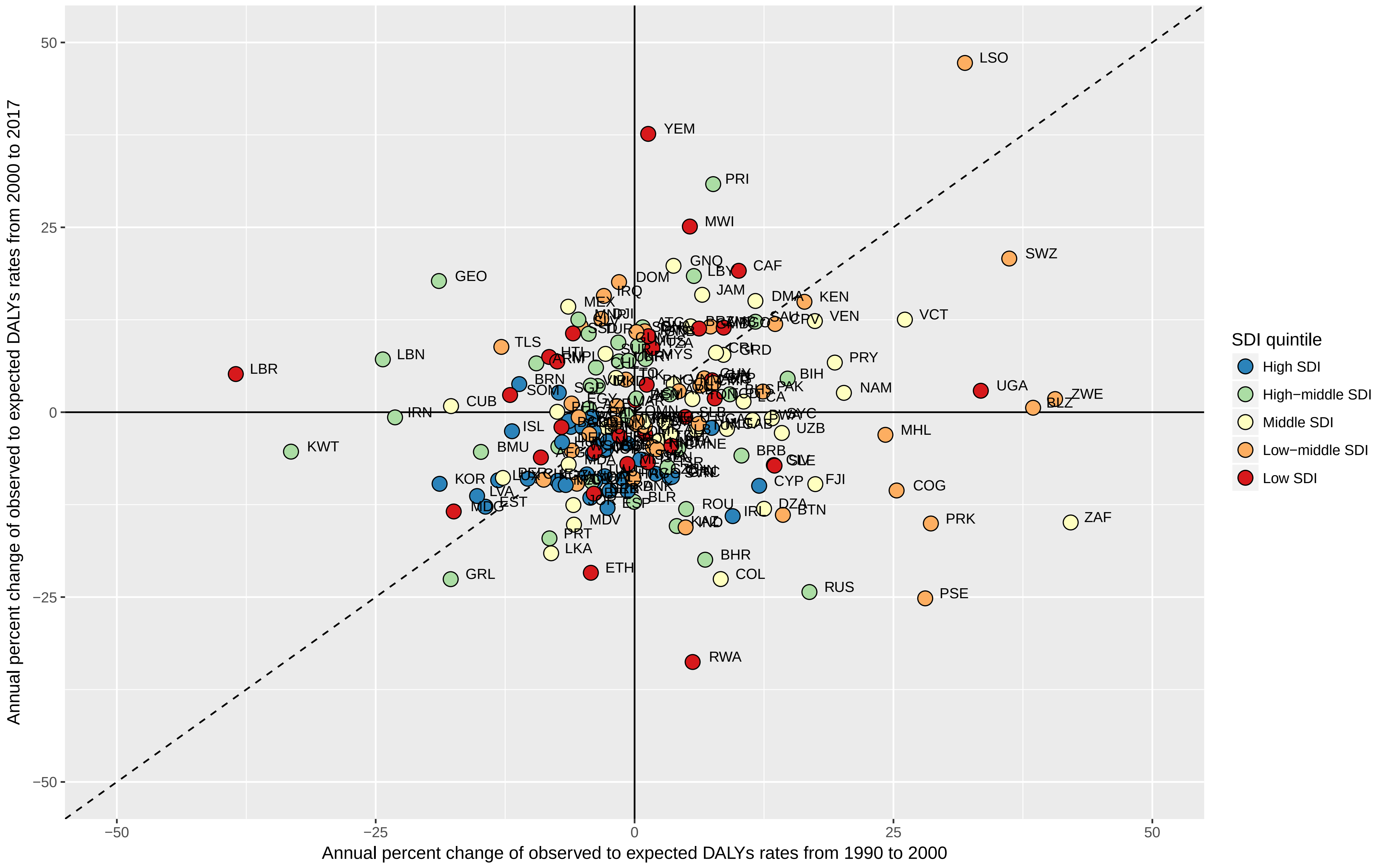

Annual percent change of observed versus expected (O:E) disability−adjusted life years (DALYs) rates by country for 1990 to 2000 versus 2000 to 2017 are plotted by country. In each pair of panels, the right panel focuses on changes within +/−50%. Panels a−d plot changes for all children and adolescents less than 20 years of age; panels e−h plot changes for children less than 1; panels i−l plot changes for children between 1 and 4; panels m−p plot changes for children between 5 and 9; and panels q−t plot changes for children between 10 and 19. For each age group, the first plot in the series is the all cause change in O:E rates (i.e., panels a, e, i, m, and q); the second plot in the series is the change in O:E rates associated with communicable, maternal, neonatal, and nutritional (CMNN) conditions (i.e., panels b, f, j, and r); the third plot in the series is the change in O:E rates associated with non−communicable diseases (NCDs – i.e., panels c, g, k, s); and the fourth plot in the series is the change in O:E rates associated with injuries (i.e., panels d, h, l, t). For each, countries in different socio−demographic index (SDI) quintiles are plotted with different countries. Abbreviations: DALY=disability−adjusted life year, AFG − Afghanistan, AGO − Angola, ALB − Albania, AND − Andorra, ARE − United Arab Emirates, ARG − Argentina, ARM − Armenia, ASM − American Samoa, ATG − Antigua and Barbuda, AUS − Australia, AUT − Austria, AZE − Azerbaijan, BDI − Burundi, BEL − Belgium, BEN − Benin, BFA − Burkina Faso, BGD − Bangladesh, BGR − Bulgaria, BHR − Bahrain, BHS − The Bahamas, BIH − Bosnia and Herzegovina, BLR − Belarus, BLZ − Belize, BMU − Bermuda, BOL − Bolivia, BRA − Brazil, BRB − Barbados, BRN − Brunei, BTN − Bhutan, BWA − Botswana, CAF − Central African Republic, CAN − Canada, CHE − Switzerland, CHL − Chile, CHN − China, CIV − Cote d'Ivoire, CMR − Cameroon, COD − Democratic Republic of the Congo, COG − Congo, COL − Colombia, COM − Comoros, CPV − Cape Verde, CRI − Costa Rica, CUB − Cuba, CYP − Cyprus, CZE − Czech Republic, DEU − Germany, DJI − Djibouti, DMA − Dominica, DNK − Denmark, DOM − Dominican Republic, DZA − Algeria, ECU − Ecuador, EGY − Egypt, ERI − Eritrea, ESP − Spain, EST − Estonia, ETH − Ethiopia, FIN − Finland, FJI − Fiji, FRA − France, FSM − Federated States of Micronesia, G − Global, GAB − Gabon, GBR − United Kingdom, GEO − Georgia, GHA − Ghana, GIN − Guinea, GMB − The Gambia, GNB − Guinea−Bissau, GNQ − Equatorial Guinea, GRC − Greece, GRD − Grenada, GRL − Greenland, GTM − Guatemala, GUM − Guam, GUY − Guyana, HND − Honduras, HRV − Croatia, HTI − Haiti, HUN − Hungary, IDN − Indonesia, IND − India, IRL − Ireland, IRN − Iran, IRQ − Iraq, ISL − Iceland, ISR − Israel, ITA − Italy, JAM − Jamaica, JOR − Jordan, JPN − Japan, KAZ − Kazakhstan, KEN − Kenya, KGZ − Kyrgyzstan, KHM − Cambodia, KIR − Kiribati, KOR − South Korea, KWT − Kuwait, LAO − Laos, LBN − Lebanon, LBR − Liberia, LBY − Libya, LCA − Saint Lucia, LKA − Sri Lanka, LSO − Lesotho, LTU − Lithuania, LUX − Luxembourg, LVA − Latvia, MAR − Morocco, MDA − Moldova, MDG − Madagascar, MDV − Maldives, MEX − Mexico, MHL − Marshall Islands, MKD − Macedonia, MLI − Mali, MLT − Malta, MMR − Myanmar, MNE − Montenegro, MNG − Mongolia, MNP − Northern Mariana Islands, MOZ − Mozambique, MRT − Mauritania, MUS − Mauritius, MWI − Malawi, MYS − Malaysia, NAM − Namibia, NER − Niger, NGA − Nigeria, NIC − Nicaragua, NLD − Netherlands, NOR − Norway, NPL − Nepal, NZL − New Zealand, OMN − Oman, PAK − Pakistan, PAN − Panama, PER − Peru, PHL − Philippines, PNG − Papua New Guinea, POL − Poland, PRI − Puerto Rico, PRK − North Korea, PRT − Portugal, PRY − Paraguay, PSE − Palestine, QAT − Qatar, ROU − Romania, RUS − Russian Federation, RWA − Rwanda, SAU − Saudi Arabia, SDN − Sudan, SEN − Senegal, SGP − Singapore, SLB − Solomon Islands, SLE − Sierra Leone, SLV − El Salvador, SOM − Somalia, SRB − Serbia, SSD − South Sudan, STP − Sao Tome and Principe, SUR − Suriname, SVK − Slovakia, SVN − Slovenia, SWE − Sweden, SWZ − Swaziland, SYC − Seychelles, SYR − Syria, TCD − Chad, TGO − Togo, THA − Thailand, TJK − Tajikistan, TKM − Turkmenistan, TLS − Timor−Leste, TON − Tonga, TTO − Trinidad and Tobago, TUN − Tunisia, TUR − Turkey, TWN − Taiwan, TZA − Tanzania, UGA − Uganda, UKR − Ukraine, URY − Uruguay, USA − United States, UZB − Uzbekistan, VCT − Saint Vincent and the Grenadines, VEN − Venezuela, VIR − Virgin Islands, U.S., VNM − Vietnam, VUT − Vanuatu, WSM − Samoa, YEM − Yemen, ZAF − South Africa, ZMB − Zambia, ZWE − Zimbabwe

© 2019 GBD 2017 Child and Adolescent Health Collaborators. JAMA Pediatrics.

eFigure 6r. Annual percent change of observed to expected (O:E) all−cause DALY rates in <20 years from 1990 to 2000 versus 2000 to 2017

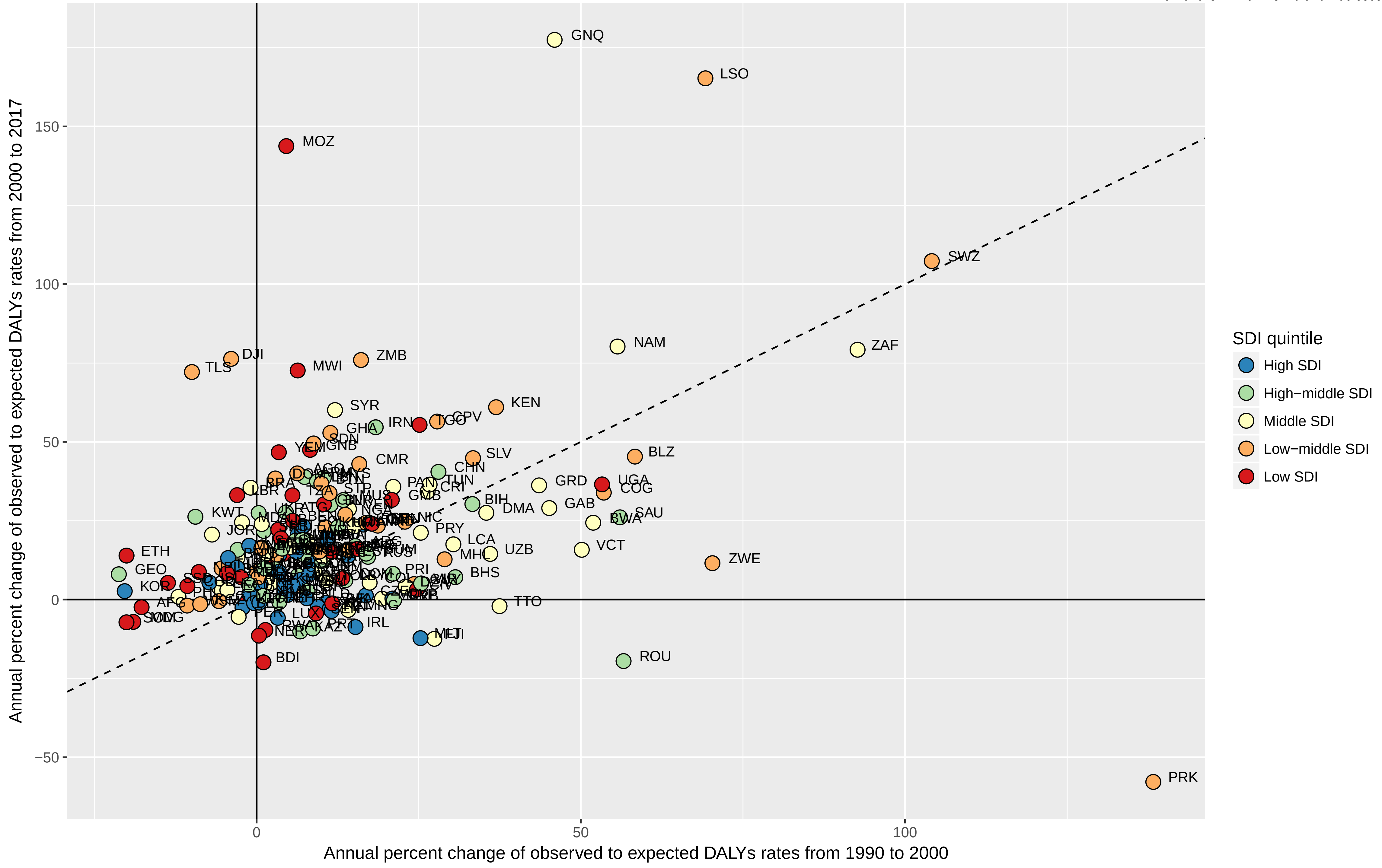

eFigure 6r. Annual percent change of observed to expected (O:E) all−cause DALY rates in <20 years from 1990 to 2000 versus 2000 to 2017 (graph subsetted to +/− 50%)

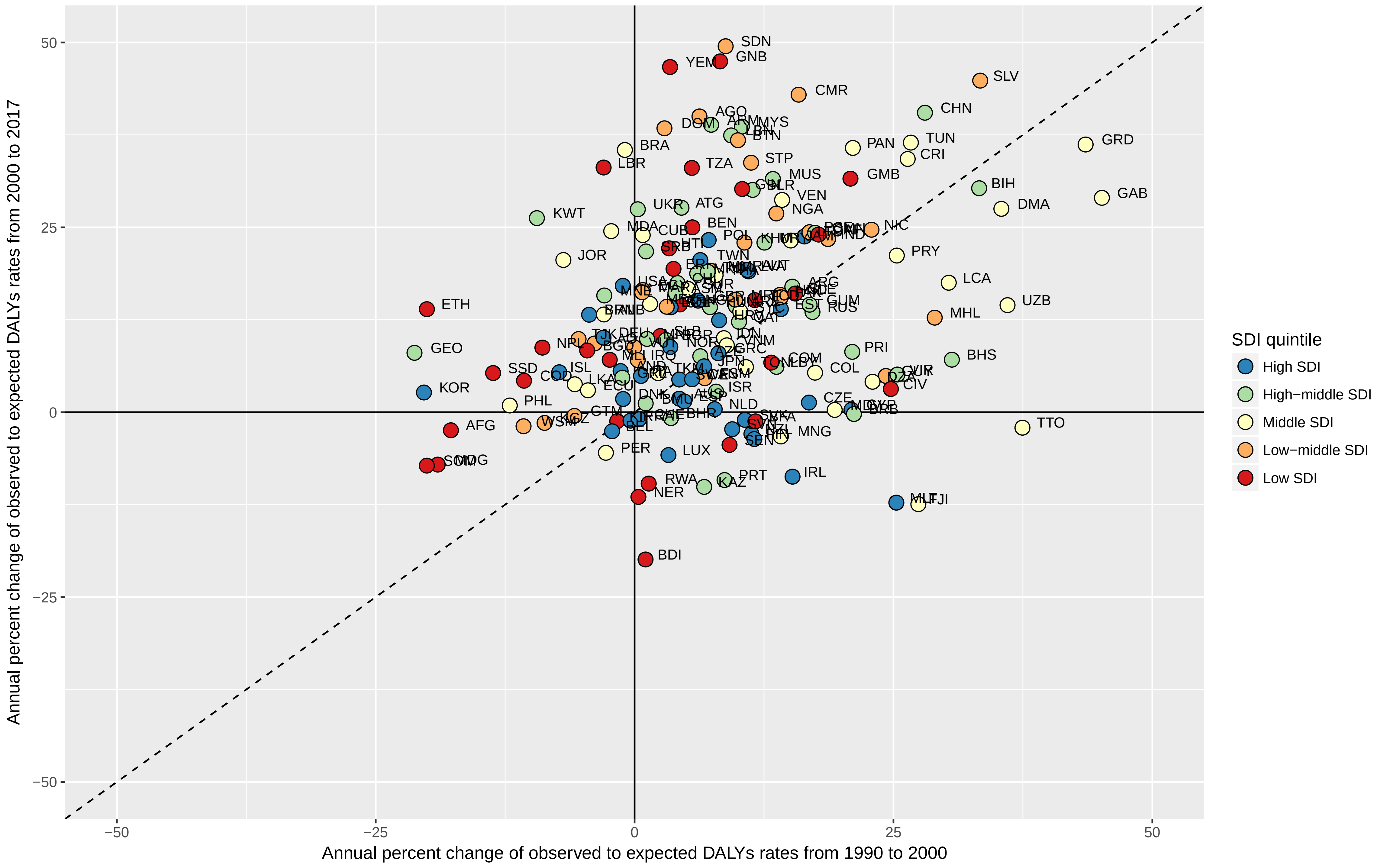

Annual percent change of observed versus expected (O:E) disability−adjusted life years (DALYs) rates by country for 1990 to 2000 versus 2000 to 2017 are plotted by country. In each pair of panels, the right panel focuses on changes within +/−50%. Panels a–d plot changes for all children and adolescents less than 20 years of age; panels e–h plot changes for children less than 1; panels i–l plot changes for children between 1 and 4; panels m–p plot changes for children between 5 and 9; and panels q–t plot changes for children between 10 and 19. For each age group, the first plot in the series is the all cause change in O:E rates (i.e., panels a, e, i, m, and q); the second plot in the series is the change in O:E rates associated with communicable, maternal, neonatal, and nutritional (CMNN) conditions (i.e., panels b, f, j, and r); the third plot in the series is the change in O:E rates associated with non−communicable diseases (NCDs – i.e., panels c, g, k, s); and the fourth plot in the series is the change in O:E rates associated with injuries (i.e., panels d, h, l, t). For each, countries in different socio−demographic index (SDI) quintiles are plotted with different countries. Abbreviations: DALY=disability−adjusted life year, AFG − Afghanistan, AGO − Angola, ALB − Albania, AND − Andorra, ARE − United Arab Emirates, ARG − Argentina, ARM − Armenia, ASM − American Samoa, ATG − Antigua and Barbuda, AUS − Australia, AUT − Austria, AZE − Azerbaijan, BDI − Burundi, BEL − Belgium, BEN − Benin, BFA − Burkina Faso, BGD − Bangladesh, BGR − Bulgaria, BHR − Bahrain, BHS − The Bahamas, BIH − Bosnia and Herzegovina, BLR − Belarus, BLZ − Belize, BMU − Bermuda, BOL − Bolivia, BRA − Brazil, BRB − Barbados, BRN − Brunei, BTN − Bhutan, BWA − Botswana, CAF − Central African Republic, CAN − Canada, CHE − Switzerland, CHL − Chile, CHN − China, CIV − Cote d'Ivoire, CMR − Cameroon, COD − Democratic Republic of the Congo, COG − Congo, COL − Colombia, COM − Comoros, CPV − Cape Verde, CRI − Costa Rica, CUB − Cuba, CYP − Cyprus, CZE − Czech Republic, DEU − Germany, DJI − Djibouti, DMA − Dominica, DNK − Denmark, DOM − Dominican Republic, DZA − Algeria, ECU − Ecuador, EGY − Egypt, ERI − Eritrea, ESP − Spain, EST − Estonia, ETH − Ethiopia, FIN − Finland, FJI − Fiji, FRA − France, FSM − Federated States of Micronesia, G − Global, GAB − Gabon, GBR − United Kingdom, GEO − Georgia, GHA − Ghana, GIN − Guinea, GMB − The Gambia, GNB − Guinea−Bissau, GNQ − Equatorial Guinea, GRC − Greece, GRD − Grenada, GRL − Greenland, GTM − Guatemala, GUM − Guam, GUY − Guyana, HND − Honduras, HRV − Croatia, HTI − Haiti, HUN − Hungary, IDN − Indonesia, IND − India, IRL − Ireland, IRN − Iran, IRQ − Iraq, ISL − Iceland, ISR − Israel, ITA − Italy, JAM − Jamaica, JOR − Jordan, JPN − Japan, KAZ − Kazakhstan, KEN − Kenya, KGZ − Kyrgyzstan, KHM − Cambodia, KIR − Kiribati, KOR − South Korea, KWT − Kuwait, LAO − Laos, LBN − Lebanon, LBR − Liberia, LBY − Libya, LCA − Saint Lucia, LKA − Sri Lanka, LSO − Lesotho, LTU − Lithuania, LUX − Luxembourg, LVA − Latvia, MAR − Morocco, MDA − Moldova, MDG − Madagascar, MDV − Maldives, MEX − Mexico, MHL − Marshall Islands, MKD − Macedonia, MLI − Mali, MLT − Malta, MMR − Myanmar, MNE − Montenegro, MNG − Mongolia, MNP − Northern Mariana Islands, MOZ − Mozambique, MRT − Mauritania, MUS − Mauritius, MWI − Malawi, MYS − Malaysia, NAM − Namibia, NER − Niger, NGA − Nigeria, NIC − Nicaragua, NLD − Netherlands, NOR − Norway, NPL − Nepal, NZL − New Zealand, OMN − Oman, PAK − Pakistan, PAN − Panama, PER − Peru, PHL − Philippines, PNG − Papua New Guinea, POL − Poland, PRI − Puerto Rico, PRK − North Korea, PRT − Portugal, PRY − Paraguay, PSE − Palestine, QAT − Qatar, ROU − Romania, RUS − Russian Federation, RWA − Rwanda, SAU − Saudi Arabia, SDN − Sudan, SEN − Senegal, SGP − Singapore, SLB − Solomon Islands, SLE − Sierra Leone, SLV − El Salvador, SOM − Somalia, SRB − Serbia, SSD − South Sudan, STP − Sao Tome and Principe, SUR − Suriname, SVK − Slovakia, SVN − Slovenia, SWE − Sweden, SWZ − Swaziland, SYC − Seychelles, SYR − Syria, TCD − Chad, TGO − Togo, THA − Thailand, TJK − Tajikistan, TKM − Turkmenistan, TLS − Timor−Leste, TON − Tonga, TTO − Trinidad and Tobago, TUN − Tunisia, TUR − Turkey, TWN − Taiwan, TZA − Tanzania, UGA − Uganda, UKR − Ukraine, URY − Uruguay, USA − United States, UZB − Uzbekistan, VCT − Saint Vincent and the Grenadines, VEN − Venezuela, VIR − Virgin Islands, U.S., VNM − Vietnam, VUT − Vanuatu, WSM − Samoa, YEM − Yemen, ZAF − South Africa, ZMB − Zambia, ZWE − Zimbabwe

eFigure 6s. Annual percent change of observed to expected (O:E) all−cause DALY rates in <20 years from 1990 to 2000 versus 2000 to 2017

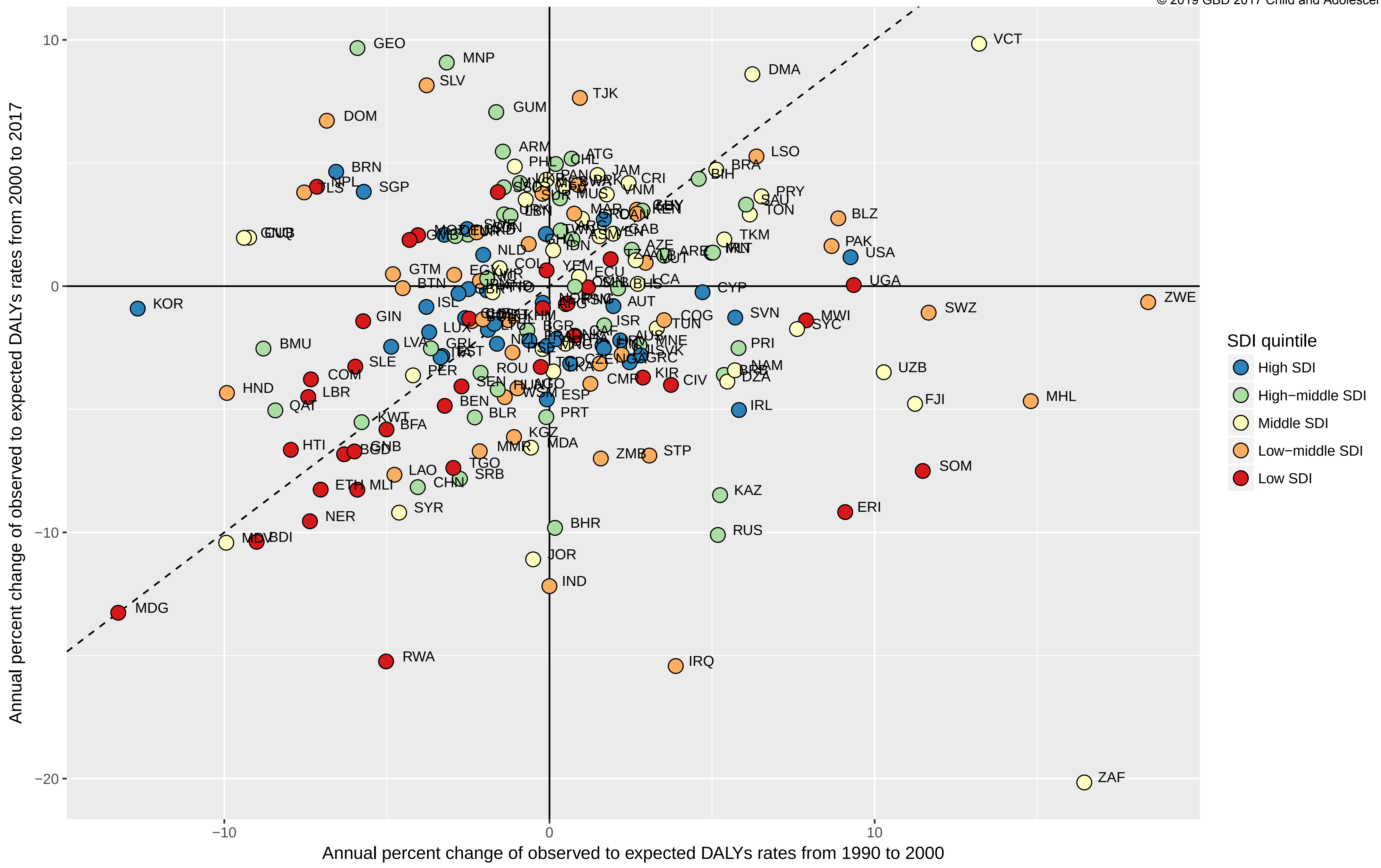

© 2019 GBD 2017 Child and Adolescent Health Collaborators. JAMA Pediatrics.

eFigure 6s. Annual percent change of observed to expected (O:E) all−cause DALY rates in <20 years from 1990 to 2000 versus 2000 to 2017 (graph subsetted to +/− 50%)

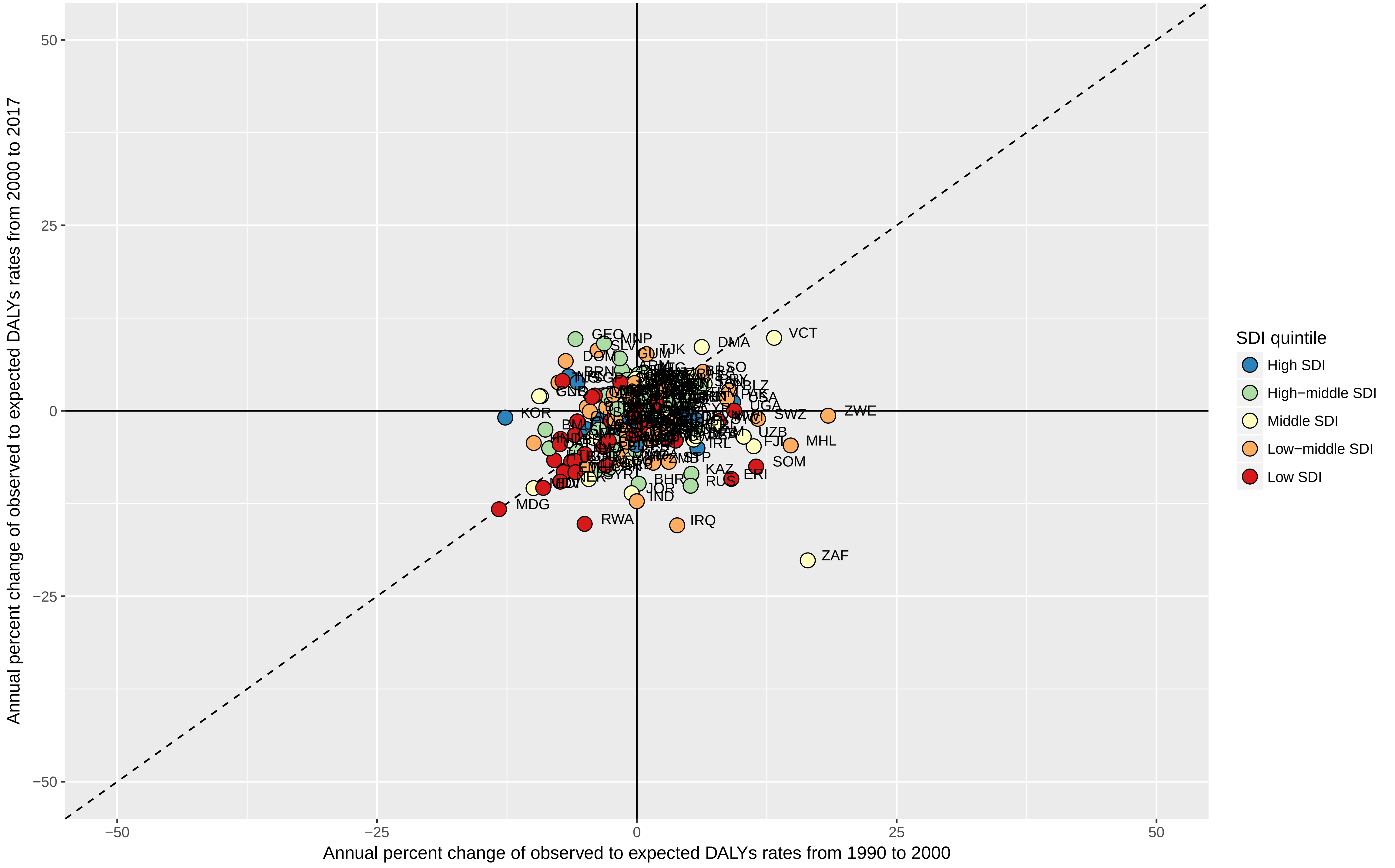

Annual percent change of observed versus expected (O:E) disability−adjusted life years (DALYs) rates by country for 1990 to 2000 versus 2000 to 2017 are plotted by country. In each pair of panels, the right panel focuses on changes within +/−50%. Panels a−d plot changes for all children and adolescents less than 20 years of age; panels e−h plot changes for children less than 1; panels i−l plot changes for children between 1 and 4; panels m−p plot changes for children between 5 and 9; and panels q−t plot changes for children between 10 and 19. For each age group, the first plot in the series is the all cause change in O:E rates (i.e., panels a, e, i, m, and q); the second plot in the series is the change in O:E rates associated with communicable, maternal, neonatal, and nutritional (CMNN) conditions (i.e., panels b, f, j, and r); the third plot in the series is the change in O:E rates associated with non−communicable diseases (NCDs – i.e., panels c, g, k, s); and the fourth plot in the series is the change in O:E rates associated with injuries (i.e., panels d, h, l, t). For each, countries in different socio−demographic index (SDI) quintiles are plotted with different countries. Abbreviations: DALY=disability−adjusted life year, AFG − Afghanistan, AGO − Angola, ALB − Albania, AND − Andorra, ARE − United Arab Emirates, ARG − Argentina, ARM − Armenia, ASM − American Samoa, ATG − Antigua and Barbuda, AUS − Australia, AUT − Austria, AZE − Azerbaijan, BDI − Burundi, BEL − Belgium, BEN − Benin, BFA − Burkina Faso, BGD − Bangladesh, BGR − Bulgaria, BHR − Bahrain, BHS − The Bahamas, BIH − Bosnia and Herzegovina, BLR − Belarus, BLZ − Belize, BMU − Bermuda, BOL − Bolivia, BRA − Brazil, BRB − Barbados, BRN − Brunei, BTN − Bhutan, BWA − Botswana, CAF − Central African Republic, CAN − Canada, CHE − Switzerland, CHL − Chile, CHN − China, CIV − Cote d'Ivoire, CMR − Cameroon, COD − Democratic Republic of the Congo, COG − Congo, COL − Colombia, COM − Comoros, CPV − Cape Verde, CRI − Costa Rica, CUB − Cuba, CYP − Cyprus, CZE − Czech Republic, DEU − Germany, DJI − Djibouti, DMA − Dominica, DNK − Denmark, DOM − Dominican Republic, DZA − Algeria, ECU − Ecuador, EGY − Egypt, ERI − Eritrea, ESP − Spain, EST − Estonia, ETH − Ethiopia, FIN − Finland, FJI − Fiji, FRA − France, FSM − Federated States of Micronesia, G − Global, GAB − Gabon, GBR − United Kingdom, GEO − Georgia, GHA − Ghana, GIN − Guinea, GMB − The Gambia, GNB − Guinea−Bissau, GNQ − Equatorial Guinea, GRC − Greece, GRD − Grenada, GRL − Greenland, GTM − Guatemala, GUM − Guam, GUY − Guyana, HND − Honduras, HRV − Croatia, HTI − Haiti, HUN − Hungary, IDN − Indonesia, IND − India, IRL − Ireland, IRN − Iran, IRQ − Iraq, ISL − Iceland, ISR − Israel, ITA − Italy, JAM − Jamaica, JOR − Jordan, JPN − Japan, KAZ − Kazakhstan, KEN − Kenya, KGZ − Kyrgyzstan, KHM − Cambodia, KIR − Kiribati, KOR − South Korea, KWT − Kuwait, LAO − Laos, LBN − Lebanon, LBR − Liberia, LBY − Libya, LCA − Saint Lucia, LKA − Sri Lanka, LSO − Lesotho, LTU − Lithuania, LUX − Luxembourg, LVA − Latvia, MAR − Morocco, MDA − Moldova, MDG − Madagascar, MDV − Maldives, MEX − Mexico, MHL − Marshall Islands, MKD − Macedonia, MLI − Mali, MLT − Malta, MMR − Myanmar, MNE − Montenegro, MNG − Mongolia, MNP − Northern Mariana Islands, MOZ − Mozambique, MRT − Mauritania, MUS − Mauritius, MWI − Malawi, MYS − Malaysia, NAM − Namibia, NER − Niger, NGA − Nigeria, NIC − Nicaragua, NLD − Netherlands, NOR − Norway, NPL − Nepal, NZL − New Zealand, OMN − Oman, PAK − Pakistan, PAN − Panama, PER − Peru, PHL − Philippines, PNG − Papua New Guinea, POL − Poland, PRI − Puerto Rico, PRK − North Korea, PRT − Portugal, PRY − Paraguay, PSE − Palestine, QAT − Qatar, ROU − Romania, RUS − Russian Federation, RWA − Rwanda, SAU − Saudi Arabia, SDN − Sudan, SEN − Senegal, SGP − Singapore, SLB − Solomon Islands, SLE − Sierra Leone, SLV − El Salvador, SOM − Somalia, SRB − Serbia, SSD − South Sudan, STP − Sao Tome and Principe, SUR − Suriname, SVK − Slovakia, SVN − Slovenia, SWE − Sweden, SWZ − Swaziland, SYC − Seychelles, SYR − Syria, TCD − Chad, TGO − Togo, THA − Thailand, TJK − Tajikistan, TKM − Turkmenistan, TLS − Timor−Leste, TON − Tonga, TTO − Trinidad and Tobago, TUN − Tunisia, TUR − Turkey, TWN − Taiwan, TZA − Tanzania, UGA − Uganda, UKR − Ukraine, URY − Uruguay, USA − United States, UZB − Uzbekistan, VCT − Saint Vincent and the Grenadines, VEN − Venezuela, VIR − Virgin Islands, U.S., VNM − Vietnam, VUT − Vanuatu, WSM − Samoa, YEM − Yemen, ZAF − South Africa, ZMB − Zambia, ZWE − Zimbabwe

© 2019 GBD 2017 Child and Adolescent Health Collaborators. JAMA Pediatrics.

eFigure 6t. Annual percent change of observed to expected (O:E) all−cause DALY rates in <20 years from 1990 to 2000 versus 2000 to 2017

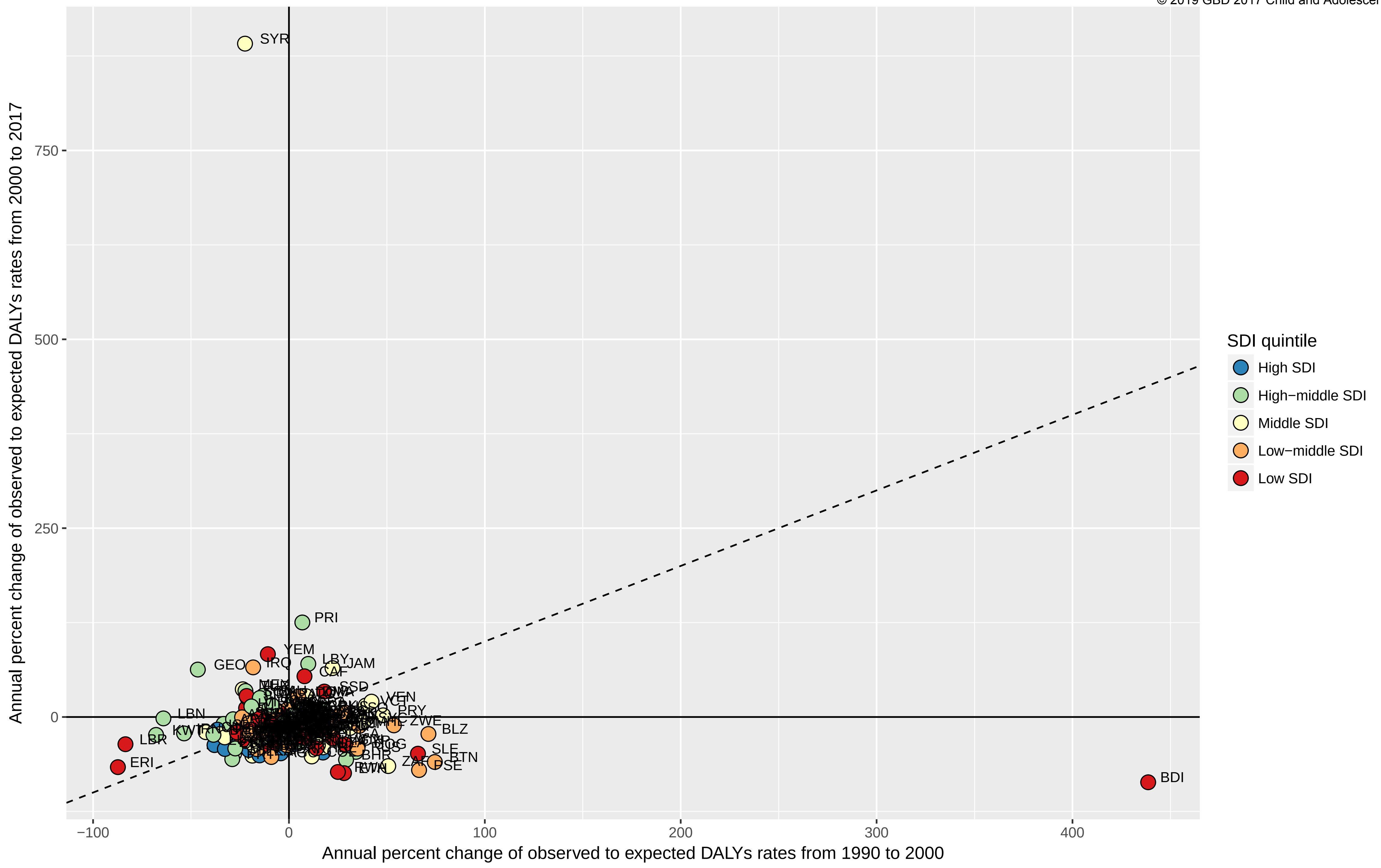

eFigure 6t. Annual percent change of observed to expected (O:E) all−cause DALY rates in <20 years from 1990 to 2000 versus 2000 to 2017 (graph subsetted to +/− 50%)

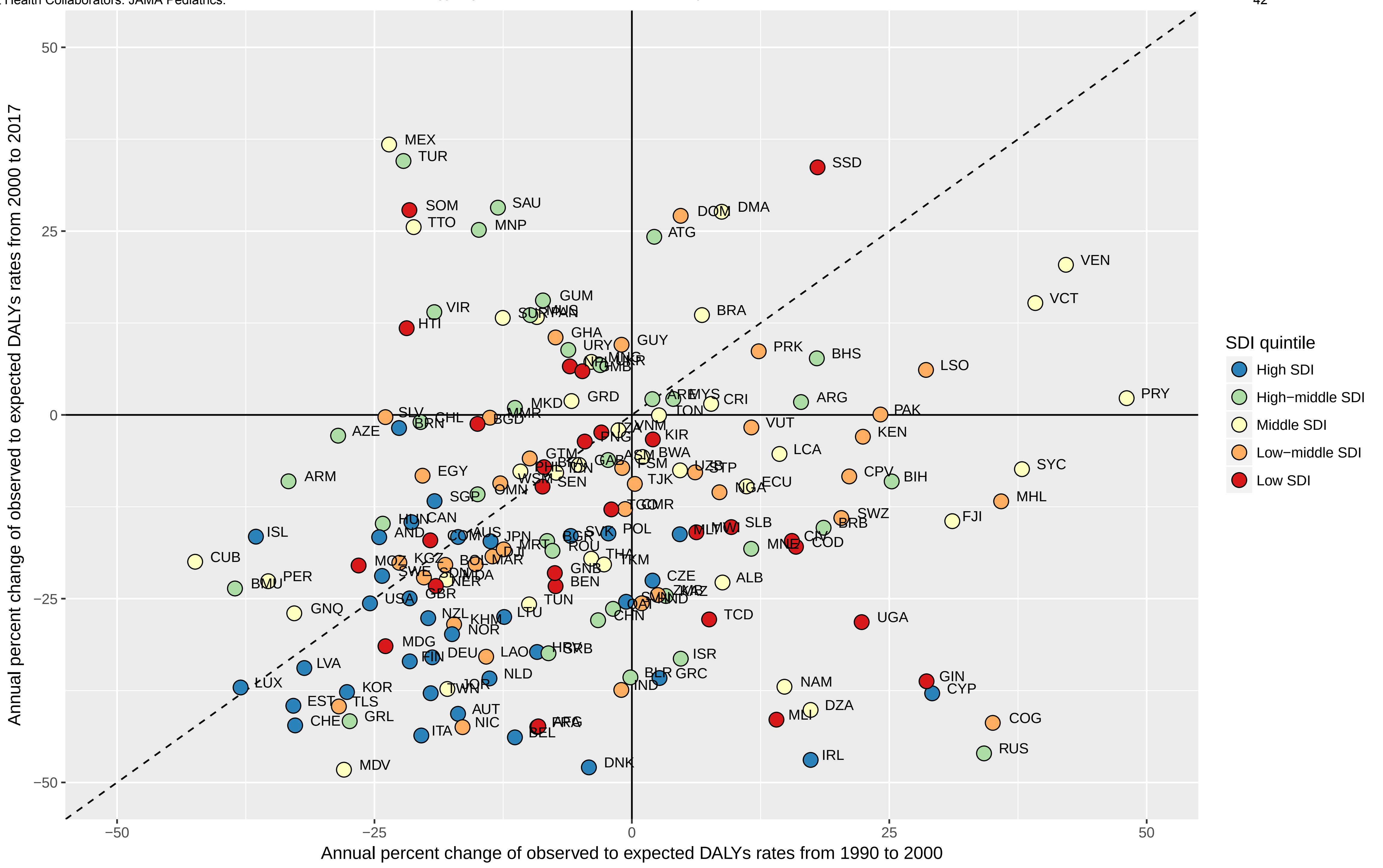

Annual percent change of observed versus expected (O:E) disability−adjusted life years (DALYs) rates by country for 1990 to 2000 versus 2000 to 2017 are plotted by country. In each pair of panels, the right panel focuses on changes within +/−50%. Panels a−d plot changes for all children and adolescents less than 20 years of age; panels e−h plot changes for children less than 1; panels i−l plot changes for children between 1 and 4; panels m−p plot changes for children between 5 and 9; and panels q−t plot changes for children between 10 and 19. For each age group, the first plot in the series is the all cause change in O:E rates (i.e., panels a, e, i, m, and q); the second plot in the series is the change in O:E rates associated with communicable, maternal, neonatal, and nutritional (CMNN) conditions (i.e., panels b, f, j, and r); the third plot in the series is the change in O:E rates associated with non−communicable diseases (NCDs – i.e., panels c, g, k, s); and the fourth plot in the series is the change in O:E rates associated with injuries (i.e., panels d, h, l, t). For each, countries in different socio−demographic index (SDI) quintiles are plotted with different countries. Abbreviations: DALY=disability−adjusted life year, AFG − Afghanistan, AGO − Angola, ALB − Albania, AND − Andorra, ARE − United Arab Emirates, ARG − Argentina, ARM − Armenia, ASM − American Samoa, ATG − Antigua and Barbuda, AUS − Australia, AUT − Austria, AZE − Azerbaijan, BDI − Burundi, BEL − Belgium, BEN − Benin, BFA − Burkina Faso, BGD − Bangladesh, BGR − Bulgaria, BHR − Bahrain, BHS − The Bahamas, BIH − Bosnia and Herzegovina, BLR − Belarus, BLZ − Belize, BMU − Bermuda, BOL − Bolivia, BRA − Brazil, BRB − Barbados, BRN − Brunei, BTN − Bhutan, BWA − Botswana, CAF − Central African Republic, CAN − Canada, CHE − Switzerland, CHL − Chile, CHN − China, CIV − Cote d'Ivoire, CMR − Cameroon, COD − Democratic Republic of the Congo, COG − Congo, COL − Colombia, COM − Comoros, CPV − Cape Verde, CRI − Costa Rica, CUB − Cuba, CYP − Cyprus, CZE − Czech Republic, DEU − Germany, DJI − Djibouti, DMA − Dominica, DNK − Denmark, DOM − Dominican Republic, DZA − Algeria, ECU − Ecuador, EGY − Egypt, ERI − Eritrea, ESP − Spain, EST − Estonia, ETH − Ethiopia, FIN − Finland, FJI − Fiji, FRA − France, FSM − Federated States of Micronesia, G − Global, GAB − Gabon, GBR − United Kingdom, GEO − Georgia, GHA − Ghana, GIN − Guinea, GMB − The Gambia, GNB − Guinea−Bissau, GNQ − Equatorial Guinea, GRC − Greece, GRD − Grenada, GRL − Greenland, GTM − Guatemala, GUM − Guam, GUY − Guyana, HND − Honduras, HRV − Croatia, HTI − Haiti, HUN − Hungary, IDN − Indonesia, IND − India, IRL − Ireland, IRN − Iran, IRQ − Iraq, ISL − Iceland, ISR − Israel, ITA − Italy, JAM − Jamaica, JOR − Jordan, JPN − Japan, KAZ − Kazakhstan, KEN − Kenya, KGZ − Kyrgyzstan, KHM − Cambodia, KIR − Kiribati, KOR − South Korea, KWT − Kuwait, LAO − Laos, LBN − Lebanon, LBR − Liberia, LBY − Libya, LCA − Saint Lucia, LKA − Sri Lanka, LSO − Lesotho, LTU − Lithuania, LUX − Luxembourg, LVA − Latvia, MAR − Morocco, MDA − Moldova, MDG − Madagascar, MDV − Maldives, MEX − Mexico, MHL − Marshall Islands, MKD − Macedonia, MLI − Mali, MLT − Malta, MMR − Myanmar, MNE − Montenegro, MNG − Mongolia, MNP − Northern Mariana Islands, MOZ − Mozambique, MRT − Mauritania, MUS − Mauritius, MWI − Malawi, MYS − Malaysia, NAM − Namibia, NER − Niger, NGA − Nigeria, NIC − Nicaragua, NLD − Netherlands, NOR − Norway, NPL − Nepal, NZL − New Zealand, OMN − Oman, PAK − Pakistan, PAN − Panama, PER − Peru, PHL − Philippines, PNG − Papua New Guinea, POL − Poland, PRI − Puerto Rico, PRK − North Korea, PRT − Portugal, PRY − Paraguay, PSE − Palestine, QAT − Qatar, ROU − Romania, RUS − Russian Federation, RWA − Rwanda, SAU − Saudi Arabia, SDN − Sudan, SEN − Senegal, SGP − Singapore, SLB − Solomon Islands, SLE − Sierra Leone, SLV − El Salvador, SOM − Somalia, SRB − Serbia, SSD − South Sudan, STP − Sao Tome and Principe, SUR − Suriname, SVK − Slovakia, SVN − Slovenia, SWE − Sweden, SWZ − Swaziland, SYC − Seychelles, SYR − Syria, TCD − Chad, TGO − Togo, THA − Thailand, TJK − Tajikistan, TKM − Turkmenistan, TLS − Timor−Leste, TON − Tonga, TTO − Trinidad and Tobago, TUN − Tunisia, TUR − Turkey, TWN − Taiwan, TZA − Tanzania, UGA − Uganda, UKR − Ukraine, URY − Uruguay, USA − United States, UZB − Uzbekistan, VCT − Saint Vincent and the Grenadines, VEN − Venezuela, VIR − Virgin Islands, U.S., VNM − Vietnam, VUT − Vanuatu, WSM − Samoa, YEM − Yemen, ZAF − South Africa, ZMB − Zambia, ZWE − Zimbabwe

eFigure 7a. Leading ten causes of DALYs with the ratio of observed DALYs to DALYs expected on the basis of Socio-Demographic Index alone in 2017, <20 years, both sexes combined. The top ten causes contributing to DALYs are listed globally, by socio-demographic quintile, and then by GBD superregion, region, country, and subnationally where modeled. For each cell, the ratio of observed DALYs to DALYs expected on the basis of socio-demographic index (SDI) alone are listed. Abbreviations: DALY=disability-adjusted life year, GBD=Global Burden of Disease.

Values shown in brackets represent the ratio of observed DALYs to predicted DALYs on the basis of Socio-Demographic Index (SDI), rounded to two (2) digits. Color ranges (shown below) were calculated to place a roughly equal number of cells into each bin.

COLOR KEY:		[0.0-0.5]	[0.5-0.69]	[0.69-0.82]	[0.82-0.92]	[0.92-1.01]	[1.01-1.18]	[1.18-1.48]	[1.48-2.26]	2.26+

	1	2	3	4	5	6	7	8	9	10
Global	Neonatal [1.38]	LRI [1.83]	Diarrhea [4.96]	Congenital [0.78]	Malaria [4596.33]	Meningitis [1.54]	Road Inj [0.69]	Iron [2.08]	PEM [9.73]	HIV [10.49]
Low SDI	Neonatal [0.7]	LRI [0.51]	Diarrhea [0.63]	Malaria [21.05]	Congenital [0.61]	PEM [0.75]	Meningitis [0.45]	HIV [4.72]	Iron [1.2]	STI [0.99]
Low-middle SDI	Neonatal [1.05]	LRI [0.91]	Diarrhea [1.62]	Congenital [0.65]	Malaria [542.96]	Meningitis [0.92]	Iron [1.64]	PEM [1.7]	Road Inj [0.65]	HIV [6.08]
Middle SDI	Neonatal [0.77]	Congenital [0.58]	LRI [0.8]	Diarrhea [1.44]	Road Inj [0.64]	Drown [0.88]	Headaches [0.81]	Iron [1.12]	Violence [1.14]	HIV [5.26]
High-middle SDI	Neonatal [1.64]	Congenital [1.32]	Road Inj [1.0]	LRI [4.63]	Headaches [0.75]	Drown [2.57]	Anxiety [0.72]	Diarrhea [2.89]	Conduct [0.89]	Back Pain [0.77]
High SDI	Neonatal [0.45]	Congenital [0.4]	Headaches [0.92]	Dermatitis [1.2]	Anxiety [1.16]	Road Inj [0.44]	Depression [1.31]	Conduct [0.98]	Back Pain [1.05]	Drugs [3.05]
Central Europe, Eastern Europe, and Central Asia	Neonatal [1.16]	LRI [4.04]	Congenital [0.77]	Road Inj [0.57]	Diarrhea [2.45]	Headaches [0.82]	Falls [1.54]	Iron [2.41]	Drown [1.26]	Conduct [0.94]
Central Asia	Neonatal [1.27]	LRI [3.48]	Congenital [0.75]	Diarrhea [2.19]	Iron [2.31]	Drown [1.1]	Road Inj [0.46]	Headaches [0.78]	Epilepsy [1.47]	Conduct [0.88]
Armenia	Neonatal [0.58]	Congenital [0.66]	LRI [1.11]	Iron [3.01]	Headaches [0.79]	Conduct [0.92]	Diarrhea [0.92]	Road Inj [0.26]	Falls [0.92]	Back Pain [0.68]
Azerbaijan	Neonatal [1.93]	LRI [6.77]	Congenital [1.24]	Diarrhea [2.45]	Encepha [5.24]	Leukemia [1.51]	Epilepsy [1.76]	Headaches [0.79]	Iron [1.56]	Fire [2.46]
Georgia	Neonatal [1.01]	Congenital [0.48]	LRI [0.58]	Road Inj [0.55]	Headaches [0.79]	Conduct [0.91]	Diarrhea [0.86]	Iron [1.1]	Epilepsy [1.07]	Falls [1.05]
Kazakhstan	Neonatal [1.18]	Congenital [1.01]	LRI [1.9]	Road Inj [0.72]	Drown [1.48]	F Body [1.91]	Vit A [3.21]	Self Harm [1.44]	Headaches [0.71]	Iron [1.74]
Kyrgyzstan	Neonatal [1.11]	LRI [0.73]	Congenital [0.52]	Iron [1.88]	Diarrhea [0.66]	Drown [0.67]	Road Inj [0.42]	Headaches [0.79]	Epilepsy [1.08]	Vit A [1.14]
Mongolia	Neonatal [1.69]	LRI [2.57]	Congenital [0.87]	Road Inj [0.72]	F Body [1.56]	Fire [2.6]	Diarrhea [0.94]	Falls [1.99]	Vit A [2.22]	Drown [0.77]
Tajikistan	Neonatal [0.91]	LRI [1.61]	Diarrhea [1.72]	Congenital [0.79]	Drown [0.95]	Iron [0.9]	Meningitis [0.36]	Epilepsy [1.55]	Vit A [0.98]	Falls [1.58]
Turkmenistan	Neonatal [1.58]	LRI [4.41]	Congenital [1.2]	Diarrhea [1.38]	Iron [1.56]	Headaches [0.75]	Drown [0.85]	Epilepsy [1.36]	F Body [1.08]	Conduct [0.86]
Uzbekistan	LRI [2.4]	Neonatal [0.8]	Congenital [0.38]	Iron [2.64]	Drown [0.9]	Road Inj [0.44]	Headaches [0.83]	Encepha [2.46]	Epilepsy [1.39]	Self Harm [1.3]
Central Europe	Neonatal [0.81]	Congenital [0.67]	Falls [1.76]	Headaches [0.85]	LRI [1.69]	Road Inj [0.51]	Conduct [0.99]	Back Pain [0.89]	Diarrhea [2.06]	Anxiety [0.67]
Albania	Neonatal [0.72]	Congenital [0.75]	LRI [0.86]	Headaches [0.95]	Falls [1.95]	Road Inj [0.41]	Back Pain [1.19]	Conduct [1.0]	Epilepsy [1.24]	Anxiety [0.82]
Bosnia	Neonatal [0.51]	Congenital [0.49]	Headaches [0.93]	Falls [1.92]	Conduct [1.0]	Road Inj [0.34]	Back Pain [0.96]	Anxiety [0.79]	Vit A [1.61]	Dermatitis [0.59]
Bulgaria	Neonatal [0.78]	Congenital [0.77]	LRI [1.84]	Falls [1.87]	Road Inj [0.55]	Headaches [0.83]	Conduct [0.97]	Diarrhea [1.93]	Anxiety [0.71]	Back Pain [0.83]
Croatia	Neonatal [0.77]	Congenital [0.61]	Headaches [0.88]	Road Inj [0.56]	Conduct [1.01]	Back Pain [0.9]	Falls [1.12]	Anxiety [0.68]	Dermatitis [0.56]	Diarrhea [1.79]
Czech	Neonatal [0.56]	Congenital [0.45]	Falls [1.91]	Headaches [0.79]	Back Pain [0.93]	Conduct [0.96]	Road Inj [0.45]	Diarrhea [2.5]	Dermatitis [0.66]	Anxiety [0.61]
Hungary	Neonatal [0.75]	Congenital [0.64]	Headaches [0.87]	Falls [1.71]	Diarrhea [2.85]	Back Pain [1.01]	Conduct [1.01]	Dermatitis [0.76]	Road Inj [0.38]	Anxiety [0.68]
Macedonia	Neonatal [1.12]	Congenital [0.55]	Headaches [0.88]	Falls [1.83]	Road Inj [0.5]	LRI [0.76]	Conduct [0.97]	Iron [1.81]	Diarrhea [1.42]	Anxiety [0.72]
Montenegro	Neonatal [0.69]	Congenital [0.29]	Headaches [0.89]	Falls [1.75]	Road Inj [0.48]	Conduct [1.01]	Back Pain [0.97]	Anxiety [0.71]	Dermatitis [0.57]	Depression [0.65]
Poland	Neonatal [0.9]	Congenital [0.76]	Falls [1.66]	Headaches [0.84]	Road Inj [0.57]	Conduct [0.98]	Dermatitis [0.75]	Anxiety [0.64]	Back Pain [0.72]	Upper Digest [4.49]
Romania	Neonatal [0.73]	LRI [3.57]	Congenital [0.67]	Iron [4.07]	Falls [1.91]	Road Inj [0.58]	Headaches [0.87]	Diarrhea [2.35]	Back Pain [1.08]	Conduct [1.0]
Serbia	Neonatal [0.66]	Congenital [0.37]	Headaches [0.88]	Falls [1.79]	Conduct [1.0]	Back Pain [1.01]	Road Inj [0.33]	Anxiety [0.73]	Dermatitis [0.58]	Diarrhea [0.96]
Slovakia	Neonatal [0.87]	Congenital [0.83]	Falls [1.72]	Headaches [0.84]	Road Inj [0.51]	LRI [1.93]	Back Pain [0.97]	Conduct [0.98]	Diarrhea [2.74]	Anxiety [0.65]
Slovenia	Neonatal [0.56]	Congenital [0.53]	Falls [1.91]	Headaches [0.81]	Road Inj [0.53]	Back Pain [0.98]	Conduct [0.97]	Anxiety [0.61]	Diarrhea [2.45]	Dermatitis [0.57]

eFigure 7a. Leading ten causes of DALYs with the ratio of observed DALYs to DALYs expected on the basis of Socio-Demographic Index alone in 2017, <20 years, both sexes combined. The top ten causes contributing to DALYs are listed globally, by socio-demographic quintile, and then by GBD superregion, region, country, and subnationally where modeled. For each cell, the ratio of observed DALYs to DALYs expected on the basis of socio-demographic index (SDI) alone are listed. Abbreviations: DALY=disability-adjusted life year, GBD=Global Burden of Disease.

Values shown in brackets represent the ratio of observed DALYs to predicted DALYs on the basis of Socio-Demographic Index (SDI), rounded to two (2) digits. Color ranges (shown below) were calculated to place a roughly equal number of cells into each bin.

COLOR KEY:	[0.0-0.5]	(0.5-0.69]	(0.69-0.82]	(0.82-0.92]	(0.92-1.01]	(1.01-1.18]	(1.18-1.48]	(1.48-2.26]	2.26+

	1	2	3	4	5	6	7	8	9	10
**Eastern Europe**	Neonatal [0.87]	Congenital [0.73]	Road Inj [0.67]	LRI [1.18]	Headaches [0.84]	Falls [1.59]	Self Harm [1.33]	Conduct [0.96]	Upper Digest [5.45]	Drown [1.29]
Belarus	Neonatal [0.75]	Congenital [0.68]	Headaches [0.86]	Falls [1.65]	Road Inj [0.4]	Conduct [0.91]	Upper Digest [5.33]	Drown [1.21]	Dermatitis [0.73]	Back Pain [0.75]
Estonia	Neonatal [0.73]	Congenital [0.56]	Headaches [0.88]	Dermatitis [0.94]	Falls [1.28]	Conduct [0.97]	Upper Digest [6.15]	Road Inj [0.38]	Diarrhea [2.37]	Back Pain [0.67]
Latvia	Neonatal [0.79]	Congenital [0.65]	Headaches [0.88]	Road Inj [0.49]	Upper Digest [6.83]	Falls [1.36]	Conduct [0.95]	Drown [1.77]	Dermatitis [0.66]	Diarrhea [2.1]
Lithuania	Neonatal [0.82]	Congenital [0.84]	Headaches [1.01]	Road Inj [0.52]	Falls [1.39]	Upper Digest [6.51]	Conduct [0.98]	Self Harm [1.31]	LRI [1.79]	Diarrhea [2.39]
Moldova	Neonatal [0.76]	Congenital [0.89]	LRI [1.04]	Road Inj [0.51]	Headaches [0.97]	Upper Digest [6.37]	Iron [1.4]	Falls [1.71]	Drown [0.78]	F Body [1.05]
Russian Federation	Neonatal [0.87]	Congenital [0.67]	Road Inj [0.66]	LRI [1.28]	Headaches [0.82]	Falls [1.57]	Conduct [0.96]	Self Harm [1.27]	Upper Digest [5.35]	Drown [1.22]
Ukraine	Neonatal [0.75]	Congenital [0.82]	Road Inj [0.71]	Self Harm [1.77]	Headaches [0.89]	Falls [1.71]	Drown [1.22]	LRI [0.57]	Conduct [0.98]	Upper Digest [5.34]
**High-income**	Neonatal [0.85]	Congenital [0.67]	Dermatitis [1.2]	Headaches [0.93]	Anxiety [1.09]	Road Inj [0.57]	Depression [1.35]	Back Pain [1.02]	Conduct [1.02]	Asthma [1.05]
**Australasia**	Neonatal [0.83]	Congenital [0.61]	Asthma [1.97]	Anxiety [1.19]	Falls [1.71]	Dermatitis [1.08]	Depression [1.52]	Headaches [0.84]	Back Pain [1.14]	Road Inj [0.56]
Australia	Neonatal [0.84]	Congenital [0.62]	Asthma [2.02]	Anxiety [1.14]	Falls [1.65]	Depression [1.54]	Headaches [0.83]	Dermatitis [1.03]	Back Pain [1.08]	Road Inj [0.54]
New Zealand	Neonatal [0.79]	Congenital [0.56]	Anxiety [1.48]	Dermatitis [1.35]	Falls [2.06]	Back Pain [1.43]	Road Inj [0.68]	Asthma [1.68]	Headaches [0.85]	Depression [1.41]
**High-income Asia Pacific**	Neonatal [0.56]	Congenital [0.5]	Dermatitis [1.2]	Back Pain [1.2]	Headaches [0.77]	Anxiety [0.8]	Conduct [0.99]	Falls [1.06]	Depression [0.95]	Road Inj [0.37]
Brunei	Neonatal [1.21]	Congenital [1.31]	Road Inj [0.99]	LRI [4.12]	Dermatitis [1.13]	Headaches [0.74]	Drown [2.56]	Anxiety [0.81]	Back Pain [0.96]	Falls [1.21]
Japan	Neonatal [0.47]	Congenital [0.53]	Dermatitis [1.27]	Back Pain [1.28]	Headaches [0.72]	Conduct [1.03]	Anxiety [0.78]	Falls [1.11]	Depression [0.92]	Asthma [0.8]
Aichi	Neonatal [0.49]	Congenital [0.54]	Dermatitis [1.28]	Back Pain [1.25]	Conduct [1.02]	Anxiety [0.77]	Falls [1.09]	Headaches [0.56]	Depression [0.91]	ASD [2.01]
Akita	Neonatal [0.39]	Congenital [0.43]	Dermatitis [1.25]	Back Pain [1.38]	Headaches [0.77]	Conduct [1.05]	Anxiety [0.85]	Falls [1.19]	Depression [1.05]	Road Inj [0.33]
Aomori	Neonatal [0.41]	Congenital [0.41]	Dermatitis [1.25]	Back Pain [1.37]	Headaches [0.76]	Conduct [1.04]	Anxiety [0.84]	Falls [1.17]	Depression [1.0]	Road Inj [0.33]
Chiba	Neonatal [0.47]	Congenital [0.58]	Dermatitis [1.26]	Back Pain [1.29]	Headaches [0.74]	Conduct [1.04]	Anxiety [0.79]	Falls [1.13]	Depression [0.94]	Road Inj [0.34]
Ehime	Neonatal [0.34]	Congenital [0.39]	Dermatitis [1.27]	Back Pain [1.28]	Headaches [0.73]	Conduct [1.03]	Anxiety [0.81]	Falls [1.16]	Road Inj [0.37]	Depression [0.92]
Fukui	Neonatal [0.41]	Congenital [0.41]	Dermatitis [1.26]	Back Pain [1.29]	Headaches [0.73]	Conduct [1.04]	Anxiety [0.8]	Falls [1.12]	Depression [0.91]	Road Inj [0.33]
Fukuoka	Neonatal [0.46]	Congenital [0.5]	Dermatitis [1.28]	Back Pain [1.24]	Headaches [0.71]	Conduct [1.0]	Anxiety [0.78]	Falls [1.11]	Depression [0.89]	Asthma [0.81]
Fukushima	Neonatal [0.37]	Congenital [0.44]	Dermatitis [1.25]	Back Pain [1.34]	Headaches [0.74]	Conduct [1.04]	Anxiety [0.83]	Falls [1.17]	Depression [0.96]	Road Inj [0.36]
Gifu	Neonatal [0.44]	Congenital [0.46]	Dermatitis [1.26]	Back Pain [1.33]	Headaches [0.75]	Conduct [1.04]	Anxiety [0.81]	Falls [1.16]	Road Inj [0.4]	Depression [0.81]
Gunma	Neonatal [0.42]	Congenital [0.46]	Dermatitis [1.25]	Back Pain [1.34]	Headaches [0.75]	Conduct [1.05]	Anxiety [0.82]	Falls [1.16]	Road Inj [0.41]	Depression [0.99]
Hiroshima	Neonatal [0.47]	Congenital [0.47]	Dermatitis [1.28]	Back Pain [1.23]	Headaches [0.71]	Conduct [1.02]	Anxiety [0.77]	Falls [1.1]	Depression [0.87]	Asthma [0.81]
Hokkaido	Neonatal [0.4]	Congenital [0.46]	Dermatitis [1.26]	Back Pain [1.32]	Headaches [0.74]	Conduct [1.03]	Anxiety [0.81]	Falls [1.14]	Depression [0.95]	Asthma [0.8]
Hyogo	Neonatal [0.44]	Congenital [0.41]	Dermatitis [1.26]	Back Pain [1.29]	Headaches [0.74]	Conduct [1.03]	Anxiety [0.79]	Falls [1.12]	Depression [0.95]	Acne [1.33]
Ibaraki	Neonatal [0.47]	Congenital [0.55]	Dermatitis [1.26]	Back Pain [1.31]	Conduct [1.04]	Anxiety [0.81]	Road Inj [0.48]	Headaches [0.65]	Falls [1.14]	Depression [0.96]
Ishikawa	Neonatal [0.42]	Congenital [0.47]	Dermatitis [1.26]	Back Pain [1.31]	Headaches [0.75]	Conduct [1.04]	Anxiety [0.81]	Falls [1.13]	Depression [0.92]	Road Inj [0.35]
Iwate	Neonatal [0.4]	Congenital [0.46]	Dermatitis [1.26]	Back Pain [1.34]	Headaches [0.74]	Conduct [1.03]	Anxiety [0.83]	Falls [1.17]	Depression [1.0]	Road Inj [0.32]

eFigure 7a. Leading ten causes of DALYs with the ratio of observed DALYs to DALYs expected on the basis of Socio-Demographic Index alone in 2017, <20 years, both sexes combined. The top ten causes contributing to DALYs are listed globally, by socio-demographic quintile, and then by GBD superregion, region, country, and subnationally where modeled. For each cell, the ratio of observed DALYs to DALYs expected on the basis of socio-demographic index (SDI) alone are listed. Abbreviations: DALY=disability-adjusted life year, GBD=Global Burden of Disease.

Values shown in brackets represent the ratio of observed DALYs to predicted DALYs on the basis of Socio-Demographic Index (SDI), rounded to two (2) digits. Color ranges (shown below) were calculated to place a roughly equal number of cells into each bin.

COLOR KEY:	[0.0-0.5]	(0.5-0.69]	(0.69-0.82]	(0.82-0.92]	(0.92-1.01]	(1.01-1.18]	(1.18-1.48]	(1.48-2.26]	2.26+

	1	2	3	4	5	6	7	8	9	10
Kagawa	Neonatal [0.4]	Congenital [0.44]	Dermatitis [1.26]	Back Pain [1.28]	Headaches [0.74]	Conduct [1.04]	Anxiety [0.81]	Road Inj [0.47]	Falls [1.14]	Depression [0.91]
Kagoshima	Neonatal [0.36]	Congenital [0.45]	Dermatitis [1.29]	Back Pain [1.23]	Headaches [0.7]	Conduct [1.0]	Anxiety [0.79]	Falls [1.14]	Road Inj [0.32]	Asthma [0.78]
Kanagawa	Neonatal [0.51]	Congenital [0.61]	Dermatitis [1.27]	Back Pain [1.26]	Headaches [0.73]	Conduct [1.03]	Anxiety [0.77]	Falls [1.11]	Depression [0.95]	Asthma [0.83]
Kochi	Neonatal [0.38]	Congenital [0.43]	Dermatitis [1.26]	Back Pain [1.32]	Headaches [0.74]	Conduct [1.03]	Anxiety [0.83]	Falls [1.2]	Depression [0.94]	Road Inj [0.36]
Kumamoto	Neonatal [0.34]	Congenital [0.38]	Dermatitis [1.29]	Back Pain [1.23]	Headaches [0.69]	Conduct [0.99]	Anxiety [0.79]	Falls [1.13]	Depression [0.86]	Asthma [0.79]
Kyoto	Neonatal [0.52]	Congenital [0.64]	Back Pain [1.34]	Dermatitis [0.99]	Headaches [0.76]	Conduct [1.05]	Anxiety [0.79]	Falls [1.11]	Depression [0.97]	Asthma [0.81]
Mie	Neonatal [0.47]	Congenital [0.46]	Dermatitis [1.27]	Back Pain [1.28]	Headaches [0.73]	Conduct [1.03]	Anxiety [0.8]	Falls [1.13]	Road Inj [0.41]	Depression [0.9]
Miyagi	Neonatal [0.43]	Congenital [0.53]	Dermatitis [1.26]	Back Pain [1.32]	Headaches [0.74]	Conduct [1.03]	Anxiety [0.8]	Falls [1.14]	Depression [0.99]	Road Inj [0.32]
Miyazaki	Neonatal [0.35]	Congenital [0.41]	Dermatitis [1.29]	Back Pain [1.25]	Headaches [0.7]	Conduct [1.0]	Anxiety [0.8]	Falls [1.15]	Depression [0.9]	Iron [3.0]
Nagano	Neonatal [0.41]	Congenital [0.4]	Dermatitis [1.26]	Back Pain [1.3]	Headaches [0.74]	Conduct [1.04]	Anxiety [0.8]	Falls [1.15]	Depression [0.93]	Road Inj [0.34]
Nagasaki	Neonatal [0.35]	Congenital [0.43]	Dermatitis [1.28]	Back Pain [1.25]	Headaches [0.72]	Conduct [1.0]	Anxiety [0.81]	Falls [1.15]	Depression [0.89]	Asthma [0.78]
Nara	Neonatal [0.38]	Congenital [0.51]	Dermatitis [1.26]	Back Pain [1.37]	Headaches [0.76]	Conduct [1.05]	Anxiety [0.82]	Falls [1.17]	Depression [0.95]	Road Inj [0.34]
Niigata	Neonatal [0.41]	Congenital [0.45]	Dermatitis [1.26]	Back Pain [1.47]	Headaches [0.74]	Conduct [1.03]	Anxiety [0.81]	Falls [1.14]	Depression [0.99]	Asthma [0.79]
Oita	Neonatal [0.45]	Congenital [0.45]	Dermatitis [1.27]	Back Pain [1.26]	Headaches [0.72]	Conduct [1.02]	Anxiety [0.79]	Falls [1.14]	Depression [0.91]	Road Inj [0.34]
Okayama	Neonatal [0.43]	Congenital [0.42]	Dermatitis [1.26]	Back Pain [1.29]	Headaches [0.74]	Conduct [1.03]	Anxiety [0.79]	Falls [1.12]	Depression [0.91]	Road Inj [0.33]
Okinawa	Neonatal [0.36]	Congenital [0.35]	Dermatitis [1.3]	Back Pain [1.19]	Headaches [0.68]	Conduct [0.96]	Anxiety [0.78]	Falls [1.12]	Depression [0.84]	Road Inj [0.28]
Osaka	Neonatal [0.45]	Congenital [0.53]	Dermatitis [1.26]	Back Pain [1.3]	Headaches [0.74]	Conduct [1.04]	Anxiety [0.79]	Falls [1.12]	Depression [0.98]	Asthma [0.82]
Saga	Neonatal [0.36]	Congenital [0.35]	Dermatitis [1.28]	Back Pain [1.27]	Headaches [0.72]	Conduct [1.01]	Anxiety [0.8]	Falls [1.13]	Road Inj [0.35]	Depression [0.88]
Saitama	Neonatal [0.42]	Congenital [0.49]	Dermatitis [1.27]	Back Pain [1.33]	Headaches [0.73]	Conduct [1.02]	Anxiety [0.8]	Falls [1.15]	Depression [0.96]	Asthma [0.8]
Shiga	Neonatal [0.46]	Congenital [0.51]	Dermatitis [1.55]	Back Pain [1.27]	Headaches [0.73]	Conduct [1.04]	Anxiety [0.78]	Falls [1.09]	Depression [0.89]	Road Inj [0.34]
Shimane	Neonatal [0.35]	Congenital [0.36]	Dermatitis [1.27]	Back Pain [1.19]	Headaches [0.71]	Conduct [1.02]	Anxiety [0.8]	Falls [1.14]	Depression [0.93]	Asthma [0.77]
Shizuoka	Neonatal [0.47]	Congenital [0.46]	Dermatitis [1.27]	Back Pain [1.25]	Headaches [0.72]	Conduct [1.03]	Anxiety [0.79]	Falls [1.11]	Depression [0.9]	Asthma [0.8]
Tochigi	Neonatal [0.47]	Congenital [0.6]	Dermatitis [1.27]	Back Pain [1.29]	Headaches [0.73]	Conduct [1.03]	Anxiety [0.79]	Falls [1.12]	Road Inj [0.43]	Depression [0.91]
Tokushima	Neonatal [0.51]	Congenital [0.58]	Dermatitis [1.26]	Back Pain [1.29]	Headaches [0.74]	Conduct [1.02]	Anxiety [0.81]	Falls [1.16]	Road Inj [0.41]	Depression [0.93]
Tokyo	Neonatal [0.69]	Congenital [0.9]	Dermatitis [1.31]	Back Pain [1.19]	Headaches [0.71]	Conduct [1.02]	Anxiety [0.71]	Falls [1.01]	Depression [0.83]	Asthma [0.88]
Tottori	Neonatal [0.42]	Congenital [0.49]	Dermatitis [1.28]	Back Pain [1.26]	Headaches [0.67]	Conduct [1.01]	Anxiety [0.8]	Falls [1.15]	Road Inj [0.38]	Depression [0.9]
Toyama	Neonatal [0.43]	Congenital [0.49]	Dermatitis [1.26]	Back Pain [1.28]	Headaches [0.73]	Conduct [1.05]	Anxiety [0.8]	Falls [1.13]	Road Inj [0.39]	Depression [0.93]
Wakayama	Neonatal [0.42]	Congenital [0.41]	Back Pain [1.48]	Dermatitis [1.26]	Headaches [0.73]	Conduct [1.03]	Anxiety [0.81]	Falls [1.14]	Depression [0.94]	Asthma [0.78]
Yamagata	Neonatal [0.38]	Congenital [0.46]	Dermatitis [1.26]	Back Pain [1.34]	Headaches [0.74]	Conduct [1.04]	Anxiety [0.83]	Falls [1.17]	Depression [0.92]	Iron [2.93]
Yamaguchi	Neonatal [0.4]	Congenital [0.44]	Dermatitis [1.27]	Back Pain [1.28]	Headaches [0.73]	Conduct [1.03]	Anxiety [0.8]	Falls [1.13]	Depression [0.92]	Road Inj [0.34]
Yamanashi	Neonatal [0.45]	Congenital [0.44]	Dermatitis [1.24]	Back Pain [1.36]	Headaches [0.76]	Conduct [1.07]	Anxiety [0.82]	Falls [1.15]	Depression [0.96]	Road Inj [0.34]

eFigure 7a. Leading ten causes of DALYs with the ratio of observed DALYs to DALYs expected on the basis of Socio-Demographic Index alone in 2017, <20 years, both sexes combined. The top ten causes contributing to DALYs are listed globally, by socio-demographic quintile, and then by GBD superregion, region, country, and subnationally where modeled. For each cell, the ratio of observed DALYs to DALYs expected on the basis of socio-demographic index (SDI) alone are listed. Abbreviations: DALY=disability-adjusted life year, GBD=Global Burden of Disease.

Values shown in brackets represent the ratio of observed DALYs to predicted DALYs on the basis of Socio-Demographic Index (SDI), rounded to two (2) digits. Color ranges (shown below) were calculated to place a roughly equal number of cells into each bin.

COLOR KEY:	[0.0-0.5]	(0.5-0.69]	(0.69-0.82]	(0.82-0.92]	(0.92-1.01]	(1.01-1.18]	(1.18-1.48]	(1.48-2.26]	2.26+

	1	2	3	4	5	6	7	8	9	10
S Korea	Neonatal [0.74]	Congenital [0.42]	Headaches [0.89]	Dermatitis [1.08]	Back Pain [1.08]	Anxiety [0.85]	Road Inj [0.46]	Conduct [0.9]	Depression [0.99]	Falls [0.98]
Singapore	Neonatal [0.48]	Congenital [0.4]	Dermatitis [1.02]	Headaches [0.71]	Depression [1.2]	Anxiety [0.8]	Back Pain [0.88]	Conduct [0.87]	Falls [0.98]	Asthma [0.79]
**High-income North America**	Neonatal [1.1]	Congenital [0.78]	Drugs [5.28]	Road Inj [0.82]	Dermatitis [1.34]	Headaches [1.01]	Depression [1.77]	Anxiety [1.01]	Conduct [1.19]	Asthma [1.2]
Canada	Neonatal [0.96]	Congenital [0.78]	Dermatitis [1.45]	Headaches [1.0]	Drugs [4.05]	Road Inj [0.66]	Depression [1.28]	Asthma [1.28]	Anxiety [0.8]	Conduct [0.98]
Greenland	Neonatal [1.19]	Self Harm [6.42]	Congenital [0.7]	Depression [2.35]	Violence [3.53]	Dermatitis [1.39]	SIDS [2.74]	Headaches [0.97]	Drown [1.61]	LRI [0.83]
USA	Neonatal [1.09]	Congenital [0.77]	Drugs [5.43]	Road Inj [0.83]	Dermatitis [1.33]	Depression [1.82]	Headaches [1.01]	Anxiety [1.03]	Conduct [1.21]	Violence [3.48]
Alabama	Neonatal [1.23]	Congenital [0.83]	Road Inj [1.37]	Drugs [6.93]	Headaches [1.04]	Violence [4.45]	Depression [1.82]	Dermatitis [1.16]	Anxiety [1.08]	SIDS [2.21]
Alaska	Neonatal [0.82]	Congenital [0.73]	Drugs [5.68]	Headaches [0.95]	Depression [1.71]	Dermatitis [1.2]	Self Harm [1.95]	Road Inj [0.68]	Anxiety [0.99]	SIDS [2.27]
Arizona	Neonatal [0.81]	Congenital [0.68]	Drugs [7.13]	Depression [2.1]	Road Inj [0.83]	Dermatitis [1.36]	Headaches [1.03]	Anxiety [1.07]	Conduct [1.19]	Asthma [1.26]
Arkansas	Neonatal [0.88]	Congenital [0.73]	Road Inj [1.28]	Drugs [6.45]	Depression [2.11]	Headaches [1.02]	SIDS [2.7]	Anxiety [1.08]	Violence [3.46]	Dermatitis [1.07]
California	Neonatal [0.89]	Congenital [0.71]	Dermatitis [1.49]	Headaches [1.02]	Depression [1.76]	Drugs [3.9]	Anxiety [1.0]	Road Inj [0.61]	Conduct [1.2]	Violence [3.61]
Colorado	Neonatal [1.09]	Congenital [0.78]	Depression [2.02]	Drugs [4.73]	Headaches [1.03]	Road Inj [0.77]	Dermatitis [1.19]	Anxiety [1.02]	Self Harm [1.75]	Conduct [1.22]
Connecticut	Neonatal [1.3]	Drugs [6.52]	Congenital [0.65]	Dermatitis [1.49]	Headaches [1.07]	Depression [1.67]	Anxiety [1.03]	Asthma [1.53]	Conduct [1.25]	Road Inj [0.63]
Delaware	Neonatal [1.6]	Congenital [0.86]	Drugs [7.28]	Road Inj [0.9]	Depression [1.92]	Headaches [1.04]	Dermatitis [1.19]	Anxiety [1.04]	Conduct [1.2]	Asthma [1.28]
DC	Neonatal [3.43]	Congenital [1.73]	Violence [17.47]	SIDS [4.38]	Dermatitis [1.85]	Drugs [4.46]	F Body [5.64]	Headaches [0.89]	LRI [4.78]	Road Inj [0.61]
Florida	Neonatal [1.24]	Congenital [0.81]	Drugs [5.74]	Road Inj [0.91]	Dermatitis [1.31]	Headaches [1.03]	Depression [1.8]	Anxiety [1.04]	Violence [4.31]	Conduct [1.2]
Georgia	Neonatal [1.15]	Congenital [0.73]	Road Inj [0.97]	Drugs [5.23]	Dermatitis [1.43]	Headaches [1.04]	Depression [1.76]	Violence [4.13]	Anxiety [1.06]	Conduct [1.2]
Hawaii	Neonatal [1.17]	Congenital [0.73]	Dermatitis [1.58]	Headaches [0.97]	Depression [1.71]	Road Inj [0.64]	Anxiety [0.99]	Conduct [1.16]	Asthma [1.28]	Drugs [2.61]
Idaho	Neonatal [0.67]	Congenital [0.57]	Road Inj [0.89]	Drugs [5.29]	Depression [2.02]	Headaches [1.01]	Dermatitis [1.1]	Anxiety [1.06]	Conduct [1.19]	Self Harm [1.57]
Illinois	Neonatal [1.31]	Congenital [0.8]	Drugs [4.74]	Headaches [1.03]	Violence [5.54]	Depression [1.67]	Dermatitis [1.16]	Anxiety [1.03]	Road Inj [0.66]	Conduct [1.21]
Indiana	Neonatal [1.06]	Congenital [0.82]	Drugs [6.73]	Road Inj [0.95]	Depression [1.94]	Headaches [1.02]	Dermatitis [1.15]	Anxiety [1.06]	Conduct [1.19]	Violence [3.5]
Iowa	Neonatal [0.75]	Congenital [0.68]	Road Inj [0.88]	Headaches [1.01]	Back Pain [1.44]	Depression [1.71]	Anxiety [1.03]	Dermatitis [1.05]	Conduct [1.19]	Drugs [2.78]
Kansas	Neonatal [0.97]	Congenital [0.75]	Road Inj [1.0]	Depression [1.8]	Headaches [1.0]	Dermatitis [1.11]	Anxiety [1.02]	Conduct [1.19]	Drugs [3.48]	Back Pain [1.11]
Kentucky	Neonatal [0.91]	Congenital [0.77]	Drugs [9.9]	Road Inj [1.08]	Depression [1.94]	Headaches [1.01]	Dermatitis [1.17]	Anxiety [1.07]	SIDS [2.19]	Conduct [1.17]
Louisiana	Neonatal [1.16]	Congenital [0.85]	Road Inj [1.13]	Violence [6.42]	Drugs [6.96]	Headaches [0.99]	Dermatitis [1.26]	Depression [1.69]	Anxiety [1.04]	SIDS [2.23]
Maine	Neonatal [1.15]	Congenital [0.89]	Drugs [7.5]	Road Inj [1.01]	Depression [1.97]	Headaches [1.06]	Anxiety [1.06]	Conduct [1.23]	Dermatitis [0.99]	Asthma [1.3]
Maryland	Neonatal [1.65]	Congenital [0.88]	Dermatitis [1.51]	Headaches [1.01]	Violence [5.85]	Depression [1.7]	Road Inj [0.73]	Drugs [3.84]	Anxiety [1.0]	Conduct [1.21]
Massachusetts	Neonatal [1.39]	Congenital [0.8]	Drugs [6.42]	Dermatitis [1.35]	Headaches [1.06]	Depression [1.79]	Anxiety [1.01]	Conduct [1.24]	Asthma [1.34]	Road Inj [0.56]
Michigan	Neonatal [1.24]	Congenital [0.82]	Drugs [6.46]	Depression [1.92]	Road Inj [0.81]	Dermatitis [1.19]	Anxiety [1.05]	Headaches [0.85]	Violence [4.22]	Conduct [1.21]
Minnesota	Neonatal [1.04]	Congenital [0.87]	Headaches [1.01]	Depression [1.74]	Anxiety [1.0]	Dermatitis [1.05]	Road Inj [0.66]	Conduct [1.21]	Drugs [2.68]	Self Harm [1.28]
Mississippi	Neonatal [1.12]	Congenital [0.81]	Road Inj [1.58]	Drugs [6.45]	Violence [4.22]	Headaches [1.03]	Depression [1.77]	Anxiety [1.1]	Dermatitis [1.06]	F Body [3.19]

eFigure 7a. Leading ten causes of DALYs with the ratio of observed DALYs to DALYs expected on the basis of Socio-Demographic Index alone in 2017, <20 years, both sexes combined. The top ten causes contributing to DALYs are listed globally, by socio-demographic quintile, and then by GBD superregion, region, country, and subnationally where modeled. For each cell, the ratio of observed DALYs to DALYs expected on the basis of socio-demographic index (SDI) alone are listed. Abbreviations: DALY=disability-adjusted life year, GBD=Global Burden of Disease.

Values shown in brackets represent the ratio of observed DALYs to predicted DALYs on the basis of Socio-Demographic Index (SDI), rounded to two (2) digits. Color ranges (shown below) were calculated to place a roughly equal number of cells into each bin.

COLOR KEY:	[0.0-0.5]	(0.5-0.69]	(0.69-0.82]	(0.82-0.92]	(0.92-1.01]	(1.01-1.18]	(1.18-1.48]	(1.48-2.26]	2.26+

	1	2	3	4	5	6	7	8	9	10
Missouri	Neonatal [1.04]	Congenital [0.77]	Road Inj [1.04]	Drugs [5.85]	Depression [1.94]	Headaches [1.01]	Violence [4.34]	Anxiety [1.04]	Dermatitis [1.07]	Conduct [1.18]
Montana	Neonatal [0.92]	Congenital [0.85]	Road Inj [1.45]	Drugs [5.27]	Depression [1.87]	Headaches [0.99]	Self Harm [2.03]	Anxiety [1.02]	Dermatitis [1.05]	Conduct [1.18]
Nebraska	Neonatal [0.94]	Congenital [0.78]	Road Inj [1.0]	Headaches [0.99]	Depression [1.65]	Dermatitis [1.06]	Anxiety [1.0]	Back Pain [1.19]	Conduct [1.18]	Drugs [2.7]
Nevada	Neonatal [0.83]	Congenital [0.64]	Drugs [7.15]	Road Inj [0.8]	Depression [1.88]	Dermatitis [1.3]	Headaches [1.0]	Violence [3.95]	Anxiety [1.04]	Conduct [1.17]
New Hampshire	Neonatal [1.07]	Drugs [7.55]	Congenital [0.74]	Depression [2.06]	Headaches [1.08]	Road Inj [0.74]	Anxiety [1.04]	Dermatitis [1.04]	Conduct [1.27]	Asthma [1.2]
New Jersey	Neonatal [1.14]	Congenital [0.73]	Dermatitis [1.62]	Drugs [5.16]	Headaches [1.03]	Depression [1.53]	Anxiety [1.01]	Asthma [1.53]	Conduct [1.22]	Road Inj [0.53]
New Mexico	Neonatal [0.79]	Congenital [0.67]	Road Inj [1.04]	Drugs [6.65]	Depression [2.11]	Headaches [1.02]	Dermatitis [1.17]	Anxiety [1.08]	Self Harm [1.7]	Conduct [1.19]
New York	Neonatal [1.26]	Congenital [0.83]	Dermatitis [1.68]	Depression [1.84]	Drugs [4.49]	Headaches [1.01]	Asthma [1.68]	Anxiety [0.99]	Conduct [1.19]	Road Inj [0.51]
N Carolina	Neonatal [1.18]	Congenital [0.75]	Road Inj [0.97]	Drugs [5.63]	Headaches [1.03]	Depression [1.79]	Dermatitis [1.23]	Anxiety [0.99]	Conduct [1.11]	Violence [3.27]
N Dakota	Neonatal [1.19]	Congenital [1.06]	Road Inj [1.19]	Headaches [0.93]	Depression [1.49]	SIDS [2.4]	Dermatitis [1.04]	Anxiety [0.94]	Conduct [1.12]	Self Harm [1.51]
Ohio	Neonatal [1.22]	Congenital [0.86]	Drugs [7.92]	Road Inj [0.78]	Depression [1.86]	Headaches [1.03]	Dermatitis [1.26]	Anxiety [1.05]	Conduct [1.19]	Violence [3.41]
Oklahoma	Neonatal [1.07]	Congenital [0.9]	Road Inj [1.25]	Drugs [7.8]	Depression [1.9]	Dermatitis [1.32]	Headaches [1.01]	Anxiety [1.05]	Violence [3.57]	SIDS [2.14]
Oregon	Neonatal [0.98]	Congenital [0.7]	Drugs [6.53]	Depression [1.99]	Dermatitis [1.39]	Headaches [1.02]	Road Inj [0.67]	Anxiety [0.95]	Conduct [1.08]	Self Harm [1.36]
Pennsylvania	Neonatal [1.32]	Congenital [0.81]	Drugs [7.79]	Dermatitis [1.3]	Depression [1.8]	Road Inj [0.78]	Headaches [0.94]	Anxiety [1.03]	Conduct [1.21]	Back Pain [1.05]
Rhode Island	Neonatal [1.46]	Drugs [8.15]	Congenital [0.81]	Depression [2.02]	Headaches [1.07]	Dermatitis [1.18]	Anxiety [1.04]	Conduct [1.23]	Road Inj [0.57]	Asthma [1.26]
S Carolina	Neonatal [1.16]	Congenital [0.71]	Road Inj [1.22]	Drugs [6.64]	Dermatitis [1.42]	Depression [1.88]	Headaches [1.03]	Anxiety [1.07]	Violence [3.82]	Conduct [1.19]
S Dakota	Neonatal [0.91]	Congenital [0.87]	Road Inj [1.37]	Headaches [0.96]	Self Harm [1.83]	Depression [1.52]	Dermatitis [1.03]	Anxiety [1.0]	Back Pain [1.17]	Conduct [1.16]
Tennessee	Neonatal [0.94]	Congenital [0.77]	Drugs [8.1]	Road Inj [0.99]	Depression [1.9]	Headaches [1.02]	Dermatitis [1.18]	Violence [3.77]	Anxiety [1.06]	Conduct [1.17]
Texas	Neonatal [0.88]	Congenital [0.69]	Road Inj [0.89]	Dermatitis [1.39]	Headaches [1.01]	Depression [1.76]	Conduct [1.36]	Anxiety [1.06]	Drugs [3.73]	Violence [2.99]
Utah	Neonatal [0.71]	Congenital [0.63]	Drugs [6.0]	Depression [2.14]	Headaches [1.0]	Road Inj [0.67]	Dermatitis [1.12]	Self Harm [1.86]	Anxiety [1.03]	Conduct [1.18]
Vermont	Neonatal [1.08]	Congenital [0.79]	Drugs [5.68]	Headaches [1.08]	Depression [1.91]	Road Inj [0.85]	Anxiety [1.05]	Dermatitis [1.02]	Conduct [1.26]	Asthma [1.08]
Virginia	Neonatal [1.29]	Congenital [0.82]	Dermatitis [1.51]	Depression [1.9]	Headaches [1.02]	Drugs [4.34]	Road Inj [0.75]	Anxiety [1.01]	Conduct [1.2]	Asthma [1.21]
Washington	Neonatal [0.95]	Congenital [0.78]	Drugs [5.22]	Dermatitis [1.36]	Depression [1.92]	Headaches [1.0]	Anxiety [1.0]	Road Inj [0.6]	Conduct [1.19]	Self Harm [1.26]
W Virginia	Neonatal [0.96]	Drugs [12.77]	Congenital [0.77]	Road Inj [1.16]	Depression [1.97]	Headaches [1.02]	Dermatitis [1.19]	Anxiety [1.08]	SIDS [2.23]	Conduct [1.17]
Wisconsin	Neonatal [1.12]	Congenital [0.79]	Drugs [5.47]	Depression [1.92]	Headaches [1.03]	Road Inj [0.75]	Anxiety [1.03]	Dermatitis [1.05]	Conduct [1.21]	Back Pain [0.95]
Wyoming	Neonatal [1.04]	Congenital [0.75]	Road Inj [1.27]	Depression [1.98]	Headaches [0.99]	Self Harm [1.96]	Drugs [3.97]	Dermatitis [1.1]	Anxiety [1.01]	Back Pain [1.13]
**Southern Latin America**	Neonatal [0.75]	Congenital [0.71]	Road Inj [0.6]	Anxiety [1.34]	LRI [0.48]	Back Pain [1.3]	Diarrhea [1.54]	Dermatitis [1.03]	Depression [1.33]	Self Harm [1.3]
Argentina	Neonatal [0.79]	Congenital [0.69]	Road Inj [0.63]	LRI [0.51]	Anxiety [1.35]	Diarrhea [1.54]	Self Harm [1.43]	Dermatitis [0.97]	Back Pain [1.17]	Depression [1.3]
Chile	Neonatal [0.64]	Congenital [0.77]	Back Pain [1.71]	Anxiety [1.3]	Dermatitis [1.21]	Road Inj [0.47]	Depression [1.46]	Headaches [0.69]	Diarrhea [1.53]	Conduct [0.9]
Uruguay	Neonatal [0.56]	Congenital [0.64]	Road Inj [0.59]	Anxiety [1.4]	LRI [0.37]	Dermatitis [0.95]	Headaches [0.67]	Self Harm [1.27]	Back Pain [1.07]	Asthma [1.01]
**Western Europe**	Neonatal [0.67]	Congenital [0.57]	Anxiety [1.23]	Headaches [0.97]	Dermatitis [1.12]	Back Pain [1.08]	Depression [1.08]	Conduct [0.91]	Asthma [0.95]	Road Inj [0.37]

eFigure 7a. Leading ten causes of DALYs with the ratio of observed DALYs to DALYs expected on the basis of Socio-Demographic Index alone in 2017, <20 years, both sexes combined. The top ten causes contributing to DALYs are listed globally, by socio-demographic quintile, and then by GBD superregion, region, country, and subnationally where modeled. For each cell, the ratio of observed DALYs to DALYs expected on the basis of socio-demographic index (SDI) alone are listed. Abbreviations: DALY=disability-adjusted life year, GBD=Global Burden of Disease.

Values shown in brackets represent the ratio of observed DALYs to predicted DALYs on the basis of Socio-Demographic Index (SDI), rounded to two (2) digits. Color ranges (shown below) were calculated to place a roughly equal number of cells into each bin.

COLOR KEY:								
[0.0-0.5]	(0.5-0.69]	(0.69-0.82]	(0.82-0.92]	(0.92-1.01]	(1.01-1.18]	(1.18-1.48]	(1.48-2.26]	2.26+

	1	2	3	4	5	6	7	8	9	10
Andorra	Neonatal [0.48]	Dermatitis [1.4]	Headaches [1.07]	Anxiety [1.21]	Congenital [0.44]	Back Pain [1.1]	Depression [1.11]	Conduct [0.98]	Asthma [1.13]	Road Inj [0.43]
Austria	Neonatal [0.83]	Congenital [0.64]	Headaches [1.02]	Anxiety [1.17]	Dermatitis [0.76]	Back Pain [0.85]	Conduct [0.89]	Road Inj [0.43]	Falls [0.87]	Asthma [0.83]
Belgium	Neonatal [0.65]	Congenital [0.59]	Headaches [1.2]	Anxiety [1.1]	Dermatitis [0.96]	Back Pain [0.92]	Conduct [0.89]	Depression [1.01]	Road Inj [0.43]	Falls [0.93]
Cyprus	Neonatal [0.72]	Congenital [0.39]	Road Inj [0.74]	Headaches [0.97]	Anxiety [1.15]	Back Pain [1.07]	Dermatitis [0.8]	Conduct [0.88]	Depression [0.98]	Asthma [0.85]
Denmark	Neonatal [0.89]	Congenital [0.79]	Dermatitis [1.32]	Anxiety [1.15]	Back Pain [1.37]	Headaches [0.85]	Conduct [0.92]	Asthma [0.99]	Road Inj [0.39]	Depression [0.81]
Finland	Neonatal [0.82]	Congenital [0.58]	Dermatitis [1.39]	Headaches [0.98]	Depression [1.34]	Anxiety [0.83]	Conduct [0.89]	Road Inj [0.45]	Asthma [0.98]	Falls [0.86]
France	Neonatal [0.65]	Congenital [0.54]	Anxiety [1.4]	Dermatitis [1.25]	Headaches [0.81]	Back Pain [1.06]	Depression [1.12]	Conduct [0.9]	Road Inj [0.43]	Asthma [0.93]
Germany	Neonatal [0.79]	Congenital [0.63]	Anxiety [1.41]	Back Pain [1.41]	Headaches [0.88]	Dermatitis [0.92]	Depression [1.06]	Conduct [0.91]	Road Inj [0.42]	Epilepsy [1.8]
Greece	Neonatal [0.55]	Congenital [0.63]	Headaches [1.18]	Anxiety [1.34]	Road Inj [0.61]	Depression [1.37]	Back Pain [0.95]	Conduct [0.88]	Dermatitis [0.66]	Asthma [0.78]
Iceland	Neonatal [0.53]	Congenital [0.56]	Dermatitis [1.45]	Headaches [0.97]	Anxiety [1.11]	Asthma [1.47]	Back Pain [0.98]	Conduct [0.9]	Road Inj [0.39]	Depression [0.85]
Ireland	Neonatal [0.63]	Congenital [0.81]	Anxiety [1.23]	Dermatitis [1.19]	Headaches [0.94]	Asthma [1.28]	Depression [1.14]	Conduct [0.88]	Back Pain [0.76]	Falls [0.8]
Israel	Neonatal [0.56]	Congenital [0.56]	Headaches [0.88]	Dermatitis [1.06]	Back Pain [1.05]	Depression [1.04]	Road Inj [0.38]	Blindness [2.78]	Anxiety [0.69]	Conduct [0.81]
Italy	Neonatal [0.52]	Congenital [0.46]	Headaches [1.24]	Anxiety [1.28]	Dermatitis [0.94]	Back Pain [1.03]	Depression [1.11]	Conduct [0.9]	Road Inj [0.38]	Acne [1.39]
Luxembourg	Neonatal [0.73]	Headaches [1.13]	Dermatitis [1.41]	Anxiety [1.11]	Congenital [0.45]	Back Pain [0.96]	Asthma [1.18]	Road Inj [0.5]	Conduct [0.91]	Depression [0.94]
Malta	Neonatal [0.88]	Congenital [1.03]	Headaches [0.97]	Anxiety [1.18]	Back Pain [1.08]	Dermatitis [0.82]	Conduct [0.85]	Depression [1.0]	Falls [1.03]	Asthma [0.93]
Netherlands	Neonatal [0.94]	Congenital [0.77]	Anxiety [1.41]	Headaches [1.08]	Dermatitis [1.22]	Conduct [1.03]	Depression [1.11]	Back Pain [0.81]	Asthma [1.04]	Acne [1.28]
Norway	Neonatal [0.7]	Congenital [0.66]	Anxiety [1.55]	Dermatitis [1.37]	Headaches [1.03]	Asthma [1.39]	Back Pain [0.91]	Conduct [0.98]	Falls [0.97]	Depression [0.98]
Portugal	Neonatal [0.34]	Congenital [0.31]	Headaches [1.07]	Anxiety [1.35]	Back Pain [1.19]	Asthma [1.3]	Depression [1.4]	Dermatitis [0.86]	Conduct [0.91]	Road Inj [0.35]
Spain	Neonatal [0.48]	Congenital [0.41]	Headaches [1.15]	Anxiety [1.15]	Back Pain [1.15]	Conduct [0.88]	Depression [1.02]	Dermatitis [0.71]	Asthma [0.76]	Road Inj [0.29]
Sweden	Neonatal [0.63]	Congenital [0.65]	Dermatitis [1.76]	Headaches [0.99]	Anxiety [1.09]	Asthma [1.38]	Depression [1.13]	Conduct [0.95]	Back Pain [0.85]	Falls [0.94]
Stockholm	Neonatal [0.65]	Congenital [0.82]	Dermatitis [1.42]	Headaches [0.95]	Anxiety [1.04]	Asthma [1.23]	Conduct [0.95]	Falls [0.88]	Depression [0.88]	Acne [1.27]
Sweden w/o Stockholm	Neonatal [0.62]	Congenital [0.6]	Dermatitis [1.86]	Headaches [1.0]	Anxiety [1.11]	Asthma [1.43]	Depression [1.2]	Back Pain [0.95]	Conduct [0.95]	Falls [0.96]
Switzerland	Neonatal [0.88]	Congenital [0.81]	Back Pain [1.96]	Dermatitis [1.41]	Anxiety [1.12]	Headaches [0.81]	Conduct [0.89]	Asthma [0.97]	Depression [0.92]	Falls [0.7]
UK	Neonatal [0.83]	Congenital [0.65]	Dermatitis [1.5]	Headaches [0.88]	Asthma [1.52]	Anxiety [1.0]	Conduct [0.94]	Depression [1.05]	Back Pain [0.86]	Falls [0.84]
England	Neonatal [0.88]	Congenital [0.67]	Dermatitis [1.53]	Headaches [0.86]	Asthma [1.54]	Anxiety [0.97]	Conduct [0.95]	Back Pain [0.86]	Depression [1.03]	Falls [0.82]
E Midlands	Neonatal [0.81]	Congenital [0.62]	Dermatitis [1.5]	Headaches [0.88]	Asthma [1.45]	Anxiety [1.0]	Conduct [0.96]	Back Pain [0.88]	Depression [1.06]	Road Inj [0.31]
Derby	Neonatal [1.04]	Congenital [0.78]	Dermatitis [1.51]	Headaches [0.84]	Asthma [1.45]	Anxiety [0.96]	Conduct [0.94]	Back Pain [0.85]	Depression [1.0]	Falls [0.84]
Derbyshire	Neonatal [0.6]	Congenital [0.5]	Dermatitis [1.49]	Headaches [0.9]	Asthma [1.43]	Anxiety [1.04]	Conduct [0.97]	Depression [1.09]	Back Pain [0.8]	Falls [0.91]
Leicester	Neonatal [1.12]	Congenital [0.86]	Dermatitis [1.5]	Headaches [0.86]	Asthma [1.47]	Anxiety [0.97]	Conduct [0.94]	Back Pain [0.88]	Depression [1.03]	Falls [0.86]
Leicestershire	Neonatal [0.76]	Congenital [0.59]	Dermatitis [1.48]	Headaches [0.92]	Anxiety [1.01]	Asthma [1.45]	Conduct [0.99]	Back Pain [0.94]	Depression [1.1]	Falls [0.84]
Lincolnshire	Neonatal [0.64]	Congenital [0.52]	Dermatitis [1.5]	Headaches [0.89]	Asthma [1.43]	Anxiety [1.03]	Conduct [0.95]	Back Pain [0.92]	Road Inj [0.4]	Depression [1.07]

eFigure 7a. Leading ten causes of DALYs with the ratio of observed DALYs to DALYs expected on the basis of Socio-Demographic Index alone in 2017, <20 years, both sexes combined. The top ten causes contributing to DALYs are listed globally, by socio-demographic quintile, and then by GBD superregion, region, country, and subnationally where modeled. For each cell, the ratio of observed DALYs to DALYs expected on the basis of socio-demographic index (SDI) alone are listed. Abbreviations: DALY=disability-adjusted life year, GBD=Global Burden of Disease.

Values shown in brackets represent the ratio of observed DALYs to predicted DALYs on the basis of Socio-Demographic Index (SDI), rounded to two (2) digits. Color ranges (shown below) were calculated to place a roughly equal number of cells into each bin.

COLOR KEY:	[0.0-0.5]	(0.5-0.69]	(0.69-0.82]	(0.82-0.92]	(0.92-1.01]	(1.01-1.18]	(1.18-1.48]	(1.48-2.26]	2.26+

	1	2	3	4	5	6	7	8	9	10
Northamptonshire	Neonatal [0.86]	Congenital [0.62]	Dermatitis [1.51]	Headaches [0.85]	Asthma [1.44]	Anxiety [0.98]	Conduct [0.94]	Back Pain [0.87]	Depression [1.01]	Road Inj [0.31]
Nottingham	Neonatal [1.25]	Congenital [0.86]	Dermatitis [1.49]	Headaches [0.9]	Asthma [1.53]	Anxiety [0.96]	Back Pain [0.91]	Conduct [0.96]	Depression [1.07]	Falls [0.81]
Nottinghamshire	Neonatal [0.73]	Congenital [0.55]	Dermatitis [1.5]	Headaches [0.87]	Asthma [1.43]	Anxiety [1.01]	Conduct [0.95]	Back Pain [0.9]	Depression [1.05]	Falls [0.85]
Rutland	Neonatal [0.37]	Congenital [0.41]	Dermatitis [1.45]	Headaches [0.99]	Anxiety [1.08]	Asthma [1.44]	Back Pain [1.02]	Conduct [1.04]	Depression [1.2]	Road Inj [0.36]
E England	Neonatal [0.71]	Congenital [0.55]	Dermatitis [1.5]	Headaches [0.87]	Asthma [1.45]	Anxiety [0.98]	Conduct [0.96]	Back Pain [0.87]	Depression [1.03]	Falls [0.82]
Bedford	Neonatal [0.9]	Congenital [0.65]	Dermatitis [1.5]	Headaches [0.88]	Asthma [1.45]	Anxiety [0.99]	Conduct [0.96]	Back Pain [0.9]	Depression [1.05]	Falls [0.83]
Cambridgeshire	Neonatal [0.81]	Congenital [0.61]	Dermatitis [1.5]	Headaches [0.88]	Asthma [1.49]	Anxiety [0.96]	Conduct [0.98]	Depression [1.05]	Back Pain [0.82]	Falls [0.8]
Cen Bedfordshire	Neonatal [0.61]	Congenital [0.49]	Dermatitis [1.51]	Headaches [0.85]	Asthma [1.44]	Anxiety [0.98]	Conduct [0.95]	Back Pain [0.86]	Depression [1.02]	Falls [0.83]
Essex	Neonatal [0.66]	Congenital [0.5]	Dermatitis [1.5]	Headaches [0.88]	Asthma [1.43]	Anxiety [1.01]	Conduct [0.97]	Back Pain [0.89]	Depression [1.06]	Falls [0.83]
Hertfordshire	Neonatal [0.73]	Congenital [0.55]	Dermatitis [1.51]	Headaches [0.86]	Asthma [1.48]	Anxiety [0.95]	Conduct [0.97]	Back Pain [0.85]	Depression [1.01]	Falls [0.77]
Luton	Neonatal [1.11]	Congenital [0.86]	Dermatitis [1.52]	Asthma [1.48]	Headaches [0.82]	Anxiety [0.95]	Conduct [0.93]	Back Pain [0.83]	Depression [0.97]	Falls [0.84]
Norfolk	Neonatal [0.66]	Congenital [0.55]	Dermatitis [1.5]	Headaches [0.88]	Asthma [1.43]	Anxiety [1.01]	Conduct [0.96]	Back Pain [0.9]	Depression [1.06]	Drugs [2.09]
Peterborough	Neonatal [0.71]	Congenital [0.59]	Dermatitis [1.53]	Asthma [1.48]	Headaches [0.8]	Anxiety [0.95]	Conduct [0.91]	Back Pain [0.82]	Depression [0.95]	Road Inj [0.32]
Southend-on-Sea	Neonatal [0.63]	Congenital [0.51]	Dermatitis [1.52]	Headaches [0.84]	Asthma [1.42]	Anxiety [0.99]	Conduct [0.93]	Back Pain [0.85]	Depression [1.01]	Iron [2.4]
Suffolk	Neonatal [0.6]	Congenital [0.47]	Dermatitis [1.5]	Headaches [0.88]	Asthma [1.41]	Anxiety [1.01]	Conduct [0.96]	Back Pain [0.89]	Depression [1.06]	Iron [2.57]
Thurrock	Neonatal [0.59]	Congenital [0.46]	Dermatitis [1.53]	Asthma [1.45]	Headaches [0.82]	Anxiety [0.99]	Conduct [0.92]	Back Pain [0.83]	Depression [0.99]	Falls [0.84]
Greater London	Neonatal [1.14]	Congenital [0.8]	Dermatitis [1.54]	Asthma [1.77]	Headaches [0.81]	Anxiety [0.88]	Conduct [0.93]	Back Pain [0.79]	Depression [0.94]	Falls [0.73]
Barking & Dagenham	Neonatal [0.72]	Congenital [0.55]	Dermatitis [2.0]	Asthma [1.61]	Headaches [0.75]	Anxiety [0.93]	Iron [2.93]	Conduct [0.87]	Back Pain [0.78]	Depression [0.89]
Barnet	Neonatal [0.71]	Congenital [0.55]	Dermatitis [1.52]	Asthma [1.7]	Headaches [0.8]	Anxiety [0.91]	Conduct [0.93]	Back Pain [0.8]	Depression [0.95]	Falls [0.76]
Bexley	Neonatal [0.6]	Congenital [0.48]	Dermatitis [1.5]	Asthma [1.67]	Headaches [0.86]	Anxiety [0.99]	Conduct [0.94]	Back Pain [0.89]	Depression [1.04]	Falls [0.83]
Brent	Neonatal [0.95]	Congenital [0.69]	Dermatitis [1.5]	Asthma [1.7]	Headaches [0.81]	Anxiety [0.93]	Conduct [0.94]	Back Pain [0.83]	Depression [0.97]	Falls [0.79]
Bromley	Neonatal [0.7]	Congenital [0.52]	Dermatitis [1.51]	Asthma [1.68]	Headaches [0.84]	Anxiety [0.95]	Conduct [0.94]	Back Pain [0.85]	Depression [1.0]	Falls [0.79]
Camden	Neonatal [1.13]	Congenital [0.79]	Dermatitis [1.52]	Asthma [1.85]	Headaches [0.88]	Anxiety [0.89]	Conduct [0.96]	Back Pain [0.84]	Depression [1.02]	Drugs [2.06]
Croydon	Neonatal [0.81]	Congenital [0.6]	Dermatitis [1.53]	Asthma [1.67]	Headaches [0.8]	Anxiety [0.93]	Conduct [0.89]	Back Pain [0.81]	Depression [0.94]	Iron [2.79]
Ealing	Neonatal [0.85]	Congenital [0.6]	Dermatitis [1.53]	Asthma [1.71]	Headaches [0.78]	Anxiety [0.89]	Conduct [0.91]	Back Pain [0.78]	Depression [0.92]	Falls [0.75]
Enfield	Neonatal [0.83]	Congenital [0.57]	Dermatitis [1.52]	Asthma [1.67]	Headaches [0.82]	Anxiety [0.95]	Conduct [0.93]	Back Pain [0.83]	Depression [0.97]	Falls [0.79]
Greenwich	Neonatal [1.01]	Congenital [0.69]	Dermatitis [1.53]	Asthma [1.64]	Headaches [0.79]	Anxiety [0.92]	Conduct [0.88]	Back Pain [0.81]	Depression [0.94]	Falls [0.77]
Hackney	Neonatal [1.55]	Congenital [1.0]	Dermatitis [1.52]	Asthma [1.82]	Headaches [0.79]	Anxiety [0.87]	Conduct [0.9]	Back Pain [0.78]	Depression [0.92]	SIDS [1.44]
Hammersmith & Fulham	Neonatal [1.29]	Congenital [0.89]	Dermatitis [1.55]	Asthma [1.8]	Headaches [0.78]	Anxiety [0.83]	Conduct [0.9]	Back Pain [0.74]	Depression [0.9]	Falls [0.71]
Haringey	Neonatal [0.97]	Congenital [0.66]	Dermatitis [1.5]	Asthma [1.71]	Headaches [0.84]	Anxiety [0.95]	Conduct [0.95]	Back Pain [0.85]	Depression [0.99]	Falls [0.79]
Harrow	Neonatal [1.04]	Congenital [0.69]	Dermatitis [1.51]	Asthma [1.68]	Headaches [0.83]	Anxiety [0.95]	Conduct [0.94]	Back Pain [0.84]	Depression [0.99]	Falls [0.79]

eFigure 7a. Leading ten causes of DALYs with the ratio of observed DALYs to DALYs expected on the basis of Socio-Demographic Index alone in 2017, <20 years, both sexes combined. The top ten causes contributing to DALYs are listed globally, by socio-demographic quintile, and then by GBD superregion, region, country, and subnationally where modeled. For each cell, the ratio of observed DALYs to DALYs expected on the basis of socio-demographic index (SDI) alone are listed. Abbreviations: DALY=disability-adjusted life year, GBD=Global Burden of Disease.

Values shown in brackets represent the ratio of observed DALYs to predicted DALYs on the basis of Socio-Demographic Index (SDI), rounded to two (2) digits. Color ranges (shown below) were calculated to place a roughly equal number of cells into each bin.

COLOR KEY:	[0.0-0.5]	(0.5-0.69]	(0.69-0.82]	(0.82-0.92]	(0.92-1.01]	(1.01-1.18]	(1.18-1.48]	(1.48-2.26]	2.26+

	1	2	3	4	5	6	7	8	9	10
Havering	Neonatal [0.61]	Congenital [0.48]	Dermatitis [1.5]	Asthma [1.66]	Headaches [0.85]	Anxiety [0.98]	Conduct [0.93]	Back Pain [0.88]	Depression [1.03]	Falls [0.83]
Hillingdon	Neonatal [0.92]	Congenital [0.74]	Dermatitis [1.51]	Asthma [1.8]	Headaches [0.83]	Anxiety [0.91]	Conduct [0.94]	Back Pain [0.83]	Depression [0.98]	Falls [0.76]
Hounslow	Neonatal [1.14]	Congenital [0.8]	Dermatitis [1.54]	Asthma [1.74]	Headaches [0.77]	Anxiety [0.86]	Conduct [0.89]	Back Pain [0.75]	Depression [0.9]	Falls [0.74]
Islington	Neonatal [1.18]	Congenital [0.9]	Dermatitis [1.53]	Asthma [1.85]	Headaches [0.82]	Anxiety [0.85]	Conduct [0.91]	Back Pain [0.79]	Depression [0.95]	Drugs [2.03]
Kensington & Chelsea	Neonatal [0.98]	Congenital [0.71]	Dermatitis [1.53]	Asthma [1.82]	Headaches [0.83]	Anxiety [0.85]	Conduct [0.94]	Back Pain [0.79]	Depression [0.96]	Drugs [1.94]
Kingston upon Thames	Neonatal [1.01]	Congenital [0.75]	Dermatitis [1.53]	Asthma [1.76]	Headaches [0.84]	Anxiety [0.9]	Conduct [0.92]	Back Pain [0.82]	Depression [0.98]	Falls [0.73]
Lambeth	Neonatal [1.44]	Congenital [1.01]	Dermatitis [1.53]	Asthma [1.77]	Headaches [0.8]	Anxiety [0.86]	Conduct [0.91]	Back Pain [0.77]	Depression [0.92]	SIDS [1.35]
Lewisham	Neonatal [1.12]	Congenital [0.68]	Dermatitis [1.53]	Asthma [1.66]	Headaches [0.76]	Anxiety [0.89]	Conduct [0.87]	Back Pain [0.77]	Depression [0.9]	Falls [0.76]
Merton	Neonatal [1.01]	Congenital [0.76]	Dermatitis [1.54]	Asthma [1.71]	Headaches [0.75]	Anxiety [0.85]	Conduct [0.88]	Back Pain [0.74]	Depression [0.88]	Falls [0.73]
Newham	Neonatal [0.97]	Congenital [0.72]	Dermatitis [1.51]	Asthma [1.68]	Headaches [0.8]	Anxiety [0.92]	Conduct [0.89]	Back Pain [0.82]	Depression [0.95]	Falls [0.78]
Redbridge	Neonatal [0.64]	Congenital [0.51]	Dermatitis [1.52]	Asthma [1.65]	Headaches [0.8]	Anxiety [0.94]	Conduct [0.91]	Back Pain [0.82]	Depression [0.95]	Falls [0.8]
Richmond upon Thames	Neonatal [0.85]	Congenital [0.68]	Dermatitis [1.54]	Asthma [1.76]	Headaches [0.79]	Anxiety [0.87]	Conduct [0.92]	Back Pain [0.76]	Depression [0.91]	Falls [0.72]
Southwark	Neonatal [1.49]	Congenital [0.98]	Dermatitis [1.54]	Asthma [1.79]	Headaches [0.78]	Anxiety [0.84]	Conduct [0.9]	Back Pain [0.75]	Depression [0.9]	Falls [0.72]
Sutton	Neonatal [0.78]	Congenital [0.64]	Dermatitis [1.52]	Asthma [1.69]	Headaches [0.82]	Anxiety [0.94]	Conduct [0.92]	Back Pain [0.83]	Depression [0.98]	Falls [0.79]
Tower Hamlets	Neonatal [1.63]	Congenital [1.11]	Dermatitis [1.52]	Asthma [1.81]	Headaches [0.79]	Anxiety [0.85]	Conduct [0.91]	Back Pain [0.77]	Depression [0.92]	Falls [0.73]
Waltham Forest	Neonatal [0.77]	Congenital [0.57]	Dermatitis [1.53]	Asthma [1.61]	Headaches [0.77]	Anxiety [0.92]	Conduct [0.87]	Back Pain [0.79]	Depression [0.92]	Falls [0.81]
Wandsworth	Neonatal [1.26]	Congenital [0.86]	Dermatitis [1.55]	Asthma [1.73]	Headaches [0.74]	Anxiety [0.8]	Conduct [0.86]	Back Pain [0.7]	Depression [0.85]	URI [1.29]
Westminster	Neonatal [1.1]	Congenital [0.77]	Dermatitis [1.52]	Asthma [1.87]	Headaches [0.85]	Anxiety [0.88]	Conduct [0.96]	Back Pain [0.82]	Depression [0.99]	Drugs [1.9]
NE England	Neonatal [0.66]	Congenital [0.57]	Dermatitis [1.61]	Headaches [0.88]	Asthma [1.45]	Anxiety [1.01]	Conduct [0.95]	Back Pain [0.89]	Depression [1.05]	Iron [2.8]
County Durham	Neonatal [0.69]	Congenital [0.56]	Dermatitis [1.78]	Headaches [0.88]	Asthma [1.47]	Anxiety [1.02]	Conduct [0.94]	Depression [1.06]	Iron [3.0]	Back Pain [0.84]
Darlington	Neonatal [0.69]	Congenital [0.58]	Dermatitis [1.5]	Headaches [0.87]	Asthma [1.48]	Anxiety [1.0]	Conduct [0.96]	Back Pain [0.9]	Depression [1.04]	Iron [2.94]
Gateshead	Neonatal [0.82]	Congenital [0.63]	Dermatitis [1.77]	Headaches [0.85]	Asthma [1.47]	Anxiety [0.98]	Conduct [0.93]	Back Pain [0.88]	Depression [1.02]	Iron [3.11]
Hartlepool	Neonatal [0.65]	Congenital [0.51]	Dermatitis [1.5]	Headaches [0.88]	Asthma [1.47]	Anxiety [1.04]	Conduct [0.94]	Back Pain [0.91]	Depression [1.07]	Drugs [2.39]
Middlesbrough	Neonatal [0.7]	Congenital [0.63]	Dermatitis [1.51]	Headaches [0.85]	Asthma [1.47]	Anxiety [0.99]	Conduct [0.92]	Back Pain [0.87]	Depression [1.02]	Iron [2.35]
Newcastle upon Tyne	Neonatal [0.82]	Congenital [0.73]	Dermatitis [1.48]	Headaches [0.91]	Asthma [1.54]	Anxiety [0.96]	Back Pain [0.92]	Conduct [0.96]	Depression [1.09]	Drugs [2.17]
N Tyneside	Neonatal [0.56]	Congenital [0.52]	Dermatitis [1.5]	Headaches [0.85]	Asthma [1.45]	Anxiety [0.98]	Conduct [0.94]	Back Pain [0.86]	Depression [1.01]	Falls [0.91]
Northumberland	Neonatal [0.53]	Congenital [0.51]	Dermatitis [1.48]	Headaches [0.91]	Asthma [1.45]	Anxiety [1.05]	Conduct [0.99]	Back Pain [0.94]	Depression [1.1]	Iron [2.58]
Redcar & Cleveland	Neonatal [0.51]	Congenital [0.46]	Dermatitis [1.5]	Headaches [0.87]	Asthma [1.43]	Anxiety [1.04]	Conduct [0.95]	Back Pain [0.91]	Depression [1.06]	Iron [2.23]
S Tyneside	Neonatal [0.49]	Congenital [0.47]	Dermatitis [1.79]	Headaches [0.87]	Asthma [1.42]	Anxiety [1.02]	Conduct [0.92]	Back Pain [0.9]	Depression [1.05]	Iron [2.57]
Stockton-on-Tees	Neonatal [0.77]	Congenital [0.59]	Dermatitis [1.51]	Headaches [0.85]	Asthma [1.48]	Anxiety [0.98]	Conduct [0.94]	Back Pain [0.87]	Depression [1.02]	Iron [2.8]
Sunderland	Neonatal [0.6]	Congenital [0.54]	Dermatitis [1.77]	Headaches [0.89]	Anxiety [1.02]	Asthma [1.3]	Conduct [0.96]	Back Pain [0.91]	Depression [1.07]	Iron [2.71]

eFigure 7a. Leading ten causes of DALYs with the ratio of observed DALYs to DALYs expected on the basis of Socio-Demographic Index alone in 2017, <20 years, both sexes combined. The top ten causes contributing to DALYs are listed globally, by socio-demographic quintile, and then by GBD superregion, region, country, and subnationally where modeled. For each cell, the ratio of observed DALYs to DALYs expected on the basis of socio-demographic index (SDI) alone are listed. Abbreviations: DALY=disability-adjusted life year, GBD=Global Burden of Disease.

Values shown in brackets represent the ratio of observed DALYs to predicted DALYs on the basis of Socio-Demographic Index (SDI), rounded to two (2) digits. Color ranges (shown below) were calculated to place a roughly equal number of cells into each bin.

COLOR KEY:	[0.0-0.5]	(0.5-0.69]	(0.69-0.82]	(0.82-0.92]	(0.92-1.01]	(1.01-1.18]	(1.18-1.48]	(1.48-2.26]	2.26+

	1	2	3	4	5	6	7	8	9	10
NW England	Neonatal [0.91]	Congenital [0.71]	Dermatitis [1.5]	Headaches [0.87]	Asthma [1.5]	Anxiety [0.98]	Conduct [0.95]	Back Pain [0.87]	Depression [1.05]	Falls [0.87]
Blackburn with Darwen	Neonatal [0.81]	Congenital [0.64]	Dermatitis [1.51]	Asthma [1.49]	Headaches [0.85]	Anxiety [1.01]	Conduct [0.94]	Back Pain [0.89]	Depression [1.02]	Iron [2.44]
Blackpool	Neonatal [0.73]	Congenital [0.59]	Dermatitis [1.52]	Headaches [0.87]	Asthma [1.47]	Anxiety [1.05]	Conduct [0.93]	Back Pain [0.91]	Depression [1.06]	Drugs [2.94]
Bolton	Neonatal [0.73]	Congenital [0.59]	Dermatitis [1.5]	Headaches [0.85]	Asthma [1.45]	Anxiety [1.0]	Conduct [0.94]	Back Pain [0.9]	Depression [1.03]	Iron [2.34]
Bury	Neonatal [0.77]	Congenital [0.64]	Dermatitis [1.51]	Headaches [0.84]	Asthma [1.43]	Anxiety [0.99]	Conduct [0.94]	Back Pain [0.88]	Depression [1.0]	Iron [2.59]
Cheshire E	Neonatal [0.95]	Congenital [0.7]	Dermatitis [1.49]	Headaches [0.9]	Asthma [1.53]	Anxiety [0.99]	Conduct [1.0]	Back Pain [0.91]	Depression [1.07]	Falls [0.82]
Cheshire W & Chester	Neonatal [0.9]	Congenital [0.73]	Dermatitis [1.49]	Headaches [0.9]	Asthma [1.52]	Anxiety [0.99]	Conduct [0.98]	Back Pain [0.9]	Depression [1.07]	Road Inj [0.37]
Cumbria	Neonatal [0.73]	Congenital [0.58]	Dermatitis [1.49]	Headaches [0.9]	Asthma [1.49]	Anxiety [1.02]	Conduct [0.98]	Back Pain [0.92]	Depression [1.08]	Road Inj [0.36]
Halton	Neonatal [0.75]	Congenital [0.59]	Dermatitis [1.51]	Headaches [0.87]	Asthma [1.51]	Anxiety [1.01]	Conduct [0.96]	Back Pain [0.88]	Depression [1.04]	Falls [0.88]
Knowsley	Neonatal [0.67]	Congenital [0.62]	Dermatitis [1.5]	Asthma [1.53]	Headaches [0.87]	Anxiety [1.0]	Conduct [0.94]	Back Pain [0.89]	Depression [1.04]	Road Inj [0.34]
Lancashire	Neonatal [0.88]	Congenital [0.71]	Dermatitis [1.49]	Headaches [0.89]	Asthma [1.5]	Anxiety [1.0]	Conduct [0.96]	Depression [1.06]	Back Pain [0.84]	Falls [0.9]
Liverpool	Neonatal [1.27]	Congenital [0.92]	Dermatitis [1.49]	Headaches [0.9]	Asthma [1.51]	Anxiety [0.9]	Depression [1.24]	Conduct [0.94]	Back Pain [0.9]	Drugs [2.41]
Manchester	Neonatal [1.6]	Congenital [1.1]	Dermatitis [1.51]	Asthma [1.57]	Headaches [0.84]	Anxiety [0.91]	Back Pain [0.87]	Conduct [0.93]	Depression [0.99]	Falls [0.81]
Oldham	Neonatal [0.84]	Congenital [0.71]	Dermatitis [1.51]	Asthma [1.53]	Headaches [0.86]	Anxiety [1.03]	Conduct [0.94]	Back Pain [0.91]	Depression [1.04]	Falls [0.92]
Rochdale	Neonatal [0.67]	Congenital [0.59]	Dermatitis [1.51]	Headaches [0.85]	Asthma [1.47]	Anxiety [1.02]	Conduct [0.93]	Back Pain [0.9]	Depression [1.03]	Iron [2.35]
Salford	Neonatal [1.05]	Congenital [0.86]	Dermatitis [1.53]	Asthma [1.52]	Headaches [0.8]	Anxiety [0.93]	Conduct [0.9]	Back Pain [0.82]	Depression [0.95]	Falls [0.86]
Sefton	Neonatal [0.77]	Congenital [0.61]	Dermatitis [1.5]	Headaches [0.87]	Asthma [1.45]	Anxiety [1.01]	Conduct [0.95]	Back Pain [0.89]	Depression [1.05]	Drugs [2.37]
St Helens	Neonatal [0.65]	Congenital [0.55]	Dermatitis [1.51]	Headaches [0.86]	Asthma [1.45]	Anxiety [1.01]	Conduct [0.94]	Back Pain [0.89]	Depression [1.03]	Drugs [2.29]
Stockport	Neonatal [0.94]	Congenital [0.76]	Dermatitis [1.51]	Headaches [0.85]	Asthma [1.47]	Anxiety [0.97]	Conduct [0.95]	Back Pain [0.87]	Depression [1.01]	Falls [0.86]
Tameside	Neonatal [0.62]	Congenital [0.55]	Dermatitis [1.52]	Asthma [1.45]	Headaches [0.83]	Anxiety [1.0]	Conduct [0.91]	Depression [1.0]	Back Pain [0.7]	Falls [0.89]
Trafford	Neonatal [0.91]	Congenital [0.64]	Dermatitis [1.51]	Headaches [0.87]	Asthma [1.56]	Anxiety [0.96]	Conduct [1.0]	Back Pain [0.87]	Depression [1.02]	Falls [0.82]
Warrington	Neonatal [1.04]	Congenital [0.73]	Dermatitis [1.5]	Headaches [0.87]	Asthma [1.54]	Anxiety [0.97]	Conduct [0.98]	Back Pain [0.88]	Depression [1.04]	Drugs [2.04]
Wigan	Neonatal [0.65]	Congenital [0.52]	Dermatitis [1.51]	Headaches [0.86]	Asthma [1.45]	Anxiety [1.02]	Conduct [0.95]	Back Pain [0.9]	Depression [1.03]	Iron [2.18]
Wirral	Neonatal [0.79]	Congenital [0.65]	Dermatitis [1.5]	Headaches [0.88]	Asthma [1.45]	Anxiety [1.03]	Conduct [0.96]	Back Pain [0.9]	Depression [1.06]	Drugs [2.32]
SE England	Neonatal [0.76]	Congenital [0.57]	Dermatitis [1.64]	Headaches [0.88]	Asthma [1.58]	Anxiety [0.98]	Conduct [0.97]	Back Pain [0.86]	Depression [1.04]	Falls [0.81]
Bracknell Forest	Neonatal [0.56]	Congenital [0.53]	Dermatitis [1.52]	Headaches [0.84]	Asthma [1.46]	Anxiety [0.93]	Conduct [0.95]	Back Pain [0.83]	Depression [0.99]	Falls [0.78]
Brighton & Hove	Neonatal [1.12]	Congenital [0.82]	Dermatitis [1.74]	Headaches [0.95]	Anxiety [0.98]	Asthma [1.53]	Conduct [1.01]	Back Pain [0.92]	Depression [1.12]	Drugs [2.44]
Buckinghamshire	Neonatal [0.93]	Congenital [0.66]	Dermatitis [1.5]	Headaches [0.89]	Anxiety [0.98]	Asthma [1.46]	Conduct [0.99]	Back Pain [0.88]	Depression [1.06]	Epilepsy [1.69]
E Sussex	Neonatal [0.61]	Congenital [0.51]	Dermatitis [1.73]	Headaches [0.91]	Anxiety [1.04]	Asthma [1.36]	Conduct [0.98]	Back Pain [0.93]	Depression [1.1]	Drugs [2.15]
Hampshire	Neonatal [0.7]	Congenital [0.51]	Asthma [2.18]	Dermatitis [1.49]	Headaches [0.89]	Anxiety [0.99]	Conduct [0.98]	Back Pain [0.88]	Depression [1.05]	Falls [0.81]
Isle of Wight	Neonatal [0.41]	Congenital [0.38]	Asthma [2.13]	Dermatitis [1.44]	Headaches [0.93]	Anxiety [1.05]	Conduct [0.99]	Back Pain [0.95]	Depression [1.13]	Drugs [2.15]

eFigure 7a. Leading ten causes of DALYs with the ratio of observed DALYs to DALYs expected on the basis of Socio-Demographic Index alone in 2017, <20 years, both sexes combined. The top ten causes contributing to DALYs are listed globally, by socio-demographic quintile, and then by GBD superregion, region, country, and subnationally where modeled. For each cell, the ratio of observed DALYs to DALYs expected on the basis of socio-demographic index (SDI) alone are listed. Abbreviations: DALY=disability-adjusted life year, GBD=Global Burden of Disease.

Values shown in brackets represent the ratio of observed DALYs to predicted DALYs on the basis of Socio-Demographic Index (SDI), rounded to two (2) digits. Color ranges (shown below) were calculated to place a roughly equal number of cells into each bin.

COLOR KEY:	[0.0-0.5]	(0.5-0.69]	(0.69-0.82]	(0.82-0.92]	(0.92-1.01]	(1.01-1.18]	(1.18-1.48]	(1.48-2.26]	2.26+

	1	2	3	4	5	6	7	8	9	10
Kent	Neonatal [0.65]	Congenital [0.49]	Dermatitis [1.73]	Headaches [0.89]	Anxiety [1.01]	Asthma [1.4]	Conduct [0.97]	Back Pain [0.9]	Depression [1.07]	Falls [0.84]
Medway	Neonatal [0.68]	Congenital [0.53]	Dermatitis [1.5]	Headaches [0.87]	Asthma [1.4]	Anxiety [1.01]	Conduct [0.94]	Back Pain [0.88]	Depression [1.05]	Falls [0.85]
Milton Keynes	Neonatal [0.99]	Congenital [0.7]	Dermatitis [1.54]	Asthma [1.49]	Headaches [0.8]	Anxiety [0.92]	Conduct [0.93]	Back Pain [0.79]	Depression [0.94]	Falls [0.8]
Oxfordshire	Neonatal [0.82]	Dermatitis [1.93]	Congenital [0.63]	Headaches [0.89]	Asthma [1.48]	Anxiety [0.96]	Conduct [0.98]	Back Pain [0.87]	Depression [1.05]	Falls [0.77]
Portsmouth	Neonatal [0.71]	Congenital [0.53]	Dermatitis [1.5]	Headaches [0.87]	Asthma [1.45]	Anxiety [0.96]	Conduct [0.95]	Back Pain [0.86]	Depression [1.04]	Drugs [2.06]
Reading	Neonatal [1.27]	Congenital [0.88]	Dermatitis [1.99]	Asthma [1.51]	Headaches [0.78]	Anxiety [0.86]	Conduct [0.9]	Back Pain [0.76]	Depression [0.91]	Falls [0.74]
Slough	Neonatal [1.04]	Congenital [0.81]	Dermatitis [1.54]	Asthma [1.55]	Headaches [0.77]	Anxiety [0.9]	Conduct [0.92]	Back Pain [0.76]	Depression [0.9]	Falls [0.79]
Southampton	Neonatal [0.92]	Congenital [0.71]	Dermatitis [1.5]	Headaches [0.85]	Asthma [1.45]	Anxiety [0.93]	Conduct [0.92]	Back Pain [0.84]	Depression [1.01]	Falls [0.8]
Surrey	Neonatal [0.8]	Congenital [0.59]	Dermatitis [1.74]	Headaches [0.88]	Anxiety [0.95]	Asthma [1.48]	Conduct [0.99]	Depression [1.03]	Back Pain [0.79]	Falls [0.76]
West Berkshire	Neonatal [0.7]	Congenital [0.59]	Dermatitis [1.5]	Headaches [0.89]	Asthma [1.5]	Anxiety [0.98]	Conduct [1.0]	Back Pain [0.88]	Depression [1.06]	Falls [0.8]
W Sussex	Neonatal [0.69]	Congenital [0.54]	Dermatitis [1.55]	Asthma [1.63]	Headaches [0.86]	Anxiety [0.97]	Conduct [0.97]	Back Pain [0.86]	Depression [1.03]	Falls [0.8]
Windsor & Maidenhead	Neonatal [0.85]	Congenital [0.7]	Dermatitis [1.5]	Headaches [0.89]	Asthma [1.51]	Anxiety [0.96]	Conduct [1.02]	Back Pain [0.87]	Depression [1.04]	Falls [0.77]
Wokingham	Neonatal [0.76]	Congenital [0.64]	Dermatitis [1.51]	Headaches [0.88]	Asthma [1.5]	Anxiety [0.96]	Conduct [1.01]	Back Pain [0.86]	Depression [1.03]	Falls [0.79]
SW England	Neonatal [0.72]	Congenital [0.55]	Dermatitis [1.49]	Headaches [0.89]	Asthma [1.44]	Anxiety [1.0]	Conduct [0.97]	Back Pain [0.89]	Depression [1.06]	Falls [0.83]
Bath & NE Somerset	Neonatal [0.77]	Congenital [0.53]	Dermatitis [1.46]	Headaches [0.96]	Anxiety [1.01]	Asthma [1.48]	Back Pain [0.96]	Conduct [1.01]	Depression [1.15]	Drugs [2.04]
Bournemouth	Neonatal [0.81]	Congenital [0.63]	Dermatitis [1.51]	Headaches [0.83]	Asthma [1.42]	Anxiety [0.92]	Conduct [0.9]	Back Pain [0.82]	Depression [0.99]	Iron [3.7]
Bristol	Neonatal [1.07]	Congenital [0.76]	Dermatitis [1.52]	Headaches [0.83]	Asthma [1.49]	Anxiety [0.9]	Conduct [0.92]	Back Pain [0.81]	Depression [0.98]	Falls [0.73]
Cornwall	Neonatal [0.73]	Congenital [0.53]	Dermatitis [1.49]	Headaches [0.9]	Anxiety [1.03]	Asthma [1.4]	Conduct [0.96]	Depression [1.09]	Iron [3.3]	Back Pain [0.86]
Devon	Neonatal [0.71]	Congenital [0.55]	Dermatitis [1.48]	Headaches [0.91]	Anxiety [1.02]	Asthma [1.43]	Conduct [0.99]	Back Pain [0.92]	Depression [1.1]	Falls [0.87]
Dorset	Neonatal [0.62]	Congenital [0.47]	Dermatitis [1.47]	Headaches [0.93]	Anxiety [1.06]	Asthma [1.44]	Conduct [1.01]	Back Pain [0.95]	Depression [1.12]	Road Inj [0.33]
Gloucestershire	Neonatal [0.71]	Congenital [0.55]	Dermatitis [1.49]	Headaches [0.9]	Anxiety [0.99]	Asthma [1.45]	Conduct [0.97]	Back Pain [0.9]	Depression [1.07]	Falls [0.83]
N Somerset	Neonatal [0.66]	Congenital [0.57]	Dermatitis [1.5]	Headaches [0.87]	Asthma [1.44]	Anxiety [1.0]	Conduct [0.96]	Back Pain [0.87]	Depression [1.05]	Drugs [2.15]
Plymouth	Neonatal [0.7]	Congenital [0.58]	Dermatitis [1.5]	Headaches [0.87]	Asthma [1.42]	Anxiety [0.98]	Conduct [0.94]	Back Pain [0.87]	Depression [1.04]	Drugs [2.16]
Poole	Neonatal [0.61]	Congenital [0.47]	Dermatitis [1.5]	Headaches [0.87]	Asthma [1.43]	Anxiety [0.99]	Conduct [0.96]	Back Pain [0.86]	Depression [1.03]	Falls [0.83]
Somerset	Neonatal [0.65]	Congenital [0.5]	Dermatitis [1.49]	Headaches [0.91]	Anxiety [1.04]	Asthma [1.4]	Conduct [0.97]	Back Pain [0.93]	Depression [1.1]	Falls [0.87]
S Gloucestershire	Neonatal [0.72]	Congenital [0.57]	Dermatitis [1.5]	Headaches [0.88]	Asthma [1.47]	Anxiety [0.97]	Conduct [0.97]	Back Pain [0.87]	Depression [1.04]	Falls [0.78]
Swindon	Neonatal [0.64]	Congenital [0.53]	Dermatitis [1.51]	Headaches [0.83]	Asthma [1.46]	Anxiety [0.95]	Conduct [0.94]	Back Pain [0.84]	Depression [0.99]	Iron [3.46]
Torbay	Neonatal [0.62]	Congenital [0.49]	Dermatitis [1.5]	Headaches [0.87]	Asthma [1.38]	Anxiety [1.04]	Conduct [0.95]	Back Pain [0.9]	Depression [1.06]	Iron [2.06]
Wiltshire	Neonatal [0.67]	Congenital [0.48]	Dermatitis [1.49]	Headaches [0.89]	Anxiety [1.02]	Asthma [1.42]	Conduct [0.97]	Back Pain [0.91]	Depression [1.07]	Iron [3.31]
W Midlands	Neonatal [1.07]	Congenital [0.82]	Dermatitis [1.5]	Headaches [0.87]	Asthma [1.47]	Anxiety [1.0]	Conduct [0.96]	Back Pain [0.88]	Depression [1.05]	Falls [0.89]
Birmingham	Neonatal [1.47]	Congenital [1.12]	Dermatitis [1.5]	Headaches [0.86]	Asthma [1.47]	Anxiety [0.98]	Conduct [0.95]	Back Pain [0.89]	Depression [1.03]	Falls [0.91]

eFigure 7a. Leading ten causes of DALYs with the ratio of observed DALYs to DALYs expected on the basis of Socio-Demographic Index alone in 2017, <20 years, both sexes combined. The top ten causes contributing to DALYs are listed globally, by socio-demographic quintile, and then by GBD superregion, region, country, and subnationally where modeled. For each cell, the ratio of observed DALYs to DALYs expected on the basis of socio-demographic index (SDI) alone are listed. Abbreviations: DALY=disability-adjusted life year, GBD=Global Burden of Disease.

Values shown in brackets represent the ratio of observed DALYs to predicted DALYs on the basis of Socio-Demographic Index (SDI), rounded to two (2) digits. Color ranges (shown below) were calculated to place a roughly equal number of cells into each bin.

COLOR KEY:	[0.0-0.5]	(0.5-0.69]	(0.69-0.82]	(0.82-0.92]	(0.92-1.01]	(1.01-1.18]	(1.18-1.48]	(1.48-2.26]	2.26+

	1	2	3	4	5	6	7	8	9	10
Coventry	Neonatal [1.14]	Congenital [0.87]	Dermatitis [1.51]	Headaches [0.85]	Asthma [1.5]	Anxiety [0.95]	Conduct [0.93]	Back Pain [0.86]	Depression [1.01]	Falls [0.81]
Dudley	Neonatal [0.81]	Congenital [0.67]	Dermatitis [1.51]	Headaches [0.87]	Asthma [1.45]	Anxiety [1.03]	Conduct [0.95]	Back Pain [0.91]	Depression [1.05]	Falls [0.92]
Herefordshire	Neonatal [0.75]	Congenital [0.65]	Dermatitis [1.49]	Headaches [0.9]	Asthma [1.46]	Anxiety [1.02]	Conduct [0.97]	Road Inj [0.43]	Back Pain [0.92]	Depression [1.07]
Sandwell	Neonatal [1.07]	Congenital [0.79]	Dermatitis [1.52]	Asthma [1.47]	Headaches [0.81]	Anxiety [0.99]	Conduct [0.92]	Back Pain [0.86]	Depression [0.98]	Falls [0.91]
Shropshire	Neonatal [0.64]	Congenital [0.53]	Dermatitis [1.48]	Headaches [0.94]	Anxiety [1.05]	Asthma [1.44]	Conduct [1.0]	Back Pain [0.97]	Depression [1.13]	Road Inj [0.31]
Solihull	Neonatal [0.83]	Congenital [0.74]	Dermatitis [1.49]	Headaches [0.89]	Asthma [1.5]	Anxiety [0.99]	Conduct [0.99]	Back Pain [0.9]	Depression [1.06]	Road Inj [0.35]
Staffordshire	Neonatal [0.92]	Congenital [0.69]	Dermatitis [1.49]	Headaches [0.9]	Asthma [1.46]	Anxiety [1.03]	Conduct [0.99]	Depression [1.09]	Back Pain [0.83]	Road Inj [0.34]
Stoke-on-Trent	Neonatal [1.23]	Congenital [0.92]	Dermatitis [1.52]	Headaches [0.84]	Asthma [1.45]	Anxiety [1.0]	Conduct [0.93]	Depression [1.01]	Back Pain [0.78]	Falls [1.0]
Telford & Wrekin	Neonatal [0.9]	Congenital [0.71]	Dermatitis [1.51]	Headaches [0.87]	Asthma [1.46]	Anxiety [1.01]	Conduct [0.94]	Back Pain [0.9]	Depression [1.04]	Road Inj [0.33]
Walsall	Neonatal [1.03]	Congenital [0.78]	Dermatitis [1.52]	Headaches [0.86]	Asthma [1.45]	Anxiety [1.03]	Conduct [0.94]	Back Pain [0.91]	Depression [1.04]	Iron [2.1]
Warwickshire	Neonatal [0.96]	Congenital [0.72]	Dermatitis [1.5]	Headaches [0.88]	Asthma [1.5]	Anxiety [0.98]	Conduct [0.98]	Back Pain [0.89]	Depression [1.05]	Falls [0.84]
Wolverhampton	Neonatal [0.93]	Congenital [0.74]	Dermatitis [1.52]	Headaches [0.85]	Asthma [1.43]	Anxiety [0.99]	Conduct [0.93]	Back Pain [0.88]	Depression [1.02]	Falls [0.86]
Worcestershire	Neonatal [0.88]	Congenital [0.68]	Dermatitis [1.49]	Headaches [0.9]	Asthma [1.45]	Anxiety [1.02]	Conduct [0.98]	Back Pain [0.92]	Depression [1.08]	Falls [0.88]
Yorkshire & Humber	Neonatal [0.76]	Congenital [0.63]	Dermatitis [1.5]	Headaches [0.87]	Asthma [1.48]	Anxiety [0.99]	Conduct [0.95]	Back Pain [0.89]	Depression [1.04]	Falls [0.84]
Barnsley	Neonatal [0.54]	Congenital [0.47]	Dermatitis [1.52]	Asthma [1.49]	Headaches [0.85]	Anxiety [1.03]	Conduct [0.92]	Back Pain [0.9]	Depression [1.03]	Iron [2.17]
Bradford	Neonatal [0.84]	Congenital [0.69]	Dermatitis [1.51]	Headaches [0.85]	Asthma [1.44]	Anxiety [1.01]	Conduct [0.94]	Back Pain [0.89]	Depression [1.02]	Iron [2.21]
Calderdale	Neonatal [0.72]	Congenital [0.61]	Dermatitis [1.51]	Headaches [0.87]	Asthma [1.48]	Anxiety [1.0]	Conduct [0.95]	Back Pain [0.9]	Depression [1.04]	Road Inj [0.35]
Doncaster	Neonatal [0.73]	Congenital [0.58]	Dermatitis [1.52]	Headaches [0.85]	Asthma [1.45]	Anxiety [1.03]	Conduct [0.93]	Back Pain [0.9]	Depression [1.03]	Road Inj [0.29]
E Riding of Yorkshire	Neonatal [0.52]	Congenital [0.48]	Dermatitis [1.48]	Headaches [0.93]	Asthma [1.49]	Anxiety [1.05]	Conduct [0.99]	Back Pain [0.96]	Depression [1.12]	Road Inj [0.37]
Kingston upon Hull	Neonatal [0.69]	Congenital [0.57]	Dermatitis [1.53]	Asthma [1.5]	Headaches [0.8]	Anxiety [0.95]	Conduct [0.9]	Back Pain [0.82]	Depression [0.96]	Drugs [2.17]
Kirklees	Neonatal [0.76]	Congenital [0.65]	Dermatitis [1.51]	Headaches [0.87]	Asthma [1.45]	Anxiety [1.01]	Conduct [0.94]	Back Pain [0.9]	Depression [1.04]	Iron [2.46]
Leeds	Neonatal [0.97]	Congenital [0.77]	Dermatitis [1.52]	Headaches [0.84]	Asthma [1.51]	Anxiety [0.92]	Conduct [0.93]	Back Pain [0.84]	Depression [0.99]	Falls [0.78]
NE Lincolnshire	Neonatal [0.61]	Congenital [0.53]	Dermatitis [1.52]	Asthma [1.49]	Headaches [0.84]	Anxiety [1.0]	Conduct [0.93]	Back Pain [0.86]	Depression [1.01]	Road Inj [0.31]
N Lincolnshire	Neonatal [0.59]	Congenital [0.5]	Dermatitis [1.51]	Headaches [0.86]	Asthma [1.5]	Anxiety [1.01]	Conduct [0.95]	Back Pain [0.89]	Road Inj [0.4]	Depression [1.03]
N Yorkshire	Neonatal [0.61]	Congenital [0.5]	Dermatitis [1.48]	Headaches [0.9]	Asthma [1.46]	Anxiety [1.02]	Conduct [1.0]	Back Pain [0.93]	Depression [1.08]	Falls [0.84]
Rotherham	Neonatal [0.62]	Congenital [0.55]	Dermatitis [1.51]	Headaches [0.87]	Asthma [1.47]	Anxiety [1.03]	Conduct [0.94]	Back Pain [0.91]	Depression [1.05]	Iron [2.09]
Sheffield	Neonatal [0.9]	Congenital [0.75]	Dermatitis [1.49]	Headaches [0.9]	Asthma [1.49]	Anxiety [0.98]	Conduct [0.96]	Back Pain [0.91]	Depression [1.08]	Drugs [2.03]
Wakefield	Neonatal [0.62]	Congenital [0.51]	Dermatitis [1.51]	Asthma [1.5]	Headaches [0.85]	Anxiety [1.01]	Conduct [0.93]	Back Pain [0.89]	Depression [1.02]	Road Inj [0.33]
York	Neonatal [0.79]	Congenital [0.67]	Dermatitis [1.48]	Headaches [0.93]	Asthma [1.55]	Anxiety [0.98]	Back Pain [0.92]	Conduct [0.98]	Depression [1.11]	Road Inj [0.38]
N Ireland	Neonatal [0.88]	Congenital [0.77]	Anxiety [1.61]	Dermatitis [1.25]	Headaches [0.95]	Asthma [1.32]	Depression [1.17]	Back Pain [0.93]	Conduct [0.81]	Road Inj [0.38]
Scotland	Neonatal [0.55]	Congenital [0.53]	Dermatitis [1.35]	Headaches [0.96]	Anxiety [1.12]	Asthma [1.47]	Depression [1.15]	Conduct [0.91]	Back Pain [0.91]	Iron [2.7]

eFigure 7a. Leading ten causes of DALYs with the ratio of observed DALYs to DALYs expected on the basis of Socio-Demographic Index alone in 2017, <20 years, both sexes combined. The top ten causes contributing to DALYs are listed globally, by socio-demographic quintile, and then by GBD superregion, region, country, and subnationally where modeled. For each cell, the ratio of observed DALYs to DALYs expected on the basis of socio-demographic index (SDI) alone are listed. Abbreviations: DALY=disability-adjusted life year, GBD=Global Burden of Disease.

Values shown in brackets represent the ratio of observed DALYs to predicted DALYs on the basis of Socio-Demographic Index (SDI), rounded to two (2) digits. Color ranges (shown below) were calculated to place a roughly equal number of cells into each bin.

COLOR KEY:	[0.0-0.5]	(0.5-0.69]	(0.69-0.82]	(0.82-0.92]	(0.92-1.01]	(1.01-1.18]	(1.18-1.48]	(1.48-2.26]	2.26+

	1	2	3	4	5	6	7	8	9	10
Wales	Neonatal [0.56]	Congenital [0.48]	Dermatitis [1.25]	Headaches [0.97]	Anxiety [1.11]	Asthma [1.3]	Depression [1.2]	Conduct [0.89]	Back Pain [0.77]	Falls [1.01]
**Latin America and Caribbean**	Neonatal [0.69]	Congenital [0.66]	LRI [0.6]	Violence [3.69]	Diarrhea [1.34]	Road Inj [0.69]	Headaches [1.19]	Asthma [1.19]	Back Pain [1.29]	F Body [0.86]
**Andean Latin America**	Neonatal [0.69]	Congenital [0.64]	LRI [0.82]	F Body [1.82]	Road Inj [0.69]	Diarrhea [0.94]	HIV [8.39]	Dermatitis [1.45]	Headaches [1.08]	Asthma [1.44]
Bolivia	Neonatal [0.83]	LRI [0.8]	Congenital [0.78]	Diarrhea [0.72]	Iron [1.71]	F Body [1.7]	Road Inj [0.71]	STI [4.0]	HIV [6.57]	Asthma [1.62]
Ecuador	Neonatal [0.61]	Congenital [0.6]	LRI [0.69]	Road Inj [1.0]	Headaches [1.38]	Diarrhea [0.79]	Dermatitis [1.27]	Asthma [1.3]	Self Harm [1.68]	Violence [1.31]
Peru	Neonatal [0.6]	Congenital [0.57]	LRI [0.78]	F Body [2.24]	HIV [12.19]	Diarrhea [0.96]	Iron [1.53]	Road Inj [0.51]	Dermatitis [1.53]	Asthma [1.41]
**Caribbean**	Neonatal [1.19]	Congenital [0.93]	LRI [1.34]	Diarrhea [4.59]	Road Inj [1.15]	Iron [1.94]	Asthma [2.0]	Meningitis [1.22]	Violence [2.2]	STI [6.91]
Antigua	Neonatal [0.68]	Congenital [0.47]	Iron [4.88]	LRI [0.96]	Headaches [1.2]	Asthma [1.81]	Drown [1.35]	Dermatitis [1.01]	Road Inj [0.42]	Anxiety [1.0]
Bahamas	Neonatal [0.88]	Congenital [0.53]	LRI [1.51]	Violence [4.5]	Headaches [1.21]	Asthma [1.93]	Road Inj [0.69]	Drown [1.68]	Endocrine [2.56]	Dermatitis [1.0]
Barbados	Neonatal [0.94]	Congenital [0.59]	Headaches [1.21]	LRI [0.93]	Asthma [1.71]	Violence [2.35]	Road Inj [0.48]	Dermatitis [0.98]	Anxiety [0.99]	Conduct [0.9]
Belize	Neonatal [0.64]	Congenital [0.44]	LRI [0.4]	Violence [2.48]	Road Inj [0.6]	Iron [1.49]	Asthma [1.72]	Headaches [1.26]	Diarrhea [0.52]	Drown [0.67]
Bermuda	Neonatal [0.62]	Asthma [2.25]	Congenital [0.38]	Headaches [1.15]	Dermatitis [1.0]	Anxiety [0.88]	Road Inj [0.41]	Conduct [0.88]	Depression [0.86]	Back Pain [0.71]
Cuba	Neonatal [0.28]	Congenital [0.32]	Headaches [1.18]	Asthma [1.81]	Dermatitis [1.05]	Iron [1.36]	Anxiety [1.01]	Road Inj [0.33]	LRI [0.25]	Conduct [0.86]
Dominica	Neonatal [1.59]	Congenital [1.02]	LRI [1.28]	Road Inj [0.91]	Drown [1.38]	Asthma [1.95]	Headaches [1.26]	Iron [2.18]	Violence [2.25]	Endocrine [2.64]
Dominican Rep	Neonatal [1.2]	Congenital [0.6]	LRI [0.4]	Road Inj [0.88]	Diarrhea [0.77]	STI [5.38]	Violence [2.05]	Headaches [1.22]	Iron [1.02]	Asthma [1.29]
Grenada	Neonatal [0.6]	Congenital [0.34]	LRI [0.43]	Headaches [1.21]	Asthma [1.61]	Drown [0.73]	Iron [1.12]	Dermatitis [1.09]	Road Inj [0.33]	Anxiety [1.08]
Guyana	Neonatal [0.95]	Congenital [0.42]	LRI [0.33]	Diarrhea [0.7]	Asthma [1.59]	Iron [1.18]	Road Inj [0.49]	Headaches [1.23]	Drown [0.65]	Violence [1.46]
Haiti	Neonatal [0.63]	Diarrhea [1.07]	LRI [0.63]	Congenital [1.02]	Road Inj [1.45]	Meningitis [0.59]	Whooping [1.3]	Iron [1.39]	Drown [0.77]	STI [1.34]
Jamaica	Neonatal [1.18]	Congenital [0.53]	Violence [3.11]	Headaches [1.25]	Asthma [1.83]	STI [8.93]	Iron [1.64]	LRI [0.29]	Dermatitis [1.04]	Road Inj [0.38]
Puerto Rico	Disaster [5993.81]	Neonatal [0.9]	Congenital [0.54]	Asthma [2.67]	Violence [5.36]	Headaches [1.25]	Dermatitis [0.96]	Road Inj [0.51]	Anxiety [0.94]	Conduct [0.94]
St Lucia	Neonatal [0.74]	Congenital [0.34]	STI [9.53]	Headaches [1.25]	Asthma [1.76]	Violence [1.96]	LRI [0.29]	Road Inj [0.44]	Dermatitis [1.07]	Anxiety [1.09]
St Vincent	Neonatal [0.67]	Congenital [0.41]	LRI [0.31]	Violence [2.01]	Asthma [1.66]	Headaches [1.24]	Drown [0.69]	Iron [1.13]	Diarrhea [0.48]	Road Inj [0.37]
Suriname	Neonatal [1.36]	Congenital [0.71]	LRI [0.61]	Diarrhea [1.58]	Road Inj [0.7]	Asthma [1.85]	Headaches [1.16]	Drown [0.74]	Self Harm [1.84]	Iron [1.14]
Trinidad Tobago	Neonatal [1.19]	Congenital [0.86]	LRI [0.69]	Violence [3.03]	Asthma [2.1]	Headaches [1.11]	Road Inj [0.58]	Dermatitis [1.07]	Anxiety [0.95]	Diarrhea [0.97]
Virgin Isl US	Neonatal [1.17]	Congenital [0.67]	Violence [5.56]	Asthma [2.04]	Headaches [1.12]	Dermatitis [1.0]	Anxiety [0.86]	Road Inj [0.44]	Disaster [435.0]	Conduct [0.87]
**Central Latin America**	Neonatal [0.59]	Congenital [0.58]	LRI [0.47]	Violence [3.76]	Diarrhea [1.04]	Road Inj [0.57]	Headaches [1.0]	Epilepsy [1.26]	Conduct [0.95]	F Body [0.73]
Colombia	Neonatal [0.6]	Congenital [0.52]	Violence [3.36]	LRI [0.36]	Headaches [1.08]	Road Inj [0.48]	Diarrhea [0.74]	Epilepsy [1.23]	Dermatitis [0.93]	Conduct [0.95]
Costa Rica	Neonatal [0.48]	Congenital [0.5]	Headaches [1.03]	Road Inj [0.43]	Asthma [1.24]	Diarrhea [0.79]	Conduct [0.94]	Dermatitis [0.87]	Violence [1.06]	Epilepsy [1.05]
El Salvador	Neonatal [0.4]	Violence [5.89]	Congenital [0.48]	LRI [0.26]	Diarrhea [0.58]	HIV [6.91]	Road Inj [0.46]	Headaches [1.1]	Asthma [1.05]	Iron [0.71]
Guatemala	Neonatal [0.54]	LRI [0.78]	Diarrhea [0.95]	Congenital [0.51]	Violence [2.74]	PEM [1.11]	Road Inj [0.4]	F Body [0.92]	Headaches [1.09]	Iron [0.6]
Honduras	Neonatal [0.37]	Diarrhea [0.4]	Violence [3.11]	Congenital [0.2]	LRI [0.07]	Endocrine [2.1]	Headaches [1.1]	Road Inj [0.35]	Asthma [1.1]	Iron [0.66]

eFigure 7a. Leading ten causes of DALYs with the ratio of observed DALYs to DALYs expected on the basis of Socio-Demographic Index alone in 2017, <20 years, both sexes combined. The top ten causes contributing to DALYs are listed globally, by socio-demographic quintile, and then by GBD superregion, region, country, and subnationally where modeled. For each cell, the ratio of observed DALYs to DALYs expected on the basis of socio-demographic index (SDI) alone are listed. Abbreviations: DALY=disability-adjusted life year, GBD=Global Burden of Disease.

Values shown in brackets represent the ratio of observed DALYs to predicted DALYs on the basis of Socio-Demographic Index (SDI), rounded to two (2) digits. Color ranges (shown below) were calculated to place a roughly equal number of cells into each bin.

COLOR KEY:	[0.0-0.5]	(0.5-0.69]	(0.69-0.82]	(0.82-0.92]	(0.92-1.01]	(1.01-1.18]	(1.18-1.48]	(1.48-2.26]	2.26+

	1	2	3	4	5	6	7	8	9	10
Mexico	Neonatal [0.53]	Congenital [0.64]	Violence [3.59]	LRI [0.36]	Road Inj [0.6]	Diarrhea [0.71]	Headaches [0.97]	Epilepsy [1.38]	F Body [0.83]	Conduct [0.97]
Aguascalientes	Neonatal [0.62]	Congenital [0.72]	Violence [2.8]	Road Inj [0.73]	LRI [0.33]	Headaches [0.97]	Diarrhea [0.86]	F Body [1.13]	Epilepsy [1.46]	Conduct [0.98]
Baja CA	Neonatal [0.46]	Congenital [0.51]	Violence [3.73]	LRI [0.3]	F Body [1.36]	Headaches [0.99]	Road Inj [0.47]	Conduct [1.0]	Epilepsy [1.27]	Diarrhea [0.47]
Baja CA Sur	Neonatal [0.54]	Congenital [0.56]	Violence [3.24]	Road Inj [0.67]	LRI [0.31]	Headaches [0.97]	Epilepsy [1.32]	Conduct [0.99]	Diarrhea [0.67]	Asthma [0.84]
Campeche	Neonatal [0.6]	Congenital [0.57]	Violence [2.7]	LRI [0.26]	Road Inj [0.49]	Diarrhea [0.54]	Headaches [0.96]	Epilepsy [1.17]	Conduct [0.96]	Self Harm [1.25]
Chiapas	Neonatal [0.25]	Congenital [0.42]	LRI [0.28]	Diarrhea [0.54]	Violence [2.18]	Road Inj [0.39]	Epilepsy [1.34]	Headaches [1.0]	Leukemia [1.08]	PEM [0.78]
Chihuahua	Neonatal [0.72]	Congenital [0.75]	Violence [5.39]	LRI [0.47]	Road Inj [0.79]	F Body [1.8]	Headaches [1.0]	Diarrhea [0.76]	Epilepsy [1.44]	Conduct [1.0]
Coahuila	Neonatal [0.74]	Congenital [0.72]	Violence [3.27]	LRI [0.36]	Road Inj [0.54]	Headaches [0.97]	F Body [1.16]	Diarrhea [0.74]	Epilepsy [1.35]	Conduct [0.97]
Colima	Neonatal [0.58]	Congenital [0.64]	Violence [3.92]	Road Inj [0.66]	LRI [0.32]	Headaches [0.97]	Diarrhea [0.73]	Epilepsy [1.39]	Conduct [0.98]	Asthma [0.82]
Mexico City	Neonatal [0.98]	Congenital [1.11]	Violence [5.82]	LRI [1.29]	Headaches [0.95]	Road Inj [0.52]	Epilepsy [1.75]	Diarrhea [1.22]	Conduct [0.94]	Dermatitis [0.82]
Durango	Neonatal [0.67]	Congenital [0.66]	Violence [3.7]	Road Inj [0.72]	LRI [0.28]	Headaches [0.97]	F Body [0.99]	Diarrhea [0.53]	Epilepsy [1.26]	Conduct [0.97]
Guanajuato	Neonatal [0.49]	Congenital [0.59]	Violence [2.99]	Road Inj [0.71]	LRI [0.23]	Headaches [0.98]	Epilepsy [1.32]	Diarrhea [0.47]	Conduct [0.98]	Asthma [0.83]
Guerrero	Neonatal [0.36]	Congenital [0.63]	Violence [5.94]	LRI [0.24]	Diarrhea [0.48]	Road Inj [0.67]	Headaches [1.01]	F Body [0.81]	PEM [1.16]	Epilepsy [1.17]
Hidalgo	Neonatal [0.37]	Congenital [0.41]	Violence [2.56]	Road Inj [0.59]	LRI [0.15]	Headaches [1.0]	Epilepsy [1.32]	F Body [0.76]	Conduct [0.99]	Diarrhea [0.25]
Jalisco	Neonatal [0.54]	Congenital [0.68]	Violence [3.63]	Road Inj [0.77]	LRI [0.34]	Headaches [0.96]	Epilepsy [1.54]	Conduct [0.97]	Diarrhea [0.58]	Leukemia [0.95]
Mexico	Neonatal [0.56]	Congenital [0.58]	Violence [3.79]	LRI [0.39]	Road Inj [0.48]	Headaches [0.93]	Diarrhea [0.65]	F Body [0.92]	Epilepsy [1.31]	Conduct [0.98]
Michoacan	Neonatal [0.36]	Congenital [0.62]	Violence [3.56]	LRI [0.27]	Road Inj [0.79]	Diarrhea [0.42]	F Body [1.03]	Headaches [0.98]	Epilepsy [1.22]	Conduct [0.97]
Morelos	Neonatal [0.48]	Congenital [0.59]	Violence [4.37]	Road Inj [0.5]	LRI [0.22]	Headaches [0.97]	Epilepsy [1.38]	Diarrhea [0.57]	Conduct [0.97]	Disaster [121.51]
Nayarit	Neonatal [0.4]	Congenital [0.53]	Violence [3.34]	Road Inj [0.78]	LRI [0.23]	Headaches [0.96]	Diarrhea [0.51]	Epilepsy [1.27]	Conduct [0.97]	Asthma [0.81]
Nuevo Leon	Neonatal [0.62]	Congenital [0.79]	Violence [3.8]	LRI [0.4]	Headaches [0.95]	Road Inj [0.45]	F Body [1.23]	Epilepsy [1.36]	Conduct [0.96]	Diarrhea [0.67]
Oaxaca	Neonatal [0.28]	Congenital [0.57]	Violence [3.29]	LRI [0.24]	Diarrhea [0.54]	Road Inj [0.54]	PEM [1.75]	Epilepsy [1.48]	Headaches [1.02]	Leukemia [0.99]
Puebla	Neonatal [0.57]	Congenital [0.77]	LRI [0.4]	Violence [2.73]	Road Inj [0.66]	Diarrhea [0.52]	Headaches [0.99]	F Body [0.84]	PEM [1.64]	Leukemia [1.02]
Queretaro	Neonatal [0.54]	Congenital [0.68]	Violence [2.76]	LRI [0.31]	Road Inj [0.63]	Headaches [0.96]	F Body [0.98]	Diarrhea [0.63]	Epilepsy [1.37]	Conduct [0.97]
Quintana Roo	Neonatal [0.53]	Congenital [0.55]	Violence [3.07]	LRI [0.26]	Road Inj [0.52]	Diarrhea [0.68]	Headaches [0.96]	F Body [0.89]	Epilepsy [1.25]	Conduct [0.97]
San Luis Potosi	Neonatal [0.44]	Congenital [0.75]	Violence [3.22]	LRI [0.36]	Road Inj [0.71]	Diarrhea [0.62]	Headaches [0.99]	Epilepsy [1.55]	F Body [0.84]	Conduct [0.99]
Sinaloa	Neonatal [0.5]	Congenital [0.59]	Violence [4.59]	Road Inj [0.76]	LRI [0.3]	Headaches [0.97]	Epilepsy [1.3]	Conduct [0.98]	Diarrhea [0.61]	Asthma [0.82]
Sonora	Neonatal [0.51]	Congenital [0.56]	Violence [3.52]	Road Inj [0.69]	LRI [0.35]	Headaches [0.98]	Diarrhea [0.72]	Epilepsy [1.4]	Conduct [0.99]	F Body [0.77]
Tabasco	Neonatal [0.7]	Congenital [0.76]	Violence [2.98]	Road Inj [0.87]	LRI [0.31]	Diarrhea [0.64]	Drown [0.65]	Headaches [0.98]	Epilepsy [1.34]	Leukemia [1.08]
Tamaulipas	Neonatal [0.64]	Congenital [0.63]	Violence [3.88]	Road Inj [0.53]	LRI [0.27]	Headaches [0.97]	Diarrhea [0.62]	Conduct [0.98]	Epilepsy [1.24]	F Body [0.81]
Tlaxcala	Neonatal [0.5]	Congenital [0.59]	LRI [0.34]	Violence [2.54]	Road Inj [0.61]	Headaches [1.0]	Diarrhea [0.45]	Epilepsy [1.38]	F Body [0.85]	Conduct [0.99]
Veracruz	Neonatal [0.43]	Congenital [0.49]	Violence [3.0]	LRI [0.16]	Headaches [1.01]	Road Inj [0.39]	Diarrhea [0.35]	Epilepsy [1.31]	Conduct [1.0]	Asthma [0.76]

eFigure 7a. Leading ten causes of DALYs with the ratio of observed DALYs to DALYs expected on the basis of Socio-Demographic Index alone in 2017, <20 years, both sexes combined. The top ten causes contributing to DALYs are listed globally, by socio-demographic quintile, and then by GBD superregion, region, country, and subnationally where modeled. For each cell, the ratio of observed DALYs to DALYs expected on the basis of socio-demographic index (SDI) alone are listed. Abbreviations: DALY=disability-adjusted life year, GBD=Global Burden of Disease.

Values shown in brackets represent the ratio of observed DALYs to predicted DALYs on the basis of Socio-Demographic Index (SDI), rounded to two (2) digits. Color ranges (shown below) were calculated to place a roughly equal number of cells into each bin.

COLOR KEY:	[0.0-0.5]	(0.5-0.69]	(0.69-0.82]	(0.82-0.92]	(0.92-1.01]	(1.01-1.18]	(1.18-1.48]	(1.48-2.26]	2.26+

	1	2	3	4	5	6	7	8	9	10
Yucatan	Neonatal [0.48]	Congenital [0.63]	LRI [0.4]	Violence [2.54]	Headaches [0.99]	Diarrhea [0.67]	Road Inj [0.43]	Epilepsy [1.33]	Conduct [0.99]	Leukemia [0.87]
Zacatecas	Neonatal [0.41]	Congenital [0.5]	Violence [3.18]	Road Inj [0.94]	LRI [0.27]	Headaches [0.97]	Epilepsy [1.39]	Diarrhea [0.4]	F Body [0.79]	Conduct [0.97]
Nicaragua	Neonatal [0.39]	Congenital [0.44]	LRI [0.23]	Diarrhea [0.31]	Headaches [1.08]	Dermatitis [1.06]	HIV [2.44]	Road Inj [0.25]	Conduct [0.95]	Asthma [0.76]
Panama	Neonatal [0.7]	Congenital [0.82]	LRI [0.95]	Diarrhea [2.63]	Violence [2.84]	Headaches [0.98]	Road Inj [0.47]	Dermatitis [1.16]	Asthma [1.3]	PEM [9.39]
Venezuela	Neonatal [0.85]	Violence [7.18]	Congenital [0.63]	Diarrhea [1.96]	LRI [0.53]	Road Inj [0.92]	STI [6.61]	Headaches [0.97]	F Body [1.04]	Dermatitis [0.99]
**Tropical Latin America**	Neonatal [0.73]	Congenital [0.72]	Violence [5.08]	LRI [0.51]	Road Inj [0.77]	Headaches [1.49]	Back Pain [2.15]	Diarrhea [1.03]	Asthma [1.37]	Anxiety [1.22]
Brazil	Neonatal [0.77]	Congenital [0.75]	Violence [5.36]	LRI [0.55]	Road Inj [0.78]	Headaches [1.49]	Back Pain [2.17]	Diarrhea [1.08]	Asthma [1.37]	Anxiety [1.21]
Acre	Neonatal [0.65]	Congenital [0.67]	Violence [4.43]	LRI [0.42]	Diarrhea [0.74]	Road Inj [0.66]	Headaches [1.59]	Iron [1.44]	Back Pain [2.41]	Drown [0.61]
Alagoas	Neonatal [0.45]	Violence [7.96]	Congenital [0.52]	LRI [0.29]	Diarrhea [0.62]	Road Inj [0.69]	Headaches [1.56]	Back Pain [2.33]	Iron [0.82]	Asthma [1.03]
Amapa	Neonatal [1.04]	Violence [8.2]	Congenital [0.66]	LRI [0.65]	Headaches [1.56]	Drown [1.24]	Road Inj [0.65]	Back Pain [2.19]	Iron [1.58]	Asthma [1.31]
Amazonas	Neonatal [0.64]	Congenital [0.66]	Violence [4.84]	LRI [0.49]	Diarrhea [1.09]	Headaches [1.51]	Back Pain [2.22]	Iron [1.37]	Road Inj [0.49]	Drown [0.77]
Bahia	Neonatal [0.66]	Congenital [0.8]	LRI [0.38]	Violence [3.63]	Diarrhea [0.8]	Headaches [1.49]	Back Pain [2.23]	Road Inj [0.49]	Iron [1.09]	Asthma [1.11]
Ceara	Neonatal [0.56]	Congenital [0.71]	Violence [5.79]	LRI [0.31]	Road Inj [0.75]	Headaches [1.47]	Diarrhea [0.63]	Back Pain [2.18]	Iron [1.01]	Asthma [1.17]
Distrito Federal	Neonatal [1.43]	Congenital [1.11]	Violence [11.11]	Headaches [1.46]	Road Inj [0.88]	Back Pain [1.96]	LRI [1.45]	Asthma [1.61]	Anxiety [1.06]	Depression [1.24]
Espirito Santo	Neonatal [0.75]	Congenital [0.81]	Violence [8.93]	Road Inj [0.83]	Headaches [1.49]	LRI [0.43]	Back Pain [2.1]	Iron [1.85]	Asthma [1.61]	Anxiety [1.21]
Goias	Neonatal [0.68]	Violence [7.69]	Congenital [0.64]	Road Inj [0.98]	Headaches [1.56]	Back Pain [2.3]	LRI [0.31]	Asthma [1.37]	Anxiety [1.28]	Depression [1.44]
Maranhao	Neonatal [0.36]	Congenital [0.54]	LRI [0.2]	Diarrhea [0.26]	Headaches [1.54]	Iron [0.91]	Road Inj [0.48]	Violence [1.38]	Back Pain [2.31]	Vit A [1.13]
Mato Grosso	Neonatal [0.75]	Congenital [0.66]	Violence [5.09]	Road Inj [1.14]	LRI [0.5]	Headaches [1.5]	Back Pain [2.19]	Asthma [1.47]	Diarrhea [0.99]	STI [5.72]
Mato Grosso do Sul	Neonatal [0.67]	Congenital [0.72]	Violence [3.72]	Road Inj [0.89]	LRI [0.43]	Headaches [1.52]	Back Pain [2.24]	Diarrhea [1.08]	Asthma [1.34]	Anxiety [1.25]
Minas Gerais	Neonatal [0.69]	Congenital [0.64]	Violence [4.82]	Road Inj [0.8]	Headaches [1.53]	Back Pain [2.27]	LRI [0.33]	Asthma [1.42]	Anxiety [1.26]	Depression [1.35]
Para	Neonatal [0.48]	Congenital [0.49]	LRI [0.36]	Violence [3.71]	Headaches [1.55]	Diarrhea [0.52]	Road Inj [0.55]	Back Pain [2.3]	Iron [0.94]	Asthma [1.18]
Paraiba	Neonatal [0.37]	Congenital [0.53]	Violence [4.99]	LRI [0.27]	Road Inj [0.72]	Headaches [1.47]	Back Pain [2.21]	Diarrhea [0.39]	Iron [0.84]	Vit A [1.31]
Parana	Neonatal [0.76]	Congenital [0.86]	Violence [5.62]	Road Inj [1.1]	Headaches [1.54]	Back Pain [2.24]	LRI [0.39]	Asthma [1.52]	Anxiety [1.23]	Depression [1.38]
Pernambuco	Neonatal [0.57]	Congenital [0.76]	Violence [6.74]	LRI [0.31]	Diarrhea [0.73]	Road Inj [0.68]	Headaches [1.49]	Back Pain [2.21]	Iron [1.16]	Asthma [1.22]
Piaui	Neonatal [0.56]	Congenital [0.6]	Road Inj [0.86]	LRI [0.18]	Violence [2.26]	Diarrhea [0.35]	Headaches [1.52]	Back Pain [2.32]	Asthma [1.08]	Anxiety [1.32]
Rio de Janeiro	Neonatal [0.96]	Violence [10.62]	Congenital [0.77]	LRI [0.91]	Road Inj [0.93]	Headaches [1.51]	Back Pain [1.89]	F Body [1.79]	Asthma [1.41]	Anxiety [1.18]
Rio Grande do Norte	Neonatal [0.43]	Violence [7.86]	Congenital [0.65]	LRI [0.39]	Road Inj [0.69]	Headaches [1.46]	Diarrhea [0.7]	Back Pain [2.17]	Iron [1.06]	Asthma [1.18]
Rio Grande do Sul	Neonatal [0.73]	Congenital [0.78]	Violence [5.89]	Back Pain [2.95]	Headaches [1.56]	Road Inj [0.8]	LRI [0.46]	STI [10.46]	Asthma [1.56]	Anxiety [1.22]
Rondonia	Neonatal [0.57]	Congenital [0.64]	Violence [4.4]	Road Inj [1.02]	LRI [0.31]	Headaches [1.58]	Back Pain [2.37]	Diarrhea [0.57]	Drown [0.63]	Asthma [1.23]
Roraima	Neonatal [0.73]	Congenital [0.8]	LRI [0.82]	Violence [3.99]	Road Inj [1.11]	Diarrhea [1.37]	Headaches [1.44]	Back Pain [2.11]	Drown [0.91]	Iron [1.44]
Santa Catarina	Neonatal [0.82]	Congenital [0.8]	Road Inj [1.23]	Headaches [1.82]	Violence [2.82]	Back Pain [2.14]	LRI [0.53]	Asthma [1.67]	Anxiety [1.19]	Depression [1.51]

eFigure 7a. Leading ten causes of DALYs with the ratio of observed DALYs to DALYs expected on the basis of Socio-Demographic Index alone in 2017, <20 years, both sexes combined. The top ten causes contributing to DALYs are listed globally, by socio-demographic quintile, and then by GBD superregion, region, country, and subnationally where modeled. For each cell, the ratio of observed DALYs to DALYs expected on the basis of socio-demographic index (SDI) alone are listed. Abbreviations: DALY=disability-adjusted life year, GBD=Global Burden of Disease.

Values shown in brackets represent the ratio of observed DALYs to predicted DALYs on the basis of Socio-Demographic Index (SDI), rounded to two (2) digits. Color ranges (shown below) were calculated to place a roughly equal number of cells into each bin.

COLOR KEY:	[0.0-0.5]	(0.5-0.69]	(0.69-0.82]	(0.82-0.92]	(0.92-1.01]	(1.01-1.18]	(1.18-1.48]	(1.48-2.26]	2.26+

	1	2	3	4	5	6	7	8	9	10
Sao Paulo	Neonatal [0.95]	Congenital [0.78]	Violence [3.45]	Headaches [1.44]	Back Pain [2.12]	LRI [0.75]	Road Inj [0.69]	Asthma [1.51]	Anxiety [1.15]	Depression [1.43]
Sergipe	Neonatal [0.72]	Violence [7.91]	Congenital [0.64]	LRI [0.42]	Road Inj [0.97]	Headaches [1.51]	Diarrhea [0.8]	Back Pain [2.21]	Iron [1.24]	Asthma [1.31]
Tocantins	Neonatal [0.6]	Congenital [0.61]	Violence [3.75]	Road Inj [1.09]	LRI [0.27]	Headaches [1.51]	Back Pain [2.27]	Diarrhea [0.57]	Leish [202.73]	Iron [1.12]
Paraguay	Neonatal [0.33]	Congenital [0.47]	STI [9.91]	Road Inj [0.69]	HIV [9.32]	LRI [0.25]	Headaches [1.43]	Diarrhea [0.8]	Asthma [1.49]	Back Pain [1.41]
**North Africa and Middle East**	Neonatal [1.06]	Congenital [1.16]	LRI [1.01]	Diarrhea [2.79]	Conflict Terror [1273.33]	Road Inj [1.13]	Iron [1.71]	Headaches [0.96]	Meningitis [0.79]	Anxiety [1.07]
Afghanistan	Neonatal [0.53]	LRI [0.43]	Congenital [1.02]	Diarrhea [0.19]	Meningitis [0.57]	Measles [0.23]	Whooping [0.65]	Road Inj [0.61]	Falls [2.32]	Drown [0.41]
Algeria	Neonatal [1.24]	Congenital [1.64]	Road Inj [1.07]	LRI [0.66]	Headaches [0.84]	Diarrhea [1.13]	Asthma [1.06]	Anxiety [0.95]	Conduct [0.86]	Back Pain [0.96]
Bahrain	Neonatal [0.52]	Congenital [0.61]	Road Inj [0.57]	Headaches [0.89]	Anxiety [0.98]	Dermatitis [0.89]	Conduct [0.91]	Back Pain [1.01]	Asthma [0.97]	Diarrhea [1.02]
Egypt	LRI [0.98]	Neonatal [0.4]	Congenital [0.74]	Diarrhea [2.12]	Road Inj [1.24]	Iron [1.33]	Headaches [0.99]	Asthma [1.23]	Back Pain [1.27]	Conduct [0.91]
Iran	Neonatal [1.1]	Congenital [1.14]	Road Inj [1.61]	Headaches [1.25]	LRI [0.57]	Anxiety [1.33]	Diarrhea [1.14]	Depression [1.28]	Conduct [0.98]	Back Pain [0.98]
Iraq	Neonatal [0.85]	Conflict Terror [3204.14]	Congenital [0.88]	LRI [0.32]	Road Inj [0.55]	Iron [1.18]	Diarrhea [0.43]	Headaches [0.94]	Violence [1.13]	Mech [2.03]
Jordan	Neonatal [1.22]	Congenital [1.24]	LRI [0.82]	Road Inj [0.79]	Headaches [0.94]	Iron [1.48]	Asthma [1.16]	Anxiety [1.05]	Conduct [0.95]	Back Pain [1.08]
Kuwait	Neonatal [0.87]	Congenital [1.16]	Road Inj [0.85]	Headaches [0.72]	Asthma [1.18]	LRI [0.94]	Anxiety [0.88]	Conduct [0.92]	Back Pain [0.92]	Diarrhea [1.87]
Lebanon	Neonatal [0.64]	Congenital [0.56]	Headaches [0.91]	Anxiety [1.18]	Asthma [1.27]	Diarrhea [1.62]	Dermatitis [0.88]	Conflict Terror [509.24]	Conduct [0.89]	Road Inj [0.33]
Libya	Neonatal [0.78]	Conflict Terror [3442.49]	Congenital [0.84]	Road Inj [1.21]	Diarrhea [2.48]	Headaches [0.91]	Oth Cardio [9.11]	LRI [0.72]	Anxiety [0.95]	Oth Trans [6.25]
Morocco	Neonatal [0.63]	Congenital [0.94]	Road Inj [0.89]	LRI [0.25]	Diarrhea [0.66]	Headaches [1.04]	Iron [0.91]	Depression [1.79]	Back Pain [1.47]	Anxiety [1.25]
Palestine	Neonatal [0.5]	Congenital [0.53]	Road Inj [0.42]	Headaches [0.99]	LRI [0.07]	Depression [1.63]	Conflict Terror [141.66]	Anxiety [1.2]	Diarrhea [0.15]	Iron [0.54]
Oman	Neonatal [1.21]	Congenital [1.01]	Road Inj [1.94]	LRI [0.66]	Headaches [0.78]	Iron [2.12]	Diarrhea [1.67]	Stroke [3.82]	Oth Cardio [7.46]	Anxiety [0.85]
Qatar	Neonatal [0.74]	Congenital [0.98]	Road Inj [1.12]	Headaches [0.77]	Dermatitis [0.88]	Anxiety [0.83]	Conduct [0.86]	Back Pain [0.89]	Asthma [0.87]	Depression [0.87]
Saudi Arabia	Neonatal [1.05]	Congenital [0.79]	Road Inj [1.04]	Headaches [1.01]	Conflict Terror [1133.81]	Anxiety [0.98]	Back Pain [1.09]	Conduct [0.99]	Dermatitis [0.85]	Diarrhea [1.87]
Sudan	Neonatal [0.97]	Congenital [1.61]	Diarrhea [0.98]	LRI [0.38]	Road Inj [1.46]	Iron [1.22]	Malaria [32.96]	STI [1.25]	Whooping [0.84]	Meningitis [0.22]
Syria	Conflict Terror [11497.8]	Neonatal [0.24]	Congenital [0.34]	Iron [1.25]	Headaches [1.11]	Road Inj [0.42]	LRI [0.15]	Whooping [5.68]	Anxiety [1.31]	Back Pain [1.36]
Tunisia	Neonatal [0.64]	Congenital [0.59]	Road Inj [0.81]	Headaches [0.93]	Anxiety [1.06]	Mech [2.32]	Conduct [0.94]	Dermatitis [0.87]	LRI [0.21]	Diarrhea [0.74]
Turkey	Neonatal [1.02]	Congenital [1.03]	Road Inj [0.68]	Headaches [0.8]	Back Pain [1.24]	LRI [0.5]	Conduct [0.96]	Epilepsy [1.44]	Depression [1.12]	Anxiety [0.82]
UAE	Congenital [1.79]	Neonatal [1.09]	Road Inj [1.86]	Asthma [1.41]	Headaches [0.81]	Dermatitis [0.87]	Diarrhea [2.25]	Anxiety [0.82]	Conduct [0.86]	Back Pain [0.84]
Yemen	Neonatal [0.74]	Diarrhea [0.8]	Congenital [0.78]	Conflict Terror [684.8]	Iron [3.34]	LRI [0.24]	Road Inj [1.22]	Vit A [1.51]	Whooping [0.73]	PEM [0.31]

eFigure 7a. Leading ten causes of DALYs with the ratio of observed DALYs to DALYs expected on the basis of Socio-Demographic Index alone in 2017, <20 years, both sexes combined. The top ten causes contributing to DALYs are listed globally, by socio-demographic quintile, and then by GBD superregion, region, country, and subnationally where modeled. For each cell, the ratio of observed DALYs to DALYs expected on the basis of socio-demographic index (SDI) alone are listed. Abbreviations: DALY=disability-adjusted life year, GBD=Global Burden of Disease.

Values shown in brackets represent the ratio of observed DALYs to predicted DALYs on the basis of Socio-Demographic Index (SDI), rounded to two (2) digits. Color ranges (shown below) were calculated to place a roughly equal number of cells into each bin.

COLOR KEY:	[0.0-0.5]	(0.5-0.69]	(0.69-0.82]	(0.82-0.92]	(0.92-1.01]	(1.01-1.18]	(1.18-1.48]	(1.48-2.26]	2.26+

	1	2	3	4	5	6	7	8	9	10
**South Asia**	Neonatal [1.03]	LRI [0.7]	Diarrhea [1.13]	Congenital [0.48]	Iron [1.92]	Typh + Paratyph [37.58]	Drown [0.63]	Malaria [126.58]	Vit A [1.53]	Meningitis [0.42]
Bangladesh	Neonatal [0.67]	LRI [0.39]	Congenital [0.34]	Drown [1.3]	Typh + Paratyph [14.69]	Diarrhea [0.18]	Iron [0.94]	STI [0.88]	PEM [0.41]	Headaches [1.2]
Bhutan	Neonatal [0.77]	LRI [0.46]	Congenital [0.46]	Iron [3.03]	Diarrhea [0.43]	Headaches [1.02]	Vit A [1.35]	Mech [2.0]	Encepha [1.55]	Malaria [156.8]
India	Neonatal [0.96]	LRI [0.78]	Diarrhea [1.37]	Congenital [0.49]	Iron [2.16]	Typh + Paratyph [51.21]	Malaria [209.67]	Vit A [1.88]	PEM [1.54]	Headaches [1.02]
Andhra Pradesh	Neonatal [0.68]	LRI [0.29]	Congenital [0.35]	Diarrhea [0.61]	Iron [1.76]	Typh + Paratyph [27.73]	Vit A [1.55]	Headaches [1.08]	Self Harm [1.57]	PEM [0.69]
Arunachal Pradesh	Neonatal [0.62]	LRI [0.45]	Diarrhea [0.8]	Iron [1.95]	Congenital [0.25]	Malaria [329.54]	Vit A [1.62]	Typh + Paratyph [32.66]	Headaches [1.02]	Hep [5.54]
Assam	Neonatal [1.13]	LRI [0.84]	Diarrhea [1.45]	Congenital [0.71]	Malaria [282.75]	Iron [1.88]	Drown [1.06]	Hep [9.68]	Typh + Paratyph [29.16]	PEM [1.24]
Bihar	Neonatal [0.64]	LRI [0.5]	Diarrhea [0.82]	Congenital [0.31]	Iron [1.46]	Vit A [1.28]	Typh + Paratyph [5.09]	Malaria [7.49]	Drown [0.46]	Encepha [1.81]
Chhattisgarh	Neonatal [0.98]	LRI [0.63]	Diarrhea [0.67]	Congenital [0.33]	Typh + Paratyph [29.9]	Iron [1.75]	Malaria [124.33]	PEM [1.21]	Drown [0.62]	Vit A [1.4]
Delhi	Neonatal [2.12]	LRI [2.48]	Iron [7.92]	Congenital [0.65]	Diarrhea [4.73]	Typh + Paratyph [1774.67]	Vit A [3.91]	Headaches [0.93]	PEM [18.21]	STI [9.75]
Goa	Neonatal [1.53]	LRI [2.75]	Congenital [0.7]	Diarrhea [2.86]	Headaches [0.99]	Vit A [3.24]	Drown [1.2]	Iron [1.96]	STI [12.13]	Encepha [6.07]
Gujarat	Neonatal [1.09]	LRI [0.71]	Congenital [0.55]	Iron [3.76]	Diarrhea [0.99]	Malaria [496.36]	Vit A [2.22]	Typh + Paratyph [71.45]	PEM [2.13]	Headaches [0.98]
Haryana	Neonatal [1.03]	LRI [0.99]	Iron [4.49]	Congenital [0.49]	Diarrhea [1.65]	Typh + Paratyph [177.65]	Vit A [2.56]	PEM [3.55]	Malaria [357.3]	Road Inj [0.4]
Himachal Pradesh	Neonatal [1.14]	LRI [1.08]	Congenital [0.57]	Iron [4.58]	Diarrhea [1.56]	Typh + Paratyph [216.74]	Vit A [2.54]	Headaches [0.99]	Road Inj [0.37]	STI [3.76]
Jammu & Kashmir	Neonatal [0.86]	LRI [0.97]	Congenital [0.57]	Diarrhea [0.84]	Iron [1.82]	Vit A [2.16]	Road Inj [0.46]	Headaches [1.05]	Typh + Paratyph [43.83]	Conduct [0.96]
Jharkhand	Neonatal [0.58]	LRI [0.44]	Diarrhea [0.77]	Congenital [0.34]	Iron [1.8]	Malaria [50.76]	Drown [0.73]	Vit A [1.5]	Typh + Paratyph [10.85]	Road Inj [0.41]
Karnataka	Neonatal [0.85]	Congenital [0.6]	LRI [0.41]	Diarrhea [0.7]	Iron [1.68]	Self Harm [3.35]	Vit A [1.9]	Drown [0.62]	PEM [1.78]	Headaches [1.0]
Kerala	Neonatal [0.59]	Congenital [0.57]	LRI [0.29]	Headaches [0.97]	Iron [1.36]	Vit A [2.13]	STI [4.32]	Self Harm [1.21]	Conduct [0.86]	Anxiety [0.83]
Madhya Pradesh	Neonatal [0.77]	LRI [0.63]	Diarrhea [0.55]	Congenital [0.39]	Iron [2.19]	Typh + Paratyph [23.18]	Malaria [49.5]	PEM [0.92]	Vit A [1.62]	Drown [0.6]
Maharashtra	Neonatal [0.99]	LRI [0.57]	Congenital [0.4]	Iron [3.09]	Diarrhea [1.04]	Vit A [2.29]	Typh + Paratyph [127.04]	Headaches [1.0]	PEM [3.59]	STI [2.88]
Manipur	Neonatal [0.92]	LRI [0.66]	Congenital [0.46]	Diarrhea [1.32]	Typh + Paratyph [119.5]	HIV [7.28]	Malaria [335.61]	Drown [0.68]	Headaches [1.04]	Road Inj [0.41]
Meghalaya	Neonatal [0.87]	LRI [0.91]	Malaria [1232.76]	Congenital [0.54]	Diarrhea [1.2]	Typh + Paratyph [89.17]	Iron [2.14]	Vit A [1.79]	Meningitis [0.47]	Headaches [1.03]

eFigure 7a. Leading ten causes of DALYs with the ratio of observed DALYs to DALYs expected on the basis of Socio-Demographic Index alone in 2017, <20 years, both sexes combined. The top ten causes contributing to DALYs are listed globally, by socio-demographic quintile, and then by GBD superregion, region, country, and subnationally where modeled. For each cell, the ratio of observed DALYs to DALYs expected on the basis of socio-demographic index (SDI) alone are listed. Abbreviations: DALY=disability-adjusted life year, GBD=Global Burden of Disease.

Values shown in brackets represent the ratio of observed DALYs to predicted DALYs on the basis of Socio-Demographic Index (SDI), rounded to two (2) digits. Color ranges (shown below) were calculated to place a roughly equal number of cells into each bin.

COLOR KEY:	[0.0-0.5]	(0.5-0.69]	(0.69-0.82]	(0.82-0.92]	(0.92-1.01]	(1.01-1.18]	(1.18-1.48]	(1.48-2.26]	2.26+

	1	2	3	4	5	6	7	8	9	10
Mizoram	Neonatal [1.13]	LRI [1.71]	Congenital [0.56]	Malaria [1622.17]	Typh + Paratyph [326.55]	Diarrhea [1.08]	HIV [6.99]	Headaches [1.0]	Meningitis [0.64]	Vit A [1.64]
Nagaland	Neonatal [1.38]	LRI [2.45]	Malaria [2520.32]	Congenital [0.4]	Diarrhea [1.76]	Typh + Paratyph [348.37]	Iron [2.53]	HIV [13.04]	Hep [15.58]	Drown [1.0]
Odisha	Neonatal [0.98]	LRI [0.66]	Diarrhea [1.11]	Malaria [448.51]	Congenital [0.47]	Typh + Paratyph [29.36]	PEM [1.34]	Iron [1.0]	Vit A [1.59]	Drown [0.63]
Punjab	Neonatal [1.06]	LRI [0.91]	Congenital [0.63]	Diarrhea [1.89]	Iron [3.65]	Typh + Paratyph [231.07]	Vit A [2.36]	Headaches [0.98]	Road Inj [0.42]	STI [2.99]
Rajasthan	Neonatal [0.83]	LRI [0.92]	Diarrhea [0.53]	Congenital [0.36]	Typh + Paratyph [26.54]	Iron [1.61]	Malaria [48.4]	Vit A [1.51]	PEM [0.82]	Measles [1.43]
Sikkim	Neonatal [0.6]	LRI [0.93]	Iron [3.05]	Congenital [0.32]	Diarrhea [1.32]	Typh + Paratyph [239.92]	Headaches [1.05]	Vit A [2.11]	Measles [20.83]	STI [3.15]
Tamil Nadu	Neonatal [0.58]	Congenital [0.43]	LRI [0.38]	Iron [2.31]	Self Harm [3.5]	Diarrhea [0.9]	Vit A [2.45]	Typh + Paratyph [105.44]	Headaches [0.99]	Road Inj [0.39]
Telangana	Neonatal [0.77]	Iron [2.72]	LRI [0.32]	Congenital [0.34]	Diarrhea [0.86]	Typh + Paratyph [62.18]	Vit A [1.94]	Headaches [1.05]	Self Harm [1.68]	PEM [1.4]
Tripura	Neonatal [0.95]	LRI [0.99]	Congenital [0.61]	Diarrhea [0.81]	Malaria [327.17]	Iron [1.54]	Vit A [1.71]	Self Harm [2.16]	Headaches [1.07]	PEM [1.05]
Uttar Pradesh	Neonatal [0.99]	LRI [0.75]	Diarrhea [1.24]	Congenital [0.59]	Typh + Paratyph [23.74]	Iron [1.88]	PEM [1.13]	Malaria [51.07]	Meningitis [0.42]	Vit A [1.58]
Uttarakhand	Neonatal [0.83]	LRI [0.87]	Congenital [0.4]	Iron [3.32]	Typh + Paratyph [209.96]	Diarrhea [1.18]	Road Inj [0.53]	Vit A [2.06]	Headaches [1.04]	STI [2.69]
W Bengal	Neonatal [0.68]	LRI [0.49]	Congenital [0.43]	Diarrhea [0.45]	Iron [1.42]	Vit A [1.69]	Self Harm [2.26]	Drown [0.56]	Headaches [1.09]	Measles [3.71]
UTs other than Delhi	Neonatal [1.48]	LRI [1.22]	Congenital [0.67]	Iron [3.92]	Diarrhea [1.86]	Vit A [2.75]	Headaches [1.01]	STI [6.23]	Malaria [1324.16]	Meningitis [0.86]
Nepal	Neonatal [0.47]	LRI [0.31]	Diarrhea [0.16]	Congenital [0.18]	Typh + Paratyph [4.35]	Road Inj [0.43]	Iron [0.7]	Headaches [1.54]	STI [0.61]	Whooping [0.39]
Pakistan	Neonatal [1.54]	LRI [0.58]	Diarrhea [0.96]	Congenital [0.54]	Meningitis [0.78]	Road Inj [0.92]	Iron [1.49]	Drown [0.82]	Typh + Paratyph [10.42]	TB [1.35]
**Southeast Asia, East Asia, and Oceania**	Neonatal [0.78]	Congenital [0.68]	LRI [1.08]	Road Inj [0.75]	Drown [1.45]	Diarrhea [1.74]	Asthma [0.92]	Headaches [0.6]	Conduct [0.79]	STI [4.98]
**East Asia**	Neonatal [0.65]	Congenital [0.62]	LRI [0.9]	Drown [1.86]	Road Inj [0.75]	F Body [1.07]	Leukemia [1.05]	Anxiety [0.71]	Conduct [0.7]	Falls [1.02]
China	Neonatal [0.63]	Congenital [0.61]	LRI [0.83]	Drown [1.8]	Road Inj [0.73]	F Body [1.04]	Leukemia [1.04]	Anxiety [0.71]	Conduct [0.7]	Falls [1.03]
N Korea	Neonatal [0.38]	Congenital [0.53]	LRI [0.39]	Drown [1.09]	Road Inj [0.78]	Diarrhea [0.34]	F Body [0.73]	Leukemia [0.92]	Iron [0.55]	Anxiety [1.06]
Taiwan	Neonatal [0.63]	Congenital [0.62]	Road Inj [0.93]	Headaches [0.72]	Asthma [1.29]	Back Pain [0.88]	Anxiety [0.73]	Conduct [0.87]	Dermatitis [0.57]	Diarrhea [2.19]
**Oceania**	Neonatal [0.65]	Congenital [0.83]	LRI [0.44]	Diarrhea [0.7]	STI [4.02]	PEM [1.43]	Meningitis [0.57]	Drown [0.98]	Iron [1.19]	Asthma [1.97]
Am Samoa	Neonatal [0.56]	Congenital [0.36]	LRI [0.73]	Asthma [1.88]	Diarrhea [1.41]	Iron [1.69]	Drown [0.97]	Headaches [0.7]	Road Inj [0.34]	Dermatitis [0.85]
Micronesia	Neonatal [0.47]	Congenital [0.42]	LRI [0.32]	Iron [1.29]	Road Inj [0.53]	Asthma [1.52]	Self Harm [2.32]	Whooping [3.73]	Drown [0.59]	Diarrhea [0.33]
Fiji	Neonatal [0.95]	Congenital [0.94]	LRI [1.05]	Diarrhea [1.89]	Drown [1.13]	Asthma [1.9]	Iron [1.75]	Road Inj [0.5]	F Body [1.13]	Meningitis [0.78]
Guam	Neonatal [1.58]	Congenital [1.09]	LRI [3.83]	Self Harm [2.73]	Diarrhea [4.62]	Asthma [1.9]	Road Inj [0.66]	Dermatitis [0.84]	Headaches [0.62]	Iron [2.71]

eFigure 7a. Leading ten causes of DALYs with the ratio of observed DALYs to DALYs expected on the basis of Socio-Demographic Index alone in 2017, <20 years, both sexes combined. The top ten causes contributing to DALYs are listed globally, by socio-demographic quintile, and then by GBD superregion, region, country, and subnationally where modeled. For each cell, the ratio of observed DALYs to DALYs expected on the basis of socio-demographic index (SDI) alone are listed. Abbreviations: DALY=disability-adjusted life year, GBD=Global Burden of Disease.

Values shown in brackets represent the ratio of observed DALYs to predicted DALYs on the basis of Socio-Demographic Index (SDI), rounded to two (2) digits. Color ranges (shown below) were calculated to place a roughly equal number of cells into each bin.

COLOR KEY:	[0.0-0.5]	(0.5-0.69]	(0.69-0.82]	(0.82-0.92]	(0.92-1.01]	(1.01-1.18]	(1.18-1.48]	(1.48-2.26]	2.26+

	1	2	3	4	5	6	7	8	9	10
Kiribati	Neonatal [0.67]	Congenital [0.88]	LRI [0.2]	Diarrhea [0.28]	PEM [1.05]	Measles [1.39]	Meningitis [0.57]	Whooping [0.94]	Iron [0.91]	Asthma [1.64]
Marshall	Neonatal [0.55]	Congenital [0.58]	LRI [0.4]	Road Inj [0.63]	Whooping [3.37]	Self Harm [2.68]	Iron [1.04]	Drown [0.65]	Asthma [1.43]	Measles [5.48]
N Mariana	Neonatal [0.48]	Congenital [0.33]	Asthma [1.79]	Self Harm [2.01]	Drown [1.61]	Road Inj [0.51]	Diarrhea [2.21]	LRI [0.82]	Headaches [0.82]	Conduct [0.96]
PNG	Neonatal [0.58]	Congenital [0.81]	LRI [0.37]	Diarrhea [0.53]	STI [2.88]	PEM [1.01]	Meningitis [0.5]	Drown [0.96]	Iron [1.06]	Asthma [1.96]
Samoa	Neonatal [0.41]	Congenital [0.36]	LRI [0.27]	Iron [1.22]	Measles [11.49]	Asthma [1.45]	Diarrhea [0.34]	Whooping [3.22]	Vit A [1.28]	Road Inj [0.34]
Solomon	Neonatal [0.31]	STI [4.94]	LRI [0.26]	Congenital [0.43]	Iron [1.23]	Diarrhea [0.14]	Road Inj [0.5]	Drown [0.51]	Asthma [1.12]	Vit A [0.8]
Tonga	Neonatal [0.57]	LRI [0.45]	Congenital [0.34]	Meningitis [1.23]	Asthma [2.05]	Road Inj [0.53]	Drown [0.74]	Diarrhea [0.61]	Dermatitis [0.94]	Headaches [0.68]
Vanuatu	Neonatal [0.51]	LRI [0.4]	Congenital [0.6]	STI [2.79]	Diarrhea [0.24]	Iron [1.14]	Road Inj [0.56]	Whooping [1.16]	Drown [0.63]	Asthma [1.5]
**Southeast Asia**	Neonatal [0.8]	Congenital [0.69]	LRI [0.95]	Diarrhea [1.9]	Road Inj [0.73]	Drown [0.94]	STI [5.27]	Asthma [1.26]	Meningitis [0.74]	Headaches [0.84]
Cambodia	Neonatal [0.52]	LRI [0.64]	Congenital [0.64]	Drown [0.91]	Iron [1.31]	Road Inj [0.65]	Diarrhea [0.18]	Mech [3.02]	Malaria [27.58]	Headaches [0.9]
Indonesia	Neonatal [0.86]	Congenital [0.56]	Diarrhea [3.0]	LRI [0.72]	STI [11.26]	Road Inj [0.79]	Dengue [60.02]	Measles [57.05]	Typh + Paratyph [224.96]	Meningitis [0.87]
Laos	Neonatal [1.15]	LRI [1.4]	Congenital [1.23]	Diarrhea [1.34]	Road Inj [1.13]	Meningitis [0.73]	Drown [0.97]	TB [1.75]	Whooping [1.51]	Typh + Paratyph [14.38]
Malaysia	Neonatal [0.57]	Congenital [0.63]	Road Inj [1.01]	HIV [22.49]	LRI [0.85]	Headaches [0.75]	Depression [1.34]	Dermatitis [0.93]	Diarrhea [1.84]	Conduct [0.9]
Maldives	Neonatal [0.47]	Congenital [0.35]	Headaches [0.77]	Iron [1.03]	Dermatitis [0.94]	Asthma [0.95]	Dengue [32.58]	Conduct [0.86]	Drown [0.5]	Road Inj [0.26]
Mauritius	Neonatal [0.91]	Congenital [0.65]	Iron [3.11]	Road Inj [0.53]	LRI [0.55]	Headaches [0.89]	Diarrhea [1.49]	Asthma [1.23]	Depression [1.27]	Conduct [0.96]
Myanmar	Neonatal [0.81]	Congenital [1.08]	LRI [0.83]	Diarrhea [0.77]	Drown [1.1]	Road Inj [0.7]	Meningitis [0.59]	Oth Un Inf [3.52]	Typh + Paratyph [35.45]	Exec & Police [203.21]
Philippines	Neonatal [0.93]	LRI [1.27]	Congenital [0.78]	Diarrhea [1.45]	Asthma [2.07]	Drown [0.94]	Meningitis [0.87]	Dengue [34.06]	Road Inj [0.44]	TB [4.27]
Sri Lanka	Neonatal [0.48]	Congenital [0.46]	Headaches [0.81]	Dermatitis [0.89]	Self Harm [1.21]	Conduct [0.9]	Asthma [0.94]	LRI [0.21]	Typh + Paratyph [249.77]	Iron [0.86]
Seychelles	Neonatal [0.69]	Congenital [0.7]	LRI [1.2]	Drown [1.52]	Meningitis [1.16]	Road Inj [0.41]	Headaches [0.78]	Dermatitis [0.91]	Asthma [0.98]	Conduct [0.86]
Thailand	Neonatal [0.4]	Road Inj [1.45]	Congenital [0.4]	Drown [1.33]	LRI [0.4]	Headaches [0.99]	Typh + Paratyph [358.85]	Dermatitis [0.94]	Conduct [0.95]	Asthma [0.93]
Timor-Leste	Neonatal [0.62]	LRI [0.59]	Congenital [0.77]	Diarrhea [0.47]	Iron [1.39]	HIV [5.73]	Drown [0.67]	Road Inj [0.42]	Meningitis [0.31]	PEM [0.6]
Vietnam	Neonatal [0.33]	Congenital [0.61]	LRI [0.31]	Drown [1.27]	Road Inj [0.63]	Headaches [0.81]	Conduct [0.88]	Dermatitis [0.83]	Asthma [0.73]	Typh + Paratyph [42.83]
**Sub-Saharan Africa**	Neonatal [0.89]	LRI [0.81]	Malaria [123.33]	Diarrhea [1.18]	Congenital [0.71]	HIV [11.53]	Meningitis [0.95]	PEM [1.53]	STI [1.91]	Measles [1.37]
**Central Sub-Saharan Africa**	Neonatal [0.89]	Malaria [242.79]	LRI [0.83]	Diarrhea [1.16]	Congenital [1.05]	PEM [1.94]	Road Inj [1.35]	STI [2.94]	Meningitis [0.72]	Whooping [1.95]
Angola	Neonatal [0.85]	Diarrhea [1.47]	LRI [0.6]	Congenital [0.8]	Malaria [95.12]	PEM [2.01]	STI [2.98]	Road Inj [1.18]	HIV [7.74]	Measles [2.41]
C African Rep	Neonatal [0.9]	Diarrhea [1.36]	LRI [0.79]	Malaria [9.47]	Congenital [1.03]	Road Inj [2.81]	PEM [1.25]	HIV [10.35]	Whooping [1.59]	TB [1.91]
Congo	Neonatal [1.0]	HIV [52.02]	Malaria [2273.17]	Diarrhea [2.91]	Congenital [0.78]	LRI [0.73]	STI [9.2]	Road Inj [1.28]	Iron [1.85]	Meningitis [0.97]
Congo DR	Malaria [22.86]	Neonatal [0.63]	LRI [0.57]	Congenital [0.95]	Diarrhea [0.47]	PEM [0.82]	Road Inj [1.1]	Whooping [1.11]	STI [1.42]	Meningitis [0.46]

eFigure 7a. Leading ten causes of DALYs with the ratio of observed DALYs to DALYs expected on the basis of Socio-Demographic Index alone in 2017, <20 years, both sexes combined. The top ten causes contributing to DALYs are listed globally, by socio-demographic quintile, and then by GBD superregion, region, country, and subnationally where modeled. For each cell, the ratio of observed DALYs to DALYs expected on the basis of socio-demographic index (SDI) alone are listed. Abbreviations: DALY=disability-adjusted life year, GBD=Global Burden of Disease.

Values shown in brackets represent the ratio of observed DALYs to predicted DALYs on the basis of Socio-Demographic Index (SDI), rounded to two (2) digits. Color ranges (shown below) were calculated to place a roughly equal number of cells into each bin.

COLOR KEY:	[0.0-0.5]	(0.5-0.69]	(0.69-0.82]	(0.82-0.92]	(0.92-1.01]	(1.01-1.18]	(1.18-1.48]	(1.48-2.26]	2.26+

	1	2	3	4	5	6	7	8	9	10
Eq Guinea	Malaria [10485.87]	Neonatal [1.09]	HIV [69.0]	Congenital [0.82]	LRI [0.79]	Diarrhea [2.46]	STI [15.6]	Road Inj [0.97]	Measles [40.22]	Meningitis [1.1]
Gabon	Neonatal [1.68]	Malaria [12947.65]	Congenital [1.06]	LRI [1.23]	Diarrhea [3.5]	Road Inj [1.33]	Iron [3.79]	HIV [21.46]	STI [13.53]	Meningitis [1.49]
**Eastern Sub-Saharan Africa**	Neonatal [0.63]	LRI [0.41]	Diarrhea [0.56]	Congenital [0.64]	HIV [12.67]	Malaria [8.71]	PEM [0.82]	Meningitis [0.45]	STI [1.28]	Whooping [0.78]
Burundi	Neonatal [0.5]	Malaria [4.73]	LRI [0.36]	Congenital [0.89]	Diarrhea [0.35]	PEM [0.78]	Measles [0.31]	Meningitis [0.34]	TB [0.76]	HIV [4.0]
Comoros	Neonatal [0.61]	LRI [0.34]	Congenital [0.6]	Diarrhea [0.45]	PEM [0.57]	Meningitis [0.33]	Iron [0.89]	STI [1.03]	Whooping [0.77]	Road Inj [0.4]
Djibouti	Neonatal [0.62]	LRI [0.51]	Congenital [0.66]	Diarrhea [0.67]	PEM [1.67]	HIV [7.71]	STI [2.71]	Whooping [1.94]	Meningitis [0.49]	Road Inj [0.5]
Eritrea	Neonatal [0.62]	LRI [0.55]	Diarrhea [0.82]	Congenital [0.67]	PEM [1.26]	Meningitis [0.54]	TB [1.51]	HIV [5.43]	Iron [1.25]	Road Inj [0.61]
Ethiopia	Neonatal [0.55]	Diarrhea [0.46]	LRI [0.28]	Congenital [0.35]	PEM [0.48]	Whooping [0.75]	Meningitis [0.34]	HIV [3.79]	STI [0.71]	Measles [0.24]
Kenya	Neonatal [0.65]	Diarrhea [1.4]	LRI [0.61]	HIV [18.3]	Congenital [0.59]	Meningitis [0.61]	PEM [1.37]	Malaria [71.49]	iNTS [6.19]	Tetanus [22.63]
Baringo	Neonatal [0.45]	Diarrhea [0.78]	LRI [0.36]	Congenital [0.42]	PEM [0.64]	Meningitis [0.36]	iNTS [3.83]	Leish [22.38]	HIV [2.63]	Vit A [0.95]
Bomet	Neonatal [0.51]	HIV [31.72]	Diarrhea [0.68]	LRI [0.21]	Congenital [0.3]	iNTS [3.64]	Meningitis [0.23]	Dermatitis [1.12]	Vit A [0.7]	Conduct [1.0]
Bungoma	Neonatal [0.65]	Diarrhea [1.3]	LRI [0.51]	Congenital [0.58]	Malaria [54.4]	PEM [1.01]	Meningitis [0.49]	iNTS [6.16]	Whooping [1.22]	HIV [4.01]
Busia	Neonatal [0.65]	LRI [0.66]	Diarrhea [0.96]	Malaria [80.78]	Congenital [0.73]	Meningitis [0.53]	PEM [0.76]	HIV [4.72]	iNTS [4.99]	Whooping [0.75]
Elgeyo-Marakwet	Neonatal [0.8]	Diarrhea [1.39]	LRI [0.44]	Congenital [0.52]	HIV [7.13]	Meningitis [0.47]	PEM [0.94]	iNTS [4.82]	STI [1.28]	Whooping [0.85]
Embu	Neonatal [0.73]	Diarrhea [2.26]	LRI [0.76]	Congenital [0.67]	HIV [13.82]	Meningitis [0.77]	PEM [2.17]	iNTS [6.23]	Malaria [99.64]	Vit A [1.09]
Garissa	Neonatal [0.23]	Diarrhea [0.28]	LRI [0.15]	HIV [7.11]	Congenital [0.23]	iNTS [2.23]	Tetanus [2.31]	Meningitis [0.16]	Vit A [0.61]	Iron [0.47]
HomaBay	HIV [54.88]	Neonatal [0.61]	LRI [0.92]	Diarrhea [1.09]	Congenital [0.96]	Malaria [24.03]	PEM [1.33]	Meningitis [0.8]	Road Inj [0.92]	iNTS [6.79]
Isiolo	Neonatal [0.56]	LRI [0.67]	Congenital [0.78]	Diarrhea [0.4]	PEM [0.72]	Meningitis [0.54]	HIV [6.5]	Conflict Terror [134.28]	Tetanus [6.91]	Whooping [0.72]
Kajiado	Neonatal [0.58]	Diarrhea [1.01]	LRI [0.39]	Congenital [0.39]	HIV [8.22]	Meningitis [0.53]	Whooping [2.1]	Vit A [1.29]	iNTS [5.45]	PEM [0.94]
Kakamega	Neonatal [0.61]	Diarrhea [1.18]	LRI [0.65]	Congenital [0.68]	HIV [11.95]	Malaria [36.43]	PEM [1.18]	Meningitis [0.6]	iNTS [5.32]	Whooping [0.82]
Kericho	Neonatal [0.9]	Diarrhea [1.34]	LRI [0.49]	Congenital [0.59]	HIV [16.6]	iNTS [9.28]	Meningitis [0.46]	PEM [1.03]	STI [1.28]	Whooping [1.16]
Kiambu	Neonatal [1.43]	LRI [1.21]	Diarrhea [2.87]	Congenital [0.96]	HIV [16.85]	Meningitis [1.29]	PEM [3.73]	Whooping [5.7]	STI [3.81]	iNTS [9.12]
Kilifi	Neonatal [0.58]	Diarrhea [1.33]	LRI [0.47]	Congenital [0.54]	HIV [11.01]	PEM [1.08]	Meningitis [0.45]	Malaria [21.04]	Whooping [0.76]	Vit A [0.97]
Kirinyaga	Neonatal [0.98]	Diarrhea [2.63]	LRI [0.93]	Congenital [0.91]	HIV [11.9]	Meningitis [0.95]	PEM [2.39]	iNTS [6.7]	Malaria [125.59]	Road Inj [0.42]
Kisii	Neonatal [0.9]	Diarrhea [2.15]	LRI [0.69]	Congenital [0.74]	Meningitis [0.65]	HIV [6.12]	iNTS [7.77]	PEM [1.15]	STI [1.46]	Road Inj [0.36]
Kisumu	Neonatal [0.79]	HIV [47.45]	LRI [0.86]	Congenital [1.0]	Diarrhea [1.17]	Malaria [219.3]	Meningitis [1.08]	iNTS [14.51]	PEM [1.57]	TB [2.39]
Kitui	Neonatal [0.47]	Diarrhea [0.83]	HIV [12.12]	LRI [0.25]	Congenital [0.33]	iNTS [4.65]	Meningitis [0.3]	PEM [0.54]	Whooping [0.73]	Typh + Paratyph [3.84]
Kwale	Neonatal [0.53]	Diarrhea [0.56]	LRI [0.3]	HIV [9.76]	Congenital [0.39]	Malaria [28.38]	iNTS [4.91]	Meningitis [0.32]	PEM [0.5]	Iron [0.67]
Laikipia	Neonatal [0.65]	HIV [33.4]	Diarrhea [1.35]	LRI [0.34]	Congenital [0.37]	Meningitis [0.43]	iNTS [5.01]	Dermatitis [1.14]	Epilepsy [1.07]	STI [1.43]

eFigure 7a. Leading ten causes of DALYs with the ratio of observed DALYs to DALYs expected on the basis of Socio-Demographic Index alone in 2017, <20 years, both sexes combined. The top ten causes contributing to DALYs are listed globally, by socio-demographic quintile, and then by GBD superregion, region, country, and subnationally where modeled. For each cell, the ratio of observed DALYs to DALYs expected on the basis of socio-demographic index (SDI) alone are listed. Abbreviations: DALY=disability-adjusted life year, GBD=Global Burden of Disease.

Values shown in brackets represent the ratio of observed DALYs to predicted DALYs on the basis of Socio-Demographic Index (SDI), rounded to two (2) digits. Color ranges (shown below) were calculated to place a roughly equal number of cells into each bin.

COLOR KEY:	[0.0-0.5]	(0.5-0.69]	(0.69-0.82]	(0.82-0.92]	(0.92-1.01]	(1.01-1.18]	(1.18-1.48]	(1.48-2.26]	2.26+

	1	2	3	4	5	6	7	8	9	10
Lamu	Neonatal [0.97]	LRI [0.8]	Congenital [1.01]	Diarrhea [0.96]	Conflict Terror [1032.86]	PEM [1.51]	Meningitis [0.79]	HIV [6.36]	Whooping [1.56]	Malaria [23.59]
Machakos	Neonatal [1.1]	Diarrhea [2.26]	HIV [29.13]	LRI [0.64]	Congenital [0.7]	Meningitis [0.63]	iNTS [8.37]	PEM [1.33]	STI [1.67]	Whooping [1.34]
Makueni	HIV [27.61]	Neonatal [0.22]	Diarrhea [0.39]	LRI [0.1]	Congenital [0.17]	Dermatitis [1.11]	Meningitis [0.17]	Typh + Paratyph [3.95]	Conduct [1.06]	Epilepsy [0.87]
Mandera	Diarrhea [0.24]	Neonatal [0.13]	LRI [0.16]	HIV [4.69]	PEM [0.24]	Congenital [0.16]	Tetanus [1.8]	Meningitis [0.18]	iNTS [1.48]	Vit A [0.69]
Marsabit	Neonatal [0.47]	Diarrhea [0.47]	LRI [0.3]	HIV [13.28]	Congenital [0.39]	Tetanus [5.09]	PEM [0.43]	Meningitis [0.26]	iNTS [1.98]	Measles [0.14]
Meru	Neonatal [0.87]	Diarrhea [2.21]	LRI [0.9]	HIV [25.36]	Congenital [0.82]	PEM [2.29]	Meningitis [0.87]	Malaria [80.84]	TB [1.7]	iNTS [5.4]
Migori	Neonatal [0.76]	LRI [0.94]	HIV [41.75]	Diarrhea [1.31]	Congenital [1.03]	Malaria [20.8]	PEM [1.25]	Meningitis [0.78]	iNTS [5.78]	TB [1.29]
Mombasa	Neonatal [1.09]	LRI [1.21]	Diarrhea [3.19]	Congenital [0.95]	HIV [13.71]	Meningitis [1.29]	PEM [4.25]	iNTS [17.14]	Whooping [4.59]	STI [2.95]
Murang'a	Neonatal [0.71]	LRI [0.72]	Congenital [0.84]	HIV [20.28]	Diarrhea [0.61]	Meningitis [0.95]	Whooping [3.41]	iNTS [7.01]	PEM [1.22]	Road Inj [0.39]
Nairobi	Neonatal [1.4]	LRI [1.77]	Congenital [0.81]	Diarrhea [3.65]	Meningitis [2.13]	HIV [15.96]	STI [13.11]	PEM [12.65]	Dermatitis [1.36]	Road Inj [0.5]
Nakuru	Neonatal [0.76]	LRI [0.85]	Diarrhea [1.85]	Congenital [0.77]	HIV [16.63]	Meningitis [0.84]	PEM [2.53]	Epilepsy [1.41]	iNTS [5.19]	Typh + Paratyph [18.92]
Nandi	Neonatal [0.58]	Diarrhea [0.97]	HIV [14.77]	LRI [0.32]	Congenital [0.39]	iNTS [6.31]	Meningitis [0.35]	Malaria [40.52]	PEM [0.61]	Dermatitis [1.13]
Narok	Neonatal [0.22]	Diarrhea [0.27]	LRI [0.15]	Congenital [0.21]	Vit A [0.96]	HIV [2.39]	Meningitis [0.2]	iNTS [2.12]	PEM [0.2]	Typh + Paratyph [1.81]
Nyamira	Neonatal [0.84]	Diarrhea [1.43]	LRI [0.51]	Congenital [0.57]	HIV [17.87]	Meningitis [0.69]	iNTS [8.36]	Road Inj [0.51]	Epilepsy [1.56]	STI [1.75]
Nyandarua	Neonatal [0.94]	LRI [0.99]	Diarrhea [2.0]	Congenital [0.86]	HIV [13.43]	Meningitis [1.08]	PEM [2.24]	Whooping [2.95]	TB [2.6]	Road Inj [0.51]
Nyeri	Neonatal [0.84]	LRI [1.15]	Congenital [0.91]	Diarrhea [1.84]	HIV [23.9]	Meningitis [1.38]	PEM [3.47]	Whooping [5.14]	TB [2.98]	Road Inj [0.47]
Samburu	Neonatal [0.32]	HIV [20.5]	Diarrhea [0.29]	LRI [0.17]	Congenital [0.23]	Tetanus [2.75]	PEM [0.21]	Meningitis [0.15]	iNTS [1.58]	Whooping [0.27]
Siaya	Neonatal [0.6]	LRI [0.68]	Diarrhea [1.08]	Malaria [125.7]	Congenital [0.8]	HIV [17.92]	Meningitis [0.56]	iNTS [6.14]	PEM [0.76]	Vit A [1.02]
TaitaTaveta	Neonatal [0.56]	HIV [28.05]	LRI [0.64]	Diarrhea [1.44]	Congenital [0.58]	Meningitis [0.81]	PEM [2.1]	iNTS [8.94]	Malaria [126.96]	Whooping [1.99]
TanaRiver	Neonatal [0.68]	LRI [0.47]	HIV [19.32]	Diarrhea [0.51]	Congenital [0.57]	PEM [0.88]	Tetanus [9.32]	Meningitis [0.44]	iNTS [4.65]	TB [0.81]
TharakaNithi	Diarrhea [4.65]	Neonatal [1.01]	LRI [1.28]	Congenital [1.02]	PEM [4.92]	Meningitis [1.21]	iNTS [9.89]	Malaria [154.59]	TB [2.57]	Road Inj [0.48]
TransNzoia	Neonatal [0.44]	Diarrhea [1.26]	HIV [26.68]	LRI [0.39]	Congenital [0.37]	PEM [1.01]	Meningitis [0.44]	iNTS [5.7]	Whooping [1.13]	Malaria [34.63]
Turkana	Neonatal [0.3]	Diarrhea [0.36]	HIV [16.81]	LRI [0.22]	Congenital [0.32]	Tetanus [2.83]	PEM [0.34]	Meningitis [0.22]	Malaria [0.31]	TB [0.33]
UasinGishu	Neonatal [0.69]	Diarrhea [2.24]	LRI [0.58]	Congenital [0.54]	HIV [13.01]	Meningitis [0.63]	PEM [1.71]	Dermatitis [1.64]	iNTS [6.04]	Vit A [1.0]
Vihiga	Neonatal [0.32]	Diarrhea [0.58]	LRI [0.28]	Congenital [0.36]	Malaria [44.31]	HIV [5.08]	Meningitis [0.39]	Road Inj [0.36]	Vit A [0.98]	iNTS [3.56]
Wajir	Neonatal [0.25]	Diarrhea [0.3]	LRI [0.18]	Congenital [0.27]	Tetanus [1.63]	Measles [0.1]	PEM [0.14]	Meningitis [0.17]	iNTS [1.59]	HIV [2.86]
WestPokot	Neonatal [0.41]	Diarrhea [0.76]	LRI [0.27]	Congenital [0.32]	HIV [4.74]	PEM [0.48]	Tetanus [6.13]	Leish [11.5]	Meningitis [0.25]	iNTS [3.14]
Madagascar	Neonatal [0.55]	Diarrhea [0.81]	LRI [0.49]	PEM [1.48]	Congenital [0.6]	Malaria [1.52]	STI [1.29]	Measles [0.32]	Meningitis [0.32]	Whooping [0.44]
Malawi	Neonatal [0.57]	HIV [29.59]	LRI [0.29]	Congenital [0.67]	Malaria [4.87]	Diarrhea [0.29]	STI [1.23]	PEM [0.46]	Meningitis [0.4]	Iron [1.19]

eFigure 7a. Leading ten causes of DALYs with the ratio of observed DALYs to DALYs expected on the basis of Socio-Demographic Index alone in 2017, <20 years, both sexes combined. The top ten causes contributing to DALYs are listed globally, by socio-demographic quintile, and then by GBD superregion, region, country, and subnationally where modeled. For each cell, the ratio of observed DALYs to DALYs expected on the basis of socio-demographic index (SDI) alone are listed. Abbreviations: DALY=disability-adjusted life year, GBD=Global Burden of Disease.

Values shown in brackets represent the ratio of observed DALYs to predicted DALYs on the basis of Socio-Demographic Index (SDI), rounded to two (2) digits. Color ranges (shown below) were calculated to place a roughly equal number of cells into each bin.

COLOR KEY:	[0.0-0.5]	(0.5-0.69]	(0.69-0.82]	(0.82-0.92]	(0.92-1.01]	(1.01-1.18]	(1.18-1.48]	(1.48-2.26]	2.26+

	1	2	3	4	5	6	7	8	9	10
Mozambique	HIV [42.86]	Neonatal [0.52]	Malaria [6.38]	Congenital [0.68]	LRI [0.22]	Diarrhea [0.25]	STI [1.06]	Meningitis [0.27]	TB [0.68]	Iron [0.96]
Rwanda	Neonatal [0.56]	LRI [0.39]	Congenital [0.75]	Diarrhea [0.43]	Malaria [11.56]	PEM [0.87]	Meningitis [0.44]	HIV [4.47]	Road Inj [0.6]	TB [0.72]
Somalia	Neonatal [0.4]	LRI [0.32]	Diarrhea [0.29]	Measles [0.53]	Whooping [1.84]	PEM [0.37]	Congenital [0.34]	STI [0.93]	Conflict Terror [43.35]	Meningitis [0.38]
S Sudan	Neonatal [0.65]	LRI [0.71]	Diarrhea [0.71]	Malaria [1.25]	PEM [0.73]	Meningitis [0.68]	Congenital [0.46]	Conflict Terror [92.22]	STI [1.41]	Measles [0.32]
Tanzania	Neonatal [0.72]	LRI [0.54]	Congenital [1.14]	Malaria [21.49]	Diarrhea [0.29]	HIV [8.53]	PEM [0.91]	STI [1.72]	Meningitis [0.41]	Iron [1.22]
Uganda	Neonatal [0.65]	Malaria [25.21]	HIV [18.15]	Congenital [0.65]	LRI [0.25]	Diarrhea [0.33]	STI [2.13]	Meningitis [0.51]	PEM [0.51]	Whooping [0.62]
Zambia	Neonatal [0.8]	HIV [43.79]	LRI [0.62]	Diarrhea [1.04]	Congenital [0.91]	Malaria [117.17]	PEM [2.06]	Meningitis [0.77]	STI [1.92]	TB [1.7]
**Southern Sub-Saharan Africa**	Neonatal [1.32]	HIV [80.72]	LRI [1.82]	Diarrhea [5.49]	Congenital [0.44]	PEM [15.24]	TB [15.23]	Road Inj [0.79]	STI [7.77]	Violence [2.09]
Botswana	Neonatal [1.11]	Diarrhea [6.44]	LRI [0.99]	HIV [22.65]	Road Inj [0.83]	Congenital [0.27]	Iron [2.34]	PEM [10.1]	Dermatitis [1.28]	TB [10.1]
Lesotho	Neonatal [0.89]	HIV [61.79]	Diarrhea [1.9]	LRI [0.75]	TB [4.3]	Congenital [0.37]	Road Inj [1.29]	PEM [1.78]	Violence [2.97]	STI [1.65]
Namibia	Neonatal [1.27]	HIV [85.18]	Diarrhea [4.71]	LRI [1.1]	Congenital [0.35]	Road Inj [0.9]	PEM [6.82]	TB [8.82]	Iron [1.75]	Violence [1.72]
S Africa	Neonatal [1.28]	HIV [109.77]	Diarrhea [6.98]	LRI [1.67]	Congenital [0.47]	STI [14.07]	Road Inj [0.84]	Violence [2.8]	PEM [14.55]	TB [14.9]
Swaziland	HIV [80.03]	Neonatal [0.91]	Diarrhea [4.5]	LRI [1.23]	Congenital [0.39]	Road Inj [1.29]	PEM [4.91]	TB [7.66]	Violence [2.07]	Meningitis [0.68]
Zimbabwe	Neonatal [0.85]	LRI [0.89]	HIV [20.02]	Diarrhea [0.76]	PEM [2.66]	TB [3.45]	Congenital [0.34]	Meningitis [0.5]	Malaria [23.24]	Road Inj [0.46]
**Western Sub-Saharan Africa**	Neonatal [1.0]	Malaria [162.53]	LRI [1.06]	Diarrhea [1.46]	Congenital [0.66]	Meningitis [1.37]	PEM [1.53]	HIV [7.1]	Iron [1.73]	Measles [1.57]
Benin	Neonatal [0.79]	Malaria [23.67]	LRI [0.47]	Diarrhea [0.61]	Congenital [0.63]	Meningitis [0.56]	PEM [0.71]	Road Inj [0.87]	Measles [0.55]	iNTS [4.54]
Burkina Faso	Malaria [5.99]	Neonatal [0.47]	LRI [0.47]	Diarrhea [0.36]	Congenital [0.81]	Meningitis [0.71]	PEM [0.54]	iNTS [5.5]	Hemog [3.33]	Iron [1.56]
Cameroon	Neonatal [0.73]	Malaria [457.76]	Diarrhea [1.48]	LRI [0.6]	Congenital [0.56]	HIV [11.8]	Meningitis [0.68]	STI [2.42]	PEM [1.2]	Iron [1.13]
Cape Verde	Neonatal [0.73]	Congenital [0.41]	LRI [0.23]	Iron [1.37]	Diarrhea [0.31]	Violence [1.44]	HIV [3.81]	Headaches [1.01]	Drown [0.47]	Epilepsy [1.02]
Chad	Diarrhea [0.95]	Neonatal [0.6]	LRI [0.63]	Malaria [1.06]	PEM [0.62]	Meningitis [0.67]	Congenital [0.46]	Iron [1.6]	Measles [0.18]	Whooping [0.57]
Cote d'Ivoire	Neonatal [0.93]	Malaria [48.61]	LRI [0.61]	Diarrhea [0.85]	Congenital [0.79]	HIV [8.55]	Meningitis [0.6]	PEM [0.88]	Iron [1.42]	iNTS [6.06]
Gambia	Neonatal [0.65]	LRI [0.31]	Diarrhea [0.39]	Congenital [0.42]	Hemog [6.72]	Iron [1.73]	Meningitis [0.47]	PEM [0.65]	STI [1.44]	HIV [3.92]
Ghana	Neonatal [1.25]	Malaria [1273.21]	Diarrhea [1.38]	LRI [0.53]	Congenital [0.66]	HIV [10.24]	Meningitis [0.99]	Iron [2.11]	PEM [2.28]	STI [3.23]
Guinea	Neonatal [0.65]	Malaria [8.24]	LRI [0.58]	Diarrhea [0.33]	Congenital [0.65]	PEM [0.72]	Meningitis [0.63]	Whooping [0.94]	iNTS [4.76]	Measles [0.29]
Guinea-Bissau	Neonatal [0.78]	Diarrhea [0.67]	LRI [0.31]	Congenital [0.61]	Measles [1.15]	HIV [8.75]	Hemog [6.01]	STI [1.8]	Meningitis [0.54]	PEM [0.52]
Liberia	Neonatal [0.44]	Malaria [4.81]	Diarrhea [0.4]	LRI [0.25]	STI [2.09]	Congenital [0.44]	Measles [0.65]	Whooping [0.78]	Meningitis [0.36]	PEM [0.34]
Mali	Neonatal [0.76]	Malaria [3.78]	Diarrhea [0.37]	LRI [0.32]	Congenital [0.78]	PEM [0.81]	Meningitis [0.72]	iNTS [6.26]	STI [1.14]	Whooping [0.71]
Mauritania	Neonatal [0.77]	Diarrhea [0.83]	LRI [0.42]	Congenital [0.41]	PEM [1.28]	Iron [1.42]	STI [1.88]	Meningitis [0.44]	Road Inj [0.62]	Vit A [1.23]
Niger	Malaria [1.17]	Diarrhea [0.4]	Neonatal [0.33]	LRI [0.35]	Meningitis [0.83]	Congenital [0.42]	PEM [0.17]	iNTS [2.1]	Measles [0.09]	Whooping [0.46]
Nigeria	Neonatal [1.35]	LRI [1.92]	Malaria [788.81]	Diarrhea [2.79]	Meningitis [2.39]	Congenital [0.65]	PEM [2.8]	Measles [6.62]	HIV [11.89]	Iron [2.24]

eFigure 7a. Leading ten causes of DALYs with the ratio of observed DALYs to DALYs expected on the basis of Socio-Demographic Index alone in 2017, <20 years, both sexes combined. The top ten causes contributing to DALYs are listed globally, by socio-demographic quintile, and then by GBD superregion, region, country, and subnationally where modeled. For each cell, the ratio of observed DALYs to DALYs expected on the basis of socio-demographic index (SDI) alone are listed. Abbreviations: DALY=disability-adjusted life year, GBD=Global Burden of Disease.

Values shown in brackets represent the ratio of observed DALYs to predicted DALYs on the basis of Socio-Demographic Index (SDI), rounded to two (2) digits. Color ranges (shown below) were calculated to place a roughly equal number of cells into each bin.

COLOR KEY:	[0.0-0.5]	(0.5-0.69]	(0.69-0.82]	(0.82-0.92]	(0.92-1.01]	(1.01-1.18]	(1.18-1.48]	(1.48-2.26]	2.26+

	1	2	3	4	5	6	7	8	9	10
Sao Tome Principe	**Neonatal** [0.52]	**LRI** [0.33]	**Congenital** [0.48]	**Diarrhea** [0.33]	**Other MN** [3.58]	**PEM** [0.83]	**Iron** [0.94]	**Vit A** [1.29]	**Road Inj** [0.42]	**Oth NTD** [3.07]
Senegal	**Neonatal** [0.55]	**Diarrhea** [0.5]	**LRI** [0.31]	**Congenital** [0.45]	**Iron** [1.48]	**Meningitis** [0.42]	**Malaria** [2.55]	**Hemog** [2.29]	**PEM** [0.31]	**Vit A** [0.94]
Sierra Leone	**Malaria** [28.23]	**Neonatal** [0.83]	**LRI** [0.65]	**Diarrhea** [0.56]	**Congenital** [0.95]	**PEM** [1.05]	**Meningitis** [0.77]	**STI** [1.12]	**Iron** [1.4]	**iNTS** [4.43]
Togo	**Neonatal** [0.67]	**Malaria** [75.86]	**Diarrhea** [0.67]	**LRI** [0.33]	**Congenital** [0.47]	**HIV** [6.77]	**iNTS** [6.43]	**Hemog** [3.38]	**Iron** [1.16]	**Meningitis** [0.35]

eFigure 7b. Leading ten causes of DALYs with the ratio of observed DALYs to DALYs expected on the basis of Socio-Demographic Index alone in 2017, <1 years, both sexes combined. The top ten causes contributing to DALYs are listed globally, by socio-demographic quintile, and then by GBD superregion, region, country, and subnationally where modeled. For each cell, the ratio of observed DALYs to DALYs expected on the basis of socio-demographic index (SDI) alone are listed. Abbreviations: DALY=disability-adjusted life year, GBD=Global Burden of Disease.

Values shown in brackets represent the ratio of observed DALYs to predicted DALYs on the basis of Socio-Demographic Index (SDI), rounded to two (2) digits. Color ranges (shown below) were calculated to place a roughly equal number of cells into each bin.

COLOR KEY:									
[0.0-0.33]	(0.33-0.46]	(0.46-0.58]	(0.58-0.7]	(0.7-0.84]	(0.84-1.03]	(1.03-1.38]	(1.38-2.46]	2.46+	

	1	2	3	4	5	6	7	8	9	10
Global	Neonatal [1.31]	LRI [1.77]	Congenital [0.71]	Diarrhea [4.95]	Malaria [53940.25]	STI [6.21]	Meningitis [1.38]	PEM [6.73]	HIV [8.34]	Whooping [7.53]
Low SDI	Neonatal [0.76]	LRI [0.61]	Diarrhea [0.69]	Congenital [0.67]	Malaria [37.06]	STI [1.13]	Meningitis [0.5]	PEM [0.75]	Whooping [0.8]	HIV [4.73]
Low-middle SDI	Neonatal [1.06]	LRI [0.95]	Congenital [0.64]	Diarrhea [1.52]	Malaria [2292.63]	Meningitis [0.91]	STI [2.04]	PEM [1.34]	HIV [6.54]	Whooping [1.42]
Middle SDI	Neonatal [0.7]	Congenital [0.55]	LRI [0.83]	Diarrhea [1.47]	STI [3.89]	F Body [0.68]	Meningitis [0.39]	PEM [2.4]	HIV [3.39]	Ileus [1.19]
High-middle SDI	Neonatal [1.51]	Congenital [1.17]	LRI [5.01]	F Body [2.14]	Diarrhea [4.98]	STI [16.42]	SIDS [0.31]	Meningitis [1.42]	Endocrine [0.87]	Ileus [3.89]
High SDI	Neonatal [0.37]	Congenital [0.35]	SIDS [0.68]	F Body [0.57]	LRI [0.14]	Diarrhea [0.45]	Endocrine [0.47]	Violence [0.82]	STI [0.75]	Oth Un Inf [0.54]
Central Europe, Eastern Europe, and Central Asia	Neonatal [1.02]	LRI [4.06]	Congenital [0.65]	Diarrhea [3.47]	F Body [0.9]	SIDS [0.36]	Oth Un Inf [2.68]	Meningitis [0.59]	Encepha [3.49]	HIV [4.88]
Central Asia	Neonatal [1.11]	LRI [3.09]	Congenital [0.62]	Diarrhea [2.55]	F Body [0.67]	Encepha [2.95]	Ileus [1.85]	Meningitis [0.4]	SIDS [0.38]	TB [4.7]
Armenia	Neonatal [0.5]	Congenital [0.57]	LRI [1.09]	Ileus [2.09]	Diarrhea [0.54]	Iron [3.88]	F Body [0.3]	SIDS [0.19]	Oth Un Inf [0.56]	HIV [0.97]
Azerbaijan	Neonatal [1.89]	LRI [7.22]	Congenital [1.23]	Diarrhea [3.62]	Encepha [8.11]	Endocrine [2.13]	TB [13.15]	Ileus [2.68]	F Body [0.6]	Oth Un Inf [2.58]
Georgia	Neonatal [0.91]	Congenital [0.37]	LRI [0.39]	Diarrhea [0.51]	Ileus [1.19]	Oth Un Inf [1.05]	F Body [0.18]	Meningitis [0.18]	Med Treat [0.86]	STI [0.57]
Kazakhstan	Neonatal [0.97]	Congenital [0.76]	LRI [1.62]	F Body [1.68]	SIDS [0.59]	Encepha [4.07]	Diarrhea [0.94]	Oth Un Inf [2.53]	Ileus [2.51]	Meningitis [0.62]
Kyrgyzstan	Neonatal [0.97]	LRI [0.57]	Congenital [0.42]	Diarrhea [0.58]	F Body [0.47]	SIDS [0.37]	HIV [1.94]	Meningitis [0.14]	Oth Un Inf [0.79]	Iron [1.94]
Mongolia	Neonatal [1.33]	LRI [2.09]	Congenital [0.65]	F Body [1.34]	Diarrhea [0.78]	STI [2.73]	Encepha [3.52]	Ileus [2.16]	TB [5.03]	SIDS [0.36]
Tajikistan	Neonatal [0.75]	LRI [1.4]	Diarrhea [1.72]	Congenital [0.65]	Meningitis [0.36]	Ileus [1.5]	TB [1.18]	Whooping [0.82]	SIDS [0.47]	PEM [0.33]
Turkmenistan	Neonatal [1.35]	LRI [3.44]	Congenital [0.99]	Diarrhea [1.6]	Ileus [4.04]	F Body [0.72]	Encepha [2.98]	TB [9.21]	Meningitis [0.48]	SIDS [0.32]
Uzbekistan	Neonatal [0.73]	LRI [2.23]	Congenital [0.32]	Encepha [2.95]	Diarrhea [0.29]	F Body [0.42]	Ileus [0.91]	URI [5.88]	Oth Un Inf [1.05]	TB [1.92]
Central Europe	Neonatal [0.65]	Congenital [0.58]	LRI [1.87]	SIDS [0.24]	Diarrhea [1.49]	F Body [0.55]	Meningitis [0.47]	Endocrine [0.42]	Vit A [3.35]	Ileus [0.98]
Albania	Neonatal [0.64]	Congenital [0.7]	LRI [0.9]	SIDS [0.51]	Oth Cardio [3.66]	F Body [0.34]	Meningitis [0.28]	Ileus [1.18]	CMP [1.36]	Stroke [2.4]
Bosnia	Neonatal [0.46]	Congenital [0.52]	LRI [0.16]	Whooping [5.73]	Diarrhea [0.46]	SIDS [0.25]	Meningitis [0.27]	Vit A [1.54]	Stroke [1.28]	Med Treat [0.83]
Bulgaria	Neonatal [0.68]	Congenital [0.68]	LRI [2.09]	Diarrhea [1.9]	F Body [0.8]	Meningitis [0.71]	Endocrine [0.52]	Ileus [1.56]	Encepha [2.73]	SIDS [0.14]
Croatia	Neonatal [0.59]	Congenital [0.53]	SIDS [0.44]	LRI [0.31]	Diarrhea [1.18]	Endocrine [0.42]	F Body [0.24]	Meningitis [0.3]	Ileus [0.72]	Oth Neuro [0.4]
Czech	Neonatal [0.39]	Congenital [0.28]	SIDS [0.32]	LRI [0.63]	F Body [0.67]	Diarrhea [1.4]	Endocrine [0.55]	Meningitis [0.38]	Oth Un Inf [0.71]	Oth Neuro [0.42]
Hungary	Neonatal [0.59]	Congenital [0.51]	LRI [0.71]	SIDS [0.38]	Endocrine [0.73]	Diarrhea [1.24]	F Body [0.41]	Meningitis [0.48]	Oth Un Inf [0.77]	Oth Neuro [0.55]
Macedonia	Neonatal [1.03]	Congenital [0.47]	LRI [0.77]	Diarrhea [1.23]	SIDS [0.39]	Whooping [10.12]	Meningitis [0.52]	Vit A [2.62]	Endocrine [0.35]	F Body [0.18]
Montenegro	Neonatal [0.55]	Congenital [0.16]	LRI [0.36]	SIDS [0.25]	Whooping [9.81]	F Body [0.36]	Diarrhea [0.51]	Vit A [2.27]	Endocrine [0.23]	Violence [0.61]
Poland	Neonatal [0.67]	Congenital [0.66]	LRI [0.7]	SIDS [0.23]	Diarrhea [1.04]	F Body [0.38]	Endocrine [0.35]	Meningitis [0.4]	Vit A [3.56]	Ileus [0.84]
Romania	Neonatal [0.61]	LRI [4.3]	Congenital [0.66]	Diarrhea [2.32]	F Body [1.05]	Oth Un Inf [1.7]	STI [3.08]	Meningitis [0.44]	Vit A [3.64]	Ileus [1.2]
Serbia	Neonatal [0.53]	Congenital [0.32]	LRI [0.26]	SIDS [0.24]	Diarrhea [0.43]	Whooping [4.4]	Vit A [2.08]	Endocrine [0.32]	Meningitis [0.18]	F Body [0.15]
Slovakia	Neonatal [0.78]	Congenital [0.71]	LRI [1.92]	Diarrhea [2.17]	SIDS [0.25]	F Body [0.76]	Meningitis [0.98]	Endocrine [0.58]	Ileus [2.02]	Oth Neuro [0.91]
Slovenia	Neonatal [0.34]	Congenital [0.33]	SIDS [0.11]	Endocrine [0.48]	LRI [0.27]	Diarrhea [1.11]	Oth Neuro [0.59]	Vit A [3.38]	Hernia [1.0]	Iron [3.33]

eFigure 7b. Leading ten causes of DALYs with the ratio of observed DALYs to DALYs expected on the basis of Socio-Demographic Index alone in 2017, <1 years, both sexes combined. The top ten causes contributing to DALYs are listed globally, by socio-demographic quintile, and then by GBD superregion, region, country, and subnationally where modeled. For each cell, the ratio of observed DALYs to DALYs expected on the basis of socio-demographic index (SDI) alone are listed. Abbreviations: DALY=disability-adjusted life year, GBD=Global Burden of Disease.

Values shown in brackets represent the ratio of observed DALYs to predicted DALYs on the basis of Socio-Demographic Index (SDI), rounded to two (2) digits. Color ranges (shown below) were calculated to place a roughly equal number of cells into each bin.

COLOR KEY:	[0.0-0.33]	(0.33-0.46]	(0.46-0.58]	(0.58-0.7]	(0.7-0.84]	(0.84-1.03]	(1.03-1.38]	(1.38-2.46]	2.46+

	1	2	3	4	5	6	7	8	9	10
**Eastern Europe**	Neonatal [0.64]	Congenital [0.59]	LRI [1.09]	Oth Un Inf [4.7]	F Body [1.06]	HIV [11.88]	SIDS [0.41]	Diarrhea [0.99]	Meningitis [0.54]	Endocrine [0.49]
Belarus	Neonatal [0.52]	Congenital [0.53]	F Body [1.11]	LRI [0.39]	SIDS [0.4]	Encepha [4.74]	Oth Un Inf [2.2]	Ileus [1.43]	Meningitis [0.39]	Endocrine [0.38]
Estonia	Neonatal [0.36]	Congenital [0.35]	LRI [1.4]	F Body [1.04]	SIDS [0.2]	Diarrhea [1.6]	Oth Un Inf [2.01]	Oth Neuro [0.72]	Endocrine [0.35]	Meningitis [0.44]
Latvia	Neonatal [0.51]	Congenital [0.46]	SIDS [1.02]	LRI [0.98]	Oth Un Inf [2.86]	Diarrhea [1.24]	F Body [0.45]	Meningitis [0.49]	Endocrine [0.32]	Fire [2.46]
Lithuania	Neonatal [0.47]	Congenital [0.71]	LRI [1.62]	F Body [1.51]	SIDS [0.47]	Oth Un Inf [2.27]	Diarrhea [1.51]	Meningitis [0.75]	Endocrine [0.47]	Ileus [1.82]
Moldova	Neonatal [0.72]	Congenital [1.04]	LRI [1.32]	F Body [1.26]	SIDS [0.63]	Oth Un Inf [2.16]	Diarrhea [0.44]	Meningitis [0.26]	Violence [1.18]	Iron [1.68]
Russian Federation	Neonatal [0.63]	Congenital [0.53]	LRI [1.2]	HIV [16.37]	Oth Un Inf [5.07]	SIDS [0.42]	F Body [0.94]	Diarrhea [1.19]	Meningitis [0.46]	Endocrine [0.4]
Ukraine	Neonatal [0.59]	Congenital [0.66]	LRI [0.46]	F Body [1.15]	Oth Un Inf [3.86]	Meningitis [0.62]	SIDS [0.33]	Endocrine [0.78]	Encepha [2.24]	Diarrhea [0.33]
**High-income**	Neonatal [0.83]	Congenital [0.63]	SIDS [0.77]	F Body [1.34]	LRI [0.76]	Diarrhea [1.93]	Endocrine [0.73]	Violence [1.34]	STI [3.54]	Meningitis [0.55]
**Australasia**	Neonatal [0.73]	Congenital [0.54]	SIDS [0.69]	LRI [0.72]	F Body [0.72]	Endocrine [0.75]	Diarrhea [1.34]	Oth Un Inf [1.29]	ALS [2.26]	Oth Neuro [0.64]
Australia	Neonatal [0.74]	Congenital [0.55]	SIDS [0.66]	LRI [0.75]	Endocrine [0.79]	Diarrhea [1.43]	F Body [0.46]	ALS [2.44]	Oth Un Inf [1.36]	Oth Neuro [0.67]
New Zealand	Neonatal [0.67]	Congenital [0.48]	SIDS [0.82]	F Body [1.69]	LRI [0.55]	Endocrine [0.57]	Diarrhea [0.91]	Meningitis [0.51]	Oth Un Inf [0.96]	Violence [0.79]
**High-income Asia Pacific**	Neonatal [0.36]	Congenital [0.44]	SIDS [0.29]	F Body [0.97]	LRI [0.56]	Diarrhea [1.03]	Endocrine [0.36]	CMP [0.76]	URI [1.02]	Violence [0.61]
Brunei	Neonatal [1.15]	Congenital [1.4]	LRI [3.81]	Endocrine [2.73]	F Body [2.09]	SIDS [0.44]	CMP [3.84]	Meningitis [1.26]	Road Inj [2.3]	Oth Un Inf [2.11]
Japan	Congenital [0.45]	Neonatal [0.24]	SIDS [0.28]	F Body [0.93]	LRI [0.52]	Diarrhea [1.06]	Endocrine [0.34]	CMP [0.69]	URI [1.02]	Violence [0.6]
Aichi	Congenital [0.46]	Neonatal [0.24]	SIDS [0.26]	F Body [0.95]	LRI [0.56]	Diarrhea [1.23]	Endocrine [0.36]	ASD [2.04]	CMP [0.76]	URI [1.03]
Akita	Congenital [0.35]	Neonatal [0.18]	F Body [1.05]	SIDS [0.25]	LRI [0.32]	Diarrhea [0.83]	Endocrine [0.31]	CMP [0.57]	URI [0.99]	ASD [1.31]
Aomori	Congenital [0.37]	Neonatal [0.23]	F Body [0.74]	SIDS [0.26]	LRI [0.31]	Diarrhea [0.69]	Endocrine [0.28]	CMP [0.53]	URI [0.98]	ASD [1.31]
Chiba	Congenital [0.53]	Neonatal [0.27]	SIDS [0.32]	F Body [0.99]	LRI [0.55]	Diarrhea [1.04]	Endocrine [0.37]	CMP [0.8]	Violence [0.69]	URI [1.02]
Ehime	Congenital [0.31]	Neonatal [0.17]	SIDS [0.25]	F Body [0.78]	LRI [0.29]	Diarrhea [0.71]	Endocrine [0.28]	URI [0.99]	CMP [0.48]	ASD [1.34]
Fukui	Congenital [0.35]	Neonatal [0.19]	F Body [0.78]	SIDS [0.21]	LRI [0.32]	Diarrhea [1.08]	Endocrine [0.32]	URI [1.0]	ASD [1.35]	CMP [0.48]
Fukuoka	Congenital [0.41]	Neonatal [0.24]	SIDS [0.35]	F Body [0.77]	LRI [0.43]	Diarrhea [0.94]	Endocrine [0.33]	Violence [0.67]	URI [1.01]	CMP [0.55]
Fukushima	Congenital [0.37]	Neonatal [0.19]	F Body [0.9]	SIDS [0.3]	LRI [0.32]	Diarrhea [0.69]	Endocrine [0.32]	CMP [0.64]	URI [0.99]	ASD [1.32]
Gifu	Congenital [0.45]	Neonatal [0.24]	F Body [0.99]	SIDS [0.27]	LRI [0.38]	Diarrhea [1.02]	Endocrine [0.34]	CMP [0.66]	URI [1.0]	ASD [1.34]
Gunma	Congenital [0.41]	Neonatal [0.23]	F Body [0.7]	SIDS [0.2]	LRI [0.39]	Diarrhea [0.8]	Endocrine [0.3]	CMP [0.6]	URI [1.01]	Violence [0.57]
Hiroshima	Congenital [0.4]	Neonatal [0.22]	F Body [0.97]	SIDS [0.25]	LRI [0.46]	Diarrhea [1.04]	Endocrine [0.31]	CMP [0.62]	URI [1.02]	Violence [0.59]
Hokkaido	Congenital [0.4]	Neonatal [0.22]	SIDS [0.36]	F Body [0.7]	LRI [0.36]	Diarrhea [0.76]	Endocrine [0.31]	CMP [0.56]	Violence [0.54]	URI [0.99]
Hyogo	Congenital [0.35]	Neonatal [0.21]	SIDS [0.26]	F Body [0.88]	LRI [0.42]	Diarrhea [1.08]	Endocrine [0.32]	CMP [0.62]	URI [1.01]	Violence [0.57]
Ibaraki	Congenital [0.51]	Neonatal [0.27]	F Body [0.97]	SIDS [0.26]	LRI [0.46]	Diarrhea [0.99]	Endocrine [0.36]	CMP [0.75]	Violence [0.61]	URI [1.01]
Ishikawa	Congenital [0.39]	Neonatal [0.21]	F Body [0.93]	SIDS [0.17]	LRI [0.4]	Diarrhea [0.96]	Endocrine [0.3]	URI [1.01]	ASD [1.34]	CMP [0.5]
Iwate	Congenital [0.39]	Neonatal [0.21]	F Body [0.98]	SIDS [0.24]	LRI [0.32]	Diarrhea [0.68]	Endocrine [0.32]	CMP [0.53]	URI [0.98]	ASD [1.31]

eFigure 7b. Leading ten causes of DALYs with the ratio of observed DALYs to DALYs expected on the basis of Socio-Demographic Index alone in 2017, <1 years, both sexes combined. The top ten causes contributing to DALYs are listed globally, by socio-demographic quintile, and then by GBD superregion, region, country, and subnationally where modeled. For each cell, the ratio of observed DALYs to DALYs expected on the basis of socio-demographic index (SDI) alone are listed. Abbreviations: DALY=disability-adjusted life year, GBD=Global Burden of Disease.

Values shown in brackets represent the ratio of observed DALYs to predicted DALYs on the basis of Socio-Demographic Index (SDI), rounded to two (2) digits. Color ranges (shown below) were calculated to place a roughly equal number of cells into each bin.

COLOR KEY:	[0.0-0.33]	(0.33-0.46]	(0.46-0.58]	(0.58-0.7]	(0.7-0.84]	(0.84-1.03]	(1.03-1.38]	(1.38-2.46]	2.46+

	1	2	3	4	5	6	7	8	9	10
Kagawa	Congenital [0.37]	Neonatal [0.19]	SIDS [0.32]	F Body [0.96]	LRI [0.4]	Diarrhea [1.04]	Endocrine [0.3]	URI [1.0]	Violence [0.57]	CMP [0.54]
Kagoshima	Congenital [0.36]	Neonatal [0.19]	F Body [0.84]	LRI [0.37]	SIDS [0.23]	Diarrhea [0.94]	Endocrine [0.33]	CMP [0.61]	URI [1.0]	Violence [0.5]
Kanagawa	Congenital [0.51]	Neonatal [0.27]	SIDS [0.34]	F Body [0.96]	LRI [0.6]	Diarrhea [0.99]	Endocrine [0.34]	Violence [0.8]	CMP [0.74]	ASD [1.65]
Kochi	Congenital [0.37]	Neonatal [0.22]	F Body [1.0]	SIDS [0.33]	LRI [0.33]	Diarrhea [0.8]	Endocrine [0.33]	CMP [0.63]	Violence [0.61]	URI [0.98]
Kumamoto	Congenital [0.29]	Neonatal [0.14]	SIDS [0.24]	F Body [0.56]	LRI [0.25]	Diarrhea [0.68]	Endocrine [0.28]	URI [0.98]	ASD [1.32]	CMP [0.42]
Kyoto	Congenital [0.54]	Neonatal [0.29]	SIDS [0.3]	F Body [0.96]	LRI [0.64]	Diarrhea [1.22]	Endocrine [0.37]	CMP [0.72]	Violence [0.66]	URI [1.02]
Mie	Congenital [0.42]	Neonatal [0.27]	F Body [1.33]	SIDS [0.34]	LRI [0.47]	Diarrhea [1.35]	Endocrine [0.37]	CMP [0.77]	Violence [0.63]	URI [1.02]
Miyagi	Congenital [0.44]	Neonatal [0.23]	SIDS [0.34]	F Body [0.91]	LRI [0.4]	Diarrhea [0.85]	Endocrine [0.33]	CMP [0.72]	URI [1.01]	Violence [0.54]
Miyazaki	Congenital [0.34]	Neonatal [0.18]	SIDS [0.33]	F Body [0.83]	LRI [0.32]	Diarrhea [0.72]	Endocrine [0.32]	CMP [0.59]	URI [0.98]	Violence [0.46]
Nagano	Congenital [0.3]	Neonatal [0.18]	SIDS [0.22]	F Body [0.67]	LRI [0.24]	Diarrhea [0.73]	Endocrine [0.26]	URI [1.01]	ASD [1.34]	CMP [0.45]
Nagasaki	Congenital [0.33]	Neonatal [0.17]	SIDS [0.23]	F Body [0.57]	LRI [0.26]	Diarrhea [0.62]	Endocrine [0.27]	Vit A [2.26]	URI [0.98]	ASD [1.31]
Nara	Congenital [0.43]	Neonatal [0.21]	F Body [0.93]	SIDS [0.25]	LRI [0.45]	Diarrhea [0.95]	Endocrine [0.33]	CMP [0.67]	Violence [0.62]	URI [1.01]
Niigata	Congenital [0.38]	Neonatal [0.19]	F Body [0.82]	SIDS [0.21]	LRI [0.29]	Diarrhea [0.79]	Endocrine [0.28]	URI [1.0]	CMP [0.51]	ASD [1.33]
Oita	Congenital [0.39]	Neonatal [0.23]	SIDS [0.24]	F Body [0.73]	LRI [0.35]	Diarrhea [1.04]	Endocrine [0.31]	CMP [0.57]	Violence [0.57]	URI [1.0]
Okayama	Congenital [0.35]	Neonatal [0.18]	F Body [0.84]	SIDS [0.23]	LRI [0.43]	Diarrhea [0.95]	Endocrine [0.29]	URI [1.0]	CMP [0.56]	ASD [1.34]
Okinawa	Congenital [0.28]	Neonatal [0.17]	SIDS [0.31]	F Body [0.54]	LRI [0.24]	Diarrhea [0.83]	Endocrine [0.33]	Violence [0.57]	Vit A [2.03]	URI [0.98]
Osaka	Congenital [0.43]	Neonatal [0.23]	F Body [0.95]	LRI [0.65]	SIDS [0.17]	Diarrhea [1.09]	Endocrine [0.35]	Violence [0.83]	CMP [0.74]	URI [1.02]
Saga	Congenital [0.26]	Neonatal [0.16]	F Body [0.67]	SIDS [0.21]	LRI [0.28]	Diarrhea [0.69]	Endocrine [0.26]	Vit A [2.52]	URI [0.99]	ASD [1.31]
Saitama	Congenital [0.44]	Neonatal [0.21]	SIDS [0.28]	F Body [0.69]	LRI [0.42]	Diarrhea [0.83]	Endocrine [0.32]	CMP [0.69]	URI [1.01]	Violence [0.52]
Shiga	Congenital [0.44]	Neonatal [0.25]	F Body [1.06]	SIDS [0.23]	LRI [0.48]	Diarrhea [1.34]	Endocrine [0.33]	URI [1.02]	CMP [0.61]	ASD [1.38]
Shimane	Congenital [0.3]	Neonatal [0.16]	SIDS [0.35]	F Body [0.67]	LRI [0.23]	Diarrhea [0.67]	Endocrine [0.27]	URI [0.98]	Iron [3.03]	ASD [1.32]
Shizuoka	Congenital [0.41]	Neonatal [0.22]	SIDS [0.29]	F Body [0.98]	LRI [0.39]	Diarrhea [0.95]	Endocrine [0.34]	Iron [5.34]	CMP [0.61]	URI [1.01]
Tochigi	Congenital [0.47]	Neonatal [0.24]	SIDS [0.28]	F Body [0.87]	LRI [0.5]	Diarrhea [0.99]	Endocrine [0.33]	CMP [0.7]	Iron [4.45]	URI [1.02]
Tokushima	Congenital [0.59]	Neonatal [0.34]	F Body [1.34]	LRI [0.64]	SIDS [0.31]	Diarrhea [1.34]	Endocrine [0.41]	CMP [0.84]	Violence [0.84]	Ileus [0.9]
Tokyo	Congenital [0.73]	Neonatal [0.37]	SIDS [0.29]	LRI [1.54]	F Body [1.27]	Diarrhea [1.68]	Endocrine [0.45]	CMP [1.17]	URI [1.08]	Violence [0.77]
Tottori	Congenital [0.41]	Neonatal [0.23]	SIDS [0.35]	F Body [0.81]	LRI [0.28]	Diarrhea [0.82]	Endocrine [0.35]	URI [0.98]	Violence [0.5]	CMP [0.48]
Toyama	Congenital [0.45]	Neonatal [0.23]	F Body [1.22]	SIDS [0.22]	LRI [0.54]	Diarrhea [1.15]	Endocrine [0.34]	CMP [0.61]	URI [1.02]	ASD [1.36]
Wakayama	Congenital [0.39]	Neonatal [0.21]	F Body [0.8]	SIDS [0.25]	LRI [0.31]	Diarrhea [0.76]	Endocrine [0.31]	Vit A [2.83]	Violence [0.62]	CMP [0.58]
Yamagata	Congenital [0.43]	Neonatal [0.24]	F Body [0.96]	SIDS [0.29]	LRI [0.32]	Diarrhea [0.74]	Endocrine [0.32]	Vit A [2.82]	CMP [0.61]	URI [0.99]
Yamaguchi	Congenital [0.4]	Neonatal [0.23]	SIDS [0.27]	F Body [0.86]	LRI [0.48]	Diarrhea [0.95]	Endocrine [0.35]	CMP [0.65]	Violence [0.65]	URI [1.0]
Yamanashi	Congenital [0.39]	Neonatal [0.21]	F Body [0.85]	SIDS [0.24]	LRI [0.38]	Diarrhea [1.0]	Endocrine [0.31]	CMP [0.58]	URI [1.01]	ASD [1.34]

eFigure 7b. Leading ten causes of DALYs with the ratio of observed DALYs to DALYs expected on the basis of Socio-Demographic Index alone in 2017, <1 years, both sexes combined. The top ten causes contributing to DALYs are listed globally, by socio-demographic quintile, and then by GBD superregion, region, country, and subnationally where modeled. For each cell, the ratio of observed DALYs to DALYs expected on the basis of socio-demographic index (SDI) alone are listed. Abbreviations: DALY=disability-adjusted life year, GBD=Global Burden of Disease.

Values shown in brackets represent the ratio of observed DALYs to predicted DALYs on the basis of Socio-Demographic Index (SDI), rounded to two (2) digits. Color ranges (shown below) were calculated to place a roughly equal number of cells into each bin.

COLOR KEY:	[0.0-0.33]	(0.33-0.46]	(0.46-0.58]	(0.58-0.7]	(0.7-0.84]	(0.84-1.03]	(1.03-1.38]	(1.38-2.46]	2.46+

	1	2	3	4	5	6	7	8	9	10
S Korea	Neonatal [0.62]	Congenital [0.37]	SIDS [0.34]	F Body [1.05]	LRI [0.39]	Endocrine [0.38]	Diarrhea [0.93]	Falls [1.44]	Road Inj [0.92]	Iron [6.39]
Singapore	Congenital [0.31]	Neonatal [0.2]	LRI [1.08]	Iron [9.47]	SIDS [0.08]	CMP [0.85]	Endocrine [0.23]	F Body [0.19]	URI [0.99]	Falls [1.1]
**High-income North America**	Neonatal [1.16]	Congenital [0.77]	SIDS [1.28]	F Body [2.55]	LRI [0.98]	Diarrhea [2.97]	Violence [2.93]	Endocrine [0.83]	Oth Un Inf [1.78]	CMP [1.57]
Canada	Neonatal [1.05]	Congenital [0.77]	SIDS [0.81]	F Body [1.19]	LRI [0.85]	Endocrine [1.15]	Diarrhea [1.8]	Oth Neuro [1.28]	Meningitis [0.99]	CKD [2.99]
Greenland	Neonatal [1.14]	Congenital [0.6]	SIDS [2.44]	LRI [0.7]	F Body [1.69]	Meningitis [1.54]	Diarrhea [2.08]	Violence [3.65]	Endocrine [0.92]	CMP [1.47]
USA	Neonatal [1.14]	Congenital [0.76]	SIDS [1.32]	F Body [2.59]	LRI [0.92]	Diarrhea [2.95]	Violence [3.08]	Endocrine [0.79]	Oth Un Inf [1.78]	CMP [1.57]
Alabama	Neonatal [1.37]	Congenital [0.91]	SIDS [2.27]	F Body [3.55]	LRI [1.02]	Diarrhea [3.91]	Violence [4.95]	Endocrine [0.92]	Oth Un Inf [2.29]	Road Inj [2.43]
Alaska	Neonatal [0.76]	Congenital [0.66]	SIDS [2.16]	F Body [5.18]	LRI [1.04]	Diarrhea [2.75]	Violence [3.02]	Endocrine [0.87]	Oth Un Inf [1.77]	CMP [1.62]
Arizona	Neonatal [0.79]	Congenital [0.67]	SIDS [1.0]	F Body [1.91]	LRI [0.69]	Diarrhea [2.88]	Violence [2.78]	Oth Un Inf [2.22]	Endocrine [0.73]	CMP [1.23]
Arkansas	Neonatal [0.96]	Congenital [0.8]	SIDS [2.84]	F Body [2.67]	LRI [0.87]	Violence [3.99]	Diarrhea [2.02]	Endocrine [0.8]	Road Inj [1.88]	Oth Un Inf [1.71]
California	Neonatal [0.91]	Congenital [0.67]	SIDS [1.02]	F Body [1.07]	LRI [0.67]	Violence [2.59]	Diarrhea [1.9]	Endocrine [0.66]	Oth Un Inf [1.5]	CMP [1.38]
Colorado	Neonatal [1.17]	Congenital [0.78]	SIDS [0.97]	F Body [2.51]	LRI [0.9]	Diarrhea [2.4]	Endocrine [0.71]	Violence [1.82]	Oth Un Inf [1.74]	Ileus [1.98]
Connecticut	Neonatal [1.41]	Congenital [0.63]	SIDS [0.98]	F Body [2.13]	Diarrhea [4.93]	LRI [1.38]	Endocrine [0.72]	Violence [1.89]	Oth Un Inf [1.73]	CMP [1.41]
Delaware	Neonatal [1.84]	Congenital [0.88]	SIDS [1.6]	F Body [2.56]	Diarrhea [5.56]	LRI [1.29]	Endocrine [1.23]	Violence [2.7]	Oth Un Inf [2.13]	CMP [1.98]
DC	Neonatal [3.06]	Congenital [1.42]	SIDS [2.98]	Violence [21.67]	F Body [6.57]	LRI [4.65]	Diarrhea [13.51]	Endocrine [2.53]	Oth Un Inf [6.12]	Meningitis [3.38]
Florida	Neonatal [1.32]	Congenital [0.82]	SIDS [1.21]	F Body [3.88]	Diarrhea [3.89]	LRI [0.96]	Violence [4.35]	Endocrine [0.83]	CMP [2.35]	Oth Un Inf [2.01]
Georgia	Neonatal [1.26]	Congenital [0.74]	SIDS [2.02]	F Body [2.7]	LRI [0.89]	Violence [3.91]	Diarrhea [2.67]	Endocrine [0.75]	Oth Un Inf [1.79]	CMP [1.63]
Hawaii	Neonatal [1.19]	Congenital [0.67]	SIDS [1.03]	F Body [2.42]	LRI [1.2]	Endocrine [0.85]	Oth Un Inf [2.04]	Diarrhea [1.58]	Violence [1.72]	CMP [1.6]
Idaho	Neonatal [0.67]	Congenital [0.57]	SIDS [1.25]	F Body [1.21]	LRI [0.38]	Diarrhea [1.34]	Endocrine [0.64]	Oth Un Inf [1.02]	Road Inj [1.01]	Ileus [1.11]
Illinois	Neonatal [1.39]	Congenital [0.78]	SIDS [0.93]	F Body [3.43]	LRI [1.18]	Violence [3.85]	Diarrhea [3.53]	Endocrine [0.77]	CMP [1.82]	Oth Un Inf [1.75]
Indiana	Neonatal [1.14]	Congenital [0.83]	SIDS [1.46]	F Body [3.44]	LRI [0.88]	Diarrhea [2.98]	Violence [3.05]	Endocrine [0.85]	CMP [1.57]	Oth Un Inf [1.66]
Iowa	Neonatal [0.7]	Congenital [0.63]	SIDS [1.44]	F Body [1.6]	LRI [0.73]	Diarrhea [2.09]	Endocrine [0.73]	Oth Un Inf [1.53]	CMP [1.05]	Road Inj [1.18]
Kansas	Neonatal [0.97]	Congenital [0.74]	SIDS [1.36]	F Body [2.09]	LRI [0.7]	Diarrhea [2.21]	Endocrine [0.76]	Violence [1.75]	Oth Un Inf [1.64]	CMP [1.12]
Kentucky	Neonatal [0.95]	Congenital [0.81]	SIDS [2.29]	F Body [2.72]	LRI [0.7]	Diarrhea [2.37]	Violence [2.5]	Endocrine [0.85]	Oth Un Inf [1.7]	CMP [1.35]
Louisiana	Neonatal [1.24]	Congenital [0.85]	SIDS [2.2]	F Body [5.13]	LRI [1.17]	Violence [5.77]	Diarrhea [2.89]	Endocrine [0.96]	CMP [2.19]	Oth Un Inf [2.03]
Maine	Neonatal [1.36]	Congenital [1.02]	SIDS [1.72]	F Body [3.16]	Diarrhea [5.81]	LRI [1.24]	Endocrine [1.22]	CMP [1.8]	Oth Un Inf [1.88]	Road Inj [1.92]
Maryland	Neonatal [1.83]	Congenital [0.86]	SIDS [1.78]	F Body [1.9]	Diarrhea [5.15]	LRI [1.37]	Violence [3.96]	Endocrine [1.04]	Oth Un Inf [2.44]	CMP [1.98]
Massachusetts	Neonatal [1.51]	Congenital [0.74]	SIDS [0.91]	F Body [1.72]	LRI [1.45]	Diarrhea [3.96]	Endocrine [0.75]	Oth Un Inf [1.72]	Violence [1.43]	CMP [1.25]
Michigan	Neonatal [1.4]	Congenital [0.83]	SIDS [1.24]	F Body [4.28]	LRI [1.08]	Violence [4.08]	Diarrhea [3.26]	Endocrine [0.91]	CMP [1.93]	Oth Un Inf [1.79]
Minnesota	Neonatal [1.03]	Congenital [0.84]	SIDS [0.95]	F Body [2.55]	LRI [1.04]	Diarrhea [2.58]	Endocrine [0.73]	Oth Un Inf [1.89]	Violence [1.22]	CMP [1.11]
Mississippi	Neonatal [1.28]	Congenital [0.92]	SIDS [2.18]	F Body [5.3]	LRI [1.02]	Violence [5.29]	Diarrhea [2.9]	Road Inj [2.64]	Endocrine [0.9]	CMP [2.28]

eFigure 7b. Leading ten causes of DALYs with the ratio of observed DALYs to DALYs expected on the basis of Socio-Demographic Index alone in 2017, <1 years, both sexes combined. The top ten causes contributing to DALYs are listed globally, by socio-demographic quintile, and then by GBD superregion, region, country, and subnationally where modeled. For each cell, the ratio of observed DALYs to DALYs expected on the basis of socio-demographic index (SDI) alone are listed. Abbreviations: DALY=disability-adjusted life year, GBD=Global Burden of Disease.

Values shown in brackets represent the ratio of observed DALYs to predicted DALYs on the basis of Socio-Demographic Index (SDI), rounded to two (2) digits. Color ranges (shown below) were calculated to place a roughly equal number of cells into each bin.

COLOR KEY:	[0.0-0.33]	(0.33-0.46]	(0.46-0.58]	(0.58-0.7]	(0.7-0.84]	(0.84-1.03]	(1.03-1.38]	(1.38-2.46]	2.46+

	1	2	3	4	5	6	7	8	9	10
Missouri	Neonatal [1.07]	Congenital [0.76]	SIDS [1.14]	F Body [3.96]	LRI [0.8]	Violence [3.56]	Diarrhea [2.45]	Endocrine [0.73]	Oth Un Inf [1.62]	CMP [1.52]
Montana	Neonatal [0.92]	Congenital [0.86]	SIDS [1.93]	F Body [2.24]	LRI [1.0]	Diarrhea [2.51]	Endocrine [0.9]	Road Inj [1.98]	Oth Un Inf [1.74]	Violence [1.58]
Nebraska	Neonatal [0.91]	Congenital [0.72]	SIDS [1.38]	F Body [1.53]	LRI [0.7]	Endocrine [0.75]	Diarrhea [1.88]	Oth Un Inf [1.47]	Violence [1.14]	Road Inj [1.06]
Nevada	Neonatal [0.79]	Congenital [0.62]	SIDS [0.82]	F Body [1.81]	LRI [0.71]	Violence [3.0]	Diarrhea [2.22]	Endocrine [0.57]	CMP [1.26]	Oth Un Inf [1.16]
New Hampshire	Neonatal [1.26]	Congenital [0.77]	SIDS [1.11]	F Body [2.04]	Diarrhea [5.79]	LRI [1.24]	Endocrine [0.97]	Oth Un Inf [1.66]	CMP [1.37]	Ileus [2.13]
New Jersey	Neonatal [1.19]	Congenital [0.64]	SIDS [0.95]	F Body [1.72]	LRI [1.21]	Diarrhea [3.94]	Endocrine [0.82]	Violence [2.0]	Oth Un Inf [1.78]	CMP [1.45]
New Mexico	Neonatal [0.79]	Congenital [0.67]	SIDS [0.96]	F Body [1.51]	LRI [0.59]	Diarrhea [2.45]	Endocrine [0.86]	Violence [2.34]	Ileus [2.05]	Oth Un Inf [1.46]
New York	Neonatal [1.24]	Congenital [0.71]	SIDS [0.75]	F Body [2.01]	LRI [1.42]	Diarrhea [4.46]	Violence [3.34]	Endocrine [0.79]	Oth Un Inf [1.99]	CMP [1.73]
N Carolina	Neonatal [1.29]	Congenital [0.77]	SIDS [1.15]	F Body [2.58]	LRI [0.9]	Diarrhea [3.11]	Violence [3.37]	Endocrine [0.88]	CMP [1.75]	Oth Un Inf [1.78]
N Dakota	Neonatal [1.06]	Congenital [0.91]	SIDS [1.98]	F Body [2.38]	LRI [1.14]	Endocrine [1.12]	Diarrhea [2.53]	Oth Un Inf [1.97]	Road Inj [1.93]	CMP [1.38]
Ohio	Neonatal [1.35]	Congenital [0.87]	SIDS [1.61]	F Body [3.24]	LRI [0.93]	Diarrhea [3.09]	Violence [3.0]	Endocrine [0.93]	CMP [1.7]	Oth Un Inf [1.75]
Oklahoma	Neonatal [1.18]	Congenital [0.95]	SIDS [2.22]	F Body [2.55]	LRI [0.86]	Diarrhea [2.99]	Violence [3.73]	Endocrine [0.93]	Road Inj [1.99]	CMP [1.72]
Oregon	Neonatal [1.03]	Congenital [0.69]	SIDS [1.68]	F Body [2.01]	LRI [0.69]	Diarrhea [2.41]	Endocrine [0.93]	Violence [1.69]	Oth Un Inf [1.61]	Road Inj [1.32]
Pennsylvania	Neonatal [1.49]	Congenital [0.79]	SIDS [1.26]	F Body [2.68]	LRI [1.18]	Diarrhea [3.87]	Violence [2.73]	Endocrine [0.81]	CMP [1.67]	Oth Un Inf [1.7]
Rhode Island	Neonatal [1.69]	Congenital [0.83]	SIDS [0.96]	Diarrhea [6.71]	F Body [2.2]	LRI [1.19]	Endocrine [0.97]	Violence [1.92]	Oth Un Inf [1.76]	CMP [1.43]
S Carolina	Neonatal [1.31]	Congenital [0.74]	SIDS [1.61]	F Body [4.12]	LRI [0.79]	Violence [3.65]	Diarrhea [2.61]	Endocrine [0.86]	Oth Un Inf [1.82]	CMP [1.65]
S Dakota	Neonatal [0.9]	Congenital [0.82]	SIDS [1.81]	F Body [2.1]	LRI [1.01]	Diarrhea [3.08]	Endocrine [0.93]	Oth Un Inf [1.75]	Road Inj [1.88]	Violence [1.43]
Tennessee	Neonatal [0.96]	Congenital [0.8]	F Body [4.24]	SIDS [1.33]	LRI [0.9]	Diarrhea [2.94]	Violence [3.93]	Endocrine [0.81]	Oth Un Inf [1.85]	CMP [1.7]
Texas	Neonatal [0.85]	Congenital [0.69]	SIDS [1.34]	F Body [1.91]	LRI [0.59]	Violence [3.1]	Diarrhea [1.77]	Endocrine [0.63]	Oth Un Inf [1.7]	CMP [1.41]
Utah	Neonatal [0.65]	Congenital [0.58]	SIDS [0.96]	F Body [1.14]	LRI [0.45]	Endocrine [0.74]	Diarrhea [1.38]	Oth Un Inf [1.26]	Ileus [1.24]	Violence [0.75]
Vermont	Neonatal [1.22]	Congenital [0.85]	SIDS [1.03]	F Body [2.1]	Diarrhea [4.89]	LRI [1.06]	Endocrine [1.11]	Oth Un Inf [2.0]	CMP [1.34]	Ileus [2.1]
Virginia	Neonatal [1.35]	Congenital [0.82]	SIDS [1.61]	F Body [2.93]	LRI [1.31]	Diarrhea [4.06]	Violence [3.39]	Endocrine [0.8]	Oth Un Inf [2.03]	CMP [1.75]
Washington	Neonatal [0.96]	Congenital [0.74]	SIDS [1.57]	F Body [2.05]	LRI [0.8]	Diarrhea [2.49]	Endocrine [0.79]	Violence [1.78]	Oth Un Inf [1.6]	CMP [1.18]
W Virginia	Neonatal [1.05]	Congenital [0.82]	SIDS [2.37]	F Body [2.32]	LRI [0.75]	Diarrhea [2.96]	Endocrine [1.16]	Violence [2.55]	Oth Un Inf [1.64]	CMP [1.55]
Wisconsin	Neonatal [1.2]	Congenital [0.78]	SIDS [1.18]	F Body [2.18]	LRI [1.03]	Diarrhea [2.7]	Endocrine [0.78]	Violence [1.72]	Oth Un Inf [1.85]	CMP [1.42]
Wyoming	Neonatal [1.21]	Congenital [0.77]	SIDS [1.36]	F Body [2.32]	LRI [0.97]	Diarrhea [2.2]	Endocrine [0.86]	Oth Un Inf [1.74]	Road Inj [1.68]	Ileus [2.25]
**Southern Latin America**	Neonatal [0.67]	Congenital [0.69]	LRI [0.41]	STI [5.46]	F Body [0.96]	SIDS [0.66]	Diarrhea [0.84]	Meningitis [0.31]	Endocrine [0.6]	CMP [0.82]
Argentina	Neonatal [0.71]	Congenital [0.66]	LRI [0.41]	STI [6.26]	F Body [1.06]	SIDS [0.74]	Diarrhea [0.84]	Meningitis [0.33]	Endocrine [0.6]	CMP [1.01]
Chile	Neonatal [0.55]	Congenital [0.79]	LRI [0.3]	SIDS [0.42]	F Body [0.44]	Diarrhea [0.66]	Endocrine [0.57]	Meningitis [0.26]	STI [0.91]	Oth Neuro [0.68]
Uruguay	Neonatal [0.49]	Congenital [0.65]	LRI [0.36]	F Body [1.21]	STI [3.77]	SIDS [0.63]	Diarrhea [0.78]	PEM [2.77]	Endocrine [0.57]	Encepha [0.87]
**Western Europe**	Neonatal [0.61]	Congenital [0.48]	SIDS [0.49]	Endocrine [0.76]	LRI [0.4]	Diarrhea [1.17]	F Body [0.37]	Oth Neuro [0.8]	Meningitis [0.46]	Oth Un Inf [0.75]

eFigure 7b. Leading ten causes of DALYs with the ratio of observed DALYs to DALYs expected on the basis of Socio-Demographic Index alone in 2017, <1 years, both sexes combined. The top ten causes contributing to DALYs are listed globally, by socio-demographic quintile, and then by GBD superregion, region, country, and subnationally where modeled. For each cell, the ratio of observed DALYs to DALYs expected on the basis of socio-demographic index (SDI) alone are listed. Abbreviations: DALY=disability-adjusted life year, GBD=Global Burden of Disease.

Values shown in brackets represent the ratio of observed DALYs to predicted DALYs on the basis of Socio-Demographic Index (SDI), rounded to two (2) digits. Color ranges (shown below) were calculated to place a roughly equal number of cells into each bin.

COLOR KEY:	[0.0-0.33]	(0.33-0.46]	(0.46-0.58]	(0.58-0.7]	(0.7-0.84]	(0.84-1.03]	(1.03-1.38]	(1.38-2.46]	2.46+

	1	2	3	4	5	6	7	8	9	10
Andorra	Neonatal [0.48]	Congenital [0.29]	SIDS [0.47]	LRI [0.44]	Diarrhea [1.36]	Endocrine [0.44]	Oth Un Inf [1.0]	URI [1.2]	F Body [0.26]	Hernia [0.91]
Austria	Neonatal [0.58]	Congenital [0.5]	SIDS [0.45]	Endocrine [1.04]	Diarrhea [1.36]	LRI [0.29]	F Body [0.36]	Hernia [1.99]	Oth Neuro [0.63]	Meningitis [0.42]
Belgium	Neonatal [0.66]	Congenital [0.5]	SIDS [0.75]	F Body [0.94]	Endocrine [0.93]	LRI [0.59]	Diarrhea [1.88]	Oth Neuro [0.93]	Oth Un Inf [1.44]	Meningitis [0.74]
Cyprus	Neonatal [0.62]	Congenital [0.28]	SIDS [0.58]	LRI [0.37]	Diarrhea [1.15]	Oth Neuro [0.78]	Hernia [2.11]	Endocrine [0.34]	F Body [0.29]	URI [1.12]
Denmark	Neonatal [0.98]	Congenital [0.71]	SIDS [0.35]	Endocrine [1.48]	Diarrhea [3.43]	LRI [1.15]	Oth Neuro [1.45]	F Body [0.48]	Meningitis [1.16]	Oth Un Inf [1.42]
Finland	Neonatal [0.43]	Congenital [0.43]	SIDS [0.46]	Endocrine [0.64]	Diarrhea [1.36]	Oth Neuro [0.76]	F Body [0.36]	URI [1.15]	LRI [0.2]	Ileus [1.24]
France	Neonatal [0.65]	Congenital [0.45]	SIDS [0.71]	Endocrine [0.97]	F Body [0.53]	Diarrhea [1.29]	LRI [0.28]	Oth Neuro [0.84]	Meningitis [0.6]	Oth Un Inf [0.95]
Germany	Neonatal [0.72]	Congenital [0.55]	SIDS [0.65]	Endocrine [0.67]	Diarrhea [1.56]	Oth Neuro [1.15]	LRI [0.32]	F Body [0.42]	Hernia [1.56]	Violence [0.77]
Greece	Neonatal [0.48]	Congenital [0.58]	LRI [0.47]	F Body [0.54]	SIDS [0.19]	Meningitis [0.38]	Endocrine [0.33]	ALS [3.24]	Diarrhea [0.47]	URI [1.12]
Iceland	Neonatal [0.35]	Congenital [0.36]	SIDS [0.4]	LRI [0.8]	Diarrhea [1.83]	Endocrine [0.35]	Oth Neuro [0.66]	Meningitis [0.65]	URI [1.17]	F Body [0.26]
Ireland	Neonatal [0.57]	Congenital [0.76]	SIDS [0.84]	Endocrine [0.77]	LRI [0.44]	Diarrhea [1.39]	ALS [2.16]	Meningitis [0.65]	URI [1.14]	Oth Un Inf [0.78]
Israel	Neonatal [0.38]	Congenital [0.41]	SIDS [0.4]	Endocrine [0.8]	LRI [0.24]	Diarrhea [0.89]	F Body [0.29]	Oth Neuro [0.57]	Oth Un Inf [0.68]	Meningitis [0.27]
Italy	Neonatal [0.53]	Congenital [0.38]	Endocrine [0.66]	LRI [0.32]	SIDS [0.11]	Diarrhea [0.88]	Oth Neuro [0.61]	F Body [0.2]	URI [1.17]	ALS [1.56]
Luxembourg	Neonatal [0.49]	Congenital [0.26]	SIDS [0.63]	Diarrhea [2.74]	LRI [0.68]	F Body [0.64]	Endocrine [0.57]	Oth Un Inf [1.58]	Hernia [1.67]	URI [1.21]
Malta	Neonatal [0.83]	Congenital [0.94]	LRI [0.74]	F Body [0.44]	Endocrine [0.56]	Oth Neuro [1.05]	SIDS [0.1]	Meningitis [0.46]	Diarrhea [0.58]	Hernia [1.97]
Netherlands	Neonatal [0.99]	Congenital [0.73]	SIDS [0.24]	Endocrine [0.98]	LRI [0.71]	Oth Neuro [1.28]	F Body [0.57]	Meningitis [1.26]	Diarrhea [1.35]	Oth Un Inf [1.37]
Norway	Neonatal [0.52]	Congenital [0.47]	Poison [98.41]	SIDS [0.34]	Road Inj [3.24]	Endocrine [0.63]	LRI [0.44]	F Body [0.33]	URI [1.28]	Diarrhea [0.89]
Portugal	Neonatal [0.32]	Congenital [0.27]	LRI [0.24]	F Body [0.37]	Endocrine [0.55]	Diarrhea [0.6]	SIDS [0.09]	Meningitis [0.19]	ALS [3.71]	URI [1.07]
Spain	Neonatal [0.44]	Congenital [0.33]	SIDS [0.29]	Endocrine [0.68]	LRI [0.24]	Diarrhea [0.74]	Oth Neuro [0.74]	F Body [0.24]	Meningitis [0.34]	URI [1.12]
Sweden	Neonatal [0.51]	Congenital [0.47]	SIDS [0.51]	LRI [0.45]	Diarrhea [1.41]	Endocrine [0.44]	Oth Neuro [0.58]	URI [1.21]	Oth Un Inf [0.77]	F Body [0.19]
Stockholm	Neonatal [0.5]	Congenital [0.55]	SIDS [0.38]	Endocrine [0.83]	Oth Neuro [1.27]	Diarrhea [1.67]	LRI [0.5]	Oth Un Inf [1.24]	URI [1.23]	Meningitis [0.64]
Sweden w/o Stockholm	Neonatal [0.5]	Congenital [0.45]	SIDS [0.54]	LRI [0.43]	Diarrhea [1.32]	Endocrine [0.33]	URI [1.2]	CMP [0.61]	Oth Un Inf [0.65]	Oth Neuro [0.39]
Switzerland	Neonatal [0.81]	Congenital [0.71]	SIDS [0.3]	Diarrhea [3.01]	Endocrine [0.71]	LRI [0.49]	Oth Neuro [0.68]	Oth Un Inf [0.89]	URI [1.18]	F Body [0.22]
UK	Neonatal [0.73]	Congenital [0.53]	SIDS [0.66]	LRI [0.61]	Endocrine [0.77]	Diarrhea [1.06]	Meningitis [0.56]	Oth Neuro [0.82]	F Body [0.32]	Oth Un Inf [1.01]
England	Neonatal [0.76]	Congenital [0.54]	SIDS [0.66]	LRI [0.67]	Endocrine [0.8]	Diarrhea [1.11]	Oth Neuro [0.85]	Meningitis [0.59]	F Body [0.33]	Oth Un Inf [1.05]
E Midlands	Neonatal [0.73]	Congenital [0.51]	SIDS [0.58]	LRI [0.49]	Endocrine [0.79]	Diarrhea [0.92]	F Body [0.31]	Meningitis [0.48]	Oth Neuro [0.8]	Oth Un Inf [0.9]
Derby	Neonatal [0.98]	Congenital [0.68]	SIDS [0.74]	LRI [0.85]	Endocrine [0.97]	Meningitis [0.74]	F Body [0.39]	Diarrhea [1.01]	Oth Neuro [0.84]	Oth Un Inf [1.15]
Derbyshire	Neonatal [0.53]	Congenital [0.4]	SIDS [0.55]	LRI [0.35]	Endocrine [0.6]	Diarrhea [0.75]	F Body [0.28]	Oth Neuro [0.8]	Meningitis [0.38]	Oth Un Inf [0.63]
Leicester	Neonatal [1.04]	Congenital [0.72]	SIDS [0.83]	LRI [0.82]	Endocrine [1.01]	Diarrhea [1.21]	Oth Neuro [0.99]	Meningitis [0.64]	F Body [0.38]	Oth Un Inf [1.16]
Leicestershire	Neonatal [0.77]	Congenital [0.51]	SIDS [0.47]	LRI [0.53]	Endocrine [0.79]	Diarrhea [0.99]	Oth Neuro [0.71]	Oth Un Inf [0.93]	Meningitis [0.41]	F Body [0.22]
Lincolnshire	Neonatal [0.53]	Congenital [0.4]	SIDS [0.56]	LRI [0.29]	Endocrine [0.68]	Diarrhea [0.64]	Oth Neuro [0.71]	F Body [0.22]	Meningitis [0.32]	Iron [3.78]

eFigure 7b. Leading ten causes of DALYs with the ratio of observed DALYs to DALYs expected on the basis of Socio-Demographic Index alone in 2017, <1 years, both sexes combined. The top ten causes contributing to DALYs are listed globally, by socio-demographic quintile, and then by GBD superregion, region, country, and subnationally where modeled. For each cell, the ratio of observed DALYs to DALYs expected on the basis of socio-demographic index (SDI) alone are listed. Abbreviations: DALY=disability-adjusted life year, GBD=Global Burden of Disease.

Values shown in brackets represent the ratio of observed DALYs to predicted DALYs on the basis of Socio-Demographic Index (SDI), rounded to two (2) digits. Color ranges (shown below) were calculated to place a roughly equal number of cells into each bin.

COLOR KEY:	[0.0-0.33]	(0.33-0.46]	(0.46-0.58]	(0.58-0.7]	(0.7-0.84]	(0.84-1.03]	(1.03-1.38]	(1.38-2.46]	2.46+

	1	2	3	4	5	6	7	8	9	10
Northamptonshire	Neonatal [0.75]	Congenital [0.51]	SIDS [0.58]	LRI [0.47]	Endocrine [0.76]	Diarrhea [1.04]	F Body [0.32]	Meningitis [0.46]	Oth Neuro [0.7]	Oth Un Inf [0.92]
Nottingham	Neonatal [1.12]	Congenital [0.73]	SIDS [0.46]	LRI [1.08]	Endocrine [1.1]	F Body [0.6]	Meningitis [0.96]	Diarrhea [1.41]	Oth Un Inf [1.66]	Oth Neuro [1.04]
Nottinghamshire	Neonatal [0.64]	Congenital [0.47]	SIDS [0.57]	LRI [0.38]	Endocrine [0.75]	F Body [0.31]	Diarrhea [0.74]	Oth Neuro [0.84]	Meningitis [0.41]	Oth Un Inf [0.78]
Rutland	Neonatal [0.29]	Congenital [0.28]	SIDS [0.52]	LRI [0.25]	Endocrine [0.48]	Diarrhea [0.69]	Meningitis [0.34]	Oth Neuro [0.49]	URI [1.13]	F Body [0.16]
E England	Neonatal [0.62]	Congenital [0.44]	SIDS [0.57]	LRI [0.45]	Endocrine [0.7]	Diarrhea [0.89]	Oth Neuro [0.75]	F Body [0.28]	Meningitis [0.41]	Oth Un Inf [0.87]
Bedford	Neonatal [0.79]	Congenital [0.54]	SIDS [0.7]	LRI [0.54]	Endocrine [0.83]	F Body [0.48]	Oth Neuro [1.21]	Diarrhea [1.08]	Oth Un Inf [1.13]	Meningitis [0.45]
Cambridgeshire	Neonatal [0.74]	Congenital [0.5]	SIDS [0.52]	LRI [0.6]	Endocrine [0.77]	Diarrhea [1.3]	F Body [0.38]	Oth Neuro [0.74]	Oth Un Inf [0.99]	Meningitis [0.51]
Cen Bedfordshire	Neonatal [0.46]	Congenital [0.35]	SIDS [0.51]	LRI [0.36]	Endocrine [0.57]	Diarrhea [0.91]	F Body [0.27]	Oth Neuro [0.69]	Oth Un Inf [0.93]	Meningitis [0.34]
Essex	Neonatal [0.58]	Congenital [0.41]	SIDS [0.55]	LRI [0.36]	Endocrine [0.69]	Diarrhea [0.71]	Oth Neuro [0.69]	Meningitis [0.37]	Oth Un Inf [0.84]	F Body [0.22]
Hertfordshire	Neonatal [0.64]	Congenital [0.43]	SIDS [0.48]	LRI [0.62]	Endocrine [0.6]	Diarrhea [1.17]	Oth Neuro [0.65]	F Body [0.29]	Oth Un Inf [0.9]	Meningitis [0.45]
Luton	Neonatal [0.99]	Congenital [0.73]	SIDS [0.78]	LRI [0.76]	Endocrine [1.15]	F Body [0.46]	Oth Neuro [1.15]	Diarrhea [1.1]	Meningitis [0.61]	Oth Un Inf [1.19]
Norfolk	Neonatal [0.62]	Congenital [0.47]	SIDS [0.67]	LRI [0.36]	Endocrine [0.73]	Diarrhea [0.79]	Oth Neuro [0.87]	F Body [0.26]	Meningitis [0.4]	Oth Un Inf [0.86]
Peterborough	Neonatal [0.61]	Congenital [0.42]	SIDS [0.64]	LRI [0.39]	Endocrine [0.78]	Diarrhea [0.84]	Oth Neuro [0.85]	F Body [0.27]	Oth Un Inf [0.83]	Meningitis [0.31]
Southend-on-Sea	Neonatal [0.47]	Congenital [0.37]	SIDS [0.57]	LRI [0.31]	Endocrine [0.61]	Diarrhea [0.56]	Meningitis [0.33]	Oth Neuro [0.57]	Iron [3.58]	F Body [0.17]
Suffolk	Neonatal [0.5]	Congenital [0.39]	SIDS [0.63]	LRI [0.32]	Endocrine [0.65]	Diarrhea [0.7]	Oth Neuro [0.71]	F Body [0.24]	Meningitis [0.33]	Oth Un Inf [0.74]
Thurrock	Neonatal [0.45]	Congenital [0.34]	SIDS [0.52]	LRI [0.28]	Endocrine [0.66]	Meningitis [0.35]	Diarrhea [0.51]	Oth Neuro [0.61]	F Body [0.19]	Oth Un Inf [0.58]
Greater London	Neonatal [0.9]	Congenital [0.57]	SIDS [0.64]	LRI [1.38]	Endocrine [0.86]	Diarrhea [1.71]	Meningitis [1.07]	Oth Un Inf [1.52]	Oth Neuro [0.87]	F Body [0.44]
Barking & Dagenham	Neonatal [0.54]	Congenital [0.41]	SIDS [0.66]	LRI [0.4]	Endocrine [0.61]	Diarrhea [0.65]	Meningitis [0.35]	F Body [0.24]	Oth Neuro [0.64]	Oth Un Inf [0.66]
Barnet	Neonatal [0.52]	Congenital [0.38]	SIDS [0.57]	LRI [0.73]	Endocrine [0.66]	Diarrhea [1.03]	Oth Neuro [0.81]	F Body [0.3]	Meningitis [0.56]	Oth Un Inf [0.93]
Bexley	Neonatal [0.46]	Congenital [0.35]	SIDS [0.57]	LRI [0.38]	Endocrine [0.68]	Oth Neuro [0.86]	Diarrhea [0.74]	Meningitis [0.43]	F Body [0.2]	Oth Un Inf [0.74]
Brent	Neonatal [0.73]	Congenital [0.51]	SIDS [0.71]	LRI [0.79]	Endocrine [0.76]	Oth Neuro [1.05]	Diarrhea [1.07]	F Body [0.39]	Meningitis [0.68]	Oth Un Inf [1.19]
Bromley	Neonatal [0.5]	Congenital [0.36]	SIDS [0.38]	LRI [0.44]	Endocrine [0.57]	Diarrhea [0.83]	Oth Neuro [0.66]	Meningitis [0.46]	Oth Un Inf [0.84]	URI [1.16]
Camden	Neonatal [0.94]	Congenital [0.51]	SIDS [0.58]	LRI [1.93]	Endocrine [0.8]	Diarrhea [1.97]	F Body [0.59]	Oth Un Inf [1.88]	Meningitis [1.48]	URI [1.44]
Croydon	Neonatal [0.62]	Congenital [0.44]	SIDS [0.52]	LRI [0.49]	Endocrine [0.63]	Diarrhea [0.77]	Meningitis [0.49]	Oth Neuro [0.76]	F Body [0.27]	Oth Un Inf [0.91]
Ealing	Neonatal [0.59]	Congenital [0.39]	SIDS [0.5]	LRI [0.81]	Endocrine [0.63]	Diarrhea [1.21]	Oth Un Inf [1.23]	Oth Neuro [0.67]	F Body [0.28]	Meningitis [0.5]
Enfield	Neonatal [0.66]	Congenital [0.44]	SIDS [0.67]	LRI [0.59]	Endocrine [0.66]	Diarrhea [0.96]	Meningitis [0.57]	Oth Neuro [0.81]	F Body [0.27]	CMP [0.83]
Greenwich	Neonatal [0.78]	Congenital [0.5]	SIDS [0.61]	LRI [0.5]	Endocrine [0.62]	Meningitis [0.57]	Diarrhea [0.88]	Oth Un Inf [0.91]	Oth Neuro [0.65]	F Body [0.22]
Hackney	Neonatal [1.18]	Congenital [0.69]	SIDS [0.97]	LRI [1.7]	Endocrine [1.31]	Diarrhea [2.14]	Meningitis [1.47]	F Body [0.68]	Oth Un Inf [2.21]	Oth Neuro [1.08]
Hammersmith & Fulham	Neonatal [0.95]	Congenital [0.55]	SIDS [0.62]	LRI [1.66]	Endocrine [0.89]	Diarrhea [2.17]	Meningitis [1.55]	Oth Un Inf [1.88]	F Body [0.53]	Oth Neuro [0.65]
Haringey	Neonatal [0.75]	Congenital [0.47]	SIDS [0.71]	LRI [0.81]	Endocrine [0.83]	Meningitis [0.82]	Diarrhea [1.25]	Oth Un Inf [1.49]	F Body [0.39]	Oth Neuro [0.82]
Harrow	Neonatal [0.86]	Congenital [0.55]	SIDS [0.63]	LRI [0.63]	Endocrine [0.88]	Diarrhea [1.08]	Meningitis [0.68]	Oth Neuro [0.8]	Oth Un Inf [1.14]	CMP [0.88]

eFigure 7b. Leading ten causes of DALYs with the ratio of observed DALYs to DALYs expected on the basis of Socio-Demographic Index alone in 2017, <1 years, both sexes combined. The top ten causes contributing to DALYs are listed globally, by socio-demographic quintile, and then by GBD superregion, region, country, and subnationally where modeled. For each cell, the ratio of observed DALYs to DALYs expected on the basis of socio-demographic index (SDI) alone are listed. Abbreviations: DALY=disability-adjusted life year, GBD=Global Burden of Disease.

Values shown in brackets represent the ratio of observed DALYs to predicted DALYs on the basis of Socio-Demographic Index (SDI), rounded to two (2) digits. Color ranges (shown below) were calculated to place a roughly equal number of cells into each bin.

COLOR KEY:	[0.0-0.33]	(0.33-0.46]	(0.46-0.58]	(0.58-0.7]	(0.7-0.84]	(0.84-1.03]	(1.03-1.38]	(1.38-2.46]	2.46+

	1	2	3	4	5	6	7	8	9	10
Havering	Neonatal [0.42]	Congenital [0.32]	SIDS [0.4]	LRI [0.35]	Endocrine [0.51]	Diarrhea [0.63]	Meningitis [0.33]	F Body [0.2]	Oth Neuro [0.53]	URI [1.2]
Hillingdon	Neonatal [0.67]	Congenital [0.49]	SIDS [0.52]	LRI [0.99]	Endocrine [0.75]	Diarrhea [1.67]	Oth Neuro [0.84]	Meningitis [0.67]	F Body [0.3]	Oth Un Inf [1.01]
Hounslow	Neonatal [0.85]	Congenital [0.55]	SIDS [0.6]	LRI [1.04]	Endocrine [0.83]	Diarrhea [1.68]	Oth Un Inf [1.44]	Meningitis [0.8]	Oth Neuro [0.76]	F Body [0.32]
Islington	Neonatal [0.86]	Congenital [0.51]	SIDS [0.69]	LRI [1.89]	Endocrine [0.77]	Diarrhea [2.1]	F Body [0.68]	Oth Un Inf [1.73]	Meningitis [1.19]	Oth Neuro [0.68]
Kensington & Chelsea	Neonatal [0.73]	Congenital [0.42]	SIDS [0.57]	LRI [1.58]	Endocrine [0.75]	Diarrhea [2.09]	F Body [0.64]	Oth Un Inf [1.84]	Oth Neuro [0.87]	Meningitis [1.2]
Kingston upon Thames	Neonatal [0.78]	Congenital [0.53]	SIDS [0.73]	LRI [1.27]	Endocrine [0.87]	Diarrhea [1.7]	Oth Neuro [0.95]	Meningitis [0.93]	F Body [0.38]	Oth Un Inf [1.13]
Lambeth	Neonatal [1.2]	Congenital [0.71]	SIDS [0.93]	LRI [1.96]	Endocrine [1.04]	Oth Un Inf [2.81]	Meningitis [1.5]	F Body [0.66]	Diarrhea [1.79]	Oth Neuro [1.03]
Lewisham	Neonatal [0.81]	Congenital [0.47]	SIDS [0.65]	LRI [0.8]	Endocrine [0.71]	Diarrhea [0.93]	Oth Neuro [0.73]	Oth Un Inf [1.05]	Meningitis [0.45]	F Body [0.25]
Merton	Neonatal [0.7]	Congenital [0.48]	SIDS [0.61]	LRI [0.81]	Endocrine [0.77]	Diarrhea [1.27]	Oth Neuro [0.81]	Oth Un Inf [1.19]	Meningitis [0.65]	F Body [0.31]
Newham	Neonatal [0.66]	Congenital [0.48]	SIDS [0.6]	LRI [0.8]	Endocrine [0.91]	Diarrhea [1.3]	Meningitis [0.79]	Oth Un Inf [1.58]	F Body [0.37]	Oth Neuro [0.92]
Redbridge	Neonatal [0.45]	Congenital [0.34]	SIDS [0.46]	LRI [0.46]	Endocrine [0.59]	Diarrhea [0.8]	Meningitis [0.43]	Oth Neuro [0.68]	F Body [0.24]	Oth Un Inf [0.65]
Richmond upon Thames	Neonatal [0.58]	Congenital [0.41]	SIDS [0.33]	LRI [0.82]	Endocrine [0.55]	Diarrhea [1.39]	Oth Un Inf [1.05]	URI [1.29]	Meningitis [0.65]	Oth Neuro [0.56]
Southwark	Neonatal [1.22]	Congenital [0.65]	SIDS [0.67]	LRI [1.76]	Endocrine [0.92]	Oth Un Inf [2.09]	Diarrhea [1.79]	Meningitis [1.25]	F Body [0.46]	Oth Neuro [0.73]
Sutton	Neonatal [0.52]	Congenital [0.46]	SIDS [0.57]	LRI [0.59]	Endocrine [0.68]	Diarrhea [1.01]	Oth Neuro [0.85]	F Body [0.31]	Meningitis [0.52]	Oth Un Inf [1.08]
Tower Hamlets	Neonatal [1.23]	Congenital [0.78]	SIDS [0.69]	LRI [2.06]	Endocrine [1.14]	Meningitis [1.73]	Diarrhea [2.24]	F Body [0.64]	Oth Un Inf [1.87]	Oth Neuro [0.92]
Waltham Forest	Neonatal [0.52]	Congenital [0.38]	SIDS [0.57]	LRI [0.52]	Endocrine [0.66]	Diarrhea [0.86]	F Body [0.29]	Oth Neuro [0.82]	Meningitis [0.42]	Oth Un Inf [0.76]
Wandsworth	Neonatal [0.89]	Congenital [0.52]	SIDS [0.5]	LRI [1.44]	Endocrine [0.74]	Diarrhea [1.73]	Oth Neuro [0.96]	Oth Un Inf [1.71]	Meningitis [1.15]	F Body [0.37]
Westminster	Neonatal [0.93]	Congenital [0.49]	SIDS [0.55]	LRI [1.58]	Endocrine [0.83]	Diarrhea [2.21]	Oth Un Inf [2.08]	Meningitis [1.45]	F Body [0.57]	Oth Neuro [0.69]
NE England	Neonatal [0.56]	Congenital [0.42]	SIDS [0.72]	LRI [0.42]	Endocrine [0.65]	Diarrhea [0.83]	Oth Neuro [0.82]	F Body [0.26]	Meningitis [0.39]	Oth Un Inf [0.74]
County Durham	Neonatal [0.59]	Congenital [0.45]	SIDS [0.81]	LRI [0.37]	Endocrine [0.68]	Diarrhea [0.73]	Oth Neuro [0.93]	Meningitis [0.38]	F Body [0.24]	Oth Un Inf [0.68]
Darlington	Neonatal [0.6]	Congenital [0.41]	SIDS [0.67]	LRI [0.34]	Endocrine [0.63]	Oth Neuro [0.78]	Diarrhea [0.69]	Meningitis [0.43]	Oth Un Inf [0.74]	URI [1.19]
Gateshead	Neonatal [0.68]	Congenital [0.48]	SIDS [0.92]	LRI [0.55]	Endocrine [0.74]	Diarrhea [0.89]	Oth Neuro [0.94]	F Body [0.33]	Meningitis [0.41]	Oth Un Inf [0.86]
Hartlepool	Neonatal [0.53]	Congenital [0.35]	SIDS [0.66]	LRI [0.29]	Endocrine [0.55]	F Body [0.3]	Diarrhea [0.61]	Meningitis [0.28]	Oth Neuro [0.61]	Oth Un Inf [0.56]
Middlesbrough	Neonatal [0.59]	Congenital [0.46]	SIDS [0.85]	LRI [0.54]	Endocrine [0.67]	Diarrhea [0.82]	Oth Neuro [0.98]	Meningitis [0.41]	Oth Un Inf [1.0]	F Body [0.24]
Newcastle upon Tyne	Neonatal [0.7]	Congenital [0.51]	SIDS [0.8]	LRI [0.91]	Endocrine [0.88]	Diarrhea [1.56]	F Body [0.48]	Oth Neuro [0.98]	Meningitis [0.82]	Oth Un Inf [1.1]
N Tyneside	Neonatal [0.44]	Congenital [0.34]	SIDS [0.59]	LRI [0.33]	Diarrhea [0.97]	Endocrine [0.53]	F Body [0.22]	Oth Neuro [0.61]	Meningitis [0.31]	Oth Un Inf [0.67]
Northumberland	Neonatal [0.46]	Congenital [0.41]	SIDS [0.5]	LRI [0.28]	Endocrine [0.6]	Diarrhea [0.63]	F Body [0.26]	Oth Neuro [0.68]	Meningitis [0.31]	Iron [3.95]
Redcar & Cleveland	Neonatal [0.42]	Congenital [0.31]	SIDS [0.64]	LRI [0.24]	Endocrine [0.44]	Diarrhea [0.49]	Oth Neuro [0.61]	Meningitis [0.23]	Oth Un Inf [0.58]	F Body [0.14]
S Tyneside	Neonatal [0.36]	Congenital [0.31]	SIDS [0.53]	LRI [0.22]	Endocrine [0.5]	Diarrhea [0.58]	F Body [0.2]	Oth Neuro [0.61]	Meningitis [0.21]	Oth Un Inf [0.51]
Stockton-on-Tees	Neonatal [0.62]	Congenital [0.43]	SIDS [0.7]	LRI [0.44]	Endocrine [0.64]	Diarrhea [0.81]	Oth Neuro [0.81]	Meningitis [0.35]	URI [1.23]	F Body [0.16]
Sunderland	Neonatal [0.53]	Congenital [0.39]	SIDS [0.77]	LRI [0.44]	Endocrine [0.67]	Diarrhea [0.93]	F Body [0.32]	Meningitis [0.48]	Oth Neuro [0.92]	Oth Un Inf [0.78]

eFigure 7b. Leading ten causes of DALYs with the ratio of observed DALYs to DALYs expected on the basis of Socio-Demographic Index alone in 2017, <1 years, both sexes combined. The top ten causes contributing to DALYs are listed globally, by socio-demographic quintile, and then by GBD superregion, region, country, and subnationally where modeled. For each cell, the ratio of observed DALYs to DALYs expected on the basis of socio-demographic index (SDI) alone are listed. Abbreviations: DALY=disability-adjusted life year, GBD=Global Burden of Disease.

Values shown in brackets represent the ratio of observed DALYs to predicted DALYs on the basis of Socio-Demographic Index (SDI), rounded to two (2) digits. Color ranges (shown below) were calculated to place a roughly equal number of cells into each bin.

COLOR KEY:	[0.0-0.33]	(0.33-0.46]	(0.46-0.58]	(0.58-0.7]	(0.7-0.84]	(0.84-1.03]	(1.03-1.38]	(1.38-2.46]	2.46+

	1	2	3	4	5	6	7	8	9	10
NW England	Neonatal [0.8]	Congenital [0.59]	SIDS [0.77]	LRI [0.68]	Endocrine [0.85]	Diarrhea [1.06]	Meningitis [0.61]	F Body [0.37]	Oth Neuro [0.97]	Oth Un Inf [1.03]
Blackburn with Darwen	Neonatal [0.69]	Congenital [0.5]	SIDS [0.91]	LRI [0.41]	Endocrine [0.84]	Diarrhea [0.73]	Oth Neuro [1.06]	Meningitis [0.4]	Oth Un Inf [0.83]	CMP [0.76]
Blackpool	Neonatal [0.61]	Congenital [0.47]	SIDS [1.1]	LRI [0.38]	Endocrine [0.77]	F Body [0.34]	Meningitis [0.37]	Diarrhea [0.5]	Oth Neuro [0.91]	CMP [0.67]
Bolton	Neonatal [0.6]	Congenital [0.46]	SIDS [0.82]	LRI [0.46]	Endocrine [0.8]	Meningitis [0.5]	Oth Neuro [0.93]	Diarrhea [0.62]	F Body [0.24]	Oth Un Inf [0.73]
Bury	Neonatal [0.67]	Congenital [0.52]	SIDS [0.65]	LRI [0.47]	Endocrine [0.77]	F Body [0.36]	Diarrhea [0.82]	Oth Neuro [0.89]	Meningitis [0.37]	Oth Un Inf [0.89]
Cheshire E	Neonatal [0.8]	Congenital [0.55]	SIDS [0.53]	LRI [0.78]	Endocrine [0.76]	Diarrhea [1.36]	Oth Neuro [0.89]	F Body [0.39]	Meningitis [0.64]	Oth Un Inf [1.02]
Cheshire W & Chester	Neonatal [0.78]	Congenital [0.57]	SIDS [0.62]	LRI [0.76]	Endocrine [0.84]	Diarrhea [1.35]	F Body [0.46]	Meningitis [0.7]	Oth Neuro [0.83]	Oth Un Inf [1.02]
Cumbria	Neonatal [0.64]	Congenital [0.49]	SIDS [0.71]	LRI [0.41]	Endocrine [0.66]	Diarrhea [0.95]	Oth Neuro [0.98]	F Body [0.32]	Meningitis [0.46]	Oth Un Inf [0.94]
Halton	Neonatal [0.63]	Congenital [0.43]	SIDS [0.68]	LRI [0.48]	Endocrine [0.68]	Diarrhea [0.97]	Meningitis [0.57]	Oth Neuro [0.82]	F Body [0.29]	Oth Un Inf [0.77]
Knowsley	Neonatal [0.51]	Congenital [0.41]	SIDS [0.71]	LRI [0.46]	Endocrine [0.65]	Diarrhea [0.82]	F Body [0.26]	Meningitis [0.39]	Oth Neuro [0.73]	Oth Un Inf [0.66]
Lancashire	Neonatal [0.8]	Congenital [0.61]	SIDS [0.67]	LRI [0.68]	Endocrine [0.83]	F Body [0.41]	Meningitis [0.65]	Diarrhea [1.01]	Oth Neuro [1.01]	Oth Un Inf [0.98]
Liverpool	Neonatal [1.09]	Congenital [0.75]	SIDS [0.93]	LRI [1.2]	Endocrine [1.05]	Diarrhea [1.51]	Meningitis [0.86]	Oth Neuro [1.16]	F Body [0.45]	Oth Un Inf [1.37]
Manchester	Neonatal [1.4]	Congenital [0.91]	SIDS [0.96]	LRI [1.77]	Endocrine [1.26]	Diarrhea [2.03]	Meningitis [1.27]	Oth Un Inf [1.99]	F Body [0.58]	Oth Neuro [1.16]
Oldham	Neonatal [0.79]	Congenital [0.6]	SIDS [0.85]	LRI [0.35]	Endocrine [0.9]	Meningitis [0.49]	Oth Neuro [1.19]	F Body [0.29]	Diarrhea [0.62]	Oth Un Inf [0.79]
Rochdale	Neonatal [0.59]	Congenital [0.47]	SIDS [0.91]	LRI [0.43]	Endocrine [0.79]	Oth Neuro [1.25]	Meningitis [0.45]	Diarrhea [0.64]	F Body [0.29]	Oth Un Inf [0.83]
Salford	Neonatal [0.82]	Congenital [0.66]	SIDS [0.89]	LRI [0.89]	Endocrine [1.06]	Oth Neuro [1.25]	F Body [0.46]	Diarrhea [1.19]	Meningitis [0.72]	Oth Un Inf [1.48]
Sefton	Neonatal [0.67]	Congenital [0.5]	SIDS [0.82]	LRI [0.4]	Endocrine [0.73]	Diarrhea [0.68]	Meningitis [0.4]	F Body [0.27]	Oth Neuro [0.81]	Oth Un Inf [0.91]
St Helens	Neonatal [0.51]	Congenital [0.4]	SIDS [0.58]	LRI [0.41]	Endocrine [0.65]	Diarrhea [0.73]	F Body [0.27]	Oth Neuro [0.72]	Meningitis [0.28]	Oth Un Inf [0.74]
Stockport	Neonatal [0.9]	Congenital [0.67]	SIDS [0.79]	LRI [0.74]	Endocrine [0.97]	Diarrhea [1.23]	Oth Neuro [1.07]	F Body [0.41]	Meningitis [0.63]	Oth Un Inf [1.02]
Tameside	Neonatal [0.5]	Congenital [0.42]	SIDS [0.72]	LRI [0.34]	Endocrine [0.62]	Diarrhea [0.74]	Meningitis [0.43]	F Body [0.25]	Oth Neuro [0.75]	Oth Un Inf [0.61]
Trafford	Neonatal [0.8]	Congenital [0.5]	SIDS [0.53]	LRI [0.74]	Endocrine [0.8]	Diarrhea [1.34]	Oth Neuro [0.83]	F Body [0.36]	Meningitis [0.69]	Oth Un Inf [1.15]
Warrington	Neonatal [0.92]	Congenital [0.61]	SIDS [0.74]	LRI [1.01]	Endocrine [0.7]	Diarrhea [1.29]	Meningitis [0.69]	F Body [0.35]	Oth Neuro [0.76]	Oth Un Inf [1.09]
Wigan	Neonatal [0.54]	Congenital [0.42]	SIDS [0.74]	LRI [0.37]	Endocrine [0.62]	Diarrhea [0.62]	F Body [0.24]	Meningitis [0.32]	Oth Neuro [0.56]	Oth Un Inf [0.66]
Wirral	Neonatal [0.67]	Congenital [0.55]	SIDS [0.91]	LRI [0.48]	Endocrine [0.9]	F Body [0.34]	Diarrhea [0.75]	Oth Neuro [0.96]	Meningitis [0.43]	Oth Un Inf [0.84]
SE England	Neonatal [0.65]	Congenital [0.46]	SIDS [0.59]	LRI [0.54]	Endocrine [0.67]	Diarrhea [0.99]	Oth Neuro [0.67]	Meningitis [0.49]	F Body [0.25]	Oth Un Inf [0.9]
Bracknell Forest	Neonatal [0.42]	Congenital [0.34]	SIDS [0.43]	LRI [0.53]	Endocrine [0.51]	Diarrhea [0.96]	F Body [0.31]	Oth Neuro [0.53]	URI [1.22]	Oth Un Inf [0.71]
Brighton & Hove	Neonatal [1.05]	Congenital [0.7]	SIDS [0.8]	LRI [1.13]	Endocrine [0.99]	Meningitis [1.16]	F Body [0.55]	Oth Un Inf [1.73]	Diarrhea [1.42]	Oth Neuro [0.81]
Buckinghamshire	Neonatal [0.85]	Congenital [0.6]	SIDS [0.53]	LRI [0.73]	Endocrine [0.83]	Diarrhea [1.24]	Meningitis [0.71]	F Body [0.37]	Oth Neuro [0.76]	Oth Un Inf [1.19]
E Sussex	Neonatal [0.5]	Congenital [0.43]	SIDS [0.65]	LRI [0.29]	Endocrine [0.53]	Diarrhea [0.59]	Oth Neuro [0.6]	Meningitis [0.28]	Oth Un Inf [0.7]	CMP [0.64]
Hampshire	Neonatal [0.61]	Congenital [0.42]	SIDS [0.53]	LRI [0.4]	Endocrine [0.63]	Diarrhea [0.92]	Oth Neuro [0.61]	Oth Un Inf [0.8]	F Body [0.22]	Meningitis [0.36]
Isle of Wight	Neonatal [0.28]	Congenital [0.26]	SIDS [0.5]	LRI [0.2]	Endocrine [0.44]	Diarrhea [0.55]	Oth Neuro [0.52]	F Body [0.16]	URI [1.15]	Meningitis [0.2]

eFigure 7b. Leading ten causes of DALYs with the ratio of observed DALYs to DALYs expected on the basis of Socio-Demographic Index alone in 2017, <1 years, both sexes combined. The top ten causes contributing to DALYs are listed globally, by socio-demographic quintile, and then by GBD superregion, region, country, and subnationally where modeled. For each cell, the ratio of observed DALYs to DALYs expected on the basis of socio-demographic index (SDI) alone are listed. Abbreviations: DALY=disability-adjusted life year, GBD=Global Burden of Disease.

Values shown in brackets represent the ratio of observed DALYs to predicted DALYs on the basis of Socio-Demographic Index (SDI), rounded to two (2) digits. Color ranges (shown below) were calculated to place a roughly equal number of cells into each bin.

COLOR KEY:	[0.0-0.33]	(0.33-0.46]	(0.46-0.58]	(0.58-0.7]	(0.7-0.84]	(0.84-1.03]	(1.03-1.38]	(1.38-2.46]	2.46+

	1	2	3	4	5	6	7	8	9	10
Kent	Neonatal [0.55]	Congenital [0.4]	SIDS [0.69]	LRI [0.38]	Endocrine [0.63]	Diarrhea [0.73]	Meningitis [0.4]	Oth Neuro [0.66]	F Body [0.21]	Oth Un Inf [0.75]
Medway	Neonatal [0.55]	Congenital [0.42]	SIDS [0.53]	LRI [0.28]	Endocrine [0.55]	Oth Neuro [0.73]	Diarrhea [0.55]	Meningitis [0.35]	URI [1.21]	F Body [0.15]
Milton Keynes	Neonatal [0.94]	Congenital [0.58]	SIDS [0.75]	LRI [0.86]	Endocrine [0.77]	Diarrhea [1.25]	Meningitis [0.6]	Oth Un Inf [1.02]	F Body [0.28]	Oth Neuro [0.6]
Oxfordshire	Neonatal [0.73]	Congenital [0.51]	SIDS [0.55]	LRI [0.73]	Endocrine [0.78]	Diarrhea [1.34]	Oth Neuro [0.78]	Meningitis [0.7]	Oth Un Inf [1.18]	F Body [0.28]
Portsmouth	Neonatal [0.56]	Congenital [0.37]	SIDS [0.71]	LRI [0.53]	Endocrine [0.69]	Diarrhea [0.97]	Meningitis [0.49]	Oth Neuro [0.6]	F Body [0.22]	URI [1.18]
Reading	Neonatal [0.99]	Congenital [0.62]	SIDS [0.53]	LRI [1.27]	Endocrine [0.93]	Diarrhea [1.56]	Oth Un Inf [1.41]	Meningitis [0.78]	Oth Neuro [0.68]	F Body [0.31]
Slough	Neonatal [0.89]	Congenital [0.64]	SIDS [0.56]	LRI [1.04]	Endocrine [0.87]	Diarrhea [1.28]	Meningitis [0.79]	F Body [0.42]	Oth Un Inf [1.12]	Oth Neuro [0.65]
Southampton	Neonatal [0.76]	Congenital [0.54]	SIDS [0.81]	LRI [0.64]	Endocrine [0.8]	Diarrhea [1.19]	F Body [0.37]	Oth Neuro [0.74]	Meningitis [0.5]	Oth Un Inf [0.91]
Surrey	Neonatal [0.66]	Congenital [0.47]	SIDS [0.47]	LRI [0.74]	Endocrine [0.61]	Diarrhea [1.26]	Oth Neuro [0.64]	Meningitis [0.54]	URI [1.25]	Oth Un Inf [0.9]
West Berkshire	Neonatal [0.64]	Congenital [0.48]	SIDS [0.52]	LRI [0.64]	Endocrine [0.66]	Diarrhea [1.16]	Meningitis [0.63]	Oth Un Inf [1.02]	F Body [0.28]	Oth Neuro [0.62]
W Sussex	Neonatal [0.56]	Congenital [0.42]	SIDS [0.6]	LRI [0.43]	Endocrine [0.61]	Diarrhea [0.89]	Oth Neuro [0.8]	Meningitis [0.47]	Oth Un Inf [0.87]	F Body [0.23]
Windsor & Maidenhead	Neonatal [0.8]	Congenital [0.58]	SIDS [0.55]	LRI [1.14]	Endocrine [0.72]	Diarrhea [1.58]	Oth Neuro [0.81]	F Body [0.36]	Oth Un Inf [1.17]	URI [1.29]
Wokingham	Neonatal [0.71]	Congenital [0.54]	SIDS [0.49]	LRI [0.84]	Endocrine [0.67]	Diarrhea [1.29]	Oth Un Inf [1.3]	Oth Neuro [0.75]	F Body [0.31]	Meningitis [0.56]
SW England	Neonatal [0.59]	Congenital [0.45]	SIDS [0.66]	LRI [0.43]	Endocrine [0.65]	Diarrhea [0.91]	Oth Neuro [0.75]	Meningitis [0.42]	F Body [0.25]	Oth Un Inf [0.87]
Bath & NE Somerset	Neonatal [0.51]	Congenital [0.41]	SIDS [0.62]	LRI [0.62]	Endocrine [0.68]	Diarrhea [1.62]	Oth Neuro [0.95]	Meningitis [0.61]	Oth Un Inf [0.98]	F Body [0.26]
Bournemouth	Neonatal [0.57]	Congenital [0.45]	SIDS [0.46]	LRI [0.56]	Endocrine [0.72]	F Body [0.37]	Oth Neuro [0.83]	Diarrhea [0.96]	Oth Un Inf [0.99]	Meningitis [0.4]
Bristol	Neonatal [0.79]	Congenital [0.57]	SIDS [0.85]	LRI [0.99]	Endocrine [0.82]	Diarrhea [1.78]	F Body [0.43]	Meningitis [0.87]	Oth Neuro [0.86]	Oth Un Inf [1.2]
Cornwall	Neonatal [0.64]	Congenital [0.47]	SIDS [0.73]	LRI [0.29]	Endocrine [0.65]	Diarrhea [0.57]	Oth Neuro [0.7]	Meningitis [0.33]	Oth Un Inf [0.81]	F Body [0.2]
Devon	Neonatal [0.66]	Congenital [0.48]	SIDS [0.63]	LRI [0.37]	Endocrine [0.68]	Diarrhea [0.81]	Oth Neuro [0.66]	Oth Un Inf [0.87]	Meningitis [0.37]	F Body [0.21]
Dorset	Neonatal [0.56]	Congenital [0.41]	SIDS [0.63]	LRI [0.3]	Endocrine [0.67]	Diarrhea [0.67]	F Body [0.24]	Oth Neuro [0.66]	Meningitis [0.34]	Oth Un Inf [0.79]
Gloucestershire	Neonatal [0.59]	Congenital [0.44]	SIDS [0.7]	LRI [0.55]	Endocrine [0.64]	Diarrhea [1.14]	Oth Neuro [0.83]	F Body [0.3]	Meningitis [0.5]	Oth Un Inf [0.94]
N Somerset	Neonatal [0.5]	Congenital [0.46]	SIDS [0.81]	LRI [0.37]	Endocrine [0.51]	Diarrhea [0.88]	Oth Neuro [0.93]	Meningitis [0.41]	F Body [0.24]	Oth Un Inf [0.79]
Plymouth	Neonatal [0.52]	Congenital [0.43]	SIDS [0.85]	LRI [0.42]	Endocrine [0.66]	Oth Neuro [0.82]	Diarrhea [0.76]	Meningitis [0.46]	Oth Un Inf [0.83]	F Body [0.22]
Poole	Neonatal [0.45]	Congenital [0.32]	SIDS [0.5]	LRI [0.32]	Endocrine [0.55]	Oth Un Inf [1.01]	Diarrhea [0.72]	F Body [0.24]	Oth Neuro [0.6]	Iron [4.84]
Somerset	Neonatal [0.53]	Congenital [0.43]	SIDS [0.58]	LRI [0.3]	Endocrine [0.62]	Diarrhea [0.69]	Oth Neuro [0.76]	Oth Un Inf [0.8]	F Body [0.2]	Meningitis [0.3]
S Gloucestershire	Neonatal [0.54]	Congenital [0.45]	SIDS [0.59]	LRI [0.53]	Endocrine [0.6]	Diarrhea [1.18]	Oth Neuro [0.78]	F Body [0.25]	Oth Un Inf [0.88]	Meningitis [0.45]
Swindon	Neonatal [0.52]	Congenital [0.38]	SIDS [0.53]	LRI [0.52]	Endocrine [0.55]	Diarrhea [1.0]	F Body [0.29]	Oth Neuro [0.66]	Meningitis [0.43]	Oth Un Inf [0.78]
Torbay	Neonatal [0.53]	Congenital [0.4]	SIDS [0.69]	LRI [0.18]	Endocrine [0.61]	Diarrhea [0.4]	Oth Neuro [0.58]	Oth Un Inf [0.65]	F Body [0.16]	Meningitis [0.19]
Wiltshire	Neonatal [0.52]	Congenital [0.4]	SIDS [0.63]	LRI [0.39]	Endocrine [0.59]	Diarrhea [0.82]	Meningitis [0.43]	Oth Neuro [0.72]	Oth Un Inf [0.76]	F Body [0.2]
W Midlands	Neonatal [1.07]	Congenital [0.8]	SIDS [0.78]	LRI [0.7]	Endocrine [1.14]	Diarrhea [1.13]	F Body [0.41]	Oth Neuro [1.06]	Meningitis [0.61]	Oth Un Inf [1.22]
Birmingham	Neonatal [1.49]	Congenital [1.12]	SIDS [0.94]	LRI [1.15]	Endocrine [1.62]	F Body [0.64]	Diarrhea [1.54]	Oth Neuro [1.45]	Meningitis [0.86]	Oth Un Inf [1.78]

eFigure 7b. Leading ten causes of DALYs with the ratio of observed DALYs to DALYs expected on the basis of Socio-Demographic Index alone in 2017, <1 years, both sexes combined. The top ten causes contributing to DALYs are listed globally, by socio-demographic quintile, and then by GBD superregion, region, country, and subnationally where modeled. For each cell, the ratio of observed DALYs to DALYs expected on the basis of socio-demographic index (SDI) alone are listed. Abbreviations: DALY=disability-adjusted life year, GBD=Global Burden of Disease.

Values shown in brackets represent the ratio of observed DALYs to predicted DALYs on the basis of Socio-Demographic Index (SDI), rounded to two (2) digits. Color ranges (shown below) were calculated to place a roughly equal number of cells into each bin.

COLOR KEY:	[0.0-0.33]	(0.33-0.46]	(0.46-0.58]	(0.58-0.7]	(0.7-0.84]	(0.84-1.03]	(1.03-1.38]	(1.38-2.46]	2.46+

	1	2	3	4	5	6	7	8	9	10
Coventry	Neonatal [1.09]	Congenital [0.79]	SIDS [0.92]	LRI [0.91]	Endocrine [1.25]	Meningitis [1.09]	Diarrhea [1.65]	Oth Neuro [1.31]	F Body [0.51]	CMP [1.18]
Dudley	Neonatal [0.74]	Congenital [0.62]	SIDS [0.66]	LRI [0.36]	Endocrine [0.99]	Diarrhea [0.78]	Meningitis [0.38]	Oth Un Inf [0.94]	Oth Neuro [0.75]	F Body [0.22]
Herefordshire	Neonatal [0.69]	Congenital [0.6]	SIDS [0.74]	LRI [0.44]	Endocrine [0.79]	Diarrhea [0.96]	F Body [0.34]	Oth Neuro [0.91]	Oth Un Inf [0.94]	Meningitis [0.39]
Sandwell	Neonatal [0.99]	Congenital [0.74]	SIDS [0.78]	LRI [0.47]	Endocrine [1.33]	Diarrhea [0.79]	F Body [0.36]	Meningitis [0.4]	Oth Un Inf [1.01]	Oth Neuro [0.81]
Shropshire	Neonatal [0.59]	Congenital [0.45]	SIDS [0.47]	LRI [0.31]	Endocrine [0.47]	Diarrhea [0.68]	Oth Neuro [0.64]	Meningitis [0.3]	URI [1.17]	Oth Un Inf [0.58]
Solihull	Neonatal [0.89]	Congenital [0.67]	SIDS [0.56]	LRI [0.6]	Endocrine [0.83]	Diarrhea [1.3]	F Body [0.33]	Oth Un Inf [1.13]	Oth Neuro [0.71]	Meningitis [0.5]
Staffordshire	Neonatal [0.96]	Congenital [0.7]	SIDS [0.7]	LRI [0.53]	Endocrine [0.88]	Diarrhea [0.97]	Oth Neuro [0.94]	F Body [0.33]	Oth Un Inf [1.15]	Meningitis [0.48]
Stoke-on-Trent	Neonatal [1.17]	Congenital [0.9]	SIDS [1.11]	LRI [0.55]	Endocrine [1.06]	Meningitis [0.65]	Oth Neuro [1.25]	Diarrhea [0.78]	F Body [0.31]	CMP [0.98]
Telford & Wrekin	Neonatal [0.87]	Congenital [0.65]	SIDS [0.88]	LRI [0.56]	Endocrine [0.82]	Meningitis [0.71]	Oth Neuro [1.1]	F Body [0.35]	Diarrhea [0.8]	Oth Un Inf [0.85]
Walsall	Neonatal [1.0]	Congenital [0.77]	SIDS [0.68]	LRI [0.49]	Endocrine [1.31]	F Body [0.42]	Meningitis [0.49]	Oth Neuro [1.19]	Diarrhea [0.69]	Oth Un Inf [1.2]
Warwickshire	Neonatal [0.92]	Congenital [0.66]	SIDS [0.49]	LRI [0.7]	Endocrine [0.87]	Diarrhea [1.4]	Oth Neuro [0.73]	Oth Un Inf [1.07]	F Body [0.28]	Meningitis [0.49]
Wolverhampton	Neonatal [0.93]	Congenital [0.69]	SIDS [0.85]	LRI [0.54]	Endocrine [1.04]	Diarrhea [0.91]	F Body [0.38]	Oth Neuro [0.95]	Meningitis [0.46]	Oth Un Inf [0.95]
Worcestershire	Neonatal [0.83]	Congenital [0.65]	SIDS [0.74]	LRI [0.62]	Endocrine [0.91]	Diarrhea [1.0]	F Body [0.39]	Oth Neuro [0.98]	Meningitis [0.55]	Oth Un Inf [1.04]
Yorkshire & Humber	Neonatal [0.66]	Congenital [0.52]	SIDS [0.71]	LRI [0.54]	Endocrine [0.77]	Oth Neuro [0.98]	Diarrhea [0.85]	Meningitis [0.52]	F Body [0.28]	Oth Un Inf [0.91]
Barnsley	Neonatal [0.41]	Congenital [0.34]	SIDS [0.79]	LRI [0.27]	Endocrine [0.52]	Oth Neuro [1.04]	Meningitis [0.38]	Diarrhea [0.57]	F Body [0.23]	Iron [2.93]
Bradford	Neonatal [0.74]	Congenital [0.62]	SIDS [0.8]	LRI [0.53]	Endocrine [0.93]	Oth Neuro [1.58]	Meningitis [0.46]	F Body [0.31]	Diarrhea [0.71]	Oth Un Inf [0.97]
Calderdale	Neonatal [0.63]	Congenital [0.47]	SIDS [0.9]	LRI [0.53]	Endocrine [0.86]	Meningitis [0.58]	F Body [0.33]	Diarrhea [0.82]	Oth Neuro [0.87]	Oth Un Inf [0.83]
Doncaster	Neonatal [0.6]	Congenital [0.51]	SIDS [0.76]	LRI [0.34]	Endocrine [0.73]	Oth Neuro [1.0]	Meningitis [0.35]	Diarrhea [0.51]	Oth Un Inf [0.78]	F Body [0.2]
E Riding of Yorkshire	Neonatal [0.44]	Congenital [0.39]	SIDS [0.6]	LRI [0.34]	Endocrine [0.59]	Oth Neuro [0.76]	Diarrhea [0.64]	Meningitis [0.36]	F Body [0.21]	Iron [4.35]
Kingston upon Hull	Neonatal [0.55]	Congenital [0.4]	SIDS [0.71]	LRI [0.41]	Endocrine [0.62]	Oth Neuro [0.92]	F Body [0.3]	Meningitis [0.41]	Diarrhea [0.64]	Iron [4.6]
Kirklees	Neonatal [0.69]	Congenital [0.56]	SIDS [0.68]	LRI [0.48]	Endocrine [0.82]	Oth Neuro [0.86]	Diarrhea [0.67]	F Body [0.27]	Meningitis [0.39]	Oth Un Inf [0.8]
Leeds	Neonatal [0.85]	Congenital [0.6]	SIDS [0.74]	LRI [0.92]	Endocrine [0.93]	Diarrhea [1.44]	Meningitis [0.88]	Oth Un Inf [1.41]	Oth Neuro [0.85]	F Body [0.35]
NE Lincolnshire	Neonatal [0.49]	Congenital [0.38]	SIDS [0.8]	LRI [0.35]	Endocrine [0.73]	Oth Neuro [1.08]	F Body [0.24]	Diarrhea [0.48]	Meningitis [0.29]	Oth Un Inf [0.77]
N Lincolnshire	Neonatal [0.53]	Congenital [0.4]	SIDS [0.65]	LRI [0.37]	Endocrine [0.68]	Diarrhea [0.66]	Oth Neuro [0.86]	F Body [0.27]	Meningitis [0.34]	Iron [3.44]
N Yorkshire	Neonatal [0.49]	Congenital [0.4]	SIDS [0.64]	LRI [0.38]	Endocrine [0.58]	Diarrhea [0.8]	Oth Neuro [0.7]	Meningitis [0.4]	Oth Un Inf [0.75]	URI [1.22]
Rotherham	Neonatal [0.55]	Congenital [0.45]	SIDS [0.57]	LRI [0.34]	Endocrine [0.58]	Meningitis [0.42]	Oth Neuro [0.82]	F Body [0.23]	Diarrhea [0.45]	Iron [2.76]
Sheffield	Neonatal [0.82]	Congenital [0.66]	SIDS [0.66]	LRI [0.76]	Endocrine [0.9]	Diarrhea [1.3]	Oth Neuro [1.11]	Meningitis [0.79]	F Body [0.35]	Oth Un Inf [1.16]
Wakefield	Neonatal [0.55]	Congenital [0.41]	SIDS [0.63]	LRI [0.39]	Endocrine [0.64]	Diarrhea [0.69]	Oth Neuro [0.77]	Meningitis [0.34]	F Body [0.23]	Oth Un Inf [0.65]
York	Neonatal [0.71]	Congenital [0.51]	SIDS [0.74]	LRI [0.81]	Endocrine [0.6]	Oth Neuro [1.03]	Diarrhea [1.22]	Meningitis [0.77]	F Body [0.33]	Oth Un Inf [1.12]
N Ireland	Neonatal [0.84]	Congenital [0.74]	SIDS [0.54]	LRI [0.48]	Endocrine [0.67]	Diarrhea [1.03]	Meningitis [0.49]	F Body [0.27]	Oth Un Inf [0.95]	ALS [2.29]
Scotland	Neonatal [0.5]	Congenital [0.43]	SIDS [0.76]	LRI [0.29]	Endocrine [0.57]	Diarrhea [0.68]	Meningitis [0.37]	F Body [0.24]	Oth Neuro [0.68]	Oth Un Inf [0.79]

eFigure 7b. Leading ten causes of DALYs with the ratio of observed DALYs to DALYs expected on the basis of Socio-Demographic Index alone in 2017, <1 years, both sexes combined. The top ten causes contributing to DALYs are listed globally, by socio-demographic quintile, and then by GBD superregion, region, country, and subnationally where modeled. For each cell, the ratio of observed DALYs to DALYs expected on the basis of socio-demographic index (SDI) alone are listed. Abbreviations: DALY=disability-adjusted life year, GBD=Global Burden of Disease.

Values shown in brackets represent the ratio of observed DALYs to predicted DALYs on the basis of Socio-Demographic Index (SDI), rounded to two (2) digits. Color ranges (shown below) were calculated to place a roughly equal number of cells into each bin.

COLOR KEY:									
	[0.0-0.33]	(0.33-0.46]	(0.46-0.58]	(0.58-0.7]	(0.7-0.84]	(0.84-1.03]	(1.03-1.38]	(1.38-2.46]	2.46+

	1	2	3	4	5	6	7	8	9	10
Wales	Neonatal [0.49]	Congenital [0.39]	SIDS [0.6]	LRI [0.31]	Endocrine [0.56]	Diarrhea [0.69]	Meningitis [0.36]	F Body [0.22]	Oth Neuro [0.67]	Oth Un Inf [0.77]
**Latin America and Caribbean**	Neonatal [0.63]	Congenital [0.67]	LRI [0.6]	Diarrhea [1.34]	STI [3.06]	F Body [0.93]	PEM [2.61]	Meningitis [0.37]	Ileus [1.21]	SIDS [0.32]
**Andean Latin America**	Neonatal [0.57]	Congenital [0.59]	LRI [0.7]	F Body [1.72]	STI [3.25]	Diarrhea [0.63]	PEM [1.84]	HIV [3.43]	Whooping [2.23]	Meningitis [0.21]
Bolivia	Neonatal [0.69]	Congenital [0.71]	LRI [0.69]	STI [3.58]	F Body [1.58]	Diarrhea [0.46]	PEM [1.89]	Meningitis [0.24]	Whooping [1.52]	HIV [2.59]
Ecuador	Neonatal [0.58]	Congenital [0.6]	LRI [0.59]	STI [4.63]	F Body [0.86]	Diarrhea [0.49]	PEM [1.75]	Ileus [0.86]	Meningitis [0.19]	Endocrine [0.67]
Peru	Neonatal [0.47]	Congenital [0.51]	LRI [0.65]	F Body [2.08]	Diarrhea [0.66]	STI [1.81]	HIV [4.55]	Whooping [2.88]	Iron [3.82]	PEM [1.2]
**Caribbean**	Neonatal [1.17]	Congenital [0.93]	LRI [1.43]	Diarrhea [6.13]	STI [7.23]	Meningitis [1.48]	Whooping [9.69]	PEM [4.64]	F Body [0.9]	Endocrine [1.63]
Antigua	Neonatal [0.71]	Congenital [0.43]	LRI [0.87]	Endocrine [1.94]	Diarrhea [0.89]	STI [2.71]	Meningitis [0.44]	SIDS [0.3]	PEM [4.94]	F Body [0.34]
Bahamas	Neonatal [1.01]	Congenital [0.57]	LRI [1.85]	Endocrine [3.96]	STI [8.01]	F Body [0.96]	HIV [8.57]	Diarrhea [1.34]	SIDS [0.41]	Meningitis [0.75]
Barbados	Neonatal [1.1]	Congenital [0.69]	LRI [1.15]	STI [8.19]	Endocrine [1.78]	Diarrhea [0.83]	Meningitis [0.59]	Bac Skin [17.15]	Urinary [8.3]	SIDS [0.23]
Belize	Neonatal [0.68]	Congenital [0.49]	LRI [0.46]	Diarrhea [0.58]	Endocrine [2.16]	SIDS [0.8]	PEM [1.43]	F Body [0.52]	STI [0.92]	HIV [2.77]
Bermuda	Neonatal [0.56]	Congenital [0.29]	LRI [0.59]	STI [7.99]	SIDS [0.38]	Endocrine [1.22]	Diarrhea [0.65]	HIV [2.45]	Meningitis [0.28]	Iron [3.33]
Cuba	Neonatal [0.21]	Congenital [0.27]	LRI [0.25]	Endocrine [0.63]	F Body [0.26]	Diarrhea [0.28]	Meningitis [0.22]	STI [0.78]	SIDS [0.1]	HIV [1.08]
Dominica	Neonatal [2.11]	Congenital [1.36]	LRI [1.78]	SIDS [2.24]	Endocrine [4.53]	Diarrhea [1.58]	F Body [1.07]	PEM [7.89]	Meningitis [0.81]	Bac Skin [20.06]
Dominican Rep	Neonatal [1.13]	Congenital [0.57]	LRI [0.39]	STI [5.28]	Diarrhea [0.67]	Meningitis [0.44]	Whooping [2.72]	F Body [0.46]	SIDS [0.55]	HIV [2.85]
Grenada	Neonatal [0.62]	Congenital [0.34]	LRI [0.44]	Endocrine [1.25]	STI [0.87]	Diarrhea [0.21]	F Body [0.26]	PEM [1.04]	Iron [2.46]	Meningitis [0.12]
Guyana	Neonatal [0.92]	Congenital [0.4]	LRI [0.33]	Diarrhea [0.75]	PEM [1.75]	STI [1.2]	F Body [0.62]	Endocrine [1.65]	HIV [2.91]	Ileus [0.88]
Haiti	Neonatal [0.64]	LRI [0.74]	Diarrhea [1.46]	Congenital [1.12]	Meningitis [0.82]	STI [1.42]	Whooping [1.39]	PEM [0.77]	F Body [1.32]	Tetanus [10.88]
Jamaica	Neonatal [1.37]	Congenital [0.57]	STI [11.53]	LRI [0.32]	SIDS [0.46]	Diarrhea [0.5]	PEM [2.85]	Endocrine [0.9]	Meningitis [0.28]	F Body [0.31]
Puerto Rico	Neonatal [1.15]	Congenital [0.7]	LRI [1.54]	Disaster [7219.55]	Endocrine [1.8]	STI [10.49]	Diarrhea [2.5]	F Body [0.63]	SIDS [0.15]	Meningitis [0.59]
St Lucia	Neonatal [0.83]	Congenital [0.38]	STI [12.39]	LRI [0.33]	Diarrhea [0.46]	Endocrine [1.06]	SIDS [0.39]	PEM [1.36]	F Body [0.25]	Meningitis [0.19]
St Vincent	Neonatal [0.76]	Congenital [0.46]	LRI [0.36]	Diarrhea [0.5]	Endocrine [2.41]	PEM [2.14]	STI [1.13]	F Body [0.37]	Meningitis [0.25]	HIV [1.8]
Suriname	Neonatal [1.47]	Congenital [0.81]	LRI [0.71]	Diarrhea [2.22]	STI [3.5]	F Body [1.11]	Endocrine [2.04]	PEM [2.94]	Meningitis [0.51]	Ileus [2.08]
Trinidad Tobago	Neonatal [1.24]	Congenital [0.92]	LRI [0.76]	F Body [1.05]	Diarrhea [1.14]	Endocrine [1.99]	STI [3.19]	SIDS [0.33]	Meningitis [0.31]	Ileus [1.14]
Virgin Isl US	Neonatal [1.19]	Congenital [0.64]	LRI [1.1]	SIDS [0.71]	STI [10.73]	Endocrine [1.56]	Violence [2.25]	F Body [0.53]	Diarrhea [1.11]	PEM [14.5]
**Central Latin America**	Neonatal [0.5]	Congenital [0.56]	LRI [0.46]	Diarrhea [0.8]	F Body [0.75]	PEM [1.95]	STI [1.39]	Ileus [1.11]	SIDS [0.38]	Meningitis [0.19]
Colombia	Neonatal [0.55]	Congenital [0.56]	LRI [0.37]	PEM [3.34]	Diarrhea [0.51]	F Body [0.58]	STI [1.32]	Meningitis [0.25]	Ileus [0.87]	Violence [1.17]
Costa Rica	Neonatal [0.41]	Congenital [0.53]	LRI [0.14]	Diarrhea [0.36]	STI [1.1]	Meningitis [0.15]	F Body [0.13]	Endocrine [0.31]	SIDS [0.12]	Oth Un Inf [0.44]
El Salvador	Neonatal [0.37]	Congenital [0.55]	LRI [0.29]	Diarrhea [0.53]	HIV [3.62]	Whooping [1.51]	PEM [0.64]	Meningitis [0.16]	SIDS [0.31]	STI [0.41]
Guatemala	Neonatal [0.49]	LRI [0.74]	Congenital [0.51]	Diarrhea [0.65]	PEM [0.98]	F Body [0.7]	SIDS [0.91]	Ileus [1.33]	Meningitis [0.19]	STI [0.45]
Honduras	Neonatal [0.32]	Congenital [0.18]	Diarrhea [0.33]	LRI [0.07]	Endocrine [3.33]	Ileus [1.19]	Whooping [0.53]	Violence [2.24]	F Body [0.26]	Encepha [0.72]

eFigure 7b. Leading ten causes of DALYs with the ratio of observed DALYs to DALYs expected on the basis of Socio-Demographic Index alone in 2017, <1 years, both sexes combined. The top ten causes contributing to DALYs are listed globally, by socio-demographic quintile, and then by GBD superregion, region, country, and subnationally where modeled. For each cell, the ratio of observed DALYs to DALYs expected on the basis of socio-demographic index (SDI) alone are listed. Abbreviations: DALY=disability-adjusted life year, GBD=Global Burden of Disease.

Values shown in brackets represent the ratio of observed DALYs to predicted DALYs on the basis of Socio-Demographic Index (SDI), rounded to two (2) digits. Color ranges (shown below) were calculated to place a roughly equal number of cells into each bin.

COLOR KEY:									
[0.0-0.33]	(0.33-0.46]	(0.46-0.58]	(0.58-0.7]	(0.7-0.84]	(0.84-1.03]	(1.03-1.38]	(1.38-2.46]	2.46+	

	1	2	3	4	5	6	7	8	9	10
Mexico	Neonatal [0.43]	Congenital [0.59]	LRI [0.37]	Diarrhea [0.62]	F Body [0.87]	PEM [1.69]	Ileus [1.21]	SIDS [0.43]	STI [0.88]	Violence [1.91]
Aguascalientes	Neonatal [0.51]	Congenital [0.7]	LRI [0.36]	F Body [1.26]	Diarrhea [0.84]	PEM [2.42]	Ileus [1.12]	STI [0.99]	SIDS [0.31]	Violence [1.48]
Baja CA	Neonatal [0.37]	Congenital [0.49]	LRI [0.32]	F Body [1.59]	Diarrhea [0.42]	STI [1.31]	Violence [1.96]	SIDS [0.32]	Ileus [0.9]	Meningitis [0.17]
Baja CA Sur	Neonatal [0.43]	Congenital [0.53]	LRI [0.33]	F Body [0.63]	Diarrhea [0.53]	SIDS [0.31]	PEM [1.61]	Violence [1.72]	STI [0.81]	Ileus [0.67]
Campeche	Neonatal [0.49]	Congenital [0.54]	LRI [0.27]	Diarrhea [0.46]	F Body [0.53]	PEM [1.23]	SIDS [0.44]	Ileus [0.99]	STI [0.66]	Violence [1.57]
Chiapas	Neonatal [0.17]	Congenital [0.36]	LRI [0.27]	Diarrhea [0.45]	PEM [0.73]	F Body [0.41]	Ileus [0.98]	STI [0.34]	SIDS [0.28]	Violence [1.2]
Chihuahua	Neonatal [0.66]	Congenital [0.75]	LRI [0.52]	F Body [2.16]	Diarrhea [0.7]	SIDS [0.63]	Violence [3.03]	PEM [2.03]	STI [1.27]	Ileus [1.15]
Coahuila	Neonatal [0.66]	Congenital [0.69]	LRI [0.39]	F Body [1.31]	Diarrhea [0.57]	SIDS [0.4]	STI [1.02]	PEM [1.53]	Violence [1.83]	Ileus [0.99]
Colima	Neonatal [0.47]	Congenital [0.62]	LRI [0.35]	Diarrhea [0.76]	F Body [0.61]	PEM [2.67]	STI [1.19]	Violence [2.2]	SIDS [0.35]	Ileus [1.14]
Mexico City	Neonatal [0.82]	Congenital [1.06]	LRI [1.43]	F Body [1.11]	Diarrhea [1.39]	Ileus [2.66]	Violence [2.81]	STI [2.32]	Meningitis [0.32]	PEM [3.72]
Durango	Neonatal [0.57]	Congenital [0.62]	LRI [0.28]	F Body [1.01]	Diarrhea [0.41]	SIDS [0.46]	Violence [2.24]	STI [0.87]	PEM [0.98]	Ileus [0.87]
Guanajuato	Neonatal [0.41]	Congenital [0.57]	LRI [0.24]	Diarrhea [0.4]	F Body [0.58]	STI [0.9]	SIDS [0.46]	PEM [1.08]	Ileus [0.84]	Violence [1.46]
Guerrero	Neonatal [0.27]	Congenital [0.54]	LRI [0.22]	Diarrhea [0.38]	F Body [0.69]	PEM [0.95]	Violence [2.75]	SIDS [0.56]	Ileus [0.91]	STI [0.44]
Hidalgo	Neonatal [0.27]	Congenital [0.36]	LRI [0.14]	F Body [0.75]	SIDS [0.78]	Diarrhea [0.2]	Ileus [0.92]	PEM [0.57]	STI [0.37]	Violence [1.3]
Jalisco	Neonatal [0.43]	Congenital [0.64]	LRI [0.34]	F Body [0.77]	Diarrhea [0.49]	STI [1.11]	PEM [1.67]	Ileus [1.06]	Violence [1.72]	SIDS [0.21]
Mexico	Neonatal [0.47]	Congenital [0.57]	LRI [0.45]	F Body [1.14]	Diarrhea [0.61]	Ileus [1.7]	PEM [1.56]	Violence [2.23]	STI [0.95]	SIDS [0.23]
Michoacan	Neonatal [0.27]	Congenital [0.56]	LRI [0.28]	F Body [1.04]	Diarrhea [0.39]	PEM [1.06]	SIDS [0.51]	Ileus [1.03]	STI [0.6]	Violence [2.18]
Morelos	Neonatal [0.39]	Congenital [0.53]	LRI [0.21]	Diarrhea [0.52]	F Body [0.51]	PEM [1.43]	Violence [2.04]	Ileus [0.98]	STI [0.79]	SIDS [0.32]
Nayarit	Neonatal [0.29]	Congenital [0.47]	LRI [0.21]	Diarrhea [0.38]	F Body [0.42]	STI [0.86]	SIDS [0.38]	Violence [1.8]	PEM [0.78]	Ileus [0.69]
Nuevo Leon	Neonatal [0.51]	Congenital [0.75]	LRI [0.41]	F Body [1.38]	Diarrhea [0.48]	STI [1.43]	Violence [1.88]	Meningitis [0.21]	Ileus [0.87]	SIDS [0.21]
Oaxaca	Neonatal [0.2]	Congenital [0.45]	LRI [0.2]	Diarrhea [0.39]	PEM [1.18]	F Body [0.53]	Ileus [0.88]	STI [0.44]	Violence [1.82]	SIDS [0.3]
Puebla	Neonatal [0.47]	Congenital [0.68]	LRI [0.38]	Diarrhea [0.44]	SIDS [1.2]	F Body [0.83]	PEM [1.37]	Ileus [1.73]	STI [0.52]	Violence [1.56]
Queretaro	Neonatal [0.42]	Congenital [0.64]	LRI [0.32]	F Body [1.06]	Diarrhea [0.6]	SIDS [0.5]	PEM [1.78]	Ileus [1.29]	STI [0.72]	Violence [1.49]
Quintana Roo	Neonatal [0.4]	Congenital [0.51]	LRI [0.27]	Diarrhea [0.54]	F Body [0.88]	PEM [1.18]	Ileus [0.98]	Violence [1.73]	STI [0.63]	HIV [1.55]
San Luis Potosi	Neonatal [0.36]	Congenital [0.73]	LRI [0.37]	Diarrhea [0.61]	F Body [0.93]	SIDS [0.95]	PEM [1.78]	Ileus [1.16]	STI [0.85]	Violence [1.8]
Sinaloa	Neonatal [0.4]	Congenital [0.55]	LRI [0.31]	Diarrhea [0.47]	F Body [0.48]	STI [1.16]	Violence [2.32]	PEM [1.22]	SIDS [0.25]	Ileus [0.54]
Sonora	Neonatal [0.42]	Congenital [0.53]	LRI [0.36]	Diarrhea [0.67]	F Body [0.82]	SIDS [0.6]	PEM [1.87]	STI [0.96]	Violence [1.8]	Ileus [0.8]
Tabasco	Neonatal [0.63]	Congenital [0.75]	LRI [0.34]	Diarrhea [0.59]	F Body [0.75]	PEM [1.76]	Ileus [0.91]	STI [0.65]	Violence [1.74]	SIDS [0.34]
Tamaulipas	Neonatal [0.53]	Congenital [0.61]	LRI [0.29]	F Body [0.92]	Diarrhea [0.45]	STI [1.05]	Violence [2.06]	PEM [1.34]	Ileus [0.78]	SIDS [0.25]
Tlaxcala	Neonatal [0.41]	Congenital [0.56]	LRI [0.35]	SIDS [1.51]	F Body [0.93]	Diarrhea [0.39]	PEM [1.29]	Ileus [1.3]	STI [0.73]	Violence [1.46]
Veracruz	Neonatal [0.34]	Congenital [0.44]	LRI [0.16]	Diarrhea [0.26]	PEM [0.97]	F Body [0.42]	Ileus [0.76]	STI [0.47]	Violence [1.48]	SIDS [0.29]

eFigure 7b. Leading ten causes of DALYs with the ratio of observed DALYs to DALYs expected on the basis of Socio-Demographic Index alone in 2017, <1 years, both sexes combined. The top ten causes contributing to DALYs are listed globally, by socio-demographic quintile, and then by GBD superregion, region, country, and subnationally where modeled. For each cell, the ratio of observed DALYs to DALYs expected on the basis of socio-demographic index (SDI) alone are listed. Abbreviations: DALY=disability-adjusted life year, GBD=Global Burden of Disease.

Values shown in brackets represent the ratio of observed DALYs to predicted DALYs on the basis of Socio-Demographic Index (SDI), rounded to two (2) digits. Color ranges (shown below) were calculated to place a roughly equal number of cells into each bin.

COLOR KEY:	[0.0-0.33]	(0.33-0.46]	(0.46-0.58]	(0.58-0.7]	(0.7-0.84]	(0.84-1.03]	(1.03-1.38]	(1.38-2.46]	2.46+

	1	2	3	4	5	6	7	8	9	10
Yucatan	Neonatal [0.42]	Congenital [0.63]	LRI [0.45]	Diarrhea [0.68]	F Body [0.62]	PEM [1.8]	Ileus [1.24]	SIDS [0.4]	STI [0.75]	Violence [1.43]
Zacatecas	Neonatal [0.34]	Congenital [0.47]	LRI [0.28]	F Body [0.78]	Diarrhea [0.33]	SIDS [0.82]	PEM [0.98]	STI [0.55]	Ileus [0.73]	Violence [1.55]
Nicaragua	Neonatal [0.36]	Congenital [0.5]	LRI [0.29]	Diarrhea [0.25]	PEM [0.62]	F Body [0.39]	Meningitis [0.11]	SIDS [0.26]	HIV [1.18]	Ileus [0.41]
Panama	Neonatal [0.63]	Congenital [0.85]	LRI [0.91]	Diarrhea [1.94]	STI [6.97]	F Body [0.92]	PEM [6.28]	Meningitis [0.53]	Ileus [1.33]	HIV [2.34]
Venezuela	Neonatal [0.73]	Congenital [0.57]	LRI [0.49]	STI [6.7]	Diarrhea [1.38]	F Body [1.12]	PEM [2.77]	Meningitis [0.31]	Ileus [0.61]	Violence [1.03]
**Tropical Latin America**	Neonatal [0.74]	Congenital [0.82]	LRI [0.55]	STI [4.99]	Diarrhea [1.24]	F Body [0.86]	PEM [3.44]	Meningitis [0.46]	Ileus [1.54]	HIV [2.5]
Brazil	Neonatal [0.78]	Congenital [0.85]	LRI [0.59]	Diarrhea [1.33]	STI [4.69]	F Body [0.89]	PEM [3.66]	Meningitis [0.48]	Ileus [1.6]	CMP [1.28]
Acre	Neonatal [0.76]	Congenital [0.87]	LRI [0.51]	Diarrhea [0.98]	STI [2.05]	PEM [2.01]	Ileus [1.48]	Meningitis [0.3]	F Body [0.41]	Oth Un Inf [0.98]
Alagoas	Neonatal [0.44]	Congenital [0.57]	LRI [0.32]	Diarrhea [0.78]	STI [1.47]	PEM [1.37]	Ileus [1.24]	Meningitis [0.19]	HIV [1.8]	Violence [1.67]
Amapa	Neonatal [1.32]	Congenital [0.86]	LRI [0.7]	STI [8.59]	Diarrhea [1.38]	PEM [3.26]	Meningitis [0.43]	SIDS [0.48]	Ileus [1.58]	Bac Skin [11.94]
Amazonas	Neonatal [0.72]	Congenital [0.79]	LRI [0.55]	STI [6.11]	Diarrhea [1.46]	PEM [3.24]	Ileus [1.47]	Meningitis [0.32]	F Body [0.3]	Endocrine [0.6]
Bahia	Neonatal [0.63]	Congenital [0.91]	LRI [0.44]	Diarrhea [0.95]	PEM [2.42]	F Body [1.0]	Meningitis [0.53]	STI [1.34]	Ileus [1.93]	CMP [1.86]
Ceara	Neonatal [0.5]	Congenital [0.74]	LRI [0.33]	Diarrhea [0.67]	STI [1.93]	Meningitis [0.34]	PEM [1.18]	F Body [0.43]	Ileus [1.11]	HIV [2.25]
Distrito Federal	Neonatal [1.59]	Congenital [1.24]	LRI [1.56]	STI [19.37]	F Body [1.67]	Diarrhea [2.75]	Meningitis [1.05]	HIV [6.63]	PEM [16.52]	Ileus [2.06]
Espirito Santo	Neonatal [0.79]	Congenital [0.93]	LRI [0.45]	STI [5.6]	Diarrhea [0.95]	F Body [0.72]	PEM [3.46]	Meningitis [0.43]	Ileus [1.07]	Violence [1.6]
Goias	Neonatal [0.78]	Congenital [0.79]	LRI [0.32]	STI [4.23]	Diarrhea [0.54]	F Body [0.53]	Meningitis [0.35]	Ileus [0.96]	PEM [1.11]	Endocrine [0.54]
Maranhao	Neonatal [0.33]	Congenital [0.58]	LRI [0.22]	Diarrhea [0.28]	STI [0.87]	PEM [0.61]	F Body [0.41]	Meningitis [0.15]	Ileus [0.93]	HIV [1.91]
Mato Grosso	Neonatal [0.82]	Congenital [0.77]	LRI [0.49]	STI [7.2]	Diarrhea [1.14]	F Body [0.84]	PEM [3.25]	Meningitis [0.44]	Ileus [1.49]	SIDS [0.43]
Mato Grosso do Sul	Neonatal [0.71]	Congenital [0.85]	LRI [0.44]	Diarrhea [1.39]	STI [4.08]	F Body [0.94]	PEM [2.58]	Meningitis [0.37]	Ileus [1.26]	Endocrine [0.83]
Minas Gerais	Neonatal [0.75]	Congenital [0.75]	LRI [0.3]	STI [4.18]	Diarrhea [0.64]	F Body [0.62]	Meningitis [0.34]	Ileus [1.17]	PEM [1.64]	Endocrine [0.65]
Para	Neonatal [0.5]	Congenital [0.56]	LRI [0.37]	Diarrhea [0.44]	STI [1.65]	PEM [0.94]	Meningitis [0.24]	Ileus [0.99]	F Body [0.26]	HIV [1.88]
Paraiba	Neonatal [0.32]	Congenital [0.51]	LRI [0.26]	STI [1.48]	Diarrhea [0.27]	PEM [0.93]	Meningitis [0.18]	Ileus [0.78]	F Body [0.21]	HIV [1.6]
Parana	Neonatal [0.85]	Congenital [1.07]	LRI [0.45]	F Body [1.6]	STI [5.59]	Diarrhea [0.8]	Meningitis [0.54]	Ileus [1.47]	PEM [2.71]	Road Inj [1.41]
Pernambuco	Neonatal [0.53]	Congenital [0.81]	LRI [0.34]	Diarrhea [0.88]	STI [1.93]	PEM [1.66]	F Body [0.63]	Meningitis [0.28]	Ileus [1.25]	Violence [1.85]
Piaui	Neonatal [0.52]	Congenital [0.62]	LRI [0.17]	Diarrhea [0.33]	STI [1.37]	PEM [0.55]	F Body [0.28]	Meningitis [0.13]	Ileus [0.71]	HIV [1.77]
Rio de Janeiro	Neonatal [1.03]	Congenital [0.9]	LRI [0.91]	F Body [2.57]	STI [6.49]	Diarrhea [1.13]	Meningitis [0.61]	Ileus [1.56]	PEM [3.91]	Violence [1.74]
Rio Grande do Norte	Neonatal [0.37]	Congenital [0.66]	LRI [0.41]	Diarrhea [0.66]	STI [2.18]	PEM [2.67]	Meningitis [0.36]	F Body [0.49]	Ileus [1.18]	Urinary [3.51]
Rio Grande do Sul	Neonatal [0.83]	Congenital [0.9]	STI [14.46]	LRI [0.52]	F Body [1.0]	Diarrhea [0.62]	Meningitis [0.39]	Ileus [1.08]	Endocrine [0.54]	HIV [1.97]
Rondonia	Neonatal [0.65]	Congenital [0.78]	LRI [0.34]	STI [4.38]	Diarrhea [0.73]	F Body [0.45]	PEM [1.38]	Meningitis [0.29]	Ileus [1.0]	Bac Skin [6.32]
Roraima	Neonatal [0.7]	Congenital [0.83]	LRI [0.81]	Diarrhea [1.78]	STI [3.98]	PEM [5.2]	F Body [0.63]	Ileus [1.4]	Endocrine [1.0]	HIV [3.21]
Santa Catarina	Neonatal [0.93]	Congenital [0.97]	LRI [0.57]	STI [7.17]	F Body [0.97]	Diarrhea [1.04]	Meningitis [0.59]	SIDS [0.42]	Ileus [1.48]	Road Inj [1.57]

eFigure 7b. Leading ten causes of DALYs with the ratio of observed DALYs to DALYs expected on the basis of Socio-Demographic Index alone in 2017, <1 years, both sexes combined. The top ten causes contributing to DALYs are listed globally, by socio-demographic quintile, and then by GBD superregion, region, country, and subnationally where modeled. For each cell, the ratio of observed DALYs to DALYs expected on the basis of socio-demographic index (SDI) alone are listed. Abbreviations: DALY=disability-adjusted life year, GBD=Global Burden of Disease.

Values shown in brackets represent the ratio of observed DALYs to predicted DALYs on the basis of Socio-Demographic Index (SDI), rounded to two (2) digits. Color ranges (shown below) were calculated to place a roughly equal number of cells into each bin.

COLOR KEY:	[0.0-0.33]	(0.33-0.46]	(0.46-0.58]	(0.58-0.7]	(0.7-0.84]	(0.84-1.03]	(1.03-1.38]	(1.38-2.46]	2.46+

	1	2	3	4	5	6	7	8	9	10
Sao Paulo	Neonatal [1.05]	Congenital [0.95]	LRI [0.79]	F Body [1.57]	STI [7.54]	Diarrhea [0.96]	Meningitis [0.66]	Ileus [1.86]	CMP [1.68]	Endocrine [0.67]
Sergipe	Neonatal [0.71]	Congenital [0.69]	LRI [0.43]	Diarrhea [0.91]	STI [2.32]	PEM [2.05]	F Body [0.61]	Meningitis [0.3]	Ileus [1.15]	HIV [2.08]
Tocantins	Neonatal [0.59]	Congenital [0.68]	LRI [0.29]	Diarrhea [0.63]	STI [2.4]	PEM [1.35]	Meningitis [0.23]	F Body [0.31]	Leish [207.89]	Ileus [0.85]
Paraguay	Neonatal [0.29]	Congenital [0.49]	STI [10.53]	LRI [0.28]	Diarrhea [0.61]	PEM [2.76]	HIV [4.88]	F Body [0.52]	Meningitis [0.33]	Ileus [1.19]
**North Africa and Middle East**	Neonatal [0.98]	Congenital [1.11]	LRI [0.99]	Diarrhea [2.94]	Meningitis [0.82]	SIDS [0.89]	Whooping [5.42]	STI [1.28]	PEM [1.92]	Conflict Terror [1411.5]
Afghanistan	Neonatal [0.56]	LRI [0.57]	Congenital [1.27]	Diarrhea [0.26]	Meningitis [0.75]	Whooping [0.71]	SIDS [0.95]	Tetanus [1.06]	PEM [0.24]	Measles [0.26]
Algeria	Neonatal [0.92]	Congenital [1.31]	LRI [0.49]	Diarrhea [0.67]	SIDS [0.37]	F Body [0.44]	Road Inj [1.73]	Meningitis [0.25]	Oth Cardio [2.58]	CKD [1.9]
Bahrain	Neonatal [0.39]	Congenital [0.51]	LRI [0.11]	Endocrine [0.96]	SIDS [0.3]	Diarrhea [0.26]	F Body [0.15]	Road Inj [0.57]	Stroke [1.15]	Hemog [2.23]
Egypt	Neonatal [0.35]	LRI [0.96]	Congenital [0.7]	Diarrhea [2.58]	SIDS [0.76]	Road Inj [1.88]	Ileus [1.32]	Endocrine [0.94]	Encepha [1.12]	CKD [2.01]
Iran	Neonatal [1.01]	Congenital [1.1]	LRI [0.53]	Road Inj [3.0]	SIDS [0.55]	Diarrhea [0.57]	Endocrine [0.97]	F Body [0.43]	Ileus [1.46]	Oth Cardio [3.68]
Iraq	Neonatal [0.72]	Congenital [0.78]	LRI [0.28]	Conflict Terror [4007.63]	Diarrhea [0.33]	Meningitis [0.41]	CMP [3.01]	SIDS [0.69]	Whooping [1.35]	CKD [2.22]
Jordan	Neonatal [1.11]	Congenital [1.22]	LRI [0.89]	SIDS [0.61]	F Body [0.53]	Endocrine [1.17]	Diarrhea [0.55]	Road Inj [1.65]	Meningitis [0.29]	Ileus [0.92]
Kuwait	Neonatal [0.67]	Congenital [1.04]	LRI [0.63]	SIDS [0.34]	Diarrhea [0.91]	Endocrine [0.66]	Road Inj [1.05]	F Body [0.21]	Meningitis [0.26]	Ileus [0.81]
Lebanon	Neonatal [0.52]	Congenital [0.48]	LRI [0.25]	Diarrhea [1.21]	SIDS [0.34]	Whooping [5.48]	F Body [0.28]	Ileus [0.85]	Endocrine [0.39]	Conflict Terror [611.93]
Libya	Neonatal [0.68]	Congenital [0.55]	Oth Cardio [15.01]	LRI [0.38]	Conflict Terror [3754.35]	CMP [3.41]	Diarrhea [1.19]	Stroke [5.15]	SIDS [0.28]	Whooping [9.5]
Morocco	Neonatal [0.66]	Congenital [1.07]	LRI [0.28]	Diarrhea [0.68]	Meningitis [0.49]	SIDS [0.83]	STI [1.01]	Whooping [1.12]	Tetanus [62.43]	F Body [0.26]
Palestine	Neonatal [0.44]	Congenital [0.48]	LRI [0.05]	SIDS [0.48]	Endocrine [0.98]	Diarrhea [0.06]	Meningitis [0.11]	Whooping [0.34]	CKD [0.93]	Ileus [0.37]
Oman	Neonatal [0.87]	Congenital [0.8]	LRI [0.42]	Oth Cardio [11.54]	Road Inj [4.16]	Stroke [8.51]	SIDS [0.48]	CMP [2.38]	Encepha [3.04]	Diarrhea [0.56]
Qatar	Neonatal [0.52]	Congenital [0.75]	LRI [0.27]	SIDS [0.29]	Road Inj [1.46]	Endocrine [0.6]	Diarrhea [0.48]	Ileus [1.0]	Oth Neuro [0.83]	CKD [1.33]
Saudi Arabia	Neonatal [1.05]	Congenital [0.67]	SIDS [0.49]	STI [4.15]	Diarrhea [1.03]	Endocrine [0.65]	Conflict Terror [1311.93]	LRI [0.1]	Med Treat [1.57]	Whooping [4.3]
Sudan	Neonatal [0.93]	Congenital [1.65]	Diarrhea [0.87]	LRI [0.37]	STI [1.25]	SIDS [0.99]	Whooping [0.8]	Meningitis [0.23]	Road Inj [2.17]	HIV [3.05]
Syria	Neonatal [0.3]	Conflict Terror [15226.87]	Congenital [0.44]	Whooping [6.89]	LRI [0.06]	SIDS [0.67]	Measles [7.08]	Endocrine [0.54]	Meningitis [0.1]	Diarrhea [0.07]
Tunisia	Neonatal [0.58]	Congenital [0.58]	LRI [0.2]	SIDS [0.45]	Diarrhea [0.39]	Mech [4.9]	F Body [0.22]	Whooping [2.46]	Ileus [0.74]	Endocrine [0.39]
Turkey	Neonatal [1.11]	Congenital [1.1]	LRI [0.43]	Endocrine [1.96]	SIDS [0.49]	Diarrhea [0.7]	F Body [0.31]	Epilepsy [2.44]	Ileus [0.9]	Oth Neuro [1.07]
UAE	Neonatal [1.03]	Congenital [1.7]	SIDS [0.56]	Med Treat [7.65]	LRI [0.4]	Endocrine [1.11]	Road Inj [2.56]	F Body [0.59]	Diarrhea [1.27]	Stroke [3.78]
Yemen	Neonatal [0.72]	Congenital [0.87]	Diarrhea [0.9]	LRI [0.28]	Whooping [0.75]	Conflict Terror [956.05]	PEM [0.38]	SIDS [0.83]	Road Inj [1.84]	Meningitis [0.13]
**South Asia**	Neonatal [1.17]	LRI [0.95]	Congenital [0.55]	Diarrhea [1.3]	STI [1.59]	PEM [1.31]	Meningitis [0.49]	Malaria [682.13]	SIDS [0.87]	Whooping [1.21]

eFigure 7b. Leading ten causes of DALYs with the ratio of observed DALYs to DALYs expected on the basis of Socio-Demographic Index alone in 2017, <1 years, both sexes combined. The top ten causes contributing to DALYs are listed globally, by socio-demographic quintile, and then by GBD superregion, region, country, and subnationally where modeled. For each cell, the ratio of observed DALYs to DALYs expected on the basis of socio-demographic index (SDI) alone are listed. Abbreviations: DALY=disability-adjusted life year, GBD=Global Burden of Disease.

Values shown in brackets represent the ratio of observed DALYs to predicted DALYs on the basis of Socio-Demographic Index (SDI), rounded to two (2) digits. Color ranges (shown below) were calculated to place a roughly equal number of cells into each bin.

COLOR KEY:	[0.0-0.33]	(0.33-0.46]	(0.46-0.58]	(0.58-0.7]	(0.7-0.84]	(0.84-1.03]	(1.03-1.38]	(1.38-2.46]	2.46+

	1	2	3	4	5	6	7	8	9	10
Bangladesh	Neonatal [0.83]	LRI [0.58]	Congenital [0.41]	STI [1.17]	Diarrhea [0.16]	PEM [0.5]	SIDS [0.89]	Whooping [0.62]	Tetanus [5.77]	Ileus [0.61]
Bhutan	Neonatal [0.81]	LRI [0.56]	Congenital [0.47]	STI [1.61]	Diarrhea [0.24]	SIDS [0.71]	Meningitis [0.23]	Encepha [1.43]	PEM [0.66]	Iron [3.49]
India	Neonatal [1.09]	LRI [1.09]	Diarrhea [1.66]	Congenital [0.58]	PEM [1.83]	STI [1.65]	Malaria [1481.13]	Meningitis [0.44]	SIDS [0.82]	TB [1.87]
Andhra Pradesh	Neonatal [0.82]	LRI [0.5]	Congenital [0.47]	Diarrhea [0.96]	STI [1.47]	Hep [9.7]	PEM [0.74]	Meningitis [0.28]	SIDS [0.77]	Encepha [1.79]
Arunachal Pradesh	Neonatal [0.69]	LRI [0.61]	Diarrhea [0.85]	Congenital [0.26]	Malaria [2380.43]	STI [1.46]	Hep [9.2]	PEM [0.82]	Whooping [1.28]	SIDS [0.56]
Assam	Neonatal [1.3]	LRI [1.07]	Congenital [0.88]	Diarrhea [1.55]	PEM [1.45]	Malaria [1071.71]	STI [1.36]	Hep [14.41]	Whooping [1.31]	SIDS [0.85]
Bihar	Neonatal [0.82]	LRI [0.73]	Diarrhea [1.08]	Congenital [0.4]	STI [0.58]	Meningitis [0.31]	PEM [0.37]	Encepha [1.85]	Malaria [14.84]	Whooping [0.41]
Chhattisgarh	Neonatal [1.13]	LRI [0.9]	Diarrhea [0.72]	Congenital [0.36]	PEM [1.52]	STI [1.21]	Malaria [499.34]	SIDS [0.68]	Meningitis [0.19]	TB [1.09]
Delhi	Neonatal [2.6]	LRI [3.4]	Congenital [0.76]	Diarrhea [7.73]	STI [13.75]	PEM [18.43]	Meningitis [1.07]	HIV [7.77]	Iron [9.58]	SIDS [0.56]
Goa	Neonatal [1.82]	LRI [4.15]	Congenital [0.83]	STI [19.13]	Diarrhea [4.31]	SIDS [0.67]	Encepha [7.3]	PEM [16.67]	Falls [6.05]	Malaria [100686.78]
Gujarat	Neonatal [1.17]	LRI [0.93]	Congenital [0.61]	Diarrhea [1.08]	STI [2.49]	Malaria [5256.81]	PEM [1.87]	Meningitis [0.48]	SIDS [0.74]	Whooping [2.0]
Haryana	Neonatal [1.15]	LRI [1.42]	Congenital [0.58]	Diarrhea [2.24]	PEM [4.92]	STI [3.34]	Meningitis [0.68]	Malaria [6163.97]	SIDS [0.96]	TB [3.56]
Himachal Pradesh	Neonatal [1.33]	LRI [1.61]	Congenital [0.7]	Diarrhea [2.03]	STI [4.99]	Falls [6.06]	SIDS [0.74]	Meningitis [0.43]	Malaria [7156.63]	F Body [0.48]
Jammu & Kashmir	Neonatal [1.15]	LRI [1.73]	Congenital [0.83]	Diarrhea [1.17]	STI [2.17]	PEM [1.51]	SIDS [0.85]	Meningitis [0.37]	Hep [10.68]	Malaria [2777.33]
Jharkhand	Neonatal [0.69]	LRI [0.6]	Diarrhea [0.8]	Congenital [0.38]	STI [0.83]	PEM [0.55]	Malaria [126.19]	Meningitis [0.16]	SIDS [0.56]	TB [0.7]
Karnataka	Neonatal [0.94]	Congenital [0.68]	LRI [0.57]	Diarrhea [0.68]	STI [2.01]	PEM [1.94]	Meningitis [0.4]	SIDS [0.72]	Encepha [1.89]	Whooping [1.58]
Kerala	Neonatal [0.51]	Congenital [0.67]	LRI [0.38]	STI [5.54]	SIDS [0.32]	PEM [1.69]	Whooping [2.55]	Vit A [2.28]	Measles [18.3]	Encepha [1.12]
Madhya Pradesh	Neonatal [0.9]	LRI [0.9]	Congenital [0.48]	Diarrhea [0.65]	PEM [1.16]	Malaria [199.29]	STI [0.87]	Meningitis [0.32]	TB [1.21]	SIDS [0.77]
Maharashtra	Neonatal [1.1]	LRI [0.75]	Congenital [0.41]	Diarrhea [1.09]	STI [3.62]	PEM [2.97]	Meningitis [0.31]	SIDS [0.53]	Whooping [2.71]	Encepha [1.56]
Manipur	Neonatal [0.98]	LRI [0.8]	Congenital [0.47]	Diarrhea [1.37]	HIV [10.41]	STI [2.48]	Malaria [3293.35]	Whooping [2.45]	F Body [0.53]	PEM [1.0]
Meghalaya	Neonatal [1.02]	LRI [1.24]	Congenital [0.64]	Malaria [12335.38]	Diarrhea [1.4]	STI [2.09]	Meningitis [0.53]	TB [2.87]	SIDS [0.88]	PEM [0.92]
Mizoram	Neonatal [1.38]	LRI [2.46]	Congenital [0.71]	Malaria [23946.13]	Diarrhea [1.19]	STI [3.63]	HIV [12.18]	Meningitis [0.84]	PEM [2.66]	Whooping [5.07]
Nagaland	Neonatal [1.81]	LRI [3.93]	Congenital [0.49]	Diarrhea [2.52]	Malaria [45765.89]	HIV [21.87]	STI [6.47]	Hep [40.9]	Meningitis [0.87]	TB [9.69]
Odisha	Neonatal [1.13]	LRI [0.92]	Congenital [0.57]	Diarrhea [1.23]	Malaria [2475.82]	PEM [1.7]	STI [1.19]	Hep [12.28]	Meningitis [0.33]	F Body [0.66]
Punjab	Neonatal [1.22]	LRI [1.29]	Congenital [0.76]	Diarrhea [2.49]	STI [3.85]	PEM [3.33]	Meningitis [0.45]	SIDS [0.71]	Malaria [3990.56]	Falls [3.95]
Rajasthan	Neonatal [0.96]	LRI [1.39]	Congenital [0.45]	Diarrhea [0.68]	PEM [1.09]	Malaria [226.58]	STI [0.83]	TB [1.78]	Meningitis [0.3]	SIDS [0.84]
Sikkim	Neonatal [0.68]	LRI [1.36]	Congenital [0.35]	Diarrhea [1.58]	STI [4.6]	SIDS [0.66]	Measles [25.96]	Encepha [1.69]	Meningitis [0.27]	Hep [6.52]
Tamil Nadu	Neonatal [0.58]	Congenital [0.46]	LRI [0.49]	Diarrhea [0.81]	STI [3.08]	PEM [1.77]	Meningitis [0.31]	SIDS [0.5]	Falls [3.66]	Encepha [1.53]
Telangana	Neonatal [0.85]	LRI [0.48]	Congenital [0.39]	Diarrhea [1.08]	STI [2.3]	HIV [4.57]	PEM [1.11]	Meningitis [0.27]	SIDS [0.64]	Encepha [1.67]
Tripura	Neonatal [1.2]	LRI [1.68]	Congenital [0.86]	Diarrhea [1.15]	Malaria [3105.23]	STI [1.76]	F Body [1.18]	PEM [1.37]	Hep [12.66]	SIDS [1.12]
Uttar Pradesh	Neonatal [1.17]	LRI [1.11]	Diarrhea [1.78]	Congenital [0.78]	PEM [1.74]	Meningitis [0.55]	Tetanus [38.9]	STI [0.96]	Malaria [224.22]	TB [1.75]

eFigure 7b. Leading ten causes of DALYs with the ratio of observed DALYs to DALYs expected on the basis of Socio-Demographic Index alone in 2017, <1 years, both sexes combined. The top ten causes contributing to DALYs are listed globally, by socio-demographic quintile, and then by GBD superregion, region, country, and subnationally where modeled. For each cell, the ratio of observed DALYs to DALYs expected on the basis of socio-demographic index (SDI) alone are listed. Abbreviations: DALY=disability-adjusted life year, GBD=Global Burden of Disease.

Values shown in brackets represent the ratio of observed DALYs to predicted DALYs on the basis of Socio-Demographic Index (SDI), rounded to two (2) digits. Color ranges (shown below) were calculated to place a roughly equal number of cells into each bin.

COLOR KEY:	[0.0-0.33]	(0.33-0.46]	(0.46-0.58]	(0.58-0.7]	(0.7-0.84]	(0.84-1.03]	(1.03-1.38]	(1.38-2.46]	2.46+

	1	2	3	4	5	6	7	8	9	10
Uttarakhand	Neonatal [0.96]	LRI [1.31]	Congenital [0.48]	Diarrhea [1.48]	STI [3.59]	PEM [2.61]	Meningitis [0.5]	Tetanus [276.41]	SIDS [0.74]	TB [2.89]
W Bengal	Neonatal [0.84]	LRI [0.8]	Congenital [0.54]	Diarrhea [0.51]	STI [1.51]	PEM [0.98]	Measles [5.62]	SIDS [0.73]	Meningitis [0.24]	F Body [0.49]
UTs other than Delhi	Neonatal [1.77]	LRI [1.81]	Congenital [0.85]	Diarrhea [2.61]	STI [8.47]	Meningitis [0.98]	SIDS [0.93]	Falls [7.72]	Malaria [19411.65]	PEM [4.04]
Nepal	Neonatal [0.56]	LRI [0.5]	Congenital [0.23]	Diarrhea [0.16]	STI [0.8]	Whooping [0.57]	PEM [0.31]	Tetanus [5.3]	SIDS [0.6]	Meningitis [0.13]
Pakistan	Neonatal [1.6]	LRI [0.65]	Diarrhea [0.93]	Congenital [0.55]	Meningitis [0.82]	STI [1.29]	Whooping [1.02]	SIDS [1.0]	PEM [0.39]	TB [0.89]
**Southeast Asia, East Asia, and Oceania**	Neonatal [0.65]	Congenital [0.59]	LRI [1.04]	Diarrhea [1.84]	STI [5.01]	F Body [0.87]	Meningitis [0.59]	Ileus [1.82]	PEM [2.31]	SIDS [0.25]
**East Asia**	Neonatal [0.44]	Congenital [0.49]	LRI [0.87]	F Body [1.2]	Diarrhea [0.75]	STI [1.92]	Ileus [1.67]	Meningitis [0.26]	Leukemia [1.9]	Road Inj [0.9]
China	Neonatal [0.42]	Congenital [0.47]	LRI [0.79]	F Body [1.15]	Diarrhea [0.66]	STI [1.81]	Ileus [1.59]	Meningitis [0.24]	Leukemia [1.86]	Road Inj [0.87]
N Korea	Neonatal [0.5]	LRI [0.63]	Congenital [0.73]	Diarrhea [0.41]	F Body [1.41]	PEM [0.66]	Ileus [1.27]	Meningitis [0.19]	Whooping [0.92]	TB [1.11]
Taiwan	Neonatal [0.72]	Congenital [0.61]	F Body [2.34]	LRI [1.42]	SIDS [0.48]	Diarrhea [2.88]	Oth Un Inf [1.08]	STI [3.4]	Endocrine [0.26]	Falls [1.34]
**Oceania**	Neonatal [0.58]	Congenital [0.73]	LRI [0.37]	STI [3.74]	Diarrhea [0.45]	Meningitis [0.57]	PEM [1.01]	Whooping [0.99]	Stroke [2.88]	F Body [0.55]
Am Samoa	Neonatal [0.56]	Congenital [0.3]	LRI [0.66]	STI [5.05]	F Body [0.83]	Endocrine [0.92]	Med Treat [2.69]	PEM [3.95]	Diarrhea [0.46]	Meningitis [0.37]
Micronesia	Neonatal [0.5]	Congenital [0.4]	LRI [0.3]	STI [2.74]	Whooping [3.25]	Meningitis [0.21]	Diarrhea [0.1]	PEM [0.51]	SIDS [0.37]	F Body [0.25]
Fiji	Neonatal [0.91]	Congenital [0.82]	LRI [0.84]	Diarrhea [1.09]	F Body [0.75]	Meningitis [0.55]	STI [1.16]	Oth Un Inf [1.65]	Whooping [2.88]	PEM [1.48]
Guam	Neonatal [1.48]	Congenital [0.97]	LRI [3.79]	STI [10.27]	F Body [0.96]	Meningitis [1.11]	CMP [2.52]	SIDS [0.28]	Diarrhea [1.38]	PEM [23.55]
Kiribati	Neonatal [0.64]	Congenital [0.8]	LRI [0.17]	Diarrhea [0.19]	PEM [0.7]	Meningitis [0.42]	Whooping [0.88]	Measles [1.37]	Med Treat [1.5]	SIDS [0.43]
Marshall	Neonatal [0.56]	Congenital [0.55]	LRI [0.37]	Whooping [2.82]	STI [0.66]	Meningitis [0.24]	Measles [5.07]	PEM [0.52]	Diarrhea [0.1]	F Body [0.32]
N Mariana	Neonatal [0.5]	Congenital [0.26]	LRI [0.62]	STI [5.55]	Diarrhea [0.68]	F Body [0.32]	SIDS [0.14]	Measles [42.84]	PEM [3.67]	Med Treat [1.01]
PNG	Neonatal [0.53]	Congenital [0.75]	LRI [0.32]	STI [2.76]	Diarrhea [0.36]	Meningitis [0.52]	PEM [0.79]	Whooping [0.71]	Stroke [2.92]	F Body [0.62]
Samoa	Neonatal [0.34]	Congenital [0.26]	LRI [0.2]	STI [0.99]	Whooping [2.16]	Measles [8.68]	Diarrhea [0.08]	SIDS [0.3]	Meningitis [0.12]	F Body [0.16]
Solomon	Neonatal [0.29]	STI [4.96]	Congenital [0.36]	LRI [0.19]	Diarrhea [0.07]	Whooping [0.38]	PEM [0.16]	Meningitis [0.09]	Vit A [1.06]	SIDS [0.23]
Tonga	Neonatal [0.58]	Congenital [0.3]	LRI [0.37]	Meningitis [0.9]	PEM [1.18]	Whooping [2.23]	F Body [0.3]	Measles [13.15]	SIDS [0.35]	Dengue [79.4]
Vanuatu	Neonatal [0.48]	LRI [0.38]	Congenital [0.55]	STI [2.78]	Diarrhea [0.14]	Whooping [0.94]	Meningitis [0.18]	PEM [0.33]	Stroke [1.52]	F Body [0.33]
**Southeast Asia**	Neonatal [0.85]	Congenital [0.71]	LRI [1.01]	Diarrhea [2.17]	STI [6.03]	Meningitis [0.82]	Ileus [1.87]	F Body [0.47]	Dengue [146.03]	TB [3.99]
Cambodia	Neonatal [0.53]	LRI [0.75]	Congenital [0.71]	Diarrhea [0.17]	Ileus [1.95]	Oth Un Inf [1.88]	Meningitis [0.23]	HIV [2.62]	TB [0.87]	Malaria [61.6]
Indonesia	Neonatal [0.99]	Congenital [0.65]	LRI [0.91]	Diarrhea [4.25]	STI [14.23]	Meningitis [1.17]	Dengue [402.59]	Measles [63.02]	Ileus [2.67]	PEM [3.03]
Laos	Neonatal [1.15]	LRI [1.66]	Congenital [1.38]	Diarrhea [1.53]	Meningitis [1.06]	F Body [1.18]	TB [2.61]	PEM [0.8]	Whooping [1.47]	Road Inj [2.96]
Malaysia	Neonatal [0.5]	Congenital [0.61]	LRI [0.65]	HIV [8.58]	F Body [0.68]	Diarrhea [1.11]	Meningitis [0.57]	SIDS [0.21]	PEM [5.83]	Whooping [5.11]
Maldives	Neonatal [0.43]	Congenital [0.27]	LRI [0.08]	Endocrine [0.89]	SIDS [0.3]	Diarrhea [0.17]	STI [0.63]	Dengue [73.88]	PEM [0.77]	F Body [0.12]
Mauritius	Neonatal [1.15]	Congenital [0.76]	LRI [0.64]	SIDS [0.74]	Diarrhea [1.41]	F Body [0.89]	Meningitis [0.47]	Med Treat [2.87]	Iron [3.34]	PEM [3.43]
Myanmar	Neonatal [0.97]	Congenital [1.34]	LRI [1.13]	Diarrhea [0.97]	Meningitis [0.91]	Oth Un Inf [6.11]	STI [1.58]	Ileus [2.66]	F Body [0.79]	Whooping [2.17]

eFigure 7b. Leading ten causes of DALYs with the ratio of observed DALYs to DALYs expected on the basis of Socio-Demographic Index alone in 2017, <1 years, both sexes combined. The top ten causes contributing to DALYs are listed globally, by socio-demographic quintile, and then by GBD superregion, region, country, and subnationally where modeled. For each cell, the ratio of observed DALYs to DALYs expected on the basis of socio-demographic index (SDI) alone are listed. Abbreviations: DALY=disability-adjusted life year, GBD=Global Burden of Disease.

Values shown in brackets represent the ratio of observed DALYs to predicted DALYs on the basis of Socio-Demographic Index (SDI), rounded to two (2) digits. Color ranges (shown below) were calculated to place a roughly equal number of cells into each bin.

COLOR KEY:	[0.0-0.33]	(0.33-0.46]	(0.46-0.58]	(0.58-0.7]	(0.7-0.84]	(0.84-1.03]	(1.03-1.38]	(1.38-2.46]	2.46+

	1	2	3	4	5	6	7	8	9	10
Philippines	Neonatal [0.84]	LRI [0.95]	Congenital [0.6]	Diarrhea [1.06]	STI [2.94]	Meningitis [0.59]	F Body [0.46]	SIDS [0.59]	PEM [1.28]	TB [2.83]
Sri Lanka	Neonatal [0.51]	Congenital [0.39]	LRI [0.17]	F Body [0.3]	SIDS [0.23]	PEM [1.33]	Meningitis [0.16]	Diarrhea [0.15]	Whooping [1.82]	CMP [0.64]
Seychelles	Neonatal [0.64]	Congenital [0.64]	LRI [1.08]	Meningitis [1.02]	SIDS [0.42]	Diarrhea [0.48]	Whooping [5.39]	F Body [0.28]	Ileus [0.99]	Endocrine [0.49]
Thailand	Neonatal [0.45]	Congenital [0.39]	LRI [0.34]	Diarrhea [0.57]	F Body [0.32]	SIDS [0.28]	Whooping [3.84]	Iron [2.53]	Oth Un Inf [0.86]	Meningitis [0.18]
Timor-Leste	Neonatal [0.62]	LRI [0.62]	Congenital [0.79]	Diarrhea [0.42]	Meningitis [0.37]	STI [0.73]	PEM [0.48]	F Body [0.53]	HIV [2.56]	Whooping [0.66]
Vietnam	Neonatal [0.31]	Congenital [0.61]	LRI [0.33]	Whooping [2.33]	F Body [0.38]	Encepha [1.29]	Meningitis [0.16]	SIDS [0.31]	Diarrhea [0.1]	Drown [1.65]
**Sub-Saharan Africa**	Neonatal [0.85]	LRI [0.76]	Diarrhea [1.05]	Congenital [0.64]	Malaria [239.46]	STI [1.89]	Meningitis [0.88]	HIV [10.69]	PEM [1.14]	Whooping [1.16]
**Central Sub-Saharan Africa**	Neonatal [0.84]	LRI [0.78]	Congenital [0.96]	Malaria [559.64]	Diarrhea [1.06]	STI [2.91]	PEM [1.54]	Meningitis [0.69]	Whooping [1.83]	TB [1.88]
Angola	Neonatal [0.78]	Diarrhea [1.28]	LRI [0.55]	Congenital [0.7]	STI [2.88]	Malaria [226.92]	PEM [1.54]	Meningitis [0.65]	HIV [8.42]	Measles [2.28]
C African Rep	Neonatal [1.14]	Diarrhea [1.84]	LRI [1.08]	Congenital [1.35]	Malaria [17.14]	STI [2.34]	PEM [1.63]	Meningitis [0.99]	Whooping [2.04]	TB [2.28]
Congo	Neonatal [0.93]	Congenital [0.64]	HIV [49.91]	Diarrhea [2.26]	LRI [0.57]	STI [9.18]	Malaria [13959.58]	Meningitis [0.64]	PEM [1.87]	Whooping [2.82]
Congo DR	Neonatal [0.64]	LRI [0.59]	Malaria [32.78]	Congenital [0.96]	Diarrhea [0.49]	STI [1.53]	PEM [0.79]	Whooping [1.16]	Meningitis [0.47]	TB [1.12]
Eq Guinea	Neonatal [1.09]	Malaria [145294.81]	Congenital [0.8]	STI [16.58]	LRI [0.79]	HIV [50.51]	Diarrhea [2.39]	Meningitis [1.01]	PEM [3.37]	Measles [34.96]
Gabon	Neonatal [1.6]	Congenital [1.02]	Malaria [189880.94]	LRI [1.19]	Diarrhea [3.53]	STI [13.95]	Meningitis [1.31]	HIV [12.61]	PEM [5.36]	SIDS [0.8]
**Eastern Sub-Saharan Africa**	Neonatal [0.64]	LRI [0.44]	Diarrhea [0.57]	Congenital [0.64]	STI [1.38]	Malaria [15.11]	HIV [12.31]	PEM [0.73]	Meningitis [0.45]	Whooping [0.79]
Burundi	Neonatal [0.53]	LRI [0.39]	Congenital [0.95]	Malaria [7.95]	Diarrhea [0.34]	PEM [0.82]	Meningitis [0.35]	Measles [0.34]	TB [0.69]	Oth Un Inf [1.23]
Comoros	Neonatal [0.67]	LRI [0.42]	Congenital [0.71]	Diarrhea [0.48]	STI [1.2]	PEM [0.6]	Meningitis [0.36]	Whooping [0.82]	Oth Un Inf [1.52]	SIDS [0.72]
Djibouti	Neonatal [0.5]	LRI [0.4]	Congenital [0.51]	Diarrhea [0.51]	STI [2.29]	PEM [0.92]	Whooping [1.52]	Meningitis [0.37]	HIV [4.39]	Oth Un Inf [1.95]
Eritrea	Neonatal [0.67]	LRI [0.55]	Diarrhea [0.73]	Congenital [0.66]	STI [1.11]	PEM [0.91]	Meningitis [0.49]	HIV [5.44]	Oth Un Inf [2.48]	TB [1.09]
Ethiopia	Neonatal [0.58]	Diarrhea [0.53]	LRI [0.31]	Congenital [0.38]	STI [0.79]	PEM [0.52]	Whooping [0.79]	Meningitis [0.35]	Oth Un Inf [1.71]	SIDS [0.68]
Kenya	Neonatal [0.65]	LRI [0.69]	Diarrhea [1.5]	Congenital [0.6]	HIV [19.23]	Meningitis [0.63]	PEM [1.09]	STI [1.09]	Tetanus [44.09]	Malaria [302.41]
Baringo	Neonatal [0.52]	LRI [0.5]	Diarrhea [1.01]	Congenital [0.5]	Meningitis [0.38]	PEM [0.62]	STI [0.62]	Oth Un Inf [1.48]	HIV [2.46]	Whooping [0.42]
Bomet	Neonatal [0.46]	HIV [42.44]	Diarrhea [0.71]	LRI [0.25]	Congenital [0.27]	STI [0.72]	Meningitis [0.2]	PEM [0.23]	Oth Un Inf [0.9]	iNTS [8.97]
Bungoma	Neonatal [0.67]	Diarrhea [1.51]	LRI [0.61]	Congenital [0.62]	Malaria [155.04]	Meningitis [0.56]	PEM [0.86]	STI [0.88]	Whooping [1.32]	Tetanus [17.92]
Busia	Neonatal [0.71]	LRI [0.97]	Diarrhea [1.33]	Congenital [0.93]	Malaria [299.38]	Meningitis [0.79]	PEM [1.05]	Whooping [0.95]	Tetanus [12.88]	STI [0.55]
Elgeyo-Marakwet	Neonatal [0.95]	Diarrhea [1.97]	LRI [0.63]	Congenital [0.65]	STI [1.65]	Meningitis [0.52]	HIV [7.66]	PEM [1.0]	Whooping [1.09]	Oth Un Inf [2.24]
Embu	Neonatal [0.8]	LRI [1.02]	Diarrhea [2.86]	Congenital [0.78]	PEM [2.51]	Meningitis [0.97]	HIV [13.67]	STI [1.7]	SIDS [1.08]	Whooping [1.59]
Garissa	Neonatal [0.23]	Diarrhea [0.39]	LRI [0.2]	Congenital [0.24]	HIV [9.84]	Tetanus [2.47]	STI [0.24]	Meningitis [0.15]	SIDS [0.58]	PEM [0.15]
HomaBay	Neonatal [0.65]	LRI [1.06]	HIV [59.54]	Diarrhea [1.23]	Congenital [1.03]	Meningitis [0.89]	Malaria [55.12]	PEM [1.16]	TB [1.83]	STI [0.62]
Isiolo	Neonatal [0.65]	LRI [0.98]	Congenital [1.01]	Diarrhea [0.54]	Meningitis [0.76]	PEM [1.0]	Tetanus [9.31]	HIV [8.49]	Whooping [0.91]	TB [1.31]
Kajiado	Neonatal [0.52]	LRI [0.39]	Diarrhea [0.92]	Congenital [0.34]	STI [1.11]	Meningitis [0.4]	Whooping [1.87]	Tetanus [49.45]	HIV [3.15]	PEM [0.54]

eFigure 7b. Leading ten causes of DALYs with the ratio of observed DALYs to DALYs expected on the basis of Socio-Demographic Index alone in 2017, <1 years, both sexes combined. The top ten causes contributing to DALYs are listed globally, by socio-demographic quintile, and then by GBD superregion, region, country, and subnationally where modeled. For each cell, the ratio of observed DALYs to DALYs expected on the basis of socio-demographic index (SDI) alone are listed. Abbreviations: DALY=disability-adjusted life year, GBD=Global Burden of Disease.

Values shown in brackets represent the ratio of observed DALYs to predicted DALYs on the basis of Socio-Demographic Index (SDI), rounded to two (2) digits. Color ranges (shown below) were calculated to place a roughly equal number of cells into each bin.

COLOR KEY:	[0.0-0.33]	(0.33-0.46]	(0.46-0.58]	(0.58-0.7]	(0.7-0.84]	(0.84-1.03]	(1.03-1.38]	(1.38-2.46]	2.46+

	1	2	3	4	5	6	7	8	9	10
Kakamega	Neonatal [0.62]	LRI [0.75]	Diarrhea [1.28]	Congenital [0.71]	Meningitis [0.66]	Malaria [94.42]	PEM [0.99]	HIV [8.34]	STI [0.67]	Whooping [0.84]
Kericho	Neonatal [0.83]	Diarrhea [1.27]	LRI [0.51]	Congenital [0.56]	HIV [18.7]	STI [1.27]	Meningitis [0.43]	Whooping [1.04]	PEM [0.58]	iNTS [21.0]
Kiambu	Neonatal [1.36]	LRI [1.32]	Congenital [0.95]	Diarrhea [3.04]	Meningitis [1.33]	STI [3.89]	PEM [2.84]	HIV [9.65]	Whooping [4.89]	SIDS [1.28]
Kilifi	Neonatal [0.62]	Diarrhea [1.57]	LRI [0.58]	Congenital [0.58]	HIV [12.88]	PEM [1.03]	Meningitis [0.49]	Tetanus [16.98]	STI [0.68]	Malaria [57.29]
Kirinyaga	Neonatal [0.98]	LRI [0.96]	Diarrhea [2.71]	Congenital [0.9]	Meningitis [0.9]	STI [1.72]	PEM [1.35]	HIV [7.2]	SIDS [0.84]	Malaria [574.43]
Kisii	Neonatal [1.05]	Diarrhea [3.01]	LRI [0.99]	Congenital [0.95]	Meningitis [0.82]	STI [1.8]	PEM [1.13]	HIV [6.51]	iNTS [23.95]	Oth Un Inf [1.86]
Kisumu	Neonatal [0.88]	LRI [1.01]	Congenital [1.11]	HIV [56.76]	Diarrhea [1.34]	Meningitis [1.34]	Malaria [1157.03]	STI [2.08]	Whooping [2.23]	PEM [1.1]
Kitui	Neonatal [0.5]	Diarrhea [0.88]	LRI [0.29]	Congenital [0.34]	HIV [13.96]	STI [0.61]	Whooping [0.84]	Meningitis [0.26]	Tetanus [12.86]	PEM [0.36]
Kwale	Neonatal [0.59]	LRI [0.42]	Diarrhea [0.73]	Congenital [0.46]	HIV [11.31]	Malaria [92.69]	Meningitis [0.34]	PEM [0.55]	Tetanus [14.45]	STI [0.51]
Laikipia	Neonatal [0.61]	HIV [37.88]	LRI [0.35]	Diarrhea [1.1]	Congenital [0.33]	STI [1.56]	Meningitis [0.33]	Oth Un Inf [1.57]	Tetanus [45.63]	Whooping [0.9]
Lamu	Neonatal [1.2]	LRI [1.15]	Congenital [1.34]	Diarrhea [1.34]	Meningitis [1.13]	PEM [1.83]	Tetanus [35.77]	Whooping [1.96]	HIV [7.86]	STI [0.96]
Machakos	Neonatal [1.28]	Diarrhea [2.85]	LRI [0.88]	Congenital [0.87]	HIV [51.62]	STI [2.07]	Meningitis [0.82]	PEM [1.23]	Whooping [1.58]	Oth Un Inf [2.61]
Makueni	Neonatal [0.19]	HIV [28.35]	Diarrhea [0.38]	LRI [0.12]	Congenital [0.13]	STI [0.28]	Meningitis [0.1]	Whooping [0.31]	PEM [0.14]	iNTS [5.33]
Mandera	Neonatal [0.15]	Diarrhea [0.27]	LRI [0.16]	Tetanus [1.79]	Congenital [0.13]	HIV [4.4]	PEM [0.17]	Meningitis [0.12]	STI [0.15]	Whooping [0.18]
Marsabit	Neonatal [0.52]	LRI [0.44]	Diarrhea [0.66]	HIV [24.65]	Congenital [0.49]	Tetanus [6.34]	PEM [0.53]	Meningitis [0.32]	STI [0.39]	Whooping [0.35]
Meru	Neonatal [0.97]	LRI [1.13]	Diarrhea [2.6]	Congenital [0.93]	HIV [35.76]	Meningitis [1.05]	PEM [1.99]	STI [1.48]	Whooping [1.53]	Malaria [391.0]
Migori	Neonatal [0.76]	LRI [1.05]	Diarrhea [1.39]	Congenital [1.09]	HIV [52.0]	Meningitis [0.82]	Malaria [42.08]	PEM [0.95]	STI [0.66]	Tetanus [9.23]
Mombasa	Neonatal [1.04]	LRI [1.32]	Diarrhea [3.45]	Congenital [0.93]	Meningitis [1.43]	PEM [3.36]	STI [2.99]	HIV [10.25]	Whooping [4.07]	SIDS [1.41]
Murang'a	Neonatal [0.8]	LRI [0.97]	Congenital [1.03]	HIV [24.49]	Meningitis [1.27]	Diarrhea [0.49]	Whooping [3.92]	STI [1.89]	PEM [1.29]	SIDS [1.3]
Nairobi	Neonatal [1.13]	LRI [1.54]	Congenital [0.63]	Diarrhea [3.61]	STI [12.16]	Meningitis [1.48]	HIV [9.71]	PEM [7.13]	SIDS [0.77]	Oth Un Inf [3.05]
Nakuru	Neonatal [0.77]	LRI [1.07]	Congenital [0.86]	Diarrhea [2.11]	Meningitis [1.04]	PEM [2.7]	STI [1.48]	HIV [6.21]	TB [2.16]	SIDS [0.88]
Nandi	Neonatal [0.6]	Diarrhea [1.18]	LRI [0.41]	Congenital [0.42]	HIV [18.54]	STI [1.05]	Meningitis [0.35]	PEM [0.52]	iNTS [18.2]	Malaria [140.86]
Narok	Neonatal [0.17]	LRI [0.16]	Diarrhea [0.26]	Congenital [0.17]	Meningitis [0.13]	STI [0.22]	PEM [0.19]	Tetanus [1.98]	Whooping [0.22]	HIV [1.21]
Nyamira	Neonatal [0.86]	LRI [0.59]	Congenital [0.6]	Diarrhea [1.43]	HIV [19.45]	STI [1.95]	Meningitis [0.53]	iNTS [21.89]	Oth Un Inf [1.5]	Tetanus [42.65]
Nyandarua	Neonatal [1.04]	LRI [1.35]	Congenital [1.06]	Diarrhea [2.49]	Meningitis [1.34]	PEM [2.43]	Whooping [3.38]	HIV [9.51]	STI [1.59]	TB [2.9]
Nyeri	Neonatal [0.93]	LRI [1.56]	Congenital [1.08]	Diarrhea [2.28]	Meningitis [1.85]	PEM [3.92]	HIV [17.13]	Whooping [5.67]	STI [2.45]	TB [4.04]
Samburu	Neonatal [0.33]	HIV [35.32]	Diarrhea [0.37]	LRI [0.24]	Congenital [0.25]	Tetanus [3.02]	PEM [0.26]	STI [0.27]	Meningitis [0.16]	Whooping [0.35]
Siaya	Neonatal [0.64]	LRI [0.84]	Diarrhea [1.21]	Congenital [0.9]	Malaria [387.98]	HIV [18.22]	Meningitis [0.66]	PEM [0.64]	STI [0.63]	TB [1.05]
TaitaTaveta	Neonatal [0.68]	LRI [0.8]	Diarrhea [1.76]	Congenital [0.65]	HIV [31.94]	Meningitis [0.89]	STI [1.86]	PEM [1.76]	Whooping [2.31]	SIDS [1.28]
TanaRiver	Neonatal [0.82]	LRI [0.55]	Diarrhea [0.58]	HIV [30.6]	Congenital [0.66]	Tetanus [13.67]	PEM [0.62]	Meningitis [0.41]	STI [0.49]	Whooping [0.71]
TharakaNithi	Neonatal [1.22]	Diarrhea [6.36]	LRI [1.72]	Congenital [1.25]	PEM [5.78]	Meningitis [1.61]	STI [2.46]	TB [3.73]	SIDS [1.76]	Whooping [2.28]

eFigure 7b. Leading ten causes of DALYs with the ratio of observed DALYs to DALYs expected on the basis of Socio-Demographic Index alone in 2017, <1 years, both sexes combined. The top ten causes contributing to DALYs are listed globally, by socio-demographic quintile, and then by GBD superregion, region, country, and subnationally where modeled. For each cell, the ratio of observed DALYs to DALYs expected on the basis of socio-demographic index (SDI) alone are listed. Abbreviations: DALY=disability-adjusted life year, GBD=Global Burden of Disease.

Values shown in brackets represent the ratio of observed DALYs to predicted DALYs on the basis of Socio-Demographic Index (SDI), rounded to two (2) digits. Color ranges (shown below) were calculated to place a roughly equal number of cells into each bin.

COLOR KEY:	[0.0-0.33]	(0.33-0.46]	(0.46-0.58]	(0.58-0.7]	(0.7-0.84]	(0.84-1.03]	(1.03-1.38]	(1.38-2.46]	2.46+

	1	2	3	4	5	6	7	8	9	10
TransNzoia	Neonatal [0.44]	Diarrhea [1.18]	LRI [0.38]	HIV [28.1]	Congenital [0.32]	STI [1.01]	Meningitis [0.35]	Whooping [1.01]	Tetanus [24.74]	PEM [0.49]
Turkana	Neonatal [0.32]	Diarrhea [0.44]	LRI [0.28]	HIV [15.78]	Congenital [0.35]	Tetanus [2.97]	PEM [0.35]	Meningitis [0.21]	Malaria [0.47]	STI [0.18]
UasinGishu	Neonatal [0.66]	Diarrhea [2.45]	LRI [0.66]	Congenital [0.53]	Meningitis [0.63]	PEM [1.56]	STI [1.52]	HIV [6.97]	Whooping [1.4]	Oth Un Inf [1.78]
Vihiga	Neonatal [0.33]	LRI [0.34]	Diarrhea [0.64]	Congenital [0.38]	Malaria [141.88]	Meningitis [0.31]	PEM [0.35]	STI [0.31]	Whooping [0.37]	Tetanus [7.08]
Wajir	Neonatal [0.29]	Diarrhea [0.53]	LRI [0.29]	Congenital [0.36]	Tetanus [1.86]	PEM [0.26]	Meningitis [0.22]	Whooping [0.44]	Measles [0.18]	SIDS [0.7]
WestPokot	Neonatal [0.42]	Diarrhea [0.92]	LRI [0.35]	Congenital [0.34]	Tetanus [7.55]	HIV [6.03]	PEM [0.47]	STI [0.48]	Meningitis [0.25]	Whooping [0.53]
Madagascar	Neonatal [0.56]	LRI [0.56]	Diarrhea [0.85]	Congenital [0.59]	PEM [1.31]	STI [1.4]	Malaria [2.5]	Meningitis [0.34]	Oth Un Inf [1.68]	Whooping [0.47]
Malawi	Neonatal [0.6]	LRI [0.31]	Congenital [0.68]	HIV [27.08]	STI [1.35]	Diarrhea [0.25]	Malaria [6.83]	Meningitis [0.36]	PEM [0.34]	Whooping [0.55]
Mozambique	Neonatal [0.62]	HIV [62.22]	Congenital [0.81]	Malaria [11.55]	LRI [0.28]	Diarrhea [0.27]	STI [1.32]	Meningitis [0.31]	SIDS [0.98]	TB [0.59]
Rwanda	Neonatal [0.54]	LRI [0.38]	Congenital [0.71]	Diarrhea [0.41]	Malaria [20.25]	PEM [0.73]	Meningitis [0.43]	STI [0.35]	SIDS [0.8]	HIV [2.78]
Somalia	Neonatal [0.44]	LRI [0.38]	Diarrhea [0.35]	Whooping [1.85]	STI [1.07]	Congenital [0.37]	Measles [0.5]	PEM [0.45]	Meningitis [0.4]	Tetanus [0.75]
S Sudan	Neonatal [0.7]	LRI [0.81]	Diarrhea [0.83]	STI [1.6]	Congenital [0.5]	Malaria [2.16]	Meningitis [0.77]	PEM [0.82]	TB [0.9]	Measles [0.33]
Tanzania	Neonatal [0.68]	Congenital [1.07]	LRI [0.5]	STI [1.71]	Malaria [39.88]	Diarrhea [0.23]	PEM [0.7]	Meningitis [0.4]	SIDS [1.16]	Whooping [0.63]
Uganda	Neonatal [0.64]	Malaria [44.07]	Congenital [0.64]	LRI [0.24]	STI [2.2]	Diarrhea [0.29]	HIV [12.48]	Meningitis [0.52]	PEM [0.47]	Whooping [0.6]
Zambia	Neonatal [0.73]	LRI [0.55]	HIV [46.93]	Congenital [0.75]	Diarrhea [0.8]	Malaria [296.89]	STI [1.79]	PEM [1.16]	Meningitis [0.61]	TB [1.12]
**Southern Sub-Saharan Africa**	Neonatal [1.21]	LRI [1.77]	Diarrhea [5.47]	HIV [48.92]	Congenital [0.35]	STI [7.59]	PEM [9.34]	TB [11.71]	Meningitis [0.75]	Whooping [5.96]
Botswana	Neonatal [1.01]	Diarrhea [5.49]	LRI [0.74]	Congenital [0.19]	HIV [15.94]	STI [2.75]	PEM [3.84]	Oth Un Inf [2.06]	SIDS [0.33]	TB [3.9]
Lesotho	Neonatal [0.93]	LRI [0.81]	Diarrhea [1.84]	HIV [61.23]	Congenital [0.37]	STI [1.78]	PEM [1.4]	TB [2.62]	Meningitis [0.28]	Oth Un Inf [1.27]
Namibia	Neonatal [1.2]	HIV [90.73]	LRI [0.98]	Diarrhea [3.97]	Congenital [0.29]	PEM [3.55]	STI [1.94]	TB [5.24]	Oth Un Inf [2.51]	Meningitis [0.38]
S Africa	Neonatal [1.17]	LRI [1.62]	Diarrhea [8.49]	HIV [64.42]	Congenital [0.36]	STI [14.31]	PEM [11.12]	TB [12.8]	SIDS [0.78]	Oth Un Inf [3.41]
Swaziland	Neonatal [0.86]	LRI [1.29]	Diarrhea [4.45]	HIV [58.05]	Congenital [0.33]	PEM [4.39]	STI [3.04]	TB [6.58]	Meningitis [0.54]	SIDS [0.75]
Zimbabwe	Neonatal [0.83]	LRI [1.0]	Diarrhea [0.66]	Congenital [0.33]	PEM [1.84]	HIV [11.79]	TB [2.74]	Meningitis [0.46]	STI [0.75]	Whooping [0.8]
**Western Sub-Saharan Africa**	Neonatal [0.95]	LRI [0.94]	Diarrhea [1.22]	Malaria [271.09]	Congenital [0.57]	Meningitis [1.22]	STI [1.52]	PEM [1.07]	HIV [7.27]	Whooping [1.01]
Benin	Neonatal [0.81]	LRI [0.46]	Malaria [33.56]	Diarrhea [0.57]	Congenital [0.64]	Meningitis [0.58]	PEM [0.58]	Whooping [0.73]	STI [0.49]	SIDS [0.88]
Burkina Faso	Neonatal [0.49]	LRI [0.48]	Malaria [7.51]	Congenital [0.83]	Diarrhea [0.38]	Meningitis [0.72]	PEM [0.54]	iNTS [9.03]	SIDS [0.94]	TB [0.51]
Cameroon	Neonatal [0.75]	Diarrhea [1.36]	LRI [0.57]	Malaria [1324.5]	Congenital [0.52]	STI [2.58]	Meningitis [0.66]	HIV [7.84]	PEM [0.94]	Whooping [0.86]
Cape Verde	Neonatal [0.77]	Congenital [0.44]	LRI [0.25]	Diarrhea [0.25]	SIDS [0.98]	STI [0.9]	Meningitis [0.23]	F Body [0.45]	HIV [2.97]	PEM [0.39]
Chad	Neonatal [0.61]	Diarrhea [0.81]	LRI [0.59]	Congenital [0.45]	Malaria [1.28]	Meningitis [0.64]	PEM [0.54]	Whooping [0.57]	STI [0.32]	TB [0.61]
Cote d'Ivoire	Neonatal [0.91]	LRI [0.6]	Diarrhea [0.77]	Congenital [0.78]	Malaria [81.23]	HIV [10.39]	Meningitis [0.6]	STI [0.99]	PEM [0.74]	SIDS [1.09]
Gambia	Neonatal [0.68]	LRI [0.31]	Congenital [0.41]	Diarrhea [0.3]	STI [1.58]	Meningitis [0.4]	PEM [0.42]	Whooping [0.65]	HIV [3.84]	Hemog [5.62]
Ghana	Neonatal [1.16]	Congenital [0.59]	LRI [0.46]	Malaria [5267.9]	Diarrhea [1.15]	STI [3.04]	Meningitis [0.76]	PEM [1.48]	HIV [7.3]	SIDS [0.82]

eFigure 7b. Leading ten causes of DALYs with the ratio of observed DALYs to DALYs expected on the basis of Socio-Demographic Index alone in 2017, <1 years, both sexes combined. The top ten causes contributing to DALYs are listed globally, by socio-demographic quintile, and then by GBD superregion, region, country, and subnationally where modeled. For each cell, the ratio of observed DALYs to DALYs expected on the basis of socio-demographic index (SDI) alone are listed. Abbreviations: DALY=disability-adjusted life year, GBD=Global Burden of Disease.

Values shown in brackets represent the ratio of observed DALYs to predicted DALYs on the basis of Socio-Demographic Index (SDI), rounded to two (2) digits. Color ranges (shown below) were calculated to place a roughly equal number of cells into each bin.

COLOR KEY:	[0.0-0.33]	(0.33-0.46]	(0.46-0.58]	(0.58-0.7]	(0.7-0.84]	(0.84-1.03]	(1.03-1.38]	(1.38-2.46]	2.46+

	1	2	3	4	5	6	7	8	9	10
Guinea	Neonatal [0.7]	LRI [0.6]	Malaria [10.64]	Congenital [0.67]	Diarrhea [0.32]	Meningitis [0.65]	PEM [0.67]	STI [0.75]	Whooping [0.98]	SIDS [0.93]
Guinea-Bissau	Neonatal [0.79]	Diarrhea [0.59]	LRI [0.28]	Congenital [0.6]	STI [1.89]	Measles [1.09]	Meningitis [0.45]	Whooping [0.72]	PEM [0.4]	HIV [5.09]
Liberia	Neonatal [0.49]	STI [2.47]	LRI [0.29]	Diarrhea [0.42]	Malaria [7.82]	Congenital [0.5]	Meningitis [0.39]	Measles [0.73]	Whooping [0.86]	PEM [0.34]
Mali	Neonatal [0.79]	Malaria [4.73]	LRI [0.3]	Diarrhea [0.36]	Congenital [0.77]	STI [1.21]	Meningitis [0.75]	PEM [0.52]	Whooping [0.66]	iNTS [7.85]
Mauritania	Neonatal [0.79]	LRI [0.47]	Diarrhea [0.78]	Congenital [0.43]	STI [2.02]	Meningitis [0.47]	PEM [0.82]	Whooping [0.84]	SIDS [0.87]	Malaria [65.28]
Niger	Neonatal [0.35]	LRI [0.3]	Diarrhea [0.33]	Malaria [0.97]	Congenital [0.38]	Meningitis [0.63]	Whooping [0.44]	PEM [0.14]	Measles [0.08]	SIDS [0.47]
Nigeria	Neonatal [1.21]	LRI [1.61]	Diarrhea [2.21]	Malaria [1877.36]	Congenital [0.51]	Meningitis [1.98]	STI [2.55]	HIV [12.98]	PEM [1.8]	Measles [5.89]
Sao Tome Principe	Neonatal [0.56]	Congenital [0.55]	LRI [0.38]	Diarrhea [0.34]	PEM [1.16]	SIDS [1.13]	Other MN [7.5]	STI [0.57]	Whooping [0.84]	Ileus [1.53]
Senegal	Neonatal [0.56]	LRI [0.32]	Diarrhea [0.43]	Congenital [0.44]	Meningitis [0.37]	STI [0.54]	PEM [0.31]	Malaria [3.28]	SIDS [0.8]	Whooping [0.38]
Sierra Leone	Neonatal [0.89]	Malaria [45.68]	LRI [0.73]	Congenital [1.06]	Diarrhea [0.64]	PEM [1.23]	Meningitis [0.89]	STI [1.25]	TB [1.03]	SIDS [1.25]
Togo	Neonatal [0.72]	Malaria [117.28]	Diarrhea [0.68]	LRI [0.35]	Congenital [0.49]	STI [0.89]	Meningitis [0.33]	PEM [0.39]	Whooping [0.61]	iNTS [13.37]

eFigure 7c. Leading ten causes of DALYs with the ratio of observed DALYs to DALYs expected on the basis of Socio-Demographic Index alone in 2017, 1-4 years, both sexes combined. The top ten causes contributing to DALYs are listed globally, by socio-demographic quintile, and then by GBD superregion, region, country, and subnationally where modeled. For each cell, the ratio of observed DALYs to DALYs expected on the basis of socio-demographic index (SDI) alone are listed. Abbreviations: DALY=disability-adjusted life year, GBD=Global Burden of Disease.

Values shown in brackets represent the ratio of observed DALYs to predicted DALYs on the basis of Socio-Demographic Index (SDI), rounded to two (2) digits. Color ranges (shown below) were calculated to place a roughly equal number of cells into each bin.

COLOR KEY:									
[0.0-0.5]	(0.5-0.72]	(0.72-0.86]	(0.86-0.97]	(0.97-1.1]	(1.1-1.23]	(1.23-1.41]	(1.41-2.15]	2.15+	

	1	2	3	4	5	6	7	8	9	10
Global	Malaria [17944.77]	Diarrhea [5.99]	LRI [1.96]	Congenital [0.83]	PEM [16.24]	Meningitis [1.62]	Measles [37.97]	Drown [1.07]	Neonatal [1.06]	Whooping [13.27]
Low SDI	Malaria [32.4]	Diarrhea [0.61]	LRI [0.46]	PEM [0.89]	Congenital [0.61]	Measles [0.52]	Meningitis [0.45]	Whooping [0.79]	Drown [0.54]	Iron [1.16]
Low-middle SDI	Malaria [1479.55]	Diarrhea [1.91]	LRI [0.93]	Meningitis [1.02]	PEM [2.43]	Congenital [0.62]	Measles [3.93]	Drown [0.62]	Neonatal [0.98]	Iron [1.65]
Middle SDI	LRI [0.65]	Congenital [0.5]	Neonatal [1.04]	Diarrhea [1.37]	Drown [0.78]	Road Inj [0.54]	Asthma [0.97]	PEM [4.3]	Measles [13.73]	Dermatitis [0.69]
High-middle SDI	Congenital [1.4]	Neonatal [1.5]	Road Inj [1.91]	LRI [3.59]	Drown [2.32]	Diarrhea [2.43]	Dermatitis [0.57]	Asthma [1.06]	Leukemia [1.62]	Falls [1.32]
High SDI	Dermatitis [1.32]	Neonatal [0.8]	Congenital [0.42]	Asthma [0.87]	Road Inj [0.39]	URI [1.16]	Diarrhea [0.58]	Skin Viral [0.9]	Drown [0.36]	Urticaria [0.74]
Central Europe, Eastern Europe, and Central Asia	LRI [3.5]	Congenital [0.82]	Neonatal [1.26]	Diarrhea [2.0]	Drown [1.13]	Dermatitis [0.54]	Iron [3.69]	Fire [1.29]	Road Inj [0.49]	Falls [1.23]
Central Asia	LRI [2.89]	Congenital [0.68]	Drown [1.2]	Diarrhea [1.67]	Neonatal [0.86]	Iron [2.76]	Fire [1.01]	Vit A [1.26]	Encepha [1.78]	Road Inj [0.39]
Armenia	Congenital [0.64]	LRI [0.87]	Iron [5.43]	Neonatal [0.83]	Diarrhea [0.81]	Dermatitis [0.48]	Urticaria [1.05]	Skin Viral [0.83]	Brain C [0.95]	F Body [0.53]
Azerbaijan	LRI [4.46]	Congenital [0.78]	Neonatal [0.87]	Fire [2.1]	Diarrhea [1.19]	Encepha [3.46]	Leukemia [1.24]	Drown [0.57]	Whooping [12.14]	Hemog [5.03]
Georgia	Congenital [0.52]	LRI [0.69]	Neonatal [0.87]	Diarrhea [0.77]	Dermatitis [0.44]	Fire [0.79]	Urticaria [1.06]	Asthma [0.53]	Leukemia [0.65]	Vit A [0.83]
Kazakhstan	Congenital [1.15]	LRI [1.61]	Neonatal [0.99]	Drown [1.38]	Road Inj [0.98]	Vit A [2.49]	F Body [1.7]	Encepha [3.98]	Diarrhea [0.93]	Fire [1.29]
Kyrgyzstan	LRI [0.84]	Congenital [0.48]	Drown [0.77]	Neonatal [0.85]	Diarrhea [0.63]	Iron [2.03]	Vit A [0.92]	Hernia [5.31]	Road Inj [0.28]	F Body [0.57]
Mongolia	LRI [2.03]	Congenital [0.68]	Fire [2.64]	Neonatal [0.96]	Drown [0.57]	Road Inj [0.61]	F Body [1.14]	Diarrhea [0.67]	Vit A [1.44]	Falls [1.62]
Tajikistan	LRI [1.27]	Diarrhea [1.24]	Congenital [0.67]	Drown [0.89]	Neonatal [0.76]	Whooping [1.15]	Fire [0.75]	Iron [0.75]	Meningitis [0.19]	Epilepsy [1.12]
Turkmenistan	LRI [4.91]	Congenital [1.15]	Neonatal [0.94]	Drown [0.99]	Diarrhea [0.96]	F Body [1.11]	Encepha [1.97]	Dermatitis [0.48]	TB [7.01]	Fire [0.8]
Uzbekistan	LRI [1.98]	Drown [1.08]	Iron [3.11]	Congenital [0.32]	Neonatal [0.75]	Encepha [1.52]	Vit A [0.98]	F Body [0.66]	Road Inj [0.31]	Diarrhea [0.31]
Central Europe	Neonatal [1.31]	Congenital [0.76]	Diarrhea [1.89]	Dermatitis [0.59]	LRI [1.3]	Urticaria [1.31]	Falls [1.2]	Asthma [0.57]	Vit A [2.15]	Skin Viral [0.85]
Albania	Congenital [0.71]	LRI [0.73]	Neonatal [1.0]	Diarrhea [0.65]	Epilepsy [1.2]	Dermatitis [0.46]	Leukemia [0.75]	Drown [0.29]	Hernia [3.59]	Brain C [1.23]
Bosnia	Neonatal [1.08]	Congenital [0.45]	Whooping [14.0]	Dermatitis [0.55]	Diarrhea [0.79]	Vit A [1.3]	Urticaria [1.08]	Hernia [3.41]	Asthma [0.48]	Falls [0.86]
Bulgaria	Congenital [0.89]	Neonatal [1.16]	LRI [1.28]	Diarrhea [1.72]	Dermatitis [0.49]	Falls [1.35]	Urticaria [1.24]	Vit A [1.85]	Hernia [2.91]	Skin Viral [0.84]
Croatia	Neonatal [1.39]	Congenital [0.78]	Diarrhea [1.71]	Dermatitis [0.51]	Urticaria [1.35]	Hernia [3.77]	Skin Viral [0.86]	Asthma [0.55]	Road Inj [0.47]	Epilepsy [1.04]
Czech	Neonatal [0.99]	Congenital [0.63]	Diarrhea [2.11]	Dermatitis [0.57]	Falls [1.33]	Urticaria [1.44]	Hernia [3.47]	Skin Viral [0.88]	Asthma [0.49]	Epilepsy [1.05]
Hungary	Neonatal [1.27]	Congenital [0.82]	Diarrhea [2.68]	Dermatitis [0.68]	Urticaria [1.31]	Falls [1.08]	Skin Viral [0.85]	Endocrine [0.9]	Epilepsy [0.96]	Asthma [0.45]
Macedonia	Neonatal [1.12]	Congenital [0.49]	Diarrhea [1.3]	Vit A [2.05]	Dermatitis [0.51]	LRI [0.52]	Whooping [18.16]	Urticaria [1.15]	Falls [1.15]	Road Inj [0.38]
Montenegro	Neonatal [1.22]	Congenital [0.41]	Dermatitis [0.53]	Whooping [21.39]	Urticaria [1.24]	Diarrhea [0.84]	Vit A [1.75]	Falls [1.05]	Hernia [3.12]	Skin Viral [0.84]
Poland	Neonatal [1.52]	Congenital [0.89]	Dermatitis [0.66]	Diarrhea [1.89]	Urticaria [1.42]	Asthma [0.74]	Falls [1.14]	Skin Viral [0.87]	Upper Digest [5.19]	Vit A [2.35]
Romania	LRI [2.65]	Neonatal [1.26]	Congenital [0.68]	Diarrhea [2.1]	Dermatitis [0.56]	Iron [4.98]	Falls [1.46]	Vit A [2.16]	Road Inj [0.51]	Urticaria [1.2]
Serbia	Neonatal [1.41]	Congenital [0.4]	Dermatitis [0.54]	Diarrhea [0.99]	Vit A [1.4]	Urticaria [1.15]	Hernia [3.33]	Falls [0.93]	Skin Viral [0.83]	Whooping [8.37]
Slovakia	Congenital [1.03]	Neonatal [1.07]	Diarrhea [2.35]	Dermatitis [0.56]	LRI [1.76]	Urticaria [1.4]	Falls [1.3]	Hernia [3.75]	Skin Viral [0.87]	Road Inj [0.53]
Slovenia	Neonatal [1.11]	Congenital [0.72]	Diarrhea [2.32]	Dermatitis [0.51]	Urticaria [1.48]	Falls [1.31]	Asthma [0.69]	Hernia [3.19]	Skin Viral [0.89]	Endocrine [0.97]

eFigure 7c. Leading ten causes of DALYs with the ratio of observed DALYs to DALYs expected on the basis of Socio-Demographic Index alone in 2017, 1-4 years, both sexes combined. The top ten causes contributing to DALYs are listed globally, by socio-demographic quintile, and then by GBD superregion, region, country, and subnationally where modeled. For each cell, the ratio of observed DALYs to DALYs expected on the basis of socio-demographic index (SDI) alone are listed. Abbreviations: DALY=disability-adjusted life year, GBD=Global Burden of Disease.

Values shown in brackets represent the ratio of observed DALYs to predicted DALYs on the basis of Socio-Demographic Index (SDI), rounded to two (2) digits. Color ranges (shown below) were calculated to place a roughly equal number of cells into each bin.

COLOR KEY:	[0.0-0.5]	(0.5-0.72]	(0.72-0.86]	(0.86-0.97]	(0.97-1.1]	(1.1-1.23]	(1.23-1.41]	(1.41-2.15]	2.15+

	1	2	3	4	5	6	7	8	9	10
**Eastern Europe**	Neonatal [1.55]	Congenital [0.86]	Dermatitis [0.57]	Diarrhea [1.37]	LRI [0.83]	Drown [0.7]	Falls [1.43]	Fire [1.44]	Urticaria [1.28]	Road Inj [0.52]
Belarus	Neonatal [1.4]	Congenital [0.87]	Dermatitis [0.62]	Fire [1.72]	Drown [0.75]	Falls [1.57]	Urticaria [1.21]	Diarrhea [0.79]	Encepha [3.21]	LRI [0.44]
Estonia	Neonatal [1.59]	Congenital [0.85]	Dermatitis [0.79]	Diarrhea [2.42]	Urticaria [1.49]	Drown [1.13]	LRI [1.59]	Falls [1.17]	Fire [2.16]	Skin Viral [0.89]
Latvia	Neonatal [1.39]	Congenital [0.77]	Diarrhea [2.15]	Dermatitis [0.58]	Drown [1.12]	Fire [2.19]	Urticaria [1.35]	LRI [0.9]	Falls [1.15]	Asthma [0.57]
Lithuania	Neonatal [1.63]	Congenital [0.93]	Diarrhea [2.4]	Drown [1.26]	LRI [1.39]	Dermatitis [0.36]	Urticaria [1.41]	Falls [1.3]	Upper Digest [5.79]	Skin Viral [0.88]
Moldova	Neonatal [1.3]	Congenital [0.6]	LRI [0.59]	Drown [0.49]	Dermatitis [0.68]	F Body [0.88]	Diarrhea [0.56]	Poison [2.76]	Road Inj [0.35]	Falls [1.19]
Russian Federation	Neonatal [1.59]	Congenital [0.77]	Diarrhea [1.61]	Dermatitis [0.57]	LRI [0.86]	Falls [1.43]	Road Inj [0.56]	Drown [0.66]	Urticaria [1.29]	Fire [1.44]
Ukraine	Congenital [1.02]	Neonatal [1.4]	Dermatitis [0.59]	LRI [0.5]	Drown [0.63]	Meningitis [0.82]	Leukemia [0.95]	Fire [1.13]	Falls [1.33]	Urticaria [1.18]
**High-income**	Dermatitis [1.31]	Neonatal [0.93]	Congenital [0.73]	Asthma [1.13]	Diarrhea [1.21]	URI [1.16]	Road Inj [0.76]	Skin Viral [0.96]	Drown [0.87]	ASD [1.15]
**Australasia**	Dermatitis [1.14]	Neonatal [0.93]	Asthma [2.09]	Congenital [0.57]	Falls [1.49]	Drown [1.24]	Road Inj [0.85]	URI [1.02]	Skin Viral [1.0]	Urticaria [1.26]
Australia	Dermatitis [1.08]	Neonatal [0.91]	Asthma [2.15]	Congenital [0.57]	Falls [1.44]	Drown [1.31]	Road Inj [0.84]	URI [1.02]	Skin Viral [1.0]	Urticaria [1.27]
New Zealand	Dermatitis [1.42]	Neonatal [1.03]	Asthma [1.8]	Congenital [0.56]	Falls [1.73]	Road Inj [0.83]	Drown [0.92]	URI [1.03]	Urticaria [1.19]	Skin Viral [0.96]
**High-income Asia Pacific**	Dermatitis [1.26]	Neonatal [1.18]	Congenital [0.77]	Asthma [1.03]	Diarrhea [1.35]	URI [1.02]	Skin Viral [0.99]	Falls [1.04]	Urticaria [1.17]	LRI [1.42]
Brunei	Neonatal [1.29]	Dermatitis [1.2]	Congenital [0.86]	LRI [3.16]	Road Inj [1.23]	Asthma [1.01]	Drown [1.42]	Falls [1.44]	Endocrine [1.58]	Diarrhea [1.23]
Japan	Dermatitis [1.32]	Neonatal [1.14]	Congenital [0.84]	Asthma [1.02]	Diarrhea [1.32]	LRI [1.56]	URI [1.03]	Skin Viral [0.99]	Urticaria [1.17]	ASD [1.36]
Aichi	Dermatitis [1.32]	Neonatal [1.17]	Congenital [0.84]	Asthma [1.05]	ASD [2.01]	Diarrhea [1.42]	URI [1.03]	Skin Viral [1.0]	LRI [1.61]	Urticaria [1.21]
Akita	Neonatal [1.19]	Dermatitis [1.33]	Congenital [0.77]	Asthma [0.94]	Diarrhea [1.27]	Drown [0.89]	LRI [1.07]	URI [1.03]	Skin Viral [0.96]	Endocrine [1.07]
Aomori	Dermatitis [1.33]	Neonatal [1.12]	Congenital [0.78]	Asthma [0.94]	LRI [1.26]	Diarrhea [1.26]	URI [1.03]	Skin Viral [0.95]	Endocrine [1.08]	Urticaria [1.05]
Chiba	Dermatitis [1.32]	Neonatal [1.14]	Congenital [0.95]	Asthma [1.02]	LRI [1.69]	Diarrhea [1.23]	URI [1.03]	Skin Viral [0.98]	Endocrine [1.17]	Urticaria [1.15]
Ehime	Dermatitis [1.32]	Neonatal [0.92]	Congenital [0.63]	Asthma [0.95]	Diarrhea [1.1]	URI [1.03]	Skin Viral [0.96]	Urticaria [1.09]	ASD [1.35]	Endocrine [1.01]
Fukui	Dermatitis [1.32]	Neonatal [1.09]	Congenital [0.68]	Asthma [0.97]	Diarrhea [1.43]	URI [1.02]	Skin Viral [0.97]	Urticaria [1.12]	ASD [1.35]	Drown [0.75]
Fukuoka	Dermatitis [1.32]	Neonatal [1.11]	Congenital [0.82]	Asthma [1.0]	Diarrhea [1.27]	LRI [1.51]	URI [1.02]	Skin Viral [0.98]	Urticaria [1.14]	Endocrine [1.14]
Fukushima	Dermatitis [1.32]	Neonatal [1.02]	Congenital [0.75]	Asthma [0.95]	Diarrhea [1.24]	LRI [0.99]	URI [1.03]	Skin Viral [0.95]	Urticaria [1.06]	ASD [1.34]
Gifu	Dermatitis [1.32]	Neonatal [1.15]	Congenital [0.77]	Asthma [0.98]	Diarrhea [1.44]	URI [1.02]	LRI [1.22]	Skin Viral [0.97]	Endocrine [1.17]	Urticaria [1.12]
Gunma	Dermatitis [1.32]	Neonatal [1.08]	Congenital [0.77]	Asthma [0.99]	Diarrhea [1.34]	LRI [1.25]	URI [1.03]	Skin Viral [0.97]	Endocrine [1.17]	Urticaria [1.12]
Hiroshima	Dermatitis [1.32]	Neonatal [1.17]	Congenital [0.74]	Asthma [1.02]	Diarrhea [1.41]	URI [1.02]	Skin Viral [0.99]	Urticaria [1.17]	ASD [1.34]	LRI [1.31]
Hokkaido	Dermatitis [1.32]	Neonatal [1.07]	Congenital [0.72]	Asthma [0.97]	Diarrhea [1.3]	LRI [1.14]	URI [1.03]	Skin Viral [0.96]	Urticaria [1.09]	ASD [1.34]
Hyogo	Dermatitis [1.32]	Neonatal [1.14]	Congenital [0.73]	Diarrhea [1.42]	Asthma [0.88]	URI [1.03]	Skin Viral [0.98]	LRI [1.38]	Urticaria [1.15]	ASD [1.35]
Ibaraki	Dermatitis [1.32]	Neonatal [1.16]	Congenital [0.8]	Asthma [0.99]	Diarrhea [1.1]	URI [1.03]	Skin Viral [0.97]	LRI [1.17]	Urticaria [1.12]	Road Inj [0.57]
Ishikawa	Dermatitis [1.32]	Neonatal [1.08]	Congenital [0.75]	Asthma [0.99]	Diarrhea [1.35]	URI [1.03]	Skin Viral [0.98]	LRI [1.24]	Urticaria [1.14]	ASD [1.34]
Iwate	Dermatitis [1.33]	Neonatal [1.14]	Congenital [0.8]	Asthma [0.94]	LRI [1.22]	Diarrhea [1.2]	URI [1.03]	Skin Viral [0.95]	Drown [0.7]	F Body [1.33]

eFigure 7c. Leading ten causes of DALYs with the ratio of observed DALYs to DALYs expected on the basis of Socio-Demographic Index alone in 2017, 1-4 years, both sexes combined. The top ten causes contributing to DALYs are listed globally, by socio-demographic quintile, and then by GBD superregion, region, country, and subnationally where modeled. For each cell, the ratio of observed DALYs to DALYs expected on the basis of socio-demographic index (SDI) alone are listed. Abbreviations: DALY=disability-adjusted life year, GBD=Global Burden of Disease.

Values shown in brackets represent the ratio of observed DALYs to predicted DALYs on the basis of Socio-Demographic Index (SDI), rounded to two (2) digits. Color ranges (shown below) were calculated to place a roughly equal number of cells into each bin.

COLOR KEY:	[0.0-0.5]	(0.5-0.72]	(0.72-0.86]	(0.86-0.97]	(0.97-1.1]	(1.1-1.23]	(1.23-1.41]	(1.41-2.15]	2.15+

	1	2	3	4	5	6	7	8	9	10
Kagawa	Dermatitis [1.32]	Neonatal [1.06]	Congenital [0.73]	Diarrhea [1.59]	Asthma [0.98]	URI [1.03]	LRI [1.2]	Skin Viral [0.97]	Urticaria [1.12]	Road Inj [0.58]
Kagoshima	Dermatitis [1.33]	Neonatal [0.95]	Congenital [0.67]	Asthma [0.94]	Diarrhea [1.24]	LRI [0.96]	URI [1.03]	Skin Viral [0.95]	Urticaria [1.06]	ASD [1.34]
Kanagawa	Dermatitis [1.32]	Neonatal [1.19]	Congenital [0.96]	Asthma [1.06]	ASD [1.62]	LRI [1.84]	Diarrhea [1.14]	URI [1.03]	Skin Viral [1.0]	Urticaria [1.21]
Kochi	Dermatitis [1.32]	Neonatal [1.03]	Congenital [0.77]	Asthma [0.93]	Diarrhea [1.36]	LRI [1.2]	Drown [0.84]	URI [1.03]	Skin Viral [0.95]	Endocrine [1.09]
Kumamoto	Dermatitis [1.33]	Neonatal [0.98]	Congenital [0.65]	Asthma [0.94]	Diarrhea [1.18]	URI [1.03]	Skin Viral [0.95]	LRI [0.86]	Endocrine [1.07]	Urticaria [1.06]
Kyoto	Congenital [1.08]	Neonatal [1.2]	Dermatitis [1.09]	Asthma [1.05]	Diarrhea [1.45]	LRI [2.02]	URI [1.02]	Skin Viral [1.0]	Endocrine [1.26]	Urticaria [1.21]
Mie	Dermatitis [1.32]	Neonatal [1.16]	Congenital [0.75]	Diarrhea [1.65]	Asthma [0.99]	URI [1.03]	LRI [1.29]	Skin Viral [0.98]	Endocrine [1.2]	Road Inj [0.62]
Miyagi	Dermatitis [1.32]	Congenital [0.96]	Neonatal [1.08]	Diarrhea [1.31]	Asthma [0.83]	LRI [1.48]	URI [1.03]	Skin Viral [0.97]	Drown [0.88]	Endocrine [1.2]
Miyazaki	Dermatitis [1.33]	Neonatal [0.97]	Congenital [0.55]	Asthma [0.92]	URI [1.03]	Diarrhea [0.91]	Skin Viral [0.95]	Urticaria [1.05]	ASD [1.33]	Endocrine [0.91]
Nagano	Dermatitis [1.32]	Neonatal [1.16]	Congenital [0.72]	Diarrhea [1.59]	Asthma [0.98]	URI [1.03]	Skin Viral [0.97]	Urticaria [1.12]	ASD [1.34]	Endocrine [1.09]
Nagasaki	Dermatitis [1.33]	Neonatal [0.99]	Congenital [0.71]	Asthma [0.92]	Diarrhea [1.06]	LRI [0.9]	URI [1.03]	Skin Viral [0.95]	Urticaria [1.05]	Endocrine [1.04]
Nara	Dermatitis [1.32]	Congenital [0.87]	Neonatal [1.01]	Asthma [0.99]	Diarrhea [1.31]	LRI [1.37]	URI [1.03]	Skin Viral [0.97]	Urticaria [1.12]	ASD [1.34]
Niigata	Dermatitis [1.32]	Neonatal [1.11]	Congenital [0.67]	Asthma [0.96]	Diarrhea [1.17]	URI [1.03]	Skin Viral [0.97]	Urticaria [1.11]	ASD [1.34]	Endocrine [1.0]
Oita	Dermatitis [1.32]	Neonatal [1.16]	Congenital [0.67]	Asthma [0.96]	Diarrhea [1.36]	URI [1.03]	Skin Viral [0.96]	Urticaria [1.1]	ASD [1.36]	LRI [0.98]
Okayama	Dermatitis [1.32]	Neonatal [1.18]	Congenital [0.7]	Asthma [1.0]	Diarrhea [1.3]	URI [1.02]	LRI [1.3]	Skin Viral [0.98]	Urticaria [1.13]	ASD [1.34]
Okinawa	Dermatitis [1.33]	Neonatal [1.0]	Congenital [0.44]	Asthma [0.8]	Diarrhea [0.99]	URI [1.03]	Skin Viral [0.95]	Urticaria [1.04]	ASD [1.33]	Endocrine [0.94]
Osaka	Dermatitis [1.32]	Neonatal [1.08]	Congenital [0.82]	Asthma [1.05]	Diarrhea [1.27]	LRI [1.72]	URI [1.02]	Skin Viral [0.99]	Urticaria [1.19]	Endocrine [1.15]
Saga	Dermatitis [1.33]	Neonatal [1.03]	Congenital [0.58]	Asthma [0.95]	Diarrhea [1.17]	URI [1.03]	Skin Viral [0.96]	Urticaria [1.07]	ASD [1.32]	LRI [0.84]
Saitama	Dermatitis [1.32]	Neonatal [1.1]	Congenital [0.82]	Asthma [0.99]	Diarrhea [1.25]	LRI [1.45]	URI [1.03]	Skin Viral [0.97]	Endocrine [1.17]	Urticaria [1.13]
Shiga	Dermatitis [1.57]	Neonatal [1.08]	Congenital [0.8]	Asthma [1.03]	Diarrhea [1.44]	URI [1.03]	Skin Viral [0.99]	Urticaria [1.19]	Endocrine [1.19]	ASD [1.36]
Shimane	Dermatitis [1.32]	Neonatal [1.0]	Congenital [0.57]	Asthma [0.93]	Diarrhea [1.22]	URI [1.02]	Skin Viral [0.95]	Urticaria [1.06]	ASD [1.35]	Endocrine [0.93]
Shizuoka	Dermatitis [1.32]	Neonatal [1.22]	Congenital [0.72]	Asthma [1.0]	Diarrhea [1.28]	URI [1.02]	Skin Viral [0.98]	Urticaria [1.16]	ASD [1.35]	Endocrine [1.13]
Tochigi	Congenital [1.07]	Dermatitis [1.32]	Neonatal [1.2]	Asthma [1.01]	LRI [1.83]	Diarrhea [1.22]	Road Inj [0.72]	URI [1.03]	Skin Viral [0.98]	Endocrine [1.16]
Tokushima	Dermatitis [1.32]	Neonatal [1.11]	Congenital [0.67]	Asthma [0.97]	Diarrhea [1.34]	URI [1.02]	Skin Viral [0.97]	Urticaria [1.11]	LRI [1.02]	ASD [1.34]
Tokyo	Dermatitis [1.33]	Neonatal [1.3]	Congenital [1.27]	Asthma [1.21]	LRI [3.99]	Diarrhea [1.44]	URI [1.03]	Skin Viral [1.06]	Urticaria [1.4]	Endocrine [1.41]
Tottori	Dermatitis [1.32]	Neonatal [1.1]	Congenital [0.73]	Asthma [0.95]	Diarrhea [1.15]	Drown [0.8]	URI [1.02]	Skin Viral [0.96]	LRI [0.93]	Endocrine [1.11]
Toyama	Dermatitis [1.32]	Neonatal [1.09]	Congenital [0.76]	Asthma [1.0]	Diarrhea [1.35]	Drown [1.14]	URI [1.03]	LRI [1.39]	Skin Viral [0.98]	Urticaria [1.15]
Wakayama	Dermatitis [1.32]	Neonatal [1.14]	Congenital [0.56]	Asthma [0.95]	Diarrhea [1.12]	URI [1.03]	Skin Viral [0.96]	Urticaria [1.09]	ASD [1.35]	Endocrine [0.97]
Yamagata	Dermatitis [1.33]	Neonatal [0.95]	Congenital [0.69]	Asthma [0.94]	Diarrhea [1.25]	URI [1.03]	Skin Viral [0.96]	LRI [0.86]	Drown [0.7]	Urticaria [1.06]
Yamaguchi	Dermatitis [1.32]	Neonatal [1.0]	Congenital [0.72]	Asthma [0.98]	Diarrhea [1.4]	LRI [1.4]	URI [1.03]	Skin Viral [0.97]	Urticaria [1.12]	ASD [1.35]
Yamanashi	Dermatitis [1.32]	Neonatal [1.23]	Congenital [0.75]	Asthma [0.99]	Diarrhea [1.25]	URI [1.03]	Skin Viral [0.98]	Urticaria [1.14]	ASD [1.32]	Falls [0.92]

eFigure 7c. Leading ten causes of DALYs with the ratio of observed DALYs to DALYs expected on the basis of Socio-Demographic Index alone in 2017, 1-4 years, both sexes combined. The top ten causes contributing to DALYs are listed globally, by socio-demographic quintile, and then by GBD superregion, region, country, and subnationally where modeled. For each cell, the ratio of observed DALYs to DALYs expected on the basis of socio-demographic index (SDI) alone are listed. Abbreviations: DALY=disability-adjusted life year, GBD=Global Burden of Disease.

Values shown in brackets represent the ratio of observed DALYs to predicted DALYs on the basis of Socio-Demographic Index (SDI), rounded to two (2) digits. Color ranges (shown below) were calculated to place a roughly equal number of cells into each bin.

COLOR KEY:	[0.0-0.5]	(0.5-0.72]	(0.72-0.86]	(0.86-0.97]	(0.97-1.1]	(1.1-1.23]	(1.23-1.41]	(1.41-2.15]	2.15+

	1	2	3	4	5	6	7	8	9	10
S Korea	Neonatal [1.24]	Dermatitis [1.16]	Congenital [0.6]	Asthma [1.02]	Iron [13.49]	Diarrhea [1.36]	Road Inj [0.91]	Falls [1.29]	Skin Viral [1.0]	URI [1.0]
Singapore	Neonatal [1.12]	Dermatitis [1.1]	Congenital [0.42]	Asthma [1.02]	Diarrhea [1.25]	Skin Viral [1.0]	URI [1.01]	Urticaria [1.14]	Falls [0.92]	ASD [1.18]
**High-income North America**	Dermatitis [1.61]	Neonatal [0.96]	Congenital [0.77]	Asthma [1.49]	Road Inj [1.25]	Drown [1.49]	Violence [3.61]	URI [1.17]	Skin Viral [0.97]	Urticaria [1.16]
Canada	Dermatitis [1.75]	Congenital [0.7]	Neonatal [0.7]	Asthma [1.68]	URI [1.18]	Skin Viral [1.1]	Road Inj [0.81]	ASD [1.53]	Diarrhea [1.05]	Urticaria [1.2]
Greenland	Congenital [0.89]	Dermatitis [1.68]	Violence [7.73]	Fire [4.31]	Drown [1.78]	Neonatal [0.93]	Meningitis [2.37]	LRI [1.12]	HIV [12.77]	Asthma [1.32]
USA	Dermatitis [1.6]	Neonatal [0.98]	Congenital [0.76]	Asthma [1.46]	Road Inj [1.26]	Violence [3.79]	Drown [1.5]	URI [1.17]	Skin Viral [0.95]	Urticaria [1.14]
Alabama	Dermatitis [1.41]	Neonatal [0.99]	Congenital [0.71]	Road Inj [1.9]	Violence [4.69]	Asthma [1.34]	Drown [1.67]	Fire [2.22]	URI [1.18]	Skin Viral [1.06]
Alaska	Dermatitis [1.45]	Neonatal [0.9]	Congenital [0.75]	Drown [2.81]	Road Inj [1.23]	Asthma [1.12]	F Body [2.39]	Violence [3.23]	Fire [2.7]	URI [1.18]
Arizona	Dermatitis [1.67]	Neonatal [0.94]	Congenital [0.74]	Road Inj [1.56]	Asthma [1.42]	Drown [1.81]	Violence [3.98]	URI [1.17]	Urticaria [1.08]	ASD [1.26]
Arkansas	Dermatitis [1.29]	Neonatal [0.85]	Congenital [0.61]	Road Inj [1.39]	Drown [1.26]	Violence [3.64]	Asthma [1.0]	URI [1.18]	Fire [1.6]	Skin Viral [0.89]
California	Dermatitis [1.8]	Neonatal [0.82]	Congenital [0.69]	Asthma [1.42]	Road Inj [0.94]	Drown [1.36]	Violence [3.19]	URI [1.17]	ASD [1.41]	Urticaria [1.12]
Colorado	Dermatitis [1.46]	Neonatal [0.98]	Congenital [0.8]	Asthma [1.34]	Road Inj [1.4]	Drown [1.38]	URI [1.19]	Violence [3.09]	Urticaria [1.14]	F Body [1.68]
Connecticut	Dermatitis [1.78]	Neonatal [1.16]	Asthma [1.99]	Congenital [0.65]	URI [1.16]	Skin Viral [1.14]	Urticaria [1.44]	Road Inj [0.76]	ASD [1.17]	Diarrhea [0.86]
Delaware	Dermatitis [1.42]	Neonatal [0.99]	Congenital [0.79]	Asthma [1.59]	Road Inj [1.47]	URI [1.17]	Violence [3.06]	Drown [1.24]	Skin Viral [1.1]	Urticaria [1.21]
DC	Dermatitis [2.06]	Violence [18.18]	Congenital [1.13]	Neonatal [1.16]	Asthma [1.69]	Road Inj [1.29]	Endocrine [2.08]	LRI [3.13]	Drown [1.8]	Fire [3.67]
Florida	Dermatitis [1.6]	Neonatal [1.08]	Drown [3.74]	Congenital [0.83]	Asthma [1.77]	Violence [5.37]	Road Inj [1.43]	Skin Viral [1.15]	URI [1.17]	Urticaria [1.21]
Georgia	Dermatitis [1.73]	Neonatal [0.94]	Congenital [0.7]	Asthma [1.55]	Road Inj [1.52]	Violence [4.88]	Drown [1.55]	URI [1.17]	Skin Viral [1.03]	LRI [1.05]
Hawaii	Dermatitis [1.83]	Neonatal [0.88]	Congenital [0.66]	Asthma [1.65]	Drown [2.04]	Road Inj [0.93]	URI [1.16]	Skin Viral [1.13]	Urticaria [1.38]	ASD [1.16]
Idaho	Dermatitis [1.35]	Neonatal [0.76]	Congenital [0.51]	Road Inj [0.99]	Asthma [0.82]	URI [1.18]	Drown [0.89]	Skin Viral [1.04]	ASD [1.15]	Diarrhea [0.67]
Illinois	Dermatitis [1.41]	Neonatal [1.13]	Congenital [0.79]	Asthma [1.32]	Violence [4.28]	Road Inj [0.9]	URI [1.16]	Urticaria [1.25]	Skin Viral [0.94]	Drown [1.05]
Indiana	Dermatitis [1.37]	Neonatal [0.94]	Congenital [0.75]	Asthma [1.35]	Road Inj [1.15]	Violence [3.36]	Drown [1.11]	URI [1.17]	Skin Viral [0.81]	Urticaria [0.97]
Iowa	Dermatitis [1.23]	Congenital [0.8]	Neonatal [0.91]	Road Inj [1.38]	Asthma [0.91]	URI [1.17]	Drown [1.14]	Skin Viral [0.93]	Urticaria [1.1]	ASD [1.15]
Kansas	Dermatitis [1.31]	Neonatal [0.99]	Congenital [0.72]	Asthma [1.35]	Road Inj [1.41]	URI [1.17]	Drown [1.15]	Violence [2.7]	Skin Viral [0.86]	Urticaria [1.08]
Kentucky	Dermatitis [1.39]	Neonatal [0.94]	Congenital [0.72]	Road Inj [1.37]	Asthma [1.33]	Drown [1.16]	Violence [2.73]	URI [1.17]	Fire [1.89]	Skin Viral [0.97]
Louisiana	Dermatitis [1.48]	Congenital [0.89]	Neonatal [0.95]	Violence [7.38]	Road Inj [1.88]	Drown [2.35]	Asthma [1.24]	Fire [2.44]	Skin Viral [1.32]	F Body [2.06]
Maine	Dermatitis [1.21]	Neonatal [0.99]	Congenital [0.74]	Asthma [1.61]	Road Inj [1.17]	Drown [1.28]	URI [1.2]	Urticaria [1.15]	Skin Viral [0.81]	Diarrhea [0.85]
Maryland	Dermatitis [1.78]	Neonatal [1.17]	Congenital [0.92]	Asthma [1.77]	Violence [6.38]	Road Inj [1.37]	URI [1.17]	Drown [1.52]	Urticaria [1.29]	Skin Viral [0.94]
Massachusetts	Dermatitis [1.59]	Neonatal [1.15]	Congenital [0.89]	Asthma [1.78]	URI [1.17]	Skin Viral [1.1]	ASD [1.55]	Urticaria [1.51]	Road Inj [0.95]	Drown [1.37]
Michigan	Dermatitis [1.43]	Neonatal [1.01]	Congenital [0.82]	Asthma [1.56]	Violence [4.47]	Road Inj [1.14]	URI [1.16]	Drown [1.21]	Skin Viral [1.02]	Urticaria [1.18]
Minnesota	Dermatitis [1.26]	Neonatal [1.01]	Congenital [0.82]	Asthma [1.15]	URI [1.17]	Road Inj [0.99]	Skin Viral [1.01]	Urticaria [1.27]	ASD [1.37]	Drown [1.11]
Mississippi	Dermatitis [1.29]	Road Inj [2.22]	Congenital [0.72]	Neonatal [0.93]	Violence [5.21]	Drown [1.59]	Fire [2.7]	Asthma [1.0]	F Body [1.79]	URI [1.18]

eFigure 7c. Leading ten causes of DALYs with the ratio of observed DALYs to DALYs expected on the basis of Socio-Demographic Index alone in 2017, 1-4 years, both sexes combined. The top ten causes contributing to DALYs are listed globally, by socio-demographic quintile, and then by GBD superregion, region, country, and subnationally where modeled. For each cell, the ratio of observed DALYs to DALYs expected on the basis of socio-demographic index (SDI) alone are listed. Abbreviations: DALY=disability-adjusted life year, GBD=Global Burden of Disease.

Values shown in brackets represent the ratio of observed DALYs to predicted DALYs on the basis of Socio-Demographic Index (SDI), rounded to two (2) digits. Color ranges (shown below) were calculated to place a roughly equal number of cells into each bin.

COLOR KEY:	[0.0-0.5]	(0.5-0.72]	(0.72-0.86]	(0.86-0.97]	(0.97-1.1]	(1.1-1.23]	(1.23-1.41]	(1.41-2.15]	2.15+

	1	2	3	4	5	6	7	8	9	10
Missouri	Dermatitis [1.24]	Neonatal [0.98]	Congenital [0.75]	Asthma [1.36]	Road Inj [1.34]	Violence [4.37]	Drown [1.11]	URI [1.17]	Skin Viral [0.87]	F Body [1.46]
Montana	Dermatitis [1.26]	Neonatal [0.97]	Congenital [0.81]	Road Inj [2.02]	Drown [1.72]	Asthma [1.06]	URI [1.22]	Skin Viral [0.94]	Violence [2.26]	F Body [1.51]
Nebraska	Dermatitis [1.25]	Neonatal [0.92]	Congenital [0.78]	Road Inj [1.28]	Asthma [0.94]	URI [1.17]	Skin Viral [0.96]	Drown [0.99]	Urticaria [1.14]	ASD [1.17]
Nevada	Dermatitis [1.58]	Neonatal [0.91]	Congenital [0.65]	Asthma [1.27]	Road Inj [1.23]	Violence [4.21]	Drown [1.35]	URI [1.17]	Urticaria [1.02]	LRI [0.94]
New Hampshire	Dermatitis [1.23]	Neonatal [0.97]	Congenital [0.8]	Asthma [1.5]	Road Inj [1.1]	URI [1.17]	Skin Viral [0.92]	Drown [1.22]	Urticaria [1.2]	ASD [1.17]
New Jersey	Dermatitis [1.94]	Neonatal [1.07]	Congenital [0.85]	Asthma [2.05]	URI [1.15]	ASD [1.7]	Skin Viral [1.12]	Urticaria [1.37]	Road Inj [0.85]	Violence [2.77]
New Mexico	Dermatitis [1.46]	Neonatal [1.01]	Congenital [0.76]	Road Inj [1.77]	Asthma [1.38]	Violence [3.61]	Drown [1.2]	URI [1.2]	Endocrine [1.0]	Urticaria [0.96]
New York	Dermatitis [1.95]	Neonatal [1.12]	Congenital [0.96]	Asthma [2.17]	Violence [4.44]	URI [1.16]	Skin Viral [1.17]	Road Inj [0.87]	Urticaria [1.38]	Drown [1.06]
N Carolina	Dermatitis [1.49]	Neonatal [0.97]	Congenital [0.74]	Road Inj [1.51]	Asthma [1.29]	Violence [4.06]	Drown [1.35]	URI [1.17]	Skin Viral [0.97]	Urticaria [1.11]
N Dakota	Dermatitis [1.23]	Neonatal [1.0]	Congenital [0.89]	Road Inj [1.49]	Asthma [1.21]	URI [1.2]	Drown [1.31]	Skin Viral [0.91]	ASD [1.16]	Urticaria [0.98]
Ohio	Dermatitis [1.51]	Congenital [0.81]	Neonatal [0.95]	Asthma [1.4]	Violence [3.64]	Road Inj [0.99]	Drown [1.2]	URI [1.19]	Urticaria [1.15]	Skin Viral [0.9]
Oklahoma	Dermatitis [1.58]	Congenital [0.91]	Neonatal [0.93]	Road Inj [2.01]	Drown [2.15]	Asthma [1.43]	Violence [4.78]	URI [1.18]	Fire [2.04]	LRI [1.03]
Oregon	Dermatitis [1.67]	Neonatal [0.85]	Congenital [0.65]	Asthma [1.16]	Road Inj [1.0]	Drown [1.39]	URI [1.16]	Skin Viral [0.94]	Urticaria [1.11]	ASD [1.16]
Pennsylvania	Dermatitis [1.53]	Neonatal [1.0]	Congenital [0.82]	Asthma [1.59]	Road Inj [1.21]	Violence [3.39]	URI [1.16]	Drown [1.12]	Skin Viral [0.93]	Urticaria [1.21]
Rhode Island	Dermatitis [1.41]	Neonatal [0.97]	Congenital [0.78]	Asthma [1.63]	URI [1.18]	Skin Viral [1.14]	Road Inj [0.91]	Drown [1.26]	Urticaria [1.34]	ASD [1.16]
S Carolina	Dermatitis [1.72]	Neonatal [0.93]	Congenital [0.7]	Road Inj [1.62]	Asthma [1.35]	Violence [4.57]	Drown [1.64]	Fire [2.24]	URI [1.17]	F Body [1.89]
S Dakota	Dermatitis [1.23]	Congenital [0.75]	Neonatal [0.83]	Road Inj [1.52]	Asthma [0.89]	Drown [1.19]	URI [1.17]	Skin Viral [0.98]	ASD [1.15]	Urticaria [0.93]
Tennessee	Dermatitis [1.4]	Neonatal [0.92]	Congenital [0.64]	Road Inj [1.16]	Violence [3.61]	Asthma [1.04]	Drown [1.06]	URI [1.17]	ASD [1.49]	Skin Viral [0.88]
Texas	Dermatitis [1.71]	Neonatal [0.99]	Congenital [0.66]	Asthma [1.45]	Road Inj [1.37]	Violence [4.02]	Drown [1.4]	URI [1.16]	ASD [1.61]	Skin Viral [0.88]
Utah	Dermatitis [1.39]	Neonatal [0.85]	Congenital [0.63]	Road Inj [0.99]	Asthma [0.89]	ASD [1.92]	URI [1.17]	Skin Viral [1.08]	Drown [0.89]	Urticaria [0.94]
Vermont	Dermatitis [1.23]	Neonatal [1.04]	Congenital [0.76]	Asthma [1.39]	Road Inj [1.15]	URI [1.17]	Drown [1.18]	Skin Viral [0.86]	ASD [1.17]	Diarrhea [0.88]
Virginia	Dermatitis [1.81]	Neonatal [1.08]	Congenital [0.78]	Asthma [1.51]	Violence [4.11]	Road Inj [1.13]	Drown [1.52]	URI [1.16]	Urticaria [1.29]	Skin Viral [0.84]
Washington	Dermatitis [1.64]	Neonatal [0.91]	Congenital [0.77]	Asthma [1.12]	Road Inj [1.1]	Drown [1.42]	URI [1.17]	Urticaria [1.29]	Skin Viral [0.96]	Violence [2.45]
W Virginia	Dermatitis [1.43]	Congenital [0.71]	Neonatal [0.94]	Road Inj [1.36]	Asthma [1.39]	Drown [1.05]	Violence [2.72]	URI [1.17]	Endocrine [1.15]	Fire [1.53]
Wisconsin	Dermatitis [1.26]	Neonatal [1.04]	Congenital [0.8]	Asthma [1.13]	Road Inj [1.07]	URI [1.16]	Drown [1.09]	Skin Viral [0.93]	Urticaria [1.13]	Violence [2.21]
Wyoming	Dermatitis [1.37]	Congenital [0.82]	Road Inj [2.18]	Neonatal [0.83]	Asthma [1.11]	Drown [1.68]	URI [1.17]	F Body [1.83]	LRI [1.39]	Violence [2.21]
**Southern Latin America**	Congenital [0.56]	Diarrhea [2.21]	Neonatal [0.85]	Dermatitis [1.05]	LRI [0.42]	Asthma [0.88]	Road Inj [0.5]	Drown [0.5]	F Body [0.77]	Skin Viral [0.92]
Argentina	Diarrhea [2.29]	Congenital [0.56]	Neonatal [0.81]	Dermatitis [1.0]	LRI [0.43]	Drown [0.52]	Asthma [0.85]	Road Inj [0.49]	F Body [0.87]	Skin Viral [0.92]
Chile	Congenital [0.57]	Neonatal [0.95]	Dermatitis [1.22]	Diarrhea [1.92]	Asthma [0.94]	Road Inj [0.55]	Drown [0.45]	Skin Viral [0.93]	Epilepsy [0.9]	Urticaria [0.86]
Uruguay	Congenital [0.49]	Neonatal [0.86]	Dermatitis [0.99]	Diarrhea [1.08]	Asthma [0.97]	LRI [0.35]	F Body [0.78]	Road Inj [0.33]	Skin Viral [0.92]	Drown [0.27]
**Western Europe**	Dermatitis [1.12]	Neonatal [0.83]	Congenital [0.68]	URI [1.29]	Asthma [0.78]	Skin Viral [0.95]	Diarrhea [0.91]	Endocrine [1.0]	ASD [1.04]	Epilepsy [1.01]

eFigure 7c. Leading ten causes of DALYs with the ratio of observed DALYs to DALYs expected on the basis of Socio-Demographic Index alone in 2017, 1-4 years, both sexes combined. The top ten causes contributing to DALYs are listed globally, by socio-demographic quintile, and then by GBD superregion, region, country, and subnationally where modeled. For each cell, the ratio of observed DALYs to DALYs expected on the basis of socio-demographic index (SDI) alone are listed. Abbreviations: DALY=disability-adjusted life year, GBD=Global Burden of Disease.

Values shown in brackets represent the ratio of observed DALYs to predicted DALYs on the basis of Socio-Demographic Index (SDI), rounded to two (2) digits. Color ranges (shown below) were calculated to place a roughly equal number of cells into each bin.

COLOR KEY:	[0.0-0.5]	(0.5-0.72]	(0.72-0.86]	(0.86-0.97]	(0.97-1.1]	(1.1-1.23]	(1.23-1.41]	(1.41-2.15]	2.15+

	1	2	3	4	5	6	7	8	9	10
Andorra	Dermatitis [1.31]	Congenital [1.13]	Neonatal [0.96]	Asthma [0.99]	URI [1.3]	Skin Viral [1.01]	Road Inj [0.76]	Diarrhea [0.97]	Endocrine [1.05]	Leukemia [1.12]
Austria	Neonatal [1.29]	Congenital [0.74]	Dermatitis [0.83]	Diarrhea [1.38]	URI [1.29]	Endocrine [1.39]	Asthma [0.72]	Hernia [3.66]	Skin Viral [0.96]	Falls [0.78]
Belgium	Dermatitis [0.98]	Congenital [0.68]	Neonatal [0.6]	URI [1.29]	Diarrhea [1.34]	Asthma [0.71]	Skin Viral [0.98]	Falls [0.97]	Endocrine [1.08]	Epilepsy [1.28]
Cyprus	Neonatal [1.12]	Dermatitis [0.85]	Congenital [0.5]	URI [1.27]	Asthma [0.73]	Skin Viral [0.96]	Diarrhea [1.01]	ASD [1.04]	Falls [0.67]	Road Inj [0.4]
Denmark	Dermatitis [1.22]	Congenital [0.88]	Neonatal [0.75]	URI [1.27]	Asthma [0.96]	Diarrhea [1.45]	Skin Viral [1.14]	Endocrine [1.31]	ASD [1.22]	Falls [0.63]
Finland	Neonatal [1.69]	Dermatitis [1.3]	Congenital [0.75]	URI [1.27]	Asthma [0.88]	Diarrhea [1.37]	Skin Viral [1.01]	ASD [1.05]	Falls [0.74]	Endocrine [0.91]
France	Dermatitis [1.21]	Congenital [0.72]	Neonatal [0.68]	URI [1.31]	Asthma [0.81]	Endocrine [1.26]	Skin Viral [0.96]	Drown [0.92]	Road Inj [0.61]	Falls [0.91]
Germany	Neonatal [1.0]	Dermatitis [0.97]	Congenital [0.76]	Diarrhea [1.47]	URI [1.27]	Epilepsy [1.85]	Skin Viral [0.98]	Asthma [0.59]	Endocrine [0.98]	Falls [0.79]
Greece	Congenital [0.73]	Neonatal [0.8]	Dermatitis [0.74]	Road Inj [0.67]	URI [1.3]	Asthma [0.63]	Skin Viral [0.92]	Brain C [1.13]	LRI [0.62]	Diarrhea [0.64]
Iceland	Dermatitis [1.35]	Congenital [1.03]	Neonatal [0.93]	Asthma [1.26]	URI [1.27]	Skin Viral [1.1]	Diarrhea [1.2]	Brain C [1.33]	LRI [1.94]	Falls [0.82]
Ireland	Dermatitis [1.15]	Congenital [0.72]	Neonatal [0.78]	Asthma [1.06]	URI [1.26]	Skin Viral [0.98]	ASD [1.11]	Diarrhea [0.74]	Endocrine [0.87]	Falls [0.71]
Israel	Dermatitis [1.05]	Neonatal [0.87]	Congenital [0.59]	URI [1.27]	Asthma [0.68]	Road Inj [0.57]	Blindness [2.53]	Endocrine [1.1]	Skin Viral [0.9]	Falls [0.74]
Italy	Dermatitis [0.98]	Congenital [0.56]	Neonatal [0.64]	URI [1.29]	Skin Viral [0.95]	Endocrine [0.89]	ASD [1.03]	Asthma [0.42]	Diarrhea [0.63]	Leukemia [0.67]
Luxembourg	Dermatitis [1.31]	Neonatal [1.17]	Congenital [0.65]	Asthma [1.05]	URI [1.28]	Diarrhea [1.3]	Skin Viral [1.02]	ASD [1.05]	Falls [0.73]	Epilepsy [1.2]
Malta	Congenital [0.81]	Dermatitis [0.88]	Neonatal [0.7]	URI [1.32]	Asthma [0.76]	Skin Viral [0.93]	Falls [0.94]	ASD [1.04]	LRI [0.62]	Brain C [0.71]
Netherlands	Dermatitis [1.22]	Neonatal [0.93]	Congenital [0.86]	URI [1.32]	Asthma [0.85]	Skin Viral [0.95]	Endocrine [1.22]	ASD [1.15]	Epilepsy [1.25]	Falls [0.71]
Norway	Dermatitis [1.31]	Neonatal [1.06]	Diarrhea [4.08]	Congenital [0.81]	Asthma [1.3]	URI [1.26]	Poison [10.21]	Skin Viral [1.09]	Falls [0.81]	ASD [0.98]
Portugal	Dermatitis [0.94]	Congenital [0.41]	Neonatal [0.56]	Asthma [0.94]	URI [1.27]	Skin Viral [0.86]	Fire [1.01]	Road Inj [0.34]	Endocrine [0.75]	ASD [1.03]
Spain	Neonatal [0.69]	Congenital [0.52]	Dermatitis [0.75]	URI [1.29]	Asthma [0.6]	Skin Viral [0.92]	Endocrine [0.9]	ASD [1.04]	Falls [0.74]	Diarrhea [0.53]
Sweden	Dermatitis [1.63]	Congenital [0.78]	Neonatal [0.83]	Asthma [1.09]	URI [1.27]	Diarrhea [1.29]	Skin Viral [1.07]	ASD [1.13]	Falls [0.74]	Urticaria [0.77]
Stockholm	Dermatitis [1.34]	Congenital [0.98]	Neonatal [0.83]	Asthma [1.01]	URI [1.26]	Diarrhea [1.41]	Skin Viral [1.11]	ASD [1.43]	Endocrine [1.24]	Vit A [2.98]
Sweden w/o Stockholm	Dermatitis [1.73]	Neonatal [0.82]	Congenital [0.72]	Asthma [1.11]	URI [1.27]	Diarrhea [1.24]	Skin Viral [1.06]	ASD [1.04]	Falls [0.75]	Urticaria [0.75]
Switzerland	Dermatitis [1.3]	Congenital [0.83]	Neonatal [0.84]	URI [1.28]	Asthma [0.85]	Diarrhea [1.29]	Skin Viral [0.99]	ASD [1.04]	Endocrine [0.91]	Falls [0.7]
UK	Dermatitis [1.43]	Neonatal [0.99]	Congenital [0.71]	Asthma [1.19]	URI [1.3]	Diarrhea [0.9]	Skin Viral [0.88]	Endocrine [0.98]	ASD [1.11]	LRI [0.82]
England	Dermatitis [1.46]	Neonatal [1.03]	Congenital [0.73]	Asthma [1.23]	URI [1.3]	Diarrhea [0.93]	Skin Viral [0.87]	Endocrine [1.02]	LRI [0.92]	ASD [1.12]
E Midlands	Dermatitis [1.44]	Neonatal [0.95]	Congenital [0.64]	Asthma [1.12]	URI [1.31]	Diarrhea [0.86]	Skin Viral [0.85]	Endocrine [0.93]	ASD [1.1]	LRI [0.63]
Derby	Dermatitis [1.43]	Congenital [0.83]	Neonatal [1.01]	Asthma [1.18]	URI [1.33]	LRI [1.24]	Endocrine [1.18]	Skin Viral [0.86]	Diarrhea [0.8]	Epilepsy [1.05]
Derbyshire	Dermatitis [1.44]	Neonatal [0.87]	Congenital [0.59]	Asthma [1.07]	URI [1.3]	Diarrhea [0.83]	Skin Viral [0.84]	Endocrine [0.84]	ASD [1.1]	Falls [0.7]
Leicester	Dermatitis [1.44]	Congenital [0.73]	Neonatal [0.86]	Asthma [1.15]	URI [1.28]	Diarrhea [0.9]	Skin Viral [0.86]	Endocrine [1.06]	LRI [0.91]	ASD [1.1]
Leicestershire	Dermatitis [1.44]	Congenital [0.68]	Neonatal [0.83]	Asthma [1.15]	URI [1.27]	Skin Viral [0.86]	Diarrhea [0.83]	Iron [6.52]	Endocrine [0.97]	ASD [1.1]
Lincolnshire	Dermatitis [1.44]	Neonatal [0.91]	Congenital [0.55]	Asthma [1.06]	URI [1.32]	Diarrhea [0.8]	Skin Viral [0.84]	Endocrine [0.84]	ASD [1.1]	Epilepsy [0.73]

eFigure 7c. Leading ten causes of DALYs with the ratio of observed DALYs to DALYs expected on the basis of Socio-Demographic Index alone in 2017, 1-4 years, both sexes combined. The top ten causes contributing to DALYs are listed globally, by socio-demographic quintile, and then by GBD superregion, region, country, and subnationally where modeled. For each cell, the ratio of observed DALYs to DALYs expected on the basis of socio-demographic index (SDI) alone are listed. Abbreviations: DALY=disability-adjusted life year, GBD=Global Burden of Disease.

Values shown in brackets represent the ratio of observed DALYs to predicted DALYs on the basis of Socio-Demographic Index (SDI), rounded to two (2) digits. Color ranges (shown below) were calculated to place a roughly equal number of cells into each bin.

COLOR KEY:	[0.0-0.5]	(0.5-0.72]	(0.72-0.86]	(0.86-0.97]	(0.97-1.1]	(1.1-1.23]	(1.23-1.41]	(1.41-2.15]	2.15+

	1	2	3	4	5	6	7	8	9	10
Northamptonshire	Dermatitis [1.44]	Neonatal [1.03]	Congenital [0.63]	Asthma [1.12]	URI [1.31]	Diarrhea [0.96]	Skin Viral [0.85]	Endocrine [0.93]	ASD [1.1]	LRI [0.61]
Nottingham	Dermatitis [1.43]	Neonatal [1.2]	Congenital [0.86]	Asthma [1.24]	URI [1.39]	Endocrine [1.21]	Skin Viral [0.88]	LRI [1.31]	Diarrhea [0.9]	ASD [1.11]
Nottinghamshire	Dermatitis [1.44]	Neonatal [0.99]	Congenital [0.54]	Asthma [1.08]	URI [1.31]	Diarrhea [0.8]	Skin Viral [0.84]	ASD [1.1]	Endocrine [0.77]	Falls [0.65]
Rutland	Dermatitis [1.44]	Congenital [0.66]	Neonatal [0.69]	Asthma [1.13]	URI [1.27]	Skin Viral [0.86]	Diarrhea [0.78]	Endocrine [0.93]	ASD [1.09]	Road Inj [0.41]
E England	Dermatitis [1.44]	Neonatal [0.91]	Congenital [0.63]	Asthma [1.14]	URI [1.3]	Skin Viral [0.86]	Diarrhea [0.84]	Endocrine [0.94]	ASD [1.17]	LRI [0.68]
Bedford	Dermatitis [1.44]	Neonatal [1.14]	Congenital [0.8]	Asthma [1.15]	URI [1.29]	Endocrine [1.25]	LRI [0.96]	Skin Viral [0.86]	Diarrhea [0.84]	Other MN [1.48]
Cambridgeshire	Dermatitis [1.43]	Neonatal [0.96]	Congenital [0.68]	Asthma [1.22]	URI [1.28]	ASD [1.67]	Skin Viral [0.89]	Diarrhea [0.96]	Endocrine [0.98]	Other MN [1.23]
Cen Bedfordshire	Dermatitis [1.44]	Neonatal [1.0]	Congenital [0.73]	Asthma [1.15]	URI [1.36]	Diarrhea [0.89]	LRI [0.92]	Endocrine [1.09]	Skin Viral [0.86]	Other MN [1.34]
Essex	Dermatitis [1.44]	Neonatal [0.89]	Congenital [0.56]	Asthma [1.1]	URI [1.29]	Skin Viral [0.85]	Diarrhea [0.73]	ASD [1.11]	Endocrine [0.84]	Epilepsy [0.82]
Hertfordshire	Dermatitis [1.43]	Neonatal [0.93]	Congenital [0.65]	Asthma [1.22]	URI [1.28]	Skin Viral [0.89]	Diarrhea [0.89]	ASD [1.12]	Endocrine [0.85]	Falls [0.66]
Luton	Dermatitis [1.44]	Congenital [0.93]	Neonatal [1.08]	Asthma [1.19]	LRI [1.32]	URI [1.31]	Endocrine [1.52]	Diarrhea [1.01]	Epilepsy [1.29]	Skin Viral [0.85]
Norfolk	Dermatitis [1.44]	Neonatal [0.76]	Congenital [0.53]	Asthma [1.09]	URI [1.29]	Diarrhea [0.86]	Skin Viral [0.85]	ASD [1.11]	Iron [4.43]	Endocrine [0.79]
Peterborough	Dermatitis [1.44]	Congenital [0.77]	Neonatal [0.8]	Asthma [1.13]	URI [1.36]	Endocrine [1.43]	LRI [0.96]	Diarrhea [0.88]	Skin Viral [0.84]	Other MN [1.36]
Southend-on-Sea	Dermatitis [1.44]	Neonatal [1.05]	Congenital [0.7]	Asthma [1.07]	URI [1.3]	LRI [0.75]	Endocrine [1.08]	Skin Viral [0.84]	Epilepsy [0.99]	Diarrhea [0.66]
Suffolk	Dermatitis [1.44]	Neonatal [0.91]	Congenital [0.5]	Asthma [1.07]	URI [1.28]	Diarrhea [0.81]	Skin Viral [0.84]	ASD [1.11]	Endocrine [0.77]	Falls [0.63]
Thurrock	Dermatitis [1.45]	Neonatal [0.99]	Congenital [0.6]	Asthma [1.08]	URI [1.32]	Endocrine [1.07]	Skin Viral [0.84]	LRI [0.56]	Diarrhea [0.64]	ASD [1.09]
Greater London	Dermatitis [1.44]	Neonatal [1.13]	Congenital [0.9]	Asthma [1.52]	URI [1.28]	Skin Viral [0.91]	LRI [1.94]	Diarrhea [1.0]	Endocrine [1.16]	ASD [1.12]
Barking & Dagenham	Dermatitis [1.81]	Neonatal [0.96]	Congenital [0.56]	Asthma [1.21]	URI [1.28]	LRI [0.57]	Iron [4.13]	Skin Viral [0.83]	Diarrhea [0.7]	Endocrine [0.86]
Barnet	Dermatitis [1.43]	Neonatal [1.02]	Congenital [0.7]	Asthma [1.38]	URI [1.27]	Skin Viral [0.88]	Diarrhea [0.84]	LRI [1.1]	ASD [1.11]	Endocrine [0.92]
Bexley	Dermatitis [1.44]	Neonatal [0.95]	Congenital [0.63]	Asthma [1.28]	URI [1.32]	Skin Viral [0.85]	Diarrhea [0.79]	Endocrine [1.0]	ASD [1.18]	Other MN [1.25]
Brent	Dermatitis [1.43]	Neonatal [1.08]	Congenital [0.75]	Asthma [1.36]	URI [1.26]	Skin Viral [0.87]	Diarrhea [0.85]	LRI [1.01]	Endocrine [1.02]	ASD [1.1]
Bromley	Dermatitis [1.43]	Neonatal [1.09]	Congenital [0.66]	Asthma [1.34]	URI [1.28]	Skin Viral [0.87]	ASD [1.18]	Diarrhea [0.72]	Endocrine [0.83]	Other MN [1.22]
Camden	Dermatitis [1.44]	Neonatal [1.06]	Congenital [1.02]	Asthma [1.66]	URI [1.37]	Skin Viral [0.95]	LRI [3.2]	Other MN [2.17]	Endocrine [1.27]	Diarrhea [1.07]
Croydon	Dermatitis [1.44]	Neonatal [0.99]	Congenital [0.58]	Asthma [1.3]	URI [1.28]	Skin Viral [0.86]	Diarrhea [0.75]	ASD [1.1]	Endocrine [0.79]	Epilepsy [0.83]
Ealing	Dermatitis [1.43]	Neonatal [1.17]	Congenital [0.75]	Asthma [1.41]	URI [1.26]	LRI [1.45]	Diarrhea [0.98]	Endocrine [1.17]	Skin Viral [0.88]	ASD [1.1]
Enfield	Dermatitis [1.44]	Neonatal [1.04]	Congenital [0.62]	Asthma [1.32]	URI [1.31]	Skin Viral [0.86]	Diarrhea [0.85]	ASD [1.11]	Endocrine [0.88]	LRI [0.75]
Greenwich	Dermatitis [1.44]	Neonatal [0.99]	Congenital [0.69]	Asthma [1.32]	URI [1.31]	Skin Viral [0.86]	Diarrhea [0.82]	LRI [0.82]	ASD [1.17]	Endocrine [0.85]
Hackney	Dermatitis [1.43]	Neonatal [1.12]	Congenital [0.84]	Asthma [1.52]	URI [1.3]	Endocrine [1.26]	LRI [1.79]	Skin Viral [0.9]	Diarrhea [0.98]	ASD [1.09]
Hammersmith & Fulham	Dermatitis [1.44]	Neonatal [1.15]	Congenital [0.98]	Asthma [1.63]	URI [1.26]	Skin Viral [0.95]	Diarrhea [1.15]	Endocrine [1.26]	LRI [2.59]	ASD [1.11]
Haringey	Dermatitis [1.43]	Neonatal [1.14]	Congenital [0.79]	Asthma [1.37]	URI [1.31]	LRI [1.32]	Endocrine [1.21]	Skin Viral [0.87]	Diarrhea [0.85]	Other MN [1.4]
Harrow	Dermatitis [1.43]	Neonatal [1.15]	Congenital [0.72]	Asthma [1.35]	URI [1.3]	Endocrine [1.1]	Skin Viral [0.87]	Diarrhea [0.86]	Other MN [1.47]	ASD [1.1]

eFigure 7c. Leading ten causes of DALYs with the ratio of observed DALYs to DALYs expected on the basis of Socio-Demographic Index alone in 2017, 1-4 years, both sexes combined. The top ten causes contributing to DALYs are listed globally, by socio-demographic quintile, and then by GBD superregion, region, country, and subnationally where modeled. For each cell, the ratio of observed DALYs to DALYs expected on the basis of socio-demographic index (SDI) alone are listed. Abbreviations: DALY=disability-adjusted life year, GBD=Global Burden of Disease.

Values shown in brackets represent the ratio of observed DALYs to predicted DALYs on the basis of Socio-Demographic Index (SDI), rounded to two (2) digits. Color ranges (shown below) were calculated to place a roughly equal number of cells into each bin.

COLOR KEY:	[0.0-0.5]	(0.5-0.72]	(0.72-0.86]	(0.86-0.97]	(0.97-1.1]	(1.1-1.23]	(1.23-1.41]	(1.41-2.15]	2.15+

	1	2	3	4	5	6	7	8	9	10
Havering	Dermatitis [1.44]	Neonatal [0.96]	Congenital [0.61]	Asthma [1.27]	URI [1.32]	Skin Viral [0.85]	Diarrhea [0.75]	LRI [0.68]	Endocrine [0.88]	ASD [1.11]
Hillingdon	Dermatitis [1.43]	Neonatal [1.22]	Congenital [0.92]	Asthma [1.48]	URI [1.28]	LRI [1.81]	Diarrhea [1.1]	Skin Viral [0.89]	Endocrine [1.15]	Other MN [1.75]
Hounslow	Dermatitis [1.43]	Neonatal [1.16]	Congenital [0.88]	Asthma [1.48]	URI [1.29]	LRI [1.73]	Endocrine [1.25]	Skin Viral [0.89]	Diarrhea [0.97]	ASD [1.11]
Islington	Dermatitis [1.44]	Congenital [1.14]	Neonatal [1.05]	Asthma [1.64]	URI [1.31]	LRI [3.72]	Endocrine [1.43]	Skin Viral [0.94]	Diarrhea [1.11]	Other MN [2.12]
Kensington & Chelsea	Dermatitis [1.44]	Neonatal [1.02]	Congenital [0.95]	Asthma [1.65]	URI [1.29]	Skin Viral [0.95]	Other MN [2.36]	Endocrine [1.28]	ASD [1.14]	Diarrhea [1.0]
Kingston upon Thames	Dermatitis [1.43]	Neonatal [1.18]	Congenital [0.95]	Asthma [1.51]	URI [1.26]	LRI [2.32]	Endocrine [1.28]	Skin Viral [0.9]	Other MN [1.81]	Diarrhea [0.94]
Lambeth	Dermatitis [1.43]	Congenital [0.95]	Neonatal [0.88]	Asthma [1.54]	URI [1.27]	LRI [2.35]	Skin Viral [0.92]	Endocrine [1.16]	Diarrhea [0.94]	ASD [1.11]
Lewisham	Dermatitis [1.43]	Neonatal [1.19]	Congenital [0.62]	Asthma [1.32]	URI [1.29]	Skin Viral [0.86]	LRI [0.95]	ASD [1.17]	Diarrhea [0.75]	Endocrine [0.87]
Merton	Dermatitis [1.43]	Neonatal [1.1]	Congenital [0.89]	Asthma [1.46]	URI [1.27]	Skin Viral [0.89]	Endocrine [1.17]	LRI [1.5]	Diarrhea [0.85]	Other MN [1.55]
Newham	Dermatitis [1.43]	Neonatal [1.12]	Congenital [0.8]	Asthma [1.36]	URI [1.28]	LRI [1.25]	Endocrine [1.43]	Diarrhea [0.93]	Skin Viral [0.86]	Other MN [1.53]
Redbridge	Dermatitis [1.44]	Neonatal [0.99]	Congenital [0.63]	Asthma [1.28]	URI [1.28]	Skin Viral [0.85]	Diarrhea [0.81]	LRI [0.75]	Endocrine [0.88]	ASD [1.11]
Richmond upon Thames	Dermatitis [1.43]	Neonatal [1.24]	Congenital [1.03]	Asthma [1.53]	URI [1.34]	Other MN [2.11]	LRI [2.18]	Skin Viral [0.91]	Endocrine [1.25]	ASD [1.12]
Southwark	Dermatitis [1.43]	Congenital [0.94]	Neonatal [0.89]	Asthma [1.57]	URI [1.27]	Skin Viral [0.93]	LRI [2.49]	Endocrine [1.22]	Diarrhea [0.99]	ASD [1.17]
Sutton	Dermatitis [1.43]	Neonatal [1.24]	Congenital [0.78]	Asthma [1.35]	URI [1.25]	Skin Viral [0.86]	LRI [0.97]	Endocrine [1.01]	Diarrhea [0.81]	ASD [1.11]
Tower Hamlets	Dermatitis [1.43]	Neonatal [1.23]	Congenital [0.95]	Asthma [1.58]	URI [1.28]	Skin Viral [0.92]	Endocrine [1.28]	Diarrhea [1.06]	LRI [2.19]	ASD [1.12]
Waltham Forest	Dermatitis [1.44]	Neonatal [0.99]	Congenital [0.59]	Asthma [1.25]	URI [1.28]	Diarrhea [0.79]	LRI [0.71]	Skin Viral [0.84]	Endocrine [0.88]	ASD [1.11]
Wandsworth	Dermatitis [1.43]	Neonatal [1.0]	Congenital [0.81]	Asthma [1.56]	URI [1.27]	Skin Viral [0.93]	ASD [1.12]	Diarrhea [0.89]	LRI [1.89]	Endocrine [0.99]
Westminster	Dermatitis [1.44]	Congenital [1.08]	Neonatal [1.0]	Asthma [1.65]	URI [1.28]	Other MN [2.45]	Endocrine [1.46]	LRI [3.21]	Skin Viral [0.94]	Diarrhea [1.06]
NE England	Dermatitis [1.54]	Congenital [0.68]	Neonatal [0.9]	Asthma [1.1]	URI [1.3]	Diarrhea [1.02]	Skin Viral [0.84]	Endocrine [0.91]	LRI [0.63]	ASD [1.1]
County Durham	Dermatitis [1.68]	Neonatal [0.92]	Congenital [0.58]	Asthma [1.09]	URI [1.29]	Diarrhea [0.98]	Skin Viral [0.84]	Endocrine [0.82]	ASD [1.1]	Falls [0.66]
Darlington	Dermatitis [1.44]	Congenital [0.77]	Neonatal [0.89]	Asthma [1.13]	URI [1.34]	Diarrhea [1.0]	Endocrine [1.05]	Skin Viral [0.85]	LRI [0.74]	Epilepsy [0.97]
Gateshead	Dermatitis [1.66]	Neonatal [0.98]	Congenital [0.62]	Asthma [1.11]	URI [1.28]	Diarrhea [0.98]	Skin Viral [0.85]	ASD [1.1]	Endocrine [0.8]	LRI [0.56]
Hartlepool	Dermatitis [1.45]	Congenital [0.71]	Neonatal [1.01]	Asthma [1.09]	URI [1.26]	LRI [0.67]	Diarrhea [0.83]	Skin Viral [0.83]	Endocrine [0.93]	Epilepsy [0.87]
Middlesbrough	Dermatitis [1.45]	Congenital [0.72]	Neonatal [0.82]	Asthma [1.11]	URI [1.28]	Diarrhea [1.06]	LRI [0.75]	Skin Viral [0.84]	Endocrine [0.92]	ASD [1.08]
Newcastle upon Tyne	Dermatitis [1.43]	Congenital [1.0]	Neonatal [0.95]	Asthma [1.27]	URI [1.32]	Diarrhea [1.27]	Endocrine [1.31]	LRI [1.57]	Skin Viral [0.88]	Epilepsy [1.25]
N Tyneside	Dermatitis [1.44]	Congenital [0.68]	Neonatal [0.85]	Asthma [1.11]	Diarrhea [1.14]	URI [1.26]	Skin Viral [0.84]	LRI [0.64]	ASD [1.12]	Endocrine [0.84]
Northumberland	Dermatitis [1.44]	Neonatal [0.83]	Congenital [0.59]	Asthma [1.08]	URI [1.34]	Diarrhea [0.92]	Skin Viral [0.84]	ASD [1.11]	Endocrine [0.79]	Falls [0.67]
Redcar & Cleveland	Dermatitis [1.45]	Congenital [0.55]	Neonatal [0.79]	Asthma [1.04]	URI [1.29]	Diarrhea [0.89]	Skin Viral [0.83]	ASD [1.11]	LRI [0.38]	Endocrine [0.71]
S Tyneside	Dermatitis [1.68]	Congenital [0.61]	Neonatal [0.83]	Asthma [1.05]	URI [1.3]	Diarrhea [0.87]	Skin Viral [0.83]	LRI [0.46]	Endocrine [0.79]	ASD [1.1]
Stockton-on-Tees	Dermatitis [1.44]	Neonatal [1.07]	Congenital [0.72]	Asthma [1.13]	URI [1.33]	Diarrhea [0.99]	Skin Viral [0.85]	LRI [0.75]	Endocrine [0.94]	Other MN [1.2]
Sunderland	Dermatitis [1.67]	Congenital [0.69]	Neonatal [0.8]	Asthma [0.99]	URI [1.28]	Diarrhea [1.03]	LRI [0.72]	Endocrine [1.01]	Skin Viral [0.84]	ASD [1.1]

eFigure 7c. Leading ten causes of DALYs with the ratio of observed DALYs to DALYs expected on the basis of Socio-Demographic Index alone in 2017, 1-4 years, both sexes combined. The top ten causes contributing to DALYs are listed globally, by socio-demographic quintile, and then by GBD superregion, region, country, and subnationally where modeled. For each cell, the ratio of observed DALYs to DALYs expected on the basis of socio-demographic index (SDI) alone are listed. Abbreviations: DALY=disability-adjusted life year, GBD=Global Burden of Disease.

Values shown in brackets represent the ratio of observed DALYs to predicted DALYs on the basis of Socio-Demographic Index (SDI), rounded to two (2) digits. Color ranges (shown below) were calculated to place a roughly equal number of cells into each bin.

COLOR KEY:	[0.0-0.5]	(0.5-0.72]	(0.72-0.86]	(0.86-0.97]	(0.97-1.1]	(1.1-1.23]	(1.23-1.41]	(1.41-2.15]	2.15+

	1	2	3	4	5	6	7	8	9	10
NW England	Dermatitis [1.44]	Neonatal [1.11]	Congenital [0.79]	Asthma [1.16]	URI [1.3]	Diarrhea [0.99]	LRI [0.94]	Endocrine [1.1]	Skin Viral [0.85]	ASD [1.1]
Blackburn with Darwen	Congenital [0.91]	Dermatitis [1.45]	Neonatal [1.13]	Asthma [1.15]	LRI [1.04]	Endocrine [1.57]	URI [1.34]	Diarrhea [1.02]	Other MN [1.48]	Iron [4.45]
Blackpool	Dermatitis [1.47]	Congenital [0.73]	Neonatal [1.04]	Asthma [1.08]	LRI [0.68]	URI [1.29]	Endocrine [1.17]	Diarrhea [0.7]	Skin Viral [0.83]	Falls [0.92]
Bolton	Dermatitis [1.45]	Neonatal [1.03]	Congenital [0.67]	Asthma [1.08]	URI [1.28]	Diarrhea [0.86]	Endocrine [1.12]	LRI [0.65]	Skin Viral [0.84]	Iron [4.15]
Bury	Dermatitis [1.44]	Congenital [0.76]	Neonatal [1.01]	Asthma [1.09]	URI [1.31]	LRI [0.81]	Diarrhea [0.93]	Iron [5.53]	Endocrine [1.05]	Skin Viral [0.84]
Cheshire E	Neonatal [1.37]	Dermatitis [1.43]	Congenital [0.9]	Asthma [1.23]	URI [1.29]	Diarrhea [1.06]	Skin Viral [0.88]	LRI [1.2]	Endocrine [1.03]	ASD [1.11]
Cheshire W & Chester	Dermatitis [1.43]	Neonatal [1.14]	Congenital [0.88]	Asthma [1.2]	URI [1.28]	Diarrhea [1.05]	Skin Viral [0.87]	LRI [1.1]	Endocrine [1.06]	ASD [1.11]
Cumbria	Dermatitis [1.44]	Neonatal [1.07]	Congenital [0.7]	Asthma [1.13]	URI [1.28]	Diarrhea [1.02]	Skin Viral [0.85]	Iron [5.68]	Endocrine [0.89]	ASD [1.1]
Halton	Dermatitis [1.44]	Congenital [0.88]	Neonatal [1.16]	Asthma [1.15]	LRI [1.24]	URI [1.37]	Endocrine [1.28]	Diarrhea [0.99]	Meningitis [1.34]	Skin Viral [0.85]
Knowsley	Dermatitis [1.44]	Congenital [0.88]	Neonatal [1.09]	Asthma [1.12]	LRI [1.04]	URI [1.35]	Diarrhea [0.9]	Endocrine [1.16]	Skin Viral [0.84]	Epilepsy [1.1]
Lancashire	Dermatitis [1.44]	Neonatal [1.08]	Congenital [0.75]	Asthma [1.14]	URI [1.31]	Diarrhea [1.05]	LRI [0.83]	Endocrine [1.05]	Skin Viral [0.85]	ASD [1.1]
Liverpool	Dermatitis [1.43]	Neonatal [1.21]	Congenital [0.82]	Asthma [1.19]	URI [1.29]	LRI [1.25]	Diarrhea [0.92]	Skin Viral [0.87]	Endocrine [1.08]	ASD [1.1]
Manchester	Dermatitis [1.43]	Neonatal [1.28]	Congenital [0.94]	Asthma [1.32]	URI [1.28]	Diarrhea [1.24]	Endocrine [1.34]	Skin Viral [0.9]	LRI [1.74]	ASD [1.1]
Oldham	Dermatitis [1.46]	Congenital [0.77]	Neonatal [0.85]	Asthma [1.09]	URI [1.33]	LRI [0.62]	Endocrine [1.21]	Diarrhea [0.83]	Skin Viral [0.83]	Epilepsy [0.97]
Rochdale	Dermatitis [1.45]	Congenital [0.67]	Neonatal [0.86]	Asthma [1.09]	URI [1.3]	Iron [5.08]	LRI [0.62]	Diarrhea [0.86]	Endocrine [1.12]	Skin Viral [0.83]
Salford	Dermatitis [1.43]	Neonatal [1.23]	Congenital [0.83]	Asthma [1.18]	URI [1.28]	LRI [1.07]	Endocrine [1.17]	Diarrhea [0.92]	Skin Viral [0.86]	ASD [1.1]
Sefton	Dermatitis [1.44]	Neonatal [1.06]	Congenital [0.67]	Asthma [1.08]	URI [1.31]	Diarrhea [0.8]	Skin Viral [0.84]	Endocrine [0.96]	LRI [0.57]	ASD [1.11]
St Helens	Dermatitis [1.45]	Neonatal [1.08]	Congenital [0.7]	Asthma [1.07]	URI [1.29]	LRI [0.7]	Diarrhea [0.84]	Endocrine [1.04]	Skin Viral [0.84]	ASD [1.1]
Stockport	Dermatitis [1.43]	Neonatal [0.95]	Congenital [0.72]	Asthma [1.16]	URI [1.29]	Diarrhea [1.11]	Skin Viral [0.86]	Endocrine [1.0]	Iron [6.08]	ASD [1.11]
Tameside	Dermatitis [1.45]	Congenital [0.63]	Neonatal [0.89]	Asthma [1.07]	URI [1.27]	Diarrhea [0.88]	Skin Viral [0.83]	LRI [0.48]	Endocrine [0.89]	ASD [1.1]
Trafford	Dermatitis [1.43]	Neonatal [1.26]	Congenital [0.86]	Asthma [1.27]	URI [1.27]	Diarrhea [1.03]	Skin Viral [0.89]	Endocrine [1.12]	ASD [1.1]	Other MN [1.46]
Warrington	Dermatitis [1.43]	Neonatal [1.31]	Congenital [0.86]	Asthma [1.23]	URI [1.27]	LRI [1.51]	Diarrhea [1.04]	Skin Viral [0.88]	Endocrine [0.96]	ASD [1.1]
Wigan	Dermatitis [1.45]	Neonatal [1.05]	Congenital [0.56]	Asthma [1.05]	URI [1.32]	Diarrhea [0.76]	Iron [4.1]	Skin Viral [0.83]	LRI [0.45]	Endocrine [0.8]
Wirral	Dermatitis [1.44]	Neonatal [1.16]	Congenital [0.73]	Asthma [1.07]	URI [1.33]	LRI [0.68]	Diarrhea [0.86]	Endocrine [1.07]	Skin Viral [0.83]	Epilepsy [0.91]
SE England	Dermatitis [1.54]	Neonatal [1.02]	Congenital [0.67]	Asthma [1.26]	URI [1.3]	Skin Viral [0.87]	Diarrhea [0.89]	ASD [1.19]	Endocrine [0.89]	Epilepsy [0.92]
Bracknell Forest	Dermatitis [1.43]	Congenital [0.8]	Neonatal [0.86]	Asthma [1.21]	URI [1.31]	Skin Viral [0.89]	LRI [1.3]	Iron [8.48]	Diarrhea [0.87]	Endocrine [0.99]
Brighton & Hove	Dermatitis [1.65]	Neonatal [1.16]	Congenital [0.84]	Asthma [1.27]	URI [1.28]	Skin Viral [0.9]	Endocrine [1.1]	Diarrhea [0.87]	ASD [1.1]	LRI [1.36]
Buckinghamshire	Dermatitis [1.43]	Neonatal [1.19]	Congenital [0.73]	Asthma [1.19]	URI [1.33]	Epilepsy [1.72]	Diarrhea [0.98]	Skin Viral [0.88]	Endocrine [0.96]	ASD [1.11]
E Sussex	Dermatitis [1.64]	Neonatal [1.03]	Congenital [0.63]	Asthma [1.03]	URI [1.3]	Diarrhea [0.8]	Skin Viral [0.84]	ASD [1.18]	Endocrine [0.83]	Iron [4.08]
Hampshire	Dermatitis [1.43]	Neonatal [0.98]	Congenital [0.59]	Asthma [1.68]	URI [1.3]	Skin Viral [0.87]	Diarrhea [0.89]	Iron [6.7]	ASD [1.11]	Endocrine [0.85]
Isle of Wight	Dermatitis [1.42]	Neonatal [0.92]	Congenital [0.66]	Asthma [1.58]	URI [1.3]	Skin Viral [0.84]	Diarrhea [0.76]	LRI [0.59]	Endocrine [0.87]	ASD [1.1]

eFigure 7c. Leading ten causes of DALYs with the ratio of observed DALYs to DALYs expected on the basis of Socio-Demographic Index alone in 2017, 1-4 years, both sexes combined. The top ten causes contributing to DALYs are listed globally, by socio-demographic quintile, and then by GBD superregion, region, country, and subnationally where modeled. For each cell, the ratio of observed DALYs to DALYs expected on the basis of socio-demographic index (SDI) alone are listed. Abbreviations: DALY=disability-adjusted life year, GBD=Global Burden of Disease.

Values shown in brackets represent the ratio of observed DALYs to predicted DALYs on the basis of Socio-Demographic Index (SDI), rounded to two (2) digits. Color ranges (shown below) were calculated to place a roughly equal number of cells into each bin.

COLOR KEY:	[0.0-0.5]	(0.5-0.72]	(0.72-0.86]	(0.86-0.97]	(0.97-1.1]	(1.1-1.23]	(1.23-1.41]	(1.41-2.15]	2.15+

	1	2	3	4	5	6	7	8	9	10
Kent	Dermatitis [1.63]	Neonatal [0.95]	Congenital [0.54]	Asthma [1.09]	URI [1.28]	ASD [1.55]	Skin Viral [0.85]	Diarrhea [0.79]	Endocrine [0.77]	Falls [0.65]
Medway	Dermatitis [1.44]	Neonatal [1.0]	Congenital [0.55]	Asthma [1.05]	URI [1.32]	Diarrhea [0.8]	Skin Viral [0.84]	ASD [1.1]	Endocrine [0.75]	Epilepsy [0.8]
Milton Keynes	Dermatitis [1.43]	Neonatal [0.93]	Congenital [0.74]	Asthma [1.21]	URI [1.28]	Diarrhea [1.01]	LRI [1.27]	Skin Viral [0.88]	Endocrine [0.96]	ASD [1.11]
Oxfordshire	Dermatitis [1.79]	Neonatal [0.98]	Congenital [0.74]	Asthma [1.24]	URI [1.28]	Skin Viral [0.9]	Diarrhea [0.91]	Endocrine [1.0]	ASD [1.11]	Other MN [1.27]
Portsmouth	Dermatitis [1.43]	Neonatal [1.01]	Congenital [0.66]	Asthma [1.18]	URI [1.27]	Diarrhea [0.96]	Skin Viral [0.88]	Endocrine [0.95]	ASD [1.1]	Epilepsy [0.99]
Reading	Dermatitis [1.79]	Neonatal [1.3]	Congenital [1.05]	Asthma [1.33]	URI [1.3]	LRI [2.61]	Endocrine [1.44]	Diarrhea [1.06]	Skin Viral [0.91]	Epilepsy [1.37]
Slough	Dermatitis [1.43]	Congenital [0.97]	Neonatal [1.13]	Asthma [1.28]	LRI [1.91]	URI [1.28]	Endocrine [1.27]	Diarrhea [1.04]	Skin Viral [0.88]	Epilepsy [1.16]
Southampton	Dermatitis [1.43]	Neonatal [1.01]	Congenital [0.82]	Asthma [1.2]	URI [1.3]	Diarrhea [0.95]	Skin Viral [0.87]	Endocrine [1.12]	LRI [1.15]	Other MN [1.46]
Surrey	Dermatitis [1.61]	Neonatal [1.12]	Congenital [0.66]	Asthma [1.24]	URI [1.29]	Skin Viral [0.9]	Diarrhea [0.88]	ASD [1.12]	Iron [7.35]	Falls [0.64]
West Berkshire	Dermatitis [1.43]	Neonatal [0.98]	Congenital [0.79]	Asthma [1.23]	URI [1.29]	Skin Viral [0.88]	Diarrhea [0.92]	ASD [1.12]	Endocrine [0.95]	Other MN [1.46]
W Sussex	Dermatitis [1.48]	Neonatal [1.0]	Congenital [0.62]	Asthma [1.28]	URI [1.3]	Skin Viral [0.86]	Diarrhea [0.84]	ASD [1.11]	Endocrine [0.8]	Falls [0.64]
Windsor & Maidenhead	Dermatitis [1.43]	Congenital [1.02]	Neonatal [1.06]	Asthma [1.3]	URI [1.33]	LRI [2.27]	Diarrhea [1.06]	Endocrine [1.24]	Skin Viral [0.9]	Other MN [1.9]
Wokingham	Dermatitis [1.43]	Neonatal [1.05]	Congenital [0.87]	Asthma [1.26]	URI [1.3]	Skin Viral [0.9]	Diarrhea [0.99]	LRI [1.53]	Other MN [1.66]	Endocrine [1.04]
SW England	Dermatitis [1.44]	Neonatal [1.04]	Congenital [0.62]	Asthma [1.13]	URI [1.3]	Skin Viral [0.86]	Diarrhea [0.85]	ASD [1.15]	Iron [5.66]	Endocrine [0.88]
Bath & NE Somerset	Neonatal [1.4]	Dermatitis [1.43]	Congenital [0.79]	Asthma [1.23]	URI [1.27]	Diarrhea [1.03]	Skin Viral [0.89]	ASD [1.32]	Other MN [1.56]	Endocrine [0.92]
Bournemouth	Dermatitis [1.43]	Neonatal [1.06]	Congenital [0.69]	Asthma [1.18]	URI [1.26]	Skin Viral [0.88]	Diarrhea [0.92]	Endocrine [1.07]	Iron [7.49]	ASD [1.1]
Bristol	Dermatitis [1.43]	Neonatal [1.25]	Congenital [0.75]	Asthma [1.27]	URI [1.28]	Skin Viral [0.9]	ASD [1.31]	Diarrhea [0.92]	Endocrine [0.98]	Epilepsy [1.02]
Cornwall	Dermatitis [1.44]	Neonatal [1.0]	Congenital [0.5]	Asthma [1.05]	URI [1.29]	Diarrhea [0.81]	Iron [5.03]	Skin Viral [0.84]	ASD [1.11]	Endocrine [0.79]
Devon	Dermatitis [1.44]	Neonatal [0.94]	Congenital [0.59]	Asthma [1.1]	URI [1.3]	Iron [7.6]	Diarrhea [0.86]	Skin Viral [0.85]	Endocrine [0.89]	ASD [1.11]
Dorset	Dermatitis [1.44]	Neonatal [1.0]	Congenital [0.61]	Asthma [1.09]	URI [1.3]	Diarrhea [0.8]	Skin Viral [0.85]	Endocrine [0.93]	ASD [1.1]	Other MN [1.13]
Gloucestershire	Dermatitis [1.43]	Neonatal [0.97]	Congenital [0.59]	Asthma [1.14]	URI [1.28]	Iron [9.51]	Diarrhea [0.94]	Skin Viral [0.87]	ASD [1.1]	Endocrine [0.83]
N Somerset	Dermatitis [1.44]	Neonatal [1.11]	Congenital [0.62]	Asthma [1.1]	URI [1.29]	Skin Viral [0.85]	ASD [1.32]	Diarrhea [0.73]	Endocrine [0.72]	Epilepsy [0.81]
Plymouth	Dermatitis [1.44]	Neonatal [1.03]	Congenital [0.67]	Asthma [1.11]	URI [1.26]	Diarrhea [0.9]	Skin Viral [0.85]	Endocrine [0.96]	ASD [1.1]	LRI [0.71]
Poole	Dermatitis [1.43]	Neonatal [1.07]	Congenital [0.84]	Asthma [1.15]	URI [1.33]	LRI [1.14]	Endocrine [1.18]	Diarrhea [0.88]	Skin Viral [0.86]	Other MN [1.49]
Somerset	Dermatitis [1.44]	Neonatal [1.04]	Congenital [0.54]	Asthma [1.06]	URI [1.33]	Iron [6.08]	Diarrhea [0.78]	Skin Viral [0.84]	Endocrine [0.87]	ASD [1.1]
S Gloucestershire	Dermatitis [1.43]	Neonatal [1.11]	Congenital [0.67]	Asthma [1.2]	URI [1.29]	Skin Viral [0.88]	ASD [1.3]	Diarrhea [0.79]	Endocrine [0.8]	Other MN [1.22]
Swindon	Dermatitis [1.44]	Neonatal [0.8]	Congenital [0.63]	Asthma [1.15]	URI [1.38]	Skin Viral [0.86]	Diarrhea [0.87]	LRI [0.9]	ASD [1.11]	Endocrine [0.79]
Torbay	Dermatitis [1.46]	Neonatal [0.84]	Congenital [0.55]	Asthma [1.02]	URI [1.32]	Iron [4.39]	Endocrine [1.05]	Skin Viral [0.83]	Diarrhea [0.67]	ASD [1.09]
Wiltshire	Dermatitis [1.44]	Neonatal [1.15]	Congenital [0.57]	Asthma [1.1]	URI [1.32]	Skin Viral [0.85]	Diarrhea [0.77]	ASD [1.1]	Endocrine [0.79]	LRI [0.59]
W Midlands	Dermatitis [1.44]	Neonatal [1.0]	Congenital [0.72]	Asthma [1.13]	URI [1.3]	Diarrhea [0.92]	Endocrine [1.15]	Skin Viral [0.85]	LRI [0.77]	Epilepsy [0.93]
Birmingham	Dermatitis [1.44]	Congenital [0.81]	Neonatal [1.02]	Asthma [1.16]	URI [1.27]	Endocrine [1.38]	Diarrhea [0.98]	LRI [1.04]	Skin Viral [0.86]	Falls [0.84]

eFigure 7c. Leading ten causes of DALYs with the ratio of observed DALYs to DALYs expected on the basis of Socio-Demographic Index alone in 2017, 1-4 years, both sexes combined. The top ten causes contributing to DALYs are listed globally, by socio-demographic quintile, and then by GBD superregion, region, country, and subnationally where modeled. For each cell, the ratio of observed DALYs to DALYs expected on the basis of socio-demographic index (SDI) alone are listed. Abbreviations: DALY=disability-adjusted life year, GBD=Global Burden of Disease.

Values shown in brackets represent the ratio of observed DALYs to predicted DALYs on the basis of Socio-Demographic Index (SDI), rounded to two (2) digits. Color ranges (shown below) were calculated to place a roughly equal number of cells into each bin.

COLOR KEY:	[0.0-0.5]	(0.5-0.72]	(0.72-0.86]	(0.86-0.97]	(0.97-1.1]	(1.1-1.23]	(1.23-1.41]	(1.41-2.15]	2.15+

	1	2	3	4	5	6	7	8	9	10
Coventry	Dermatitis [1.43]	Neonatal [0.98]	Congenital [0.78]	Asthma [1.2]	URI [1.28]	Endocrine [1.22]	Diarrhea [0.9]	Skin Viral [0.86]	LRI [0.94]	Epilepsy [1.09]
Dudley	Dermatitis [1.45]	Neonatal [1.05]	Congenital [0.67]	Asthma [1.06]	URI [1.31]	Endocrine [1.19]	Diarrhea [0.86]	LRI [0.54]	Skin Viral [0.84]	Epilepsy [0.88]
Herefordshire	Dermatitis [1.43]	Neonatal [1.02]	Congenital [0.71]	Asthma [1.13]	URI [1.29]	Diarrhea [0.93]	Endocrine [1.09]	Skin Viral [0.85]	ASD [1.11]	Other MN [1.19]
Sandwell	Dermatitis [1.45]	Neonatal [1.02]	Congenital [0.58]	Asthma [1.06]	URI [1.32]	Endocrine [1.18]	Diarrhea [0.84]	Skin Viral [0.83]	LRI [0.46]	ASD [1.1]
Shropshire	Dermatitis [1.44]	Congenital [0.81]	Neonatal [1.0]	Asthma [1.12]	URI [1.31]	Diarrhea [0.87]	LRI [0.84]	Skin Viral [0.85]	Other MN [1.33]	Endocrine [0.92]
Solihull	Dermatitis [1.43]	Congenital [0.86]	Neonatal [0.79]	Asthma [1.19]	URI [1.35]	Diarrhea [0.92]	Skin Viral [0.87]	Endocrine [1.09]	Other MN [1.57]	LRI [1.01]
Staffordshire	Dermatitis [1.44]	Neonatal [0.96]	Congenital [0.63]	Asthma [1.1]	URI [1.31]	Diarrhea [0.96]	Skin Viral [0.85]	Endocrine [0.93]	ASD [1.04]	Falls [0.75]
Stoke-on-Trent	Dermatitis [1.45]	Neonatal [1.15]	Congenital [0.73]	Asthma [1.08]	URI [1.27]	Diarrhea [0.92]	Endocrine [1.1]	LRI [0.61]	Skin Viral [0.84]	Falls [0.92]
Telford & Wrekin	Dermatitis [1.44]	Neonatal [1.01]	Congenital [0.72]	Asthma [1.11]	URI [1.33]	Diarrhea [0.86]	LRI [0.73]	Endocrine [1.01]	Skin Viral [0.84]	Iron [5.17]
Walsall	Dermatitis [1.45]	Neonatal [0.97]	Congenital [0.62]	Asthma [1.05]	URI [1.3]	Endocrine [1.23]	LRI [0.53]	Diarrhea [0.73]	Skin Viral [0.83]	ASD [1.1]
Warwickshire	Dermatitis [1.43]	Neonatal [1.02]	Congenital [0.68]	Asthma [1.19]	URI [1.29]	Skin Viral [0.87]	Diarrhea [0.91]	Endocrine [0.94]	ASD [1.1]	LRI [0.81]
Wolverhampton	Dermatitis [1.45]	Congenital [0.62]	Neonatal [0.75]	Asthma [1.08]	URI [1.33]	Diarrhea [0.93]	Endocrine [1.14]	Skin Viral [0.84]	LRI [0.62]	ASD [1.09]
Worcestershire	Dermatitis [1.43]	Neonatal [1.09]	Congenital [0.68]	Asthma [1.13]	URI [1.36]	Skin Viral [0.85]	Endocrine [1.04]	Diarrhea [0.83]	LRI [0.75]	ASD [1.11]
Yorkshire & Humber	Dermatitis [1.44]	Neonatal [0.95]	Congenital [0.7]	Asthma [1.14]	URI [1.3]	Diarrhea [0.92]	Skin Viral [0.85]	Endocrine [1.0]	LRI [0.76]	ASD [1.1]
Barnsley	Dermatitis [1.46]	Neonatal [0.97]	Congenital [0.6]	Asthma [1.06]	URI [1.3]	Diarrhea [0.83]	LRI [0.49]	Skin Viral [0.83]	Endocrine [0.88]	Epilepsy [0.84]
Bradford	Dermatitis [1.45]	Congenital [0.74]	Neonatal [1.02]	Asthma [1.11]	URI [1.29]	LRI [0.76]	Endocrine [1.2]	Diarrhea [0.85]	Skin Viral [0.84]	Epilepsy [0.89]
Calderdale	Dermatitis [1.44]	Congenital [0.85]	Neonatal [0.96]	Asthma [1.14]	URI [1.34]	LRI [1.1]	Endocrine [1.37]	Diarrhea [0.95]	Other MN [1.55]	Skin Viral [0.85]
Doncaster	Dermatitis [1.46]	Neonatal [1.12]	Congenital [0.55]	Asthma [1.05]	URI [1.33]	Diarrhea [0.78]	Skin Viral [0.83]	LRI [0.41]	Endocrine [0.8]	ASD [1.1]
E Riding of Yorkshire	Dermatitis [1.44]	Neonatal [0.91]	Congenital [0.64]	Asthma [1.1]	URI [1.28]	Diarrhea [0.82]	Skin Viral [0.84]	ASD [1.11]	Endocrine [0.83]	LRI [0.56]
Kingston upon Hull	Dermatitis [1.44]	Neonatal [0.87]	Congenital [0.63]	Asthma [1.12]	URI [1.28]	Diarrhea [0.85]	Skin Viral [0.84]	LRI [0.66]	Endocrine [0.92]	Epilepsy [0.96]
Kirklees	Dermatitis [1.45]	Congenital [0.7]	Neonatal [0.89]	Asthma [1.11]	URI [1.31]	Diarrhea [0.9]	LRI [0.73]	Endocrine [1.1]	Skin Viral [0.84]	Iron [4.88]
Leeds	Dermatitis [1.43]	Neonatal [0.91]	Congenital [0.74]	Asthma [1.24]	URI [1.31]	Skin Viral [0.89]	Diarrhea [0.97]	Endocrine [1.02]	ASD [1.1]	LRI [1.02]
NE Lincolnshire	Dermatitis [1.45]	Congenital [0.7]	Neonatal [0.92]	Asthma [1.1]	URI [1.3]	LRI [0.72]	Endocrine [1.16]	Skin Viral [0.84]	Diarrhea [0.7]	Epilepsy [0.9]
N Lincolnshire	Dermatitis [1.44]	Neonatal [0.84]	Congenital [0.6]	Asthma [1.1]	URI [1.3]	Diarrhea [0.79]	Skin Viral [0.84]	LRI [0.54]	Endocrine [0.86]	ASD [1.1]
N Yorkshire	Dermatitis [1.43]	Neonatal [1.05]	Congenital [0.7]	Asthma [1.15]	URI [1.3]	Diarrhea [1.0]	Skin Viral [0.86]	Endocrine [0.94]	Other MN [1.3]	ASD [1.11]
Rotherham	Dermatitis [1.45]	Neonatal [0.83]	Congenital [0.56]	Asthma [1.06]	URI [1.29]	Diarrhea [0.76]	Skin Viral [0.83]	LRI [0.45]	ASD [1.11]	Endocrine [0.77]
Sheffield	Dermatitis [1.43]	Neonatal [1.01]	Congenital [0.74]	Asthma [1.19]	URI [1.29]	Diarrhea [1.01]	Skin Viral [0.87]	Endocrine [0.99]	ASD [1.11]	Iron [6.22]
Wakefield	Dermatitis [1.45]	Neonatal [0.74]	Congenital [0.51]	Asthma [1.09]	URI [1.29]	Diarrhea [0.91]	Skin Viral [0.84]	ASD [1.1]	Endocrine [0.8]	LRI [0.45]
York	Dermatitis [1.43]	Congenital [0.91]	Neonatal [0.94]	Asthma [1.28]	URI [1.34]	Diarrhea [1.05]	Skin Viral [0.9]	Other MN [1.81]	LRI [1.48]	ASD [1.1]
N Ireland	Dermatitis [1.19]	Neonatal [0.87]	Congenital [0.63]	Asthma [1.01]	URI [1.31]	Skin Viral [0.94]	Diarrhea [0.75]	Endocrine [0.85]	ASD [1.03]	Falls [0.71]
Scotland	Dermatitis [1.29]	Congenital [0.61]	Neonatal [0.8]	Asthma [1.02]	URI [1.31]	Skin Viral [0.91]	Diarrhea [0.64]	Endocrine [0.82]	ASD [1.04]	Epilepsy [0.79]

eFigure 7c. Leading ten causes of DALYs with the ratio of observed DALYs to DALYs expected on the basis of Socio-Demographic Index alone in 2017, 1-4 years, both sexes combined. The top ten causes contributing to DALYs are listed globally, by socio-demographic quintile, and then by GBD superregion, region, country, and subnationally where modeled. For each cell, the ratio of observed DALYs to DALYs expected on the basis of socio-demographic index (SDI) alone are listed. Abbreviations: DALY=disability-adjusted life year, GBD=Global Burden of Disease.

Values shown in brackets represent the ratio of observed DALYs to predicted DALYs on the basis of Socio-Demographic Index (SDI), rounded to two (2) digits. Color ranges (shown below) were calculated to place a roughly equal number of cells into each bin.

COLOR KEY:	[0.0-0.5]	(0.5-0.72]	(0.72-0.86]	(0.86-0.97]	(0.97-1.1]	(1.1-1.23]	(1.23-1.41]	(1.41-2.15]	2.15+

	1	2	3	4	5	6	7	8	9	10
Wales	Dermatitis [1.21]	Neonatal [0.8]	Congenital [0.53]	Asthma [1.01]	URI [1.32]	Skin Viral [1.02]	Diarrhea [0.68]	Endocrine [0.73]	ASD [0.98]	Falls [0.69]
Latin America and Caribbean	LRI [0.52]	Diarrhea [1.35]	Congenital [0.49]	Neonatal [1.0]	Asthma [1.27]	Road Inj [0.55]	Dermatitis [0.88]	HIV [3.21]	Drown [0.37]	PEM [2.45]
Andean Latin America	HIV [13.61]	LRI [0.68]	Congenital [0.44]	Diarrhea [1.02]	Neonatal [0.9]	Dermatitis [1.43]	Asthma [1.55]	Road Inj [0.72]	F Body [1.31]	Iron [1.92]
Bolivia	LRI [0.65]	HIV [9.5]	Congenital [0.5]	Diarrhea [0.72]	Iron [2.5]	Asthma [1.65]	Road Inj [0.77]	Neonatal [0.85]	Dermatitis [1.53]	F Body [1.34]
Ecuador	LRI [0.94]	Congenital [0.66]	Road Inj [1.1]	Diarrhea [1.06]	Neonatal [0.97]	Dermatitis [1.3]	Asthma [1.47]	Drown [0.61]	F Body [0.99]	Leukemia [0.92]
Peru	HIV [19.81]	LRI [0.47]	Diarrhea [0.96]	Neonatal [0.88]	Dermatitis [1.47]	Asthma [1.48]	Congenital [0.3]	F Body [1.37]	Iron [2.1]	Road Inj [0.49]
Caribbean	Diarrhea [3.8]	LRI [1.19]	Congenital [0.89]	Road Inj [1.5]	Asthma [2.83]	Whooping [15.1]	Drown [0.92]	Neonatal [0.89]	Meningitis [0.96]	PEM [5.48]
Antigua	LRI [1.63]	Congenital [0.78]	Asthma [2.37]	Neonatal [0.96]	Drown [1.04]	Dermatitis [1.03]	Iron [4.03]	Endocrine [2.34]	Diarrhea [1.13]	PEM [15.54]
Bahamas	Asthma [2.59]	LRI [1.51]	Congenital [0.6]	Neonatal [0.97]	Dermatitis [1.01]	Endocrine [2.8]	Drown [1.07]	Diarrhea [1.17]	HIV [7.04]	Road Inj [0.49]
Barbados	Asthma [2.18]	Neonatal [0.94]	Dermatitis [1.0]	Congenital [0.39]	LRI [0.57]	Diarrhea [0.92]	Urticaria [1.03]	Endocrine [0.99]	Skin Viral [0.81]	URI [0.84]
Belize	LRI [0.32]	Asthma [1.86]	Congenital [0.36]	Neonatal [0.87]	Dermatitis [1.19]	Drown [0.43]	Diarrhea [0.4]	PEM [1.66]	Iron [1.18]	Road Inj [0.35]
Bermuda	Asthma [3.23]	Congenital [0.75]	Neonatal [1.02]	Dermatitis [0.98]	LRI [1.42]	Drown [1.26]	Diarrhea [1.45]	Endocrine [1.73]	Leukemia [1.2]	Road Inj [0.55]
Cuba	Asthma [2.17]	Congenital [0.4]	Neonatal [0.79]	Dermatitis [1.06]	LRI [0.28]	Iron [1.55]	Diarrhea [0.42]	Urticaria [0.94]	Drown [0.26]	Leukemia [0.54]
Dominica	Congenital [0.97]	LRI [1.25]	Asthma [2.61]	Neonatal [1.13]	Drown [0.92]	Dermatitis [1.07]	Endocrine [2.55]	Road Inj [0.66]	Diarrhea [0.96]	Leukemia [1.23]
Dominican Rep	Congenital [0.5]	LRI [0.32]	Diarrhea [0.72]	Neonatal [0.84]	Asthma [1.39]	Whooping [4.01]	Road Inj [0.57]	Dermatitis [1.2]	Violence [1.73]	Meningitis [0.29]
Grenada	LRI [0.41]	Neonatal [0.89]	Asthma [1.74]	Congenital [0.32]	Dermatitis [1.11]	Iron [2.28]	Diarrhea [0.51]	Drown [0.33]	Endocrine [1.17]	Vit A [0.78]
Guyana	LRI [0.23]	Neonatal [0.93]	Asthma [1.63]	Diarrhea [0.51]	Congenital [0.31]	PEM [2.0]	Dermatitis [1.2]	Drown [0.33]	Iron [1.11]	Endocrine [1.27]
Haiti	Diarrhea [0.69]	LRI [0.46]	Congenital [0.8]	Road Inj [1.53]	Whooping [1.27]	Drown [0.72]	Asthma [2.23]	Meningitis [0.45]	PEM [0.5]	Iron [1.47]
Jamaica	Neonatal [1.43]	Congenital [0.64]	Asthma [2.16]	Dermatitis [1.07]	LRI [0.28]	Iron [2.02]	Diarrhea [0.57]	HIV [3.42]	Vit A [0.98]	Violence [1.44]
Puerto Rico	Disaster [7969.54]	Asthma [3.91]	Neonatal [1.0]	Dermatitis [0.97]	Congenital [0.48]	Diarrhea [1.32]	Urticaria [1.19]	Endocrine [1.02]	Skin Viral [0.83]	Road Inj [0.44]
St Lucia	Asthma [1.99]	Neonatal [0.9]	Dermatitis [1.1]	Congenital [0.27]	Diarrhea [0.66]	LRI [0.23]	Drown [0.22]	Urticaria [0.91]	Endocrine [0.79]	Epilepsy [0.66]
St Vincent	Asthma [1.71]	Neonatal [0.85]	Congenital [0.29]	LRI [0.2]	Dermatitis [1.17]	Diarrhea [0.36]	PEM [1.87]	Vit A [0.93]	Endocrine [1.39]	Drown [0.23]
Suriname	Asthma [2.06]	Congenital [0.41]	Neonatal [0.96]	LRI [0.39]	Diarrhea [0.87]	Dermatitis [1.12]	Drown [0.45]	HIV [3.02]	Road Inj [0.37]	PEM [2.44]
Trinidad Tobago	Congenital [0.75]	Asthma [2.54]	Neonatal [0.97]	Dermatitis [1.05]	LRI [0.48]	Road Inj [0.55]	Diarrhea [0.77]	Endocrine [1.41]	Violence [1.87]	F Body [0.93]
Virgin Isl US	Asthma [2.88]	Neonatal [1.02]	Congenital [0.67]	Dermatitis [0.98]	Diarrhea [1.62]	Disaster [568.38]	Endocrine [1.57]	LRI [0.91]	Drown [0.74]	Urticaria [1.17]
Central Latin America	Diarrhea [1.22]	Congenital [0.49]	LRI [0.42]	Neonatal [1.15]	Road Inj [0.45]	Dermatitis [0.78]	Asthma [0.81]	PEM [2.16]	Violence [1.61]	Drown [0.28]
Colombia	Neonatal [1.18]	Congenital [0.41]	LRI [0.33]	Diarrhea [0.82]	Drown [0.47]	Dermatitis [0.86]	PEM [3.12]	Asthma [0.83]	Road Inj [0.35]	Epilepsy [1.07]
Costa Rica	Neonatal [1.05]	Congenital [0.38]	Diarrhea [0.93]	Asthma [1.19]	Dermatitis [0.81]	LRI [0.13]	Epilepsy [0.84]	Road Inj [0.26]	Drown [0.22]	Urticaria [0.91]
El Salvador	HIV [12.7]	Neonatal [1.06]	Diarrhea [0.46]	Congenital [0.22]	Asthma [0.9]	Dermatitis [0.82]	LRI [0.11]	Vit A [0.53]	Whooping [1.26]	Epilepsy [0.67]
Guatemala	LRI [0.83]	Diarrhea [1.32]	Congenital [0.39]	Neonatal [1.03]	PEM [1.09]	F Body [0.93]	Dermatitis [1.01]	Asthma [0.66]	Iron [0.66]	Road Inj [0.25]
Honduras	Diarrhea [0.29]	Neonatal [1.11]	Asthma [1.06]	Violence [1.72]	Congenital [0.15]	Dermatitis [1.01]	Endocrine [1.67]	Whooping [0.55]	Leukemia [0.79]	LRI [0.05]

eFigure 7c. Leading ten causes of DALYs with the ratio of observed DALYs to DALYs expected on the basis of Socio-Demographic Index alone in 2017, 1-4 years, both sexes combined. The top ten causes contributing to DALYs are listed globally, by socio-demographic quintile, and then by GBD superregion, region, country, and subnationally where modeled. For each cell, the ratio of observed DALYs to DALYs expected on the basis of socio-demographic index (SDI) alone are listed. Abbreviations: DALY=disability-adjusted life year, GBD=Global Burden of Disease.

Values shown in brackets represent the ratio of observed DALYs to predicted DALYs on the basis of Socio-Demographic Index (SDI), rounded to two (2) digits. Color ranges (shown below) were calculated to place a roughly equal number of cells into each bin.

COLOR KEY:	[0.0-0.5]	(0.5-0.72]	(0.72-0.86]	(0.86-0.97]	(0.97-1.1]	(1.1-1.23]	(1.23-1.41]	(1.41-2.15]	2.15+

	1	2	3	4	5	6	7	8	9	10
Mexico	Congenital [0.57]	Neonatal [1.17]	Diarrhea [0.79]	LRI [0.26]	Road Inj [0.54]	Violence [2.1]	Epilepsy [1.24]	Dermatitis [0.65]	Asthma [0.67]	Vit A [0.88]
Aguascalientes	Congenital [0.58]	Neonatal [1.18]	Diarrhea [0.86]	Road Inj [0.63]	LRI [0.2]	Asthma [0.8]	Dermatitis [0.64]	Epilepsy [1.29]	F Body [0.8]	Violence [1.6]
Baja CA	Neonatal [1.13]	Congenital [0.41]	Violence [2.13]	F Body [0.94]	Diarrhea [0.54]	Road Inj [0.41]	Dermatitis [0.64]	LRI [0.18]	Epilepsy [1.09]	Vit A [0.92]
Baja CA Sur	Neonatal [1.2]	Congenital [0.43]	Diarrhea [0.79]	Road Inj [0.52]	LRI [0.21]	Dermatitis [0.64]	Violence [1.87]	Vit A [1.12]	Asthma [0.74]	Epilepsy [1.13]
Campeche	Neonatal [1.22]	Congenital [0.43]	Diarrhea [0.55]	LRI [0.16]	Road Inj [0.38]	Vit A [0.95]	Dermatitis [0.67]	Violence [1.57]	Asthma [0.65]	Drown [0.24]
Chiapas	Diarrhea [0.52]	LRI [0.2]	Congenital [0.36]	Neonatal [1.1]	Vit A [0.76]	PEM [0.51]	Epilepsy [1.12]	Dermatitis [0.72]	Leukemia [0.67]	Asthma [0.52]
Chihuahua	Congenital [0.65]	Neonatal [1.15]	Road Inj [0.97]	LRI [0.36]	Diarrhea [0.87]	Violence [3.29]	F Body [1.46]	Drown [0.39]	Epilepsy [1.38]	Asthma [0.81]
Coahuila	Congenital [0.6]	Neonatal [1.03]	Diarrhea [0.83]	Road Inj [0.6]	LRI [0.21]	Violence [1.92]	F Body [0.87]	Asthma [0.77]	Dermatitis [0.65]	Epilepsy [1.23]
Colima	Neonatal [1.24]	Congenital [0.51]	Diarrhea [0.73]	Road Inj [0.55]	Violence [2.31]	LRI [0.22]	Dermatitis [0.64]	Asthma [0.73]	Epilepsy [1.22]	Drown [0.33]
Mexico City	Congenital [0.92]	Neonatal [1.33]	LRI [0.75]	Diarrhea [1.24]	Violence [3.12]	Dermatitis [0.77]	Epilepsy [1.62]	Road Inj [0.54]	Leukemia [1.04]	Vit A [1.26]
Durango	Congenital [0.56]	Neonatal [1.08]	Road Inj [0.75]	Diarrhea [0.58]	LRI [0.2]	Violence [2.38]	Dermatitis [0.86]	F Body [0.86]	Asthma [0.76]	Drown [0.3]
Guanajuato	Congenital [0.45]	Neonatal [1.02]	Diarrhea [0.49]	Road Inj [0.52]	LRI [0.15]	Vit A [0.99]	Dermatitis [0.67]	Asthma [0.71]	Epilepsy [1.13]	Violence [1.5]
Guerrero	Congenital [0.74]	Diarrhea [0.59]	LRI [0.25]	Neonatal [1.27]	Violence [3.77]	Road Inj [0.82]	PEM [1.16]	Drown [0.35]	F Body [0.95]	Leukemia [1.01]
Hidalgo	Neonatal [1.31]	Congenital [0.35]	Diarrhea [0.27]	LRI [0.1]	Dermatitis [0.7]	Epilepsy [1.08]	Road Inj [0.3]	Asthma [0.6]	Violence [1.32]	F Body [0.5]
Jalisco	Congenital [0.55]	Neonatal [1.13]	Road Inj [0.7]	Diarrhea [0.64]	LRI [0.21]	Violence [1.93]	Epilepsy [1.36]	Dermatitis [0.65]	Vit A [0.95]	Drown [0.31]
Mexico	Neonatal [1.33]	Congenital [0.46]	Diarrhea [0.66]	LRI [0.19]	Violence [2.18]	Vit A [1.23]	Road Inj [0.38]	Iron [1.25]	Epilepsy [1.11]	Dermatitis [0.57]
Michoacan	Congenital [0.56]	Neonatal [1.02]	Road Inj [0.76]	LRI [0.19]	Diarrhea [0.39]	Violence [2.21]	Drown [0.35]	F Body [0.81]	Asthma [0.67]	Leukemia [0.75]
Morelos	Congenital [0.56]	Neonatal [1.13]	Diarrhea [0.61]	Violence [2.32]	LRI [0.18]	Road Inj [0.42]	Epilepsy [1.2]	Disaster [126.68]	Dermatitis [0.56]	Asthma [0.63]
Nayarit	Congenital [0.5]	Neonatal [1.23]	Road Inj [0.71]	Diarrhea [0.59]	LRI [0.2]	Violence [2.17]	Drown [0.32]	Dermatitis [0.67]	Asthma [0.7]	Epilepsy [1.14]
Nuevo Leon	Congenital [0.55]	Neonatal [0.97]	Diarrhea [0.78]	LRI [0.24]	Violence [2.03]	Road Inj [0.45]	Dermatitis [0.54]	Epilepsy [1.18]	F Body [0.8]	Asthma [0.6]
Oaxaca	Congenital [0.86]	Diarrhea [0.77]	LRI [0.3]	PEM [2.14]	Neonatal [1.18]	Road Inj [0.62]	Violence [2.54]	Epilepsy [1.62]	F Body [0.81]	Drown [0.28]
Puebla	Congenital [0.74]	LRI [0.31]	Neonatal [1.13]	Diarrhea [0.55]	Road Inj [0.6]	PEM [1.42]	Violence [1.67]	F Body [0.74]	Leukemia [0.87]	Drown [0.26]
Queretaro	Congenital [0.51]	Neonatal [1.2]	Diarrhea [0.61]	Road Inj [0.49]	LRI [0.18]	Dermatitis [0.65]	Asthma [0.73]	Epilepsy [1.17]	Vit A [0.93]	Violence [1.57]
Quintana Roo	Neonatal [1.4]	Congenital [0.43]	Diarrhea [0.7]	LRI [0.17]	Road Inj [0.43]	Violence [1.75]	Dermatitis [0.67]	Asthma [0.67]	Vit A [0.88]	F Body [0.65]
San Luis Potosi	Congenital [0.66]	Neonatal [1.04]	LRI [0.28]	Diarrhea [0.66]	Road Inj [0.65]	Epilepsy [1.5]	Vit A [1.06]	Violence [1.84]	Drown [0.32]	Asthma [0.73]
Sinaloa	Congenital [0.53]	Neonatal [1.07]	Road Inj [0.76]	Diarrhea [0.71]	Violence [2.5]	LRI [0.22]	Vit A [1.31]	Drown [0.4]	Dermatitis [0.64]	Asthma [0.75]
Sonora	Congenital [0.48]	Neonatal [1.07]	Road Inj [0.68]	Diarrhea [0.77]	LRI [0.24]	Violence [2.01]	Vit A [1.19]	Dermatitis [0.64]	Asthma [0.75]	Epilepsy [1.24]
Tabasco	Congenital [0.62]	Neonatal [1.25]	Diarrhea [0.63]	Drown [0.52]	LRI [0.21]	Road Inj [0.64]	Violence [1.7]	Epilepsy [1.19]	Leukemia [0.81]	Dermatitis [0.67]
Tamaulipas	Congenital [0.54]	Neonatal [1.27]	Diarrhea [0.72]	Road Inj [0.6]	Violence [2.14]	Vit A [1.24]	LRI [0.17]	Drown [0.33]	Asthma [0.7]	Dermatitis [0.54]
Tlaxcala	Neonatal [1.19]	Congenital [0.43]	LRI [0.19]	Diarrhea [0.43]	Road Inj [0.42]	Epilepsy [1.16]	Dermatitis [0.69]	Asthma [0.64]	Violence [1.41]	F Body [0.51]
Veracruz	Congenital [0.45]	Neonatal [1.16]	Diarrhea [0.41]	LRI [0.12]	Violence [1.51]	Dermatitis [0.7]	Epilepsy [1.09]	Asthma [0.63]	Iron [0.83]	PEM [0.82]

eFigure 7c. Leading ten causes of DALYs with the ratio of observed DALYs to DALYs expected on the basis of Socio-Demographic Index alone in 2017, 1-4 years, both sexes combined. The top ten causes contributing to DALYs are listed globally, by socio-demographic quintile, and then by GBD superregion, region, country, and subnationally where modeled. For each cell, the ratio of observed DALYs to DALYs expected on the basis of socio-demographic index (SDI) alone are listed. Abbreviations: DALY=disability-adjusted life year, GBD=Global Burden of Disease.

Values shown in brackets represent the ratio of observed DALYs to predicted DALYs on the basis of Socio-Demographic Index (SDI), rounded to two (2) digits. Color ranges (shown below) were calculated to place a roughly equal number of cells into each bin.

COLOR KEY:	[0.0-0.5]	(0.5-0.72]	(0.72-0.86]	(0.86-0.97]	(0.97-1.1]	(1.1-1.23]	(1.23-1.41]	(1.41-2.15]	2.15+

	1	2	3	4	5	6	7	8	9	10
Yucatan	Congenital [0.52]	Neonatal [1.07]	LRI [0.27]	Diarrhea [0.7]	Road Inj [0.38]	Dermatitis [0.63]	Epilepsy [1.16]	Violence [1.51]	Asthma [0.66]	Vit A [0.83]
Zacatecas	Congenital [0.42]	Neonatal [0.82]	Road Inj [0.67]	LRI [0.17]	Diarrhea [0.43]	Epilepsy [1.21]	Violence [1.6]	Asthma [0.7]	Dermatitis [0.68]	F Body [0.61]
Nicaragua	HIV [3.83]	Neonatal [1.03]	Diarrhea [0.24]	LRI [0.12]	Congenital [0.22]	Dermatitis [1.01]	Asthma [0.62]	Iron [0.51]	Epilepsy [0.62]	PEM [0.26]
Panama	Diarrhea [3.47]	LRI [1.15]	Congenital [0.69]	PEM [16.33]	Neonatal [1.11]	Asthma [1.51]	Dermatitis [1.01]	Drown [0.59]	Road Inj [0.47]	Iron [1.53]
Venezuela	Diarrhea [2.01]	Congenital [0.55]	Neonatal [1.15]	LRI [0.44]	Road Inj [0.6]	Dermatitis [0.9]	Asthma [0.96]	PEM [3.83]	Drown [0.35]	F Body [0.81]
**Tropical Latin America**	Congenital [0.41]	Neonatal [0.84]	LRI [0.38]	Asthma [1.48]	Diarrhea [0.81]	Dermatitis [0.77]	Road Inj [0.4]	Vit A [1.1]	Drown [0.33]	Urticaria [0.95]
Brazil	Congenital [0.43]	Neonatal [0.85]	LRI [0.42]	Asthma [1.5]	Diarrhea [0.84]	Dermatitis [0.75]	Road Inj [0.43]	Vit A [1.13]	Drown [0.36]	Urticaria [0.95]
Acre	LRI [0.33]	Congenital [0.39]	Neonatal [0.85]	Diarrhea [0.55]	Asthma [1.17]	Iron [1.37]	Drown [0.35]	Dermatitis [0.83]	Road Inj [0.34]	Vit A [0.84]
Alagoas	LRI [0.18]	Diarrhea [0.37]	Congenital [0.3]	Neonatal [0.73]	Asthma [0.92]	Road Inj [0.33]	Vit A [0.66]	Dermatitis [0.71]	PEM [0.55]	Drown [0.19]
Amapa	LRI [0.76]	Congenital [0.46]	Drown [0.9]	Neonatal [0.89]	Asthma [1.4]	Diarrhea [0.74]	Dermatitis [0.76]	Road Inj [0.46]	Vit A [1.04]	Iron [1.29]
Amazonas	LRI [0.46]	Congenital [0.45]	Diarrhea [1.05]	Drown [0.69]	Neonatal [0.75]	Asthma [1.3]	Dermatitis [0.79]	Vit A [0.96]	Road Inj [0.36]	PEM [1.88]
Bahia	Neonatal [0.78]	Congenital [0.27]	Diarrhea [0.39]	Asthma [1.08]	LRI [0.14]	Dermatitis [1.09]	Vit A [1.09]	Drown [0.23]	Road Inj [0.24]	Urticaria [0.91]
Ceara	Neonatal [0.7]	Asthma [1.09]	Congenital [0.21]	Diarrhea [0.32]	Dermatitis [0.83]	Vit A [0.92]	LRI [0.09]	Iron [0.68]	Leish [31.01]	Urticaria [0.91]
Distrito Federal	Congenital [0.81]	Neonatal [1.01]	Asthma [2.06]	Dermatitis [0.68]	LRI [1.11]	Road Inj [0.87]	Diarrhea [1.26]	Urticaria [1.18]	Drown [0.5]	Vit A [1.63]
Espirito Santo	Congenital [0.52]	Neonatal [0.84]	Asthma [1.76]	LRI [0.36]	Dermatitis [0.75]	Diarrhea [0.74]	Road Inj [0.51]	Drown [0.43]	Vit A [1.15]	Iron [1.18]
Goias	Congenital [0.42]	Neonatal [0.75]	Asthma [1.47]	LRI [0.29]	Road Inj [0.5]	Dermatitis [0.77]	Diarrhea [0.57]	Vit A [0.99]	Drown [0.24]	Urticaria [0.94]
Maranhao	Congenital [0.28]	LRI [0.11]	Diarrhea [0.16]	Neonatal [0.74]	Vit A [0.95]	Asthma [0.74]	Road Inj [0.31]	Dermatitis [0.86]	Drown [0.17]	Leish [9.0]
Mato Grosso	LRI [0.62]	Congenital [0.49]	Neonatal [0.82]	Asthma [1.59]	Diarrhea [0.95]	Road Inj [0.71]	Drown [0.54]	Dermatitis [0.76]	PEM [3.27]	Vit A [0.89]
Mato Grosso do Sul	Congenital [0.49]	LRI [0.46]	Neonatal [0.88]	Diarrhea [0.91]	Asthma [1.44]	Leish [155.04]	Road Inj [0.56]	Dermatitis [0.77]	Vit A [1.3]	Drown [0.33]
Minas Gerais	Congenital [0.49]	Neonatal [0.9]	Asthma [1.59]	LRI [0.38]	Diarrhea [0.73]	Road Inj [0.47]	Dermatitis [0.66]	Drown [0.35]	Vit A [1.01]	Leukemia [0.56]
Para	LRI [0.36]	Congenital [0.37]	Diarrhea [0.54]	Neonatal [0.79]	Drown [0.49]	Asthma [1.11]	Road Inj [0.46]	Iron [1.02]	Vit A [0.92]	Dermatitis [0.85]
Paraiba	Congenital [0.31]	LRI [0.17]	Neonatal [0.69]	Diarrhea [0.34]	Asthma [0.99]	Dermatitis [0.85]	Vit A [0.72]	Road Inj [0.29]	Drown [0.17]	Iron [0.45]
Parana	Congenital [0.55]	Neonatal [0.87]	Asthma [1.76]	Road Inj [0.64]	LRI [0.33]	Diarrhea [0.79]	Dermatitis [0.67]	Vit A [1.22]	Drown [0.35]	Epilepsy [0.89]
Pernambuco	Congenital [0.27]	Neonatal [0.72]	Asthma [1.15]	Diarrhea [0.37]	LRI [0.12]	Dermatitis [0.81]	Vit A [0.81]	Urticaria [0.91]	Drown [0.15]	Road Inj [0.19]
Piaui	Congenital [0.29]	Neonatal [0.76]	Diarrhea [0.24]	LRI [0.11]	Asthma [0.93]	Dermatitis [0.86]	Leish [24.68]	Road Inj [0.25]	Vit A [0.47]	Drown [0.13]
Rio de Janeiro	Neonatal [1.08]	Congenital [0.53]	LRI [0.86]	Asthma [1.67]	Dermatitis [0.73]	Diarrhea [0.83]	F Body [1.04]	Road Inj [0.44]	Vit A [1.21]	Drown [0.4]
Rio Grande do Norte	Neonatal [0.66]	Congenital [0.25]	Diarrhea [0.46]	Asthma [1.11]	Vit A [1.22]	LRI [0.15]	Dermatitis [0.82]	Urticaria [0.91]	Road Inj [0.2]	Drown [0.13]
Rio Grande do Sul	Congenital [0.64]	Neonatal [0.89]	Asthma [1.93]	Dermatitis [0.95]	LRI [0.42]	Epilepsy [1.42]	Diarrhea [0.65]	Road Inj [0.43]	Drown [0.39]	Vit A [0.99]
Rondonia	Congenital [0.39]	Neonatal [0.84]	LRI [0.27]	Asthma [1.23]	Diarrhea [0.47]	Dermatitis [0.81]	Road Inj [0.4]	Drown [0.32]	Vit A [0.98]	Urticaria [0.92]
Roraima	LRI [0.68]	Congenital [0.49]	Neonatal [0.92]	Diarrhea [0.97]	Road Inj [0.7]	Drown [0.6]	Asthma [1.31]	Dermatitis [0.78]	PEM [3.1]	Vit A [0.9]
Santa Catarina	Congenital [0.54]	Asthma [1.94]	Neonatal [0.82]	LRI [0.52]	Road Inj [0.78]	Dermatitis [0.73]	Diarrhea [0.76]	Drown [0.48]	Vit A [1.29]	Leukemia [0.67]

eFigure 7c. Leading ten causes of DALYs with the ratio of observed DALYs to DALYs expected on the basis of Socio-Demographic Index alone in 2017, 1-4 years, both sexes combined. The top ten causes contributing to DALYs are listed globally, by socio-demographic quintile, and then by GBD superregion, region, country, and subnationally where modeled. For each cell, the ratio of observed DALYs to DALYs expected on the basis of socio-demographic index (SDI) alone are listed. Abbreviations: DALY=disability-adjusted life year, GBD=Global Burden of Disease.

Values shown in brackets represent the ratio of observed DALYs to predicted DALYs on the basis of Socio-Demographic Index (SDI), rounded to two (2) digits. Color ranges (shown below) were calculated to place a roughly equal number of cells into each bin.

COLOR KEY:		[0.0-0.5]	(0.5-0.72]	(0.72-0.86]	(0.86-0.97]	(0.97-1.1]	(1.1-1.23]	(1.23-1.41]	(1.41-2.15]	2.15+

	1	2	3	4	5	6	7	8	9	10
Sao Paulo	Congenital [0.51]	Neonatal [0.95]	Asthma [1.84]	LRI [0.71]	Dermatitis [0.63]	Diarrhea [0.77]	Road Inj [0.44]	Drown [0.4]	Urticaria [1.04]	Epilepsy [0.89]
Sergipe	LRI [0.37]	Congenital [0.41]	Neonatal [0.92]	Diarrhea [0.7]	Asthma [1.31]	Road Inj [0.61]	Drown [0.44]	Vit A [1.21]	Dermatitis [0.68]	PEM [1.53]
Tocantins	Leish [132.03]	Neonatal [0.82]	Congenital [0.29]	Asthma [1.16]	LRI [0.18]	Diarrhea [0.44]	Vit A [1.26]	Road Inj [0.42]	Dermatitis [0.82]	Iron [1.04]
Paraguay	HIV [15.02]	Asthma [1.42]	Diarrhea [0.65]	Neonatal [0.6]	Dermatitis [1.04]	Vit A [0.84]	Congenital [0.13]	Iron [0.86]	Urticaria [0.88]	Skin Viral [0.83]
**North Africa and Middle East**	Diarrhea [2.83]	Congenital [0.97]	LRI [0.78]	Conflict Terror [1172.11]	Road Inj [1.31]	Neonatal [1.11]	Whooping [8.46]	Iron [2.44]	Measles [14.2]	Asthma [1.08]
Afghanistan	LRI [0.22]	Measles [0.24]	Diarrhea [0.12]	Congenital [0.5]	Whooping [0.64]	Meningitis [0.37]	Drown [0.33]	Falls [2.81]	Neonatal [1.06]	Road Inj [0.34]
Algeria	Congenital [1.04]	Neonatal [1.11]	Road Inj [1.23]	Diarrhea [1.14]	Asthma [1.2]	LRI [0.39]	Dermatitis [0.64]	Drown [0.36]	Epilepsy [1.09]	Falls [1.17]
Bahrain	Congenital [0.65]	Neonatal [1.08]	Asthma [1.14]	Road Inj [0.67]	Diarrhea [1.05]	Dermatitis [0.72]	Epilepsy [1.08]	Endocrine [1.04]	Urticaria [1.05]	LRI [0.18]
Egypt	LRI [0.72]	Diarrhea [1.54]	Congenital [0.6]	Road Inj [1.29]	Neonatal [0.95]	Asthma [1.32]	Iron [1.03]	Encepha [0.9]	Dermatitis [0.57]	Cirrhosis [1.61]
Iran	Congenital [0.9]	Road Inj [1.66]	Neonatal [1.27]	Diarrhea [0.93]	LRI [0.34]	Asthma [0.83]	Dermatitis [0.51]	Epilepsy [1.21]	Fire [0.83]	Iron [1.61]
Iraq	Conflict Terror [3196.23]	Congenital [0.67]	Neonatal [1.14]	LRI [0.24]	Iron [1.33]	Road Inj [0.47]	Diarrhea [0.31]	Asthma [0.81]	Whooping [1.96]	Meningitis [0.26]
Jordan	Congenital [1.1]	Neonatal [1.52]	Road Inj [1.46]	LRI [0.55]	Asthma [1.34]	Diarrhea [1.07]	Dermatitis [0.64]	Vit A [1.17]	Iron [1.75]	Drown [0.37]
Kuwait	Congenital [0.91]	Neonatal [1.22]	Asthma [1.53]	Diarrhea [1.7]	Road Inj [0.95]	Dermatitis [0.61]	LRI [0.83]	Urticaria [1.2]	Epilepsy [1.14]	Skin Viral [0.8]
Lebanon	Neonatal [1.01]	Asthma [1.52]	Congenital [0.37]	Diarrhea [1.5]	Dermatitis [0.73]	Conflict Terror [569.26]	HIV [6.35]	Urticaria [1.08]	Epilepsy [0.87]	Iron [1.59]
Libya	Congenital [1.77]	Conflict Terror [3643.11]	Neonatal [1.27]	Road Inj [1.83]	Diarrhea [2.49]	Oth Cardio [20.47]	LRI [0.99]	Whooping [32.0]	Measles [127.66]	Asthma [1.28]
Morocco	Congenital [0.67]	Diarrhea [0.66]	Neonatal [0.99]	LRI [0.13]	Road Inj [0.48]	Meningitis [0.29]	Iron [0.93]	Dermatitis [0.78]	HIV [1.86]	Asthma [0.65]
Palestine	Congenital [0.51]	Neonatal [1.37]	Road Inj [0.53]	Diarrhea [0.16]	Asthma [0.71]	LRI [0.07]	Dermatitis [0.77]	Vit A [0.53]	Falls [0.96]	Epilepsy [0.73]
Oman	Road Inj [3.05]	Neonatal [1.18]	Congenital [0.64]	Diarrhea [1.54]	Drown [0.96]	Iron [4.92]	Asthma [1.05]	Dermatitis [0.57]	Oth Cardio [9.51]	LRI [0.51]
Qatar	Congenital [0.71]	Neonatal [1.02]	Road Inj [1.1]	Dermatitis [0.69]	Asthma [1.06]	Diarrhea [0.98]	Urticaria [1.16]	Epilepsy [1.07]	Drown [0.39]	Skin Viral [0.8]
Saudi Arabia	Congenital [1.04]	Neonatal [1.01]	Conflict Terror [1299.5]	Diarrhea [1.84]	Dermatitis [0.69]	Road Inj [0.83]	Asthma [0.94]	Urticaria [1.19]	Epilepsy [1.24]	Drown [0.56]
Sudan	Diarrhea [1.16]	Congenital [1.44]	LRI [0.37]	Road Inj [2.02]	Malaria [71.74]	Neonatal [1.07]	Drown [0.45]	Iron [1.29]	Whooping [0.8]	PEM [0.41]
Syria	Conflict Terror [12418.82]	Congenital [0.59]	Whooping [10.66]	LRI [0.39]	Neonatal [0.95]	PEM [2.99]	Asthma [1.08]	Measles [7.56]	Iron [1.37]	Diarrhea [0.34]
Tunisia	Congenital [0.52]	Neonatal [1.0]	Road Inj [0.62]	Diarrhea [0.8]	Mech [2.89]	Dermatitis [0.72]	Asthma [0.87]	HIV [3.06]	Iron [1.31]	LRI [0.16]
Turkey	Congenital [1.3]	Neonatal [1.14]	Road Inj [0.91]	LRI [0.62]	Endocrine [2.34]	Epilepsy [2.16]	Diarrhea [1.19]	Asthma [1.07]	Dermatitis [0.58]	Conflict Terror [473.99]
UAE	Congenital [2.05]	Road Inj [2.92]	Neonatal [1.12]	Asthma [1.77]	Diarrhea [2.15]	Dermatitis [0.68]	Epilepsy [1.86]	Drown [0.87]	PEM [47.26]	Other MN [1.85]
Yemen	Diarrhea [0.67]	Conflict Terror [754.88]	Iron [3.88]	Road Inj [0.95]	Congenital [0.41]	LRI [0.13]	Whooping [0.65]	Vit A [1.27]	Neonatal [1.05]	PEM [0.22]

eFigure 7c. Leading ten causes of DALYs with the ratio of observed DALYs to DALYs expected on the basis of Socio-Demographic Index alone in 2017, 1-4 years, both sexes combined. The top ten causes contributing to DALYs are listed globally, by socio-demographic quintile, and then by GBD superregion, region, country, and subnationally where modeled. For each cell, the ratio of observed DALYs to DALYs expected on the basis of socio-demographic index (SDI) alone are listed. Abbreviations: DALY=disability-adjusted life year, GBD=Global Burden of Disease.

Values shown in brackets represent the ratio of observed DALYs to predicted DALYs on the basis of Socio-Demographic Index (SDI), rounded to two (2) digits. Color ranges (shown below) were calculated to place a roughly equal number of cells into each bin.

COLOR KEY:		[0.0-0.5]	(0.5-0.72]	(0.72-0.86]	(0.86-0.97]	(0.97-1.1]	(1.1-1.23]	(1.23-1.41]	(1.41-2.15]	2.15+
	1	2	3	4	5	6	7	8	9	10
**South Asia**	Diarrhea [0.89]	LRI [0.44]	Drown [0.69]	Typh + Paratyph [49.05]	Iron [1.94]	Neonatal [1.12]	PEM [1.33]	Congenital [0.33]	Malaria [210.94]	Measles [2.32]
Bangladesh	Drown [1.55]	LRI [0.27]	Typh + Paratyph [22.19]	PEM [0.47]	Diarrhea [0.13]	Neonatal [1.0]	Congenital [0.25]	Whooping [0.55]	Iron [0.96]	Vit A [0.46]
Bhutan	Iron [4.45]	LRI [0.25]	Congenital [0.38]	Neonatal [1.05]	HIV [3.34]	Vit A [1.33]	Encepha [1.24]	Diarrhea [0.26]	Measles [2.76]	Malaria [262.96]
India	Diarrhea [0.99]	LRI [0.45]	Malaria [396.97]	Typh + Paratyph [64.94]	PEM [1.77]	Measles [3.92]	Iron [2.09]	Neonatal [1.12]	Congenital [0.31]	Drown [0.51]
Andhra Pradesh	Iron [2.01]	Neonatal [1.13]	PEM [1.06]	Vit A [1.31]	Diarrhea [0.23]	Typh + Paratyph [31.82]	Measles [1.49]	Congenital [0.18]	LRI [0.07]	Encepha [0.74]
Arunachal Pradesh	Diarrhea [0.76]	LRI [0.33]	Malaria [626.67]	Neonatal [1.14]	Measles [4.1]	PEM [1.63]	Iron [1.77]	Congenital [0.25]	Drown [0.4]	Typh + Paratyph [41.88]
Assam	Diarrhea [1.57]	LRI [0.75]	Malaria [576.55]	Drown [1.48]	Hep [10.74]	Measles [3.42]	Congenital [0.49]	PEM [1.45]	Neonatal [1.13]	Typh + Paratyph [40.11]
Bihar	Diarrhea [0.63]	LRI [0.43]	Measles [0.57]	Malaria [13.24]	Drown [0.49]	Neonatal [1.06]	PEM [0.36]	Iron [1.21]	Congenital [0.23]	Encepha [1.67]
Chhattisgarh	LRI [0.29]	Diarrhea [0.4]	Malaria [183.41]	Typh + Paratyph [36.58]	PEM [1.12]	Measles [1.87]	Neonatal [1.09]	Drown [0.41]	Congenital [0.23]	Vit A [0.91]
Delhi	LRI [1.94]	Iron [13.69]	Diarrhea [3.82]	Typh + Paratyph [1960.66]	Neonatal [1.31]	Congenital [0.58]	PEM [31.17]	Vit A [3.77]	Meningitis [1.07]	Measles [47.64]
Goa	LRI [1.97]	Neonatal [1.59]	Congenital [0.7]	Encepha [8.58]	Drown [1.5]	PEM [44.38]	Iron [7.18]	Diarrhea [2.11]	Measles [118.49]	Vit A [2.4]
Gujarat	LRI [0.38]	Iron [3.13]	Malaria [894.27]	Neonatal [1.17]	PEM [3.22]	Diarrhea [0.67]	Congenital [0.4]	Measles [6.48]	Typh + Paratyph [87.78]	Vit A [1.59]
Haryana	Iron [5.03]	LRI [0.39]	Typh + Paratyph [199.45]	Diarrhea [0.97]	Neonatal [1.18]	Vit A [2.03]	PEM [2.81]	Congenital [0.3]	Measles [8.06]	Malaria [504.32]
Himachal Pradesh	LRI [0.51]	Iron [4.54]	Neonatal [1.31]	Congenital [0.41]	Diarrhea [1.05]	Typh + Paratyph [248.12]	Vit A [2.12]	Measles [9.93]	Falls [1.47]	PEM [2.52]
Jammu & Kashmir	Iron [2.98]	Neonatal [1.13]	LRI [0.25]	Vit A [1.7]	Congenital [0.27]	Diarrhea [0.31]	Measles [4.15]	Typh + Paratyph [44.94]	Drown [0.23]	ID [7.1]
Jharkhand	Diarrhea [0.67]	LRI [0.35]	Drown [0.72]	Malaria [81.48]	PEM [0.87]	Measles [1.13]	Iron [1.71]	Congenital [0.35]	Neonatal [1.1]	Typh + Paratyph [13.96]
Karnataka	Congenital [0.49]	Neonatal [1.3]	Iron [2.26]	PEM [2.36]	Measles [5.88]	LRI [0.19]	Diarrhea [0.39]	Drown [0.45]	Vit A [1.38]	Encepha [1.2]
Kerala	Neonatal [1.58]	PEM [7.85]	Congenital [0.3]	Iron [2.36]	Measles [17.54]	Vit A [1.39]	Dermatitis [0.43]	Urticaria [0.96]	LRI [0.11]	Encepha [0.92]
Madhya Pradesh	LRI [0.36]	Diarrhea [0.33]	Typh + Paratyph [33.02]	Measles [1.46]	PEM [0.95]	Malaria [72.66]	Iron [1.82]	Drown [0.55]	Neonatal [1.01]	Vit A [1.21]
Maharashtra	Neonatal [1.27]	PEM [6.4]	LRI [0.4]	Iron [3.26]	Congenital [0.38]	Diarrhea [0.72]	Measles [12.0]	Typh + Paratyph [149.29]	Vit A [1.57]	Drown [0.48]
Manipur	LRI [0.64]	Diarrhea [1.42]	Drown [1.06]	Typh + Paratyph [165.54]	Congenital [0.52]	Neonatal [1.29]	Malaria [819.45]	Measles [7.19]	Whooping [3.95]	Road Inj [0.47]
Meghalaya	Malaria [2957.46]	LRI [0.89]	Diarrhea [1.33]	Congenital [0.65]	Typh + Paratyph [129.07]	Neonatal [1.25]	Measles [5.09]	Meningitis [0.55]	Drown [0.47]	Whooping [2.25]

eFigure 7c. Leading ten causes of DALYs with the ratio of observed DALYs to DALYs expected on the basis of Socio-Demographic Index alone in 2017, 1-4 years, both sexes combined. The top ten causes contributing to DALYs are listed globally, by socio-demographic quintile, and then by GBD superregion, region, country, and subnationally where modeled. For each cell, the ratio of observed DALYs to DALYs expected on the basis of socio-demographic index (SDI) alone are listed. Abbreviations: DALY=disability-adjusted life year, GBD=Global Burden of Disease.

Values shown in brackets represent the ratio of observed DALYs to predicted DALYs on the basis of Socio-Demographic Index (SDI), rounded to two (2) digits. Color ranges (shown below) were calculated to place a roughly equal number of cells into each bin.

COLOR KEY:	[0.0-0.5]	(0.5-0.72]	(0.72-0.86]	(0.86-0.97]	(0.97-1.1]	(1.1-1.23]	(1.23-1.41]	(1.41-2.15]	2.15+

	1	2	3	4	5	6	7	8	9	10
Mizoram	LRI [1.58]	Malaria [4504.71]	Typh + Paratyph [430.03]	Congenital [0.55]	Neonatal [1.31]	Diarrhea [0.96]	Drown [0.66]	PEM [3.59]	Meningitis [0.62]	Whooping [5.08]
Nagaland	LRI [1.97]	Malaria [6787.94]	Drown [1.95]	Typh + Paratyph [480.24]	Diarrhea [1.82]	Neonatal [1.45]	Hep [21.23]	Whooping [12.94]	Measles [24.75]	Congenital [0.46]
Odisha	Malaria [813.6]	Diarrhea [0.81]	LRI [0.36]	Drown [0.74]	PEM [1.35]	Typh + Paratyph [41.25]	Neonatal [1.1]	Congenital [0.3]	Hep [5.54]	Encepha [1.23]
Punjab	Diarrhea [1.69]	LRI [0.61]	Typh + Paratyph [310.53]	Congenital [0.54]	Neonatal [1.24]	Iron [3.6]	Vit A [1.78]	Measles [10.25]	PEM [3.15]	Drown [0.35]
Rajasthan	LRI [0.37]	Diarrhea [0.33]	Typh + Paratyph [35.08]	Measles [1.35]	PEM [0.82]	Malaria [69.29]	Iron [1.62]	Neonatal [0.96]	Drown [0.36]	Vit A [1.13]
Sikkim	LRI [0.82]	Measles [27.57]	Neonatal [1.33]	Diarrhea [1.12]	Typh + Paratyph [231.29]	Congenital [0.35]	Iron [2.75]	Vit A [1.57]	Encepha [1.69]	Drown [0.28]
Tamil Nadu	Neonatal [1.19]	PEM [4.51]	Iron [2.4]	Vit A [1.55]	Congenital [0.23]	Typh + Paratyph [94.52]	Diarrhea [0.34]	Measles [5.77]	LRI [0.11]	Encepha [0.75]
Telangana	Iron [2.96]	Neonatal [1.18]	PEM [2.54]	Vit A [1.61]	Typh + Paratyph [61.69]	Diarrhea [0.3]	Measles [3.09]	Congenital [0.17]	LRI [0.07]	Encepha [0.66]
Tripura	LRI [0.32]	Measles [4.71]	Diarrhea [0.49]	Neonatal [1.15]	Malaria [332.3]	Congenital [0.33]	PEM [1.3]	Whooping [1.84]	Drown [0.38]	Iron [1.03]
Uttar Pradesh	Diarrhea [0.78]	LRI [0.35]	Typh + Paratyph [32.41]	Malaria [82.27]	Iron [1.88]	PEM [0.85]	Measles [1.26]	Neonatal [0.98]	Congenital [0.27]	Meningitis [0.29]
Uttarakhand	Typh + Paratyph [256.62]	LRI [0.45]	Iron [4.04]	Neonatal [1.27]	Diarrhea [0.8]	Congenital [0.32]	Vit A [1.82]	PEM [2.27]	Malaria [504.77]	Drown [0.38]
W Bengal	Measles [3.98]	LRI [0.19]	Drown [0.62]	Neonatal [1.06]	Congenital [0.3]	Iron [1.43]	Diarrhea [0.24]	Vit A [1.14]	PEM [0.84]	Malaria [109.15]
UTs other than Delhi	LRI [0.77]	Neonatal [1.44]	Iron [5.65]	Congenital [0.5]	Diarrhea [1.46]	Drown [0.85]	Vit A [2.3]	PEM [7.11]	Malaria [3247.36]	Encepha [2.57]
Nepal	LRI [0.16]	Neonatal [1.05]	Diarrhea [0.1]	Whooping [0.41]	Typh + Paratyph [6.09]	HIV [1.51]	Iron [0.67]	Drown [0.2]	Malaria [3.73]	Encepha [0.8]
Pakistan	Diarrhea [0.94]	LRI [0.48]	Meningitis [0.75]	Drown [0.83]	Congenital [0.42]	Iron [1.95]	Neonatal [1.17]	Whooping [1.03]	Road Inj [0.62]	PEM [0.58]
**Southeast Asia, East Asia, and Oceania**	LRI [0.87]	Congenital [0.59]	Drown [1.36]	Diarrhea [1.81]	Neonatal [1.0]	Road Inj [0.7]	Asthma [1.19]	Measles [26.96]	Dermatitis [0.67]	Leukemia [0.89]
**East Asia**	Neonatal [1.26]	Drown [1.49]	Congenital [0.56]	Road Inj [1.01]	LRI [0.51]	Diarrhea [0.84]	Dermatitis [0.55]	Leukemia [0.95]	Falls [1.39]	Asthma [0.71]
China	Neonatal [1.27]	Drown [1.43]	Congenital [0.54]	Road Inj [0.99]	LRI [0.46]	Diarrhea [0.76]	Dermatitis [0.55]	Leukemia [0.93]	Falls [1.38]	Asthma [0.68]
N Korea	Drown [1.01]	LRI [0.3]	Congenital [0.55]	Diarrhea [0.41]	Road Inj [0.63]	Neonatal [0.69]	Whooping [1.15]	HIV [1.58]	Leukemia [0.84]	Vit A [0.64]
Taiwan	Asthma [2.58]	Congenital [0.82]	Diarrhea [2.64]	Neonatal [0.64]	Dermatitis [0.62]	Road Inj [0.9]	LRI [1.84]	Skin Viral [1.04]	Urticaria [1.24]	Drown [0.95]
**Oceania**	Diarrhea [0.86]	PEM [1.77]	LRI [0.33]	Congenital [0.77]	Drown [0.89]	Whooping [1.11]	Asthma [1.88]	Measles [0.93]	Meningitis [0.42]	Iron [1.23]
Am Samoa	Asthma [2.32]	LRI [0.71]	Congenital [0.45]	Diarrhea [1.61]	Measles [58.23]	Neonatal [0.72]	Dermatitis [0.91]	Drown [0.74]	Vit A [1.63]	PEM [9.59]
Micronesia	LRI [0.31]	Whooping [5.11]	Congenital [0.46]	HIV [4.54]	Asthma [1.47]	Diarrhea [0.4]	Neonatal [0.77]	Drown [0.41]	PEM [1.32]	Dermatitis [1.05]
Fiji	LRI [1.19]	Congenital [1.15]	Diarrhea [2.81]	Drown [1.32]	Asthma [2.09]	Measles [25.33]	Neonatal [0.87]	PEM [5.02]	Iron [2.37]	F Body [1.31]
Guam	Diarrhea [4.44]	Congenital [0.87]	Asthma [2.63]	LRI [2.49]	Neonatal [0.85]	Dermatitis [0.85]	Drown [1.11]	Measles [203.81]	Road Inj [0.86]	Vit A [2.28]

eFigure 7c. Leading ten causes of DALYs with the ratio of observed DALYs to DALYs expected on the basis of Socio-Demographic Index alone in 2017, 1-4 years, both sexes combined. The top ten causes contributing to DALYs are listed globally, by socio-demographic quintile, and then by GBD superregion, region, country, and subnationally where modeled. For each cell, the ratio of observed DALYs to DALYs expected on the basis of socio-demographic index (SDI) alone are listed. Abbreviations: DALY=disability-adjusted life year, GBD=Global Burden of Disease.

Values shown in brackets represent the ratio of observed DALYs to predicted DALYs on the basis of Socio-Demographic Index (SDI), rounded to two (2) digits. Color ranges (shown below) were calculated to place a roughly equal number of cells into each bin.

COLOR KEY:	[0.0-0.5]	(0.5-0.72]	(0.72-0.86]	(0.86-0.97]	(0.97-1.1]	(1.1-1.23]	(1.23-1.41]	(1.41-2.15]	2.15+

	1	2	3	4	5	6	7	8	9	10
Kiribati	PEM [1.34]	Measles [1.26]	Congenital [0.91]	Diarrhea [0.31]	LRI [0.17]	Whooping [0.89]	Meningitis [0.41]	Neonatal [1.46]	Asthma [1.34]	Drown [0.25]
Marshall	LRI [0.37]	Congenital [0.59]	Whooping [3.95]	Measles [5.34]	Iron [1.54]	PEM [1.27]	Diarrhea [0.3]	Asthma [1.28]	Drown [0.44]	Neonatal [0.74]
N Mariana	Asthma [2.6]	Congenital [0.67]	Diarrhea [2.86]	LRI [1.49]	Drown [1.61]	Neonatal [0.86]	Dermatitis [0.87]	Measles [112.46]	Road Inj [0.68]	PEM [28.78]
PNG	Diarrhea [0.61]	PEM [1.17]	LRI [0.25]	Congenital [0.7]	Drown [0.83]	Whooping [0.74]	Asthma [1.79]	Measles [0.45]	Meningitis [0.38]	Iron [1.12]
Samoa	Measles [10.66]	LRI [0.27]	Congenital [0.4]	Whooping [3.66]	Asthma [1.39]	Diarrhea [0.38]	Neonatal [0.74]	Vit A [1.11]	Drown [0.35]	Dermatitis [1.05]
Solomon	LRI [0.24]	Congenital [0.47]	Diarrhea [0.17]	PEM [0.36]	Whooping [0.54]	Drown [0.37]	Neonatal [0.8]	Iron [0.87]	Asthma [0.86]	STI [5.54]
Tonga	LRI [0.59]	Asthma [2.22]	Congenital [0.45]	Drown [0.87]	Meningitis [0.93]	Measles [16.69]	Neonatal [0.8]	Diarrhea [0.72]	PEM [3.96]	Road Inj [0.69]
Vanuatu	LRI [0.36]	Congenital [0.55]	Diarrhea [0.3]	Whooping [1.2]	PEM [0.71]	Drown [0.46]	Asthma [1.2]	Neonatal [0.81]	Iron [0.86]	Road Inj [0.39]
**Southeast Asia**	LRI [0.91]	Diarrhea [1.97]	Congenital [0.58]	Drown [1.08]	Measles [20.81]	Asthma [1.67]	Dengue [77.67]	Neonatal [0.64]	Meningitis [0.59]	PEM [3.93]
Cambodia	LRI [0.41]	Drown [0.8]	Congenital [0.34]	Iron [1.26]	Diarrhea [0.15]	Mech [2.59]	Malaria [25.66]	Neonatal [0.56]	Dermatitis [1.05]	Asthma [0.63]
Indonesia	Diarrhea [2.76]	Measles [59.69]	LRI [0.58]	Dengue [153.44]	Congenital [0.35]	Drown [0.74]	PEM [6.86]	Asthma [1.32]	Neonatal [0.63]	HIV [4.65]
Laos	LRI [0.82]	Diarrhea [1.16]	Drown [0.91]	Congenital [0.57]	Whooping [1.49]	Asthma [1.44]	Road Inj [0.69]	HIV [3.13]	Meningitis [0.35]	Measles [1.23]
Malaysia	HIV [50.94]	Neonatal [0.73]	Dermatitis [0.93]	Diarrhea [1.92]	Congenital [0.32]	Asthma [1.12]	PEM [28.16]	Dengue [821.44]	Urticaria [1.02]	Skin Viral [0.94]
Maldives	Congenital [0.38]	Dengue [86.15]	Neonatal [0.56]	Dermatitis [0.93]	Asthma [1.13]	Drown [0.52]	Diarrhea [0.44]	LRI [0.14]	PEM [2.74]	Vit A [0.81]
Mauritius	Congenital [0.63]	Asthma [1.84]	Diarrhea [1.76]	Neonatal [0.73]	Dermatitis [0.87]	Iron [4.14]	LRI [0.55]	PEM [15.17]	Epilepsy [1.23]	Leukemia [0.76]
Myanmar	LRI [0.57]	Drown [1.5]	Congenital [0.83]	Diarrhea [0.76]	Whooping [2.77]	Meningitis [0.47]	Asthma [1.39]	Neonatal [0.66]	Typh + Paratyph [38.13]	Malaria [202.51]
Philippines	LRI [1.51]	Congenital [0.93]	Diarrhea [1.97]	Asthma [3.13]	Drown [0.94]	Dengue [77.65]	Meningitis [0.82]	Neonatal [0.73]	PEM [3.29]	Leukemia [1.35]
Sri Lanka	Congenital [0.48]	Neonatal [0.64]	Dermatitis [0.9]	Asthma [1.09]	Diarrhea [0.58]	Typh + Paratyph [221.87]	LRI [0.2]	PEM [4.5]	Vit A [0.87]	Epilepsy [0.88]
Seychelles	LRI [0.89]	Congenital [0.47]	Drown [0.91]	Neonatal [0.68]	Dermatitis [0.9]	Asthma [1.16]	Diarrhea [0.86]	Meningitis [0.54]	Leukemia [0.59]	Urticaria [0.91]
Thailand	Drown [1.27]	Congenital [0.5]	LRI [0.47]	Dermatitis [0.98]	Neonatal [0.58]	Road Inj [0.66]	Asthma [1.16]	HIV [5.74]	Typh + Paratyph [328.16]	Diarrhea [0.74]
Timor-Leste	LRI [0.58]	Diarrhea [0.53]	HIV [7.14]	Congenital [0.57]	Drown [0.63]	Iron [1.92]	PEM [0.73]	Asthma [1.11]	Whooping [0.82]	Vit A [0.94]
Vietnam	Drown [1.33]	Congenital [0.43]	LRI [0.25]	Neonatal [0.49]	Whooping [3.18]	Dermatitis [0.88]	Asthma [0.76]	Diarrhea [0.3]	Encepha [0.94]	Iron [0.82]
**Sub-Saharan Africa**	Malaria [225.14]	Diarrhea [1.34]	LRI [0.91]	PEM [1.95]	Meningitis [1.04]	Congenital [0.89]	Measles [1.35]	Whooping [1.29]	HIV [3.84]	iNTS [6.66]
**Central Sub-Saharan Africa**	Malaria [421.1]	LRI [0.88]	Diarrhea [1.18]	PEM [2.34]	Congenital [1.36]	Road Inj [2.3]	Drown [1.25]	Whooping [1.94]	TB [2.74]	Measles [1.18]
Angola	Diarrhea [1.49]	Malaria [152.63]	LRI [0.6]	PEM [2.34]	Measles [2.28]	Congenital [0.99]	Road Inj [1.78]	Drown [1.08]	Meningitis [0.51]	Iron [2.04]
C African Rep	Diarrhea [1.57]	Malaria [11.68]	LRI [0.84]	PEM [1.48]	Road Inj [4.59]	Whooping [1.93]	Congenital [1.18]	TB [2.26]	Measles [0.55]	Drown [1.36]
Congo	Malaria [6093.11]	Diarrhea [3.73]	HIV [25.2]	LRI [0.97]	Congenital [1.34]	PEM [6.15]	Road Inj [2.13]	Drown [1.36]	Measles [12.59]	Whooping [5.1]
Congo DR	Malaria [29.76]	LRI [0.6]	Diarrhea [0.43]	Congenital [1.15]	PEM [0.93]	Road Inj [1.82]	Whooping [1.15]	Drown [0.99]	TB [1.43]	Meningitis [0.41]
Eq Guinea	Malaria [27791.16]	HIV [21.75]	Diarrhea [2.51]	Measles [42.63]	Congenital [0.96]	LRI [0.68]	Road Inj [1.52]	Drown [1.14]	PEM [6.48]	Neonatal [0.94]

eFigure 7c. Leading ten causes of DALYs with the ratio of observed DALYs to DALYs expected on the basis of Socio-Demographic Index alone in 2017, 1-4 years, both sexes combined. The top ten causes contributing to DALYs are listed globally, by socio-demographic quintile, and then by GBD superregion, region, country, and subnationally where modeled. For each cell, the ratio of observed DALYs to DALYs expected on the basis of socio-demographic index (SDI) alone are listed. Abbreviations: DALY=disability-adjusted life year, GBD=Global Burden of Disease.

Values shown in brackets represent the ratio of observed DALYs to predicted DALYs on the basis of Socio-Demographic Index (SDI), rounded to two (2) digits. Color ranges (shown below) were calculated to place a roughly equal number of cells into each bin.

COLOR KEY:	[0.0-0.5]	(0.5-0.72]	(0.72-0.86]	(0.86-0.97]	(0.97-1.1]	(1.1-1.23]	(1.23-1.41]	(1.41-2.15]	2.15+

	1	2	3	4	5	6	7	8	9	10
Gabon	Malaria [38826.8]	Diarrhea [3.4]	Congenital [1.13]	LRI [1.03]	Road Inj [2.25]	Measles [50.37]	Drown [1.52]	Neonatal [1.35]	PEM [11.18]	Iron [4.2]
Eastern Sub-Saharan Africa	Diarrhea [0.54]	LRI [0.4]	Malaria [10.27]	PEM [0.95]	Congenital [0.73]	Measles [0.51]	Whooping [0.84]	HIV [3.65]	Meningitis [0.42]	TB [0.74]
Burundi	Malaria [4.64]	Diarrhea [0.38]	LRI [0.35]	PEM [0.84]	Congenital [1.01]	Measles [0.33]	TB [0.75]	Meningitis [0.34]	Drown [0.32]	Whooping [0.3]
Comoros	Diarrhea [0.39]	LRI [0.23]	Congenital [0.51]	PEM [0.63]	Whooping [0.88]	Measles [0.5]	Neonatal [1.11]	Meningitis [0.21]	Vit A [0.72]	Iron [0.59]
Djibouti	LRI [0.5]	Diarrhea [0.63]	PEM [2.13]	Congenital [0.8]	Whooping [1.83]	Neonatal [1.11]	Meningitis [0.37]	Iron [1.28]	TB [1.1]	Drown [0.28]
Eritrea	Diarrhea [1.0]	LRI [0.69]	PEM [1.83]	Congenital [1.02]	Meningitis [0.58]	TB [1.66]	Oth Un Inf [3.44]	Neonatal [1.26]	Drown [0.42]	Iron [1.15]
Ethiopia	Diarrhea [0.42]	LRI [0.26]	PEM [0.5]	Whooping [0.82]	Measles [0.26]	Congenital [0.35]	Meningitis [0.31]	Iron [0.9]	TB [0.39]	Neonatal [0.94]
Kenya	Diarrhea [1.32]	LRI [0.5]	PEM [1.75]	Congenital [0.56]	iNTS [10.78]	HIV [4.87]	Malaria [100.85]	Neonatal [1.14]	Whooping [1.17]	Meningitis [0.39]
Baringo	Diarrhea [0.71]	LRI [0.26]	PEM [0.76]	iNTS [6.47]	Congenital [0.36]	Leish [18.44]	Neonatal [1.04]	Vit A [1.07]	Measles [0.31]	Meningitis [0.2]
Bomet	Diarrhea [0.42]	HIV [5.84]	Neonatal [1.19]	iNTS [5.6]	LRI [0.09]	Congenital [0.19]	Vit A [0.79]	PEM [0.36]	Dermatitis [1.03]	Iron [0.45]
Bungoma	Diarrhea [1.21]	LRI [0.44]	PEM [1.29]	iNTS [11.22]	Malaria [65.67]	Congenital [0.55]	Whooping [1.29]	Meningitis [0.36]	Neonatal [1.12]	Measles [0.64]
Busia	Diarrhea [0.66]	Malaria [67.8]	LRI [0.25]	iNTS [7.73]	PEM [0.59]	Congenital [0.37]	Whooping [0.7]	Neonatal [1.06]	Meningitis [0.21]	Measles [0.31]
Elgeyo-Marakwet	Diarrhea [0.99]	LRI [0.26]	iNTS [9.33]	PEM [1.06]	Congenital [0.36]	Neonatal [1.2]	Whooping [0.95]	Measles [0.85]	Meningitis [0.21]	Dermatitis [1.03]
Embu	Diarrhea [2.13]	LRI [0.53]	PEM [2.43]	Congenital [0.62]	iNTS [12.59]	Neonatal [1.26]	Meningitis [0.45]	Whooping [1.7]	Measles [1.98]	HIV [2.74]
Garissa	Diarrhea [0.15]	iNTS [2.9]	LRI [0.07]	HIV [1.95]	Neonatal [1.05]	Congenital [0.15]	Measles [0.07]	PEM [0.08]	Vit A [0.57]	Whooping [0.16]
HomaBay	LRI [0.87]	Diarrhea [1.02]	HIV [17.89]	PEM [1.68]	Congenital [1.03]	Malaria [29.57]	iNTS [11.24]	Meningitis [0.7]	TB [1.49]	Whooping [0.67]
Isiolo	LRI [0.35]	Diarrhea [0.28]	PEM [0.6]	Congenital [0.46]	Whooping [0.72]	iNTS [4.37]	Measles [0.27]	Conflict Terror [146.44]	Meningitis [0.27]	Iron [1.08]
Kajiado	Diarrhea [0.92]	LRI [0.31]	iNTS [9.8]	Neonatal [1.3]	Whooping [2.31]	Congenital [0.4]	Iron [2.07]	Vit A [1.93]	PEM [1.27]	Meningitis [0.3]
Kakamega	Diarrhea [1.19]	LRI [0.58]	PEM [1.5]	Malaria [44.11]	Congenital [0.69]	iNTS [8.96]	Meningitis [0.46]	Whooping [0.88]	HIV [2.3]	Neonatal [1.01]
Kericho	Diarrhea [1.3]	LRI [0.39]	iNTS [16.94]	PEM [1.49]	Congenital [0.5]	HIV [4.29]	Neonatal [1.31]	Whooping [1.24]	Meningitis [0.31]	Measles [0.98]
Kiambu	Diarrhea [2.69]	LRI [0.86]	Congenital [0.85]	PEM [5.09]	iNTS [17.82]	Whooping [6.71]	Neonatal [1.4]	Meningitis [0.8]	Measles [6.38]	Other MN [2.3]
Kilifi	Diarrhea [1.32]	LRI [0.4]	PEM [1.26]	Congenital [0.51]	HIV [3.06]	Whooping [0.8]	Malaria [24.15]	Neonatal [1.06]	Meningitis [0.31]	Measles [0.49]
Kirinyaga	Diarrhea [2.98]	LRI [1.1]	Congenital [1.19]	PEM [4.33]	Meningitis [0.9]	iNTS [13.89]	Neonatal [1.33]	Malaria [248.3]	Measles [2.11]	TB [2.2]
Kisii	Diarrhea [1.45]	LRI [0.3]	iNTS [13.66]	PEM [1.3]	Congenital [0.4]	Neonatal [1.07]	Vit A [0.95]	Meningitis [0.22]	Measles [0.9]	Dermatitis [1.03]
Kisumu	LRI [0.84]	HIV [15.01]	iNTS [30.19]	Diarrhea [0.95]	Congenital [1.22]	Malaria [313.76]	PEM [2.58]	Meningitis [0.79]	Whooping [2.29]	TB [2.24]
Kitui	Diarrhea [0.92]	LRI [0.23]	iNTS [8.17]	PEM [0.78]	HIV [3.58]	Congenital [0.33]	Whooping [0.81]	Neonatal [1.18]	Measles [0.43]	Meningitis [0.19]
Kwale	Diarrhea [0.41]	iNTS [7.78]	LRI [0.13]	Malaria [27.03]	Neonatal [1.07]	PEM [0.43]	Congenital [0.24]	HIV [1.62]	Whooping [0.43]	Vit A [0.77]
Laikipia	Diarrhea [1.72]	HIV [10.55]	LRI [0.32]	Neonatal [1.34]	Congenital [0.41]	iNTS [9.87]	PEM [1.74]	Whooping [1.41]	Meningitis [0.28]	Dermatitis [1.02]
Lamu	Diarrhea [0.91]	LRI [0.64]	Conflict Terror [1198.31]	PEM [1.83]	Congenital [0.88]	iNTS [13.17]	Whooping [1.85]	Meningitis [0.61]	Malaria [30.72]	Measles [0.76]
Machakos	Diarrhea [2.46]	LRI [0.43]	iNTS [17.56]	HIV [8.19]	PEM [1.83]	Congenital [0.51]	Neonatal [1.26]	Whooping [1.54]	Measles [1.65]	Meningitis [0.37]

eFigure 7c. Leading ten causes of DALYs with the ratio of observed DALYs to DALYs expected on the basis of Socio-Demographic Index alone in 2017, 1-4 years, both sexes combined. The top ten causes contributing to DALYs are listed globally, by socio-demographic quintile, and then by GBD superregion, region, country, and subnationally where modeled. For each cell, the ratio of observed DALYs to DALYs expected on the basis of socio-demographic index (SDI) alone are listed. Abbreviations: DALY=disability-adjusted life year, GBD=Global Burden of Disease.

Values shown in brackets represent the ratio of observed DALYs to predicted DALYs on the basis of Socio-Demographic Index (SDI), rounded to two (2) digits. Color ranges (shown below) were calculated to place a roughly equal number of cells into each bin.

COLOR KEY:	[0.0-0.5]	(0.5-0.72]	(0.72-0.86]	(0.86-0.97]	(0.97-1.1]	(1.1-1.23]	(1.23-1.41]	(1.41-2.15]	2.15+

	1	2	3	4	5	6	7	8	9	10
Makueni	HIV [4.89]	Diarrhea [0.27]	Neonatal [1.11]	iNTS [2.85]	Congenital [0.15]	Dermatitis [1.04]	LRI [0.04]	Vit A [0.44]	Typh + Paratyph [4.15]	Whooping [0.22]
Mandera	Diarrhea [0.31]	LRI [0.27]	PEM [0.36]	Measles [0.12]	iNTS [2.16]	HIV [2.54]	Congenital [0.25]	Meningitis [0.19]	Vit A [0.91]	Whooping [0.22]
Marsabit	Diarrhea [0.33]	LRI [0.17]	PEM [0.4]	HIV [3.83]	iNTS [2.69]	Measles [0.15]	Congenital [0.22]	Neonatal [1.07]	Whooping [0.29]	Meningitis [0.13]
Meru	Diarrhea [2.64]	LRI [0.92]	PEM [3.51]	Congenital [0.99]	HIV [9.61]	iNTS [11.65]	Meningitis [0.72]	Whooping [1.87]	Neonatal [1.29]	Malaria [124.06]
Migori	Diarrhea [1.22]	LRI [0.77]	HIV [13.92]	PEM [1.54]	Malaria [23.53]	Congenital [0.88]	iNTS [8.69]	Meningitis [0.59]	TB [1.19]	Measles [0.39]
Mombasa	Diarrhea [3.01]	LRI [0.91]	iNTS [33.22]	Congenital [0.94]	PEM [5.6]	Whooping [5.16]	Meningitis [0.83]	Neonatal [1.22]	Measles [4.24]	Malaria [395.23]
Murang’a	LRI [0.63]	Congenital [0.93]	Whooping [4.34]	iNTS [15.86]	Meningitis [0.75]	Diarrhea [0.47]	HIV [5.5]	PEM [1.77]	Neonatal [1.31]	Measles [1.95]
Nairobi	LRI [1.23]	Diarrhea [2.97]	Congenital [0.78]	Neonatal [1.42]	PEM [17.08]	iNTS [17.46]	Meningitis [1.13]	Dermatitis [1.15]	Other MN [2.28]	Whooping [10.62]
Nakuru	Diarrhea [1.64]	LRI [0.53]	PEM [2.66]	Congenital [0.62]	Neonatal [1.38]	iNTS [9.22]	Meningitis [0.44]	Whooping [1.57]	Typh + Paratyph [27.56]	Dermatitis [1.02]
Nandi	Diarrhea [0.75]	iNTS [10.87]	LRI [0.19]	Neonatal [1.23]	HIV [3.21]	Congenital [0.3]	PEM [0.71]	Dermatitis [1.03]	Measles [0.56]	Malaria [36.85]
Narok	Diarrhea [0.15]	Neonatal [1.15]	Vit A [1.05]	LRI [0.05]	iNTS [2.03]	Congenital [0.14]	Iron [0.49]	PEM [0.11]	Dermatitis [1.06]	Measles [0.09]
Nyamira	Diarrhea [1.05]	iNTS [11.94]	Neonatal [1.19]	LRI [0.19]	Congenital [0.29]	PEM [1.0]	HIV [2.48]	Dermatitis [1.03]	Typh + Paratyph [17.53]	Meningitis [0.17]
Nyandarua	Diarrhea [1.71]	LRI [0.52]	PEM [2.39]	Whooping [3.26]	Congenital [0.58]	Neonatal [1.35]	iNTS [8.28]	Meningitis [0.45]	Measles [1.45]	Dermatitis [1.02]
Nyeri	LRI [0.85]	Diarrhea [1.69]	Congenital [0.92]	PEM [4.27]	Whooping [6.43]	Meningitis [0.87]	iNTS [12.55]	Neonatal [1.35]	HIV [3.62]	Measles [3.18]
Samburu	Diarrhea [0.2]	HIV [5.18]	LRI [0.07]	PEM [0.15]	iNTS [1.86]	Measles [0.09]	Neonatal [1.06]	Whooping [0.23]	Vit A [0.83]	Congenital [0.13]
Siaya	Malaria [207.06]	Diarrhea [1.1]	LRI [0.59]	Congenital [0.77]	iNTS [9.86]	PEM [1.0]	HIV [5.18]	Meningitis [0.44]	Neonatal [0.88]	Vit A [1.13]
TaitaTaveta	Diarrhea [1.89]	LRI [0.82]	PEM [3.8]	HIV [11.26]	Congenital [0.97]	iNTS [21.21]	Whooping [2.8]	Meningitis [0.73]	Neonatal [1.25]	Malaria [197.48]
TanaRiver	LRI [0.58]	Diarrhea [0.6]	PEM [1.31]	HIV [8.74]	iNTS [8.37]	Congenital [0.67]	Whooping [0.8]	Measles [0.37]	Meningitis [0.45]	TB [0.87]
TharakaNithi	Diarrhea [5.14]	LRI [1.22]	PEM [6.38]	Congenital [1.22]	iNTS [22.6]	Meningitis [0.94]	Whooping [2.64]	Measles [2.88]	Neonatal [1.18]	Malaria [201.94]
TransNzoia	Diarrhea [1.66]	LRI [0.56]	HIV [10.99]	PEM [1.87]	iNTS [11.65]	Congenital [0.6]	Whooping [1.48]	Meningitis [0.45]	Neonatal [1.2]	Malaria [59.27]
Turkana	Diarrhea [0.31]	LRI [0.18]	PEM [0.35]	HIV [4.04]	iNTS [1.81]	Congenital [0.25]	Malaria [0.26]	Measles [0.08]	Meningitis [0.16]	Whooping [0.23]
UasinGishu	Diarrhea [2.1]	LRI [0.42]	Congenital [0.5]	iNTS [10.96]	PEM [1.94]	Neonatal [1.23]	Whooping [1.7]	Dermatitis [1.44]	Meningitis [0.34]	Vit A [0.92]
Vihiga	Diarrhea [0.43]	LRI [0.18]	Malaria [54.98]	Neonatal [1.09]	Congenital [0.3]	PEM [0.52]	iNTS [4.35]	Vit A [1.05]	Typh + Paratyph [7.92]	Meningitis [0.17]
Wajir	Diarrhea [0.13]	Measles [0.1]	iNTS [1.78]	LRI [0.06]	Whooping [0.23]	PEM [0.07]	Neonatal [0.98]	Congenital [0.13]	Vit A [0.63]	Tetanus [1.23]
WestPokot	Diarrhea [0.63]	LRI [0.18]	PEM [0.48]	iNTS [4.57]	Leish [10.23]	Whooping [0.43]	Neonatal [1.1]	Measles [0.19]	Congenital [0.23]	Vit A [0.96]
Madagascar	Diarrhea [0.77]	PEM [1.57]	LRI [0.38]	Measles [0.33]	Congenital [0.55]	Malaria [1.3]	Whooping [0.45]	Meningitis [0.27]	Neonatal [1.24]	Oth Un Inf [2.25]
Malawi	Malaria [5.77]	Diarrhea [0.3]	LRI [0.29]	HIV [9.17]	Congenital [0.81]	PEM [0.61]	Measles [0.38]	Meningitis [0.43]	Whooping [0.61]	TB [0.53]
Mozambique	Malaria [6.78]	HIV [16.11]	Diarrhea [0.22]	Congenital [0.74]	LRI [0.17]	PEM [0.31]	Meningitis [0.25]	TB [0.54]	iNTS [2.11]	Measles [0.12]
Rwanda	LRI [0.41]	Diarrhea [0.43]	Congenital [0.95]	PEM [1.04]	Malaria [14.13]	Meningitis [0.45]	TB [0.76]	Road Inj [0.52]	Neonatal [1.01]	Drown [0.3]

eFigure 7c. Leading ten causes of DALYs with the ratio of observed DALYs to DALYs expected on the basis of Socio-Demographic Index alone in 2017, 1-4 years, both sexes combined. The top ten causes contributing to DALYs are listed globally, by socio-demographic quintile, and then by GBD superregion, region, country, and subnationally where modeled. For each cell, the ratio of observed DALYs to DALYs expected on the basis of socio-demographic index (SDI) alone are listed. Abbreviations: DALY=disability-adjusted life year, GBD=Global Burden of Disease.

Values shown in brackets represent the ratio of observed DALYs to predicted DALYs on the basis of Socio-Demographic Index (SDI), rounded to two (2) digits. Color ranges (shown below) were calculated to place a roughly equal number of cells into each bin.

COLOR KEY:									
[0.0-0.5]	(0.5-0.72]	(0.72-0.86]	(0.86-0.97]	(0.97-1.1]	(1.1-1.23]	(1.23-1.41]	(1.41-2.15]	2.15+	

	1	2	3	4	5	6	7	8	9	10
Somalia	Measles [0.61]	Whooping [2.16]	Diarrhea [0.25]	LRI [0.28]	PEM [0.37]	Conflict Terror [51.23]	Meningitis [0.35]	Congenital [0.33]	TB [0.41]	Road Inj [0.43]
S Sudan	Diarrhea [0.68]	LRI [0.71]	PEM [0.77]	Malaria [1.12]	Measles [0.36]	Meningitis [0.71]	Conflict Terror [106.82]	Leish [6.56]	Congenital [0.47]	TB [0.74]
Tanzania	LRI [0.59]	Malaria [27.85]	Congenital [1.44]	PEM [1.09]	Diarrhea [0.26]	Meningitis [0.39]	Whooping [0.66]	TB [1.13]	Drown [0.39]	Neonatal [1.06]
Uganda	Malaria [30.59]	Diarrhea [0.29]	LRI [0.24]	Congenital [0.73]	PEM [0.56]	Meningitis [0.54]	HIV [3.85]	Whooping [0.65]	Measles [0.32]	TB [0.63]
Zambia	Diarrhea [1.14]	HIV [17.17]	LRI [0.66]	Malaria [201.61]	PEM [2.93]	Congenital [1.32]	Meningitis [0.75]	TB [1.97]	Drown [0.5]	Iron [1.35]
**Southern Sub-Saharan Africa**	Diarrhea [5.74]	LRI [1.45]	PEM [25.43]	HIV [15.85]	TB [15.42]	Congenital [0.46]	Neonatal [1.03]	Measles [24.03]	Road Inj [0.98]	Dermatitis [1.19]
Botswana	Diarrhea [8.77]	LRI [1.27]	PEM [23.06]	Neonatal [1.14]	Congenital [0.36]	Road Inj [0.83]	HIV [6.98]	TB [11.34]	Dermatitis [1.17]	Vit A [1.31]
Lesotho	Diarrhea [2.05]	HIV [14.02]	LRI [0.56]	PEM [2.52]	TB [3.48]	Road Inj [0.71]	Congenital [0.3]	Measles [0.95]	Neonatal [0.82]	Iron [1.15]
Namibia	Diarrhea [5.94]	HIV [28.7]	LRI [1.13]	PEM [12.92]	TB [10.11]	Neonatal [1.04]	Road Inj [0.93]	Congenital [0.39]	Iron [2.52]	Dermatitis [1.24]
S Africa	Diarrhea [6.37]	LRI [1.43]	HIV [22.39]	PEM [22.01]	Road Inj [1.37]	Measles [55.41]	Neonatal [1.13]	Congenital [0.51]	TB [16.34]	Violence [3.83]
Swaziland	Diarrhea [4.02]	LRI [0.71]	HIV [13.28]	PEM [5.81]	TB [5.3]	Road Inj [0.8]	Congenital [0.33]	Neonatal [0.86]	Dermatitis [1.3]	Meningitis [0.33]
Zimbabwe	PEM [3.57]	LRI [0.57]	Diarrhea [0.79]	TB [3.0]	Malaria [45.85]	HIV [2.9]	Whooping [0.75]	Congenital [0.29]	Meningitis [0.32]	Measles [0.49]
**Western Sub-Saharan Africa**	Malaria [307.59]	Diarrhea [1.8]	LRI [1.28]	Meningitis [1.67]	PEM [2.04]	Measles [1.54]	Congenital [0.84]	iNTS [11.58]	Whooping [1.16]	TB [1.96]
Benin	Malaria [32.59]	Diarrhea [0.69]	LRI [0.53]	PEM [0.91]	Measles [0.6]	Congenital [0.7]	iNTS [7.14]	Meningitis [0.61]	Whooping [0.83]	Road Inj [1.14]
Burkina Faso	Malaria [7.23]	LRI [0.52]	Diarrhea [0.35]	iNTS [7.77]	PEM [0.59]	Meningitis [0.84]	Congenital [0.92]	Hemog [4.46]	Drown [0.55]	Iron [1.82]
Cameroon	Malaria [935.54]	Diarrhea [1.87]	LRI [0.71]	PEM [1.65]	Congenital [0.72]	Meningitis [0.64]	iNTS [8.68]	HIV [3.43]	Whooping [0.95]	Drown [0.42]
Cape Verde	Neonatal [1.45]	LRI [0.21]	Congenital [0.37]	Drown [0.49]	Iron [1.44]	Diarrhea [0.23]	Violence [1.87]	iNTS [4.08]	Dermatitis [0.83]	Epilepsy [1.08]
Chad	Diarrhea [1.17]	LRI [0.73]	Malaria [1.07]	PEM [0.69]	Meningitis [0.74]	Measles [0.19]	Congenital [0.49]	Whooping [0.58]	TB [0.63]	iNTS [2.11]
Cote d'Ivoire	Malaria [67.82]	Diarrhea [0.97]	LRI [0.59]	PEM [1.03]	Congenital [0.77]	iNTS [9.25]	Meningitis [0.54]	HIV [3.66]	Whooping [0.66]	Drown [0.49]
Gambia	Diarrhea [0.52]	LRI [0.38]	Hemog [10.1]	PEM [0.95]	Congenital [0.52]	Meningitis [0.5]	Whooping [0.79]	iNTS [4.86]	Iron [1.77]	Neonatal [1.28]
Ghana	Malaria [2769.22]	Diarrhea [1.55]	LRI [0.54]	PEM [3.2]	Congenital [0.67]	Meningitis [0.77]	Hemog [6.62]	iNTS [9.27]	Neonatal [1.08]	Iron [1.84]
Guinea	Malaria [10.38]	LRI [0.67]	Diarrhea [0.35]	PEM [0.85]	iNTS [6.66]	Meningitis [0.71]	Congenital [0.73]	Whooping [1.03]	Measles [0.32]	TB [0.78]
Guinea-Bissau	Diarrhea [0.76]	Measles [1.21]	LRI [0.36]	Hemog [8.32]	PEM [0.64]	Congenital [0.64]	Meningitis [0.6]	Whooping [0.84]	Malaria [1.54]	Iron [1.35]
Liberia	Malaria [5.47]	Diarrhea [0.42]	Measles [0.73]	LRI [0.26]	Whooping [0.88]	PEM [0.41]	Congenital [0.45]	iNTS [3.66]	Meningitis [0.36]	Hemog [2.65]
Mali	Malaria [4.58]	PEM [1.11]	Diarrhea [0.38]	LRI [0.4]	iNTS [8.54]	Congenital [0.97]	Meningitis [0.76]	Whooping [0.85]	Measles [0.17]	Drown [0.76]
Mauritania	Diarrhea [0.85]	PEM [2.0]	LRI [0.32]	Iron [1.83]	Whooping [1.01]	Congenital [0.33]	Neonatal [1.14]	Malaria [31.54]	Vit A [1.32]	Meningitis [0.28]
Niger	Malaria [1.47]	Diarrhea [0.51]	LRI [0.51]	Meningitis [1.35]	PEM [0.21]	Measles [0.11]	Congenital [0.59]	iNTS [2.46]	Whooping [0.53]	TB [0.36]
Nigeria	Malaria [1941.47]	LRI [2.36]	Diarrhea [3.54]	Meningitis [2.98]	Measles [6.15]	PEM [3.84]	Congenital [0.9]	TB [4.54]	iNTS [14.2]	HIV [7.04]
Sao Tome Principe	Other MN [5.13]	Diarrhea [0.27]	LRI [0.17]	Neonatal [1.28]	Congenital [0.33]	PEM [0.62]	iNTS [5.03]	Whooping [0.86]	Oth NTD [3.49]	Vit A [0.83]
Senegal	Diarrhea [0.57]	LRI [0.3]	Congenital [0.48]	Malaria [3.03]	Meningitis [0.41]	PEM [0.35]	Hemog [3.08]	Iron [1.26]	Whooping [0.41]	Neonatal [1.17]

eFigure 7c. Leading ten causes of DALYs with the ratio of observed DALYs to DALYs expected on the basis of Socio-Demographic Index alone in 2017, 1-4 years, both sexes combined. The top ten causes contributing to DALYs are listed globally, by socio-demographic quintile, and then by GBD superregion, region, country, and subnationally where modeled. For each cell, the ratio of observed DALYs to DALYs expected on the basis of socio-demographic index (SDI) alone are listed. Abbreviations: DALY=disability-adjusted life year, GBD=Global Burden of Disease.

Values shown in brackets represent the ratio of observed DALYs to predicted DALYs on the basis of Socio-Demographic Index (SDI), rounded to two (2) digits. Color ranges (shown below) were calculated to place a roughly equal number of cells into each bin.

COLOR KEY:	[0.0-0.5]	(0.5-0.72]	(0.72-0.86]	(0.86-0.97]	(0.97-1.1]	(1.1-1.23]	(1.23-1.41]	(1.41-2.15]	2.15+

	1	2	3	4	5	6	7	8	9	10
Sierra Leone	**Malaria** [35.87]	**LRI** [0.62]	**Diarrhea** [0.51]	**PEM** [1.06]	**Congenital** [0.94]	**Meningitis** [0.75]	**iNTS** [6.34]	**Drown** [0.54]	**Iron** [1.6]	**TB** [0.83]
Togo	**Malaria** [125.31]	**Diarrhea** [0.74]	**LRI** [0.38]	**iNTS** [9.77]	**PEM** [0.75]	**Congenital** [0.53]	**Hemog** [4.61]	**Meningitis** [0.34]	**Whooping** [0.61]	**Iron** [1.35]

eFigure 7d. Leading ten causes of DALYs with the ratio of observed DALYs to DALYs expected on the basis of Socio-Demographic Index alone in 2017, 5-9 years, both sexes combined. The top ten causes contributing to DALYs are listed globally, by socio-demographic quintile, and then by GBD superregion, region, country, and subnationally where modeled. For each cell, the ratio of observed DALYs to DALYs expected on the basis of socio-demographic index (SDI) alone are listed. Abbreviations: DALY=disability-adjusted life year, GBD=Global Burden of Disease.

Values shown in brackets represent the ratio of observed DALYs to predicted DALYs on the basis of Socio-Demographic Index (SDI), rounded to two (2) digits. Color ranges (shown below) were calculated to place a roughly equal number of cells into each bin.

COLOR KEY:	[0.0-0.62]	(0.62-0.79]	(0.79-0.89]	(0.89-0.97]	(0.97-1.07]	(1.07-1.26]	(1.26-1.44]	(1.44-2.32]	2.32+

	1	2	3	4	5	6	7	8	9	10
Global	Iron [2.11]	Vit A [1.74]	Diarrhea [2.82]	Malaria [964.64]	Neonatal [1.07]	Typh + Paratyph [205.52]	Road Inj [0.59]	Congenital [0.73]	LRI [1.16]	Drown [0.8]
Low SDI	Iron [1.24]	Malaria [8.63]	Diarrhea [0.78]	Vit A [1.04]	Typh + Paratyph [3.06]	HIV [11.63]	LRI [0.45]	Road Inj [0.36]	Neonatal [1.42]	Drown [0.46]
Low-middle SDI	Iron [1.77]	Malaria [158.24]	Vit A [1.32]	Diarrhea [1.29]	Road Inj [0.5]	Typh + Paratyph [16.82]	LRI [0.6]	Neonatal [1.13]	Congenital [0.69]	Drown [0.46]
Middle SDI	Neonatal [1.18]	Drown [1.05]	Road Inj [0.56]	Iron [1.07]	Congenital [0.71]	Vit A [1.14]	Asthma [0.88]	LRI [0.82]	Dermatitis [0.69]	Diarrhea [1.34]
High-middle SDI	Neonatal [1.31]	Road Inj [1.73]	Congenital [1.17]	Drown [3.22]	Asthma [1.0]	Dermatitis [0.59]	Iron [3.49]	Skin Viral [0.94]	Leukemia [1.41]	Conduct [0.86]
High SDI	Dermatitis [1.18]	Neonatal [0.88]	Asthma [0.96]	Congenital [0.56]	Anxiety [1.16]	Skin Viral [1.01]	Conduct [1.0]	URI [1.15]	Road Inj [0.29]	Falls [0.96]
Central Europe, Eastern Europe, and Central Asia	Neonatal [1.35]	Iron [2.39]	Congenital [0.89]	LRI [2.97]	Road Inj [0.68]	Drown [1.34]	Dermatitis [0.59]	Falls [1.49]	Vit A [1.28]	Conduct [0.92]
Central Asia	LRI [2.95]	Iron [2.18]	Vit A [1.53]	Road Inj [0.64]	Neonatal [0.94]	Drown [1.08]	Congenital [0.75]	Epilepsy [1.47]	Leukemia [0.77]	Dermatitis [0.52]
Armenia	Iron [3.52]	Neonatal [0.96]	Congenital [0.85]	LRI [1.25]	Dermatitis [0.52]	Road Inj [0.33]	Diarrhea [1.31]	Conduct [0.91]	Epilepsy [1.0]	Brain C [1.12]
Azerbaijan	LRI [5.12]	Congenital [1.02]	Leukemia [1.7]	Neonatal [0.92]	Epilepsy [2.03]	Drown [0.95]	Iron [1.07]	Road Inj [0.48]	Vit A [0.88]	Fire [2.24]
Georgia	Neonatal [0.96]	Congenital [0.67]	Road Inj [0.5]	Iron [1.08]	Vit A [1.07]	Leukemia [0.81]	Epilepsy [1.11]	Asthma [0.57]	Conduct [0.91]	Dermatitis [0.47]
Kazakhstan	Vit A [3.06]	Road Inj [1.06]	Neonatal [1.02]	Congenital [0.98]	Iron [1.74]	Drown [1.54]	Epilepsy [1.36]	LRI [1.36]	Dermatitis [0.47]	Conduct [0.9]
Kyrgyzstan	Iron [1.79]	Vit A [1.2]	Neonatal [1.01]	Road Inj [0.45]	Congenital [0.6]	LRI [0.66]	Drown [0.53]	Epilepsy [1.07]	Dermatitis [0.56]	Asthma [0.54]
Mongolia	Road Inj [1.14]	Vit A [2.03]	Iron [1.5]	Congenital [0.85]	Neonatal [0.98]	LRI [1.34]	Drown [0.82]	Falls [1.7]	Epilepsy [1.19]	Fire [1.79]
Tajikistan	LRI [1.43]	Drown [0.91]	Iron [1.02]	Vit A [1.15]	Congenital [0.83]	Diarrhea [0.87]	Neonatal [1.05]	Epilepsy [1.56]	Road Inj [0.31]	Dermatitis [0.58]
Turkmenistan	LRI [2.97]	Iron [1.36]	Congenital [0.78]	Neonatal [0.84]	Vit A [1.29]	Drown [0.84]	Epilepsy [1.3]	Dermatitis [0.52]	Leukemia [0.8]	Road Inj [0.32]
Uzbekistan	LRI [2.71]	Iron [2.48]	Road Inj [0.58]	Drown [0.9]	Neonatal [0.9]	Vit A [0.99]	Congenital [0.51]	Epilepsy [1.33]	Leukemia [0.73]	Dermatitis [0.55]
Central Europe	Neonatal [1.39]	Congenital [0.87]	Falls [1.77]	Dermatitis [0.64]	Vit A [2.77]	Diarrhea [2.68]	Asthma [0.66]	Conduct [0.9]	Iron [1.98]	Road Inj [0.53]
Albania	Neonatal [1.17]	Congenital [0.96]	Vit A [1.65]	Falls [2.04]	Road Inj [0.41]	Epilepsy [1.31]	Brain C [1.5]	Leukemia [0.78]	LRI [0.85]	Iron [0.66]
Bosnia	Neonatal [1.35]	Vit A [1.87]	Congenital [0.69]	Falls [1.96]	Dermatitis [0.58]	Iron [0.83]	Asthma [0.61]	Conduct [0.9]	Epilepsy [1.06]	Diarrhea [1.3]
Bulgaria	Neonatal [1.24]	Congenital [0.99]	Falls [1.88]	Vit A [2.07]	Diarrhea [2.27]	Dermatitis [0.52]	Conduct [0.9]	Road Inj [0.48]	Skin Viral [0.79]	Asthma [0.53]
Croatia	Neonatal [1.51]	Congenital [0.88]	Dermatitis [0.54]	Diarrhea [2.36]	Road Inj [0.62]	Falls [1.17]	Conduct [0.9]	Asthma [0.62]	Vit A [1.96]	Skin Viral [0.8]
Czech	Neonatal [0.93]	Falls [2.0]	Dermatitis [0.66]	Congenital [0.78]	Diarrhea [3.21]	Conduct [0.91]	Skin Viral [0.81]	Asthma [0.53]	Anxiety [0.63]	Urticaria [1.65]
Hungary	Neonatal [1.34]	Diarrhea [4.03]	Dermatitis [0.75]	Congenital [0.89]	Falls [1.73]	Vit A [2.02]	Conduct [0.9]	Skin Viral [0.8]	Asthma [0.51]	Anxiety [0.64]
Macedonia	Neonatal [1.33]	Vit A [2.38]	Iron [1.79]	Congenital [0.73]	Falls [1.91]	Road Inj [0.59]	Dermatitis [0.54]	Diarrhea [1.8]	Asthma [0.64]	Conduct [0.9]
Montenegro	Neonatal [1.31]	Vit A [2.67]	Falls [1.78]	Congenital [0.7]	Dermatitis [0.56]	Road Inj [0.59]	Iron [1.57]	Conduct [0.9]	Asthma [0.58]	Skin Viral [0.79]
Poland	Neonatal [1.55]	Congenital [0.96]	Dermatitis [0.73]	Falls [1.65]	Asthma [0.85]	Diarrhea [2.65]	Vit A [2.68]	Upper Digest [7.33]	Conduct [0.91]	Skin Viral [0.81]
Romania	Neonatal [1.43]	Iron [4.52]	Vit A [3.43]	Congenital [0.77]	Diarrhea [2.93]	Falls [1.87]	Upper Digest [8.96]	Road Inj [0.67]	Dermatitis [0.6]	LRI [2.56]
Serbia	Neonatal [1.72]	Vit A [1.57]	Congenital [0.66]	Falls [1.85]	Dermatitis [0.56]	Conduct [0.91]	Diarrhea [1.58]	Skin Viral [0.8]	Anxiety [0.7]	Asthma [0.44]

eFigure 7d. Leading ten causes of DALYs with the ratio of observed DALYs to DALYs expected on the basis of Socio-Demographic Index alone in 2017, 5-9 years, both sexes combined. The top ten causes contributing to DALYs are listed globally, by socio-demographic quintile, and then by GBD superregion, region, country, and subnationally where modeled. For each cell, the ratio of observed DALYs to DALYs expected on the basis of socio-demographic index (SDI) alone are listed. Abbreviations: DALY=disability-adjusted life year, GBD=Global Burden of Disease.

Values shown in brackets represent the ratio of observed DALYs to predicted DALYs on the basis of Socio-Demographic Index (SDI), rounded to two (2) digits. Color ranges (shown below) were calculated to place a roughly equal number of cells into each bin.

COLOR KEY:	[0.0-0.62]	(0.62-0.79]	(0.79-0.89]	(0.89-0.97]	(0.97-1.07]	(1.07-1.26]	(1.26-1.44]	(1.44-2.32]	2.32+

	1	2	3	4	5	6	7	8	9	10
Slovakia	Neonatal [1.03]	Congenital [0.96]	Falls [1.72]	Dermatitis [0.66]	Diarrhea [3.45]	Vit A [3.42]	Upper Digest [7.57]	Road Inj [0.66]	Conduct [0.91]	Skin Viral [0.8]
Slovenia	Neonatal [1.1]	Falls [2.01]	Congenital [0.89]	Diarrhea [3.16]	Dermatitis [0.54]	Asthma [0.76]	Vit A [2.75]	Conduct [0.92]	Skin Viral [0.81]	Upper Digest [5.87]
**Eastern Europe**	Neonatal [1.62]	Congenital [0.97]	Iron [2.37]	Upper Digest [9.57]	Road Inj [0.67]	Dermatitis [0.62]	Drown [1.53]	Falls [1.52]	Conduct [0.94]	Asthma [0.6]
Belarus	Neonatal [1.59]	Congenital [0.94]	Drown [1.72]	Dermatitis [0.74]	Upper Digest [9.95]	Falls [1.62]	Road Inj [0.56]	Asthma [0.71]	Conduct [0.9]	Skin Viral [0.8]
Estonia	Neonatal [1.69]	Dermatitis [0.91]	Congenital [1.0]	Upper Digest [10.9]	Falls [1.24]	Iron [3.49]	Conduct [0.92]	Diarrhea [2.47]	Skin Viral [0.82]	Drown [1.83]
Latvia	Neonatal [1.52]	Congenital [1.07]	Upper Digest [11.02]	Dermatitis [0.64]	Drown [2.07]	Falls [1.35]	Diarrhea [2.38]	Asthma [0.72]	Road Inj [0.59]	Conduct [0.9]
Lithuania	Neonatal [1.74]	Upper Digest [13.05]	Congenital [0.89]	Diarrhea [2.81]	Falls [1.26]	Conduct [0.91]	Skin Viral [0.81]	Asthma [0.62]	Dermatitis [0.37]	Urticaria [1.59]
Moldova	Neonatal [1.67]	Iron [1.67]	Congenital [0.78]	Drown [1.01]	Upper Digest [9.8]	Road Inj [0.54]	Dermatitis [0.79]	Falls [1.68]	LRI [0.84]	Conduct [0.9]
Russian Federation	Neonatal [1.62]	Congenital [0.88]	Iron [2.55]	Upper Digest [9.48]	Road Inj [0.72]	Dermatitis [0.62]	Drown [1.54]	Falls [1.53]	Conduct [0.94]	Diarrhea [1.74]
Ukraine	Neonatal [1.6]	Congenital [1.21]	Iron [1.66]	Upper Digest [8.86]	Drown [1.16]	Dermatitis [0.64]	Road Inj [0.47]	Falls [1.53]	Asthma [0.69]	Conduct [0.95]
**High-income**	Dermatitis [1.16]	Neonatal [0.89]	Asthma [1.11]	Anxiety [1.08]	Congenital [0.75]	Skin Viral [1.03]	Conduct [1.02]	URI [1.07]	Road Inj [0.55]	Falls [0.76]
**Australasia**	Asthma [2.05]	Dermatitis [1.07]	Falls [2.18]	Neonatal [0.9]	Anxiety [1.15]	Conduct [1.07]	Skin Viral [1.01]	Congenital [0.66]	Road Inj [0.67]	URI [0.95]
Australia	Asthma [2.1]	Dermatitis [1.01]	Falls [2.08]	Neonatal [0.88]	Anxiety [1.11]	Conduct [1.07]	Skin Viral [1.01]	Congenital [0.64]	Road Inj [0.6]	URI [0.94]
New Zealand	Dermatitis [1.36]	Asthma [1.84]	Falls [2.74]	Neonatal [1.01]	Anxiety [1.38]	Road Inj [0.94]	Congenital [0.74]	Conduct [1.09]	Skin Viral [0.98]	Mech [3.65]
**High-income Asia Pacific**	Dermatitis [1.24]	Neonatal [1.16]	Asthma [0.93]	Congenital [0.72]	Skin Viral [1.01]	Falls [1.1]	Anxiety [0.8]	Conduct [0.88]	Diarrhea [2.36]	URI [0.97]
Brunei	Dermatitis [1.16]	Neonatal [1.13]	Road Inj [1.15]	Iron [5.03]	Congenital [0.86]	Drown [2.68]	Asthma [0.86]	Skin Viral [1.0]	LRI [4.84]	Falls [1.18]
Japan	Dermatitis [1.3]	Neonatal [1.14]	Asthma [0.94]	Congenital [0.78]	Skin Viral [1.0]	Falls [1.14]	Conduct [0.91]	Anxiety [0.78]	Diarrhea [2.22]	URI [0.99]
Aichi	Dermatitis [1.31]	Neonatal [1.17]	Asthma [0.96]	Congenital [0.75]	Skin Viral [1.01]	ASD [1.98]	Falls [1.12]	Conduct [0.91]	Anxiety [0.78]	Diarrhea [2.36]
Akita	Dermatitis [1.31]	Neonatal [1.23]	Congenital [0.89]	Asthma [0.9]	Skin Viral [0.98]	Falls [1.21]	Conduct [0.89]	Diarrhea [2.09]	Anxiety [0.81]	Iron [1.95]
Aomori	Dermatitis [1.31]	Neonatal [1.15]	Asthma [0.89]	Congenital [0.66]	Skin Viral [0.98]	Falls [1.2]	Conduct [0.89]	Diarrhea [2.05]	Anxiety [0.81]	Vit A [1.9]
Chiba	Dermatitis [1.31]	Neonatal [1.13]	Asthma [0.94]	Congenital [0.78]	Skin Viral [1.0]	Falls [1.15]	Conduct [0.9]	Anxiety [0.79]	URI [0.99]	Diarrhea [1.9]
Ehime	Dermatitis [1.31]	Neonatal [0.94]	Asthma [0.9]	Congenital [0.74]	Skin Viral [0.98]	Falls [1.22]	Conduct [0.9]	Anxiety [0.8]	Vit A [1.94]	Road Inj [0.5]
Fukui	Dermatitis [1.31]	Neonatal [1.12]	Asthma [0.91]	Congenital [0.72]	Skin Viral [0.99]	Falls [1.18]	Conduct [0.9]	Diarrhea [2.34]	Anxiety [0.79]	Road Inj [0.55]
Fukuoka	Dermatitis [1.31]	Neonatal [1.12]	Asthma [0.93]	Congenital [0.74]	Skin Viral [1.0]	Falls [1.16]	Conduct [0.9]	Anxiety [0.79]	Diarrhea [1.96]	Iron [2.35]
Fukushima	Dermatitis [1.31]	Neonatal [1.06]	Asthma [0.89]	Congenital [0.72]	Skin Viral [0.98]	Falls [1.19]	Diarrhea [2.33]	Conduct [0.89]	Anxiety [0.8]	URI [1.01]
Gifu	Dermatitis [1.31]	Neonatal [1.16]	Asthma [0.92]	Congenital [0.66]	Skin Viral [0.99]	Falls [1.18]	Iron [3.04]	Conduct [0.9]	Diarrhea [2.33]	Anxiety [0.79]
Gunma	Dermatitis [1.3]	Neonatal [1.1]	Asthma [0.92]	Congenital [0.72]	Skin Viral [0.99]	Falls [1.16]	Conduct [0.9]	Diarrhea [2.31]	Anxiety [0.79]	Iron [2.23]
Hiroshima	Dermatitis [1.31]	Neonatal [1.18]	Asthma [0.94]	Skin Viral [1.0]	Congenital [0.65]	Falls [1.12]	Conduct [0.91]	Anxiety [0.78]	Diarrhea [2.28]	URI [0.98]
Hokkaido	Dermatitis [1.31]	Neonatal [1.1]	Asthma [0.91]	Congenital [0.71]	Skin Viral [0.98]	Falls [1.16]	Diarrhea [2.3]	Conduct [0.89]	Anxiety [0.8]	Vit A [2.18]
Hyogo	Dermatitis [1.31]	Neonatal [1.15]	Asthma [0.79]	Skin Viral [1.0]	Falls [1.14]	Congenital [0.62]	Conduct [0.9]	Diarrhea [2.36]	Anxiety [0.78]	URI [1.0]
Ibaraki	Dermatitis [1.31]	Neonatal [1.19]	Congenital [0.8]	Asthma [0.92]	Skin Viral [0.99]	Falls [1.15]	Conduct [0.9]	Anxiety [0.79]	Vit A [2.37]	Diarrhea [1.99]

eFigure 7d. Leading ten causes of DALYs with the ratio of observed DALYs to DALYs expected on the basis of Socio-Demographic Index alone in 2017, 5-9 years, both sexes combined. The top ten causes contributing to DALYs are listed globally, by socio-demographic quintile, and then by GBD superregion, region, country, and subnationally where modeled. For each cell, the ratio of observed DALYs to DALYs expected on the basis of socio-demographic index (SDI) alone are listed. Abbreviations: DALY=disability-adjusted life year, GBD=Global Burden of Disease.

Values shown in brackets represent the ratio of observed DALYs to predicted DALYs on the basis of Socio-Demographic Index (SDI), rounded to two (2) digits. Color ranges (shown below) were calculated to place a roughly equal number of cells into each bin.

COLOR KEY:	[0.0-0.62]	(0.62-0.79]	(0.79-0.89]	(0.89-0.97]	(0.97-1.07]	(1.07-1.26]	(1.26-1.44]	(1.44-2.32]	2.32+

	1	2	3	4	5	6	7	8	9	10
Ishikawa	Dermatitis [1.31]	Neonatal [1.1]	Congenital [0.81]	Asthma [0.93]	Skin Viral [0.99]	Falls [1.16]	Conduct [0.89]	Diarrhea [2.33]	Anxiety [0.79]	URI [0.99]
Iwate	Dermatitis [1.31]	Neonatal [1.18]	Congenital [0.78]	Asthma [0.89]	Skin Viral [0.97]	Falls [1.21]	Conduct [0.89]	Diarrhea [2.04]	Anxiety [0.81]	Vit A [1.62]
Kagawa	Dermatitis [1.3]	Neonatal [1.08]	Asthma [0.92]	Diarrhea [3.03]	Congenital [0.69]	Skin Viral [0.99]	Falls [1.14]	Conduct [0.9]	Anxiety [0.79]	URI [1.0]
Kagoshima	Dermatitis [1.31]	Neonatal [0.98]	Congenital [0.77]	Asthma [0.89]	Skin Viral [0.98]	Falls [1.19]	Conduct [0.89]	Diarrhea [2.15]	Anxiety [0.81]	Iron [2.0]
Kanagawa	Dermatitis [1.31]	Neonatal [1.19]	Congenital [0.87]	Asthma [0.98]	Skin Viral [1.01]	Falls [1.13]	Conduct [0.91]	Anxiety [0.78]	ASD [1.59]	URI [0.99]
Kochi	Dermatitis [1.31]	Neonatal [1.05]	Asthma [0.89]	Congenital [0.7]	Skin Viral [0.98]	Falls [1.21]	Conduct [0.88]	Diarrhea [2.07]	Anxiety [0.81]	Iron [1.79]
Kumamoto	Dermatitis [1.31]	Neonatal [1.03]	Asthma [0.89]	Congenital [0.64]	Skin Viral [0.98]	Falls [1.19]	Conduct [0.89]	Anxiety [0.81]	Iron [2.05]	Diarrhea [1.84]
Kyoto	Neonatal [1.19]	Dermatitis [1.04]	Congenital [0.98]	Asthma [0.97]	Skin Viral [1.01]	Falls [1.13]	Conduct [0.92]	Anxiety [0.77]	Diarrhea [2.41]	URI [0.98]
Mie	Dermatitis [1.31]	Neonatal [1.17]	Asthma [0.92]	Skin Viral [0.99]	Diarrhea [2.72]	Congenital [0.65]	Falls [1.14]	Conduct [0.9]	Anxiety [0.79]	URI [0.99]
Miyagi	Dermatitis [1.31]	Neonatal [1.09]	Congenital [0.84]	Asthma [0.77]	Skin Viral [0.99]	Falls [1.16]	Conduct [0.9]	Anxiety [0.79]	Diarrhea [2.09]	URI [1.0]
Miyazaki	Dermatitis [1.31]	Neonatal [1.03]	Asthma [0.88]	Congenital [0.69]	Skin Viral [0.98]	Falls [1.21]	Conduct [0.89]	Anxiety [0.81]	URI [1.01]	ASD [1.34]
Nagano	Dermatitis [1.31]	Neonatal [1.19]	Asthma [0.92]	Congenital [0.72]	Diarrhea [2.82]	Skin Viral [0.99]	Falls [1.18]	Conduct [0.9]	Anxiety [0.79]	URI [1.0]
Nagasaki	Dermatitis [1.31]	Neonatal [1.03]	Congenital [0.73]	Asthma [0.88]	Skin Viral [0.98]	Falls [1.19]	Conduct [0.89]	Anxiety [0.81]	Diarrhea [1.69]	URI [1.01]
Nara	Dermatitis [1.31]	Neonatal [1.02]	Congenital [0.87]	Asthma [0.92]	Skin Viral [0.99]	Falls [1.17]	Conduct [0.9]	Anxiety [0.8]	Diarrhea [2.24]	Vit A [2.1]
Niigata	Dermatitis [1.3]	Neonatal [1.15]	Asthma [0.91]	Congenital [0.74]	Skin Viral [0.99]	Falls [1.17]	Iron [2.9]	Conduct [0.89]	Anxiety [0.8]	Diarrhea [2.14]
Oita	Dermatitis [1.3]	Neonatal [1.2]	Asthma [0.9]	Congenital [0.74]	Skin Viral [0.99]	Falls [1.2]	Diarrhea [2.51]	Conduct [0.9]	Anxiety [0.79]	URI [1.0]
Okayama	Dermatitis [1.3]	Neonatal [1.21]	Asthma [0.93]	Skin Viral [0.99]	Congenital [0.64]	Falls [1.15]	Conduct [0.91]	Anxiety [0.79]	Diarrhea [2.08]	URI [0.99]
Okinawa	Dermatitis [1.31]	Neonatal [1.08]	Asthma [0.75]	Skin Viral [0.98]	Falls [1.21]	Congenital [0.56]	Vit A [2.04]	Conduct [0.88]	Anxiety [0.81]	Diarrhea [1.76]
Osaka	Dermatitis [1.31]	Neonatal [1.09]	Asthma [0.96]	Congenital [0.82]	Skin Viral [1.01]	Falls [1.15]	Conduct [0.91]	Anxiety [0.78]	Diarrhea [2.15]	URI [0.99]
Saga	Dermatitis [1.31]	Neonatal [1.08]	Asthma [0.9]	Congenital [0.68]	Skin Viral [0.98]	Falls [1.19]	Conduct [0.89]	Anxiety [0.8]	Diarrhea [2.07]	URI [1.0]
Saitama	Dermatitis [1.31]	Neonatal [1.12]	Asthma [0.93]	Congenital [0.68]	Skin Viral [0.99]	Falls [1.18]	Conduct [0.9]	Anxiety [0.79]	Diarrhea [2.07]	URI [1.0]
Shiga	Dermatitis [1.59]	Neonatal [1.08]	Asthma [0.95]	Congenital [0.76]	Skin Viral [1.0]	Falls [1.13]	Conduct [0.91]	Anxiety [0.78]	Diarrhea [2.4]	URI [0.99]
Shimane	Dermatitis [1.31]	Neonatal [1.06]	Asthma [0.88]	Congenital [0.63]	Skin Viral [0.98]	Falls [1.2]	Conduct [0.9]	Diarrhea [2.1]	Anxiety [0.8]	URI [1.01]
Shizuoka	Dermatitis [1.31]	Neonatal [1.25]	Asthma [0.93]	Congenital [0.7]	Skin Viral [1.0]	Falls [1.13]	Conduct [0.91]	Anxiety [0.79]	Diarrhea [2.14]	Vit A [2.24]
Tochigi	Dermatitis [1.31]	Neonatal [1.2]	Congenital [0.94]	Asthma [0.93]	Skin Viral [0.99]	Falls [1.13]	Conduct [0.9]	Anxiety [0.79]	URI [0.99]	ASD [1.33]
Tokushima	Dermatitis [1.3]	Neonatal [1.14]	Congenital [0.84]	Asthma [0.91]	Skin Viral [0.99]	Falls [1.21]	Diarrhea [2.51]	Road Inj [0.66]	Conduct [0.9]	Anxiety [0.79]
Tokyo	Dermatitis [1.33]	Neonatal [1.25]	Congenital [1.07]	Asthma [1.07]	Skin Viral [1.05]	Falls [1.05]	Conduct [0.95]	Anxiety [0.76]	Diarrhea [2.52]	URI [0.97]
Tottori	Dermatitis [1.3]	Neonatal [1.14]	Congenital [0.82]	Asthma [0.89]	Skin Viral [0.98]	Falls [1.2]	Conduct [0.9]	Anxiety [0.8]	Iron [2.27]	Road Inj [0.52]
Toyama	Dermatitis [1.3]	Neonatal [1.1]	Asthma [0.94]	Congenital [0.74]	Skin Viral [1.0]	Falls [1.13]	Conduct [0.91]	Diarrhea [2.43]	Iron [3.01]	Anxiety [0.78]
Wakayama	Dermatitis [1.31]	Neonatal [1.19]	Asthma [0.9]	Skin Viral [0.98]	Congenital [0.62]	Falls [1.18]	Conduct [0.9]	Iron [2.56]	Anxiety [0.8]	Diarrhea [2.11]
Yamagata	Dermatitis [1.31]	Neonatal [0.97]	Asthma [0.9]	Congenital [0.69]	Skin Viral [0.98]	Diarrhea [2.44]	Falls [1.21]	Conduct [0.89]	Anxiety [0.8]	URI [1.01]

eFigure 7d. Leading ten causes of DALYs with the ratio of observed DALYs to DALYs expected on the basis of Socio-Demographic Index alone in 2017, 5-9 years, both sexes combined. The top ten causes contributing to DALYs are listed globally, by socio-demographic quintile, and then by GBD superregion, region, country, and subnationally where modeled. For each cell, the ratio of observed DALYs to DALYs expected on the basis of socio-demographic index (SDI) alone are listed. Abbreviations: DALY=disability-adjusted life year, GBD=Global Burden of Disease.

Values shown in brackets represent the ratio of observed DALYs to predicted DALYs on the basis of Socio-Demographic Index (SDI), rounded to two (2) digits. Color ranges (shown below) were calculated to place a roughly equal number of cells into each bin.

COLOR KEY:	[0.0-0.62]	(0.62-0.79]	(0.79-0.89]	(0.89-0.97]	(0.97-1.07]	(1.07-1.26]	(1.26-1.44]	(1.44-2.32]	2.32+

	1	2	3	4	5	6	7	8	9	10
Yamaguchi	Dermatitis [1.31]	Neonatal [1.01]	Asthma [0.91]	Skin Viral [0.99]	Congenital [0.61]	Falls [1.13]	Conduct [0.9]	Diarrhea [2.33]	Anxiety [0.79]	URI [1.0]
Yamanashi	Dermatitis [1.31]	Neonatal [1.26]	Asthma [0.93]	Congenital [0.74]	Skin Viral [1.0]	Falls [1.16]	Conduct [0.9]	Anxiety [0.79]	Diarrhea [2.13]	URI [0.99]
S Korea	Neonatal [1.18]	Dermatitis [1.11]	Asthma [0.89]	Skin Viral [1.01]	Anxiety [0.85]	Diarrhea [2.57]	Falls [1.04]	Congenital [0.57]	Road Inj [0.7]	Conduct [0.82]
Singapore	Neonatal [1.17]	Dermatitis [1.03]	Asthma [0.88]	Skin Viral [1.01]	Anxiety [0.83]	Falls [1.04]	Diarrhea [2.4]	Conduct [0.85]	Congenital [0.56]	Iron [2.96]
**High-income North America**	Dermatitis [1.28]	Asthma [1.44]	Neonatal [0.88]	Skin Viral [1.16]	Conduct [1.18]	Congenital [0.77]	Anxiety [0.92]	Road Inj [0.81]	Psoriasis [3.75]	URI [0.98]
Canada	Dermatitis [1.38]	Asthma [1.58]	Skin Viral [1.28]	Neonatal [0.61]	Congenital [0.68]	Conduct [0.97]	Anxiety [0.77]	Psoriasis [3.73]	ASD [1.51]	URI [1.03]
Greenland	Dermatitis [1.33]	Asthma [1.43]	Drown [1.61]	Congenital [0.7]	Fire [3.81]	Neonatal [0.6]	Skin Viral [1.18]	Conduct [1.0]	Violence [3.0]	Anxiety [0.96]
USA	Dermatitis [1.27]	Asthma [1.42]	Neonatal [0.9]	Conduct [1.19]	Skin Viral [1.15]	Congenital [0.76]	Anxiety [0.94]	Road Inj [0.81]	Psoriasis [3.76]	URI [0.98]
Alabama	Dermatitis [1.12]	Asthma [1.39]	Neonatal [0.89]	Road Inj [1.22]	Skin Viral [1.2]	Conduct [1.14]	Congenital [0.67]	Anxiety [0.96]	Iron [2.52]	Psoriasis [3.43]
Alaska	Dermatitis [1.12]	Neonatal [0.85]	Asthma [1.08]	Congenital [0.82]	Conduct [1.16]	Anxiety [0.94]	Road Inj [0.72]	Drown [1.96]	Skin Viral [0.83]	Oth Trans [6.44]
Arizona	Dermatitis [1.3]	Asthma [1.5]	Neonatal [0.89]	Conduct [1.15]	Congenital [0.7]	Road Inj [0.79]	Anxiety [0.95]	Skin Viral [0.89]	Iron [2.81]	Psoriasis [3.42]
Arkansas	Dermatitis [1.03]	Road Inj [1.16]	Neonatal [0.78]	Asthma [1.05]	Congenital [0.68]	Conduct [1.14]	Anxiety [0.97]	Skin Viral [0.97]	Iron [1.99]	URI [0.99]
California	Dermatitis [1.42]	Asthma [1.36]	Neonatal [0.75]	Conduct [1.18]	Congenital [0.71]	Anxiety [0.93]	Skin Viral [0.97]	Psoriasis [3.89]	Road Inj [0.6]	ASD [1.39]
Colorado	Dermatitis [1.14]	Neonatal [0.92]	Asthma [1.37]	Conduct [1.18]	Congenital [0.74]	Anxiety [0.93]	Skin Viral [0.98]	Road Inj [0.75]	URI [0.97]	Psoriasis [2.68]
Connecticut	Dermatitis [1.44]	Asthma [1.94]	Neonatal [1.08]	Skin Viral [1.53]	Conduct [1.19]	Anxiety [0.91]	Congenital [0.74]	Psoriasis [4.13]	URI [0.97]	Epilepsy [1.37]
Delaware	Dermatitis [1.14]	Asthma [1.58]	Neonatal [0.88]	Skin Viral [1.36]	Conduct [1.17]	Congenital [0.73]	Anxiety [0.93]	Road Inj [0.79]	Psoriasis [4.09]	Epilepsy [1.38]
DC	Dermatitis [1.69]	Asthma [1.6]	Neonatal [0.93]	Violence [7.8]	Skin Viral [1.36]	Congenital [0.94]	Conduct [1.17]	Anxiety [0.93]	Road Inj [0.86]	Psoriasis [3.68]
Florida	Dermatitis [1.28]	Neonatal [1.01]	Asthma [1.55]	Skin Viral [1.35]	Conduct [1.16]	Congenital [0.76]	Road Inj [0.86]	Anxiety [0.94]	Psoriasis [4.71]	Iron [2.76]
Georgia	Dermatitis [1.39]	Asthma [1.49]	Neonatal [0.83]	Road Inj [0.98]	Skin Viral [1.19]	Conduct [1.15]	Congenital [0.7]	Anxiety [0.95]	Psoriasis [4.18]	Epilepsy [1.24]
Hawaii	Dermatitis [1.49]	Asthma [1.55]	Neonatal [0.82]	Skin Viral [1.37]	Conduct [1.17]	Congenital [0.74]	Anxiety [0.94]	Road Inj [0.71]	Psoriasis [3.86]	URI [0.98]
Idaho	Dermatitis [1.04]	Neonatal [0.74]	Skin Viral [1.26]	Road Inj [0.81]	Conduct [1.15]	Congenital [0.66]	Asthma [0.78]	Anxiety [0.96]	URI [0.99]	Psoriasis [2.82]
Illinois	Dermatitis [1.12]	Neonatal [1.06]	Asthma [1.33]	Congenital [0.82]	Skin Viral [1.16]	Conduct [1.17]	Anxiety [0.93]	Road Inj [0.68]	Psoriasis [3.57]	URI [0.97]
Indiana	Dermatitis [1.09]	Asthma [1.33]	Neonatal [0.85]	Congenital [0.81]	Iron [3.79]	Conduct [1.15]	Road Inj [0.8]	Anxiety [0.95]	Skin Viral [1.0]	Epilepsy [1.23]
Iowa	Dermatitis [1.0]	Neonatal [0.87]	Skin Viral [1.17]	Conduct [1.17]	Congenital [0.76]	Asthma [0.83]	Road Inj [0.89]	Anxiety [0.94]	Iron [3.53]	URI [0.97]
Kansas	Dermatitis [1.06]	Neonatal [0.92]	Asthma [1.38]	Road Inj [1.11]	Conduct [1.16]	Congenital [0.77]	Skin Viral [1.05]	Anxiety [0.94]	URI [0.97]	Psoriasis [3.14]
Kentucky	Dermatitis [1.12]	Asthma [1.37]	Neonatal [0.87]	Road Inj [0.93]	Skin Viral [1.21]	Congenital [0.74]	Conduct [1.14]	Anxiety [0.97]	Iron [2.03]	Epilepsy [1.22]
Louisiana	Dermatitis [1.2]	Neonatal [0.81]	Skin Viral [1.59]	Asthma [1.1]	Road Inj [1.0]	Congenital [0.76]	Conduct [1.14]	Anxiety [0.97]	Psoriasis [4.06]	Epilepsy [1.25]
Maine	Dermatitis [0.97]	Asthma [1.52]	Neonatal [0.91]	Congenital [0.8]	Conduct [1.17]	Road Inj [0.9]	Skin Viral [1.05]	Anxiety [0.94]	Psoriasis [3.46]	URI [0.99]
Maryland	Dermatitis [1.44]	Neonatal [1.06]	Asthma [1.65]	Skin Viral [1.18]	Conduct [1.19]	Congenital [0.84]	Anxiety [0.92]	Road Inj [0.88]	Psoriasis [4.11]	URI [0.96]
Massachusetts	Dermatitis [1.29]	Neonatal [1.03]	Asthma [1.69]	Skin Viral [1.42]	Conduct [1.2]	Congenital [0.87]	Anxiety [0.91]	Psoriasis [4.13]	ASD [1.5]	URI [0.97]
Michigan	Dermatitis [1.13]	Asthma [1.53]	Neonatal [0.89]	Skin Viral [1.36]	Congenital [0.83]	Conduct [1.16]	Anxiety [0.94]	Road Inj [0.76]	Iron [3.26]	Psoriasis [3.59]

eFigure 7d. Leading ten causes of DALYs with the ratio of observed DALYs to DALYs expected on the basis of Socio-Demographic Index alone in 2017, 5-9 years, both sexes combined. The top ten causes contributing to DALYs are listed globally, by socio-demographic quintile, and then by GBD superregion, region, country, and subnationally where modeled. For each cell, the ratio of observed DALYs to DALYs expected on the basis of socio-demographic index (SDI) alone are listed. Abbreviations: DALY=disability-adjusted life year, GBD=Global Burden of Disease.

Values shown in brackets represent the ratio of observed DALYs to predicted DALYs on the basis of Socio-Demographic Index (SDI), rounded to two (2) digits. Color ranges (shown below) were calculated to place a roughly equal number of cells into each bin.

COLOR KEY:	[0.0-0.62]	(0.62-0.79]	(0.79-0.89]	(0.89-0.97]	(0.97-1.07]	(1.07-1.26]	(1.26-1.44]	(1.44-2.32]	2.32+

	1	2	3	4	5	6	7	8	9	10
Minnesota	Dermatitis [1.0]	Neonatal [0.96]	Asthma [1.19]	Skin Viral [1.34]	Congenital [0.89]	Conduct [1.19]	Anxiety [0.93]	Road Inj [0.88]	URI [0.97]	ASD [1.34]
Mississippi	Dermatitis [1.02]	Road Inj [1.19]	Neonatal [0.85]	Asthma [0.95]	Conduct [1.13]	Iron [2.62]	Congenital [0.63]	Anxiety [0.98]	Skin Viral [0.97]	URI [0.99]
Missouri	Dermatitis [1.01]	Neonatal [0.92]	Asthma [1.33]	Road Inj [0.96]	Congenital [0.8]	Conduct [1.16]	Skin Viral [1.04]	Anxiety [0.95]	URI [0.98]	Psoriasis [3.22]
Montana	Dermatitis [0.99]	Neonatal [0.87]	Road Inj [1.23]	Asthma [1.02]	Skin Viral [1.19]	Conduct [1.16]	Congenital [0.77]	Anxiety [0.94]	Iron [2.55]	URI [0.98]
Nebraska	Dermatitis [1.0]	Neonatal [0.87]	Asthma [0.96]	Skin Viral [1.18]	Congenital [0.82]	Road Inj [1.0]	Conduct [1.17]	Anxiety [0.94]	URI [0.97]	Psoriasis [3.01]
Nevada	Dermatitis [1.23]	Neonatal [0.84]	Asthma [1.22]	Conduct [1.15]	Anxiety [0.96]	Road Inj [0.74]	Congenital [0.63]	Skin Viral [0.71]	URI [0.99]	Psoriasis [2.99]
New Hampshire	Dermatitis [1.01]	Asthma [1.46]	Neonatal [0.87]	Skin Viral [1.2]	Conduct [1.19]	Congenital [0.82]	Anxiety [0.92]	Psoriasis [3.83]	Road Inj [0.82]	URI [0.97]
New Jersey	Dermatitis [1.57]	Asthma [1.95]	Neonatal [0.97]	Skin Viral [1.4]	Congenital [0.88]	Conduct [1.19]	Anxiety [0.92]	Psoriasis [4.59]	ASD [1.65]	URI [0.96]
New Mexico	Dermatitis [1.13]	Asthma [1.48]	Neonatal [0.95]	Road Inj [0.99]	Congenital [0.73]	Conduct [1.14]	Anxiety [0.97]	Skin Viral [0.72]	URI [0.99]	Epilepsy [1.06]
New York	Dermatitis [1.62]	Asthma [2.11]	Neonatal [1.01]	Skin Viral [1.45]	Congenital [0.91]	Conduct [1.18]	Psoriasis [5.0]	Anxiety [0.92]	URI [0.96]	Epilepsy [1.39]
N Carolina	Dermatitis [1.18]	Neonatal [0.9]	Asthma [1.28]	Skin Viral [1.16]	Road Inj [0.87]	Congenital [0.72]	Conduct [1.06]	Anxiety [0.9]	Psoriasis [3.76]	URI [0.98]
N Dakota	Dermatitis [0.96]	Neonatal [0.95]	Asthma [1.16]	Congenital [0.89]	Skin Viral [1.14]	Conduct [1.18]	Anxiety [0.93]	Road Inj [0.85]	URI [0.97]	Iron [2.63]
Ohio	Dermatitis [1.2]	Asthma [1.41]	Neonatal [0.86]	Congenital [0.84]	Conduct [1.16]	Skin Viral [1.12]	Anxiety [0.94]	Road Inj [0.68]	Psoriasis [3.44]	Epilepsy [1.31]
Oklahoma	Dermatitis [1.25]	Asthma [1.5]	Road Inj [1.26]	Neonatal [0.81]	Congenital [0.82]	Conduct [1.15]	Anxiety [0.96]	Skin Viral [0.94]	Epilepsy [1.22]	URI [0.98]
Oregon	Dermatitis [1.32]	Neonatal [0.78]	Asthma [1.08]	Skin Viral [1.16]	Congenital [0.7]	Conduct [1.02]	Anxiety [0.87]	Psoriasis [3.64]	Road Inj [0.63]	URI [0.97]
Pennsylvania	Dermatitis [1.24]	Asthma [1.59]	Neonatal [0.9]	Congenital [0.86]	Skin Viral [1.2]	Conduct [1.17]	Anxiety [0.93]	Road Inj [0.75]	Psoriasis [3.7]	URI [0.97]
Rhode Island	Dermatitis [1.14]	Asthma [1.57]	Neonatal [0.88]	Skin Viral [1.43]	Conduct [1.18]	Anxiety [0.92]	Congenital [0.74]	Psoriasis [3.87]	URI [0.98]	Epilepsy [1.29]
S Carolina	Dermatitis [1.37]	Neonatal [0.85]	Asthma [1.28]	Iron [4.38]	Road Inj [0.99]	Skin Viral [1.19]	Congenital [0.72]	Conduct [1.14]	Anxiety [0.96]	Psoriasis [3.97]
S Dakota	Dermatitis [0.97]	Road Inj [1.5]	Neonatal [0.77]	Congenital [0.9]	Skin Viral [1.29]	Asthma [0.89]	Conduct [1.16]	Anxiety [0.95]	Iron [2.43]	URI [0.98]
Tennessee	Dermatitis [1.12]	Neonatal [0.84]	Asthma [1.08]	Road Inj [0.89]	Conduct [1.14]	Congenital [0.68]	Skin Viral [1.01]	Anxiety [0.96]	ASD [1.49]	Psoriasis [3.39]
Texas	Dermatitis [1.33]	Neonatal [0.96]	Asthma [1.48]	Conduct [1.49]	Road Inj [0.86]	Congenital [0.69]	Anxiety [0.96]	Skin Viral [1.0]	Psoriasis [3.97]	ASD [1.6]
Utah	Dermatitis [1.06]	Neonatal [0.83]	Skin Viral [1.3]	Conduct [1.15]	Asthma [0.85]	Congenital [0.7]	Anxiety [0.95]	ASD [1.9]	Iron [3.2]	Road Inj [0.63]
Vermont	Dermatitis [0.99]	Neonatal [0.97]	Asthma [1.31]	Conduct [1.19]	Congenital [0.82]	Anxiety [0.92]	Skin Viral [1.04]	Road Inj [0.87]	Psoriasis [3.31]	URI [0.96]
Virginia	Dermatitis [1.45]	Neonatal [0.98]	Asthma [1.49]	Conduct [1.18]	Skin Viral [1.06]	Congenital [0.74]	Anxiety [0.93]	Road Inj [0.78]	Psoriasis [3.75]	URI [0.97]
Washington	Dermatitis [1.28]	Neonatal [0.83]	Asthma [1.06]	Skin Viral [1.22]	Conduct [1.18]	Congenital [0.78]	Anxiety [0.93]	Psoriasis [3.56]	Road Inj [0.63]	URI [0.97]
W Virginia	Dermatitis [1.14]	Asthma [1.42]	Neonatal [0.85]	Road Inj [0.96]	Congenital [0.74]	Conduct [1.14]	Anxiety [0.97]	Skin Viral [0.97]	Iron [2.25]	Epilepsy [1.25]
Wisconsin	Dermatitis [1.0]	Neonatal [0.96]	Asthma [1.07]	Skin Viral [1.2]	Congenital [0.82]	Conduct [1.18]	Anxiety [0.93]	Road Inj [0.73]	URI [0.97]	Psoriasis [3.2]
Wyoming	Dermatitis [1.05]	Road Inj [1.41]	Neonatal [0.73]	Asthma [1.09]	Congenital [0.8]	Conduct [1.17]	Anxiety [0.94]	Skin Viral [0.91]	URI [0.98]	Epilepsy [1.16]
**Southern Latin America**	Neonatal [0.95]	Dermatitis [1.0]	Asthma [0.94]	Anxiety [1.37]	Congenital [0.57]	Road Inj [0.42]	Diarrhea [1.77]	Skin Viral [0.99]	Conduct [0.92]	Leukemia [0.58]
Argentina	Neonatal [0.94]	Dermatitis [0.94]	Asthma [0.92]	Congenital [0.56]	Anxiety [1.38]	Road Inj [0.41]	Diarrhea [1.72]	Skin Viral [0.99]	Conduct [0.93]	Leukemia [0.59]
Chile	Dermatitis [1.19]	Neonatal [1.0]	Asthma [0.99]	Anxiety [1.32]	Congenital [0.59]	Diarrhea [2.07]	Road Inj [0.46]	Skin Viral [0.99]	Conduct [0.89]	Epilepsy [0.97]

eFigure 7d. Leading ten causes of DALYs with the ratio of observed DALYs to DALYs expected on the basis of Socio-Demographic Index alone in 2017, 5-9 years, both sexes combined. The top ten causes contributing to DALYs are listed globally, by socio-demographic quintile, and then by GBD superregion, region, country, and subnationally where modeled. For each cell, the ratio of observed DALYs to DALYs expected on the basis of socio-demographic index (SDI) alone are listed. Abbreviations: DALY=disability-adjusted life year, GBD=Global Burden of Disease.

Values shown in brackets represent the ratio of observed DALYs to predicted DALYs on the basis of Socio-Demographic Index (SDI), rounded to two (2) digits. Color ranges (shown below) were calculated to place a roughly equal number of cells into each bin.

COLOR KEY:	[0.0-0.62]	(0.62-0.79]	(0.79-0.89]	(0.89-0.97]	(0.97-1.07]	(1.07-1.26]	(1.26-1.44]	(1.44-2.32]	2.32+

	1	2	3	4	5	6	7	8	9	10
Uruguay	Neonatal [1.0]	Dermatitis [0.93]	Asthma [1.07]	Anxiety [1.39]	Congenital [0.53]	Skin Viral [0.99]	Road Inj [0.34]	Conduct [0.93]	Iron [0.6]	Vit A [0.52]
**Western Europe**	Dermatitis [1.08]	Neonatal [0.81]	Anxiety [1.29]	Congenital [0.78]	Asthma [0.83]	Skin Viral [0.95]	Conduct [0.95]	URI [1.26]	Falls [0.85]	Epilepsy [0.99]
Andorra	Dermatitis [1.48]	Neonatal [0.86]	Anxiety [1.22]	Asthma [1.01]	Congenital [0.87]	Skin Viral [0.99]	Conduct [0.96]	URI [1.24]	Falls [0.81]	Headaches [1.03]
Austria	Neonatal [1.34]	Dermatitis [0.75]	Anxiety [1.24]	Congenital [0.87]	Skin Viral [0.96]	Asthma [0.74]	Conduct [0.95]	URI [1.26]	Falls [0.89]	Headaches [1.01]
Belgium	Dermatitis [0.91]	Anxiety [1.19]	Neonatal [0.56]	Congenital [0.78]	Skin Viral [0.98]	Conduct [0.96]	Falls [1.01]	URI [1.24]	Asthma [0.7]	Headaches [1.28]
Cyprus	Neonatal [1.07]	Dermatitis [0.77]	Anxiety [1.23]	Congenital [0.71]	Asthma [0.76]	Skin Viral [0.96]	Conduct [0.95]	URI [1.26]	Road Inj [0.58]	Falls [0.82]
Denmark	Dermatitis [1.26]	Anxiety [1.2]	Neonatal [0.69]	Congenital [1.05]	Skin Viral [1.15]	Asthma [0.93]	Iron [5.37]	URI [1.22]	Conduct [0.9]	Falls [0.75]
Finland	Neonatal [1.68]	Dermatitis [1.32]	Congenital [1.0]	Asthma [0.9]	Anxiety [0.9]	Skin Viral [1.01]	Conduct [0.93]	URI [1.23]	Falls [0.91]	Road Inj [0.62]
France	Dermatitis [1.2]	Anxiety [1.46]	Neonatal [0.65]	Congenital [0.75]	Asthma [0.78]	Skin Viral [0.96]	Conduct [0.94]	URI [1.26]	Falls [0.88]	Epilepsy [1.07]
Germany	Neonatal [0.93]	Dermatitis [0.91]	Anxiety [1.45]	Congenital [0.82]	Skin Viral [0.98]	Epilepsy [1.9]	Conduct [0.95]	URI [1.25]	Falls [0.85]	Asthma [0.57]
Greece	Neonatal [0.84]	Anxiety [1.39]	Congenital [0.79]	Dermatitis [0.64]	Skin Viral [0.94]	Road Inj [0.59]	Asthma [0.68]	Conduct [0.92]	URI [1.31]	Falls [0.91]
Iceland	Dermatitis [1.38]	Neonatal [0.87]	Asthma [1.39]	Anxiety [1.21]	Congenital [0.88]	Skin Viral [1.09]	Conduct [0.97]	URI [1.23]	Falls [0.8]	Brain C [0.98]
Ireland	Dermatitis [1.12]	Asthma [1.17]	Anxiety [1.33]	Neonatal [0.72]	Congenital [0.9]	Skin Viral [0.97]	Conduct [0.95]	URI [1.24]	Falls [0.86]	Headaches [1.01]
Israel	Dermatitis [1.0]	Neonatal [0.92]	Congenital [0.71]	Blindness [2.66]	Asthma [0.72]	Skin Viral [0.91]	Conduct [0.92]	URI [1.28]	Anxiety [0.81]	Falls [0.93]
Italy	Dermatitis [0.91]	Anxiety [1.32]	Neonatal [0.64]	Congenital [0.74]	Skin Viral [0.95]	Conduct [0.94]	URI [1.27]	Headaches [1.21]	Falls [0.72]	Asthma [0.42]
Luxembourg	Dermatitis [1.48]	Neonatal [1.13]	Anxiety [1.2]	Asthma [1.11]	Congenital [0.8]	Skin Viral [1.0]	Conduct [0.98]	URI [1.23]	Falls [0.79]	Headaches [1.16]
Malta	Congenital [1.08]	Dermatitis [0.79]	Neonatal [0.71]	Anxiety [1.27]	Asthma [0.82]	Skin Viral [0.94]	Conduct [0.93]	Falls [1.13]	URI [1.28]	Iron [1.96]
Netherlands	Dermatitis [1.18]	Neonatal [0.87]	Anxiety [1.43]	Congenital [0.89]	Asthma [0.91]	Conduct [1.09]	Skin Viral [0.92]	URI [1.23]	Falls [0.68]	Headaches [1.14]
Norway	Dermatitis [1.33]	Neonatal [1.04]	Anxiety [1.6]	Asthma [1.35]	Congenital [1.02]	Skin Viral [1.07]	Conduct [0.98]	Falls [0.98]	URI [1.24]	Headaches [1.04]
Portugal	Dermatitis [0.86]	Asthma [1.12]	Anxiety [1.34]	Neonatal [0.59]	Conduct [0.92]	Congenital [0.48]	Skin Viral [0.84]	Vit A [1.25]	URI [1.31]	Iron [0.96]
Spain	Neonatal [0.68]	Dermatitis [0.67]	Anxiety [1.21]	Congenital [0.66]	Skin Viral [0.92]	Conduct [0.93]	Asthma [0.67]	URI [1.29]	Iron [1.91]	Falls [0.86]
Sweden	Dermatitis [1.69]	Asthma [1.25]	Neonatal [0.76]	Congenital [0.97]	Anxiety [1.19]	Skin Viral [1.07]	Conduct [0.98]	Falls [1.01]	URI [1.25]	ASD [1.1]
Stockholm	Dermatitis [1.35]	Congenital [1.34]	Neonatal [0.75]	Asthma [1.17]	Anxiety [1.17]	Skin Viral [1.1]	Conduct [0.99]	Falls [0.97]	URI [1.23]	ASD [1.38]
Sweden w/o Stockholm	Dermatitis [1.79]	Asthma [1.27]	Neonatal [0.77]	Anxiety [1.2]	Congenital [0.87]	Skin Viral [1.06]	Conduct [0.97]	Falls [1.02]	URI [1.26]	Headaches [0.96]
Switzerland	Dermatitis [1.47]	Neonatal [0.83]	Anxiety [1.22]	Congenital [0.82]	Asthma [0.89]	Skin Viral [0.98]	Conduct [0.96]	URI [1.24]	Falls [0.67]	ASD [1.02]
UK	Dermatitis [1.43]	Neonatal [0.97]	Asthma [1.39]	Iron [4.12]	Congenital [0.83]	Anxiety [1.08]	Conduct [0.98]	Skin Viral [0.89]	URI [1.28]	Falls [0.88]
England	Dermatitis [1.46]	Neonatal [0.99]	Asthma [1.42]	Iron [4.26]	Congenital [0.83]	Anxiety [1.05]	Conduct [0.98]	Skin Viral [0.88]	URI [1.28]	Falls [0.87]
E Midlands	Dermatitis [1.44]	Neonatal [0.94]	Asthma [1.31]	Iron [3.51]	Congenital [0.78]	Anxiety [1.07]	Conduct [0.97]	Skin Viral [0.87]	URI [1.29]	Falls [0.9]
Derby	Dermatitis [1.43]	Neonatal [0.95]	Asthma [1.32]	Iron [3.85]	Congenital [0.77]	Anxiety [1.05]	Conduct [0.98]	Skin Viral [0.88]	URI [1.27]	Falls [0.87]
Derbyshire	Dermatitis [1.44]	Neonatal [0.86]	Asthma [1.27]	Iron [3.92]	Congenital [0.77]	Anxiety [1.08]	Conduct [0.97]	Skin Viral [0.87]	URI [1.31]	Falls [0.94]
Leicester	Dermatitis [1.44]	Asthma [1.36]	Neonatal [0.81]	Congenital [0.9]	Anxiety [1.06]	Conduct [0.98]	Iron [2.84]	Skin Viral [0.88]	URI [1.27]	Falls [0.91]

eFigure 7d. Leading ten causes of DALYs with the ratio of observed DALYs to DALYs expected on the basis of Socio-Demographic Index alone in 2017, 5-9 years, both sexes combined. The top ten causes contributing to DALYs are listed globally, by socio-demographic quintile, and then by GBD superregion, region, country, and subnationally where modeled. For each cell, the ratio of observed DALYs to DALYs expected on the basis of socio-demographic index (SDI) alone are listed. Abbreviations: DALY=disability-adjusted life year, GBD=Global Burden of Disease.

Values shown in brackets represent the ratio of observed DALYs to predicted DALYs on the basis of Socio-Demographic Index (SDI), rounded to two (2) digits. Color ranges (shown below) were calculated to place a roughly equal number of cells into each bin.

COLOR KEY:		[0.0-0.62]	(0.62-0.79]	(0.79-0.89]	(0.89-0.97]	(0.97-1.07]	(1.07-1.26]	(1.26-1.44]	(1.44-2.32]	2.32+
	1	2	3	4	5	6	7	8	9	10
Leicestershire	Dermatitis [1.44]	Asthma [1.32]	Neonatal [0.79]	Iron [4.19]	Congenital [0.79]	Anxiety [1.05]	Conduct [0.98]	Skin Viral [0.88]	URI [1.28]	Falls [0.87]
Lincolnshire	Dermatitis [1.44]	Neonatal [0.92]	Asthma [1.28]	Congenital [0.82]	Iron [2.98]	Anxiety [1.09]	Conduct [0.96]	Skin Viral [0.87]	URI [1.32]	Falls [0.93]
Northamptonshire	Dermatitis [1.44]	Neonatal [1.03]	Asthma [1.3]	Iron [3.52]	Congenital [0.76]	Anxiety [1.07]	Conduct [0.97]	Skin Viral [0.87]	URI [1.29]	Falls [0.89]
Nottingham	Dermatitis [1.44]	Neonatal [1.15]	Asthma [1.41]	Congenital [0.84]	Anxiety [1.03]	Iron [3.96]	Conduct [1.0]	Skin Viral [0.89]	URI [1.26]	Falls [0.84]
Nottinghamshire	Dermatitis [1.44]	Neonatal [1.03]	Asthma [1.27]	Congenital [0.69]	Anxiety [1.08]	Iron [2.48]	Conduct [0.97]	Skin Viral [0.87]	URI [1.31]	Falls [0.9]
Rutland	Dermatitis [1.44]	Asthma [1.29]	Iron [3.92]	Neonatal [0.66]	Congenital [0.72]	Anxiety [1.07]	Conduct [0.97]	Skin Viral [0.88]	URI [1.3]	Falls [0.87]
E England	Dermatitis [1.44]	Neonatal [0.9]	Asthma [1.33]	Congenital [0.76]	Anxiety [1.06]	Iron [2.97]	Conduct [0.98]	Skin Viral [0.88]	URI [1.29]	Falls [0.88]
Bedford	Dermatitis [1.44]	Neonatal [1.1]	Asthma [1.32]	Congenital [0.76]	Iron [3.44]	Anxiety [1.06]	Conduct [0.98]	Skin Viral [0.88]	URI [1.27]	Falls [0.87]
Cambridgeshire	Dermatitis [1.43]	Neonatal [0.92]	Asthma [1.41]	Congenital [0.88]	Anxiety [1.03]	Conduct [1.0]	Skin Viral [0.89]	URI [1.27]	ASD [1.65]	Iron [2.92]
Cen Bedfordshire	Dermatitis [1.44]	Neonatal [0.98]	Asthma [1.3]	Congenital [0.7]	Anxiety [1.07]	Conduct [0.97]	Skin Viral [0.88]	Iron [2.51]	URI [1.3]	Falls [0.88]
Essex	Dermatitis [1.44]	Neonatal [0.89]	Asthma [1.29]	Congenital [0.7]	Anxiety [1.08]	Conduct [0.97]	Skin Viral [0.88]	URI [1.3]	Iron [2.3]	Falls [0.88]
Hertfordshire	Dermatitis [1.43]	Neonatal [0.89]	Asthma [1.39]	Congenital [0.78]	Anxiety [1.03]	Conduct [1.0]	Iron [3.54]	Skin Viral [0.89]	URI [1.27]	Falls [0.83]
Luton	Dermatitis [1.43]	Neonatal [1.02]	Asthma [1.37]	Congenital [0.9]	Anxiety [1.06]	Conduct [0.99]	Skin Viral [0.87]	URI [1.28]	Iron [2.29]	Falls [0.93]
Norfolk	Dermatitis [1.44]	Asthma [1.3]	Neonatal [0.76]	Congenital [0.74]	Iron [2.99]	Anxiety [1.07]	Conduct [0.98]	Skin Viral [0.87]	URI [1.3]	Falls [0.91]
Peterborough	Dermatitis [1.44]	Asthma [1.35]	Neonatal [0.71]	Congenital [0.81]	Iron [2.94]	Anxiety [1.07]	Conduct [0.98]	Skin Viral [0.87]	URI [1.29]	Falls [0.94]
Southend-on-Sea	Dermatitis [1.44]	Neonatal [1.06]	Asthma [1.3]	Congenital [0.85]	Iron [2.78]	Anxiety [1.09]	Conduct [0.97]	Skin Viral [0.87]	URI [1.31]	Falls [0.95]
Suffolk	Dermatitis [1.44]	Neonatal [0.94]	Asthma [1.28]	Iron [3.15]	Congenital [0.71]	Anxiety [1.07]	Conduct [0.97]	Skin Viral [0.87]	URI [1.31]	Falls [0.91]
Thurrock	Dermatitis [1.45]	Neonatal [0.99]	Asthma [1.29]	Congenital [0.69]	Iron [2.41]	Anxiety [1.1]	Conduct [0.96]	Skin Viral [0.87]	URI [1.32]	Falls [0.92]
Greater London	Dermatitis [1.46]	Asthma [1.75]	Neonatal [1.05]	Iron [6.32]	Congenital [0.93]	Anxiety [1.02]	Conduct [1.01]	Skin Viral [0.91]	URI [1.24]	Falls [0.8]
Barking & Dagenham	Dermatitis [1.87]	Iron [4.98]	Asthma [1.49]	Neonatal [0.98]	Congenital [0.7]	Anxiety [1.09]	Conduct [0.97]	Skin Viral [0.86]	URI [1.31]	Falls [0.93]
Barnet	Dermatitis [1.43]	Asthma [1.63]	Neonatal [0.97]	Iron [5.0]	Congenital [0.8]	Anxiety [1.04]	Conduct [0.99]	Skin Viral [0.89]	URI [1.27]	Falls [0.84]
Bexley	Dermatitis [1.43]	Asthma [1.55]	Neonatal [0.92]	Iron [4.25]	Congenital [0.76]	Anxiety [1.07]	Conduct [0.97]	Skin Viral [0.87]	URI [1.31]	Falls [0.91]
Brent	Dermatitis [1.43]	Asthma [1.63]	Neonatal [1.03]	Iron [5.4]	Congenital [0.82]	Anxiety [1.04]	Conduct [0.99]	Skin Viral [0.88]	URI [1.26]	Falls [0.86]
Bromley	Dermatitis [1.44]	Neonatal [1.1]	Asthma [1.6]	Congenital [0.82]	Anxiety [1.05]	Conduct [0.98]	Skin Viral [0.89]	URI [1.29]	Falls [0.87]	Iron [2.14]
Camden	Dermatitis [1.46]	Asthma [1.88]	Neonatal [0.92]	Congenital [1.05]	Anxiety [1.0]	Iron [6.63]	Conduct [1.04]	Skin Viral [0.94]	URI [1.24]	Falls [0.79]
Croydon	Dermatitis [1.44]	Asthma [1.56]	Neonatal [0.98]	Iron [4.66]	Congenital [0.71]	Anxiety [1.06]	Conduct [0.97]	Skin Viral [0.88]	URI [1.29]	Falls [0.87]
Ealing	Dermatitis [1.43]	Neonatal [1.11]	Asthma [1.64]	Iron [6.63]	Anxiety [1.04]	Congenital [0.76]	Conduct [0.99]	Skin Viral [0.89]	URI [1.26]	Falls [0.83]
Enfield	Dermatitis [1.44]	Asthma [1.59]	Neonatal [1.03]	Iron [4.61]	Congenital [0.83]	Anxiety [1.06]	Conduct [0.98]	Skin Viral [0.88]	URI [1.29]	Falls [0.89]
Greenwich	Dermatitis [1.43]	Asthma [1.54]	Neonatal [0.95]	Iron [3.76]	Anxiety [1.06]	Congenital [0.69]	Conduct [0.98]	Skin Viral [0.87]	URI [1.28]	Falls [0.85]
Hackney	Dermatitis [1.45]	Asthma [1.8]	Neonatal [1.02]	Congenital [0.93]	Anxiety [1.03]	Iron [4.28]	Conduct [0.99]	Skin Viral [0.91]	URI [1.24]	Falls [0.83]
Hammersmith & Fulham	Dermatitis [1.46]	Asthma [1.85]	Neonatal [1.03]	Congenital [0.94]	Anxiety [1.01]	Conduct [1.03]	Skin Viral [0.94]	URI [1.23]	Falls [0.76]	Iron [3.1]

eFigure 7d. Leading ten causes of DALYs with the ratio of observed DALYs to DALYs expected on the basis of Socio-Demographic Index alone in 2017, 5-9 years, both sexes combined. The top ten causes contributing to DALYs are listed globally, by socio-demographic quintile, and then by GBD superregion, region, country, and subnationally where modeled. For each cell, the ratio of observed DALYs to DALYs expected on the basis of socio-demographic index (SDI) alone are listed. Abbreviations: DALY=disability-adjusted life year, GBD=Global Burden of Disease.

Values shown in brackets represent the ratio of observed DALYs to predicted DALYs on the basis of Socio-Demographic Index (SDI), rounded to two (2) digits. Color ranges (shown below) were calculated to place a roughly equal number of cells into each bin.

COLOR KEY:	[0.0-0.62]	(0.62-0.79]	(0.79-0.89]	(0.89-0.97]	(0.97-1.07]	(1.07-1.26]	(1.26-1.44]	(1.44-2.32]	2.32+

	1	2	3	4	5	6	7	8	9	10
Haringey	Dermatitis [1.44]	Neonatal [1.08]	Asthma [1.61]	Iron [4.5]	Congenital [0.79]	Anxiety [1.05]	Conduct [0.98]	Skin Viral [0.89]	URI [1.26]	Falls [0.84]
Harrow	Dermatitis [1.44]	Neonatal [1.12]	Asthma [1.58]	Iron [5.13]	Congenital [0.8]	Anxiety [1.05]	Conduct [0.98]	Skin Viral [0.89]	URI [1.28]	Falls [0.85]
Havering	Dermatitis [1.44]	Asthma [1.54]	Neonatal [0.97]	Congenital [0.74]	Iron [3.14]	Anxiety [1.07]	Conduct [0.97]	Skin Viral [0.87]	URI [1.31]	Falls [0.9]
Hillingdon	Dermatitis [1.44]	Neonatal [1.15]	Asthma [1.79]	Congenital [1.25]	Iron [5.48]	Anxiety [1.03]	Other MN [3.5]	Conduct [1.0]	Brain C [1.48]	Skin Viral [0.9]
Hounslow	Dermatitis [1.44]	Neonatal [1.09]	Asthma [1.7]	Congenital [0.88]	Iron [5.0]	Anxiety [1.03]	Conduct [1.0]	Skin Viral [0.9]	URI [1.25]	Falls [0.83]
Islington	Dermatitis [1.45]	Asthma [1.89]	Neonatal [0.89]	Congenital [1.08]	Anxiety [1.01]	Conduct [1.03]	Skin Viral [0.94]	URI [1.23]	Iron [4.37]	Falls [0.8]
Kensington & Chelsea	Dermatitis [1.45]	Asthma [1.86]	Neonatal [0.89]	Iron [8.37]	Congenital [0.92]	Anxiety [0.99]	Conduct [1.05]	Skin Viral [0.94]	URI [1.23]	Falls [0.76]
Kingston upon Thames	Dermatitis [1.44]	Neonatal [1.09]	Asthma [1.73]	Congenital [0.9]	Anxiety [1.02]	Conduct [1.0]	Iron [4.1]	Skin Viral [0.91]	URI [1.26]	Falls [0.8]
Lambeth	Dermatitis [1.44]	Asthma [1.76]	Iron [7.47]	Neonatal [0.75]	Congenital [0.94]	Anxiety [1.02]	Conduct [1.01]	Skin Viral [0.92]	URI [1.23]	Falls [0.79]
Lewisham	Dermatitis [1.43]	Neonatal [1.18]	Asthma [1.58]	Iron [3.96]	Congenital [0.73]	Anxiety [1.06]	Conduct [0.98]	Skin Viral [0.88]	URI [1.27]	Falls [0.85]
Merton	Dermatitis [1.43]	Asthma [1.68]	Neonatal [1.04]	Iron [5.71]	Congenital [0.84]	Anxiety [1.03]	Conduct [0.99]	Skin Viral [0.9]	URI [1.26]	Falls [0.82]
Newham	Dermatitis [1.44]	Neonatal [1.06]	Asthma [1.61]	Congenital [0.8]	Anxiety [1.06]	Iron [3.29]	Conduct [0.98]	Skin Viral [0.88]	URI [1.27]	Falls [0.86]
Redbridge	Dermatitis [1.44]	Asthma [1.55]	Neonatal [0.98]	Iron [4.4]	Congenital [0.79]	Anxiety [1.06]	Conduct [0.98]	Skin Viral [0.87]	URI [1.29]	Falls [0.89]
Richmond upon Thames	Dermatitis [1.44]	Neonatal [1.15]	Asthma [1.76]	Congenital [1.08]	Iron [6.01]	Anxiety [1.02]	Conduct [1.01]	Skin Viral [0.91]	URI [1.26]	Other MN [2.77]
Southwark	Dermatitis [1.45]	Asthma [1.84]	Neonatal [0.77]	Congenital [1.01]	Anxiety [1.01]	Iron [5.39]	Conduct [1.02]	Skin Viral [0.92]	URI [1.23]	Falls [0.82]
Sutton	Dermatitis [1.43]	Neonatal [1.25]	Asthma [1.6]	Iron [4.31]	Congenital [0.82]	Anxiety [1.05]	Conduct [0.98]	Skin Viral [0.88]	URI [1.28]	Falls [0.87]
Tower Hamlets	Dermatitis [1.45]	Neonatal [1.13]	Asthma [1.84]	Congenital [1.02]	Anxiety [1.01]	Iron [5.16]	Conduct [1.01]	Skin Viral [0.92]	URI [1.23]	Falls [0.81]
Waltham Forest	Dermatitis [1.44]	Asthma [1.53]	Neonatal [0.99]	Iron [4.15]	Congenital [0.76]	Anxiety [1.07]	Conduct [0.97]	Skin Viral [0.87]	URI [1.28]	Falls [0.91]
Wandsworth	Dermatitis [1.45]	Asthma [1.77]	Neonatal [0.92]	Congenital [0.91]	Anxiety [1.01]	Iron [5.41]	Conduct [1.02]	Skin Viral [0.93]	URI [1.24]	Falls [0.77]
Westminster	Dermatitis [1.46]	Asthma [1.88]	Neonatal [0.83]	Iron [6.92]	Congenital [0.92]	Anxiety [1.0]	Conduct [1.04]	Skin Viral [0.94]	URI [1.22]	Falls [0.77]
NE England	Dermatitis [1.55]	Iron [4.59]	Neonatal [0.88]	Asthma [1.3]	Congenital [0.84]	Anxiety [1.07]	Conduct [0.97]	Skin Viral [0.87]	URI [1.3]	Falls [0.9]
County Durham	Dermatitis [1.72]	Iron [4.73]	Neonatal [0.95]	Asthma [1.29]	Congenital [0.79]	Anxiety [1.09]	Conduct [0.96]	Skin Viral [0.87]	URI [1.31]	Falls [0.91]
Darlington	Dermatitis [1.43]	Iron [5.15]	Asthma [1.33]	Neonatal [0.86]	Congenital [0.89]	Anxiety [1.06]	Conduct [0.98]	Skin Viral [0.87]	URI [1.3]	Falls [0.9]
Gateshead	Dermatitis [1.69]	Iron [5.85]	Neonatal [0.98]	Asthma [1.32]	Congenital [0.85]	Anxiety [1.07]	Conduct [0.96]	Skin Viral [0.87]	URI [1.29]	Falls [0.9]
Hartlepool	Dermatitis [1.45]	Neonatal [1.0]	Asthma [1.29]	Iron [2.77]	Congenital [0.79]	Anxiety [1.1]	Conduct [0.97]	Skin Viral [0.86]	URI [1.31]	Falls [0.92]
Middlesbrough	Dermatitis [1.44]	Asthma [1.33]	Congenital [0.94]	Neonatal [0.77]	Iron [3.06]	Anxiety [1.09]	Conduct [0.97]	Skin Viral [0.87]	URI [1.3]	Falls [0.94]
Newcastle upon Tyne	Dermatitis [1.43]	Asthma [1.42]	Neonatal [0.84]	Congenital [0.91]	Iron [5.02]	Anxiety [1.03]	Conduct [1.0]	Skin Viral [0.89]	URI [1.26]	Falls [0.81]
N Tyneside	Dermatitis [1.43]	Asthma [1.32]	Iron [4.64]	Neonatal [0.82]	Congenital [0.87]	Anxiety [1.07]	Conduct [0.98]	Skin Viral [0.87]	URI [1.29]	Falls [0.93]
Northumberland	Dermatitis [1.44]	Neonatal [0.85]	Asthma [1.28]	Iron [3.64]	Congenital [0.77]	Anxiety [1.08]	Conduct [0.98]	Skin Viral [0.86]	URI [1.31]	Falls [0.91]
Redcar & Cleveland	Dermatitis [1.45]	Iron [3.71]	Asthma [1.29]	Congenital [0.95]	Neonatal [0.8]	Anxiety [1.1]	Conduct [0.98]	Skin Viral [0.86]	URI [1.33]	Falls [1.0]
S Tyneside	Dermatitis [1.73]	Iron [3.29]	Asthma [1.27]	Neonatal [0.84]	Congenital [0.79]	Anxiety [1.1]	Conduct [0.96]	Skin Viral [0.87]	URI [1.33]	Falls [0.94]

eFigure 7d. Leading ten causes of DALYs with the ratio of observed DALYs to DALYs expected on the basis of Socio-Demographic Index alone in 2017, 5-9 years, both sexes combined. The top ten causes contributing to DALYs are listed globally, by socio-demographic quintile, and then by GBD superregion, region, country, and subnationally where modeled. For each cell, the ratio of observed DALYs to DALYs expected on the basis of socio-demographic index (SDI) alone are listed. Abbreviations: DALY=disability-adjusted life year, GBD=Global Burden of Disease.

Values shown in brackets represent the ratio of observed DALYs to predicted DALYs on the basis of Socio-Demographic Index (SDI), rounded to two (2) digits. Color ranges (shown below) were calculated to place a roughly equal number of cells into each bin.

COLOR KEY:	[0.0-0.62]	(0.62-0.79]	(0.79-0.89]	(0.89-0.97]	(0.97-1.07]	(1.07-1.26]	(1.26-1.44]	(1.44-2.32]	2.32+

	1	2	3	4	5	6	7	8	9	10
Stockton-on-Tees	Dermatitis [1.44]	Neonatal [1.07]	Asthma [1.34]	Iron [4.7]	Congenital [0.87]	Anxiety [1.08]	Conduct [0.97]	Skin Viral [0.87]	URI [1.3]	Falls [0.9]
Sunderland	Dermatitis [1.71]	Iron [4.99]	Asthma [1.15]	Neonatal [0.75]	Congenital [0.83]	Anxiety [1.08]	Conduct [0.97]	Skin Viral [0.87]	URI [1.3]	Falls [0.91]
NW England	Dermatitis [1.44]	Neonatal [1.09]	Asthma [1.36]	Congenital [0.9]	Iron [3.3]	Anxiety [1.06]	Conduct [0.98]	Skin Viral [0.88]	URI [1.28]	Falls [0.91]
Blackburn with Darwen	Dermatitis [1.45]	Neonatal [1.07]	Asthma [1.33]	Congenital [0.88]	Iron [2.41]	Anxiety [1.1]	Conduct [0.97]	Skin Viral [0.87]	URI [1.3]	Falls [0.96]
Blackpool	Dermatitis [1.46]	Neonatal [1.03]	Asthma [1.27]	Iron [2.14]	Congenital [0.68]	Anxiety [1.12]	Conduct [0.97]	Skin Viral [0.87]	URI [1.33]	Falls [0.98]
Bolton	Dermatitis [1.44]	Neonatal [1.02]	Asthma [1.32]	Congenital [0.91]	Iron [2.58]	Anxiety [1.09]	Conduct [0.97]	Skin Viral [0.87]	URI [1.3]	Falls [0.97]
Bury	Dermatitis [1.44]	Neonatal [0.98]	Asthma [1.29]	Iron [3.34]	Congenital [0.83]	Anxiety [1.07]	Conduct [0.98]	Skin Viral [0.87]	URI [1.29]	Falls [0.93]
Cheshire E	Dermatitis [1.43]	Neonatal [1.35]	Asthma [1.42]	Congenital [1.03]	Anxiety [1.03]	Conduct [1.0]	Skin Viral [0.89]	URI [1.27]	Iron [3.15]	Falls [0.85]
Cheshire W & Chester	Dermatitis [1.44]	Neonatal [1.11]	Asthma [1.39]	Congenital [1.03]	Anxiety [1.05]	Iron [3.29]	Conduct [0.99]	Skin Viral [0.89]	URI [1.27]	Falls [0.88]
Cumbria	Dermatitis [1.43]	Neonatal [1.08]	Asthma [1.31]	Congenital [0.77]	Iron [3.23]	Anxiety [1.07]	Conduct [0.97]	Skin Viral [0.87]	URI [1.29]	Falls [0.89]
Halton	Dermatitis [1.44]	Neonatal [1.11]	Asthma [1.38]	Congenital [1.05]	Iron [3.56]	Anxiety [1.08]	Conduct [0.97]	Skin Viral [0.87]	URI [1.29]	Falls [0.96]
Knowsley	Dermatitis [1.44]	Neonatal [1.05]	Asthma [1.33]	Congenital [0.98]	Iron [3.6]	Anxiety [1.08]	Conduct [0.97]	Skin Viral [0.87]	URI [1.29]	Falls [0.95]
Lancashire	Dermatitis [1.43]	Neonatal [1.05]	Asthma [1.35]	Congenital [0.91]	Iron [3.21]	Anxiety [1.07]	Conduct [0.98]	Skin Viral [0.87]	URI [1.29]	Falls [0.93]
Liverpool	Dermatitis [1.44]	Neonatal [1.19]	Asthma [1.39]	Congenital [1.06]	Anxiety [0.97]	Conduct [0.98]	Skin Viral [0.88]	URI [1.27]	Falls [0.91]	Iron [2.39]
Manchester	Dermatitis [1.44]	Neonatal [1.2]	Asthma [1.5]	Congenital [0.99]	Anxiety [1.02]	Conduct [1.0]	Skin Viral [0.91]	URI [1.24]	Falls [0.84]	Iron [3.07]
Oldham	Dermatitis [1.45]	Asthma [1.32]	Neonatal [0.8]	Congenital [0.77]	Iron [2.06]	Anxiety [1.12]	Conduct [0.96]	Skin Viral [0.87]	URI [1.32]	Falls [0.95]
Rochdale	Dermatitis [1.44]	Iron [3.26]	Asthma [1.28]	Neonatal [0.83]	Congenital [0.68]	Anxiety [1.1]	Conduct [0.97]	Skin Viral [0.86]	URI [1.32]	Falls [0.92]
Salford	Dermatitis [1.44]	Neonatal [1.22]	Asthma [1.38]	Congenital [0.92]	Anxiety [1.06]	Conduct [0.98]	Skin Viral [0.88]	URI [1.27]	Falls [0.91]	Iron [2.18]
Sefton	Dermatitis [1.44]	Neonatal [1.08]	Asthma [1.29]	Congenital [0.86]	Iron [3.1]	Anxiety [1.08]	Conduct [0.97]	Skin Viral [0.86]	URI [1.31]	Falls [0.94]
St Helens	Dermatitis [1.44]	Neonatal [1.09]	Asthma [1.28]	Congenital [0.84]	Iron [2.86]	Anxiety [1.09]	Conduct [0.96]	Skin Viral [0.87]	URI [1.3]	Falls [0.94]
Stockport	Dermatitis [1.43]	Neonatal [0.92]	Asthma [1.34]	Congenital [0.84]	Iron [3.93]	Anxiety [1.05]	Conduct [0.99]	Skin Viral [0.88]	URI [1.29]	Falls [0.89]
Tameside	Dermatitis [1.44]	Neonatal [0.89]	Asthma [1.3]	Congenital [0.83]	Iron [2.32]	Anxiety [1.1]	Conduct [0.97]	Skin Viral [0.87]	URI [1.32]	Falls [0.98]
Trafford	Dermatitis [1.44]	Neonatal [1.21]	Asthma [1.44]	Congenital [0.96]	Iron [4.83]	Anxiety [1.03]	Conduct [1.0]	Skin Viral [0.9]	URI [1.25]	Falls [0.85]
Warrington	Dermatitis [1.43]	Neonatal [1.28]	Asthma [1.41]	Iron [5.78]	Congenital [0.9]	Anxiety [1.04]	Conduct [0.99]	Skin Viral [0.89]	URI [1.26]	Falls [0.85]
Wigan	Dermatitis [1.44]	Neonatal [1.08]	Asthma [1.29]	Congenital [0.78]	Iron [2.34]	Anxiety [1.09]	Conduct [0.97]	Skin Viral [0.86]	URI [1.33]	Falls [0.95]
Wirral	Dermatitis [1.44]	Neonatal [1.21]	Asthma [1.29]	Congenital [0.89]	Anxiety [1.09]	Iron [1.98]	Conduct [0.97]	Skin Viral [0.87]	URI [1.31]	Falls [0.93]
SE England	Dermatitis [1.57]	Neonatal [1.02]	Asthma [1.47]	Congenital [0.8]	Anxiety [1.05]	Iron [3.57]	Conduct [0.99]	Skin Viral [0.89]	URI [1.28]	Falls [0.85]
Bracknell Forest	Dermatitis [1.43]	Asthma [1.38]	Neonatal [0.8]	Congenital [0.89]	Anxiety [1.04]	Conduct [1.0]	Skin Viral [0.9]	URI [1.27]	Falls [0.85]	Iron [2.17]
Brighton & Hove	Dermatitis [1.7]	Neonatal [1.12]	Asthma [1.46]	Congenital [1.1]	Iron [5.15]	Anxiety [1.03]	Conduct [1.0]	Skin Viral [0.91]	URI [1.24]	Falls [0.86]
Buckinghamshire	Dermatitis [1.43]	Neonatal [1.15]	Asthma [1.36]	Congenital [0.87]	Anxiety [1.04]	Iron [3.68]	Conduct [0.99]	Skin Viral [0.89]	URI [1.28]	Epilepsy [1.62]
E Sussex	Dermatitis [1.67]	Neonatal [1.06]	Asthma [1.22]	Congenital [0.76]	Iron [2.83]	Anxiety [1.08]	Conduct [0.98]	Skin Viral [0.86]	URI [1.31]	Falls [0.91]

eFigure 7d. Leading ten causes of DALYs with the ratio of observed DALYs to DALYs expected on the basis of Socio-Demographic Index alone in 2017, 5-9 years, both sexes combined. The top ten causes contributing to DALYs are listed globally, by socio-demographic quintile, and then by GBD superregion, region, country, and subnationally where modeled. For each cell, the ratio of observed DALYs to DALYs expected on the basis of socio-demographic index (SDI) alone are listed. Abbreviations: DALY=disability-adjusted life year, GBD=Global Burden of Disease.

Values shown in brackets represent the ratio of observed DALYs to predicted DALYs on the basis of Socio-Demographic Index (SDI), rounded to two (2) digits. Color ranges (shown below) were calculated to place a roughly equal number of cells into each bin.

COLOR KEY:	[0.0-0.62]	(0.62-0.79]	(0.79-0.89]	(0.89-0.97]	(0.97-1.07]	(1.07-1.26]	(1.26-1.44]	(1.44-2.32]	2.32+

	1	2	3	4	5	6	7	8	9	10
Hampshire	Dermatitis [1.43]	Asthma [2.06]	Neonatal [0.99]	Iron [3.84]	Congenital [0.73]	Anxiety [1.05]	Conduct [0.98]	Skin Viral [0.88]	URI [1.29]	Falls [0.85]
Isle of Wight	Dermatitis [1.41]	Asthma [1.96]	Neonatal [0.92]	Iron [3.33]	Congenital [0.69]	Anxiety [1.08]	Conduct [0.97]	Skin Viral [0.87]	URI [1.3]	Falls [0.9]
Kent	Dermatitis [1.67]	Neonatal [0.98]	Asthma [1.26]	Congenital [0.7]	Anxiety [1.07]	Iron [2.67]	Conduct [0.98]	Skin Viral [0.87]	URI [1.3]	ASD [1.55]
Medway	Dermatitis [1.44]	Neonatal [1.03]	Asthma [1.26]	Iron [2.98]	Congenital [0.78]	Anxiety [1.09]	Conduct [0.96]	Skin Viral [0.87]	URI [1.31]	Falls [0.92]
Milton Keynes	Dermatitis [1.43]	Asthma [1.41]	Neonatal [0.88]	Congenital [0.93]	Anxiety [1.04]	Conduct [0.99]	Skin Viral [0.89]	URI [1.27]	Iron [2.87]	Falls [0.9]
Oxfordshire	Dermatitis [1.87]	Neonatal [0.94]	Asthma [1.39]	Iron [5.55]	Congenital [0.85]	Anxiety [1.03]	Conduct [0.99]	Skin Viral [0.9]	URI [1.26]	Falls [0.81]
Portsmouth	Dermatitis [1.44]	Neonatal [0.97]	Asthma [1.33]	Iron [4.95]	Anxiety [1.05]	Congenital [0.74]	Conduct [0.99]	Skin Viral [0.89]	URI [1.26]	Falls [0.84]
Reading	Dermatitis [1.87]	Neonatal [1.22]	Asthma [1.47]	Congenital [0.93]	Anxiety [1.02]	Conduct [1.01]	Skin Viral [0.91]	URI [1.24]	Iron [3.78]	Falls [0.81]
Slough	Dermatitis [1.43]	Neonatal [1.06]	Asthma [1.43]	Congenital [0.89]	Anxiety [1.05]	Conduct [0.99]	Skin Viral [0.89]	URI [1.26]	Falls [0.86]	Iron [2.41]
Southampton	Dermatitis [1.43]	Neonatal [0.96]	Asthma [1.34]	Iron [4.07]	Congenital [0.75]	Anxiety [1.04]	Conduct [0.99]	Skin Viral [0.89]	URI [1.27]	Falls [0.84]
Surrey	Dermatitis [1.66]	Neonatal [1.11]	Asthma [1.4]	Congenital [0.88]	Anxiety [1.02]	Conduct [1.0]	Skin Viral [0.91]	URI [1.27]	Falls [0.81]	Iron [2.81]
West Berkshire	Dermatitis [1.43]	Neonatal [0.93]	Asthma [1.39]	Congenital [0.88]	Anxiety [1.04]	Iron [3.72]	Conduct [1.0]	Skin Viral [0.89]	URI [1.26]	Falls [0.84]
W Sussex	Dermatitis [1.48]	Neonatal [1.02]	Asthma [1.51]	Iron [4.18]	Congenital [0.73]	Anxiety [1.05]	Conduct [0.99]	Skin Viral [0.88]	URI [1.29]	Falls [0.85]
Windsor & Maidenhead	Dermatitis [1.44]	Neonatal [0.97]	Asthma [1.43]	Congenital [0.87]	Anxiety [1.02]	Conduct [1.02]	Skin Viral [0.9]	URI [1.25]	Iron [3.45]	Falls [0.8]
Wokingham	Dermatitis [1.44]	Neonatal [1.0]	Asthma [1.41]	Congenital [0.92]	Anxiety [1.02]	Iron [4.25]	Conduct [1.01]	Skin Viral [0.91]	URI [1.27]	Falls [0.83]
SW England	Dermatitis [1.43]	Neonatal [1.05]	Iron [5.7]	Asthma [1.3]	Congenital [0.73]	Anxiety [1.06]	Conduct [0.98]	Skin Viral [0.88]	URI [1.29]	Falls [0.88]
Bath & NE Somerset	Dermatitis [1.43]	Neonatal [1.41]	Iron [8.02]	Asthma [1.37]	Anxiety [1.03]	Congenital [0.75]	Conduct [1.0]	Skin Viral [0.89]	URI [1.26]	Falls [0.82]
Bournemouth	Dermatitis [1.44]	Iron [7.82]	Neonatal [1.04]	Asthma [1.34]	Anxiety [1.05]	Congenital [0.72]	Conduct [0.98]	Skin Viral [0.89]	URI [1.27]	Falls [0.84]
Bristol	Dermatitis [1.44]	Neonatal [1.22]	Iron [8.25]	Asthma [1.42]	Anxiety [1.03]	Congenital [0.75]	Conduct [1.0]	Skin Viral [0.9]	URI [1.25]	Falls [0.79]
Cornwall	Dermatitis [1.44]	Iron [6.67]	Neonatal [1.03]	Asthma [1.26]	Congenital [0.7]	Anxiety [1.08]	Conduct [0.97]	Skin Viral [0.87]	URI [1.3]	Falls [0.93]
Devon	Dermatitis [1.43]	Neonatal [0.95]	Asthma [1.28]	Iron [4.2]	Congenital [0.72]	Anxiety [1.06]	Conduct [0.98]	Skin Viral [0.87]	URI [1.29]	Falls [0.9]
Dorset	Dermatitis [1.44]	Iron [5.78]	Neonatal [1.01]	Asthma [1.28]	Congenital [0.72]	Anxiety [1.07]	Conduct [0.97]	Skin Viral [0.87]	URI [1.29]	Falls [0.91]
Gloucestershire	Dermatitis [1.43]	Neonatal [0.97]	Asthma [1.33]	Iron [3.94]	Congenital [0.78]	Anxiety [1.06]	Conduct [0.98]	Skin Viral [0.88]	URI [1.28]	Falls [0.88]
N Somerset	Dermatitis [1.43]	Neonatal [1.13]	Iron [6.29]	Asthma [1.3]	Congenital [0.81]	Anxiety [1.06]	Conduct [0.98]	Skin Viral [0.87]	URI [1.29]	Falls [0.89]
Plymouth	Dermatitis [1.44]	Neonatal [1.04]	Asthma [1.31]	Iron [4.17]	Congenital [0.81]	Anxiety [1.06]	Conduct [0.97]	Skin Viral [0.88]	URI [1.28]	Falls [0.91]
Poole	Dermatitis [1.43]	Neonatal [1.03]	Iron [5.41]	Asthma [1.3]	Anxiety [1.05]	Congenital [0.69]	Conduct [0.99]	Skin Viral [0.87]	URI [1.29]	Falls [0.87]
Somerset	Dermatitis [1.44]	Neonatal [1.09]	Asthma [1.24]	Anxiety [1.09]	Iron [2.52]	Congenital [0.62]	Conduct [0.97]	Skin Viral [0.87]	URI [1.31]	Falls [0.91]
S Gloucestershire	Dermatitis [1.44]	Neonatal [1.1]	Asthma [1.37]	Iron [5.57]	Congenital [0.81]	Anxiety [1.04]	Conduct [0.99]	Skin Viral [0.89]	URI [1.27]	Falls [0.83]
Swindon	Dermatitis [1.43]	Iron [8.33]	Asthma [1.35]	Neonatal [0.75]	Congenital [0.83]	Anxiety [1.05]	Conduct [0.98]	Skin Viral [0.88]	URI [1.29]	Brain C [1.05]
Torbay	Dermatitis [1.45]	Neonatal [0.85]	Iron [2.96]	Asthma [1.23]	Congenital [0.62]	Anxiety [1.11]	Conduct [0.97]	Skin Viral [0.87]	URI [1.34]	Falls [0.96]
Wiltshire	Dermatitis [1.43]	Iron [7.49]	Neonatal [1.2]	Asthma [1.27]	Anxiety [1.07]	Congenital [0.68]	Conduct [0.97]	Skin Viral [0.87]	URI [1.29]	Falls [0.88]

eFigure 7d. Leading ten causes of DALYs with the ratio of observed DALYs to DALYs expected on the basis of Socio-Demographic Index alone in 2017, 5-9 years, both sexes combined. The top ten causes contributing to DALYs are listed globally, by socio-demographic quintile, and then by GBD superregion, region, country, and subnationally where modeled. For each cell, the ratio of observed DALYs to DALYs expected on the basis of socio-demographic index (SDI) alone are listed. Abbreviations: DALY=disability-adjusted life year, GBD=Global Burden of Disease.

Values shown in brackets represent the ratio of observed DALYs to predicted DALYs on the basis of Socio-Demographic Index (SDI), rounded to two (2) digits. Color ranges (shown below) were calculated to place a roughly equal number of cells into each bin.

COLOR KEY:	[0.0-0.62]	(0.62-0.79]	(0.79-0.89]	(0.89-0.97]	(0.97-1.07]	(1.07-1.26]	(1.26-1.44]	(1.44-2.32]	2.32+

	1	2	3	4	5	6	7	8	9	10
W Midlands	Dermatitis [1.44]	Neonatal [0.98]	Asthma [1.33]	Congenital [0.83]	Iron [3.62]	Anxiety [1.07]	Conduct [0.98]	Skin Viral [0.87]	URI [1.29]	Falls [0.94]
Birmingham	Dermatitis [1.44]	Neonatal [0.96]	Asthma [1.35]	Congenital [0.89]	Iron [3.55]	Anxiety [1.06]	Conduct [0.98]	Skin Viral [0.88]	URI [1.28]	Falls [0.97]
Coventry	Dermatitis [1.44]	Neonatal [0.93]	Asthma [1.38]	Congenital [0.83]	Anxiety [1.05]	Iron [3.23]	Conduct [0.98]	Skin Viral [0.88]	URI [1.26]	Falls [0.86]
Dudley	Dermatitis [1.45]	Neonatal [1.07]	Asthma [1.31]	Congenital [0.89]	Iron [2.44]	Anxiety [1.1]	Conduct [0.96]	Skin Viral [0.87]	URI [1.31]	Falls [1.0]
Herefordshire	Dermatitis [1.43]	Neonatal [1.01]	Asthma [1.29]	Iron [3.93]	Congenital [0.71]	Anxiety [1.07]	Conduct [0.98]	Skin Viral [0.87]	URI [1.29]	Falls [0.88]
Sandwell	Dermatitis [1.45]	Neonatal [1.06]	Asthma [1.32]	Iron [2.69]	Congenital [0.81]	Anxiety [1.1]	Conduct [0.97]	Skin Viral [0.86]	URI [1.32]	Falls [1.0]
Shropshire	Dermatitis [1.44]	Neonatal [0.98]	Asthma [1.29]	Congenital [0.77]	Anxiety [1.07]	Iron [2.82]	Conduct [0.97]	Skin Viral [0.87]	URI [1.29]	Falls [0.89]
Solihull	Dermatitis [1.43]	Asthma [1.36]	Neonatal [0.72]	Congenital [0.86]	Iron [3.96]	Anxiety [1.05]	Conduct [0.99]	Skin Viral [0.89]	URI [1.28]	Falls [0.91]
Staffordshire	Dermatitis [1.44]	Neonatal [0.95]	Iron [4.87]	Asthma [1.3]	Congenital [0.79]	Anxiety [1.07]	Conduct [0.98]	Skin Viral [0.87]	URI [1.3]	Falls [0.97]
Stoke-on-Trent	Dermatitis [1.45]	Neonatal [1.17]	Asthma [1.31]	Congenital [0.88]	Iron [2.63]	Anxiety [1.09]	Conduct [0.96]	Skin Viral [0.87]	URI [1.3]	Falls [1.09]
Telford & Wrekin	Dermatitis [1.44]	Neonatal [0.99]	Asthma [1.32]	Congenital [0.89]	Anxiety [1.08]	Iron [2.59]	Conduct [0.96]	Skin Viral [0.87]	URI [1.3]	Falls [0.95]
Walsall	Dermatitis [1.45]	Neonatal [0.98]	Asthma [1.28]	Iron [2.26]	Congenital [0.7]	Anxiety [1.1]	Conduct [0.97]	Skin Viral [0.86]	URI [1.32]	Falls [0.98]
Warwickshire	Dermatitis [1.44]	Neonatal [1.0]	Asthma [1.37]	Iron [4.97]	Congenital [0.82]	Anxiety [1.05]	Conduct [0.99]	Skin Viral [0.89]	URI [1.28]	Falls [0.88]
Wolverhampton	Dermatitis [1.44]	Asthma [1.27]	Neonatal [0.72]	Congenital [0.7]	Iron [2.44]	Anxiety [1.09]	Conduct [0.97]	Skin Viral [0.87]	URI [1.3]	Falls [0.92]
Worcestershire	Dermatitis [1.44]	Neonatal [1.1]	Asthma [1.32]	Congenital [0.81]	Iron [3.6]	Anxiety [1.07]	Conduct [0.98]	Skin Viral [0.88]	URI [1.3]	Falls [0.92]
Yorkshire & Humber	Dermatitis [1.44]	Neonatal [0.92]	Asthma [1.35]	Iron [3.62]	Congenital [0.8]	Anxiety [1.07]	Conduct [0.97]	Skin Viral [0.87]	URI [1.29]	Falls [0.89]
Barnsley	Dermatitis [1.45]	Neonatal [0.97]	Asthma [1.3]	Iron [2.31]	Congenital [0.73]	Anxiety [1.12]	Conduct [0.97]	Skin Viral [0.87]	URI [1.34]	Falls [0.95]
Bradford	Dermatitis [1.44]	Neonatal [1.02]	Asthma [1.3]	Iron [2.89]	Congenital [0.74]	Anxiety [1.1]	Conduct [0.97]	Skin Viral [0.87]	URI [1.3]	Falls [0.93]
Calderdale	Dermatitis [1.44]	Neonatal [0.89]	Asthma [1.33]	Congenital [0.78]	Anxiety [1.08]	Conduct [0.96]	Skin Viral [0.88]	URI [1.29]	Iron [2.18]	Falls [0.9]
Doncaster	Dermatitis [1.45]	Neonatal [1.19]	Asthma [1.3]	Congenital [0.76]	Anxiety [1.11]	Iron [1.8]	Conduct [0.96]	Skin Viral [0.87]	URI [1.34]	Falls [0.96]
E Riding of Yorkshire	Dermatitis [1.44]	Neonatal [0.91]	Asthma [1.29]	Iron [3.21]	Congenital [0.66]	Anxiety [1.08]	Conduct [0.98]	Skin Viral [0.87]	URI [1.3]	Falls [0.88]
Kingston upon Hull	Dermatitis [1.44]	Asthma [1.37]	Neonatal [0.84]	Congenital [0.81]	Anxiety [1.08]	Conduct [0.97]	Iron [2.16]	Skin Viral [0.87]	URI [1.3]	Falls [0.94]
Kirklees	Dermatitis [1.44]	Asthma [1.32]	Neonatal [0.86]	Congenital [0.81]	Iron [3.18]	Anxiety [1.08]	Conduct [0.97]	Skin Viral [0.87]	URI [1.3]	Falls [0.92]
Leeds	Dermatitis [1.43]	Asthma [1.43]	Neonatal [0.84]	Iron [5.7]	Congenital [0.91]	Anxiety [1.03]	Conduct [0.99]	Skin Viral [0.9]	URI [1.27]	Falls [0.84]
NE Lincolnshire	Dermatitis [1.44]	Asthma [1.35]	Neonatal [0.88]	Iron [3.3]	Congenital [0.84]	Anxiety [1.1]	Conduct [0.97]	Skin Viral [0.87]	URI [1.31]	Falls [0.94]
N Lincolnshire	Dermatitis [1.44]	Asthma [1.3]	Neonatal [0.82]	Iron [3.44]	Congenital [0.68]	Anxiety [1.09]	Conduct [0.97]	Skin Viral [0.87]	URI [1.3]	Falls [0.9]
N Yorkshire	Dermatitis [1.43]	Neonatal [1.05]	Asthma [1.32]	Iron [3.75]	Congenital [0.75]	Anxiety [1.06]	Conduct [0.98]	Skin Viral [0.88]	URI [1.28]	Falls [0.87]
Rotherham	Dermatitis [1.44]	Asthma [1.3]	Neonatal [0.83]	Congenital [0.82]	Iron [2.29]	Anxiety [1.1]	Conduct [0.97]	Skin Viral [0.86]	URI [1.32]	Falls [0.96]
Sheffield	Dermatitis [1.44]	Neonatal [0.97]	Asthma [1.37]	Congenital [0.81]	Iron [4.14]	Anxiety [1.05]	Conduct [0.98]	Skin Viral [0.89]	URI [1.28]	Falls [0.85]
Wakefield	Dermatitis [1.44]	Asthma [1.34]	Iron [3.62]	Neonatal [0.73]	Congenital [0.76]	Anxiety [1.09]	Conduct [0.97]	Skin Viral [0.86]	URI [1.31]	Falls [0.93]
York	Dermatitis [1.44]	Asthma [1.45]	Neonatal [0.86]	Congenital [0.89]	Anxiety [1.03]	Conduct [0.99]	Iron [3.89]	Skin Viral [0.9]	URI [1.26]	Falls [0.82]

eFigure 7d. Leading ten causes of DALYs with the ratio of observed DALYs to DALYs expected on the basis of Socio-Demographic Index alone in 2017, 5-9 years, both sexes combined. The top ten causes contributing to DALYs are listed globally, by socio-demographic quintile, and then by GBD superregion, region, country, and subnationally where modeled. For each cell, the ratio of observed DALYs to DALYs expected on the basis of socio-demographic index (SDI) alone are listed. Abbreviations: DALY=disability-adjusted life year, GBD=Global Burden of Disease.

Values shown in brackets represent the ratio of observed DALYs to predicted DALYs on the basis of Socio-Demographic Index (SDI), rounded to two (2) digits. Color ranges (shown below) were calculated to place a roughly equal number of cells into each bin.

COLOR KEY:									
	[0.0-0.62]	(0.62-0.79]	(0.79-0.89]	(0.89-0.97]	(0.97-1.07]	(1.07-1.26]	(1.26-1.44]	(1.44-2.32]	2.32+

	1	2	3	4	5	6	7	8	9	10
N Ireland	Dermatitis [1.19]	Anxiety [1.71]	Neonatal [0.84]	Asthma [1.15]	Congenital [0.75]	Skin Viral [0.94]	Conduct [0.88]	URI [1.27]	Falls [0.87]	Headaches [0.97]
Scotland	Dermatitis [1.29]	Asthma [1.24]	Iron [3.4]	Neonatal [0.79]	Congenital [0.82]	Anxiety [1.17]	Skin Viral [0.93]	Conduct [0.95]	Vit A [1.78]	URI [1.3]
Wales	Dermatitis [1.2]	Neonatal [0.81]	Asthma [1.17]	Iron [3.27]	Congenital [0.72]	Anxiety [1.19]	Skin Viral [1.01]	Conduct [0.95]	URI [1.31]	Falls [1.05]
**Latin America and Caribbean**	Neonatal [1.15]	Asthma [1.28]	Road Inj [0.48]	Dermatitis [0.9]	Congenital [0.6]	Vit A [0.94]	Iron [0.72]	Diarrhea [1.3]	LRI [0.58]	Leukemia [0.68]
**Andean Latin America**	Asthma [1.6]	Road Inj [0.68]	Dermatitis [1.48]	Iron [1.31]	Neonatal [1.09]	LRI [1.04]	Congenital [0.55]	Leukemia [0.93]	Vit A [0.77]	Diarrhea [1.2]
Bolivia	Iron [1.64]	Asthma [1.78]	Road Inj [0.7]	Dermatitis [1.58]	LRI [0.81]	Neonatal [1.07]	Diarrhea [1.11]	Congenital [0.55]	Leukemia [0.92]	Vit A [0.63]
Ecuador	Road Inj [0.97]	Asthma [1.48]	Neonatal [1.12]	Dermatitis [1.3]	LRI [0.98]	Congenital [0.59]	Leukemia [1.04]	Diarrhea [1.27]	Epilepsy [1.11]	Drown [0.47]
Peru	Iron [1.63]	Dermatitis [1.54]	Asthma [1.58]	Neonatal [1.1]	LRI [1.09]	Road Inj [0.51]	Vit A [1.0]	Congenital [0.53]	Leukemia [0.86]	F Body [2.18]
**Caribbean**	Road Inj [0.96]	Iron [1.84]	Asthma [1.85]	Dermatitis [1.15]	Neonatal [0.93]	Congenital [0.71]	Drown [0.73]	Vit A [1.02]	Disaster [169.05]	LRI [0.74]
Antigua	Iron [4.36]	Asthma [1.97]	Dermatitis [1.08]	Neonatal [0.96]	Drown [1.23]	Congenital [0.63]	Vit A [0.97]	Epilepsy [1.17]	Road Inj [0.32]	Skin Viral [0.8]
Bahamas	Asthma [2.13]	Dermatitis [1.07]	Neonatal [0.96]	Drown [1.61]	Congenital [0.69]	Road Inj [0.62]	Iron [1.35]	Skin Viral [0.8]	Anxiety [0.85]	Diarrhea [1.46]
Barbados	Asthma [1.85]	Neonatal [1.04]	Dermatitis [1.05]	Congenital [0.62]	Iron [1.04]	Skin Viral [0.8]	Anxiety [0.86]	Headaches [1.31]	Epilepsy [0.94]	Road Inj [0.3]
Belize	Iron [1.58]	Asthma [1.84]	Neonatal [1.02]	Dermatitis [1.21]	Road Inj [0.36]	Drown [0.53]	Congenital [0.45]	LRI [0.38]	Vit A [0.48]	Diarrhea [0.63]
Bermuda	Asthma [2.53]	Dermatitis [1.03]	Neonatal [0.98]	Congenital [0.56]	Skin Viral [0.79]	Anxiety [0.79]	Iron [1.57]	Headaches [1.33]	Diarrhea [1.71]	Conduct [0.68]
Cuba	Asthma [1.96]	Dermatitis [1.11]	Neonatal [0.92]	Iron [1.31]	Congenital [0.52]	Skin Viral [0.81]	Road Inj [0.27]	Anxiety [0.91]	Headaches [1.3]	Drown [0.42]
Dominica	Iron [2.17]	Asthma [1.91]	Neonatal [1.1]	Dermatitis [1.11]	Congenital [0.75]	Road Inj [0.52]	Drown [0.92]	Epilepsy [1.31]	Leukemia [0.82]	Vit A [0.78]
Dominican Rep	Iron [1.37]	Road Inj [0.6]	Asthma [1.36]	Neonatal [1.03]	Dermatitis [1.21]	Congenital [0.62]	Diarrhea [0.97]	Vit A [0.61]	LRI [0.45]	Violence [1.7]
Grenada	Asthma [1.58]	Neonatal [1.06]	Dermatitis [1.15]	Iron [0.96]	Drown [0.72]	Vit A [0.96]	Congenital [0.54]	LRI [0.62]	Diarrhea [1.12]	Road Inj [0.25]
Guyana	Iron [1.41]	Asthma [1.7]	Neonatal [1.1]	Dermatitis [1.22]	Drown [0.63]	Road Inj [0.35]	Congenital [0.51]	Vit A [0.69]	LRI [0.47]	Diarrhea [0.77]
Haiti	Road Inj [1.13]	Iron [1.22]	Asthma [1.74]	Drown [0.75]	Vit A [0.74]	Congenital [0.75]	LRI [0.38]	Dermatitis [1.2]	HIV [6.94]	Neonatal [1.08]
Jamaica	Asthma [1.96]	Neonatal [1.02]	Dermatitis [1.12]	Congenital [0.62]	Iron [0.99]	Vit A [0.89]	Road Inj [0.3]	Epilepsy [0.98]	Skin Viral [0.81]	Anxiety [0.93]
Puerto Rico	Disaster [6687.47]	Asthma [3.09]	Neonatal [1.04]	Dermatitis [1.03]	Congenital [0.6]	Iron [2.02]	Vit A [1.84]	Skin Viral [0.79]	Anxiety [0.78]	Headaches [1.32]
St Lucia	Asthma [1.87]	Neonatal [1.08]	Dermatitis [1.14]	Iron [0.88]	Congenital [0.48]	Drown [0.52]	Road Inj [0.3]	Diarrhea [1.13]	Vit A [0.61]	Epilepsy [0.86]
St Vincent	Iron [1.79]	Asthma [1.75]	Neonatal [1.03]	Dermatitis [1.19]	Drown [0.63]	Congenital [0.53]	Vit A [0.67]	Epilepsy [0.84]	Diarrhea [0.7]	Road Inj [0.21]
Suriname	Asthma [1.91]	Iron [1.55]	Neonatal [1.02]	Dermatitis [1.15]	Road Inj [0.4]	Congenital [0.53]	Vit A [0.84]	Drown [0.52]	Diarrhea [1.09]	Epilepsy [0.93]
Trinidad Tobago	Asthma [2.14]	Neonatal [1.04]	Dermatitis [1.1]	Road Inj [0.54]	Congenital [0.68]	Leukemia [0.67]	Skin Viral [0.8]	Anxiety [0.9]	Epilepsy [0.92]	Headaches [1.3]
Virgin Isl US	Asthma [2.26]	Neonatal [1.0]	Dermatitis [1.03]	Congenital [0.74]	Disaster [480.83]	Iron [2.2]	Diarrhea [2.11]	Road Inj [0.54]	Drown [1.23]	Skin Viral [0.79]
**Central Latin America**	Neonatal [1.37]	Congenital [0.57]	Diarrhea [1.39]	Asthma [0.88]	Road Inj [0.37]	Dermatitis [0.81]	Epilepsy [1.21]	Vit A [0.69]	Leukemia [0.75]	Violence [1.72]
Colombia	Neonatal [1.37]	Dermatitis [0.89]	Asthma [0.89]	Diarrhea [1.36]	Road Inj [0.34]	Congenital [0.47]	Epilepsy [1.22]	Leukemia [0.76]	Drown [0.36]	Skin Viral [0.83]
Costa Rica	Neonatal [1.27]	Asthma [1.28]	Dermatitis [0.83]	Diarrhea [1.65]	Congenital [0.5]	Road Inj [0.32]	Epilepsy [1.07]	Skin Viral [0.82]	Conduct [0.81]	Vit A [0.48]
El Salvador	Neonatal [1.39]	Iron [1.16]	Vit A [1.07]	Asthma [1.11]	Diarrhea [1.14]	Congenital [0.53]	Road Inj [0.34]	Dermatitis [0.82]	Epilepsy [0.93]	LRI [0.36]

eFigure 7d. Leading ten causes of DALYs with the ratio of observed DALYs to DALYs expected on the basis of Socio-Demographic Index alone in 2017, 5-9 years, both sexes combined. The top ten causes contributing to DALYs are listed globally, by socio-demographic quintile, and then by GBD superregion, region, country, and subnationally where modeled. For each cell, the ratio of observed DALYs to DALYs expected on the basis of socio-demographic index (SDI) alone are listed. Abbreviations: DALY=disability-adjusted life year, GBD=Global Burden of Disease.

Values shown in brackets represent the ratio of observed DALYs to predicted DALYs on the basis of Socio-Demographic Index (SDI), rounded to two (2) digits. Color ranges (shown below) were calculated to place a roughly equal number of cells into each bin.

COLOR KEY:	[0.0-0.62]	(0.62-0.79]	(0.79-0.89]	(0.89-0.97]	(0.97-1.07]	(1.07-1.26]	(1.26-1.44]	(1.44-2.32]	2.32+

	1	2	3	4	5	6	7	8	9	10
Guatemala	Diarrhea [1.41]	LRI [0.85]	Neonatal [1.46]	Iron [0.71]	Dermatitis [1.07]	Congenital [0.45]	Asthma [0.73]	Road Inj [0.22]	Epilepsy [0.86]	Vit A [0.39]
Honduras	Diarrhea [1.12]	Iron [0.88]	Neonatal [1.58]	Violence [2.95]	Asthma [1.14]	Road Inj [0.34]	Dermatitis [1.06]	Vit A [0.6]	Leukemia [0.87]	Epilepsy [1.11]
Mexico	Neonatal [1.38]	Congenital [0.63]	Road Inj [0.39]	Vit A [0.83]	Asthma [0.81]	Epilepsy [1.32]	Leukemia [0.82]	Dermatitis [0.66]	Violence [1.94]	Conduct [0.87]
Aguascalientes	Neonatal [1.36]	Asthma [0.91]	Congenital [0.55]	Epilepsy [1.34]	Dermatitis [0.65]	Road Inj [0.32]	Vit A [0.68]	Leukemia [0.62]	Conduct [0.87]	Skin Viral [0.81]
Baja CA	Neonatal [1.34]	Vit A [1.1]	Congenital [0.52]	Asthma [0.78]	Epilepsy [1.25]	Dermatitis [0.65]	Road Inj [0.28]	Violence [1.9]	Leukemia [0.62]	Conduct [0.87]
Baja CA Sur	Neonatal [1.42]	Road Inj [0.52]	Congenital [0.66]	Vit A [1.09]	Asthma [0.88]	Epilepsy [1.33]	Dermatitis [0.65]	Leukemia [0.8]	Violence [1.81]	Conduct [0.87]
Campeche	Neonatal [1.51]	Vit A [0.97]	Congenital [0.55]	Asthma [0.8]	Road Inj [0.32]	Epilepsy [1.16]	Dermatitis [0.68]	Leukemia [0.75]	Iron [0.51]	Violence [1.49]
Chiapas	Neonatal [1.45]	Leukemia [1.25]	Congenital [0.65]	Diarrhea [0.73]	Epilepsy [1.34]	Vit A [0.62]	Road Inj [0.28]	Asthma [0.75]	LRI [0.31]	Dermatitis [0.7]
Chihuahua	Neonatal [1.27]	Road Inj [0.48]	Congenital [0.62]	Asthma [0.92]	Epilepsy [1.32]	Violence [2.52]	Leukemia [0.7]	Dermatitis [0.56]	Vit A [0.61]	Conduct [0.87]
Coahuila	Neonatal [1.16]	Road Inj [0.42]	Congenital [0.58]	Asthma [0.91]	Epilepsy [1.31]	Dermatitis [0.66]	Vit A [0.72]	Leukemia [0.74]	Diarrhea [1.01]	Iron [0.5]
Colima	Neonatal [1.45]	Road Inj [0.51]	Congenital [0.65]	Vit A [0.98]	Asthma [0.85]	Epilepsy [1.37]	Dermatitis [0.65]	Violence [2.35]	Leukemia [0.72]	Conduct [0.87]
Mexico City	Neonatal [1.45]	Congenital [0.84]	Dermatitis [0.81]	Epilepsy [1.69]	Leukemia [1.15]	Asthma [0.82]	Road Inj [0.42]	Violence [2.88]	Vit A [0.77]	Skin Viral [0.8]
Durango	Neonatal [1.22]	Dermatitis [0.88]	Asthma [0.89]	Road Inj [0.34]	Vit A [0.79]	Congenital [0.48]	Epilepsy [1.19]	Violence [1.77]	Conduct [0.87]	Skin Viral [0.83]
Guanajuato	Neonatal [1.25]	Road Inj [0.43]	Vit A [1.01]	Congenital [0.59]	Asthma [0.86]	Epilepsy [1.3]	Dermatitis [0.68]	Leukemia [0.76]	Conduct [0.88]	Violence [1.47]
Guerrero	Neonatal [1.49]	Violence [3.09]	Road Inj [0.43]	Congenital [0.62]	Vit A [0.67]	Asthma [0.77]	Epilepsy [1.13]	Leukemia [0.77]	Dermatitis [0.71]	Diarrhea [0.48]
Hidalgo	Neonatal [1.72]	Congenital [0.59]	Road Inj [0.36]	Epilepsy [1.31]	Asthma [0.82]	Leukemia [0.76]	Vit A [0.6]	Dermatitis [0.7]	Violence [1.44]	Conduct [0.9]
Jalisco	Neonatal [1.31]	Road Inj [0.54]	Congenital [0.69]	Epilepsy [1.53]	Leukemia [0.98]	Asthma [0.77]	Vit A [0.8]	Dermatitis [0.66]	Violence [1.88]	Conduct [0.87]
Mexico	Neonatal [1.63]	Congenital [0.57]	Epilepsy [1.27]	Vit A [0.79]	Asthma [0.75]	Road Inj [0.31]	Violence [2.04]	Leukemia [0.7]	Dermatitis [0.57]	Conduct [0.87]
Michoacan	Neonatal [1.2]	Road Inj [0.47]	Congenital [0.61]	Vit A [0.74]	Asthma [0.83]	Leukemia [0.8]	Epilepsy [1.15]	Violence [1.95]	Dermatitis [0.7]	Conduct [0.9]
Morelos	Neonatal [1.34]	Congenital [0.67]	Vit A [0.85]	Epilepsy [1.32]	Asthma [0.77]	Road Inj [0.33]	Violence [2.19]	Leukemia [0.77]	Dermatitis [0.57]	Disaster [118.29]
Nayarit	Neonatal [1.47]	Road Inj [0.62]	Congenital [0.66]	Vit A [0.95]	Asthma [0.83]	Epilepsy [1.27]	Violence [2.21]	Leukemia [0.8]	Dermatitis [0.67]	Conduct [0.88]
Nuevo Leon	Neonatal [1.1]	Vit A [1.34]	Congenital [0.63]	Asthma [0.79]	Epilepsy [1.36]	Road Inj [0.32]	Dermatitis [0.55]	Leukemia [0.77]	Violence [1.92]	Conduct [0.86]
Oaxaca	Neonatal [1.42]	Congenital [0.74]	Road Inj [0.36]	Epilepsy [1.38]	Leukemia [0.93]	Violence [2.25]	Asthma [0.78]	Diarrhea [0.66]	Dermatitis [0.71]	Vit A [0.51]
Puebla	Neonatal [1.32]	Congenital [0.71]	Road Inj [0.4]	Leukemia [1.01]	Asthma [0.82]	Vit A [0.61]	Dermatitis [0.7]	Epilepsy [1.05]	Violence [1.56]	Disaster [74.38]
Queretaro	Neonatal [1.44]	Road Inj [0.49]	Congenital [0.68]	Asthma [0.88]	Epilepsy [1.35]	Dermatitis [0.66]	Leukemia [0.77]	Vit A [0.62]	Conduct [0.87]	Violence [1.59]
Quintana Roo	Neonatal [1.73]	Vit A [1.11]	Congenital [0.64]	Road Inj [0.4]	Asthma [0.81]	Iron [0.67]	Epilepsy [1.26]	Leukemia [0.81]	Dermatitis [0.67]	Diarrhea [0.96]
San Luis Potosi	Neonatal [1.19]	Congenital [0.62]	Road Inj [0.37]	Asthma [0.85]	Epilepsy [1.35]	Vit A [0.83]	Dermatitis [0.68]	Leukemia [0.65]	Violence [1.6]	Conduct [0.88]
Sinaloa	Neonatal [1.24]	Road Inj [0.53]	Vit A [1.05]	Congenital [0.59]	Asthma [0.85]	Epilepsy [1.29]	Dermatitis [0.66]	Violence [2.28]	Leukemia [0.64]	Iron [0.48]
Sonora	Neonatal [1.21]	Road Inj [0.56]	Congenital [0.65]	Asthma [0.89]	Vit A [0.97]	Epilepsy [1.39]	Leukemia [0.91]	Dermatitis [0.66]	Violence [2.01]	Iron [0.51]
Tabasco	Neonatal [1.49]	Road Inj [0.48]	Congenital [0.69]	Vit A [1.04]	Iron [0.77]	Leukemia [0.98]	Epilepsy [1.26]	Asthma [0.75]	Dermatitis [0.67]	Diarrhea [0.78]
Tamaulipas	Neonatal [1.52]	Congenital [0.59]	Road Inj [0.41]	Asthma [0.84]	Vit A [0.88]	Epilepsy [1.24]	Violence [2.0]	Dermatitis [0.55]	Leukemia [0.67]	Diarrhea [0.92]

eFigure 7d. Leading ten causes of DALYs with the ratio of observed DALYs to DALYs expected on the basis of Socio-Demographic Index alone in 2017, 5-9 years, both sexes combined. The top ten causes contributing to DALYs are listed globally, by socio-demographic quintile, and then by GBD superregion, region, country, and subnationally where modeled. For each cell, the ratio of observed DALYs to DALYs expected on the basis of socio-demographic index (SDI) alone are listed. Abbreviations: DALY=disability-adjusted life year, GBD=Global Burden of Disease.

Values shown in brackets represent the ratio of observed DALYs to predicted DALYs on the basis of Socio-Demographic Index (SDI), rounded to two (2) digits. Color ranges (shown below) were calculated to place a roughly equal number of cells into each bin.

COLOR KEY:	[0.0-0.62]	(0.62-0.79]	(0.79-0.89]	(0.89-0.97]	(0.97-1.07]	(1.07-1.26]	(1.26-1.44]	(1.44-2.32]	2.32+

	1	2	3	4	5	6	7	8	9	10
Tlaxcala	Neonatal [1.48]	Congenital [0.58]	Asthma [0.84]	Road Inj [0.34]	Vit A [0.77]	Epilepsy [1.28]	Dermatitis [0.69]	Leukemia [0.73]	Conduct [0.88]	Violence [1.37]
Veracruz	Neonatal [1.48]	Congenital [0.58]	Vit A [0.74]	Epilepsy [1.23]	Asthma [0.78]	Dermatitis [0.7]	Leukemia [0.66]	Violence [1.48]	Road Inj [0.19]	Conduct [0.89]
Yucatan	Neonatal [1.27]	Congenital [0.61]	Asthma [0.78]	Epilepsy [1.28]	Vit A [0.77]	Leukemia [0.77]	Dermatitis [0.63]	Road Inj [0.26]	Iron [0.51]	Conduct [0.87]
Zacatecas	Road Inj [0.61]	Neonatal [0.95]	Congenital [0.56]	Asthma [0.87]	Epilepsy [1.33]	Vit A [0.71]	Dermatitis [0.68]	Leukemia [0.62]	Violence [1.61]	Conduct [0.88]
Nicaragua	Neonatal [1.47]	Diarrhea [0.78]	Dermatitis [1.07]	Asthma [0.83]	Congenital [0.42]	Epilepsy [0.85]	Road Inj [0.18]	Leukemia [0.5]	Iron [0.26]	Skin Viral [0.87]
Panama	Neonatal [1.25]	Diarrhea [2.99]	Asthma [1.27]	Dermatitis [1.1]	Iron [1.17]	Congenital [0.61]	Road Inj [0.35]	Vit A [0.71]	Epilepsy [0.99]	LRI [0.71]
Venezuela	Neonatal [1.36]	Diarrhea [2.8]	Road Inj [0.49]	Dermatitis [0.95]	Congenital [0.63]	Asthma [0.97]	Epilepsy [1.14]	Leukemia [0.69]	Vit A [0.62]	LRI [0.53]
**Tropical Latin America**	Asthma [1.6]	Vit A [1.38]	Neonatal [0.91]	Congenital [0.66]	Road Inj [0.44]	Dermatitis [0.77]	Iron [0.74]	Headaches [1.67]	Diarrhea [1.08]	Anxiety [1.06]
Brazil	Asthma [1.6]	Vit A [1.42]	Neonatal [0.92]	Congenital [0.67]	Road Inj [0.46]	Dermatitis [0.75]	Iron [0.75]	Headaches [1.66]	Diarrhea [1.07]	Anxiety [1.05]
Acre	Iron [1.07]	Asthma [1.37]	Neonatal [0.99]	Vit A [1.07]	Congenital [0.69]	Road Inj [0.42]	LRI [0.56]	Dermatitis [0.8]	Drown [0.46]	Diarrhea [0.95]
Alagoas	Vit A [1.22]	Asthma [1.23]	Road Inj [0.42]	Neonatal [0.85]	Congenital [0.54]	Diarrhea [0.61]	LRI [0.35]	Dermatitis [0.68]	Iron [0.36]	Drown [0.31]
Amapa	Drown [1.25]	Asthma [1.52]	Neonatal [0.96]	Road Inj [0.54]	Congenital [0.73]	Iron [1.0]	Vit A [1.04]	LRI [0.99]	Dermatitis [0.76]	Headaches [1.67]
Amazonas	Asthma [1.47]	Vit A [1.33]	Iron [1.1]	Congenital [0.69]	Neonatal [0.82]	Drown [0.7]	Road Inj [0.36]	Dermatitis [0.78]	LRI [0.59]	Headaches [1.68]
Bahia	Vit A [1.46]	Asthma [1.31]	Iron [0.82]	Neonatal [0.91]	Dermatitis [1.09]	Congenital [0.56]	Road Inj [0.36]	Diarrhea [0.87]	Drown [0.42]	LRI [0.36]
Ceara	Vit A [1.51]	Asthma [1.39]	Neonatal [0.86]	Road Inj [0.41]	Congenital [0.59]	Dermatitis [0.81]	Diarrhea [0.85]	Drown [0.34]	Headaches [1.7]	LRI [0.37]
Distrito Federal	Asthma [1.95]	Neonatal [1.01]	Road Inj [1.1]	Congenital [1.06]	Dermatitis [0.69]	Vit A [1.85]	Headaches [1.68]	Anxiety [0.9]	Skin Viral [0.79]	Brain C [1.11]
Espirito Santo	Asthma [1.86]	Vit A [2.01]	Neonatal [0.9]	Congenital [0.78]	Road Inj [0.52]	Iron [1.0]	Dermatitis [0.75]	Drown [0.53]	Headaches [1.66]	Anxiety [1.04]
Goias	Asthma [1.62]	Road Inj [0.58]	Neonatal [0.83]	Vit A [1.16]	Congenital [0.65]	Dermatitis [0.77]	Iron [0.66]	Headaches [1.68]	Anxiety [1.07]	Diarrhea [0.95]
Maranhao	Vit A [1.09]	Asthma [1.16]	Iron [0.55]	Road Inj [0.35]	Neonatal [0.95]	Congenital [0.54]	Dermatitis [0.81]	Diarrhea [0.37]	LRI [0.22]	Drown [0.26]
Mato Grosso	Asthma [1.71]	Road Inj [0.59]	Vit A [1.35]	Neonatal [0.87]	Congenital [0.63]	Dermatitis [0.76]	Headaches [1.67]	Drown [0.48]	Diarrhea [1.07]	Anxiety [1.06]
Mato Grosso do Sul	Asthma [1.56]	Neonatal [1.0]	Road Inj [0.5]	Congenital [0.67]	Vit A [0.98]	Dermatitis [0.77]	Headaches [1.68]	Diarrhea [1.04]	Anxiety [1.07]	LRI [0.51]
Minas Gerais	Asthma [1.69]	Neonatal [0.96]	Vit A [1.31]	Congenital [0.64]	Road Inj [0.45]	Dermatitis [0.65]	Headaches [1.66]	Diarrhea [1.08]	Iron [0.55]	Anxiety [1.06]
Para	Asthma [1.38]	Vit A [0.99]	Iron [0.79]	Neonatal [0.88]	Congenital [0.55]	Diarrhea [0.97]	Road Inj [0.33]	Dermatitis [0.83]	Drown [0.45]	LRI [0.47]
Paraiba	Vit A [1.52]	Asthma [1.3]	Road Inj [0.42]	Congenital [0.61]	Neonatal [0.82]	Diarrhea [0.86]	Dermatitis [0.82]	LRI [0.36]	Drown [0.34]	Headaches [1.74]
Parana	Asthma [1.83]	Neonatal [0.94]	Congenital [0.72]	Road Inj [0.53]	Vit A [1.18]	Dermatitis [0.67]	Headaches [1.67]	Anxiety [1.03]	Diarrhea [1.1]	Skin Viral [0.8]
Pernambuco	Vit A [1.4]	Asthma [1.43]	Iron [1.05]	Congenital [0.67]	Neonatal [0.86]	Road Inj [0.37]	Dermatitis [0.79]	LRI [0.38]	Headaches [1.69]	Diarrhea [0.68]
Piaui	Asthma [1.31]	Road Inj [0.46]	Vit A [0.83]	Congenital [0.65]	Neonatal [0.98]	Dermatitis [0.83]	Diarrhea [0.61]	Drown [0.35]	LRI [0.31]	Headaches [1.78]
Rio de Janeiro	Asthma [1.68]	Neonatal [1.14]	Congenital [0.7]	Dermatitis [0.73]	Road Inj [0.38]	Headaches [1.67]	Vit A [0.84]	LRI [1.03]	Anxiety [0.99]	Skin Viral [0.79]
Rio Grande do Norte	Asthma [1.39]	Vit A [1.12]	Neonatal [0.79]	Iron [0.69]	Congenital [0.54]	Dermatitis [0.8]	Diarrhea [0.98]	Road Inj [0.29]	Headaches [1.69]	LRI [0.4]
Rio Grande do Sul	Asthma [1.88]	Congenital [1.01]	Neonatal [0.96]	Dermatitis [0.98]	Epilepsy [1.58]	Vit A [1.01]	Road Inj [0.38]	Back Pain [3.24]	Headaches [1.71]	Anxiety [1.01]
Rondonia	Asthma [1.44]	Congenital [0.83]	Neonatal [1.01]	Road Inj [0.53]	Vit A [1.09]	Iron [0.71]	Dermatitis [0.79]	Drown [0.47]	Headaches [1.68]	LRI [0.45]

eFigure 7d. Leading ten causes of DALYs with the ratio of observed DALYs to DALYs expected on the basis of Socio-Demographic Index alone in 2017, 5-9 years, both sexes combined. The top ten causes contributing to DALYs are listed globally, by socio-demographic quintile, and then by GBD superregion, region, country, and subnationally where modeled. For each cell, the ratio of observed DALYs to DALYs expected on the basis of socio-demographic index (SDI) alone are listed. Abbreviations: DALY=disability-adjusted life year, GBD=Global Burden of Disease.

Values shown in brackets represent the ratio of observed DALYs to predicted DALYs on the basis of Socio-Demographic Index (SDI), rounded to two (2) digits. Color ranges (shown below) were calculated to place a roughly equal number of cells into each bin.

COLOR KEY:	[0.0-0.62]	(0.62-0.79]	(0.79-0.89]	(0.89-0.97]	(0.97-1.07]	(1.07-1.26]	(1.26-1.44]	(1.44-2.32]	2.32+

	1	2	3	4	5	6	7	8	9	10
Roraima	Road Inj [0.77]	Asthma [1.48]	Neonatal [1.01]	Vit A [1.38]	Congenital [0.78]	Drown [0.74]	Iron [0.83]	LRI [0.88]	Dermatitis [0.77]	Headaches [1.67]
Santa Catarina	Asthma [1.99]	Road Inj [0.76]	Vit A [1.63]	Congenital [0.79]	Neonatal [0.86]	Iron [1.1]	Dermatitis [0.74]	Headaches [2.07]	Anxiety [1.01]	Skin Viral [0.79]
Sao Paulo	Asthma [1.84]	Neonatal [0.98]	Vit A [1.26]	Congenital [0.62]	Dermatitis [0.63]	Road Inj [0.37]	Headaches [1.6]	Anxiety [0.97]	Iron [0.74]	Skin Viral [0.79]
Sergipe	Vit A [1.59]	Asthma [1.51]	Neonatal [1.0]	Road Inj [0.45]	Congenital [0.57]	Iron [0.59]	Dermatitis [0.66]	Headaches [1.69]	Drown [0.35]	Anxiety [1.13]
Tocantins	Road Inj [0.77]	Asthma [1.42]	Neonatal [0.98]	Vit A [1.09]	Congenital [0.68]	Leish [223.65]	Dermatitis [0.8]	Drown [0.49]	Iron [0.5]	Headaches [1.69]
Paraguay	Asthma [1.62]	Dermatitis [1.05]	Iron [0.82]	Neonatal [0.78]	Diarrhea [1.46]	Congenital [0.58]	Vit A [0.89]	Road Inj [0.32]	Headaches [1.72]	Anxiety [1.14]
**North Africa and Middle East**	Conflict Terror [1335.19]	Road Inj [1.08]	Iron [1.81]	Congenital [0.92]	Neonatal [0.96]	Asthma [1.0]	Vit A [1.04]	LRI [0.92]	Diarrhea [1.44]	Dermatitis [0.7]
Afghanistan	LRI [0.47]	Road Inj [0.49]	Meningitis [0.6]	Congenital [0.79]	Vit A [0.47]	Measles [0.25]	Drown [0.44]	Iron [0.38]	Falls [1.78]	Asthma [1.04]
Algeria	Road Inj [1.41]	Congenital [1.19]	Neonatal [1.07]	Asthma [1.13]	Iron [1.1]	Dermatitis [0.68]	Vit A [0.98]	Conduct [1.07]	Epilepsy [1.11]	LRI [0.89]
Bahrain	Neonatal [1.03]	Road Inj [0.77]	Dermatitis [0.9]	Asthma [1.06]	Congenital [0.63]	Diarrhea [1.71]	Conduct [1.06]	Hemog [3.44]	Vit A [0.8]	Epilepsy [1.07]
Egypt	Road Inj [1.06]	LRI [1.29]	Iron [1.38]	Congenital [0.89]	Neonatal [1.04]	Asthma [1.2]	Diarrhea [1.06]	Conduct [1.1]	Dermatitis [0.57]	Epilepsy [0.88]
Iran	Road Inj [1.73]	Neonatal [1.24]	Congenital [1.04]	Iron [1.1]	Asthma [0.85]	Diarrhea [1.83]	Anxiety [1.26]	Conduct [1.12]	Epilepsy [1.21]	Dermatitis [0.54]
Iraq	Conflict Terror [3235.72]	Iron [1.59]	Road Inj [0.73]	Congenital [1.07]	Neonatal [1.12]	Vit A [0.97]	Asthma [0.94]	Mech [2.34]	Violence [2.21]	Oth Unint [3.3]
Jordan	Neonatal [1.39]	Road Inj [0.98]	Congenital [0.97]	Asthma [1.27]	Iron [1.23]	Vit A [1.07]	Dermatitis [0.68]	Conduct [1.06]	Diarrhea [1.24]	Anxiety [0.97]
Kuwait	Neonatal [1.16]	Road Inj [1.03]	Congenital [1.01]	Asthma [1.28]	Iron [2.31]	Dermatitis [0.67]	Diarrhea [2.22]	Conduct [1.06]	Anxiety [0.87]	Epilepsy [1.17]
Lebanon	Neonatal [1.03]	Asthma [1.4]	Congenital [0.73]	Dermatitis [0.8]	Iron [1.26]	Diarrhea [1.78]	Anxiety [1.11]	Conduct [1.02]	Conflict Terror [520.3]	Leukemia [0.77]
Libya	Conflict Terror [3712.72]	Road Inj [1.32]	Congenital [1.3]	Iron [2.54]	Neonatal [1.03]	Dermatitis [0.87]	Diarrhea [3.02]	Asthma [1.08]	Leukemia [1.22]	Conduct [1.06]
Morocco	Road Inj [0.93]	Congenital [1.08]	Iron [1.11]	Vit A [1.24]	Neonatal [1.06]	Dermatitis [0.82]	Asthma [0.76]	Diarrhea [0.74]	LRI [0.4]	Conduct [1.11]
Palestine	Neonatal [1.14]	Road Inj [0.43]	Iron [0.58]	Congenital [0.58]	Asthma [0.93]	Dermatitis [0.79]	Vit A [0.47]	Conduct [1.13]	Epilepsy [0.76]	Diarrhea [0.4]
Oman	Road Inj [1.99]	Iron [2.35]	Neonatal [1.07]	Asthma [0.93]	Congenital [0.62]	Diarrhea [2.21]	Dermatitis [0.6]	Conduct [1.06]	Anxiety [0.91]	Epilepsy [1.08]
Qatar	Road Inj [1.24]	Neonatal [0.97]	Congenital [0.97]	Dermatitis [0.87]	Asthma [0.93]	Iron [1.36]	Conduct [1.06]	Anxiety [0.89]	Skin Viral [0.78]	Epilepsy [1.09]
Saudi Arabia	Dermatitis [0.85]	Congenital [0.85]	Neonatal [0.73]	Road Inj [0.76]	Conflict Terror [1131.51]	Asthma [0.84]	Diarrhea [2.25]	Conduct [1.07]	Anxiety [0.88]	Skin Viral [0.78]
Sudan	Road Inj [1.18]	Iron [1.09]	Congenital [0.92]	Diarrhea [0.61]	Vit A [0.7]	Asthma [0.95]	Neonatal [0.86]	LRI [0.29]	Hemog [1.42]	Dermatitis [0.72]
Syria	Conflict Terror [12320.41]	Iron [1.53]	Neonatal [0.93]	Congenital [0.59]	Asthma [0.94]	Road Inj [0.33]	Vit A [0.7]	LRI [0.52]	Conduct [1.08]	Leukemia [0.62]
Tunisia	Road Inj [0.7]	Neonatal [1.03]	Congenital [0.61]	Dermatitis [0.79]	Asthma [0.84]	Mech [2.32]	Conduct [1.07]	Diarrhea [1.1]	Anxiety [1.0]	Skin Viral [0.79]
Turkey	Road Inj [0.68]	Congenital [0.77]	Neonatal [0.75]	Iron [1.24]	Asthma [0.9]	Vit A [1.24]	Dermatitis [0.61]	Epilepsy [1.38]	Conduct [1.04]	Diarrhea [1.58]
UAE	Road Inj [1.51]	Congenital [1.2]	Neonatal [1.02]	Asthma [1.43]	Iron [3.32]	Dermatitis [0.85]	Diarrhea [2.45]	Conduct [1.02]	Epilepsy [1.41]	Anxiety [0.82]

eFigure 7d. Leading ten causes of DALYs with the ratio of observed DALYs to DALYs expected on the basis of Socio-Demographic Index alone in 2017, 5-9 years, both sexes combined. The top ten causes contributing to DALYs are listed globally, by socio-demographic quintile, and then by GBD superregion, region, country, and subnationally where modeled. For each cell, the ratio of observed DALYs to DALYs expected on the basis of socio-demographic index (SDI) alone are listed. Abbreviations: DALY=disability-adjusted life year, GBD=Global Burden of Disease.

Values shown in brackets represent the ratio of observed DALYs to predicted DALYs on the basis of Socio-Demographic Index (SDI), rounded to two (2) digits. Color ranges (shown below) were calculated to place a roughly equal number of cells into each bin.

COLOR KEY:	[0.0-0.62]	(0.62-0.79]	(0.79-0.89]	(0.89-0.97]	(0.97-1.07]	(1.07-1.26]	(1.26-1.44]	(1.44-2.32]	2.32+

	1	2	3	4	5	6	7	8	9	10
Yemen	Iron [3.47]	Conflict Terror [682.95]	Road Inj [1.17]	Vit A [1.37]	Diarrhea [0.5]	Congenital [0.63]	Neonatal [1.29]	Dermatitis [0.97]	Hemog [1.5]	Oth NTD [1.51]
**South Asia**	Iron [1.96]	Typh + Paratyph [39.04]	Vit A [1.56]	Diarrhea [1.67]	Neonatal [1.5]	LRI [0.61]	Drown [0.64]	Malaria [88.37]	Congenital [0.64]	Road Inj [0.32]
Bangladesh	Typh + Paratyph [14.34]	Drown [1.55]	Iron [0.99]	Diarrhea [0.62]	LRI [0.48]	Neonatal [1.56]	Congenital [0.7]	Vit A [0.48]	Road Inj [0.28]	Dermatitis [0.89]
Bhutan	Iron [3.55]	Vit A [1.46]	Neonatal [1.33]	Diarrhea [1.29]	Typh + Paratyph [29.07]	Congenital [0.65]	LRI [0.49]	Dermatitis [0.9]	Malaria [103.05]	Encepha [1.98]
India	Iron [2.09]	Typh + Paratyph [53.8]	Vit A [1.89]	Diarrhea [1.96]	Neonatal [1.52]	LRI [0.64]	Malaria [121.43]	Congenital [0.6]	Drown [0.47]	ID [6.37]
Andhra Pradesh	Iron [1.62]	Typh + Paratyph [27.99]	Vit A [1.51]	Neonatal [1.69]	Diarrhea [0.68]	Congenital [0.44]	ID [5.32]	Malaria [42.81]	Dermatitis [0.52]	Skin Viral [0.85]
Arunachal Pradesh	Iron [1.78]	Vit A [1.71]	Neonatal [1.59]	Typh + Paratyph [34.03]	Malaria [189.29]	Diarrhea [1.1]	Congenital [0.47]	LRI [0.38]	Drown [0.29]	ID [5.2]
Assam	Malaria [205.55]	Iron [1.47]	Typh + Paratyph [28.8]	Diarrhea [1.68]	Vit A [1.47]	Neonatal [1.47]	Drown [0.72]	LRI [0.6]	Hep [6.38]	Congenital [0.59]
Bihar	Diarrhea [1.87]	Iron [1.46]	Vit A [1.35]	Typh + Paratyph [4.65]	LRI [0.64]	Neonatal [1.79]	Drown [0.53]	Encepha [3.38]	Animal [5.89]	Congenital [0.52]
Chhattisgarh	Typh + Paratyph [31.78]	Diarrhea [1.81]	Iron [1.33]	Malaria [98.48]	Drown [0.94]	LRI [0.77]	Vit A [1.12]	Neonatal [1.59]	Congenital [0.59]	Road Inj [0.3]
Delhi	Iron [6.17]	Typh + Paratyph [2464.07]	Vit A [3.26]	Neonatal [1.46]	Diarrhea [2.65]	Congenital [0.65]	LRI [1.53]	Leukemia [0.86]	Dengue [94.83]	Road Inj [0.33]
Goa	Neonatal [1.8]	Vit A [3.35]	Congenital [0.88]	Diarrhea [2.45]	Drown [1.17]	LRI [1.75]	Epilepsy [1.25]	Dermatitis [0.46]	Road Inj [0.34]	Skin Viral [0.79]
Gujarat	Iron [3.69]	Vit A [2.16]	Typh + Paratyph [78.64]	Neonatal [1.53]	Malaria [269.63]	Diarrhea [1.34]	Congenital [0.61]	LRI [0.49]	Hemog [1.72]	ID [5.12]
Haryana	Iron [4.72]	Typh + Paratyph [187.72]	Vit A [2.53]	Neonatal [1.51]	Diarrhea [1.17]	Congenital [0.53]	Malaria [142.96]	LRI [0.44]	Hemog [2.1]	Oth NTD [6.06]
Himachal Pradesh	Iron [5.53]	Vit A [2.49]	Typh + Paratyph [254.29]	Neonatal [1.63]	Congenital [0.6]	Diarrhea [1.5]	LRI [0.58]	Hemog [2.2]	Dermatitis [0.49]	ID [5.11]
Jammu & Kashmir	Iron [1.95]	Vit A [2.1]	Neonatal [1.51]	Typh + Paratyph [52.65]	Congenital [0.77]	Diarrhea [1.5]	LRI [0.77]	Road Inj [0.4]	Drown [0.46]	ID [6.22]
Jharkhand	Diarrhea [1.99]	Iron [1.8]	Vit A [1.55]	Typh + Paratyph [10.58]	Malaria [48.0]	Drown [0.91]	LRI [0.61]	Neonatal [1.7]	Congenital [0.73]	Road Inj [0.31]
Karnataka	Iron [1.84]	Vit A [1.92]	Neonatal [1.77]	Typh + Paratyph [43.18]	Congenital [0.82]	Diarrhea [1.29]	Drown [0.52]	LRI [0.36]	Epilepsy [0.91]	ID [5.35]
Kerala	Neonatal [2.01]	Vit A [2.41]	Iron [1.54]	Congenital [0.74]	Diarrhea [1.06]	Dermatitis [0.48]	Skin Viral [0.8]	Conduct [0.78]	ID [4.9]	Epilepsy [0.7]
Madhya Pradesh	Typh + Paratyph [21.21]	Iron [2.13]	Vit A [1.67]	Diarrhea [1.01]	Neonatal [1.51]	LRI [0.54]	Malaria [30.9]	Drown [0.58]	Congenital [0.48]	Animal [5.77]
Maharashtra	Iron [3.24]	Vit A [2.29]	Neonatal [1.62]	Typh + Paratyph [143.63]	Congenital [0.66]	Diarrhea [1.52]	Malaria [208.67]	LRI [0.58]	Drown [0.39]	ID [5.18]

eFigure 7d. Leading ten causes of DALYs with the ratio of observed DALYs to DALYs expected on the basis of Socio-Demographic Index alone in 2017, 5-9 years, both sexes combined. The top ten causes contributing to DALYs are listed globally, by socio-demographic quintile, and then by GBD superregion, region, country, and subnationally where modeled. For each cell, the ratio of observed DALYs to DALYs expected on the basis of socio-demographic index (SDI) alone are listed. Abbreviations: DALY=disability-adjusted life year, GBD=Global Burden of Disease.

Values shown in brackets represent the ratio of observed DALYs to predicted DALYs on the basis of Socio-Demographic Index (SDI), rounded to two (2) digits. Color ranges (shown below) were calculated to place a roughly equal number of cells into each bin.

COLOR KEY:		[0.0-0.62]	(0.62-0.79]	(0.79-0.89]	(0.89-0.97]	(0.97-1.07]	(1.07-1.26]	(1.26-1.44]	(1.44-2.32]	2.32+
	1	2	3	4	5	6	7	8	9	10
Manipur	Typh + Paratyph [123.5]	Neonatal [1.69]	Vit A [1.36]	Diarrhea [1.38]	Iron [0.7]	Congenital [0.56]	Malaria [157.49]	Drown [0.42]	ID [6.81]	LRI [0.43]
Meghalaya	Iron [2.3]	Typh + Paratyph [82.44]	Malaria [457.64]	Vit A [2.07]	Neonatal [1.63]	Diarrhea [1.11]	Congenital [0.56]	LRI [0.48]	ID [6.25]	Dermatitis [0.52]
Mizoram	Typh + Paratyph [322.98]	Malaria [565.28]	Neonatal [1.66]	Vit A [1.85]	Congenital [0.54]	Diarrhea [1.21]	LRI [0.64]	Iron [0.63]	ID [6.14]	Dermatitis [0.51]
Nagaland	Typh + Paratyph [368.82]	Iron [2.14]	Malaria [993.73]	Neonatal [1.72]	Vit A [1.81]	LRI [0.77]	Congenital [0.47]	Drown [0.52]	Diarrhea [1.22]	HIV [11.2]
Odisha	Malaria [230.48]	Typh + Paratyph [29.14]	Diarrhea [1.95]	Vit A [1.7]	Neonatal [1.48]	LRI [0.58]	Iron [0.73]	Drown [0.54]	Congenital [0.54]	Animal [7.16]
Punjab	Iron [3.8]	Typh + Paratyph [198.42]	Vit A [2.38]	Neonatal [1.54]	Congenital [0.46]	Diarrhea [0.89]	Dermatitis [0.48]	ID [5.05]	Hemog [1.81]	Conduct [0.83]
Rajasthan	Typh + Paratyph [26.06]	Iron [1.8]	Vit A [1.58]	Diarrhea [0.85]	Neonatal [1.48]	LRI [0.53]	Malaria [27.37]	Drown [0.42]	Congenital [0.44]	ID [6.0]
Sikkim	Iron [3.65]	Typh + Paratyph [280.78]	Vit A [2.31]	Neonatal [1.67]	LRI [0.91]	Diarrhea [1.64]	Congenital [0.58]	Dermatitis [0.49]	Skin Viral [0.81]	Conduct [0.81]
Tamil Nadu	Iron [3.36]	Vit A [2.4]	Neonatal [1.57]	Typh + Paratyph [134.79]	Diarrhea [1.99]	Congenital [0.84]	Drown [0.56]	LRI [0.66]	Road Inj [0.28]	Encepha [2.41]
Telangana	Iron [2.67]	Typh + Paratyph [71.74]	Vit A [1.93]	Neonatal [1.65]	Diarrhea [1.46]	Congenital [0.53]	Malaria [89.05]	ID [5.31]	Drown [0.3]	LRI [0.29]
Tripura	Iron [1.76]	Vit A [2.04]	Malaria [231.63]	Neonatal [1.62]	Typh + Paratyph [22.67]	Diarrhea [1.11]	LRI [0.55]	Congenital [0.64]	Drown [0.4]	ID [6.25]
Uttar Pradesh	Typh + Paratyph [23.9]	Iron [1.66]	Vit A [1.67]	Diarrhea [1.42]	LRI [0.63]	Neonatal [1.46]	Malaria [33.74]	Congenital [0.59]	Meningitis [0.59]	Road Inj [0.27]
Uttarakhand	Typh + Paratyph [229.9]	Iron [2.99]	Vit A [1.94]	Neonatal [1.64]	Diarrhea [1.44]	Congenital [0.56]	LRI [0.63]	Road Inj [0.26]	Malaria [123.28]	Dermatitis [0.51]
W Bengal	Iron [1.61]	Vit A [1.84]	Neonatal [1.5]	Typh + Paratyph [15.43]	Diarrhea [0.87]	Congenital [0.59]	Drown [0.45]	ID [6.74]	LRI [0.3]	Malaria [54.38]
UTs other than Delhi	Iron [4.77]	Vit A [2.89]	Neonatal [1.75]	Typh + Paratyph [246.32]	Malaria [812.61]	Congenital [0.63]	Diarrhea [1.64]	LRI [0.68]	Drown [0.48]	Road Inj [0.27]
Nepal	Typh + Paratyph [3.98]	Iron [0.81]	Neonatal [1.9]	Diarrhea [0.52]	Vit A [0.65]	Road Inj [0.26]	Dermatitis [0.89]	Malaria [4.11]	LRI [0.2]	Drown [0.26]
Pakistan	Iron [1.95]	Diarrhea [1.25]	Road Inj [0.77]	Typh + Paratyph [10.61]	Drown [0.84]	LRI [0.61]	Neonatal [1.42]	Congenital [0.79]	Vit A [0.8]	Malaria [32.73]
**Southeast Asia, East Asia, and Oceania**	Drown [1.82]	Road Inj [0.75]	Neonatal [1.06]	Congenital [0.78]	Asthma [0.99]	Dermatitis [0.64]	LRI [1.0]	Leukemia [0.87]	Skin Viral [1.05]	Typh + Paratyph [225.32]
**East Asia**	Drown [2.65]	Road Inj [1.04]	Neonatal [1.35]	Congenital [0.75]	Asthma [0.81]	Leukemia [1.02]	Skin Viral [1.11]	Dermatitis [0.52]	Falls [1.22]	Hernia [4.38]
China	Drown [2.59]	Road Inj [1.03]	Neonatal [1.37]	Congenital [0.74]	Asthma [0.79]	Leukemia [1.01]	Skin Viral [1.11]	Dermatitis [0.51]	Falls [1.24]	Hernia [4.38]
N Korea	Drown [1.04]	Road Inj [0.47]	Congenital [0.54]	Vit A [0.58]	Neonatal [0.72]	Diarrhea [0.59]	Asthma [0.69]	LRI [0.32]	Leukemia [0.71]	Dermatitis [0.65]
Taiwan	Asthma [1.9]	Dermatitis [0.6]	Neonatal [0.57]	Congenital [0.77]	Skin Viral [1.04]	Conduct [0.81]	Anxiety [0.72]	Diarrhea [2.03]	Road Inj [0.51]	Urticaria [1.35]

eFigure 7d. Leading ten causes of DALYs with the ratio of observed DALYs to DALYs expected on the basis of Socio-Demographic Index alone in 2017, 5-9 years, both sexes combined. The top ten causes contributing to DALYs are listed globally, by socio-demographic quintile, and then by GBD superregion, region, country, and subnationally where modeled. For each cell, the ratio of observed DALYs to DALYs expected on the basis of socio-demographic index (SDI) alone are listed. Abbreviations: DALY=disability-adjusted life year, GBD=Global Burden of Disease.

Values shown in brackets represent the ratio of observed DALYs to predicted DALYs on the basis of Socio-Demographic Index (SDI), rounded to two (2) digits. Color ranges (shown below) were calculated to place a roughly equal number of cells into each bin.

COLOR KEY:	[0.0-0.62]	(0.62-0.79]	(0.79-0.89]	(0.89-0.97]	(0.97-1.07]	(1.07-1.26]	(1.26-1.44]	(1.44-2.32]	2.32+

	1	2	3	4	5	6	7	8	9	10
**Oceania**	LRI [1.0]	Iron [1.23]	Diarrhea [0.9]	Asthma [1.92]	Congenital [0.91]	Drown [0.73]	Road Inj [0.45]	RHD [4.01]	Dermatitis [1.02]	Typh + Paratyph [3.1]
Am Samoa	Asthma [2.17]	Iron [2.17]	Dermatitis [0.93]	Diarrhea [2.33]	Vit A [1.25]	Neonatal [0.69]	Congenital [0.58]	Skin Viral [0.99]	Road Inj [0.34]	Drown [0.6]
Micronesia	Iron [1.64]	Vit A [1.67]	Asthma [1.71]	Congenital [0.79]	Road Inj [0.5]	Dermatitis [1.03]	LRI [0.56]	Diarrhea [0.96]	Drown [0.47]	Neonatal [0.71]
Fiji	Iron [1.86]	Asthma [1.88]	Congenital [1.04]	LRI [1.33]	Road Inj [0.56]	Drown [0.8]	Diarrhea [1.86]	Dermatitis [0.97]	Neonatal [0.65]	Vit A [0.86]
Guam	Asthma [2.22]	Diarrhea [5.79]	Iron [3.91]	Dermatitis [0.87]	Congenital [0.91]	Road Inj [0.89]	Neonatal [0.74]	Skin Viral [0.97]	LRI [2.33]	Conduct [0.79]
Kiribati	Iron [1.28]	Congenital [1.31]	Meningitis [1.03]	Vit A [0.95]	Asthma [1.82]	Measles [1.74]	LRI [0.45]	Diarrhea [0.49]	Road Inj [0.3]	Typh + Paratyph [1.68]
Marshall	Vit A [1.17]	Asthma [1.57]	Congenital [0.85]	Road Inj [0.51]	LRI [0.5]	Dermatitis [1.04]	Iron [0.57]	Diarrhea [0.75]	Neonatal [0.74]	Drown [0.43]
N Mariana	Asthma [2.28]	Neonatal [0.84]	Dermatitis [0.9]	Diarrhea [3.19]	Vit A [1.94]	Congenital [0.6]	Iron [1.24]	Skin Viral [0.98]	Road Inj [0.43]	Drown [0.8]
PNG	LRI [0.94]	Iron [1.09]	Diarrhea [0.72]	Asthma [1.99]	Drown [0.76]	Congenital [0.91]	Road Inj [0.43]	RHD [4.08]	Typh + Paratyph [1.62]	Meningitis [0.43]
Samoa	Iron [1.55]	Vit A [1.34]	Asthma [1.62]	Dermatitis [1.04]	Congenital [0.57]	Diarrhea [0.97]	Neonatal [0.73]	Road Inj [0.27]	Skin Viral [1.06]	LRI [0.31]
Solomon	Iron [1.62]	LRI [0.65]	Vit A [0.77]	Road Inj [0.45]	Asthma [1.48]	Congenital [0.78]	Drown [0.41]	Dermatitis [1.02]	Diarrhea [0.3]	Falls [1.79]
Tonga	Asthma [2.26]	Meningitis [2.17]	Dermatitis [1.0]	Road Inj [0.43]	Iron [0.79]	Diarrhea [1.44]	Vit A [0.88]	Neonatal [0.69]	Congenital [0.52]	LRI [0.62]
Vanuatu	Iron [1.09]	Asthma [1.76]	Road Inj [0.55]	Vit A [0.98]	Congenital [0.88]	LRI [0.47]	Drown [0.55]	Dermatitis [1.02]	Diarrhea [0.42]	Typh + Paratyph [3.14]
**Southeast Asia**	Drown [0.96]	Congenital [0.78]	Asthma [1.17]	Typh + Paratyph [142.28]	LRI [0.92]	Road Inj [0.42]	Neonatal [0.72]	Diarrhea [1.52]	Dermatitis [0.82]	Dengue [22.23]
Cambodia	Iron [1.59]	Drown [0.92]	Mech [2.85]	Vit A [0.71]	LRI [0.41]	Congenital [0.6]	Road Inj [0.33]	Typh + Paratyph [3.92]	Dermatitis [1.02]	Asthma [0.82]
Indonesia	Typh + Paratyph [254.58]	Asthma [1.17]	Diarrhea [1.98]	Dengue [33.36]	Vit A [1.07]	Road Inj [0.43]	Neonatal [0.73]	Congenital [0.58]	Drown [0.64]	Dermatitis [0.61]
Laos	Drown [1.03]	Road Inj [0.69]	Typh + Paratyph [15.71]	Vit A [0.99]	Congenital [0.79]	LRI [0.57]	Asthma [1.11]	Diarrhea [0.69]	Iron [0.53]	Dermatitis [1.03]
Malaysia	Dermatitis [0.95]	Neonatal [0.81]	Asthma [0.99]	Congenital [0.7]	Diarrhea [2.36]	Skin Viral [0.98]	Road Inj [0.46]	Dengue [427.11]	Leukemia [0.96]	LRI [1.59]
Maldives	Dermatitis [0.96]	Asthma [1.01]	Congenital [0.63]	Neonatal [0.66]	Dengue [27.39]	Iron [0.69]	Drown [0.53]	Skin Viral [1.0]	Conduct [0.85]	Road Inj [0.22]
Mauritius	Iron [2.61]	Asthma [1.27]	Dermatitis [0.91]	Neonatal [0.82]	Congenital [0.72]	Diarrhea [1.82]	Skin Viral [0.98]	Epilepsy [1.05]	Conduct [0.83]	Vit A [0.7]
Myanmar	Typh + Paratyph [36.54]	Congenital [1.06]	Drown [0.9]	Iron [0.83]	LRI [0.67]	Vit A [0.82]	Asthma [1.0]	Dermatitis [1.04]	Road Inj [0.34]	Diarrhea [0.84]
Philippines	LRI [1.96]	Congenital [1.19]	Drown [1.18]	Asthma [1.72]	Dengue [34.36]	Road Inj [0.5]	Dermatitis [1.05]	Diarrhea [1.42]	Neonatal [0.77]	Leukemia [1.04]
Sri Lanka	Congenital [0.83]	Dermatitis [0.9]	Asthma [1.0]	Typh + Paratyph [308.81]	Neonatal [0.74]	Vit A [1.15]	Iron [1.02]	Skin Viral [1.02]	Epilepsy [1.0]	Conduct [0.83]
Seychelles	Drown [1.77]	LRI [1.91]	Congenital [0.88]	Dermatitis [0.94]	Neonatal [0.81]	Asthma [1.04]	Meningitis [2.15]	Vit A [0.8]	Skin Viral [0.99]	Road Inj [0.3]
Thailand	Drown [1.97]	Typh + Paratyph [475.62]	Road Inj [0.66]	Congenital [0.79]	Dermatitis [0.98]	Asthma [0.98]	Neonatal [0.67]	Skin Viral [0.99]	LRI [0.85]	Conduct [0.84]

eFigure 7d. Leading ten causes of DALYs with the ratio of observed DALYs to DALYs expected on the basis of Socio-Demographic Index alone in 2017, 5-9 years, both sexes combined. The top ten causes contributing to DALYs are listed globally, by socio-demographic quintile, and then by GBD superregion, region, country, and subnationally where modeled. For each cell, the ratio of observed DALYs to DALYs expected on the basis of socio-demographic index (SDI) alone are listed. Abbreviations: DALY=disability-adjusted life year, GBD=Global Burden of Disease.

Values shown in brackets represent the ratio of observed DALYs to predicted DALYs on the basis of Socio-Demographic Index (SDI), rounded to two (2) digits. Color ranges (shown below) were calculated to place a roughly equal number of cells into each bin.

COLOR KEY:	[0.0-0.62]	(0.62-0.79]	(0.79-0.89]	(0.89-0.97]	(0.97-1.07]	(1.07-1.26]	(1.26-1.44]	(1.44-2.32]	2.32+

	1	2	3	4	5	6	7	8	9	10
Timor-Leste	Iron [1.05]	Drown [0.72]	Congenital [0.8]	Typh + Paratyph [8.5]	Vit A [0.66]	LRI [0.39]	Asthma [0.96]	Dermatitis [1.02]	Diarrhea [0.53]	Road Inj [0.26]
Vietnam	Drown [1.01]	Congenital [0.6]	Dermatitis [0.84]	Typh + Paratyph [44.47]	Neonatal [0.61]	Asthma [0.74]	Skin Viral [1.04]	Road Inj [0.23]	Vit A [0.5]	Conduct [0.87]
**Sub-Saharan Africa**	Malaria [37.89]	Iron [1.43]	HIV [21.71]	Diarrhea [0.93]	Vit A [1.22]	LRI [0.51]	Road Inj [0.43]	Meningitis [0.6]	Congenital [0.59]	Dermatitis [1.0]
**Central Sub-Saharan Africa**	Malaria [71.78]	Iron [1.51]	Vit A [1.34]	Diarrhea [1.0]	Road Inj [0.73]	HIV [13.54]	Meningitis [0.77]	LRI [0.45]	Hemog [2.07]	Congenital [0.59]
Angola	Iron [1.97]	Diarrhea [1.28]	Malaria [30.76]	Road Inj [0.72]	HIV [15.37]	Vit A [1.16]	Meningitis [0.77]	LRI [0.42]	Neonatal [1.21]	Dermatitis [1.16]
C African Rep	Malaria [5.24]	Road Inj [1.65]	Conflict Terror [167.03]	Iron [1.66]	HIV [29.25]	Diarrhea [0.84]	Vit A [1.06]	Meningitis [0.87]	LRI [0.55]	Hemog [2.44]
Congo	HIV [77.88]	Malaria [611.67]	Iron [2.16]	Vit A [2.43]	Diarrhea [2.9]	Road Inj [0.86]	LRI [0.82]	Neonatal [1.13]	Meningitis [1.62]	Asthma [1.22]
Congo DR	Malaria [9.21]	Iron [1.07]	Vit A [1.03]	Diarrhea [0.51]	Road Inj [0.59]	LRI [0.33]	Meningitis [0.51]	HIV [8.58]	Hemog [1.71]	Oncho [144.34]
Eq Guinea	HIV [186.66]	Malaria [2142.75]	Iron [1.59]	Road Inj [0.69]	Diarrhea [2.16]	Vit A [1.3]	Dermatitis [1.12]	Asthma [1.1]	Epilepsy [1.67]	Neonatal [0.73]
Gabon	Iron [5.13]	Malaria [2274.7]	HIV [38.52]	Road Inj [0.99]	Vit A [2.23]	Neonatal [1.22]	Diarrhea [2.68]	Dermatitis [1.1]	Asthma [1.2]	Epilepsy [1.8]
**Eastern Sub-Saharan Africa**	HIV [32.87]	Diarrhea [0.73]	Vit A [0.89]	Iron [0.79]	Malaria [3.72]	LRI [0.41]	Road Inj [0.33]	Congenital [0.59]	Neonatal [1.46]	Meningitis [0.4]
Burundi	Malaria [2.34]	Diarrhea [0.48]	LRI [0.39]	Iron [0.61]	Road Inj [0.43]	HIV [11.55]	Congenital [0.72]	Vit A [0.43]	Typh + Paratyph [0.57]	Dermatitis [1.18]
Comoros	Iron [1.39]	Diarrhea [0.88]	Vit A [0.87]	LRI [0.49]	Neonatal [1.61]	Road Inj [0.38]	Congenital [0.64]	Dermatitis [1.16]	Asthma [0.96]	Meningitis [0.44]
Djibouti	HIV [18.31]	Diarrhea [0.85]	LRI [0.56]	Iron [0.69]	Road Inj [0.45]	Neonatal [1.35]	Congenital [0.66]	Dermatitis [1.16]	Typh + Paratyph [4.73]	Meningitis [0.54]
Eritrea	Diarrhea [1.22]	Iron [1.37]	LRI [0.56]	Vit A [0.74]	Road Inj [0.48]	HIV [8.72]	Neonatal [1.64]	Meningitis [0.52]	Congenital [0.63]	Typh + Paratyph [1.59]
Ethiopia	Diarrhea [0.59]	Vit A [0.97]	Iron [0.6]	HIV [10.88]	LRI [0.31]	Meningitis [0.39]	Neonatal [1.77]	Typh + Paratyph [0.67]	Road Inj [0.25]	Dermatitis [1.05]
Kenya	HIV [26.8]	Diarrhea [1.49]	Neonatal [1.73]	Vit A [1.02]	Congenital [0.71]	LRI [0.47]	Typh + Paratyph [6.85]	Dermatitis [1.15]	iNTS [3.09]	Meningitis [0.68]
Baringo	Diarrhea [1.04]	Vit A [0.95]	Neonatal [1.88]	Leish [23.69]	Congenital [0.68]	LRI [0.37]	Typh + Paratyph [2.65]	iNTS [2.35]	Dermatitis [1.12]	Meningitis [0.5]
Bomet	HIV [50.85]	Neonatal [2.03]	Diarrhea [1.09]	Vit A [0.68]	Dermatitis [1.12]	Congenital [0.54]	Typh + Paratyph [5.06]	Asthma [0.67]	iNTS [1.9]	LRI [0.2]
Bungoma	Diarrhea [1.09]	Vit A [1.31]	Neonatal [1.89]	Malaria [25.13]	Congenital [0.62]	Iron [0.52]	iNTS [2.45]	Dermatitis [1.12]	HIV [6.87]	Typh + Paratyph [2.96]
Busia	Malaria [33.66]	Diarrhea [0.89]	Vit A [1.16]	Neonatal [1.89]	iNTS [2.69]	Congenital [0.67]	LRI [0.37]	Dermatitis [1.12]	Typh + Paratyph [2.03]	Meningitis [0.47]
Elgeyo-Marakwet	Diarrhea [1.58]	Neonatal [2.01]	HIV [13.84]	Congenital [0.65]	Dermatitis [1.13]	Vit A [0.62]	Typh + Paratyph [5.25]	LRI [0.35]	Meningitis [0.52]	Asthma [0.68]
Embu	Diarrhea [2.07]	HIV [20.53]	Vit A [1.51]	Neonatal [1.87]	Congenital [0.71]	LRI [0.51]	Iron [0.62]	Dermatitis [1.13]	Typh + Paratyph [12.88]	Meningitis [0.77]

eFigure 7d. Leading ten causes of DALYs with the ratio of observed DALYs to DALYs expected on the basis of Socio-Demographic Index alone in 2017, 5-9 years, both sexes combined. The top ten causes contributing to DALYs are listed globally, by socio-demographic quintile, and then by GBD superregion, region, country, and subnationally where modeled. For each cell, the ratio of observed DALYs to DALYs expected on the basis of socio-demographic index (SDI) alone are listed. Abbreviations: DALY=disability-adjusted life year, GBD=Global Burden of Disease.

Values shown in brackets represent the ratio of observed DALYs to predicted DALYs on the basis of Socio-Demographic Index (SDI), rounded to two (2) digits. Color ranges (shown below) were calculated to place a roughly equal number of cells into each bin.

COLOR KEY:	[0.0-0.62]	(0.62-0.79]	(0.79-0.89]	(0.89-0.97]	(0.97-1.07]	(1.07-1.26]	(1.26-1.44]	(1.44-2.32]	2.32+

	1	2	3	4	5	6	7	8	9	10
Garissa	Diarrhea [0.5]	HIV [13.49]	Neonatal [2.59]	Vit A [0.58]	iNTS [1.38]	Congenital [0.59]	Dermatitis [1.13]	Typh + Paratyph [0.58]	LRI [0.21]	Meningitis [0.28]
HomaBay	HIV [88.02]	Diarrhea [1.24]	Vit A [1.34]	Road Inj [0.8]	LRI [0.59]	iNTS [3.72]	Malaria [10.1]	Congenital [0.85]	Meningitis [0.71]	Iron [0.65]
Isiolo	Diarrhea [0.64]	HIV [13.72]	LRI [0.46]	Conflict Terror [123.04]	Vit A [0.73]	Congenital [0.79]	Meningitis [0.58]	Neonatal [1.76]	Typh + Paratyph [1.32]	iNTS [1.76]
Kajiado	Neonatal [2.03]	Diarrhea [1.45]	Vit A [0.89]	Congenital [0.67]	Typh + Paratyph [13.48]	Dermatitis [1.13]	HIV [9.55]	LRI [0.4]	Meningitis [0.69]	Asthma [0.71]
Kakamega	HIV [21.41]	Diarrhea [1.17]	Vit A [1.28]	Malaria [18.35]	LRI [0.49]	Neonatal [1.61]	Congenital [0.75]	iNTS [2.82]	Typh + Paratyph [3.02]	Meningitis [0.63]
Kericho	HIV [26.69]	Neonatal [2.14]	Diarrhea [1.24]	Vit A [0.91]	Congenital [0.63]	iNTS [3.17]	Dermatitis [1.12]	Typh + Paratyph [6.17]	LRI [0.32]	Asthma [0.68]
Kiambu	Neonatal [1.93]	Diarrhea [2.11]	HIV [20.96]	Congenital [0.77]	LRI [0.7]	Dermatitis [1.14]	Typh + Paratyph [32.81]	Meningitis [1.15]	Vit A [0.68]	Road Inj [0.31]
Kilifi	HIV [18.98]	Diarrhea [1.07]	Vit A [0.99]	Neonatal [1.79]	Iron [0.61]	Congenital [0.68]	Typh + Paratyph [3.05]	LRI [0.37]	Dermatitis [1.12]	Meningitis [0.52]
Kirinyaga	HIV [26.0]	Diarrhea [2.09]	Neonatal [1.94]	Congenital [0.76]	LRI [0.58]	Typh + Paratyph [13.48]	Dermatitis [1.14]	Meningitis [0.86]	Road Inj [0.32]	Asthma [0.72]
Kisii	Diarrhea [2.21]	Neonatal [1.56]	Congenital [0.73]	LRI [0.52]	iNTS [4.12]	Typh + Paratyph [10.71]	Dermatitis [1.13]	Vit A [0.7]	Meningitis [0.73]	Road Inj [0.29]
Kisumu	HIV [46.25]	Diarrhea [1.76]	Vit A [1.21]	Malaria [73.26]	LRI [0.66]	iNTS [4.71]	Congenital [0.82]	Typh + Paratyph [8.38]	Meningitis [0.89]	Neonatal [1.1]
Kitui	HIV [21.59]	Diarrhea [1.1]	Neonatal [2.15]	Typh + Paratyph [3.41]	Congenital [0.61]	iNTS [2.36]	Dermatitis [1.12]	LRI [0.29]	Meningitis [0.41]	Vit A [0.39]
Kwale	HIV [17.37]	Diarrhea [0.85]	Vit A [0.95]	Neonatal [1.84]	Malaria [15.17]	iNTS [2.81]	Typh + Paratyph [3.21]	Congenital [0.67]	LRI [0.37]	Dermatitis [1.12]
Laikipia	HIV [51.49]	Neonatal [2.01]	Diarrhea [1.71]	Vit A [0.94]	Dermatitis [1.14]	Congenital [0.61]	Typh + Paratyph [17.58]	Asthma [0.74]	LRI [0.33]	Epilepsy [0.85]
Lamu	Conflict Terror [1064.98]	Diarrhea [0.89]	LRI [0.5]	Neonatal [1.6]	Congenital [0.77]	Vit A [0.72]	HIV [9.39]	Typh + Paratyph [3.39]	Meningitis [0.66]	iNTS [2.39]
Machakos	HIV [31.21]	Diarrhea [1.51]	Neonatal [1.95]	Typh + Paratyph [10.29]	Congenital [0.65]	Dermatitis [1.13]	Vit A [0.66]	LRI [0.38]	iNTS [2.64]	Meningitis [0.56]
Makueni	HIV [60.19]	Diarrhea [0.9]	Neonatal [2.0]	Typh + Paratyph [3.59]	Dermatitis [1.12]	Vit A [0.57]	Congenital [0.55]	Asthma [0.64]	iNTS [1.54]	LRI [0.19]
Mandera	Diarrhea [0.48]	Vit A [0.87]	HIV [16.81]	Iron [0.55]	LRI [0.29]	iNTS [1.41]	Neonatal [2.19]	Typh + Paratyph [0.58]	Meningitis [0.37]	Congenital [0.6]
Marsabit	HIV [24.64]	Diarrhea [0.67]	Neonatal [2.69]	Vit A [0.59]	iNTS [1.37]	Congenital [0.59]	LRI [0.24]	Typh + Paratyph [0.65]	Dermatitis [1.13]	Meningitis [0.34]
Meru	HIV [29.78]	Diarrhea [1.72]	Neonatal [1.94]	LRI [0.59]	Congenital [0.77]	Vit A [0.85]	Meningitis [0.82]	Typh + Paratyph [7.91]	Dermatitis [1.13]	Road Inj [0.33]
Migori	HIV [60.36]	Diarrhea [1.24]	LRI [0.63]	Vit A [0.98]	Malaria [8.96]	iNTS [3.52]	Meningitis [0.75]	Congenital [0.87]	Road Inj [0.44]	Typh + Paratyph [2.26]

eFigure 7d. Leading ten causes of DALYs with the ratio of observed DALYs to DALYs expected on the basis of Socio-Demographic Index alone in 2017, 5-9 years, both sexes combined. The top ten causes contributing to DALYs are listed globally, by socio-demographic quintile, and then by GBD superregion, region, country, and subnationally where modeled. For each cell, the ratio of observed DALYs to DALYs expected on the basis of socio-demographic index (SDI) alone are listed. Abbreviations: DALY=disability-adjusted life year, GBD=Global Burden of Disease.

Values shown in brackets represent the ratio of observed DALYs to predicted DALYs on the basis of Socio-Demographic Index (SDI), rounded to two (2) digits. Color ranges (shown below) were calculated to place a roughly equal number of cells into each bin.

COLOR KEY:		[0.0-0.62]	(0.62-0.79]	(0.79-0.89]	(0.89-0.97]	(0.97-1.07]	(1.07-1.26]	(1.26-1.44]	(1.44-2.32]	2.32+
	1	2	3	4	5	6	7	8	9	10
Mombasa	Neonatal [1.57]	Diarrhea [1.78]	Congenital [0.77]	Typh + Paratyph [33.74]	LRI [0.65]	Dermatitis [1.14]	HIV [12.02]	Vit A [0.86]	Meningitis [1.07]	iNTS [4.44]
Murang'a	HIV [34.18]	Diarrhea [2.0]	Neonatal [1.95]	Congenital [0.71]	LRI [0.49]	Typh + Paratyph [12.01]	Dermatitis [1.13]	Meningitis [0.76]	Road Inj [0.28]	Vit A [0.52]
Nairobi	Neonatal [1.73]	Dermatitis [1.37]	Congenital [0.92]	Diarrhea [2.67]	LRI [1.63]	Typh + Paratyph [293.31]	Vit A [1.24]	Road Inj [0.49]	Meningitis [2.81]	Asthma [0.83]
Nakuru	HIV [26.41]	Diarrhea [2.0]	Neonatal [1.82]	Congenital [0.79]	LRI [0.63]	Typh + Paratyph [19.04]	Dermatitis [1.14]	Meningitis [0.95]	Vit A [0.7]	Epilepsy [1.29]
Nandi	HIV [25.02]	Neonatal [2.05]	Diarrhea [1.17]	Vit A [0.88]	Typh + Paratyph [6.63]	Congenital [0.62]	Dermatitis [1.12]	iNTS [3.07]	LRI [0.31]	Asthma [0.68]
Narok	Diarrhea [0.72]	Vit A [0.9]	Neonatal [2.59]	Typh + Paratyph [1.57]	iNTS [2.04]	HIV [7.53]	Congenital [0.61]	LRI [0.27]	Dermatitis [1.12]	Meningitis [0.37]
Nyamira	Diarrhea [2.51]	HIV [23.47]	Neonatal [1.77]	LRI [0.73]	iNTS [5.97]	Congenital [0.81]	Typh + Paratyph [18.61]	Meningitis [1.09]	Road Inj [0.39]	Dermatitis [1.14]
Nyandarua	HIV [30.45]	Diarrhea [2.44]	Neonatal [2.06]	LRI [0.91]	Congenital [0.92]	Meningitis [1.31]	Typh + Paratyph [15.94]	Road Inj [0.42]	Vit A [0.82]	Dermatitis [1.14]
Nyeri	HIV [42.48]	Diarrhea [2.15]	Neonatal [1.92]	LRI [0.8]	Congenital [0.84]	Meningitis [1.24]	Road Inj [0.4]	Dermatitis [1.14]	Typh + Paratyph [19.93]	Asthma [0.76]
Samburu	HIV [42.12]	Diarrhea [0.49]	Neonatal [3.02]	iNTS [1.1]	Vit A [0.35]	Dermatitis [1.13]	Congenital [0.52]	Typh + Paratyph [0.45]	Meningitis [0.23]	LRI [0.15]
Siaya	Malaria [44.53]	HIV [18.75]	Diarrhea [1.11]	Vit A [0.87]	iNTS [3.58]	Congenital [0.75]	LRI [0.44]	Typh + Paratyph [3.2]	Neonatal [1.22]	Meningitis [0.57]
TaitaTaveta	HIV [42.95]	Diarrhea [1.49]	Neonatal [1.79]	Congenital [0.77]	LRI [0.59]	Typh + Paratyph [13.71]	Dermatitis [1.14]	Meningitis [0.85]	Road Inj [0.29]	iNTS [2.99]
TanaRiver	HIV [32.01]	Vit A [1.31]	Iron [1.08]	Diarrhea [0.58]	LRI [0.43]	Neonatal [2.16]	Congenital [0.75]	Typh + Paratyph [1.33]	iNTS [2.01]	Meningitis [0.52]
TharakaNithi	Diarrhea [2.32]	Neonatal [1.52]	LRI [0.64]	Congenital [0.78]	Typh + Paratyph [12.97]	Dermatitis [1.13]	Meningitis [0.91]	Road Inj [0.33]	Vit A [0.64]	iNTS [2.91]
TransNzoia	HIV [38.87]	Diarrhea [1.37]	Neonatal [1.95]	Vit A [1.0]	Congenital [0.6]	Dermatitis [1.12]	Typh + Paratyph [5.34]	iNTS [2.48]	LRI [0.31]	Meningitis [0.48]
Turkana	HIV [61.45]	Diarrhea [0.63]	Vit A [0.7]	Neonatal [2.63]	iNTS [1.25]	Congenital [0.62]	Meningitis [0.34]	LRI [0.22]	PEM [0.64]	Dermatitis [1.12]
UasinGishu	HIV [26.68]	Diarrhea [1.93]	Neonatal [1.83]	Dermatitis [1.66]	Vit A [0.96]	Congenital [0.69]	Typh + Paratyph [17.51]	LRI [0.47]	Meningitis [0.74]	Asthma [0.72]
Vihiga	Diarrhea [1.43]	Vit A [1.05]	LRI [0.63]	Neonatal [1.84]	iNTS [3.85]	Congenital [0.81]	Typh + Paratyph [5.2]	Malaria [25.05]	Meningitis [0.77]	HIV [8.69]
Wajir	Diarrhea [0.55]	Neonatal [2.97]	Vit A [0.41]	iNTS [1.21]	LRI [0.24]	Congenital [0.67]	Typh + Paratyph [0.36]	Meningitis [0.35]	Dermatitis [1.12]	Measles [0.11]
WestPokot	Diarrhea [0.77]	Neonatal [2.49]	HIV [10.79]	Leish [10.31]	Vit A [0.62]	iNTS [1.66]	Congenital [0.55]	Dermatitis [1.12]	Typh + Paratyph [0.88]	LRI [0.2]
Madagascar	Diarrhea [0.93]	PEM [2.1]	Iron [0.96]	LRI [0.47]	Vit A [0.71]	Congenital [0.94]	Malaria [0.87]	Road Inj [0.29]	Asthma [1.06]	Dermatitis [1.17]

eFigure 7d. Leading ten causes of DALYs with the ratio of observed DALYs to DALYs expected on the basis of Socio-Demographic Index alone in 2017, 5-9 years, both sexes combined. The top ten causes contributing to DALYs are listed globally, by socio-demographic quintile, and then by GBD superregion, region, country, and subnationally where modeled. For each cell, the ratio of observed DALYs to DALYs expected on the basis of socio-demographic index (SDI) alone are listed. Abbreviations: DALY=disability-adjusted life year, GBD=Global Burden of Disease.

Values shown in brackets represent the ratio of observed DALYs to predicted DALYs on the basis of Socio-Demographic Index (SDI), rounded to two (2) digits. Color ranges (shown below) were calculated to place a roughly equal number of cells into each bin.

COLOR KEY:	[0.0-0.62]	(0.62-0.79]	(0.79-0.89]	(0.89-0.97]	(0.97-1.07]	(1.07-1.26]	(1.26-1.44]	(1.44-2.32]	2.32+

	1	2	3	4	5	6	7	8	9	10
Malawi	HIV [76.22]	Iron [1.16]	Malaria [2.28]	Diarrhea [0.58]	Vit A [0.86]	LRI [0.33]	Congenital [0.62]	Meningitis [0.39]	Road Inj [0.29]	Dermatitis [1.17]
Mozambique	HIV [116.58]	Malaria [3.76]	Iron [1.06]	Diarrhea [0.55]	Vit A [0.87]	LRI [0.4]	Typh + Paratyph [0.97]	Congenital [0.72]	Road Inj [0.27]	Asthma [0.89]
Rwanda	Iron [0.78]	Diarrhea [0.54]	Vit A [0.75]	Road Inj [0.48]	Malaria [4.42]	LRI [0.37]	HIV [8.25]	Asthma [1.14]	Congenital [0.61]	Neonatal [1.34]
Somalia	Measles [0.46]	Conflict Terror [36.17]	Diarrhea [0.45]	Vit A [0.81]	Road Inj [0.64]	Iron [0.72]	LRI [0.42]	Whooping [1.78]	Meningitis [0.48]	Dermatitis [1.16]
S Sudan	Conflict Terror [78.21]	Diarrhea [0.64]	Malaria [0.72]	Iron [0.89]	LRI [0.47]	Leish [4.18]	Vit A [0.62]	Road Inj [0.44]	Meningitis [0.47]	Measles [0.21]
Tanzania	HIV [26.53]	Iron [1.17]	Diarrhea [0.69]	Malaria [6.92]	LRI [0.51]	Vit A [0.75]	Congenital [0.65]	Asthma [1.11]	Neonatal [1.28]	Hemog [1.61]
Uganda	HIV [76.87]	Malaria [8.9]	Diarrhea [0.61]	Vit A [0.73]	Iron [0.67]	Road Inj [0.37]	LRI [0.33]	Neonatal [1.31]	Dermatitis [1.07]	Asthma [0.94]
Zambia	HIV [60.76]	Diarrhea [1.34]	Vit A [1.5]	Malaria [38.76]	Iron [0.77]	LRI [0.55]	Congenital [0.69]	Meningitis [0.71]	Road Inj [0.38]	Dermatitis [1.16]
**Southern Sub-Saharan Africa**	HIV [137.73]	Road Inj [0.93]	Diarrhea [2.99]	Vit A [1.77]	Dermatitis [1.31]	LRI [1.28]	Neonatal [1.02]	Iron [1.16]	TB [17.2]	Congenital [0.6]
Botswana	Diarrhea [4.43]	Vit A [2.36]	Iron [2.06]	Road Inj [0.94]	Neonatal [1.26]	Dermatitis [1.3]	LRI [1.31]	Congenital [0.47]	HIV [13.29]	Asthma [0.66]
Lesotho	HIV [116.35]	Road Inj [1.51]	Diarrhea [2.25]	LRI [1.02]	TB [6.88]	Vit A [1.5]	Iron [1.06]	Dermatitis [1.39]	Drown [0.57]	Meningitis [0.72]
Namibia	HIV [84.09]	Diarrhea [3.45]	Road Inj [0.93]	Iron [1.71]	Dermatitis [1.36]	Neonatal [1.07]	LRI [0.96]	Vit A [1.09]	TB [9.24]	Congenital [0.44]
S Africa	HIV [173.82]	Road Inj [1.09]	Vit A [1.89]	Dermatitis [1.27]	Neonatal [1.05]	Diarrhea [2.52]	Congenital [0.68]	LRI [1.21]	Iron [0.96]	Violence [3.11]
Swaziland	HIV [181.37]	Diarrhea [4.14]	Road Inj [1.42]	Iron [1.65]	Vit A [1.75]	LRI [1.22]	TB [9.19]	Dermatitis [1.4]	Neonatal [0.84]	Drown [0.57]
Zimbabwe	HIV [50.23]	Diarrhea [1.0]	TB [4.56]	LRI [0.69]	Iron [0.82]	Vit A [0.96]	Road Inj [0.45]	Dermatitis [1.39]	PEM [2.67]	Drown [0.53]
**Western Sub-Saharan Africa**	Malaria [48.76]	Iron [1.97]	Vit A [1.35]	Diarrhea [0.81]	LRI [0.53]	iNTS [3.31]	Meningitis [0.66]	Road Inj [0.4]	HIV [8.62]	Hemog [2.12]
Benin	Malaria [10.05]	Vit A [1.13]	Iron [1.03]	Diarrhea [0.54]	Road Inj [0.57]	iNTS [1.93]	LRI [0.32]	HIV [6.74]	Congenital [0.52]	Meningitis [0.34]
Burkina Faso	Malaria [2.56]	Iron [1.6]	Vit A [0.97]	Typh + Paratyph [0.94]	Diarrhea [0.33]	Hemog [1.74]	iNTS [1.52]	LRI [0.31]	Road Inj [0.3]	Meningitis [0.37]
Cameroon	Malaria [157.47]	HIV [24.39]	Iron [1.55]	Vit A [1.57]	Diarrhea [1.0]	LRI [0.53]	Road Inj [0.37]	iNTS [2.68]	Congenital [0.57]	Meningitis [0.57]
Cape Verde	Iron [1.64]	Neonatal [1.41]	Dermatitis [0.97]	Diarrhea [0.65]	Congenital [0.44]	Drown [0.37]	Epilepsy [0.98]	Asthma [0.63]	HIV [6.17]	Conduct [0.94]
Chad	Iron [1.92]	Malaria [0.8]	Diarrhea [0.6]	Vit A [0.88]	LRI [0.49]	Road Inj [0.47]	Meningitis [0.54]	iNTS [1.22]	HIV [11.5]	Typh + Paratyph [0.4]
Cote d'Ivoire	Malaria [19.42]	Iron [1.58]	Vit A [1.34]	Diarrhea [0.58]	iNTS [3.0]	LRI [0.48]	Road Inj [0.4]	Congenital [0.63]	HIV [7.33]	Meningitis [0.46]
Gambia	Iron [1.52]	Vit A [1.4]	Hemog [4.42]	Diarrhea [0.53]	HIV [9.85]	LRI [0.4]	Neonatal [1.64]	Meningitis [0.5]	Road Inj [0.3]	Congenital [0.53]
Ghana	Malaria [435.42]	Iron [2.18]	Vit A [2.23]	HIV [20.22]	Diarrhea [1.17]	Typh + Paratyph [18.42]	Road Inj [0.44]	Meningitis [1.1]	Hemog [3.04]	LRI [0.52]
Guinea	Malaria [4.23]	Iron [1.16]	Vit A [0.93]	iNTS [2.26]	Diarrhea [0.37]	LRI [0.4]	HIV [11.09]	Road Inj [0.35]	Meningitis [0.42]	Congenital [0.59]
Guinea-Bissau	HIV [38.56]	Diarrhea [0.84]	Iron [1.33]	Hemog [4.11]	Vit A [1.13]	Measles [0.97]	LRI [0.46]	Meningitis [0.53]	Congenital [0.67]	Road Inj [0.34]
Liberia	Malaria [2.47]	Iron [1.47]	Diarrhea [0.62]	Vit A [0.84]	Oncho [211.61]	Measles [0.5]	Hemog [1.85]	HIV [9.78]	iNTS [1.48]	LRI [0.28]
Mali	Malaria [1.54]	Iron [1.82]	Vit A [1.11]	Diarrhea [0.53]	iNTS [2.86]	Meningitis [0.56]	Road Inj [0.38]	HIV [10.79]	LRI [0.25]	Hemog [1.09]

eFigure 7d. Leading ten causes of DALYs with the ratio of observed DALYs to DALYs expected on the basis of Socio-Demographic Index alone in 2017, 5-9 years, both sexes combined. The top ten causes contributing to DALYs are listed globally, by socio-demographic quintile, and then by GBD superregion, region, country, and subnationally where modeled. For each cell, the ratio of observed DALYs to DALYs expected on the basis of socio-demographic index (SDI) alone are listed. Abbreviations: DALY=disability-adjusted life year, GBD=Global Burden of Disease.

Values shown in brackets represent the ratio of observed DALYs to predicted DALYs on the basis of Socio-Demographic Index (SDI), rounded to two (2) digits. Color ranges (shown below) were calculated to place a roughly equal number of cells into each bin.

COLOR KEY:	[0.0-0.62]	(0.62-0.79]	(0.79-0.89]	(0.89-0.97]	(0.97-1.07]	(1.07-1.26]	(1.26-1.44]	(1.44-2.32]	2.32+

	1	2	3	4	5	6	7	8	9	10
Mauritania	Iron [1.41]	Diarrhea [1.11]	Vit A [1.25]	Road Inj [0.53]	LRI [0.46]	Neonatal [1.24]	Congenital [0.51]	Meningitis [0.51]	Dermatitis [0.95]	Asthma [0.78]
Niger	Malaria [0.78]	Iron [1.3]	Vit A [0.7]	Diarrhea [0.33]	iNTS [1.44]	LRI [0.32]	Meningitis [0.4]	Road Inj [0.26]	Congenital [0.55]	Hemog [0.7]
Nigeria	Malaria [184.1]	Iron [2.58]	Vit A [1.56]	Diarrhea [1.15]	LRI [0.76]	iNTS [5.18]	Measles [6.38]	Meningitis [0.98]	Road Inj [0.43]	Hemog [2.72]
Sao Tome Principe	Vit A [1.53]	Iron [1.23]	LRI [0.74]	Neonatal [1.24]	Congenital [0.68]	Diarrhea [0.61]	Road Inj [0.34]	Other MN [2.47]	Dermatitis [0.95]	Typh + Paratyph [4.29]
Senegal	Iron [1.45]	Diarrhea [0.65]	Vit A [0.99]	Meningitis [0.48]	LRI [0.3]	Hemog [1.67]	Neonatal [1.54]	Malaria [1.52]	Congenital [0.53]	Road Inj [0.27]
Sierra Leone	Malaria [11.98]	Iron [1.68]	Vit A [1.09]	Diarrhea [0.58]	LRI [0.46]	iNTS [1.96]	Meningitis [0.5]	Road Inj [0.35]	Hemog [1.59]	Congenital [0.6]
Togo	Malaria [32.37]	Vit A [1.15]	Iron [1.02]	HIV [15.28]	Diarrhea [0.61]	iNTS [3.09]	Hemog [2.38]	LRI [0.3]	Neonatal [1.12]	Congenital [0.49]

eFigure 7e. Leading ten causes of DALYs with the ratio of observed DALYs to DALYs expected on the basis of Socio-Demographic Index alone in 2017, 10-19 years, both sexes combined. The top ten causes contributing to DALYs are listed globally, by socio-demographic quintile, and then by GBD superregion, region, country, and subnationally where modeled. For each cell, the ratio of observed DALYs to DALYs expected on the basis of socio-demographic index (SDI) alone are listed. Abbreviations: DALY=disability-adjusted life year, GBD=Global Burden of Disease.

Values shown in brackets represent the ratio of observed DALYs to predicted DALYs on the basis of Socio-Demographic Index (SDI), rounded to two (2) digits. Color ranges (shown below) were calculated to place a roughly equal number of cells into each bin.

COLOR KEY:									
[0.0-0.73]	(0.73-0.88]	(0.88-0.95]	(0.95-1.01]	(1.01-1.09]	(1.09-1.26]	(1.26-1.55]	(1.55-2.09]	2.09+	

	1	2	3	4	5	6	7	8	9	10
**Global**	Road Inj [0.66]	Headaches [0.85]	Iron [1.82]	Neonatal [1.09]	Conduct [0.9]	Anxiety [0.88]	HIV [25.29]	Depression [0.9]	Back Pain [0.82]	Diarrhea [2.7]
**Low SDI**	Iron [1.17]	HIV [18.81]	Road Inj [0.53]	Headaches [0.92]	Diarrhea [1.02]	Malaria [6.45]	Typh + Paratyph [3.25]	Conduct [0.9]	Neonatal [1.43]	Anxiety [0.94]
**Low-middle SDI**	Iron [1.53]	Road Inj [0.7]	Headaches [0.95]	Neonatal [1.15]	HIV [16.25]	Conduct [0.89]	Diarrhea [1.64]	Anxiety [0.92]	Back Pain [0.91]	Malaria [101.42]
**Middle SDI**	Road Inj [0.68]	Headaches [0.81]	Neonatal [1.21]	Violence [1.4]	Conduct [0.88]	Anxiety [0.78]	Back Pain [0.75]	Drown [0.86]	Iron [1.1]	Depression [0.73]
**High-middle SDI**	Road Inj [0.8]	Headaches [0.79]	Neonatal [1.25]	Back Pain [0.82]	Anxiety [0.76]	Conduct [0.95]	Depression [0.87]	Congenital [0.98]	Self Harm [0.68]	Violence [2.61]
**High SDI**	Headaches [0.93]	Anxiety [1.16]	Depression [1.33]	Back Pain [1.05]	Road Inj [0.49]	Drugs [3.06]	Conduct [0.98]	Neonatal [0.89]	Acne [1.63]	Self Harm [0.68]
**Central Europe, Eastern Europe, and Central Asia**	Headaches [0.9]	Road Inj [0.61]	Neonatal [1.38]	Self Harm [1.19]	Conduct [1.03]	Falls [1.74]	Back Pain [0.8]	Anxiety [0.62]	Depression [0.71]	Congenital [0.96]
**Central Asia**	Headaches [0.88]	Iron [2.39]	Self Harm [1.2]	Conduct [0.98]	Road Inj [0.46]	Epilepsy [1.85]	Neonatal [0.93]	LRI [2.27]	Back Pain [0.74]	Depression [0.79]
Armenia	Headaches [0.86]	Conduct [1.0]	Neonatal [0.98]	Iron [1.82]	Back Pain [0.74]	Congenital [0.94]	Depression [0.71]	Road Inj [0.28]	Anxiety [0.57]	Falls [1.21]
Azerbaijan	Headaches [0.87]	Conduct [1.0]	Neonatal [0.89]	Iron [1.74]	Epilepsy [1.79]	Back Pain [0.72]	LRI [2.38]	Congenital [0.9]	Road Inj [0.29]	Falls [1.31]
Georgia	Road Inj [0.7]	Headaches [0.87]	Conduct [1.0]	Neonatal [0.97]	Depression [0.72]	Back Pain [0.64]	Congenital [0.86]	Anxiety [0.58]	Falls [1.23]	Iron [1.27]
Kazakhstan	Self Harm [1.8]	Headaches [0.86]	Road Inj [0.6]	Conduct [1.01]	Neonatal [1.01]	Depression [0.91]	Back Pain [0.75]	Iron [2.14]	Congenital [0.98]	Drown [1.39]
Kyrgyzstan	Headaches [0.9]	Iron [1.86]	Self Harm [1.24]	Road Inj [0.5]	Conduct [0.97]	Neonatal [1.03]	Epilepsy [1.46]	Depression [0.84]	Back Pain [0.78]	Congenital [0.87]
Mongolia	Self Harm [2.02]	Headaches [0.88]	Road Inj [0.65]	Conduct [0.98]	Falls [2.21]	Neonatal [0.98]	Depression [0.96]	Congenital [1.0]	Iron [1.27]	Back Pain [0.74]
Tajikistan	Headaches [0.93]	LRI [1.55]	Epilepsy [1.98]	Drown [0.95]	Conduct [0.97]	Iron [0.94]	Neonatal [1.04]	Congenital [0.99]	Falls [2.11]	Road Inj [0.33]
Turkmenistan	Headaches [0.87]	Conduct [0.99]	Self Harm [1.05]	Iron [1.83]	LRI [2.55]	Epilepsy [1.78]	Neonatal [0.83]	Cirrhosis [8.37]	Back Pain [0.71]	Congenital [0.94]
Uzbekistan	Iron [2.73]	Headaches [0.89]	Self Harm [1.41]	LRI [2.26]	Conduct [0.97]	Road Inj [0.46]	Epilepsy [1.88]	Neonatal [0.91]	Back Pain [0.76]	Depression [0.81]
**Central Europe**	Headaches [0.87]	Neonatal [1.43]	Falls [1.95]	Road Inj [0.54]	Back Pain [0.91]	Conduct [1.03]	Anxiety [0.69]	Congenital [1.0]	Self Harm [0.63]	Depression [0.59]
Albania	Headaches [0.9]	Back Pain [1.13]	Falls [2.38]	Neonatal [1.19]	Conduct [0.97]	Road Inj [0.45]	Anxiety [0.79]	Congenital [0.96]	Depression [0.65]	Epilepsy [1.17]
Bosnia	Headaches [0.89]	Neonatal [1.42]	Falls [2.31]	Conduct [0.98]	Back Pain [0.92]	Road Inj [0.4]	Anxiety [0.77]	Congenital [0.91]	Depression [0.66]	Self Harm [0.54]
Bulgaria	Headaches [0.87]	Road Inj [0.61]	Falls [2.09]	Neonatal [1.26]	Conduct [1.02]	Back Pain [0.86]	Anxiety [0.74]	Congenital [1.14]	Depression [0.61]	Self Harm [0.56]
Croatia	Headaches [0.88]	Neonatal [1.55]	Road Inj [0.57]	Conduct [1.03]	Back Pain [0.89]	Anxiety [0.69]	Falls [1.2]	Depression [0.71]	Congenital [0.99]	Self Harm [0.61]
Czech	Headaches [0.87]	Falls [2.17]	Back Pain [1.01]	Conduct [1.06]	Road Inj [0.49]	Neonatal [0.93]	Anxiety [0.67]	Depression [0.65]	Congenital [0.89]	Self Harm [0.55]
Hungary	Headaches [0.88]	Neonatal [1.37]	Back Pain [1.01]	Falls [1.9]	Conduct [1.03]	Anxiety [0.69]	Road Inj [0.41]	Congenital [1.16]	Depression [0.62]	Self Harm [0.56]
Macedonia	Headaches [0.88]	Neonatal [1.38]	Falls [2.06]	Road Inj [0.5]	Conduct [1.0]	Anxiety [0.73]	Back Pain [0.69]	Congenital [0.89]	Depression [0.61]	Iron [1.74]
Montenegro	Headaches [0.88]	Neonatal [1.35]	Falls [1.95]	Back Pain [0.96]	Conduct [1.02]	Road Inj [0.48]	Anxiety [0.71]	Depression [0.64]	Congenital [0.87]	Self Harm [0.47]
Poland	Headaches [0.88]	Neonatal [1.59]	Road Inj [0.61]	Falls [1.87]	Conduct [1.04]	Back Pain [0.74]	Anxiety [0.68]	Self Harm [0.89]	Congenital [1.04]	Depression [0.52]
Romania	Headaches [0.87]	Neonatal [1.47]	Road Inj [0.58]	Back Pain [1.06]	Falls [2.04]	Conduct [1.01]	Iron [3.64]	Anxiety [0.67]	Upper Digest [4.67]	Congenital [0.94]
Serbia	Neonatal [1.82]	Headaches [0.87]	Falls [2.07]	Back Pain [1.0]	Conduct [1.01]	Anxiety [0.73]	Road Inj [0.37]	Congenital [0.89]	Depression [0.6]	Dermatitis [0.61]
Slovakia	Headaches [0.88]	Falls [1.9]	Back Pain [1.01]	Conduct [1.04]	Road Inj [0.5]	Neonatal [1.03]	Anxiety [0.68]	Congenital [1.05]	Depression [0.58]	Upper Digest [3.32]

eFigure 7e. Leading ten causes of DALYs with the ratio of observed DALYs to DALYs expected on the basis of Socio-Demographic Index alone in 2017, 10-19 years, both sexes combined. The top ten causes contributing to DALYs are listed globally, by socio-demographic quintile, and then by GBD superregion, region, country, and subnationally where modeled. For each cell, the ratio of observed DALYs to DALYs expected on the basis of socio-demographic index (SDI) alone are listed. Abbreviations: DALY=disability-adjusted life year, GBD=Global Burden of Disease.

Values shown in brackets represent the ratio of observed DALYs to predicted DALYs on the basis of Socio-Demographic Index (SDI), rounded to two (2) digits. Color ranges (shown below) were calculated to place a roughly equal number of cells into each bin.

COLOR KEY:	[0.0-0.73]	(0.73-0.88]	(0.88-0.95]	(0.95-1.01]	(1.01-1.09]	(1.09-1.26]	(1.26-1.55]	(1.55-2.09]	2.09+

	1	2	3	4	5	6	7	8	9	10
Slovenia	Headaches [0.88]	Falls [2.18]	Back Pain [1.08]	Road Inj [0.6]	Conduct [1.06]	Neonatal [1.09]	Anxiety [0.67]	Self Harm [0.83]	Congenital [1.12]	Depression [0.68]
**Eastern Europe**	Headaches [0.95]	Road Inj [0.76]	Neonatal [1.65]	Self Harm [1.52]	Conduct [1.08]	Falls [1.8]	Back Pain [0.79]	Depression [0.74]	Anxiety [0.6]	Congenital [1.0]
Belarus	Headaches [0.96]	Neonatal [1.66]	Conduct [1.01]	Falls [1.78]	Back Pain [0.82]	Road Inj [0.41]	Self Harm [0.84]	Depression [0.82]	Anxiety [0.62]	Upper Digest [4.68]
Estonia	Headaches [0.97]	Neonatal [1.72]	Conduct [1.07]	Falls [1.4]	Back Pain [0.73]	Depression [0.83]	Road Inj [0.41]	Self Harm [0.84]	Anxiety [0.57]	Upper Digest [5.47]
Latvia	Headaches [0.96]	Neonatal [1.57]	Road Inj [0.54]	Conduct [1.04]	Back Pain [0.81]	Falls [1.48]	Upper Digest [6.67]	Self Harm [0.81]	Anxiety [0.59]	Depression [0.68]
Lithuania	Headaches [1.05]	Neonatal [1.79]	Self Harm [1.37]	Road Inj [0.56]	Conduct [1.03]	Falls [1.48]	Back Pain [0.78]	Depression [0.88]	Anxiety [0.59]	Upper Digest [5.41]
Moldova	Headaches [0.98]	Neonatal [1.79]	Road Inj [0.57]	Conduct [0.98]	Upper Digest [6.02]	Falls [2.0]	Back Pain [0.89]	Depression [0.76]	Anxiety [0.69]	Self Harm [0.74]
Russian Federation	Headaches [0.94]	Road Inj [0.74]	Neonatal [1.65]	Self Harm [1.47]	Conduct [1.09]	Falls [1.79]	Back Pain [0.73]	Anxiety [0.6]	Depression [0.7]	Upper Digest [4.82]
Ukraine	Road Inj [0.92]	Self Harm [1.94]	Headaches [0.97]	Neonatal [1.65]	Conduct [1.06]	Back Pain [0.98]	Falls [1.99]	Congenital [1.34]	Drown [1.77]	Depression [0.85]
**High-income**	Headaches [0.94]	Anxiety [1.1]	Depression [1.38]	Back Pain [1.03]	Road Inj [0.55]	Drugs [2.69]	Conduct [1.03]	Neonatal [0.89]	Self Harm [0.81]	Acne [1.26]
**Australasia**	Depression [1.62]	Anxiety [1.26]	Headaches [0.88]	Back Pain [1.22]	Falls [1.71]	Road Inj [0.55]	Asthma [1.88]	Conduct [1.02]	Self Harm [1.04]	Neonatal [0.89]
Australia	Depression [1.65]	Anxiety [1.21]	Headaches [0.88]	Back Pain [1.17]	Falls [1.65]	Asthma [1.95]	Road Inj [0.53]	Conduct [1.0]	Self Harm [0.97]	Neonatal [0.87]
New Zealand	Anxiety [1.55]	Back Pain [1.51]	Headaches [0.89]	Depression [1.49]	Falls [2.03]	Road Inj [0.64]	Self Harm [1.36]	Conduct [1.09]	Neonatal [1.02]	Asthma [1.54]
**High-income Asia Pacific**	Back Pain [1.19]	Headaches [0.75]	Neonatal [1.12]	Anxiety [0.78]	Conduct [0.99]	Depression [0.94]	Acne [1.26]	Falls [1.05]	Self Harm [0.77]	Dermatitis [1.15]
Brunei	Road Inj [0.91]	Headaches [0.74]	Back Pain [0.96]	Neonatal [1.13]	Anxiety [0.8]	Conduct [0.93]	Depression [0.86]	Falls [1.11]	Drown [3.13]	Dermatitis [1.07]
Japan	Back Pain [1.27]	Headaches [0.7]	Neonatal [1.11]	Conduct [1.03]	Anxiety [0.77]	Depression [0.92]	Falls [1.14]	Acne [1.27]	Dermatitis [1.21]	Self Harm [0.73]
Aichi	Back Pain [1.26]	Neonatal [1.13]	Conduct [1.04]	Anxiety [0.76]	Headaches [0.56]	Depression [0.92]	Falls [1.12]	Acne [1.24]	Dermatitis [1.22]	Self Harm [0.71]
Akita	Back Pain [1.29]	Headaches [0.71]	Neonatal [1.22]	Conduct [1.01]	Depression [0.98]	Anxiety [0.79]	Self Harm [0.87]	Falls [1.2]	Dermatitis [1.22]	Acne [1.36]
Aomori	Back Pain [1.29]	Headaches [0.71]	Neonatal [1.14]	Conduct [1.0]	Anxiety [0.79]	Depression [0.95]	Falls [1.19]	Self Harm [0.77]	Dermatitis [1.22]	Acne [1.35]
Chiba	Back Pain [1.26]	Headaches [0.71]	Conduct [1.03]	Neonatal [1.1]	Anxiety [0.77]	Depression [0.92]	Falls [1.15]	Acne [1.28]	Dermatitis [1.22]	Self Harm [0.79]
Ehime	Back Pain [1.25]	Headaches [0.71]	Conduct [1.02]	Anxiety [0.78]	Depression [0.91]	Neonatal [0.93]	Falls [1.19]	Self Harm [0.79]	Road Inj [0.35]	Dermatitis [1.22]
Fukui	Back Pain [1.26]	Headaches [0.71]	Conduct [1.02]	Neonatal [1.1]	Anxiety [0.77]	Depression [0.89]	Falls [1.14]	Acne [1.31]	Dermatitis [1.22]	Road Inj [0.28]
Fukuoka	Back Pain [1.26]	Headaches [0.71]	Conduct [1.03]	Neonatal [1.09]	Anxiety [0.77]	Depression [0.91]	Falls [1.15]	Acne [1.3]	Dermatitis [1.21]	Iron [3.75]
Fukushima	Back Pain [1.28]	Headaches [0.7]	Conduct [1.01]	Neonatal [1.05]	Anxiety [0.79]	Depression [0.92]	Self Harm [0.88]	Falls [1.2]	Dermatitis [1.22]	Road Inj [0.34]
Gifu	Back Pain [1.29]	Headaches [0.71]	Neonatal [1.14]	Conduct [1.02]	Anxiety [0.78]	Depression [0.78]	Falls [1.17]	Road Inj [0.36]	Dermatitis [1.22]	Acne [1.3]
Gunma	Back Pain [1.28]	Headaches [0.71]	Conduct [1.02]	Neonatal [1.08]	Anxiety [0.78]	Depression [0.95]	Road Inj [0.39]	Falls [1.18]	Self Harm [0.85]	Acne [1.3]
Hiroshima	Back Pain [1.24]	Headaches [0.71]	Neonatal [1.15]	Conduct [1.04]	Anxiety [0.76]	Depression [0.88]	Falls [1.13]	Acne [1.26]	Dermatitis [1.22]	Road Inj [0.31]
Hokkaido	Back Pain [1.28]	Headaches [0.71]	Conduct [1.01]	Neonatal [1.08]	Anxiety [0.78]	Depression [0.93]	Falls [1.17]	Self Harm [0.78]	Dermatitis [1.21]	Acne [1.33]
Hyogo	Back Pain [1.26]	Headaches [0.72]	Neonatal [1.12]	Conduct [1.03]	Anxiety [0.77]	Depression [0.94]	Falls [1.14]	Acne [1.29]	Dermatitis [1.22]	Self Harm [0.75]
Ibaraki	Back Pain [1.27]	Headaches [0.62]	Neonatal [1.16]	Conduct [1.03]	Anxiety [0.77]	Depression [0.93]	Road Inj [0.45]	Falls [1.16]	Acne [1.31]	Dermatitis [1.21]
Ishikawa	Back Pain [1.26]	Headaches [0.72]	Conduct [1.02]	Neonatal [1.08]	Anxiety [0.77]	Depression [0.9]	Falls [1.16]	Acne [1.31]	Dermatitis [1.21]	Road Inj [0.31]

eFigure 7e. Leading ten causes of DALYs with the ratio of observed DALYs to DALYs expected on the basis of Socio-Demographic Index alone in 2017, 10-19 years, both sexes combined. The top ten causes contributing to DALYs are listed globally, by socio-demographic quintile, and then by GBD superregion, region, country, and subnationally where modeled. For each cell, the ratio of observed DALYs to DALYs expected on the basis of socio-demographic index (SDI) alone are listed. Abbreviations: DALY=disability-adjusted life year, GBD=Global Burden of Disease.

Values shown in brackets represent the ratio of observed DALYs to predicted DALYs on the basis of Socio-Demographic Index (SDI), rounded to two (2) digits. Color ranges (shown below) were calculated to place a roughly equal number of cells into each bin.

COLOR KEY:		[0.0-0.73]	(0.73-0.88]	(0.88-0.95]	(0.95-1.01]	(1.01-1.09]	(1.09-1.26]	(1.26-1.55]	(1.55-2.09]	2.09+
	1	2	3	4	5	6	7	8	9	10
Iwate	Back Pain [1.29]	Headaches [0.71]	Neonatal [1.17]	Conduct [1.01]	Anxiety [0.79]	Depression [0.96]	Falls [1.19]	Self Harm [0.81]	Dermatitis [1.22]	Acne [1.35]
Kagawa	Back Pain [1.24]	Headaches [0.71]	Conduct [1.02]	Neonatal [1.06]	Anxiety [0.78]	Road Inj [0.45]	Depression [0.88]	Falls [1.16]	Dermatitis [1.22]	Acne [1.3]
Kagoshima	Back Pain [1.25]	Headaches [0.7]	Conduct [1.02]	Anxiety [0.79]	Neonatal [0.97]	Depression [0.78]	Falls [1.2]	Dermatitis [1.23]	Acne [1.31]	Road Inj [0.33]
Kanagawa	Back Pain [1.26]	Headaches [0.72]	Neonatal [1.15]	Conduct [1.04]	Anxiety [0.76]	Depression [0.95]	Falls [1.13]	Acne [1.26]	Dermatitis [1.21]	Self Harm [0.73]
Kochi	Back Pain [1.27]	Headaches [0.71]	Conduct [1.01]	Neonatal [1.04]	Anxiety [0.79]	Depression [0.9]	Falls [1.21]	Iron [3.9]	Dermatitis [1.22]	Acne [1.35]
Kumamoto	Back Pain [1.25]	Headaches [0.7]	Conduct [1.02]	Anxiety [0.79]	Neonatal [1.02]	Depression [0.89]	Falls [1.19]	Dermatitis [1.22]	Acne [1.34]	Iron [3.14]
Kyoto	Back Pain [1.29]	Headaches [0.73]	Neonatal [1.15]	Conduct [1.03]	Anxiety [0.76]	Depression [0.94]	Falls [1.12]	Acne [1.27]	Congenital [0.9]	Road Inj [0.28]
Mie	Back Pain [1.25]	Headaches [0.71]	Neonatal [1.14]	Conduct [1.03]	Anxiety [0.77]	Depression [0.89]	Road Inj [0.38]	Falls [1.15]	Acne [1.29]	Dermatitis [1.22]
Miyagi	Back Pain [1.29]	Headaches [0.72]	Conduct [1.02]	Neonatal [1.06]	Anxiety [0.78]	Depression [0.97]	Falls [1.16]	Acne [1.34]	Dermatitis [1.21]	Self Harm [0.66]
Miyazaki	Back Pain [1.26]	Headaches [0.7]	Conduct [1.01]	Anxiety [0.79]	Neonatal [1.02]	Depression [0.91]	Iron [4.08]	Falls [1.22]	Dermatitis [1.22]	Self Harm [0.76]
Nagano	Back Pain [1.26]	Headaches [0.71]	Neonatal [1.17]	Conduct [1.03]	Anxiety [0.77]	Depression [0.9]	Self Harm [0.88]	Falls [1.17]	Acne [1.31]	Dermatitis [1.22]
Nagasaki	Back Pain [1.25]	Headaches [0.71]	Conduct [1.01]	Anxiety [0.79]	Neonatal [1.02]	Depression [0.89]	Falls [1.21]	Dermatitis [1.22]	Acne [1.35]	Iron [3.42]
Nara	Back Pain [1.31]	Headaches [0.72]	Conduct [1.02]	Anxiety [0.78]	Neonatal [1.0]	Depression [0.91]	Falls [1.18]	Acne [1.31]	Dermatitis [1.21]	Road Inj [0.31]
Niigata	Back Pain [1.43]	Headaches [0.71]	Neonatal [1.13]	Conduct [1.02]	Anxiety [0.78]	Depression [0.97]	Falls [1.17]	Self Harm [0.82]	Acne [1.33]	Dermatitis [1.22]
Oita	Back Pain [1.25]	Headaches [0.71]	Neonatal [1.17]	Conduct [1.02]	Anxiety [0.78]	Depression [0.91]	Falls [1.18]	Dermatitis [1.22]	Acne [1.32]	Self Harm [0.7]
Okayama	Back Pain [1.27]	Headaches [0.72]	Neonatal [1.18]	Conduct [1.03]	Anxiety [0.77]	Depression [0.9]	Falls [1.14]	Acne [1.3]	Dermatitis [1.21]	Road Inj [0.3]
Okinawa	Back Pain [1.25]	Headaches [0.7]	Conduct [1.01]	Neonatal [1.07]	Anxiety [0.79]	Depression [0.88]	Falls [1.22]	Dermatitis [1.22]	Acne [1.34]	Iron [3.46]
Osaka	Back Pain [1.28]	Headaches [0.72]	Conduct [1.03]	Anxiety [0.76]	Neonatal [1.05]	Depression [0.96]	Falls [1.14]	Acne [1.26]	Dermatitis [1.21]	Self Harm [0.71]
Saga	Back Pain [1.26]	Headaches [0.71]	Conduct [1.01]	Neonatal [1.06]	Anxiety [0.79]	Depression [0.88]	Falls [1.18]	Dermatitis [1.22]	Acne [1.33]	Road Inj [0.33]
Saitama	Back Pain [1.31]	Headaches [0.71]	Conduct [1.02]	Neonatal [1.09]	Anxiety [0.78]	Depression [0.95]	Falls [1.18]	Self Harm [0.83]	Acne [1.31]	Dermatitis [1.21]
Shiga	Back Pain [1.26]	Headaches [0.71]	Conduct [1.04]	Anxiety [0.76]	Neonatal [1.04]	Depression [0.89]	Dermatitis [1.5]	Falls [1.12]	Acne [1.26]	Road Inj [0.3]
Shimane	Back Pain [1.18]	Headaches [0.7]	Conduct [1.02]	Neonatal [1.05]	Anxiety [0.79]	Depression [0.92]	Self Harm [0.83]	Falls [1.2]	Dermatitis [1.21]	Acne [1.33]
Shizuoka	Back Pain [1.24]	Headaches [0.71]	Neonatal [1.22]	Conduct [1.04]	Anxiety [0.77]	Depression [0.9]	Falls [1.13]	Acne [1.28]	Dermatitis [1.22]	Road Inj [0.3]
Tochigi	Back Pain [1.27]	Headaches [0.71]	Neonatal [1.17]	Conduct [1.03]	Anxiety [0.77]	Depression [0.9]	Road Inj [0.41]	Falls [1.15]	Self Harm [0.84]	Dermatitis [1.22]
Tokushima	Back Pain [1.26]	Headaches [0.72]	Neonatal [1.11]	Conduct [1.01]	Anxiety [0.78]	Depression [0.91]	Falls [1.18]	Road Inj [0.37]	Dermatitis [1.22]	Acne [1.3]
Tokyo	Back Pain [1.24]	Headaches [0.73]	Neonatal [1.15]	Conduct [1.08]	Anxiety [0.73]	Depression [0.88]	Falls [1.04]	Acne [1.16]	Dermatitis [1.23]	Self Harm [0.81]
Tottori	Back Pain [1.26]	Headaches [0.66]	Neonatal [1.13]	Conduct [1.01]	Anxiety [0.79]	Depression [0.91]	Falls [1.19]	Road Inj [0.35]	Dermatitis [1.22]	Acne [1.33]
Toyama	Back Pain [1.25]	Headaches [0.71]	Conduct [1.04]	Neonatal [1.07]	Anxiety [0.77]	Depression [0.91]	Self Harm [0.9]	Falls [1.15]	Road Inj [0.37]	Acne [1.28]
Wakayama	Back Pain [1.44]	Headaches [0.71]	Neonatal [1.17]	Conduct [1.02]	Anxiety [0.78]	Depression [0.92]	Falls [1.17]	Dermatitis [1.22]	Acne [1.31]	Road Inj [0.28]
Yamagata	Back Pain [1.28]	Headaches [0.71]	Conduct [1.01]	Anxiety [0.79]	Neonatal [0.96]	Depression [0.88]	Falls [1.21]	Self Harm [0.78]	Dermatitis [1.22]	Acne [1.35]
Yamaguchi	Back Pain [1.25]	Headaches [0.71]	Conduct [1.02]	Anxiety [0.78]	Neonatal [0.98]	Depression [0.91]	Falls [1.15]	Acne [1.31]	Dermatitis [1.22]	Road Inj [0.33]

eFigure 7e. Leading ten causes of DALYs with the ratio of observed DALYs to DALYs expected on the basis of Socio-Demographic Index alone in 2017, 10-19 years, both sexes combined. The top ten causes contributing to DALYs are listed globally, by socio-demographic quintile, and then by GBD superregion, region, country, and subnationally where modeled. For each cell, the ratio of observed DALYs to DALYs expected on the basis of socio-demographic index (SDI) alone are listed. Abbreviations: DALY=disability-adjusted life year, GBD=Global Burden of Disease.

Values shown in brackets represent the ratio of observed DALYs to predicted DALYs on the basis of Socio-Demographic Index (SDI), rounded to two (2) digits. Color ranges (shown below) were calculated to place a roughly equal number of cells into each bin.

COLOR KEY: [0.0-0.73] | (0.73-0.88] | (0.88-0.95] | (0.95-1.01] | (1.01-1.09] | (1.09-1.26] | (1.26-1.55] | (1.55-2.09] | 2.09+

	1	2	3	4	5	6	7	8	9	10
Yamanashi	Back Pain [1.28]	Headaches [0.71]	Neonatal [1.23]	Conduct [1.03]	Anxiety [0.77]	Depression [0.91]	Falls [1.15]	Acne [1.29]	Dermatitis [1.2]	Iron [3.84]
S Korea	Headaches [0.86]	Back Pain [1.06]	Neonatal [1.14]	Anxiety [0.83]	Depression [0.97]	Conduct [0.89]	Self Harm [0.88]	Road Inj [0.38]	Acne [1.29]	Falls [0.89]
Singapore	Headaches [0.72]	Depression [1.24]	Back Pain [0.91]	Neonatal [1.14]	Anxiety [0.82]	Conduct [0.89]	Acne [1.23]	Falls [0.99]	Dermatitis [0.95]	Road Inj [0.27]
**High-income North America**	Drugs [5.3]	Depression [1.83]	Headaches [1.02]	Road Inj [0.77]	Anxiety [1.02]	Conduct [1.2]	Back Pain [0.83]	Self Harm [1.09]	Violence [3.77]	Neonatal [0.86]
Canada	Headaches [1.04]	Drugs [4.22]	Depression [1.36]	Road Inj [0.67]	Back Pain [0.93]	Anxiety [0.83]	Self Harm [1.23]	Conduct [1.02]	Acne [1.25]	Dermatitis [1.22]
Greenland	Self Harm [6.67]	Depression [2.51]	Headaches [1.01]	Drugs [3.74]	Anxiety [1.04]	Violence [2.62]	Conduct [0.98]	Back Pain [0.79]	Road Inj [0.35]	Dermatitis [1.17]
USA	Drugs [5.43]	Depression [1.87]	Headaches [1.01]	Road Inj [0.78]	Anxiety [1.05]	Conduct [1.21]	Back Pain [0.82]	Self Harm [1.07]	Violence [3.88]	Neonatal [0.88]
Alabama	Drugs [6.81]	Road Inj [1.29]	Depression [1.84]	Headaches [1.02]	Anxiety [1.08]	Back Pain [1.05]	Violence [4.72]	Conduct [1.17]	Self Harm [1.09]	Neonatal [0.87]
Alaska	Drugs [6.02]	Depression [1.87]	Self Harm [2.07]	Headaches [1.01]	Anxiety [1.05]	Road Inj [0.62]	Conduct [1.21]	Back Pain [0.93]	Neonatal [0.84]	Oth MSK [5.87]
Arizona	Drugs [7.0]	Depression [2.13]	Headaches [1.01]	Road Inj [0.73]	Anxiety [1.07]	Conduct [1.18]	Self Harm [1.33]	Back Pain [0.9]	Neonatal [0.88]	Violence [3.01]
Arkansas	Road Inj [1.27]	Drugs [6.4]	Depression [2.15]	Headaches [1.02]	Anxiety [1.09]	Self Harm [1.36]	Conduct [1.17]	Violence [3.6]	Back Pain [0.82]	Neonatal [0.77]
California	Depression [1.83]	Headaches [1.02]	Drugs [3.92]	Anxiety [1.02]	Conduct [1.21]	Road Inj [0.58]	Violence [4.41]	Back Pain [0.7]	Dermatitis [1.31]	Acne [1.3]
Colorado	Depression [2.07]	Drugs [4.71]	Headaches [1.02]	Self Harm [1.74]	Road Inj [0.71]	Anxiety [1.04]	Conduct [1.22]	Back Pain [0.83]	Neonatal [0.89]	Oth MSK [5.77]
Connecticut	Drugs [6.31]	Headaches [1.04]	Depression [1.67]	Anxiety [1.02]	Conduct [1.23]	Road Inj [0.6]	Neonatal [1.03]	Back Pain [0.7]	Dermatitis [1.37]	Acne [1.27]
Delaware	Drugs [7.23]	Depression [1.97]	Headaches [1.03]	Road Inj [0.84]	Anxiety [1.05]	Conduct [1.2]	Back Pain [0.72]	Self Harm [0.95]	Neonatal [0.85]	Violence [3.57]
DC	Violence [17.57]	Drugs [5.4]	Headaches [1.06]	Depression [1.63]	Anxiety [1.06]	Conduct [1.2]	Road Inj [0.54]	Dermatitis [1.53]	Acne [1.45]	Neonatal [0.89]
Florida	Drugs [5.68]	Depression [1.83]	Headaches [1.03]	Road Inj [0.84]	Anxiety [1.06]	Conduct [1.2]	Violence [4.48]	Neonatal [0.98]	Back Pain [0.73]	Self Harm [0.99]
Georgia	Drugs [5.12]	Headaches [1.02]	Road Inj [0.89]	Depression [1.77]	Anxiety [1.07]	Conduct [1.19]	Violence [4.4]	Back Pain [0.8]	Self Harm [0.96]	Neonatal [0.81]
Hawaii	Depression [1.85]	Headaches [1.02]	Anxiety [1.04]	Road Inj [0.62]	Conduct [1.21]	Drugs [2.76]	Self Harm [1.04]	Dermatitis [1.41]	Neonatal [0.81]	Acne [1.32]
Idaho	Drugs [5.27]	Depression [2.07]	Road Inj [0.89]	Headaches [1.01]	Self Harm [1.57]	Anxiety [1.07]	Conduct [1.19]	Back Pain [0.84]	Oth MSK [6.21]	Neonatal [0.73]
Illinois	Drugs [4.7]	Headaches [1.03]	Depression [1.7]	Violence [6.91]	Anxiety [1.04]	Road Inj [0.63]	Conduct [1.21]	Back Pain [0.9]	Neonatal [1.03]	Oth MSK [5.89]
Indiana	Drugs [6.68]	Depression [1.98]	Road Inj [0.93]	Headaches [1.02]	Anxiety [1.07]	Conduct [1.18]	Back Pain [0.96]	Self Harm [1.28]	Violence [4.02]	Neonatal [0.84]
Iowa	Headaches [1.02]	Depression [1.78]	Back Pain [1.43]	Road Inj [0.83]	Anxiety [1.05]	Conduct [1.2]	Drugs [2.8]	Self Harm [1.25]	Oth MSK [7.04]	Neonatal [0.85]
Kansas	Depression [1.89]	Road Inj [0.95]	Headaches [1.02]	Drugs [3.55]	Back Pain [1.13]	Anxiety [1.05]	Conduct [1.2]	Self Harm [1.24]	Neonatal [0.9]	Oth MSK [5.85]
Kentucky	Drugs [9.86]	Road Inj [1.06]	Depression [1.99]	Headaches [1.01]	Anxiety [1.08]	Back Pain [1.09]	Conduct [1.17]	Self Harm [1.2]	Neonatal [0.86]	Oth MSK [6.3]
Louisiana	Drugs [7.14]	Road Inj [1.06]	Violence [7.22]	Headaches [1.02]	Depression [1.78]	Anxiety [1.08]	Conduct [1.18]	Self Harm [1.14]	Back Pain [0.87]	Neonatal [0.79]
Maine	Drugs [7.24]	Depression [1.95]	Road Inj [0.96]	Headaches [1.02]	Anxiety [1.05]	Conduct [1.2]	Self Harm [1.37]	Back Pain [0.89]	Oth MSK [7.51]	Neonatal [0.88]
Maryland	Depression [1.78]	Headaches [1.03]	Drugs [3.92]	Anxiety [1.03]	Violence [6.92]	Road Inj [0.66]	Conduct [1.23]	Self Harm [1.15]	Neonatal [1.02]	Back Pain [0.66]
Massachusetts	Drugs [6.31]	Depression [1.81]	Headaches [1.05]	Anxiety [1.02]	Conduct [1.24]	Road Inj [0.51]	Neonatal [0.98]	Back Pain [0.68]	Oth MSK [5.46]	Self Harm [0.94]
Michigan	Drugs [6.29]	Depression [1.93]	Road Inj [0.76]	Headaches [0.83]	Anxiety [1.05]	Conduct [1.2]	Self Harm [1.25]	Violence [4.6]	Back Pain [0.79]	Neonatal [0.87]
Minnesota	Depression [1.83]	Headaches [1.03]	Anxiety [1.03]	Conduct [1.23]	Road Inj [0.61]	Drugs [2.74]	Self Harm [1.31]	Back Pain [0.72]	Neonatal [0.92]	Oth MSK [5.58]

eFigure 7e. Leading ten causes of DALYs with the ratio of observed DALYs to DALYs expected on the basis of Socio-Demographic Index alone in 2017, 10-19 years, both sexes combined. The top ten causes contributing to DALYs are listed globally, by socio-demographic quintile, and then by GBD superregion, region, country, and subnationally where modeled. For each cell, the ratio of observed DALYs to DALYs expected on the basis of socio-demographic index (SDI) alone are listed. Abbreviations: DALY=disability-adjusted life year, GBD=Global Burden of Disease.

Values shown in brackets represent the ratio of observed DALYs to predicted DALYs on the basis of Socio-Demographic Index (SDI), rounded to two (2) digits. Color ranges (shown below) were calculated to place a roughly equal number of cells into each bin.

COLOR KEY:	[0.0-0.73]	(0.73-0.88]	(0.88-0.95]	(0.95-1.01]	(1.01-1.09]	(1.09-1.26]	(1.26-1.55]	(1.55-2.09]	2.09+

	1	2	3	4	5	6	7	8	9	10
Mississippi	Road Inj [1.52]	Drugs [6.35]	Headaches [1.02]	Depression [1.8]	Anxiety [1.1]	Violence [4.25]	Conduct [1.17]	Self Harm [1.0]	Back Pain [0.74]	Neonatal [0.84]
Missouri	Drugs [5.89]	Road Inj [1.01]	Depression [2.01]	Headaches [1.02]	Anxiety [1.06]	Conduct [1.19]	Violence [5.01]	Self Harm [1.23]	Back Pain [0.9]	Neonatal [0.9]
Montana	Road Inj [1.44]	Drugs [5.4]	Depression [1.97]	Self Harm [2.08]	Headaches [1.01]	Anxiety [1.05]	Conduct [1.21]	Back Pain [0.94]	Oth MSK [6.81]	Neonatal [0.85]
Nebraska	Road Inj [1.0]	Headaches [1.02]	Depression [1.76]	Back Pain [1.22]	Anxiety [1.04]	Conduct [1.22]	Drugs [2.8]	Self Harm [1.2]	Neonatal [0.85]	Oth MSK [5.51]
Nevada	Drugs [7.25]	Depression [1.96]	Headaches [1.01]	Road Inj [0.77]	Anxiety [1.07]	Conduct [1.19]	Violence [4.52]	Self Harm [1.24]	Back Pain [0.71]	Neonatal [0.83]
New Hampshire	Drugs [7.22]	Depression [2.03]	Headaches [1.04]	Anxiety [1.02]	Road Inj [0.67]	Conduct [1.23]	Back Pain [0.83]	Self Harm [1.04]	Oth MSK [5.97]	Neonatal [0.83]
New Jersey	Drugs [5.15]	Headaches [1.03]	Depression [1.57]	Anxiety [1.02]	Conduct [1.23]	Back Pain [0.77]	Road Inj [0.49]	Neonatal [0.93]	Dermatitis [1.43]	Acne [1.3]
New Mexico	Drugs [6.55]	Depression [2.14]	Road Inj [0.96]	Headaches [1.01]	Self Harm [1.67]	Anxiety [1.08]	Conduct [1.18]	Back Pain [0.96]	Violence [3.38]	Neonatal [0.93]
New York	Drugs [4.59]	Depression [1.94]	Headaches [1.03]	Anxiety [1.03]	Conduct [1.22]	Road Inj [0.48]	Neonatal [0.97]	Acne [1.48]	Dermatitis [1.5]	Back Pain [0.69]
N Carolina	Drugs [5.57]	Depression [1.83]	Road Inj [0.9]	Headaches [1.02]	Anxiety [1.0]	Conduct [1.11]	Self Harm [0.99]	Back Pain [0.72]	Neonatal [0.89]	Violence [3.4]
N Dakota	Road Inj [1.29]	Headaches [1.02]	Depression [1.7]	Self Harm [1.68]	Anxiety [1.04]	Back Pain [1.02]	Conduct [1.22]	Oth MSK [8.75]	Drugs [2.71]	Neonatal [0.92]
Ohio	Drugs [7.85]	Depression [1.9]	Headaches [1.02]	Road Inj [0.76]	Anxiety [1.06]	Conduct [1.19]	Back Pain [0.87]	Self Harm [1.15]	Violence [3.82]	Neonatal [0.83]
Oklahoma	Drugs [7.85]	Road Inj [1.16]	Depression [1.96]	Headaches [1.01]	Self Harm [1.51]	Anxiety [1.07]	Conduct [1.18]	Back Pain [0.98]	Violence [3.62]	Oth MSK [6.38]
Oregon	Drugs [6.55]	Depression [2.05]	Headaches [1.02]	Road Inj [0.63]	Anxiety [0.97]	Self Harm [1.36]	Conduct [1.1]	Back Pain [0.76]	Oth MSK [6.04]	Dermatitis [1.22]
Pennsylvania	Drugs [7.72]	Depression [1.84]	Headaches [0.93]	Road Inj [0.73]	Anxiety [1.04]	Back Pain [1.04]	Conduct [1.21]	Self Harm [1.03]	Violence [4.25]	Oth MSK [6.4]
Rhode Island	Drugs [7.93]	Depression [2.02]	Headaches [1.04]	Anxiety [1.04]	Conduct [1.21]	Road Inj [0.53]	Back Pain [0.68]	Neonatal [0.84]	Self Harm [0.85]	Oth MSK [5.21]
S Carolina	Drugs [6.56]	Road Inj [1.2]	Depression [1.91]	Headaches [1.02]	Anxiety [1.07]	Conduct [1.18]	Back Pain [0.86]	Violence [4.1]	Self Harm [1.05]	Neonatal [0.83]
S Dakota	Road Inj [1.38]	Headaches [1.01]	Self Harm [1.94]	Depression [1.65]	Back Pain [1.23]	Anxiety [1.05]	Conduct [1.21]	Drugs [2.77]	Oth MSK [7.55]	Neonatal [0.75]
Tennessee	Drugs [8.07]	Road Inj [0.96]	Depression [1.95]	Headaches [1.02]	Anxiety [1.08]	Conduct [1.17]	Violence [4.17]	Back Pain [0.85]	Self Harm [1.07]	Neonatal [0.83]
Texas	Depression [1.81]	Headaches [1.01]	Road Inj [0.83]	Drugs [3.73]	Anxiety [1.07]	Conduct [1.33]	Self Harm [1.04]	Back Pain [0.8]	Neonatal [0.96]	Violence [2.98]
Utah	Drugs [6.1]	Depression [2.24]	Headaches [1.01]	Self Harm [1.89]	Anxiety [1.06]	Road Inj [0.65]	Conduct [1.19]	Back Pain [0.83]	Neonatal [0.81]	Oth MSK [5.26]
Vermont	Drugs [5.45]	Depression [1.89]	Headaches [1.04]	Road Inj [0.79]	Anxiety [1.04]	Conduct [1.23]	Self Harm [1.13]	Back Pain [0.77]	Neonatal [0.93]	Oth MSK [5.43]
Virginia	Depression [1.98]	Drugs [4.39]	Headaches [1.03]	Road Inj [0.71]	Anxiety [1.04]	Conduct [1.22]	Self Harm [1.11]	Back Pain [0.75]	Neonatal [0.95]	Dermatitis [1.33]
Washington	Drugs [5.36]	Depression [2.03]	Headaches [1.02]	Anxiety [1.03]	Conduct [1.22]	Self Harm [1.29]	Road Inj [0.55]	Back Pain [0.83]	Oth MSK [6.64]	Neonatal [0.81]
W Virginia	Drugs [12.62]	Road Inj [1.15]	Depression [2.0]	Headaches [1.01]	Anxiety [1.09]	Conduct [1.16]	Self Harm [1.22]	Back Pain [0.92]	Neonatal [0.83]	Oth MSK [5.37]
Wisconsin	Drugs [5.45]	Depression [1.97]	Headaches [1.02]	Road Inj [0.71]	Anxiety [1.04]	Conduct [1.21]	Back Pain [0.94]	Self Harm [1.26]	Neonatal [0.93]	Oth MSK [5.61]
Wyoming	Road Inj [1.18]	Depression [2.08]	Headaches [1.01]	Self Harm [2.0]	Drugs [4.06]	Back Pain [1.15]	Anxiety [1.04]	Conduct [1.22]	Oth MSK [6.28]	Neonatal [0.71]
**Southern Latin America**	Anxiety [1.36]	Road Inj [0.67]	Back Pain [1.36]	Depression [1.38]	Self Harm [1.33]	Headaches [0.66]	Conduct [0.93]	Neonatal [0.99]	Violence [1.67]	Dermatitis [1.02]
Argentina	Road Inj [0.74]	Anxiety [1.38]	Self Harm [1.46]	Back Pain [1.23]	Depression [1.35]	Headaches [0.63]	Conduct [0.93]	Violence [1.73]	Neonatal [0.97]	Drugs [1.62]
Chile	Back Pain [1.8]	Anxiety [1.32]	Depression [1.51]	Headaches [0.71]	Road Inj [0.47]	Conduct [0.92]	Neonatal [1.01]	Self Harm [0.94]	Dermatitis [1.22]	Violence [1.43]
Uruguay	Road Inj [0.73]	Anxiety [1.39]	Self Harm [1.26]	Headaches [0.67]	Back Pain [1.08]	Depression [1.12]	Conduct [0.93]	Neonatal [1.03]	Violence [1.53]	Drown [0.89]

eFigure 7e. Leading ten causes of DALYs with the ratio of observed DALYs to DALYs expected on the basis of Socio-Demographic Index alone in 2017, 10-19 years, both sexes combined. The top ten causes contributing to DALYs are listed globally, by socio-demographic quintile, and then by GBD superregion, region, country, and subnationally where modeled. For each cell, the ratio of observed DALYs to DALYs expected on the basis of socio-demographic index (SDI) alone are listed. Abbreviations: DALY=disability-adjusted life year, GBD=Global Burden of Disease.

Values shown in brackets represent the ratio of observed DALYs to predicted DALYs on the basis of Socio-Demographic Index (SDI), rounded to two (2) digits. Color ranges (shown below) were calculated to place a roughly equal number of cells into each bin.

COLOR KEY:	[0.0-0.73]	(0.73-0.88]	(0.88-0.95]	(0.95-1.01]	(1.01-1.09]	(1.09-1.26]	(1.26-1.55]	(1.55-2.09]	2.09+

	1	2	3	4	5	6	7	8	9	10
**Western Europe**	Headaches [1.0]	Anxiety [1.24]	Back Pain [1.1]	Depression [1.09]	Conduct [0.91]	Road Inj [0.38]	Neonatal [0.81]	Acne [1.34]	Dermatitis [1.13]	Asthma [1.08]
Andorra	Headaches [1.02]	Anxiety [1.14]	Back Pain [1.06]	Depression [1.05]	Conduct [0.92]	Dermatitis [1.4]	Acne [1.27]	Neonatal [0.84]	Road Inj [0.39]	Asthma [1.2]
Austria	Headaches [1.04]	Anxiety [1.17]	Neonatal [1.36]	Back Pain [0.86]	Road Inj [0.46]	Conduct [0.89]	Depression [0.77]	Acne [1.35]	Self Harm [0.74]	Falls [0.91]
Belgium	Headaches [1.25]	Anxiety [1.13]	Back Pain [0.94]	Depression [1.05]	Conduct [0.91]	Road Inj [0.41]	Acne [1.14]	Falls [0.93]	Dermatitis [0.95]	Self Harm [0.63]
Cyprus	Headaches [1.02]	Road Inj [0.83]	Anxiety [1.18]	Back Pain [1.13]	Neonatal [1.09]	Depression [1.0]	Conduct [0.89]	Acne [1.29]	Falls [0.86]	Asthma [0.94]
Denmark	Back Pain [1.38]	Anxiety [1.13]	Headaches [0.85]	Conduct [0.91]	Dermatitis [1.45]	Depression [0.79]	Acne [1.2]	Road Inj [0.35]	Neonatal [0.66]	Asthma [1.03]
Finland	Headaches [1.01]	Neonatal [1.7]	Depression [1.37]	Anxiety [0.83]	Conduct [0.9]	Dermatitis [1.48]	Road Inj [0.44]	Back Pain [0.62]	Acne [1.27]	Self Harm [0.9]
France	Anxiety [1.41]	Headaches [0.83]	Back Pain [1.08]	Depression [1.12]	Conduct [0.9]	Road Inj [0.42]	Acne [1.48]	Dermatitis [1.31]	Neonatal [0.64]	Asthma [1.05]
Germany	Anxiety [1.4]	Back Pain [1.38]	Headaches [0.87]	Depression [1.04]	Conduct [0.9]	Road Inj [0.44]	Neonatal [0.92]	Acne [1.16]	Falls [0.84]	Dermatitis [0.91]
Greece	Headaches [1.19]	Anxiety [1.33]	Depression [1.35]	Road Inj [0.62]	Back Pain [0.95]	Conduct [0.87]	Neonatal [0.86]	Acne [1.49]	Congenital [0.89]	Falls [0.89]
Iceland	Headaches [1.02]	Anxiety [1.14]	Back Pain [1.01]	Dermatitis [1.55]	Conduct [0.92]	Depression [0.88]	Asthma [1.6]	Acne [1.3]	Neonatal [0.85]	Road Inj [0.4]
Ireland	Headaches [1.01]	Anxiety [1.27]	Depression [1.21]	Back Pain [0.81]	Conduct [0.9]	Asthma [1.42]	Acne [1.27]	Dermatitis [1.22]	Neonatal [0.72]	Road Inj [0.31]
Israel	Headaches [0.99]	Back Pain [1.17]	Depression [1.14]	Anxiety [0.74]	Neonatal [0.95]	Conduct [0.87]	Road Inj [0.38]	Acne [1.29]	Blindness [3.01]	Dermatitis [1.03]
Italy	Headaches [1.24]	Anxiety [1.26]	Back Pain [1.01]	Depression [1.08]	Conduct [0.89]	Road Inj [0.41]	Acne [1.39]	Neonatal [0.64]	Dermatitis [0.94]	Congenital [0.8]
Luxembourg	Headaches [1.17]	Anxiety [1.13]	Back Pain [0.99]	Neonatal [1.1]	Road Inj [0.52]	Depression [0.96]	Conduct [0.93]	Dermatitis [1.41]	Acne [1.27]	Asthma [1.28]
Malta	Headaches [1.02]	Anxiety [1.21]	Back Pain [1.12]	Depression [1.02]	Conduct [0.87]	Acne [1.37]	Congenital [1.07]	Falls [1.06]	Road Inj [0.33]	Neonatal [0.71]
Netherlands	Anxiety [1.39]	Headaches [1.06]	Depression [1.08]	Back Pain [0.8]	Conduct [1.01]	Acne [1.27]	Neonatal [0.85]	Dermatitis [1.25]	Asthma [1.16]	Road Inj [0.32]
Norway	Anxiety [1.58]	Headaches [1.07]	Back Pain [0.95]	Depression [1.02]	Conduct [1.0]	Neonatal [1.0]	Dermatitis [1.44]	Acne [1.29]	Asthma [1.46]	Falls [1.02]
Portugal	Headaches [1.0]	Anxiety [1.27]	Depression [1.28]	Back Pain [1.11]	Conduct [0.85]	Asthma [1.53]	Road Inj [0.36]	Acne [1.49]	Neonatal [0.6]	Dermatitis [0.86]
Spain	Headaches [1.18]	Anxiety [1.15]	Back Pain [1.19]	Depression [1.03]	Conduct [0.88]	Acne [1.36]	Neonatal [0.7]	Road Inj [0.3]	Falls [0.85]	Drugs [1.3]
Sweden	Headaches [1.06]	Anxiety [1.14]	Depression [1.22]	Back Pain [0.92]	Dermatitis [1.85]	Conduct [1.01]	Acne [1.51]	Asthma [1.6]	Falls [1.01]	Neonatal [0.75]
Stockholm	Headaches [1.06]	Anxiety [1.12]	Conduct [1.04]	Depression [0.99]	Acne [1.43]	Dermatitis [1.46]	Back Pain [0.61]	Asthma [1.38]	Falls [0.96]	Neonatal [0.73]
Sweden w/o Stockholm	Headaches [1.06]	Anxiety [1.15]	Depression [1.28]	Back Pain [1.01]	Dermatitis [1.96]	Conduct [1.0]	Asthma [1.66]	Acne [1.53]	Neonatal [0.76]	Falls [1.03]
Switzerland	Back Pain [2.12]	Anxiety [1.15]	Headaches [0.86]	Depression [0.95]	Conduct [0.91]	Dermatitis [1.4]	Neonatal [0.83]	Acne [1.28]	Asthma [1.07]	Road Inj [0.3]
UK	Headaches [0.93]	Anxiety [1.04]	Depression [1.11]	Back Pain [0.92]	Conduct [0.98]	Asthma [1.73]	Neonatal [0.98]	Dermatitis [1.56]	Drugs [2.01]	Acne [1.33]
England	Headaches [0.92]	Anxiety [1.0]	Back Pain [0.92]	Depression [1.1]	Conduct [1.0]	Asthma [1.75]	Neonatal [1.01]	Dermatitis [1.59]	Drugs [2.01]	Acne [1.32]
E Midlands	Headaches [0.93]	Anxiety [1.02]	Depression [1.1]	Back Pain [0.93]	Conduct [0.99]	Asthma [1.67]	Neonatal [0.96]	Dermatitis [1.55]	Drugs [2.02]	Road Inj [0.35]
Derby	Headaches [0.92]	Anxiety [1.01]	Back Pain [0.94]	Depression [1.09]	Conduct [1.0]	Asthma [1.67]	Neonatal [0.96]	Dermatitis [1.55]	Drugs [2.12]	Acne [1.3]
Derbyshire	Headaches [0.92]	Anxiety [1.04]	Depression [1.1]	Conduct [0.98]	Back Pain [0.83]	Asthma [1.66]	Dermatitis [1.56]	Neonatal [0.89]	Drugs [2.08]	Road Inj [0.35]
Leicester	Headaches [0.93]	Anxiety [1.01]	Back Pain [0.95]	Depression [1.11]	Conduct [0.99]	Asthma [1.7]	Dermatitis [1.55]	Drugs [2.1]	Neonatal [0.82]	Acne [1.32]
Leicestershire	Headaches [0.93]	Anxiety [1.01]	Back Pain [0.95]	Depression [1.1]	Conduct [1.0]	Dermatitis [1.55]	Asthma [1.64]	Drugs [1.93]	Neonatal [0.8]	Acne [1.39]

eFigure 7e. Leading ten causes of DALYs with the ratio of observed DALYs to DALYs expected on the basis of Socio-Demographic Index alone in 2017, 10-19 years, both sexes combined. The top ten causes contributing to DALYs are listed globally, by socio-demographic quintile, and then by GBD superregion, region, country, and subnationally where modeled. For each cell, the ratio of observed DALYs to DALYs expected on the basis of socio-demographic index (SDI) alone are listed. Abbreviations: DALY=disability-adjusted life year, GBD=Global Burden of Disease.

Values shown in brackets represent the ratio of observed DALYs to predicted DALYs on the basis of Socio-Demographic Index (SDI), rounded to two (2) digits. Color ranges (shown below) were calculated to place a roughly equal number of cells into each bin.

COLOR KEY:	[0.0-0.73]	(0.73-0.88]	(0.88-0.95]	(0.95-1.01]	(1.01-1.09]	(1.09-1.26]	(1.26-1.55]	(1.55-2.09]	2.09+

	1	2	3	4	5	6	7	8	9	10
Lincolnshire	Headaches [0.93]	Anxiety [1.05]	Back Pain [0.96]	Depression [1.11]	Conduct [0.97]	Road Inj [0.46]	Asthma [1.68]	Neonatal [0.96]	Dermatitis [1.55]	Drugs [2.1]
Northamptonshire	Headaches [0.91]	Anxiety [1.02]	Back Pain [0.94]	Depression [1.08]	Conduct [0.99]	Neonatal [1.06]	Asthma [1.67]	Dermatitis [1.57]	Drugs [1.99]	Road Inj [0.36]
Nottingham	Headaches [0.96]	Anxiety [1.0]	Back Pain [0.97]	Depression [1.13]	Neonatal [1.16]	Conduct [1.0]	Asthma [1.74]	Dermatitis [1.54]	Drugs [2.04]	Acne [1.3]
Nottinghamshire	Headaches [0.91]	Anxiety [1.03]	Back Pain [0.95]	Depression [1.09]	Conduct [0.98]	Neonatal [1.07]	Asthma [1.67]	Dermatitis [1.55]	Drugs [1.93]	Acne [1.39]
Rutland	Headaches [0.93]	Anxiety [1.01]	Back Pain [0.96]	Depression [1.12]	Conduct [0.99]	Dermatitis [1.53]	Asthma [1.61]	Drugs [1.95]	Road Inj [0.37]	Acne [1.42]
E England	Headaches [0.92]	Anxiety [1.01]	Depression [1.09]	Back Pain [0.92]	Conduct [1.0]	Asthma [1.65]	Dermatitis [1.55]	Neonatal [0.92]	Drugs [1.94]	Acne [1.37]
Bedford	Headaches [0.92]	Anxiety [1.01]	Back Pain [0.94]	Depression [1.09]	Neonatal [1.13]	Conduct [0.99]	Dermatitis [1.55]	Asthma [1.64]	Drugs [2.09]	Acne [1.35]
Cambridgeshire	Headaches [0.93]	Anxiety [0.99]	Depression [1.1]	Back Pain [0.86]	Conduct [1.02]	Dermatitis [1.55]	Asthma [1.65]	Neonatal [0.92]	Drugs [1.88]	Acne [1.35]
Cen Bedfordshire	Headaches [0.91]	Anxiety [1.01]	Back Pain [0.92]	Depression [1.08]	Conduct [1.0]	Neonatal [1.0]	Dermatitis [1.56]	Asthma [1.64]	Drugs [1.87]	Acne [1.37]
Essex	Headaches [0.92]	Anxiety [1.02]	Back Pain [0.93]	Depression [1.09]	Conduct [0.99]	Dermatitis [1.55]	Asthma [1.64]	Neonatal [0.91]	Drugs [1.96]	Acne [1.4]
Hertfordshire	Headaches [0.92]	Anxiety [0.99]	Back Pain [0.91]	Depression [1.08]	Conduct [1.02]	Dermatitis [1.56]	Asthma [1.63]	Neonatal [0.9]	Acne [1.3]	Drugs [1.75]
Luton	Headaches [0.91]	Anxiety [1.01]	Back Pain [0.92]	Depression [1.07]	Conduct [1.0]	Neonatal [1.04]	Asthma [1.71]	Dermatitis [1.57]	Drugs [1.84]	Acne [1.28]
Norfolk	Headaches [0.93]	Anxiety [1.03]	Back Pain [0.95]	Depression [1.1]	Conduct [0.98]	Asthma [1.65]	Dermatitis [1.55]	Drugs [2.19]	Neonatal [0.78]	Acne [1.43]
Peterborough	Headaches [0.91]	Anxiety [1.03]	Back Pain [0.93]	Depression [1.07]	Conduct [0.99]	Asthma [1.75]	Dermatitis [1.56]	Drugs [2.17]	Road Inj [0.37]	Neonatal [0.73]
Southend-on-Sea	Headaches [0.91]	Anxiety [1.04]	Depression [1.09]	Back Pain [0.92]	Neonatal [1.11]	Conduct [0.98]	Asthma [1.65]	Dermatitis [1.56]	Drugs [2.29]	Acne [1.4]
Suffolk	Headaches [0.91]	Anxiety [1.03]	Depression [1.09]	Back Pain [0.93]	Conduct [0.98]	Neonatal [0.97]	Dermatitis [1.55]	Asthma [1.63]	Drugs [1.99]	Acne [1.42]
Thurrock	Headaches [0.91]	Anxiety [1.04]	Depression [1.08]	Back Pain [0.91]	Conduct [0.98]	Neonatal [1.03]	Asthma [1.73]	Dermatitis [1.57]	Drugs [1.83]	Acne [1.39]
Greater London	Headaches [0.93]	Anxiety [0.97]	Back Pain [0.91]	Depression [1.08]	Asthma [1.96]	Conduct [1.04]	Neonatal [1.04]	Dermatitis [1.58]	Drugs [1.84]	Acne [1.2]
Barking & Dagenham	Headaches [0.9]	Anxiety [1.04]	Dermatitis [2.06]	Back Pain [0.94]	Depression [1.07]	Asthma [1.93]	Conduct [0.98]	Neonatal [1.02]	Drugs [2.01]	Acne [1.34]
Barnet	Headaches [0.91]	Anxiety [0.99]	Back Pain [0.91]	Depression [1.07]	Asthma [1.9]	Conduct [1.02]	Neonatal [0.98]	Dermatitis [1.56]	Drugs [1.85]	Acne [1.3]
Bexley	Headaches [0.92]	Anxiety [1.03]	Back Pain [0.95]	Depression [1.1]	Asthma [1.93]	Conduct [0.98]	Dermatitis [1.56]	Neonatal [0.95]	Drugs [1.85]	Acne [1.41]
Brent	Headaches [0.91]	Anxiety [1.0]	Back Pain [0.92]	Depression [1.07]	Asthma [1.94]	Conduct [1.01]	Neonatal [1.05]	Dermatitis [1.56]	Drugs [1.88]	Acne [1.32]
Bromley	Headaches [0.92]	Anxiety [1.0]	Back Pain [0.93]	Depression [1.08]	Neonatal [1.12]	Asthma [1.9]	Conduct [1.0]	Dermatitis [1.56]	Drugs [1.82]	Acne [1.35]
Camden	Headaches [0.97]	Anxiety [0.96]	Back Pain [0.94]	Depression [1.13]	Asthma [1.98]	Drugs [2.29]	Conduct [1.05]	Dermatitis [1.57]	Neonatal [0.88]	Acne [1.24]
Croydon	Headaches [0.92]	Anxiety [1.02]	Back Pain [0.93]	Depression [1.08]	Asthma [1.95]	Conduct [0.99]	Neonatal [1.01]	Dermatitis [1.57]	Drugs [1.84]	Acne [1.34]
Ealing	Headaches [0.91]	Anxiety [0.99]	Back Pain [0.91]	Depression [1.07]	Asthma [1.95]	Neonatal [1.13]	Conduct [1.02]	Dermatitis [1.57]	Drugs [1.77]	Acne [1.27]
Enfield	Headaches [0.91]	Anxiety [1.01]	Back Pain [0.93]	Depression [1.07]	Asthma [1.9]	Conduct [1.0]	Neonatal [1.05]	Dermatitis [1.56]	Drugs [1.78]	Acne [1.34]
Greenwich	Headaches [0.92]	Anxiety [1.02]	Back Pain [0.94]	Depression [1.09]	Asthma [1.92]	Conduct [0.99]	Neonatal [0.97]	Dermatitis [1.56]	Drugs [2.04]	Acne [1.34]
Hackney	Headaches [0.93]	Anxiety [0.98]	Asthma [2.09]	Back Pain [0.92]	Depression [1.08]	Conduct [1.03]	Neonatal [1.01]	Dermatitis [1.57]	Drugs [2.1]	Acne [1.21]
Hammersmith & Fulham	Headaches [0.95]	Anxiety [0.95]	Back Pain [0.91]	Depression [1.1]	Asthma [1.99]	Conduct [1.06]	Drugs [2.13]	Neonatal [0.99]	Dermatitis [1.58]	Acne [1.19]
Haringey	Headaches [0.91]	Anxiety [1.0]	Back Pain [0.92]	Depression [1.07]	Asthma [1.96]	Neonatal [1.1]	Conduct [1.01]	Dermatitis [1.56]	Drugs [1.89]	Acne [1.29]

eFigure 7e. Leading ten causes of DALYs with the ratio of observed DALYs to DALYs expected on the basis of Socio-Demographic Index alone in 2017, 10-19 years, both sexes combined. The top ten causes contributing to DALYs are listed globally, by socio-demographic quintile, and then by GBD superregion, region, country, and subnationally where modeled. For each cell, the ratio of observed DALYs to DALYs expected on the basis of socio-demographic index (SDI) alone are listed. Abbreviations: DALY=disability-adjusted life year, GBD=Global Burden of Disease.

Values shown in brackets represent the ratio of observed DALYs to predicted DALYs on the basis of Socio-Demographic Index (SDI), rounded to two (2) digits. Color ranges (shown below) were calculated to place a roughly equal number of cells into each bin.

COLOR KEY:	[0.0-0.73]	(0.73-0.88]	(0.88-0.95]	(0.95-1.01]	(1.01-1.09]	(1.09-1.26]	(1.26-1.55]	(1.55-2.09]	2.09+

	1	2	3	4	5	6	7	8	9	10
Harrow	Headaches [0.91]	Anxiety [1.0]	Back Pain [0.92]	Depression [1.08]	Neonatal [1.15]	Asthma [1.9]	Conduct [1.01]	Dermatitis [1.56]	Drugs [1.79]	Acne [1.34]
Havering	Headaches [0.93]	Anxiety [1.03]	Back Pain [0.96]	Depression [1.1]	Asthma [1.93]	Conduct [0.98]	Neonatal [1.0]	Dermatitis [1.55]	Drugs [1.92]	Acne [1.4]
Hillingdon	Headaches [0.93]	Anxiety [0.98]	Back Pain [0.93]	Depression [1.1]	Asthma [2.0]	Neonatal [1.15]	Conduct [1.02]	Dermatitis [1.56]	Drugs [1.9]	Acne [1.29]
Hounslow	Headaches [0.92]	Anxiety [0.98]	Back Pain [0.91]	Depression [1.08]	Asthma [1.96]	Conduct [1.03]	Neonatal [1.09]	Dermatitis [1.57]	Drugs [1.79]	Acne [1.24]
Islington	Headaches [0.96]	Anxiety [0.96]	Back Pain [0.93]	Asthma [2.07]	Depression [1.11]	Drugs [2.4]	Conduct [1.04]	Dermatitis [1.58]	Neonatal [0.86]	Acne [1.2]
Kensington & Chelsea	Headaches [0.96]	Anxiety [0.95]	Back Pain [0.92]	Depression [1.12]	Asthma [1.97]	Drugs [2.27]	Conduct [1.05]	Dermatitis [1.58]	Acne [1.25]	Neonatal [0.85]
Kingston upon Thames	Headaches [0.95]	Anxiety [0.98]	Back Pain [0.93]	Depression [1.11]	Asthma [1.95]	Neonatal [1.08]	Conduct [1.02]	Dermatitis [1.57]	Drugs [1.97]	Acne [1.29]
Lambeth	Headaches [0.94]	Anxiety [0.97]	Back Pain [0.91]	Depression [1.08]	Asthma [1.98]	Conduct [1.04]	Dermatitis [1.58]	Drugs [2.03]	Acne [1.19]	Neonatal [0.73]
Lewisham	Headaches [0.92]	Anxiety [1.01]	Neonatal [1.21]	Back Pain [0.93]	Depression [1.08]	Asthma [1.95]	Conduct [1.0]	Dermatitis [1.56]	Drugs [1.83]	Acne [1.29]
Merton	Headaches [0.91]	Anxiety [0.98]	Back Pain [0.9]	Depression [1.07]	Asthma [1.95]	Conduct [1.03]	Neonatal [1.04]	Dermatitis [1.56]	Acne [1.26]	Drugs [1.69]
Newham	Headaches [0.93]	Anxiety [1.02]	Back Pain [0.95]	Depression [1.1]	Asthma [1.94]	Neonatal [1.08]	Conduct [0.99]	Dermatitis [1.56]	Drugs [1.89]	Acne [1.32]
Redbridge	Headaches [0.9]	Anxiety [1.02]	Back Pain [0.93]	Depression [1.07]	Asthma [1.92]	Conduct [1.0]	Neonatal [1.01]	Dermatitis [1.56]	Drugs [1.81]	Acne [1.34]
Richmond upon Thames	Headaches [0.93]	Anxiety [0.97]	Back Pain [0.9]	Depression [1.07]	Neonatal [1.14]	Asthma [1.91]	Conduct [1.04]	Dermatitis [1.58]	Drugs [1.76]	Acne [1.23]
Southwark	Headaches [0.94]	Anxiety [0.96]	Back Pain [0.91]	Depression [1.1]	Asthma [1.99]	Conduct [1.05]	Dermatitis [1.58]	Drugs [1.92]	Acne [1.19]	Neonatal [0.75]
Sutton	Headaches [0.92]	Anxiety [1.01]	Neonatal [1.29]	Back Pain [0.93]	Depression [1.09]	Asthma [1.93]	Conduct [1.0]	Dermatitis [1.56]	Drugs [1.88]	Acne [1.35]
Tower Hamlets	Headaches [0.94]	Anxiety [0.96]	Back Pain [0.92]	Asthma [2.03]	Depression [1.09]	Neonatal [1.11]	Conduct [1.04]	Dermatitis [1.57]	Drugs [1.91]	Acne [1.19]
Waltham Forest	Headaches [0.91]	Anxiety [1.03]	Back Pain [0.93]	Depression [1.08]	Asthma [1.91]	Conduct [0.99]	Neonatal [1.02]	Dermatitis [1.57]	Drugs [2.01]	Acne [1.35]
Wandsworth	Headaches [0.94]	Anxiety [0.96]	Back Pain [0.9]	Depression [1.09]	Asthma [1.94]	Conduct [1.04]	Dermatitis [1.59]	Neonatal [0.9]	Drugs [1.76]	Acne [1.19]
Westminster	Headaches [0.95]	Anxiety [0.95]	Back Pain [0.93]	Depression [1.11]	Asthma [2.04]	Conduct [1.06]	Drugs [2.14]	Dermatitis [1.56]	Acne [1.23]	Neonatal [0.8]
NE England	Headaches [0.92]	Anxiety [1.03]	Depression [1.1]	Back Pain [0.94]	Conduct [0.98]	Dermatitis [1.68]	Asthma [1.7]	Drugs [2.25]	Neonatal [0.91]	Congenital [1.05]
County Durham	Headaches [0.93]	Anxiety [1.04]	Depression [1.11]	Dermatitis [1.87]	Back Pain [0.89]	Conduct [0.97]	Asthma [1.74]	Neonatal [0.98]	Drugs [2.17]	Congenital [1.03]
Darlington	Headaches [0.92]	Anxiety [1.03]	Back Pain [0.95]	Depression [1.09]	Conduct [0.99]	Asthma [1.72]	Dermatitis [1.56]	Drugs [2.25]	Neonatal [0.88]	Road Inj [0.37]
Gateshead	Headaches [0.92]	Anxiety [1.03]	Back Pain [0.96]	Depression [1.1]	Dermatitis [1.85]	Drugs [2.58]	Conduct [0.98]	Neonatal [1.01]	Asthma [1.74]	Congenital [1.08]
Hartlepool	Headaches [0.92]	Anxiety [1.05]	Back Pain [0.96]	Depression [1.11]	Conduct [0.96]	Neonatal [1.05]	Asthma [1.75]	Drugs [2.5]	Dermatitis [1.55]	Congenital [1.01]
Middlesbrough	Headaches [0.92]	Anxiety [1.04]	Back Pain [0.95]	Depression [1.1]	Conduct [0.97]	Asthma [1.74]	Dermatitis [1.55]	Drugs [2.22]	Neonatal [0.8]	Congenital [1.01]
Newcastle upon Tyne	Headaches [0.96]	Anxiety [0.99]	Back Pain [0.97]	Depression [1.14]	Conduct [1.0]	Drugs [2.29]	Asthma [1.74]	Dermatitis [1.53]	Neonatal [0.84]	Acne [1.35]
N Tyneside	Headaches [0.91]	Anxiety [1.02]	Back Pain [0.93]	Depression [1.08]	Conduct [0.99]	Asthma [1.69]	Dermatitis [1.56]	Drugs [2.17]	Neonatal [0.84]	Congenital [1.07]
Northumberland	Headaches [0.91]	Anxiety [1.03]	Back Pain [0.94]	Depression [1.09]	Conduct [0.98]	Asthma [1.68]	Dermatitis [1.55]	Drugs [2.24]	Neonatal [0.88]	Congenital [1.04]
Redcar & Cleveland	Headaches [0.92]	Anxiety [1.05]	Depression [1.1]	Back Pain [0.95]	Conduct [0.97]	Asthma [1.69]	Dermatitis [1.55]	Drugs [2.2]	Neonatal [0.83]	Road Inj [0.34]
S Tyneside	Headaches [0.93]	Anxiety [1.06]	Back Pain [0.96]	Depression [1.11]	Dermatitis [1.87]	Conduct [0.96]	Asthma [1.7]	Drugs [2.27]	Neonatal [0.88]	Congenital [1.0]
Stockton-on-Tees	Headaches [0.91]	Anxiety [1.02]	Back Pain [0.94]	Depression [1.09]	Neonatal [1.12]	Conduct [0.99]	Asthma [1.73]	Dermatitis [1.55]	Drugs [2.24]	Road Inj [0.37]

eFigure 7e. Leading ten causes of DALYs with the ratio of observed DALYs to DALYs expected on the basis of Socio-Demographic Index alone in 2017, 10-19 years, both sexes combined. The top ten causes contributing to DALYs are listed globally, by socio-demographic quintile, and then by GBD superregion, region, country, and subnationally where modeled. For each cell, the ratio of observed DALYs to DALYs expected on the basis of socio-demographic index (SDI) alone are listed. Abbreviations: DALY=disability-adjusted life year, GBD=Global Burden of Disease.

Values shown in brackets represent the ratio of observed DALYs to predicted DALYs on the basis of Socio-Demographic Index (SDI), rounded to two (2) digits. Color ranges (shown below) were calculated to place a roughly equal number of cells into each bin.

COLOR KEY:		[0.0-0.73]	(0.73-0.88]	(0.88-0.95]	(0.95-1.01]	(1.01-1.09]	(1.09-1.26]	(1.26-1.55]	(1.55-2.09]	2.09+

	1	2	3	4	5	6	7	8	9	10
Sunderland	Headaches [0.92]	Anxiety [1.03]	Back Pain [0.95]	Depression [1.1]	Dermatitis [1.87]	Conduct [0.98]	Drugs [2.2]	Asthma [1.53]	Neonatal [0.77]	Congenital [1.04]
NW England	Headaches [0.92]	Anxiety [1.01]	Depression [1.11]	Back Pain [0.93]	Neonatal [1.12]	Conduct [0.99]	Asthma [1.74]	Dermatitis [1.55]	Drugs [2.24]	Congenital [1.06]
Blackburn with Darwen	Headaches [0.91]	Anxiety [1.05]	Back Pain [0.95]	Depression [1.08]	Neonatal [1.11]	Conduct [0.98]	Asthma [1.74]	Dermatitis [1.56]	Drugs [2.17]	Acne [1.35]
Blackpool	Headaches [0.92]	Anxiety [1.07]	Drugs [3.09]	Back Pain [0.97]	Depression [1.1]	Neonatal [1.08]	Conduct [0.96]	Asthma [1.78]	Dermatitis [1.57]	Congenital [0.92]
Bolton	Headaches [0.91]	Anxiety [1.04]	Back Pain [0.96]	Depression [1.09]	Conduct [0.98]	Neonatal [1.06]	Asthma [1.71]	Drugs [2.4]	Dermatitis [1.55]	Congenital [0.98]
Bury	Headaches [0.9]	Anxiety [1.02]	Back Pain [0.95]	Depression [1.08]	Conduct [0.99]	Neonatal [1.01]	Asthma [1.66]	Dermatitis [1.55]	Drugs [2.16]	Acne [1.34]
Cheshire E	Headaches [0.93]	Neonatal [1.38]	Anxiety [0.99]	Back Pain [0.94]	Depression [1.09]	Conduct [1.01]	Asthma [1.7]	Dermatitis [1.56]	Drugs [1.91]	Acne [1.3]
Cheshire W & Chester	Headaches [0.93]	Anxiety [1.0]	Back Pain [0.94]	Depression [1.1]	Neonatal [1.12]	Conduct [1.0]	Asthma [1.72]	Dermatitis [1.55]	Drugs [2.15]	Road Inj [0.4]
Cumbria	Headaches [0.92]	Anxiety [1.02]	Back Pain [0.94]	Depression [1.1]	Neonatal [1.11]	Conduct [0.99]	Asthma [1.72]	Dermatitis [1.55]	Drugs [2.27]	Road Inj [0.41]
Halton	Headaches [0.92]	Anxiety [1.03]	Back Pain [0.93]	Depression [1.09]	Neonatal [1.14]	Conduct [0.98]	Asthma [1.73]	Dermatitis [1.56]	Drugs [2.14]	Acne [1.34]
Knowsley	Headaches [0.92]	Anxiety [1.03]	Back Pain [0.95]	Depression [1.1]	Neonatal [1.09]	Asthma [1.84]	Conduct [0.98]	Drugs [2.37]	Dermatitis [1.55]	Congenital [1.21]
Lancashire	Headaches [0.93]	Anxiety [1.02]	Depression [1.1]	Back Pain [0.88]	Neonatal [1.08]	Conduct [0.99]	Asthma [1.75]	Dermatitis [1.55]	Drugs [2.24]	Congenital [1.07]
Liverpool	Headaches [0.96]	Depression [1.31]	Anxiety [0.93]	Back Pain [0.96]	Neonatal [1.21]	Drugs [2.55]	Conduct [0.98]	Asthma [1.74]	Dermatitis [1.54]	Congenital [1.15]
Manchester	Headaches [0.95]	Anxiety [0.98]	Back Pain [0.98]	Neonatal [1.2]	Depression [1.11]	Conduct [1.02]	Asthma [1.77]	Dermatitis [1.55]	Drugs [2.05]	Acne [1.22]
Oldham	Headaches [0.91]	Anxiety [1.05]	Back Pain [0.96]	Depression [1.09]	Asthma [1.88]	Conduct [0.97]	Dermatitis [1.56]	Drugs [2.36]	Congenital [1.13]	Neonatal [0.83]
Rochdale	Headaches [0.91]	Anxiety [1.05]	Back Pain [0.96]	Depression [1.09]	Conduct [0.97]	Asthma [1.78]	Drugs [2.47]	Dermatitis [1.56]	Neonatal [0.86]	Congenital [1.0]
Salford	Headaches [0.92]	Anxiety [1.02]	Neonatal [1.25]	Back Pain [0.95]	Depression [1.09]	Conduct [0.99]	Asthma [1.79]	Dermatitis [1.56]	Drugs [2.23]	Road Inj [0.37]
Sefton	Headaches [0.92]	Anxiety [1.04]	Back Pain [0.94]	Depression [1.09]	Neonatal [1.12]	Conduct [0.98]	Drugs [2.49]	Asthma [1.71]	Dermatitis [1.55]	Congenital [1.08]
St Helens	Headaches [0.91]	Anxiety [1.04]	Back Pain [0.95]	Depression [1.09]	Neonatal [1.13]	Conduct [0.98]	Asthma [1.73]	Drugs [2.45]	Dermatitis [1.55]	Road Inj [0.38]
Stockport	Headaches [0.92]	Anxiety [1.01]	Back Pain [0.93]	Depression [1.09]	Conduct [1.0]	Asthma [1.69]	Dermatitis [1.56]	Neonatal [0.94]	Drugs [2.09]	Acne [1.31]
Tameside	Headaches [0.91]	Anxiety [1.05]	Depression [1.09]	Conduct [0.97]	Asthma [1.74]	Dermatitis [1.56]	Back Pain [0.77]	Neonatal [0.92]	Drugs [2.19]	Congenital [0.95]
Trafford	Headaches [0.91]	Anxiety [0.98]	Neonatal [1.22]	Back Pain [0.91]	Depression [1.07]	Conduct [1.03]	Asthma [1.73]	Dermatitis [1.57]	Drugs [1.79]	Acne [1.25]
Warrington	Headaches [0.92]	Anxiety [0.99]	Neonatal [1.3]	Back Pain [0.93]	Depression [1.09]	Conduct [1.01]	Asthma [1.74]	Dermatitis [1.55]	Drugs [2.15]	Acne [1.29]
Wigan	Headaches [0.91]	Anxiety [1.04]	Back Pain [0.95]	Depression [1.08]	Neonatal [1.13]	Conduct [0.98]	Asthma [1.72]	Dermatitis [1.55]	Drugs [2.28]	Congenital [0.99]
Wirral	Headaches [0.91]	Anxiety [1.04]	Neonatal [1.27]	Back Pain [0.94]	Depression [1.09]	Conduct [0.98]	Asthma [1.7]	Drugs [2.39]	Dermatitis [1.55]	Congenital [1.08]
SE England	Headaches [0.92]	Anxiety [1.0]	Depression [1.09]	Back Pain [0.91]	Conduct [1.0]	Dermatitis [1.71]	Asthma [1.78]	Neonatal [1.03]	Drugs [1.91]	Acne [1.34]
Bracknell Forest	Headaches [0.92]	Anxiety [0.99]	Back Pain [0.91]	Depression [1.08]	Conduct [1.02]	Dermatitis [1.56]	Asthma [1.62]	Acne [1.32]	Neonatal [0.8]	Drugs [1.73]
Brighton & Hove	Headaches [0.96]	Anxiety [0.98]	Depression [1.13]	Back Pain [0.93]	Dermatitis [1.85]	Drugs [2.46]	Neonatal [1.12]	Conduct [1.01]	Asthma [1.66]	Acne [1.32]
Buckinghamshire	Headaches [0.92]	Anxiety [0.99]	Back Pain [0.92]	Depression [1.09]	Neonatal [1.15]	Conduct [1.01]	Dermatitis [1.56]	Asthma [1.62]	Drugs [1.83]	Acne [1.31]
E Sussex	Headaches [0.92]	Anxiety [1.04]	Depression [1.1]	Back Pain [0.94]	Dermatitis [1.83]	Neonatal [1.1]	Conduct [0.97]	Asthma [1.57]	Drugs [2.17]	Acne [1.42]
Hampshire	Headaches [0.92]	Asthma [2.43]	Anxiety [1.0]	Back Pain [0.92]	Depression [1.09]	Conduct [1.0]	Neonatal [1.01]	Dermatitis [1.56]	Acne [1.38]	Drugs [1.84]

eFigure 7e. Leading ten causes of DALYs with the ratio of observed DALYs to DALYs expected on the basis of Socio-Demographic Index alone in 2017, 10-19 years, both sexes combined. The top ten causes contributing to DALYs are listed globally, by socio-demographic quintile, and then by GBD superregion, region, country, and subnationally where modeled. For each cell, the ratio of observed DALYs to DALYs expected on the basis of socio-demographic index (SDI) alone are listed. Abbreviations: DALY=disability-adjusted life year, GBD=Global Burden of Disease.

Values shown in brackets represent the ratio of observed DALYs to predicted DALYs on the basis of Socio-Demographic Index (SDI), rounded to two (2) digits. Color ranges (shown below) were calculated to place a roughly equal number of cells into each bin.

COLOR KEY:	[0.0-0.73]	(0.73-0.88]	(0.88-0.95]	(0.95-1.01]	(1.01-1.09]	(1.09-1.26]	(1.26-1.55]	(1.55-2.09]	2.09+

	1	2	3	4	5	6	7	8	9	10
Isle of Wight	Headaches [0.92]	Asthma [2.43]	Anxiety [1.03]	Depression [1.1]	Back Pain [0.94]	Conduct [0.98]	Neonatal [0.95]	Dermatitis [1.5]	Drugs [2.1]	Acne [1.46]
Kent	Headaches [0.92]	Anxiety [1.02]	Back Pain [0.93]	Depression [1.1]	Dermatitis [1.83]	Conduct [0.99]	Neonatal [1.0]	Asthma [1.6]	Drugs [1.98]	Acne [1.39]
Medway	Headaches [0.92]	Anxiety [1.04]	Depression [1.1]	Back Pain [0.93]	Neonatal [1.07]	Conduct [0.97]	Asthma [1.65]	Dermatitis [1.55]	Drugs [2.08]	Acne [1.4]
Milton Keynes	Headaches [0.91]	Anxiety [1.0]	Back Pain [0.9]	Depression [1.06]	Conduct [1.02]	Dermatitis [1.58]	Asthma [1.66]	Neonatal [0.88]	Drugs [1.73]	Acne [1.25]
Oxfordshire	Headaches [0.94]	Anxiety [0.98]	Dermatitis [2.04]	Back Pain [0.92]	Depression [1.1]	Conduct [1.02]	Asthma [1.63]	Neonatal [0.93]	Drugs [1.84]	Acne [1.32]
Portsmouth	Headaches [0.94]	Anxiety [1.0]	Depression [1.12]	Back Pain [0.93]	Conduct [1.0]	Neonatal [0.98]	Drugs [2.22]	Asthma [1.65]	Dermatitis [1.54]	Acne [1.35]
Reading	Headaches [0.94]	Anxiety [0.97]	Dermatitis [2.06]	Neonatal [1.21]	Back Pain [0.91]	Depression [1.1]	Conduct [1.03]	Asthma [1.69]	Drugs [2.0]	Acne [1.23]
Slough	Headaches [0.9]	Anxiety [0.99]	Back Pain [0.89]	Depression [1.05]	Conduct [1.02]	Neonatal [1.07]	Asthma [1.77]	Dermatitis [1.58]	Drugs [1.83]	Acne [1.22]
Southampton	Headaches [0.94]	Anxiety [1.0]	Depression [1.12]	Back Pain [0.93]	Conduct [1.0]	Neonatal [0.97]	Asthma [1.66]	Dermatitis [1.54]	Drugs [2.14]	Acne [1.36]
Surrey	Headaches [0.93]	Anxiety [0.98]	Depression [1.09]	Dermatitis [1.83]	Neonatal [1.12]	Back Pain [0.84]	Conduct [1.03]	Asthma [1.62]	Acne [1.29]	Drugs [1.73]
West Berkshire	Headaches [0.92]	Anxiety [0.99]	Back Pain [0.92]	Depression [1.09]	Conduct [1.02]	Dermatitis [1.56]	Asthma [1.66]	Neonatal [0.93]	Acne [1.32]	Drugs [1.76]
W Sussex	Headaches [0.91]	Anxiety [1.0]	Back Pain [0.92]	Depression [1.08]	Conduct [1.0]	Asthma [1.86]	Neonatal [1.05]	Dermatitis [1.62]	Drugs [1.94]	Acne [1.35]
Windsor & Maidenhead	Headaches [0.92]	Anxiety [0.96]	Back Pain [0.9]	Depression [1.07]	Conduct [1.04]	Dermatitis [1.56]	Neonatal [0.96]	Asthma [1.63]	Acne [1.24]	Drugs [1.71]
Wokingham	Headaches [0.92]	Anxiety [0.97]	Back Pain [0.9]	Depression [1.08]	Conduct [1.03]	Neonatal [0.99]	Dermatitis [1.57]	Asthma [1.62]	Acne [1.27]	Drugs [1.63]
SW England	Headaches [0.93]	Anxiety [1.01]	Depression [1.1]	Back Pain [0.93]	Neonatal [1.08]	Conduct [0.99]	Dermatitis [1.55]	Asthma [1.64]	Drugs [2.04]	Acne [1.39]
Bath & NE Somerset	Headaches [0.96]	Neonatal [1.43]	Anxiety [0.99]	Back Pain [0.95]	Depression [1.14]	Conduct [1.0]	Dermatitis [1.54]	Asthma [1.61]	Drugs [2.01]	Acne [1.4]
Bournemouth	Headaches [0.95]	Anxiety [1.0]	Depression [1.12]	Back Pain [0.94]	Conduct [1.0]	Neonatal [1.06]	Drugs [2.21]	Dermatitis [1.54]	Asthma [1.63]	Acne [1.38]
Bristol	Headaches [0.95]	Anxiety [0.99]	Neonatal [1.22]	Back Pain [0.92]	Depression [1.12]	Conduct [1.01]	Asthma [1.68]	Dermatitis [1.56]	Drugs [2.05]	Acne [1.28]
Cornwall	Headaches [0.93]	Anxiety [1.04]	Depression [1.11]	Back Pain [0.89]	Neonatal [1.07]	Conduct [0.98]	Dermatitis [1.55]	Asthma [1.62]	Drugs [2.08]	Acne [1.44]
Devon	Headaches [0.92]	Anxiety [1.01]	Back Pain [0.94]	Depression [1.11]	Conduct [0.99]	Neonatal [0.98]	Dermatitis [1.55]	Asthma [1.63]	Drugs [2.12]	Acne [1.42]
Dorset	Headaches [0.92]	Anxiety [1.02]	Back Pain [0.93]	Depression [1.09]	Conduct [0.99]	Neonatal [1.05]	Dermatitis [1.55]	Asthma [1.64]	Drugs [2.04]	Acne [1.43]
Gloucestershire	Headaches [0.93]	Anxiety [1.01]	Back Pain [0.93]	Depression [1.1]	Conduct [1.0]	Neonatal [0.99]	Dermatitis [1.55]	Asthma [1.64]	Drugs [1.97]	Acne [1.39]
N Somerset	Headaches [0.92]	Anxiety [1.02]	Neonatal [1.17]	Depression [1.09]	Back Pain [0.92]	Conduct [0.99]	Asthma [1.66]	Dermatitis [1.56]	Drugs [2.25]	Acne [1.35]
Plymouth	Headaches [0.93]	Anxiety [1.02]	Depression [1.11]	Back Pain [0.93]	Neonatal [1.07]	Conduct [0.98]	Drugs [2.32]	Dermatitis [1.55]	Asthma [1.64]	Acne [1.38]
Poole	Headaches [0.92]	Anxiety [1.01]	Depression [1.09]	Back Pain [0.92]	Conduct [1.0]	Neonatal [1.05]	Dermatitis [1.56]	Asthma [1.62]	Drugs [1.82]	Acne [1.37]
Somerset	Headaches [0.92]	Anxiety [1.03]	Back Pain [0.95]	Depression [1.11]	Neonatal [1.13]	Conduct [0.98]	Dermatitis [1.55]	Asthma [1.63]	Drugs [2.04]	Acne [1.45]
S Gloucestershire	Headaches [0.93]	Anxiety [0.99]	Back Pain [0.92]	Depression [1.09]	Neonatal [1.12]	Conduct [1.01]	Dermatitis [1.55]	Asthma [1.65]	Acne [1.35]	Drugs [1.82]
Swindon	Headaches [0.92]	Anxiety [1.01]	Back Pain [0.93]	Depression [1.08]	Conduct [1.0]	Asthma [1.67]	Dermatitis [1.56]	Drugs [1.89]	Neonatal [0.77]	Acne [1.32]
Torbay	Headaches [0.91]	Anxiety [1.05]	Depression [1.1]	Back Pain [0.94]	Conduct [0.97]	Dermatitis [1.56]	Asthma [1.63]	Drugs [2.38]	Neonatal [0.88]	Acne [1.46]
Wiltshire	Headaches [0.92]	Anxiety [1.02]	Neonatal [1.25]	Back Pain [0.94]	Depression [1.09]	Conduct [0.99]	Dermatitis [1.55]	Asthma [1.63]	Drugs [1.93]	Acne [1.41]
W Midlands	Headaches [0.92]	Anxiety [1.02]	Back Pain [0.93]	Depression [1.09]	Conduct [0.99]	Neonatal [1.01]	Asthma [1.69]	Dermatitis [1.55]	Drugs [2.02]	Acne [1.34]

eFigure 7e. Leading ten causes of DALYs with the ratio of observed DALYs to DALYs expected on the basis of Socio-Demographic Index alone in 2017, 10-19 years, both sexes combined. The top ten causes contributing to DALYs are listed globally, by socio-demographic quintile, and then by GBD superregion, region, country, and subnationally where modeled. For each cell, the ratio of observed DALYs to DALYs expected on the basis of socio-demographic index (SDI) alone are listed. Abbreviations: DALY=disability-adjusted life year, GBD=Global Burden of Disease.

Values shown in brackets represent the ratio of observed DALYs to predicted DALYs on the basis of Socio-Demographic Index (SDI), rounded to two (2) digits. Color ranges (shown below) were calculated to place a roughly equal number of cells into each bin.

COLOR KEY:	[0.0-0.73]	(0.73-0.88]	(0.88-0.95]	(0.95-1.01]	(1.01-1.09]	(1.09-1.26]	(1.26-1.55]	(1.55-2.09]	2.09+

	1	2	3	4	5	6	7	8	9	10
Birmingham	Headaches [0.92]	Anxiety [1.01]	Back Pain [0.94]	Depression [1.09]	Conduct [0.99]	Neonatal [0.98]	Asthma [1.68]	Dermatitis [1.56]	Drugs [2.0]	Acne [1.3]
Coventry	Headaches [0.94]	Anxiety [1.01]	Back Pain [0.95]	Depression [1.12]	Conduct [0.99]	Asthma [1.73]	Dermatitis [1.55]	Neonatal [0.95]	Drugs [2.03]	Acne [1.32]
Dudley	Headaches [0.91]	Anxiety [1.05]	Back Pain [0.96]	Depression [1.09]	Neonatal [1.12]	Conduct [0.97]	Asthma [1.71]	Dermatitis [1.56]	Drugs [2.11]	Acne [1.38]
Herefordshire	Headaches [0.93]	Anxiety [1.02]	Back Pain [0.95]	Depression [1.1]	Road Inj [0.5]	Conduct [0.98]	Neonatal [1.03]	Asthma [1.68]	Dermatitis [1.56]	Drugs [2.15]
Sandwell	Headaches [0.9]	Anxiety [1.04]	Back Pain [0.95]	Depression [1.08]	Neonatal [1.1]	Conduct [0.98]	Asthma [1.77]	Dermatitis [1.56]	Drugs [1.94]	Congenital [0.92]
Shropshire	Headaches [0.93]	Anxiety [1.02]	Back Pain [0.96]	Depression [1.11]	Conduct [0.98]	Neonatal [1.0]	Dermatitis [1.55]	Asthma [1.64]	Drugs [2.0]	Acne [1.41]
Solihull	Headaches [0.92]	Anxiety [1.0]	Back Pain [0.93]	Depression [1.09]	Conduct [1.01]	Asthma [1.7]	Dermatitis [1.55]	Drugs [1.91]	Road Inj [0.38]	Acne [1.33]
Staffordshire	Headaches [0.92]	Anxiety [1.02]	Depression [1.1]	Conduct [0.99]	Back Pain [0.85]	Neonatal [0.99]	Asthma [1.68]	Dermatitis [1.55]	Drugs [2.09]	Road Inj [0.37]
Stoke-on-Trent	Headaches [0.91]	Anxiety [1.04]	Neonatal [1.21]	Depression [1.09]	Conduct [0.98]	Back Pain [0.85]	Asthma [1.71]	Dermatitis [1.56]	Drugs [2.25]	Acne [1.34]
Telford & Wrekin	Headaches [0.93]	Anxiety [1.04]	Back Pain [0.95]	Depression [1.1]	Conduct [0.97]	Neonatal [1.01]	Asthma [1.7]	Dermatitis [1.56]	Drugs [2.14]	Road Inj [0.35]
Walsall	Headaches [0.91]	Anxiety [1.05]	Back Pain [0.97]	Depression [1.09]	Conduct [0.97]	Neonatal [1.02]	Asthma [1.73]	Dermatitis [1.56]	Drugs [2.17]	Acne [1.38]
Warwickshire	Headaches [0.92]	Anxiety [1.0]	Back Pain [0.93]	Depression [1.09]	Conduct [1.01]	Neonatal [1.02]	Asthma [1.69]	Dermatitis [1.56]	Drugs [1.91]	Acne [1.32]
Wolverhampton	Headaches [0.92]	Anxiety [1.04]	Back Pain [0.95]	Depression [1.1]	Conduct [0.98]	Asthma [1.69]	Dermatitis [1.55]	Drugs [1.99]	Neonatal [0.74]	Acne [1.35]
Worcestershire	Headaches [0.92]	Anxiety [1.02]	Back Pain [0.94]	Depression [1.1]	Neonatal [1.14]	Conduct [0.99]	Dermatitis [1.55]	Asthma [1.64]	Drugs [1.96]	Acne [1.36]
Yorkshire & Humber	Headaches [0.92]	Anxiety [1.02]	Back Pain [0.95]	Depression [1.1]	Conduct [0.99]	Asthma [1.72]	Dermatitis [1.55]	Neonatal [0.95]	Drugs [2.12]	Acne [1.36]
Barnsley	Headaches [0.92]	Anxiety [1.06]	Back Pain [0.97]	Depression [1.1]	Conduct [0.96]	Asthma [1.81]	Neonatal [1.01]	Dermatitis [1.56]	Drugs [2.38]	Road Inj [0.34]
Bradford	Headaches [0.91]	Anxiety [1.04]	Back Pain [0.95]	Depression [1.08]	Conduct [0.98]	Neonatal [1.07]	Asthma [1.68]	Dermatitis [1.56]	Drugs [1.98]	Acne [1.35]
Calderdale	Headaches [0.92]	Anxiety [1.03]	Back Pain [0.95]	Depression [1.09]	Conduct [0.98]	Asthma [1.73]	Drugs [2.35]	Dermatitis [1.56]	Neonatal [0.91]	Road Inj [0.39]
Doncaster	Headaches [0.92]	Anxiety [1.06]	Neonatal [1.24]	Back Pain [0.96]	Depression [1.09]	Conduct [0.96]	Asthma [1.74]	Dermatitis [1.57]	Drugs [2.23]	Road Inj [0.35]
E Riding of Yorkshire	Headaches [0.93]	Anxiety [1.03]	Back Pain [0.95]	Depression [1.11]	Conduct [0.98]	Asthma [1.73]	Dermatitis [1.55]	Road Inj [0.43]	Neonatal [0.94]	Drugs [2.09]
Kingston upon Hull	Headaches [0.91]	Anxiety [1.03]	Back Pain [0.94]	Depression [1.09]	Conduct [0.98]	Asthma [1.8]	Drugs [2.5]	Dermatitis [1.55]	Neonatal [0.87]	Acne [1.36]
Kirklees	Headaches [0.92]	Anxiety [1.04]	Back Pain [0.95]	Depression [1.1]	Conduct [0.98]	Asthma [1.69]	Dermatitis [1.56]	Neonatal [0.88]	Drugs [2.11]	Acne [1.38]
Leeds	Headaches [0.94]	Anxiety [0.99]	Back Pain [0.94]	Depression [1.11]	Conduct [1.01]	Asthma [1.71]	Dermatitis [1.55]	Drugs [2.01]	Neonatal [0.84]	Acne [1.29]
NE Lincolnshire	Headaches [0.92]	Anxiety [1.04]	Back Pain [0.94]	Depression [1.09]	Conduct [0.97]	Asthma [1.77]	Dermatitis [1.56]	Drugs [2.29]	Neonatal [0.91]	Road Inj [0.35]
N Lincolnshire	Headaches [0.91]	Anxiety [1.04]	Back Pain [0.94]	Depression [1.08]	Conduct [0.98]	Road Inj [0.48]	Asthma [1.78]	Dermatitis [1.56]	Drugs [2.09]	Neonatal [0.84]
N Yorkshire	Headaches [0.91]	Anxiety [1.01]	Back Pain [0.94]	Depression [1.09]	Conduct [1.0]	Neonatal [1.08]	Asthma [1.65]	Dermatitis [1.55]	Drugs [1.9]	Acne [1.38]
Rotherham	Headaches [0.92]	Anxiety [1.05]	Back Pain [0.97]	Depression [1.1]	Conduct [0.97]	Asthma [1.75]	Dermatitis [1.56]	Drugs [2.2]	Neonatal [0.86]	Road Inj [0.34]
Sheffield	Headaches [0.95]	Anxiety [1.0]	Back Pain [0.96]	Depression [1.12]	Conduct [0.99]	Neonatal [0.98]	Asthma [1.69]	Dermatitis [1.54]	Drugs [2.13]	Acne [1.35]
Wakefield	Headaches [0.92]	Anxiety [1.04]	Back Pain [0.96]	Depression [1.09]	Conduct [0.97]	Asthma [1.79]	Drugs [2.43]	Dermatitis [1.56]	Road Inj [0.4]	Neonatal [0.75]
York	Headaches [0.96]	Anxiety [0.99]	Back Pain [0.96]	Depression [1.14]	Conduct [1.01]	Asthma [1.71]	Drugs [2.18]	Dermatitis [1.54]	Neonatal [0.85]	Road Inj [0.4]
N Ireland	Anxiety [1.67]	Headaches [1.0]	Depression [1.21]	Back Pain [0.98]	Road Inj [0.43]	Conduct [0.83]	Asthma [1.55]	Neonatal [0.84]	Dermatitis [1.3]	Acne [1.36]

eFigure 7e. Leading ten causes of DALYs with the ratio of observed DALYs to DALYs expected on the basis of Socio-Demographic Index alone in 2017, 10-19 years, both sexes combined. The top ten causes contributing to DALYs are listed globally, by socio-demographic quintile, and then by GBD superregion, region, country, and subnationally where modeled. For each cell, the ratio of observed DALYs to DALYs expected on the basis of socio-demographic index (SDI) alone are listed. Abbreviations: DALY=disability-adjusted life year, GBD=Global Burden of Disease.

Values shown in brackets represent the ratio of observed DALYs to predicted DALYs on the basis of Socio-Demographic Index (SDI), rounded to two (2) digits. Color ranges (shown below) were calculated to place a roughly equal number of cells into each bin.

COLOR KEY:	[0.0-0.73]	(0.73-0.88]	(0.88-0.95]	(0.95-1.01]	(1.01-1.09]	(1.09-1.26]	(1.26-1.55]	(1.55-2.09]	2.09+

	1	2	3	4	5	6	7	8	9	10
Scotland	Headaches [0.98]	Anxiety [1.12]	Depression [1.14]	Back Pain [0.92]	Asthma [1.78]	Drugs [2.55]	Conduct [0.91]	Dermatitis [1.44]	Neonatal [0.81]	Road Inj [0.35]
Wales	Headaches [1.0]	Anxiety [1.12]	Depression [1.2]	Conduct [0.9]	Back Pain [0.77]	Asthma [1.51]	Neonatal [0.83]	Dermatitis [1.31]	Road Inj [0.36]	Acne [1.42]
Latin America and Caribbean	Violence [4.99]	Headaches [1.17]	Road Inj [0.81]	Back Pain [1.28]	Neonatal [1.18]	Conduct [0.95]	Anxiety [0.94]	Depression [0.93]	Self Harm [0.71]	Congenital [0.75]
**Andean Latin America**	Headaches [1.12]	Road Inj [0.67]	Back Pain [1.08]	Neonatal [1.12]	Conduct [0.94]	Anxiety [0.97]	Self Harm [0.81]	Violence [0.96]	Dermatitis [1.4]	Depression [0.77]
Bolivia	Headaches [1.16]	Road Inj [0.66]	Back Pain [1.21]	Iron [1.2]	Conduct [0.93]	Neonatal [1.08]	Anxiety [1.02]	Violence [0.89]	Asthma [1.38]	Dermatitis [1.46]
Ecuador	Road Inj [1.02]	Headaches [1.33]	Self Harm [1.65]	Violence [1.63]	Neonatal [1.13]	Back Pain [1.0]	Conduct [0.93]	Anxiety [0.97]	Depression [0.98]	Epilepsy [1.36]
Peru	Headaches [0.99]	Back Pain [1.08]	Road Inj [0.47]	Neonatal [1.13]	Conduct [0.94]	Anxiety [0.96]	Dermatitis [1.48]	LRI [1.35]	Asthma [1.16]	Iron [0.9]
**Caribbean**	Road Inj [1.05]	Headaches [1.15]	Violence [2.27]	Disaster [363.5]	Iron [1.79]	Anxiety [1.05]	Conduct [0.86]	Depression [0.95]	Neonatal [0.95]	Asthma [1.56]
Antigua	Iron [5.53]	Headaches [1.13]	Anxiety [0.97]	Drown [1.71]	Road Inj [0.45]	Conduct [0.87]	Neonatal [0.98]	Asthma [1.49]	Depression [0.83]	Violence [1.55]
Bahamas	Violence [5.83]	Headaches [1.13]	Road Inj [0.72]	Anxiety [0.94]	Drown [2.14]	Conduct [0.89]	Neonatal [0.97]	Asthma [1.56]	Depression [0.82]	Back Pain [0.71]
Barbados	Headaches [1.13]	Violence [3.02]	Road Inj [0.56]	Anxiety [0.95]	Neonatal [1.05]	Conduct [0.88]	Depression [0.87]	Back Pain [0.73]	Drown [1.55]	Asthma [1.44]
Belize	Violence [2.97]	Headaches [1.16]	Road Inj [0.8]	Iron [1.57]	Anxiety [1.1]	Drown [0.99]	Neonatal [1.05]	Conduct [0.86]	Asthma [1.61]	Depression [0.94]
Bermuda	Headaches [1.13]	Anxiety [0.89]	Conduct [0.91]	Neonatal [0.98]	Asthma [1.68]	Depression [0.87]	Road Inj [0.41]	Back Pain [0.7]	Acne [1.21]	Dermatitis [0.98]
Cuba	Headaches [1.14]	Anxiety [1.01]	Conduct [0.87]	Neonatal [0.96]	Depression [0.93]	Road Inj [0.38]	Asthma [1.53]	Iron [1.37]	Back Pain [0.64]	Violence [0.96]
Dominica	Road Inj [1.02]	Headaches [1.13]	Violence [2.5]	Drown [1.94]	Iron [2.3]	Anxiety [1.0]	Neonatal [1.11]	Epilepsy [2.09]	Asthma [1.75]	Conduct [0.87]
Dominican Rep	Road Inj [1.14]	Headaches [1.17]	Violence [2.18]	Anxiety [1.11]	Neonatal [1.05]	Conduct [0.86]	Depression [1.01]	Iron [1.03]	Back Pain [0.85]	Asthma [1.21]
Grenada	Headaches [1.15]	Anxiety [1.05]	Neonatal [1.09]	Drown [1.09]	Road Inj [0.42]	Conduct [0.87]	Asthma [1.58]	Depression [0.92]	Back Pain [0.8]	Congenital [0.74]
Guyana	Headaches [1.18]	Self Harm [1.77]	Road Inj [0.69]	Violence [1.65]	Depression [1.41]	Drown [1.03]	Anxiety [1.12]	Neonatal [1.11]	Conduct [0.85]	Iron [0.99]
Haiti	Road Inj [1.51]	Disaster [299.53]	Iron [1.4]	Headaches [1.23]	Violence [1.83]	HIV [19.49]	Drown [0.88]	Anxiety [1.14]	Asthma [1.51]	Conduct [0.85]
Jamaica	Violence [3.82]	Headaches [1.14]	Anxiety [1.01]	Iron [1.91]	Neonatal [1.04]	Road Inj [0.42]	Conduct [0.86]	Asthma [1.63]	Back Pain [0.79]	Depression [0.86]
Puerto Rico	Disaster [5205.94]	Violence [7.58]	Headaches [1.13]	Asthma [2.03]	Anxiety [0.89]	Road Inj [0.49]	Neonatal [1.05]	Conduct [0.91]	Back Pain [0.68]	Depression [0.77]
St Lucia	Headaches [1.15]	Violence [2.43]	Road Inj [0.56]	Anxiety [1.04]	Neonatal [1.11]	Conduct [0.86]	Depression [0.94]	Asthma [1.61]	Drown [1.01]	Back Pain [0.8]
St Vincent	Violence [2.46]	Headaches [1.16]	Road Inj [0.53]	Drown [1.18]	Anxiety [1.09]	Neonatal [1.07]	Conduct [0.86]	Depression [0.96]	Asthma [1.58]	Back Pain [0.83]
Suriname	Road Inj [0.92]	Headaches [1.14]	Self Harm [1.84]	Depression [1.29]	Anxiety [1.05]	Violence [1.36]	Drown [1.11]	Neonatal [1.04]	Conduct [0.87]	Asthma [1.67]
Trinidad Tobago	Violence [3.93]	Headaches [1.12]	Road Inj [0.59]	Anxiety [0.98]	Depression [1.05]	Neonatal [1.06]	Asthma [1.79]	Conduct [0.88]	Self Harm [0.86]	Back Pain [0.75]
Virgin Isl US	Violence [7.82]	Headaches [1.12]	Anxiety [0.89]	Neonatal [1.01]	Conduct [0.92]	Road Inj [0.44]	Depression [0.87]	Asthma [1.55]	Back Pain [0.69]	Disaster [380.64]
**Central Latin America**	Violence [4.95]	Headaches [1.0]	Road Inj [0.71]	Neonatal [1.41]	Conduct [0.98]	Back Pain [0.81]	Self Harm [0.84]	Epilepsy [1.37]	Depression [0.74]	Anxiety [0.69]
Colombia	Violence [4.5]	Headaches [1.05]	Road Inj [0.58]	Neonatal [1.39]	Back Pain [1.11]	Conduct [0.95]	Self Harm [0.8]	Epilepsy [1.33]	Anxiety [0.6]	Depression [0.63]
Costa Rica	Headaches [1.0]	Neonatal [1.31]	Road Inj [0.53]	Conduct [0.95]	Violence [1.4]	Back Pain [0.85]	Depression [0.82]	Anxiety [0.67]	Asthma [1.25]	Self Harm [0.68]
El Salvador	Violence [8.19]	Headaches [1.02]	Road Inj [0.64]	Neonatal [1.41]	Self Harm [1.26]	Conduct [0.94]	Back Pain [0.92]	Depression [0.95]	Drown [0.76]	Anxiety [0.72]
Guatemala	Violence [3.72]	Headaches [1.04]	Road Inj [0.57]	Neonatal [1.48]	Conduct [0.94]	Back Pain [1.06]	LRI [1.03]	Diarrhea [1.51]	Depression [0.94]	Anxiety [0.73]

eFigure 7e. Leading ten causes of DALYs with the ratio of observed DALYs to DALYs expected on the basis of Socio-Demographic Index alone in 2017, 10-19 years, both sexes combined. The top ten causes contributing to DALYs are listed globally, by socio-demographic quintile, and then by GBD superregion, region, country, and subnationally where modeled. For each cell, the ratio of observed DALYs to DALYs expected on the basis of socio-demographic index (SDI) alone are listed. Abbreviations: DALY=disability-adjusted life year, GBD=Global Burden of Disease.

Values shown in brackets represent the ratio of observed DALYs to predicted DALYs on the basis of Socio-Demographic Index (SDI), rounded to two (2) digits. Color ranges (shown below) were calculated to place a roughly equal number of cells into each bin.

COLOR KEY:	[0.0-0.73]	(0.73-0.88]	(0.88-0.95]	(0.95-1.01]	(1.01-1.09]	(1.09-1.26]	(1.26-1.55]	(1.55-2.09]	2.09+

	1	2	3	4	5	6	7	8	9	10
Honduras	Violence [3.63]	Headaches [1.05]	Neonatal [1.62]	Road Inj [0.48]	Conduct [0.94]	Back Pain [0.97]	Epilepsy [1.33]	Diarrhea [1.15]	Anxiety [0.73]	Iron [0.58]
Mexico	Violence [4.49]	Headaches [0.97]	Road Inj [0.71]	Neonatal [1.42]	Conduct [1.01]	Self Harm [0.83]	Epilepsy [1.45]	Anxiety [0.72]	Congenital [0.84]	Depression [0.73]
Aguascalientes	Violence [3.53]	Road Inj [0.89]	Headaches [0.97]	Neonatal [1.4]	Conduct [1.01]	Self Harm [0.94]	Epilepsy [1.56]	Congenital [0.93]	Anxiety [0.71]	Back Pain [0.67]
Baja CA	Violence [4.61]	Headaches [0.97]	Neonatal [1.4]	Road Inj [0.54]	Conduct [1.01]	Anxiety [0.71]	Back Pain [0.66]	Epilepsy [1.37]	Depression [0.67]	Congenital [0.74]
Baja CA Sur	Violence [4.03]	Road Inj [0.76]	Headaches [0.96]	Neonatal [1.47]	Conduct [1.01]	Self Harm [0.8]	Anxiety [0.7]	Epilepsy [1.43]	Depression [0.73]	Congenital [0.85]
Campeche	Violence [3.42]	Headaches [0.98]	Neonatal [1.57]	Road Inj [0.62]	Self Harm [1.29]	Conduct [1.01]	Depression [0.86]	Anxiety [0.74]	Epilepsy [1.28]	Back Pain [0.68]
Chiapas	Violence [2.85]	Headaches [1.01]	Road Inj [0.56]	Neonatal [1.48]	Conduct [1.01]	Epilepsy [1.43]	Self Harm [0.86]	Leukemia [1.44]	Anxiety [0.77]	Back Pain [0.72]
Chihuahua	Violence [6.6]	Road Inj [0.84]	Headaches [0.97]	Neonatal [1.3]	Conduct [1.01]	Self Harm [1.07]	Epilepsy [1.48]	Congenital [0.91]	Anxiety [0.72]	Depression [0.76]
Coahuila	Violence [4.09]	Headaches [0.97]	Road Inj [0.55]	Neonatal [1.19]	Conduct [1.01]	Anxiety [0.71]	Epilepsy [1.4]	Back Pain [0.68]	Self Harm [0.72]	Depression [0.72]
Colima	Violence [4.83]	Road Inj [0.75]	Headaches [0.97]	Neonatal [1.5]	Conduct [1.01]	Epilepsy [1.47]	Anxiety [0.71]	Back Pain [0.66]	Depression [0.71]	Congenital [0.79]
Mexico City	Violence [7.49]	Headaches [0.96]	Neonatal [1.48]	Road Inj [0.52]	Conduct [0.98]	Self Harm [1.0]	Epilepsy [1.8]	Congenital [0.96]	Depression [0.74]	Back Pain [0.64]
Durango	Violence [4.59]	Road Inj [0.87]	Headaches [0.98]	Neonatal [1.26]	Conduct [1.01]	Anxiety [0.73]	Epilepsy [1.35]	Back Pain [0.69]	Depression [0.72]	Self Harm [0.69]
Guanajuato	Violence [3.83]	Road Inj [0.91]	Headaches [0.98]	Neonatal [1.29]	Conduct [1.01]	Self Harm [1.11]	Epilepsy [1.41]	Anxiety [0.74]	Depression [0.77]	Back Pain [0.69]
Guerrero	Violence [7.57]	Road Inj [0.73]	Headaches [1.0]	Neonatal [1.53]	Conduct [1.01]	Epilepsy [1.23]	Anxiety [0.77]	Congenital [0.83]	Back Pain [0.71]	Depression [0.71]
Hidalgo	Violence [3.28]	Road Inj [0.84]	Headaches [0.99]	Neonatal [1.78]	Conduct [1.01]	Epilepsy [1.44]	Anxiety [0.76]	Self Harm [0.74]	Back Pain [0.7]	Congenital [0.8]
Jalisco	Violence [4.67]	Road Inj [0.9]	Headaches [0.97]	Neonatal [1.35]	Conduct [1.01]	Self Harm [1.06]	Epilepsy [1.64]	Anxiety [0.71]	Congenital [0.91]	Depression [0.76]
Mexico	Violence [4.72]	Headaches [0.93]	Neonatal [1.7]	Road Inj [0.57]	Conduct [1.01]	Anxiety [0.72]	Epilepsy [1.4]	Self Harm [0.76]	Congenital [0.82]	Depression [0.71]
Michoacan	Violence [4.45]	Road Inj [0.97]	Headaches [1.0]	Neonatal [1.23]	Conduct [1.01]	Self Harm [0.87]	Epilepsy [1.28]	Anxiety [0.76]	Congenital [0.86]	Back Pain [0.71]
Morelos	Violence [5.65]	Headaches [0.98]	Road Inj [0.6]	Neonatal [1.39]	Conduct [1.01]	Epilepsy [1.5]	Congenital [0.94]	Anxiety [0.72]	Self Harm [0.7]	Depression [0.7]
Nayarit	Violence [4.13]	Road Inj [0.9]	Headaches [0.98]	Neonatal [1.52]	Conduct [1.01]	Anxiety [0.73]	Epilepsy [1.35]	Back Pain [0.68]	Depression [0.7]	Congenital [0.79]
Nuevo Leon	Violence [4.91]	Headaches [0.96]	Road Inj [0.49]	Conduct [1.02]	Neonatal [1.14]	Anxiety [0.69]	Self Harm [0.73]	Epilepsy [1.45]	Back Pain [0.66]	Depression [0.71]
Oaxaca	Violence [3.9]	Headaches [1.01]	Road Inj [0.62]	Neonatal [1.45]	Conduct [1.01]	Epilepsy [1.45]	Congenital [0.89]	Anxiety [0.77]	Back Pain [0.71]	Depression [0.7]
Puebla	Violence [3.39]	Road Inj [0.83]	Headaches [1.0]	Neonatal [1.35]	Conduct [1.01]	Self Harm [0.87]	Congenital [0.91]	Anxiety [0.76]	Back Pain [0.71]	Epilepsy [1.19]
Queretaro	Violence [3.49]	Road Inj [0.74]	Headaches [0.97]	Neonatal [1.49]	Conduct [1.01]	Epilepsy [1.44]	Anxiety [0.72]	Back Pain [0.67]	Depression [0.73]	Congenital [0.81]
Quintana Roo	Violence [3.86]	Neonatal [1.81]	Headaches [0.98]	Road Inj [0.6]	Self Harm [1.17]	Conduct [1.01]	Depression [0.84]	Anxiety [0.73]	Epilepsy [1.35]	Back Pain [0.66]
San Luis Potosi	Violence [4.02]	Road Inj [0.88]	Headaches [0.98]	Neonatal [1.23]	Conduct [1.01]	Self Harm [1.13]	Epilepsy [1.55]	Congenital [0.95]	Anxiety [0.74]	Depression [0.74]
Sinaloa	Violence [5.82]	Road Inj [0.85]	Headaches [0.97]	Neonatal [1.28]	Conduct [1.01]	Anxiety [0.71]	Epilepsy [1.37]	Back Pain [0.66]	Depression [0.68]	Congenital [0.7]
Sonora	Violence [4.36]	Road Inj [0.74]	Headaches [0.97]	Neonatal [1.24]	Conduct [1.01]	Epilepsy [1.47]	Anxiety [0.71]	Self Harm [0.76]	Back Pain [0.67]	Depression [0.72]
Tabasco	Violence [3.72]	Road Inj [1.14]	Headaches [0.98]	Neonatal [1.55]	Self Harm [1.4]	Conduct [1.01]	Drown [0.98]	Congenital [0.96]	Epilepsy [1.42]	Depression [0.81]
Tamaulipas	Violence [4.89]	Headaches [0.97]	Neonatal [1.59]	Road Inj [0.55]	Conduct [1.01]	Anxiety [0.71]	Back Pain [0.67]	Epilepsy [1.32]	Depression [0.68]	Congenital [0.72]
Tlaxcala	Violence [3.15]	Road Inj [0.8]	Headaches [0.99]	Neonatal [1.53]	Conduct [1.01]	Epilepsy [1.49]	Anxiety [0.75]	Congenital [0.87]	Back Pain [0.7]	CKD [2.75]

eFigure 7e. Leading ten causes of DALYs with the ratio of observed DALYs to DALYs expected on the basis of Socio-Demographic Index alone in 2017, 10-19 years, both sexes combined. The top ten causes contributing to DALYs are listed globally, by socio-demographic quintile, and then by GBD superregion, region, country, and subnationally where modeled. For each cell, the ratio of observed DALYs to DALYs expected on the basis of socio-demographic index (SDI) alone are listed. Abbreviations: DALY=disability-adjusted life year, GBD=Global Burden of Disease.

Values shown in brackets represent the ratio of observed DALYs to predicted DALYs on the basis of Socio-Demographic Index (SDI), rounded to two (2) digits. Color ranges (shown below) were calculated to place a roughly equal number of cells into each bin.

COLOR KEY:	[0.0-0.73]	(0.73-0.88]	(0.88-0.95]	(0.95-1.01]	(1.01-1.09]	(1.09-1.26]	(1.26-1.55]	(1.55-2.09]	2.09+

	1	2	3	4	5	6	7	8	9	10
Veracruz	Violence [3.82]	Headaches [0.99]	Neonatal [1.53]	Road Inj [0.55]	Conduct [1.01]	Epilepsy [1.46]	Congenital [0.9]	Anxiety [0.76]	Depression [0.75]	Self Harm [0.72]
Yucatan	Violence [3.13]	Headaches [0.98]	Neonatal [1.32]	Road Inj [0.51]	Conduct [1.01]	Self Harm [1.06]	Depression [0.82]	Epilepsy [1.42]	Anxiety [0.73]	Congenital [0.85]
Zacatecas	Violence [4.16]	Road Inj [1.23]	Headaches [0.98]	Conduct [1.01]	Neonatal [0.98]	Epilepsy [1.48]	Anxiety [0.74]	Back Pain [0.7]	Self Harm [0.69]	Depression [0.69]
Nicaragua	Headaches [1.04]	Neonatal [1.51]	Conduct [0.95]	Back Pain [0.95]	Road Inj [0.37]	Self Harm [0.85]	Depression [0.88]	Violence [0.73]	Anxiety [0.73]	Epilepsy [0.95]
Panama	Violence [3.94]	Headaches [0.99]	Neonatal [1.28]	Road Inj [0.51]	Conduct [0.96]	Back Pain [0.82]	Dermatitis [1.31]	Depression [0.74]	Anxiety [0.65]	Asthma [1.19]
Venezuela	Violence [10.66]	Road Inj [1.22]	Headaches [0.98]	Neonatal [1.4]	Self Harm [1.13]	Conduct [0.96]	Back Pain [0.75]	Depression [0.79]	Congenital [0.9]	Mech [2.34]
**Tropical Latin America**	Violence [7.16]	Headaches [1.43]	Road Inj [0.95]	Back Pain [2.06]	Anxiety [1.22]	Depression [1.23]	Conduct [0.94]	Iron [1.63]	Neonatal [0.93]	Bipolar [1.73]
Brazil	Violence [7.54]	Headaches [1.43]	Back Pain [2.08]	Road Inj [0.95]	Anxiety [1.21]	Depression [1.23]	Conduct [0.94]	Iron [1.69]	Neonatal [0.94]	Bipolar [1.72]
Acre	Violence [5.94]	Headaches [1.45]	Back Pain [2.2]	Road Inj [0.86]	Iron [1.75]	Anxiety [1.29]	Depression [1.23]	Conduct [0.95]	Drown [0.99]	Neonatal [1.01]
Alagoas	Violence [11.74]	Headaches [1.49]	Road Inj [0.96]	Back Pain [2.22]	Iron [1.33]	Depression [1.4]	Anxiety [1.33]	Conduct [0.95]	Neonatal [0.86]	Drown [0.63]
Amapa	Violence [11.13]	Headaches [1.43]	Back Pain [2.01]	Road Inj [0.71]	Drown [1.59]	Anxiety [1.22]	Iron [2.0]	Depression [1.23]	Conduct [0.95]	Neonatal [0.99]
Amazonas	Violence [6.78]	Headaches [1.44]	Back Pain [2.12]	Road Inj [0.57]	Anxiety [1.25]	Iron [1.74]	Depression [1.13]	Conduct [0.95]	Drown [0.94]	Self Harm [0.88]
Bahia	Violence [5.06]	Headaches [1.46]	Back Pain [2.18]	Iron [1.63]	Road Inj [0.56]	Anxiety [1.31]	Depression [1.11]	Conduct [0.86]	Neonatal [0.93]	Bipolar [1.8]
Ceara	Violence [8.72]	Road Inj [1.11]	Headaches [1.46]	Back Pain [2.17]	Iron [1.63]	Anxiety [1.3]	Depression [1.3]	Conduct [0.94]	Neonatal [0.88]	Self Harm [0.7]
Distrito Federal	Violence [16.95]	Headaches [1.42]	Back Pain [1.9]	Road Inj [0.81]	Anxiety [1.07]	Depression [1.17]	Conduct [0.99]	Neonatal [1.02]	Asthma [1.23]	Congenital [0.94]
Espirito Santo	Violence [12.72]	Headaches [1.43]	Road Inj [0.99]	Back Pain [2.0]	Iron [2.54]	Anxiety [1.2]	Depression [1.26]	Conduct [0.95]	Neonatal [0.91]	Asthma [1.38]
Goias	Violence [10.61]	Road Inj [1.22]	Headaches [1.44]	Back Pain [2.11]	Anxiety [1.24]	Depression [1.28]	Conduct [0.95]	Iron [1.35]	Neonatal [0.85]	Asthma [1.19]
Maranhao	Headaches [1.5]	Back Pain [2.25]	Iron [1.55]	Violence [1.87]	Road Inj [0.61]	Anxiety [1.33]	Depression [1.19]	Conduct [0.95]	Neonatal [0.97]	Bipolar [1.85]
Mato Grosso	Violence [7.11]	Road Inj [1.45]	Headaches [1.43]	Back Pain [2.08]	Anxiety [1.22]	Depression [1.18]	Conduct [0.95]	Iron [1.38]	Neonatal [0.89]	Drown [0.98]
Mato Grosso do Sul	Violence [5.04]	Road Inj [1.1]	Headaches [1.44]	Back Pain [2.12]	Anxiety [1.23]	Depression [1.32]	Conduct [0.95]	Self Harm [1.06]	Neonatal [1.03]	Iron [1.26]
Minas Gerais	Violence [6.65]	Headaches [1.42]	Road Inj [0.97]	Back Pain [2.1]	Anxiety [1.23]	Depression [1.21]	Conduct [0.95]	Neonatal [0.99]	Iron [1.46]	Drown [0.84]
Para	Violence [5.13]	Headaches [1.47]	Back Pain [2.17]	Road Inj [0.69]	Anxiety [1.32]	Conduct [0.95]	Depression [1.04]	Iron [1.03]	Neonatal [0.9]	Bipolar [1.8]
Paraiba	Violence [7.51]	Headaches [1.47]	Road Inj [1.07]	Back Pain [2.2]	Anxiety [1.32]	Iron [1.34]	Depression [1.24]	Conduct [0.95]	Neonatal [0.83]	Congenital [0.78]
Parana	Violence [7.76]	Road Inj [1.33]	Headaches [1.43]	Back Pain [2.08]	Anxiety [1.2]	Depression [1.24]	Conduct [0.95]	Neonatal [0.96]	Iron [1.63]	Asthma [1.24]
Pernambuco	Violence [9.87]	Headaches [1.46]	Road Inj [1.0]	Back Pain [2.17]	Depression [1.41]	Iron [1.54]	Anxiety [1.31]	Conduct [0.95]	Neonatal [0.87]	Congenital [0.81]
Piaui	Road Inj [1.39]	Violence [3.36]	Headaches [1.49]	Back Pain [2.26]	Anxiety [1.34]	Depression [1.17]	Conduct [0.95]	Neonatal [1.0]	Drown [0.7]	Self Harm [0.78]
Rio de Janeiro	Violence [15.09]	Road Inj [1.15]	Headaches [1.43]	Back Pain [1.77]	Anxiety [1.16]	Depression [1.14]	Neonatal [1.15]	Conduct [0.96]	Iron [1.47]	Congenital [0.84]
Rio Grande do Norte	Violence [11.99]	Headaches [1.46]	Road Inj [1.03]	Back Pain [2.16]	Anxiety [1.3]	Iron [1.58]	Depression [1.28]	Conduct [0.95]	Self Harm [0.82]	Neonatal [0.8]
Rio Grande do Sul	Violence [8.11]	Back Pain [2.7]	Headaches [1.43]	Road Inj [0.96]	Anxiety [1.17]	Depression [1.29]	Conduct [0.88]	Congenital [1.2]	Neonatal [0.96]	Self Harm [0.86]
Rondonia	Violence [5.84]	Road Inj [1.38]	Headaches [1.45]	Back Pain [2.16]	Anxiety [1.27]	Depression [1.25]	Conduct [0.95]	Drown [1.04]	Neonatal [1.05]	Congenital [1.13]
Roraima	Violence [5.38]	Road Inj [1.36]	Headaches [1.43]	Back Pain [2.08]	Iron [2.08]	Depression [1.41]	Anxiety [1.23]	Drown [1.33]	Conduct [0.95]	Self Harm [1.05]

eFigure 7e. Leading ten causes of DALYs with the ratio of observed DALYs to DALYs expected on the basis of Socio-Demographic Index alone in 2017, 10-19 years, both sexes combined. The top ten causes contributing to DALYs are listed globally, by socio-demographic quintile, and then by GBD superregion, region, country, and subnationally where modeled. For each cell, the ratio of observed DALYs to DALYs expected on the basis of socio-demographic index (SDI) alone are listed. Abbreviations: DALY=disability-adjusted life year, GBD=Global Burden of Disease.

Values shown in brackets represent the ratio of observed DALYs to predicted DALYs on the basis of Socio-Demographic Index (SDI), rounded to two (2) digits. Color ranges (shown below) were calculated to place a roughly equal number of cells into each bin.

COLOR KEY:	[0.0-0.73]	(0.73-0.88]	(0.88-0.95]	(0.95-1.01]	(1.01-1.09]	(1.09-1.26]	(1.26-1.55]	(1.55-2.09]	2.09+

	1	2	3	4	5	6	7	8	9	10
Santa Catarina	Road Inj [1.39]	Headaches [1.7]	Violence [3.87]	Back Pain [2.0]	Depression [1.37]	Anxiety [1.17]	Conduct [0.96]	Iron [1.74]	Neonatal [0.87]	Asthma [1.36]
Sao Paulo	Headaches [1.36]	Violence [4.7]	Back Pain [2.0]	Road Inj [0.79]	Depression [1.3]	Anxiety [1.13]	Conduct [0.97]	Neonatal [0.97]	Iron [1.8]	Bipolar [1.71]
Sergipe	Violence [11.37]	Road Inj [1.31]	Headaches [1.45]	Back Pain [2.12]	Iron [1.86]	Anxiety [1.28]	Depression [1.23]	Conduct [0.95]	Drown [0.97]	Neonatal [1.03]
Tocantins	Violence [5.29]	Road Inj [1.47]	Headaches [1.45]	Back Pain [2.17]	Anxiety [1.28]	Iron [1.58]	Depression [1.3]	Conduct [0.95]	Neonatal [1.01]	Drown [0.8]
Paraguay	Road Inj [1.1]	Headaches [1.44]	Back Pain [1.41]	Violence [1.69]	Anxiety [1.3]	Depression [1.06]	Conduct [0.9]	Self Harm [0.99]	Iron [1.09]	Asthma [1.43]
**North Africa and Middle East**	Conflict Terror [1294.15]	Road Inj [1.04]	Headaches [1.02]	Back Pain [1.24]	Anxiety [1.15]	Depression [1.17]	Conduct [0.94]	Iron [1.42]	Neonatal [0.96]	Congenital [1.0]
Afghanistan	Road Inj [1.03]	Headaches [1.19]	Conflict Terror [22.45]	Back Pain [1.56]	Anxiety [1.31]	Violence [0.93]	Depression [1.17]	Meningitis [0.6]	Conduct [0.95]	LRI [0.56]
Algeria	Road Inj [0.9]	Headaches [0.99]	Back Pain [1.13]	Anxiety [1.1]	Depression [1.11]	Conduct [0.95]	Neonatal [1.07]	Congenital [1.05]	Asthma [0.88]	Dermatitis [0.87]
Bahrain	Headaches [0.98]	Back Pain [1.11]	Anxiety [1.08]	Depression [1.18]	Road Inj [0.5]	Conduct [0.95]	Neonatal [1.05]	Dermatitis [1.01]	Hemog [3.94]	Iron [1.06]
Egypt	Road Inj [1.25]	Headaches [1.08]	Back Pain [1.4]	Iron [1.39]	Anxiety [1.08]	Congenital [1.24]	Conduct [0.94]	LRI [1.59]	Neonatal [1.04]	Depression [1.0]
Iran	Road Inj [1.52]	Headaches [1.38]	Anxiety [1.45]	Depression [1.42]	Back Pain [1.09]	Neonatal [1.21]	Conduct [1.02]	Congenital [1.12]	Self Harm [0.72]	Drugs [1.76]
Iraq	Conflict Terror [3082.11]	Headaches [1.01]	Back Pain [1.24]	Anxiety [1.16]	Neonatal [1.11]	Conduct [0.92]	Road Inj [0.43]	Depression [1.06]	Congenital [1.0]	Violence [0.88]
Jordan	Headaches [0.97]	Neonatal [1.38]	Back Pain [1.13]	Anxiety [1.09]	Road Inj [0.51]	Depression [1.1]	Conduct [0.96]	Iron [1.55]	Congenital [0.97]	Asthma [0.98]
Kuwait	Road Inj [0.84]	Headaches [0.81]	Back Pain [1.05]	Anxiety [0.99]	Neonatal [1.15]	Depression [1.06]	Conduct [0.99]	Congenital [0.95]	Dermatitis [0.88]	Asthma [0.9]
Lebanon	Headaches [0.98]	Anxiety [1.28]	Depression [1.05]	Neonatal [1.04]	Conduct [0.92]	Back Pain [0.84]	Road Inj [0.38]	Congenital [0.88]	Conflict Terror [469.59]	Dermatitis [1.05]
Libya	Conflict Terror [3299.91]	Road Inj [1.13]	Headaches [0.98]	Back Pain [1.1]	Anxiety [1.02]	Depression [1.11]	Conduct [0.97]	Neonatal [1.01]	Congenital [1.25]	Oth Trans [6.46]
Morocco	Road Inj [1.04]	Headaches [1.02]	Depression [1.72]	Back Pain [1.46]	Anxiety [1.24]	Conduct [0.94]	Neonatal [1.06]	Congenital [1.06]	Iron [0.84]	Self Harm [0.67]
Palestine	Headaches [1.03]	Depression [1.66]	Back Pain [1.27]	Anxiety [1.25]	Conduct [0.94]	Neonatal [1.16]	Conflict Terror [194.05]	Road Inj [0.36]	Iron [0.65]	Violence [0.71]
Oman	Road Inj [1.66]	Headaches [0.97]	Back Pain [1.08]	Anxiety [1.04]	Depression [1.09]	Conduct [0.97]	Neonatal [1.07]	Congenital [0.73]	Dermatitis [0.77]	Iron [1.24]
Qatar	Road Inj [1.23]	Headaches [0.95]	Back Pain [1.12]	Anxiety [1.0]	Depression [1.07]	Conduct [1.0]	Neonatal [0.95]	Congenital [0.95]	Self Harm [0.61]	Dermatitis [0.98]
Saudi Arabia	Road Inj [1.16]	Headaches [1.03]	Back Pain [1.11]	Anxiety [1.01]	Depression [1.11]	Conduct [0.98]	Conflict Terror [1053.22]	Congenital [1.06]	Neonatal [0.72]	Dermatitis [0.97]
Sudan	Road Inj [1.14]	Iron [1.32]	Headaches [1.04]	Back Pain [1.39]	Anxiety [1.25]	Depression [1.13]	Conduct [0.94]	Congenital [0.9]	Malaria [19.83]	Diarrhea [0.83]
Syria	Conflict Terror [11074.46]	Headaches [0.98]	Back Pain [1.2]	Anxiety [1.18]	Road Inj [0.48]	Depression [1.11]	Conduct [0.94]	Iron [1.06]	Neonatal [0.9]	Congenital [0.79]
Tunisia	Road Inj [0.94]	Headaches [0.99]	Anxiety [1.13]	Depression [1.15]	Back Pain [1.04]	Conduct [0.95]	Neonatal [1.04]	Dermatitis [1.03]	Congenital [0.69]	Mech [1.68]
Turkey	Headaches [0.8]	Road Inj [0.63]	Back Pain [1.26]	Depression [1.11]	Conduct [0.94]	Anxiety [0.83]	Neonatal [0.74]	Congenital [0.78]	Conflict Terror [399.16]	Epilepsy [1.11]
UAE	Road Inj [1.98]	Headaches [0.97]	Back Pain [1.02]	Anxiety [0.96]	Conduct [0.96]	Neonatal [1.01]	Congenital [1.33]	Depression [0.85]	Drugs [1.84]	Asthma [1.13]

eFigure 7e. Leading ten causes of DALYs with the ratio of observed DALYs to DALYs expected on the basis of Socio-Demographic Index alone in 2017, 10-19 years, both sexes combined. The top ten causes contributing to DALYs are listed globally, by socio-demographic quintile, and then by GBD superregion, region, country, and subnationally where modeled. For each cell, the ratio of observed DALYs to DALYs expected on the basis of socio-demographic index (SDI) alone are listed. Abbreviations: DALY=disability-adjusted life year, GBD=Global Burden of Disease.

Values shown in brackets represent the ratio of observed DALYs to predicted DALYs on the basis of Socio-Demographic Index (SDI), rounded to two (2) digits. Color ranges (shown below) were calculated to place a roughly equal number of cells into each bin.

COLOR KEY:	[0.0-0.73]	(0.73-0.88]	(0.88-0.95]	(0.95-1.01]	(1.01-1.09]	(1.09-1.26]	(1.26-1.55]	(1.55-2.09]	2.09+

	1	2	3	4	5	6	7	8	9	10
Yemen	Conflict Terror [614.02]	Iron [2.95]	Road Inj [1.33]	Headaches [1.05]	Back Pain [1.3]	Anxiety [1.24]	Depression [1.16]	Conduct [0.92]	Neonatal [1.28]	Hemog [1.8]
**South Asia**	Iron [1.94]	Headaches [0.97]	Typh + Paratyph [32.66]	Neonatal [1.51]	Self Harm [1.24]	Road Inj [0.5]	Diarrhea [2.04]	Conduct [0.86]	Anxiety [0.84]	Congenital [0.8]
Bangladesh	Typh + Paratyph [12.21]	Headaches [1.07]	Iron [0.85]	Drown [0.98]	Neonatal [1.56]	Self Harm [1.05]	Anxiety [1.05]	Road Inj [0.38]	Depression [1.0]	Conduct [0.8]
Bhutan	Iron [2.02]	Headaches [1.01]	Neonatal [1.31]	Road Inj [0.4]	Conduct [0.81]	Anxiety [0.92]	Diarrhea [1.94]	Back Pain [0.83]	Mech [2.15]	Depression [0.77]
India	Iron [2.34]	Headaches [0.97]	Typh + Paratyph [45.11]	Neonatal [1.54]	Self Harm [1.37]	Diarrhea [2.29]	Conduct [0.87]	Road Inj [0.38]	Anxiety [0.8]	Congenital [0.8]
Andhra Pradesh	Iron [1.84]	Headaches [0.99]	Neonatal [1.72]	Self Harm [1.42]	Typh + Paratyph [26.69]	Conduct [0.86]	Depression [0.94]	Anxiety [0.82]	Diarrhea [1.35]	Road Inj [0.28]
Arunachal Pradesh	Iron [2.19]	Headaches [0.98]	Neonatal [1.62]	Self Harm [1.13]	Conduct [0.87]	Depression [0.88]	Diarrhea [1.66]	Typh + Paratyph [28.38]	Anxiety [0.81]	Road Inj [0.31]
Assam	Iron [2.93]	Headaches [0.98]	Self Harm [1.29]	Neonatal [1.45]	Diarrhea [2.14]	Typh + Paratyph [25.39]	Conduct [0.86]	Malaria [101.02]	Drown [0.73]	Hep [8.88]
Bihar	Iron [1.62]	Diarrhea [2.07]	Headaches [1.01]	Neonatal [1.82]	Typh + Paratyph [4.83]	Conduct [0.88]	Vit A [2.06]	Road Inj [0.31]	Anxiety [0.81]	ID [6.78]
Chhattisgarh	Iron [2.61]	Typh + Paratyph [25.67]	Headaches [0.99]	Neonatal [1.61]	Self Harm [1.28]	Diarrhea [1.64]	Conduct [0.86]	Road Inj [0.38]	Vit A [3.22]	Malaria [49.27]
Delhi	Iron [7.64]	Headaches [0.92]	Neonatal [1.47]	Typh + Paratyph [1757.38]	Conduct [0.89]	Anxiety [0.68]	Road Inj [0.34]	Diarrhea [3.83]	Self Harm [0.69]	Depression [0.62]
Goa	Neonatal [1.82]	Headaches [0.93]	Conduct [0.9]	Self Harm [0.92]	Road Inj [0.35]	Anxiety [0.63]	Depression [0.68]	Congenital [0.87]	Epilepsy [1.38]	Diarrhea [3.21]
Gujarat	Iron [4.26]	Headaches [0.95]	Neonatal [1.55]	Self Harm [1.27]	Conduct [0.87]	Typh + Paratyph [62.94]	Anxiety [0.76]	Diarrhea [1.83]	Vit A [3.97]	Congenital [0.75]
Haryana	Iron [4.26]	Typh + Paratyph [170.73]	Headaches [0.94]	Neonatal [1.55]	Road Inj [0.48]	Self Harm [1.14]	Conduct [0.88]	Diarrhea [2.19]	Depression [0.84]	Anxiety [0.78]
Himachal Pradesh	Iron [4.26]	Headaches [0.94]	Neonatal [1.69]	Road Inj [0.45]	Self Harm [1.05]	Typh + Paratyph [201.42]	Conduct [0.87]	Anxiety [0.75]	Congenital [0.84]	Diarrhea [2.38]
Jammu & Kashmir	Headaches [0.96]	Neonatal [1.52]	Iron [1.43]	Road Inj [0.51]	Conduct [0.88]	Self Harm [0.86]	Diarrhea [2.05]	Anxiety [0.79]	Congenital [0.9]	Depression [0.67]
Jharkhand	Iron [1.89]	Diarrhea [2.12]	Headaches [1.0]	Road Inj [0.57]	Neonatal [1.74]	Typh + Paratyph [9.87]	Conduct [0.95]	Drown [0.68]	Anxiety [0.85]	Congenital [0.86]
Karnataka	Self Harm [3.15]	Neonatal [1.82]	Headaches [0.95]	Iron [1.35]	Road Inj [0.44]	Congenital [1.18]	Diarrhea [2.34]	Drown [0.83]	Conduct [0.74]	Anxiety [0.81]
Kerala	Neonatal [2.08]	Headaches [0.95]	Self Harm [1.17]	Conduct [0.85]	Anxiety [0.82]	Congenital [0.89]	Depression [0.71]	Iron [1.0]	Road Inj [0.26]	Back Pain [0.53]
Madhya Pradesh	Iron [2.54]	Typh + Paratyph [20.59]	Self Harm [1.68]	Headaches [1.0]	Diarrhea [1.62]	Neonatal [1.54]	Road Inj [0.44]	Conduct [0.86]	Drown [0.67]	Vit A [2.52]
Maharashtra	Iron [3.1]	Headaches [0.95]	Neonatal [1.66]	Conduct [0.92]	Self Harm [1.01]	Typh + Paratyph [117.69]	Anxiety [0.77]	Depression [0.76]	Diarrhea [2.11]	Congenital [0.78]
Manipur	Headaches [0.97]	Neonatal [1.73]	Typh + Paratyph [105.88]	Road Inj [0.45]	Conduct [0.87]	Anxiety [0.89]	Self Harm [0.83]	HIV [17.0]	Depression [0.84]	Diarrhea [1.99]

eFigure 7e. Leading ten causes of DALYs with the ratio of observed DALYs to DALYs expected on the basis of Socio-Demographic Index alone in 2017, 10-19 years, both sexes combined. The top ten causes contributing to DALYs are listed globally, by socio-demographic quintile, and then by GBD superregion, region, country, and subnationally where modeled. For each cell, the ratio of observed DALYs to DALYs expected on the basis of socio-demographic index (SDI) alone are listed. Abbreviations: DALY=disability-adjusted life year, GBD=Global Burden of Disease.

Values shown in brackets represent the ratio of observed DALYs to predicted DALYs on the basis of Socio-Demographic Index (SDI), rounded to two (2) digits. Color ranges (shown below) were calculated to place a roughly equal number of cells into each bin.

COLOR KEY:		[0.0-0.73]	(0.73-0.88]	(0.88-0.95]	(0.95-1.01]	(1.01-1.09]	(1.09-1.26]	(1.26-1.55]	(1.55-2.09]	2.09+
	**1**	**2**	**3**	**4**	**5**	**6**	**7**	**8**	**9**	**10**
Meghalaya	Iron [2.47]	Typh + Paratyph [81.31]	Headaches [0.97]	Neonatal [1.68]	Malaria [302.03]	Conduct [0.87]	Diarrhea [1.85]	Depression [0.87]	Anxiety [0.8]	Congenital [0.77]
Mizoram	Typh + Paratyph [306.02]	Headaches [0.96]	Neonatal [1.7]	Conduct [0.88]	Malaria [478.44]	Anxiety [0.78]	Road Inj [0.34]	Diarrhea [2.21]	Iron [0.87]	HIV [15.42]
Nagaland	Iron [3.29]	Neonatal [1.76]	Headaches [0.94]	Typh + Paratyph [315.74]	Conduct [0.87]	HIV [25.88]	Malaria [869.1]	Anxiety [0.76]	Road Inj [0.3]	Depression [0.71]
Odisha	Diarrhea [2.8]	Headaches [0.99]	Malaria [143.72]	Self Harm [1.42]	Iron [1.23]	Typh + Paratyph [25.03]	Neonatal [1.51]	Conduct [0.86]	Road Inj [0.37]	Depression [0.89]
Punjab	Iron [3.72]	Typh + Paratyph [238.77]	Headaches [0.93]	Neonatal [1.6]	Road Inj [0.5]	Conduct [0.88]	Diarrhea [2.51]	Self Harm [0.8]	Congenital [0.89]	Anxiety [0.73]
Rajasthan	Typh + Paratyph [23.61]	Iron [1.59]	Headaches [0.98]	Neonatal [1.5]	Conduct [0.87]	Diarrhea [1.14]	Road Inj [0.32]	Anxiety [0.84]	Self Harm [0.71]	Vit A [2.3]
Sikkim	Iron [2.85]	Headaches [0.96]	Neonatal [1.73]	Typh + Paratyph [242.85]	Conduct [0.86]	Self Harm [0.9]	Anxiety [0.77]	Road Inj [0.32]	Diarrhea [2.44]	Depression [0.72]
Tamil Nadu	Self Harm [3.39]	Headaches [0.96]	Neonatal [1.62]	Iron [1.69]	Road Inj [0.55]	Conduct [0.87]	Depression [0.95]	Congenital [1.07]	Diarrhea [2.57]	Anxiety [0.77]
Telangana	Iron [2.8]	Headaches [0.98]	Neonatal [1.68]	Self Harm [1.54]	Typh + Paratyph [59.33]	Conduct [0.86]	Depression [0.92]	Diarrhea [2.01]	Anxiety [0.81]	Road Inj [0.33]
Tripura	Self Harm [1.97]	Iron [1.61]	Headaches [0.98]	Neonatal [1.65]	Conduct [0.87]	Malaria [124.77]	Road Inj [0.34]	Diarrhea [1.52]	Anxiety [0.81]	Congenital [0.8]
Uttar Pradesh	Iron [2.12]	Typh + Paratyph [20.65]	Headaches [1.0]	Self Harm [1.28]	Diarrhea [1.54]	Neonatal [1.46]	Conduct [0.87]	Road Inj [0.38]	Anxiety [0.8]	Vit A [2.37]
Uttarakhand	Iron [3.4]	Typh + Paratyph [194.57]	Headaches [0.96]	Road Inj [0.7]	Neonatal [1.69]	Conduct [0.87]	Self Harm [0.82]	Anxiety [0.78]	Diarrhea [2.18]	Congenital [0.77]
W Bengal	Self Harm [2.02]	Headaches [0.99]	Iron [1.33]	Neonatal [1.5]	Conduct [0.86]	Diarrhea [1.63]	Road Inj [0.36]	Anxiety [0.83]	Congenital [0.91]	Vit A [2.96]
UTs other than Delhi	Iron [3.18]	Neonatal [1.81]	Headaches [0.94]	Self Harm [1.26]	Conduct [0.88]	Road Inj [0.39]	Anxiety [0.74]	Depression [0.77]	Congenital [0.75]	Back Pain [0.54]
Nepal	Headaches [1.34]	Road Inj [0.66]	Neonatal [1.87]	Typh + Paratyph [4.12]	Back Pain [1.1]	Iron [0.61]	Self Harm [0.89]	Anxiety [0.95]	Conduct [0.81]	Diarrhea [0.75]
Pakistan	Road Inj [1.18]	Headaches [0.89]	Diarrhea [1.73]	Neonatal [1.38]	Iron [0.84]	TB [2.66]	Drown [0.79]	Typh + Paratyph [8.85]	Conduct [0.82]	Anxiety [0.93]
**Southeast Asia, East Asia, and Oceania**	Road Inj [0.76]	Headaches [0.61]	Neonatal [1.08]	Drown [1.33]	Conduct [0.82]	Anxiety [0.72]	Back Pain [0.67]	Congenital [0.85]	Depression [0.65]	Leukemia [0.91]
**East Asia**	Road Inj [0.6]	Neonatal [1.36]	Drown [1.83]	Anxiety [0.73]	Headaches [0.45]	Conduct [0.73]	Back Pain [0.63]	Depression [0.59]	Congenital [0.73]	Leukemia [0.95]
China	Neonatal [1.39]	Road Inj [0.58]	Drown [1.77]	Anxiety [0.73]	Conduct [0.73]	Headaches [0.44]	Back Pain [0.62]	Depression [0.59]	Congenital [0.73]	Leukemia [0.94]
N Korea	Road Inj [0.99]	Drown [1.47]	Headaches [0.58]	Self Harm [0.95]	Back Pain [0.94]	Anxiety [0.92]	Iron [0.74]	Conduct [0.72]	Congenital [0.88]	Neonatal [0.72]
Taiwan	Road Inj [0.95]	Headaches [0.7]	Back Pain [0.85]	Anxiety [0.71]	Conduct [0.86]	Depression [0.53]	Neonatal [0.55]	Congenital [0.71]	Self Harm [0.43]	Drugs [0.88]
**Oceania**	Self Harm [2.73]	Road Inj [0.96]	Drown [1.46]	LRI [1.46]	Iron [1.02]	RHD [4.35]	Diarrhea [1.47]	Asthma [2.08]	Headaches [0.73]	Conduct [0.87]
Am Samoa	Headaches [0.68]	Conduct [0.88]	Drown [1.41]	Iron [1.79]	Self Harm [0.87]	Anxiety [0.78]	Asthma [1.5]	Back Pain [0.75]	Road Inj [0.35]	Depression [0.76]
Micronesia	Self Harm [2.17]	Road Inj [0.64]	Iron [1.44]	Headaches [0.7]	Drown [0.95]	Conduct [0.87]	Anxiety [0.88]	Asthma [1.47]	Depression [0.87]	Back Pain [0.82]
Fiji	Self Harm [1.43]	Headaches [0.69]	Drown [1.18]	Road Inj [0.45]	Iron [1.45]	Conduct [0.87]	Asthma [1.71]	LRI [1.77]	Anxiety [0.83]	Congenital [1.03]

eFigure 7e. Leading ten causes of DALYs with the ratio of observed DALYs to DALYs expected on the basis of Socio-Demographic Index alone in 2017, 10-19 years, both sexes combined. The top ten causes contributing to DALYs are listed globally, by socio-demographic quintile, and then by GBD superregion, region, country, and subnationally where modeled. For each cell, the ratio of observed DALYs to DALYs expected on the basis of socio-demographic index (SDI) alone are listed. Abbreviations: DALY=disability-adjusted life year, GBD=Global Burden of Disease.

Values shown in brackets represent the ratio of observed DALYs to predicted DALYs on the basis of Socio-Demographic Index (SDI), rounded to two (2) digits. Color ranges (shown below) were calculated to place a roughly equal number of cells into each bin.

COLOR KEY:	[0.0-0.73]	(0.73-0.88]	(0.88-0.95]	(0.95-1.01]	(1.01-1.09]	(1.09-1.26]	(1.26-1.55]	(1.55-2.09]	2.09+

	1	2	3	4	5	6	7	8	9	10
Guam	Self Harm [2.98]	Headaches [0.68]	Road Inj [0.57]	Depression [0.97]	Conduct [0.91]	Diarrhea [6.68]	Anxiety [0.71]	Back Pain [0.68]	Asthma [1.33]	Neonatal [0.74]
Kiribati	Self Harm [3.37]	Iron [1.08]	TB [2.4]	Meningitis [1.64]	Congenital [1.38]	Asthma [1.9]	Diarrhea [0.99]	Headaches [0.76]	LRI [0.78]	Road Inj [0.38]
Marshall	Self Harm [2.7]	Road Inj [0.84]	Drown [1.16]	Iron [1.2]	Headaches [0.71]	Conduct [0.87]	Congenital [1.08]	LRI [1.12]	RHD [4.06]	Anxiety [0.89]
N Mariana	Self Harm [1.7]	Headaches [0.71]	Road Inj [0.47]	Drown [2.07]	Conduct [0.87]	Anxiety [0.74]	Depression [0.87]	Back Pain [0.73]	Neonatal [0.87]	Asthma [1.38]
PNG	Self Harm [2.9]	Road Inj [1.01]	Drown [1.61]	LRI [1.36]	RHD [4.4]	Diarrhea [1.2]	Iron [0.88]	Asthma [2.16]	Headaches [0.76]	Maternal [1.75]
Samoa	Self Harm [1.53]	Headaches [0.7]	Iron [1.13]	Road Inj [0.44]	Conduct [0.87]	Anxiety [0.88]	Back Pain [0.81]	Depression [0.84]	Asthma [1.32]	Drown [0.66]
Solomon	Self Harm [2.05]	Iron [1.23]	LRI [1.39]	Road Inj [0.69]	Drown [1.05]	Headaches [0.75]	Conduct [0.87]	Congenital [0.98]	Anxiety [0.9]	Depression [0.84]
Tonga	Road Inj [0.52]	Headaches [0.69]	Asthma [1.73]	Conduct [0.87]	Drown [0.96]	Anxiety [0.85]	Self Harm [0.88]	Back Pain [0.81]	Meningitis [3.58]	Depression [0.78]
Vanuatu	Self Harm [2.19]	Iron [1.41]	Road Inj [0.71]	Drown [1.15]	Headaches [0.72]	Asthma [1.61]	Conduct [0.87]	LRI [0.8]	Congenital [0.98]	RHD [2.9]
**Southeast Asia**	Road Inj [0.97]	Headaches [0.83]	Conduct [0.93]	Congenital [0.98]	Back Pain [0.74]	Anxiety [0.72]	Depression [0.73]	Drown [0.74]	Neonatal [0.72]	Typh + Paratyph [144.45]
Cambodia	Road Inj [1.02]	Iron [1.08]	Headaches [0.88]	Mech [3.26]	Drown [0.98]	Conduct [0.89]	Back Pain [1.05]	Malaria [26.73]	Congenital [0.92]	Depression [0.87]
Indonesia	Road Inj [0.99]	Headaches [0.83]	Conduct [0.98]	Typh + Paratyph [264.69]	Back Pain [0.74]	Anxiety [0.72]	Neonatal [0.74]	TB [11.28]	Congenital [0.78]	Depression [0.64]
Laos	Road Inj [1.31]	Headaches [0.86]	Iron [0.91]	Drown [0.82]	Conduct [0.89]	Anxiety [1.01]	Back Pain [0.89]	Congenital [0.95]	Typh + Paratyph [13.74]	Depression [0.79]
Malaysia	Road Inj [1.31]	Depression [1.38]	Headaches [0.76]	Conduct [0.92]	Anxiety [0.82]	Neonatal [0.8]	Back Pain [0.64]	Congenital [0.85]	Dermatitis [0.91]	Drown [1.14]
Maldives	Headaches [0.82]	Conduct [0.91]	Iron [1.4]	Depression [0.85]	Anxiety [0.72]	Back Pain [0.7]	Road Inj [0.28]	Congenital [0.78]	Neonatal [0.66]	Dermatitis [0.87]
Mauritius	Headaches [0.81]	Road Inj [0.6]	Iron [3.15]	Depression [1.15]	Conduct [0.9]	Self Harm [0.92]	Congenital [1.05]	Neonatal [0.83]	Back Pain [0.72]	Anxiety [0.68]
Myanmar	Road Inj [1.09]	Exec & Police [289.4]	Headaches [0.85]	Congenital [1.41]	Typh + Paratyph [35.02]	Conduct [0.89]	Drown [0.76]	Iron [0.81]	Malaria [148.79]	Anxiety [0.8]
Philippines	Headaches [0.83]	LRI [1.92]	Road Inj [0.47]	Congenital [1.26]	Conduct [0.9]	Violence [1.12]	Back Pain [0.88]	Asthma [1.46]	Anxiety [0.76]	Drown [0.76]
Sri Lanka	Headaches [0.81]	Self Harm [1.21]	Conduct [0.91]	Congenital [1.19]	Depression [0.9]	Anxiety [0.71]	Neonatal [0.75]	Road Inj [0.31]	Back Pain [0.63]	Drown [0.76]
Seychelles	Headaches [0.82]	Drown [1.88]	Road Inj [0.53]	Conduct [0.91]	Congenital [1.13]	Neonatal [0.82]	Anxiety [0.7]	Depression [0.77]	Back Pain [0.69]	LRI [1.85]
Thailand	Road Inj [1.82]	Headaches [0.92]	Conduct [0.9]	Drown [1.17]	Anxiety [0.71]	Depression [0.79]	Violence [1.22]	Back Pain [0.7]	Self Harm [0.75]	Congenital [0.88]
Timor-Leste	Iron [1.32]	Road Inj [0.59]	Headaches [0.86]	Conduct [0.89]	Congenital [1.02]	Depression [0.94]	HIV [11.92]	Drown [0.68]	Anxiety [0.8]	Back Pain [0.77]
Vietnam	Road Inj [1.06]	Headaches [0.84]	Drown [1.32]	Conduct [0.9]	Congenital [1.0]	Depression [0.75]	Back Pain [0.66]	Neonatal [0.62]	Epilepsy [0.93]	Anxiety [0.5]
**Sub-Saharan Africa**	HIV [45.35]	Malaria [26.89]	Iron [1.14]	Road Inj [0.57]	Diarrhea [1.25]	Headaches [0.8]	Conduct [0.94]	Depression [1.05]	Anxiety [0.96]	Epilepsy [1.23]
**Central Sub-Saharan Africa**	Malaria [58.01]	Road Inj [1.06]	HIV [28.33]	Diarrhea [1.4]	Iron [0.88]	Oncho [244.55]	Headaches [0.79]	Depression [1.21]	TB [2.06]	Epilepsy [1.5]
Angola	Road Inj [1.05]	Iron [1.62]	Diarrhea [1.79]	Malaria [28.2]	HIV [19.03]	Headaches [0.78]	Epilepsy [1.72]	Depression [1.21]	Conduct [0.93]	Yellow Fev [15.47]
C African Rep	HIV [66.55]	Road Inj [2.34]	Conflict Terror [167.57]	Malaria [5.45]	TB [2.24]	Diarrhea [1.05]	Iron [0.99]	Hemog [2.77]	Meningitis [1.02]	LRI [0.81]
Congo	HIV [125.31]	Malaria [781.81]	Road Inj [1.18]	Iron [1.78]	Diarrhea [3.26]	Headaches [0.75]	Epilepsy [1.96]	Depression [1.21]	Conduct [0.94]	Neonatal [1.06]

eFigure 7e. Leading ten causes of DALYs with the ratio of observed DALYs to DALYs expected on the basis of Socio-Demographic Index alone in 2017, 10-19 years, both sexes combined. The top ten causes contributing to DALYs are listed globally, by socio-demographic quintile, and then by GBD superregion, region, country, and subnationally where modeled. For each cell, the ratio of observed DALYs to DALYs expected on the basis of socio-demographic index (SDI) alone are listed. Abbreviations: DALY=disability-adjusted life year, GBD=Global Burden of Disease.

Values shown in brackets represent the ratio of observed DALYs to predicted DALYs on the basis of Socio-Demographic Index (SDI), rounded to two (2) digits. Color ranges (shown below) were calculated to place a roughly equal number of cells into each bin.

COLOR KEY:	[0.0-0.73]	(0.73-0.88]	(0.88-0.95]	(0.95-1.01]	(1.01-1.09]	(1.09-1.26]	(1.26-1.55]	(1.55-2.09]	2.09+

	1	2	3	4	5	6	7	8	9	10
Congo DR	Malaria [9.15]	Road Inj [0.86]	HIV [18.48]	Oncho [150.6]	Diarrhea [0.73]	Headaches [0.83]	TB [1.13]	Hemog [2.0]	Depression [1.19]	Conduct [0.94]
Eq Guinea	HIV [170.14]	Malaria [3225.92]	Road Inj [0.89]	Headaches [0.72]	Iron [1.62]	Depression [1.22]	Epilepsy [2.06]	Conduct [0.98]	Anxiety [0.86]	Back Pain [0.82]
Gabon	Malaria [3901.58]	Road Inj [1.16]	HIV [75.92]	Iron [2.79]	Headaches [0.75]	Depression [1.22]	Epilepsy [2.26]	Neonatal [1.2]	Conduct [0.93]	Anxiety [0.87]
**Eastern Sub-Saharan Africa**	HIV [52.29]	Diarrhea [1.0]	Malaria [4.25]	Iron [0.78]	Road Inj [0.49]	Epilepsy [1.36]	Depression [1.12]	Conduct [0.96]	TB [1.09]	Anxiety [1.02]
Burundi	HIV [33.04]	Malaria [2.93]	Road Inj [0.64]	TB [1.28]	Diarrhea [0.67]	Epilepsy [1.25]	Depression [1.13]	LRI [0.58]	Conduct [0.94]	Anxiety [1.05]
Comoros	Road Inj [0.55]	Diarrhea [1.22]	Epilepsy [1.51]	Neonatal [1.54]	Iron [0.67]	Conduct [0.92]	Headaches [0.61]	Anxiety [1.01]	LRI [0.72]	Depression [0.99]
Djibouti	HIV [39.79]	Road Inj [0.65]	Epilepsy [1.65]	Depression [1.14]	Conduct [0.95]	Neonatal [1.28]	Headaches [0.58]	Iron [0.7]	Anxiety [0.98]	Diarrhea [1.06]
Eritrea	HIV [23.83]	Diarrhea [1.72]	Road Inj [0.8]	Iron [1.28]	TB [2.68]	Epilepsy [1.75]	LRI [0.88]	Meningitis [1.21]	Maternal [1.55]	Depression [1.15]
Ethiopia	HIV [22.66]	Diarrhea [0.84]	Conduct [1.01]	Road Inj [0.35]	Depression [1.04]	Epilepsy [1.15]	Meningitis [0.66]	Anxiety [1.04]	Neonatal [1.72]	Headaches [0.62]
Kenya	HIV [71.59]	Neonatal [1.71]	Diarrhea [1.88]	Road Inj [0.47]	Conduct [1.0]	Epilepsy [1.59]	Headaches [0.59]	Meningitis [1.76]	Anxiety [0.99]	Congenital [1.0]
Baringo	Diarrhea [1.48]	Neonatal [1.83]	HIV [12.4]	Conduct [1.0]	Road Inj [0.42]	Meningitis [1.38]	Epilepsy [1.5]	Headaches [0.61]	Congenital [1.0]	Anxiety [0.99]
Bomet	HIV [99.01]	Neonatal [2.08]	Diarrhea [1.73]	Conduct [1.0]	Headaches [0.6]	Anxiety [1.0]	Depression [0.94]	Epilepsy [1.19]	Congenital [0.87]	Road Inj [0.31]
Bungoma	HIV [22.63]	Malaria [35.22]	Neonatal [1.88]	Diarrhea [1.44]	Conduct [1.0]	Headaches [0.6]	Anxiety [0.99]	Road Inj [0.36]	Depression [0.92]	Epilepsy [1.22]
Busia	HIV [37.51]	Malaria [35.29]	Neonatal [1.82]	Diarrhea [1.15]	Iron [0.77]	Conduct [0.99]	Road Inj [0.38]	Headaches [0.61]	Anxiety [0.99]	Epilepsy [1.22]
Elgeyo-Marakwet	HIV [30.7]	Diarrhea [2.3]	Neonatal [2.03]	Epilepsy [1.66]	Conduct [1.0]	Meningitis [1.9]	Road Inj [0.42]	Headaches [0.59]	Anxiety [0.99]	Congenital [1.03]
Embu	HIV [58.06]	Neonatal [1.9]	Diarrhea [2.39]	Conduct [1.0]	Epilepsy [1.56]	Headaches [0.59]	Road Inj [0.41]	Anxiety [0.98]	Depression [0.96]	Congenital [1.0]
Garissa	HIV [27.19]	Iron [0.82]	Diarrhea [0.72]	Neonatal [2.54]	Conduct [1.03]	Road Inj [0.36]	Epilepsy [1.25]	Meningitis [0.69]	Headaches [0.64]	TB [0.75]
HomaBay	HIV [224.99]	Road Inj [1.09]	Diarrhea [1.42]	Malaria [11.99]	Violence [1.29]	Meningitis [1.35]	Epilepsy [1.6]	TB [1.61]	Conduct [0.99]	LRI [0.78]
Isiolo	HIV [27.04]	Conflict Terror [127.51]	Diarrhea [0.81]	Meningitis [1.08]	Road Inj [0.42]	Epilepsy [1.39]	Conduct [1.01]	Neonatal [1.67]	TB [1.1]	Congenital [1.04]
Kajiado	HIV [55.27]	Neonatal [2.08]	Epilepsy [1.78]	Conduct [1.0]	Diarrhea [1.93]	Road Inj [0.42]	Headaches [0.59]	Meningitis [2.24]	Anxiety [0.99]	Depression [0.96]
Kakamega	HIV [68.13]	Malaria [20.59]	Diarrhea [1.38]	Iron [0.81]	Road Inj [0.44]	Neonatal [1.53]	Conduct [0.99]	Epilepsy [1.35]	Headaches [0.61]	Meningitis [1.23]
Kericho	HIV [55.09]	Neonatal [2.21]	Diarrhea [1.64]	Conduct [1.0]	Headaches [0.59]	Anxiety [0.99]	Epilepsy [1.34]	Depression [0.95]	Congenital [0.95]	Road Inj [0.34]
Kiambu	HIV [87.75]	Neonatal [1.97]	Road Inj [0.53]	Epilepsy [2.04]	Conduct [1.0]	Headaches [0.58]	Violence [1.09]	Anxiety [0.98]	Diarrhea [2.24]	Meningitis [2.99]
Kilifi	HIV [42.5]	Neonatal [1.77]	Diarrhea [1.29]	Conduct [1.0]	Road Inj [0.39]	Headaches [0.6]	Anxiety [0.99]	Malaria [13.81]	Epilepsy [1.27]	Congenital [0.96]
Kirinyaga	HIV [55.29]	Neonatal [1.95]	Diarrhea [2.33]	Road Inj [0.49]	Conduct [0.99]	Epilepsy [1.71]	Headaches [0.59]	Anxiety [0.99]	Congenital [1.03]	Depression [0.96]
Kisii	HIV [30.13]	Diarrhea [2.91]	Neonatal [1.48]	Road Inj [0.52]	Epilepsy [1.76]	Conduct [1.0]	Meningitis [2.28]	Headaches [0.59]	Congenital [1.07]	Anxiety [0.99]
Kisumu	HIV [180.55]	Malaria [97.97]	Diarrhea [2.14]	Epilepsy [2.12]	Meningitis [2.27]	Violence [1.16]	TB [3.39]	Road Inj [0.47]	Conduct [0.98]	LRI [1.06]
Kitui	HIV [46.31]	Neonatal [2.16]	Diarrhea [1.52]	Conduct [1.0]	Epilepsy [1.43]	Headaches [0.61]	Road Inj [0.37]	Anxiety [1.0]	Meningitis [1.24]	Depression [0.94]
Kwale	HIV [44.6]	Iron [0.98]	Neonatal [1.77]	Malaria [19.3]	Diarrhea [1.11]	Conduct [0.99]	Epilepsy [1.41]	Road Inj [0.38]	Headaches [0.6]	Anxiety [0.99]
Laikipia	HIV [98.37]	Neonatal [2.08]	Diarrhea [2.28]	Conduct [1.01]	Headaches [0.58]	Epilepsy [1.53]	Anxiety [0.97]	Depression [0.96]	Congenital [0.95]	Road Inj [0.32]

eFigure 7e. Leading ten causes of DALYs with the ratio of observed DALYs to DALYs expected on the basis of Socio-Demographic Index alone in 2017, 10-19 years, both sexes combined. The top ten causes contributing to DALYs are listed globally, by socio-demographic quintile, and then by GBD superregion, region, country, and subnationally where modeled. For each cell, the ratio of observed DALYs to DALYs expected on the basis of socio-demographic index (SDI) alone are listed. Abbreviations: DALY=disability-adjusted life year, GBD=Global Burden of Disease.

Values shown in brackets represent the ratio of observed DALYs to predicted DALYs on the basis of Socio-Demographic Index (SDI), rounded to two (2) digits. Color ranges (shown below) were calculated to place a roughly equal number of cells into each bin.

COLOR KEY:	[0.0-0.73]	(0.73-0.88]	(0.88-0.95]	(0.95-1.01]	(1.01-1.09]	(1.09-1.26]	(1.26-1.55]	(1.55-2.09]	2.09+

	1	2	3	4	5	6	7	8	9	10
Lamu	Conflict Terror [903.69]	HIV [27.19]	Exec & Police [244.81]	Neonatal [1.52]	Conduct [1.0]	Diarrhea [1.1]	Epilepsy [1.48]	Meningitis [1.36]	Headaches [0.61]	Road Inj [0.36]
Machakos	HIV [69.75]	Neonatal [1.97]	Diarrhea [1.83]	Conduct [1.0]	Headaches [0.59]	Epilepsy [1.47]	Anxiety [0.98]	Road Inj [0.37]	Depression [0.95]	Congenital [0.95]
Makueni	HIV [114.86]	Neonatal [2.0]	Diarrhea [1.49]	Conduct [0.99]	Headaches [0.61]	Anxiety [0.99]	Epilepsy [1.29]	Depression [0.95]	Congenital [0.89]	Road Inj [0.3]
Mandera	HIV [19.25]	Diarrhea [0.6]	Road Inj [0.4]	Tetanus [6.08]	Meningitis [0.67]	TB [0.76]	Conduct [1.04]	Epilepsy [1.23]	Neonatal [2.1]	Iron [0.49]
Marsabit	HIV [30.27]	Diarrhea [0.9]	Neonatal [2.69]	Conduct [1.02]	Meningitis [0.76]	Road Inj [0.36]	Epilepsy [1.17]	Tetanus [8.35]	Headaches [0.65]	Anxiety [1.01]
Meru	HIV [73.72]	Neonatal [1.94]	Diarrhea [1.91]	Conduct [0.99]	Road Inj [0.43]	Epilepsy [1.51]	Headaches [0.6]	Anxiety [0.99]	Congenital [1.0]	Depression [0.96]
Migori	HIV [142.69]	Malaria [12.3]	Diarrhea [1.47]	Road Inj [0.6]	Meningitis [1.52]	Epilepsy [1.68]	LRI [0.88]	TB [1.61]	Conduct [1.0]	Congenital [1.11]
Mombasa	HIV [66.53]	Neonatal [1.52]	Iron [1.29]	Conduct [0.98]	Epilepsy [1.68]	Headaches [0.59]	Anxiety [0.99]	Depression [0.97]	Road Inj [0.38]	Diarrhea [2.0]
Murang'a	HIV [67.96]	Neonatal [1.98]	Diarrhea [2.28]	Conduct [1.0]	Road Inj [0.44]	Headaches [0.59]	Epilepsy [1.54]	Anxiety [0.98]	Depression [0.95]	Congenital [0.99]
Nairobi	HIV [59.85]	Neonatal [1.71]	Road Inj [0.61]	Epilepsy [2.73]	Violence [1.69]	Conduct [0.99]	Headaches [0.58]	Meningitis [6.24]	Anxiety [0.9]	Depression [0.95]
Nakuru	HIV [102.15]	Neonatal [1.74]	Epilepsy [1.79]	Diarrhea [2.14]	Conduct [1.0]	Headaches [0.58]	Anxiety [0.98]	Depression [0.95]	Congenital [0.99]	Road Inj [0.34]
Nandi	HIV [52.01]	Neonatal [2.11]	Diarrhea [1.61]	Conduct [1.0]	Road Inj [0.43]	Epilepsy [1.52]	Headaches [0.6]	Anxiety [0.99]	Depression [0.95]	Congenital [0.96]
Narok	Neonatal [2.64]	HIV [15.69]	Diarrhea [1.11]	Road Inj [0.45]	Meningitis [1.17]	Epilepsy [1.54]	Conduct [1.0]	Violence [0.9]	Headaches [0.63]	Congenital [0.99]
Nyamira	HIV [72.16]	Road Inj [0.81]	Diarrhea [3.25]	Epilepsy [2.45]	Neonatal [1.7]	Meningitis [3.77]	LRI [1.58]	Violence [1.29]	TB [5.42]	Conduct [1.0]
Nyandarua	HIV [61.49]	Neonatal [2.06]	Road Inj [0.7]	Epilepsy [2.35]	Diarrhea [2.62]	Meningitis [3.26]	Violence [1.32]	LRI [1.38]	TB [4.68]	Conduct [1.0]
Nyeri	HIV [108.54]	Neonatal [1.92]	Road Inj [0.52]	Epilepsy [1.9]	Conduct [1.0]	Diarrhea [2.25]	Meningitis [2.86]	Headaches [0.59]	Anxiety [0.98]	Congenital [1.09]
Samburu	HIV [53.98]	Neonatal [3.13]	Diarrhea [0.71]	Conduct [1.03]	Tetanus [6.21]	Headaches [0.66]	TB [0.68]	Anxiety [1.01]	Meningitis [0.56]	Road Inj [0.29]
Siaya	HIV [85.45]	Malaria [40.82]	Diarrhea [1.36]	Conduct [0.99]	Headaches [0.61]	Anxiety [1.0]	Depression [0.95]	Congenital [0.95]	Epilepsy [1.22]	Neonatal [1.13]
TaitaTaveta	HIV [96.85]	Neonatal [1.76]	Epilepsy [1.9]	Road Inj [0.47]	Conduct [1.0]	Diarrhea [1.83]	Meningitis [2.3]	Headaches [0.58]	Congenital [1.07]	Anxiety [0.98]
TanaRiver	HIV [49.99]	TB [1.71]	Road Inj [0.51]	Meningitis [1.23]	Iron [0.77]	Epilepsy [1.65]	Neonatal [2.1]	Diarrhea [0.8]	LRI [0.77]	Violence [1.03]
TharakaNithi	Diarrhea [2.67]	Road Inj [0.58]	Epilepsy [2.0]	Neonatal [1.48]	Conduct [0.99]	Meningitis [2.49]	Malaria [113.17]	Violence [1.03]	Headaches [0.59]	Anxiety [0.99]
TransNzoia	HIV [94.13]	Neonatal [1.99]	Diarrhea [1.77]	Conduct [1.0]	Headaches [0.6]	Anxiety [0.99]	Depression [0.96]	Epilepsy [1.28]	Road Inj [0.34]	Congenital [0.91]
Turkana	HIV [85.75]	Diarrhea [0.8]	Tetanus [6.84]	Neonatal [2.63]	Iron [0.61]	Conduct [1.03]	Meningitis [0.64]	TB [0.69]	Road Inj [0.31]	Headaches [0.66]
UasinGishu	HIV [66.31]	Neonatal [1.83]	Diarrhea [2.35]	Conduct [0.99]	Headaches [0.59]	Anxiety [0.99]	Epilepsy [1.49]	Dermatitis [1.8]	Depression [0.95]	Congenital [0.97]
Vihiga	HIV [37.54]	Diarrhea [1.76]	Neonatal [1.77]	Road Inj [0.52]	Malaria [29.69]	Epilepsy [1.68]	Meningitis [1.77]	Conduct [0.99]	LRI [0.91]	Headaches [0.61]
Wajir	HIV [21.0]	Diarrhea [0.69]	Tetanus [4.41]	Neonatal [2.99]	Maternal [0.62]	Road Inj [0.37]	Meningitis [0.6]	Conduct [1.06]	Epilepsy [1.13]	TB [0.54]
WestPokot	HIV [16.28]	Diarrhea [1.11]	Neonatal [2.51]	Conduct [1.01]	Leish [22.18]	Malaria [2.8]	Headaches [0.63]	Meningitis [0.83]	Anxiety [1.01]	Depression [0.94]
Madagascar	Diarrhea [1.17]	Iron [0.91]	Congenital [1.48]	Road Inj [0.46]	Malaria [1.35]	LRI [0.77]	PEM [3.96]	Epilepsy [1.29]	Depression [1.13]	Conduct [0.93]
Malawi	HIV [137.69]	Iron [1.3]	Malaria [3.5]	Diarrhea [0.83]	Road Inj [0.46]	Epilepsy [1.3]	Meningitis [0.77]	TB [0.88]	Conduct [0.93]	Headaches [0.64]
Mozambique	HIV [120.15]	Malaria [4.41]	TB [1.49]	Iron [0.91]	Road Inj [0.58]	Diarrhea [0.77]	Epilepsy [1.37]	LRI [0.65]	Depression [1.14]	Conduct [0.93]

eFigure 7e. Leading ten causes of DALYs with the ratio of observed DALYs to DALYs expected on the basis of Socio-Demographic Index alone in 2017, 10-19 years, both sexes combined. The top ten causes contributing to DALYs are listed globally, by socio-demographic quintile, and then by GBD superregion, region, country, and subnationally where modeled. For each cell, the ratio of observed DALYs to DALYs expected on the basis of socio-demographic index (SDI) alone are listed. Abbreviations: DALY=disability-adjusted life year, GBD=Global Burden of Disease.

Values shown in brackets represent the ratio of observed DALYs to predicted DALYs on the basis of Socio-Demographic Index (SDI), rounded to two (2) digits. Color ranges (shown below) were calculated to place a roughly equal number of cells into each bin.

COLOR KEY:	[0.0-0.73]	(0.73-0.88]	(0.88-0.95]	(0.95-1.01]	(1.01-1.09]	(1.09-1.26]	(1.26-1.55]	(1.55-2.09]	2.09+

	1	2	3	4	5	6	7	8	9	10
Rwanda	HIV [28.63]	Road Inj [0.71]	Malaria [6.22]	Depression [1.24]	Diarrhea [0.77]	Iron [0.6]	Epilepsy [1.31]	Conduct [0.93]	Headaches [0.62]	Anxiety [1.03]
Somalia	Conflict Terror [40.6]	Road Inj [0.92]	Iron [1.03]	TB [0.98]	Diarrhea [0.54]	Meningitis [0.69]	Epilepsy [1.28]	LRI [0.63]	Depression [1.11]	HIV [8.09]
S Sudan	Conflict Terror [86.81]	Diarrhea [0.86]	Malaria [0.88]	Iron [0.9]	HIV [14.78]	Oncho [79.0]	Road Inj [0.56]	Leish [6.68]	TB [0.77]	Meningitis [0.67]
Tanzania	HIV [58.48]	Iron [1.34]	Malaria [7.27]	Diarrhea [0.87]	Epilepsy [1.45]	Depression [1.14]	Conduct [0.92]	Road Inj [0.36]	Anxiety [1.02]	Hemog [1.87]
Uganda	HIV [77.69]	Malaria [9.95]	Iron [1.11]	Road Inj [0.56]	Depression [1.59]	Diarrhea [0.87]	Epilepsy [1.36]	Conduct [0.93]	TB [1.08]	Headaches [0.61]
Zambia	HIV [135.54]	Malaria [34.5]	Diarrhea [1.73]	Road Inj [0.52]	Epilepsy [1.79]	Headaches [0.77]	TB [2.41]	Meningitis [1.53]	Conduct [0.92]	Iron [0.69]
**Southern Sub-Saharan Africa**	HIV [300.73]	Violence [2.09]	Road Inj [0.64]	Headaches [0.75]	TB [16.43]	Conduct [0.99]	Anxiety [0.95]	Depression [0.98]	Diarrhea [3.21]	Neonatal [0.91]
Botswana	HIV [81.66]	Road Inj [0.83]	Iron [2.73]	Headaches [0.74]	Neonatal [1.23]	Diarrhea [4.35]	Violence [1.54]	Conduct [0.95]	Depression [1.07]	Anxiety [0.89]
Lesotho	HIV [244.55]	Violence [3.84]	Road Inj [1.56]	TB [7.6]	Diarrhea [2.74]	Depression [1.76]	LRI [1.48]	Self Harm [1.45]	Headaches [0.79]	Conduct [0.93]
Namibia	HIV [225.65]	Road Inj [0.88]	Violence [2.13]	Headaches [0.75]	Diarrhea [3.94]	TB [11.64]	Conduct [0.94]	Iron [1.32]	Anxiety [0.94]	Neonatal [1.0]
S Africa	HIV [374.34]	Violence [2.59]	Headaches [0.75]	Road Inj [0.54]	Conduct [1.02]	Anxiety [0.97]	Depression [1.0]	Neonatal [0.92]	Congenital [0.87]	Dermatitis [1.27]
Swaziland	HIV [296.57]	Road Inj [1.43]	Violence [2.66]	Diarrhea [4.67]	TB [10.85]	Headaches [0.76]	LRI [1.78]	Self Harm [1.25]	Depression [1.14]	Conduct [0.94]
Zimbabwe	HIV [110.78]	TB [4.88]	Road Inj [0.62]	Headaches [0.8]	Diarrhea [1.37]	Self Harm [1.21]	LRI [1.05]	Iron [0.73]	Violence [1.01]	Conduct [0.93]
**Western Sub-Saharan Africa**	Malaria [31.04]	Iron [1.53]	HIV [22.55]	Headaches [1.02]	Road Inj [0.48]	Diarrhea [1.08]	Conduct [0.93]	Back Pain [1.12]	Hemog [2.26]	Meningitis [1.12]
Benin	Malaria [8.49]	Iron [1.34]	Road Inj [0.81]	Headaches [1.0]	HIV [12.11]	Diarrhea [0.79]	Conduct [0.93]	Back Pain [1.11]	Hemog [1.56]	Anxiety [0.91]
Burkina Faso	Malaria [2.39]	Iron [1.22]	Hemog [2.45]	Headaches [1.1]	Road Inj [0.44]	HIV [10.83]	Diarrhea [0.52]	Typh + Paratyph [1.04]	Conduct [0.96]	Meningitis [0.57]
Cameroon	HIV [56.53]	Malaria [107.6]	Headaches [0.97]	Road Inj [0.58]	Diarrhea [1.44]	Iron [0.8]	Conduct [0.94]	Back Pain [1.04]	Anxiety [0.9]	LRI [0.76]
Cape Verde	Headaches [0.97]	Violence [1.38]	Neonatal [1.39]	Iron [1.18]	Conduct [0.93]	HIV [17.73]	Back Pain [0.96]	Depression [1.0]	Anxiety [0.9]	Epilepsy [1.04]
Chad	Iron [1.44]	HIV [17.19]	Malaria [0.73]	Diarrhea [0.73]	Maternal [0.98]	Road Inj [0.53]	Headaches [1.12]	Meningitis [0.66]	Back Pain [1.38]	LRI [0.56]
Cote d'Ivoire	Malaria [15.45]	HIV [28.77]	Iron [1.13]	Headaches [1.01]	Road Inj [0.55]	Diarrhea [0.85]	Conduct [0.95]	LRI [0.65]	Meningitis [0.92]	Violence [0.82]
Gambia	Iron [1.86]	Hemog [6.03]	Headaches [1.02]	HIV [15.24]	Road Inj [0.46]	Meningitis [1.05]	Diarrhea [0.78]	Vit A [2.15]	Depression [1.13]	Neonatal [1.59]
Ghana	Malaria [394.38]	HIV [42.39]	Iron [2.26]	Road Inj [0.81]	Headaches [0.96]	Hemog [4.49]	Meningitis [2.76]	Conduct [0.93]	Diarrhea [1.57]	Anxiety [0.89]
Guinea	Malaria [3.66]	Iron [0.93]	HIV [13.91]	Headaches [1.08]	Road Inj [0.51]	Diarrhea [0.56]	Meningitis [0.75]	Maternal [0.88]	Conduct [0.95]	LRI [0.56]
Guinea-Bissau	HIV [45.59]	Hemog [5.44]	Road Inj [0.92]	Iron [1.29]	Diarrhea [1.06]	Headaches [1.06]	Meningitis [0.98]	LRI [0.67]	Conduct [0.94]	TB [0.81]
Liberia	Oncho [194.57]	Malaria [2.82]	HIV [20.29]	Iron [0.93]	Diarrhea [0.85]	Headaches [1.06]	Hemog [2.16]	Road Inj [0.35]	Conduct [0.94]	Back Pain [1.09]
Mali	Iron [1.62]	Malaria [1.26]	Diarrhea [0.68]	Headaches [1.12]	HIV [10.87]	Meningitis [0.72]	Road Inj [0.41]	Vit A [1.36]	Maternal [0.6]	Hemog [1.26]
Mauritania	Road Inj [0.85]	Iron [1.19]	Headaches [0.98]	Diarrhea [1.39]	Conduct [0.93]	Neonatal [1.22]	Back Pain [0.96]	Anxiety [0.91]	Depression [0.74]	Meningitis [0.99]
Niger	Malaria [0.77]	Iron [0.94]	Headaches [1.17]	Diarrhea [0.47]	Maternal [0.46]	Road Inj [0.34]	Conduct [0.97]	Meningitis [0.49]	Back Pain [1.24]	Anxiety [0.95]
Nigeria	Malaria [107.06]	Iron [1.99]	HIV [26.56]	Headaches [1.01]	Back Pain [1.24]	Conduct [0.92]	Diarrhea [1.35]	Depression [1.03]	Road Inj [0.38]	Hemog [2.5]

eFigure 7e. Leading ten causes of DALYs with the ratio of observed DALYs to DALYs expected on the basis of Socio-Demographic Index alone in 2017, 10-19 years, both sexes combined. The top ten causes contributing to DALYs are listed globally, by socio-demographic quintile, and then by GBD superregion, region, country, and subnationally where modeled. For each cell, the ratio of observed DALYs to DALYs expected on the basis of socio-demographic index (SDI) alone are listed. Abbreviations: DALY=disability-adjusted life year, GBD=Global Burden of Disease.

Values shown in brackets represent the ratio of observed DALYs to predicted DALYs on the basis of Socio-Demographic Index (SDI), rounded to two (2) digits. Color ranges (shown below) were calculated to place a roughly equal number of cells into each bin.

COLOR KEY:	[0.0-0.73]	(0.73-0.88]	(0.88-0.95]	(0.95-1.01]	(1.01-1.09]	(1.09-1.26]	(1.26-1.55]	(1.55-2.09]	2.09+

	1	2	3	4	5	6	7	8	9	10
Sao Tome Principe	**Headaches** [0.97]	**LRI** [1.11]	**Iron** [0.91]	**Road Inj** [0.47]	**Conduct** [0.94]	**Neonatal** [1.23]	**Back Pain** [0.92]	**Anxiety** [0.9]	**Depression** [0.85]	**Congenital** [0.88]
Senegal	**Iron** [1.62]	**Headaches** [1.04]	**Diarrhea** [0.89]	**Hemog** [2.3]	**Road Inj** [0.42]	**Meningitis** [0.93]	**Conduct** [0.95]	**Neonatal** [1.5]	**HIV** [7.65]	**Anxiety** [0.91]
Sierra Leone	**Malaria** [8.59]	**Iron** [0.97]	**Diarrhea** [0.89]	**Headaches** [1.07]	**Road Inj** [0.51]	**Maternal** [1.31]	**Meningitis** [0.9]	**Conduct** [0.93]	**LRI** [0.62]	**HIV** [8.28]
Togo	**HIV** [48.7]	**Malaria** [26.86]	**Road Inj** [0.65]	**Iron** [1.04]	**Hemog** [3.4]	**Headaches** [1.0]	**Diarrhea** [0.98]	**Conduct** [0.94]	**Back Pain** [1.04]	**Anxiety** [0.9]

Appendix Figure 8: Co−evolution of YLL to YLD ratios and SDI for GBD regions and Level 1 causes, 1990 to 2017, both sexes combined.

(a) All causes

© 2019 GBD 2017 Child and Adolescent Health Collaborators. JAMA Pediatrics.

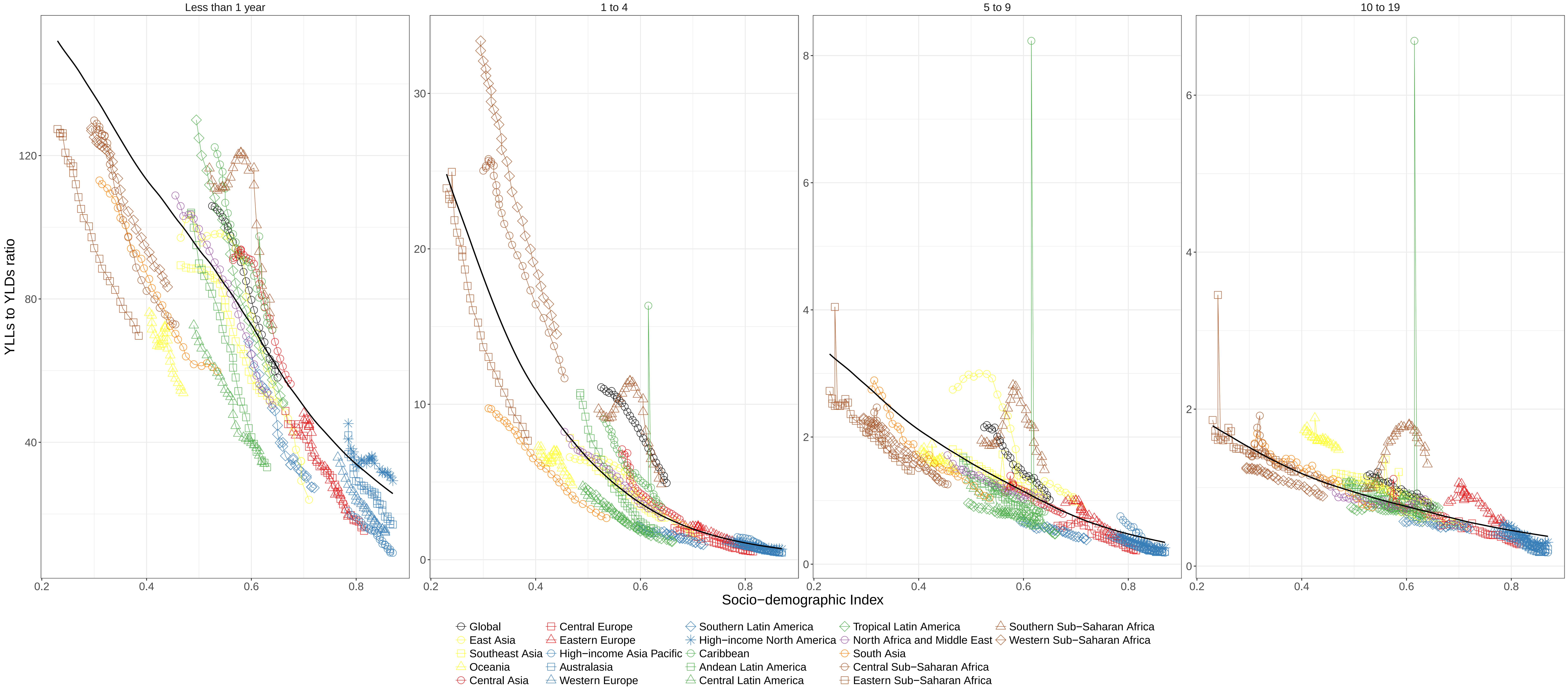

The coevolution of YLL to YLD ratio is plotted for each GBD region through time. In panel a) changes across all causes are plotted; in panel b) changes in the YLL to YLD ratio for maternal, neonatal, and nutritional (CMNN) conditions are plotted; in panel c), changes in the YLL to YLD ratio for non−communicable diseases (NCDs) is plotted and in panel d) changes in the YLL to YLD ratio for injuries is plotted. Within each panel, trends for children less than 1 are plotted in left sub−panel, between 1 and 4 in the second sub−panel, between 5 and 9 in the third sub−panel, and between 10 and 19 in the right sub−panel. Abbreviations: YLD=year of life lived with disability, YLL=year of life lost, GBD=Global Burden of Disease.

Appendix Figure 8: Co−evolution of YLL to YLD ratios and SDI for GBD regions and Level 1 causes, 1990 to 2017, both sexes combined.

(b) Communicable, maternal, neonatal, and nutritional diseases

© 2019 GBD 2017 Child and Adolescent Health Collaborators. JAMA Pediatrics.

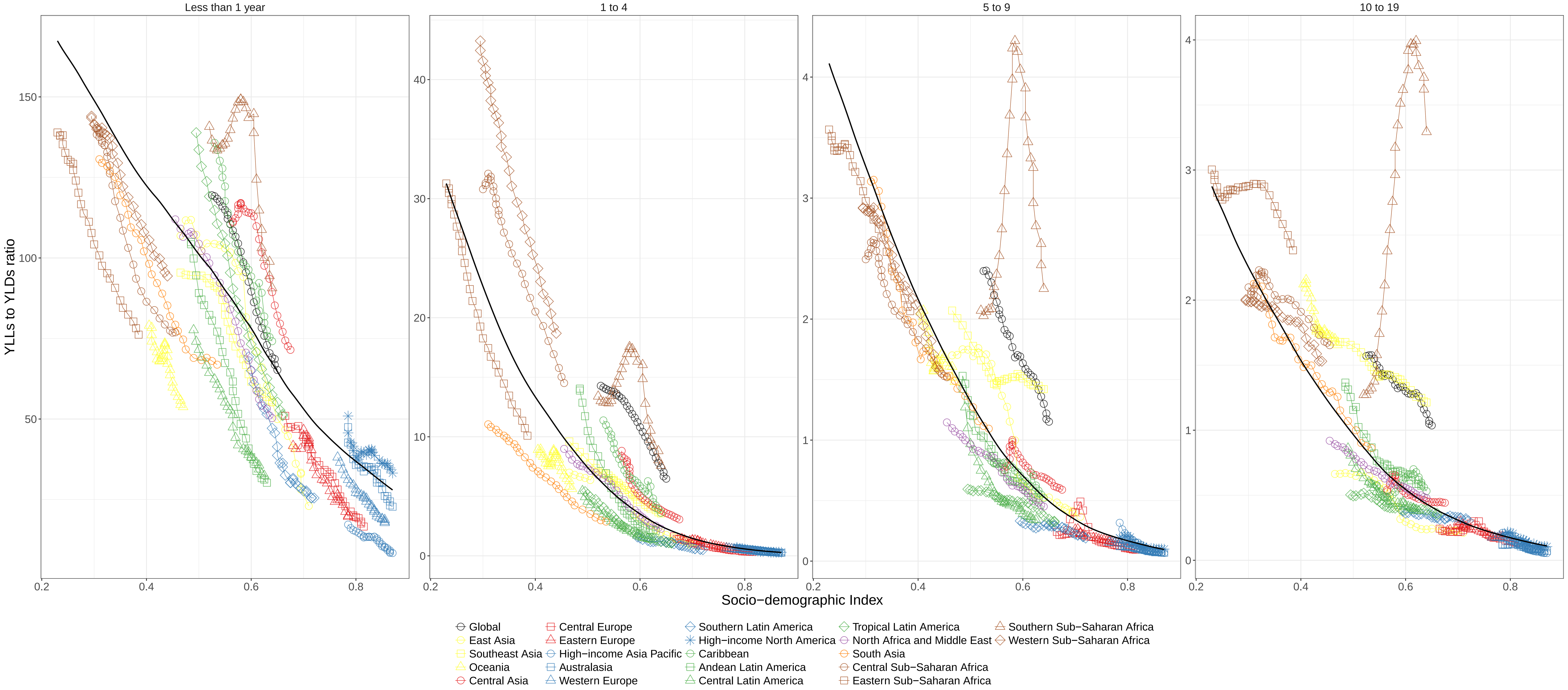

The coevolution of YLL to YLD ratio is plotted for each GBD region through time. In panel a) changes across all causes are plotted; in panel b) changes in the YLL to YLD ratio for maternal, neonatal, and nutritional (CMNN) conditions are plotted; in panel c), changes in the YLL to YLD ratio for non−communicable diseases (NCDs) is plotted and in panel d) changes in the YLL to YLD ratio for injuries is plotted. Within each panel, trends for children less than 1 are plotted in left sub−panel, between 1 and 4 in the second sub−panel, between 5 and 9 in the third sub−panel, and between 10 and 19 in the right sub−panel. Abbreviations: YLD=year of life lived with disability, YLL=year of life lost, GBD=Global Burden of Disease.

Appendix Figure 8: Co−evolution of YLL to YLD ratios and SDI for GBD regions and Level 1 causes, 1990 to 2017, both sexes combined.

(c) Non−communicable diseases

© 2019 GBD 2017 Child and Adolescent Health Collaborators. JAMA Pediatrics.

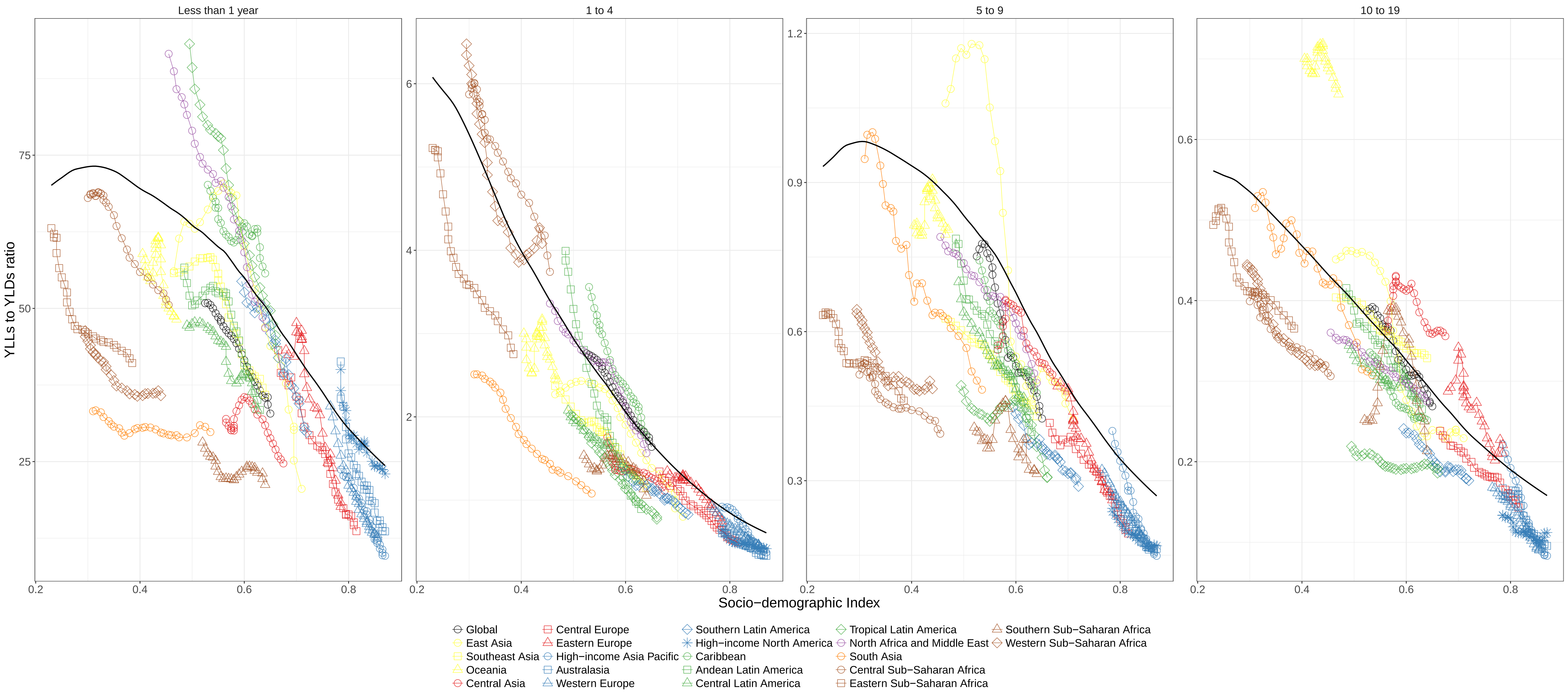

The coevolution of YLL to YLD ratio is plotted for each GBD region through time. In panel a) changes across all causes are plotted; in panel b) changes in the YLL to YLD ratio for maternal, neonatal, and nutritional (CMNN) conditions are plotted; in panel c), changes in the YLL to YLD ratio for non−communicable diseases (NCDs) is plotted and in panel d) changes in the YLL to YLD ratio for injuries is plotted. Within each panel, trends for children less than 1 are plotted in left sub−panel, between 1 and 4 in the second sub−panel, between 5 and 9 in the third sub−panel, and between 10 and 19 in the right sub−panel. Abbreviations: YLD=year of life lived with disability, YLL=year of life lost, GBD=Global Burden of Disease.

Appendix Figure 8: Co−evolution of YLL to YLD ratios and SDI for GBD regions and Level 1 causes, 1990 to 2017, both sexes combined.

(d) Injuries

© 2019 GBD 2017 Child and Adolescent Health Collaborators. JAMA Pediatrics.

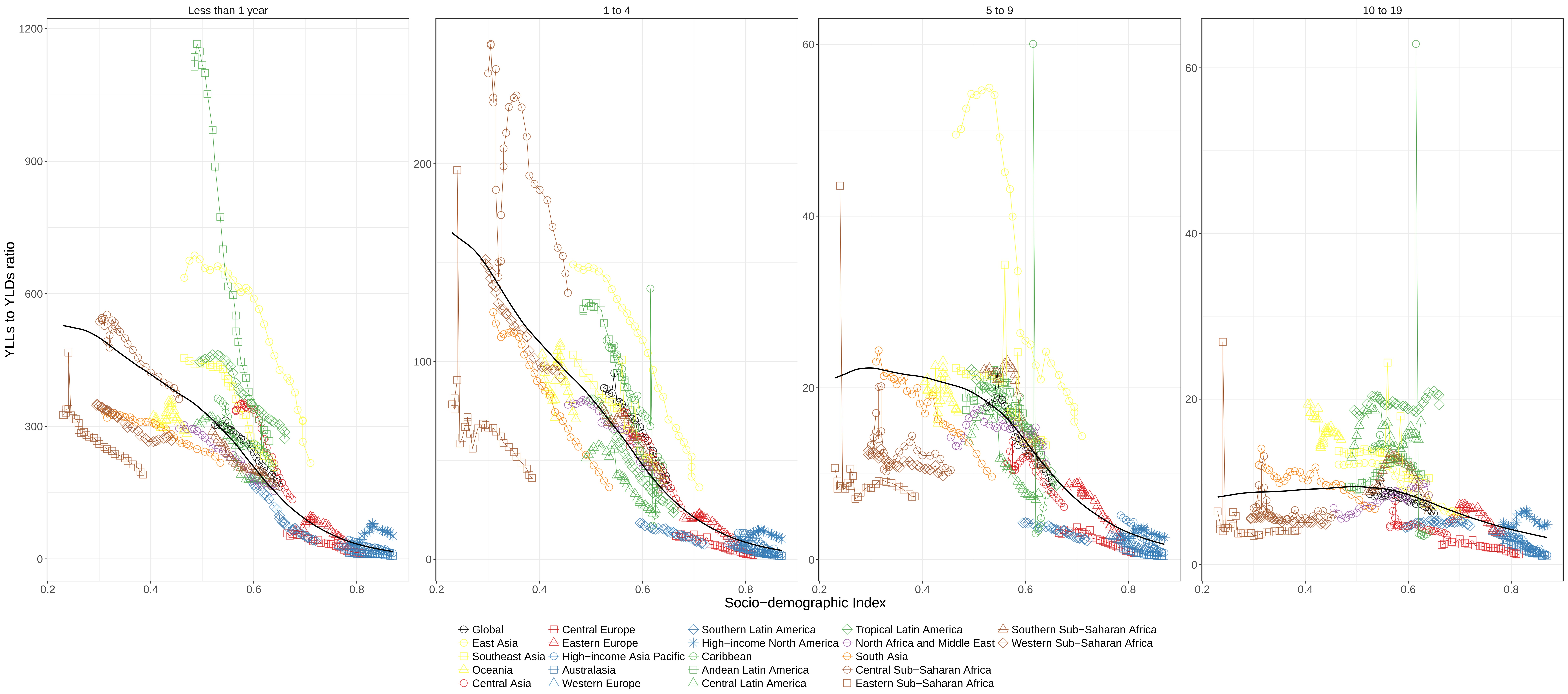

The coevolution of YLL to YLD ratio is plotted for each GBD region through time. In panel a) changes across all causes are plotted; in panel b) changes in the YLL to YLD ratio for maternal, neonatal, and nutritional (CMNN) conditions are plotted; in panel c), changes in the YLL to YLD ratio for non−communicable diseases (NCDs) is plotted and in panel d) changes in the YLL to YLD ratio for injuries is plotted. Within each panel, trends for children less than 1 are plotted in left sub−panel, between 1 and 4 in the second sub−panel, between 5 and 9 in the third sub−panel, and between 10 and 19 in the right sub−panel. Abbreviations: YLD=year of life lived with disability, YLL=year of life lost, GBD=Global Burden of Disease.

eFigure 9a. Leading ten causes of YLDs with the ratio of observed YLDs to YLDs expected on the basis of Socio-Demographic Index alone in 2017, <20 years, both sexes combined.
The top ten causes contributing to YLDs are listed globally, by socio-demographic quintile, and then by GBD superregion, region, country, and subnationally where modeled. For each cell, the ratio of observed YLDs to YLDs expected on the basis of socio-demographic index (SDI) alone are listed. Abbreviations: YLD=year of life lived with disability, GBD=Global Burden of Disease.

Values shown in brackets represent the ratio of observed YLDs to predicted YLDs on the basis of Socio-Demographic Index (SDI), rounded to two (2) digits. Color ranges (shown below) were calculated to place a roughly equal number of cells into each bin.

COLOR KEY:	[0.0-0.78]	(0.78-0.88]	(0.88-0.94]	(0.94-1.0]	(1.0-1.06]	(1.06-1.18]	(1.18-1.38]	(1.38-1.66]	1.66+

	1	2	3	4	5	6	7	8	9	10
Global	Iron [2.08]	Neonatal [1.13]	Headaches [0.82]	Vit A [1.88]	Conduct [0.87]	Dermatitis [0.79]	Anxiety [0.86]	Congenital [0.99]	Back Pain [0.79]	Depression [0.87]
Low SDI	Iron [1.2]	Vit A [1.01]	Neonatal [1.34]	Headaches [0.96]	Dermatitis [0.85]	Conduct [0.94]	Diarrhea [0.94]	Congenital [1.04]	Anxiety [0.98]	Depression [0.96]
Low-middle SDI	Iron [1.64]	Neonatal [1.16]	Vit A [1.29]	Headaches [0.95]	Conduct [0.89]	Dermatitis [0.79]	Anxiety [0.92]	Diarrhea [1.13]	Congenital [1.05]	Back Pain [0.92]
Middle SDI	Neonatal [1.2]	Headaches [0.81]	Iron [1.12]	Conduct [0.87]	Dermatitis [0.7]	Anxiety [0.77]	Vit A [1.16]	Asthma [0.79]	Back Pain [0.76]	Congenital [0.91]
High-middle SDI	Neonatal [1.34]	Headaches [0.75]	Anxiety [0.72]	Conduct [0.89]	Back Pain [0.77]	Dermatitis [0.6]	Depression [0.82]	Asthma [0.83]	Congenital [0.92]	Iron [2.91]
High SDI	Neonatal [0.88]	Headaches [0.92]	Dermatitis [1.2]	Anxiety [1.16]	Depression [1.31]	Conduct [0.98]	Back Pain [1.05]	Asthma [0.96]	Drugs [3.12]	Congenital [0.89]
Central Europe, Eastern Europe, and Central Asia	Neonatal [1.38]	Headaches [0.82]	Iron [2.41]	Conduct [0.94]	Falls [1.7]	Congenital [1.08]	Dermatitis [0.61]	Back Pain [0.73]	Anxiety [0.56]	Upper Digest [3.75]
Central Asia	Iron [2.31]	Neonatal [0.98]	Headaches [0.78]	Conduct [0.88]	Vit A [1.55]	Congenital [1.07]	Epilepsy [1.3]	Dermatitis [0.54]	Back Pain [0.66]	Depression [0.69]
Armenia	Iron [3.01]	Neonatal [0.99]	Headaches [0.79]	Conduct [0.92]	Congenital [1.06]	Back Pain [0.68]	Diarrhea [1.37]	Dermatitis [0.53]	Anxiety [0.53]	Epilepsy [1.22]
Azerbaijan	Neonatal [0.96]	Headaches [0.79]	Iron [1.56]	Conduct [0.91]	Congenital [1.04]	Back Pain [0.66]	Dermatitis [0.53]	Epilepsy [1.34]	Diarrhea [1.25]	Anxiety [0.53]
Georgia	Neonatal [0.99]	Headaches [0.79]	Conduct [0.91]	Iron [1.1]	Congenital [1.08]	Epilepsy [1.26]	Anxiety [0.53]	Diarrhea [1.23]	Dermatitis [0.49]	Back Pain [0.59]
Kazakhstan	Neonatal [1.07]	Vit A [3.21]	Headaches [0.71]	Iron [1.74]	Conduct [0.84]	Congenital [1.1]	Depression [0.73]	Back Pain [0.62]	Epilepsy [1.43]	Dermatitis [0.5]
Kyrgyzstan	Iron [1.88]	Neonatal [1.03]	Headaches [0.79]	Vit A [1.14]	Conduct [0.87]	Congenital [1.09]	Hernia [6.09]	Dermatitis [0.58]	Back Pain [0.69]	Epilepsy [1.05]
Mongolia	Neonatal [1.03]	Vit A [2.22]	Iron [1.23]	Headaches [0.72]	Conduct [0.82]	Congenital [1.1]	Dermatitis [0.56]	Diarrhea [1.19]	Depression [0.78]	Epilepsy [1.25]
Tajikistan	Iron [0.9]	Neonatal [1.05]	Vit A [0.98]	Headaches [0.86]	Conduct [0.91]	Congenital [1.1]	Diarrhea [0.99]	Epilepsy [1.09]	Dermatitis [0.58]	Back Pain [0.75]
Turkmenistan	Neonatal [0.9]	Iron [1.56]	Headaches [0.75]	Conduct [0.86]	Congenital [1.07]	Dermatitis [0.54]	Epilepsy [1.31]	Vit A [1.14]	Back Pain [0.61]	Anxiety [0.51]
Uzbekistan	Iron [2.64]	Neonatal [0.93]	Headaches [0.83]	Conduct [0.91]	Congenital [1.05]	Vit A [1.04]	Epilepsy [1.25]	Back Pain [0.71]	Dermatitis [0.56]	Depression [0.75]
Central Europe	Neonatal [1.42]	Headaches [0.85]	Falls [2.16]	Conduct [0.99]	Back Pain [0.89]	Congenital [1.22]	Anxiety [0.67]	Dermatitis [0.65]	Diarrhea [2.26]	Upper Digest [3.99]
Albania	Neonatal [1.18]	Headaches [0.95]	Falls [3.38]	Back Pain [1.19]	Conduct [1.0]	Congenital [1.17]	Anxiety [0.82]	Vit A [1.31]	Iron [0.78]	Upper Digest [3.38]
Bosnia	Neonatal [1.34]	Headaches [0.93]	Falls [3.21]	Conduct [1.0]	Back Pain [0.96]	Anxiety [0.79]	Congenital [1.17]	Vit A [1.61]	Dermatitis [0.59]	Iron [0.85]
Bulgaria	Neonatal [1.26]	Headaches [0.83]	Falls [2.22]	Conduct [0.97]	Congenital [1.26]	Anxiety [0.71]	Back Pain [0.83]	Diarrhea [1.97]	Dermatitis [0.53]	Vit A [2.25]
Croatia	Neonatal [1.53]	Headaches [0.88]	Conduct [1.01]	Back Pain [0.9]	Anxiety [0.68]	Congenital [1.2]	Falls [1.35]	Dermatitis [0.56]	Diarrhea [1.95]	Depression [0.7]
Czech	Neonatal [0.96]	Falls [2.33]	Headaches [0.79]	Back Pain [0.93]	Conduct [0.96]	Dermatitis [0.66]	Congenital [1.17]	Anxiety [0.61]	Diarrhea [2.68]	Depression [0.59]
Hungary	Neonatal [1.36]	Headaches [0.87]	Falls [2.24]	Diarrhea [3.35]	Back Pain [1.01]	Conduct [1.01]	Dermatitis [0.76]	Congenital [1.25]	Anxiety [0.68]	Depression [0.62]
Macedonia	Neonatal [1.34]	Headaches [0.88]	Falls [2.55]	Conduct [0.97]	Iron [1.81]	Congenital [1.16]	Anxiety [0.72]	Vit A [2.35]	Back Pain [0.7]	Dermatitis [0.54]
Montenegro	Neonatal [1.32]	Headaches [0.89]	Falls [2.36]	Conduct [1.01]	Back Pain [0.97]	Congenital [1.18]	Anxiety [0.71]	Dermatitis [0.57]	Depression [0.65]	Vit A [2.4]
Poland	Neonatal [1.59]	Headaches [0.84]	Falls [1.96]	Conduct [0.98]	Dermatitis [0.75]	Congenital [1.23]	Anxiety [0.64]	Back Pain [0.72]	Upper Digest [4.72]	Asthma [0.78]
Romania	Neonatal [1.44]	Iron [4.07]	Headaches [0.87]	Falls [2.31]	Back Pain [1.08]	Conduct [1.0]	Upper Digest [5.78]	Diarrhea [2.51]	Congenital [1.21]	Vit A [3.22]
Serbia	Neonatal [1.73]	Headaches [0.88]	Falls [2.71]	Conduct [1.0]	Back Pain [1.01]	Congenital [1.26]	Anxiety [0.73]	Dermatitis [0.58]	Vit A [1.71]	Diarrhea [1.39]
Slovakia	Neonatal [1.06]	Headaches [0.84]	Falls [1.99]	Back Pain [0.97]	Conduct [0.98]	Congenital [1.3]	Diarrhea [2.83]	Anxiety [0.65]	Dermatitis [0.65]	Upper Digest [4.28]
Slovenia	Neonatal [1.13]	Falls [2.23]	Headaches [0.81]	Back Pain [0.98]	Conduct [0.97]	Congenital [1.28]	Anxiety [0.61]	Diarrhea [2.71]	Dermatitis [0.57]	Asthma [0.71]

eFigure 9a. Leading ten causes of YLDs with the ratio of observed YLDs to YLDs expected on the basis of Socio-Demographic Index alone in 2017, <20 years, both sexes combined.
The top ten causes contributing to YLDs are listed globally, by socio-demographic quintile, and then by GBD superregion, region, country, and subnationally where modeled. For each cell, the ratio of observed YLDs to YLDs expected on the basis of socio-demographic index (SDI) alone are listed. Abbreviations: YLD=year of life lived with disability, GBD=Global Burden of Disease.

Values shown in brackets represent the ratio of observed YLDs to predicted YLDs on the basis of Socio-Demographic Index (SDI), rounded to two (2) digits. Color ranges (shown below) were calculated to place a roughly equal number of cells into each bin.

COLOR KEY:	[0.0-0.78]	(0.78-0.88]	(0.88-0.94]	(0.94-1.0]	(1.0-1.06]	(1.06-1.18]	(1.18-1.38]	(1.38-1.66]	1.66+

	1	2	3	4	5	6	7	8	9	10
**Eastern Europe**	Neonatal [1.66]	Headaches [0.84]	Conduct [0.96]	Upper Digest [5.86]	Falls [1.67]	Dermatitis [0.65]	Iron [1.95]	Congenital [1.03]	Back Pain [0.71]	Anxiety [0.54]
Belarus	Neonatal [1.62]	Headaches [0.86]	Conduct [0.91]	Upper Digest [5.8]	Dermatitis [0.73]	Falls [1.7]	Back Pain [0.75]	Congenital [1.01]	Anxiety [0.56]	Depression [0.72]
Estonia	Neonatal [1.72]	Headaches [0.88]	Dermatitis [0.94]	Conduct [0.97]	Upper Digest [6.43]	Falls [1.35]	Congenital [1.06]	Back Pain [0.67]	Diarrhea [2.58]	Anxiety [0.53]
Latvia	Neonatal [1.55]	Headaches [0.88]	Upper Digest [7.25]	Conduct [0.95]	Falls [1.49]	Dermatitis [0.66]	Back Pain [0.76]	Diarrhea [2.38]	Congenital [0.97]	Anxiety [0.54]
Lithuania	Neonatal [1.78]	Headaches [1.01]	Conduct [0.98]	Upper Digest [6.86]	Falls [1.47]	Back Pain [0.77]	Congenital [1.14]	Diarrhea [2.62]	Depression [0.83]	Anxiety [0.56]
Moldova	Neonatal [1.68]	Headaches [0.97]	Iron [1.4]	Upper Digest [7.47]	Conduct [0.97]	Falls [2.67]	Dermatitis [0.77]	Back Pain [0.9]	Congenital [0.99]	Anxiety [0.68]
Russian Federation	Neonatal [1.66]	Headaches [0.82]	Conduct [0.96]	Upper Digest [5.74]	Falls [1.65]	Dermatitis [0.64]	Iron [2.14]	Congenital [1.01]	Back Pain [0.65]	Diarrhea [1.88]
Ukraine	Neonatal [1.62]	Headaches [0.89]	Conduct [0.98]	Upper Digest [5.88]	Back Pain [0.91]	Falls [1.92]	Dermatitis [0.66]	Congenital [1.06]	Iron [1.25]	Anxiety [0.59]
**High-income**	Dermatitis [1.2]	Neonatal [0.9]	Headaches [0.93]	Anxiety [1.09]	Depression [1.35]	Back Pain [1.02]	Conduct [1.02]	Asthma [1.04]	Drugs [2.85]	Congenital [0.93]
**Australasia**	Asthma [1.97]	Anxiety [1.19]	Neonatal [0.91]	Falls [2.01]	Dermatitis [1.08]	Depression [1.52]	Headaches [0.84]	Back Pain [1.14]	Conduct [0.99]	Congenital [0.78]
Australia	Asthma [2.03]	Anxiety [1.14]	Neonatal [0.88]	Depression [1.54]	Falls [1.92]	Headaches [0.83]	Dermatitis [1.03]	Back Pain [1.08]	Conduct [0.97]	Congenital [0.78]
New Zealand	Anxiety [1.48]	Dermatitis [1.35]	Neonatal [1.02]	Falls [2.49]	Back Pain [1.43]	Asthma [1.66]	Headaches [0.85]	Depression [1.41]	Conduct [1.06]	Mech [5.29]
**High-income Asia Pacific**	Neonatal [1.15]	Dermatitis [1.2]	Back Pain [1.2]	Headaches [0.77]	Anxiety [0.8]	Conduct [0.99]	Depression [0.95]	Falls [1.18]	Asthma [0.81]	Congenital [0.93]
Brunei	Neonatal [1.15]	Dermatitis [1.13]	Headaches [0.74]	Anxiety [0.81]	Back Pain [0.96]	Conduct [0.92]	Depression [0.85]	Iron [3.52]	Falls [1.09]	Asthma [0.77]
Japan	Neonatal [1.13]	Dermatitis [1.27]	Back Pain [1.28]	Headaches [0.72]	Conduct [1.03]	Anxiety [0.78]	Falls [1.29]	Depression [0.92]	Congenital [1.02]	Asthma [0.82]
Aichi	Neonatal [1.16]	Dermatitis [1.28]	Back Pain [1.25]	Conduct [1.02]	Anxiety [0.77]	Headaches [0.56]	Falls [1.24]	Depression [0.91]	ASD [2.01]	Asthma [0.83]
Akita	Neonatal [1.21]	Dermatitis [1.25]	Back Pain [1.38]	Headaches [0.77]	Conduct [1.05]	Anxiety [0.85]	Depression [1.05]	Falls [1.45]	Congenital [1.09]	Asthma [0.8]
Aomori	Neonatal [1.14]	Dermatitis [1.25]	Back Pain [1.37]	Headaches [0.76]	Conduct [1.04]	Anxiety [0.84]	Falls [1.46]	Depression [1.0]	Asthma [0.8]	Acne [1.46]
Chiba	Neonatal [1.12]	Dermatitis [1.26]	Back Pain [1.29]	Headaches [0.74]	Conduct [1.04]	Anxiety [0.79]	Falls [1.31]	Depression [0.94]	Congenital [1.05]	Asthma [0.82]
Ehime	Dermatitis [1.27]	Neonatal [0.93]	Back Pain [1.28]	Headaches [0.73]	Conduct [1.03]	Anxiety [0.81]	Falls [1.37]	Depression [0.92]	Asthma [0.8]	Congenital [0.93]
Fukui	Neonatal [1.11]	Dermatitis [1.26]	Back Pain [1.29]	Headaches [0.73]	Conduct [1.04]	Anxiety [0.8]	Falls [1.33]	Depression [0.91]	Asthma [0.81]	Congenital [0.91]
Fukuoka	Neonatal [1.11]	Dermatitis [1.28]	Back Pain [1.24]	Headaches [0.71]	Conduct [1.0]	Anxiety [0.78]	Falls [1.3]	Depression [0.89]	Asthma [0.82]	Congenital [0.97]
Fukushima	Neonatal [1.04]	Dermatitis [1.25]	Back Pain [1.34]	Headaches [0.74]	Conduct [1.04]	Anxiety [0.83]	Falls [1.43]	Depression [0.96]	Congenital [1.01]	Asthma [0.79]
Gifu	Neonatal [1.15]	Dermatitis [1.26]	Back Pain [1.33]	Headaches [0.75]	Conduct [1.04]	Anxiety [0.81]	Falls [1.35]	Depression [0.81]	Iron [3.19]	Asthma [0.81]
Gunma	Neonatal [1.08]	Dermatitis [1.25]	Back Pain [1.34]	Headaches [0.75]	Conduct [1.05]	Anxiety [0.82]	Falls [1.37]	Depression [0.99]	Iron [3.47]	Asthma [0.81]
Hiroshima	Neonatal [1.17]	Dermatitis [1.28]	Back Pain [1.23]	Headaches [0.71]	Conduct [1.02]	Anxiety [0.77]	Falls [1.26]	Depression [0.87]	Asthma [0.83]	Congenital [0.91]
Hokkaido	Neonatal [1.08]	Dermatitis [1.26]	Back Pain [1.32]	Headaches [0.74]	Conduct [1.03]	Anxiety [0.81]	Falls [1.39]	Depression [0.95]	Congenital [1.0]	Iron [3.02]
Hyogo	Neonatal [1.14]	Dermatitis [1.26]	Back Pain [1.29]	Headaches [0.74]	Conduct [1.03]	Anxiety [0.79]	Falls [1.3]	Depression [0.95]	Acne [1.33]	Asthma [0.68]
Ibaraki	Neonatal [1.17]	Dermatitis [1.26]	Back Pain [1.31]	Conduct [1.04]	Anxiety [0.81]	Headaches [0.65]	Falls [1.34]	Depression [0.96]	Congenital [1.08]	Asthma [0.81]
Ishikawa	Neonatal [1.09]	Dermatitis [1.26]	Back Pain [1.31]	Headaches [0.75]	Conduct [1.04]	Anxiety [0.81]	Falls [1.33]	Depression [0.92]	Congenital [1.0]	Asthma [0.82]
Iwate	Neonatal [1.17]	Dermatitis [1.26]	Back Pain [1.34]	Headaches [0.74]	Conduct [1.03]	Anxiety [0.83]	Depression [1.0]	Falls [1.44]	Congenital [1.07]	Asthma [0.8]

eFigure 9a. Leading ten causes of YLDs with the ratio of observed YLDs to YLDs expected on the basis of Socio-Demographic Index alone in 2017, <20 years, both sexes combined.
The top ten causes contributing to YLDs are listed globally, by socio-demographic quintile, and then by GBD superregion, region, country, and subnationally where modeled. For each cell, the ratio of observed YLDs to YLDs expected on the basis of socio-demographic index (SDI) alone are listed. Abbreviations: YLD=year of life lived with disability, GBD=Global Burden of Disease.

Values shown in brackets represent the ratio of observed YLDs to predicted YLDs on the basis of Socio-Demographic Index (SDI), rounded to two (2) digits. Color ranges (shown below) were calculated to place a roughly equal number of cells into each bin.

COLOR KEY:	[0.0-0.78]	(0.78-0.88]	(0.88-0.94]	(0.94-1.0]	(1.0-1.06]	(1.06-1.18]	(1.18-1.38]	(1.38-1.66]	1.66+

	1	2	3	4	5	6	7	8	9	10
Kagawa	Neonatal [1.07]	Dermatitis [1.26]	Back Pain [1.28]	Headaches [0.74]	Conduct [1.04]	Anxiety [0.81]	Falls [1.33]	Depression [0.91]	Asthma [0.81]	Congenital [0.96]
Kagoshima	Neonatal [0.98]	Dermatitis [1.29]	Back Pain [1.23]	Headaches [0.7]	Conduct [1.0]	Anxiety [0.79]	Falls [1.38]	Congenital [1.13]	Asthma [0.8]	Depression [0.76]
Kanagawa	Neonatal [1.18]	Dermatitis [1.27]	Back Pain [1.26]	Headaches [0.73]	Conduct [1.03]	Anxiety [0.77]	Falls [1.27]	Depression [0.95]	Congenital [1.16]	Asthma [0.84]
Kochi	Neonatal [1.04]	Dermatitis [1.26]	Back Pain [1.32]	Headaches [0.74]	Conduct [1.03]	Anxiety [0.83]	Falls [1.42]	Depression [0.94]	Iron [3.11]	Asthma [0.79]
Kumamoto	Neonatal [1.02]	Dermatitis [1.29]	Back Pain [1.23]	Headaches [0.69]	Conduct [0.99]	Anxiety [0.79]	Falls [1.39]	Depression [0.86]	Asthma [0.81]	Iron [2.6]
Kyoto	Neonatal [1.17]	Back Pain [1.34]	Dermatitis [0.99]	Headaches [0.76]	Conduct [1.05]	Anxiety [0.79]	Falls [1.28]	Depression [0.97]	Congenital [1.25]	Asthma [0.83]
Mie	Neonatal [1.16]	Dermatitis [1.27]	Back Pain [1.28]	Headaches [0.73]	Conduct [1.03]	Anxiety [0.8]	Falls [1.31]	Depression [0.9]	Iron [3.27]	Asthma [0.82]
Miyagi	Neonatal [1.07]	Dermatitis [1.26]	Back Pain [1.32]	Headaches [0.74]	Conduct [1.03]	Anxiety [0.8]	Falls [1.36]	Depression [0.99]	Congenital [1.1]	Acne [1.4]
Miyazaki	Neonatal [1.02]	Dermatitis [1.29]	Back Pain [1.25]	Headaches [0.7]	Conduct [1.0]	Anxiety [0.8]	Falls [1.42]	Depression [0.9]	Iron [3.0]	Congenital [1.01]
Nagano	Neonatal [1.18]	Dermatitis [1.26]	Back Pain [1.3]	Headaches [0.74]	Conduct [1.04]	Anxiety [0.8]	Falls [1.36]	Depression [0.93]	Asthma [0.82]	Iron [3.15]
Nagasaki	Neonatal [1.02]	Dermatitis [1.28]	Back Pain [1.25]	Headaches [0.72]	Conduct [1.0]	Anxiety [0.81]	Falls [1.42]	Congenital [1.12]	Depression [0.89]	Asthma [0.8]
Nara	Neonatal [1.0]	Dermatitis [1.26]	Back Pain [1.37]	Headaches [0.76]	Conduct [1.05]	Anxiety [0.82]	Falls [1.38]	Congenital [1.23]	Depression [0.95]	Asthma [0.81]
Niigata	Neonatal [1.14]	Dermatitis [1.26]	Back Pain [1.47]	Headaches [0.74]	Conduct [1.03]	Anxiety [0.81]	Falls [1.37]	Depression [0.99]	Congenital [1.06]	Asthma [0.81]
Oita	Neonatal [1.19]	Dermatitis [1.27]	Back Pain [1.26]	Headaches [0.72]	Conduct [1.02]	Anxiety [0.79]	Falls [1.34]	Depression [0.91]	Asthma [0.8]	Iron [2.79]
Okayama	Neonatal [1.2]	Dermatitis [1.26]	Back Pain [1.29]	Headaches [0.74]	Conduct [1.03]	Anxiety [0.79]	Falls [1.31]	Depression [0.91]	Asthma [0.81]	Acne [1.34]
Okinawa	Neonatal [1.08]	Dermatitis [1.3]	Back Pain [1.19]	Headaches [0.68]	Conduct [0.96]	Anxiety [0.78]	Falls [1.42]	Depression [0.84]	Iron [2.34]	Asthma [0.67]
Osaka	Neonatal [1.07]	Dermatitis [1.26]	Back Pain [1.3]	Headaches [0.74]	Conduct [1.04]	Anxiety [0.79]	Falls [1.3]	Depression [0.98]	Congenital [1.09]	Asthma [0.83]
Saga	Neonatal [1.07]	Dermatitis [1.28]	Back Pain [1.27]	Headaches [0.72]	Conduct [1.01]	Anxiety [0.8]	Falls [1.38]	Depression [0.88]	Asthma [0.81]	Congenital [0.91]
Saitama	Neonatal [1.1]	Dermatitis [1.27]	Back Pain [1.33]	Headaches [0.73]	Conduct [1.02]	Anxiety [0.8]	Falls [1.36]	Depression [0.96]	Asthma [0.82]	Acne [1.35]
Shiga	Dermatitis [1.55]	Neonatal [1.06]	Back Pain [1.27]	Headaches [0.73]	Conduct [1.04]	Anxiety [0.78]	Falls [1.27]	Depression [0.89]	Congenital [1.03]	Asthma [0.83]
Shimane	Neonatal [1.05]	Dermatitis [1.27]	Back Pain [1.19]	Headaches [0.71]	Conduct [1.02]	Anxiety [0.8]	Falls [1.4]	Depression [0.93]	Asthma [0.79]	Iron [2.45]
Shizuoka	Neonatal [1.24]	Dermatitis [1.27]	Back Pain [1.25]	Headaches [0.72]	Conduct [1.03]	Anxiety [0.79]	Falls [1.29]	Depression [0.9]	Asthma [0.82]	Iron [3.14]
Tochigi	Neonatal [1.19]	Dermatitis [1.27]	Back Pain [1.29]	Headaches [0.73]	Conduct [1.03]	Anxiety [0.79]	Congenital [1.37]	Falls [1.31]	Depression [0.91]	Asthma [0.82]
Tokushima	Neonatal [1.12]	Dermatitis [1.26]	Back Pain [1.29]	Headaches [0.74]	Conduct [1.02]	Anxiety [0.81]	Falls [1.34]	Depression [0.93]	Asthma [0.8]	Congenital [0.93]
Tokyo	Neonatal [1.22]	Dermatitis [1.31]	Back Pain [1.19]	Headaches [0.71]	Conduct [1.02]	Anxiety [0.71]	Falls [1.09]	Congenital [1.26]	Depression [0.83]	Asthma [0.89]
Tottori	Neonatal [1.14]	Dermatitis [1.28]	Back Pain [1.26]	Headaches [0.67]	Conduct [1.01]	Anxiety [0.8]	Falls [1.38]	Depression [0.9]	Congenital [1.08]	Asthma [0.81]
Toyama	Neonatal [1.09]	Dermatitis [1.26]	Back Pain [1.28]	Headaches [0.73]	Conduct [1.05]	Anxiety [0.8]	Falls [1.3]	Depression [0.93]	Iron [3.7]	Asthma [0.82]
Wakayama	Neonatal [1.17]	Back Pain [1.48]	Dermatitis [1.26]	Headaches [0.73]	Conduct [1.03]	Anxiety [0.81]	Falls [1.38]	Depression [0.94]	Asthma [0.8]	Iron [2.83]
Yamagata	Neonatal [0.96]	Dermatitis [1.26]	Back Pain [1.34]	Headaches [0.74]	Conduct [1.04]	Anxiety [0.83]	Falls [1.45]	Depression [0.92]	Iron [2.93]	Asthma [0.8]
Yamaguchi	Neonatal [0.99]	Dermatitis [1.27]	Back Pain [1.28]	Headaches [0.73]	Conduct [1.03]	Anxiety [0.8]	Falls [1.33]	Depression [0.92]	Asthma [0.81]	Acne [1.35]
Yamanashi	Neonatal [1.24]	Dermatitis [1.24]	Back Pain [1.36]	Headaches [0.76]	Conduct [1.07]	Anxiety [0.82]	Falls [1.35]	Depression [0.96]	Congenital [0.99]	Asthma [0.81]

eFigure 9a. Leading ten causes of YLDs with the ratio of observed YLDs to YLDs expected on the basis of Socio-Demographic Index alone in 2017, <20 years, both sexes combined.
The top ten causes contributing to YLDs are listed globally, by socio-demographic quintile, and then by GBD superregion, region, country, and subnationally where modeled. For each cell, the ratio of observed YLDs to YLDs expected on the basis of socio-demographic index (SDI) alone are listed. Abbreviations: YLD=year of life lived with disability, GBD=Global Burden of Disease.

Values shown in brackets represent the ratio of observed YLDs to predicted YLDs on the basis of Socio-Demographic Index (SDI), rounded to two (2) digits. Color ranges (shown below) were calculated to place a roughly equal number of cells into each bin.

COLOR KEY:	[0.0-0.78]	[0.78-0.88]	[0.88-0.94]	[0.94-1.0]	[1.0-1.06]	[1.06-1.18]	[1.18-1.38]	[1.38-1.66]	1.66+

	1	2	3	4	5	6	7	8	9	10
S Korea	Neonatal [1.17]	Headaches [0.89]	Dermatitis [1.08]	Back Pain [1.08]	Anxiety [0.85]	Conduct [0.9]	Depression [0.99]	Falls [1.0]	Asthma [0.8]	Acne [1.34]
Singapore	Neonatal [1.17]	Dermatitis [1.02]	Headaches [0.71]	Depression [1.2]	Anxiety [0.8]	Back Pain [0.88]	Conduct [0.87]	Falls [1.03]	Asthma [0.8]	Acne [1.21]
**High-income North America**	Dermatitis [1.34]	Headaches [1.01]	Depression [1.77]	Drugs [5.58]	Neonatal [0.89]	Anxiety [1.01]	Conduct [1.19]	Asthma [1.16]	Back Pain [0.83]	Skin Viral [1.29]
Canada	Dermatitis [1.45]	Headaches [1.0]	Drugs [4.89]	Depression [1.28]	Asthma [1.3]	Anxiety [0.8]	Conduct [0.98]	Back Pain [0.91]	Neonatal [0.62]	Skin Viral [1.39]
Greenland	Depression [2.35]	Dermatitis [1.39]	Headaches [0.97]	Anxiety [0.99]	Asthma [1.26]	Drugs [4.34]	Neonatal [0.61]	Conduct [0.96]	Back Pain [0.77]	Skin Viral [1.3]
USA	Dermatitis [1.33]	Depression [1.82]	Headaches [1.01]	Drugs [5.66]	Neonatal [0.91]	Anxiety [1.03]	Conduct [1.21]	Asthma [1.14]	Back Pain [0.82]	Congenital [0.88]
Alabama	Drugs [6.99]	Headaches [1.04]	Depression [1.82]	Neonatal [0.89]	Dermatitis [1.16]	Anxiety [1.08]	Conduct [1.18]	Back Pain [1.08]	Asthma [1.08]	Skin Viral [1.28]
Alaska	Headaches [0.95]	Depression [1.71]	Dermatitis [1.2]	Drugs [5.48]	Neonatal [0.86]	Anxiety [0.99]	Conduct [1.16]	Back Pain [0.88]	Asthma [0.88]	Congenital [0.97]
Arizona	Drugs [7.48]	Depression [2.1]	Dermatitis [1.36]	Headaches [1.03]	Neonatal [0.9]	Anxiety [1.07]	Conduct [1.19]	Asthma [1.22]	Back Pain [0.91]	Congenital [0.85]
Arkansas	Depression [2.11]	Drugs [6.98]	Headaches [1.02]	Anxiety [1.08]	Dermatitis [1.07]	Neonatal [0.79]	Conduct [1.18]	Back Pain [0.83]	Asthma [0.83]	Skin Viral [1.09]
California	Dermatitis [1.49]	Headaches [1.02]	Depression [1.76]	Anxiety [1.0]	Conduct [1.2]	Neonatal [0.76]	Drugs [4.22]	Asthma [1.12]	Back Pain [0.7]	Acne [1.3]
Colorado	Depression [2.02]	Headaches [1.03]	Dermatitis [1.19]	Neonatal [0.92]	Anxiety [1.02]	Drugs [4.86]	Conduct [1.22]	Asthma [1.14]	Back Pain [0.83]	Oth MSK [6.32]
Connecticut	Drugs [7.31]	Dermatitis [1.49]	Neonatal [1.08]	Headaches [1.07]	Depression [1.67]	Anxiety [1.03]	Conduct [1.25]	Asthma [1.52]	Back Pain [0.72]	Skin Viral [1.59]
Delaware	Drugs [7.73]	Depression [1.92]	Headaches [1.04]	Dermatitis [1.19]	Neonatal [0.9]	Anxiety [1.04]	Conduct [1.2]	Asthma [1.25]	Back Pain [0.72]	Skin Viral [1.36]
DC	Dermatitis [1.85]	Neonatal [0.94]	Headaches [0.89]	Drugs [4.89]	Anxiety [0.89]	Depression [1.31]	Conduct [1.03]	Asthma [1.25]	Skin Viral [1.45]	Congenital [0.94]
Florida	Dermatitis [1.31]	Neonatal [1.01]	Headaches [1.03]	Depression [1.8]	Drugs [5.89]	Anxiety [1.04]	Conduct [1.2]	Asthma [1.21]	Back Pain [0.73]	Psoriasis [3.51]
Georgia	Dermatitis [1.43]	Headaches [1.04]	Depression [1.76]	Drugs [5.49]	Neonatal [0.84]	Anxiety [1.06]	Conduct [1.2]	Asthma [1.17]	Back Pain [0.82]	Acne [1.32]
Hawaii	Dermatitis [1.58]	Headaches [0.97]	Depression [1.71]	Neonatal [0.83]	Anxiety [0.99]	Conduct [1.16]	Asthma [1.26]	Drugs [2.76]	Skin Viral [1.5]	Back Pain [0.56]
Idaho	Depression [2.02]	Headaches [1.01]	Drugs [5.52]	Dermatitis [1.1]	Anxiety [1.06]	Conduct [1.19]	Neonatal [0.74]	Back Pain [0.85]	Skin Viral [1.4]	Oth MSK [6.95]
Illinois	Neonatal [1.06]	Headaches [1.03]	Depression [1.67]	Dermatitis [1.16]	Anxiety [1.03]	Drugs [4.88]	Conduct [1.21]	Back Pain [0.92]	Asthma [1.07]	Oth MSK [6.54]
Indiana	Drugs [6.73]	Depression [1.94]	Headaches [1.02]	Dermatitis [1.15]	Neonatal [0.86]	Anxiety [1.06]	Conduct [1.19]	Back Pain [0.97]	Asthma [1.1]	Congenital [0.96]
Iowa	Headaches [1.01]	Back Pain [1.44]	Depression [1.71]	Neonatal [0.87]	Anxiety [1.03]	Dermatitis [1.05]	Conduct [1.19]	Oth MSK [7.75]	Drugs [2.81]	Skin Viral [1.36]
Kansas	Depression [1.8]	Headaches [1.0]	Neonatal [0.93]	Dermatitis [1.11]	Anxiety [1.02]	Conduct [1.19]	Back Pain [1.11]	Drugs [3.49]	Asthma [1.11]	Oth MSK [6.32]
Kentucky	Drugs [10.24]	Depression [1.94]	Headaches [1.01]	Dermatitis [1.17]	Neonatal [0.88]	Anxiety [1.07]	Conduct [1.17]	Back Pain [1.1]	Asthma [1.13]	Oth MSK [7.22]
Louisiana	Drugs [7.39]	Headaches [0.99]	Dermatitis [1.26]	Depression [1.69]	Neonatal [0.82]	Anxiety [1.04]	Conduct [1.15]	Back Pain [0.85]	Skin Viral [1.66]	Asthma [0.9]
Maine	Drugs [7.65]	Depression [1.97]	Headaches [1.06]	Neonatal [0.91]	Anxiety [1.06]	Conduct [1.23]	Dermatitis [0.99]	Asthma [1.25]	Back Pain [0.92]	Oth MSK [8.65]
Maryland	Dermatitis [1.51]	Neonatal [1.06]	Headaches [1.01]	Depression [1.7]	Anxiety [1.0]	Drugs [4.49]	Conduct [1.21]	Asthma [1.29]	Back Pain [0.64]	Psoriasis [3.09]
Massachusetts	Drugs [7.16]	Dermatitis [1.35]	Headaches [1.06]	Neonatal [1.04]	Depression [1.79]	Anxiety [1.01]	Conduct [1.24]	Asthma [1.32]	Back Pain [0.69]	Skin Viral [1.51]
Michigan	Drugs [6.49]	Depression [1.92]	Dermatitis [1.19]	Neonatal [0.9]	Anxiety [1.05]	Headaches [0.85]	Conduct [1.21]	Asthma [1.23]	Back Pain [0.81]	Skin Viral [1.56]
Minnesota	Headaches [1.01]	Depression [1.74]	Neonatal [0.96]	Anxiety [1.0]	Dermatitis [1.05]	Conduct [1.21]	Back Pain [0.71]	Asthma [0.95]	Drugs [2.79]	Skin Viral [1.49]
Mississippi	Drugs [6.5]	Headaches [1.03]	Depression [1.77]	Neonatal [0.85]	Anxiety [1.1]	Dermatitis [1.06]	Conduct [1.18]	Back Pain [0.76]	Asthma [0.75]	Skin Viral [1.04]

eFigure 9a. Leading ten causes of YLDs with the ratio of observed YLDs to YLDs expected on the basis of Socio-Demographic Index alone in 2017, <20 years, both sexes combined. The top ten causes contributing to YLDs are listed globally, by socio-demographic quintile, and then by GBD superregion, region, country, and subnationally where modeled. For each cell, the ratio of observed YLDs to YLDs expected on the basis of socio-demographic index (SDI) alone are listed. Abbreviations: YLD=year of life lived with disability, GBD=Global Burden of Disease.

Values shown in brackets represent the ratio of observed YLDs to predicted YLDs on the basis of Socio-Demographic Index (SDI), rounded to two (2) digits. Color ranges (shown below) were calculated to place a roughly equal number of cells into each bin.

COLOR KEY:	[0.0-0.78]	(0.78-0.88]	(0.88-0.94]	(0.94-1.0]	(1.0-1.06]	(1.06-1.18]	(1.18-1.38]	(1.38-1.66]	1.66+

	1	2	3	4	5	6	7	8	9	10
Missouri	Depression [1.94]	Headaches [1.01]	Drugs [5.71]	Neonatal [0.92]	Anxiety [1.04]	Dermatitis [1.07]	Conduct [1.18]	Back Pain [0.9]	Asthma [1.07]	Oth MSK [6.64]
Montana	Depression [1.87]	Headaches [0.99]	Neonatal [0.89]	Drugs [5.19]	Anxiety [1.02]	Dermatitis [1.05]	Conduct [1.18]	Back Pain [0.92]	Oth MSK [7.24]	Asthma [0.83]
Nebraska	Headaches [0.99]	Depression [1.65]	Neonatal [0.88]	Dermatitis [1.06]	Anxiety [1.0]	Back Pain [1.19]	Conduct [1.18]	Drugs [2.9]	Asthma [0.75]	Congenital [0.91]
Nevada	Drugs [7.24]	Depression [1.88]	Dermatitis [1.3]	Headaches [1.0]	Neonatal [0.85]	Anxiety [1.04]	Conduct [1.17]	Asthma [1.0]	Back Pain [0.7]	Oth MSK [5.42]
New Hampshire	Drugs [8.33]	Depression [2.06]	Headaches [1.08]	Anxiety [1.04]	Neonatal [0.87]	Dermatitis [1.04]	Conduct [1.27]	Back Pain [0.87]	Asthma [1.16]	Oth MSK [6.8]
New Jersey	Dermatitis [1.62]	Headaches [1.03]	Neonatal [0.98]	Drugs [5.53]	Depression [1.53]	Anxiety [1.01]	Asthma [1.52]	Conduct [1.22]	Back Pain [0.78]	Congenital [1.07]
New Mexico	Depression [2.11]	Drugs [6.56]	Headaches [1.02]	Neonatal [0.95]	Dermatitis [1.17]	Anxiety [1.08]	Conduct [1.19]	Asthma [1.22]	Back Pain [0.98]	Oth MSK [6.71]
New York	Dermatitis [1.68]	Depression [1.84]	Neonatal [1.02]	Headaches [1.01]	Drugs [4.99]	Asthma [1.65]	Anxiety [0.99]	Conduct [1.19]	Back Pain [0.68]	Acne [1.45]
N Carolina	Headaches [1.03]	Depression [1.79]	Dermatitis [1.23]	Drugs [5.79]	Neonatal [0.91]	Anxiety [0.99]	Conduct [1.11]	Asthma [1.0]	Back Pain [0.73]	Skin Viral [1.25]
N Dakota	Neonatal [0.96]	Headaches [0.93]	Depression [1.49]	Dermatitis [1.04]	Anxiety [0.94]	Conduct [1.12]	Back Pain [0.93]	Oth MSK [8.67]	Congenital [1.17]	Asthma [0.95]
Ohio	Drugs [8.24]	Depression [1.86]	Headaches [1.03]	Dermatitis [1.26]	Neonatal [0.87]	Anxiety [1.05]	Conduct [1.19]	Asthma [1.15]	Back Pain [0.88]	Congenital [0.99]
Oklahoma	Drugs [7.74]	Depression [1.9]	Dermatitis [1.32]	Headaches [1.01]	Neonatal [0.82]	Anxiety [1.05]	Conduct [1.17]	Back Pain [0.98]	Asthma [1.19]	Oth MSK [7.14]
Oregon	Drugs [7.2]	Depression [1.99]	Dermatitis [1.39]	Headaches [1.02]	Neonatal [0.78]	Anxiety [0.95]	Conduct [1.08]	Back Pain [0.76]	Asthma [0.91]	Oth MSK [6.66]
Pennsylvania	Drugs [8.0]	Dermatitis [1.3]	Depression [1.8]	Headaches [0.94]	Neonatal [0.91]	Anxiety [1.03]	Conduct [1.21]	Back Pain [1.05]	Asthma [1.29]	Oth MSK [7.16]
Rhode Island	Drugs [9.2]	Depression [2.02]	Headaches [1.07]	Dermatitis [1.18]	Neonatal [0.88]	Anxiety [1.04]	Conduct [1.23]	Asthma [1.25]	Back Pain [0.7]	Skin Viral [1.47]
S Carolina	Drugs [7.01]	Dermatitis [1.42]	Depression [1.88]	Headaches [1.03]	Neonatal [0.85]	Anxiety [1.07]	Conduct [1.19]	Back Pain [0.88]	Asthma [1.03]	Skin Viral [1.28]
S Dakota	Headaches [0.96]	Depression [1.52]	Dermatitis [1.03]	Anxiety [1.0]	Back Pain [1.17]	Neonatal [0.78]	Conduct [1.16]	Oth MSK [7.81]	Drugs [2.67]	Skin Viral [1.45]
Tennessee	Drugs [8.26]	Depression [1.9]	Headaches [1.02]	Dermatitis [1.18]	Neonatal [0.85]	Anxiety [1.06]	Conduct [1.17]	Back Pain [0.85]	Asthma [0.88]	ASD [1.51]
Texas	Dermatitis [1.39]	Headaches [1.01]	Neonatal [0.97]	Depression [1.76]	Conduct [1.36]	Anxiety [1.06]	Asthma [1.18]	Drugs [3.63]	Back Pain [0.8]	ASD [1.63]
Utah	Depression [2.14]	Drugs [6.41]	Headaches [1.0]	Dermatitis [1.12]	Neonatal [0.83]	Anxiety [1.03]	Conduct [1.18]	Back Pain [0.81]	ASD [1.92]	Skin Viral [1.49]
Vermont	Drugs [6.23]	Headaches [1.08]	Depression [1.91]	Neonatal [0.97]	Anxiety [1.05]	Dermatitis [1.02]	Conduct [1.26]	Back Pain [0.8]	Asthma [1.04]	Oth MSK [6.17]
Virginia	Dermatitis [1.51]	Depression [1.9]	Headaches [1.02]	Neonatal [0.99]	Anxiety [1.01]	Drugs [4.65]	Conduct [1.2]	Asthma [1.16]	Back Pain [0.74]	Psoriasis [2.85]
Washington	Dermatitis [1.36]	Depression [1.92]	Headaches [1.0]	Drugs [5.49]	Neonatal [0.84]	Anxiety [1.0]	Conduct [1.19]	Back Pain [0.81]	Oth MSK [7.13]	Asthma [0.87]
W Virginia	Drugs [12.4]	Depression [1.97]	Headaches [1.02]	Dermatitis [1.19]	Neonatal [0.86]	Anxiety [1.08]	Conduct [1.17]	Asthma [1.15]	Back Pain [0.93]	Congenital [0.78]
Wisconsin	Depression [1.92]	Headaches [1.03]	Drugs [5.99]	Neonatal [0.96]	Anxiety [1.03]	Dermatitis [1.05]	Conduct [1.21]	Back Pain [0.95]	Asthma [0.89]	Congenital [0.95]
Wyoming	Depression [1.98]	Headaches [0.99]	Dermatitis [1.1]	Anxiety [1.01]	Back Pain [1.13]	Conduct [1.2]	Neonatal [0.74]	Drugs [3.42]	Asthma [0.88]	Oth MSK [6.74]
**Southern Latin America**	Neonatal [0.96]	Anxiety [1.34]	Back Pain [1.3]	Dermatitis [1.03]	Depression [1.33]	Headaches [0.64]	Diarrhea [2.29]	Conduct [0.91]	Asthma [0.94]	Congenital [0.75]
Argentina	Neonatal [0.94]	Anxiety [1.35]	Dermatitis [0.97]	Back Pain [1.17]	Depression [1.3]	Diarrhea [2.36]	Conduct [0.91]	Headaches [0.61]	Asthma [0.92]	Congenital [0.76]
Chile	Neonatal [1.01]	Back Pain [1.71]	Anxiety [1.3]	Dermatitis [1.21]	Depression [1.46]	Headaches [0.69]	Conduct [0.9]	Diarrhea [2.26]	Asthma [0.96]	Congenital [0.73]
Uruguay	Neonatal [1.0]	Anxiety [1.4]	Dermatitis [0.95]	Headaches [0.67]	Back Pain [1.07]	Conduct [0.93]	Asthma [1.06]	Depression [1.12]	Iron [0.92]	Diarrhea [1.17]
**Western Europe**	Anxiety [1.23]	Headaches [0.97]	Dermatitis [1.12]	Neonatal [0.83]	Back Pain [1.08]	Depression [1.08]	Conduct [0.91]	Asthma [0.96]	Congenital [1.04]	Acne [1.31]

eFigure 9a. Leading ten causes of YLDs with the ratio of observed YLDs to YLDs expected on the basis of Socio-Demographic Index alone in 2017, <20 years, both sexes combined.
The top ten causes contributing to YLDs are listed globally, by socio-demographic quintile, and then by GBD superregion, region, country, and subnationally where modeled. For each cell, the ratio of observed YLDs to YLDs expected on the basis of socio-demographic index (SDI) alone are listed. Abbreviations: YLD=year of life lived with disability, GBD=Global Burden of Disease.

Values shown in brackets represent the ratio of observed YLDs to predicted YLDs on the basis of Socio-Demographic Index (SDI), rounded to two (2) digits. Color ranges (shown below) were calculated to place a roughly equal number of cells into each bin.

COLOR KEY:	[0.0-0.78]	(0.78-0.88]	(0.88-0.94]	(0.94-1.0]	(1.0-1.06]	(1.06-1.18]	(1.18-1.38]	(1.38-1.66]	1.66+

	1	2	3	4	5	6	7	8	9	10
Andorra	Dermatitis [1.4]	Headaches [1.07]	Anxiety [1.21]	Neonatal [0.86]	Back Pain [1.1]	Depression [1.11]	Conduct [0.98]	Asthma [1.12]	Congenital [1.06]	Acne [1.32]
Austria	Neonatal [1.37]	Headaches [1.02]	Anxiety [1.17]	Dermatitis [0.76]	Back Pain [0.85]	Conduct [0.89]	Congenital [1.06]	Asthma [0.85]	Depression [0.77]	Falls [0.93]
Belgium	Headaches [1.2]	Anxiety [1.1]	Dermatitis [0.96]	Back Pain [0.92]	Neonatal [0.58]	Conduct [0.89]	Depression [1.01]	Falls [1.0]	Congenital [1.02]	Asthma [0.77]
Cyprus	Neonatal [1.09]	Headaches [0.97]	Anxiety [1.15]	Back Pain [1.07]	Dermatitis [0.8]	Conduct [0.88]	Depression [0.98]	Asthma [0.87]	Congenital [1.0]	Falls [0.9]
Denmark	Dermatitis [1.32]	Anxiety [1.15]	Back Pain [1.37]	Headaches [0.85]	Neonatal [0.69]	Conduct [0.92]	Asthma [1.0]	Congenital [1.16]	Depression [0.81]	Acne [1.2]
Finland	Neonatal [1.72]	Dermatitis [1.39]	Headaches [0.98]	Depression [1.34]	Anxiety [0.83]	Conduct [0.89]	Asthma [1.0]	Congenital [1.07]	Falls [0.96]	Back Pain [0.62]
France	Anxiety [1.4]	Dermatitis [1.25]	Headaches [0.81]	Back Pain [1.06]	Neonatal [0.66]	Depression [1.12]	Conduct [0.9]	Asthma [0.95]	Congenital [1.03]	Acne [1.45]
Germany	Anxiety [1.41]	Neonatal [0.96]	Back Pain [1.41]	Headaches [0.88]	Dermatitis [0.92]	Depression [1.06]	Conduct [0.91]	Congenital [1.0]	Falls [0.92]	Epilepsy [2.0]
Greece	Headaches [1.18]	Anxiety [1.34]	Neonatal [0.85]	Depression [1.37]	Back Pain [0.95]	Conduct [0.88]	Dermatitis [0.66]	Asthma [0.81]	Congenital [0.99]	Acne [1.47]
Iceland	Dermatitis [1.45]	Headaches [0.97]	Anxiety [1.11]	Neonatal [0.89]	Asthma [1.5]	Back Pain [0.98]	Conduct [0.9]	Depression [0.85]	Congenital [0.99]	Acne [1.22]
Ireland	Anxiety [1.23]	Dermatitis [1.19]	Headaches [0.94]	Neonatal [0.74]	Asthma [1.29]	Depression [1.14]	Conduct [0.88]	Back Pain [0.76]	Congenital [1.14]	Falls [0.9]
Israel	Neonatal [0.95]	Headaches [0.88]	Dermatitis [1.06]	Back Pain [1.05]	Depression [1.04]	Blindness [2.78]	Anxiety [0.69]	Conduct [0.81]	Congenital [1.03]	Asthma [0.84]
Italy	Headaches [1.24]	Anxiety [1.28]	Dermatitis [0.94]	Back Pain [1.03]	Neonatal [0.65]	Depression [1.11]	Conduct [0.9]	Congenital [1.0]	Acne [1.39]	URI [1.2]
Luxembourg	Neonatal [1.15]	Headaches [1.13]	Dermatitis [1.41]	Anxiety [1.11]	Back Pain [0.96]	Asthma [1.19]	Conduct [0.91]	Depression [0.94]	Congenital [1.07]	Acne [1.22]
Malta	Headaches [0.97]	Anxiety [1.18]	Neonatal [0.72]	Back Pain [1.08]	Dermatitis [0.82]	Conduct [0.85]	Depression [1.0]	Congenital [1.21]	Asthma [0.95]	Falls [1.18]
Netherlands	Anxiety [1.41]	Headaches [1.08]	Dermatitis [1.22]	Neonatal [0.88]	Conduct [1.03]	Depression [1.11]	Back Pain [0.81]	Asthma [1.06]	Congenital [1.09]	Acne [1.28]
Norway	Anxiety [1.55]	Dermatitis [1.37]	Neonatal [1.05]	Headaches [1.03]	Asthma [1.41]	Back Pain [0.91]	Conduct [0.98]	Congenital [1.47]	Depression [0.98]	Falls [1.06]
Portugal	Headaches [1.07]	Anxiety [1.35]	Back Pain [1.19]	Depression [1.4]	Asthma [1.37]	Dermatitis [0.86]	Neonatal [0.59]	Conduct [0.91]	Acne [1.6]	Congenital [0.82]
Spain	Headaches [1.15]	Anxiety [1.15]	Back Pain [1.15]	Neonatal [0.7]	Conduct [0.88]	Depression [1.02]	Dermatitis [0.71]	Asthma [0.78]	Congenital [0.95]	Falls [0.95]
Sweden	Dermatitis [1.76]	Headaches [0.99]	Anxiety [1.09]	Neonatal [0.78]	Asthma [1.42]	Depression [1.13]	Conduct [0.95]	Back Pain [0.85]	Congenital [1.23]	Falls [1.09]
Stockholm	Dermatitis [1.42]	Headaches [0.95]	Anxiety [1.04]	Neonatal [0.76]	Asthma [1.25]	Conduct [0.95]	Congenital [1.29]	Depression [0.88]	Falls [0.99]	Acne [1.27]
Sweden w/o Stockholm	Dermatitis [1.86]	Headaches [1.0]	Anxiety [1.11]	Neonatal [0.78]	Asthma [1.46]	Depression [1.2]	Back Pain [0.95]	Conduct [0.95]	Congenital [1.22]	Falls [1.12]
Switzerland	Back Pain [1.96]	Dermatitis [1.41]	Anxiety [1.12]	Neonatal [0.85]	Headaches [0.81]	Conduct [0.89]	Depression [0.92]	Asthma [0.99]	Congenital [1.04]	Acne [1.21]
UK	Dermatitis [1.5]	Neonatal [0.99]	Headaches [0.88]	Anxiety [1.0]	Asthma [1.51]	Conduct [0.94]	Depression [1.05]	Back Pain [0.86]	Congenital [1.16]	Falls [0.98]
England	Dermatitis [1.53]	Neonatal [1.02]	Headaches [0.86]	Asthma [1.53]	Anxiety [0.97]	Conduct [0.95]	Back Pain [0.86]	Depression [1.03]	Congenital [1.16]	Falls [0.96]
E Midlands	Dermatitis [1.5]	Neonatal [0.95]	Headaches [0.88]	Anxiety [1.0]	Asthma [1.45]	Conduct [0.96]	Back Pain [0.88]	Depression [1.06]	Congenital [1.19]	Falls [1.03]
Derby	Dermatitis [1.51]	Neonatal [0.97]	Headaches [0.84]	Asthma [1.46]	Anxiety [0.96]	Conduct [0.94]	Back Pain [0.85]	Depression [1.0]	Congenital [1.2]	Falls [0.97]
Derbyshire	Dermatitis [1.49]	Neonatal [0.88]	Headaches [0.9]	Anxiety [1.04]	Asthma [1.44]	Conduct [0.97]	Depression [1.09]	Back Pain [0.8]	Congenital [1.17]	Iron [2.39]
Leicester	Dermatitis [1.5]	Headaches [0.86]	Neonatal [0.83]	Asthma [1.45]	Anxiety [0.97]	Conduct [0.94]	Back Pain [0.88]	Depression [1.03]	Congenital [1.21]	Falls [0.99]
Leicestershire	Dermatitis [1.48]	Headaches [0.92]	Neonatal [0.8]	Anxiety [1.01]	Asthma [1.46]	Conduct [0.99]	Back Pain [0.94]	Depression [1.1]	Congenital [1.21]	Falls [1.01]
Lincolnshire	Dermatitis [1.5]	Neonatal [0.94]	Headaches [0.89]	Anxiety [1.03]	Asthma [1.43]	Conduct [0.95]	Back Pain [0.92]	Depression [1.07]	Congenital [1.17]	Falls [1.08]

eFigure 9a. Leading ten causes of YLDs with the ratio of observed YLDs to YLDs expected on the basis of Socio-Demographic Index alone in 2017, <20 years, both sexes combined. The top ten causes contributing to YLDs are listed globally, by socio-demographic quintile, and then by GBD superregion, region, country, and subnationally where modeled. For each cell, the ratio of observed YLDs to YLDs expected on the basis of socio-demographic index (SDI) alone are listed. Abbreviations: YLD=year of life lived with disability, GBD=Global Burden of Disease.

Values shown in brackets represent the ratio of observed YLDs to predicted YLDs on the basis of Socio-Demographic Index (SDI), rounded to two (2) digits. Color ranges (shown below) were calculated to place a roughly equal number of cells into each bin.

COLOR KEY:	[0.0-0.78]	(0.78-0.88]	(0.88-0.94]	(0.94-1.0]	(1.0-1.06]	(1.06-1.18]	(1.18-1.38]	(1.38-1.66]	1.66+

	1	2	3	4	5	6	7	8	9	10
Northamptonshire	Dermatitis [1.51]	Neonatal [1.05]	Headaches [0.85]	Anxiety [0.98]	Asthma [1.44]	Conduct [0.94]	Back Pain [0.87]	Depression [1.01]	Congenital [1.19]	Falls [1.01]
Nottingham	Neonatal [1.17]	Dermatitis [1.49]	Headaches [0.9]	Asthma [1.51]	Anxiety [0.96]	Back Pain [0.91]	Conduct [0.96]	Depression [1.07]	Congenital [1.22]	Falls [0.93]
Nottinghamshire	Dermatitis [1.5]	Neonatal [1.05]	Headaches [0.87]	Anxiety [1.01]	Asthma [1.43]	Conduct [0.95]	Back Pain [0.9]	Depression [1.05]	Congenital [1.18]	Falls [1.08]
Rutland	Dermatitis [1.45]	Headaches [0.99]	Anxiety [1.08]	Asthma [1.47]	Neonatal [0.67]	Back Pain [1.02]	Conduct [1.04]	Depression [1.2]	Congenital [1.18]	Acne [1.53]
E England	Dermatitis [1.5]	Neonatal [0.92]	Headaches [0.87]	Anxiety [0.98]	Asthma [1.46]	Conduct [0.96]	Back Pain [0.87]	Depression [1.03]	Congenital [1.09]	Falls [1.0]
Bedford	Dermatitis [1.5]	Neonatal [1.12]	Headaches [0.88]	Anxiety [0.99]	Asthma [1.45]	Conduct [0.96]	Back Pain [0.9]	Depression [1.05]	Congenital [1.09]	Falls [0.99]
Cambridgeshire	Dermatitis [1.5]	Neonatal [0.94]	Headaches [0.88]	Anxiety [0.96]	Asthma [1.5]	Conduct [0.98]	Depression [1.05]	Back Pain [0.82]	Congenital [1.13]	Falls [0.92]
Cen Bedfordshire	Dermatitis [1.51]	Neonatal [1.0]	Headaches [0.85]	Anxiety [0.98]	Asthma [1.44]	Conduct [0.95]	Back Pain [0.86]	Depression [1.02]	Congenital [1.06]	Falls [1.01]
Essex	Dermatitis [1.5]	Neonatal [0.9]	Headaches [0.88]	Anxiety [1.01]	Asthma [1.44]	Conduct [0.97]	Back Pain [0.89]	Depression [1.06]	Congenital [1.08]	Falls [1.03]
Hertfordshire	Dermatitis [1.51]	Neonatal [0.91]	Headaches [0.86]	Asthma [1.49]	Anxiety [0.95]	Conduct [0.97]	Back Pain [0.85]	Depression [1.01]	Congenital [1.1]	Falls [0.91]
Luton	Dermatitis [1.52]	Neonatal [1.04]	Headaches [0.82]	Asthma [1.45]	Anxiety [0.95]	Conduct [0.93]	Back Pain [0.83]	Depression [0.97]	Congenital [1.1]	Falls [0.99]
Norfolk	Dermatitis [1.5]	Headaches [0.88]	Neonatal [0.78]	Anxiety [1.01]	Asthma [1.44]	Conduct [0.96]	Back Pain [0.9]	Depression [1.06]	Congenital [1.08]	Falls [1.05]
Peterborough	Dermatitis [1.53]	Headaches [0.8]	Asthma [1.45]	Neonatal [0.74]	Anxiety [0.95]	Conduct [0.91]	Back Pain [0.82]	Depression [0.95]	Congenital [1.07]	Iron [2.33]
Southend-on-Sea	Dermatitis [1.52]	Neonatal [1.09]	Headaches [0.84]	Asthma [1.42]	Anxiety [0.99]	Conduct [0.93]	Back Pain [0.85]	Depression [1.01]	Congenital [1.07]	Iron [2.4]
Suffolk	Dermatitis [1.5]	Neonatal [0.95]	Headaches [0.88]	Anxiety [1.01]	Asthma [1.44]	Conduct [0.96]	Back Pain [0.89]	Depression [1.06]	Congenital [1.07]	Iron [2.57]
Thurrock	Dermatitis [1.53]	Neonatal [1.02]	Headaches [0.82]	Asthma [1.43]	Anxiety [0.99]	Conduct [0.92]	Back Pain [0.83]	Depression [0.99]	Congenital [1.06]	Falls [1.08]
Greater London	Dermatitis [1.54]	Neonatal [1.07]	Asthma [1.76]	Headaches [0.81]	Anxiety [0.88]	Conduct [0.93]	Back Pain [0.79]	Depression [0.94]	Congenital [1.17]	URI [1.26]
Barking & Dagenham	Dermatitis [2.0]	Neonatal [1.01]	Asthma [1.62]	Headaches [0.75]	Anxiety [0.93]	Iron [2.93]	Conduct [0.87]	Back Pain [0.78]	Depression [0.89]	Congenital [1.07]
Barnet	Dermatitis [1.52]	Neonatal [1.0]	Asthma [1.72]	Headaches [0.8]	Anxiety [0.91]	Conduct [0.93]	Back Pain [0.8]	Depression [0.95]	Congenital [1.1]	Falls [0.89]
Bexley	Dermatitis [1.5]	Neonatal [0.94]	Asthma [1.69]	Headaches [0.86]	Anxiety [0.99]	Conduct [0.94]	Back Pain [0.89]	Depression [1.04]	Congenital [1.07]	Falls [1.03]
Brent	Dermatitis [1.5]	Neonatal [1.06]	Asthma [1.7]	Headaches [0.81]	Anxiety [0.93]	Conduct [0.94]	Back Pain [0.83]	Depression [0.97]	Congenital [1.09]	Falls [0.92]
Bromley	Dermatitis [1.51]	Neonatal [1.12]	Asthma [1.7]	Headaches [0.84]	Anxiety [0.95]	Conduct [0.94]	Back Pain [0.85]	Depression [1.0]	Congenital [1.11]	Falls [0.95]
Camden	Dermatitis [1.52]	Asthma [1.84]	Neonatal [0.94]	Headaches [0.88]	Anxiety [0.89]	Conduct [0.96]	Back Pain [0.84]	Depression [1.02]	Congenital [1.2]	Acne [1.11]
Croydon	Dermatitis [1.53]	Neonatal [1.01]	Asthma [1.67]	Headaches [0.8]	Anxiety [0.93]	Conduct [0.89]	Back Pain [0.81]	Depression [0.94]	Congenital [1.1]	Iron [2.79]
Ealing	Dermatitis [1.53]	Neonatal [1.14]	Asthma [1.71]	Headaches [0.78]	Anxiety [0.89]	Conduct [0.91]	Back Pain [0.78]	Depression [0.92]	Congenital [1.1]	Iron [3.13]
Enfield	Dermatitis [1.52]	Neonatal [1.05]	Asthma [1.69]	Headaches [0.82]	Anxiety [0.95]	Conduct [0.93]	Back Pain [0.83]	Depression [0.97]	Congenital [1.09]	Falls [0.97]
Greenwich	Dermatitis [1.53]	Neonatal [0.99]	Asthma [1.66]	Headaches [0.79]	Anxiety [0.92]	Conduct [0.88]	Back Pain [0.81]	Depression [0.94]	Congenital [1.11]	URI [1.28]
Hackney	Dermatitis [1.52]	Neonatal [1.06]	Asthma [1.8]	Headaches [0.79]	Anxiety [0.87]	Conduct [0.9]	Back Pain [0.78]	Depression [0.92]	Congenital [1.16]	URI [1.26]
Hammersmith & Fulham	Dermatitis [1.55]	Neonatal [1.06]	Asthma [1.79]	Headaches [0.78]	Anxiety [0.83]	Conduct [0.9]	Back Pain [0.74]	Depression [0.9]	Congenital [1.2]	URI [1.27]
Haringey	Dermatitis [1.5]	Neonatal [1.1]	Asthma [1.7]	Headaches [0.84]	Anxiety [0.95]	Conduct [0.95]	Back Pain [0.85]	Depression [0.99]	Congenital [1.13]	Falls [0.92]
Harrow	Dermatitis [1.51]	Neonatal [1.15]	Asthma [1.7]	Headaches [0.83]	Anxiety [0.95]	Conduct [0.94]	Back Pain [0.84]	Depression [0.99]	Congenital [1.11]	Falls [0.94]

eFigure 9a. Leading ten causes of YLDs with the ratio of observed YLDs to YLDs expected on the basis of Socio-Demographic Index alone in 2017, <20 years, both sexes combined. The top ten causes contributing to YLDs are listed globally, by socio-demographic quintile, and then by GBD superregion, region, country, and subnationally where modeled. For each cell, the ratio of observed YLDs to YLDs expected on the basis of socio-demographic index (SDI) alone are listed. Abbreviations: YLD=year of life lived with disability, GBD=Global Burden of Disease.

Values shown in brackets represent the ratio of observed YLDs to predicted YLDs on the basis of Socio-Demographic Index (SDI), rounded to two (2) digits. Color ranges (shown below) were calculated to place a roughly equal number of cells into each bin.

COLOR KEY:	[0.0-0.78]	(0.78-0.88]	(0.88-0.94]	(0.94-1.0]	(1.0-1.06]	(1.06-1.18]	(1.18-1.38]	(1.38-1.66]	1.66+

	1	2	3	4	5	6	7	8	9	10
Havering	Dermatitis [1.5]	Neonatal [0.99]	Asthma [1.68]	Headaches [0.85]	Anxiety [0.98]	Conduct [0.93]	Back Pain [0.88]	Depression [1.03]	Congenital [1.08]	Falls [1.03]
Hillingdon	Dermatitis [1.51]	Neonatal [1.18]	Asthma [1.77]	Headaches [0.83]	Anxiety [0.91]	Conduct [0.94]	Back Pain [0.83]	Depression [0.98]	Congenital [1.13]	Falls [0.86]
Hounslow	Dermatitis [1.54]	Neonatal [1.12]	Asthma [1.73]	Headaches [0.77]	Anxiety [0.86]	Conduct [0.89]	Back Pain [0.75]	Depression [0.9]	Congenital [1.13]	URI [1.27]
Islington	Dermatitis [1.53]	Asthma [1.8]	Neonatal [0.92]	Headaches [0.82]	Anxiety [0.85]	Conduct [0.91]	Back Pain [0.79]	Depression [0.95]	Congenital [1.21]	URI [1.25]
Kensington & Chelsea	Dermatitis [1.53]	Asthma [1.81]	Neonatal [0.91]	Headaches [0.83]	Anxiety [0.85]	Conduct [0.94]	Back Pain [0.79]	Depression [0.96]	Congenital [1.2]	Falls [0.74]
Kingston upon Thames	Dermatitis [1.53]	Neonatal [1.12]	Asthma [1.76]	Headaches [0.84]	Anxiety [0.9]	Conduct [0.92]	Back Pain [0.82]	Depression [0.98]	Congenital [1.17]	URI [1.27]
Lambeth	Dermatitis [1.53]	Asthma [1.74]	Headaches [0.8]	Neonatal [0.78]	Anxiety [0.86]	Conduct [0.91]	Back Pain [0.77]	Depression [0.92]	Congenital [1.19]	URI [1.26]
Lewisham	Neonatal [1.22]	Dermatitis [1.53]	Asthma [1.66]	Headaches [0.76]	Anxiety [0.89]	Conduct [0.87]	Back Pain [0.77]	Depression [0.9]	Congenital [1.12]	URI [1.28]
Merton	Dermatitis [1.54]	Neonatal [1.07]	Asthma [1.7]	Headaches [0.75]	Anxiety [0.85]	Conduct [0.88]	Back Pain [0.74]	Congenital [1.15]	Depression [0.88]	Iron [3.27]
Newham	Dermatitis [1.51]	Neonatal [1.09]	Asthma [1.66]	Headaches [0.8]	Anxiety [0.92]	Conduct [0.89]	Back Pain [0.82]	Depression [0.95]	Congenital [1.11]	URI [1.28]
Redbridge	Dermatitis [1.52]	Neonatal [1.01]	Asthma [1.67]	Headaches [0.8]	Anxiety [0.94]	Conduct [0.91]	Back Pain [0.82]	Depression [0.95]	Congenital [1.16]	Iron [2.57]
Richmond upon Thames	Dermatitis [1.54]	Neonatal [1.18]	Asthma [1.76]	Headaches [0.79]	Anxiety [0.87]	Conduct [0.92]	Back Pain [0.76]	Depression [0.91]	Congenital [1.18]	URI [1.28]
Southwark	Dermatitis [1.54]	Asthma [1.77]	Neonatal [0.8]	Headaches [0.78]	Anxiety [0.84]	Conduct [0.9]	Back Pain [0.75]	Depression [0.9]	Congenital [1.21]	URI [1.27]
Sutton	Neonatal [1.28]	Dermatitis [1.52]	Asthma [1.69]	Headaches [0.82]	Anxiety [0.94]	Conduct [0.92]	Back Pain [0.83]	Depression [0.98]	Congenital [1.11]	Falls [0.95]
Tower Hamlets	Dermatitis [1.52]	Neonatal [1.16]	Asthma [1.79]	Headaches [0.79]	Anxiety [0.85]	Conduct [0.91]	Back Pain [0.77]	Depression [0.92]	Congenital [1.19]	URI [1.26]
Waltham Forest	Dermatitis [1.53]	Neonatal [1.02]	Asthma [1.63]	Headaches [0.77]	Anxiety [0.92]	Conduct [0.87]	Back Pain [0.79]	Depression [0.92]	Congenital [1.11]	URI [1.29]
Wandsworth	Dermatitis [1.55]	Neonatal [0.95]	Asthma [1.72]	Headaches [0.74]	Anxiety [0.8]	Conduct [0.86]	Congenital [1.22]	Back Pain [0.7]	Depression [0.85]	URI [1.28]
Westminster	Dermatitis [1.52]	Asthma [1.86]	Headaches [0.85]	Neonatal [0.85]	Anxiety [0.88]	Conduct [0.96]	Back Pain [0.82]	Depression [0.99]	Congenital [1.19]	Falls [0.77]
NE England	Dermatitis [1.61]	Neonatal [0.9]	Headaches [0.88]	Anxiety [1.01]	Asthma [1.45]	Conduct [0.95]	Congenital [1.35]	Back Pain [0.89]	Depression [1.05]	Iron [2.8]
County Durham	Dermatitis [1.78]	Neonatal [0.97]	Headaches [0.88]	Asthma [1.46]	Anxiety [1.02]	Conduct [0.94]	Congenital [1.33]	Depression [1.06]	Iron [3.0]	Back Pain [0.84]
Darlington	Dermatitis [1.5]	Neonatal [0.88]	Headaches [0.87]	Asthma [1.48]	Anxiety [1.0]	Conduct [0.96]	Congenital [1.35]	Back Pain [0.9]	Depression [1.04]	Iron [2.94]
Gateshead	Dermatitis [1.77]	Neonatal [1.01]	Headaches [0.85]	Asthma [1.46]	Anxiety [0.98]	Conduct [0.93]	Congenital [1.35]	Back Pain [0.88]	Depression [1.02]	Iron [3.11]
Hartlepool	Dermatitis [1.5]	Neonatal [1.02]	Headaches [0.88]	Asthma [1.46]	Anxiety [1.04]	Conduct [0.94]	Congenital [1.32]	Back Pain [0.91]	Depression [1.07]	Iron [2.04]
Middlesbrough	Dermatitis [1.51]	Headaches [0.85]	Neonatal [0.79]	Asthma [1.46]	Anxiety [0.99]	Conduct [0.92]	Congenital [1.34]	Back Pain [0.87]	Depression [1.02]	Iron [2.35]
Newcastle upon Tyne	Dermatitis [1.48]	Headaches [0.91]	Neonatal [0.86]	Asthma [1.53]	Anxiety [0.96]	Back Pain [0.92]	Conduct [0.96]	Depression [1.09]	Congenital [1.41]	Drugs [2.36]
N Tyneside	Dermatitis [1.5]	Neonatal [0.84]	Headaches [0.85]	Asthma [1.46]	Anxiety [0.98]	Conduct [0.94]	Congenital [1.36]	Back Pain [0.86]	Depression [1.01]	Iron [2.77]
Northumberland	Dermatitis [1.48]	Headaches [0.91]	Neonatal [0.86]	Anxiety [1.05]	Asthma [1.47]	Conduct [0.99]	Back Pain [0.94]	Congenital [1.34]	Depression [1.1]	Iron [2.58]
Redcar & Cleveland	Dermatitis [1.5]	Headaches [0.87]	Neonatal [0.81]	Asthma [1.44]	Anxiety [1.04]	Conduct [0.95]	Congenital [1.32]	Back Pain [0.91]	Depression [1.06]	Iron [2.23]
S Tyneside	Dermatitis [1.79]	Neonatal [0.86]	Headaches [0.87]	Asthma [1.43]	Anxiety [1.02]	Conduct [0.92]	Congenital [1.32]	Back Pain [0.9]	Depression [1.05]	Iron [2.57]
Stockton-on-Tees	Dermatitis [1.51]	Neonatal [1.1]	Headaches [0.85]	Asthma [1.47]	Anxiety [0.98]	Conduct [0.94]	Congenital [1.35]	Back Pain [0.87]	Depression [1.02]	Iron [2.8]
Sunderland	Dermatitis [1.77]	Headaches [0.89]	Neonatal [0.77]	Anxiety [1.02]	Asthma [1.3]	Conduct [0.96]	Back Pain [0.91]	Congenital [1.34]	Depression [1.07]	Iron [2.71]

eFigure 9a. Leading ten causes of YLDs with the ratio of observed YLDs to YLDs expected on the basis of Socio-Demographic Index alone in 2017, <20 years, both sexes combined.
The top ten causes contributing to YLDs are listed globally, by socio-demographic quintile, and then by GBD superregion, region, country, and subnationally where modeled. For each cell, the ratio of observed YLDs to YLDs expected on the basis of socio-demographic index (SDI) alone are listed. Abbreviations: YLD=year of life lived with disability, GBD=Global Burden of Disease.

Values shown in brackets represent the ratio of observed YLDs to predicted YLDs on the basis of Socio-Demographic Index (SDI), rounded to two (2) digits. Color ranges (shown below) were calculated to place a roughly equal number of cells into each bin.

COLOR KEY:	[0.0-0.78]	(0.78-0.88]	(0.88-0.94]	(0.94-1.0]	(1.0-1.06]	(1.06-1.18]	(1.18-1.38]	(1.38-1.66]	1.66+

	1	2	3	4	5	6	7	8	9	10
NW England	Dermatitis [1.5]	Neonatal [1.11]	Headaches [0.87]	Asthma [1.48]	Anxiety [0.98]	Conduct [0.95]	Back Pain [0.87]	Depression [1.05]	Congenital [1.28]	Falls [1.01]
Blackburn with Darwen	Dermatitis [1.51]	Neonatal [1.09]	Headaches [0.85]	Asthma [1.45]	Anxiety [1.01]	Conduct [0.94]	Back Pain [0.89]	Depression [1.02]	Congenital [1.21]	Iron [2.44]
Blackpool	Dermatitis [1.52]	Neonatal [1.05]	Headaches [0.87]	Asthma [1.46]	Anxiety [1.05]	Conduct [0.93]	Back Pain [0.91]	Depression [1.06]	Congenital [1.21]	Iron [1.78]
Bolton	Dermatitis [1.5]	Neonatal [1.04]	Headaches [0.85]	Asthma [1.44]	Anxiety [1.0]	Conduct [0.94]	Back Pain [0.9]	Depression [1.03]	Congenital [1.22]	Iron [2.34]
Bury	Dermatitis [1.51]	Neonatal [1.0]	Headaches [0.84]	Asthma [1.43]	Anxiety [0.99]	Conduct [0.94]	Back Pain [0.88]	Congenital [1.24]	Depression [1.0]	Iron [2.59]
Cheshire E	Neonatal [1.37]	Dermatitis [1.49]	Headaches [0.9]	Anxiety [0.99]	Asthma [1.52]	Conduct [1.0]	Back Pain [0.91]	Congenital [1.39]	Depression [1.07]	Falls [0.94]
Cheshire W & Chester	Dermatitis [1.49]	Neonatal [1.12]	Headaches [0.9]	Anxiety [0.99]	Asthma [1.49]	Conduct [0.98]	Back Pain [0.9]	Congenital [1.39]	Depression [1.07]	Falls [0.96]
Cumbria	Dermatitis [1.49]	Neonatal [1.09]	Headaches [0.9]	Anxiety [1.02]	Asthma [1.48]	Conduct [0.98]	Back Pain [0.92]	Depression [1.08]	Congenital [1.23]	Falls [1.04]
Halton	Dermatitis [1.51]	Neonatal [1.13]	Headaches [0.87]	Asthma [1.48]	Anxiety [1.01]	Conduct [0.96]	Back Pain [0.88]	Depression [1.04]	Congenital [1.23]	Iron [2.63]
Knowsley	Dermatitis [1.5]	Neonatal [1.08]	Headaches [0.87]	Asthma [1.48]	Anxiety [1.0]	Conduct [0.94]	Congenital [1.34]	Back Pain [0.89]	Depression [1.04]	Iron [2.29]
Lancashire	Dermatitis [1.49]	Neonatal [1.07]	Headaches [0.89]	Anxiety [1.0]	Asthma [1.47]	Conduct [0.96]	Depression [1.06]	Back Pain [0.84]	Congenital [1.24]	Falls [1.04]
Liverpool	Neonatal [1.22]	Dermatitis [1.49]	Headaches [0.9]	Asthma [1.48]	Anxiety [0.9]	Depression [1.24]	Congenital [1.41]	Conduct [0.94]	Back Pain [0.9]	Falls [0.97]
Manchester	Neonatal [1.23]	Dermatitis [1.51]	Headaches [0.84]	Asthma [1.54]	Anxiety [0.91]	Back Pain [0.87]	Conduct [0.93]	Congenital [1.32]	Depression [0.99]	Falls [0.85]
Oldham	Dermatitis [1.51]	Neonatal [0.82]	Headaches [0.86]	Asthma [1.47]	Anxiety [1.03]	Conduct [0.94]	Back Pain [0.91]	Depression [1.04]	Congenital [1.22]	Iron [1.95]
Rochdale	Dermatitis [1.51]	Neonatal [0.84]	Headaches [0.85]	Asthma [1.45]	Anxiety [1.02]	Conduct [0.93]	Back Pain [0.9]	Depression [1.03]	Congenital [1.21]	Iron [2.35]
Salford	Neonatal [1.25]	Dermatitis [1.53]	Headaches [0.8]	Asthma [1.47]	Anxiety [0.93]	Conduct [0.9]	Congenital [1.26]	Back Pain [0.82]	Depression [0.95]	Falls [0.96]
Sefton	Dermatitis [1.5]	Neonatal [1.1]	Headaches [0.87]	Asthma [1.45]	Anxiety [1.01]	Conduct [0.95]	Congenital [1.34]	Back Pain [0.89]	Depression [1.05]	Iron [2.41]
St Helens	Dermatitis [1.51]	Neonatal [1.11]	Headaches [0.86]	Asthma [1.44]	Anxiety [1.01]	Conduct [0.94]	Congenital [1.33]	Back Pain [0.89]	Depression [1.03]	Iron [2.15]
Stockport	Dermatitis [1.51]	Neonatal [0.94]	Headaches [0.85]	Asthma [1.47]	Anxiety [0.97]	Conduct [0.95]	Back Pain [0.87]	Depression [1.01]	Congenital [1.27]	Falls [0.98]
Tameside	Dermatitis [1.52]	Neonatal [0.91]	Headaches [0.83]	Asthma [1.44]	Anxiety [1.0]	Conduct [0.91]	Congenital [1.21]	Depression [1.0]	Back Pain [0.7]	Iron [1.98]
Trafford	Neonatal [1.23]	Dermatitis [1.51]	Headaches [0.87]	Asthma [1.53]	Anxiety [0.96]	Conduct [1.0]	Back Pain [0.87]	Depression [1.02]	Congenital [1.28]	Falls [0.9]
Warrington	Neonatal [1.3]	Dermatitis [1.5]	Headaches [0.87]	Asthma [1.52]	Anxiety [0.97]	Conduct [0.98]	Back Pain [0.88]	Depression [1.04]	Congenital [1.28]	Iron [3.16]
Wigan	Dermatitis [1.51]	Neonatal [1.1]	Headaches [0.86]	Asthma [1.44]	Anxiety [1.02]	Conduct [0.95]	Back Pain [0.9]	Depression [1.03]	Congenital [1.21]	Iron [2.18]
Wirral	Neonatal [1.23]	Dermatitis [1.5]	Headaches [0.88]	Anxiety [1.03]	Asthma [1.44]	Conduct [0.96]	Congenital [1.34]	Back Pain [0.9]	Depression [1.06]	Falls [1.1]
SE England	Dermatitis [1.64]	Neonatal [1.03]	Headaches [0.88]	Asthma [1.6]	Anxiety [0.98]	Conduct [0.97]	Back Pain [0.86]	Depression [1.04]	Congenital [1.16]	Falls [0.96]
Bracknell Forest	Dermatitis [1.52]	Headaches [0.84]	Neonatal [0.82]	Asthma [1.47]	Anxiety [0.93]	Conduct [0.95]	Back Pain [0.83]	Depression [0.99]	Congenital [1.17]	Falls [0.9]
Brighton & Hove	Dermatitis [1.74]	Neonatal [1.13]	Headaches [0.95]	Anxiety [0.98]	Asthma [1.5]	Conduct [1.01]	Back Pain [0.92]	Depression [1.12]	Congenital [1.21]	Acne [1.31]
Buckinghamshire	Neonatal [1.18]	Dermatitis [1.5]	Headaches [0.89]	Anxiety [0.98]	Asthma [1.46]	Conduct [0.99]	Back Pain [0.88]	Depression [1.06]	Congenital [1.17]	Falls [0.94]
E Sussex	Dermatitis [1.73]	Neonatal [1.08]	Headaches [0.91]	Anxiety [1.04]	Asthma [1.37]	Conduct [0.98]	Back Pain [0.93]	Depression [1.1]	Congenital [1.12]	Falls [1.09]
Hampshire	Asthma [2.23]	Dermatitis [1.49]	Neonatal [1.01]	Headaches [0.89]	Anxiety [0.99]	Conduct [0.98]	Back Pain [0.88]	Depression [1.05]	Congenital [1.15]	Falls [0.98]
Isle of Wight	Asthma [2.19]	Dermatitis [1.44]	Neonatal [0.93]	Headaches [0.93]	Anxiety [1.05]	Conduct [0.99]	Back Pain [0.95]	Depression [1.13]	Congenital [1.1]	Falls [1.1]

eFigure 9a. Leading ten causes of YLDs with the ratio of observed YLDs to YLDs expected on the basis of Socio-Demographic Index alone in 2017, <20 years, both sexes combined. The top ten causes contributing to YLDs are listed globally, by socio-demographic quintile, and then by GBD superregion, region, country, and subnationally where modeled. For each cell, the ratio of observed YLDs to YLDs expected on the basis of socio-demographic index (SDI) alone are listed. Abbreviations: YLD=year of life lived with disability, GBD=Global Burden of Disease.

Values shown in brackets represent the ratio of observed YLDs to predicted YLDs on the basis of Socio-Demographic Index (SDI), rounded to two (2) digits. Color ranges (shown below) were calculated to place a roughly equal number of cells into each bin.

COLOR KEY:	[0.0-0.78]	(0.78-0.88]	(0.88-0.94]	(0.94-1.0]	(1.0-1.06]	(1.06-1.18]	(1.18-1.38]	(1.38-1.66]	1.66+

	1	2	3	4	5	6	7	8	9	10
Kent	Dermatitis [1.73]	Neonatal [0.99]	Headaches [0.89]	Anxiety [1.01]	Asthma [1.41]	Conduct [0.97]	Back Pain [0.9]	Depression [1.07]	Congenital [1.13]	Falls [1.03]
Medway	Dermatitis [1.5]	Neonatal [1.05]	Headaches [0.87]	Anxiety [1.01]	Asthma [1.4]	Conduct [0.94]	Back Pain [0.88]	Depression [1.05]	Congenital [1.11]	Falls [1.09]
Milton Keynes	Dermatitis [1.54]	Neonatal [0.9]	Headaches [0.8]	Asthma [1.47]	Anxiety [0.92]	Conduct [0.93]	Back Pain [0.79]	Depression [0.94]	Congenital [1.16]	Falls [0.92]
Oxfordshire	Dermatitis [1.93]	Neonatal [0.95]	Headaches [0.89]	Anxiety [0.96]	Asthma [1.49]	Conduct [0.98]	Back Pain [0.87]	Depression [1.05]	Congenital [1.18]	Falls [0.89]
Portsmouth	Dermatitis [1.5]	Neonatal [0.99]	Headaches [0.87]	Anxiety [0.96]	Asthma [1.45]	Conduct [0.95]	Back Pain [0.86]	Depression [1.04]	Congenital [1.14]	Falls [0.93]
Reading	Dermatitis [1.99]	Neonatal [1.25]	Headaches [0.78]	Asthma [1.5]	Anxiety [0.86]	Conduct [0.9]	Back Pain [0.76]	Congenital [1.22]	Depression [0.91]	URI [1.27]
Slough	Dermatitis [1.54]	Neonatal [1.09]	Asthma [1.5]	Headaches [0.77]	Anxiety [0.9]	Conduct [0.92]	Back Pain [0.76]	Congenital [1.16]	Depression [0.9]	URI [1.27]
Southampton	Dermatitis [1.5]	Neonatal [0.99]	Headaches [0.85]	Asthma [1.44]	Anxiety [0.93]	Conduct [0.92]	Back Pain [0.84]	Depression [1.01]	Congenital [1.16]	Falls [0.92]
Surrey	Dermatitis [1.74]	Neonatal [1.13]	Headaches [0.88]	Anxiety [0.95]	Asthma [1.49]	Conduct [0.99]	Depression [1.03]	Back Pain [0.79]	Congenital [1.19]	Falls [0.88]
West Berkshire	Dermatitis [1.5]	Neonatal [0.94]	Headaches [0.89]	Anxiety [0.98]	Asthma [1.5]	Conduct [1.0]	Back Pain [0.88]	Depression [1.06]	Congenital [1.17]	Falls [0.93]
W Sussex	Dermatitis [1.55]	Neonatal [1.04]	Asthma [1.66]	Headaches [0.86]	Anxiety [0.97]	Conduct [0.97]	Back Pain [0.86]	Depression [1.03]	Congenital [1.15]	Falls [0.98]
Windsor & Maidenhead	Dermatitis [1.5]	Neonatal [0.99]	Headaches [0.89]	Anxiety [0.96]	Asthma [1.52]	Conduct [1.02]	Back Pain [0.87]	Depression [1.04]	Congenital [1.2]	Falls [0.87]
Wokingham	Dermatitis [1.51]	Neonatal [1.01]	Headaches [0.88]	Anxiety [0.96]	Asthma [1.5]	Conduct [1.01]	Back Pain [0.86]	Depression [1.03]	Congenital [1.2]	Falls [0.89]
SW England	Dermatitis [1.49]	Neonatal [1.07]	Headaches [0.89]	Anxiety [1.0]	Asthma [1.44]	Conduct [0.97]	Back Pain [0.89]	Depression [1.06]	Congenital [1.04]	Iron [2.87]
Bath & NE Somerset	Neonatal [1.43]	Dermatitis [1.46]	Headaches [0.96]	Anxiety [1.01]	Asthma [1.5]	Back Pain [0.96]	Conduct [1.01]	Depression [1.15]	Congenital [1.07]	Acne [1.42]
Bournemouth	Dermatitis [1.51]	Neonatal [1.07]	Headaches [0.83]	Asthma [1.43]	Anxiety [0.92]	Conduct [0.9]	Back Pain [0.82]	Depression [0.99]	Iron [3.7]	Congenital [1.07]
Bristol	Neonatal [1.25]	Dermatitis [1.52]	Headaches [0.83]	Asthma [1.49]	Anxiety [0.9]	Conduct [0.92]	Back Pain [0.81]	Depression [0.98]	Congenital [1.1]	URI [1.27]
Cornwall	Dermatitis [1.49]	Neonatal [1.05]	Headaches [0.9]	Anxiety [1.03]	Asthma [1.41]	Conduct [0.96]	Depression [1.09]	Iron [3.3]	Back Pain [0.86]	Congenital [1.03]
Devon	Dermatitis [1.48]	Neonatal [0.96]	Headaches [0.91]	Anxiety [1.02]	Asthma [1.43]	Conduct [0.99]	Back Pain [0.92]	Depression [1.1]	Congenital [1.04]	Falls [1.04]
Dorset	Dermatitis [1.47]	Neonatal [1.03]	Headaches [0.93]	Anxiety [1.06]	Asthma [1.45]	Conduct [1.01]	Back Pain [0.95]	Depression [1.12]	Congenital [1.02]	Iron [2.59]
Gloucestershire	Dermatitis [1.49]	Neonatal [0.99]	Headaches [0.9]	Anxiety [0.99]	Asthma [1.45]	Conduct [0.97]	Back Pain [0.9]	Depression [1.07]	Congenital [1.05]	Falls [0.97]
N Somerset	Neonatal [1.15]	Dermatitis [1.5]	Headaches [0.87]	Anxiety [1.0]	Asthma [1.43]	Conduct [0.96]	Back Pain [0.87]	Depression [1.05]	Congenital [1.04]	Iron [2.75]
Plymouth	Dermatitis [1.5]	Neonatal [1.06]	Headaches [0.87]	Anxiety [0.98]	Asthma [1.41]	Conduct [0.94]	Back Pain [0.87]	Depression [1.04]	Congenital [1.04]	Falls [1.0]
Poole	Dermatitis [1.5]	Neonatal [1.05]	Headaches [0.87]	Anxiety [0.99]	Asthma [1.44]	Conduct [0.96]	Back Pain [0.86]	Depression [1.03]	Congenital [1.04]	Falls [0.99]
Somerset	Dermatitis [1.49]	Neonatal [1.11]	Headaches [0.91]	Anxiety [1.04]	Asthma [1.42]	Conduct [0.97]	Back Pain [0.93]	Depression [1.1]	Congenital [1.02]	Falls [1.1]
S Gloucestershire	Dermatitis [1.5]	Neonatal [1.13]	Headaches [0.88]	Anxiety [0.97]	Asthma [1.48]	Conduct [0.97]	Back Pain [0.87]	Depression [1.04]	Congenital [1.06]	Falls [0.92]
Swindon	Dermatitis [1.51]	Headaches [0.83]	Neonatal [0.78]	Asthma [1.45]	Anxiety [0.95]	Conduct [0.94]	Back Pain [0.84]	Depression [0.99]	Iron [3.46]	Congenital [1.04]
Torbay	Dermatitis [1.5]	Neonatal [0.86]	Headaches [0.87]	Anxiety [1.04]	Asthma [1.4]	Conduct [0.95]	Back Pain [0.9]	Depression [1.06]	Iron [2.06]	Congenital [1.01]
Wiltshire	Neonatal [1.22]	Dermatitis [1.49]	Headaches [0.89]	Anxiety [1.02]	Asthma [1.43]	Conduct [0.97]	Back Pain [0.91]	Depression [1.07]	Iron [3.31]	Congenital [1.03]
W Midlands	Dermatitis [1.5]	Neonatal [1.0]	Headaches [0.87]	Anxiety [1.0]	Asthma [1.45]	Conduct [0.96]	Back Pain [0.88]	Depression [1.05]	Congenital [1.11]	Falls [1.05]
Birmingham	Dermatitis [1.5]	Neonatal [0.98]	Headaches [0.86]	Anxiety [0.98]	Asthma [1.45]	Conduct [0.95]	Back Pain [0.89]	Depression [1.03]	Congenital [1.13]	Falls [1.02]

eFigure 9a. Leading ten causes of YLDs with the ratio of observed YLDs to YLDs expected on the basis of Socio-Demographic Index alone in 2017, <20 years, both sexes combined.
The top ten causes contributing to YLDs are listed globally, by socio-demographic quintile, and then by GBD superregion, region, country, and subnationally where modeled. For each cell, the ratio of observed YLDs to YLDs expected on the basis of socio-demographic index (SDI) alone are listed. Abbreviations: YLD=year of life lived with disability, GBD=Global Burden of Disease.

Values shown in brackets represent the ratio of observed YLDs to predicted YLDs on the basis of Socio-Demographic Index (SDI), rounded to two (2) digits. Color ranges (shown below) were calculated to place a roughly equal number of cells into each bin.

COLOR KEY:	[0.0-0.78]	(0.78-0.88]	(0.88-0.94]	(0.94-1.0]	(1.0-1.06]	(1.06-1.18]	(1.18-1.38]	(1.38-1.66]	1.66+

	1	2	3	4	5	6	7	8	9	10
Coventry	Dermatitis [1.51]	Neonatal [0.96]	Headaches [0.85]	Asthma [1.47]	Anxiety [0.95]	Conduct [0.93]	Back Pain [0.86]	Depression [1.01]	Congenital [1.13]	Falls [0.94]
Dudley	Dermatitis [1.51]	Neonatal [1.09]	Headaches [0.87]	Anxiety [1.03]	Asthma [1.43]	Conduct [0.95]	Back Pain [0.91]	Depression [1.05]	Congenital [1.08]	Falls [1.14]
Herefordshire	Dermatitis [1.49]	Neonatal [1.03]	Headaches [0.9]	Anxiety [1.02]	Asthma [1.45]	Conduct [0.97]	Back Pain [0.92]	Depression [1.07]	Congenital [1.1]	Iron [2.68]
Sandwell	Dermatitis [1.52]	Neonatal [1.08]	Headaches [0.81]	Asthma [1.44]	Anxiety [0.99]	Conduct [0.92]	Back Pain [0.86]	Depression [0.98]	Congenital [1.08]	Iron [1.87]
Shropshire	Dermatitis [1.48]	Neonatal [0.99]	Headaches [0.94]	Anxiety [1.05]	Asthma [1.46]	Conduct [1.0]	Back Pain [0.97]	Depression [1.13]	Congenital [1.1]	Falls [1.05]
Solihull	Dermatitis [1.49]	Headaches [0.89]	Anxiety [0.99]	Asthma [1.48]	Neonatal [0.73]	Conduct [0.99]	Back Pain [0.9]	Depression [1.06]	Congenital [1.13]	Falls [0.99]
Staffordshire	Dermatitis [1.49]	Neonatal [0.97]	Headaches [0.9]	Anxiety [1.03]	Asthma [1.45]	Conduct [0.99]	Depression [1.09]	Back Pain [0.83]	Congenital [1.1]	Iron [2.76]
Stoke-on-Trent	Neonatal [1.18]	Dermatitis [1.52]	Headaches [0.84]	Asthma [1.42]	Anxiety [1.0]	Conduct [0.93]	Depression [1.01]	Back Pain [0.78]	Congenital [1.09]	Falls [1.14]
Telford & Wrekin	Dermatitis [1.51]	Neonatal [1.0]	Headaches [0.87]	Anxiety [1.01]	Asthma [1.45]	Conduct [0.94]	Back Pain [0.9]	Depression [1.04]	Congenital [1.09]	Falls [1.06]
Walsall	Dermatitis [1.52]	Neonatal [1.0]	Headaches [0.86]	Asthma [1.43]	Anxiety [1.03]	Conduct [0.94]	Back Pain [0.91]	Depression [1.04]	Congenital [1.08]	Iron [2.1]
Warwickshire	Dermatitis [1.5]	Neonatal [1.02]	Headaches [0.88]	Anxiety [0.98]	Asthma [1.49]	Conduct [0.98]	Back Pain [0.89]	Depression [1.05]	Congenital [1.13]	Falls [0.97]
Wolverhampton	Dermatitis [1.52]	Headaches [0.85]	Asthma [1.42]	Neonatal [0.73]	Anxiety [0.99]	Conduct [0.93]	Back Pain [0.88]	Depression [1.02]	Congenital [1.09]	Iron [2.16]
Worcestershire	Dermatitis [1.49]	Neonatal [1.12]	Headaches [0.9]	Anxiety [1.02]	Asthma [1.45]	Conduct [0.98]	Back Pain [0.92]	Depression [1.08]	Congenital [1.11]	Falls [1.04]
Yorkshire & Humber	Dermatitis [1.5]	Neonatal [0.94]	Headaches [0.87]	Asthma [1.47]	Anxiety [0.99]	Conduct [0.95]	Back Pain [0.89]	Depression [1.04]	Congenital [1.11]	Iron [2.59]
Barnsley	Dermatitis [1.52]	Neonatal [0.99]	Headaches [0.85]	Asthma [1.45]	Anxiety [1.03]	Conduct [0.92]	Back Pain [0.9]	Depression [1.03]	Iron [2.17]	Congenital [1.07]
Bradford	Dermatitis [1.51]	Neonatal [1.04]	Headaches [0.85]	Asthma [1.45]	Anxiety [1.01]	Conduct [0.94]	Back Pain [0.89]	Depression [1.02]	Congenital [1.1]	Iron [2.21]
Calderdale	Dermatitis [1.51]	Neonatal [0.91]	Headaches [0.87]	Asthma [1.47]	Anxiety [1.0]	Conduct [0.95]	Back Pain [0.9]	Depression [1.04]	Congenital [1.1]	Falls [1.03]
Doncaster	Neonatal [1.21]	Dermatitis [1.52]	Headaches [0.85]	Asthma [1.45]	Anxiety [1.03]	Conduct [0.93]	Back Pain [0.9]	Depression [1.03]	Congenital [1.08]	Falls [1.14]
E Riding of Yorkshire	Dermatitis [1.48]	Neonatal [0.92]	Headaches [0.93]	Anxiety [1.05]	Asthma [1.48]	Conduct [0.99]	Back Pain [0.96]	Depression [1.12]	Congenital [1.09]	Falls [1.08]
Kingston upon Hull	Dermatitis [1.53]	Neonatal [0.87]	Headaches [0.8]	Asthma [1.46]	Anxiety [0.95]	Conduct [0.9]	Back Pain [0.82]	Depression [0.96]	Congenital [1.1]	Falls [1.03]
Kirklees	Dermatitis [1.51]	Neonatal [0.88]	Headaches [0.87]	Anxiety [1.01]	Asthma [1.45]	Conduct [0.94]	Back Pain [0.9]	Depression [1.04]	Congenital [1.1]	Iron [2.46]
Leeds	Dermatitis [1.52]	Neonatal [0.87]	Headaches [0.84]	Asthma [1.5]	Anxiety [0.92]	Conduct [0.93]	Back Pain [0.84]	Depression [0.99]	Congenital [1.16]	Falls [0.88]
NE Lincolnshire	Dermatitis [1.52]	Neonatal [0.9]	Headaches [0.84]	Asthma [1.47]	Anxiety [1.0]	Conduct [0.93]	Back Pain [0.86]	Depression [1.01]	Congenital [1.09]	Iron [2.31]
N Lincolnshire	Dermatitis [1.51]	Neonatal [0.83]	Headaches [0.86]	Asthma [1.47]	Anxiety [1.01]	Conduct [0.95]	Back Pain [0.89]	Depression [1.03]	Congenital [1.08]	Iron [2.6]
N Yorkshire	Dermatitis [1.48]	Neonatal [1.06]	Headaches [0.9]	Anxiety [1.02]	Asthma [1.48]	Conduct [1.0]	Back Pain [0.93]	Depression [1.08]	Congenital [1.12]	Iron [2.81]
Rotherham	Dermatitis [1.51]	Neonatal [0.84]	Headaches [0.87]	Asthma [1.46]	Anxiety [1.03]	Conduct [0.94]	Back Pain [0.91]	Depression [1.05]	Congenital [1.08]	Iron [2.09]
Sheffield	Dermatitis [1.49]	Neonatal [0.99]	Headaches [0.9]	Anxiety [0.98]	Asthma [1.49]	Conduct [0.96]	Back Pain [0.91]	Depression [1.08]	Congenital [1.14]	Falls [0.95]
Wakefield	Dermatitis [1.51]	Headaches [0.85]	Asthma [1.47]	Neonatal [0.75]	Anxiety [1.01]	Conduct [0.93]	Back Pain [0.89]	Depression [1.02]	Congenital [1.08]	Iron [2.43]
York	Dermatitis [1.48]	Headaches [0.93]	Neonatal [0.87]	Anxiety [0.98]	Asthma [1.54]	Back Pain [0.92]	Conduct [0.98]	Depression [1.11]	Congenital [1.16]	Acne [1.3]
N Ireland	Anxiety [1.61]	Dermatitis [1.25]	Headaches [0.95]	Neonatal [0.86]	Asthma [1.29]	Depression [1.17]	Back Pain [0.93]	Conduct [0.81]	Congenital [1.04]	Falls [0.96]
Scotland	Dermatitis [1.35]	Headaches [0.96]	Anxiety [1.12]	Neonatal [0.81]	Asthma [1.45]	Depression [1.15]	Conduct [0.91]	Back Pain [0.91]	Congenital [1.24]	Iron [2.7]

eFigure 9a. Leading ten causes of YLDs with the ratio of observed YLDs to YLDs expected on the basis of Socio-Demographic Index alone in 2017, <20 years, both sexes combined. The top ten causes contributing to YLDs are listed globally, by socio-demographic quintile, and then by GBD superregion, region, country, and subnationally where modeled. For each cell, the ratio of observed YLDs to YLDs expected on the basis of socio-demographic index (SDI) alone are listed. Abbreviations: YLD=year of life lived with disability, GBD=Global Burden of Disease.

Values shown in brackets represent the ratio of observed YLDs to predicted YLDs on the basis of Socio-Demographic Index (SDI), rounded to two (2) digits. Color ranges (shown below) were calculated to place a roughly equal number of cells into each bin.

COLOR KEY:	[0.0-0.78]	(0.78-0.88]	(0.88-0.94]	(0.94-1.0]	(1.0-1.06]	(1.06-1.18]	(1.18-1.38]	(1.38-1.66]	1.66+

	1	2	3	4	5	6	7	8	9	10
Wales	Dermatitis [1.25]	Headaches [0.97]	Neonatal [0.83]	Anxiety [1.11]	Asthma [1.29]	Depression [1.2]	Conduct [0.89]	Back Pain [0.77]	Congenital [1.04]	Falls [1.25]
**Latin America and Caribbean**	Neonatal [1.16]	Headaches [1.19]	Asthma [1.26]	Back Pain [1.29]	Dermatitis [0.89]	Iron [0.86]	Conduct [0.92]	Anxiety [0.92]	Depression [0.95]	Diarrhea [1.33]
**Andean Latin America**	Neonatal [1.13]	Dermatitis [1.45]	Headaches [1.08]	Iron [1.28]	Asthma [1.6]	Conduct [0.86]	Back Pain [1.02]	Anxiety [0.9]	Diarrhea [1.39]	Vit A [0.86]
Bolivia	Iron [1.71]	Neonatal [1.1]	Asthma [1.84]	Dermatitis [1.54]	Headaches [1.1]	Diarrhea [1.47]	Back Pain [1.12]	Conduct [0.84]	Anxiety [0.93]	Vit A [0.77]
Ecuador	Headaches [1.38]	Neonatal [1.12]	Dermatitis [1.27]	Asthma [1.44]	Conduct [0.92]	Anxiety [0.97]	Back Pain [1.03]	Depression [0.99]	Epilepsy [1.4]	Diarrhea [1.27]
Peru	Neonatal [1.14]	Iron [1.53]	Dermatitis [1.53]	Asthma [1.6]	Headaches [0.92]	Conduct [0.84]	Back Pain [0.98]	Anxiety [0.87]	Diarrhea [1.39]	Vit A [1.05]
**Caribbean**	Iron [1.94]	Headaches [1.16]	Neonatal [0.95]	Asthma [1.76]	Dermatitis [1.1]	Anxiety [1.04]	Disaster [1034.02]	Conduct [0.83]	Vit A [1.14]	Depression [0.96]
Antigua	Iron [4.88]	Headaches [1.2]	Asthma [1.87]	Neonatal [0.96]	Dermatitis [1.01]	Anxiety [1.0]	Conduct [0.88]	Back Pain [0.79]	Depression [0.88]	Epilepsy [1.21]
Bahamas	Headaches [1.21]	Asthma [1.97]	Neonatal [0.96]	Dermatitis [1.0]	Anxiety [0.97]	Conduct [0.9]	Back Pain [0.76]	Depression [0.87]	Iron [1.28]	Diarrhea [1.37]
Barbados	Headaches [1.21]	Neonatal [1.03]	Asthma [1.73]	Dermatitis [0.98]	Anxiety [0.99]	Conduct [0.9]	Depression [0.93]	Back Pain [0.79]	Iron [1.07]	Diarrhea [1.23]
Belize	Iron [1.49]	Headaches [1.26]	Asthma [1.91]	Neonatal [1.0]	Dermatitis [1.14]	Anxiety [1.15]	Conduct [0.89]	Depression [1.01]	Back Pain [0.9]	Epilepsy [0.97]
Bermuda	Asthma [2.32]	Headaches [1.15]	Neonatal [0.99]	Dermatitis [1.0]	Anxiety [0.88]	Conduct [0.88]	Depression [0.86]	Back Pain [0.71]	Diarrhea [1.69]	Congenital [0.69]
Cuba	Headaches [1.18]	Asthma [1.95]	Neonatal [0.92]	Dermatitis [1.05]	Iron [1.36]	Anxiety [1.01]	Conduct [0.86]	Depression [0.95]	Back Pain [0.67]	Congenital [0.67]
Dominica	Headaches [1.26]	Neonatal [1.08]	Iron [2.18]	Asthma [1.8]	Dermatitis [1.03]	Anxiety [1.08]	Conduct [0.92]	Back Pain [0.87]	Depression [0.94]	Epilepsy [1.44]
Dominican Rep	Neonatal [1.02]	Headaches [1.22]	Iron [1.02]	Asthma [1.39]	Dermatitis [1.14]	Anxiety [1.12]	Diarrhea [1.44]	Conduct [0.85]	Depression [1.04]	Back Pain [0.89]
Grenada	Neonatal [1.04]	Headaches [1.21]	Asthma [1.76]	Iron [1.12]	Dermatitis [1.09]	Anxiety [1.08]	Conduct [0.88]	Diarrhea [1.4]	Depression [0.96]	Back Pain [0.84]
Guyana	Neonatal [1.07]	Iron [1.18]	Headaches [1.23]	Asthma [1.78]	Dermatitis [1.15]	Depression [1.45]	Anxiety [1.13]	Conduct [0.85]	Back Pain [0.89]	Vit A [0.64]
Haiti	Iron [1.39]	Disaster [1107.76]	Asthma [1.83]	Headaches [1.29]	Vit A [0.81]	Neonatal [1.0]	Dermatitis [1.16]	Diarrhea [1.11]	Anxiety [1.15]	Conduct [0.84]
Jamaica	Headaches [1.25]	Neonatal [1.02]	Asthma [1.85]	Iron [1.64]	Dermatitis [1.04]	Anxiety [1.07]	Conduct [0.89]	Back Pain [0.87]	Depression [0.94]	Epilepsy [1.25]
Puerto Rico	Asthma [2.74]	Headaches [1.25]	Neonatal [1.04]	Dermatitis [0.96]	Anxiety [0.94]	Conduct [0.94]	Back Pain [0.75]	Depression [0.84]	Acne [1.32]	Diarrhea [1.63]
St Lucia	Headaches [1.25]	Neonatal [1.06]	Asthma [1.84]	Dermatitis [1.07]	Anxiety [1.09]	Iron [0.99]	Conduct [0.89]	Depression [1.01]	Back Pain [0.87]	Diarrhea [1.33]
St Vincent	Headaches [1.24]	Neonatal [1.01]	Asthma [1.82]	Iron [1.13]	Dermatitis [1.13]	Anxiety [1.13]	Conduct [0.88]	Depression [1.02]	Back Pain [0.88]	Vit A [0.8]
Suriname	Asthma [1.96]	Neonatal [1.02]	Headaches [1.16]	Iron [1.14]	Dermatitis [1.1]	Anxiety [1.04]	Depression [1.28]	Conduct [0.85]	Back Pain [0.78]	Epilepsy [1.1]
Trinidad Tobago	Asthma [2.07]	Neonatal [1.05]	Headaches [1.11]	Dermatitis [1.07]	Anxiety [0.95]	Conduct [0.83]	Depression [1.01]	Back Pain [0.74]	Iron [0.88]	Congenital [0.69]
Virgin Isl US	Asthma [2.08]	Headaches [1.12]	Neonatal [1.02]	Dermatitis [1.0]	Anxiety [0.86]	Conduct [0.87]	Depression [0.86]	Back Pain [0.69]	Diarrhea [2.05]	Epilepsy [1.36]
**Central Latin America**	Neonatal [1.38]	Headaches [1.0]	Conduct [0.95]	Dermatitis [0.83]	Asthma [0.94]	Epilepsy [1.64]	Diarrhea [1.47]	Back Pain [0.81]	Anxiety [0.68]	Depression [0.74]
Colombia	Neonatal [1.38]	Headaches [1.08]	Dermatitis [0.93]	Conduct [0.95]	Back Pain [1.13]	Asthma [0.97]	Diarrhea [1.54]	Epilepsy [1.68]	Anxiety [0.61]	Congenital [0.7]
Costa Rica	Neonatal [1.28]	Headaches [1.03]	Asthma [1.37]	Conduct [0.94]	Dermatitis [0.87]	Diarrhea [1.76]	Back Pain [0.87]	Epilepsy [1.54]	Anxiety [0.67]	Iron [0.67]
El Salvador	Neonatal [1.37]	Headaches [1.1]	Asthma [1.24]	Iron [0.71]	Conduct [0.97]	Diarrhea [1.47]	Dermatitis [0.85]	Back Pain [0.99]	Depression [1.01]	Epilepsy [1.39]
Guatemala	Neonatal [1.4]	Headaches [1.09]	Diarrhea [1.79]	Iron [0.6]	Dermatitis [1.04]	Conduct [0.95]	Back Pain [1.11]	Asthma [0.84]	Depression [0.97]	Anxiety [0.74]
Honduras	Neonatal [1.53]	Headaches [1.1]	Iron [0.66]	Diarrhea [1.66]	Asthma [1.22]	Dermatitis [1.05]	Conduct [0.94]	Epilepsy [1.52]	Back Pain [1.01]	Vit A [0.5]

eFigure 9a. Leading ten causes of YLDs with the ratio of observed YLDs to YLDs expected on the basis of Socio-Demographic Index alone in 2017, <20 years, both sexes combined.
The top ten causes contributing to YLDs are listed globally, by socio-demographic quintile, and then by GBD superregion, region, country, and subnationally where modeled. For each cell, the ratio of observed YLDs to YLDs expected on the basis of socio-demographic index (SDI) alone are listed. Abbreviations: YLD=year of life lived with disability, GBD=Global Burden of Disease.

Values shown in brackets represent the ratio of observed YLDs to predicted YLDs on the basis of Socio-Demographic Index (SDI), rounded to two (2) digits. Color ranges (shown below) were calculated to place a roughly equal number of cells into each bin.

COLOR KEY:	[0.0-0.78]	(0.78-0.88]	(0.88-0.94]	(0.94-1.0]	(1.0-1.06]	(1.06-1.18]	(1.18-1.38]	(1.38-1.66]	1.66+

	1	2	3	4	5	6	7	8	9	10
Mexico	Neonatal [1.39]	Headaches [0.97]	Conduct [0.97]	Epilepsy [1.79]	Asthma [0.86]	Dermatitis [0.67]	Anxiety [0.71]	Vit A [0.89]	Congenital [0.78]	Back Pain [0.65]
Aguascalientes	Neonatal [1.39]	Headaches [0.97]	Conduct [0.98]	Epilepsy [1.91]	Asthma [0.95]	Dermatitis [0.66]	Anxiety [0.69]	Congenital [0.83]	Back Pain [0.67]	Depression [0.71]
Baja CA	Neonatal [1.36]	Headaches [0.99]	Conduct [1.0]	Epilepsy [1.85]	Dermatitis [0.66]	Asthma [0.79]	Anxiety [0.71]	Vit A [1.05]	Back Pain [0.68]	Congenital [0.78]
Baja CA Sur	Neonatal [1.44]	Headaches [0.97]	Conduct [0.99]	Epilepsy [1.88]	Asthma [0.92]	Dermatitis [0.66]	Anxiety [0.69]	Vit A [1.11]	Congenital [0.8]	Back Pain [0.65]
Campeche	Neonatal [1.52]	Headaches [0.96]	Conduct [0.96]	Epilepsy [1.66]	Asthma [0.87]	Dermatitis [0.7]	Vit A [0.95]	Iron [0.55]	Anxiety [0.71]	Depression [0.82]
Chiapas	Neonatal [1.43]	Headaches [1.0]	Conduct [0.97]	Epilepsy [1.59]	Vit A [0.75]	Asthma [0.86]	Dermatitis [0.72]	Diarrhea [1.05]	Anxiety [0.75]	Back Pain [0.71]
Chihuahua	Neonatal [1.28]	Headaches [1.0]	Conduct [1.0]	Epilepsy [1.83]	Asthma [0.97]	Anxiety [0.72]	Dermatitis [0.57]	Congenital [0.85]	Back Pain [0.7]	Depression [0.77]
Coahuila	Neonatal [1.18]	Headaches [0.97]	Conduct [0.97]	Epilepsy [1.83]	Asthma [0.96]	Dermatitis [0.67]	Diarrhea [1.29]	Anxiety [0.69]	Back Pain [0.67]	Congenital [0.76]
Colima	Neonatal [1.47]	Headaches [0.97]	Conduct [0.98]	Epilepsy [1.89]	Asthma [0.89]	Dermatitis [0.67]	Anxiety [0.69]	Vit A [0.97]	Back Pain [0.66]	Depression [0.71]
Mexico City	Neonatal [1.48]	Headaches [0.95]	Conduct [0.94]	Epilepsy [2.26]	Dermatitis [0.82]	Asthma [0.78]	Anxiety [0.61]	Congenital [0.83]	Back Pain [0.64]	Depression [0.72]
Durango	Neonatal [1.24]	Headaches [0.97]	Conduct [0.97]	Dermatitis [0.9]	Epilepsy [1.76]	Asthma [0.95]	Anxiety [0.71]	Vit A [0.86]	Back Pain [0.68]	Congenital [0.78]
Guanajuato	Neonatal [1.26]	Headaches [0.98]	Conduct [0.98]	Epilepsy [1.79]	Asthma [0.93]	Vit A [1.03]	Dermatitis [0.69]	Anxiety [0.72]	Back Pain [0.69]	Depression [0.76]
Guerrero	Neonatal [1.48]	Headaches [1.01]	Conduct [0.98]	Epilepsy [1.59]	Asthma [0.87]	Dermatitis [0.72]	Vit A [0.67]	Anxiety [0.75]	Back Pain [0.71]	Congenital [0.74]
Hidalgo	Neonatal [1.71]	Headaches [1.0]	Conduct [0.99]	Epilepsy [1.67]	Asthma [0.91]	Dermatitis [0.71]	Anxiety [0.75]	Back Pain [0.71]	Vit A [0.62]	Congenital [0.75]
Jalisco	Neonatal [1.34]	Headaches [0.96]	Epilepsy [2.17]	Conduct [0.97]	Dermatitis [0.67]	Asthma [0.78]	Anxiety [0.69]	Congenital [0.83]	Back Pain [0.66]	Depression [0.74]
Mexico	Neonatal [1.64]	Headaches [0.93]	Conduct [0.98]	Epilepsy [1.78]	Asthma [0.78]	Anxiety [0.71]	Vit A [0.96]	Dermatitis [0.59]	Depression [0.7]	Congenital [0.71]
Michoacan	Neonatal [1.2]	Headaches [0.98]	Conduct [0.97]	Epilepsy [1.6]	Asthma [0.91]	Dermatitis [0.72]	Anxiety [0.74]	Vit A [0.71]	Back Pain [0.7]	Congenital [0.75]
Morelos	Neonatal [1.36]	Headaches [0.97]	Conduct [0.97]	Epilepsy [1.83]	Asthma [0.82]	Anxiety [0.7]	Dermatitis [0.58]	Congenital [0.84]	Vit A [0.81]	Depression [0.69]
Nayarit	Neonatal [1.49]	Headaches [0.96]	Conduct [0.97]	Epilepsy [1.7]	Asthma [0.89]	Dermatitis [0.69]	Anxiety [0.71]	Congenital [0.8]	Vit A [0.76]	Back Pain [0.67]
Nuevo Leon	Neonatal [1.13]	Headaches [0.95]	Conduct [0.96]	Epilepsy [1.96]	Asthma [0.78]	Anxiety [0.66]	Vit A [1.18]	Dermatitis [0.57]	Back Pain [0.65]	Congenital [0.79]
Oaxaca	Neonatal [1.4]	Headaches [1.02]	Conduct [0.99]	Epilepsy [1.68]	Asthma [0.88]	Dermatitis [0.72]	Anxiety [0.76]	Congenital [0.81]	Back Pain [0.72]	Diarrhea [0.86]
Puebla	Neonatal [1.31]	Headaches [0.99]	Conduct [0.97]	Asthma [0.9]	Dermatitis [0.71]	Anxiety [0.74]	Epilepsy [1.24]	Back Pain [0.71]	Vit A [0.62]	Congenital [0.77]
Queretaro	Neonatal [1.46]	Headaches [0.96]	Conduct [0.97]	Epilepsy [1.81]	Asthma [0.93]	Dermatitis [0.68]	Anxiety [0.69]	Back Pain [0.66]	Congenital [0.77]	Depression [0.7]
Quintana Roo	Neonatal [1.76]	Headaches [0.96]	Conduct [0.97]	Epilepsy [1.79]	Vit A [1.07]	Asthma [0.86]	Dermatitis [0.69]	Diarrhea [1.27]	Anxiety [0.7]	Depression [0.81]
San Luis Potosi	Neonatal [1.2]	Headaches [0.99]	Conduct [0.99]	Epilepsy [1.77]	Asthma [0.92]	Dermatitis [0.69]	Vit A [0.93]	Anxiety [0.72]	Congenital [0.84]	Back Pain [0.69]
Sinaloa	Neonatal [1.26]	Headaches [0.97]	Conduct [0.98]	Epilepsy [1.8]	Asthma [0.89]	Vit A [1.17]	Dermatitis [0.67]	Anxiety [0.7]	Back Pain [0.66]	Congenital [0.75]
Sonora	Neonatal [1.23]	Headaches [0.98]	Conduct [0.99]	Epilepsy [1.89]	Asthma [0.93]	Dermatitis [0.67]	Vit A [1.09]	Anxiety [0.7]	Congenital [0.84]	Back Pain [0.68]
Tabasco	Neonatal [1.5]	Headaches [0.98]	Conduct [0.98]	Epilepsy [1.71]	Iron [0.67]	Vit A [0.97]	Dermatitis [0.69]	Asthma [0.8]	Anxiety [0.73]	Congenital [0.86]
Tamaulipas	Neonatal [1.53]	Headaches [0.97]	Conduct [0.98]	Epilepsy [1.79]	Asthma [0.89]	Anxiety [0.7]	Vit A [1.02]	Diarrhea [1.16]	Dermatitis [0.56]	Back Pain [0.67]
Tlaxcala	Neonatal [1.48]	Headaches [1.0]	Conduct [0.99]	Epilepsy [1.77]	Asthma [0.93]	Dermatitis [0.7]	Anxiety [0.74]	Congenital [0.84]	Back Pain [0.7]	Depression [0.7]
Veracruz	Neonatal [1.48]	Headaches [1.01]	Conduct [1.0]	Epilepsy [1.73]	Asthma [0.86]	Dermatitis [0.71]	Anxiety [0.75]	Vit A [0.7]	Congenital [0.81]	Back Pain [0.71]

eFigure 9a. Leading ten causes of YLDs with the ratio of observed YLDs to YLDs expected on the basis of Socio-Demographic Index alone in 2017, <20 years, both sexes combined. The top ten causes contributing to YLDs are listed globally, by socio-demographic quintile, and then by GBD superregion, region, country, and subnationally where modeled. For each cell, the ratio of observed YLDs to YLDs expected on the basis of socio-demographic index (SDI) alone are listed. Abbreviations: YLD=year of life lived with disability, GBD=Global Burden of Disease.

Values shown in brackets represent the ratio of observed YLDs to predicted YLDs on the basis of Socio-Demographic Index (SDI), rounded to two (2) digits. Color ranges (shown below) were calculated to place a roughly equal number of cells into each bin.

COLOR KEY:	[0.0-0.78]	(0.78-0.88]	(0.88-0.94]	(0.94-1.0]	(1.0-1.06]	(1.06-1.18]	(1.18-1.38]	(1.38-1.66]	1.66+

	1	2	3	4	5	6	7	8	9	10
Yucatan	Neonatal [1.28]	Headaches [0.99]	Conduct [0.99]	Epilepsy [1.8]	Asthma [0.83]	Dermatitis [0.65]	Anxiety [0.72]	Depression [0.82]	Congenital [0.81]	Vit A [0.74]
Zacatecas	Neonatal [0.97]	Headaches [0.97]	Conduct [0.97]	Epilepsy [1.78]	Asthma [0.94]	Dermatitis [0.7]	Anxiety [0.72]	Vit A [0.74]	Back Pain [0.69]	Congenital [0.73]
Nicaragua	Neonatal [1.43]	Headaches [1.08]	Diarrhea [1.7]	Dermatitis [1.06]	Conduct [0.95]	Asthma [0.98]	Back Pain [0.98]	Iron [0.38]	Epilepsy [1.24]	Depression [0.9]
Panama	Neonatal [1.27]	Headaches [0.98]	Dermatitis [1.16]	Diarrhea [2.57]	Asthma [1.29]	Iron [1.18]	Conduct [0.92]	Back Pain [0.81]	Anxiety [0.63]	Epilepsy [1.35]
Venezuela	Neonatal [1.39]	Diarrhea [3.03]	Headaches [0.97]	Dermatitis [0.99]	Conduct [0.91]	Asthma [1.03]	Epilepsy [1.58]	Back Pain [0.74]	Anxiety [0.64]	Depression [0.77]
**Tropical Latin America**	Headaches [1.49]	Back Pain [2.15]	Neonatal [0.91]	Asthma [1.49]	Anxiety [1.22]	Iron [1.21]	Depression [1.32]	Conduct [0.93]	Vit A [1.37]	Dermatitis [0.74]
Brazil	Headaches [1.49]	Back Pain [2.17]	Neonatal [0.92]	Asthma [1.48]	Anxiety [1.21]	Iron [1.25]	Depression [1.33]	Conduct [0.93]	Vit A [1.41]	Dermatitis [0.73]
Acre	Headaches [1.59]	Iron [1.44]	Back Pain [2.41]	Neonatal [0.96]	Anxiety [1.35]	Asthma [1.31]	Depression [1.39]	Conduct [0.98]	Vit A [1.08]	Dermatitis [0.77]
Alagoas	Headaches [1.56]	Back Pain [2.33]	Iron [0.82]	Neonatal [0.82]	Anxiety [1.35]	Vit A [1.07]	Asthma [1.24]	Depression [1.54]	Conduct [0.95]	Dermatitis [0.66]
Amapa	Headaches [1.56]	Back Pain [2.19]	Neonatal [0.95]	Iron [1.58]	Asthma [1.42]	Anxiety [1.27]	Depression [1.39]	Conduct [0.98]	Dermatitis [0.73]	Vit A [1.13]
Amazonas	Headaches [1.51]	Back Pain [2.22]	Iron [1.37]	Neonatal [0.81]	Asthma [1.42]	Anxiety [1.27]	Conduct [0.95]	Depression [1.22]	Vit A [1.17]	Dermatitis [0.76]
Bahia	Headaches [1.49]	Back Pain [2.23]	Iron [1.09]	Neonatal [0.9]	Vit A [1.39]	Asthma [1.29]	Anxiety [1.29]	Dermatitis [1.05]	Depression [1.17]	Conduct [0.84]
Ceara	Headaches [1.47]	Back Pain [2.18]	Neonatal [0.86]	Iron [1.01]	Asthma [1.36]	Vit A [1.45]	Anxiety [1.26]	Depression [1.36]	Conduct [0.91]	Dermatitis [0.79]
Distrito Federal	Headaches [1.46]	Back Pain [1.96]	Neonatal [1.03]	Asthma [1.64]	Anxiety [1.06]	Depression [1.24]	Conduct [0.96]	Dermatitis [0.68]	Congenital [0.96]	Iron [1.8]
Espirito Santo	Headaches [1.49]	Back Pain [2.1]	Iron [1.85]	Asthma [1.73]	Neonatal [0.89]	Anxiety [1.21]	Depression [1.37]	Conduct [0.94]	Vit A [1.75]	Dermatitis [0.73]
Goias	Headaches [1.56]	Back Pain [2.3]	Neonatal [0.82]	Asthma [1.51]	Anxiety [1.28]	Depression [1.44]	Iron [1.04]	Conduct [0.97]	Dermatitis [0.73]	Vit A [1.13]
Maranhao	Headaches [1.54]	Iron [0.91]	Back Pain [2.31]	Vit A [1.13]	Neonatal [0.91]	Asthma [1.26]	Anxiety [1.32]	Conduct [0.92]	Depression [1.28]	Dermatitis [0.8]
Mato Grosso	Headaches [1.5]	Back Pain [2.19]	Neonatal [0.86]	Asthma [1.6]	Anxiety [1.23]	Depression [1.28]	Iron [1.0]	Conduct [0.95]	Vit A [1.5]	Dermatitis [0.74]
Mato Grosso do Sul	Headaches [1.52]	Back Pain [2.24]	Neonatal [0.99]	Asthma [1.46]	Anxiety [1.25]	Depression [1.43]	Conduct [0.95]	Iron [0.84]	Dermatitis [0.74]	Vit A [1.18]
Minas Gerais	Headaches [1.53]	Back Pain [2.27]	Neonatal [0.96]	Asthma [1.54]	Anxiety [1.26]	Depression [1.35]	Iron [1.05]	Conduct [0.97]	Vit A [1.21]	Dermatitis [0.63]
Para	Headaches [1.55]	Back Pain [2.3]	Neonatal [0.86]	Iron [0.94]	Asthma [1.4]	Anxiety [1.34]	Vit A [1.12]	Conduct [0.96]	Diarrhea [1.42]	Dermatitis [0.8]
Paraiba	Headaches [1.47]	Back Pain [2.21]	Neonatal [0.81]	Iron [0.84]	Vit A [1.31]	Asthma [1.32]	Anxiety [1.28]	Depression [1.3]	Conduct [0.91]	Dermatitis [0.81]
Parana	Headaches [1.54]	Back Pain [2.24]	Neonatal [0.93]	Asthma [1.63]	Anxiety [1.23]	Depression [1.38]	Conduct [0.97]	Iron [1.07]	Dermatitis [0.64]	Congenital [0.95]
Pernambuco	Headaches [1.49]	Back Pain [2.21]	Iron [1.16]	Neonatal [0.85]	Asthma [1.42]	Anxiety [1.28]	Depression [1.49]	Vit A [1.19]	Conduct [0.92]	Dermatitis [0.77]
Piaui	Headaches [1.52]	Back Pain [2.32]	Neonatal [0.95]	Asthma [1.36]	Anxiety [1.32]	Conduct [0.93]	Depression [1.23]	Dermatitis [0.81]	Vit A [0.7]	Congenital [0.94]
Rio de Janeiro	Headaches [1.51]	Neonatal [1.14]	Back Pain [1.89]	Asthma [1.48]	Anxiety [1.18]	Depression [1.25]	Conduct [0.96]	Dermatitis [0.7]	Iron [1.0]	Congenital [0.88]
Rio Grande do Norte	Headaches [1.46]	Back Pain [2.17]	Iron [1.06]	Neonatal [0.78]	Asthma [1.37]	Anxiety [1.25]	Depression [1.34]	Vit A [1.18]	Conduct [0.91]	Dermatitis [0.79]
Rio Grande do Sul	Back Pain [2.95]	Headaches [1.56]	Neonatal [0.95]	Asthma [1.65]	Anxiety [1.22]	Depression [1.46]	Congenital [1.5]	Dermatitis [0.92]	Epilepsy [2.2]	Iron [1.22]
Rondonia	Headaches [1.58]	Back Pain [2.37]	Neonatal [0.99]	Anxiety [1.33]	Asthma [1.38]	Iron [0.91]	Congenital [1.53]	Depression [1.42]	Conduct [0.98]	Vit A [1.09]
Roraima	Headaches [1.44]	Back Pain [2.11]	Neonatal [1.01]	Iron [1.44]	Asthma [1.42]	Anxiety [1.2]	Depression [1.47]	Conduct [0.92]	Vit A [1.27]	Dermatitis [0.75]
Santa Catarina	Headaches [1.82]	Back Pain [2.14]	Asthma [1.78]	Neonatal [0.85]	Anxiety [1.19]	Depression [1.51]	Iron [1.5]	Conduct [0.97]	Dermatitis [0.7]	Vit A [1.55]

eFigure 9a. Leading ten causes of YLDs with the ratio of observed YLDs to YLDs expected on the basis of Socio-Demographic Index alone in 2017, <20 years, both sexes combined.
The top ten causes contributing to YLDs are listed globally, by socio-demographic quintile, and then by GBD superregion, region, country, and subnationally where modeled. For each cell, the ratio of observed YLDs to YLDs expected on the basis of socio-demographic index (SDI) alone are listed. Abbreviations: YLD=year of life lived with disability, GBD=Global Burden of Disease.

Values shown in brackets represent the ratio of observed YLDs to predicted YLDs on the basis of Socio-Demographic Index (SDI), rounded to two (2) digits. Color ranges (shown below) were calculated to place a roughly equal number of cells into each bin.

COLOR KEY:	[0.0-0.78]	(0.78-0.88]	(0.88-0.94]	(0.94-1.0]	(1.0-1.06]	(1.06-1.18]	(1.18-1.38]	(1.38-1.66]	1.66+

	1	2	3	4	5	6	7	8	9	10
Sao Paulo	Headaches [1.44]	Back Pain [2.12]	Neonatal [0.97]	Asthma [1.6]	Anxiety [1.15]	Depression [1.43]	Conduct [0.97]	Iron [1.32]	Dermatitis [0.61]	Bipolar [1.8]
Sergipe	Headaches [1.51]	Back Pain [2.21]	Neonatal [0.99]	Iron [1.24]	Asthma [1.46]	Anxiety [1.28]	Vit A [1.46]	Depression [1.33]	Conduct [0.94]	Dermatitis [0.65]
Tocantins	Headaches [1.51]	Back Pain [2.27]	Neonatal [0.97]	Iron [1.12]	Asthma [1.39]	Anxiety [1.29]	Depression [1.4]	Conduct [0.94]	Vit A [1.2]	Dermatitis [0.78]
Paraguay	Headaches [1.43]	Asthma [1.74]	Neonatal [0.79]	Back Pain [1.41]	Anxiety [1.24]	Iron [0.92]	Diarrhea [1.98]	Dermatitis [1.04]	Conduct [0.85]	Depression [1.04]
**North Africa and Middle East**	Iron [1.71]	Neonatal [1.0]	Headaches [0.96]	Anxiety [1.07]	Back Pain [1.15]	Diarrhea [1.83]	Conduct [0.93]	Asthma [0.96]	Depression [1.11]	Dermatitis [0.74]
Afghanistan	Diarrhea [1.38]	Vit A [0.45]	Neonatal [1.13]	Iron [0.34]	Headaches [1.25]	Dermatitis [1.0]	Anxiety [1.34]	Back Pain [1.61]	Conduct [1.03]	Asthma [1.14]
Algeria	Neonatal [1.13]	Headaches [0.84]	Anxiety [0.95]	Asthma [1.09]	Conduct [0.86]	Back Pain [0.96]	Dermatitis [0.75]	Depression [0.97]	Diarrhea [1.59]	Iron [0.89]
Bahrain	Neonatal [1.06]	Headaches [0.89]	Anxiety [0.98]	Dermatitis [0.89]	Conduct [0.91]	Back Pain [1.01]	Depression [1.09]	Asthma [0.99]	Diarrhea [1.79]	Congenital [0.8]
Egypt	Neonatal [1.06]	Iron [1.33]	Headaches [0.99]	Asthma [1.18]	Back Pain [1.27]	Conduct [0.91]	Diarrhea [1.56]	Anxiety [0.99]	Depression [0.94]	Dermatitis [0.63]
Iran	Neonatal [1.28]	Headaches [1.25]	Anxiety [1.33]	Depression [1.28]	Conduct [0.98]	Back Pain [0.98]	Diarrhea [1.78]	Asthma [0.82]	Congenital [0.99]	Dermatitis [0.59]
Iraq	Neonatal [1.13]	Iron [1.18]	Headaches [0.94]	Anxiety [1.08]	Conduct [0.91]	Asthma [1.02]	Back Pain [1.14]	Vit A [0.96]	Conflict Terror [672.19]	Dermatitis [0.71]
Jordan	Neonatal [1.4]	Headaches [0.94]	Iron [1.48]	Asthma [1.24]	Anxiety [1.05]	Conduct [0.95]	Back Pain [1.08]	Depression [1.07]	Dermatitis [0.75]	Diarrhea [1.46]
Kuwait	Neonatal [1.2]	Headaches [0.72]	Asthma [1.21]	Anxiety [0.88]	Conduct [0.92]	Back Pain [0.92]	Dermatitis [0.74]	Diarrhea [2.3]	Depression [0.94]	Iron [1.75]
Lebanon	Neonatal [1.06]	Headaches [0.91]	Anxiety [1.18]	Asthma [1.35]	Dermatitis [0.88]	Conduct [0.89]	Diarrhea [2.01]	Depression [0.99]	Back Pain [0.78]	Congenital [0.91]
Libya	Neonatal [1.05]	Headaches [0.91]	Diarrhea [2.94]	Anxiety [0.95]	Dermatitis [0.87]	Back Pain [1.02]	Conduct [0.94]	Iron [1.83]	Depression [1.05]	Asthma [1.0]
Morocco	Neonatal [1.05]	Headaches [1.04]	Iron [0.91]	Depression [1.79]	Back Pain [1.47]	Anxiety [1.25]	Conduct [0.99]	Dermatitis [0.89]	Vit A [0.98]	Diarrhea [1.3]
Palestine	Neonatal [1.11]	Headaches [0.99]	Depression [1.63]	Anxiety [1.2]	Iron [0.54]	Asthma [1.07]	Conduct [0.95]	Back Pain [1.21]	Dermatitis [0.86]	Diarrhea [1.11]
Oman	Neonatal [1.13]	Headaches [0.78]	Iron [2.12]	Diarrhea [2.43]	Anxiety [0.85]	Conduct [0.83]	Back Pain [0.85]	Asthma [0.92]	Dermatitis [0.67]	Depression [0.88]
Qatar	Neonatal [1.02]	Headaches [0.77]	Dermatitis [0.88]	Anxiety [0.83]	Conduct [0.86]	Back Pain [0.89]	Asthma [0.91]	Depression [0.87]	Congenital [0.89]	Iron [1.28]
Saudi Arabia	Headaches [1.01]	Neonatal [0.75]	Anxiety [0.98]	Back Pain [1.09]	Conduct [0.99]	Dermatitis [0.85]	Depression [1.1]	Diarrhea [2.23]	Congenital [0.95]	Asthma [0.73]
Sudan	Iron [1.22]	Diarrhea [2.02]	Neonatal [0.87]	Headaches [1.01]	Vit A [0.74]	Anxiety [1.21]	Back Pain [1.33]	Conduct [0.95]	Asthma [1.01]	Dermatitis [0.78]
Syria	Conflict Terror [2962.5]	Iron [1.25]	Headaches [1.11]	Neonatal [0.89]	Anxiety [1.31]	Back Pain [1.36]	Conduct [1.09]	Depression [1.28]	Asthma [0.88]	Dermatitis [0.58]
Tunisia	Neonatal [1.05]	Headaches [0.93]	Anxiety [1.06]	Conduct [0.94]	Dermatitis [0.87]	Back Pain [0.98]	Depression [1.1]	Asthma [0.84]	Diarrhea [1.44]	Congenital [0.8]
Turkey	Neonatal [0.77]	Headaches [0.8]	Back Pain [1.24]	Conduct [0.96]	Depression [1.12]	Anxiety [0.82]	Dermatitis [0.67]	Asthma [0.88]	Iron [1.07]	Diarrhea [1.46]
UAE	Neonatal [1.07]	Headaches [0.81]	Asthma [1.38]	Dermatitis [0.87]	Anxiety [0.82]	Conduct [0.86]	Diarrhea [2.51]	Back Pain [0.84]	Iron [2.03]	Congenital [0.92]
Yemen	Iron [3.34]	Vit A [1.51]	Diarrhea [2.1]	Neonatal [1.19]	Headaches [1.02]	Dermatitis [0.97]	Anxiety [1.2]	Hemog [2.7]	Conduct [0.94]	Back Pain [1.25]
**South Asia**	Iron [1.92]	Neonatal [1.46]	Vit A [1.53]	Headaches [1.03]	Conduct [0.89]	Anxiety [0.88]	Congenital [1.01]	ID [5.06]	Dermatitis [0.64]	Diarrhea [0.91]
Bangladesh	Iron [0.94]	Neonatal [1.43]	Headaches [1.2]	Anxiety [1.15]	Dermatitis [0.89]	Conduct [0.88]	Depression [1.11]	Vit A [0.43]	Back Pain [1.04]	Congenital [0.96]
Bhutan	Iron [3.03]	Neonatal [1.33]	Headaches [1.02]	Vit A [1.35]	Dermatitis [0.9]	Diarrhea [1.31]	Conduct [0.82]	Anxiety [0.92]	Back Pain [0.83]	Congenital [0.82]
India	Iron [2.16]	Neonatal [1.48]	Vit A [1.88]	Headaches [1.02]	Conduct [0.91]	ID [6.52]	Congenital [1.08]	Anxiety [0.84]	Diarrhea [0.88]	PEM [18.45]

eFigure 9a. Leading ten causes of YLDs with the ratio of observed YLDs to YLDs expected on the basis of Socio-Demographic Index alone in 2017, <20 years, both sexes combined. The top ten causes contributing to YLDs are listed globally, by socio-demographic quintile, and then by GBD superregion, region, country, and subnationally where modeled. For each cell, the ratio of observed YLDs to YLDs expected on the basis of socio-demographic index (SDI) alone are listed. Abbreviations: YLD=year of life lived with disability, GBD=Global Burden of Disease.

Values shown in brackets represent the ratio of observed YLDs to predicted YLDs on the basis of Socio-Demographic Index (SDI), rounded to two (2) digits. Color ranges (shown below) were calculated to place a roughly equal number of cells into each bin.

COLOR KEY:	[0.0-0.78]	(0.78-0.88]	(0.88-0.94]	(0.94-1.0]	(1.0-1.06]	(1.06-1.18]	(1.18-1.38]	(1.38-1.66]	1.66+

	1	2	3	4	5	6	7	8	9	10
Andhra Pradesh	Iron [1.76]	Neonatal [1.61]	Vit A [1.55]	Headaches [1.08]	Conduct [0.93]	Anxiety [0.88]	Congenital [1.03]	ID [5.47]	Depression [1.02]	PEM [17.82]
Arunachal Pradesh	Iron [1.95]	Neonatal [1.55]	Vit A [1.62]	Headaches [1.02]	Conduct [0.9]	Congenital [1.05]	Anxiety [0.84]	ID [5.35]	PEM [23.35]	Depression [0.91]
Assam	Iron [1.88]	Neonatal [1.42]	Vit A [1.48]	Headaches [1.03]	ID [6.92]	Conduct [0.89]	Congenital [1.09]	Anxiety [0.84]	Dermatitis [0.56]	Depression [0.77]
Bihar	Iron [1.46]	Vit A [1.28]	Neonatal [1.6]	Headaches [1.12]	ID [7.13]	Conduct [0.96]	Congenital [1.04]	Anxiety [0.89]	Diarrhea [0.81]	Hemog [1.81]
Chhattisgarh	Iron [1.75]	Neonatal [1.53]	Vit A [1.4]	Headaches [1.03]	Conduct [0.88]	ID [6.0]	Congenital [1.05]	Anxiety [0.78]	PEM [12.15]	Dermatitis [0.56]
Delhi	Iron [7.92]	Neonatal [1.47]	Vit A [3.91]	Headaches [0.93]	Conduct [0.89]	Anxiety [0.69]	Congenital [0.96]	Diarrhea [1.39]	Hemog [6.16]	Blindness [1.67]
Goa	Neonatal [1.8]	Headaches [0.99]	Vit A [3.24]	Iron [1.96]	Conduct [0.93]	Anxiety [0.66]	Congenital [0.99]	Diarrhea [1.48]	Depression [0.71]	Dermatitis [0.5]
Gujarat	Iron [3.76]	Neonatal [1.52]	Vit A [2.22]	Headaches [0.98]	Conduct [0.89]	PEM [44.45]	Congenital [1.05]	Anxiety [0.78]	ID [5.3]	Hemog [3.26]
Haryana	Iron [4.49]	Neonatal [1.51]	Vit A [2.56]	Headaches [0.95]	Conduct [0.88]	Congenital [1.03]	Anxiety [0.79]	Hemog [3.71]	Depression [0.84]	Dermatitis [0.55]
Himachal Pradesh	Iron [4.58]	Neonatal [1.64]	Vit A [2.54]	Headaches [0.99]	Conduct [0.9]	Anxiety [0.79]	Congenital [1.0]	ID [5.34]	Dermatitis [0.53]	Depression [0.74]
Jammu & Kashmir	Iron [1.82]	Neonatal [1.46]	Vit A [2.16]	Headaches [1.05]	Conduct [0.96]	ID [6.46]	Anxiety [0.86]	Congenital [1.06]	Diarrhea [0.95]	Dermatitis [0.56]
Jharkhand	Iron [1.8]	Neonatal [1.59]	Vit A [1.5]	Headaches [1.08]	Conduct [1.02]	ID [6.0]	PEM [11.98]	Congenital [1.05]	Anxiety [0.91]	Diarrhea [0.79]
Karnataka	Neonatal [1.74]	Iron [1.68]	Vit A [1.9]	Headaches [1.0]	Conduct [0.77]	Anxiety [0.85]	Congenital [1.02]	PEM [31.28]	ID [5.52]	Dermatitis [0.56]
Kerala	Neonatal [2.01]	Headaches [0.97]	Iron [1.36]	Vit A [2.13]	Conduct [0.86]	Anxiety [0.83]	Congenital [1.01]	Dermatitis [0.52]	PEM [121.78]	Depression [0.72]
Madhya Pradesh	Iron [2.19]	Vit A [1.62]	Neonatal [1.44]	Headaches [1.03]	ID [6.58]	Conduct [0.89]	PEM [12.0]	Congenital [1.05]	Hemog [2.27]	Anxiety [0.77]
Maharashtra	Iron [3.09]	Neonatal [1.61]	Vit A [2.29]	Headaches [1.0]	Conduct [0.95]	PEM [75.94]	Anxiety [0.8]	Congenital [1.02]	ID [5.4]	Depression [0.8]
Manipur	Neonatal [1.66]	Headaches [1.04]	Vit A [1.39]	Conduct [0.91]	ID [7.1]	Anxiety [0.93]	Iron [0.55]	Congenital [1.07]	Depression [0.89]	Diarrhea [0.98]
Meghalaya	Iron [2.14]	Neonatal [1.6]	Vit A [1.79]	Headaches [1.03]	Conduct [0.92]	ID [6.44]	Congenital [1.07]	Anxiety [0.85]	Depression [0.92]	Dermatitis [0.57]
Mizoram	Neonatal [1.64]	Headaches [1.0]	Vit A [1.64]	Conduct [0.9]	Iron [0.69]	Anxiety [0.81]	Congenital [1.04]	ID [6.4]	Diarrhea [1.25]	Dermatitis [0.55]
Nagaland	Neonatal [1.7]	Iron [2.53]	Headaches [1.01]	Vit A [1.98]	Conduct [0.92]	Anxiety [0.8]	Congenital [1.02]	ID [5.66]	Dermatitis [0.53]	Depression [0.76]
Odisha	Neonatal [1.44]	Iron [1.0]	Vit A [1.59]	Headaches [1.04]	ID [6.56]	Conduct [0.9]	Congenital [1.07]	Anxiety [0.85]	PEM [16.17]	Diarrhea [0.97]
Punjab	Iron [3.65]	Neonatal [1.54]	Vit A [2.36]	Headaches [0.98]	Conduct [0.91]	Congenital [1.03]	Anxiety [0.76]	ID [5.28]	Dermatitis [0.52]	Hemog [3.34]
Rajasthan	Iron [1.61]	Neonatal [1.41]	Vit A [1.51]	Headaches [1.04]	ID [6.2]	Conduct [0.91]	Congenital [1.05]	PEM [11.67]	Anxiety [0.88]	Diarrhea [0.77]
Sikkim	Iron [3.05]	Neonatal [1.65]	Headaches [1.05]	Vit A [2.11]	Conduct [0.92]	Anxiety [0.82]	Congenital [1.0]	Diarrhea [1.16]	Depression [0.79]	Dermatitis [0.54]
Tamil Nadu	Iron [2.31]	Neonatal [1.56]	Vit A [2.45]	Headaches [0.99]	Conduct [0.88]	PEM [68.31]	Anxiety [0.78]	Depression [0.97]	ID [5.53]	Congenital [0.85]
Telangana	Iron [2.72]	Neonatal [1.6]	Vit A [1.94]	Headaches [1.05]	Conduct [0.91]	PEM [36.5]	Anxiety [0.85]	Congenital [1.04]	Depression [0.98]	ID [5.48]
Tripura	Iron [1.54]	Neonatal [1.55]	Vit A [1.71]	Headaches [1.07]	Conduct [0.93]	ID [6.45]	Anxiety [0.87]	Congenital [1.05]	PEM [17.78]	Diarrhea [0.87]
Uttar Pradesh	Iron [1.88]	Vit A [1.58]	Neonatal [1.37]	Headaches [1.08]	ID [6.71]	Conduct [0.93]	Congenital [1.14]	Anxiety [0.85]	Diarrhea [0.82]	Hemog [2.21]
Uttarakhand	Iron [3.32]	Neonatal [1.62]	Vit A [2.06]	Headaches [1.04]	Conduct [0.93]	Anxiety [0.84]	Congenital [1.03]	Diarrhea [1.08]	ID [5.14]	Dermatitis [0.55]
W Bengal	Iron [1.42]	Neonatal [1.43]	Vit A [1.69]	Headaches [1.09]	ID [6.93]	Conduct [0.93]	Anxiety [0.9]	Congenital [1.05]	Dermatitis [0.56]	Depression [0.77]
UTs other than Delhi	Iron [3.92]	Neonatal [1.74]	Vit A [2.75]	Headaches [1.01]	Conduct [0.92]	Anxiety [0.78]	Congenital [1.0]	Depression [0.83]	Dermatitis [0.52]	Back Pain [0.58]

eFigure 9a. Leading ten causes of YLDs with the ratio of observed YLDs to YLDs expected on the basis of Socio-Demographic Index alone in 2017, <20 years, both sexes combined. The top ten causes contributing to YLDs are listed globally, by socio-demographic quintile, and then by GBD superregion, region, country, and subnationally where modeled. For each cell, the ratio of observed YLDs to YLDs expected on the basis of socio-demographic index (SDI) alone are listed. Abbreviations: YLD=year of life lived with disability, GBD=Global Burden of Disease.

Values shown in brackets represent the ratio of observed YLDs to predicted YLDs on the basis of Socio-Demographic Index (SDI), rounded to two (2) digits. Color ranges (shown below) were calculated to place a roughly equal number of cells into each bin.

COLOR KEY:	[0.0-0.78]	(0.78-0.88]	(0.88-0.94]	(0.94-1.0]	(1.0-1.06]	(1.06-1.18]	(1.18-1.38]	(1.38-1.66]	1.66+

	1	2	3	4	5	6	7	8	9	10
Nepal	Neonatal [1.68]	Iron [0.7]	Headaches [1.54]	Vit A [0.48]	Dermatitis [0.88]	Anxiety [1.06]	Back Pain [1.26]	Conduct [0.9]	Epilepsy [1.05]	Depression [0.93]
Pakistan	Iron [1.49]	Neonatal [1.39]	Headaches [0.93]	Vit A [0.76]	Dermatitis [0.93]	Diarrhea [1.2]	Conduct [0.84]	Anxiety [0.96]	Epilepsy [1.0]	Depression [0.77]
**Southeast Asia, East Asia, and Oceania**	Neonatal [1.09]	Headaches [0.6]	Conduct [0.79]	Asthma [0.83]	Anxiety [0.71]	Dermatitis [0.63]	Congenital [0.88]	Back Pain [0.67]	Depression [0.64]	Skin Viral [1.11]
**East Asia**	Neonatal [1.37]	Anxiety [0.71]	Conduct [0.7]	Headaches [0.44]	Asthma [0.67]	Dermatitis [0.5]	Back Pain [0.62]	Skin Viral [1.18]	Congenital [0.72]	Depression [0.58]
China	Neonatal [1.39]	Anxiety [0.71]	Conduct [0.7]	Headaches [0.43]	Asthma [0.66]	Dermatitis [0.5]	Back Pain [0.61]	Skin Viral [1.19]	Congenital [0.72]	Depression [0.58]
N Korea	Neonatal [0.67]	Iron [0.55]	Diarrhea [1.49]	Anxiety [1.06]	Headaches [0.67]	Back Pain [1.09]	Conduct [0.83]	Asthma [0.71]	Dermatitis [0.61]	Vit A [0.53]
Taiwan	Headaches [0.72]	Asthma [1.32]	Back Pain [0.88]	Anxiety [0.73]	Neonatal [0.58]	Conduct [0.87]	Dermatitis [0.57]	Diarrhea [2.31]	Congenital [0.84]	Skin Viral [1.05]
**Oceania**	Iron [1.19]	Asthma [1.86]	Neonatal [0.76]	Diarrhea [1.4]	Dermatitis [0.98]	Headaches [0.71]	Vit A [0.53]	Conduct [0.84]	Anxiety [0.88]	Congenital [0.92]
Am Samoa	Asthma [2.0]	Iron [1.69]	Neonatal [0.7]	Diarrhea [2.51]	Headaches [0.7]	Dermatitis [0.85]	Conduct [0.89]	Anxiety [0.79]	Vit A [1.65]	Back Pain [0.77]
Micronesia	Iron [1.29]	Asthma [1.72]	Vit A [1.47]	Neonatal [0.69]	Diarrhea [1.7]	Headaches [0.74]	Dermatitis [0.96]	Conduct [0.9]	Anxiety [0.93]	Back Pain [0.86]
Fiji	Iron [1.75]	Asthma [1.73]	Neonatal [0.67]	Diarrhea [2.08]	Dermatitis [0.94]	Headaches [0.64]	Conduct [0.82]	Vit A [1.13]	Anxiety [0.79]	Depression [0.79]
Guam	Diarrhea [5.92]	Asthma [1.98]	Neonatal [0.77]	Dermatitis [0.84]	Headaches [0.62]	Iron [2.71]	Conduct [0.83]	Anxiety [0.66]	Depression [0.88]	Back Pain [0.62]
Kiribati	Iron [0.91]	Vit A [0.83]	Asthma [1.86]	Diarrhea [1.33]	Neonatal [0.76]	Dermatitis [0.99]	Headaches [0.74]	Conduct [0.84]	Anxiety [0.9]	Congenital [0.91]
Marshall	Iron [1.04]	Vit A [1.4]	Asthma [1.64]	Neonatal [0.71]	Diarrhea [1.56]	Dermatitis [0.99]	Headaches [0.7]	Conduct [0.86]	Anxiety [0.9]	Congenital [0.91]
N Mariana	Asthma [1.89]	Neonatal [0.84]	Headaches [0.82]	Diarrhea [3.24]	Conduct [0.96]	Anxiety [0.83]	Dermatitis [0.77]	Iron [1.78]	Depression [1.01]	Back Pain [0.85]
PNG	Iron [1.06]	Asthma [1.93]	Neonatal [0.81]	Diarrhea [1.26]	Dermatitis [0.98]	Headaches [0.75]	Conduct [0.85]	Vit A [0.38]	Anxiety [0.9]	Congenital [0.91]
Samoa	Iron [1.22]	Asthma [1.71]	Vit A [1.28]	Neonatal [0.73]	Diarrhea [1.92]	Dermatitis [0.99]	Headaches [0.66]	Conduct [0.82]	Anxiety [0.84]	Congenital [0.89]
Solomon	Iron [1.23]	Vit A [0.8]	Asthma [1.76]	Neonatal [0.84]	Diarrhea [1.27]	Dermatitis [0.98]	Headaches [0.74]	Conduct [0.86]	Anxiety [0.9]	Congenital [0.9]
Tonga	Asthma [2.26]	Neonatal [0.69]	Diarrhea [2.01]	Dermatitis [0.94]	Headaches [0.68]	Conduct [0.86]	Iron [0.69]	Anxiety [0.85]	Vit A [1.1]	Back Pain [0.8]
Vanuatu	Iron [1.14]	Asthma [1.95]	Vit A [1.0]	Neonatal [0.78]	Dermatitis [0.99]	Diarrhea [1.24]	Headaches [0.69]	Conduct [0.84]	Anxiety [0.88]	Congenital [0.89]
**Southeast Asia**	Neonatal [0.72]	Headaches [0.84]	Conduct [0.93]	Asthma [1.03]	Dermatitis [0.8]	Congenital [1.11]	Iron [0.67]	Anxiety [0.72]	Back Pain [0.75]	Vit A [0.94]
Cambodia	Iron [1.31]	Neonatal [0.77]	Headaches [0.9]	Dermatitis [0.98]	Vit A [0.62]	Conduct [0.91]	Congenital [1.14]	Asthma [0.93]	Back Pain [1.08]	Diarrhea [0.87]
Indonesia	Neonatal [0.74]	Headaches [0.85]	Conduct [1.0]	Asthma [1.04]	Anxiety [0.74]	Congenital [1.0]	Vit A [1.08]	Back Pain [0.76]	Dermatitis [0.6]	Diarrhea [1.22]
Laos	Iron [0.65]	Headaches [0.88]	Vit A [0.89]	Dermatitis [0.98]	Neonatal [0.59]	Conduct [0.9]	Anxiety [1.03]	Asthma [0.97]	Congenital [1.17]	Back Pain [0.92]
Malaysia	Neonatal [0.82]	Headaches [0.75]	Depression [1.34]	Dermatitis [0.93]	Conduct [0.9]	Anxiety [0.81]	Diarrhea [2.24]	Congenital [1.09]	Asthma [0.9]	Back Pain [0.64]
Maldives	Neonatal [0.67]	Headaches [0.77]	Iron [1.03]	Dermatitis [0.94]	Conduct [0.86]	Asthma [0.97]	Congenital [1.09]	Anxiety [0.69]	Depression [0.79]	Scabies [3.28]
Mauritius	Iron [3.11]	Neonatal [0.82]	Headaches [0.89]	Depression [1.27]	Conduct [0.96]	Asthma [1.12]	Dermatitis [0.84]	Anxiety [0.74]	Congenital [1.05]	Diarrhea [1.77]
Myanmar	Iron [0.77]	Headaches [0.92]	Neonatal [0.73]	Congenital [1.63]	Dermatitis [0.98]	Conduct [0.95]	Vit A [0.76]	Asthma [0.86]	Anxiety [0.86]	Scabies [2.14]
Philippines	Neonatal [0.79]	Asthma [1.36]	Headaches [0.79]	Dermatitis [1.04]	Conduct [0.86]	Congenital [1.18]	Vit A [0.95]	Back Pain [0.85]	Iron [0.54]	Anxiety [0.74]
Sri Lanka	Neonatal [0.74]	Headaches [0.81]	Dermatitis [0.89]	Conduct [0.9]	Congenital [1.26]	Asthma [0.96]	Iron [0.86]	Anxiety [0.72]	Depression [0.89]	Epilepsy [1.32]
Seychelles	Neonatal [0.82]	Headaches [0.78]	Dermatitis [0.91]	Conduct [0.86]	Asthma [0.98]	Congenital [1.07]	Anxiety [0.68]	Back Pain [0.67]	Depression [0.73]	Diarrhea [1.21]

eFigure 9a. Leading ten causes of YLDs with the ratio of observed YLDs to YLDs expected on the basis of Socio-Demographic Index alone in 2017, <20 years, both sexes combined. The top ten causes contributing to YLDs are listed globally, by socio-demographic quintile, and then by GBD superregion, region, country, and subnationally where modeled. For each cell, the ratio of observed YLDs to YLDs expected on the basis of socio-demographic index (SDI) alone are listed. Abbreviations: YLD=year of life lived with disability, GBD=Global Burden of Disease.

Values shown in brackets represent the ratio of observed YLDs to predicted YLDs on the basis of Socio-Demographic Index (SDI), rounded to two (2) digits. Color ranges (shown below) were calculated to place a roughly equal number of cells into each bin.

COLOR KEY:	[0.0-0.78]	(0.78-0.88]	(0.88-0.94]	(0.94-1.0]	(1.0-1.06]	(1.06-1.18]	(1.18-1.38]	(1.38-1.66]	1.66+

	1	2	3	4	5	6	7	8	9	10
Thailand	Headaches [0.99]	Neonatal [0.66]	Dermatitis [0.94]	Conduct [0.95]	Asthma [0.95]	Congenital [1.11]	Anxiety [0.76]	Back Pain [0.76]	Depression [0.86]	Scabies [4.08]
Timor-Leste	Iron [1.39]	Headaches [0.89]	Vit A [0.8]	Neonatal [0.66]	Dermatitis [0.97]	Conduct [0.92]	Congenital [1.15]	Asthma [0.91]	Diarrhea [0.94]	Anxiety [0.83]
Vietnam	Headaches [0.81]	Neonatal [0.62]	Conduct [0.88]	Dermatitis [0.83]	Congenital [1.04]	Asthma [0.78]	Iron [0.45]	Depression [0.73]	Back Pain [0.66]	Diarrhea [0.91]
**Sub-Saharan Africa**	Iron [1.3]	Vit A [1.2]	Neonatal [1.08]	Dermatitis [0.99]	Headaches [0.78]	Diarrhea [1.09]	Congenital [1.17]	Conduct [0.92]	Anxiety [0.94]	Epilepsy [1.18]
**Central Sub-Saharan Africa**	Iron [1.16]	Vit A [1.44]	Neonatal [1.19]	Dermatitis [1.13]	Oncho [226.21]	Diarrhea [1.32]	Epilepsy [1.6]	Malaria [19.93]	Headaches [0.74]	Conduct [0.9]
Angola	Iron [1.89]	Vit A [1.25]	Neonatal [1.35]	Dermatitis [1.14]	Diarrhea [1.43]	Epilepsy [1.73]	Asthma [1.15]	Headaches [0.7]	Congenital [1.1]	Conduct [0.87]
C African Rep	Iron [1.27]	Vit A [1.05]	Neonatal [1.1]	Malaria [3.23]	Diarrhea [1.13]	Dermatitis [1.12]	Headaches [0.96]	Epilepsy [1.52]	Conduct [1.03]	Depression [1.39]
Congo	Iron [1.85]	Vit A [2.57]	Neonatal [1.29]	Dermatitis [1.13]	Epilepsy [2.03]	Asthma [1.22]	Diarrhea [1.63]	Headaches [0.7]	Conduct [0.9]	Depression [1.14]
Congo DR	Vit A [1.06]	Iron [0.69]	Neonatal [1.28]	Oncho [149.82]	Dermatitis [1.14]	Malaria [4.49]	Diarrhea [1.08]	Epilepsy [1.43]	Headaches [0.83]	Conduct [0.95]
Eq Guinea	Iron [1.72]	Neonatal [0.9]	Vit A [1.66]	Dermatitis [1.06]	Epilepsy [2.22]	Headaches [0.7]	Conduct [0.95]	Asthma [1.07]	Diarrhea [1.68]	Malaria [430.41]
Gabon	Iron [3.79]	Neonatal [1.38]	Vit A [2.29]	Dermatitis [1.05]	Epilepsy [2.35]	Headaches [0.71]	Asthma [1.15]	Diarrhea [1.83]	Conduct [0.89]	Depression [1.16]
**Eastern Sub-Saharan Africa**	Iron [0.81]	Vit A [0.87]	Neonatal [1.38]	Dermatitis [1.06]	Congenital [1.17]	Conduct [0.98]	Diarrhea [0.94]	Asthma [0.99]	Anxiety [1.03]	Depression [1.15]
Burundi	Iron [0.54]	Vit A [0.53]	Neonatal [1.16]	Dermatitis [1.15]	Diarrhea [0.84]	Congenital [1.1]	Conduct [0.96]	Epilepsy [1.2]	Anxiety [1.06]	Asthma [1.0]
Comoros	Iron [0.89]	Neonatal [1.5]	Vit A [0.78]	Dermatitis [1.11]	Conduct [0.98]	Asthma [1.07]	Diarrhea [1.02]	Anxiety [1.07]	Congenital [1.03]	Headaches [0.64]
Djibouti	Neonatal [1.4]	Iron [0.87]	Dermatitis [1.11]	PEM [13.81]	Conduct [0.89]	Congenital [1.07]	Asthma [0.9]	Epilepsy [1.3]	Anxiety [0.92]	Depression [1.06]
Eritrea	Iron [1.25]	Neonatal [1.52]	Vit A [0.78]	Dermatitis [1.11]	Conduct [0.98]	Diarrhea [0.99]	Anxiety [1.06]	Depression [1.22]	Congenital [1.05]	Epilepsy [1.24]
Ethiopia	Vit A [0.94]	Iron [0.61]	Neonatal [1.5]	Dermatitis [1.07]	Conduct [1.06]	Diarrhea [0.87]	Congenital [1.06]	Anxiety [1.08]	Asthma [0.91]	Depression [1.1]
Kenya	Neonatal [1.62]	Vit A [1.03]	Dermatitis [1.15]	Congenital [1.55]	Conduct [1.02]	Iron [0.43]	Diarrhea [1.16]	Anxiety [1.01]	Headaches [0.6]	Depression [0.98]
Baringo	Neonatal [1.64]	Vit A [0.95]	Dermatitis [1.11]	Congenital [1.5]	Conduct [1.1]	Diarrhea [1.14]	Anxiety [1.08]	Headaches [0.67]	Depression [1.04]	Asthma [0.74]
Bomet	Neonatal [1.89]	Dermatitis [1.12]	Congenital [1.52]	Vit A [0.7]	Conduct [1.0]	Anxiety [1.0]	Diarrhea [1.05]	Headaches [0.6]	Asthma [0.76]	Depression [0.95]
Bungoma	Neonatal [1.71]	Vit A [1.18]	Iron [0.49]	Dermatitis [1.13]	Congenital [1.52]	Conduct [1.02]	Diarrhea [1.11]	Anxiety [1.0]	Headaches [0.6]	Asthma [0.75]
Busia	Neonatal [1.68]	Vit A [1.0]	Iron [0.53]	Dermatitis [1.12]	Congenital [1.51]	Conduct [1.03]	Diarrhea [1.08]	Anxiety [1.03]	Headaches [0.63]	Schisto [78.59]
Elgeyo-Marakwet	Neonatal [1.83]	Dermatitis [1.13]	Congenital [1.52]	Conduct [1.07]	Vit A [0.63]	Anxiety [1.05]	Diarrhea [1.1]	Headaches [0.63]	Depression [1.02]	Epilepsy [1.16]
Embu	Neonatal [1.77]	Vit A [1.09]	Dermatitis [1.13]	Congenital [1.53]	Conduct [1.05]	Anxiety [1.03]	Diarrhea [1.23]	Iron [0.44]	Headaches [0.62]	Depression [1.02]
Garissa	Neonatal [2.03]	Vit A [0.61]	Iron [0.47]	Congenital [1.52]	Dermatitis [1.13]	Conduct [1.11]	Anxiety [1.06]	Headaches [0.69]	Diarrhea [0.67]	Depression [0.99]
HomaBay	Vit A [1.05]	Neonatal [1.12]	Iron [0.56]	HIV [102.92]	Congenital [1.54]	Dermatitis [1.12]	Diarrhea [1.23]	Conduct [1.03]	Anxiety [1.02]	Headaches [0.63]
Isiolo	Neonatal [1.52]	Vit A [0.76]	Iron [0.45]	Congenital [1.51]	Dermatitis [1.13]	Conduct [1.07]	Diarrhea [0.97]	Anxiety [1.05]	Headaches [0.67]	Depression [1.0]
Kajiado	Neonatal [1.92]	Vit A [1.29]	Iron [0.63]	Dermatitis [1.14]	Congenital [1.55]	Conduct [0.98]	Anxiety [0.96]	Headaches [0.57]	Epilepsy [1.26]	Diarrhea [1.02]
Kakamega	Neonatal [1.48]	Vit A [1.19]	Iron [0.68]	Dermatitis [1.12]	Congenital [1.52]	Diarrhea [1.2]	Conduct [1.02]	Anxiety [1.01]	Headaches [0.62]	Depression [0.96]
Kericho	Neonatal [2.0]	Dermatitis [1.13]	Congenital [1.55]	Vit A [0.78]	Conduct [0.99]	Diarrhea [1.25]	Anxiety [0.97]	Headaches [0.57]	Asthma [0.76]	Depression [0.93]
Kiambu	Neonatal [1.87]	Dermatitis [1.14]	Congenital [1.56]	Conduct [0.98]	Anxiety [0.96]	Headaches [0.56]	Diarrhea [1.21]	Epilepsy [1.33]	Depression [0.94]	Asthma [0.75]

eFigure 9a. Leading ten causes of YLDs with the ratio of observed YLDs to YLDs expected on the basis of Socio-Demographic Index alone in 2017, <20 years, both sexes combined.
The top ten causes contributing to YLDs are listed globally, by socio-demographic quintile, and then by GBD superregion, region, country, and subnationally where modeled. For each cell, the ratio of observed YLDs to YLDs expected on the basis of socio-demographic index (SDI) alone are listed. Abbreviations: YLD=year of life lived with disability, GBD=Global Burden of Disease.

Values shown in brackets represent the ratio of observed YLDs to predicted YLDs on the basis of Socio-Demographic Index (SDI), rounded to two (2) digits. Color ranges (shown below) were calculated to place a roughly equal number of cells into each bin.

COLOR KEY:	[0.0-0.78]	(0.78-0.88]	(0.88-0.94]	(0.94-1.0]	(1.0-1.06]	(1.06-1.18]	(1.18-1.38]	(1.38-1.66]	1.66+

	1	2	3	4	5	6	7	8	9	10
Kilifi	Neonatal [1.63]	Vit A [0.97]	Iron [0.52]	Dermatitis [1.13]	Congenital [1.51]	Conduct [1.03]	Diarrhea [1.06]	Anxiety [1.02]	Headaches [0.62]	Depression [0.96]
Kirinyaga	Neonatal [1.84]	Dermatitis [1.13]	Congenital [1.55]	Conduct [1.02]	Diarrhea [1.42]	Anxiety [1.01]	Headaches [0.6]	Depression [0.98]	Epilepsy [1.22]	Vit A [0.57]
Kisii	Neonatal [1.45]	Dermatitis [1.13]	Vit A [0.89]	Congenital [1.53]	Conduct [1.05]	Anxiety [1.04]	Diarrhea [1.24]	Headaches [0.62]	Depression [1.02]	Epilepsy [1.15]
Kisumu	Vit A [1.31]	Neonatal [1.1]	Iron [0.7]	Congenital [1.54]	Conduct [1.04]	Dermatitis [0.93]	Anxiety [1.04]	Diarrhea [1.1]	Headaches [0.63]	HIV [103.37]
Kitui	Neonatal [1.91]	Dermatitis [1.12]	Congenital [1.51]	Conduct [1.06]	Diarrhea [1.12]	Vit A [0.52]	Anxiety [1.06]	Headaches [0.65]	Depression [1.02]	Epilepsy [1.05]
Kwale	Neonatal [1.65]	Iron [0.67]	Vit A [0.95]	Dermatitis [1.13]	Congenital [1.49]	Conduct [1.04]	Anxiety [1.03]	Diarrhea [0.95]	Headaches [0.62]	Depression [0.98]
Laikipia	Neonatal [1.93]	Dermatitis [1.14]	Congenital [1.54]	Conduct [1.02]	Anxiety [0.98]	Diarrhea [1.24]	Headaches [0.58]	Vit A [0.7]	Asthma [0.79]	Depression [0.97]
Lamu	Neonatal [1.45]	Dermatitis [1.11]	Vit A [0.66]	Congenital [1.5]	Conduct [1.09]	Anxiety [1.08]	Diarrhea [1.06]	Iron [0.32]	Headaches [0.67]	Depression [1.05]
Machakos	Neonatal [1.81]	Dermatitis [1.13]	Congenital [1.52]	Conduct [1.05]	Vit A [0.71]	Diarrhea [1.23]	Anxiety [1.02]	Headaches [0.61]	Depression [1.0]	Epilepsy [1.16]
Makueni	Neonatal [1.8]	Dermatitis [1.11]	Congenital [1.51]	Conduct [1.06]	Anxiety [1.05]	Headaches [0.65]	Vit A [0.47]	Diarrhea [0.91]	Depression [1.03]	HIV [80.61]
Mandera	Vit A [0.69]	Neonatal [1.65]	Iron [0.49]	Congenital [1.49]	Dermatitis [1.11]	Conduct [1.22]	Anxiety [1.16]	Headaches [0.8]	Diarrhea [0.68]	Depression [1.14]
Marsabit	Neonatal [2.11]	Vit A [0.55]	Congenital [1.5]	Dermatitis [1.13]	Conduct [1.1]	Diarrhea [0.85]	Anxiety [1.09]	Iron [0.23]	PEM [2.93]	Headaches [0.71]
Meru	Neonatal [1.8]	Vit A [1.04]	Dermatitis [1.12]	Congenital [1.53]	Conduct [1.06]	Diarrhea [1.34]	Anxiety [1.05]	Headaches [0.64]	Depression [1.04]	Epilepsy [1.15]
Migori	Vit A [1.06]	Neonatal [1.08]	Iron [0.43]	Congenital [1.54]	Dermatitis [1.13]	Diarrhea [1.17]	Conduct [1.0]	HIV [69.89]	Anxiety [1.0]	Headaches [0.61]
Mombasa	Neonatal [1.53]	Iron [0.83]	Vit A [1.19]	Dermatitis [1.14]	Congenital [1.55]	Conduct [0.96]	Diarrhea [1.36]	Anxiety [0.96]	Headaches [0.57]	Epilepsy [1.3]
Murang'a	Neonatal [1.82]	Dermatitis [1.12]	Congenital [1.54]	Conduct [1.09]	Anxiety [1.06]	Headaches [0.65]	Diarrhea [1.14]	Depression [1.06]	Epilepsy [1.16]	Asthma [0.74]
Nairobi	Neonatal [1.74]	Dermatitis [1.36]	Congenital [1.48]	Conduct [0.91]	Anxiety [0.82]	Headaches [0.52]	Epilepsy [1.67]	Vit A [1.25]	Depression [0.87]	Asthma [0.76]
Nakuru	Neonatal [1.77]	Dermatitis [1.14]	Congenital [1.56]	Conduct [1.02]	Epilepsy [1.74]	Diarrhea [1.33]	Anxiety [0.99]	Vit A [0.7]	Headaches [0.59]	Asthma [0.78]
Nandi	Neonatal [1.9]	Dermatitis [1.13]	Congenital [1.53]	Vit A [0.77]	Conduct [1.04]	Anxiety [1.03]	Headaches [0.62]	Diarrhea [1.05]	Depression [1.0]	Asthma [0.75]
Narok	Neonatal [2.19]	Vit A [0.96]	Dermatitis [1.13]	Congenital [1.51]	Conduct [0.99]	Iron [0.28]	Diarrhea [0.94]	Anxiety [0.99]	Headaches [0.61]	Asthma [0.8]
Nyamira	Neonatal [1.66]	Dermatitis [1.14]	Congenital [1.55]	Conduct [1.02]	Diarrhea [1.4]	Anxiety [1.0]	Headaches [0.59]	Epilepsy [1.39]	Depression [0.99]	Asthma [0.76]
Nyandarua	Neonatal [1.92]	Dermatitis [1.12]	Congenital [1.55]	Conduct [1.07]	Anxiety [1.04]	Headaches [0.63]	Diarrhea [1.17]	Epilepsy [1.4]	Depression [1.07]	Vit A [0.61]
Nyeri	Neonatal [1.8]	Dermatitis [1.12]	Congenital [1.55]	Conduct [1.08]	Anxiety [1.05]	Headaches [0.64]	Epilepsy [1.38]	Depression [1.06]	Diarrhea [1.11]	Asthma [0.74]
Samburu	Neonatal [2.29]	Vit A [0.57]	Congenital [1.52]	Dermatitis [1.15]	Iron [0.32]	Diarrhea [0.91]	Conduct [1.06]	Anxiety [1.05]	Headaches [0.68]	Asthma [0.81]
Siaya	Neonatal [1.18]	Vit A [1.02]	Dermatitis [1.12]	Iron [0.45]	Congenital [1.52]	Conduct [1.03]	Diarrhea [1.17]	Anxiety [1.02]	Headaches [0.62]	Asthma [0.79]
TaitaTaveta	Neonatal [1.65]	Dermatitis [1.13]	Congenital [1.53]	Conduct [1.09]	Anxiety [1.06]	Headaches [0.63]	Diarrhea [1.14]	Epilepsy [1.36]	Depression [1.05]	Asthma [0.73]
TanaRiver	Iron [0.89]	Vit A [1.13]	Neonatal [1.8]	Congenital [1.51]	Dermatitis [1.13]	Conduct [1.07]	Diarrhea [0.95]	Anxiety [1.06]	Headaches [0.67]	Epilepsy [1.06]
TharakaNithi	Neonatal [1.45]	Dermatitis [1.13]	Congenital [1.53]	Conduct [1.07]	Anxiety [1.06]	Diarrhea [1.29]	Vit A [0.71]	Headaches [0.63]	Depression [1.06]	Epilepsy [1.3]
TransNzoia	Neonatal [1.8]	Dermatitis [1.12]	Vit A [0.81]	Congenital [1.51]	Conduct [1.04]	Diarrhea [1.32]	Anxiety [1.03]	Headaches [0.62]	Depression [1.01]	Asthma [0.75]
Turkana	Neonatal [1.99]	Vit A [0.63]	Iron [0.37]	Congenital [1.53]	Dermatitis [1.13]	Diarrhea [0.91]	Conduct [1.12]	Anxiety [1.09]	PEM [1.96]	Headaches [0.73]
UasinGishu	Neonatal [1.74]	Dermatitis [1.64]	Vit A [1.0]	Congenital [1.53]	Conduct [1.0]	Diarrhea [1.32]	Anxiety [0.99]	Headaches [0.59]	Iron [0.41]	Depression [0.96]

eFigure 9a. Leading ten causes of YLDs with the ratio of observed YLDs to YLDs expected on the basis of Socio-Demographic Index alone in 2017, <20 years, both sexes combined.
The top ten causes contributing to YLDs are listed globally, by socio-demographic quintile, and then by GBD superregion, region, country, and subnationally where modeled. For each cell, the ratio of observed YLDs to YLDs expected on the basis of socio-demographic index (SDI) alone are listed. Abbreviations: YLD=year of life lived with disability, GBD=Global Burden of Disease.

Values shown in brackets represent the ratio of observed YLDs to predicted YLDs on the basis of Socio-Demographic Index (SDI), rounded to two (2) digits. Color ranges (shown below) were calculated to place a roughly equal number of cells into each bin.

COLOR KEY:	[0.0-0.78]	(0.78-0.88]	(0.88-0.94]	(0.94-1.0]	(1.0-1.06]	(1.06-1.18]	(1.18-1.38]	(1.38-1.66]	1.66+

	1	2	3	4	5	6	7	8	9	10
Vihiga	Neonatal [1.66]	Vit A [0.98]	Dermatitis [1.11]	Congenital [1.51]	Conduct [1.07]	Iron [0.41]	Diarrhea [1.14]	Anxiety [1.07]	Headaches [0.66]	Depression [1.04]
Wajir	Neonatal [2.04]	Vit A [0.48]	Congenital [1.52]	Dermatitis [1.14]	Conduct [1.18]	Iron [0.22]	Diarrhea [0.65]	Anxiety [1.12]	Headaches [0.77]	Depression [1.04]
WestPokot	Neonatal [2.05]	Vit A [0.81]	Congenital [1.51]	Dermatitis [1.13]	Iron [0.31]	Diarrhea [1.05]	Conduct [1.05]	Anxiety [1.05]	Headaches [0.66]	Depression [0.99]
Madagascar	Iron [0.8]	Congenital [2.54]	Neonatal [1.45]	Vit A [0.61]	Diarrhea [1.1]	Dermatitis [1.13]	Asthma [1.13]	Conduct [0.95]	Epilepsy [1.25]	Anxiety [1.04]
Malawi	Iron [1.19]	Vit A [0.84]	Neonatal [1.28]	Diarrhea [1.21]	Dermatitis [1.13]	HIV [55.32]	Conduct [0.96]	Congenital [1.09]	Anxiety [1.07]	Asthma [0.98]
Mozambique	Iron [0.96]	Vit A [0.89]	Neonatal [1.21]	HIV [54.33]	Dermatitis [0.95]	Epilepsy [1.46]	Malaria [2.48]	Conduct [0.99]	Anxiety [1.09]	Congenital [1.04]
Rwanda	Iron [0.69]	Neonatal [1.33]	Vit A [0.66]	Asthma [1.32]	Conduct [0.93]	Depression [1.24]	Congenital [1.08]	Anxiety [1.02]	Diarrhea [0.88]	Epilepsy [1.24]
Somalia	Iron [0.85]	Vit A [0.71]	Neonatal [1.44]	Dermatitis [1.14]	Congenital [1.06]	Diarrhea [0.67]	Conduct [1.0]	Anxiety [1.08]	Epilepsy [1.04]	Depression [1.17]
S Sudan	Iron [0.9]	Vit A [0.75]	Oncho [84.59]	Neonatal [1.34]	Diarrhea [1.1]	Dermatitis [1.14]	Asthma [1.11]	Conduct [0.99]	Congenital [1.02]	PEM [1.82]
Tanzania	Iron [1.22]	Neonatal [1.29]	Vit A [0.76]	Asthma [1.28]	Dermatitis [0.95]	Conduct [0.9]	Anxiety [0.99]	Epilepsy [1.18]	Depression [1.11]	Congenital [0.89]
Uganda	Iron [0.84]	Neonatal [1.31]	Vit A [0.73]	Dermatitis [1.09]	Depression [1.59]	Epilepsy [1.43]	Asthma [1.1]	Malaria [5.76]	Diarrhea [0.96]	Conduct [0.93]
Zambia	Iron [0.9]	Vit A [1.32]	Neonatal [1.15]	Dermatitis [1.12]	Diarrhea [1.2]	Epilepsy [1.5]	Headaches [0.73]	Conduct [0.89]	Congenital [1.07]	Anxiety [0.97]
**Southern Sub-Saharan Africa**	Neonatal [1.07]	Dermatitis [1.29]	Iron [1.07]	Vit A [1.7]	Headaches [0.7]	Conduct [0.93]	Congenital [1.21]	Anxiety [0.9]	HIV [248.67]	Depression [0.91]
Botswana	Iron [2.34]	Neonatal [1.29]	Dermatitis [1.28]	Vit A [2.07]	Headaches [0.7]	Conduct [0.91]	Anxiety [0.85]	Depression [1.02]	Diarrhea [1.39]	Congenital [0.84]
Lesotho	Iron [0.94]	Vit A [1.2]	Neonatal [0.95]	Dermatitis [1.36]	Depression [1.83]	HIV [150.1]	Headaches [0.81]	Conduct [0.96]	Diarrhea [1.14]	Anxiety [1.02]
Namibia	Iron [1.75]	Neonatal [1.12]	Dermatitis [1.34]	Headaches [0.71]	Diarrhea [1.67]	Conduct [0.9]	Anxiety [0.9]	Vit A [1.05]	Depression [0.89]	HIV [188.47]
S Africa	Neonatal [1.1]	Dermatitis [1.24]	Headaches [0.69]	Congenital [1.43]	Vit A [1.84]	Conduct [0.95]	Anxiety [0.91]	HIV [339.23]	Depression [0.92]	Iron [0.8]
Swaziland	Iron [1.2]	Neonatal [0.91]	Dermatitis [1.37]	Vit A [1.27]	HIV [246.47]	Headaches [0.74]	Diarrhea [1.6]	Conduct [0.93]	Anxiety [0.95]	Depression [1.12]
Zimbabwe	Iron [0.79]	Neonatal [1.13]	Vit A [0.86]	Dermatitis [1.37]	Diarrhea [1.29]	Headaches [0.79]	Conduct [0.93]	Anxiety [0.89]	Congenital [0.76]	HIV [58.79]
**Western Sub-Saharan Africa**	Iron [1.73]	Vit A [1.27]	Neonatal [0.85]	Headaches [0.98]	Diarrhea [1.1]	Congenital [1.17]	Dermatitis [0.85]	Conduct [0.9]	Malaria [12.57]	Hemog [2.33]
Benin	Iron [1.08]	Vit A [1.05]	Neonatal [1.2]	Malaria [5.54]	Headaches [1.0]	Dermatitis [0.92]	Conduct [0.94]	Epilepsy [1.2]	Congenital [0.97]	Back Pain [1.12]
Burkina Faso	Iron [1.56]	Vit A [1.0]	Malaria [1.83]	Neonatal [1.02]	Headaches [1.11]	Diarrhea [0.92]	Dermatitis [0.93]	Hemog [1.74]	Conduct [0.96]	Congenital [0.97]
Cameroon	Iron [1.13]	Vit A [1.39]	Neonatal [0.8]	Headaches [0.96]	Diarrhea [1.51]	Dermatitis [0.92]	Conduct [0.93]	Malaria [28.78]	Epilepsy [1.36]	Oncho [183.02]
Cape Verde	Iron [1.37]	Neonatal [1.43]	Headaches [1.01]	Conduct [0.98]	Dermatitis [0.91]	Anxiety [0.94]	Diarrhea [1.18]	Back Pain [1.02]	Depression [1.05]	Epilepsy [1.32]
Chad	Iron [1.6]	Vit A [0.83]	Diarrhea [1.29]	Neonatal [0.9]	Headaches [1.09]	Dermatitis [0.94]	Conduct [0.94]	Congenital [0.95]	Back Pain [1.36]	Epilepsy [0.97]
Cote d'Ivoire	Iron [1.42]	Vit A [1.24]	Neonatal [0.98]	Headaches [0.98]	Diarrhea [1.25]	Dermatitis [0.91]	Conduct [0.93]	Epilepsy [1.23]	Congenital [1.01]	Hemog [2.03]
Gambia	Iron [1.73]	Vit A [1.33]	Neonatal [1.54]	Headaches [1.05]	Diarrhea [1.18]	Dermatitis [0.91]	Conduct [0.96]	Depression [1.18]	Anxiety [0.94]	Congenital [0.94]
Ghana	Iron [2.11]	Vit A [2.14]	Neonatal [1.11]	Headaches [0.93]	Diarrhea [1.34]	Conduct [0.91]	Epilepsy [1.39]	Anxiety [0.87]	Congenital [1.01]	Malaria [82.53]
Guinea	Iron [1.15]	Vit A [0.86]	Neonatal [1.02]	Malaria [2.34]	Headaches [1.08]	Diarrhea [0.95]	Dermatitis [0.93]	Conduct [0.96]	Hemog [1.74]	Congenital [0.97]
Guinea-Bissau	Iron [1.29]	Vit A [1.09]	Neonatal [1.24]	Headaches [1.06]	Dermatitis [0.93]	Diarrhea [0.88]	Conduct [0.94]	Congenital [1.03]	Epilepsy [1.16]	Anxiety [0.93]
Liberia	Iron [1.18]	Oncho [208.34]	Vit A [0.73]	Neonatal [1.21]	Diarrhea [1.34]	Headaches [1.12]	Dermatitis [0.92]	Epilepsy [1.38]	Conduct [0.99]	Hemog [1.85]

eFigure 9a. Leading ten causes of YLDs with the ratio of observed YLDs to YLDs expected on the basis of Socio-Demographic Index alone in 2017, <20 years, both sexes combined.
The top ten causes contributing to YLDs are listed globally, by socio-demographic quintile, and then by GBD superregion, region, country, and subnationally where modeled. For each cell, the ratio of observed YLDs to YLDs expected on the basis of socio-demographic index (SDI) alone are listed. Abbreviations: YLD=year of life lived with disability, GBD=Global Burden of Disease.

Values shown in brackets represent the ratio of observed YLDs to predicted YLDs on the basis of Socio-Demographic Index (SDI), rounded to two (2) digits. Color ranges (shown below) were calculated to place a roughly equal number of cells into each bin.

COLOR KEY:	[0.0-0.78]	(0.78-0.88]	(0.88-0.94]	(0.94-1.0]	(1.0-1.06]	(1.06-1.18]	(1.18-1.38]	(1.38-1.66]	1.66+

	1	2	3	4	5	6	7	8	9	10
Mali	**Iron** [1.69]	**Vit A** [1.03]	**Neonatal** [0.98]	**Headaches** [1.14]	**Malaria** [1.23]	**Diarrhea** [0.8]	**Conduct** [0.97]	**Dermatitis** [0.81]	**Congenital** [0.96]	**Anxiety** [0.95]
Mauritania	**Iron** [1.42]	**PEM** [27.03]	**Neonatal** [1.3]	**Vit A** [1.23]	**Headaches** [0.97]	**Diarrhea** [1.24]	**Dermatitis** [0.92]	**Conduct** [0.93]	**Asthma** [0.93]	**Epilepsy** [1.26]
Niger	**Iron** [1.02]	**Vit A** [0.71]	**Neonatal** [0.97]	**Diarrhea** [0.76]	**Malaria** [1.03]	**Headaches** [1.16]	**Dermatitis** [0.96]	**Conduct** [0.96]	**Congenital** [0.92]	**PEM** [0.87]
Nigeria	**Iron** [2.24]	**Vit A** [1.45]	**Headaches** [0.94]	**Neonatal** [0.68]	**Congenital** [1.4]	**Hemog** [3.21]	**Dermatitis** [0.84]	**Back Pain** [1.17]	**Conduct** [0.87]	**Diarrhea** [1.0]
Sao Tome Principe	**Iron** [0.94]	**Neonatal** [1.27]	**Vit A** [1.29]	**Headaches** [1.0]	**Diarrhea** [1.28]	**Dermatitis** [0.92]	**Conduct** [0.97]	**Anxiety** [0.94]	**Epilepsy** [1.29]	**Congenital** [0.98]
Senegal	**Iron** [1.48]	**Vit A** [0.94]	**Neonatal** [1.48]	**Diarrhea** [1.28]	**Headaches** [1.06]	**Dermatitis** [0.91]	**Conduct** [0.97]	**Congenital** [0.97]	**Epilepsy** [1.12]	**Anxiety** [0.93]
Sierra Leone	**Iron** [1.4]	**Vit A** [0.94]	**Malaria** [4.86]	**Headaches** [1.11]	**Neonatal** [0.87]	**Hemog** [2.32]	**Dermatitis** [0.92]	**Diarrhea** [0.93]	**Conduct** [0.97]	**Epilepsy** [1.19]
Togo	**Iron** [1.16]	**Vit A** [1.14]	**Neonatal** [1.18]	**Malaria** [15.31]	**Headaches** [1.01]	**Diarrhea** [1.26]	**Dermatitis** [0.91]	**Conduct** [0.96]	**Epilepsy** [1.23]	**Congenital** [0.98]

eFigure 9b. Leading ten causes of YLDs with the ratio of observed YLDs to YLDs expected on the basis of Socio-Demographic Index alone in 2017, <1 years, both sexes combined. The top ten causes contributing to YLDs are listed globally, by socio-demographic quintile, and then by GBD superregion, region, country, and subnationally where modeled. For each cell, the ratio of observed YLDs to YLDs expected on the basis of socio-demographic index (SDI) alone are listed. Abbreviations: YLD=year of life lived with disability, GBD=Global Burden of Disease.

Values shown in brackets represent the ratio of observed YLDs to predicted YLDs on the basis of Socio-Demographic Index (SDI), rounded to two (2) digits. Color ranges (shown below) were calculated to place a roughly equal number of cells into each bin.

COLOR KEY:	[0.0-0.68]	(0.68-0.82]	(0.82-0.91]	(0.91-1.01]	(1.01-1.1]	(1.1-1.21]	(1.21-1.45]	(1.45-2.25]	2.25+

	1	2	3	4	5	6	7	8	9	10
Global	Neonatal [1.38]	Vit A [1.88]	Iron [2.11]	Diarrhea [1.3]	Congenital [0.95]	PEM [12.99]	URI [0.88]	Epilepsy [1.09]	Urticaria [0.84]	Hemog [2.77]
Low SDI	Vit A [1.15]	Iron [1.34]	Neonatal [1.28]	Diarrhea [0.89]	Congenital [1.09]	PEM [1.35]	Hemog [1.53]	ID [3.11]	URI [0.96]	Epilepsy [0.97]
Low-middle SDI	Neonatal [1.34]	Iron [1.73]	Vit A [1.16]	Diarrhea [1.08]	Congenital [1.02]	PEM [3.14]	Hemog [2.2]	Epilepsy [1.06]	URI [0.84]	Urticaria [0.88]
Middle SDI	Neonatal [1.26]	Vit A [1.21]	Diarrhea [1.26]	Congenital [0.83]	Iron [1.01]	PEM [13.88]	URI [0.91]	Urticaria [0.85]	Epilepsy [1.07]	ASD [0.73]
High-middle SDI	Neonatal [1.66]	Diarrhea [2.33]	Congenital [0.92]	Vit A [3.85]	Iron [4.22]	URI [0.81]	Urticaria [1.22]	Other MN [8.47]	Epilepsy [1.58]	ASD [0.76]
High SDI	Neonatal [0.83]	Congenital [0.88]	URI [1.14]	Diarrhea [0.73]	ASD [1.12]	Hernia [0.94]	Urticaria [0.74]	Iron [0.41]	Epilepsy [0.74]	Vit A [0.26]
Central Europe, Eastern Europe, and Central Asia	Neonatal [1.42]	Congenital [1.0]	Diarrhea [1.45]	Iron [2.35]	Vit A [1.1]	Hernia [1.47]	Urticaria [1.07]	PEM [76.89]	ASD [0.79]	URI [0.52]
Central Asia	Neonatal [1.07]	Iron [1.67]	Congenital [0.98]	Vit A [0.94]	Diarrhea [0.86]	PEM [18.69]	Hernia [2.66]	Urticaria [0.96]	Epilepsy [1.05]	ASD [0.84]
Armenia	Iron [3.88]	Neonatal [1.03]	Congenital [0.95]	Diarrhea [0.92]	Hernia [2.32]	Urticaria [0.98]	Epilepsy [1.03]	ASD [0.83]	Oth NTD [575.48]	URI [0.51]
Azerbaijan	Neonatal [1.15]	Iron [2.56]	Congenital [0.97]	Diarrhea [1.4]	Vit A [0.77]	Hernia [2.14]	Urticaria [0.98]	Epilepsy [1.18]	ASD [0.83]	Hemog [2.61]
Georgia	Neonatal [1.03]	Congenital [0.96]	Diarrhea [0.83]	Iron [0.72]	Vit A [0.54]	Urticaria [0.99]	Epilepsy [1.11]	ASD [0.82]	Hernia [1.06]	URI [0.37]
Kazakhstan	Neonatal [1.15]	Vit A [1.74]	Congenital [0.98]	Iron [1.32]	Diarrhea [0.76]	Hernia [1.72]	Urticaria [1.03]	Epilepsy [1.35]	ASD [0.8]	URI [0.45]
Kyrgyzstan	Iron [1.94]	Neonatal [1.01]	Congenital [1.01]	Vit A [0.74]	Hernia [7.15]	Diarrhea [0.6]	Urticaria [0.92]	Epilepsy [0.87]	ASD [0.9]	Hemog [1.6]
Mongolia	Neonatal [1.09]	Vit A [1.09]	Congenital [1.02]	Diarrhea [1.21]	Iron [0.58]	Hernia [2.65]	Urticaria [0.95]	Epilepsy [1.1]	ASD [0.85]	URI [0.37]
Tajikistan	PEM [11.27]	Neonatal [0.96]	Vit A [0.68]	Diarrhea [1.15]	Congenital [1.06]	Iron [0.62]	Hernia [4.96]	Urticaria [0.95]	ASD [0.95]	Epilepsy [0.74]
Turkmenistan	Neonatal [1.09]	Congenital [0.98]	Iron [1.3]	Vit A [0.7]	Diarrhea [0.73]	Hernia [2.16]	Urticaria [0.98]	Epilepsy [1.08]	ASD [0.82]	URI [0.37]
Uzbekistan	Neonatal [0.99]	Iron [1.57]	Congenital [0.98]	Vit A [0.6]	Hernia [3.2]	Diarrhea [0.48]	Urticaria [0.93]	Epilepsy [0.96]	ASD [0.87]	PEM [2.29]
Central Europe	Neonatal [1.46]	Congenital [1.15]	Diarrhea [1.72]	Vit A [3.35]	Iron [2.14]	Hernia [1.1]	Urticaria [1.18]	ASD [0.79]	URI [0.58]	Epilepsy [1.23]
Albania	Neonatal [1.26]	Vit A [1.72]	Congenital [1.11]	Diarrhea [0.99]	Iron [0.81]	PEM [18.58]	Hernia [2.46]	Urticaria [0.96]	Epilepsy [1.16]	ASD [0.84]
Bosnia	Neonatal [1.18]	Congenital [1.07]	Vit A [1.54]	Diarrhea [1.12]	Iron [0.88]	Hernia [2.16]	Urticaria [1.0]	URI [0.61]	ASD [0.82]	Epilepsy [0.88]
Bulgaria	Neonatal [1.27]	Congenital [1.1]	Diarrhea [1.63]	Vit A [2.56]	Hernia [1.47]	Iron [1.67]	Urticaria [1.12]	URI [0.58]	ASD [0.79]	Epilepsy [1.11]
Croatia	Neonatal [1.58]	Congenital [1.09]	Diarrhea [1.4]	Hernia [1.07]	Urticaria [1.21]	Vit A [1.32]	URI [0.58]	ASD [0.8]	Epilepsy [1.17]	Blindness [1.35]
Czech	Neonatal [1.07]	Congenital [1.19]	Diarrhea [1.82]	Vit A [2.59]	Hernia [1.11]	Urticaria [1.3]	ASD [0.81]	URI [0.58]	Falls [1.56]	Epilepsy [1.38]
Hungary	Neonatal [1.38]	Congenital [1.2]	Diarrhea [2.28]	Vit A [2.44]	Hernia [1.18]	Urticaria [1.18]	URI [0.58]	ASD [0.79]	Epilepsy [1.2]	Falls [1.53]
Macedonia	Neonatal [1.3]	Vit A [2.62]	Congenital [1.09]	Diarrhea [1.48]	Iron [1.79]	Hernia [1.78]	Urticaria [1.05]	URI [0.59]	ASD [0.8]	Epilepsy [0.89]
Montenegro	Neonatal [1.31]	Congenital [1.09]	Vit A [2.27]	Iron [2.25]	Diarrhea [1.0]	Hernia [1.52]	Urticaria [1.11]	ASD [0.79]	URI [0.58]	Epilepsy [1.08]
Poland	Neonatal [1.73]	Congenital [1.15]	Diarrhea [1.74]	Vit A [3.56]	Urticaria [1.28]	Hernia [0.75]	ASD [0.81]	URI [0.58]	Epilepsy [1.45]	Iron [1.63]
Romania	Neonatal [1.45]	Vit A [3.64]	Congenital [1.15]	Diarrhea [1.99]	Iron [3.66]	Urticaria [1.1]	Hernia [1.06]	URI [0.58]	ASD [0.78]	Epilepsy [1.17]
Serbia	Neonatal [1.53]	Congenital [1.19]	Vit A [2.08]	Diarrhea [1.04]	Hernia [2.22]	Iron [1.07]	Urticaria [1.04]	URI [0.59]	ASD [0.81]	Falls [1.95]
Slovakia	Neonatal [1.16]	Congenital [1.26]	Diarrhea [1.97]	Vit A [3.78]	Hernia [1.12]	Urticaria [1.26]	ASD [0.81]	URI [0.58]	Epilepsy [1.37]	Falls [1.42]
Slovenia	Neonatal [1.25]	Congenital [1.17]	Diarrhea [1.87]	Vit A [3.38]	Hernia [1.0]	Iron [3.33]	Urticaria [1.34]	ASD [0.82]	URI [0.58]	Falls [1.34]

eFigure 9b. Leading ten causes of YLDs with the ratio of observed YLDs to YLDs expected on the basis of Socio-Demographic Index alone in 2017, <1 years, both sexes combined.
The top ten causes contributing to YLDs are listed globally, by socio-demographic quintile, and then by GBD superregion, region, country, and subnationally where modeled. For each cell, the ratio of observed YLDs to YLDs expected on the basis of socio-demographic index (SDI) alone are listed. Abbreviations: YLD=year of life lived with disability, GBD=Global Burden of Disease.

Values shown in brackets represent the ratio of observed YLDs to predicted YLDs on the basis of Socio-Demographic Index (SDI), rounded to two (2) digits. Color ranges (shown below) were calculated to place a roughly equal number of cells into each bin.

COLOR KEY:	[0.0-0.68]	(0.68-0.82]	(0.82-0.91]	(0.91-1.01]	(1.01-1.1]	(1.1-1.21]	(1.21-1.45]	(1.45-2.25]	2.25+

	1	2	3	4	5	6	7	8	9	10
**Eastern Europe**	Neonatal [1.65]	Diarrhea [1.8]	Congenital [0.96]	Iron [2.47]	Hernia [1.15]	Urticaria [1.12]	URI [0.63]	ASD [0.78]	Falls [1.67]	Epilepsy [0.68]
Belarus	Neonatal [1.52]	Congenital [0.95]	Diarrhea [1.06]	Iron [1.36]	Hernia [1.18]	Urticaria [1.09]	URI [0.62]	ASD [0.79]	Falls [1.83]	Epilepsy [0.69]
Estonia	Neonatal [1.79]	Diarrhea [2.52]	Congenital [1.03]	Iron [3.58]	Urticaria [1.35]	Hernia [0.83]	URI [0.61]	ASD [0.81]	Falls [1.26]	Other MN [6.85]
Latvia	Neonatal [1.54]	Diarrhea [1.95]	Congenital [0.92]	Iron [2.92]	Hernia [0.96]	Urticaria [1.21]	URI [0.61]	ASD [0.78]	Falls [1.51]	Epilepsy [0.74]
Lithuania	Neonatal [1.78]	Congenital [1.12]	Diarrhea [1.96]	Iron [3.58]	Urticaria [1.27]	URI [0.61]	Hernia [0.69]	ASD [0.79]	Falls [1.38]	Epilepsy [0.86]
Moldova	Neonatal [1.53]	Iron [1.68]	Congenital [0.97]	Diarrhea [1.23]	Hernia [1.97]	Urticaria [0.96]	URI [0.65]	ASD [0.84]	Falls [3.69]	Whooping [1.79]
Russian Federation	Neonatal [1.67]	Diarrhea [2.13]	Congenital [0.95]	Iron [2.55]	Hernia [1.1]	Urticaria [1.13]	URI [0.63]	ASD [0.78]	Falls [1.64]	PEM [72.55]
Ukraine	Neonatal [1.56]	Congenital [0.98]	Iron [1.78]	Diarrhea [0.74]	Hernia [1.52]	Urticaria [1.04]	URI [0.64]	ASD [0.8]	Whooping [2.93]	Falls [2.0]
**High-income**	Neonatal [0.98]	Congenital [0.97]	Diarrhea [1.12]	URI [1.14]	ASD [1.16]	Hernia [0.71]	Iron [2.2]	Urticaria [0.91]	Epilepsy [1.02]	Vit A [0.93]
**Australasia**	Neonatal [0.93]	Congenital [0.81]	Hernia [1.69]	URI [1.03]	Diarrhea [1.01]	ASD [1.05]	Urticaria [1.19]	Falls [1.21]	Other MN [2.97]	Med Treat [7.76]
Australia	Neonatal [0.91]	Congenital [0.81]	Hernia [1.71]	URI [1.03]	Diarrhea [1.07]	ASD [1.05]	Urticaria [1.21]	Falls [1.21]	Other MN [2.94]	Epilepsy [0.59]
New Zealand	Neonatal [1.04]	Congenital [0.81]	Hernia [1.63]	URI [1.04]	Diarrhea [0.73]	ASD [1.01]	Urticaria [1.09]	Falls [1.22]	Med Treat [10.67]	Epilepsy [0.53]
**High-income Asia Pacific**	Neonatal [1.19]	Congenital [1.0]	URI [1.06]	ASD [1.3]	Iron [3.67]	Vit A [1.88]	Urticaria [1.09]	Diarrhea [0.56]	Hernia [0.57]	Falls [0.91]
Brunei	Neonatal [1.23]	Congenital [0.85]	Iron [5.55]	URI [1.03]	ASD [1.19]	Diarrhea [0.57]	Urticaria [1.02]	Hernia [0.55]	Falls [1.22]	Epilepsy [0.84]
Japan	Neonatal [1.15]	Congenital [1.08]	URI [1.06]	ASD [1.38]	Vit A [1.94]	Urticaria [1.08]	Hernia [0.6]	PEM [1095.99]	Diarrhea [0.5]	Iron [1.92]
Aichi	Neonatal [1.22]	Congenital [1.04]	ASD [2.04]	URI [1.07]	Urticaria [1.12]	Vit A [1.82]	Diarrhea [0.56]	PEM [1561.09]	Hernia [0.57]	Endocrine [2.75]
Akita	Neonatal [1.2]	Congenital [1.07]	URI [1.05]	ASD [1.31]	Vit A [1.42]	PEM [510.96]	Urticaria [0.96]	Diarrhea [0.48]	Hernia [0.67]	Endocrine [2.5]
Aomori	Neonatal [1.11]	Congenital [0.99]	URI [1.05]	ASD [1.31]	Vit A [1.56]	Urticaria [0.95]	Diarrhea [0.47]	Hernia [0.68]	PEM [325.05]	Endocrine [2.53]
Chiba	Neonatal [1.14]	Congenital [1.07]	URI [1.06]	ASD [1.36]	Vit A [1.71]	Urticaria [1.06]	Hernia [0.62]	Iron [2.15]	Diarrhea [0.48]	Endocrine [2.73]
Ehime	Neonatal [0.9]	Congenital [0.99]	URI [1.06]	ASD [1.34]	Vit A [1.36]	Urticaria [0.99]	PEM [560.25]	Hernia [0.65]	Diarrhea [0.45]	Endocrine [2.34]
Fukui	Neonatal [1.12]	Congenital [1.02]	URI [1.05]	ASD [1.35]	Vit A [2.27]	Iron [2.99]	PEM [916.53]	Urticaria [1.03]	Diarrhea [0.54]	Hernia [0.62]
Fukuoka	Neonatal [1.12]	Congenital [1.01]	URI [1.06]	ASD [0.99]	PEM [1118.32]	Vit A [1.75]	Urticaria [1.04]	Hernia [0.62]	Diarrhea [0.52]	Endocrine [2.66]
Fukushima	Neonatal [1.0]	Congenital [1.21]	URI [1.05]	ASD [1.32]	Iron [2.49]	Urticaria [0.96]	Hernia [0.66]	Diarrhea [0.46]	Vit A [1.04]	PEM [356.49]
Gifu	Neonatal [1.16]	Congenital [0.92]	URI [1.06]	ASD [1.34]	Urticaria [1.02]	PEM [820.09]	Diarrhea [0.53]	Hernia [0.63]	Vit A [1.3]	Endocrine [2.75]
Gunma	Neonatal [1.08]	Congenital [1.06]	URI [1.06]	ASD [1.34]	Vit A [2.31]	Iron [2.31]	Urticaria [1.02]	Hernia [0.62]	Diarrhea [0.5]	Endocrine [3.0]
Hiroshima	Neonatal [1.21]	Congenital [1.0]	URI [1.06]	ASD [1.36]	Vit A [2.13]	Urticaria [1.08]	Hernia [0.6]	Diarrhea [0.51]	Iron [2.02]	PEM [963.23]
Hokkaido	Neonatal [1.07]	Congenital [1.02]	Iron [3.54]	URI [1.05]	ASD [1.32]	Urticaria [0.99]	Vit A [1.33]	Hernia [0.65]	Diarrhea [0.48]	PEM [451.28]
Hyogo	Neonatal [1.16]	Congenital [0.88]	URI [1.06]	ASD [1.35]	PEM [1310.21]	Urticaria [1.06]	Vit A [1.56]	Hernia [0.61]	Diarrhea [0.5]	Endocrine [2.49]
Ibaraki	Neonatal [1.19]	Congenital [1.18]	URI [1.06]	ASD [1.08]	Vit A [1.57]	Urticaria [1.02]	Iron [2.21]	Hernia [0.62]	Diarrhea [0.43]	Endocrine [2.7]
Ishikawa	Neonatal [1.1]	Congenital [0.99]	URI [1.06]	ASD [1.34]	Vit A [2.1]	Urticaria [1.04]	Diarrhea [0.55]	Hernia [0.62]	PEM [830.61]	Endocrine [2.49]
Iwate	Neonatal [1.15]	Congenital [1.19]	URI [1.05]	ASD [1.31]	Vit A [1.76]	PEM [444.01]	Iron [1.81]	Urticaria [0.95]	Diarrhea [0.47]	Hernia [0.68]

eFigure 9b. Leading ten causes of YLDs with the ratio of observed YLDs to YLDs expected on the basis of Socio-Demographic Index alone in 2017, <1 years, both sexes combined.
The top ten causes contributing to YLDs are listed globally, by socio-demographic quintile, and then by GBD superregion, region, country, and subnationally where modeled. For each cell, the ratio of observed YLDs to YLDs expected on the basis of socio-demographic index (SDI) alone are listed. Abbreviations: YLD=year of life lived with disability, GBD=Global Burden of Disease.

Values shown in brackets represent the ratio of observed YLDs to predicted YLDs on the basis of Socio-Demographic Index (SDI), rounded to two (2) digits. Color ranges (shown below) were calculated to place a roughly equal number of cells into each bin.

COLOR KEY:	[0.0-0.68]	(0.68-0.82]	(0.82-0.91]	(0.91-1.01]	(1.01-1.1]	(1.1-1.21]	(1.21-1.45]	(1.45-2.25]	2.25+

	1	2	3	4	5	6	7	8	9	10
Kagawa	Neonatal [1.07]	Congenital [0.99]	URI [1.06]	ASD [1.34]	Vit A [2.18]	PEM [909.59]	Urticaria [1.03]	Diarrhea [0.55]	Hernia [0.62]	Endocrine [2.52]
Kagoshima	Neonatal [0.93]	Congenital [1.17]	URI [1.05]	ASD [1.32]	Vit A [1.68]	Urticaria [0.97]	Hernia [0.68]	PEM [406.36]	Diarrhea [0.46]	Iron [1.5]
Kanagawa	Neonatal [1.24]	Congenital [1.14]	ASD [1.65]	URI [1.07]	Urticaria [1.12]	PEM [1641.01]	Hernia [0.59]	Vit A [1.68]	Diarrhea [0.47]	Endocrine [2.63]
Kochi	Neonatal [0.99]	Congenital [0.96]	URI [1.05]	ASD [1.32]	Vit A [1.32]	Iron [1.7]	Urticaria [0.95]	Hernia [0.7]	Diarrhea [0.46]	PEM [329.34]
Kumamoto	Neonatal [0.98]	Congenital [0.96]	URI [1.05]	ASD [1.32]	Vit A [1.79]	Iron [2.05]	Urticaria [0.96]	Diarrhea [0.49]	PEM [419.28]	Hernia [0.67]
Kyoto	Neonatal [1.24]	Congenital [1.27]	URI [1.07]	ASD [1.38]	PEM [1878.54]	Urticaria [1.12]	Iron [2.8]	Hernia [0.58]	Diarrhea [0.55]	Vit A [1.62]
Mie	Neonatal [1.18]	Congenital [1.02]	URI [1.06]	ASD [1.34]	Vit A [1.6]	Urticaria [1.04]	PEM [960.41]	Diarrhea [0.56]	Hernia [0.6]	Iron [2.09]
Miyagi	Neonatal [1.07]	Congenital [1.15]	URI [1.06]	ASD [1.34]	Vit A [2.01]	Urticaria [1.02]	Hernia [0.63]	Iron [1.93]	Diarrhea [0.49]	PEM [598.38]
Miyazaki	Neonatal [0.97]	Congenital [1.01]	URI [1.05]	ASD [1.32]	Iron [1.73]	Urticaria [0.95]	PEM [365.64]	Hernia [0.69]	Vit A [0.91]	Diarrhea [0.4]
Nagano	Neonatal [1.19]	Congenital [0.97]	URI [1.06]	ASD [1.34]	Vit A [2.14]	Iron [2.8]	PEM [917.24]	Urticaria [1.02]	Hernia [0.62]	Diarrhea [0.51]
Nagasaki	Neonatal [0.97]	Congenital [1.22]	Vit A [2.26]	URI [1.05]	ASD [1.31]	Urticaria [0.95]	Hernia [0.69]	Iron [1.44]	Diarrhea [0.44]	PEM [285.74]
Nara	Neonatal [1.0]	Congenital [1.29]	URI [1.06]	Vit A [2.68]	ASD [1.34]	PEM [881.24]	Urticaria [1.02]	Hernia [0.65]	Diarrhea [0.49]	Endocrine [2.5]
Niigata	Neonatal [1.13]	Congenital [1.05]	URI [1.05]	ASD [1.33]	PEM [819.75]	Vit A [1.63]	Urticaria [1.01]	Hernia [0.63]	Diarrhea [0.46]	Iron [1.66]
Oita	Neonatal [1.19]	Congenital [0.92]	URI [1.06]	Vit A [2.49]	ASD [1.34]	PEM [854.75]	Urticaria [1.01]	Diarrhea [0.54]	Hernia [0.63]	Iron [1.77]
Okayama	Neonatal [1.22]	Congenital [1.04]	URI [1.06]	ASD [1.34]	Vit A [1.75]	Urticaria [1.04]	Hernia [0.61]	Diarrhea [0.49]	Endocrine [2.47]	Falls [0.86]
Okinawa	Neonatal [1.01]	Congenital [0.93]	Vit A [2.03]	URI [1.05]	ASD [1.31]	PEM [399.73]	Urticaria [0.94]	Hernia [0.71]	Diarrhea [0.43]	Iron [1.06]
Osaka	Neonatal [1.1]	Congenital [1.09]	URI [1.06]	ASD [1.01]	Vit A [2.08]	Urticaria [1.09]	Hernia [0.58]	PEM [1319.04]	Diarrhea [0.49]	Iron [2.13]
Saga	Neonatal [1.03]	Congenital [0.94]	Vit A [2.52]	URI [1.05]	ASD [1.31]	Urticaria [0.98]	Hernia [0.66]	Iron [1.7]	Diarrhea [0.46]	PEM [413.13]
Saitama	Neonatal [1.1]	Congenital [1.01]	URI [1.06]	ASD [1.35]	Urticaria [1.03]	Vit A [1.5]	Hernia [0.65]	Diarrhea [0.46]	PEM [596.33]	Iron [1.43]
Shiga	Neonatal [1.09]	Congenital [1.1]	URI [1.07]	ASD [1.38]	Vit A [2.6]	Urticaria [1.09]	Iron [2.67]	Hernia [0.58]	Diarrhea [0.51]	PEM [1003.92]
Shimane	Neonatal [1.0]	Congenital [1.02]	URI [1.05]	Iron [3.03]	ASD [1.32]	Urticaria [0.96]	PEM [438.88]	Hernia [0.67]	Vit A [1.1]	Diarrhea [0.45]
Shizuoka	Neonatal [1.27]	Congenital [0.97]	Iron [5.34]	URI [1.06]	ASD [1.36]	Vit A [1.75]	Urticaria [1.06]	PEM [1039.59]	Hernia [0.6]	Diarrhea [0.49]
Tochigi	Neonatal [1.21]	Congenital [1.31]	Iron [4.45]	URI [1.06]	ASD [1.34]	Urticaria [1.04]	Hernia [0.61]	Diarrhea [0.46]	Endocrine [2.84]	Vit A [1.0]
Tokushima	Neonatal [1.13]	Congenital [0.99]	Vit A [2.74]	URI [1.05]	ASD [1.33]	PEM [904.8]	Urticaria [1.01]	Iron [2.09]	Diarrhea [0.5]	Hernia [0.63]
Tokyo	Neonatal [1.32]	Congenital [1.31]	URI [1.11]	ASD [1.48]	Urticaria [1.34]	Hernia [0.49]	Vit A [2.71]	Diarrhea [0.59]	PEM [4861.74]	Other MN [4.25]
Tottori	Neonatal [1.13]	Congenital [1.09]	URI [1.05]	ASD [1.33]	Vit A [1.67]	Iron [2.21]	PEM [576.67]	Urticaria [0.98]	Hernia [0.67]	Diarrhea [0.47]
Toyama	Neonatal [1.1]	Congenital [1.06]	URI [1.06]	ASD [1.36]	Vit A [1.74]	Urticaria [1.06]	PEM [1036.11]	Hernia [0.6]	Diarrhea [0.46]	Other MN [5.18]
Wakayama	Neonatal [1.16]	Congenital [0.89]	Vit A [2.83]	URI [1.05]	ASD [1.33]	PEM [659.98]	Urticaria [0.99]	Hernia [0.65]	Iron [1.71]	Diarrhea [0.46]
Yamagata	Neonatal [0.92]	Congenital [0.93]	Vit A [2.82]	URI [1.05]	ASD [1.31]	Urticaria [0.97]	PEM [429.44]	Diarrhea [0.47]	Hernia [0.67]	Endocrine [2.39]
Yamaguchi	Neonatal [0.98]	Congenital [0.93]	URI [1.05]	Iron [3.86]	ASD [1.34]	Vit A [1.83]	PEM [867.18]	Urticaria [1.02]	Hernia [0.62]	Diarrhea [0.49]
Yamanashi	Neonatal [1.28]	Congenital [1.21]	URI [1.06]	ASD [1.34]	Vit A [2.24]	Urticaria [1.04]	Hernia [0.61]	Diarrhea [0.48]	PEM [678.32]	Falls [0.87]

eFigure 9b. Leading ten causes of YLDs with the ratio of observed YLDs to YLDs expected on the basis of Socio-Demographic Index alone in 2017, <1 years, both sexes combined.
The top ten causes contributing to YLDs are listed globally, by socio-demographic quintile, and then by GBD superregion, region, country, and subnationally where modeled. For each cell, the ratio of observed YLDs to YLDs expected on the basis of socio-demographic index (SDI) alone are listed. Abbreviations: YLD=year of life lived with disability, GBD=Global Burden of Disease.

Values shown in brackets represent the ratio of observed YLDs to predicted YLDs on the basis of Socio-Demographic Index (SDI), rounded to two (2) digits. Color ranges (shown below) were calculated to place a roughly equal number of cells into each bin.

COLOR KEY:	[0.0-0.68]	(0.68-0.82]	(0.82-0.91]	(0.91-1.01]	(1.01-1.1]	(1.1-1.21]	(1.21-1.45]	(1.45-2.25]	2.25+

	1	2	3	4	5	6	7	8	9	10
S Korea	Neonatal [1.23]	Congenital [0.84]	Iron [6.39]	URI [1.04]	ASD [1.12]	Diarrhea [0.65]	Urticaria [1.08]	Vit A [1.64]	Hernia [0.54]	Falls [1.07]
Singapore	Neonatal [1.21]	Iron [9.47]	Congenital [0.7]	URI [1.03]	ASD [1.21]	Urticaria [1.07]	Diarrhea [0.56]	Hernia [0.53]	Falls [1.06]	Vit A [1.1]
**High-income North America**	Neonatal [1.0]	Congenital [1.0]	URI [1.22]	ASD [1.28]	Diarrhea [0.69]	Urticaria [1.12]	Iron [1.98]	Epilepsy [1.17]	Oth Neuro [1.66]	ID [2.18]
Canada	Neonatal [0.7]	Congenital [0.93]	URI [1.17]	ASD [1.58]	Diarrhea [0.84]	Urticaria [1.16]	Vit A [0.94]	Epilepsy [0.79]	Other MN [3.0]	Whooping [1.89]
Greenland	Neonatal [0.67]	Congenital [0.88]	URI [1.19]	ASD [1.27]	Diarrhea [0.56]	Iron [0.69]	Urticaria [0.86]	Vit A [0.37]	Epilepsy [0.89]	Falls [0.96]
USA	Neonatal [1.02]	Congenital [1.0]	URI [1.22]	ASD [1.25]	Diarrhea [0.66]	Urticaria [1.11]	Iron [1.98]	Epilepsy [1.19]	Oth Neuro [1.68]	ID [2.14]
Alabama	Neonatal [0.96]	Congenital [0.78]	URI [1.21]	Diarrhea [0.69]	ASD [0.78]	Urticaria [0.9]	Iron [1.47]	Epilepsy [1.08]	Vit A [0.51]	ID [2.63]
Alaska	Neonatal [0.9]	Congenital [1.13]	URI [1.22]	ASD [1.17]	Diarrhea [0.65]	Urticaria [0.96]	Epilepsy [0.83]	Iron [1.13]	Falls [0.49]	Whooping [1.71]
Arizona	Neonatal [0.95]	Congenital [0.96]	URI [1.21]	ASD [1.26]	Diarrhea [0.61]	Urticaria [1.05]	Iron [2.1]	Epilepsy [0.99]	Vit A [0.43]	Whooping [1.6]
Arkansas	Neonatal [0.84]	Congenital [0.8]	URI [1.2]	ASD [1.22]	Diarrhea [0.56]	Urticaria [0.83]	Epilepsy [0.96]	Iron [0.89]	ID [2.01]	Oth Neuro [1.37]
California	Neonatal [0.9]	Congenital [0.93]	URI [1.22]	ASD [1.45]	Urticaria [1.11]	Diarrhea [0.58]	Epilepsy [1.22]	Iron [1.9]	Oth Neuro [1.72]	Med Treat [6.44]
Colorado	Neonatal [1.02]	Congenital [1.04]	URI [1.23]	ASD [1.11]	Diarrhea [0.66]	Urticaria [1.13]	Epilepsy [1.13]	Iron [1.91]	Falls [0.45]	Oth Neuro [1.59]
Connecticut	Neonatal [1.27]	Congenital [1.05]	URI [1.25]	ASD [1.24]	Diarrhea [0.87]	Urticaria [1.41]	Epilepsy [1.43]	Oth Neuro [2.02]	Iron [1.28]	Oth Skin [1.24]
Delaware	Neonatal [1.07]	Congenital [1.06]	URI [1.22]	ASD [1.19]	Diarrhea [0.85]	Urticaria [1.19]	Epilepsy [1.54]	Iron [1.51]	Oth Neuro [2.17]	ID [2.3]
DC	Neonatal [1.03]	Congenital [1.11]	URI [1.23]	Diarrhea [0.94]	ASD [1.19]	Urticaria [1.18]	Epilepsy [0.99]	Iron [1.28]	Oth Skin [1.27]	Falls [0.37]
Florida	Neonatal [1.11]	Congenital [1.0]	URI [1.22]	Diarrhea [0.7]	Iron [2.91]	Urticaria [1.16]	ASD [0.86]	Epilepsy [1.19]	Oth Neuro [1.69]	ID [2.43]
Georgia	Neonatal [0.95]	Congenital [0.88]	URI [1.21]	ASD [1.17]	Diarrhea [0.63]	Urticaria [1.04]	Epilepsy [1.24]	Iron [0.92]	Oth Neuro [1.76]	Vit A [0.49]
Hawaii	Neonatal [0.91]	Congenital [0.96]	URI [1.22]	ASD [1.18]	Urticaria [1.32]	Diarrhea [0.58]	Iron [2.48]	Epilepsy [1.0]	Other MN [2.49]	ID [2.15]
Idaho	Neonatal [0.79]	Congenital [0.83]	URI [1.21]	ASD [1.14]	Diarrhea [0.55]	Urticaria [0.81]	Iron [1.56]	Epilepsy [0.87]	ID [2.32]	Whooping [1.52]
Illinois	Neonatal [1.18]	Congenital [1.1]	URI [1.22]	ASD [1.19]	Diarrhea [0.73]	Urticaria [1.2]	Epilepsy [1.24]	Iron [1.9]	Oth Neuro [1.76]	ID [2.41]
Indiana	Neonatal [0.97]	Congenital [1.07]	URI [1.21]	ASD [1.15]	Diarrhea [0.7]	Urticaria [0.96]	Epilepsy [1.22]	Iron [1.28]	Oth Neuro [1.73]	Med Treat [6.73]
Iowa	Neonatal [0.91]	Congenital [0.96]	URI [1.22]	ASD [1.18]	Urticaria [1.11]	Diarrhea [0.61]	Iron [1.44]	Epilepsy [0.79]	ID [2.32]	Whooping [1.6]
Kansas	Neonatal [1.02]	Congenital [0.91]	URI [1.22]	ASD [1.17]	Diarrhea [0.65]	Urticaria [1.07]	Iron [1.84]	Epilepsy [1.09]	ID [2.26]	Oth Neuro [1.55]
Kentucky	Neonatal [0.97]	Congenital [0.92]	URI [1.21]	ASD [1.13]	Diarrhea [0.57]	Urticaria [0.98]	Epilepsy [1.21]	Iron [1.17]	Oth Neuro [1.72]	Vit A [0.41]
Louisiana	Neonatal [0.91]	Congenital [0.93]	URI [1.21]	Urticaria [1.07]	Diarrhea [0.57]	ASD [0.79]	Epilepsy [1.19]	Iron [1.11]	Oth Neuro [1.69]	ID [2.15]
Maine	Neonatal [1.04]	Congenital [1.01]	URI [1.22]	Diarrhea [0.89]	ASD [1.18]	Urticaria [1.09]	Epilepsy [1.13]	Iron [1.29]	ID [2.48]	Oth Neuro [1.61]
Maryland	Neonatal [1.2]	Congenital [1.07]	URI [1.23]	ASD [1.33]	Diarrhea [0.84]	Urticaria [1.26]	Epilepsy [1.29]	Iron [1.92]	Oth Neuro [1.82]	Falls [0.4]
Massachusetts	Neonatal [1.26]	Congenital [1.17]	URI [1.26]	ASD [1.66]	Diarrhea [0.89]	Urticaria [1.48]	Epilepsy [1.51]	Med Treat [7.98]	Oth Neuro [2.13]	Oth Skin [1.42]
Michigan	Neonatal [1.02]	Congenital [1.14]	URI [1.22]	ASD [1.17]	Diarrhea [0.7]	Urticaria [1.16]	Iron [2.17]	Epilepsy [1.24]	Whooping [2.11]	Med Treat [7.71]
Minnesota	Neonatal [1.07]	Congenital [1.13]	URI [1.24]	ASD [1.42]	Urticaria [1.28]	Diarrhea [0.7]	Iron [3.24]	Epilepsy [1.08]	Med Treat [7.28]	Other MN [2.21]
Mississippi	Neonatal [0.87]	Congenital [0.75]	URI [1.2]	ASD [1.12]	Diarrhea [0.6]	Urticaria [0.85]	Epilepsy [0.91]	ID [2.29]	Oth Neuro [1.29]	Iron [0.43]

eFigure 9b. Leading ten causes of YLDs with the ratio of observed YLDs to YLDs expected on the basis of Socio-Demographic Index alone in 2017, <1 years, both sexes combined.
The top ten causes contributing to YLDs are listed globally, by socio-demographic quintile, and then by GBD superregion, region, country, and subnationally where modeled. For each cell, the ratio of observed YLDs to YLDs expected on the basis of socio-demographic index (SDI) alone are listed. Abbreviations: YLD=year of life lived with disability, GBD=Global Burden of Disease.

Values shown in brackets represent the ratio of observed YLDs to predicted YLDs on the basis of Socio-Demographic Index (SDI), rounded to two (2) digits. Color ranges (shown below) were calculated to place a roughly equal number of cells into each bin.

COLOR KEY:	[0.0-0.68]	(0.68-0.82]	(0.82-0.91]	(0.91-1.01]	(1.01-1.1]	(1.1-1.21]	(1.21-1.45]	(1.45-2.25]	2.25+

	1	2	3	4	5	6	7	8	9	10
Missouri	Neonatal [0.99]	Congenital [1.02]	URI [1.21]	ASD [1.22]	Diarrhea [0.68]	Urticaria [0.97]	Iron [1.95]	Epilepsy [1.08]	Vit A [0.54]	Oth Neuro [1.53]
Montana	Neonatal [1.0]	Congenital [0.86]	URI [1.22]	ASD [1.17]	Diarrhea [0.61]	Urticaria [0.87]	Iron [2.04]	Epilepsy [1.15]	Vit A [0.66]	ID [2.5]
Nebraska	Neonatal [0.93]	Congenital [1.0]	URI [1.22]	ASD [1.19]	Urticaria [1.18]	Diarrhea [0.61]	Iron [1.94]	Epilepsy [0.9]	ID [2.33]	Oth Skin [1.04]
Nevada	Neonatal [0.95]	Congenital [0.8]	URI [1.21]	ASD [1.15]	Diarrhea [0.59]	Urticaria [1.0]	Epilepsy [1.12]	Iron [1.46]	ID [2.4]	Oth Neuro [1.59]
New Hampshire	Neonatal [1.04]	Congenital [1.03]	URI [1.25]	ASD [1.24]	Diarrhea [0.96]	Urticaria [1.19]	Epilepsy [1.22]	Iron [2.52]	ID [2.62]	Oth Neuro [1.73]
New Jersey	Neonatal [1.17]	Congenital [1.27]	URI [1.24]	ASD [1.79]	Diarrhea [0.8]	Urticaria [1.33]	Epilepsy [1.54]	Iron [1.75]	Oth Neuro [2.17]	Oth Skin [1.29]
New Mexico	Neonatal [1.04]	Congenital [0.97]	URI [1.21]	ASD [1.13]	Diarrhea [0.6]	Urticaria [0.93]	Iron [1.46]	Epilepsy [1.19]	Oth Neuro [1.69]	ID [2.16]
New York	Neonatal [1.21]	Congenital [1.32]	URI [1.24]	Urticaria [1.28]	Diarrhea [0.72]	ASD [0.87]	Epilepsy [1.5]	Oth Neuro [2.11]	Iron [1.13]	Oth Skin [1.28]
N Carolina	Neonatal [0.96]	Congenital [0.99]	URI [1.21]	ASD [1.34]	Diarrhea [0.68]	Urticaria [1.11]	Iron [1.88]	Epilepsy [1.02]	Vit A [0.46]	Oth Neuro [1.45]
N Dakota	Neonatal [1.04]	Congenital [1.33]	URI [1.23]	ASD [1.19]	Diarrhea [0.63]	Urticaria [0.99]	Iron [2.52]	Epilepsy [0.97]	Other MN [2.1]	Oth Skin [1.08]
Ohio	Neonatal [1.0]	Congenital [1.12]	URI [1.22]	ASD [1.16]	Diarrhea [0.74]	Urticaria [1.09]	Epilepsy [1.33]	Iron [1.85]	Oth Neuro [1.88]	Oth Skin [1.09]
Oklahoma	Neonatal [0.92]	Congenital [0.93]	URI [1.21]	ASD [1.14]	Diarrhea [0.64]	Urticaria [0.93]	Epilepsy [1.16]	Iron [1.29]	Oth Neuro [1.65]	Med Treat [6.97]
Oregon	Neonatal [0.88]	Congenital [0.93]	URI [1.22]	Iron [4.36]	ASD [1.18]	Diarrhea [0.64]	Urticaria [1.07]	Epilepsy [1.08]	Vit A [0.75]	Med Treat [7.42]
Pennsylvania	Neonatal [1.06]	Congenital [1.18]	URI [1.23]	ASD [1.08]	Diarrhea [0.77]	Iron [3.36]	Urticaria [1.14]	Epilepsy [1.37]	Oth Neuro [1.93]	Vit A [0.64]
Rhode Island	Neonatal [1.02]	Congenital [1.01]	URI [1.23]	Diarrhea [0.95]	ASD [1.21]	Urticaria [1.24]	Epilepsy [1.32]	Iron [2.18]	Oth Neuro [1.86]	Oth Skin [1.17]
S Carolina	Neonatal [0.91]	Congenital [0.93]	URI [1.21]	ASD [0.98]	Diarrhea [0.63]	Urticaria [1.13]	Epilepsy [1.01]	Iron [1.25]	ID [2.54]	Oth Skin [1.23]
S Dakota	Neonatal [0.85]	Congenital [0.98]	URI [1.22]	ASD [1.16]	Diarrhea [0.63]	Urticaria [0.93]	Iron [1.8]	Epilepsy [0.97]	ID [2.23]	Vit A [0.45]
Tennessee	Neonatal [0.91]	Congenital [0.83]	URI [1.2]	ASD [1.48]	Diarrhea [0.62]	Urticaria [0.99]	Iron [1.42]	Epilepsy [1.03]	Vit A [0.47]	Oth Neuro [1.46]
Texas	Neonatal [1.04]	Congenital [0.86]	URI [1.21]	ASD [1.6]	Iron [2.45]	Urticaria [1.02]	Diarrhea [0.53]	Epilepsy [1.12]	Oth Neuro [1.58]	Med Treat [6.86]
Utah	Neonatal [0.88]	Congenital [0.87]	ASD [1.92]	URI [1.22]	Diarrhea [0.57]	Iron [2.21]	Urticaria [0.94]	Epilepsy [0.87]	Med Treat [7.53]	Other MN [2.82]
Vermont	Neonatal [1.08]	Congenital [0.98]	URI [1.24]	ASD [1.22]	Diarrhea [0.88]	Iron [3.23]	Urticaria [1.02]	Epilepsy [1.0]	ID [2.45]	Falls [0.37]
Virginia	Neonatal [1.14]	Congenital [0.9]	URI [1.23]	ASD [1.2]	Diarrhea [0.79]	Urticaria [1.26]	Epilepsy [1.32]	Iron [1.93]	Med Treat [7.4]	Oth Neuro [1.87]
Washington	Neonatal [0.96]	Congenital [1.01]	URI [1.23]	ASD [1.2]	Urticaria [1.25]	Diarrhea [0.69]	Iron [2.39]	Epilepsy [1.08]	Oth Skin [1.21]	Falls [0.4]
W Virginia	Neonatal [0.97]	Congenital [0.84]	URI [1.2]	Diarrhea [0.66]	ASD [0.85]	Urticaria [0.9]	Iron [1.51]	Epilepsy [1.29]	Oth Neuro [1.83]	ID [2.3]
Wisconsin	Neonatal [1.06]	Congenital [1.07]	URI [1.23]	ASD [1.07]	Diarrhea [0.64]	Urticaria [1.13]	Iron [2.62]	Epilepsy [1.15]	Blindness [1.28]	Falls [0.44]
Wyoming	Neonatal [0.86]	Congenital [1.01]	URI [1.22]	ASD [1.17]	Iron [3.71]	Diarrhea [0.63]	Urticaria [0.87]	Epilepsy [1.09]	Whooping [2.06]	ID [2.28]
**Southern Latin America**	Neonatal [0.93]	Diarrhea [2.35]	Congenital [0.78]	Iron [1.41]	Vit A [0.66]	Hernia [1.99]	URI [0.9]	ASD [1.08]	PEM [25.19]	Urticaria [0.78]
Argentina	Diarrhea [2.36]	Neonatal [0.88]	Congenital [0.79]	Iron [1.42]	Hernia [2.1]	Vit A [0.57]	PEM [27.55]	URI [0.9]	ASD [1.08]	Urticaria [0.77]
Chile	Neonatal [1.07]	Diarrhea [2.41]	Congenital [0.77]	Vit A [0.97]	Iron [1.25]	Hernia [1.69]	URI [0.89]	ASD [1.07]	Epilepsy [1.43]	Urticaria [0.81]
Uruguay	Neonatal [0.96]	Diarrhea [1.72]	Iron [1.67]	Congenital [0.76]	Vit A [0.71]	Hernia [2.16]	URI [0.91]	ASD [1.09]	Urticaria [0.77]	Epilepsy [0.83]
**Western Europe**	Neonatal [0.91]	Congenital [1.01]	Diarrhea [1.17]	URI [1.19]	Hernia [1.11]	ASD [1.04]	Epilepsy [1.08]	Urticaria [0.66]	Iron [1.33]	Falls [0.84]

eFigure 9b. Leading ten causes of YLDs with the ratio of observed YLDs to YLDs expected on the basis of Socio-Demographic Index alone in 2017, <1 years, both sexes combined.
The top ten causes contributing to YLDs are listed globally, by socio-demographic quintile, and then by GBD superregion, region, country, and subnationally where modeled. For each cell, the ratio of observed YLDs to YLDs expected on the basis of socio-demographic index (SDI) alone are listed. Abbreviations: YLD=year of life lived with disability, GBD=Global Burden of Disease.

Values shown in brackets represent the ratio of observed YLDs to predicted YLDs on the basis of Socio-Demographic Index (SDI), rounded to two (2) digits. Color ranges (shown below) were calculated to place a roughly equal number of cells into each bin.

COLOR KEY:	[0.0-0.68]	(0.68-0.82]	(0.82-0.91]	(0.91-1.01]	(1.01-1.1]	(1.1-1.21]	(1.21-1.45]	(1.45-2.25]	2.25+

	1	2	3	4	5	6	7	8	9	10
Andorra	Neonatal [1.02]	Congenital [1.11]	URI [1.21]	Diarrhea [1.26]	Hernia [0.91]	ASD [1.09]	Urticaria [0.79]	Epilepsy [1.06]	Falls [0.72]	Otitis [1.3]
Austria	Neonatal [1.43]	Congenital [1.05]	Hernia [1.99]	Diarrhea [1.75]	URI [1.19]	ASD [1.04]	Urticaria [0.7]	Falls [0.86]	Epilepsy [0.89]	Vit A [0.86]
Belgium	Congenital [1.08]	Neonatal [0.71]	Diarrhea [1.4]	URI [1.19]	Hernia [1.24]	ASD [1.06]	Epilepsy [1.36]	Urticaria [0.75]	Falls [0.85]	Otitis [1.3]
Cyprus	Neonatal [1.15]	Congenital [1.04]	Hernia [2.11]	Diarrhea [1.33]	URI [1.19]	ASD [1.04]	Urticaria [0.7]	Falls [0.79]	Epilepsy [0.86]	Vit A [0.85]
Denmark	Congenital [1.23]	Neonatal [0.84]	Diarrhea [2.3]	URI [1.22]	ASD [1.3]	Hernia [0.56]	Urticaria [0.91]	Iron [2.73]	Falls [0.68]	Epilepsy [1.04]
Finland	Neonatal [1.93]	Congenital [1.16]	Diarrhea [1.89]	URI [1.2]	Hernia [0.93]	ASD [1.09]	Urticaria [0.77]	Otitis [1.83]	Falls [0.82]	Epilepsy [1.07]
France	Neonatal [0.76]	Congenital [1.02]	URI [1.18]	Hernia [1.07]	Diarrhea [0.89]	ASD [0.97]	Urticaria [0.73]	Epilepsy [1.02]	Falls [0.86]	Otitis [1.28]
Germany	Neonatal [1.15]	Congenital [0.99]	Diarrhea [1.88]	Hernia [1.56]	URI [1.18]	Epilepsy [2.3]	ASD [1.05]	Urticaria [0.72]	Oth Neuro [3.23]	Falls [0.83]
Greece	Neonatal [0.85]	Congenital [0.95]	URI [1.17]	Diarrhea [0.88]	Hernia [1.25]	ASD [1.01]	Iron [1.13]	Vit A [0.67]	Urticaria [0.59]	Otitis [1.57]
Iceland	Neonatal [1.03]	Congenital [1.01]	Diarrhea [1.41]	URI [1.2]	ASD [1.06]	Urticaria [0.8]	Hernia [0.31]	Falls [0.71]	Epilepsy [0.99]	Vit A [1.27]
Ireland	Neonatal [0.86]	Congenital [1.1]	URI [1.19]	Diarrhea [1.08]	Hernia [1.0]	ASD [1.13]	Falls [0.99]	Urticaria [0.73]	Vit A [1.26]	Epilepsy [1.04]
Israel	Neonatal [0.9]	Congenital [0.95]	URI [1.17]	Hernia [1.18]	Diarrhea [0.72]	ASD [0.94]	Blindness [3.55]	Vit A [0.95]	Urticaria [0.58]	Iron [0.78]
Italy	Congenital [0.98]	Neonatal [0.67]	URI [1.18]	Hernia [1.14]	Diarrhea [0.73]	ASD [1.03]	Vit A [1.59]	Urticaria [0.49]	Epilepsy [0.74]	Falls [0.73]
Luxembourg	Neonatal [1.36]	Congenital [1.14]	Hernia [1.67]	Diarrhea [1.72]	URI [1.22]	ASD [1.11]	Urticaria [0.84]	Epilepsy [1.26]	Falls [0.68]	Otitis [1.32]
Malta	Congenital [1.15]	Neonatal [0.72]	Hernia [1.97]	URI [1.17]	ASD [1.03]	Diarrhea [0.63]	Falls [1.24]	Urticaria [0.63]	Vit A [0.78]	Epilepsy [0.74]
Netherlands	Neonatal [1.04]	Congenital [1.12]	URI [1.21]	Hernia [0.9]	ASD [1.22]	Diarrhea [0.99]	Urticaria [0.82]	Vit A [1.55]	Falls [0.68]	Epilepsy [1.04]
Norway	Neonatal [1.23]	Congenital [1.43]	URI [1.25]	ASD [1.04]	Hernia [0.47]	Urticaria [0.82]	Falls [0.78]	Other MN [4.21]	Epilepsy [1.18]	Diarrhea [0.35]
Portugal	Neonatal [0.56]	Congenital [0.77]	URI [1.17]	Diarrhea [0.65]	Vit A [0.92]	ASD [1.0]	Hernia [1.06]	Iron [0.66]	Urticaria [0.54]	Otitis [1.23]
Spain	Neonatal [0.72]	Congenital [0.88]	URI [1.17]	Diarrhea [0.81]	Hernia [1.17]	ASD [1.01]	Iron [1.73]	ID [5.51]	Urticaria [0.6]	Falls [0.94]
Sweden	Congenital [1.32]	Neonatal [0.87]	Diarrhea [1.65]	URI [1.22]	ASD [1.17]	Hernia [0.51]	Urticaria [0.82]	Falls [0.77]	Iron [1.65]	Vit A [1.05]
Stockholm	Congenital [1.42]	Neonatal [0.87]	Diarrhea [1.83]	URI [1.25]	ASD [1.53]	Iron [5.72]	Vit A [2.6]	Hernia [0.46]	Urticaria [0.91]	Falls [0.68]
Sweden w/o Stockholm	Congenital [1.29]	Neonatal [0.87]	Diarrhea [1.59]	URI [1.22]	ASD [1.06]	Hernia [0.53]	Urticaria [0.79]	Falls [0.8]	Epilepsy [0.82]	Vit A [0.7]
Switzerland	Neonatal [0.91]	Congenital [1.08]	Diarrhea [2.28]	URI [1.2]	Hernia [1.09]	ASD [1.08]	Vit A [1.8]	Urticaria [0.76]	Iron [1.92]	Falls [0.6]
UK	Neonatal [1.01]	Congenital [1.04]	Diarrhea [1.27]	URI [1.2]	Iron [3.15]	ASD [1.1]	Hernia [0.73]	Urticaria [0.65]	Epilepsy [0.86]	Vit A [0.75]
England	Neonatal [1.04]	Congenital [1.03]	Diarrhea [1.33]	URI [1.21]	Iron [3.42]	ASD [1.12]	Hernia [0.65]	Urticaria [0.66]	Epilepsy [0.87]	Falls [0.87]
E Midlands	Neonatal [0.95]	Congenital [1.02]	Diarrhea [1.24]	Iron [3.68]	URI [1.2]	ASD [1.08]	Hernia [0.73]	Urticaria [0.62]	Epilepsy [0.8]	Falls [0.94]
Derby	Neonatal [0.99]	Congenital [1.05]	Iron [5.65]	Diarrhea [1.18]	URI [1.2]	ASD [1.09]	Hernia [0.73]	Urticaria [0.65]	Epilepsy [0.86]	Falls [0.91]
Derbyshire	Neonatal [0.86]	Congenital [1.0]	Diarrhea [1.18]	URI [1.2]	Iron [2.58]	ASD [1.08]	Hernia [0.82]	Urticaria [0.6]	Vit A [0.55]	Falls [1.0]
Leicester	Neonatal [0.85]	Congenital [1.05]	Diarrhea [1.25]	URI [1.2]	Iron [3.39]	ASD [1.09]	Hernia [0.65]	Urticaria [0.64]	Vit A [0.79]	Epilepsy [0.84]
Leicestershire	Neonatal [0.84]	Congenital [1.04]	Diarrhea [1.24]	URI [1.21]	Iron [4.17]	ASD [1.1]	Hernia [0.7]	Urticaria [0.66]	Epilepsy [0.85]	Falls [0.9]
Lincolnshire	Neonatal [0.9]	Congenital [1.0]	Iron [3.78]	Diarrhea [1.18]	URI [1.19]	ASD [1.08]	Hernia [0.76]	Urticaria [0.59]	Epilepsy [0.75]	Falls [1.0]

eFigure 9b. Leading ten causes of YLDs with the ratio of observed YLDs to YLDs expected on the basis of Socio-Demographic Index alone in 2017, <1 years, both sexes combined.
The top ten causes contributing to YLDs are listed globally, by socio-demographic quintile, and then by GBD superregion, region, country, and subnationally where modeled. For each cell, the ratio of observed YLDs to YLDs expected on the basis of socio-demographic index (SDI) alone are listed. Abbreviations: YLD=year of life lived with disability, GBD=Global Burden of Disease.

Values shown in brackets represent the ratio of observed YLDs to predicted YLDs on the basis of Socio-Demographic Index (SDI), rounded to two (2) digits. Color ranges (shown below) were calculated to place a roughly equal number of cells into each bin.

COLOR KEY:	[0.0-0.68]	(0.68-0.82]	(0.82-0.91]	(0.91-1.01]	(1.01-1.1]	(1.1-1.21]	(1.21-1.45]	(1.45-2.25]	2.25+

	1	2	3	4	5	6	7	8	9	10
Northamptonshire	Neonatal [1.04]	Congenital [1.02]	Diarrhea [1.38]	URI [1.2]	Iron [3.34]	ASD [1.08]	Hernia [0.71]	Urticaria [0.63]	Epilepsy [0.8]	Falls [0.94]
Nottingham	Neonatal [1.21]	Congenital [1.09]	Diarrhea [1.3]	URI [1.22]	Iron [3.71]	ASD [1.12]	Hernia [0.66]	Urticaria [0.7]	Epilepsy [0.98]	Falls [0.83]
Nottinghamshire	Neonatal [1.0]	Congenital [1.0]	Diarrhea [1.18]	Iron [3.37]	URI [1.2]	ASD [1.08]	Hernia [0.8]	Urticaria [0.6]	Falls [1.0]	Epilepsy [0.74]
Rutland	Neonatal [0.71]	Congenital [1.02]	URI [1.2]	Iron [3.64]	Diarrhea [1.03]	ASD [1.09]	Urticaria [0.63]	Hernia [0.43]	Epilepsy [0.8]	Falls [0.91]
E England	Neonatal [0.93]	Congenital [0.97]	Diarrhea [1.24]	URI [1.2]	Iron [3.51]	ASD [1.15]	Hernia [0.56]	Urticaria [0.64]	Epilepsy [0.81]	Falls [0.9]
Bedford	Neonatal [1.12]	Congenital [0.99]	Diarrhea [1.21]	URI [1.2]	Iron [3.29]	ASD [1.09]	Urticaria [0.65]	Hernia [0.45]	Vit A [0.75]	Epilepsy [0.83]
Cambridgeshire	Neonatal [0.99]	Congenital [1.03]	Diarrhea [1.43]	URI [1.22]	ASD [1.69]	Iron [2.75]	Urticaria [0.71]	Hernia [0.41]	Falls [0.81]	Epilepsy [0.91]
Cen Bedfordshire	Neonatal [1.0]	Congenital [0.95]	Diarrhea [1.28]	URI [1.2]	Iron [3.29]	ASD [1.09]	Hernia [0.57]	Urticaria [0.63]	Epilepsy [0.79]	Falls [0.91]
Essex	Neonatal [0.9]	Congenital [0.96]	Iron [3.86]	Diarrhea [1.14]	URI [1.2]	ASD [1.09]	Hernia [0.54]	Urticaria [0.62]	Epilepsy [0.78]	Falls [0.93]
Hertfordshire	Neonatal [0.98]	Congenital [1.01]	Diarrhea [1.34]	URI [1.22]	Iron [4.07]	ASD [1.13]	Hernia [0.48]	Urticaria [0.71]	Epilepsy [0.93]	Falls [0.8]
Luton	Neonatal [1.05]	Congenital [0.98]	Diarrhea [1.37]	Iron [4.09]	URI [1.2]	ASD [1.08]	Hernia [0.68]	Urticaria [0.63]	Epilepsy [0.83]	Falls [0.95]
Norfolk	Neonatal [0.79]	Congenital [0.95]	Diarrhea [1.24]	URI [1.2]	Iron [2.99]	ASD [1.09]	Hernia [0.76]	Urticaria [0.62]	Falls [0.96]	Epilepsy [0.75]
Peterborough	Neonatal [0.76]	Congenital [0.95]	Diarrhea [1.2]	URI [1.2]	Iron [2.67]	ASD [1.07]	Hernia [0.8]	Urticaria [0.61]	Epilepsy [0.78]	Falls [0.98]
Southend-on-Sea	Neonatal [1.04]	Congenital [0.95]	Iron [3.58]	Diarrhea [0.98]	URI [1.2]	ASD [1.07]	Hernia [0.7]	Urticaria [0.59]	Vit A [0.59]	Epilepsy [0.72]
Suffolk	Neonatal [0.93]	Congenital [0.94]	Diarrhea [1.18]	URI [1.2]	Iron [2.61]	ASD [1.09]	Hernia [0.63]	Urticaria [0.61]	Falls [0.97]	Epilepsy [0.73]
Thurrock	Neonatal [0.97]	Congenital [0.93]	Iron [2.58]	Diarrhea [0.97]	URI [1.2]	ASD [1.07]	Urticaria [0.59]	Hernia [0.5]	Epilepsy [0.71]	Falls [1.04]
Greater London	Neonatal [1.17]	Congenital [1.1]	Diarrhea [1.44]	URI [1.23]	ASD [1.17]	Hernia [0.5]	Iron [2.79]	Urticaria [0.78]	Epilepsy [1.05]	Falls [0.73]
Barking & Dagenham	Neonatal [0.95]	Congenital [0.94]	Diarrhea [0.99]	URI [1.2]	ASD [1.07]	Iron [1.03]	Hernia [0.68]	Urticaria [0.58]	Epilepsy [0.74]	Falls [1.04]
Barnet	Neonatal [1.05]	Congenital [1.01]	Diarrhea [1.22]	URI [1.21]	ASD [1.13]	Hernia [0.56]	Iron [2.2]	Urticaria [0.7]	Epilepsy [0.91]	Falls [0.81]
Bexley	Neonatal [0.93]	Congenital [0.95]	Diarrhea [1.18]	URI [1.2]	ASD [1.15]	Iron [1.74]	Hernia [0.74]	Urticaria [0.61]	Falls [0.94]	Epilepsy [0.75]
Brent	Neonatal [1.07]	Congenital [0.99]	Diarrhea [1.17]	URI [1.21]	ASD [1.1]	Hernia [0.5]	Urticaria [0.67]	Iron [1.19]	Epilepsy [0.84]	Falls [0.84]
Bromley	Neonatal [1.12]	Congenital [0.99]	URI [1.21]	Diarrhea [1.11]	ASD [1.18]	Iron [2.49]	Hernia [0.58]	Urticaria [0.66]	Falls [0.86]	Epilepsy [0.83]
Camden	Neonatal [1.13]	Congenital [1.18]	URI [1.27]	Diarrhea [1.6]	ASD [1.22]	Hernia [0.4]	Iron [3.98]	Urticaria [0.89]	Epilepsy [1.21]	Falls [0.67]
Croydon	Neonatal [1.0]	Congenital [0.97]	Diarrhea [1.08]	URI [1.2]	ASD [1.09]	Iron [1.94]	Hernia [0.71]	Urticaria [0.63]	Epilepsy [0.79]	Falls [0.9]
Ealing	Neonatal [1.18]	Congenital [1.01]	Diarrhea [1.28]	URI [1.21]	ASD [1.11]	Hernia [0.56]	Iron [2.0]	Urticaria [0.7]	Epilepsy [0.91]	Falls [0.8]
Enfield	Neonatal [1.05]	Congenital [0.98]	Diarrhea [1.21]	URI [1.2]	ASD [1.1]	Hernia [0.63]	Urticaria [0.65]	Epilepsy [0.84]	Iron [1.05]	Falls [0.88]
Greenwich	Neonatal [0.98]	Congenital [0.99]	Diarrhea [1.24]	URI [1.2]	ASD [1.15]	Iron [1.88]	Hernia [0.73]	Urticaria [0.63]	Epilepsy [0.81]	Falls [0.9]
Hackney	Neonatal [1.16]	Congenital [1.07]	Diarrhea [1.43]	URI [1.23]	ASD [1.13]	Hernia [0.52]	Iron [2.21]	Urticaria [0.75]	Epilepsy [1.07]	Falls [0.78]
Hammersmith & Fulham	Neonatal [1.23]	Congenital [1.17]	Diarrhea [1.68]	URI [1.26]	ASD [1.2]	Iron [4.55]	Urticaria [0.87]	Hernia [0.37]	Epilepsy [1.21]	Falls [0.67]
Haringey	Neonatal [1.14]	Congenital [1.02]	Diarrhea [1.25]	URI [1.21]	ASD [1.1]	Hernia [0.59]	Iron [1.86]	Urticaria [0.68]	Epilepsy [0.89]	Falls [0.82]
Harrow	Neonatal [1.16]	Congenital [1.0]	Diarrhea [1.26]	URI [1.21]	ASD [1.11]	Hernia [0.6]	Iron [1.63]	Urticaria [0.66]	Other MN [4.79]	Epilepsy [0.85]

eFigure 9b. Leading ten causes of YLDs with the ratio of observed YLDs to YLDs expected on the basis of Socio-Demographic Index alone in 2017, <1 years, both sexes combined.
The top ten causes contributing to YLDs are listed globally, by socio-demographic quintile, and then by GBD superregion, region, country, and subnationally where modeled. For each cell, the ratio of observed YLDs to YLDs expected on the basis of socio-demographic index (SDI) alone are listed. Abbreviations: YLD=year of life lived with disability, GBD=Global Burden of Disease.

Values shown in brackets represent the ratio of observed YLDs to predicted YLDs on the basis of Socio-Demographic Index (SDI), rounded to two (2) digits. Color ranges (shown below) were calculated to place a roughly equal number of cells into each bin.

COLOR KEY:	[0.0-0.68]	[0.68-0.82]	[0.82-0.91]	[0.91-1.01]	[1.01-1.1]	[1.1-1.21]	[1.21-1.45]	[1.45-2.25]	2.25+

	1	2	3	4	5	6	7	8	9	10
Havering	Neonatal [0.96]	Congenital [0.95]	Diarrhea [1.09]	URI [1.2]	Iron [2.89]	ASD [1.09]	Hernia [0.65]	Urticaria [0.62]	Epilepsy [0.79]	Falls [0.95]
Hillingdon	Neonatal [1.25]	Congenital [1.05]	Diarrhea [1.47]	URI [1.22]	ASD [1.13]	Hernia [0.57]	Iron [2.37]	Urticaria [0.73]	Epilepsy [0.99]	Falls [0.76]
Hounslow	Neonatal [1.19]	Congenital [1.05]	Diarrhea [1.36]	URI [1.22]	ASD [1.14]	Hernia [0.52]	Iron [2.29]	Urticaria [0.74]	Epilepsy [0.99]	Falls [0.77]
Islington	Neonatal [1.08]	Congenital [1.16]	Diarrhea [1.57]	URI [1.26]	ASD [1.2]	Hernia [0.44]	Urticaria [0.86]	Epilepsy [1.26]	Falls [0.68]	Iron [2.26]
Kensington & Chelsea	Neonatal [1.08]	Congenital [1.18]	URI [1.27]	Diarrhea [1.53]	ASD [1.23]	Urticaria [0.89]	Hernia [0.32]	Other MN [3.71]	Epilepsy [1.2]	Falls [0.66]
Kingston upon Thames	Neonatal [1.22]	Congenital [1.08]	Diarrhea [1.44]	URI [1.23]	ASD [1.16]	Hernia [0.46]	Urticaria [0.76]	Epilepsy [1.04]	Other MN [4.26]	Iron [1.85]
Lambeth	Neonatal [0.9]	Congenital [1.13]	Diarrhea [1.42]	URI [1.24]	ASD [1.16]	Hernia [0.5]	Iron [3.16]	Urticaria [0.79]	Epilepsy [1.09]	Falls [0.72]
Lewisham	Neonatal [1.22]	Congenital [1.01]	URI [1.2]	Diarrhea [1.11]	ASD [1.17]	Hernia [0.64]	Iron [1.77]	Urticaria [0.65]	Epilepsy [0.85]	Falls [0.86]
Merton	Neonatal [1.12]	Congenital [1.05]	URI [1.22]	Diarrhea [1.26]	ASD [1.14]	Iron [2.69]	Hernia [0.57]	Urticaria [0.72]	Epilepsy [0.97]	Falls [0.78]
Newham	Neonatal [1.08]	Congenital [1.0]	Diarrhea [1.21]	URI [1.21]	ASD [1.09]	Hernia [0.51]	Urticaria [0.64]	Iron [1.17]	Epilepsy [0.83]	Falls [0.86]
Redbridge	Neonatal [0.98]	Congenital [1.01]	Diarrhea [1.12]	URI [1.2]	ASD [1.09]	Hernia [0.56]	Urticaria [0.62]	Epilepsy [0.79]	Falls [0.91]	Iron [0.83]
Richmond upon Thames	Neonatal [1.3]	Congenital [1.1]	URI [1.24]	Diarrhea [1.33]	ASD [1.18]	Hernia [0.44]	Urticaria [0.79]	Falls [0.72]	Epilepsy [1.01]	Iron [1.78]
Southwark	Neonatal [0.93]	Congenital [1.14]	Diarrhea [1.49]	URI [1.25]	ASD [1.25]	Hernia [0.51]	Urticaria [0.82]	Epilepsy [1.19]	Falls [0.71]	Otitis [1.4]
Sutton	Neonatal [1.29]	Congenital [0.99]	Diarrhea [1.18]	URI [1.2]	ASD [1.1]	Hernia [0.64]	Urticaria [0.65]	Iron [1.24]	Epilepsy [0.84]	Falls [0.87]
Tower Hamlets	Neonatal [1.3]	Congenital [1.11]	Diarrhea [1.5]	URI [1.25]	ASD [1.18]	Urticaria [0.81]	Iron [2.52]	Epilepsy [1.16]	Hernia [0.34]	Falls [0.73]
Waltham Forest	Neonatal [0.98]	Congenital [0.97]	Diarrhea [1.07]	URI [1.2]	ASD [1.08]	Iron [1.59]	Hernia [0.63]	Urticaria [0.61]	Epilepsy [0.76]	Falls [0.94]
Wandsworth	Neonatal [1.07]	Congenital [1.14]	URI [1.25]	Diarrhea [1.43]	ASD [1.19]	Hernia [0.43]	Urticaria [0.82]	Epilepsy [1.14]	Iron [2.31]	Falls [0.7]
Westminster	Neonatal [1.03]	Congenital [1.16]	Diarrhea [1.6]	URI [1.26]	ASD [1.21]	Urticaria [0.87]	Hernia [0.34]	Epilepsy [1.23]	Falls [0.68]	Other MN [3.52]
NE England	Neonatal [0.89]	Congenital [1.1]	Diarrhea [1.36]	URI [1.2]	Iron [2.83]	ASD [1.08]	Hernia [0.83]	Vit A [0.84]	Urticaria [0.61]	Epilepsy [0.77]
County Durham	Neonatal [0.93]	Congenital [1.09]	Diarrhea [1.34]	Iron [3.18]	URI [1.2]	ASD [1.07]	Hernia [0.9]	Vit A [0.79]	Urticaria [0.59]	Epilepsy [0.74]
Darlington	Neonatal [0.86]	Congenital [1.11]	Diarrhea [1.36]	URI [1.2]	Iron [2.21]	ASD [1.07]	Hernia [0.74]	Urticaria [0.62]	Vit A [0.62]	Epilepsy [0.8]
Gateshead	Neonatal [0.99]	Congenital [1.11]	Diarrhea [1.34]	URI [1.2]	Iron [3.06]	ASD [1.08]	Hernia [0.89]	Vit A [0.96]	Urticaria [0.62]	Epilepsy [0.81]
Hartlepool	Neonatal [0.95]	Congenital [1.08]	Diarrhea [1.14]	URI [1.2]	Iron [1.5]	ASD [1.07]	Hernia [0.86]	Vit A [0.57]	Urticaria [0.57]	Epilepsy [0.71]
Middlesbrough	Neonatal [0.79]	Congenital [1.09]	Diarrhea [1.37]	Iron [3.41]	URI [1.2]	ASD [1.07]	Hernia [0.79]	Vit A [0.77]	Urticaria [0.59]	Epilepsy [0.78]
Newcastle upon Tyne	Neonatal [0.95]	Congenital [1.19]	Diarrhea [1.66]	URI [1.22]	ASD [1.12]	Hernia [0.78]	Iron [3.34]	Vit A [1.28]	Urticaria [0.71]	Epilepsy [0.96]
N Tyneside	Neonatal [0.85]	Congenital [1.11]	Diarrhea [1.49]	URI [1.2]	Iron [2.46]	ASD [1.09]	Hernia [0.76]	Vit A [0.98]	Urticaria [0.61]	Epilepsy [0.79]
Northumberland	Neonatal [0.84]	Congenital [1.09]	Iron [3.95]	Diarrhea [1.27]	URI [1.2]	ASD [1.08]	Hernia [0.93]	Vit A [0.66]	Urticaria [0.59]	Epilepsy [0.74]
Redcar & Cleveland	Neonatal [0.77]	Congenital [1.07]	Diarrhea [1.23]	URI [1.2]	Iron [1.63]	ASD [1.08]	Hernia [0.85]	Vit A [0.61]	Urticaria [0.57]	Epilepsy [0.69]
S Tyneside	Neonatal [0.81]	Congenital [1.07]	Diarrhea [1.2]	URI [1.2]	Iron [1.81]	ASD [1.07]	Hernia [0.85]	Vit A [0.64]	Urticaria [0.57]	Epilepsy [0.69]
Stockton-on-Tees	Neonatal [1.06]	Congenital [1.11]	Diarrhea [1.35]	URI [1.2]	ASD [1.07]	Iron [2.15]	Hernia [0.76]	Vit A [1.08]	Urticaria [0.61]	Epilepsy [0.77]
Sunderland	Neonatal [0.77]	Congenital [1.09]	Diarrhea [1.36]	URI [1.2]	ASD [1.08]	Iron [1.82]	Hernia [0.82]	Vit A [0.74]	Urticaria [0.6]	Epilepsy [0.78]

eFigure 9b. Leading ten causes of YLDs with the ratio of observed YLDs to YLDs expected on the basis of Socio-Demographic Index alone in 2017, <1 years, both sexes combined.
The top ten causes contributing to YLDs are listed globally, by socio-demographic quintile, and then by GBD superregion, region, country, and subnationally where modeled. For each cell, the ratio of observed YLDs to YLDs expected on the basis of socio-demographic index (SDI) alone are listed. Abbreviations: YLD=year of life lived with disability, GBD=Global Burden of Disease.

Values shown in brackets represent the ratio of observed YLDs to predicted YLDs on the basis of Socio-Demographic Index (SDI), rounded to two (2) digits. Color ranges (shown below) were calculated to place a roughly equal number of cells into each bin.

COLOR KEY:	[0.0-0.68]	[0.68-0.82]	[0.82-0.91]	[0.91-1.01]	[1.01-1.1]	[1.1-1.21]	[1.21-1.45]	[1.45-2.25]	2.25+

	1	2	3	4	5	6	7	8	9	10
NW England	Neonatal [1.11]	Congenital [1.09]	Diarrhea [1.35]	URI [1.2]	Iron [3.03]	ASD [1.09]	Hernia [0.72]	Urticaria [0.63]	Epilepsy [0.82]	Falls [0.93]
Blackburn with Darwen	Neonatal [1.05]	Congenital [1.03]	Diarrhea [1.27]	URI [1.19]	Iron [2.29]	ASD [1.07]	Hernia [0.78]	Urticaria [0.58]	Vit A [0.54]	Epilepsy [0.75]
Blackpool	Neonatal [0.97]	Congenital [1.03]	Iron [2.11]	Diarrhea [0.98]	URI [1.2]	ASD [1.07]	Hernia [0.9]	Urticaria [0.56]	Vit A [0.39]	Epilepsy [0.72]
Bolton	Neonatal [1.0]	Congenital [1.03]	Diarrhea [1.16]	URI [1.2]	Iron [2.18]	ASD [1.07]	Hernia [0.81]	Urticaria [0.59]	Epilepsy [0.72]	Falls [1.02]
Bury	Neonatal [0.98]	Congenital [1.04]	Diarrhea [1.27]	URI [1.2]	Iron [2.38]	ASD [1.08]	Hernia [0.7]	Vit A [0.66]	Urticaria [0.6]	Epilepsy [0.74]
Cheshire E	Neonatal [1.42]	Congenital [1.18]	Diarrhea [1.53]	URI [1.21]	ASD [1.12]	Iron [3.36]	Hernia [0.74]	Urticaria [0.7]	Epilepsy [0.93]	Falls [0.82]
Cheshire W & Chester	Neonatal [1.15]	Congenital [1.16]	Diarrhea [1.47]	URI [1.21]	Iron [3.42]	ASD [1.11]	Hernia [0.78]	Urticaria [0.68]	Falls [0.85]	Vit A [0.79]
Cumbria	Neonatal [1.07]	Congenital [1.05]	Diarrhea [1.38]	URI [1.2]	Iron [2.95]	ASD [1.09]	Hernia [0.92]	Urticaria [0.62]	Vit A [0.69]	Epilepsy [0.78]
Halton	Neonatal [1.11]	Congenital [1.05]	Diarrhea [1.32]	URI [1.2]	Iron [2.57]	ASD [1.07]	Hernia [0.61]	Urticaria [0.62]	Epilepsy [0.81]	Falls [0.94]
Knowsley	Neonatal [1.05]	Congenital [1.1]	Diarrhea [1.2]	URI [1.2]	Iron [2.43]	ASD [1.07]	Hernia [0.7]	Urticaria [0.6]	Epilepsy [0.78]	Falls [0.99]
Lancashire	Neonatal [1.06]	Congenital [1.06]	Diarrhea [1.43]	URI [1.2]	Iron [2.54]	ASD [1.08]	Hernia [0.8]	Urticaria [0.62]	Epilepsy [0.8]	Falls [0.95]
Liverpool	Neonatal [1.23]	Congenital [1.17]	Iron [5.81]	Diarrhea [1.32]	URI [1.21]	ASD [1.1]	Hernia [0.67]	Vit A [0.98]	Urticaria [0.67]	Epilepsy [0.93]
Manchester	Neonatal [1.33]	Congenital [1.18]	Diarrhea [1.71]	URI [1.22]	ASD [1.13]	Iron [3.75]	Hernia [0.54]	Vit A [1.46]	Urticaria [0.75]	Falls [0.81]
Oldham	Neonatal [0.8]	Congenital [1.02]	Diarrhea [1.14]	URI [1.2]	Iron [1.87]	ASD [1.07]	Hernia [0.76]	Urticaria [0.57]	Vit A [0.41]	Epilepsy [0.71]
Rochdale	Neonatal [0.82]	Congenital [1.01]	Iron [2.75]	Diarrhea [1.15]	URI [1.2]	ASD [1.07]	Hernia [0.73]	Vit A [0.59]	Urticaria [0.57]	Epilepsy [0.73]
Salford	Neonatal [1.24]	Congenital [1.08]	Diarrhea [1.3]	URI [1.21]	ASD [1.09]	Iron [2.53]	Hernia [0.71]	Urticaria [0.64]	Epilepsy [0.87]	Falls [0.91]
Sefton	Neonatal [1.06]	Congenital [1.1]	Diarrhea [1.15]	URI [1.2]	Iron [2.58]	ASD [1.08]	Hernia [0.81]	Urticaria [0.59]	Epilepsy [0.74]	Falls [1.03]
St Helens	Neonatal [1.07]	Congenital [1.09]	Diarrhea [1.17]	URI [1.2]	ASD [1.07]	Iron [1.57]	Hernia [0.81]	Urticaria [0.59]	Epilepsy [0.75]	Falls [1.03]
Stockport	Neonatal [0.96]	Congenital [1.08]	Diarrhea [1.52]	URI [1.2]	Iron [3.42]	ASD [1.1]	Hernia [0.69]	Urticaria [0.65]	Falls [0.92]	Epilepsy [0.86]
Tameside	Neonatal [0.86]	Congenital [1.02]	Diarrhea [1.19]	URI [1.2]	Iron [1.49]	ASD [1.07]	Hernia [0.77]	Vit A [0.57]	Urticaria [0.58]	Epilepsy [0.71]
Trafford	Neonatal [1.29]	Congenital [1.13]	Diarrhea [1.5]	URI [1.22]	Iron [4.88]	ASD [1.13]	Hernia [0.51]	Urticaria [0.72]	Epilepsy [0.94]	Falls [0.79]
Warrington	Neonatal [1.34]	Congenital [1.11]	Diarrhea [1.48]	URI [1.21]	ASD [1.11]	Iron [3.27]	Hernia [0.59]	Urticaria [0.69]	Epilepsy [0.94]	Falls [0.83]
Wigan	Neonatal [1.05]	Congenital [1.02]	Diarrhea [1.09]	URI [1.2]	ASD [1.08]	Iron [1.38]	Hernia [0.8]	Urticaria [0.58]	Epilepsy [0.7]	Falls [1.06]
Wirral	Neonatal [1.18]	Congenital [1.1]	Diarrhea [1.21]	URI [1.2]	Iron [2.15]	ASD [1.07]	Hernia [0.89]	Urticaria [0.59]	Epilepsy [0.73]	Falls [1.04]
SE England	Neonatal [1.07]	Congenital [1.03]	Diarrhea [1.33]	URI [1.21]	ASD [1.19]	Iron [2.77]	Hernia [0.65]	Urticaria [0.67]	Epilepsy [0.94]	Falls [0.85]
Bracknell Forest	Neonatal [0.89]	Congenital [1.05]	URI [1.22]	Diarrhea [1.2]	ASD [1.13]	Iron [2.7]	Hernia [0.52]	Urticaria [0.71]	Vit A [0.99]	Falls [0.8]
Brighton & Hove	Neonatal [1.22]	Congenital [1.11]	Diarrhea [1.39]	URI [1.23]	ASD [1.14]	Hernia [0.5]	Iron [2.33]	Urticaria [0.75]	Epilepsy [1.0]	Falls [0.78]
Buckinghamshire	Neonatal [1.35]	Congenital [1.05]	Diarrhea [1.46]	URI [1.22]	Epilepsy [2.46]	ASD [1.12]	Hernia [0.66]	Iron [2.51]	Urticaria [0.7]	Oth Neuro [3.46]
E Sussex	Neonatal [1.04]	Congenital [0.97]	Diarrhea [1.18]	URI [1.2]	Iron [2.33]	ASD [1.15]	Hernia [0.77]	Urticaria [0.6]	Epilepsy [0.73]	Falls [0.99]
Hampshire	Neonatal [1.02]	Congenital [1.01]	Diarrhea [1.38]	URI [1.21]	ASD [1.11]	Iron [2.95]	Hernia [0.7]	Urticaria [0.67]	Falls [0.87]	Epilepsy [0.83]
Isle of Wight	Neonatal [0.93]	Congenital [0.96]	Diarrhea [1.08]	URI [1.2]	ASD [1.08]	Hernia [0.71]	Iron [1.26]	Urticaria [0.6]	Epilepsy [0.73]	Falls [0.99]

eFigure 9b. Leading ten causes of YLDs with the ratio of observed YLDs to YLDs expected on the basis of Socio-Demographic Index alone in 2017, <1 years, both sexes combined.
The top ten causes contributing to YLDs are listed globally, by socio-demographic quintile, and then by GBD superregion, region, country, and subnationally where modeled. For each cell, the ratio of observed YLDs to YLDs expected on the basis of socio-demographic index (SDI) alone are listed. Abbreviations: YLD=year of life lived with disability, GBD=Global Burden of Disease.

Values shown in brackets represent the ratio of observed YLDs to predicted YLDs on the basis of Socio-Demographic Index (SDI), rounded to two (2) digits. Color ranges (shown below) were calculated to place a roughly equal number of cells into each bin.

COLOR KEY:	[0.0-0.68]	[0.68-0.82]	[0.82-0.91]	[0.91-1.01]	[1.01-1.1]	[1.1-1.21]	[1.21-1.45]	[1.45-2.25]	2.25+

	1	2	3	4	5	6	7	8	9	10
Kent	Neonatal [0.98]	Congenital [0.99]	Diarrhea [1.22]	URI [1.2]	ASD [1.52]	Iron [2.46]	Hernia [0.72]	Urticaria [0.62]	Falls [0.93]	Epilepsy [0.77]
Medway	Neonatal [1.0]	Congenital [0.96]	Diarrhea [1.14]	URI [1.2]	Iron [2.25]	ASD [1.08]	Hernia [0.86]	Urticaria [0.59]	Vit A [0.57]	Epilepsy [0.76]
Milton Keynes	Neonatal [0.95]	Congenital [1.04]	Diarrhea [1.48]	URI [1.22]	ASD [1.11]	Iron [3.17]	Hernia [0.64]	Urticaria [0.68]	Falls [0.87]	Vit A [0.89]
Oxfordshire	Neonatal [1.04]	Congenital [1.07]	Diarrhea [1.42]	URI [1.23]	ASD [1.14]	Hernia [0.56]	Iron [2.71]	Urticaria [0.74]	Epilepsy [0.99]	Falls [0.78]
Portsmouth	Neonatal [1.03]	Congenital [1.03]	Diarrhea [1.35]	URI [1.22]	Iron [4.05]	ASD [1.11]	Hernia [0.79]	Urticaria [0.68]	Epilepsy [0.91]	Falls [0.82]
Reading	Neonatal [1.36]	Congenital [1.12]	Diarrhea [1.51]	URI [1.23]	Iron [5.64]	ASD [1.16]	Hernia [0.49]	Urticaria [0.78]	Epilepsy [1.11]	Vit A [1.29]
Slough	Neonatal [1.13]	Congenital [1.04]	Diarrhea [1.44]	URI [1.21]	Iron [3.38]	ASD [1.11]	Hernia [0.63]	Urticaria [0.68]	Epilepsy [0.93]	Falls [0.84]
Southampton	Neonatal [1.02]	Congenital [1.04]	Diarrhea [1.37]	URI [1.21]	ASD [1.12]	Iron [2.18]	Hernia [0.55]	Urticaria [0.69]	Epilepsy [0.93]	Falls [0.83]
Surrey	Neonatal [1.22]	Congenital [1.08]	Diarrhea [1.41]	URI [1.23]	ASD [1.16]	Hernia [0.6]	Iron [2.65]	Urticaria [0.75]	Epilepsy [1.0]	Falls [0.77]
West Berkshire	Neonatal [1.01]	Congenital [1.05]	Diarrhea [1.4]	URI [1.22]	ASD [1.13]	Iron [3.3]	Hernia [0.56]	Urticaria [0.71]	Vit A [1.01]	Epilepsy [0.94]
W Sussex	Neonatal [1.03]	Congenital [1.01]	Diarrhea [1.26]	URI [1.21]	ASD [1.11]	Iron [2.46]	Hernia [0.71]	Urticaria [0.65]	Vit A [0.88]	Falls [0.87]
Windsor & Maidenhead	Neonatal [1.09]	Congenital [1.1]	Diarrhea [1.55]	URI [1.23]	ASD [1.16]	Hernia [0.48]	Iron [2.67]	Urticaria [0.76]	Epilepsy [1.0]	Falls [0.76]
Wokingham	Neonatal [1.11]	Congenital [1.09]	Diarrhea [1.51]	URI [1.23]	ASD [1.17]	Hernia [0.5]	Iron [2.6]	Urticaria [0.75]	Other MN [4.4]	Falls [0.77]
SW England	Neonatal [1.08]	Congenital [0.95]	Diarrhea [1.25]	URI [1.2]	Iron [3.59]	ASD [1.14]	Hernia [0.76]	Urticaria [0.64]	Vit A [0.84]	Epilepsy [0.82]
Bath & NE Somerset	Neonatal [1.51]	Congenital [1.0]	Diarrhea [1.53]	URI [1.22]	ASD [1.34]	Iron [4.22]	Hernia [0.57]	Urticaria [0.72]	Epilepsy [0.94]	Falls [0.78]
Bournemouth	Neonatal [1.1]	Congenital [0.98]	Diarrhea [1.35]	URI [1.22]	Iron [4.25]	ASD [1.11]	Hernia [0.68]	Vit A [1.27]	Urticaria [0.69]	Epilepsy [0.89]
Bristol	Neonatal [1.34]	Congenital [1.04]	Diarrhea [1.39]	URI [1.23]	ASD [1.34]	Iron [4.71]	Hernia [0.57]	Urticaria [0.75]	Epilepsy [1.04]	Falls [0.75]
Cornwall	Neonatal [1.03]	Congenital [0.92]	Diarrhea [1.17]	Iron [3.05]	URI [1.2]	ASD [1.08]	Hernia [0.84]	Vit A [0.75]	Urticaria [0.6]	Epilepsy [0.75]
Devon	Neonatal [0.96]	Congenital [0.94]	Diarrhea [1.28]	URI [1.2]	Iron [2.48]	ASD [1.09]	Hernia [0.81]	Urticaria [0.63]	Epilepsy [0.81]	Falls [0.93]
Dorset	Neonatal [1.01]	Congenital [0.93]	Diarrhea [1.19]	URI [1.2]	Iron [3.3]	ASD [1.09]	Hernia [0.79]	Urticaria [0.62]	Vit A [0.64]	Epilepsy [0.77]
Gloucestershire	Neonatal [1.0]	Congenital [0.96]	Iron [5.89]	Diarrhea [1.38]	URI [1.21]	ASD [1.1]	Hernia [0.85]	Urticaria [0.66]	Epilepsy [0.84]	Falls [0.86]
N Somerset	Neonatal [1.13]	Congenital [0.94]	Diarrhea [1.08]	URI [1.2]	ASD [1.29]	Iron [2.11]	Vit A [1.21]	Hernia [0.68]	Urticaria [0.62]	Epilepsy [0.78]
Plymouth	Neonatal [1.05]	Congenital [0.95]	Diarrhea [1.24]	URI [1.2]	Iron [3.2]	Hernia [0.97]	ASD [1.09]	Vit A [0.89]	Urticaria [0.63]	Epilepsy [0.82]
Poole	Neonatal [1.05]	Congenital [0.95]	Iron [4.84]	Diarrhea [1.2]	URI [1.2]	ASD [1.1]	Hernia [0.78]	Vit A [0.99]	Urticaria [0.64]	Epilepsy [0.81]
Somerset	Neonatal [1.07]	Congenital [0.91]	Diarrhea [1.16]	Iron [3.05]	URI [1.2]	ASD [1.08]	Hernia [0.93]	Urticaria [0.6]	Vit A [0.64]	Falls [1.0]
S Gloucestershire	Neonatal [1.17]	Congenital [0.98]	Diarrhea [1.27]	URI [1.21]	ASD [1.32]	Iron [3.78]	Vit A [1.72]	Hernia [0.61]	Urticaria [0.7]	Falls [0.81]
Swindon	Neonatal [0.81]	Congenital [0.95]	Diarrhea [1.25]	URI [1.21]	ASD [1.1]	Iron [2.61]	Hernia [0.69]	Urticaria [0.65]	Vit A [0.79]	Epilepsy [0.84]
Torbay	Neonatal [0.81]	Congenital [0.9]	Diarrhea [1.04]	URI [1.2]	Iron [1.74]	ASD [1.07]	Vit A [0.78]	Hernia [0.86]	Urticaria [0.57]	Falls [1.14]
Wiltshire	Neonatal [1.19]	Congenital [0.93]	Diarrhea [1.14]	URI [1.2]	Iron [3.48]	ASD [1.09]	Hernia [0.7]	Vit A [0.8]	Urticaria [0.63]	Epilepsy [0.77]
W Midlands	Neonatal [0.99]	Congenital [0.98]	Diarrhea [1.32]	URI [1.2]	Iron [2.56]	ASD [1.01]	Hernia [0.71]	Urticaria [0.62]	Falls [1.01]	Epilepsy [0.81]
Birmingham	Neonatal [1.0]	Congenital [1.01]	Diarrhea [1.38]	URI [1.2]	Iron [3.33]	Hernia [0.71]	ASD [0.8]	Urticaria [0.64]	Falls [1.03]	Epilepsy [0.86]

eFigure 9b. Leading ten causes of YLDs with the ratio of observed YLDs to YLDs expected on the basis of Socio-Demographic Index alone in 2017, <1 years, both sexes combined.
The top ten causes contributing to YLDs are listed globally, by socio-demographic quintile, and then by GBD superregion, region, country, and subnationally where modeled. For each cell, the ratio of observed YLDs to YLDs expected on the basis of socio-demographic index (SDI) alone are listed. Abbreviations: YLD=year of life lived with disability, GBD=Global Burden of Disease.

Values shown in brackets represent the ratio of observed YLDs to predicted YLDs on the basis of Socio-Demographic Index (SDI), rounded to two (2) digits. Color ranges (shown below) were calculated to place a roughly equal number of cells into each bin.

COLOR KEY:	[0.0-0.68]	[0.68-0.82]	[0.82-0.91]	[0.91-1.01]	[1.01-1.1]	[1.1-1.21]	[1.21-1.45]	[1.45-2.25]	2.25+

	1	2	3	4	5	6	7	8	9	10
Coventry	Neonatal [0.98]	Congenital [1.02]	Diarrhea [1.34]	URI [1.21]	ASD [1.1]	Hernia [0.61]	Iron [1.53]	Urticaria [0.66]	Epilepsy [0.9]	Falls [0.89]
Dudley	Neonatal [1.04]	Congenital [0.95]	Diarrhea [1.2]	URI [1.2]	ASD [1.07]	Iron [1.28]	Hernia [0.79]	Urticaria [0.58]	Falls [1.12]	Epilepsy [0.73]
Herefordshire	Neonatal [1.03]	Congenital [0.98]	Diarrhea [1.33]	URI [1.2]	ASD [1.09]	Iron [2.07]	Hernia [0.69]	Urticaria [0.62]	Falls [0.94]	Epilepsy [0.77]
Sandwell	Neonatal [1.01]	Congenital [0.95]	Diarrhea [1.2]	URI [1.2]	ASD [1.07]	Iron [1.29]	Hernia [0.8]	Vit A [0.63]	Urticaria [0.58]	Epilepsy [0.75]
Shropshire	Neonatal [0.98]	Congenital [0.97]	Diarrhea [1.27]	URI [1.2]	Iron [2.71]	ASD [1.09]	Hernia [0.73]	Urticaria [0.62]	Epilepsy [0.79]	Falls [0.94]
Solihull	Neonatal [0.79]	Congenital [1.01]	Diarrhea [1.36]	URI [1.21]	ASD [1.11]	Iron [2.48]	Hernia [0.61]	Urticaria [0.67]	Falls [0.91]	Epilepsy [0.86]
Staffordshire	Neonatal [0.96]	Congenital [0.97]	Diarrhea [1.41]	URI [1.2]	Iron [3.03]	ASD [1.03]	Hernia [0.71]	Urticaria [0.61]	Vit A [0.71]	Falls [1.04]
Stoke-on-Trent	Neonatal [1.12]	Congenital [0.96]	Diarrhea [1.27]	URI [1.2]	ASD [1.07]	Iron [1.44]	Hernia [0.86]	Vit A [0.69]	Urticaria [0.58]	Falls [1.21]
Telford & Wrekin	Neonatal [0.98]	Congenital [0.96]	Diarrhea [1.23]	Iron [3.72]	URI [1.2]	ASD [1.08]	Hernia [0.73]	Urticaria [0.61]	Epilepsy [0.78]	Falls [1.0]
Walsall	Neonatal [0.94]	Congenital [0.95]	Diarrhea [1.07]	Iron [2.3]	URI [1.21]	ASD [1.07]	Hernia [0.76]	Urticaria [0.57]	Vit A [0.4]	Falls [1.19]
Warwickshire	Neonatal [1.04]	Congenital [1.02]	Diarrhea [1.42]	URI [1.21]	ASD [1.1]	Iron [2.78]	Hernia [0.62]	Urticaria [0.68]	Falls [0.9]	Epilepsy [0.87]
Wolverhampton	Neonatal [0.75]	Congenital [0.96]	Diarrhea [1.3]	URI [1.2]	ASD [1.07]	Iron [1.7]	Hernia [0.77]	Urticaria [0.59]	Falls [1.06]	Epilepsy [0.75]
Worcestershire	Neonatal [1.11]	Congenital [0.98]	Diarrhea [1.26]	URI [1.2]	ASD [1.1]	Iron [2.27]	Hernia [0.77]	Urticaria [0.63]	Falls [0.97]	Epilepsy [0.82]
Yorkshire & Humber	Neonatal [0.94]	Congenital [0.99]	Iron [4.39]	Diarrhea [1.26]	URI [1.2]	ASD [1.08]	Hernia [0.76]	Urticaria [0.62]	Vit A [0.71]	Epilepsy [0.77]
Barnsley	Neonatal [0.94]	Congenital [0.94]	Iron [2.93]	Diarrhea [1.13]	URI [1.2]	ASD [1.07]	Hernia [0.9]	Vit A [0.54]	Urticaria [0.56]	Epilepsy [0.71]
Bradford	Neonatal [0.98]	Congenital [0.97]	Diarrhea [1.14]	Iron [2.94]	URI [1.2]	ASD [1.07]	Hernia [0.91]	Urticaria [0.59]	Vit A [0.47]	Falls [1.02]
Calderdale	Neonatal [0.9]	Congenital [0.98]	Diarrhea [1.26]	Iron [3.97]	URI [1.2]	ASD [1.08]	Hernia [0.77]	Urticaria [0.61]	Vit A [0.64]	Epilepsy [0.8]
Doncaster	Neonatal [1.13]	Congenital [0.94]	Diarrhea [1.1]	Iron [2.03]	URI [1.2]	ASD [1.07]	Hernia [0.97]	Vit A [0.51]	Urticaria [0.57]	Epilepsy [0.69]
E Riding of Yorkshire	Neonatal [0.9]	Congenital [0.96]	Iron [4.35]	Diarrhea [1.16]	URI [1.2]	ASD [1.09]	Hernia [0.87]	Vit A [0.7]	Urticaria [0.61]	Epilepsy [0.75]
Kingston upon Hull	Neonatal [0.84]	Congenital [0.97]	Iron [4.6]	Diarrhea [1.15]	URI [1.2]	ASD [1.07]	Hernia [0.75]	Vit A [0.7]	Urticaria [0.6]	Epilepsy [0.77]
Kirklees	Neonatal [0.86]	Congenital [0.97]	Diarrhea [1.22]	Iron [3.31]	URI [1.2]	ASD [1.07]	Hernia [0.78]	Urticaria [0.6]	Epilepsy [0.74]	Falls [0.98]
Leeds	Neonatal [0.93]	Congenital [1.05]	Iron [8.34]	Diarrhea [1.41]	URI [1.22]	ASD [1.12]	Hernia [0.61]	Urticaria [0.71]	Epilepsy [0.94]	Falls [0.8]
NE Lincolnshire	Neonatal [0.87]	Congenital [0.95]	Iron [3.58]	Diarrhea [1.01]	URI [1.2]	ASD [1.07]	Hernia [0.69]	Vit A [0.64]	Urticaria [0.59]	Epilepsy [0.72]
N Lincolnshire	Neonatal [0.82]	Congenital [0.96]	Iron [3.44]	Diarrhea [1.14]	URI [1.2]	ASD [1.07]	Hernia [0.72]	Urticaria [0.59]	Vit A [0.54]	Epilepsy [0.76]
N Yorkshire	Neonatal [1.06]	Congenital [0.99]	Diarrhea [1.37]	Iron [4.11]	URI [1.2]	ASD [1.09]	Hernia [0.81]	Urticaria [0.64]	Vit A [0.76]	Falls [0.89]
Rotherham	Neonatal [0.82]	Congenital [0.95]	Iron [2.76]	Diarrhea [1.06]	URI [1.2]	ASD [1.07]	Hernia [0.81]	Vit A [0.56]	Urticaria [0.57]	Epilepsy [0.71]
Sheffield	Neonatal [1.03]	Congenital [1.02]	Diarrhea [1.41]	Iron [5.06]	URI [1.21]	ASD [1.11]	Hernia [0.73]	Urticaria [0.67]	Epilepsy [0.9]	Falls [0.84]
Wakefield	Neonatal [0.74]	Congenital [0.95]	Diarrhea [1.28]	Iron [3.04]	URI [1.2]	ASD [1.07]	Vit A [1.0]	Hernia [0.76]	Urticaria [0.59]	Epilepsy [0.72]
York	Neonatal [0.96]	Congenital [1.07]	Diarrhea [1.49]	URI [1.22]	Iron [5.25]	ASD [1.13]	Hernia [0.76]	Urticaria [0.73]	Epilepsy [0.95]	Falls [0.76]
N Ireland	Neonatal [0.91]	Congenital [1.01]	Diarrhea [1.06]	URI [1.17]	Hernia [1.19]	ASD [1.02]	Urticaria [0.63]	Epilepsy [0.86]	Falls [0.91]	Otitis [1.25]
Scotland	Neonatal [0.83]	Congenital [1.21]	URI [1.16]	Diarrhea [0.87]	Hernia [1.28]	Iron [1.88]	ASD [1.02]	Vit A [0.94]	Urticaria [0.59]	Epilepsy [0.86]

eFigure 9b. Leading ten causes of YLDs with the ratio of observed YLDs to YLDs expected on the basis of Socio-Demographic Index alone in 2017, <1 years, both sexes combined.
The top ten causes contributing to YLDs are listed globally, by socio-demographic quintile, and then by GBD superregion, region, country, and subnationally where modeled. For each cell, the ratio of observed YLDs to YLDs expected on the basis of socio-demographic index (SDI) alone are listed. Abbreviations: YLD=year of life lived with disability, GBD=Global Burden of Disease.

Values shown in brackets represent the ratio of observed YLDs to predicted YLDs on the basis of Socio-Demographic Index (SDI), rounded to two (2) digits. Color ranges (shown below) were calculated to place a roughly equal number of cells into each bin.

COLOR KEY:	[0.0-0.68]	(0.68-0.82]	(0.82-0.91]	(0.91-1.01]	(1.01-1.1]	(1.1-1.21]	(1.21-1.45]	(1.45-2.25]	2.25+

	1	2	3	4	5	6	7	8	9	10
Wales	Neonatal [0.82]	Congenital [0.95]	URI [1.17]	Diarrhea [0.93]	Iron [2.19]	Hernia [1.32]	ASD [0.96]	Vit A [0.9]	Urticaria [0.59]	Epilepsy [0.71]
**Latin America and Caribbean**	Neonatal [1.19]	Diarrhea [1.54]	Iron [1.13]	Vit A [0.82]	Congenital [0.75]	Epilepsy [1.53]	URI [0.9]	Urticaria [0.87]	ASD [0.82]	Oth Neuro [2.16]
**Andean Latin America**	Iron [2.87]	Neonatal [1.15]	Vit A [1.01]	Diarrhea [1.26]	Congenital [0.72]	URI [1.0]	Urticaria [0.85]	Epilepsy [1.02]	Oth NTD [11.48]	ASD [0.81]
Bolivia	Iron [2.91]	Neonatal [1.14]	Diarrhea [1.23]	Vit A [0.73]	Congenital [0.77]	Oth NTD [3.83]	URI [0.9]	Urticaria [0.85]	Epilepsy [0.93]	Oth Un Inf [2.83]
Ecuador	Neonatal [1.16]	Diarrhea [0.97]	Congenital [0.71]	Vit A [0.5]	URI [0.96]	Epilepsy [1.11]	Urticaria [0.86]	ASD [0.82]	Oth Neuro [1.58]	Hernia [0.82]
Peru	Iron [3.82]	Neonatal [1.15]	Vit A [1.29]	Diarrhea [1.35]	Congenital [0.72]	URI [1.07]	Oth NTD [18.57]	Urticaria [0.85]	Epilepsy [1.0]	Oth Un Inf [3.86]
**Caribbean**	Iron [2.46]	Neonatal [0.99]	Vit A [1.17]	Diarrhea [1.29]	Congenital [0.77]	URI [0.94]	Urticaria [0.85]	Epilepsy [0.9]	PEM [3.25]	Hemog [2.13]
Antigua	Neonatal [0.94]	Iron [2.21]	Congenital [0.66]	Diarrhea [0.79]	Vit A [0.52]	URI [0.89]	Urticaria [0.92]	Epilepsy [1.02]	ASD [0.75]	Scabies [2.63]
Bahamas	Neonatal [1.05]	Congenital [0.68]	Vit A [1.13]	Iron [1.32]	Diarrhea [0.83]	URI [0.87]	Urticaria [0.97]	Epilepsy [1.23]	ASD [0.73]	Hemog [3.93]
Barbados	Neonatal [1.08]	Congenital [0.66]	Iron [1.45]	Diarrhea [0.84]	Vit A [0.78]	URI [0.88]	Urticaria [0.95]	Epilepsy [1.12]	ASD [0.74]	Scabies [2.97]
Belize	Neonatal [0.91]	Iron [1.04]	Congenital [0.72]	Diarrhea [0.58]	Vit A [0.36]	URI [0.98]	Urticaria [0.85]	Epilepsy [0.9]	ASD [0.84]	PEM [1.39]
Bermuda	Neonatal [1.07]	Congenital [0.66]	Iron [3.33]	Diarrhea [0.92]	URI [0.86]	Urticaria [1.06]	Epilepsy [1.37]	Vit A [0.88]	ASD [0.73]	Scabies [5.15]
Cuba	Neonatal [0.83]	Iron [1.09]	Congenital [0.65]	URI [0.91]	Diarrhea [0.42]	Urticaria [0.89]	ASD [0.77]	Vit A [0.25]	Epilepsy [0.72]	Scabies [2.16]
Dominica	Neonatal [1.16]	Iron [1.06]	Congenital [0.7]	Diarrhea [0.83]	URI [0.91]	Vit A [0.35]	Epilepsy [1.27]	Urticaria [0.89]	ASD [0.77]	Oth Neuro [1.79]
Dominican Rep	Neonatal [0.95]	Iron [1.25]	Diarrhea [1.18]	Congenital [0.74]	Vit A [0.47]	URI [0.84]	Urticaria [0.85]	Epilepsy [0.81]	ASD [0.84]	Scabies [1.43]
Grenada	Iron [2.46]	Neonatal [0.98]	Congenital [0.68]	Vit A [0.54]	Diarrhea [0.68]	URI [0.94]	Urticaria [0.86]	Epilepsy [0.97]	ASD [0.8]	Oth NTD [14.43]
Guyana	Iron [1.73]	Neonatal [0.95]	Vit A [0.57]	Congenital [0.72]	Diarrhea [0.65]	PEM [3.27]	URI [0.95]	Urticaria [0.85]	Epilepsy [0.87]	ASD [0.85]
Haiti	Iron [1.88]	Vit A [0.89]	Neonatal [0.86]	Diarrhea [1.22]	Congenital [0.87]	PEM [0.75]	Hemog [1.61]	URI [1.13]	Oth NTD [1.44]	Urticaria [0.91]
Jamaica	Neonatal [1.08]	Iron [1.68]	Congenital [0.68]	Diarrhea [0.57]	Vit A [0.41]	URI [0.92]	Epilepsy [1.28]	Urticaria [0.88]	ASD [0.77]	Oth Neuro [1.81]
Puerto Rico	Neonatal [1.13]	Congenital [0.71]	Diarrhea [1.04]	URI [0.85]	Iron [1.94]	Urticaria [1.08]	Epilepsy [1.36]	ASD [0.73]	Scabies [5.44]	Vit A [0.46]
St Lucia	Neonatal [1.0]	Iron [0.97]	Diarrhea [0.9]	Congenital [0.67]	Vit A [0.48]	URI [0.93]	Urticaria [0.87]	Epilepsy [0.94]	ASD [0.79]	Scabies [1.74]
St Vincent	Neonatal [0.92]	Vit A [0.62]	Congenital [0.69]	Iron [0.62]	Diarrhea [0.63]	URI [0.96]	Urticaria [0.85]	Epilepsy [0.88]	ASD [0.83]	Hemog [1.11]
Suriname	Neonatal [1.04]	Iron [1.17]	Congenital [0.72]	Diarrhea [0.76]	Vit A [0.36]	URI [0.94]	Urticaria [0.86]	Epilepsy [1.02]	ASD [0.8]	Scabies [1.67]
Trinidad Tobago	Neonatal [1.04]	Congenital [0.67]	Diarrhea [0.58]	Iron [0.63]	URI [0.91]	Urticaria [0.91]	Epilepsy [1.04]	Vit A [0.28]	ASD [0.76]	Whooping [2.2]
Virgin Isl US	Neonatal [1.09]	Iron [3.88]	Congenital [0.66]	Diarrhea [1.09]	URI [0.85]	Urticaria [1.06]	Vit A [0.98]	Epilepsy [1.44]	ASD [0.72]	Whooping [2.67]
**Central Latin America**	Neonatal [1.38]	Diarrhea [1.97]	Congenital [0.73]	Vit A [0.59]	Iron [0.59]	Epilepsy [1.96]	URI [0.9]	Urticaria [0.86]	Oth Neuro [2.76]	ASD [0.85]
Colombia	Neonatal [1.43]	Diarrhea [2.05]	Iron [0.84]	Congenital [0.74]	Vit A [0.45]	Epilepsy [1.98]	URI [1.07]	Urticaria [0.86]	Oth Neuro [2.79]	ASD [0.82]
Costa Rica	Neonatal [1.3]	Diarrhea [1.94]	Congenital [0.67]	Epilepsy [1.79]	URI [0.84]	Vit A [0.3]	Urticaria [0.87]	ASD [0.8]	Oth Neuro [2.52]	LRI [1.48]
El Salvador	Neonatal [1.33]	Diarrhea [2.08]	Iron [1.1]	Vit A [0.86]	Congenital [0.77]	Epilepsy [1.51]	URI [0.88]	Urticaria [0.85]	ASD [0.85]	Oth Neuro [2.13]
Guatemala	Neonatal [1.22]	Diarrhea [2.17]	Iron [1.41]	Congenital [0.79]	Vit A [0.39]	Epilepsy [1.06]	Urticaria [0.88]	URI [0.81]	ASD [0.89]	Oth NTD [1.34]
Honduras	Neonatal [1.32]	Diarrhea [2.3]	Iron [1.29]	Congenital [0.77]	Vit A [0.34]	Epilepsy [1.69]	URI [0.93]	Urticaria [0.88]	Oth Neuro [2.38]	ASD [0.91]

eFigure 9b. Leading ten causes of YLDs with the ratio of observed YLDs to YLDs expected on the basis of Socio-Demographic Index alone in 2017, <1 years, both sexes combined.
The top ten causes contributing to YLDs are listed globally, by socio-demographic quintile, and then by GBD superregion, region, country, and subnationally where modeled. For each cell, the ratio of observed YLDs to YLDs expected on the basis of socio-demographic index (SDI) alone are listed. Abbreviations: YLD=year of life lived with disability, GBD=Global Burden of Disease.

Values shown in brackets represent the ratio of observed YLDs to predicted YLDs on the basis of Socio-Demographic Index (SDI), rounded to two (2) digits. Color ranges (shown below) were calculated to place a roughly equal number of cells into each bin.

COLOR KEY:	[0.0-0.68]	(0.68-0.82]	(0.82-0.91]	(0.91-1.01]	(1.01-1.1]	(1.1-1.21]	(1.21-1.45]	(1.45-2.25]	2.25+

	1	2	3	4	5	6	7	8	9	10
Mexico	Neonatal [1.39]	Diarrhea [1.36]	Vit A [0.74]	Congenital [0.72]	Epilepsy [2.25]	URI [0.88]	Urticaria [0.87]	Oth Neuro [3.17]	ASD [0.89]	Iron [0.15]
Aguascalientes	Neonatal [1.43]	Diarrhea [1.57]	Vit A [1.0]	Congenital [0.78]	Epilepsy [2.45]	URI [0.87]	Urticaria [0.89]	Oth Neuro [3.46]	ASD [0.83]	Iron [0.18]
Baja CA	Neonatal [1.36]	Vit A [0.86]	Diarrhea [1.08]	Congenital [0.74]	Epilepsy [2.35]	URI [0.86]	Urticaria [0.89]	Oth Neuro [3.31]	ASD [0.84]	Iron [0.2]
Baja CA Sur	Neonatal [1.46]	Diarrhea [1.51]	Vit A [0.74]	Congenital [0.69]	Epilepsy [2.37]	URI [0.86]	Urticaria [0.89]	Oth Neuro [3.33]	ASD [0.84]	Whooping [1.42]
Campeche	Neonatal [1.45]	Diarrhea [1.51]	Vit A [0.62]	Congenital [0.73]	Epilepsy [2.05]	URI [0.89]	Urticaria [0.87]	Oth Neuro [2.88]	ASD [0.87]	Iron [0.18]
Chiapas	Neonatal [1.3]	Diarrhea [1.4]	Vit A [0.63]	Congenital [0.72]	Epilepsy [2.04]	URI [0.93]	Urticaria [0.9]	Oth Neuro [2.87]	ASD [0.94]	PEM [0.4]
Chihuahua	Neonatal [1.32]	Diarrhea [1.64]	Congenital [0.78]	Epilepsy [2.38]	Vit A [0.51]	URI [0.87]	Urticaria [0.88]	Oth Neuro [3.35]	ASD [0.85]	Iron [0.15]
Coahuila	Neonatal [1.21]	Diarrhea [1.84]	Congenital [0.7]	Epilepsy [2.33]	Vit A [0.4]	URI [0.87]	Urticaria [0.88]	Oth Neuro [3.28]	ASD [0.84]	Whooping [1.33]
Colima	Neonatal [1.51]	Vit A [1.18]	Diarrhea [1.29]	Congenital [0.7]	Epilepsy [2.36]	PEM [7.75]	Iron [0.39]	URI [0.87]	Urticaria [0.88]	Oth Neuro [3.33]
Mexico City	Neonatal [1.58]	Diarrhea [1.58]	Vit A [1.33]	Congenital [0.76]	Epilepsy [3.03]	URI [0.83]	Oth Neuro [4.27]	Urticaria [0.94]	ASD [0.79]	Whooping [2.23]
Durango	Neonatal [1.26]	Diarrhea [1.34]	Vit A [0.66]	Congenital [0.74]	Epilepsy [2.21]	Iron [0.33]	URI [0.89]	Urticaria [0.87]	Oth Neuro [3.11]	ASD [0.86]
Guanajuato	Neonatal [1.24]	Vit A [0.99]	Diarrhea [1.19]	Congenital [0.75]	Epilepsy [2.24]	ASD [1.52]	URI [0.89]	Urticaria [0.87]	Oth Neuro [3.15]	Iron [0.19]
Guerrero	Neonatal [1.4]	Vit A [0.79]	Diarrhea [1.11]	Congenital [0.69]	Epilepsy [1.98]	URI [0.93]	Urticaria [0.89]	Oth Neuro [2.79]	ASD [0.92]	Iron [0.11]
Hidalgo	Neonatal [1.57]	Diarrhea [0.91]	Congenital [0.74]	Epilepsy [2.1]	Vit A [0.34]	URI [0.92]	Urticaria [0.88]	Oth Neuro [2.95]	ASD [0.9]	Whooping [0.79]
Jalisco	Neonatal [1.4]	Diarrhea [1.24]	Congenital [0.75]	Vit A [0.7]	Epilepsy [2.87]	Oth Neuro [4.04]	URI [0.87]	Urticaria [0.89]	ASD [0.84]	Whooping [1.28]
Mexico	Neonatal [1.61]	Diarrhea [1.39]	Vit A [0.81]	Congenital [0.64]	Epilepsy [2.26]	URI [0.88]	Urticaria [0.88]	Oth Neuro [3.18]	ASD [0.85]	Iron [0.21]
Michoacan	Neonatal [1.16]	Vit A [0.79]	Diarrhea [1.02]	Congenital [0.69]	Epilepsy [2.04]	URI [0.92]	Urticaria [0.87]	Oth Neuro [2.87]	ASD [0.9]	Iron [0.16]
Morelos	Neonatal [1.35]	Diarrhea [1.16]	Congenital [0.76]	Vit A [0.68]	Epilepsy [2.25]	URI [0.88]	Urticaria [0.88]	Oth Neuro [3.17]	ASD [0.85]	Iron [0.09]
Nayarit	Neonatal [1.47]	Diarrhea [1.37]	Congenital [0.74]	Epilepsy [2.1]	Vit A [0.38]	URI [0.89]	Urticaria [0.87]	Oth Neuro [2.96]	ASD [0.86]	Whooping [1.01]
Nuevo Leon	Neonatal [1.19]	Diarrhea [1.39]	Vit A [1.07]	Congenital [0.7]	Epilepsy [2.5]	URI [0.85]	Urticaria [0.9]	Oth Neuro [3.53]	Iron [0.35]	ASD [0.82]
Oaxaca	Neonatal [1.29]	Diarrhea [1.21]	Congenital [0.75]	Vit A [0.38]	Epilepsy [2.19]	URI [0.93]	Urticaria [0.89]	Oth Neuro [3.09]	ASD [0.92]	LRI [1.15]
Puebla	Neonatal [1.22]	Diarrhea [1.27]	Congenital [0.73]	Vit A [0.44]	Epilepsy [1.27]	URI [0.92]	Urticaria [0.87]	ASD [0.9]	Iron [0.18]	Oth Neuro [1.8]
Queretaro	Neonatal [1.45]	Diarrhea [1.19]	Congenital [0.72]	Epilepsy [2.3]	Vit A [0.49]	URI [0.87]	Urticaria [0.88]	Oth Neuro [3.23]	ASD [0.85]	Zika [5693.67]
Quintana Roo	Neonatal [1.7]	Diarrhea [1.76]	Congenital [0.74]	Vit A [0.53]	Epilepsy [2.22]	URI [0.88]	Urticaria [0.87]	Oth Neuro [3.13]	ASD [0.86]	LRI [1.39]
San Luis Potosi	Neonatal [1.22]	Diarrhea [1.18]	Vit A [0.77]	Congenital [0.79]	Epilepsy [2.35]	URI [0.89]	Urticaria [0.87]	Oth Neuro [3.31]	ASD [0.87]	Iron [0.13]
Sinaloa	Neonatal [1.3]	Diarrhea [1.43]	Vit A [0.69]	Congenital [0.69]	Epilepsy [2.25]	URI [0.87]	Urticaria [0.88]	Oth Neuro [3.17]	ASD [0.84]	Zika [6292.53]
Sonora	Neonatal [1.28]	Diarrhea [1.49]	Congenital [0.78]	Vit A [0.64]	Epilepsy [2.37]	URI [0.87]	Iron [0.35]	Urticaria [0.88]	Oth Neuro [3.34]	ASD [0.84]
Tabasco	Neonatal [1.46]	Diarrhea [1.7]	Vit A [0.86]	Congenital [0.8]	Epilepsy [2.16]	URI [0.89]	Urticaria [0.87]	Oth Neuro [3.04]	ASD [0.87]	Iron [0.1]
Tamaulipas	Neonatal [1.5]	Diarrhea [1.58]	Vit A [0.79]	Congenital [0.72]	Epilepsy [2.23]	URI [0.87]	Urticaria [0.88]	Oth Neuro [3.14]	ASD [0.84]	Whooping [1.17]
Tlaxcala	Neonatal [1.44]	Diarrhea [1.24]	Congenital [0.78]	Epilepsy [2.24]	Vit A [0.39]	URI [0.9]	Urticaria [0.87]	Oth Neuro [3.15]	ASD [0.88]	Whooping [0.91]
Veracruz	Neonatal [1.42]	Diarrhea [1.19]	Vit A [0.61]	Congenital [0.75]	Epilepsy [2.09]	URI [0.91]	Urticaria [0.87]	Oth Neuro [2.94]	ASD [0.9]	PEM [0.87]

eFigure 9b. Leading ten causes of YLDs with the ratio of observed YLDs to YLDs expected on the basis of Socio-Demographic Index alone in 2017, <1 years, both sexes combined.
The top ten causes contributing to YLDs are listed globally, by socio-demographic quintile, and then by GBD superregion, region, country, and subnationally where modeled. For each cell, the ratio of observed YLDs to YLDs expected on the basis of socio-demographic index (SDI) alone are listed. Abbreviations: YLD=year of life lived with disability, GBD=Global Burden of Disease.

Values shown in brackets represent the ratio of observed YLDs to predicted YLDs on the basis of Socio-Demographic Index (SDI), rounded to two (2) digits. Color ranges (shown below) were calculated to place a roughly equal number of cells into each bin.

COLOR KEY:	[0.0-0.68]	(0.68-0.82]	(0.82-0.91]	(0.91-1.01]	(1.01-1.1]	(1.1-1.21]	(1.21-1.45]	(1.45-2.25]	2.25+

	1	2	3	4	5	6	7	8	9	10
Yucatan	Neonatal [1.28]	Diarrhea [1.32]	Congenital [0.74]	Epilepsy [2.26]	Vit A [0.4]	URI [0.88]	Urticaria [0.88]	Oth Neuro [3.18]	ASD [0.86]	Iron [0.18]
Zacatecas	Neonatal [0.97]	Diarrhea [1.13]	Congenital [0.69]	Epilepsy [2.25]	Vit A [0.42]	URI [0.9]	Urticaria [0.87]	Oth Neuro [3.17]	ASD [0.88]	Whooping [0.97]
Nicaragua	Neonatal [1.26]	Diarrhea [1.93]	Iron [0.84]	Congenital [0.81]	Epilepsy [1.36]	Urticaria [0.88]	URI [0.79]	ASD [0.9]	Vit A [0.11]	Oth Neuro [1.92]
Panama	Diarrhea [3.49]	Neonatal [1.33]	Congenital [0.69]	Iron [0.64]	Vit A [0.5]	Epilepsy [1.41]	URI [0.83]	Urticaria [0.88]	ASD [0.79]	Oth Neuro [1.99]
Venezuela	Diarrhea [3.68]	Neonatal [1.42]	Congenital [0.71]	PEM [8.95]	Epilepsy [1.8]	Vit A [0.31]	URI [0.84]	Urticaria [0.86]	ASD [0.77]	Oth Neuro [2.55]
**Tropical Latin America**	Neonatal [0.92]	Vit A [1.04]	Congenital [0.78]	Diarrhea [0.95]	Iron [0.83]	URI [0.86]	Epilepsy [1.18]	Urticaria [0.89]	ASD [0.79]	Scabies [2.76]
Brazil	Neonatal [0.93]	Vit A [1.06]	Congenital [0.78]	Diarrhea [0.92]	Iron [0.88]	URI [0.86]	Epilepsy [1.2]	Urticaria [0.89]	ASD [0.79]	Scabies [2.85]
Acre	Neonatal [0.91]	Vit A [0.98]	Diarrhea [1.11]	Congenital [0.92]	Iron [0.68]	URI [0.91]	Urticaria [0.87]	Epilepsy [0.92]	ASD [0.85]	Scabies [1.77]
Alagoas	Vit A [1.0]	Neonatal [0.75]	Diarrhea [0.98]	Congenital [0.82]	Iron [0.24]	URI [0.93]	Urticaria [0.89]	Epilepsy [0.89]	ASD [0.89]	Scabies [1.76]
Amapa	Neonatal [0.93]	Vit A [1.09]	Iron [1.1]	Congenital [0.75]	Diarrhea [0.86]	URI [0.87]	Urticaria [0.88]	Epilepsy [1.04]	ASD [0.79]	Scabies [2.7]
Amazonas	Neonatal [0.79]	Vit A [0.87]	Iron [0.89]	Congenital [0.76]	Diarrhea [0.87]	URI [0.88]	Urticaria [0.87]	Epilepsy [0.96]	ASD [0.82]	Scabies [2.34]
Bahia	Neonatal [0.85]	Vit A [1.02]	Diarrhea [0.99]	Congenital [0.82]	Iron [0.65]	URI [0.91]	Urticaria [0.87]	Epilepsy [0.93]	ASD [0.86]	Scabies [1.88]
Ceara	Neonatal [0.81]	Vit A [0.86]	Congenital [0.83]	Iron [0.73]	Diarrhea [0.86]	URI [0.91]	Urticaria [0.87]	Epilepsy [0.92]	Scabies [2.31]	ASD [0.85]
Distrito Federal	Neonatal [1.13]	Congenital [0.81]	Diarrhea [1.08]	Vit A [1.6]	Iron [1.64]	URI [0.79]	Urticaria [1.04]	Epilepsy [1.46]	ASD [0.73]	Scabies [6.92]
Espirito Santo	Neonatal [0.9]	Iron [1.6]	Vit A [1.2]	Congenital [0.73]	Diarrhea [0.81]	URI [0.85]	Urticaria [0.89]	Epilepsy [1.11]	ASD [0.79]	Scabies [2.95]
Goias	Neonatal [0.81]	Vit A [1.01]	Iron [0.98]	Congenital [0.75]	Diarrhea [0.81]	URI [0.87]	Urticaria [0.88]	Epilepsy [1.03]	ASD [0.81]	Scabies [2.56]
Maranhao	Vit A [0.8]	Neonatal [0.74]	Iron [0.53]	Diarrhea [0.72]	Congenital [0.78]	URI [0.93]	Urticaria [0.91]	Epilepsy [0.8]	ASD [0.91]	Scabies [1.69]
Mato Grosso	Neonatal [0.86]	Vit A [0.92]	Congenital [0.75]	Diarrhea [0.96]	Iron [0.93]	URI [0.86]	Urticaria [0.89]	Epilepsy [1.06]	ASD [0.8]	Scabies [2.72]
Mato Grosso do Sul	Neonatal [0.95]	Congenital [0.85]	Vit A [0.8]	Diarrhea [1.0]	Iron [0.67]	URI [0.86]	Urticaria [0.88]	Epilepsy [1.05]	ASD [0.8]	Scabies [2.55]
Minas Gerais	Neonatal [0.94]	Vit A [0.98]	Iron [0.98]	Congenital [0.76]	Diarrhea [0.91]	URI [0.86]	Urticaria [0.88]	Epilepsy [1.09]	ASD [0.8]	Scabies [2.71]
Para	Neonatal [0.79]	Vit A [0.89]	Diarrhea [1.05]	Iron [0.78]	Congenital [0.78]	URI [0.92]	Urticaria [0.88]	Epilepsy [0.88]	ASD [0.87]	Scabies [1.96]
Paraiba	Neonatal [0.73]	Vit A [0.88]	Iron [0.89]	Diarrhea [0.86]	Congenital [0.84]	URI [0.92]	Urticaria [0.88]	Epilepsy [0.86]	ASD [0.87]	Scabies [1.81]
Parana	Neonatal [0.95]	Vit A [0.99]	Congenital [0.81]	Iron [1.08]	Diarrhea [0.79]	URI [0.85]	Urticaria [0.9]	Epilepsy [1.15]	ASD [0.78]	Scabies [3.06]
Pernambuco	Neonatal [0.81]	Vit A [1.06]	Iron [0.81]	Congenital [0.82]	Diarrhea [0.81]	URI [0.91]	Urticaria [0.87]	Epilepsy [0.94]	ASD [0.86]	Scabies [1.91]
Piaui	Neonatal [0.82]	Diarrhea [0.81]	Congenital [0.82]	Vit A [0.31]	URI [0.93]	Iron [0.2]	Urticaria [0.89]	Epilepsy [0.87]	ASD [0.89]	Scabies [1.73]
Rio de Janeiro	Neonatal [1.22]	Vit A [1.06]	Congenital [0.72]	Diarrhea [0.73]	Epilepsy [1.81]	Iron [0.65]	URI [0.83]	Urticaria [0.93]	ASD [0.76]	Scabies [3.8]
Rio Grande do Norte	Neonatal [0.75]	Vit A [1.01]	Diarrhea [1.12]	Iron [0.8]	Congenital [0.78]	URI [0.9]	Urticaria [0.87]	Epilepsy [0.94]	ASD [0.84]	Scabies [1.98]
Rio Grande do Sul	Neonatal [1.0]	Congenital [1.08]	Vit A [1.14]	Iron [1.41]	Epilepsy [2.67]	Diarrhea [0.72]	URI [0.84]	Urticaria [0.91]	Oth Neuro [3.77]	Scabies [3.41]
Rondonia	Neonatal [0.93]	Congenital [1.1]	Vit A [0.84]	Diarrhea [0.78]	Iron [0.46]	URI [0.88]	Urticaria [0.87]	Epilepsy [0.94]	ASD [0.83]	Scabies [2.38]
Roraima	Neonatal [0.99]	Vit A [0.98]	Congenital [0.77]	Diarrhea [0.94]	Iron [0.54]	URI [0.87]	Urticaria [0.88]	Epilepsy [1.0]	ASD [0.81]	Scabies [2.49]
Santa Catarina	Neonatal [0.89]	Iron [1.42]	Congenital [0.79]	Vit A [0.99]	Diarrhea [0.8]	URI [0.84]	Urticaria [0.92]	Epilepsy [1.13]	ASD [0.76]	Scabies [3.53]

eFigure 9b. Leading ten causes of YLDs with the ratio of observed YLDs to YLDs expected on the basis of Socio-Demographic Index alone in 2017, <1 years, both sexes combined. The top ten causes contributing to YLDs are listed globally, by socio-demographic quintile, and then by GBD superregion, region, country, and subnationally where modeled. For each cell, the ratio of observed YLDs to YLDs expected on the basis of socio-demographic index (SDI) alone are listed. Abbreviations: YLD=year of life lived with disability, GBD=Global Burden of Disease.

Values shown in brackets represent the ratio of observed YLDs to predicted YLDs on the basis of Socio-Demographic Index (SDI), rounded to two (2) digits. Color ranges (shown below) were calculated to place a roughly equal number of cells into each bin.

COLOR KEY:									
[0.0-0.68]	(0.68-0.82]	(0.82-0.91]	(0.91-1.01]	(1.01-1.1]	(1.1-1.21]	(1.21-1.45]	(1.45-2.25]	2.25+	

	1	2	3	4	5	6	7	8	9	10
Sao Paulo	Neonatal [1.05]	Congenital [0.69]	Diarrhea [0.7]	Vit A [0.56]	Epilepsy [1.51]	URI [0.83]	Urticaria [0.94]	Iron [0.45]	ASD [0.76]	Scabies [4.07]
Sergipe	Neonatal [0.94]	Vit A [1.21]	Iron [1.08]	Congenital [0.78]	Diarrhea [0.8]	URI [0.89]	Urticaria [0.87]	Epilepsy [0.96]	ASD [0.83]	Scabies [2.09]
Tocantins	Neonatal [0.91]	Vit A [0.85]	Iron [0.79]	Diarrhea [0.88]	Congenital [0.76]	URI [0.9]	Urticaria [0.87]	Epilepsy [0.92]	ASD [0.84]	Scabies [2.04]
Paraguay	Neonatal [0.82]	Diarrhea [1.65]	Vit A [0.98]	Congenital [0.8]	URI [0.87]	Iron [0.28]	Epilepsy [1.08]	Urticaria [0.86]	ASD [0.83]	Scabies [2.11]
**North Africa and Middle East**	Neonatal [1.15]	Vit A [1.47]	Diarrhea [1.81]	Iron [1.41]	Congenital [0.91]	PEM [8.12]	Hernia [2.66]	Epilepsy [1.37]	URI [0.9]	Urticaria [0.92]
Afghanistan	Vit A [0.57]	Neonatal [0.82]	Diarrhea [1.19]	Congenital [0.96]	Iron [0.34]	ID [4.8]	PEM [0.3]	Epilepsy [1.01]	Hernia [5.68]	URI [1.05]
Algeria	Neonatal [1.23]	Diarrhea [1.74]	Congenital [0.86]	Vit A [1.03]	PEM [38.57]	Hernia [2.16]	URI [0.88]	Epilepsy [1.38]	Urticaria [0.96]	ASD [0.64]
Bahrain	Neonatal [1.09]	Congenital [0.77]	Diarrhea [1.06]	Hernia [1.83]	Vit A [0.5]	URI [0.87]	Epilepsy [1.48]	Urticaria [0.98]	ASD [0.63]	Blindness [2.0]
Egypt	Neonatal [1.14]	Diarrhea [2.09]	Iron [1.51]	Congenital [0.89]	Vit A [0.59]	Hernia [3.21]	Epilepsy [1.47]	Urticaria [0.94]	ID [3.91]	URI [0.67]
Iran	Neonatal [1.47]	Congenital [1.02]	Diarrhea [1.02]	Vit A [0.65]	Hernia [2.18]	Epilepsy [1.73]	PEM [19.62]	URI [0.9]	Iron [0.52]	Urticaria [0.98]
Iraq	Neonatal [1.14]	Vit A [1.15]	Iron [0.92]	Diarrhea [0.91]	Congenital [0.87]	PEM [2.88]	Hernia [3.55]	Epilepsy [1.23]	URI [0.96]	Urticaria [0.91]
Jordan	Neonatal [1.47]	Vit A [2.17]	Iron [1.8]	Diarrhea [1.37]	Congenital [0.8]	Hernia [2.35]	Epilepsy [1.31]	Urticaria [0.95]	URI [0.62]	Hemog [2.94]
Kuwait	Neonatal [1.31]	Congenital [0.82]	Diarrhea [1.53]	Iron [2.55]	Vit A [1.29]	Hernia [1.24]	URI [0.84]	Epilepsy [1.67]	Urticaria [1.09]	ASD [0.61]
Lebanon	Neonatal [1.13]	Diarrhea [2.07]	Congenital [0.85]	Hernia [1.86]	Vit A [0.59]	URI [0.87]	Urticaria [1.0]	Epilepsy [1.35]	Iron [0.57]	ASD [0.64]
Libya	Neonatal [1.15]	Diarrhea [2.2]	Congenital [0.87]	Vit A [1.37]	Iron [1.42]	Hernia [1.65]	URI [0.85]	Urticaria [1.04]	Epilepsy [0.99]	ID [3.92]
Morocco	Neonatal [1.09]	Vit A [1.03]	Diarrhea [1.42]	Congenital [0.91]	Hernia [3.56]	Iron [0.3]	URI [1.05]	Epilepsy [1.18]	Urticaria [0.92]	ID [2.77]
Palestine	Neonatal [0.96]	Vit A [0.62]	Diarrhea [0.93]	Congenital [0.87]	Iron [0.35]	Hernia [4.01]	URI [0.99]	Epilepsy [1.04]	Urticaria [0.94]	ID [3.26]
Oman	Neonatal [1.2]	Diarrhea [1.7]	PEM [142.21]	Vit A [1.68]	Congenital [0.89]	Iron [1.44]	Hernia [1.59]	URI [0.85]	Epilepsy [1.49]	Urticaria [1.02]
Qatar	Neonatal [1.12]	Congenital [0.82]	Diarrhea [1.03]	URI [0.85]	Vit A [0.74]	Epilepsy [1.66]	Urticaria [1.05]	Hernia [0.99]	Blindness [2.39]	ASD [0.61]
Saudi Arabia	Neonatal [0.95]	Diarrhea [1.77]	Congenital [0.93]	Vit A [1.03]	Hernia [1.35]	URI [0.84]	Epilepsy [1.79]	Urticaria [1.08]	ASD [0.61]	Oth Neuro [2.54]
Sudan	Diarrhea [1.94]	Vit A [0.94]	Neonatal [0.95]	Iron [0.88]	PEM [3.41]	Congenital [0.97]	Hemog [2.25]	ID [4.95]	Hernia [4.78]	Epilepsy [1.18]
Syria	Neonatal [1.06]	Vit A [0.84]	Congenital [0.86]	Iron [0.82]	Diarrhea [0.78]	Hernia [3.05]	URI [0.94]	Epilepsy [1.14]	PEM [2.43]	Urticaria [0.91]
Tunisia	Neonatal [1.08]	Iron [1.62]	Diarrhea [1.53]	Congenital [0.81]	Hernia [2.37]	URI [0.9]	Urticaria [0.94]	Vit A [0.27]	Epilepsy [1.02]	Hemog [2.49]
Turkey	Neonatal [0.98]	Congenital [0.79]	Vit A [1.12]	Diarrhea [1.03]	Hernia [1.96]	URI [1.03]	Epilepsy [1.66]	Urticaria [0.97]	ASD [0.63]	Oth Neuro [2.36]
UAE	Neonatal [1.21]	Diarrhea [2.12]	Congenital [0.87]	PEM [557.19]	Vit A [1.57]	Epilepsy [1.98]	Hernia [1.2]	URI [0.83]	Iron [1.39]	Urticaria [1.11]
Yemen	Iron [3.01]	Vit A [2.15]	Diarrhea [2.13]	Neonatal [0.97]	PEM [3.18]	Congenital [0.93]	Hemog [2.57]	Oth NTD [2.28]	ID [5.06]	Oth Un Inf [3.12]
**South Asia**	Vit A [1.71]	Neonatal [1.45]	Iron [1.69]	PEM [5.26]	Congenital [0.96]	Diarrhea [0.64]	ID [6.3]	Hemog [2.22]	URI [0.92]	Urticaria [0.89]
Bangladesh	Iron [1.63]	Neonatal [1.3]	Vit A [0.51]	Congenital [0.94]	PEM [1.23]	Diarrhea [0.43]	URI [1.18]	Hemog [1.57]	Oth NTD [1.3]	Urticaria [0.95]
Bhutan	Iron [3.49]	Neonatal [1.51]	Vit A [1.21]	Congenital [0.8]	Diarrhea [0.71]	Oth NTD [4.06]	Hemog [2.21]	URI [0.92]	Epilepsy [1.08]	Oth Un Inf [3.44]
India	Vit A [2.21]	Neonatal [1.45]	Iron [1.5]	PEM [7.4]	Congenital [1.01]	ID [8.37]	Diarrhea [0.61]	Hemog [2.34]	URI [0.9]	Urticaria [0.88]
Andhra Pradesh	Vit A [1.99]	Neonatal [1.49]	PEM [9.29]	Iron [1.18]	Congenital [0.96]	ID [6.95]	Diarrhea [0.52]	Hemog [1.98]	URI [0.9]	Urticaria [0.89]

eFigure 9b. Leading ten causes of YLDs with the ratio of observed YLDs to YLDs expected on the basis of Socio-Demographic Index alone in 2017, <1 years, both sexes combined.
The top ten causes contributing to YLDs are listed globally, by socio-demographic quintile, and then by GBD superregion, region, country, and subnationally where modeled. For each cell, the ratio of observed YLDs to YLDs expected on the basis of socio-demographic index (SDI) alone are listed. Abbreviations: YLD=year of life lived with disability, GBD=Global Burden of Disease.

Values shown in brackets represent the ratio of observed YLDs to predicted YLDs on the basis of Socio-Demographic Index (SDI), rounded to two (2) digits. Color ranges (shown below) were calculated to place a roughly equal number of cells into each bin.

COLOR KEY:	[0.0-0.68]	[0.68-0.82]	[0.82-0.91]	[0.91-1.01]	[1.01-1.1]	[1.1-1.21]	[1.21-1.45]	[1.45-2.25]	2.25+

	1	2	3	4	5	6	7	8	9	10
Arunachal Pradesh	Vit A [2.03]	Neonatal [1.47]	PEM [11.2]	Iron [1.53]	Congenital [0.97]	ID [6.86]	Diarrhea [0.53]	Hemog [2.09]	URI [0.9]	Urticaria [0.88]
Assam	Vit A [1.62]	Neonatal [1.42]	PEM [3.84]	Congenital [1.05]	Iron [0.62]	ID [8.88]	Diarrhea [0.56]	URI [0.91]	Hemog [1.64]	Skin Fung [2.62]
Bihar	Vit A [1.53]	Neonatal [1.28]	Iron [1.2]	PEM [2.64]	Congenital [1.04]	ID [9.54]	Diarrhea [0.61]	Hemog [1.79]	URI [0.93]	Urticaria [0.94]
Chhattisgarh	Vit A [1.82]	Neonatal [1.43]	Iron [0.92]	PEM [3.99]	Congenital [1.0]	ID [7.76]	Diarrhea [0.46]	Hemog [2.0]	URI [0.91]	Urticaria [0.9]
Delhi	Iron [9.58]	Vit A [4.48]	Neonatal [1.66]	PEM [155.11]	Congenital [0.81]	Diarrhea [1.0]	Oth NTD [3588.08]	Hemog [7.66]	Oth Un Inf [8.9]	ID [6.08]
Goa	Neonatal [1.96]	Iron [6.15]	PEM [313.89]	Vit A [3.53]	Congenital [0.84]	Diarrhea [0.96]	URI [0.79]	ID [5.68]	Hemog [5.34]	Urticaria [0.96]
Gujarat	Vit A [2.47]	PEM [17.06]	Neonatal [1.54]	Iron [1.86]	Congenital [0.95]	Diarrhea [0.61]	ID [6.67]	Hemog [2.57]	URI [0.89]	Urticaria [0.87]
Haryana	Vit A [3.0]	Iron [3.45]	Neonatal [1.54]	Congenital [0.92]	PEM [6.21]	Diarrhea [0.64]	Hemog [3.56]	ID [5.88]	Oth NTD [5.96]	URI [0.88]
Himachal Pradesh	Vit A [3.19]	Neonatal [1.71]	Iron [1.72]	PEM [17.53]	Congenital [0.88]	Diarrhea [0.62]	ID [6.97]	Hemog [3.44]	URI [0.85]	Urticaria [0.87]
Jammu & Kashmir	Vit A [2.81]	Neonatal [1.46]	Iron [1.6]	Congenital [0.96]	PEM [4.67]	ID [8.33]	Diarrhea [0.57]	Hemog [2.74]	URI [0.89]	Urticaria [0.87]
Jharkhand	Vit A [1.86]	Iron [1.64]	Neonatal [1.4]	PEM [4.38]	Congenital [1.01]	ID [7.85]	Diarrhea [0.52]	Hemog [2.12]	URI [0.91]	Oth NTD [1.49]
Karnataka	Vit A [2.37]	Neonatal [1.68]	PEM [12.95]	Iron [1.87]	Congenital [0.92]	Diarrhea [0.55]	ID [6.8]	Hemog [2.42]	Epilepsy [1.2]	URI [0.89]
Kerala	Neonatal [1.96]	PEM [49.93]	Vit A [2.28]	Iron [1.33]	Congenital [0.85]	ID [6.84]	Diarrhea [0.45]	URI [0.84]	Urticaria [0.89]	Hemog [2.28]
Madhya Pradesh	Vit A [1.72]	Neonatal [1.3]	PEM [5.34]	Iron [1.3]	Congenital [1.03]	ID [8.58]	Diarrhea [0.52]	Hemog [1.96]	URI [0.92]	Urticaria [0.91]
Maharashtra	Vit A [2.53]	Neonatal [1.6]	PEM [29.24]	Iron [1.7]	Congenital [0.91]	Diarrhea [0.71]	ID [6.94]	URI [0.86]	Hemog [2.45]	Urticaria [0.86]
Manipur	Neonatal [1.61]	Vit A [1.76]	Congenital [0.97]	Iron [0.79]	PEM [4.67]	ID [9.09]	Diarrhea [0.66]	URI [0.89]	Urticaria [0.87]	Epilepsy [0.79]
Meghalaya	Neonatal [1.57]	Vit A [1.84]	PEM [8.23]	Iron [1.0]	Congenital [0.99]	ID [8.2]	Diarrhea [0.62]	URI [0.91]	Urticaria [0.88]	Hemog [1.74]
Mizoram	Neonatal [1.66]	PEM [20.52]	Vit A [1.64]	Congenital [0.93]	Diarrhea [0.73]	ID [8.07]	Iron [0.44]	URI [0.88]	Urticaria [0.87]	Epilepsy [0.88]
Nagaland	Neonatal [1.79]	Vit A [2.37]	PEM [23.5]	Iron [1.11]	Congenital [0.93]	Diarrhea [0.63]	ID [7.4]	Iodine [91.63]	URI [0.85]	Urticaria [0.87]
Odisha	Vit A [1.76]	Neonatal [1.39]	PEM [7.67]	Iron [1.05]	Congenital [1.03]	Diarrhea [0.64]	ID [8.29]	Hemog [1.88]	URI [0.91]	Urticaria [0.89]
Punjab	Vit A [2.65]	Neonatal [1.57]	Iron [2.11]	PEM [10.75]	Congenital [0.92]	Diarrhea [0.63]	ID [6.77]	Hemog [2.95]	URI [0.86]	Urticaria [0.87]
Rajasthan	Vit A [1.75]	Neonatal [1.29]	PEM [3.96]	Iron [0.88]	Congenital [1.0]	ID [8.1]	Diarrhea [0.53]	Hemog [1.76]	URI [0.92]	Urticaria [0.91]
Sikkim	Neonatal [1.68]	Vit A [2.48]	Iron [1.9]	Congenital [0.88]	Diarrhea [0.75]	PEM [6.06]	ID [6.03]	URI [0.86]	Hemog [2.61]	Urticaria [0.87]
Tamil Nadu	PEM [26.82]	Neonatal [1.51]	Vit A [2.03]	Iron [1.62]	Congenital [0.8]	ID [7.22]	Diarrhea [0.51]	URI [0.86]	Urticaria [0.87]	Hemog [2.18]
Telangana	Vit A [2.47]	Neonatal [1.55]	PEM [13.97]	Iron [1.8]	Congenital [0.93]	ID [6.9]	Diarrhea [0.5]	Hemog [2.49]	URI [0.89]	Urticaria [0.87]
Tripura	Vit A [2.11]	Neonatal [1.47]	PEM [10.01]	Iron [1.47]	Congenital [0.97]	ID [8.19]	Diarrhea [0.58]	Hemog [2.13]	URI [0.91]	Urticaria [0.88]
Uttar Pradesh	Vit A [2.0]	Neonatal [1.29]	Iron [1.31]	PEM [2.78]	Congenital [1.11]	ID [8.65]	Diarrhea [0.64]	Hemog [2.24]	URI [0.91]	Urticaria [0.91]
Uttarakhand	Vit A [2.88]	Neonatal [1.67]	Iron [2.64]	PEM [7.68]	Congenital [0.93]	Diarrhea [0.71]	ID [6.5]	Hemog [3.17]	URI [0.88]	Urticaria [0.84]
W Bengal	Vit A [1.99]	Neonatal [1.34]	PEM [7.01]	Iron [1.33]	Congenital [0.98]	ID [9.08]	Diarrhea [0.41]	Hemog [2.01]	URI [0.91]	Urticaria [0.89]
UTs other than Delhi	Neonatal [1.81]	Vit A [3.19]	Iron [2.37]	PEM [23.24]	Congenital [0.87]	Diarrhea [0.64]	ID [6.07]	Hemog [3.53]	URI [0.84]	Urticaria [0.88]
Nepal	Neonatal [1.4]	Iron [1.31]	Vit A [0.55]	Congenital [0.84]	Diarrhea [0.42]	Epilepsy [1.22]	URI [0.98]	Urticaria [1.0]	Oth NTD [0.97]	ID [2.29]

eFigure 9b. Leading ten causes of YLDs with the ratio of observed YLDs to YLDs expected on the basis of Socio-Demographic Index alone in 2017, <1 years, both sexes combined.
The top ten causes contributing to YLDs are listed globally, by socio-demographic quintile, and then by GBD superregion, region, country, and subnationally where modeled. For each cell, the ratio of observed YLDs to YLDs expected on the basis of socio-demographic index (SDI) alone are listed. Abbreviations: YLD=year of life lived with disability, GBD=Global Burden of Disease.

Values shown in brackets represent the ratio of observed YLDs to predicted YLDs on the basis of Socio-Demographic Index (SDI), rounded to two (2) digits. Color ranges (shown below) were calculated to place a roughly equal number of cells into each bin.

COLOR KEY:	[0.0-0.68]	(0.68-0.82]	(0.82-0.91]	(0.91-1.01]	(1.01-1.1]	(1.1-1.21]	(1.21-1.45]	(1.45-2.25]	2.25+

	1	2	3	4	5	6	7	8	9	10
Pakistan	Iron [2.25]	Neonatal [1.48]	Vit A [0.64]	Diarrhea [0.88]	Congenital [0.78]	PEM [1.63]	Hemog [2.14]	Oth NTD [1.91]	Epilepsy [1.16]	URI [0.88]
**Southeast Asia, East Asia, and Oceania**	Neonatal [1.17]	Diarrhea [1.52]	Vit A [1.01]	Congenital [0.81]	Iron [0.73]	PEM [17.04]	URI [0.92]	Urticaria [0.84]	Epilepsy [0.89]	ASD [0.72]
**East Asia**	Neonatal [1.42]	Diarrhea [1.68]	Congenital [0.72]	Vit A [0.84]	URI [0.86]	Other MN [22.29]	Urticaria [0.85]	Iron [0.35]	ASD [0.72]	Scabies [3.79]
China	Neonatal [1.42]	Diarrhea [1.6]	Congenital [0.71]	Vit A [0.81]	URI [0.87]	Other MN [23.24]	Urticaria [0.84]	ASD [0.72]	Iron [0.32]	Scabies [3.65]
N Korea	Diarrhea [3.16]	Iron [0.88]	Neonatal [0.61]	Vit A [0.64]	Congenital [0.85]	PEM [1.53]	URI [0.95]	Urticaria [0.8]	Scabies [1.7]	Epilepsy [0.63]
Taiwan	Diarrhea [4.19]	Neonatal [0.73]	Congenital [0.97]	URI [0.82]	Urticaria [1.15]	Other MN [7.34]	Epilepsy [1.44]	Vit A [1.45]	Scabies [13.27]	ASD [0.54]
**Oceania**	Iron [1.82]	Diarrhea [1.52]	Vit A [0.61]	PEM [2.88]	Neonatal [0.64]	Congenital [0.97]	URI [0.96]	Oth NTD [1.46]	Hemog [1.27]	Scabies [2.28]
Am Samoa	Diarrhea [1.83]	Neonatal [0.68]	Vit A [1.16]	Congenital [0.78]	Iron [0.67]	URI [0.85]	Urticaria [0.85]	Scabies [3.77]	Epilepsy [0.85]	PEM [9.68]
Micronesia	Vit A [1.65]	Diarrhea [1.33]	Neonatal [0.62]	Congenital [0.91]	PEM [1.72]	URI [0.94]	Urticaria [0.81]	Iron [0.18]	Scabies [2.22]	Epilepsy [0.68]
Fiji	Diarrhea [2.17]	Iron [1.74]	Vit A [1.22]	Neonatal [0.71]	Congenital [0.81]	PEM [9.23]	URI [0.88]	Urticaria [0.81]	Scabies [3.17]	Epilepsy [0.81]
Guam	Diarrhea [3.51]	Neonatal [0.83]	Vit A [3.13]	Congenital [0.76]	URI [0.81]	Urticaria [0.98]	Scabies [8.39]	Epilepsy [1.04]	ASD [0.62]	Iron [0.64]
Kiribati	Vit A [0.95]	Diarrhea [1.37]	Neonatal [0.59]	PEM [1.66]	Iron [0.49]	Congenital [0.98]	URI [0.98]	Scabies [2.25]	Urticaria [0.88]	Epilepsy [0.62]
Marshall	Vit A [2.06]	Diarrhea [1.3]	Iron [0.87]	Neonatal [0.62]	Congenital [0.91]	PEM [1.54]	URI [0.94]	Urticaria [0.82]	Scabies [2.16]	Epilepsy [0.66]
N Mariana	Diarrhea [2.74]	Neonatal [0.84]	Congenital [0.76]	Vit A [1.15]	URI [0.82]	Iron [0.88]	Urticaria [0.92]	Scabies [6.37]	ASD [0.63]	Epilepsy [0.92]
PNG	Iron [1.86]	Diarrhea [1.44]	PEM [2.46]	Neonatal [0.62]	Vit A [0.45]	Congenital [0.97]	Oth NTD [1.28]	URI [0.98]	Hemog [1.29]	Scabies [2.36]
Samoa	Vit A [1.22]	Diarrhea [1.41]	Iron [1.06]	Neonatal [0.62]	Congenital [0.84]	URI [0.93]	Urticaria [0.82]	Scabies [2.2]	Epilepsy [0.7]	ASD [0.74]
Solomon	Vit A [1.06]	Diarrhea [1.21]	Iron [0.6]	Neonatal [0.56]	Congenital [0.93]	PEM [0.6]	URI [0.98]	Urticaria [0.88]	Scabies [2.25]	Malaria [6.56]
Tonga	Vit A [1.19]	Neonatal [0.66]	Diarrhea [1.4]	Congenital [0.83]	PEM [6.67]	URI [0.9]	Urticaria [0.81]	Scabies [2.81]	Iron [0.19]	Epilepsy [0.71]
Vanuatu	Vit A [1.08]	Diarrhea [1.23]	Neonatal [0.6]	Iron [0.62]	Congenital [0.91]	PEM [0.93]	URI [0.96]	Urticaria [0.86]	Scabies [2.06]	Epilepsy [0.63]
**Southeast Asia**	Neonatal [0.79]	Vit A [1.18]	PEM [17.56]	Diarrhea [1.24]	Congenital [0.99]	Iron [1.03]	URI [1.03]	Epilepsy [1.08]	Urticaria [0.84]	Scabies [2.77]
Cambodia	Iron [1.8]	Neonatal [0.73]	Vit A [0.63]	Diarrhea [0.97]	Congenital [1.07]	PEM [1.28]	Hemog [2.0]	Epilepsy [1.2]	Oth NTD [1.54]	URI [0.86]
Indonesia	PEM [40.22]	Neonatal [0.81]	Diarrhea [1.74]	Vit A [1.07]	Congenital [0.95]	URI [1.12]	Urticaria [0.85]	Epilepsy [1.08]	Scabies [2.93]	ASD [0.71]
Laos	Vit A [0.86]	Neonatal [0.74]	Congenital [1.12]	Diarrhea [0.97]	Iron [0.45]	URI [0.95]	Epilepsy [0.99]	Urticaria [0.85]	Scabies [1.92]	ASD [0.83]
Malaysia	Neonatal [0.89]	PEM [280.02]	Diarrhea [1.67]	Congenital [0.9]	Iron [1.06]	URI [0.82]	Epilepsy [1.39]	Urticaria [0.94]	ASD [0.68]	Scabies [5.7]
Maldives	Neonatal [0.7]	Iron [1.2]	Vit A [0.92]	Congenital [0.91]	PEM [17.16]	Hemog [7.27]	Diarrhea [0.64]	URI [0.91]	Urticaria [0.84]	Epilepsy [0.98]
Mauritius	Iron [3.34]	Neonatal [0.89]	PEM [101.5]	Diarrhea [1.67]	Congenital [0.87]	Vit A [0.71]	URI [0.83]	Epilepsy [1.38]	Urticaria [0.89]	Scabies [4.35]
Myanmar	Neonatal [0.83]	Vit A [0.87]	Iron [0.98]	Congenital [1.35]	Diarrhea [1.01]	PEM [1.51]	URI [0.94]	Epilepsy [1.03]	Urticaria [0.84]	Hemog [1.47]
Philippines	Vit A [1.6]	Iron [1.59]	Neonatal [0.85]	Congenital [1.01]	Diarrhea [0.77]	PEM [4.19]	URI [1.28]	Epilepsy [1.09]	Urticaria [0.82]	Scabies [2.45]
Sri Lanka	Neonatal [0.79]	PEM [41.01]	Congenital [0.96]	Vit A [0.98]	Diarrhea [0.77]	Iron [0.76]	Epilepsy [1.79]	URI [0.86]	Urticaria [0.85]	ASD [0.74]
Seychelles	Neonatal [0.85]	Congenital [0.88]	Diarrhea [1.03]	Vit A [0.73]	PEM [16.21]	URI [0.85]	Epilepsy [1.32]	Urticaria [0.87]	Iron [0.34]	Scabies [3.54]
Thailand	Iron [2.53]	Vit A [1.65]	Neonatal [0.71]	Congenital [0.98]	Diarrhea [0.93]	Hemog [5.19]	PEM [11.05]	Urticaria [0.86]	URI [0.7]	Epilepsy [1.09]

eFigure 9b. Leading ten causes of YLDs with the ratio of observed YLDs to YLDs expected on the basis of Socio-Demographic Index alone in 2017, <1 years, both sexes combined.
The top ten causes contributing to YLDs are listed globally, by socio-demographic quintile, and then by GBD superregion, region, country, and subnationally where modeled. For each cell, the ratio of observed YLDs to YLDs expected on the basis of socio-demographic index (SDI) alone are listed. Abbreviations: YLD=year of life lived with disability, GBD=Global Burden of Disease.

Values shown in brackets represent the ratio of observed YLDs to predicted YLDs on the basis of Socio-Demographic Index (SDI), rounded to two (2) digits. Color ranges (shown below) were calculated to place a roughly equal number of cells into each bin.

COLOR KEY:	[0.0-0.68]	(0.68-0.82]	(0.82-0.91]	(0.91-1.01]	(1.01-1.1]	(1.1-1.21]	(1.21-1.45]	(1.45-2.25]	2.25+

	1	2	3	4	5	6	7	8	9	10
Timor-Leste	Iron [1.83]	Vit A [1.08]	PEM [4.87]	Neonatal [0.7]	Diarrhea [1.02]	Congenital [1.07]	URI [0.96]	Hemog [1.58]	Oth NTD [1.68]	Urticaria [0.85]
Vietnam	Neonatal [0.61]	Congenital [0.91]	Vit A [0.69]	Diarrhea [0.9]	Iron [0.76]	PEM [4.41]	URI [0.78]	Urticaria [0.82]	Epilepsy [0.78]	Scabies [2.1]
**Sub-Saharan Africa**	Iron [1.63]	Neonatal [1.42]	Vit A [1.09]	Diarrhea [1.05]	Congenital [1.19]	PEM [1.17]	Hemog [1.85]	Malaria [19.1]	Epilepsy [1.14]	Oth NTD [1.25]
**Central Sub-Saharan Africa**	Neonatal [1.76]	Vit A [1.42]	Iron [1.45]	Diarrhea [1.41]	Congenital [1.18]	PEM [1.23]	Malaria [36.65]	Epilepsy [1.71]	Hemog [2.28]	URI [1.29]
Angola	Iron [2.04]	Neonatal [1.82]	Vit A [1.11]	Diarrhea [1.46]	Congenital [1.19]	Epilepsy [1.99]	Hemog [2.3]	URI [1.4]	Oth NTD [1.65]	PEM [0.68]
C African Rep	Neonatal [1.64]	Vit A [0.98]	Iron [1.24]	Diarrhea [1.37]	Congenital [1.2]	Malaria [4.51]	PEM [0.69]	Epilepsy [1.8]	URI [1.71]	Hemog [1.37]
Congo	Vit A [2.74]	Neonatal [2.07]	Iron [1.83]	Diarrhea [1.58]	PEM [5.4]	Congenital [1.02]	Epilepsy [2.14]	URI [1.27]	Malaria [358.15]	Hemog [1.96]
Congo DR	Vit A [1.12]	Neonatal [1.57]	Iron [1.06]	Diarrhea [1.2]	Congenital [1.16]	PEM [0.85]	Malaria [7.43]	Hemog [1.85]	Epilepsy [1.5]	URI [1.28]
Eq Guinea	Neonatal [2.12]	Diarrhea [1.8]	Vit A [1.14]	Iron [1.21]	Congenital [0.98]	Epilepsy [2.5]	Malaria [965.79]	URI [1.31]	Oth Neuro [3.53]	Urticaria [0.82]
Gabon	Neonatal [2.21]	Iron [3.38]	Vit A [1.78]	Diarrhea [1.94]	Congenital [0.93]	Epilepsy [2.62]	URI [1.34]	Malaria [1818.02]	Hemog [3.21]	Oth Neuro [3.69]
**Eastern Sub-Saharan Africa**	Vit A [1.0]	Neonatal [1.28]	Iron [1.2]	Diarrhea [0.96]	Congenital [1.2]	PEM [0.65]	Epilepsy [1.04]	URI [0.96]	Hemog [0.98]	Urticaria [0.92]
Burundi	Iron [1.7]	Neonatal [1.21]	Vit A [0.47]	Diarrhea [0.81]	Congenital [1.17]	PEM [0.78]	Malaria [1.75]	Oth NTD [1.03]	Epilepsy [1.12]	Oth Un Inf [1.67]
Comoros	Neonatal [1.43]	Vit A [0.82]	Iron [0.99]	Diarrhea [1.08]	Congenital [1.07]	PEM [1.05]	URI [0.98]	Epilepsy [1.03]	Urticaria [0.89]	ASD [1.02]
Djibouti	Neonatal [1.47]	Iron [1.13]	PEM [3.06]	Congenital [1.06]	Diarrhea [0.74]	Vit A [0.35]	Epilepsy [1.14]	URI [0.95]	Urticaria [0.87]	ASD [1.02]
Eritrea	Neonatal [1.4]	Vit A [0.9]	Iron [0.85]	Diarrhea [0.98]	Congenital [1.14]	PEM [1.34]	Epilepsy [1.07]	URI [0.96]	Urticaria [0.91]	ASD [1.04]
Ethiopia	Vit A [1.33]	Iron [1.15]	Neonatal [1.06]	Diarrhea [0.86]	Congenital [1.11]	PEM [0.84]	Oth NTD [0.73]	Urticaria [0.95]	Hemog [0.83]	ASD [1.04]
Kenya	Neonatal [1.43]	Vit A [1.21]	Diarrhea [1.42]	Congenital [1.36]	Iron [0.4]	URI [1.12]	PEM [0.8]	Urticaria [0.87]	Epilepsy [0.87]	Hemog [1.24]
Baringo	Vit A [1.4]	Neonatal [1.29]	Diarrhea [1.48]	Congenital [1.36]	Iron [0.39]	PEM [0.82]	URI [1.13]	Hemog [1.44]	Urticaria [0.9]	ASD [1.02]
Bomet	Neonatal [1.48]	Diarrhea [1.28]	Vit A [0.65]	Congenital [1.27]	URI [1.12]	Urticaria [0.88]	HIV [42.92]	Epilepsy [0.79]	ASD [1.01]	Hearing [3.28]
Bungoma	Vit A [1.24]	Neonatal [1.33]	Diarrhea [1.56]	Congenital [1.34]	Iron [0.44]	PEM [1.0]	URI [1.13]	Hemog [1.27]	Urticaria [0.89]	ASD [1.01]
Busia	Vit A [1.68]	Neonatal [1.33]	Diarrhea [1.48]	Congenital [1.38]	Iron [0.57]	Malaria [25.38]	Hemog [1.79]	URI [1.13]	Urticaria [0.9]	Epilepsy [0.79]
Elgeyo-Marakwet	Neonatal [1.4]	Vit A [1.24]	Diarrhea [1.46]	Congenital [1.32]	Iron [0.58]	URI [1.11]	Urticaria [0.88]	Hemog [1.3]	Epilepsy [0.82]	ASD [1.0]
Embu	Neonatal [1.54]	Diarrhea [1.68]	Vit A [0.83]	Congenital [1.31]	Iron [0.28]	URI [1.11]	Urticaria [0.86]	Epilepsy [0.91]	ASD [0.98]	Hearing [3.47]
Garissa	Vit A [1.22]	Neonatal [1.14]	Congenital [1.31]	Diarrhea [0.85]	Iron [0.53]	Hemog [1.21]	URI [1.19]	PEM [0.33]	Urticaria [0.95]	Epilepsy [0.73]
HomaBay	Neonatal [1.24]	Vit A [0.7]	Diarrhea [1.57]	Congenital [1.48]	HIV [56.21]	PEM [0.74]	URI [1.15]	Epilepsy [0.9]	Urticaria [0.92]	ASD [1.03]
Isiolo	Vit A [0.88]	Neonatal [1.22]	Diarrhea [1.17]	Congenital [1.43]	PEM [0.87]	Iron [0.21]	URI [1.17]	Urticaria [0.94]	Hemog [0.97]	Epilepsy [0.79]
Kajiado	Neonatal [1.56]	Vit A [1.4]	Iron [0.88]	Congenital [1.3]	Diarrhea [1.18]	URI [1.11]	Hemog [1.59]	Urticaria [0.86]	Epilepsy [0.92]	ASD [0.98]
Kakamega	Vit A [1.46]	Neonatal [1.31]	Diarrhea [1.56]	Congenital [1.39]	Iron [0.7]	Hemog [1.69]	PEM [0.66]	URI [1.13]	Urticaria [0.9]	Epilepsy [0.8]
Kericho	Neonatal [1.53]	Diarrhea [1.63]	Vit A [0.85]	Congenital [1.27]	Iron [0.23]	URI [1.11]	Urticaria [0.88]	Epilepsy [0.8]	ASD [1.0]	Hearing [3.3]
Kiambu	Neonatal [1.63]	Diarrhea [1.61]	Congenital [1.31]	Vit A [0.67]	Iron [0.34]	URI [1.09]	Epilepsy [0.99]	Urticaria [0.84]	ASD [0.95]	Hearing [3.74]
Kilifi	Neonatal [1.31]	Vit A [1.04]	Diarrhea [1.39]	Congenital [1.37]	Iron [0.65]	URI [1.13]	Hemog [1.22]	Urticaria [0.9]	ASD [1.02]	Epilepsy [0.76]

eFigure 9b. Leading ten causes of YLDs with the ratio of observed YLDs to YLDs expected on the basis of Socio-Demographic Index alone in 2017, <1 years, both sexes combined.
The top ten causes contributing to YLDs are listed globally, by socio-demographic quintile, and then by GBD superregion, region, country, and subnationally where modeled. For each cell, the ratio of observed YLDs to YLDs expected on the basis of socio-demographic index (SDI) alone are listed. Abbreviations: YLD=year of life lived with disability, GBD=Global Burden of Disease.

Values shown in brackets represent the ratio of observed YLDs to predicted YLDs on the basis of Socio-Demographic Index (SDI), rounded to two (2) digits. Color ranges (shown below) were calculated to place a roughly equal number of cells into each bin.

COLOR KEY:	[0.0-0.68]	(0.68-0.82]	(0.82-0.91]	(0.91-1.01]	(1.01-1.1]	(1.1-1.21]	(1.21-1.45]	(1.45-2.25]	2.25+

	1	2	3	4	5	6	7	8	9	10
Kirinyaga	Neonatal [1.59]	Diarrhea [1.89]	Congenital [1.33]	Vit A [0.37]	URI [1.12]	Urticaria [0.86]	Epilepsy [0.89]	ASD [0.99]	PEM [0.76]	Hearing [3.49]
Kisii	Neonatal [1.39]	Vit A [1.26]	Diarrhea [1.75]	Congenital [1.35]	Iron [0.32]	URI [1.11]	PEM [0.79]	Urticaria [0.87]	Epilepsy [0.83]	Hemog [1.35]
Kisumu	Vit A [2.34]	Neonatal [1.33]	Diarrhea [1.29]	Congenital [1.44]	Iron [0.75]	Hemog [3.3]	URI [1.1]	HIV [58.63]	Epilepsy [1.0]	Urticaria [0.87]
Kitui	Neonatal [1.41]	Vit A [0.87]	Diarrhea [1.37]	Congenital [1.32]	Iron [0.31]	URI [1.13]	Urticaria [0.9]	Epilepsy [0.8]	ASD [1.01]	PEM [0.42]
Kwale	Vit A [1.33]	Neonatal [1.36]	Iron [0.8]	Diarrhea [1.17]	Congenital [1.34]	URI [1.13]	Hemog [1.49]	Urticaria [0.89]	Malaria [16.01]	Epilepsy [0.82]
Laikipia	Neonatal [1.59]	Diarrhea [1.65]	Vit A [0.84]	Congenital [1.27]	URI [1.11]	Urticaria [0.86]	Epilepsy [0.88]	ASD [0.98]	HIV [47.83]	Iron [0.12]
Lamu	Vit A [1.16]	Neonatal [1.33]	Diarrhea [1.42]	Congenital [1.38]	Iron [0.25]	URI [1.13]	Epilepsy [0.93]	Urticaria [0.9]	Hemog [1.16]	ASD [1.02]
Machakos	Neonatal [1.48]	Vit A [1.06]	Diarrhea [1.8]	Congenital [1.3]	Iron [0.66]	URI [1.11]	PEM [0.77]	Urticaria [0.86]	Epilepsy [0.87]	ASD [0.99]
Makueni	Neonatal [1.38]	Congenital [1.33]	Diarrhea [1.09]	Vit A [0.53]	URI [1.13]	HIV [50.23]	Urticaria [0.89]	Epilepsy [0.82]	ASD [1.02]	Hearing [3.16]
Mandera	Vit A [0.68]	Neonatal [1.05]	Congenital [1.27]	Diarrhea [0.8]	Iron [0.41]	PEM [0.61]	URI [1.2]	Urticaria [0.96]	Hemog [0.73]	ASD [1.03]
Marsabit	Neonatal [1.2]	Vit A [0.56]	PEM [1.67]	Diarrhea [1.09]	Congenital [1.33]	URI [1.19]	Urticaria [0.96]	ASD [1.05]	Epilepsy [0.71]	Iron [0.09]
Meru	Neonatal [1.59]	Vit A [1.07]	Diarrhea [1.85]	Congenital [1.32]	URI [1.11]	Urticaria [0.87]	Epilepsy [0.88]	ASD [1.0]	Iron [0.12]	Hemog [0.96]
Migori	Vit A [1.5]	Neonatal [1.21]	Diarrhea [1.46]	Congenital [1.46]	Iron [0.28]	HIV [43.74]	PEM [0.64]	Hemog [1.59]	URI [1.14]	Urticaria [0.91]
Mombasa	Neonatal [1.51]	Vit A [1.45]	Iron [1.46]	Diarrhea [1.77]	Congenital [1.32]	URI [1.09]	Hemog [1.98]	Epilepsy [1.0]	Urticaria [0.85]	ASD [0.95]
Murang'a	Neonatal [1.56]	Congenital [1.31]	Diarrhea [1.19]	Vit A [0.32]	URI [1.2]	Urticaria [0.87]	Epilepsy [0.87]	ASD [0.99]	Hearing [3.43]	PEM [0.5]
Nairobi	Neonatal [1.78]	Congenital [1.22]	Diarrhea [1.47]	Vit A [1.04]	URI [1.02]	Epilepsy [1.24]	Urticaria [0.86]	ASD [0.86]	Iron [0.27]	Hearing [4.42]
Nakuru	Neonatal [1.84]	Diarrhea [1.68]	Vit A [0.77]	Congenital [1.32]	Epilepsy [1.67]	URI [1.11]	Iron [0.23]	Urticaria [0.86]	ASD [0.98]	Oth Neuro [2.36]
Nandi	Neonatal [1.44]	Vit A [1.15]	Diarrhea [1.36]	Congenital [1.3]	URI [1.12]	PEM [0.7]	Iron [0.15]	Urticaria [0.88]	Epilepsy [0.83]	ASD [1.0]
Narok	Vit A [1.23]	Neonatal [1.3]	Diarrhea [0.98]	Congenital [1.3]	Iron [0.39]	URI [1.16]	Hemog [1.21]	Urticaria [0.92]	Epilepsy [0.79]	ASD [1.04]
Nyamira	Neonatal [1.52]	Diarrhea [1.66]	Congenital [1.34]	Vit A [0.5]	URI [1.11]	PEM [1.13]	Epilepsy [0.98]	Urticaria [0.86]	ASD [0.99]	Hearing [3.55]
Nyandarua	Neonatal [1.66]	Diarrhea [1.61]	Congenital [1.31]	Vit A [0.52]	PEM [1.74]	URI [1.11]	Epilepsy [1.01]	Urticaria [0.86]	ASD [0.99]	Whooping [1.56]
Nyeri	Neonatal [1.61]	Diarrhea [1.45]	Congenital [1.33]	URI [1.11]	Vit A [0.22]	Epilepsy [1.05]	Urticaria [0.86]	ASD [0.98]	Hearing [3.58]	Whooping [1.34]
Samburu	Neonatal [1.13]	Vit A [0.64]	Diarrhea [1.07]	Congenital [1.31]	PEM [0.4]	URI [1.2]	Urticaria [0.97]	Iron [0.1]	ASD [1.04]	Hemog [0.65]
Siaya	Vit A [1.34]	Neonatal [1.26]	Diarrhea [1.51]	Congenital [1.4]	Iron [0.7]	Malaria [29.43]	URI [1.13]	Hemog [1.52]	Urticaria [0.89]	Epilepsy [0.79]
TaitaTaveta	Neonatal [1.48]	Diarrhea [1.42]	Congenital [1.35]	Vit A [0.58]	URI [1.12]	Epilepsy [0.99]	Urticaria [0.87]	ASD [0.99]	Hearing [3.45]	HIV [35.18]
TanaRiver	Vit A [1.19]	Neonatal [1.25]	Diarrhea [1.19]	Iron [0.72]	Congenital [1.36]	PEM [0.85]	Hemog [1.39]	URI [1.16]	Urticaria [0.94]	Epilepsy [0.78]
TharakaNithi	Neonatal [1.45]	Vit A [1.23]	Diarrhea [2.0]	Congenital [1.4]	URI [1.11]	Epilepsy [1.02]	Iron [0.17]	Urticaria [0.86]	ASD [0.99]	Hemog [1.24]
TransNzoia	Neonatal [1.41]	Diarrhea [1.68]	Vit A [0.62]	Congenital [1.31]	URI [1.12]	Urticaria [0.88]	Epilepsy [0.79]	ASD [1.0]	Hearing [3.26]	HIV [26.63]
Turkana	Vit A [0.84]	Neonatal [1.09]	PEM [1.33]	Diarrhea [0.99]	Congenital [1.35]	Iron [0.27]	URI [1.19]	Hemog [0.89]	Urticaria [0.96]	HIV [9.91]
UasinGishu	Neonatal [1.52]	Vit A [1.03]	Diarrhea [1.7]	Congenital [1.28]	Iron [0.42]	URI [1.11]	Urticaria [0.86]	Epilepsy [0.85]	ASD [0.98]	Hemog [1.05]
Vihiga	Neonatal [1.43]	Vit A [0.87]	Diarrhea [1.31]	Congenital [1.35]	URI [1.13]	Iron [0.15]	Urticaria [0.89]	Epilepsy [0.85]	ASD [1.02]	Malaria [21.25]

eFigure 9b. Leading ten causes of YLDs with the ratio of observed YLDs to YLDs expected on the basis of Socio-Demographic Index alone in 2017, <1 years, both sexes combined.
The top ten causes contributing to YLDs are listed globally, by socio-demographic quintile, and then by GBD superregion, region, country, and subnationally where modeled. For each cell, the ratio of observed YLDs to YLDs expected on the basis of socio-demographic index (SDI) alone are listed. Abbreviations: YLD=year of life lived with disability, GBD=Global Burden of Disease.

Values shown in brackets represent the ratio of observed YLDs to predicted YLDs on the basis of Socio-Demographic Index (SDI), rounded to two (2) digits. Color ranges (shown below) were calculated to place a roughly equal number of cells into each bin.

COLOR KEY:	[0.0-0.68]	(0.68-0.82]	(0.82-0.91]	(0.91-1.01]	(1.01-1.1]	(1.1-1.21]	(1.21-1.45]	(1.45-2.25]	2.25+

	1	2	3	4	5	6	7	8	9	10
Wajir	Neonatal [1.0]	Vit A [0.56]	Diarrhea [0.81]	Congenital [1.27]	PEM [0.24]	URI [1.21]	Iron [0.12]	Urticaria [0.97]	ASD [1.01]	Hemog [0.55]
WestPokot	Vit A [1.25]	Neonatal [1.22]	Diarrhea [1.34]	Congenital [1.34]	Iron [0.43]	URI [1.16]	Hemog [1.24]	PEM [0.34]	Urticaria [0.93]	ASD [1.04]
Madagascar	Neonatal [1.34]	Iron [1.1]	Vit A [0.56]	Congenital [1.92]	Diarrhea [1.12]	Epilepsy [1.19]	Hearing [5.15]	URI [0.97]	Urticaria [0.94]	Oth NTD [0.7]
Malawi	Iron [1.86]	Vit A [0.98]	Neonatal [1.28]	Diarrhea [1.26]	Congenital [1.18]	Malaria [3.66]	Oth NTD [1.17]	HIV [25.86]	URI [1.12]	Epilepsy [1.0]
Mozambique	Vit A [0.97]	Neonatal [1.35]	Iron [1.17]	Congenital [1.15]	Diarrhea [0.65]	Malaria [4.45]	HIV [31.29]	Epilepsy [1.53]	Oth NTD [0.72]	Urticaria [0.94]
Rwanda	Neonatal [1.32]	Iron [1.28]	Vit A [0.78]	Diarrhea [0.88]	Congenital [1.15]	Epilepsy [1.17]	URI [0.98]	Oth NTD [0.86]	Urticaria [0.91]	PEM [0.35]
Somalia	Vit A [0.83]	Neonatal [1.03]	Iron [0.92]	PEM [0.93]	Diarrhea [0.66]	Congenital [1.07]	Epilepsy [0.84]	URI [1.0]	Urticaria [0.94]	ASD [0.99]
S Sudan	Vit A [0.81]	Neonatal [1.12]	Iron [1.11]	Diarrhea [1.19]	PEM [1.22]	Congenital [1.02]	Epilepsy [1.0]	Malaria [1.07]	URI [0.99]	Oth NTD [0.7]
Tanzania	Iron [1.93]	Neonatal [1.35]	Vit A [0.78]	Congenital [1.01]	Diarrhea [0.7]	Hemog [1.65]	Oth NTD [1.32]	Epilepsy [1.18]	URI [1.06]	PEM [0.4]
Uganda	Neonatal [1.37]	Iron [0.97]	Vit A [0.57]	Diarrhea [0.96]	Congenital [1.07]	Malaria [10.46]	Epilepsy [1.27]	URI [1.23]	Hemog [1.14]	Urticaria [0.92]
Zambia	Neonatal [1.51]	Vit A [1.0]	Iron [1.07]	Diarrhea [1.13]	Congenital [1.13]	Epilepsy [1.41]	URI [1.08]	Malaria [28.02]	PEM [0.61]	HIV [40.74]
**Southern Sub-Saharan Africa**	Neonatal [1.48]	Iron [1.38]	Vit A [1.07]	Congenital [1.07]	Diarrhea [0.93]	URI [0.94]	Epilepsy [1.06]	Urticaria [0.84]	ASD [0.89]	PEM [2.8]
Botswana	Neonatal [1.56]	Iron [2.14]	Vit A [1.26]	Diarrhea [1.15]	Congenital [0.82]	PEM [10.71]	URI [0.92]	Epilepsy [1.12]	Urticaria [0.84]	ASD [0.86]
Lesotho	Neonatal [1.26]	Vit A [0.92]	Iron [0.82]	Diarrhea [1.07]	Congenital [0.97]	HIV [83.66]	URI [1.0]	Epilepsy [0.96]	Urticaria [0.87]	ASD [1.0]
Namibia	Iron [2.97]	Neonatal [1.46]	Diarrhea [1.5]	Congenital [0.86]	Vit A [0.62]	PEM [4.71]	URI [1.04]	Epilepsy [1.06]	Oth NTD [6.97]	Urticaria [0.82]
S Africa	Neonatal [1.55]	Congenital [1.19]	Vit A [1.0]	Iron [0.61]	Diarrhea [0.59]	URI [0.93]	Epilepsy [1.2]	Urticaria [0.87]	ASD [0.86]	PEM [6.72]
Swaziland	Neonatal [1.31]	Iron [1.99]	Diarrhea [1.53]	Vit A [0.86]	Congenital [0.91]	HIV [125.48]	URI [0.91]	Epilepsy [0.95]	Urticaria [0.83]	ASD [0.95]
Zimbabwe	Iron [1.44]	Neonatal [1.24]	Vit A [0.7]	Diarrhea [1.12]	Congenital [0.88]	URI [1.0]	Urticaria [0.88]	Oth NTD [1.14]	Epilepsy [0.76]	ASD [1.01]
**Western Sub-Saharan Africa**	Iron [2.0]	Neonatal [1.39]	Vit A [0.94]	Diarrhea [0.99]	Congenital [1.17]	PEM [1.43]	Hemog [2.47]	Malaria [22.17]	Oth NTD [1.49]	Epilepsy [1.07]
Benin	Vit A [0.98]	Neonatal [1.32]	Iron [0.82]	Congenital [1.05]	Diarrhea [0.65]	Malaria [9.81]	PEM [0.65]	Hemog [1.59]	Epilepsy [1.17]	Urticaria [0.92]
Burkina Faso	Iron [2.28]	Vit A [0.87]	Neonatal [1.15]	PEM [1.76]	Diarrhea [0.74]	Congenital [1.03]	Malaria [2.89]	Hemog [1.98]	Oth NTD [1.39]	Oth Un Inf [2.13]
Cameroon	Neonatal [1.48]	Iron [1.48]	Vit A [1.06]	Diarrhea [1.29]	Congenital [1.07]	Malaria [56.49]	Epilepsy [1.31]	Hemog [1.84]	URI [0.97]	PEM [0.63]
Cape Verde	Neonatal [1.67]	Iron [1.3]	Congenital [0.92]	Diarrhea [0.74]	Epilepsy [1.23]	Urticaria [0.85]	URI [0.74]	ASD [0.97]	Hemog [1.07]	Oth NTD [1.37]
Chad	Vit A [0.72]	Neonatal [1.07]	Iron [1.12]	Diarrhea [1.27]	PEM [0.98]	Congenital [0.98]	Epilepsy [0.97]	Hemog [0.76]	Malaria [0.86]	URI [0.91]
Cote d'Ivoire	Iron [2.53]	Vit A [1.06]	Neonatal [1.39]	Diarrhea [1.01]	Congenital [1.06]	Hemog [2.85]	Oth NTD [1.72]	Epilepsy [1.23]	Oth Un Inf [2.59]	Malaria [8.92]
Gambia	Iron [1.89]	Neonatal [1.5]	Vit A [1.02]	Diarrhea [0.99]	Congenital [0.99]	PEM [1.37]	Hemog [1.78]	Oth NTD [1.3]	Oth Un Inf [1.95]	Urticaria [0.9]
Ghana	Neonatal [1.56]	Vit A [1.66]	Iron [1.77]	Diarrhea [1.1]	Congenital [1.02]	PEM [2.04]	Epilepsy [1.31]	Malaria [166.49]	Hemog [2.12]	Urticaria [0.84]
Guinea	Iron [1.66]	Neonatal [1.22]	Vit A [0.68]	Congenital [1.06]	Diarrhea [0.71]	PEM [0.67]	Malaria [3.73]	Hemog [2.01]	Oth NTD [1.02]	Epilepsy [1.0]
Guinea-Bissau	Vit A [1.18]	Neonatal [1.35]	Iron [0.88]	Congenital [1.13]	Diarrhea [0.78]	Epilepsy [1.1]	PEM [0.32]	Urticaria [0.93]	URI [0.79]	ASD [1.04]
Liberia	Neonatal [1.39]	Iron [1.34]	Vit A [0.49]	Diarrhea [1.03]	Congenital [1.09]	Hemog [1.98]	Epilepsy [1.46]	Malaria [2.43]	PEM [0.37]	Oth NTD [0.82]
Mali	Iron [1.68]	Vit A [0.78]	Neonatal [1.07]	Diarrhea [0.68]	Congenital [1.0]	PEM [0.62]	Malaria [1.74]	Hemog [1.02]	Oth NTD [1.01]	Oth Un Inf [1.52]

eFigure 9b. Leading ten causes of YLDs with the ratio of observed YLDs to YLDs expected on the basis of Socio-Demographic Index alone in 2017, <1 years, both sexes combined.
The top ten causes contributing to YLDs are listed globally, by socio-demographic quintile, and then by GBD superregion, region, country, and subnationally where modeled. For each cell, the ratio of observed YLDs to YLDs expected on the basis of socio-demographic index (SDI) alone are listed. Abbreviations: YLD=year of life lived with disability, GBD=Global Burden of Disease.

Values shown in brackets represent the ratio of observed YLDs to predicted YLDs on the basis of Socio-Demographic Index (SDI), rounded to two (2) digits. Color ranges (shown below) were calculated to place a roughly equal number of cells into each bin.

COLOR KEY:	[0.0-0.68]	(0.68-0.82]	(0.82-0.91]	(0.91-1.01]	(1.01-1.1]	(1.1-1.21]	(1.21-1.45]	(1.45-2.25]	2.25+

	1	2	3	4	5	6	7	8	9	10
Mauritania	**PEM** [7.56]	**Neonatal** [1.57]	**Iron** [1.52]	**Vit A** [0.76]	**Diarrhea** [1.02]	**Congenital** [0.98]	**Epilepsy** [1.24]	**Oth NTD** [1.25]	**Urticaria** [0.87]	**Hemog** [1.18]
Niger	**Vit A** [0.79]	**Neonatal** [0.92]	**Iron** [0.78]	**PEM** [0.84]	**Diarrhea** [0.68]	**Congenital** [0.92]	**Malaria** [1.69]	**Hemog** [1.05]	**Epilepsy** [0.68]	**Urticaria** [0.93]
Nigeria	**Iron** [2.6]	**Neonatal** [1.44]	**Vit A** [0.82]	**Congenital** [1.29]	**Diarrhea** [0.94]	**PEM** [1.67]	**Hemog** [3.28]	**Malaria** [71.61]	**Oth NTD** [2.24]	**Epilepsy** [1.07]
Sao Tome Principe	**Neonatal** [1.49]	**Vit A** [1.32]	**Iron** [0.97]	**Diarrhea** [1.0]	**Congenital** [1.02]	**PEM** [0.97]	**Hemog** [1.86]	**Epilepsy** [1.21]	**Urticaria** [0.86]	**URI** [0.78]
Senegal	**Iron** [1.75]	**Neonatal** [1.39]	**Vit A** [0.82]	**Diarrhea** [1.08]	**Congenital** [1.02]	**PEM** [0.45]	**Hemog** [1.38]	**Oth NTD** [1.12]	**Epilepsy** [1.07]	**Oth Un Inf** [1.84]
Sierra Leone	**Iron** [2.05]	**Neonatal** [1.33]	**Vit A** [0.79]	**Congenital** [1.05]	**Malaria** [8.96]	**Diarrhea** [0.66]	**Hemog** [2.92]	**PEM** [0.67]	**Oth NTD** [1.26]	**Epilepsy** [1.29]
Togo	**Iron** [1.93]	**Neonatal** [1.51]	**Vit A** [0.96]	**Diarrhea** [1.13]	**Congenital** [1.06]	**Malaria** [29.5]	**Hemog** [2.33]	**PEM** [0.81]	**Epilepsy** [1.3]	**Oth NTD** [1.35]

eFigure 9c. Leading ten causes of YLDs with the ratio of observed YLDs to YLDs expected on the basis of Socio-Demographic Index alone in 2017, 1-4 years, both sexes combined.
The top ten causes contributing to YLDs are listed globally, by socio-demographic quintile, and then by GBD superregion, region, country, and subnationally where modeled. For each cell, the ratio of observed YLDs to YLDs expected on the basis of socio-demographic index (SDI) alone are listed. Abbreviations: YLD=year of life lived with disability, GBD=Global Burden of Disease.

Values shown in brackets represent the ratio of observed YLDs to predicted YLDs on the basis of Socio-Demographic Index (SDI), rounded to two (2) digits. Color ranges (shown below) were calculated to place a roughly equal number of cells into each bin.

COLOR KEY:									
[0.0-0.72]	(0.72-0.84]	(0.84-0.92]	(0.92-1.01]	(1.01-1.09]	(1.09-1.17]	(1.17-1.32]	(1.32-1.67]	1.67+	

	1	2	3	4	5	6	7	8	9	10
Global	Neonatal [1.13]	Iron [2.88]	Vit A [1.51]	Dermatitis [0.74]	Diarrhea [1.2]	Asthma [0.7]	Congenital [0.97]	PEM [127.58]	Urticaria [0.88]	Skin Viral [0.86]
Low SDI	Iron [1.16]	Neonatal [1.29]	Vit A [0.84]	Diarrhea [0.85]	Dermatitis [0.81]	PEM [3.4]	Congenital [1.04]	Asthma [0.6]	Urticaria [0.91]	Epilepsy [0.93]
Low-middle SDI	Iron [1.65]	Neonatal [1.14]	Vit A [1.03]	Diarrhea [1.07]	Dermatitis [0.73]	PEM [17.92]	Congenital [1.04]	Asthma [0.6]	Urticaria [0.89]	Skin Viral [0.87]
Middle SDI	Neonatal [1.18]	Dermatitis [0.69]	Asthma [0.86]	Iron [1.37]	Diarrhea [1.04]	Vit A [0.92]	Congenital [0.88]	PEM [114.95]	Urticaria [0.9]	Skin Viral [0.89]
High-middle SDI	Neonatal [1.43]	Dermatitis [0.57]	Diarrhea [1.92]	Asthma [1.03]	Congenital [0.92]	Urticaria [1.35]	Skin Viral [0.97]	Vit A [2.61]	URI [0.75]	Iron [6.47]
High SDI	Dermatitis [1.32]	Neonatal [0.86]	Asthma [0.91]	Congenital [0.88]	URI [1.18]	Skin Viral [0.9]	Diarrhea [0.73]	Urticaria [0.74]	ASD [1.15]	Epilepsy [0.8]
Central Europe, Eastern Europe, and Central Asia	Neonatal [1.35]	Dermatitis [0.54]	Diarrhea [1.29]	Congenital [1.03]	Iron [3.69]	Urticaria [1.2]	Skin Viral [0.83]	Vit A [1.16]	Asthma [0.47]	Hernia [2.46]
Central Asia	Neonatal [0.97]	Iron [2.76]	Vit A [1.26]	Dermatitis [0.49]	Congenital [1.01]	Urticaria [1.02]	Diarrhea [0.69]	Skin Viral [0.83]	Hernia [2.89]	Asthma [0.43]
Armenia	Iron [5.43]	Neonatal [0.97]	Dermatitis [0.48]	Diarrhea [1.05]	Congenital [1.0]	Urticaria [1.05]	Skin Viral [0.83]	Hernia [2.77]	Asthma [0.42]	Epilepsy [1.06]
Azerbaijan	Neonatal [0.99]	Iron [1.86]	Dermatitis [0.48]	Congenital [0.98]	Diarrhea [0.85]	Urticaria [1.06]	Vit A [0.88]	Skin Viral [0.83]	Asthma [0.42]	Epilepsy [1.2]
Georgia	Neonatal [0.96]	Congenital [1.02]	Dermatitis [0.44]	Diarrhea [0.98]	Urticaria [1.06]	Vit A [0.83]	Skin Viral [0.83]	Epilepsy [1.12]	Asthma [0.39]	Iron [0.8]
Kazakhstan	Neonatal [1.05]	Vit A [2.49]	Congenital [1.04]	Dermatitis [0.45]	Urticaria [1.12]	Diarrhea [0.77]	Skin Viral [0.83]	Epilepsy [1.33]	Asthma [0.37]	Blindness [1.3]
Kyrgyzstan	Neonatal [0.98]	Iron [2.03]	Vit A [0.92]	Hernia [6.25]	Dermatitis [0.54]	Congenital [1.04]	Urticaria [0.96]	Asthma [0.46]	Skin Viral [0.86]	Diarrhea [0.48]
Mongolia	Neonatal [1.0]	Vit A [1.44]	Diarrhea [1.03]	Dermatitis [0.5]	Congenital [1.04]	Urticaria [1.01]	Iron [0.97]	Skin Viral [0.84]	Asthma [0.42]	Epilepsy [1.13]
Tajikistan	Neonatal [1.01]	Iron [0.75]	Vit A [0.58]	Diarrhea [0.86]	Congenital [1.05]	Dermatitis [0.56]	Urticaria [0.97]	Hernia [4.54]	PEM [7.88]	Skin Viral [0.89]
Turkmenistan	Neonatal [0.92]	Dermatitis [0.48]	Congenital [1.01]	Iron [1.48]	Urticaria [1.05]	Vit A [0.72]	Skin Viral [0.83]	Asthma [0.42]	Epilepsy [1.14]	Hernia [2.07]
Uzbekistan	Iron [3.11]	Neonatal [0.92]	Vit A [0.98]	Dermatitis [0.51]	Congenital [1.0]	Urticaria [0.97]	Asthma [0.48]	Hernia [3.06]	Skin Viral [0.84]	Epilepsy [1.02]
Central Europe	Neonatal [1.41]	Diarrhea [1.99]	Dermatitis [0.59]	Congenital [1.17]	Urticaria [1.31]	Asthma [0.58]	Vit A [2.15]	Skin Viral [0.85]	Hernia [2.94]	Falls [1.83]
Albania	Neonatal [1.18]	Diarrhea [1.13]	Congenital [1.1]	Dermatitis [0.46]	Urticaria [1.03]	Vit A [0.81]	Hernia [3.63]	Iron [1.06]	PEM [197.39]	Skin Viral [0.82]
Bosnia	Neonatal [1.26]	Dermatitis [0.55]	Congenital [1.11]	Diarrhea [1.17]	Vit A [1.3]	Urticaria [1.08]	Hernia [3.61]	Asthma [0.53]	Skin Viral [0.83]	Falls [2.86]
Bulgaria	Neonatal [1.25]	Diarrhea [1.76]	Dermatitis [0.49]	Congenital [1.19]	Urticaria [1.24]	Vit A [1.85]	Skin Viral [0.84]	Hernia [2.98]	Falls [1.91]	Asthma [0.47]
Croatia	Neonatal [1.53]	Dermatitis [0.51]	Diarrhea [1.72]	Congenital [1.14]	Urticaria [1.35]	Hernia [3.85]	Skin Viral [0.86]	Asthma [0.56]	Epilepsy [1.1]	ASD [0.81]
Czech	Neonatal [0.99]	Diarrhea [2.14]	Dermatitis [0.57]	Congenital [1.16]	Urticaria [1.44]	Falls [2.02]	Hernia [3.54]	Skin Viral [0.88]	Asthma [0.5]	Vit A [1.83]
Hungary	Neonatal [1.35]	Diarrhea [2.83]	Dermatitis [0.68]	Congenital [1.22]	Urticaria [1.31]	Skin Viral [0.85]	Falls [1.88]	Asthma [0.46]	Hernia [2.31]	Vit A [1.46]
Macedonia	Neonatal [1.27]	Diarrhea [1.5]	Vit A [2.05]	Dermatitis [0.51]	Congenital [1.09]	Urticaria [1.15]	Iron [2.32]	Hernia [3.34]	Asthma [0.57]	Skin Viral [0.83]
Montenegro	Neonatal [1.3]	Dermatitis [0.53]	Congenital [1.11]	Urticaria [1.24]	Diarrhea [0.95]	Vit A [1.75]	Hernia [3.23]	Skin Viral [0.84]	Asthma [0.51]	Falls [2.01]
Poland	Neonatal [1.6]	Dermatitis [0.66]	Diarrhea [1.98]	Congenital [1.17]	Urticaria [1.42]	Asthma [0.76]	Skin Viral [0.87]	Upper Digest [5.85]	Falls [1.64]	Vit A [2.35]
Romania	Neonatal [1.42]	Diarrhea [2.26]	Dermatitis [0.56]	Congenital [1.16]	Iron [4.98]	Vit A [2.16]	Urticaria [1.2]	Asthma [0.61]	Skin Viral [0.84]	Upper Digest [5.25]
Serbia	Neonatal [1.61]	Dermatitis [0.54]	Congenital [1.18]	Diarrhea [1.26]	Vit A [1.4]	Urticaria [1.15]	Hernia [3.5]	Skin Viral [0.83]	Falls [2.33]	Asthma [0.38]
Slovakia	Neonatal [1.09]	Diarrhea [2.38]	Dermatitis [0.56]	Congenital [1.24]	Urticaria [1.4]	Hernia [3.83]	Skin Viral [0.87]	Falls [1.68]	Upper Digest [5.11]	Asthma [0.45]
Slovenia	Neonatal [1.16]	Diarrhea [2.4]	Dermatitis [0.51]	Congenital [1.22]	Urticaria [1.48]	Asthma [0.7]	Falls [1.88]	Hernia [3.22]	Skin Viral [0.89]	Vit A [2.11]

eFigure 9c. Leading ten causes of YLDs with the ratio of observed YLDs to YLDs expected on the basis of Socio-Demographic Index alone in 2017, 1-4 years, both sexes combined.
The top ten causes contributing to YLDs are listed globally, by socio-demographic quintile, and then by GBD superregion, region, country, and subnationally where modeled. For each cell, the ratio of observed YLDs to YLDs expected on the basis of socio-demographic index (SDI) alone are listed. Abbreviations: YLD=year of life lived with disability, GBD=Global Burden of Disease.

Values shown in brackets represent the ratio of observed YLDs to predicted YLDs on the basis of Socio-Demographic Index (SDI), rounded to two (2) digits. Color ranges (shown below) were calculated to place a roughly equal number of cells into each bin.

COLOR KEY:									
	[0.0-0.72]	[0.72-0.84]	[0.84-0.92]	[0.92-1.01]	[1.01-1.09]	[1.09-1.17]	[1.17-1.32]	[1.32-1.67]	1.67+

	1	2	3	4	5	6	7	8	9	10
**Eastern Europe**	Neonatal [1.61]	Dermatitis [0.57]	Diarrhea [1.49]	Congenital [0.99]	Urticaria [1.28]	Upper Digest [5.47]	Skin Viral [0.84]	Iron [2.83]	Asthma [0.44]	Falls [1.64]
Belarus	Neonatal [1.53]	Dermatitis [0.62]	Congenital [0.97]	Urticaria [1.21]	Diarrhea [0.94]	Asthma [0.57]	Skin Viral [0.84]	Upper Digest [5.17]	Hernia [2.14]	Falls [1.64]
Estonia	Neonatal [1.7]	Dermatitis [0.79]	Diarrhea [2.52]	Congenital [1.03]	Urticaria [1.49]	Skin Viral [0.89]	Upper Digest [6.24]	Falls [1.26]	Asthma [0.42]	Hernia [1.92]
Latvia	Neonatal [1.51]	Diarrhea [2.33]	Dermatitis [0.58]	Congenital [0.95]	Urticaria [1.35]	Asthma [0.59]	Skin Viral [0.87]	Upper Digest [5.32]	Falls [1.44]	Hernia [2.1]
Lithuania	Neonatal [1.74]	Diarrhea [2.53]	Congenital [1.1]	Dermatitis [0.36]	Urticaria [1.41]	Upper Digest [6.54]	Skin Viral [0.88]	Asthma [0.5]	Falls [1.33]	Hernia [2.11]
Moldova	Neonatal [1.55]	Dermatitis [0.68]	Diarrhea [0.96]	Congenital [0.96]	Iron [1.3]	Upper Digest [5.6]	Urticaria [1.02]	Hernia [3.27]	Skin Viral [0.83]	Asthma [0.41]
Russian Federation	Neonatal [1.62]	Dermatitis [0.57]	Diarrhea [1.72]	Congenital [0.98]	Urticaria [1.29]	Upper Digest [5.46]	Skin Viral [0.84]	Iron [3.15]	Asthma [0.41]	Falls [1.64]
Ukraine	Neonatal [1.55]	Dermatitis [0.59]	Congenital [1.02]	Urticaria [1.18]	Upper Digest [5.15]	Asthma [0.5]	Skin Viral [0.83]	Iron [1.61]	Diarrhea [0.64]	Hernia [2.5]
**High-income**	Dermatitis [1.31]	Neonatal [0.94]	Asthma [1.12]	Congenital [0.94]	URI [1.16]	Diarrhea [1.14]	Skin Viral [0.96]	ASD [1.15]	Urticaria [0.91]	Epilepsy [1.06]
**Australasia**	Dermatitis [1.14]	Asthma [2.1]	Neonatal [0.92]	Falls [2.11]	Skin Viral [1.0]	Congenital [0.78]	URI [1.02]	Urticaria [1.26]	ASD [1.03]	Diarrhea [0.65]
Australia	Dermatitis [1.08]	Asthma [2.16]	Neonatal [0.9]	Falls [2.02]	Skin Viral [1.0]	Congenital [0.78]	URI [1.02]	Urticaria [1.27]	ASD [1.03]	Diarrhea [0.63]
New Zealand	Dermatitis [1.42]	Neonatal [1.02]	Asthma [1.82]	Falls [2.67]	Congenital [0.78]	URI [1.04]	Urticaria [1.19]	Skin Viral [0.96]	Diarrhea [0.73]	ASD [1.02]
**High-income Asia Pacific**	Dermatitis [1.26]	Neonatal [1.2]	Asthma [1.03]	Congenital [0.99]	Diarrhea [1.21]	URI [1.03]	Skin Viral [0.99]	Urticaria [1.17]	ASD [1.28]	Falls [1.09]
Brunei	Dermatitis [1.2]	Neonatal [1.19]	Asthma [0.98]	Diarrhea [1.2]	Iron [9.85]	Congenital [0.81]	Skin Viral [0.98]	URI [1.02]	Urticaria [1.09]	ASD [1.19]
Japan	Dermatitis [1.32]	Neonatal [1.18]	Asthma [1.02]	Congenital [1.09]	URI [1.04]	Skin Viral [0.99]	Diarrhea [1.13]	Urticaria [1.17]	ASD [1.36]	Falls [1.14]
Aichi	Dermatitis [1.32]	Neonatal [1.21]	Asthma [1.05]	ASD [2.01]	Congenital [1.02]	Diarrhea [1.22]	URI [1.04]	Skin Viral [1.0]	Urticaria [1.21]	Falls [1.1]
Akita	Dermatitis [1.33]	Neonatal [1.25]	Congenital [1.14]	Asthma [0.96]	Diarrhea [1.09]	URI [1.05]	Skin Viral [0.96]	Urticaria [1.06]	ASD [1.32]	Endocrine [3.49]
Aomori	Dermatitis [1.33]	Neonatal [1.16]	Asthma [0.95]	Congenital [0.93]	Diarrhea [1.08]	URI [1.05]	Skin Viral [0.95]	Urticaria [1.05]	ASD [1.33]	Endocrine [3.52]
Chiba	Dermatitis [1.32]	Neonatal [1.16]	Asthma [1.02]	Congenital [1.11]	URI [1.04]	Skin Viral [0.98]	Urticaria [1.15]	Diarrhea [1.01]	ASD [1.34]	Falls [1.15]
Ehime	Dermatitis [1.32]	Neonatal [0.96]	Asthma [0.96]	Congenital [1.0]	URI [1.05]	Skin Viral [0.96]	Diarrhea [0.98]	Urticaria [1.09]	ASD [1.35]	Vit A [2.22]
Fukui	Dermatitis [1.32]	Neonatal [1.15]	Asthma [0.98]	Congenital [0.99]	Diarrhea [1.26]	URI [1.04]	Skin Viral [0.97]	Urticaria [1.12]	ASD [1.35]	Falls [1.18]
Fukuoka	Dermatitis [1.32]	Neonatal [1.14]	Asthma [1.0]	Congenital [1.04]	URI [1.04]	Skin Viral [0.98]	Diarrhea [1.08]	Urticaria [1.14]	ASD [0.99]	Endocrine [3.72]
Fukushima	Dermatitis [1.32]	Neonatal [1.06]	Congenital [1.14]	Asthma [0.95]	Diarrhea [1.14]	URI [1.05]	Skin Viral [0.95]	Urticaria [1.06]	ASD [1.34]	Falls [1.26]
Gifu	Dermatitis [1.32]	Neonatal [1.19]	Asthma [0.99]	Congenital [0.89]	Diarrhea [1.23]	URI [1.04]	Skin Viral [0.97]	Urticaria [1.12]	ASD [1.35]	Endocrine [3.83]
Gunma	Dermatitis [1.32]	Neonatal [1.12]	Asthma [0.99]	Congenital [1.04]	Diarrhea [1.2]	URI [1.05]	Skin Viral [0.97]	Urticaria [1.12]	ASD [1.34]	Endocrine [4.01]
Hiroshima	Dermatitis [1.32]	Neonatal [1.22]	Asthma [1.02]	Congenital [0.98]	Diarrhea [1.23]	URI [1.04]	Skin Viral [0.99]	Urticaria [1.17]	ASD [1.34]	Falls [1.12]
Hokkaido	Dermatitis [1.32]	Neonatal [1.12]	Asthma [0.98]	Congenital [1.07]	Diarrhea [1.17]	URI [1.04]	Skin Viral [0.96]	Urticaria [1.09]	ASD [1.34]	Falls [1.24]
Hyogo	Dermatitis [1.32]	Neonatal [1.18]	Asthma [0.88]	Congenital [0.86]	Diarrhea [1.2]	URI [1.05]	Skin Viral [0.98]	Urticaria [1.15]	ASD [1.35]	Falls [1.14]
Ibaraki	Dermatitis [1.32]	Neonatal [1.21]	Asthma [1.0]	Congenital [1.14]	URI [1.04]	Skin Viral [0.97]	Urticaria [1.12]	Diarrhea [0.93]	ASD [1.08]	Falls [1.18]
Ishikawa	Dermatitis [1.32]	Neonatal [1.12]	Asthma [1.0]	Congenital [1.06]	Diarrhea [1.18]	URI [1.04]	Skin Viral [0.98]	Urticaria [1.14]	ASD [1.34]	Falls [1.17]
Iwate	Dermatitis [1.33]	Neonatal [1.2]	Congenital [1.12]	Asthma [0.94]	Diarrhea [1.08]	URI [1.05]	Skin Viral [0.95]	Urticaria [1.05]	ASD [1.34]	Falls [1.29]

eFigure 9c. Leading ten causes of YLDs with the ratio of observed YLDs to YLDs expected on the basis of Socio-Demographic Index alone in 2017, 1-4 years, both sexes combined.
The top ten causes contributing to YLDs are listed globally, by socio-demographic quintile, and then by GBD superregion, region, country, and subnationally where modeled. For each cell, the ratio of observed YLDs to YLDs expected on the basis of socio-demographic index (SDI) alone are listed. Abbreviations: YLD=year of life lived with disability, GBD=Global Burden of Disease.

Values shown in brackets represent the ratio of observed YLDs to predicted YLDs on the basis of Socio-Demographic Index (SDI), rounded to two (2) digits. Color ranges (shown below) were calculated to place a roughly equal number of cells into each bin.

COLOR KEY:	[0.0-0.72]	(0.72-0.84]	(0.84-0.92]	(0.92-1.01]	(1.01-1.09]	(1.09-1.17]	(1.17-1.32]	(1.32-1.67]	1.67+

	1	2	3	4	5	6	7	8	9	10
Kagawa	Dermatitis [1.32]	Neonatal [1.11]	Asthma [0.98]	Congenital [1.04]	Diarrhea [1.41]	URI [1.05]	Skin Viral [0.97]	Urticaria [1.12]	ASD [1.34]	Falls [1.17]
Kagoshima	Dermatitis [1.33]	Neonatal [0.99]	Congenital [1.18]	Asthma [0.94]	Diarrhea [1.09]	URI [1.05]	Skin Viral [0.95]	Urticaria [1.06]	ASD [1.34]	Falls [1.26]
Kanagawa	Dermatitis [1.32]	Neonatal [1.23]	Congenital [1.22]	Asthma [1.05]	ASD [1.62]	URI [1.04]	Skin Viral [1.0]	Urticaria [1.21]	Diarrhea [0.96]	Falls [1.12]
Kochi	Dermatitis [1.32]	Neonatal [1.06]	Asthma [0.93]	Congenital [1.01]	Diarrhea [1.17]	URI [1.05]	Skin Viral [0.95]	Urticaria [1.05]	ASD [1.35]	Endocrine [3.37]
Kumamoto	Dermatitis [1.33]	Neonatal [1.04]	Asthma [0.94]	Congenital [0.97]	Diarrhea [1.05]	URI [1.05]	Skin Viral [0.95]	Urticaria [1.06]	ASD [1.34]	Endocrine [3.54]
Kyoto	Neonatal [1.23]	Dermatitis [1.09]	Congenital [1.3]	Asthma [1.05]	Diarrhea [1.19]	URI [1.03]	Skin Viral [1.0]	Urticaria [1.21]	ASD [1.35]	Endocrine [3.98]
Mie	Dermatitis [1.32]	Neonatal [1.2]	Asthma [0.99]	Diarrhea [1.39]	Congenital [0.97]	URI [1.04]	Skin Viral [0.98]	Urticaria [1.14]	ASD [1.34]	Endocrine [3.97]
Miyagi	Dermatitis [1.32]	Neonatal [1.11]	Congenital [1.23]	Asthma [0.83]	Diarrhea [1.1]	URI [1.05]	Skin Viral [0.97]	Urticaria [1.12]	ASD [1.34]	Endocrine [3.64]
Miyazaki	Dermatitis [1.33]	Neonatal [1.03]	Asthma [0.94]	Congenital [1.06]	URI [1.05]	Skin Viral [0.95]	Urticaria [1.05]	ASD [1.33]	Diarrhea [0.84]	Falls [1.29]
Nagano	Dermatitis [1.32]	Neonatal [1.22]	Asthma [0.99]	Diarrhea [1.45]	Congenital [1.0]	URI [1.05]	Skin Viral [0.97]	Urticaria [1.12]	ASD [1.34]	Endocrine [3.66]
Nagasaki	Dermatitis [1.33]	Neonatal [1.04]	Congenital [1.26]	Asthma [0.94]	URI [1.05]	Skin Viral [0.95]	Diarrhea [0.94]	Urticaria [1.05]	ASD [1.33]	Endocrine [3.57]
Nara	Dermatitis [1.32]	Neonatal [1.04]	Congenital [1.28]	Asthma [0.99]	Diarrhea [1.13]	URI [1.04]	Skin Viral [0.97]	Urticaria [1.12]	ASD [1.34]	Falls [1.19]
Niigata	Dermatitis [1.32]	Neonatal [1.17]	Asthma [0.98]	Congenital [1.11]	URI [1.04]	Skin Viral [0.97]	Diarrhea [1.05]	Urticaria [1.11]	ASD [1.34]	Falls [1.22]
Oita	Dermatitis [1.32]	Neonatal [1.23]	Asthma [0.97]	Congenital [0.94]	Diarrhea [1.19]	URI [1.05]	Skin Viral [0.96]	Urticaria [1.1]	ASD [1.36]	Falls [1.2]
Okayama	Dermatitis [1.32]	Neonatal [1.23]	Asthma [1.0]	Congenital [0.94]	Diarrhea [1.12]	URI [1.04]	Skin Viral [0.98]	Urticaria [1.13]	ASD [1.34]	Falls [1.16]
Okinawa	Dermatitis [1.33]	Neonatal [1.08]	Asthma [0.8]	Congenital [0.86]	URI [1.06]	Skin Viral [0.95]	Urticaria [1.04]	Diarrhea [0.88]	ASD [1.33]	Falls [1.34]
Osaka	Dermatitis [1.32]	Neonatal [1.11]	Asthma [1.04]	Congenital [1.15]	URI [1.04]	Skin Viral [0.99]	Diarrhea [1.09]	Urticaria [1.19]	ASD [0.99]	Endocrine [3.81]
Saga	Dermatitis [1.33]	Neonatal [1.09]	Asthma [0.96]	Congenital [0.97]	Diarrhea [1.07]	URI [1.05]	Skin Viral [0.96]	Urticaria [1.07]	ASD [1.32]	Falls [1.25]
Saitama	Dermatitis [1.32]	Neonatal [1.14]	Asthma [0.99]	Congenital [0.95]	URI [1.04]	Diarrhea [1.08]	Skin Viral [0.97]	Urticaria [1.13]	ASD [1.35]	Endocrine [3.93]
Shiga	Dermatitis [1.57]	Neonatal [1.11]	Asthma [1.03]	Congenital [1.1]	URI [1.04]	Diarrhea [1.16]	Skin Viral [0.99]	Urticaria [1.19]	ASD [1.36]	Endocrine [3.86]
Shimane	Dermatitis [1.32]	Neonatal [1.06]	Asthma [0.94]	Congenital [0.95]	Diarrhea [1.14]	URI [1.05]	Skin Viral [0.95]	Urticaria [1.06]	ASD [1.35]	Vit A [1.7]
Shizuoka	Dermatitis [1.32]	Neonatal [1.28]	Asthma [1.01]	Congenital [0.99]	URI [1.04]	Diarrhea [1.12]	Skin Viral [0.98]	Urticaria [1.16]	ASD [1.35]	Endocrine [3.73]
Tochigi	Dermatitis [1.32]	Neonatal [1.23]	Congenital [1.41]	Asthma [1.0]	URI [1.04]	Skin Viral [0.98]	Urticaria [1.14]	ASD [1.34]	Diarrhea [0.95]	Falls [1.17]
Tokushima	Dermatitis [1.32]	Neonatal [1.16]	Asthma [0.97]	Congenital [1.01]	Diarrhea [1.18]	URI [1.04]	Skin Viral [0.97]	Urticaria [1.11]	ASD [1.34]	Falls [1.18]
Tokyo	Dermatitis [1.33]	Neonatal [1.31]	Congenital [1.33]	Asthma [1.2]	URI [1.03]	Skin Viral [1.06]	Urticaria [1.4]	ASD [1.36]	Diarrhea [1.2]	Endocrine [4.25]
Tottori	Dermatitis [1.32]	Neonatal [1.16]	Congenital [1.14]	Asthma [0.96]	URI [1.04]	Skin Viral [0.96]	Diarrhea [1.0]	Urticaria [1.07]	ASD [1.35]	Endocrine [3.8]
Toyama	Dermatitis [1.32]	Neonatal [1.13]	Asthma [1.01]	Congenital [1.02]	Diarrhea [1.15]	URI [1.04]	Skin Viral [0.98]	Urticaria [1.15]	ASD [1.34]	Falls [1.14]
Wakayama	Dermatitis [1.32]	Neonatal [1.2]	Asthma [0.96]	Congenital [0.82]	URI [1.05]	Skin Viral [0.96]	Diarrhea [1.02]	Urticaria [1.09]	ASD [1.35]	Falls [1.23]
Yamagata	Dermatitis [1.33]	Neonatal [0.99]	Asthma [0.95]	Congenital [0.97]	Diarrhea [1.16]	URI [1.05]	Skin Viral [0.96]	Urticaria [1.06]	ASD [1.32]	Falls [1.29]
Yamaguchi	Dermatitis [1.32]	Neonatal [1.03]	Asthma [0.98]	Congenital [0.93]	Diarrhea [1.23]	URI [1.04]	Skin Viral [0.97]	Urticaria [1.12]	ASD [1.35]	Falls [1.17]
Yamanashi	Dermatitis [1.32]	Neonatal [1.29]	Asthma [1.0]	Congenital [1.07]	URI [1.04]	Skin Viral [0.98]	Diarrhea [1.07]	Urticaria [1.14]	ASD [1.32]	Falls [1.16]

eFigure 9c. Leading ten causes of YLDs with the ratio of observed YLDs to YLDs expected on the basis of Socio-Demographic Index alone in 2017, 1-4 years, both sexes combined.
The top ten causes contributing to YLDs are listed globally, by socio-demographic quintile, and then by GBD superregion, region, country, and subnationally where modeled. For each cell, the ratio of observed YLDs to YLDs expected on the basis of socio-demographic index (SDI) alone are listed. Abbreviations: YLD=year of life lived with disability, GBD=Global Burden of Disease.

Values shown in brackets represent the ratio of observed YLDs to predicted YLDs on the basis of Socio-Demographic Index (SDI), rounded to two (2) digits. Color ranges (shown below) were calculated to place a roughly equal number of cells into each bin.

COLOR KEY:	[0.0-0.72]	(0.72-0.84]	(0.84-0.92]	(0.92-1.01]	(1.01-1.09]	(1.09-1.17]	(1.17-1.32]	(1.32-1.67]	1.67+

	1	2	3	4	5	6	7	8	9	10
S Korea	Neonatal [1.22]	Dermatitis [1.16]	Asthma [1.03]	Iron [13.49]	Diarrhea [1.36]	Congenital [0.8]	Skin Viral [1.0]	URI [1.02]	Urticaria [1.14]	ASD [1.1]
Singapore	Neonatal [1.21]	Dermatitis [1.1]	Asthma [1.03]	Diarrhea [1.31]	Skin Viral [1.0]	URI [1.02]	Congenital [0.69]	Urticaria [1.14]	ASD [1.18]	Falls [1.1]
**High-income North America**	Dermatitis [1.61]	Neonatal [0.94]	Asthma [1.46]	Congenital [0.9]	URI [1.16]	Skin Viral [0.97]	Urticaria [1.16]	ASD [1.26]	Diarrhea [0.73]	Psoriasis [4.3]
Canada	Dermatitis [1.75]	Asthma [1.7]	Neonatal [0.66]	URI [1.18]	Skin Viral [1.1]	ASD [1.53]	Congenital [0.81]	Urticaria [1.2]	Diarrhea [1.0]	Psoriasis [4.18]
Greenland	Dermatitis [1.68]	Asthma [1.37]	Neonatal [0.63]	URI [1.23]	Congenital [0.79]	Skin Viral [0.97]	Urticaria [0.9]	Diarrhea [0.73]	ASD [1.31]	Epilepsy [0.9]
USA	Dermatitis [1.6]	Neonatal [0.96]	Asthma [1.42]	Congenital [0.9]	URI [1.16]	Skin Viral [0.95]	Urticaria [1.14]	ASD [1.23]	Diarrhea [0.7]	Psoriasis [4.32]
Alabama	Dermatitis [1.41]	Neonatal [0.94]	Asthma [1.31]	URI [1.17]	Skin Viral [1.06]	Congenital [0.72]	Urticaria [0.93]	Diarrhea [0.71]	Epilepsy [1.22]	ASD [0.79]
Alaska	Dermatitis [1.45]	Neonatal [0.89]	Asthma [1.06]	Congenital [0.98]	URI [1.16]	ASD [1.15]	Skin Viral [0.72]	Urticaria [0.91]	Diarrhea [0.78]	Iron [5.06]
Arizona	Dermatitis [1.67]	Neonatal [0.94]	Asthma [1.36]	Congenital [0.88]	URI [1.16]	Urticaria [1.08]	ASD [1.26]	Skin Viral [0.78]	Diarrhea [0.68]	Epilepsy [1.08]
Arkansas	Dermatitis [1.29]	Neonatal [0.82]	Asthma [0.96]	URI [1.17]	Congenital [0.75]	Skin Viral [0.89]	ASD [1.24]	Urticaria [0.81]	Diarrhea [0.67]	Epilepsy [1.09]
California	Dermatitis [1.8]	Neonatal [0.82]	Asthma [1.4]	URI [1.16]	Congenital [0.84]	ASD [1.41]	Urticaria [1.12]	Skin Viral [0.85]	Psoriasis [4.45]	Diarrhea [0.64]
Colorado	Dermatitis [1.46]	Neonatal [0.97]	Asthma [1.27]	Congenital [0.93]	URI [1.16]	Urticaria [1.14]	Skin Viral [0.73]	ASD [1.08]	Diarrhea [0.69]	Epilepsy [1.17]
Connecticut	Dermatitis [1.78]	Neonatal [1.17]	Asthma [1.98]	URI [1.16]	Congenital [0.91]	Skin Viral [1.14]	Urticaria [1.44]	ASD [1.17]	Diarrhea [0.81]	Psoriasis [4.63]
Delaware	Dermatitis [1.42]	Neonatal [0.96]	Asthma [1.56]	Congenital [0.94]	URI [1.16]	Skin Viral [1.1]	Urticaria [1.21]	ASD [1.15]	Diarrhea [0.77]	Epilepsy [1.52]
DC	Dermatitis [2.06]	Neonatal [0.96]	Asthma [1.62]	Congenital [0.93]	URI [1.15]	Skin Viral [1.12]	Urticaria [1.2]	ASD [1.14]	Diarrhea [0.79]	Psoriasis [4.19]
Florida	Dermatitis [1.6]	Neonatal [1.07]	Asthma [1.73]	Skin Viral [1.15]	Congenital [0.89]	URI [1.16]	Urticaria [1.21]	Psoriasis [5.41]	Diarrhea [0.69]	Epilepsy [1.28]
Georgia	Dermatitis [1.73]	Neonatal [0.9]	Asthma [1.52]	URI [1.16]	Congenital [0.81]	Skin Viral [1.03]	Urticaria [1.04]	ASD [1.17]	Diarrhea [0.69]	Psoriasis [4.86]
Hawaii	Dermatitis [1.83]	Neonatal [0.87]	Asthma [1.64]	Skin Viral [1.13]	URI [1.16]	Congenital [0.88]	Urticaria [1.38]	ASD [1.16]	Diarrhea [0.72]	Psoriasis [4.36]
Idaho	Dermatitis [1.35]	Neonatal [0.78]	Asthma [0.79]	URI [1.17]	Skin Viral [1.04]	Congenital [0.79]	ASD [1.15]	Urticaria [0.82]	Diarrhea [0.69]	Epilepsy [0.96]
Illinois	Dermatitis [1.41]	Neonatal [1.13]	Asthma [1.28]	Congenital [0.97]	URI [1.15]	Urticaria [1.25]	Skin Viral [0.94]	ASD [1.15]	Diarrhea [0.72]	Epilepsy [1.34]
Indiana	Dermatitis [1.37]	Neonatal [0.92]	Asthma [1.33]	Congenital [0.98]	URI [1.16]	Skin Viral [0.81]	Urticaria [0.97]	ASD [1.15]	Diarrhea [0.74]	Epilepsy [1.32]
Iowa	Dermatitis [1.23]	Neonatal [0.9]	Asthma [0.86]	URI [1.16]	Congenital [0.88]	Skin Viral [0.93]	Urticaria [1.1]	ASD [1.15]	Diarrhea [0.74]	Psoriasis [3.34]
Kansas	Dermatitis [1.31]	Neonatal [0.98]	Asthma [1.32]	URI [1.16]	Congenital [0.81]	Skin Viral [0.86]	Urticaria [1.08]	ASD [1.15]	Iron [7.06]	Diarrhea [0.72]
Kentucky	Dermatitis [1.39]	Neonatal [0.93]	Asthma [1.3]	Congenital [0.89]	URI [1.17]	Skin Viral [0.97]	Urticaria [1.03]	ASD [1.15]	Epilepsy [1.31]	Diarrhea [0.59]
Louisiana	Dermatitis [1.48]	Neonatal [0.86]	Asthma [1.17]	Skin Viral [1.32]	Congenital [0.88]	URI [1.17]	Urticaria [1.12]	Epilepsy [1.32]	Diarrhea [0.61]	Psoriasis [4.44]
Maine	Dermatitis [1.21]	Neonatal [0.98]	Asthma [1.58]	Congenital [0.9]	URI [1.16]	Urticaria [1.15]	Skin Viral [0.81]	ASD [1.16]	Diarrhea [0.78]	Epilepsy [1.2]
Maryland	Dermatitis [1.78]	Neonatal [1.13]	Asthma [1.72]	URI [1.15]	Congenital [0.9]	Urticaria [1.29]	Skin Viral [0.94]	ASD [1.27]	Diarrhea [0.76]	Psoriasis [4.49]
Massachusetts	Dermatitis [1.59]	Neonatal [1.13]	Asthma [1.75]	Congenital [1.0]	URI [1.16]	Skin Viral [1.1]	ASD [1.55]	Urticaria [1.51]	Diarrhea [0.83]	Psoriasis [4.71]
Michigan	Dermatitis [1.43]	Neonatal [0.96]	Asthma [1.52]	Congenital [1.02]	URI [1.16]	Skin Viral [1.02]	Urticaria [1.18]	ASD [1.15]	Diarrhea [0.73]	Epilepsy [1.31]
Minnesota	Dermatitis [1.26]	Neonatal [1.02]	Asthma [1.11]	Congenital [0.97]	URI [1.16]	Skin Viral [1.01]	Urticaria [1.27]	ASD [1.37]	Diarrhea [0.75]	Epilepsy [1.19]
Mississippi	Dermatitis [1.29]	Neonatal [0.88]	Asthma [0.94]	URI [1.17]	Skin Viral [0.93]	Congenital [0.68]	ASD [1.14]	Urticaria [0.85]	Diarrhea [0.67]	Epilepsy [1.05]

eFigure 9c. Leading ten causes of YLDs with the ratio of observed YLDs to YLDs expected on the basis of Socio-Demographic Index alone in 2017, 1-4 years, both sexes combined. The top ten causes contributing to YLDs are listed globally, by socio-demographic quintile, and then by GBD superregion, region, country, and subnationally where modeled. For each cell, the ratio of observed YLDs to YLDs expected on the basis of socio-demographic index (SDI) alone are listed. Abbreviations: YLD=year of life lived with disability, GBD=Global Burden of Disease.

Values shown in brackets represent the ratio of observed YLDs to predicted YLDs on the basis of Socio-Demographic Index (SDI), rounded to two (2) digits. Color ranges (shown below) were calculated to place a roughly equal number of cells into each bin.

COLOR KEY:	[0.0-0.72]	(0.72-0.84]	(0.84-0.92]	(0.92-1.01]	(1.01-1.09]	(1.09-1.17]	(1.17-1.32]	(1.32-1.67]	1.67+

	1	2	3	4	5	6	7	8	9	10
Missouri	Dermatitis [1.24]	Neonatal [0.97]	Asthma [1.33]	Congenital [0.93]	URI [1.16]	Skin Viral [0.87]	ASD [1.21]	Urticaria [0.98]	Diarrhea [0.72]	Epilepsy [1.15]
Montana	Dermatitis [1.26]	Neonatal [0.95]	Asthma [0.97]	URI [1.16]	Congenital [0.82]	Skin Viral [0.94]	ASD [1.16]	Urticaria [0.88]	Diarrhea [0.66]	Epilepsy [1.23]
Nebraska	Dermatitis [1.25]	Neonatal [0.92]	Asthma [0.88]	Congenital [0.93]	URI [1.16]	Skin Viral [0.96]	Urticaria [1.14]	ASD [1.17]	Diarrhea [0.71]	Psoriasis [3.5]
Nevada	Dermatitis [1.58]	Neonatal [0.9]	Asthma [1.21]	URI [1.17]	Congenital [0.73]	Urticaria [1.02]	ASD [1.15]	Skin Viral [0.67]	Diarrhea [0.66]	Epilepsy [1.17]
New Hampshire	Dermatitis [1.23]	Neonatal [0.96]	Asthma [1.47]	URI [1.16]	Congenital [0.93]	Skin Viral [0.92]	Urticaria [1.2]	ASD [1.17]	Diarrhea [0.88]	Psoriasis [4.35]
New Jersey	Dermatitis [1.94]	Neonatal [1.06]	Asthma [2.04]	Congenital [1.08]	URI [1.15]	ASD [1.7]	Skin Viral [1.12]	Urticaria [1.37]	Psoriasis [5.31]	Diarrhea [0.81]
New Mexico	Dermatitis [1.46]	Neonatal [1.0]	Asthma [1.32]	Congenital [0.87]	URI [1.17]	Urticaria [0.96]	ASD [1.15]	Skin Viral [0.63]	Diarrhea [0.64]	Epilepsy [1.24]
New York	Dermatitis [1.95]	Neonatal [1.1]	Asthma [2.13]	Congenital [1.1]	Skin Viral [1.17]	URI [1.15]	Urticaria [1.38]	Psoriasis [5.63]	Epilepsy [1.46]	Diarrhea [0.73]
N Carolina	Dermatitis [1.49]	Neonatal [0.95]	Asthma [1.25]	Congenital [0.88]	URI [1.16]	Skin Viral [0.97]	Urticaria [1.11]	ASD [1.34]	Diarrhea [0.71]	Psoriasis [4.26]
N Dakota	Dermatitis [1.23]	Neonatal [1.0]	Asthma [1.17]	Congenital [1.16]	URI [1.16]	Skin Viral [0.91]	ASD [1.16]	Urticaria [0.98]	Diarrhea [0.71]	Epilepsy [1.1]
Ohio	Dermatitis [1.51]	Neonatal [0.92]	Asthma [1.36]	Congenital [1.02]	URI [1.16]	Urticaria [1.15]	Skin Viral [0.9]	ASD [1.15]	Diarrhea [0.73]	Epilepsy [1.39]
Oklahoma	Dermatitis [1.58]	Neonatal [0.87]	Asthma [1.36]	URI [1.16]	Congenital [0.85]	Skin Viral [0.82]	Urticaria [0.94]	ASD [1.15]	Diarrhea [0.69]	Epilepsy [1.27]
Oregon	Dermatitis [1.67]	Neonatal [0.83]	Asthma [1.12]	URI [1.16]	Congenital [0.87]	Skin Viral [0.94]	Urticaria [1.11]	ASD [1.16]	Diarrhea [0.71]	Psoriasis [4.1]
Pennsylvania	Dermatitis [1.53]	Neonatal [0.98]	Asthma [1.57]	Congenital [1.06]	URI [1.16]	Skin Viral [0.93]	Urticaria [1.21]	ASD [1.05]	Diarrhea [0.76]	Epilepsy [1.38]
Rhode Island	Dermatitis [1.41]	Neonatal [0.95]	Asthma [1.61]	Congenital [0.92]	URI [1.16]	Skin Viral [1.14]	Urticaria [1.34]	ASD [1.16]	Diarrhea [0.79]	Psoriasis [4.42]
S Carolina	Dermatitis [1.72]	Neonatal [0.89]	Asthma [1.3]	URI [1.16]	Congenital [0.83]	Skin Viral [1.01]	Urticaria [1.16]	ASD [0.98]	Diarrhea [0.69]	Psoriasis [4.45]
S Dakota	Dermatitis [1.23]	Neonatal [0.82]	Asthma [0.84]	URI [1.17]	Congenital [0.84]	Skin Viral [0.98]	ASD [1.15]	Urticaria [0.93]	Diarrhea [0.68]	Epilepsy [1.05]
Tennessee	Dermatitis [1.4]	Neonatal [0.89]	Asthma [1.01]	URI [1.17]	Congenital [0.79]	ASD [1.49]	Skin Viral [0.88]	Urticaria [1.02]	Diarrhea [0.67]	Epilepsy [1.18]
Texas	Dermatitis [1.71]	Neonatal [0.99]	Asthma [1.43]	URI [1.16]	Congenital [0.83]	ASD [1.61]	Skin Viral [0.88]	Urticaria [1.05]	Diarrhea [0.62]	Psoriasis [4.62]
Utah	Dermatitis [1.39]	Neonatal [0.87]	Asthma [0.87]	ASD [1.92]	URI [1.17]	Skin Viral [1.08]	Congenital [0.84]	Urticaria [0.94]	Diarrhea [0.72]	Iron [5.61]
Vermont	Dermatitis [1.23]	Neonatal [1.03]	Asthma [1.36]	URI [1.16]	Congenital [0.91]	Skin Viral [0.86]	ASD [1.17]	Urticaria [1.01]	Diarrhea [0.83]	Psoriasis [3.8]
Virginia	Dermatitis [1.81]	Neonatal [1.06]	Asthma [1.47]	URI [1.16]	Congenital [0.82]	Urticaria [1.29]	Skin Viral [0.84]	ASD [1.16]	Diarrhea [0.78]	Epilepsy [1.36]
Washington	Dermatitis [1.64]	Neonatal [0.9]	Asthma [1.08]	Congenital [0.95]	URI [1.16]	Urticaria [1.29]	Skin Viral [0.96]	ASD [1.16]	Diarrhea [0.76]	Psoriasis [4.09]
W Virginia	Dermatitis [1.43]	Neonatal [0.9]	Asthma [1.36]	URI [1.17]	Congenital [0.8]	Urticaria [0.97]	Skin Viral [0.79]	Diarrhea [0.71]	Epilepsy [1.37]	ASD [0.86]
Wisconsin	Dermatitis [1.26]	Neonatal [1.03]	Asthma [1.09]	Congenital [0.98]	URI [1.16]	Skin Viral [0.93]	Urticaria [1.13]	ASD [1.04]	Epilepsy [1.35]	Diarrhea [0.66]
Wyoming	Dermatitis [1.37]	Neonatal [0.8]	Asthma [1.04]	Congenital [0.91]	URI [1.16]	ASD [1.15]	Skin Viral [0.75]	Urticaria [0.85]	Diarrhea [0.73]	Epilepsy [1.2]
**Southern Latin America**	Diarrhea [2.96]	Neonatal [0.92]	Dermatitis [1.05]	Asthma [0.96]	Congenital [0.73]	Skin Viral [0.92]	Urticaria [0.81]	URI [0.92]	ASD [1.11]	Vit A [0.46]
Argentina	Diarrhea [3.18]	Neonatal [0.89]	Dermatitis [1.0]	Asthma [0.93]	Congenital [0.74]	Skin Viral [0.92]	Urticaria [0.8]	URI [0.92]	ASD [1.11]	Vit A [0.47]
Chile	Dermatitis [1.22]	Neonatal [1.01]	Diarrhea [2.48]	Asthma [1.0]	Congenital [0.72]	Skin Viral [0.93]	Urticaria [0.86]	URI [0.9]	ASD [1.1]	Epilepsy [1.18]
Uruguay	Neonatal [0.97]	Dermatitis [0.99]	Diarrhea [1.46]	Asthma [1.05]	Skin Viral [0.92]	Congenital [0.7]	Urticaria [0.79]	URI [0.93]	ASD [1.12]	Vit A [0.48]
**Western Europe**	Dermatitis [1.12]	Neonatal [0.85]	Congenital [1.04]	URI [1.29]	Asthma [0.79]	Skin Viral [0.95]	Diarrhea [0.88]	ASD [1.04]	Falls [0.91]	Epilepsy [1.06]

eFigure 9c. Leading ten causes of YLDs with the ratio of observed YLDs to YLDs expected on the basis of Socio-Demographic Index alone in 2017, 1-4 years, both sexes combined.
The top ten causes contributing to YLDs are listed globally, by socio-demographic quintile, and then by GBD superregion, region, country, and subnationally where modeled. For each cell, the ratio of observed YLDs to YLDs expected on the basis of socio-demographic index (SDI) alone are listed. Abbreviations: YLD=year of life lived with disability, GBD=Global Burden of Disease.

Values shown in brackets represent the ratio of observed YLDs to predicted YLDs on the basis of Socio-Demographic Index (SDI), rounded to two (2) digits. Color ranges (shown below) were calculated to place a roughly equal number of cells into each bin.

COLOR KEY:	[0.0-0.72]	(0.72-0.84]	(0.84-0.92]	(0.92-1.01]	(1.01-1.09]	(1.09-1.17]	(1.17-1.32]	(1.32-1.67]	1.67+

	1	2	3	4	5	6	7	8	9	10
Andorra	Dermatitis [1.31]	Neonatal [0.92]	Congenital [1.09]	Asthma [0.95]	URI [1.28]	Skin Viral [1.01]	Diarrhea [0.92]	ASD [1.03]	Falls [0.79]	Urticaria [0.68]
Austria	Neonatal [1.37]	Dermatitis [0.83]	Congenital [1.07]	URI [1.29]	Diarrhea [1.35]	Asthma [0.73]	Hernia [3.72]	Skin Viral [0.96]	ASD [1.03]	Falls [0.92]
Belgium	Dermatitis [0.98]	Neonatal [0.61]	Congenital [1.02]	URI [1.28]	Diarrhea [1.2]	Asthma [0.71]	Skin Viral [0.98]	ASD [1.03]	Falls [0.97]	Epilepsy [1.2]
Cyprus	Neonatal [1.11]	Dermatitis [0.85]	Congenital [1.01]	URI [1.29]	Asthma [0.74]	Skin Viral [0.96]	Diarrhea [1.05]	ASD [1.04]	Hernia [1.81]	Falls [0.88]
Denmark	Dermatitis [1.22]	Neonatal [0.75]	Congenital [1.18]	URI [1.27]	Asthma [0.96]	Skin Viral [1.14]	Diarrhea [1.3]	ASD [1.22]	Urticaria [0.79]	Falls [0.75]
Finland	Neonatal [1.76]	Dermatitis [1.3]	Congenital [1.1]	URI [1.28]	Asthma [0.88]	Diarrhea [1.37]	Skin Viral [1.01]	ASD [1.05]	Falls [0.91]	Urticaria [0.67]
France	Dermatitis [1.21]	Neonatal [0.7]	Congenital [1.02]	URI [1.29]	Asthma [0.81]	Skin Viral [0.96]	ASD [0.96]	Urticaria [0.65]	Epilepsy [1.15]	Falls [0.91]
Germany	Dermatitis [0.97]	Neonatal [1.01]	Congenital [1.05]	Diarrhea [1.45]	URI [1.28]	Skin Viral [0.98]	Epilepsy [2.0]	Asthma [0.6]	ASD [1.04]	Falls [0.89]
Greece	Neonatal [0.86]	Dermatitis [0.74]	Congenital [0.98]	URI [1.31]	Asthma [0.65]	Skin Viral [0.92]	Diarrhea [0.71]	ASD [1.03]	Urticaria [0.55]	Falls [1.03]
Iceland	Dermatitis [1.35]	Neonatal [0.94]	Asthma [1.27]	URI [1.28]	Congenital [1.0]	Skin Viral [1.1]	Diarrhea [1.05]	ASD [1.0]	Falls [0.78]	Urticaria [0.69]
Ireland	Dermatitis [1.15]	Neonatal [0.78]	Asthma [1.07]	Congenital [1.13]	URI [1.28]	Skin Viral [0.98]	ASD [1.11]	Diarrhea [0.69]	Falls [0.95]	Urticaria [0.64]
Israel	Dermatitis [1.05]	Neonatal [0.92]	Congenital [1.0]	URI [1.3]	Asthma [0.68]	Blindness [2.53]	Skin Viral [0.9]	ASD [0.96]	Falls [1.07]	Diarrhea [0.46]
Italy	Dermatitis [0.98]	Neonatal [0.66]	Congenital [0.99]	URI [1.3]	Skin Viral [0.95]	ASD [1.03]	Asthma [0.42]	Diarrhea [0.62]	Blindness [1.04]	Falls [0.79]
Luxembourg	Dermatitis [1.31]	Neonatal [1.21]	Asthma [1.05]	Congenital [1.07]	URI [1.28]	Skin Viral [1.02]	Diarrhea [1.15]	ASD [1.05]	Vit A [2.42]	Falls [0.75]
Malta	Dermatitis [0.88]	Neonatal [0.72]	Congenital [1.19]	URI [1.3]	Asthma [0.77]	Skin Viral [0.93]	ASD [1.04]	Falls [1.28]	Urticaria [0.56]	Diarrhea [0.42]
Netherlands	Dermatitis [1.22]	Neonatal [0.95]	Congenital [1.09]	URI [1.28]	Asthma [0.85]	Skin Viral [0.95]	ASD [1.15]	Urticaria [0.7]	Diarrhea [0.62]	Falls [0.72]
Norway	Dermatitis [1.31]	Neonatal [1.11]	Diarrhea [4.13]	Congenital [1.47]	Asthma [1.28]	URI [1.27]	Skin Viral [1.09]	ASD [0.98]	Hernia [2.31]	Falls [0.97]
Portugal	Dermatitis [0.94]	Neonatal [0.59]	Asthma [0.98]	URI [1.31]	Congenital [0.8]	Skin Viral [0.86]	ASD [1.03]	Diarrhea [0.43]	Vit A [0.75]	Urticaria [0.48]
Spain	Dermatitis [0.75]	Neonatal [0.71]	URI [1.31]	Congenital [0.93]	Asthma [0.61]	Skin Viral [0.92]	ASD [1.04]	Diarrhea [0.52]	ID [4.78]	Urticaria [0.54]
Sweden	Dermatitis [1.63]	Neonatal [0.8]	Asthma [1.1]	Congenital [1.23]	URI [1.27]	Skin Viral [1.07]	Diarrhea [1.21]	ASD [1.13]	Falls [1.0]	Urticaria [0.77]
Stockholm	Dermatitis [1.34]	Neonatal [0.8]	Congenital [1.28]	Asthma [1.01]	URI [1.26]	Skin Viral [1.11]	Diarrhea [1.33]	ASD [1.43]	Vit A [2.98]	Falls [0.9]
Sweden w/o Stockholm	Dermatitis [1.73]	Neonatal [0.8]	Asthma [1.13]	Congenital [1.21]	URI [1.27]	Skin Viral [1.06]	Diarrhea [1.17]	ASD [1.04]	Falls [1.04]	Urticaria [0.75]
Switzerland	Dermatitis [1.3]	Neonatal [0.87]	Congenital [1.08]	URI [1.28]	Asthma [0.86]	Diarrhea [1.21]	Skin Viral [0.99]	ASD [1.04]	Urticaria [0.66]	Blindness [1.05]
UK	Dermatitis [1.43]	Neonatal [0.99]	Asthma [1.18]	Congenital [1.12]	URI [1.28]	Skin Viral [0.88]	Diarrhea [0.91]	ASD [1.11]	Urticaria [0.62]	Iron [4.15]
England	Dermatitis [1.46]	Neonatal [1.02]	Asthma [1.21]	Congenital [1.12]	URI [1.28]	Skin Viral [0.87]	Diarrhea [0.95]	ASD [1.12]	Iron [4.54]	Urticaria [0.64]
E Midlands	Dermatitis [1.44]	Neonatal [0.96]	Asthma [1.12]	Congenital [1.14]	URI [1.28]	Skin Viral [0.85]	Diarrhea [0.89]	ASD [1.1]	Urticaria [0.6]	Iron [3.48]
Derby	Dermatitis [1.43]	Neonatal [0.98]	Asthma [1.15]	Congenital [1.16]	URI [1.28]	Skin Viral [0.86]	Diarrhea [0.8]	ASD [1.1]	Urticaria [0.63]	Falls [0.98]
Derbyshire	Dermatitis [1.44]	Neonatal [0.88]	Asthma [1.08]	Congenital [1.12]	URI [1.29]	Diarrhea [0.89]	Skin Viral [0.84]	ASD [1.1]	Iron [2.94]	Urticaria [0.58]
Leicester	Dermatitis [1.44]	Neonatal [0.84]	Asthma [1.14]	Congenital [1.16]	URI [1.28]	Skin Viral [0.86]	Diarrhea [0.9]	ASD [1.1]	Urticaria [0.62]	Falls [1.01]
Leicestershire	Dermatitis [1.44]	Neonatal [0.82]	Asthma [1.15]	Congenital [1.16]	URI [1.28]	Skin Viral [0.86]	Iron [6.52]	Diarrhea [0.84]	ASD [1.1]	Urticaria [0.63]
Lincolnshire	Dermatitis [1.44]	Neonatal [0.93]	Asthma [1.08]	Congenital [1.12]	URI [1.29]	Diarrhea [0.88]	Skin Viral [0.84]	ASD [1.1]	Iron [2.73]	Urticaria [0.57]

eFigure 9c. Leading ten causes of YLDs with the ratio of observed YLDs to YLDs expected on the basis of Socio-Demographic Index alone in 2017, 1-4 years, both sexes combined.
The top ten causes contributing to YLDs are listed globally, by socio-demographic quintile, and then by GBD superregion, region, country, and subnationally where modeled. For each cell, the ratio of observed YLDs to YLDs expected on the basis of socio-demographic index (SDI) alone are listed. Abbreviations: YLD=year of life lived with disability, GBD=Global Burden of Disease.

Values shown in brackets represent the ratio of observed YLDs to predicted YLDs on the basis of Socio-Demographic Index (SDI), rounded to two (2) digits. Color ranges (shown below) were calculated to place a roughly equal number of cells into each bin.

COLOR KEY:	[0.0-0.72]	(0.72-0.84]	(0.84-0.92]	(0.92-1.01]	(1.01-1.09]	(1.09-1.17]	(1.17-1.32]	(1.32-1.67]	1.67+

	1	2	3	4	5	6	7	8	9	10
Northamptonshire	Dermatitis [1.44]	Neonatal [1.05]	Asthma [1.11]	Congenital [1.14]	URI [1.28]	Diarrhea [0.99]	Skin Viral [0.85]	ASD [1.1]	Urticaria [0.6]	Falls [1.03]
Nottingham	Dermatitis [1.43]	Neonatal [1.17]	Asthma [1.21]	Congenital [1.18]	URI [1.27]	Skin Viral [0.88]	Diarrhea [0.88]	ASD [1.11]	Urticaria [0.66]	Falls [0.92]
Nottinghamshire	Dermatitis [1.44]	Neonatal [1.03]	Asthma [1.09]	Congenital [1.13]	URI [1.29]	Diarrhea [0.87]	Skin Viral [0.84]	ASD [1.1]	Iron [3.21]	Urticaria [0.58]
Rutland	Dermatitis [1.44]	Neonatal [0.69]	Asthma [1.13]	Congenital [1.13]	URI [1.29]	Skin Viral [0.86]	Diarrhea [0.8]	ASD [1.09]	Urticaria [0.61]	Falls [1.02]
E England	Dermatitis [1.44]	Neonatal [0.92]	Asthma [1.14]	Congenital [1.05]	URI [1.28]	Skin Viral [0.86]	Diarrhea [0.86]	ASD [1.17]	Urticaria [0.62]	Falls [1.0]
Bedford	Dermatitis [1.44]	Neonatal [1.11]	Asthma [1.13]	Congenital [1.06]	URI [1.27]	Skin Viral [0.86]	Diarrhea [0.82]	ASD [1.09]	Urticaria [0.62]	Falls [0.98]
Cambridgeshire	Dermatitis [1.43]	Neonatal [0.96]	Asthma [1.21]	Congenital [1.09]	URI [1.28]	ASD [1.67]	Skin Viral [0.89]	Diarrhea [0.96]	Urticaria [0.68]	Falls [0.9]
Cen Bedfordshire	Dermatitis [1.44]	Neonatal [0.99]	Asthma [1.12]	Congenital [1.02]	URI [1.28]	Skin Viral [0.86]	Diarrhea [0.89]	ASD [1.1]	Urticaria [0.61]	Falls [1.01]
Essex	Dermatitis [1.44]	Neonatal [0.91]	Asthma [1.11]	Congenital [1.04]	URI [1.29]	Skin Viral [0.85]	Diarrhea [0.77]	ASD [1.11]	Urticaria [0.6]	Falls [1.03]
Hertfordshire	Dermatitis [1.43]	Neonatal [0.94]	Asthma [1.22]	Congenital [1.07]	URI [1.27]	Skin Viral [0.89]	Diarrhea [0.89]	ASD [1.12]	Urticaria [0.68]	Falls [0.9]
Luton	Dermatitis [1.44]	Neonatal [1.04]	Asthma [1.13]	Congenital [1.06]	URI [1.28]	Diarrhea [1.02]	Skin Viral [0.85]	ASD [1.1]	Urticaria [0.61]	Falls [1.03]
Norfolk	Dermatitis [1.44]	Neonatal [0.78]	Asthma [1.1]	Congenital [1.04]	URI [1.28]	Diarrhea [0.92]	Skin Viral [0.85]	ASD [1.11]	Iron [4.43]	Urticaria [0.6]
Peterborough	Dermatitis [1.44]	Neonatal [0.74]	Asthma [1.11]	Congenital [1.03]	URI [1.28]	Diarrhea [0.89]	Skin Viral [0.84]	ASD [1.1]	Iron [3.68]	Urticaria [0.59]
Southend-on-Sea	Dermatitis [1.44]	Neonatal [1.06]	Asthma [1.07]	Congenital [1.03]	URI [1.29]	Skin Viral [0.84]	ASD [1.1]	Diarrhea [0.7]	Iron [3.29]	Urticaria [0.58]
Suffolk	Dermatitis [1.44]	Neonatal [0.95]	Asthma [1.09]	Congenital [1.02]	URI [1.29]	Skin Viral [0.84]	Diarrhea [0.87]	ASD [1.11]	Urticaria [0.59]	Falls [1.07]
Thurrock	Dermatitis [1.45]	Neonatal [1.0]	Asthma [1.07]	Congenital [1.02]	URI [1.29]	Skin Viral [0.84]	Diarrhea [0.69]	ASD [1.09]	Urticaria [0.57]	Falls [1.13]
Greater London	Dermatitis [1.44]	Neonatal [1.1]	Asthma [1.49]	Congenital [1.13]	URI [1.26]	Skin Viral [0.91]	Diarrhea [0.96]	ASD [1.12]	Urticaria [0.73]	Falls [0.82]
Barking & Dagenham	Dermatitis [1.81]	Neonatal [0.97]	Asthma [1.21]	Congenital [1.01]	URI [1.29]	Iron [4.13]	Skin Viral [0.83]	Diarrhea [0.75]	ASD [1.1]	Urticaria [0.56]
Barnet	Dermatitis [1.43]	Neonatal [1.02]	Asthma [1.38]	Congenital [1.06]	URI [1.27]	Skin Viral [0.88]	Diarrhea [0.85]	ASD [1.11]	Urticaria [0.66]	Falls [0.9]
Bexley	Dermatitis [1.44]	Neonatal [0.94]	Asthma [1.28]	Congenital [1.03]	URI [1.29]	Skin Viral [0.85]	Diarrhea [0.85]	ASD [1.18]	Urticaria [0.6]	Falls [1.05]
Brent	Dermatitis [1.43]	Neonatal [1.05]	Asthma [1.34]	Congenital [1.04]	URI [1.27]	Skin Viral [0.87]	Diarrhea [0.86]	ASD [1.1]	Iron [5.68]	Urticaria [0.64]
Bromley	Dermatitis [1.43]	Neonatal [1.11]	Asthma [1.34]	Congenital [1.07]	URI [1.28]	Skin Viral [0.87]	ASD [1.18]	Diarrhea [0.73]	Urticaria [0.63]	Falls [0.96]
Camden	Dermatitis [1.44]	Neonatal [1.0]	Asthma [1.63]	Congenital [1.17]	URI [1.26]	Skin Viral [0.95]	ASD [1.13]	Diarrhea [1.02]	Urticaria [0.81]	Falls [0.74]
Croydon	Dermatitis [1.44]	Neonatal [1.0]	Asthma [1.3]	Congenital [1.05]	URI [1.29]	Skin Viral [0.86]	Diarrhea [0.79]	ASD [1.1]	Iron [4.29]	Urticaria [0.61]
Ealing	Dermatitis [1.43]	Neonatal [1.15]	Asthma [1.39]	Congenital [1.06]	URI [1.27]	Skin Viral [0.88]	Diarrhea [0.95]	ASD [1.1]	Iron [5.47]	Urticaria [0.66]
Enfield	Dermatitis [1.44]	Neonatal [1.05]	Asthma [1.32]	Congenital [1.05]	URI [1.28]	Skin Viral [0.86]	Diarrhea [0.88]	ASD [1.11]	Urticaria [0.62]	Falls [0.98]
Greenwich	Dermatitis [1.44]	Neonatal [0.98]	Asthma [1.3]	Congenital [1.07]	URI [1.28]	Skin Viral [0.86]	Diarrhea [0.84]	ASD [1.17]	Urticaria [0.61]	Falls [1.0]
Hackney	Dermatitis [1.43]	Neonatal [1.09]	Asthma [1.49]	Congenital [1.11]	URI [1.26]	Skin Viral [0.9]	Diarrhea [0.93]	ASD [1.09]	Urticaria [0.71]	Falls [0.85]
Hammersmith & Fulham	Dermatitis [1.44]	Neonatal [1.11]	Asthma [1.59]	Congenital [1.16]	URI [1.26]	Skin Viral [0.95]	Diarrhea [1.07]	ASD [1.11]	Urticaria [0.8]	Falls [0.75]
Haringey	Dermatitis [1.43]	Neonatal [1.11]	Asthma [1.35]	Congenital [1.08]	URI [1.26]	Skin Viral [0.87]	Diarrhea [0.82]	ASD [1.09]	Iron [4.96]	Urticaria [0.64]
Harrow	Dermatitis [1.43]	Neonatal [1.14]	Asthma [1.35]	Congenital [1.06]	URI [1.28]	Skin Viral [0.87]	Diarrhea [0.87]	ASD [1.1]	Urticaria [0.64]	Falls [0.95]

eFigure 9c. Leading ten causes of YLDs with the ratio of observed YLDs to YLDs expected on the basis of Socio-Demographic Index alone in 2017, 1-4 years, both sexes combined.
The top ten causes contributing to YLDs are listed globally, by socio-demographic quintile, and then by GBD superregion, region, country, and subnationally where modeled. For each cell, the ratio of observed YLDs to YLDs expected on the basis of socio-demographic index (SDI) alone are listed. Abbreviations: YLD=year of life lived with disability, GBD=Global Burden of Disease.

Values shown in brackets represent the ratio of observed YLDs to predicted YLDs on the basis of Socio-Demographic Index (SDI), rounded to two (2) digits. Color ranges (shown below) were calculated to place a roughly equal number of cells into each bin.

COLOR KEY:	[0.0-0.72]	(0.72-0.84]	(0.84-0.92]	(0.92-1.01]	(1.01-1.09]	(1.09-1.17]	(1.17-1.32]	(1.32-1.67]	1.67+

	1	2	3	4	5	6	7	8	9	10
Havering	Dermatitis [1.44]	Neonatal [0.98]	Asthma [1.27]	Congenital [1.04]	URI [1.29]	Skin Viral [0.85]	Diarrhea [0.79]	ASD [1.11]	Urticaria [0.6]	Falls [1.05]
Hillingdon	Dermatitis [1.43]	Neonatal [1.19]	Asthma [1.45]	Congenital [1.09]	URI [1.26]	Skin Viral [0.89]	Diarrhea [1.05]	ASD [1.11]	Urticaria [0.7]	Falls [0.86]
Hounslow	Dermatitis [1.43]	Neonatal [1.14]	Asthma [1.44]	Congenital [1.08]	URI [1.26]	Skin Viral [0.89]	Diarrhea [0.91]	ASD [1.11]	Iron [8.2]	Urticaria [0.7]
Islington	Dermatitis [1.44]	Neonatal [0.97]	Asthma [1.59]	Congenital [1.17]	URI [1.26]	Skin Viral [0.94]	Diarrhea [1.03]	ASD [1.12]	Urticaria [0.78]	Falls [0.76]
Kensington & Chelsea	Dermatitis [1.44]	Neonatal [0.96]	Asthma [1.62]	Congenital [1.16]	URI [1.26]	Skin Viral [0.95]	ASD [1.14]	Diarrhea [0.92]	Urticaria [0.81]	Falls [0.74]
Kingston upon Thames	Dermatitis [1.43]	Neonatal [1.15]	Asthma [1.48]	Congenital [1.13]	URI [1.27]	Skin Viral [0.9]	ASD [1.11]	Diarrhea [0.89]	Urticaria [0.72]	Falls [0.83]
Lambeth	Dermatitis [1.43]	Neonatal [0.81]	Asthma [1.5]	Congenital [1.15]	URI [1.26]	Skin Viral [0.92]	ASD [1.11]	Diarrhea [0.9]	Urticaria [0.74]	Falls [0.8]
Lewisham	Dermatitis [1.43]	Neonatal [1.2]	Asthma [1.33]	Congenital [1.07]	URI [1.27]	Skin Viral [0.86]	ASD [1.17]	Diarrhea [0.76]	Urticaria [0.63]	Iron [4.06]
Merton	Dermatitis [1.43]	Neonatal [1.07]	Asthma [1.43]	Congenital [1.1]	URI [1.27]	Skin Viral [0.89]	ASD [1.11]	Diarrhea [0.81]	Urticaria [0.68]	Falls [0.87]
Newham	Dermatitis [1.43]	Neonatal [1.08]	Asthma [1.32]	Congenital [1.05]	URI [1.28]	Skin Viral [0.86]	Diarrhea [0.9]	ASD [1.09]	Urticaria [0.62]	Falls [0.96]
Redbridge	Dermatitis [1.44]	Neonatal [1.0]	Asthma [1.28]	Congenital [1.11]	URI [1.28]	Skin Viral [0.85]	Diarrhea [0.84]	ASD [1.11]	Iron [4.08]	Urticaria [0.6]
Richmond upon Thames	Dermatitis [1.43]	Neonatal [1.21]	Asthma [1.5]	Congenital [1.14]	URI [1.27]	Skin Viral [0.91]	ASD [1.12]	Diarrhea [0.84]	Iron [8.69]	Urticaria [0.74]
Southwark	Dermatitis [1.43]	Neonatal [0.83]	Asthma [1.54]	Congenital [1.16]	URI [1.26]	Skin Viral [0.93]	ASD [1.17]	Iron [11.76]	Diarrhea [0.94]	Urticaria [0.76]
Sutton	Dermatitis [1.43]	Neonatal [1.26]	Asthma [1.33]	Congenital [1.06]	URI [1.28]	Skin Viral [0.86]	Diarrhea [0.81]	ASD [1.11]	Urticaria [0.63]	Falls [0.96]
Tower Hamlets	Dermatitis [1.43]	Neonatal [1.2]	Asthma [1.55]	Congenital [1.15]	URI [1.26]	Skin Viral [0.92]	Diarrhea [1.0]	ASD [1.12]	Urticaria [0.75]	Falls [0.81]
Waltham Forest	Dermatitis [1.44]	Neonatal [1.0]	Asthma [1.26]	Congenital [1.05]	URI [1.28]	Skin Viral [0.84]	Diarrhea [0.82]	ASD [1.11]	Urticaria [0.59]	Falls [1.05]
Wandsworth	Dermatitis [1.43]	Neonatal [0.98]	Asthma [1.55]	Congenital [1.16]	URI [1.26]	Skin Viral [0.93]	ASD [1.12]	Diarrhea [0.85]	Urticaria [0.76]	Falls [0.78]
Westminster	Dermatitis [1.44]	Neonatal [0.91]	Asthma [1.61]	Congenital [1.15]	URI [1.26]	Skin Viral [0.94]	ASD [1.12]	Diarrhea [0.96]	Urticaria [0.8]	Falls [0.75]
NE England	Dermatitis [1.54]	Neonatal [0.9]	Asthma [1.1]	Congenital [1.28]	URI [1.29]	Diarrhea [1.09]	Skin Viral [0.84]	ASD [1.1]	Urticaria [0.59]	Falls [1.06]
County Durham	Dermatitis [1.68]	Neonatal [0.95]	Asthma [1.09]	Congenital [1.27]	URI [1.29]	Diarrhea [1.08]	Skin Viral [0.84]	ASD [1.1]	Urticaria [0.57]	Falls [1.11]
Darlington	Dermatitis [1.44]	Neonatal [0.87]	Asthma [1.12]	Congenital [1.29]	URI [1.29]	Diarrhea [1.08]	Skin Viral [0.85]	ASD [1.09]	Urticaria [0.59]	Falls [1.04]
Gateshead	Dermatitis [1.66]	Neonatal [1.0]	Asthma [1.12]	Congenital [1.29]	URI [1.28]	Diarrhea [1.05]	Skin Viral [0.85]	ASD [1.1]	Urticaria [0.59]	Falls [1.05]
Hartlepool	Dermatitis [1.45]	Neonatal [0.99]	Asthma [1.08]	Congenital [1.26]	URI [1.29]	Diarrhea [0.92]	Skin Viral [0.83]	ASD [1.1]	Urticaria [0.56]	Falls [1.15]
Middlesbrough	Dermatitis [1.45]	Neonatal [0.78]	Asthma [1.1]	Congenital [1.27]	URI [1.28]	Diarrhea [1.15]	Skin Viral [0.84]	ASD [1.08]	Urticaria [0.57]	Falls [1.09]
Newcastle upon Tyne	Dermatitis [1.43]	Neonatal [0.89]	Asthma [1.24]	Congenital [1.35]	URI [1.27]	Diarrhea [1.25]	Skin Viral [0.88]	ASD [1.11]	Urticaria [0.68]	Falls [0.89]
N Tyneside	Dermatitis [1.44]	Neonatal [0.85]	Asthma [1.12]	Congenital [1.29]	URI [1.28]	Diarrhea [1.19]	Skin Viral [0.84]	ASD [1.12]	Urticaria [0.6]	Falls [1.06]
Northumberland	Dermatitis [1.44]	Neonatal [0.86]	Asthma [1.09]	Congenital [1.28]	URI [1.3]	Diarrhea [1.02]	Skin Viral [0.84]	ASD [1.11]	Urticaria [0.58]	Falls [1.12]
Redcar & Cleveland	Dermatitis [1.45]	Neonatal [0.8]	Asthma [1.06]	Congenital [1.25]	URI [1.3]	Diarrhea [1.04]	Skin Viral [0.83]	ASD [1.11]	Urticaria [0.55]	Falls [1.2]
S Tyneside	Dermatitis [1.68]	Neonatal [0.84]	Asthma [1.06]	Congenital [1.25]	URI [1.3]	Diarrhea [0.98]	Skin Viral [0.83]	ASD [1.1]	Iron [2.44]	Urticaria [0.56]
Stockton-on-Tees	Dermatitis [1.44]	Neonatal [1.07]	Asthma [1.12]	Congenital [1.29]	URI [1.28]	Diarrhea [1.06]	Skin Viral [0.85]	ASD [1.09]	Urticaria [0.59]	Falls [1.04]
Sunderland	Dermatitis [1.67]	Neonatal [0.78]	Congenital [1.28]	Asthma [0.99]	URI [1.29]	Diarrhea [1.08]	Skin Viral [0.84]	ASD [1.1]	Urticaria [0.58]	Falls [1.08]

eFigure 9c. Leading ten causes of YLDs with the ratio of observed YLDs to YLDs expected on the basis of Socio-Demographic Index alone in 2017, 1-4 years, both sexes combined. The top ten causes contributing to YLDs are listed globally, by socio-demographic quintile, and then by GBD superregion, region, country, and subnationally where modeled. For each cell, the ratio of observed YLDs to YLDs expected on the basis of socio-demographic index (SDI) alone are listed. Abbreviations: YLD=year of life lived with disability, GBD=Global Burden of Disease.

Values shown in brackets represent the ratio of observed YLDs to predicted YLDs on the basis of Socio-Demographic Index (SDI), rounded to two (2) digits. Color ranges (shown below) were calculated to place a roughly equal number of cells into each bin.

COLOR KEY:	[0.0-0.72]	(0.72-0.84]	(0.84-0.92]	(0.92-1.01]	(1.01-1.09]	(1.09-1.17]	(1.17-1.32]	(1.32-1.67]	1.67+

	1	2	3	4	5	6	7	8	9	10
NW England	Dermatitis [1.44]	Neonatal [1.1]	Asthma [1.14]	Congenital [1.23]	URI [1.28]	Diarrhea [1.02]	Skin Viral [0.85]	ASD [1.1]	Iron [5.03]	Urticaria [0.61]
Blackburn with Darwen	Dermatitis [1.45]	Neonatal [1.07]	Asthma [1.08]	Congenital [1.15]	URI [1.28]	Diarrhea [1.08]	Iron [4.45]	Skin Viral [0.83]	ASD [1.09]	Urticaria [0.56]
Blackpool	Dermatitis [1.47]	Neonatal [1.01]	Asthma [1.05]	Congenital [1.15]	URI [1.3]	Diarrhea [0.79]	Skin Viral [0.83]	Iron [2.39]	ASD [1.09]	Urticaria [0.54]
Bolton	Dermatitis [1.45]	Neonatal [1.02]	Asthma [1.08]	Congenital [1.16]	URI [1.29]	Diarrhea [0.95]	Skin Viral [0.84]	Iron [4.15]	ASD [1.09]	Urticaria [0.57]
Bury	Dermatitis [1.44]	Neonatal [0.99]	Asthma [1.08]	Congenital [1.18]	URI [1.28]	Diarrhea [0.99]	Iron [5.53]	Skin Viral [0.84]	ASD [1.1]	Urticaria [0.58]
Cheshire E	Dermatitis [1.43]	Neonatal [1.37]	Asthma [1.21]	Congenital [1.34]	URI [1.28]	Diarrhea [1.06]	Skin Viral [0.88]	ASD [1.11]	Urticaria [0.66]	Falls [0.91]
Cheshire W & Chester	Dermatitis [1.43]	Neonatal [1.13]	Asthma [1.18]	Congenital [1.33]	URI [1.28]	Diarrhea [1.05]	Skin Viral [0.87]	ASD [1.11]	Iron [5.81]	Urticaria [0.64]
Cumbria	Dermatitis [1.44]	Neonatal [1.08]	Asthma [1.12]	Congenital [1.19]	URI [1.28]	Diarrhea [1.07]	Skin Viral [0.85]	Iron [5.68]	ASD [1.1]	Urticaria [0.6]
Halton	Dermatitis [1.44]	Neonatal [1.12]	Asthma [1.12]	Congenital [1.18]	URI [1.28]	Diarrhea [0.98]	Skin Viral [0.85]	Iron [5.57]	ASD [1.08]	Urticaria [0.6]
Knowsley	Dermatitis [1.44]	Neonatal [1.06]	Asthma [1.1]	Congenital [1.29]	URI [1.28]	Diarrhea [0.93]	Skin Viral [0.84]	ASD [1.1]	Iron [3.24]	Urticaria [0.58]
Lancashire	Dermatitis [1.44]	Neonatal [1.07]	Asthma [1.12]	Congenital [1.19]	URI [1.28]	Diarrhea [1.11]	Skin Viral [0.85]	ASD [1.1]	Iron [3.83]	Urticaria [0.6]
Liverpool	Dermatitis [1.43]	Neonatal [1.21]	Congenital [1.35]	Asthma [1.17]	URI [1.28]	Skin Viral [0.87]	Diarrhea [0.9]	ASD [1.1]	Iron [5.5]	Urticaria [0.64]
Manchester	Dermatitis [1.43]	Neonatal [1.25]	Asthma [1.29]	Congenital [1.28]	URI [1.26]	Diarrhea [1.21]	Skin Viral [0.9]	ASD [1.1]	Iron [7.9]	Urticaria [0.71]
Oldham	Dermatitis [1.46]	Neonatal [0.8]	Asthma [1.07]	Congenital [1.16]	URI [1.29]	Diarrhea [0.92]	Skin Viral [0.83]	ASD [1.09]	Iron [2.41]	Urticaria [0.55]
Rochdale	Dermatitis [1.45]	Neonatal [0.83]	Asthma [1.07]	Congenital [1.15]	URI [1.29]	Iron [5.08]	Diarrhea [0.96]	Skin Viral [0.83]	ASD [1.1]	Urticaria [0.56]
Salford	Dermatitis [1.43]	Neonatal [1.24]	Asthma [1.16]	Congenital [1.21]	URI [1.28]	Diarrhea [0.94]	Skin Viral [0.86]	ASD [1.1]	Urticaria [0.62]	Falls [0.99]
Sefton	Dermatitis [1.44]	Neonatal [1.08]	Asthma [1.08]	Congenital [1.28]	URI [1.29]	Diarrhea [0.86]	Skin Viral [0.84]	ASD [1.11]	Iron [3.82]	Urticaria [0.57]
St Helens	Dermatitis [1.45]	Neonatal [1.09]	Asthma [1.07]	Congenital [1.27]	URI [1.29]	Diarrhea [0.89]	Skin Viral [0.84]	ASD [1.1]	Iron [3.01]	Urticaria [0.57]
Stockport	Dermatitis [1.43]	Neonatal [0.95]	Asthma [1.15]	Congenital [1.22]	URI [1.28]	Diarrhea [1.15]	Skin Viral [0.86]	Iron [6.08]	ASD [1.11]	Urticaria [0.62]
Tameside	Dermatitis [1.45]	Neonatal [0.89]	Asthma [1.06]	Congenital [1.15]	URI [1.29]	Diarrhea [0.96]	Skin Viral [0.83]	ASD [1.1]	Iron [2.45]	Urticaria [0.55]
Trafford	Dermatitis [1.43]	Neonatal [1.24]	Asthma [1.24]	Congenital [1.25]	URI [1.27]	Skin Viral [0.89]	Diarrhea [1.03]	ASD [1.1]	Iron [6.61]	Urticaria [0.68]
Warrington	Dermatitis [1.43]	Neonatal [1.3]	Asthma [1.2]	Congenital [1.23]	URI [1.27]	Diarrhea [1.04]	Skin Viral [0.88]	ASD [1.1]	Iron [6.32]	Urticaria [0.66]
Wigan	Dermatitis [1.45]	Neonatal [1.08]	Asthma [1.06]	Congenital [1.16]	URI [1.3]	Iron [4.1]	Diarrhea [0.84]	Skin Viral [0.83]	ASD [1.11]	Urticaria [0.56]
Wirral	Dermatitis [1.44]	Neonatal [1.19]	Asthma [1.07]	Congenital [1.28]	URI [1.29]	Diarrhea [0.94]	Skin Viral [0.83]	ASD [1.1]	Iron [3.05]	Urticaria [0.57]
SE England	Dermatitis [1.54]	Neonatal [1.04]	Asthma [1.26]	Congenital [1.12]	URI [1.28]	Skin Viral [0.87]	Diarrhea [0.91]	ASD [1.19]	Iron [5.11]	Urticaria [0.65]
Bracknell Forest	Dermatitis [1.43]	Neonatal [0.85]	Asthma [1.2]	Congenital [1.13]	URI [1.28]	Skin Viral [0.89]	Iron [8.48]	Diarrhea [0.87]	ASD [1.1]	Urticaria [0.67]
Brighton & Hove	Dermatitis [1.65]	Neonatal [1.15]	Asthma [1.24]	Congenital [1.18]	URI [1.27]	Skin Viral [0.9]	ASD [1.1]	Diarrhea [0.86]	Urticaria [0.7]	Falls [0.86]
Buckinghamshire	Dermatitis [1.43]	Neonatal [1.22]	Asthma [1.18]	Congenital [1.13]	URI [1.28]	Skin Viral [0.88]	Diarrhea [0.98]	Epilepsy [1.85]	ASD [1.11]	Iron [6.94]
E Sussex	Dermatitis [1.64]	Neonatal [1.06]	Asthma [1.03]	Congenital [1.08]	URI [1.29]	Diarrhea [0.88]	Skin Viral [0.84]	ASD [1.18]	Iron [4.08]	Urticaria [0.58]
Hampshire	Dermatitis [1.43]	Neonatal [1.01]	Asthma [1.69]	Congenital [1.11]	URI [1.29]	Skin Viral [0.87]	Diarrhea [0.92]	Iron [6.7]	ASD [1.11]	Urticaria [0.64]
Isle of Wight	Dermatitis [1.42]	Neonatal [0.93]	Asthma [1.58]	Congenital [1.06]	URI [1.29]	Skin Viral [0.84]	Diarrhea [0.82]	ASD [1.1]	Iron [3.24]	Urticaria [0.58]

eFigure 9c. Leading ten causes of YLDs with the ratio of observed YLDs to YLDs expected on the basis of Socio-Demographic Index alone in 2017, 1-4 years, both sexes combined. The top ten causes contributing to YLDs are listed globally, by socio-demographic quintile, and then by GBD superregion, region, country, and subnationally where modeled. For each cell, the ratio of observed YLDs to YLDs expected on the basis of socio-demographic index (SDI) alone are listed. Abbreviations: YLD=year of life lived with disability, GBD=Global Burden of Disease.

Values shown in brackets represent the ratio of observed YLDs to predicted YLDs on the basis of Socio-Demographic Index (SDI), rounded to two (2) digits. Color ranges (shown below) were calculated to place a roughly equal number of cells into each bin.

COLOR KEY:	[0.0-0.72]	(0.72-0.84]	(0.84-0.92]	(0.92-1.01]	(1.01-1.09]	(1.09-1.17]	(1.17-1.32]	(1.32-1.67]	1.67+

	1	2	3	4	5	6	7	8	9	10
Kent	Dermatitis [1.63]	Neonatal [0.99]	Asthma [1.1]	Congenital [1.08]	URI [1.29]	ASD [1.55]	Skin Viral [0.85]	Diarrhea [0.85]	Urticaria [0.6]	Iron [3.54]
Medway	Dermatitis [1.44]	Neonatal [1.04]	Asthma [1.06]	Congenital [1.07]	URI [1.29]	Diarrhea [0.89]	Skin Viral [0.84]	ASD [1.1]	Iron [3.12]	Urticaria [0.57]
Milton Keynes	Dermatitis [1.43]	Neonatal [0.92]	Asthma [1.19]	Congenital [1.12]	URI [1.27]	Diarrhea [1.01]	Skin Viral [0.88]	ASD [1.11]	Iron [6.08]	Urticaria [0.66]
Oxfordshire	Dermatitis [1.79]	Neonatal [0.98]	Asthma [1.23]	Congenital [1.15]	URI [1.27]	Skin Viral [0.9]	Diarrhea [0.9]	ASD [1.11]	Urticaria [0.7]	Falls [0.87]
Portsmouth	Dermatitis [1.43]	Neonatal [1.01]	Asthma [1.17]	Congenital [1.1]	URI [1.27]	Skin Viral [0.88]	Diarrhea [0.99]	ASD [1.1]	Urticaria [0.65]	Falls [0.92]
Reading	Dermatitis [1.79]	Neonatal [1.27]	Asthma [1.29]	Congenital [1.17]	URI [1.26]	Skin Viral [0.91]	Diarrhea [1.02]	ASD [1.1]	Iron [8.64]	Urticaria [0.73]
Slough	Dermatitis [1.43]	Neonatal [1.1]	Asthma [1.21]	Congenital [1.12]	URI [1.27]	Diarrhea [1.03]	Skin Viral [0.88]	ASD [1.1]	Urticaria [0.66]	Falls [0.93]
Southampton	Dermatitis [1.43]	Neonatal [1.0]	Asthma [1.17]	Congenital [1.12]	URI [1.28]	Skin Viral [0.87]	Diarrhea [0.94]	ASD [1.11]	Urticaria [0.66]	Falls [0.93]
Surrey	Dermatitis [1.61]	Neonatal [1.16]	Asthma [1.24]	Congenital [1.16]	URI [1.28]	Skin Viral [0.9]	ASD [1.12]	Diarrhea [0.89]	Iron [7.35]	Urticaria [0.7]
West Berkshire	Dermatitis [1.43]	Neonatal [0.97]	Asthma [1.21]	Congenital [1.14]	URI [1.27]	Skin Viral [0.88]	Diarrhea [0.92]	ASD [1.12]	Urticaria [0.68]	Falls [0.91]
W Sussex	Dermatitis [1.48]	Neonatal [1.02]	Asthma [1.29]	Congenital [1.11]	URI [1.29]	Skin Viral [0.86]	Diarrhea [0.87]	ASD [1.11]	Urticaria [0.63]	Falls [0.97]
Windsor & Maidenhead	Dermatitis [1.43]	Neonatal [1.03]	Asthma [1.26]	Congenital [1.17]	URI [1.27]	Skin Viral [0.9]	Diarrhea [1.01]	ASD [1.12]	Urticaria [0.72]	Falls [0.84]
Wokingham	Dermatitis [1.43]	Neonatal [1.05]	Asthma [1.24]	Congenital [1.17]	URI [1.27]	Skin Viral [0.9]	Diarrhea [0.98]	ASD [1.13]	Iron [8.37]	Urticaria [0.7]
SW England	Dermatitis [1.44]	Neonatal [1.07]	Asthma [1.13]	Congenital [1.01]	URI [1.28]	Skin Viral [0.86]	Diarrhea [0.88]	ASD [1.15]	Iron [5.66]	Urticaria [0.62]
Bath & NE Somerset	Dermatitis [1.43]	Neonatal [1.44]	Asthma [1.22]	Congenital [1.04]	URI [1.27]	Skin Viral [0.89]	ASD [1.32]	Diarrhea [0.99]	Iron [6.35]	Urticaria [0.68]
Bournemouth	Dermatitis [1.43]	Neonatal [1.08]	Asthma [1.17]	Congenital [1.03]	URI [1.28]	Skin Viral [0.88]	Diarrhea [0.95]	Iron [7.49]	ASD [1.1]	Urticaria [0.66]
Bristol	Dermatitis [1.43]	Neonatal [1.27]	Asthma [1.26]	Congenital [1.06]	URI [1.27]	Skin Viral [0.9]	ASD [1.31]	Diarrhea [0.88]	Urticaria [0.71]	Falls [0.84]
Cornwall	Dermatitis [1.44]	Neonatal [1.04]	Asthma [1.06]	Congenital [0.99]	URI [1.29]	Diarrhea [0.89]	Iron [5.03]	Skin Viral [0.84]	ASD [1.11]	Urticaria [0.58]
Devon	Dermatitis [1.44]	Neonatal [0.96]	Asthma [1.1]	Congenital [1.0]	URI [1.28]	Iron [7.6]	Skin Viral [0.85]	Diarrhea [0.9]	ASD [1.11]	Urticaria [0.61]
Dorset	Dermatitis [1.44]	Neonatal [1.02]	Asthma [1.09]	Congenital [0.99]	URI [1.29]	Skin Viral [0.85]	Diarrhea [0.86]	ASD [1.1]	Urticaria [0.6]	Falls [1.05]
Gloucestershire	Dermatitis [1.43]	Neonatal [0.99]	Asthma [1.15]	Congenital [1.01]	URI [1.28]	Iron [9.51]	Diarrhea [0.96]	Skin Viral [0.87]	ASD [1.1]	Urticaria [0.64]
N Somerset	Dermatitis [1.44]	Neonatal [1.14]	Asthma [1.1]	Congenital [1.01]	URI [1.28]	Skin Viral [0.85]	ASD [1.32]	Diarrhea [0.74]	Urticaria [0.6]	Iron [3.41]
Plymouth	Dermatitis [1.44]	Neonatal [1.05]	Asthma [1.1]	Congenital [1.0]	URI [1.28]	Diarrhea [0.95]	Skin Viral [0.85]	ASD [1.1]	Urticaria [0.61]	Falls [1.0]
Poole	Dermatitis [1.43]	Neonatal [1.05]	Asthma [1.13]	Congenital [1.01]	URI [1.28]	Skin Viral [0.86]	Diarrhea [0.9]	ASD [1.12]	Iron [4.01]	Urticaria [0.62]
Somerset	Dermatitis [1.44]	Neonatal [1.08]	Asthma [1.07]	Congenital [0.98]	URI [1.3]	Iron [6.08]	Skin Viral [0.84]	Diarrhea [0.84]	ASD [1.1]	Urticaria [0.58]
S Gloucestershire	Dermatitis [1.43]	Neonatal [1.13]	Asthma [1.2]	Congenital [1.03]	URI [1.28]	Skin Viral [0.88]	ASD [1.3]	Diarrhea [0.78]	Urticaria [0.66]	Falls [0.9]
Swindon	Dermatitis [1.44]	Neonatal [0.79]	Asthma [1.15]	Congenital [1.0]	URI [1.28]	Skin Viral [0.86]	Diarrhea [0.87]	ASD [1.11]	Urticaria [0.63]	Falls [0.97]
Torbay	Dermatitis [1.46]	Neonatal [0.85]	Asthma [1.03]	Congenital [0.97]	URI [1.3]	Iron [4.39]	Skin Viral [0.83]	Diarrhea [0.77]	ASD [1.09]	Urticaria [0.55]
Wiltshire	Dermatitis [1.44]	Neonatal [1.2]	Asthma [1.1]	Congenital [0.99]	URI [1.28]	Skin Viral [0.85]	Diarrhea [0.79]	ASD [1.1]	Iron [4.17]	Urticaria [0.6]
W Midlands	Dermatitis [1.44]	Neonatal [1.0]	Asthma [1.11]	Congenital [1.07]	URI [1.28]	Diarrhea [0.94]	Skin Viral [0.85]	ASD [1.02]	Falls [1.07]	Urticaria [0.6]
Birmingham	Dermatitis [1.44]	Neonatal [0.99]	Asthma [1.13]	Congenital [1.09]	URI [1.27]	Diarrhea [0.97]	Skin Viral [0.86]	ASD [0.81]	Falls [1.05]	Urticaria [0.62]

eFigure 9c. Leading ten causes of YLDs with the ratio of observed YLDs to YLDs expected on the basis of Socio-Demographic Index alone in 2017, 1-4 years, both sexes combined.
The top ten causes contributing to YLDs are listed globally, by socio-demographic quintile, and then by GBD superregion, region, country, and subnationally where modeled. For each cell, the ratio of observed YLDs to YLDs expected on the basis of socio-demographic index (SDI) alone are listed. Abbreviations: YLD=year of life lived with disability, GBD=Global Burden of Disease.

Values shown in brackets represent the ratio of observed YLDs to predicted YLDs on the basis of Socio-Demographic Index (SDI), rounded to two (2) digits. Color ranges (shown below) were calculated to place a roughly equal number of cells into each bin.

COLOR KEY:	[0.0-0.72]	(0.72-0.84]	(0.84-0.92]	(0.92-1.01]	(1.01-1.09]	(1.09-1.17]	(1.17-1.32]	(1.32-1.67]	1.67+

	1	2	3	4	5	6	7	8	9	10
Coventry	Dermatitis [1.43]	Neonatal [0.97]	Asthma [1.16]	Congenital [1.09]	URI [1.27]	Skin Viral [0.86]	Diarrhea [0.87]	ASD [1.11]	Urticaria [0.64]	Falls [0.96]
Dudley	Dermatitis [1.45]	Neonatal [1.07]	Asthma [1.05]	Congenital [1.04]	URI [1.29]	Diarrhea [0.92]	Skin Viral [0.84]	ASD [1.1]	Urticaria [0.56]	Falls [1.18]
Herefordshire	Dermatitis [1.43]	Neonatal [1.03]	Asthma [1.11]	Congenital [1.07]	URI [1.28]	Diarrhea [0.96]	Skin Viral [0.85]	ASD [1.11]	Urticaria [0.6]	Falls [1.03]
Sandwell	Dermatitis [1.45]	Neonatal [1.04]	Asthma [1.06]	Congenital [1.03]	URI [1.29]	Diarrhea [0.92]	Skin Viral [0.83]	ASD [1.1]	Urticaria [0.56]	Falls [1.21]
Shropshire	Dermatitis [1.44]	Neonatal [0.99]	Asthma [1.11]	Congenital [1.06]	URI [1.28]	Diarrhea [0.92]	Skin Viral [0.85]	ASD [1.11]	Urticaria [0.6]	Falls [1.03]
Solihull	Dermatitis [1.43]	Neonatal [0.76]	Asthma [1.17]	Congenital [1.1]	URI [1.28]	Skin Viral [0.87]	Diarrhea [0.89]	ASD [1.11]	Falls [0.98]	Urticaria [0.65]
Staffordshire	Dermatitis [1.44]	Neonatal [0.97]	Asthma [1.1]	Congenital [1.06]	URI [1.29]	Diarrhea [1.02]	Skin Viral [0.85]	ASD [1.04]	Falls [1.09]	Urticaria [0.6]
Stoke-on-Trent	Dermatitis [1.45]	Neonatal [1.14]	Asthma [1.06]	Congenital [1.05]	URI [1.28]	Diarrhea [1.0]	Skin Viral [0.84]	ASD [1.09]	Falls [1.22]	Urticaria [0.57]
Telford & Wrekin	Dermatitis [1.44]	Neonatal [0.99]	Asthma [1.1]	Congenital [1.05]	URI [1.28]	Diarrhea [0.92]	Skin Viral [0.84]	Iron [5.17]	ASD [1.1]	Urticaria [0.59]
Walsall	Dermatitis [1.45]	Neonatal [0.97]	Asthma [1.04]	Congenital [1.03]	URI [1.29]	Diarrhea [0.81]	Skin Viral [0.83]	ASD [1.1]	Iron [2.23]	Urticaria [0.55]
Warwickshire	Dermatitis [1.43]	Neonatal [1.03]	Asthma [1.17]	Congenital [1.09]	URI [1.28]	Skin Viral [0.87]	Diarrhea [0.89]	ASD [1.1]	Urticaria [0.65]	Falls [0.97]
Wolverhampton	Dermatitis [1.45]	Neonatal [0.73]	Asthma [1.07]	Congenital [1.05]	URI [1.28]	Diarrhea [1.0]	Skin Viral [0.84]	ASD [1.09]	Urticaria [0.57]	Falls [1.12]
Worcestershire	Dermatitis [1.43]	Neonatal [1.11]	Asthma [1.12]	Congenital [1.07]	URI [1.28]	Skin Viral [0.85]	Diarrhea [0.84]	ASD [1.11]	Urticaria [0.61]	Falls [1.04]
Yorkshire & Humber	Dermatitis [1.44]	Neonatal [0.94]	Asthma [1.13]	Congenital [1.07]	URI [1.28]	Diarrhea [0.97]	Skin Viral [0.85]	ASD [1.1]	Iron [3.96]	Urticaria [0.6]
Barnsley	Dermatitis [1.46]	Neonatal [0.96]	Asthma [1.06]	Congenital [1.02]	URI [1.3]	Diarrhea [0.95]	Skin Viral [0.83]	ASD [1.11]	Iron [2.57]	Urticaria [0.54]
Bradford	Dermatitis [1.45]	Neonatal [1.0]	Asthma [1.08]	Congenital [1.05]	URI [1.29]	Diarrhea [0.92]	Skin Viral [0.84]	ASD [1.1]	Urticaria [0.57]	Iron [2.4]
Calderdale	Dermatitis [1.44]	Neonatal [0.91]	Asthma [1.12]	Congenital [1.06]	URI [1.28]	Diarrhea [0.99]	Skin Viral [0.85]	ASD [1.08]	Iron [3.7]	Urticaria [0.6]
Doncaster	Dermatitis [1.46]	Neonatal [1.17]	Asthma [1.05]	Congenital [1.04]	URI [1.3]	Diarrhea [0.9]	Skin Viral [0.83]	ASD [1.1]	Urticaria [0.55]	Iron [1.93]
E Riding of Yorkshire	Dermatitis [1.44]	Neonatal [0.92]	Asthma [1.1]	Congenital [1.06]	URI [1.29]	Diarrhea [0.88]	Skin Viral [0.84]	ASD [1.11]	Urticaria [0.59]	Falls [1.07]
Kingston upon Hull	Dermatitis [1.44]	Neonatal [0.86]	Asthma [1.11]	Congenital [1.05]	URI [1.29]	Diarrhea [0.92]	Skin Viral [0.84]	ASD [1.1]	Iron [3.82]	Urticaria [0.58]
Kirklees	Dermatitis [1.45]	Neonatal [0.87]	Asthma [1.1]	Congenital [1.06]	URI [1.28]	Diarrhea [0.98]	Skin Viral [0.84]	Iron [4.88]	ASD [1.09]	Urticaria [0.58]
Leeds	Dermatitis [1.43]	Neonatal [0.89]	Asthma [1.23]	Congenital [1.12]	URI [1.27]	Skin Viral [0.89]	Diarrhea [0.96]	ASD [1.1]	Urticaria [0.68]	Iron [5.38]
NE Lincolnshire	Dermatitis [1.45]	Neonatal [0.89]	Asthma [1.09]	Congenital [1.05]	URI [1.29]	Skin Viral [0.84]	Diarrhea [0.78]	ASD [1.1]	Urticaria [0.57]	Falls [1.12]
N Lincolnshire	Dermatitis [1.44]	Neonatal [0.83]	Asthma [1.09]	Congenital [1.05]	URI [1.29]	Diarrhea [0.87]	Skin Viral [0.84]	ASD [1.1]	Iron [3.46]	Urticaria [0.57]
N Yorkshire	Dermatitis [1.43]	Neonatal [1.06]	Asthma [1.15]	Congenital [1.08]	URI [1.28]	Diarrhea [1.06]	Skin Viral [0.86]	ASD [1.11]	Urticaria [0.62]	Falls [0.99]
Rotherham	Dermatitis [1.45]	Neonatal [0.84]	Asthma [1.07]	Congenital [1.04]	URI [1.29]	Diarrhea [0.88]	Skin Viral [0.83]	ASD [1.11]	Iron [2.6]	Urticaria [0.55]
Sheffield	Dermatitis [1.43]	Neonatal [1.01]	Asthma [1.18]	Congenital [1.1]	URI [1.28]	Diarrhea [1.02]	Skin Viral [0.87]	ASD [1.11]	Iron [6.22]	Urticaria [0.65]
Wakefield	Dermatitis [1.45]	Neonatal [0.74]	Asthma [1.09]	Congenital [1.04]	URI [1.29]	Diarrhea [1.01]	Skin Viral [0.84]	ASD [1.1]	Iron [2.87]	Urticaria [0.57]
York	Dermatitis [1.43]	Neonatal [0.9]	Asthma [1.26]	Congenital [1.13]	URI [1.26]	Diarrhea [1.06]	Skin Viral [0.9]	ASD [1.1]	Urticaria [0.7]	Falls [0.85]
N Ireland	Dermatitis [1.19]	Neonatal [0.88]	Asthma [0.99]	Congenital [1.03]	URI [1.29]	Skin Viral [0.94]	Diarrhea [0.75]	ASD [1.03]	Falls [0.98]	Urticaria [0.56]
Scotland	Dermatitis [1.29]	Neonatal [0.82]	Asthma [1.02]	Congenital [1.2]	URI [1.3]	Skin Viral [0.91]	Diarrhea [0.68]	ASD [1.04]	Iron [2.56]	Urticaria [0.52]

eFigure 9c. Leading ten causes of YLDs with the ratio of observed YLDs to YLDs expected on the basis of Socio-Demographic Index alone in 2017, 1-4 years, both sexes combined.
The top ten causes contributing to YLDs are listed globally, by socio-demographic quintile, and then by GBD superregion, region, country, and subnationally where modeled. For each cell, the ratio of observed YLDs to YLDs expected on the basis of socio-demographic index (SDI) alone are listed. Abbreviations: YLD=year of life lived with disability, GBD=Global Burden of Disease.

Values shown in brackets represent the ratio of observed YLDs to predicted YLDs on the basis of Socio-Demographic Index (SDI), rounded to two (2) digits. Color ranges (shown below) were calculated to place a roughly equal number of cells into each bin.

COLOR KEY:	[0.0-0.72]	(0.72-0.84]	(0.84-0.92]	(0.92-1.01]	(1.01-1.09]	(1.09-1.17]	(1.17-1.32]	(1.32-1.67]	1.67+

	1	2	3	4	5	6	7	8	9	10
Wales	Dermatitis [1.21]	Neonatal [0.82]	Asthma [1.02]	Congenital [1.02]	URI [1.31]	Skin Viral [1.02]	Diarrhea [0.74]	ASD [0.98]	Falls [1.12]	Vit A [1.03]
**Latin America and Caribbean**	Neonatal [1.13]	Asthma [1.32]	Dermatitis [0.88]	Diarrhea [1.36]	Vit A [0.85]	Iron [0.98]	Urticaria [0.91]	Congenital [0.75]	Epilepsy [1.28]	Skin Viral [0.82]
**Andean Latin America**	Neonatal [1.09]	Dermatitis [1.43]	Asthma [1.81]	Iron [1.92]	Diarrhea [1.46]	Vit A [0.79]	Urticaria [0.88]	Congenital [0.68]	URI [1.06]	Skin Viral [0.82]
Bolivia	Iron [2.5]	Neonatal [1.06]	Asthma [1.98]	Dermatitis [1.53]	Diarrhea [1.42]	Vit A [0.72]	Urticaria [0.87]	Congenital [0.7]	Skin Viral [0.85]	URI [0.97]
Ecuador	Neonatal [1.12]	Dermatitis [1.3]	Asthma [1.66]	Diarrhea [1.14]	Urticaria [0.89]	Congenital [0.69]	Vit A [0.52]	Epilepsy [1.24]	Skin Viral [0.82]	URI [1.01]
Peru	Neonatal [1.1]	Dermatitis [1.47]	Asthma [1.8]	Iron [2.1]	Diarrhea [1.57]	Vit A [0.88]	Urticaria [0.89]	Congenital [0.68]	URI [1.12]	Skin Viral [0.82]
**Caribbean**	Asthma [2.08]	Neonatal [0.94]	Iron [2.5]	Dermatitis [1.11]	Diarrhea [1.41]	Vit A [1.16]	Urticaria [0.89]	Congenital [0.71]	Skin Viral [0.81]	URI [0.91]
Antigua	Asthma [2.4]	Neonatal [0.95]	Dermatitis [1.03]	Iron [4.03]	Diarrhea [0.92]	Vit A [0.95]	Urticaria [0.98]	Congenital [0.66]	Skin Viral [0.81]	URI [0.87]
Bahamas	Asthma [2.6]	Neonatal [0.98]	Dermatitis [1.01]	Diarrhea [1.12]	Urticaria [1.06]	Congenital [0.67]	Skin Viral [0.81]	URI [0.85]	Iron [1.71]	Epilepsy [1.19]
Barbados	Asthma [2.25]	Neonatal [1.04]	Dermatitis [1.0]	Diarrhea [1.11]	Urticaria [1.03]	Congenital [0.66]	Skin Viral [0.81]	URI [0.86]	Iron [1.32]	Epilepsy [1.08]
Belize	Asthma [2.23]	Neonatal [0.96]	Dermatitis [1.19]	Iron [1.18]	Vit A [0.76]	Urticaria [0.87]	Diarrhea [0.57]	Congenital [0.68]	Skin Viral [0.84]	URI [0.98]
Bermuda	Asthma [3.24]	Neonatal [1.02]	Dermatitis [0.98]	Diarrhea [1.46]	Urticaria [1.17]	Congenital [0.67]	Skin Viral [0.83]	Iron [4.08]	URI [0.83]	Epilepsy [1.26]
Cuba	Asthma [2.42]	Neonatal [0.88]	Dermatitis [1.06]	Iron [1.55]	Urticaria [0.94]	Congenital [0.65]	Diarrhea [0.63]	Skin Viral [0.81]	URI [0.9]	Scabies [2.29]
Dominica	Neonatal [1.1]	Asthma [2.19]	Dermatitis [1.07]	Iron [1.78]	Diarrhea [0.81]	Urticaria [0.94]	Congenital [0.69]	Skin Viral [0.81]	Epilepsy [1.19]	URI [0.89]
Dominican Rep	Neonatal [0.99]	Asthma [1.57]	Dermatitis [1.2]	Diarrhea [1.61]	Vit A [0.62]	Urticaria [0.87]	Congenital [0.71]	Skin Viral [0.84]	Iron [0.4]	Epilepsy [0.82]
Grenada	Asthma [2.03]	Neonatal [1.01]	Dermatitis [1.11]	Iron [2.28]	Diarrhea [1.14]	Vit A [0.78]	Urticaria [0.89]	Congenital [0.67]	Skin Viral [0.82]	URI [0.93]
Guyana	Asthma [2.03]	Neonatal [1.02]	Dermatitis [1.2]	Iron [1.11]	Vit A [0.57]	Diarrhea [0.75]	Urticaria [0.87]	Congenital [0.69]	Skin Viral [0.85]	URI [0.95]
Haiti	Iron [1.47]	Asthma [2.16]	Vit A [0.8]	Neonatal [0.98]	Dermatitis [1.25]	Diarrhea [1.21]	Congenital [0.74]	Urticaria [0.89]	Skin Viral [0.87]	URI [1.1]
Jamaica	Asthma [2.31]	Neonatal [1.02]	Dermatitis [1.07]	Iron [2.02]	Vit A [0.98]	Urticaria [0.93]	Congenital [0.66]	Diarrhea [0.63]	Skin Viral [0.81]	Epilepsy [1.17]
Puerto Rico	Asthma [3.96]	Neonatal [1.08]	Dermatitis [0.97]	Diarrhea [1.4]	Urticaria [1.19]	Congenital [0.73]	Skin Viral [0.83]	URI [0.83]	Epilepsy [1.26]	Vit A [1.39]
St Lucia	Asthma [2.2]	Neonatal [1.03]	Dermatitis [1.1]	Diarrhea [1.18]	Urticaria [0.91]	Congenital [0.66]	Iron [0.72]	Skin Viral [0.81]	Vit A [0.49]	URI [0.91]
St Vincent	Asthma [2.1]	Neonatal [0.97]	Dermatitis [1.17]	Vit A [0.93]	Iron [0.76]	Diarrhea [0.7]	Urticaria [0.88]	Congenital [0.67]	Skin Viral [0.83]	URI [0.96]
Suriname	Asthma [2.27]	Neonatal [1.01]	Dermatitis [1.12]	Vit A [0.64]	Urticaria [0.89]	Congenital [0.69]	Iron [0.69]	Diarrhea [0.58]	Skin Viral [0.82]	URI [0.93]
Trinidad Tobago	Asthma [2.49]	Neonatal [1.02]	Dermatitis [1.05]	Urticaria [0.96]	Congenital [0.66]	Diarrhea [0.64]	Skin Viral [0.81]	URI [0.89]	Iron [0.87]	Epilepsy [1.02]
Virgin Isl US	Asthma [2.91]	Neonatal [1.04]	Dermatitis [0.98]	Diarrhea [1.63]	Urticaria [1.17]	Congenital [0.66]	Skin Viral [0.83]	URI [0.83]	Epilepsy [1.32]	Vit A [1.35]
**Central Latin America**	Neonatal [1.34]	Diarrhea [1.49]	Dermatitis [0.78]	Asthma [0.87]	Vit A [0.67]	Epilepsy [1.59]	Urticaria [0.9]	Congenital [0.71]	Iron [0.54]	Skin Viral [0.82]
Colombia	Neonatal [1.36]	Dermatitis [0.86]	Diarrhea [1.38]	Asthma [0.95]	Epilepsy [1.61]	Urticaria [0.89]	URI [1.16]	Congenital [0.66]	Skin Viral [0.82]	Vit A [0.37]
Costa Rica	Neonatal [1.25]	Asthma [1.38]	Diarrhea [1.69]	Dermatitis [0.81]	Urticaria [0.91]	Epilepsy [1.47]	Congenital [0.64]	Vit A [0.54]	Skin Viral [0.81]	URI [0.92]
El Salvador	Neonatal [1.35]	Diarrhea [1.56]	Asthma [1.2]	Dermatitis [0.82]	Vit A [0.53]	Urticaria [0.87]	Epilepsy [1.32]	Congenital [0.72]	Skin Viral [0.85]	Iron [0.41]
Guatemala	Neonatal [1.32]	Diarrhea [2.09]	Dermatitis [1.01]	Iron [0.66]	Vit A [0.55]	Asthma [0.8]	Urticaria [0.88]	Congenital [0.7]	Skin Viral [0.87]	Epilepsy [0.96]
Honduras	Neonatal [1.45]	Diarrhea [1.68]	Dermatitis [1.01]	Asthma [1.21]	Vit A [0.55]	Epilepsy [1.41]	Iron [0.32]	Urticaria [0.88]	Congenital [0.69]	Skin Viral [0.87]

eFigure 9c. Leading ten causes of YLDs with the ratio of observed YLDs to YLDs expected on the basis of Socio-Demographic Index alone in 2017, 1-4 years, both sexes combined.
The top ten causes contributing to YLDs are listed globally, by socio-demographic quintile, and then by GBD superregion, region, country, and subnationally where modeled. For each cell, the ratio of observed YLDs to YLDs expected on the basis of socio-demographic index (SDI) alone are listed. Abbreviations: YLD=year of life lived with disability, GBD=Global Burden of Disease.

Values shown in brackets represent the ratio of observed YLDs to predicted YLDs on the basis of Socio-Demographic Index (SDI), rounded to two (2) digits. Color ranges (shown below) were calculated to place a roughly equal number of cells into each bin.

COLOR KEY:	[0.0-0.72]	(0.72-0.84]	(0.84-0.92]	(0.92-1.01]	(1.01-1.09]	(1.09-1.17]	(1.17-1.32]	(1.32-1.67]	1.67+

	1	2	3	4	5	6	7	8	9	10
Mexico	Neonatal [1.33]	Dermatitis [0.65]	Diarrhea [1.08]	Vit A [0.88]	Asthma [0.75]	Epilepsy [1.78]	Urticaria [0.93]	Congenital [0.74]	Iron [0.64]	Skin Viral [0.82]
Aguascalientes	Neonatal [1.35]	Dermatitis [0.64]	Diarrhea [1.14]	Asthma [0.87]	Epilepsy [1.9]	Congenital [0.81]	Urticaria [0.95]	Vit A [0.68]	Skin Viral [0.81]	URI [0.92]
Baja CA	Neonatal [1.31]	Dermatitis [0.64]	Vit A [0.92]	Diarrhea [0.9]	Epilepsy [1.84]	Asthma [0.65]	Urticaria [0.95]	Congenital [0.74]	Skin Viral [0.81]	URI [0.93]
Baja CA Sur	Neonatal [1.4]	Diarrhea [1.27]	Dermatitis [0.64]	Vit A [1.12]	Asthma [0.84]	Epilepsy [1.86]	Urticaria [0.95]	Congenital [0.76]	Skin Viral [0.81]	URI [0.92]
Campeche	Neonatal [1.44]	Vit A [0.95]	Diarrhea [1.14]	Dermatitis [0.67]	Asthma [0.76]	Epilepsy [1.64]	Urticaria [0.92]	Congenital [0.73]	Iron [0.55]	Skin Viral [0.83]
Chiapas	Neonatal [1.35]	Diarrhea [1.24]	Vit A [0.76]	Dermatitis [0.72]	Asthma [0.74]	Epilepsy [1.58]	Urticaria [0.92]	Congenital [0.69]	Iron [0.28]	Skin Viral [0.86]
Chihuahua	Neonatal [1.24]	Diarrhea [1.23]	Asthma [0.87]	Dermatitis [0.56]	Vit A [0.83]	Epilepsy [1.84]	Congenital [0.82]	Urticaria [0.94]	Skin Viral [0.81]	URI [0.94]
Coahuila	Neonatal [1.14]	Diarrhea [1.45]	Dermatitis [0.65]	Asthma [0.87]	Epilepsy [1.82]	Vit A [0.74]	Urticaria [0.94]	Congenital [0.71]	Skin Viral [0.81]	URI [0.93]
Colima	Neonatal [1.43]	Dermatitis [0.64]	Asthma [0.8]	Vit A [0.84]	Diarrhea [0.89]	Epilepsy [1.87]	Urticaria [0.95]	Congenital [0.7]	Skin Viral [0.81]	URI [0.92]
Mexico City	Neonatal [1.44]	Dermatitis [0.77]	Diarrhea [1.17]	Epilepsy [2.3]	Vit A [1.26]	Asthma [0.69]	Congenital [0.8]	Urticaria [1.03]	Skin Viral [0.8]	URI [0.88]
Durango	Neonatal [1.2]	Dermatitis [0.86]	Diarrhea [1.11]	Asthma [0.84]	Epilepsy [1.76]	Vit A [0.6]	Urticaria [0.92]	Congenital [0.74]	Iron [0.66]	Skin Viral [0.82]
Guanajuato	Neonatal [1.2]	Vit A [0.99]	Dermatitis [0.67]	Asthma [0.83]	Diarrhea [0.91]	Epilepsy [1.78]	Urticaria [0.92]	Congenital [0.75]	ASD [1.54]	Skin Viral [0.82]
Guerrero	Neonatal [1.42]	Dermatitis [0.71]	Diarrhea [0.97]	Asthma [0.76]	Vit A [0.54]	Epilepsy [1.59]	Iron [0.41]	Urticaria [0.92]	Congenital [0.7]	Skin Viral [0.9]
Hidalgo	Neonatal [1.61]	Dermatitis [0.7]	Asthma [0.79]	Diarrhea [0.77]	Epilepsy [1.65]	Vit A [0.49]	Urticaria [0.91]	Congenital [0.73]	Skin Viral [0.85]	Iron [0.38]
Jalisco	Neonatal [1.3]	Dermatitis [0.65]	Vit A [0.95]	Epilepsy [2.19]	Diarrhea [0.95]	Asthma [0.67]	Congenital [0.79]	Urticaria [0.94]	Skin Viral [0.81]	URI [0.93]
Mexico	Neonatal [1.56]	Vit A [1.23]	Iron [1.25]	Diarrhea [1.11]	Dermatitis [0.57]	Epilepsy [1.76]	Asthma [0.64]	Urticaria [0.93]	Congenital [0.67]	Skin Viral [0.82]
Michoacan	Neonatal [1.15]	Dermatitis [0.7]	Asthma [0.79]	Vit A [0.59]	Diarrhea [0.78]	Epilepsy [1.61]	Urticaria [0.91]	Congenital [0.72]	Skin Viral [0.85]	Iron [0.36]
Morelos	Neonatal [1.3]	Dermatitis [0.56]	Asthma [0.71]	Vit A [0.76]	Epilepsy [1.8]	Diarrhea [0.8]	Congenital [0.79]	Urticaria [0.93]	Iron [0.61]	Skin Viral [0.82]
Nayarit	Neonatal [1.43]	Dermatitis [0.67]	Diarrhea [1.12]	Asthma [0.79]	Epilepsy [1.68]	Urticaria [0.92]	Congenital [0.76]	Vit A [0.43]	Skin Viral [0.82]	URI [0.96]
Nuevo Leon	Neonatal [1.1]	Diarrhea [1.21]	Dermatitis [0.54]	Epilepsy [1.96]	Asthma [0.66]	Vit A [0.79]	Urticaria [0.97]	Congenital [0.74]	Iron [0.98]	Skin Viral [0.81]
Oaxaca	Neonatal [1.33]	Diarrhea [1.14]	Dermatitis [0.71]	Asthma [0.76]	Vit A [0.51]	Epilepsy [1.71]	Urticaria [0.92]	Congenital [0.78]	Skin Viral [0.86]	Iron [0.29]
Puebla	Neonatal [1.25]	Dermatitis [0.7]	Diarrhea [0.96]	Asthma [0.79]	Vit A [0.59]	Urticaria [0.91]	Congenital [0.74]	Epilepsy [1.17]	Skin Viral [0.85]	Iron [0.35]
Queretaro	Neonatal [1.4]	Dermatitis [0.65]	Vit A [0.93]	Asthma [0.83]	Iron [1.0]	Epilepsy [1.79]	Diarrhea [0.8]	Urticaria [0.93]	Congenital [0.72]	Skin Viral [0.82]
Quintana Roo	Neonatal [1.67]	Diarrhea [1.41]	Dermatitis [0.67]	Vit A [0.88]	Asthma [0.76]	Epilepsy [1.77]	Urticaria [0.92]	Congenital [0.77]	Skin Viral [0.82]	URI [0.95]
San Luis Potosi	Neonatal [1.16]	Vit A [1.06]	Dermatitis [0.67]	Asthma [0.82]	Diarrhea [0.89]	Epilepsy [1.8]	Congenital [0.81]	Urticaria [0.92]	Skin Viral [0.83]	URI [0.96]
Sinaloa	Neonatal [1.22]	Vit A [1.31]	Dermatitis [0.64]	Diarrhea [1.15]	Asthma [0.8]	Epilepsy [1.78]	Iron [0.97]	Urticaria [0.94]	Congenital [0.7]	Skin Viral [0.81]
Sonora	Neonatal [1.19]	Vit A [1.19]	Dermatitis [0.64]	Diarrhea [1.13]	Asthma [0.84]	Epilepsy [1.87]	Congenital [0.8]	Urticaria [0.94]	Iron [0.69]	Skin Viral [0.81]
Tabasco	Neonatal [1.44]	Diarrhea [1.22]	Dermatitis [0.67]	Vit A [0.79]	Iron [0.92]	Asthma [0.69]	Epilepsy [1.71]	Congenital [0.83]	Urticaria [0.92]	Skin Viral [0.83]
Tamaulipas	Neonatal [1.46]	Vit A [1.24]	Diarrhea [1.34]	Asthma [0.79]	Dermatitis [0.54]	Epilepsy [1.77]	Urticaria [0.94]	Congenital [0.75]	Iron [0.74]	Skin Viral [0.81]
Tlaxcala	Neonatal [1.42]	Dermatitis [0.69]	Asthma [0.81]	Diarrhea [0.95]	Epilepsy [1.76]	Congenital [0.81]	Urticaria [0.92]	Vit A [0.45]	Iron [0.48]	Skin Viral [0.83]
Veracruz	Neonatal [1.41]	Dermatitis [0.7]	Iron [0.83]	Diarrhea [1.0]	Asthma [0.75]	Epilepsy [1.68]	Vit A [0.54]	Urticaria [0.91]	Congenital [0.77]	Skin Viral [0.84]

eFigure 9c. Leading ten causes of YLDs with the ratio of observed YLDs to YLDs expected on the basis of Socio-Demographic Index alone in 2017, 1-4 years, both sexes combined. The top ten causes contributing to YLDs are listed globally, by socio-demographic quintile, and then by GBD superregion, region, country, and subnationally where modeled. For each cell, the ratio of observed YLDs to YLDs expected on the basis of socio-demographic index (SDI) alone are listed. Abbreviations: YLD=year of life lived with disability, GBD=Global Burden of Disease.

Values shown in brackets represent the ratio of observed YLDs to predicted YLDs on the basis of Socio-Demographic Index (SDI), rounded to two (2) digits. Color ranges (shown below) were calculated to place a roughly equal number of cells into each bin.

COLOR KEY:	[0.0-0.72]	(0.72-0.84]	(0.84-0.92]	(0.92-1.01]	(1.01-1.09]	(1.09-1.17]	(1.17-1.32]	(1.32-1.67]	1.67+

	1	2	3	4	5	6	7	8	9	10
Yucatan	Neonatal [1.24]	Dermatitis [0.63]	Diarrhea [0.99]	Vit A [0.83]	Asthma [0.73]	Epilepsy [1.77]	Congenital [0.78]	Urticaria [0.93]	Skin Viral [0.82]	URI [0.94]
Zacatecas	Neonatal [0.93]	Dermatitis [0.68]	Asthma [0.83]	Diarrhea [0.9]	Vit A [0.67]	Epilepsy [1.77]	Urticaria [0.92]	Congenital [0.69]	Skin Viral [0.83]	URI [0.98]
Nicaragua	Neonatal [1.35]	Diarrhea [1.48]	Dermatitis [1.01]	Asthma [0.92]	Iron [0.51]	Congenital [0.75]	Urticaria [0.88]	Epilepsy [1.17]	Skin Viral [0.87]	URI [0.88]
Panama	Neonatal [1.24]	Diarrhea [2.19]	Dermatitis [1.01]	Asthma [1.33]	Iron [1.53]	Urticaria [0.92]	Vit A [0.7]	Congenital [0.65]	Skin Viral [0.81]	Epilepsy [1.25]
Venezuela	Neonatal [1.34]	Diarrhea [2.4]	Dermatitis [0.9]	Asthma [1.02]	Epilepsy [1.5]	Urticaria [0.9]	Congenital [0.67]	Skin Viral [0.81]	URI [0.92]	ASD [0.78]
**Tropical Latin America**	Neonatal [0.89]	Asthma [1.62]	Dermatitis [0.77]	Vit A [1.1]	Diarrhea [1.08]	Congenital [0.85]	Urticaria [0.95]	Iron [0.82]	Skin Viral [0.81]	Scabies [2.96]
Brazil	Neonatal [0.89]	Asthma [1.62]	Dermatitis [0.75]	Vit A [1.13]	Diarrhea [1.03]	Congenital [0.85]	Urticaria [0.95]	Iron [0.84]	Skin Viral [0.81]	Scabies [3.06]
Acre	Neonatal [0.93]	Asthma [1.38]	Iron [1.37]	Dermatitis [0.83]	Diarrhea [1.15]	Vit A [0.84]	Congenital [0.99]	Urticaria [0.91]	Skin Viral [0.83]	URI [0.94]
Alagoas	Neonatal [0.79]	Asthma [1.22]	Vit A [0.66]	Diarrhea [1.02]	Dermatitis [0.71]	Iron [0.57]	Congenital [0.87]	Urticaria [0.92]	Skin Viral [0.85]	Scabies [1.87]
Amapa	Neonatal [0.92]	Asthma [1.53]	Dermatitis [0.76]	Vit A [1.04]	Iron [1.29]	Congenital [0.83]	Diarrhea [0.76]	Urticaria [0.95]	Skin Viral [0.81]	Scabies [2.89]
Amazonas	Neonatal [0.79]	Asthma [1.5]	Dermatitis [0.79]	Vit A [0.96]	Diarrhea [0.88]	Iron [0.82]	Congenital [0.84]	Urticaria [0.93]	Skin Viral [0.82]	Scabies [2.51]
Bahia	Neonatal [0.87]	Dermatitis [1.09]	Asthma [1.38]	Vit A [1.09]	Diarrhea [1.21]	Congenital [0.86]	Urticaria [0.91]	Skin Viral [0.84]	Scabies [2.01]	URI [0.95]
Ceara	Neonatal [0.82]	Asthma [1.41]	Dermatitis [0.83]	Vit A [0.92]	Diarrhea [1.02]	Iron [0.68]	Congenital [0.88]	Urticaria [0.91]	Scabies [2.47]	Skin Viral [0.83]
Distrito Federal	Neonatal [1.03]	Asthma [2.02]	Dermatitis [0.68]	Congenital [0.93]	Urticaria [1.18]	Diarrhea [0.94]	Vit A [1.63]	Skin Viral [0.82]	Scabies [7.38]	URI [0.8]
Espirito Santo	Asthma [1.87]	Neonatal [0.87]	Dermatitis [0.75]	Vit A [1.15]	Diarrhea [0.87]	Congenital [0.84]	Iron [1.18]	Urticaria [0.97]	Skin Viral [0.8]	Scabies [3.18]
Goias	Asthma [1.65]	Neonatal [0.8]	Dermatitis [0.77]	Vit A [0.99]	Diarrhea [0.96]	Congenital [0.84]	Urticaria [0.94]	Skin Viral [0.81]	Scabies [2.75]	Iron [0.61]
Maranhao	Vit A [0.95]	Neonatal [0.84]	Asthma [1.23]	Dermatitis [0.86]	Diarrhea [0.85]	Iron [0.32]	Congenital [0.82]	Urticaria [0.92]	Skin Viral [0.86]	Scabies [1.76]
Mato Grosso	Asthma [1.72]	Neonatal [0.84]	Dermatitis [0.76]	Diarrhea [1.02]	Vit A [0.89]	Iron [1.07]	Congenital [0.83]	Urticaria [0.95]	Skin Viral [0.81]	Scabies [2.92]
Mato Grosso do Sul	Neonatal [0.94]	Asthma [1.57]	Dermatitis [0.77]	Vit A [1.3]	Diarrhea [1.08]	Congenital [0.95]	Iron [1.0]	Urticaria [0.94]	Skin Viral [0.81]	Scabies [2.75]
Minas Gerais	Neonatal [0.93]	Asthma [1.72]	Dermatitis [0.66]	Diarrhea [1.14]	Vit A [1.01]	Congenital [0.84]	Urticaria [0.95]	Skin Viral [0.81]	Scabies [2.91]	URI [0.88]
Para	Neonatal [0.82]	Asthma [1.42]	Diarrhea [1.37]	Iron [1.02]	Vit A [0.92]	Dermatitis [0.85]	Congenital [0.84]	Urticaria [0.91]	Scabies [2.1]	Skin Viral [0.84]
Paraiba	Neonatal [0.77]	Asthma [1.31]	Dermatitis [0.85]	Diarrhea [1.12]	Vit A [0.72]	Congenital [0.86]	Iron [0.45]	Urticaria [0.91]	Skin Viral [0.85]	Scabies [1.94]
Parana	Asthma [1.87]	Neonatal [0.91]	Dermatitis [0.67]	Vit A [1.22]	Diarrhea [0.93]	Congenital [0.91]	Urticaria [0.97]	Skin Viral [0.8]	Iron [0.86]	Scabies [3.3]
Pernambuco	Neonatal [0.82]	Asthma [1.47]	Dermatitis [0.81]	Vit A [0.81]	Diarrhea [0.88]	Congenital [0.91]	Urticaria [0.91]	Iron [0.49]	Skin Viral [0.84]	Scabies [2.06]
Piaui	Neonatal [0.89]	Asthma [1.37]	Dermatitis [0.86]	Diarrhea [0.94]	Vit A [0.47]	Congenital [0.88]	Urticaria [0.92]	Skin Viral [0.86]	Scabies [1.85]	URI [0.97]
Rio de Janeiro	Neonatal [1.13]	Asthma [1.71]	Dermatitis [0.73]	Vit A [1.21]	Congenital [0.83]	Urticaria [1.02]	Diarrhea [0.78]	Epilepsy [1.34]	Skin Viral [0.8]	Scabies [4.09]
Rio Grande do Norte	Neonatal [0.75]	Asthma [1.41]	Vit A [1.22]	Dermatitis [0.82]	Diarrhea [1.3]	Congenital [0.84]	Urticaria [0.91]	Skin Viral [0.83]	Iron [0.44]	Scabies [2.13]
Rio Grande do Sul	Asthma [1.99]	Neonatal [0.95]	Dermatitis [0.95]	Congenital [1.4]	Epilepsy [2.23]	Vit A [0.99]	Diarrhea [0.83]	Urticaria [0.99]	Iron [1.21]	Skin Viral [0.8]
Rondonia	Neonatal [0.96]	Asthma [1.44]	Dermatitis [0.81]	Congenital [1.42]	Vit A [0.98]	Diarrhea [0.76]	Urticaria [0.92]	Scabies [2.55]	Skin Viral [0.82]	URI [0.92]
Roraima	Neonatal [0.97]	Asthma [1.47]	Dermatitis [0.78]	Vit A [0.9]	Diarrhea [0.88]	Iron [1.01]	Congenital [0.84]	Urticaria [0.94]	Skin Viral [0.81]	Scabies [2.67]
Santa Catarina	Asthma [2.06]	Neonatal [0.85]	Dermatitis [0.73]	Vit A [1.29]	Congenital [0.89]	Diarrhea [0.83]	Iron [1.39]	Urticaria [1.0]	Skin Viral [0.8]	Scabies [3.8]

eFigure 9c. Leading ten causes of YLDs with the ratio of observed YLDs to YLDs expected on the basis of Socio-Demographic Index alone in 2017, 1-4 years, both sexes combined. The top ten causes contributing to YLDs are listed globally, by socio-demographic quintile, and then by GBD superregion, region, country, and subnationally where modeled. For each cell, the ratio of observed YLDs to YLDs expected on the basis of socio-demographic index (SDI) alone are listed. Abbreviations: YLD=year of life lived with disability, GBD=Global Burden of Disease.

Values shown in brackets represent the ratio of observed YLDs to predicted YLDs on the basis of Socio-Demographic Index (SDI), rounded to two (2) digits. Color ranges (shown below) were calculated to place a roughly equal number of cells into each bin.

COLOR KEY:	[0.0-0.72]	(0.72-0.84]	(0.84-0.92]	(0.92-1.01]	(1.01-1.09]	(1.09-1.17]	(1.17-1.32]	(1.32-1.67]	1.67+

	1	2	3	4	5	6	7	8	9	10
Sao Paulo	Neonatal [0.98]	Asthma [1.91]	Dermatitis [0.63]	Urticaria [1.04]	Congenital [0.72]	Diarrhea [0.77]	Skin Viral [0.8]	Scabies [4.36]	URI [0.84]	Vit A [0.67]
Sergipe	Neonatal [0.95]	Asthma [1.49]	Vit A [1.21]	Dermatitis [0.68]	Diarrhea [0.81]	Congenital [0.84]	Iron [0.66]	Urticaria [0.92]	Skin Viral [0.83]	Scabies [2.25]
Tocantins	Neonatal [0.93]	Asthma [1.41]	Vit A [1.26]	Dermatitis [0.82]	Iron [1.04]	Diarrhea [0.92]	Congenital [0.83]	Urticaria [0.91]	Skin Viral [0.83]	Scabies [2.19]
Paraguay	Asthma [1.83]	Diarrhea [2.11]	Neonatal [0.77]	Dermatitis [1.04]	Vit A [0.84]	Iron [0.86]	Congenital [0.89]	Urticaria [0.88]	Skin Viral [0.83]	Scabies [2.31]
**North Africa and Middle East**	Neonatal [1.01]	Iron [2.44]	Diarrhea [1.89]	Asthma [1.07]	Dermatitis [0.64]	Vit A [0.95]	Congenital [0.84]	Urticaria [0.96]	Epilepsy [1.3]	Skin Viral [0.8]
Afghanistan	Diarrhea [1.52]	Neonatal [1.08]	Vit A [0.4]	Iron [0.36]	Dermatitis [0.94]	Asthma [1.2]	ID [4.19]	Congenital [0.85]	Urticaria [1.03]	Epilepsy [0.9]
Algeria	Neonatal [1.11]	Asthma [1.25]	Diarrhea [1.52]	Dermatitis [0.64]	Iron [1.46]	Congenital [0.82]	Urticaria [1.02]	Epilepsy [1.36]	Vit A [0.66]	Skin Viral [0.79]
Bahrain	Neonatal [1.04]	Dermatitis [0.72]	Asthma [1.17]	Diarrhea [1.59]	Urticaria [1.05]	Congenital [0.75]	Epilepsy [1.4]	Skin Viral [0.79]	URI [0.86]	Blindness [1.36]
Egypt	Neonatal [1.05]	Diarrhea [1.73]	Asthma [1.27]	Iron [1.03]	Dermatitis [0.57]	Urticaria [0.98]	Congenital [0.81]	Epilepsy [1.42]	Skin Viral [0.82]	Vit A [0.37]
Iran	Neonatal [1.32]	Diarrhea [1.27]	Asthma [0.86]	Dermatitis [0.51]	Congenital [0.93]	Iron [1.61]	Urticaria [1.07]	Epilepsy [1.59]	Skin Viral [0.78]	URI [0.89]
Iraq	Neonatal [1.12]	Iron [1.33]	Asthma [1.1]	Vit A [0.8]	Dermatitis [0.65]	Diarrhea [0.94]	Congenital [0.82]	Urticaria [0.94]	Epilepsy [1.22]	Skin Viral [0.83]
Jordan	Neonatal [1.41]	Asthma [1.46]	Diarrhea [1.53]	Dermatitis [0.64]	Vit A [1.17]	Iron [1.75]	Congenital [0.8]	Urticaria [1.02]	Epilepsy [1.28]	Skin Viral [0.79]
Kuwait	Neonatal [1.21]	Asthma [1.53]	Diarrhea [1.94]	Dermatitis [0.61]	Urticaria [1.2]	Congenital [0.83]	Skin Viral [0.8]	Epilepsy [1.55]	URI [0.83]	Vit A [1.28]
Lebanon	Neonatal [1.06]	Asthma [1.64]	Diarrhea [1.83]	Dermatitis [0.73]	Congenital [0.86]	Urticaria [1.08]	Iron [1.59]	Skin Viral [0.79]	Epilepsy [1.29]	URI [0.86]
Libya	Neonatal [1.09]	Diarrhea [2.24]	Dermatitis [0.69]	Asthma [1.24]	Iron [3.77]	Congenital [0.84]	Urticaria [1.14]	Skin Viral [0.8]	Vit A [1.06]	URI [0.84]
Morocco	Neonatal [1.06]	Iron [0.93]	Diarrhea [1.2]	Dermatitis [0.78]	Asthma [0.81]	Vit A [0.62]	Congenital [0.85]	Urticaria [0.94]	Epilepsy [1.14]	Skin Viral [0.83]
Palestine	Neonatal [1.06]	Asthma [1.15]	Diarrhea [1.18]	Dermatitis [0.77]	Vit A [0.53]	Congenital [0.81]	Urticaria [0.94]	Iron [0.32]	Epilepsy [1.04]	Skin Viral [0.85]
Oman	Neonatal [1.11]	Iron [4.92]	Diarrhea [1.99]	Asthma [1.09]	Dermatitis [0.57]	Congenital [0.84]	Urticaria [1.11]	Vit A [1.09]	Skin Viral [0.79]	Epilepsy [1.4]
Qatar	Neonatal [1.02]	Dermatitis [0.69]	Asthma [1.1]	Diarrhea [1.16]	Congenital [0.82]	Urticaria [1.16]	Epilepsy [1.53]	Skin Viral [0.8]	URI [0.84]	Blindness [1.54]
Saudi Arabia	Neonatal [0.78]	Dermatitis [0.69]	Diarrhea [2.01]	Asthma [0.94]	Congenital [0.91]	Urticaria [1.19]	Epilepsy [1.66]	Skin Viral [0.8]	URI [0.83]	Blindness [1.12]
Sudan	Iron [1.29]	Diarrhea [2.34]	Neonatal [0.86]	Vit A [0.66]	Asthma [1.08]	PEM [8.25]	Dermatitis [0.72]	Congenital [0.84]	ID [4.5]	Urticaria [0.94]
Syria	Neonatal [0.99]	PEM [127.54]	Iron [1.37]	Conflict Terror [17710.55]	Asthma [0.99]	Diarrhea [1.04]	Dermatitis [0.54]	Vit A [0.66]	Congenital [0.82]	Urticaria [0.94]
Tunisia	Neonatal [1.04]	Dermatitis [0.72]	Diarrhea [1.36]	Asthma [0.96]	Iron [1.31]	Urticaria [0.99]	Congenital [0.76]	Skin Viral [0.8]	URI [0.89]	Epilepsy [1.06]
Turkey	Neonatal [0.81]	Asthma [1.09]	Dermatitis [0.58]	Diarrhea [1.28]	Vit A [1.09]	Urticaria [1.05]	Congenital [0.78]	Epilepsy [1.52]	URI [1.01]	Skin Viral [0.79]
UAE	Neonatal [1.09]	Asthma [1.7]	Diarrhea [2.16]	Dermatitis [0.68]	Congenital [0.88]	PEM [3624.32]	Urticaria [1.22]	Epilepsy [1.82]	Skin Viral [0.81]	URI [0.82]
Yemen	Iron [3.88]	Vit A [1.27]	Diarrhea [2.39]	Neonatal [1.12]	PEM [5.8]	Dermatitis [0.86]	Oth NTD [2.58]	Asthma [0.87]	Hemog [2.92]	Oth Un Inf [4.13]
**South Asia**	Iron [1.94]	Neonatal [1.42]	Vit A [1.16]	PEM [30.67]	Dermatitis [0.54]	Congenital [1.0]	ID [5.5]	Diarrhea [0.58]	Urticaria [0.92]	Skin Viral [0.84]
Bangladesh	Neonatal [1.37]	Iron [0.96]	Vit A [0.46]	Dermatitis [0.75]	PEM [6.2]	Congenital [0.95]	Urticaria [0.94]	Diarrhea [0.4]	Skin Viral [0.85]	URI [1.1]
Bhutan	Iron [4.45]	Neonatal [1.33]	Vit A [1.33]	Dermatitis [0.73]	Diarrhea [0.82]	Congenital [0.8]	Urticaria [0.91]	Oth NTD [4.42]	Skin Viral [0.83]	Epilepsy [1.01]
India	Iron [2.09]	Neonatal [1.44]	Vit A [1.34]	PEM [44.45]	ID [7.17]	Congenital [1.07]	Dermatitis [0.47]	Urticaria [0.92]	Diarrhea [0.49]	Skin Viral [0.84]
Andhra Pradesh	Iron [2.01]	Neonatal [1.54]	PEM [41.43]	Vit A [1.31]	Congenital [1.02]	ID [5.93]	Dermatitis [0.48]	Urticaria [0.92]	Skin Viral [0.84]	Hemog [2.38]

eFigure 9c. Leading ten causes of YLDs with the ratio of observed YLDs to YLDs expected on the basis of Socio-Demographic Index alone in 2017, 1-4 years, both sexes combined.
The top ten causes contributing to YLDs are listed globally, by socio-demographic quintile, and then by GBD superregion, region, country, and subnationally where modeled. For each cell, the ratio of observed YLDs to YLDs expected on the basis of socio-demographic index (SDI) alone are listed. Abbreviations: YLD=year of life lived with disability, GBD=Global Burden of Disease.

Values shown in brackets represent the ratio of observed YLDs to predicted YLDs on the basis of Socio-Demographic Index (SDI), rounded to two (2) digits. Color ranges (shown below) were calculated to place a roughly equal number of cells into each bin.

COLOR KEY:	[0.0-0.72]	(0.72-0.84]	(0.84-0.92]	(0.92-1.01]	(1.01-1.09]	(1.09-1.17]	(1.17-1.32]	(1.32-1.67]	1.67+

	1	2	3	4	5	6	7	8	9	10
Arunachal Pradesh	Neonatal [1.48]	Iron [1.77]	PEM [56.38]	Vit A [1.21]	Congenital [1.04]	ID [5.83]	Dermatitis [0.48]	Urticaria [0.92]	Skin Viral [0.84]	Diarrhea [0.42]
Assam	Neonatal [1.39]	PEM [26.06]	Vit A [0.74]	ID [7.6]	Iron [0.51]	Congenital [1.11]	Dermatitis [0.49]	Urticaria [0.93]	Skin Fung [2.42]	Diarrhea [0.45]
Bihar	Iron [1.21]	Neonatal [1.51]	Vit A [0.94]	PEM [7.21]	ID [7.79]	Congenital [1.05]	Dermatitis [0.5]	Diarrhea [0.45]	Urticaria [0.94]	Skin Viral [0.86]
Chhattisgarh	Neonatal [1.47]	PEM [25.8]	Vit A [0.91]	Iron [0.81]	ID [6.55]	Congenital [1.05]	Dermatitis [0.49]	Urticaria [0.92]	Skin Viral [0.84]	Skin Fung [1.94]
Delhi	Iron [13.69]	Neonatal [1.48]	Vit A [3.77]	PEM [1231.65]	Congenital [0.9]	Dermatitis [0.37]	Urticaria [1.02]	Diarrhea [0.72]	Hemog [8.76]	Oth NTD [2813.93]
Goa	Neonatal [1.8]	Iron [7.18]	PEM [2650.78]	Vit A [2.4]	Congenital [0.96]	Dermatitis [0.4]	Urticaria [1.08]	Diarrhea [0.73]	Skin Viral [0.79]	URI [0.8]
Gujarat	Iron [3.13]	Neonatal [1.48]	PEM [121.78]	Vit A [1.59]	Congenital [1.03]	Dermatitis [0.47]	ID [5.79]	Urticaria [0.91]	Diarrhea [0.49]	Skin Viral [0.83]
Haryana	Iron [5.03]	Neonatal [1.47]	Vit A [2.03]	PEM [73.48]	Congenital [1.0]	Dermatitis [0.46]	Urticaria [0.9]	ID [5.12]	Hemog [4.18]	Oth NTD [7.64]
Himachal Pradesh	Iron [4.54]	Neonatal [1.61]	Vit A [2.12]	PEM [95.49]	Congenital [0.97]	Dermatitis [0.44]	ID [5.95]	Urticaria [0.93]	Diarrhea [0.54]	Skin Viral [0.8]
Jammu & Kashmir	Iron [2.98]	Neonatal [1.44]	Vit A [1.7]	ID [7.1]	Congenital [1.05]	Dermatitis [0.47]	Urticaria [0.91]	PEM [23.52]	Skin Viral [0.82]	Hemog [3.11]
Jharkhand	Iron [1.71]	Neonatal [1.51]	PEM [24.97]	Vit A [1.12]	ID [6.54]	Congenital [1.05]	Dermatitis [0.49]	Urticaria [0.92]	Diarrhea [0.44]	Skin Viral [0.84]
Karnataka	Neonatal [1.67]	Iron [2.26]	PEM [84.13]	Vit A [1.38]	Congenital [1.0]	ID [5.97]	Dermatitis [0.47]	Urticaria [0.91]	Diarrhea [0.48]	Epilepsy [1.06]
Kerala	Neonatal [1.95]	PEM [425.56]	Iron [2.36]	Vit A [1.39]	Congenital [0.97]	Dermatitis [0.43]	Urticaria [0.96]	ID [5.71]	Skin Viral [0.79]	Diarrhea [0.54]
Madhya Pradesh	Iron [1.82]	Neonatal [1.37]	PEM [22.21]	Vit A [1.21]	ID [7.18]	Congenital [1.05]	Dermatitis [0.49]	Urticaria [0.92]	Hemog [2.24]	Diarrhea [0.42]
Maharashtra	Neonatal [1.57]	Iron [3.26]	PEM [246.67]	Vit A [1.57]	Congenital [1.0]	ID [5.94]	Urticaria [0.92]	Dermatitis [0.38]	Diarrhea [0.65]	Skin Viral [0.81]
Manipur	Neonatal [1.61]	ID [7.8]	Vit A [0.68]	Congenital [1.06]	Dermatitis [0.47]	Urticaria [0.92]	Iron [0.51]	Diarrhea [0.61]	Skin Viral [0.83]	PEM [20.84]
Meghalaya	Neonatal [1.56]	Iron [1.25]	Vit A [1.07]	PEM [35.27]	ID [7.04]	Congenital [1.06]	Dermatitis [0.48]	Urticaria [0.92]	Diarrhea [0.49]	Skin Viral [0.84]
Mizoram	Neonatal [1.61]	PEM [118.47]	Vit A [0.84]	Congenital [1.03]	ID [6.99]	Dermatitis [0.46]	Urticaria [0.93]	Diarrhea [0.65]	Skin Viral [0.81]	URI [0.91]
Nagaland	Neonatal [1.7]	Vit A [1.23]	PEM [151.12]	Congenital [1.01]	Dermatitis [0.44]	ID [6.13]	Urticaria [0.94]	Iron [0.74]	Iodine [76.98]	Skin Viral [0.8]
Odisha	Neonatal [1.38]	PEM [33.93]	Vit A [0.93]	Iron [0.92]	ID [7.15]	Congenital [1.07]	Dermatitis [0.49]	Urticaria [0.92]	Diarrhea [0.53]	Skin Fung [2.11]
Punjab	Iron [3.6]	Neonatal [1.51]	Vit A [1.78]	PEM [85.86]	Congenital [1.01]	Dermatitis [0.44]	ID [5.82]	Urticaria [0.92]	Skin Viral [0.81]	Diarrhea [0.47]
Rajasthan	Iron [1.62]	Neonatal [1.34]	PEM [23.8]	Vit A [1.13]	ID [6.75]	Congenital [1.04]	Dermatitis [0.49]	Urticaria [0.92]	Diarrhea [0.41]	Skin Viral [0.84]
Sikkim	Neonatal [1.63]	Iron [2.75]	Vit A [1.57]	Congenital [0.98]	Dermatitis [0.45]	Urticaria [0.93]	ID [5.19]	Diarrhea [0.6]	Skin Viral [0.8]	URI [0.88]
Tamil Nadu	Neonatal [1.51]	PEM [207.66]	Iron [2.4]	Vit A [1.55]	Dermatitis [0.45]	ID [6.13]	Congenital [0.83]	Urticaria [0.92]	Skin Viral [0.81]	Diarrhea [0.45]
Telangana	Iron [2.96]	Neonatal [1.55]	PEM [103.93]	Vit A [1.61]	Congenital [1.02]	ID [5.96]	Dermatitis [0.47]	Urticaria [0.91]	Skin Viral [0.83]	Hemog [2.99]
Tripura	Neonatal [1.51]	PEM [41.7]	Iron [1.03]	Vit A [0.97]	ID [7.04]	Congenital [1.04]	Dermatitis [0.48]	Urticaria [0.92]	Diarrhea [0.49]	Skin Viral [0.84]
Uttar Pradesh	Iron [1.88]	Neonatal [1.32]	Vit A [1.2]	PEM [11.87]	ID [7.29]	Congenital [1.14]	Dermatitis [0.47]	Urticaria [0.92]	Diarrhea [0.47]	Hemog [2.3]
Uttarakhand	Iron [4.04]	Neonatal [1.6]	Vit A [1.82]	PEM [63.93]	Congenital [1.02]	Dermatitis [0.46]	ID [5.65]	Urticaria [0.89]	Diarrhea [0.57]	Skin Viral [0.81]
W Bengal	Neonatal [1.4]	Iron [1.43]	Vit A [1.14]	PEM [27.01]	ID [7.67]	Congenital [1.06]	Dermatitis [0.48]	Urticaria [0.92]	Skin Viral [0.92]	URI [0.95]
UTs other than Delhi	Iron [5.65]	Neonatal [1.72]	Vit A [2.3]	PEM [293.26]	Congenital [0.97]	Dermatitis [0.43]	Urticaria [0.94]	ID [5.19]	Skin Viral [0.79]	Diarrhea [0.49]
Nepal	Neonatal [1.62]	Iron [0.67]	Vit A [0.48]	Dermatitis [0.76]	Congenital [0.8]	Diarrhea [0.48]	Urticaria [0.97]	Epilepsy [1.03]	Skin Viral [0.85]	PEM [1.71]

eFigure 9c. Leading ten causes of YLDs with the ratio of observed YLDs to YLDs expected on the basis of Socio-Demographic Index alone in 2017, 1-4 years, both sexes combined. The top ten causes contributing to YLDs are listed globally, by socio-demographic quintile, and then by GBD superregion, region, country, and subnationally where modeled. For each cell, the ratio of observed YLDs to YLDs expected on the basis of socio-demographic index (SDI) alone are listed. Abbreviations: YLD=year of life lived with disability, GBD=Global Burden of Disease.

Values shown in brackets represent the ratio of observed YLDs to predicted YLDs on the basis of Socio-Demographic Index (SDI), rounded to two (2) digits. Color ranges (shown below) were calculated to place a roughly equal number of cells into each bin.

COLOR KEY:	[0.0-0.72]	(0.72-0.84]	(0.84-0.92]	(0.92-1.01]	(1.01-1.09]	(1.09-1.17]	(1.17-1.32]	(1.32-1.67]	1.67+

	1	2	3	4	5	6	7	8	9	10
Pakistan	Iron [1.95]	Neonatal [1.38]	Vit A [0.86]	Diarrhea [1.06]	PEM [9.85]	Dermatitis [0.77]	Urticaria [0.92]	Congenital [0.7]	Epilepsy [1.0]	Hemog [2.11]
**Southeast Asia, East Asia, and Oceania**	Neonatal [1.08]	Dermatitis [0.67]	Asthma [0.98]	Diarrhea [1.24]	Congenital [0.87]	Skin Viral [0.98]	Vit A [0.74]	Urticaria [0.89]	URI [0.88]	Scabies [3.27]
**East Asia**	Neonatal [1.35]	Dermatitis [0.55]	Diarrhea [1.09]	Asthma [0.78]	Skin Viral [1.02]	Congenital [0.71]	Urticaria [0.91]	Vit A [0.64]	URI [0.8]	Hernia [2.34]
China	Neonatal [1.36]	Dermatitis [0.55]	Asthma [0.75]	Diarrhea [1.04]	Skin Viral [1.02]	Congenital [0.71]	Urticaria [0.9]	Vit A [0.61]	URI [0.81]	Hernia [2.36]
N Korea	Diarrhea [2.02]	Neonatal [0.67]	Vit A [0.64]	Dermatitis [0.72]	Iron [0.56]	Asthma [0.77]	Congenital [0.75]	Skin Viral [1.05]	Urticaria [0.79]	Scabies [1.59]
Taiwan	Asthma [2.63]	Diarrhea [2.74]	Dermatitis [0.62]	Neonatal [0.63]	Congenital [0.86]	Skin Viral [1.04]	Urticaria [1.24]	URI [0.73]	Scabies [11.75]	Epilepsy [1.05]
**Oceania**	Iron [1.23]	Asthma [2.16]	Diarrhea [1.41]	Dermatitis [1.08]	Neonatal [0.72]	Vit A [0.52]	PEM [7.25]	Congenital [0.92]	Skin Viral [1.01]	Urticaria [0.83]
Am Samoa	Asthma [2.57]	Diarrhea [2.25]	Dermatitis [0.91]	Neonatal [0.68]	Vit A [1.63]	Congenital [0.8]	Skin Viral [0.94]	Urticaria [0.9]	URI [0.8]	Scabies [2.72]
Micronesia	Asthma [2.02]	Diarrhea [1.63]	Dermatitis [1.05]	Vit A [1.04]	Neonatal [0.67]	Iron [0.56]	Congenital [0.91]	Skin Viral [0.99]	Urticaria [0.82]	Scabies [1.92]
Fiji	Asthma [2.04]	Iron [2.37]	Diarrhea [1.99]	Dermatitis [0.97]	Neonatal [0.66]	Vit A [1.0]	Congenital [0.82]	Skin Viral [0.95]	Urticaria [0.84]	Scabies [2.77]
Guam	Diarrhea [5.05]	Asthma [2.73]	Dermatitis [0.85]	Neonatal [0.78]	Vit A [2.28]	Congenital [0.79]	Skin Viral [0.96]	Urticaria [1.07]	Scabies [7.23]	URI [0.76]
Kiribati	Asthma [2.09]	Vit A [0.67]	Diarrhea [1.28]	Dermatitis [1.11]	Neonatal [0.72]	PEM [3.26]	Iron [0.28]	Congenital [0.91]	Skin Viral [1.02]	Urticaria [0.84]
Marshall	Iron [1.54]	Asthma [1.87]	Vit A [1.21]	Dermatitis [1.07]	Neonatal [0.68]	Diarrhea [1.36]	Congenital [0.91]	Skin Viral [1.0]	Urticaria [0.82]	Scabies [1.85]
N Mariana	Asthma [2.76]	Diarrhea [3.53]	Neonatal [0.82]	Dermatitis [0.87]	Congenital [0.81]	Skin Viral [0.95]	Urticaria [1.0]	Scabies [5.53]	URI [0.78]	Vit A [0.84]
PNG	Iron [1.12]	Asthma [2.25]	Diarrhea [1.27]	Dermatitis [1.1]	Neonatal [0.76]	PEM [4.85]	Vit A [0.41]	Congenital [0.91]	Skin Viral [1.02]	Urticaria [0.84]
Samoa	Asthma [1.94]	Diarrhea [1.8]	Vit A [1.11]	Dermatitis [1.05]	Neonatal [0.68]	Iron [0.92]	Congenital [0.87]	Skin Viral [0.99]	Urticaria [0.82]	Scabies [1.91]
Solomon	Iron [0.87]	Asthma [1.99]	Vit A [0.65]	Diarrhea [1.19]	Dermatitis [1.1]	Neonatal [0.76]	Congenital [0.9]	Skin Viral [1.02]	Urticaria [0.84]	Scabies [1.98]
Tonga	Asthma [2.66]	Diarrhea [1.77]	Dermatitis [0.99]	Neonatal [0.67]	Vit A [1.06]	Congenital [0.86]	Skin Viral [0.96]	Urticaria [0.83]	Scabies [2.44]	URI [0.87]
Vanuatu	Asthma [2.17]	Iron [0.86]	Dermatitis [1.08]	Diarrhea [1.22]	Vit A [0.67]	Neonatal [0.72]	Congenital [0.88]	Skin Viral [1.01]	Urticaria [0.83]	Scabies [1.72]
**Southeast Asia**	Neonatal [0.73]	Asthma [1.21]	Dermatitis [0.84]	Diarrhea [1.36]	Congenital [1.09]	PEM [110.33]	Vit A [0.76]	Iron [0.79]	Urticaria [0.87]	Skin Viral [0.94]
Cambodia	Iron [1.26]	Neonatal [0.74]	Dermatitis [1.05]	Vit A [0.61]	Diarrhea [1.03]	Asthma [1.03]	Congenital [1.12]	Urticaria [0.85]	Skin Viral [1.0]	Epilepsy [1.04]
Indonesia	Neonatal [0.74]	Diarrhea [1.76]	PEM [255.61]	Asthma [1.14]	Dermatitis [0.64]	Congenital [0.99]	Vit A [0.66]	Urticaria [0.91]	Skin Viral [0.93]	URI [1.14]
Laos	Dermatitis [1.04]	Asthma [1.15]	Neonatal [0.61]	Vit A [0.62]	Diarrhea [1.02]	Congenital [1.17]	Iron [0.38]	Urticaria [0.85]	Skin Viral [1.0]	Scabies [1.86]
Malaysia	Neonatal [0.84]	Dermatitis [0.93]	Diarrhea [2.32]	Asthma [1.16]	PEM [2010.56]	Congenital [1.06]	Urticaria [1.02]	Skin Viral [0.94]	Scabies [5.62]	Epilepsy [1.26]
Maldives	Dermatitis [0.93]	Neonatal [0.66]	Asthma [1.12]	Congenital [1.03]	Vit A [0.81]	Diarrhea [0.81]	PEM [117.25]	Urticaria [0.87]	Skin Viral [0.94]	Scabies [3.21]
Mauritius	Neonatal [0.83]	Dermatitis [0.87]	Iron [4.14]	Asthma [1.45]	Diarrhea [1.77]	PEM [917.63]	Congenital [1.02]	Urticaria [0.95]	Skin Viral [0.93]	Vit A [0.86]
Myanmar	Neonatal [0.75]	Dermatitis [1.04]	Vit A [0.82]	Congenital [1.59]	Diarrhea [1.13]	Asthma [1.02]	Iron [0.54]	Urticaria [0.84]	Skin Viral [0.99]	Scabies [2.05]
Philippines	Asthma [1.69]	Neonatal [0.79]	Dermatitis [1.06]	Vit A [0.8]	Congenital [1.14]	Diarrhea [0.96]	Iron [0.81]	URI [1.29]	Urticaria [0.84]	Skin Viral [0.96]
Sri Lanka	Neonatal [0.74]	Dermatitis [0.9]	Asthma [1.14]	Congenital [1.21]	Diarrhea [1.04]	PEM [247.2]	Vit A [0.87]	Skin Viral [0.96]	Epilepsy [1.48]	Urticaria [0.89]
Seychelles	Neonatal [0.8]	Dermatitis [0.9]	Asthma [1.17]	Diarrhea [1.28]	Congenital [1.02]	Urticaria [0.91]	Skin Viral [0.93]	Epilepsy [1.2]	Scabies [3.49]	URI [0.81]
Thailand	Dermatitis [0.98]	Neonatal [0.68]	Asthma [1.19]	Congenital [1.09]	Diarrhea [0.87]	Urticaria [0.9]	Skin Viral [0.94]	Vit A [0.69]	Scabies [3.92]	PEM [179.61]

eFigure 9c. Leading ten causes of YLDs with the ratio of observed YLDs to YLDs expected on the basis of Socio-Demographic Index alone in 2017, 1-4 years, both sexes combined.
The top ten causes contributing to YLDs are listed globally, by socio-demographic quintile, and then by GBD superregion, region, country, and subnationally where modeled. For each cell, the ratio of observed YLDs to YLDs expected on the basis of socio-demographic index (SDI) alone are listed. Abbreviations: YLD=year of life lived with disability, GBD=Global Burden of Disease.

Values shown in brackets represent the ratio of observed YLDs to predicted YLDs on the basis of Socio-Demographic Index (SDI), rounded to two (2) digits. Color ranges (shown below) were calculated to place a roughly equal number of cells into each bin.

COLOR KEY:	[0.0-0.72]	(0.72-0.84]	(0.84-0.92]	(0.92-1.01]	(1.01-1.09]	(1.09-1.17]	(1.17-1.32]	(1.32-1.67]	1.67+

	1	2	3	4	5	6	7	8	9	10
Timor-Leste	Iron [1.92]	Vit A [0.94]	PEM [17.77]	Dermatitis [1.04]	Neonatal [0.66]	Diarrhea [1.13]	Asthma [1.04]	Congenital [1.13]	Urticaria [0.84]	Skin Viral [0.99]
Vietnam	Dermatitis [0.88]	Neonatal [0.6]	Asthma [0.87]	Diarrhea [1.03]	Iron [0.82]	Congenital [1.01]	Vit A [0.58]	Urticaria [0.84]	Skin Viral [0.97]	Scabies [2.09]
**Sub-Saharan Africa**	Iron [1.27]	Vit A [0.99]	Neonatal [1.11]	Diarrhea [1.06]	Dermatitis [0.94]	Congenital [1.16]	Asthma [0.71]	Malaria [13.68]	PEM [3.5]	Epilepsy [1.16]
**Central Sub-Saharan Africa**	Iron [1.04]	Vit A [1.09]	Neonatal [1.24]	Diarrhea [1.45]	Dermatitis [1.13]	Asthma [0.99]	Malaria [24.01]	Congenital [1.08]	Epilepsy [1.55]	Urticaria [0.86]
Angola	Iron [2.04]	Vit A [1.15]	Neonatal [1.34]	Diarrhea [1.41]	Dermatitis [1.12]	Asthma [1.17]	Congenital [1.12]	Epilepsy [1.73]	Malaria [16.56]	Hemog [2.3]
C African Rep	Iron [1.33]	Vit A [1.24]	Diarrhea [1.28]	Neonatal [1.2]	Malaria [4.99]	Dermatitis [1.19]	Asthma [1.17]	Congenital [1.06]	Epilepsy [1.51]	Hemog [1.75]
Congo	Neonatal [1.44]	Vit A [1.68]	Diarrhea [1.99]	Iron [1.39]	Dermatitis [1.09]	Asthma [1.25]	Epilepsy [1.98]	Congenital [1.01]	Malaria [185.54]	PEM [22.0]
Congo DR	Vit A [0.77]	Neonatal [1.32]	Diarrhea [1.22]	Iron [0.48]	Dermatitis [1.18]	Malaria [5.13]	Asthma [0.97]	Congenital [1.04]	Epilepsy [1.36]	Urticaria [0.9]
Eq Guinea	Neonatal [1.05]	Iron [2.44]	Vit A [1.86]	Dermatitis [1.02]	Diarrhea [1.75]	Malaria [841.23]	Asthma [1.15]	Epilepsy [2.17]	Congenital [0.94]	URI [1.22]
Gabon	Neonatal [1.51]	Iron [4.2]	Diarrhea [2.24]	Vit A [2.0]	Dermatitis [0.99]	Asthma [1.23]	Epilepsy [2.31]	Malaria [1189.82]	Congenital [0.92]	URI [1.26]
**Eastern Sub-Saharan Africa**	Iron [0.81]	Neonatal [1.38]	Vit A [0.76]	Dermatitis [1.01]	Diarrhea [0.89]	Congenital [1.18]	Asthma [0.89]	Epilepsy [1.06]	Urticaria [0.9]	Skin Viral [0.99]
Burundi	Vit A [0.62]	Neonatal [1.24]	Iron [0.45]	Dermatitis [1.09]	Diarrhea [0.77]	Congenital [1.13]	Asthma [0.91]	Malaria [1.58]	Epilepsy [1.12]	Urticaria [0.93]
Comoros	Neonatal [1.53]	Vit A [0.72]	Iron [0.59]	Dermatitis [1.02]	Diarrhea [1.04]	Asthma [0.96]	Congenital [1.04]	PEM [3.59]	Urticaria [0.87]	Epilepsy [1.08]
Djibouti	Neonatal [1.42]	PEM [24.12]	Iron [1.28]	Dermatitis [0.99]	Vit A [0.53]	Asthma [0.8]	Diarrhea [0.71]	Congenital [1.04]	Epilepsy [1.19]	Urticaria [0.85]
Eritrea	Iron [1.15]	Neonatal [1.55]	Vit A [0.84]	Dermatitis [1.02]	Diarrhea [0.9]	Congenital [1.07]	Asthma [0.8]	PEM [2.84]	Epilepsy [1.09]	Urticaria [0.87]
Ethiopia	Iron [0.9]	Vit A [0.8]	Neonatal [1.45]	Dermatitis [1.05]	Diarrhea [0.73]	Congenital [1.08]	Asthma [0.78]	PEM [1.03]	Urticaria [0.95]	Skin Viral [1.12]
Kenya	Neonatal [1.55]	Vit A [0.97]	Dermatitis [1.05]	Diarrhea [1.1]	Congenital [1.52]	Iron [0.52]	Asthma [0.61]	Urticaria [0.89]	Skin Viral [0.92]	Epilepsy [0.99]
Baringo	Neonatal [1.56]	Vit A [1.07]	Dermatitis [1.04]	Diarrhea [1.08]	Iron [0.46]	Congenital [1.49]	Asthma [0.61]	Urticaria [0.9]	Skin Viral [0.93]	URI [1.08]
Bomet	Neonatal [1.7]	Vit A [0.79]	Dermatitis [1.03]	Congenital [1.47]	Diarrhea [0.84]	Iron [0.45]	Asthma [0.63]	Urticaria [0.89]	Skin Viral [0.93]	URI [1.07]
Bungoma	Neonatal [1.58]	Vit A [1.01]	Iron [0.72]	Dermatitis [1.03]	Diarrhea [1.06]	Congenital [1.49]	PEM [4.61]	Asthma [0.61]	Urticaria [0.89]	Skin Viral [0.93]
Busia	Neonatal [1.59]	Vit A [0.57]	Dermatitis [1.05]	Diarrhea [1.02]	Congenital [1.5]	Malaria [12.08]	Asthma [0.6]	Iron [0.24]	Urticaria [0.91]	Skin Viral [0.94]
Elgeyo-Marakwet	Neonatal [1.67]	Dermatitis [1.03]	Vit A [0.63]	Congenital [1.5]	Diarrhea [0.98]	Asthma [0.62]	Iron [0.33]	Urticaria [0.89]	Skin Viral [0.93]	Epilepsy [0.97]
Embu	Neonatal [1.66]	Dermatitis [1.02]	Vit A [0.8]	Diarrhea [1.22]	Iron [0.71]	Congenital [1.5]	Asthma [0.61]	Urticaria [0.89]	Skin Viral [0.92]	Epilepsy [1.05]
Garissa	Neonatal [1.85]	Vit A [0.57]	Dermatitis [1.11]	Congenital [1.49]	Iron [0.33]	Diarrhea [0.57]	Asthma [0.68]	Urticaria [0.96]	Skin Viral [0.98]	Epilepsy [0.84]
HomaBay	Neonatal [1.2]	Vit A [0.78]	Iron [0.6]	Diarrhea [1.2]	Dermatitis [1.05]	Congenital [1.54]	HIV [53.56]	Asthma [0.65]	PEM [2.5]	Urticaria [0.91]
Isiolo	Iron [1.08]	Vit A [0.92]	Neonatal [1.49]	Dermatitis [1.07]	Diarrhea [0.93]	PEM [3.42]	Congenital [1.49]	Asthma [0.62]	Urticaria [0.92]	Skin Viral [0.95]
Kajiado	Neonatal [1.73]	Iron [2.07]	Vit A [1.93]	Dermatitis [1.02]	Congenital [1.5]	Diarrhea [0.93]	Asthma [0.61]	Hemog [3.18]	Urticaria [0.88]	Skin Viral [0.92]
Kakamega	Neonatal [1.46]	Vit A [0.86]	Iron [0.79]	Diarrhea [1.19]	Dermatitis [1.04]	Congenital [1.5]	Asthma [0.63]	Urticaria [0.9]	Skin Viral [0.93]	Epilepsy [0.91]
Kericho	Neonatal [1.79]	Dermatitis [1.03]	Diarrhea [1.22]	Vit A [0.59]	Congenital [1.49]	Asthma [0.63]	Urticaria [0.89]	Skin Viral [0.93]	URI [1.07]	Epilepsy [0.93]
Kiambu	Neonatal [1.72]	Dermatitis [1.0]	Congenital [1.52]	Diarrhea [1.18]	Asthma [0.62]	Vit A [0.5]	Urticaria [0.88]	Skin Viral [0.91]	Epilepsy [1.15]	URI [1.06]
Kilifi	Neonatal [1.52]	Vit A [0.92]	Dermatitis [1.04]	Diarrhea [1.05]	Congenital [1.49]	Iron [0.31]	Asthma [0.57]	Urticaria [0.9]	Skin Viral [0.93]	URI [1.08]

eFigure 9c. Leading ten causes of YLDs with the ratio of observed YLDs to YLDs expected on the basis of Socio-Demographic Index alone in 2017, 1-4 years, both sexes combined.
The top ten causes contributing to YLDs are listed globally, by socio-demographic quintile, and then by GBD superregion, region, country, and subnationally where modeled. For each cell, the ratio of observed YLDs to YLDs expected on the basis of socio-demographic index (SDI) alone are listed. Abbreviations: YLD=year of life lived with disability, GBD=Global Burden of Disease.

Values shown in brackets represent the ratio of observed YLDs to predicted YLDs on the basis of Socio-Demographic Index (SDI), rounded to two (2) digits. Color ranges (shown below) were calculated to place a roughly equal number of cells into each bin.

COLOR KEY:	[0.0-0.72]	(0.72-0.84]	(0.84-0.92]	(0.92-1.01]	(1.01-1.09]	(1.09-1.17]	(1.17-1.32]	(1.32-1.67]	1.67+

	1	2	3	4	5	6	7	8	9	10
Kirinyaga	Neonatal [1.7]	Diarrhea [1.46]	Dermatitis [1.02]	Vit A [0.75]	Congenital [1.52]	Asthma [0.63]	Urticaria [0.89]	Skin Viral [0.92]	Epilepsy [1.05]	URI [1.07]
Kisii	Neonatal [1.45]	Vit A [0.95]	Dermatitis [1.03]	Diarrhea [1.12]	Congenital [1.53]	Asthma [0.61]	Urticaria [0.89]	Skin Viral [0.92]	Epilepsy [0.98]	URI [1.08]
Kisumu	Neonatal [1.2]	Vit A [1.17]	Iron [0.87]	Dermatitis [0.89]	Congenital [1.55]	Diarrhea [1.04]	Asthma [0.62]	Urticaria [0.88]	Epilepsy [1.11]	Hemog [2.42]
Kitui	Neonatal [1.73]	Vit A [0.78]	Iron [0.62]	Dermatitis [1.04]	Diarrhea [1.07]	Congenital [1.47]	Asthma [0.6]	Urticaria [0.9]	Skin Viral [0.93]	URI [1.08]
Kwale	Neonatal [1.59]	Vit A [0.77]	Dermatitis [1.04]	Iron [0.5]	Congenital [1.48]	Diarrhea [0.87]	Asthma [0.6]	Urticaria [0.9]	Skin Viral [0.93]	Epilepsy [0.94]
Laikipia	Neonatal [1.76]	Dermatitis [1.02]	Diarrhea [1.18]	Congenital [1.5]	Asthma [0.66]	Vit A [0.44]	Urticaria [0.89]	Skin Viral [0.92]	Epilepsy [1.04]	URI [1.08]
Lamu	Neonatal [1.46]	Iron [0.75]	Vit A [0.61]	Dermatitis [1.04]	Diarrhea [1.08]	Congenital [1.49]	Asthma [0.62]	Urticaria [0.9]	Epilepsy [1.02]	Skin Viral [0.93]
Machakos	Neonatal [1.67]	Dermatitis [1.02]	Diarrhea [1.31]	Vit A [0.79]	Congenital [1.49]	Iron [0.53]	Asthma [0.6]	Urticaria [0.89]	Skin Viral [0.92]	Epilepsy [1.01]
Makueni	Neonatal [1.65]	Dermatitis [1.04]	Congenital [1.49]	Vit A [0.44]	Diarrhea [0.74]	Asthma [0.62]	Urticaria [0.9]	Skin Viral [0.93]	HIV [48.42]	Epilepsy [0.93]
Mandera	Vit A [0.91]	Neonatal [1.68]	Iron [0.48]	Dermatitis [1.12]	Diarrhea [0.69]	Congenital [1.47]	PEM [1.06]	Asthma [0.64]	Urticaria [0.97]	Skin Viral [0.99]
Marsabit	Neonatal [1.89]	PEM [4.22]	Vit A [0.58]	Dermatitis [1.11]	Diarrhea [0.78]	Congenital [1.48]	Iron [0.19]	Asthma [0.62]	Urticaria [0.95]	Skin Viral [0.98]
Meru	Neonatal [1.7]	Vit A [1.17]	Diarrhea [1.43]	Dermatitis [1.02]	Congenital [1.5]	Asthma [0.63]	Iron [0.34]	Urticaria [0.89]	Skin Viral [0.92]	Epilepsy [0.99]
Migori	Vit A [0.84]	Neonatal [1.14]	Diarrhea [1.12]	Dermatitis [1.05]	Congenital [1.53]	Iron [0.28]	Asthma [0.66]	Urticaria [0.91]	HIV [41.45]	PEM [2.02]
Mombasa	Neonatal [1.5]	Vit A [1.23]	Dermatitis [1.01]	Diarrhea [1.34]	Congenital [1.52]	Iron [0.53]	Asthma [0.59]	Urticaria [0.88]	Epilepsy [1.16]	Skin Viral [0.91]
Murang'a	Neonatal [1.71]	Dermatitis [1.03]	Congenital [1.51]	Diarrhea [1.06]	Vit A [0.56]	Asthma [0.62]	Urticaria [0.89]	Skin Viral [0.92]	URI [1.16]	Epilepsy [1.01]
Nairobi	Neonatal [1.66]	Dermatitis [1.15]	Congenital [1.43]	Vit A [1.17]	Diarrhea [1.13]	Asthma [0.67]	Urticaria [0.93]	Epilepsy [1.42]	Skin Viral [0.86]	URI [0.96]
Nakuru	Neonatal [1.83]	Dermatitis [1.02]	Diarrhea [1.31]	Congenital [1.53]	Vit A [0.69]	Asthma [0.65]	Epilepsy [1.61]	Urticaria [0.89]	Skin Viral [0.92]	Iron [0.3]
Nandi	Neonatal [1.72]	Dermatitis [1.03]	Congenital [1.5]	Diarrhea [0.96]	Vit A [0.48]	Asthma [0.63]	Urticaria [0.89]	Skin Viral [0.93]	Epilepsy [0.96]	URI [1.08]
Narok	Neonatal [1.89]	Vit A [1.05]	Iron [0.49]	Dermatitis [1.06]	Congenital [1.46]	Diarrhea [0.75]	Asthma [0.67]	Urticaria [0.92]	Skin Viral [0.95]	Epilepsy [0.89]
Nyamira	Neonatal [1.6]	Dermatitis [1.03]	Diarrhea [1.16]	Congenital [1.54]	Asthma [0.62]	Urticaria [0.89]	Epilepsy [1.14]	Skin Viral [0.93]	URI [1.08]	Vit A [0.27]
Nyandarua	Neonatal [1.79]	Dermatitis [1.02]	Diarrhea [1.15]	Congenital [1.53]	Vit A [0.47]	Asthma [0.64]	PEM [10.7]	Urticaria [0.89]	Epilepsy [1.16]	Skin Viral [0.92]
Nyeri	Neonatal [1.71]	Dermatitis [1.02]	Congenital [1.53]	Diarrhea [1.13]	Vit A [0.47]	Asthma [0.62]	Urticaria [0.89]	Epilepsy [1.19]	Skin Viral [0.92]	URI [1.07]
Samburu	Neonatal [1.98]	Vit A [0.83]	Iron [0.51]	Dermatitis [1.12]	Diarrhea [0.79]	Congenital [1.48]	PEM [0.83]	Asthma [0.68]	Urticaria [0.96]	Skin Viral [0.99]
Siaya	Vit A [1.13]	Neonatal [1.26]	Iron [0.75]	Diarrhea [1.16]	Dermatitis [1.03]	Congenital [1.51]	Malaria [23.46]	Asthma [0.66]	Urticaria [0.9]	Skin Viral [0.93]
TaitaTaveta	Neonatal [1.59]	Dermatitis [1.03]	Diarrhea [1.2]	Congenital [1.52]	Vit A [0.53]	Asthma [0.61]	Urticaria [0.89]	Epilepsy [1.15]	Skin Viral [0.92]	URI [1.07]
TanaRiver	Vit A [0.99]	Neonatal [1.68]	Iron [0.89]	Dermatitis [1.07]	Diarrhea [0.95]	Congenital [1.5]	PEM [2.0]	Asthma [0.62]	Urticaria [0.92]	Skin Viral [0.96]
TharakaNithi	Neonatal [1.47]	Vit A [0.86]	Diarrhea [1.39]	Dermatitis [1.02]	Congenital [1.53]	Iron [0.51]	Asthma [0.62]	Urticaria [0.88]	Epilepsy [1.12]	Skin Viral [0.92]
TransNzoia	Neonatal [1.64]	Vit A [0.87]	Diarrhea [1.31]	Dermatitis [1.03]	Congenital [1.48]	Iron [0.39]	Asthma [0.62]	Urticaria [0.89]	Skin Viral [0.93]	URI [1.07]
Turkana	Neonatal [1.83]	PEM [2.5]	Vit A [0.5]	Diarrhea [0.91]	Dermatitis [1.12]	Congenital [1.52]	Asthma [0.64]	Urticaria [0.97]	Iron [0.13]	Skin Viral [1.0]
UasinGishu	Neonatal [1.62]	Dermatitis [1.44]	Vit A [0.92]	Diarrhea [1.29]	Iron [0.8]	Congenital [1.49]	Asthma [0.62]	Urticaria [0.89]	Skin Viral [0.92]	URI [1.07]
Vihiga	Neonatal [1.6]	Vit A [1.05]	Dermatitis [1.03]	Diarrhea [1.0]	Congenital [1.5]	Iron [0.41]	Asthma [0.63]	Urticaria [0.9]	Malaria [19.48]	Skin Viral [0.93]

eFigure 9c. Leading ten causes of YLDs with the ratio of observed YLDs to YLDs expected on the basis of Socio-Demographic Index alone in 2017, 1-4 years, both sexes combined.
The top ten causes contributing to YLDs are listed globally, by socio-demographic quintile, and then by GBD superregion, region, country, and subnationally where modeled. For each cell, the ratio of observed YLDs to YLDs expected on the basis of socio-demographic index (SDI) alone are listed. Abbreviations: YLD=year of life lived with disability, GBD=Global Burden of Disease.

Values shown in brackets represent the ratio of observed YLDs to predicted YLDs on the basis of Socio-Demographic Index (SDI), rounded to two (2) digits. Color ranges (shown below) were calculated to place a roughly equal number of cells into each bin.

COLOR KEY:	[0.0-0.72]	(0.72-0.84]	(0.84-0.92]	(0.92-1.01]	(1.01-1.09]	(1.09-1.17]	(1.17-1.32]	(1.32-1.67]	1.67+

	1	2	3	4	5	6	7	8	9	10
Wajir	Neonatal [1.94]	Vit A [0.63]	Dermatitis [1.14]	Iron [0.3]	Congenital [1.51]	Diarrhea [0.56]	Asthma [0.66]	Urticaria [0.98]	Skin Viral [1.0]	URI [1.23]
WestPokot	Neonatal [1.8]	Vit A [0.96]	Iron [0.59]	Dermatitis [1.08]	Diarrhea [0.96]	Congenital [1.47]	PEM [1.8]	Asthma [0.64]	Urticaria [0.93]	Skin Viral [0.96]
Madagascar	Neonatal [1.52]	Congenital [2.43]	Diarrhea [1.21]	Vit A [0.48]	Iron [0.42]	Dermatitis [1.08]	PEM [1.63]	Asthma [1.08]	Epilepsy [1.17]	Urticaria [0.92]
Malawi	Iron [0.91]	Vit A [0.74]	Neonatal [1.39]	Diarrhea [1.48]	Dermatitis [1.07]	Congenital [1.12]	Asthma [0.89]	Malaria [2.64]	Epilepsy [1.02]	Urticaria [0.91]
Mozambique	Iron [0.87]	Vit A [0.8]	Neonatal [1.29]	Dermatitis [0.94]	Malaria [3.32]	Diarrhea [0.6]	Asthma [0.92]	Congenital [1.09]	Epilepsy [1.39]	HIV [29.72]
Rwanda	Neonatal [1.36]	Iron [0.56]	Vit A [0.55]	Asthma [1.19]	Diarrhea [0.87]	Dermatitis [0.77]	Congenital [1.09]	Epilepsy [1.16]	Urticaria [0.88]	Skin Viral [0.95]
Somalia	Iron [0.75]	Vit A [0.63]	Neonatal [1.5]	Dermatitis [1.11]	PEM [0.85]	Diarrhea [0.61]	Congenital [1.08]	Asthma [0.86]	Epilepsy [0.91]	Urticaria [0.94]
S Sudan	Vit A [0.95]	Iron [0.89]	PEM [2.17]	Neonatal [1.4]	Diarrhea [1.01]	Dermatitis [1.09]	Asthma [1.07]	Malaria [1.26]	Congenital [1.02]	Epilepsy [0.98]
Tanzania	Iron [0.9]	Neonatal [1.33]	Vit A [0.69]	Dermatitis [0.91]	Asthma [1.16]	Diarrhea [0.71]	Congenital [0.91]	Epilepsy [1.11]	Urticaria [0.87]	Skin Viral [0.91]
Uganda	Neonatal [1.37]	Iron [0.58]	Vit A [0.6]	Dermatitis [1.03]	Diarrhea [0.92]	Asthma [0.98]	Malaria [7.12]	Congenital [0.94]	Epilepsy [1.35]	Urticaria [0.89]
Zambia	Iron [1.35]	Neonatal [1.24]	Vit A [1.11]	Diarrhea [1.25]	Dermatitis [1.0]	Congenital [1.08]	Asthma [0.73]	Epilepsy [1.39]	Malaria [21.49]	Urticaria [0.86]
**Southern Sub-Saharan Africa**	Neonatal [1.17]	Dermatitis [1.19]	Vit A [1.06]	Congenital [1.17]	Iron [1.14]	Diarrhea [0.85]	Urticaria [0.89]	Skin Viral [0.83]	URI [0.9]	Epilepsy [1.0]
Botswana	Neonatal [1.31]	Dermatitis [1.17]	Vit A [1.31]	Iron [1.78]	Diarrhea [1.07]	Congenital [0.82]	Urticaria [0.89]	Skin Viral [0.83]	Asthma [0.41]	Epilepsy [1.06]
Lesotho	Iron [1.15]	Neonatal [1.07]	Vit A [0.95]	Dermatitis [1.33]	Diarrhea [0.96]	Congenital [0.91]	HIV [81.41]	Urticaria [0.85]	Skin Viral [0.88]	Skin Fung [1.79]
Namibia	Neonatal [1.19]	Iron [2.52]	Dermatitis [1.24]	Diarrhea [1.63]	Vit A [1.05]	Congenital [0.85]	PEM [49.17]	Urticaria [0.85]	Skin Viral [0.85]	URI [1.0]
S Africa	Neonatal [1.22]	Dermatitis [1.14]	Congenital [1.37]	Vit A [1.08]	Urticaria [0.94]	Skin Viral [0.82]	URI [0.89]	Epilepsy [1.09]	Iron [0.71]	ASD [0.87]
Swaziland	Neonatal [1.02]	Dermatitis [1.3]	Diarrhea [1.49]	Iron [1.08]	Vit A [0.77]	Congenital [0.88]	Urticaria [0.85]	HIV [122.68]	Asthma [0.43]	Skin Viral [0.87]
Zimbabwe	Neonatal [1.15]	Dermatitis [1.34]	Diarrhea [1.44]	Iron [0.67]	Vit A [0.6]	Congenital [0.78]	Urticaria [0.87]	Skin Viral [0.89]	Skin Fung [1.66]	URI [0.94]
**Western Sub-Saharan Africa**	Iron [1.67]	Vit A [1.07]	Neonatal [0.89]	Diarrhea [1.06]	Dermatitis [0.82]	PEM [5.0]	Congenital [1.16]	Malaria [16.69]	Hemog [2.33]	Asthma [0.56]
Benin	Iron [0.82]	Vit A [0.82]	Neonatal [1.23]	Malaria [7.19]	Dermatitis [0.88]	PEM [2.14]	Diarrhea [0.55]	Congenital [0.98]	Epilepsy [1.22]	Asthma [0.6]
Burkina Faso	Iron [1.82]	Vit A [1.02]	Malaria [2.66]	Neonatal [1.05]	Diarrhea [0.81]	Dermatitis [0.91]	PEM [1.05]	Hemog [1.92]	Congenital [0.99]	Asthma [0.76]
Cameroon	Iron [1.04]	Vit A [1.03]	Diarrhea [1.69]	Neonatal [0.91]	Dermatitis [0.87]	Malaria [37.55]	Congenital [1.03]	Epilepsy [1.4]	Asthma [0.54]	Urticaria [0.86]
Cape Verde	Neonatal [1.5]	Iron [1.44]	Dermatitis [0.83]	Diarrhea [0.76]	Congenital [0.93]	Asthma [0.58]	Epilepsy [1.32]	Urticaria [0.85]	Skin Viral [0.88]	URI [0.7]
Chad	Iron [1.55]	Vit A [0.8]	Diarrhea [1.39]	Neonatal [0.89]	PEM [1.07]	Dermatitis [0.92]	Congenital [0.96]	Malaria [0.83]	Oth NTD [0.84]	Epilepsy [1.0]
Cote d'Ivoire	Iron [1.36]	Vit A [1.12]	Neonatal [1.04]	Diarrhea [1.33]	Dermatitis [0.85]	Congenital [1.01]	Hemog [2.31]	Asthma [0.65]	Epilepsy [1.28]	Malaria [6.92]
Gambia	Iron [1.77]	Vit A [1.03]	Neonatal [1.53]	Diarrhea [1.15]	Dermatitis [0.86]	PEM [2.6]	Congenital [0.95]	Asthma [0.57]	Urticaria [0.87]	Hemog [1.71]
Ghana	Iron [1.84]	Vit A [1.6]	Neonatal [1.22]	Diarrhea [1.26]	Congenital [1.02]	Dermatitis [0.54]	Malaria [104.25]	Epilepsy [1.41]	Urticaria [0.85]	Asthma [0.45]
Guinea	Iron [1.34]	Vit A [0.86]	Neonatal [1.06]	Diarrhea [0.98]	Malaria [3.45]	Dermatitis [0.9]	Congenital [1.0]	Hemog [1.98]	Asthma [0.75]	Epilepsy [1.05]
Guinea-Bissau	Iron [1.35]	Vit A [0.98]	Neonatal [1.3]	Diarrhea [0.77]	Dermatitis [0.89]	Congenital [1.06]	Epilepsy [1.16]	Asthma [0.58]	Urticaria [0.91]	Skin Viral [0.93]
Liberia	Iron [1.2]	Vit A [0.68]	Diarrhea [1.48]	Neonatal [1.23]	Dermatitis [0.9]	Malaria [2.22]	Congenital [1.0]	Hemog [1.93]	Epilepsy [1.38]	Asthma [0.63]
Mali	Iron [1.67]	Vit A [0.89]	Neonatal [1.02]	PEM [1.04]	Diarrhea [0.61]	Malaria [1.55]	Dermatitis [0.85]	Congenital [0.97]	Oth NTD [0.93]	Urticaria [0.93]

eFigure 9c. Leading ten causes of YLDs with the ratio of observed YLDs to YLDs expected on the basis of Socio-Demographic Index alone in 2017, 1-4 years, both sexes combined.
The top ten causes contributing to YLDs are listed globally, by socio-demographic quintile, and then by GBD superregion, region, country, and subnationally where modeled. For each cell, the ratio of observed YLDs to YLDs expected on the basis of socio-demographic index (SDI) alone are listed. Abbreviations: YLD=year of life lived with disability, GBD=Global Burden of Disease.

Values shown in brackets represent the ratio of observed YLDs to predicted YLDs on the basis of Socio-Demographic Index (SDI), rounded to two (2) digits. Color ranges (shown below) were calculated to place a roughly equal number of cells into each bin.

COLOR KEY:	[0.0-0.72]	(0.72-0.84]	(0.84-0.92]	(0.92-1.01]	(1.01-1.09]	(1.09-1.17]	(1.17-1.32]	(1.32-1.67]	1.67+

	1	2	3	4	5	6	7	8	9	10
Mauritania	PEM [53.68]	Iron [1.83]	Vit A [1.32]	Neonatal [1.36]	Diarrhea [1.04]	Dermatitis [0.83]	Asthma [0.76]	Congenital [0.94]	Epilepsy [1.25]	Urticaria [0.84]
Niger	Iron [0.86]	Vit A [0.65]	PEM [0.89]	Neonatal [1.01]	Diarrhea [0.67]	Malaria [1.32]	Dermatitis [0.95]	Congenital [0.95]	Asthma [0.68]	Hemog [0.86]
Nigeria	Iron [2.01]	Vit A [1.2]	Neonatal [0.74]	Dermatitis [0.81]	PEM [10.78]	Diarrhea [0.96]	Congenital [1.38]	Malaria [48.34]	Hemog [3.21]	Asthma [0.54]
Sao Tome Principe	Neonatal [1.37]	Vit A [0.83]	Diarrhea [1.24]	Dermatitis [0.84]	Iron [0.54]	Congenital [1.0]	Asthma [0.56]	Epilepsy [1.29]	Urticaria [0.86]	Skin Viral [0.89]
Senegal	Iron [1.26]	Neonatal [1.52]	Vit A [0.85]	Diarrhea [1.3]	Dermatitis [0.88]	Congenital [0.98]	Epilepsy [1.12]	Urticaria [0.89]	Asthma [0.5]	Skin Viral [0.92]
Sierra Leone	Iron [1.6]	Vit A [0.87]	Malaria [6.98]	Neonatal [0.89]	Diarrhea [0.76]	Dermatitis [0.88]	Hemog [2.53]	Congenital [0.9]	Epilepsy [1.26]	Asthma [0.62]
Togo	Iron [1.35]	Vit A [0.97]	Neonatal [1.26]	Diarrhea [1.29]	Malaria [21.01]	Dermatitis [0.85]	PEM [3.06]	Congenital [1.0]	Asthma [0.66]	Epilepsy [1.23]

eFigure 9d. Leading ten causes of YLDs with the ratio of observed YLDs to YLDs expected on the basis of Socio-Demographic Index alone in 2017, 5-9 years, both sexes combined.
The top ten causes contributing to YLDs are listed globally, by socio-demographic quintile, and then by GBD superregion, region, country, and subnationally where modeled. For each cell, the ratio of observed YLDs to YLDs expected on the basis of socio-demographic index (SDI) alone are listed. Abbreviations: YLD=year of life lived with disability, GBD=Global Burden of Disease.

Values shown in brackets represent the ratio of observed YLDs to predicted YLDs on the basis of Socio-Demographic Index (SDI), rounded to two (2) digits. Color ranges (shown below) were calculated to place a roughly equal number of cells into each bin.

COLOR KEY:	[0.0-0.8]	(0.8-0.89]	(0.89-0.97]	(0.97-1.02]	(1.02-1.1]	(1.1-1.24]	(1.24-1.42]	(1.42-1.91]	1.91+

	1	2	3	4	5	6	7	8	9	10
Global	Iron [2.11]	Vit A [1.74]	Neonatal [1.07]	Dermatitis [0.78]	Asthma [0.74]	Skin Viral [0.9]	Congenital [0.99]	Conduct [0.86]	Anxiety [0.85]	Diarrhea [1.06]
Low SDI	Iron [1.24]	Vit A [1.04]	Neonatal [1.42]	Dermatitis [0.85]	Congenital [1.05]	Asthma [0.66]	Skin Viral [0.93]	Diarrhea [0.91]	Conduct [0.92]	Epilepsy [0.92]
Low-middle SDI	Iron [1.77]	Vit A [1.32]	Neonatal [1.13]	Dermatitis [0.77]	Asthma [0.65]	Congenital [1.06]	Skin Viral [0.89]	Conduct [0.9]	Diarrhea [1.0]	Hemog [2.18]
Middle SDI	Neonatal [1.18]	Iron [1.07]	Vit A [1.14]	Asthma [0.87]	Dermatitis [0.69]	Skin Viral [0.93]	Congenital [0.92]	Conduct [0.81]	Anxiety [0.76]	Epilepsy [0.94]
High-middle SDI	Neonatal [1.31]	Asthma [1.0]	Dermatitis [0.59]	Iron [3.49]	Skin Viral [0.94]	Conduct [0.86]	Vit A [2.36]	Congenital [0.91]	Anxiety [0.71]	Diarrhea [1.9]
High SDI	Dermatitis [1.18]	Neonatal [0.88]	Asthma [0.97]	Anxiety [1.16]	Skin Viral [1.01]	Conduct [1.0]	Congenital [0.89]	URI [1.16]	Falls [1.28]	ASD [1.2]
Central Europe, Eastern Europe, and Central Asia	Neonatal [1.35]	Iron [2.39]	Dermatitis [0.59]	Vit A [1.28]	Congenital [1.06]	Conduct [0.92]	Asthma [0.61]	Upper Digest [6.45]	Falls [1.62]	Skin Viral [0.8]
Central Asia	Iron [2.18]	Vit A [1.53]	Neonatal [0.94]	Dermatitis [0.52]	Congenital [1.04]	Conduct [0.91]	Asthma [0.55]	Skin Viral [0.81]	Epilepsy [1.28]	Urticaria [1.1]
Armenia	Iron [3.52]	Neonatal [0.96]	Dermatitis [0.52]	Congenital [1.04]	Conduct [0.91]	Diarrhea [1.44]	Skin Viral [0.81]	Asthma [0.53]	Epilepsy [1.21]	Urticaria [1.15]
Azerbaijan	Neonatal [0.92]	Iron [1.07]	Vit A [0.88]	Dermatitis [0.52]	Congenital [1.02]	Conduct [0.92]	Skin Viral [0.81]	Epilepsy [1.34]	Asthma [0.52]	Diarrhea [1.27]
Georgia	Neonatal [0.96]	Iron [1.08]	Vit A [1.07]	Congenital [1.06]	Conduct [0.91]	Dermatitis [0.47]	Skin Viral [0.81]	Diarrhea [1.43]	Epilepsy [1.26]	Asthma [0.5]
Kazakhstan	Vit A [3.06]	Neonatal [1.02]	Iron [1.74]	Congenital [1.08]	Dermatitis [0.47]	Conduct [0.9]	Skin Viral [0.8]	Epilepsy [1.45]	Asthma [0.45]	Urticaria [1.25]
Kyrgyzstan	Iron [1.79]	Vit A [1.2]	Neonatal [1.01]	Dermatitis [0.56]	Congenital [1.07]	Asthma [0.6]	Hernia [5.31]	Conduct [0.93]	Skin Viral [0.85]	Epilepsy [1.06]
Mongolia	Vit A [2.03]	Iron [1.5]	Neonatal [0.98]	Dermatitis [0.54]	Congenital [1.07]	Conduct [0.91]	Epilepsy [1.27]	Skin Viral [0.82]	Asthma [0.53]	Urticaria [1.08]
Tajikistan	Iron [1.02]	Vit A [1.15]	Neonatal [1.05]	Dermatitis [0.58]	Congenital [1.07]	Conduct [0.96]	Epilepsy [1.08]	Skin Viral [0.87]	Diarrhea [0.93]	Asthma [0.47]
Turkmenistan	Iron [1.36]	Neonatal [0.84]	Vit A [1.29]	Dermatitis [0.52]	Congenital [1.05]	Conduct [0.91]	Skin Viral [0.81]	Epilepsy [1.3]	Asthma [0.52]	Urticaria [1.15]
Uzbekistan	Iron [2.48]	Neonatal [0.9]	Vit A [0.99]	Dermatitis [0.55]	Asthma [0.62]	Congenital [1.03]	Conduct [0.92]	Epilepsy [1.22]	Skin Viral [0.83]	Urticaria [1.04]
Central Europe	Neonatal [1.39]	Dermatitis [0.64]	Vit A [2.77]	Falls [2.06]	Diarrhea [2.76]	Congenital [1.19]	Asthma [0.67]	Conduct [0.9]	Iron [1.98]	Skin Viral [0.79]
Albania	Neonatal [1.17]	Vit A [1.65]	Falls [3.22]	Congenital [1.13]	Iron [0.66]	Conduct [0.91]	Diarrhea [1.44]	Dermatitis [0.46]	Skin Viral [0.8]	Upper Digest [5.33]
Bosnia	Neonatal [1.35]	Vit A [1.87]	Falls [3.05]	Dermatitis [0.58]	Congenital [1.15]	Iron [0.83]	Asthma [0.64]	Conduct [0.9]	Diarrhea [1.49]	Skin Viral [0.8]
Bulgaria	Neonatal [1.24]	Vit A [2.07]	Falls [2.12]	Congenital [1.23]	Dermatitis [0.52]	Diarrhea [2.32]	Conduct [0.9]	Skin Viral [0.79]	Asthma [0.54]	Anxiety [0.69]
Croatia	Neonatal [1.51]	Dermatitis [0.54]	Congenital [1.17]	Diarrhea [2.42]	Conduct [0.9]	Falls [1.34]	Asthma [0.63]	Vit A [1.96]	Skin Viral [0.8]	Anxiety [0.64]
Czech	Neonatal [0.93]	Falls [2.31]	Dermatitis [0.66]	Diarrhea [3.24]	Congenital [1.16]	Conduct [0.91]	Skin Viral [0.81]	Asthma [0.54]	Anxiety [0.63]	Urticaria [1.65]
Hungary	Neonatal [1.34]	Diarrhea [4.17]	Dermatitis [0.75]	Falls [2.11]	Congenital [1.23]	Vit A [2.02]	Conduct [0.9]	Skin Viral [0.8]	Asthma [0.52]	Anxiety [0.64]
Macedonia	Neonatal [1.33]	Vit A [2.38]	Iron [1.79]	Falls [2.44]	Dermatitis [0.54]	Congenital [1.13]	Diarrhea [1.96]	Asthma [0.66]	Conduct [0.9]	Skin Viral [0.8]
Montenegro	Neonatal [1.31]	Vit A [2.67]	Falls [2.22]	Dermatitis [0.56]	Congenital [1.15]	Iron [1.57]	Conduct [0.9]	Skin Viral [0.79]	Asthma [0.59]	Upper Digest [5.32]
Poland	Neonatal [1.55]	Dermatitis [0.73]	Falls [1.86]	Asthma [0.87]	Congenital [1.2]	Diarrhea [2.71]	Vit A [2.68]	Upper Digest [7.88]	Conduct [0.91]	Skin Viral [0.81]
Romania	Neonatal [1.43]	Iron [4.52]	Vit A [3.43]	Diarrhea [3.1]	Upper Digest [9.95]	Falls [2.17]	Dermatitis [0.6]	Congenital [1.18]	Asthma [0.7]	Conduct [0.89]
Serbia	Neonatal [1.72]	Vit A [1.57]	Falls [2.6]	Dermatitis [0.56]	Congenital [1.22]	Conduct [0.91]	Diarrhea [1.73]	Skin Viral [0.8]	Anxiety [0.7]	Asthma [0.45]
Slovakia	Neonatal [1.03]	Dermatitis [0.66]	Diarrhea [3.52]	Vit A [3.42]	Falls [1.89]	Congenital [1.26]	Upper Digest [8.12]	Conduct [0.91]	Skin Viral [0.8]	Anxiety [0.63]
Slovenia	Neonatal [1.1]	Falls [2.23]	Diarrhea [3.23]	Dermatitis [0.54]	Congenital [1.26]	Asthma [0.77]	Vit A [2.75]	Conduct [0.92]	Skin Viral [0.81]	Upper Digest [6.21]

eFigure 9d. Leading ten causes of YLDs with the ratio of observed YLDs to YLDs expected on the basis of Socio-Demographic Index alone in 2017, 5-9 years, both sexes combined.
The top ten causes contributing to YLDs are listed globally, by socio-demographic quintile, and then by GBD superregion, region, country, and subnationally where modeled. For each cell, the ratio of observed YLDs to YLDs expected on the basis of socio-demographic index (SDI) alone are listed. Abbreviations: YLD=year of life lived with disability, GBD=Global Burden of Disease.

Values shown in brackets represent the ratio of observed YLDs to predicted YLDs on the basis of Socio-Demographic Index (SDI), rounded to two (2) digits. Color ranges (shown below) were calculated to place a roughly equal number of cells into each bin.

COLOR KEY:	[0.0-0.8]	(0.8-0.89]	(0.89-0.97]	(0.97-1.02]	(1.02-1.1]	(1.1-1.24]	(1.24-1.42]	(1.42-1.91]	1.91+

	1	2	3	4	5	6	7	8	9	10
**Eastern Europe**	Neonatal [1.62]	Iron [2.37]	Upper Digest [10.59]	Dermatitis [0.62]	Conduct [0.94]	Falls [1.64]	Congenital [1.01]	Asthma [0.62]	Skin Viral [0.79]	Diarrhea [1.58]
Belarus	Neonatal [1.59]	Dermatitis [0.74]	Upper Digest [11.12]	Asthma [0.73]	Falls [1.65]	Conduct [0.9]	Congenital [0.99]	Skin Viral [0.8]	Urticaria [1.35]	Iron [0.84]
Estonia	Neonatal [1.69]	Dermatitis [0.91]	Upper Digest [11.64]	Iron [3.49]	Falls [1.3]	Conduct [0.92]	Diarrhea [2.52]	Congenital [1.04]	Skin Viral [0.82]	Asthma [0.52]
Latvia	Neonatal [1.52]	Upper Digest [11.97]	Dermatitis [0.64]	Diarrhea [2.46]	Asthma [0.73]	Falls [1.44]	Conduct [0.9]	Iron [2.29]	Congenital [0.96]	Skin Viral [0.81]
Lithuania	Neonatal [1.74]	Upper Digest [14.1]	Diarrhea [2.89]	Congenital [1.11]	Falls [1.37]	Conduct [0.91]	Skin Viral [0.81]	Asthma [0.63]	Dermatitis [0.37]	Urticaria [1.59]
Moldova	Neonatal [1.67]	Iron [1.67]	Upper Digest [11.88]	Dermatitis [0.79]	Falls [2.61]	Congenital [0.99]	Conduct [0.9]	Asthma [0.56]	Skin Viral [0.81]	Urticaria [1.11]
Russian Federation	Neonatal [1.62]	Iron [2.55]	Upper Digest [10.45]	Dermatitis [0.62]	Falls [1.63]	Conduct [0.94]	Congenital [1.0]	Skin Viral [0.79]	Asthma [0.58]	Diarrhea [1.8]
Ukraine	Neonatal [1.6]	Iron [1.66]	Upper Digest [10.12]	Dermatitis [0.64]	Asthma [0.71]	Congenital [1.06]	Conduct [0.95]	Falls [1.84]	Skin Viral [0.8]	Urticaria [1.37]
**High-income**	Dermatitis [1.16]	Neonatal [0.89]	Asthma [1.1]	Anxiety [1.08]	Skin Viral [1.03]	Conduct [1.02]	Congenital [0.92]	URI [1.07]	Falls [0.81]	ASD [1.14]
**Australasia**	Asthma [2.05]	Dermatitis [1.07]	Falls [2.43]	Neonatal [0.9]	Anxiety [1.15]	Conduct [1.07]	Skin Viral [1.01]	URI [0.95]	Congenital [0.76]	Mech [5.31]
Australia	Asthma [2.09]	Dermatitis [1.01]	Neonatal [0.88]	Falls [2.31]	Anxiety [1.11]	Conduct [1.07]	Skin Viral [1.01]	URI [0.94]	Congenital [0.76]	ASD [1.02]
New Zealand	Dermatitis [1.36]	Asthma [1.82]	Falls [3.16]	Neonatal [1.01]	Anxiety [1.38]	Conduct [1.09]	Skin Viral [0.98]	Mech [7.47]	URI [0.99]	Congenital [0.76]
**High-income Asia Pacific**	Dermatitis [1.24]	Neonatal [1.16]	Asthma [0.93]	Skin Viral [1.01]	Anxiety [0.8]	Falls [1.16]	Conduct [0.88]	Diarrhea [2.36]	Congenital [0.94]	URI [0.97]
Brunei	Dermatitis [1.16]	Neonatal [1.13]	Iron [5.03]	Asthma [0.85]	Skin Viral [1.0]	Anxiety [0.83]	Conduct [0.85]	Diarrhea [2.32]	Falls [1.1]	Congenital [0.76]
Japan	Dermatitis [1.3]	Neonatal [1.14]	Asthma [0.94]	Skin Viral [1.0]	Falls [1.25]	Conduct [0.91]	Congenital [1.04]	Anxiety [0.78]	Diarrhea [2.22]	URI [0.99]
Aichi	Dermatitis [1.31]	Neonatal [1.17]	Asthma [0.96]	Skin Viral [1.01]	ASD [1.98]	Falls [1.21]	Conduct [0.91]	Anxiety [0.78]	Congenital [0.95]	Diarrhea [2.35]
Akita	Dermatitis [1.31]	Neonatal [1.23]	Asthma [0.91]	Skin Viral [0.98]	Congenital [1.13]	Falls [1.37]	Conduct [0.89]	Anxiety [0.81]	Diarrhea [2.08]	Iron [1.95]
Aomori	Dermatitis [1.31]	Neonatal [1.15]	Asthma [0.9]	Skin Viral [0.98]	Falls [1.39]	Conduct [0.89]	Anxiety [0.81]	Diarrhea [2.07]	Vit A [1.9]	Congenital [0.86]
Chiba	Dermatitis [1.31]	Neonatal [1.13]	Asthma [0.94]	Skin Viral [1.0]	Falls [1.27]	Congenital [1.07]	Conduct [0.9]	Anxiety [0.79]	URI [1.0]	Vit A [2.06]
Ehime	Dermatitis [1.31]	Neonatal [0.94]	Asthma [0.9]	Skin Viral [0.98]	Falls [1.33]	Conduct [0.9]	Anxiety [0.8]	Congenital [0.95]	Vit A [1.94]	URI [1.01]
Fukui	Dermatitis [1.31]	Neonatal [1.12]	Asthma [0.92]	Skin Viral [0.99]	Falls [1.29]	Conduct [0.9]	Anxiety [0.79]	Diarrhea [2.31]	Congenital [0.92]	URI [1.0]
Fukuoka	Dermatitis [1.31]	Neonatal [1.12]	Asthma [0.94]	Skin Viral [1.0]	Falls [1.28]	Conduct [0.9]	Anxiety [0.79]	Congenital [0.98]	Diarrhea [1.95]	Iron [2.35]
Fukushima	Dermatitis [1.31]	Neonatal [1.06]	Asthma [0.89]	Skin Viral [0.98]	Diarrhea [2.38]	Falls [1.37]	Congenital [1.03]	Conduct [0.89]	Anxiety [0.8]	URI [1.01]
Gifu	Dermatitis [1.31]	Neonatal [1.16]	Asthma [0.93]	Skin Viral [0.99]	Iron [3.04]	Falls [1.3]	Conduct [0.9]	Diarrhea [2.33]	Anxiety [0.79]	Congenital [0.82]
Gunma	Dermatitis [1.3]	Neonatal [1.1]	Asthma [0.93]	Skin Viral [0.99]	Falls [1.3]	Conduct [0.9]	Diarrhea [2.34]	Anxiety [0.79]	Congenital [0.98]	Iron [2.23]
Hiroshima	Dermatitis [1.31]	Neonatal [1.18]	Asthma [0.95]	Skin Viral [1.0]	Falls [1.23]	Conduct [0.91]	Anxiety [0.78]	Diarrhea [2.3]	Congenital [0.92]	URI [0.99]
Hokkaido	Dermatitis [1.31]	Neonatal [1.1]	Asthma [0.92]	Skin Viral [0.98]	Falls [1.34]	Diarrhea [2.34]	Conduct [0.89]	Congenital [1.02]	Anxiety [0.8]	Vit A [2.18]
Hyogo	Dermatitis [1.31]	Neonatal [1.15]	Asthma [0.8]	Skin Viral [1.0]	Falls [1.25]	Conduct [0.9]	Anxiety [0.78]	Diarrhea [2.35]	URI [1.0]	Iron [2.39]
Ibaraki	Dermatitis [1.31]	Neonatal [1.19]	Asthma [0.93]	Skin Viral [0.99]	Congenital [1.09]	Falls [1.28]	Conduct [0.9]	Anxiety [0.79]	Vit A [2.37]	Diarrhea [1.99]
Ishikawa	Dermatitis [1.31]	Neonatal [1.1]	Asthma [0.94]	Skin Viral [0.99]	Falls [1.28]	Conduct [0.89]	Congenital [1.02]	Anxiety [0.79]	Diarrhea [2.32]	URI [1.0]
Iwate	Dermatitis [1.31]	Neonatal [1.18]	Asthma [0.9]	Skin Viral [0.97]	Congenital [1.08]	Falls [1.39]	Conduct [0.89]	Anxiety [0.81]	Diarrhea [2.07]	Vit A [1.62]

eFigure 9d. Leading ten causes of YLDs with the ratio of observed YLDs to YLDs expected on the basis of Socio-Demographic Index alone in 2017, 5-9 years, both sexes combined.
The top ten causes contributing to YLDs are listed globally, by socio-demographic quintile, and then by GBD superregion, region, country, and subnationally where modeled. For each cell, the ratio of observed YLDs to YLDs expected on the basis of socio-demographic index (SDI) alone are listed. Abbreviations: YLD=year of life lived with disability, GBD=Global Burden of Disease.

Values shown in brackets represent the ratio of observed YLDs to predicted YLDs on the basis of Socio-Demographic Index (SDI), rounded to two (2) digits. Color ranges (shown below) were calculated to place a roughly equal number of cells into each bin.

COLOR KEY:	[0.0-0.8]	(0.8-0.89]	(0.89-0.97]	(0.97-1.02]	(1.02-1.1]	(1.1-1.24]	(1.24-1.42]	(1.42-1.91]	1.91+

	1	2	3	4	5	6	7	8	9	10
Kagawa	Dermatitis [1.3]	Neonatal [1.08]	Asthma [0.92]	Diarrhea [3.07]	Skin Viral [0.99]	Falls [1.28]	Conduct [0.9]	Anxiety [0.79]	Congenital [0.99]	URI [1.0]
Kagoshima	Dermatitis [1.31]	Neonatal [0.98]	Asthma [0.89]	Skin Viral [0.98]	Congenital [1.14]	Falls [1.36]	Conduct [0.89]	Diarrhea [2.17]	Anxiety [0.81]	Iron [2.0]
Kanagawa	Dermatitis [1.31]	Neonatal [1.19]	Asthma [0.98]	Skin Viral [1.01]	Congenital [1.2]	Falls [1.23]	Conduct [0.91]	Anxiety [0.78]	ASD [1.59]	URI [0.99]
Kochi	Dermatitis [1.31]	Neonatal [1.05]	Asthma [0.89]	Skin Viral [0.98]	Falls [1.36]	Conduct [0.88]	Congenital [0.97]	Anxiety [0.81]	Diarrhea [2.09]	Iron [1.79]
Kumamoto	Dermatitis [1.31]	Neonatal [1.03]	Asthma [0.9]	Skin Viral [0.98]	Falls [1.37]	Conduct [0.89]	Anxiety [0.81]	Congenital [0.92]	Iron [2.05]	Diarrhea [1.87]
Kyoto	Neonatal [1.19]	Dermatitis [1.04]	Asthma [0.97]	Congenital [1.29]	Skin Viral [1.01]	Falls [1.22]	Conduct [0.92]	Anxiety [0.77]	Diarrhea [2.39]	URI [0.98]
Mie	Dermatitis [1.31]	Neonatal [1.17]	Asthma [0.93]	Skin Viral [0.99]	Diarrhea [2.73]	Falls [1.26]	Conduct [0.9]	Anxiety [0.79]	Congenital [0.91]	URI [1.0]
Miyagi	Dermatitis [1.31]	Neonatal [1.09]	Skin Viral [0.99]	Asthma [0.77]	Congenital [1.15]	Falls [1.3]	Conduct [0.9]	Anxiety [0.79]	Diarrhea [2.1]	URI [1.0]
Miyazaki	Dermatitis [1.31]	Neonatal [1.03]	Asthma [0.89]	Skin Viral [0.98]	Falls [1.4]	Congenital [1.02]	Conduct [0.89]	Anxiety [0.81]	URI [1.01]	ASD [1.34]
Nagano	Dermatitis [1.31]	Neonatal [1.19]	Asthma [0.93]	Diarrhea [2.84]	Skin Viral [0.99]	Falls [1.31]	Conduct [0.9]	Anxiety [0.79]	Congenital [0.95]	URI [1.01]
Nagasaki	Dermatitis [1.31]	Neonatal [1.03]	Asthma [0.89]	Congenital [1.15]	Skin Viral [0.98]	Falls [1.39]	Conduct [0.89]	Anxiety [0.81]	Diarrhea [1.72]	URI [1.02]
Nara	Dermatitis [1.31]	Neonatal [1.02]	Asthma [0.92]	Congenital [1.26]	Skin Viral [0.99]	Falls [1.31]	Conduct [0.9]	Anxiety [0.8]	Diarrhea [2.26]	Vit A [2.1]
Niigata	Dermatitis [1.3]	Neonatal [1.15]	Asthma [0.93]	Skin Viral [0.99]	Iron [2.9]	Congenital [1.07]	Falls [1.33]	Conduct [0.89]	Anxiety [0.8]	Diarrhea [2.16]
Oita	Dermatitis [1.3]	Neonatal [1.2]	Asthma [0.91]	Skin Viral [0.99]	Diarrhea [2.5]	Falls [1.31]	Conduct [0.9]	Anxiety [0.79]	Congenital [0.88]	URI [1.0]
Okayama	Dermatitis [1.3]	Neonatal [1.21]	Asthma [0.94]	Skin Viral [0.99]	Falls [1.27]	Conduct [0.91]	Anxiety [0.79]	Diarrhea [2.08]	Congenital [0.87]	URI [0.99]
Okinawa	Dermatitis [1.31]	Neonatal [1.08]	Skin Viral [0.98]	Asthma [0.75]	Vit A [2.04]	Falls [1.43]	Conduct [0.88]	Anxiety [0.81]	Diarrhea [1.76]	Congenital [0.79]
Osaka	Dermatitis [1.31]	Neonatal [1.09]	Asthma [0.97]	Skin Viral [1.01]	Congenital [1.12]	Falls [1.25]	Conduct [0.91]	Anxiety [0.78]	Diarrhea [2.15]	URI [0.99]
Saga	Dermatitis [1.31]	Neonatal [1.08]	Asthma [0.91]	Skin Viral [0.98]	Falls [1.35]	Conduct [0.89]	Anxiety [0.8]	Congenital [0.93]	Diarrhea [2.07]	URI [1.01]
Saitama	Dermatitis [1.31]	Neonatal [1.12]	Asthma [0.94]	Skin Viral [0.99]	Falls [1.32]	Conduct [0.9]	Anxiety [0.79]	Diarrhea [2.08]	Congenital [0.88]	URI [1.0]
Shiga	Dermatitis [1.59]	Neonatal [1.08]	Asthma [0.96]	Skin Viral [1.0]	Falls [1.24]	Conduct [0.91]	Congenital [1.05]	Anxiety [0.78]	Diarrhea [2.38]	URI [0.99]
Shimane	Dermatitis [1.31]	Neonatal [1.06]	Asthma [0.89]	Skin Viral [0.98]	Falls [1.36]	Conduct [0.9]	Anxiety [0.8]	Diarrhea [2.12]	Congenital [0.86]	URI [1.01]
Shizuoka	Dermatitis [1.31]	Neonatal [1.25]	Asthma [0.94]	Skin Viral [1.0]	Falls [1.25]	Conduct [0.91]	Anxiety [0.79]	Congenital [0.94]	Diarrhea [2.15]	Vit A [2.24]
Tochigi	Dermatitis [1.31]	Neonatal [1.2]	Asthma [0.94]	Congenital [1.4]	Skin Viral [0.99]	Falls [1.27]	Conduct [0.9]	Anxiety [0.79]	URI [0.99]	ASD [1.33]
Tokushima	Dermatitis [1.3]	Neonatal [1.14]	Asthma [0.91]	Skin Viral [0.99]	Diarrhea [2.49]	Falls [1.29]	Conduct [0.9]	Anxiety [0.79]	Congenital [0.95]	URI [1.0]
Tokyo	Dermatitis [1.33]	Neonatal [1.25]	Asthma [1.08]	Congenital [1.3]	Skin Viral [1.05]	Falls [1.08]	Conduct [0.95]	Anxiety [0.76]	URI [0.97]	Diarrhea [2.49]
Tottori	Dermatitis [1.3]	Neonatal [1.14]	Asthma [0.9]	Skin Viral [0.98]	Congenital [1.11]	Falls [1.34]	Conduct [0.9]	Anxiety [0.8]	Iron [2.27]	Diarrhea [1.78]
Toyama	Dermatitis [1.3]	Neonatal [1.1]	Asthma [0.95]	Skin Viral [1.0]	Falls [1.25]	Conduct [0.91]	Iron [3.01]	Diarrhea [2.43]	Anxiety [0.78]	Congenital [0.96]
Wakayama	Dermatitis [1.31]	Neonatal [1.19]	Asthma [0.9]	Skin Viral [0.98]	Falls [1.33]	Conduct [0.9]	Iron [2.56]	Anxiety [0.8]	Diarrhea [2.12]	Vit A [1.91]
Yamagata	Dermatitis [1.31]	Neonatal [0.97]	Asthma [0.91]	Skin Viral [0.98]	Diarrhea [2.48]	Falls [1.4]	Conduct [0.89]	Anxiety [0.8]	Congenital [0.92]	URI [1.01]
Yamaguchi	Dermatitis [1.31]	Neonatal [1.01]	Asthma [0.92]	Skin Viral [0.99]	Falls [1.28]	Conduct [0.9]	Diarrhea [2.36]	Anxiety [0.79]	Congenital [0.87]	URI [1.0]
Yamanashi	Dermatitis [1.31]	Neonatal [1.26]	Asthma [0.94]	Skin Viral [1.0]	Falls [1.27]	Conduct [0.9]	Congenital [1.0]	Anxiety [0.79]	Diarrhea [2.13]	URI [1.0]

eFigure 9d. Leading ten causes of YLDs with the ratio of observed YLDs to YLDs expected on the basis of Socio-Demographic Index alone in 2017, 5-9 years, both sexes combined.
The top ten causes contributing to YLDs are listed globally, by socio-demographic quintile, and then by GBD superregion, region, country, and subnationally where modeled. For each cell, the ratio of observed YLDs to YLDs expected on the basis of socio-demographic index (SDI) alone are listed. Abbreviations: YLD=year of life lived with disability, GBD=Global Burden of Disease.

Values shown in brackets represent the ratio of observed YLDs to predicted YLDs on the basis of Socio-Demographic Index (SDI), rounded to two (2) digits. Color ranges (shown below) were calculated to place a roughly equal number of cells into each bin.

COLOR KEY:	[0.0-0.8]	(0.8-0.89]	(0.89-0.97]	(0.97-1.02]	(1.02-1.1]	(1.1-1.24]	(1.24-1.42]	(1.42-1.91]	1.91+

	1	2	3	4	5	6	7	8	9	10
S Korea	Neonatal [1.18]	Dermatitis [1.11]	Asthma [0.9]	Skin Viral [1.01]	Anxiety [0.85]	Diarrhea [2.58]	Conduct [0.82]	Iron [2.97]	Falls [1.01]	URI [0.93]
Singapore	Neonatal [1.17]	Dermatitis [1.03]	Asthma [0.88]	Skin Viral [1.01]	Anxiety [0.83]	Diarrhea [2.45]	Conduct [0.85]	Falls [1.06]	Iron [2.96]	URI [0.95]
**High-income North America**	Dermatitis [1.28]	Neonatal [0.88]	Asthma [1.4]	Skin Viral [1.16]	Conduct [1.18]	Anxiety [0.92]	Psoriasis [3.75]	Congenital [0.86]	URI [0.98]	ASD [1.24]
Canada	Dermatitis [1.38]	Asthma [1.59]	Skin Viral [1.28]	Neonatal [0.61]	Conduct [0.97]	Anxiety [0.77]	Psoriasis [3.73]	ASD [1.51]	URI [1.03]	Congenital [0.75]
Greenland	Dermatitis [1.33]	Asthma [1.42]	Neonatal [0.6]	Skin Viral [1.18]	Conduct [1.0]	Anxiety [0.96]	Vit A [0.85]	URI [1.13]	Iron [0.81]	Psoriasis [3.65]
USA	Dermatitis [1.27]	Neonatal [0.9]	Asthma [1.37]	Conduct [1.19]	Skin Viral [1.15]	Anxiety [0.94]	Psoriasis [3.76]	Congenital [0.86]	URI [0.97]	ASD [1.21]
Alabama	Dermatitis [1.12]	Neonatal [0.89]	Asthma [1.33]	Skin Viral [1.2]	Conduct [1.14]	Anxiety [0.96]	Iron [2.52]	Psoriasis [3.43]	URI [0.99]	Epilepsy [1.35]
Alaska	Dermatitis [1.12]	Neonatal [0.85]	Asthma [1.01]	Conduct [1.16]	Anxiety [0.94]	Skin Viral [0.83]	Congenital [0.93]	URI [0.97]	Psoriasis [3.07]	ASD [1.14]
Arizona	Dermatitis [1.3]	Asthma [1.45]	Neonatal [0.89]	Conduct [1.15]	Anxiety [0.95]	Skin Viral [0.89]	Iron [2.81]	Congenital [0.84]	Psoriasis [3.42]	URI [0.98]
Arkansas	Dermatitis [1.03]	Neonatal [0.78]	Asthma [0.97]	Conduct [1.14]	Anxiety [0.97]	Skin Viral [0.97]	Iron [1.99]	URI [0.99]	Congenital [0.71]	ASD [1.24]
California	Dermatitis [1.42]	Asthma [1.33]	Neonatal [0.75]	Conduct [1.18]	Anxiety [0.93]	Skin Viral [0.97]	Psoriasis [3.89]	ASD [1.39]	Congenital [0.81]	URI [0.97]
Colorado	Dermatitis [1.14]	Neonatal [0.92]	Asthma [1.32]	Conduct [1.18]	Anxiety [0.93]	Skin Viral [0.98]	Congenital [0.89]	URI [0.97]	Psoriasis [2.68]	Headaches [1.0]
Connecticut	Dermatitis [1.44]	Asthma [1.92]	Neonatal [1.08]	Skin Viral [1.53]	Conduct [1.19]	Anxiety [0.91]	Psoriasis [4.13]	Congenital [0.88]	URI [0.96]	Epilepsy [1.58]
Delaware	Dermatitis [1.14]	Asthma [1.55]	Neonatal [0.88]	Skin Viral [1.36]	Conduct [1.17]	Anxiety [0.93]	Psoriasis [4.09]	Congenital [0.89]	URI [0.97]	Epilepsy [1.62]
DC	Dermatitis [1.69]	Asthma [1.53]	Neonatal [0.93]	Skin Viral [1.36]	Conduct [1.17]	Anxiety [0.93]	Psoriasis [3.68]	Congenital [0.88]	URI [0.95]	ASD [1.1]
Florida	Dermatitis [1.28]	Neonatal [1.01]	Asthma [1.5]	Skin Viral [1.35]	Conduct [1.16]	Anxiety [0.94]	Psoriasis [4.71]	Iron [2.76]	Congenital [0.84]	URI [0.97]
Georgia	Dermatitis [1.39]	Asthma [1.44]	Neonatal [0.83]	Skin Viral [1.19]	Conduct [1.15]	Anxiety [0.95]	Psoriasis [4.18]	URI [0.98]	Congenital [0.78]	Epilepsy [1.45]
Hawaii	Dermatitis [1.49]	Asthma [1.52]	Neonatal [0.82]	Skin Viral [1.37]	Conduct [1.17]	Anxiety [0.94]	Psoriasis [3.86]	Congenital [0.84]	URI [0.98]	ASD [1.14]
Idaho	Dermatitis [1.04]	Neonatal [0.74]	Skin Viral [1.26]	Conduct [1.15]	Anxiety [0.96]	Asthma [0.72]	URI [0.99]	Congenital [0.77]	Psoriasis [2.82]	ASD [1.15]
Illinois	Dermatitis [1.12]	Neonatal [1.06]	Asthma [1.27]	Skin Viral [1.16]	Conduct [1.17]	Anxiety [0.93]	Congenital [0.93]	Psoriasis [3.57]	URI [0.97]	Epilepsy [1.5]
Indiana	Dermatitis [1.09]	Neonatal [0.85]	Asthma [1.28]	Iron [3.79]	Conduct [1.15]	Anxiety [0.95]	Skin Viral [1.0]	Congenital [0.94]	Psoriasis [3.24]	URI [0.98]
Iowa	Dermatitis [1.0]	Neonatal [0.87]	Skin Viral [1.17]	Conduct [1.17]	Anxiety [0.94]	Asthma [0.78]	Iron [3.53]	Congenital [0.84]	URI [0.97]	Psoriasis [2.86]
Kansas	Dermatitis [1.06]	Neonatal [0.92]	Asthma [1.33]	Conduct [1.16]	Skin Viral [1.05]	Anxiety [0.94]	URI [0.97]	Psoriasis [3.14]	Congenital [0.78]	ASD [1.14]
Kentucky	Dermatitis [1.12]	Neonatal [0.87]	Asthma [1.32]	Skin Viral [1.21]	Conduct [1.14]	Anxiety [0.97]	Iron [2.03]	Congenital [0.86]	Psoriasis [3.52]	URI [0.99]
Louisiana	Dermatitis [1.2]	Neonatal [0.81]	Skin Viral [1.59]	Asthma [1.04]	Conduct [1.14]	Anxiety [0.97]	Psoriasis [4.06]	Congenital [0.83]	URI [0.99]	Epilepsy [1.47]
Maine	Dermatitis [0.97]	Asthma [1.47]	Neonatal [0.91]	Conduct [1.17]	Skin Viral [1.05]	Anxiety [0.94]	Psoriasis [3.46]	Congenital [0.86]	URI [0.98]	ASD [1.15]
Maryland	Dermatitis [1.44]	Neonatal [1.06]	Asthma [1.6]	Skin Viral [1.18]	Conduct [1.19]	Anxiety [0.92]	Psoriasis [4.11]	Congenital [0.86]	URI [0.96]	ASD [1.23]
Massachusetts	Dermatitis [1.29]	Neonatal [1.03]	Asthma [1.65]	Skin Viral [1.42]	Conduct [1.2]	Anxiety [0.91]	Psoriasis [4.13]	Congenital [0.97]	ASD [1.5]	URI [0.97]
Michigan	Dermatitis [1.13]	Asthma [1.48]	Neonatal [0.89]	Skin Viral [1.36]	Conduct [1.16]	Anxiety [0.94]	Iron [3.26]	Congenital [0.95]	Psoriasis [3.59]	URI [0.97]
Minnesota	Dermatitis [1.0]	Neonatal [0.96]	Asthma [1.13]	Skin Viral [1.34]	Conduct [1.19]	Anxiety [0.93]	Congenital [0.94]	ASD [1.34]	URI [0.96]	Psoriasis [3.1]
Mississippi	Dermatitis [1.02]	Neonatal [0.85]	Asthma [0.89]	Conduct [1.13]	Iron [2.62]	Anxiety [0.98]	Skin Viral [0.97]	URI [0.99]	Psoriasis [3.08]	Congenital [0.66]

eFigure 9d. Leading ten causes of YLDs with the ratio of observed YLDs to YLDs expected on the basis of Socio-Demographic Index alone in 2017, 5-9 years, both sexes combined.
The top ten causes contributing to YLDs are listed globally, by socio-demographic quintile, and then by GBD superregion, region, country, and subnationally where modeled. For each cell, the ratio of observed YLDs to YLDs expected on the basis of socio-demographic index (SDI) alone are listed. Abbreviations: YLD=year of life lived with disability, GBD=Global Burden of Disease.

Values shown in brackets represent the ratio of observed YLDs to predicted YLDs on the basis of Socio-Demographic Index (SDI), rounded to two (2) digits. Color ranges (shown below) were calculated to place a roughly equal number of cells into each bin.

COLOR KEY:	[0.0-0.8]	(0.8-0.89]	(0.89-0.97]	(0.97-1.02]	(1.02-1.1]	(1.1-1.24]	(1.24-1.42]	(1.42-1.91]	1.91+

	1	2	3	4	5	6	7	8	9	10
Missouri	Dermatitis [1.01]	Neonatal [0.92]	Asthma [1.27]	Conduct [1.16]	Skin Viral [1.04]	Anxiety [0.95]	Congenital [0.88]	URI [0.98]	Psoriasis [3.22]	ASD [1.2]
Montana	Dermatitis [0.99]	Neonatal [0.87]	Skin Viral [1.19]	Asthma [0.93]	Conduct [1.16]	Anxiety [0.94]	Iron [2.55]	Congenital [0.8]	URI [0.96]	Psoriasis [2.85]
Nebraska	Dermatitis [1.0]	Neonatal [0.87]	Skin Viral [1.18]	Conduct [1.17]	Asthma [0.88]	Anxiety [0.94]	Congenital [0.88]	URI [0.97]	Psoriasis [3.01]	ASD [1.15]
Nevada	Dermatitis [1.23]	Neonatal [0.84]	Asthma [1.16]	Conduct [1.15]	Anxiety [0.96]	Skin Viral [0.71]	URI [0.99]	Psoriasis [2.99]	Congenital [0.7]	ASD [1.14]
New Hampshire	Dermatitis [1.01]	Neonatal [0.87]	Asthma [1.41]	Skin Viral [1.2]	Conduct [1.19]	Anxiety [0.92]	Psoriasis [3.83]	Congenital [0.9]	URI [0.97]	Iron [3.13]
New Jersey	Dermatitis [1.57]	Asthma [1.92]	Neonatal [0.97]	Skin Viral [1.4]	Conduct [1.19]	Anxiety [0.92]	Psoriasis [4.59]	ASD [1.65]	Congenital [1.03]	URI [0.96]
New Mexico	Dermatitis [1.13]	Neonatal [0.95]	Asthma [1.42]	Conduct [1.14]	Anxiety [0.97]	Skin Viral [0.72]	Congenital [0.83]	URI [0.99]	Epilepsy [1.28]	ASD [1.15]
New York	Dermatitis [1.62]	Asthma [2.07]	Neonatal [1.01]	Skin Viral [1.45]	Conduct [1.18]	Psoriasis [5.0]	Anxiety [0.92]	Congenital [1.04]	URI [0.96]	Epilepsy [1.6]
N Carolina	Dermatitis [1.18]	Neonatal [0.9]	Asthma [1.23]	Skin Viral [1.16]	Conduct [1.06]	Anxiety [0.9]	Psoriasis [3.76]	Congenital [0.83]	URI [0.98]	ASD [1.33]
N Dakota	Dermatitis [0.96]	Neonatal [0.95]	Asthma [1.12]	Skin Viral [1.14]	Conduct [1.18]	Anxiety [0.93]	Congenital [1.11]	URI [0.97]	Iron [2.63]	Psoriasis [2.9]
Ohio	Dermatitis [1.2]	Neonatal [0.86]	Asthma [1.36]	Conduct [1.16]	Skin Viral [1.12]	Anxiety [0.94]	Congenital [0.97]	Psoriasis [3.44]	URI [0.98]	Epilepsy [1.54]
Oklahoma	Dermatitis [1.25]	Asthma [1.41]	Neonatal [0.81]	Conduct [1.15]	Anxiety [0.96]	Skin Viral [0.94]	Congenital [0.81]	Iron [1.98]	URI [0.98]	Epilepsy [1.42]
Oregon	Dermatitis [1.32]	Neonatal [0.78]	Asthma [1.04]	Skin Viral [1.16]	Conduct [1.02]	Anxiety [0.87]	Psoriasis [3.64]	Congenital [0.85]	URI [0.97]	ASD [1.14]
Pennsylvania	Dermatitis [1.24]	Asthma [1.55]	Neonatal [0.9]	Skin Viral [1.2]	Conduct [1.17]	Anxiety [0.93]	Congenital [1.0]	Psoriasis [3.7]	URI [0.97]	Iron [2.72]
Rhode Island	Dermatitis [1.14]	Asthma [1.55]	Neonatal [0.88]	Skin Viral [1.43]	Conduct [1.18]	Anxiety [0.92]	Psoriasis [3.87]	Congenital [0.88]	URI [0.97]	ASD [1.14]
S Carolina	Dermatitis [1.37]	Neonatal [0.85]	Asthma [1.23]	Iron [4.38]	Skin Viral [1.19]	Conduct [1.14]	Anxiety [0.96]	Psoriasis [3.97]	Congenital [0.79]	URI [0.98]
S Dakota	Dermatitis [0.97]	Neonatal [0.77]	Skin Viral [1.29]	Conduct [1.16]	Anxiety [0.95]	Asthma [0.79]	Iron [2.43]	Congenital [0.81]	URI [0.97]	Psoriasis [2.92]
Tennessee	Dermatitis [1.12]	Neonatal [0.84]	Asthma [1.03]	Conduct [1.14]	Skin Viral [1.01]	Anxiety [0.96]	ASD [1.49]	Psoriasis [3.39]	URI [0.99]	Congenital [0.77]
Texas	Dermatitis [1.33]	Neonatal [0.96]	Asthma [1.44]	Conduct [1.49]	Anxiety [0.96]	Skin Viral [1.0]	Psoriasis [3.97]	ASD [1.6]	Congenital [0.79]	URI [0.98]
Utah	Dermatitis [1.06]	Neonatal [0.83]	Skin Viral [1.3]	Conduct [1.15]	Asthma [0.81]	Anxiety [0.95]	ASD [1.9]	Iron [3.2]	Congenital [0.81]	URI [0.98]
Vermont	Dermatitis [0.99]	Neonatal [0.97]	Asthma [1.26]	Conduct [1.19]	Anxiety [0.92]	Skin Viral [1.04]	Congenital [0.88]	Psoriasis [3.31]	URI [0.96]	ASD [1.14]
Virginia	Dermatitis [1.45]	Neonatal [0.98]	Asthma [1.43]	Conduct [1.18]	Skin Viral [1.06]	Anxiety [0.93]	Psoriasis [3.75]	URI [0.96]	Congenital [0.79]	Epilepsy [1.48]
Washington	Dermatitis [1.28]	Neonatal [0.83]	Asthma [1.02]	Skin Viral [1.22]	Conduct [1.18]	Anxiety [0.93]	Psoriasis [3.56]	Congenital [0.92]	URI [0.97]	ASD [1.14]
W Virginia	Dermatitis [1.14]	Asthma [1.35]	Neonatal [0.85]	Conduct [1.14]	Anxiety [0.97]	Skin Viral [0.97]	Iron [2.25]	Epilepsy [1.51]	Psoriasis [3.43]	Congenital [0.77]
Wisconsin	Dermatitis [1.0]	Neonatal [0.96]	Asthma [1.03]	Skin Viral [1.2]	Conduct [1.18]	Anxiety [0.93]	Congenital [0.94]	Psoriasis [3.2]	URI [0.97]	Epilepsy [1.52]
Wyoming	Dermatitis [1.05]	Neonatal [0.73]	Asthma [1.0]	Conduct [1.17]	Anxiety [0.94]	Skin Viral [0.91]	Congenital [0.87]	URI [0.97]	ASD [1.13]	Headaches [0.99]
**Southern Latin America**	Neonatal [0.95]	Dermatitis [1.0]	Asthma [0.98]	Anxiety [1.37]	Diarrhea [1.97]	Skin Viral [0.99]	Conduct [0.92]	Congenital [0.73]	Iron [0.5]	URI [0.89]
Argentina	Neonatal [0.94]	Dermatitis [0.94]	Asthma [0.96]	Anxiety [1.38]	Diarrhea [1.94]	Skin Viral [0.99]	Conduct [0.93]	Iron [0.54]	Congenital [0.74]	URI [0.89]
Chile	Dermatitis [1.19]	Neonatal [1.0]	Asthma [1.02]	Anxiety [1.32]	Diarrhea [2.24]	Skin Viral [0.99]	Conduct [0.89]	Congenital [0.72]	Epilepsy [1.15]	Vit A [0.62]
Uruguay	Neonatal [1.0]	Dermatitis [0.93]	Asthma [1.11]	Anxiety [1.39]	Skin Viral [0.99]	Conduct [0.93]	Iron [0.6]	Congenital [0.69]	Vit A [0.52]	Diarrhea [0.98]
**Western Europe**	Dermatitis [1.08]	Neonatal [0.81]	Anxiety [1.29]	Asthma [0.83]	Skin Viral [0.95]	Conduct [0.95]	Congenital [1.03]	URI [1.27]	Falls [0.92]	Headaches [0.96]

eFigure 9d. Leading ten causes of YLDs with the ratio of observed YLDs to YLDs expected on the basis of Socio-Demographic Index alone in 2017, 5-9 years, both sexes combined.
The top ten causes contributing to YLDs are listed globally, by socio-demographic quintile, and then by GBD superregion, region, country, and subnationally where modeled. For each cell, the ratio of observed YLDs to YLDs expected on the basis of socio-demographic index (SDI) alone are listed. Abbreviations: YLD=year of life lived with disability, GBD=Global Burden of Disease.

Values shown in brackets represent the ratio of observed YLDs to predicted YLDs on the basis of Socio-Demographic Index (SDI), rounded to two (2) digits. Color ranges (shown below) were calculated to place a roughly equal number of cells into each bin.

COLOR KEY:	[0.0-0.8]	(0.8-0.89]	(0.89-0.97]	(0.97-1.02]	(1.02-1.1]	(1.1-1.24]	(1.24-1.42]	(1.42-1.91]	1.91+

	1	2	3	4	5	6	7	8	9	10
Andorra	Dermatitis [1.48]	Neonatal [0.86]	Anxiety [1.22]	Asthma [1.0]	Skin Viral [0.99]	Conduct [0.96]	URI [1.24]	Congenital [1.07]	Falls [0.83]	Headaches [1.03]
Austria	Neonatal [1.34]	Dermatitis [0.75]	Anxiety [1.24]	Skin Viral [0.96]	Asthma [0.75]	Conduct [0.95]	Congenital [1.05]	URI [1.26]	Falls [0.93]	Headaches [1.01]
Belgium	Dermatitis [0.91]	Anxiety [1.19]	Neonatal [0.56]	Skin Viral [0.98]	Conduct [0.96]	URI [1.24]	Asthma [0.7]	Congenital [1.0]	Falls [1.02]	Headaches [1.28]
Cyprus	Neonatal [1.07]	Dermatitis [0.77]	Anxiety [1.23]	Asthma [0.77]	Skin Viral [0.96]	Conduct [0.95]	URI [1.26]	Congenital [0.98]	Falls [0.9]	Headaches [0.99]
Denmark	Dermatitis [1.26]	Anxiety [1.2]	Neonatal [0.69]	Skin Viral [1.15]	Asthma [0.93]	Iron [5.37]	Congenital [1.16]	URI [1.22]	Conduct [0.9]	Falls [0.79]
Finland	Neonatal [1.68]	Dermatitis [1.32]	Asthma [0.91]	Anxiety [0.9]	Skin Viral [1.01]	Conduct [0.93]	Congenital [1.08]	URI [1.23]	Falls [0.96]	Headaches [1.02]
France	Dermatitis [1.2]	Anxiety [1.46]	Neonatal [0.65]	Asthma [0.78]	Skin Viral [0.96]	Conduct [0.94]	URI [1.26]	Congenital [1.01]	Falls [0.93]	Epilepsy [1.17]
Germany	Neonatal [0.93]	Dermatitis [0.91]	Anxiety [1.45]	Skin Viral [0.98]	Conduct [0.95]	URI [1.25]	Congenital [1.02]	Epilepsy [2.05]	Asthma [0.57]	Falls [0.91]
Greece	Neonatal [0.84]	Anxiety [1.39]	Dermatitis [0.64]	Skin Viral [0.94]	Asthma [0.69]	Conduct [0.92]	URI [1.31]	Congenital [0.97]	Headaches [1.16]	Falls [1.03]
Iceland	Dermatitis [1.38]	Neonatal [0.87]	Asthma [1.4]	Anxiety [1.21]	Skin Viral [1.09]	Conduct [0.97]	URI [1.23]	Congenital [0.98]	Falls [0.82]	Headaches [1.03]
Ireland	Dermatitis [1.12]	Anxiety [1.33]	Asthma [1.18]	Neonatal [0.72]	Skin Viral [0.97]	Congenital [1.14]	Conduct [0.95]	URI [1.24]	Falls [0.92]	Headaches [1.01]
Israel	Dermatitis [1.0]	Neonatal [0.92]	Blindness [2.66]	Asthma [0.73]	Skin Viral [0.91]	Conduct [0.92]	Congenital [1.0]	URI [1.29]	Anxiety [0.81]	Falls [1.07]
Italy	Dermatitis [0.91]	Anxiety [1.32]	Neonatal [0.64]	Skin Viral [0.95]	Conduct [0.94]	URI [1.27]	Congenital [0.98]	Headaches [1.21]	Asthma [0.42]	Falls [0.79]
Luxembourg	Dermatitis [1.48]	Neonatal [1.13]	Anxiety [1.2]	Asthma [1.11]	Skin Viral [1.0]	Conduct [0.98]	URI [1.23]	Congenital [1.05]	Falls [0.79]	Headaches [1.16]
Malta	Dermatitis [0.79]	Neonatal [0.71]	Anxiety [1.27]	Asthma [0.83]	Congenital [1.19]	Skin Viral [0.94]	Conduct [0.93]	URI [1.27]	Falls [1.21]	Iron [1.96]
Netherlands	Dermatitis [1.18]	Neonatal [0.87]	Anxiety [1.43]	Asthma [0.92]	Conduct [1.09]	Skin Viral [0.92]	URI [1.23]	Congenital [1.08]	Headaches [1.14]	Falls [0.7]
Norway	Dermatitis [1.33]	Neonatal [1.04]	Anxiety [1.6]	Asthma [1.36]	Congenital [1.48]	Skin Viral [1.07]	Conduct [0.98]	URI [1.24]	Falls [1.05]	Headaches [1.04]
Portugal	Dermatitis [0.86]	Asthma [1.15]	Anxiety [1.34]	Neonatal [0.59]	Conduct [0.92]	Skin Viral [0.84]	Vit A [1.25]	URI [1.32]	Congenital [0.81]	Iron [0.96]
Spain	Neonatal [0.68]	Dermatitis [0.67]	Anxiety [1.21]	Skin Viral [0.92]	Conduct [0.93]	Asthma [0.68]	URI [1.29]	Congenital [0.93]	Iron [1.91]	Headaches [1.18]
Sweden	Dermatitis [1.69]	Asthma [1.26]	Neonatal [0.76]	Anxiety [1.19]	Skin Viral [1.07]	Congenital [1.2]	Conduct [0.98]	URI [1.25]	Falls [1.09]	ASD [1.1]
Stockholm	Dermatitis [1.35]	Neonatal [0.75]	Asthma [1.18]	Anxiety [1.17]	Skin Viral [1.1]	Congenital [1.25]	Conduct [0.99]	URI [1.23]	Falls [1.01]	ASD [1.38]
Sweden w/o Stockholm	Dermatitis [1.79]	Asthma [1.28]	Neonatal [0.77]	Anxiety [1.2]	Skin Viral [1.06]	Congenital [1.18]	Conduct [0.97]	URI [1.26]	Falls [1.12]	Headaches [0.96]
Switzerland	Dermatitis [1.47]	Neonatal [0.83]	Anxiety [1.22]	Asthma [0.9]	Skin Viral [0.98]	Conduct [0.96]	URI [1.24]	Congenital [1.04]	Falls [0.66]	ASD [1.02]
UK	Dermatitis [1.43]	Neonatal [0.97]	Asthma [1.38]	Iron [4.12]	Anxiety [1.08]	Congenital [1.15]	Conduct [0.98]	Skin Viral [0.89]	URI [1.28]	Falls [0.99]
England	Dermatitis [1.46]	Neonatal [0.99]	Asthma [1.41]	Iron [4.26]	Anxiety [1.05]	Congenital [1.15]	Conduct [0.98]	Skin Viral [0.88]	URI [1.27]	Falls [0.97]
E Midlands	Dermatitis [1.44]	Neonatal [0.94]	Asthma [1.3]	Iron [3.51]	Anxiety [1.07]	Congenital [1.19]	Conduct [0.97]	Skin Viral [0.87]	URI [1.29]	Falls [1.04]
Derby	Dermatitis [1.43]	Neonatal [0.95]	Asthma [1.33]	Iron [3.85]	Anxiety [1.05]	Congenital [1.2]	Conduct [0.98]	Skin Viral [0.88]	URI [1.27]	Falls [0.99]
Derbyshire	Dermatitis [1.44]	Neonatal [0.86]	Asthma [1.27]	Iron [3.92]	Anxiety [1.08]	Congenital [1.17]	Conduct [0.97]	Skin Viral [0.87]	URI [1.31]	Falls [1.09]
Leicester	Dermatitis [1.44]	Asthma [1.34]	Neonatal [0.81]	Anxiety [1.06]	Congenital [1.2]	Conduct [0.98]	Iron [2.84]	Skin Viral [0.88]	URI [1.27]	Falls [1.01]
Leicestershire	Dermatitis [1.44]	Asthma [1.32]	Neonatal [0.79]	Iron [4.19]	Anxiety [1.05]	Congenital [1.21]	Conduct [0.98]	Skin Viral [0.88]	URI [1.28]	Falls [0.99]
Lincolnshire	Dermatitis [1.44]	Neonatal [0.92]	Asthma [1.27]	Iron [2.98]	Anxiety [1.09]	Congenital [1.16]	Conduct [0.96]	Skin Viral [0.87]	URI [1.31]	Falls [1.1]

eFigure 9d. Leading ten causes of YLDs with the ratio of observed YLDs to YLDs expected on the basis of Socio-Demographic Index alone in 2017, 5-9 years, both sexes combined.
The top ten causes contributing to YLDs are listed globally, by socio-demographic quintile, and then by GBD superregion, region, country, and subnationally where modeled. For each cell, the ratio of observed YLDs to YLDs expected on the basis of socio-demographic index (SDI) alone are listed. Abbreviations: YLD=year of life lived with disability, GBD=Global Burden of Disease.

Values shown in brackets represent the ratio of observed YLDs to predicted YLDs on the basis of Socio-Demographic Index (SDI), rounded to two (2) digits. Color ranges (shown below) were calculated to place a roughly equal number of cells into each bin.

COLOR KEY:	[0.0-0.8]	(0.8-0.89]	(0.89-0.97]	(0.97-1.02]	(1.02-1.1]	(1.1-1.24]	(1.24-1.42]	(1.42-1.91]	1.91+

	1	2	3	4	5	6	7	8	9	10
Northamptonshire	Dermatitis [1.44]	Neonatal [1.03]	Asthma [1.3]	Iron [3.52]	Anxiety [1.07]	Congenital [1.19]	Conduct [0.97]	Skin Viral [0.87]	URI [1.29]	Falls [1.03]
Nottingham	Dermatitis [1.44]	Neonatal [1.15]	Asthma [1.4]	Anxiety [1.03]	Iron [3.96]	Congenital [1.22]	Conduct [1.0]	Skin Viral [0.89]	URI [1.26]	Falls [0.93]
Nottinghamshire	Dermatitis [1.44]	Neonatal [1.03]	Asthma [1.28]	Anxiety [1.08]	Iron [2.48]	Congenital [1.18]	Conduct [0.97]	Skin Viral [0.87]	URI [1.31]	Falls [1.09]
Rutland	Dermatitis [1.44]	Asthma [1.3]	Iron [3.92]	Neonatal [0.66]	Anxiety [1.07]	Congenital [1.18]	Conduct [0.97]	Skin Viral [0.88]	URI [1.3]	Falls [1.03]
E England	Dermatitis [1.44]	Neonatal [0.9]	Asthma [1.32]	Anxiety [1.06]	Iron [2.97]	Conduct [0.98]	Congenital [1.08]	Skin Viral [0.88]	URI [1.29]	Falls [1.01]
Bedford	Dermatitis [1.44]	Neonatal [1.1]	Asthma [1.32]	Iron [3.44]	Anxiety [1.06]	Conduct [0.98]	Congenital [1.08]	Skin Viral [0.88]	URI [1.27]	Falls [0.99]
Cambridgeshire	Dermatitis [1.43]	Neonatal [0.92]	Asthma [1.39]	Anxiety [1.03]	Conduct [1.0]	Congenital [1.12]	Skin Viral [0.89]	URI [1.27]	ASD [1.65]	Iron [2.92]
Cen Bedfordshire	Dermatitis [1.44]	Neonatal [0.98]	Asthma [1.3]	Anxiety [1.07]	Conduct [0.97]	Congenital [1.05]	Skin Viral [0.88]	Iron [2.51]	URI [1.29]	Falls [1.02]
Essex	Dermatitis [1.44]	Neonatal [0.89]	Asthma [1.3]	Anxiety [1.08]	Conduct [0.97]	Congenital [1.08]	Skin Viral [0.88]	URI [1.3]	Iron [2.3]	Falls [1.04]
Hertfordshire	Dermatitis [1.43]	Neonatal [0.89]	Asthma [1.39]	Anxiety [1.03]	Conduct [1.0]	Iron [3.54]	Congenital [1.09]	Skin Viral [0.89]	URI [1.28]	Falls [0.92]
Luton	Dermatitis [1.43]	Neonatal [1.02]	Asthma [1.33]	Anxiety [1.06]	Conduct [0.99]	Congenital [1.09]	Skin Viral [0.87]	URI [1.28]	Iron [2.29]	Falls [1.03]
Norfolk	Dermatitis [1.44]	Asthma [1.29]	Neonatal [0.76]	Iron [2.99]	Anxiety [1.07]	Conduct [0.98]	Congenital [1.07]	Skin Viral [0.87]	URI [1.3]	Falls [1.05]
Peterborough	Dermatitis [1.44]	Asthma [1.32]	Neonatal [0.71]	Iron [2.94]	Anxiety [1.07]	Conduct [0.98]	Congenital [1.06]	Skin Viral [0.87]	URI [1.29]	Falls [1.07]
Southend-on-Sea	Dermatitis [1.44]	Neonatal [1.06]	Asthma [1.28]	Iron [2.78]	Anxiety [1.09]	Conduct [0.97]	Congenital [1.06]	Skin Viral [0.87]	URI [1.31]	Falls [1.1]
Suffolk	Dermatitis [1.44]	Neonatal [0.94]	Asthma [1.29]	Iron [3.15]	Anxiety [1.07]	Conduct [0.97]	Congenital [1.06]	Skin Viral [0.87]	URI [1.31]	Falls [1.07]
Thurrock	Dermatitis [1.45]	Neonatal [0.99]	Asthma [1.29]	Iron [2.41]	Anxiety [1.1]	Conduct [0.96]	Congenital [1.05]	Skin Viral [0.87]	URI [1.32]	Falls [1.12]
Greater London	Dermatitis [1.46]	Asthma [1.72]	Neonatal [1.05]	Iron [6.32]	Anxiety [1.02]	Conduct [1.01]	Congenital [1.15]	Skin Viral [0.91]	URI [1.24]	Falls [0.85]
Barking & Dagenham	Dermatitis [1.87]	Iron [4.98]	Neonatal [0.98]	Asthma [1.49]	Anxiety [1.09]	Conduct [0.97]	Congenital [1.05]	Skin Viral [0.86]	URI [1.31]	Falls [1.14]
Barnet	Dermatitis [1.43]	Asthma [1.63]	Neonatal [0.97]	Iron [5.0]	Anxiety [1.04]	Conduct [0.99]	Congenital [1.08]	Skin Viral [0.89]	URI [1.27]	Falls [0.92]
Bexley	Dermatitis [1.43]	Asthma [1.54]	Neonatal [0.92]	Iron [4.25]	Anxiety [1.07]	Conduct [0.97]	Congenital [1.06]	Skin Viral [0.87]	URI [1.3]	Falls [1.05]
Brent	Dermatitis [1.43]	Neonatal [1.03]	Asthma [1.6]	Iron [5.4]	Anxiety [1.04]	Conduct [0.99]	Congenital [1.06]	Skin Viral [0.88]	URI [1.26]	Falls [0.95]
Bromley	Dermatitis [1.44]	Neonatal [1.1]	Asthma [1.59]	Anxiety [1.05]	Conduct [0.98]	Congenital [1.1]	Skin Viral [0.89]	URI [1.29]	Falls [0.97]	Iron [2.14]
Camden	Dermatitis [1.46]	Asthma [1.85]	Neonatal [0.92]	Anxiety [1.0]	Iron [6.63]	Conduct [1.04]	Congenital [1.19]	Skin Viral [0.94]	URI [1.23]	Falls [0.78]
Croydon	Dermatitis [1.44]	Asthma [1.56]	Neonatal [0.98]	Iron [4.66]	Anxiety [1.06]	Conduct [0.97]	Congenital [1.08]	Skin Viral [0.88]	URI [1.29]	Falls [1.01]
Ealing	Dermatitis [1.43]	Neonatal [1.11]	Asthma [1.64]	Iron [6.63]	Anxiety [1.04]	Conduct [0.99]	Congenital [1.08]	Skin Viral [0.89]	URI [1.26]	Falls [0.92]
Enfield	Dermatitis [1.44]	Neonatal [1.03]	Asthma [1.57]	Iron [4.61]	Anxiety [1.06]	Conduct [0.98]	Congenital [1.08]	Skin Viral [0.88]	URI [1.28]	Falls [1.0]
Greenwich	Dermatitis [1.43]	Asthma [1.55]	Neonatal [0.95]	Iron [3.76]	Anxiety [1.06]	Conduct [0.98]	Congenital [1.09]	Skin Viral [0.87]	URI [1.28]	Falls [1.0]
Hackney	Dermatitis [1.45]	Asthma [1.76]	Neonatal [1.02]	Anxiety [1.03]	Iron [4.28]	Conduct [0.99]	Congenital [1.14]	Skin Viral [0.91]	URI [1.24]	Falls [0.87]
Hammersmith & Fulham	Dermatitis [1.46]	Asthma [1.83]	Neonatal [1.03]	Anxiety [1.01]	Conduct [1.03]	Congenital [1.18]	Skin Viral [0.94]	URI [1.23]	Falls [0.78]	Iron [3.1]
Haringey	Dermatitis [1.44]	Neonatal [1.08]	Asthma [1.6]	Iron [4.5]	Anxiety [1.05]	Conduct [0.98]	Congenital [1.11]	Skin Viral [0.89]	URI [1.25]	Falls [0.93]
Harrow	Dermatitis [1.44]	Neonatal [1.12]	Asthma [1.58]	Iron [5.13]	Anxiety [1.05]	Conduct [0.98]	Congenital [1.1]	Skin Viral [0.89]	URI [1.28]	Falls [0.96]

eFigure 9d. Leading ten causes of YLDs with the ratio of observed YLDs to YLDs expected on the basis of Socio-Demographic Index alone in 2017, 5-9 years, both sexes combined.
The top ten causes contributing to YLDs are listed globally, by socio-demographic quintile, and then by GBD superregion, region, country, and subnationally where modeled. For each cell, the ratio of observed YLDs to YLDs expected on the basis of socio-demographic index (SDI) alone are listed. Abbreviations: YLD=year of life lived with disability, GBD=Global Burden of Disease.

Values shown in brackets represent the ratio of observed YLDs to predicted YLDs on the basis of Socio-Demographic Index (SDI), rounded to two (2) digits. Color ranges (shown below) were calculated to place a roughly equal number of cells into each bin.

COLOR KEY:	[0.0-0.8]	(0.8-0.89]	(0.89-0.97]	(0.97-1.02]	(1.02-1.1]	(1.1-1.24]	(1.24-1.42]	(1.42-1.91]	1.91+

	1	2	3	4	5	6	7	8	9	10
Havering	Dermatitis [1.44]	Asthma [1.54]	Neonatal [0.97]	Iron [3.14]	Anxiety [1.07]	Conduct [0.97]	Congenital [1.07]	Skin Viral [0.87]	URI [1.3]	Falls [1.06]
Hillingdon	Dermatitis [1.44]	Neonatal [1.15]	Asthma [1.71]	Iron [5.48]	Anxiety [1.03]	Conduct [1.0]	Congenital [1.12]	Skin Viral [0.9]	URI [1.24]	Falls [0.88]
Hounslow	Dermatitis [1.44]	Neonatal [1.09]	Asthma [1.68]	Iron [5.0]	Anxiety [1.03]	Conduct [1.0]	Congenital [1.11]	Skin Viral [0.9]	URI [1.24]	Falls [0.89]
Islington	Dermatitis [1.45]	Asthma [1.83]	Neonatal [0.89]	Anxiety [1.01]	Conduct [1.03]	Congenital [1.19]	Skin Viral [0.94]	URI [1.22]	Iron [4.37]	Falls [0.8]
Kensington & Chelsea	Dermatitis [1.45]	Asthma [1.85]	Neonatal [0.89]	Iron [8.37]	Anxiety [0.99]	Conduct [1.05]	Congenital [1.18]	Skin Viral [0.94]	URI [1.23]	Falls [0.78]
Kingston upon Thames	Dermatitis [1.44]	Neonatal [1.09]	Asthma [1.71]	Anxiety [1.02]	Conduct [1.0]	Congenital [1.16]	Iron [4.1]	Skin Viral [0.91]	URI [1.26]	Falls [0.86]
Lambeth	Dermatitis [1.44]	Asthma [1.73]	Iron [7.47]	Neonatal [0.75]	Anxiety [1.02]	Conduct [1.01]	Congenital [1.17]	Skin Viral [0.92]	URI [1.23]	Falls [0.83]
Lewisham	Dermatitis [1.43]	Neonatal [1.18]	Asthma [1.58]	Iron [3.96]	Anxiety [1.06]	Conduct [0.98]	Congenital [1.1]	Skin Viral [0.88]	URI [1.28]	Falls [0.97]
Merton	Dermatitis [1.43]	Neonatal [1.04]	Asthma [1.67]	Iron [5.71]	Anxiety [1.03]	Conduct [0.99]	Congenital [1.12]	Skin Viral [0.9]	URI [1.26]	Falls [0.89]
Newham	Dermatitis [1.44]	Neonatal [1.06]	Asthma [1.58]	Anxiety [1.06]	Iron [3.29]	Conduct [0.98]	Congenital [1.08]	Skin Viral [0.88]	URI [1.27]	Falls [0.98]
Redbridge	Dermatitis [1.44]	Asthma [1.55]	Neonatal [0.98]	Iron [4.4]	Anxiety [1.06]	Congenital [1.15]	Conduct [0.98]	Skin Viral [0.87]	URI [1.29]	Falls [1.03]
Richmond upon Thames	Dermatitis [1.44]	Neonatal [1.15]	Asthma [1.73]	Iron [6.01]	Anxiety [1.02]	Conduct [1.01]	Congenital [1.17]	Skin Viral [0.91]	URI [1.25]	Falls [0.84]
Southwark	Dermatitis [1.45]	Asthma [1.79]	Neonatal [0.77]	Anxiety [1.01]	Iron [5.39]	Conduct [1.02]	Congenital [1.19]	Skin Viral [0.92]	URI [1.23]	Falls [0.83]
Sutton	Dermatitis [1.43]	Neonatal [1.25]	Asthma [1.59]	Iron [4.31]	Anxiety [1.05]	Conduct [0.98]	Congenital [1.09]	Skin Viral [0.88]	URI [1.28]	Falls [0.99]
Tower Hamlets	Dermatitis [1.45]	Neonatal [1.13]	Asthma [1.79]	Anxiety [1.01]	Iron [5.16]	Conduct [1.01]	Congenital [1.17]	Skin Viral [0.92]	URI [1.23]	Falls [0.83]
Waltham Forest	Dermatitis [1.44]	Neonatal [0.99]	Asthma [1.51]	Iron [4.15]	Anxiety [1.07]	Conduct [0.97]	Congenital [1.08]	Skin Viral [0.87]	URI [1.28]	Falls [1.05]
Wandsworth	Dermatitis [1.45]	Asthma [1.75]	Neonatal [0.92]	Anxiety [1.01]	Iron [5.41]	Conduct [1.02]	Congenital [1.19]	Skin Viral [0.93]	URI [1.24]	Falls [0.81]
Westminster	Dermatitis [1.46]	Asthma [1.87]	Neonatal [0.83]	Iron [6.92]	Anxiety [1.0]	Conduct [1.04]	Congenital [1.18]	Skin Viral [0.94]	URI [1.22]	Falls [0.79]
NE England	Dermatitis [1.55]	Iron [4.59]	Neonatal [0.88]	Asthma [1.3]	Congenital [1.35]	Anxiety [1.07]	Conduct [0.97]	Skin Viral [0.87]	URI [1.3]	Falls [1.06]
County Durham	Dermatitis [1.72]	Iron [4.73]	Neonatal [0.95]	Asthma [1.3]	Congenital [1.34]	Anxiety [1.09]	Conduct [0.96]	Skin Viral [0.87]	URI [1.31]	Falls [1.1]
Darlington	Dermatitis [1.43]	Iron [5.15]	Neonatal [0.86]	Asthma [1.33]	Congenital [1.36]	Anxiety [1.06]	Conduct [0.98]	Skin Viral [0.87]	URI [1.3]	Falls [1.04]
Gateshead	Dermatitis [1.69]	Iron [5.85]	Neonatal [0.98]	Asthma [1.32]	Congenital [1.36]	Anxiety [1.07]	Conduct [0.96]	Skin Viral [0.87]	URI [1.29]	Falls [1.05]
Hartlepool	Dermatitis [1.45]	Neonatal [1.0]	Asthma [1.29]	Iron [2.77]	Congenital [1.33]	Anxiety [1.1]	Conduct [0.97]	Skin Viral [0.86]	URI [1.32]	Falls [1.14]
Middlesbrough	Dermatitis [1.44]	Asthma [1.31]	Neonatal [0.77]	Iron [3.06]	Congenital [1.35]	Anxiety [1.09]	Conduct [0.97]	Skin Viral [0.87]	URI [1.3]	Falls [1.09]
Newcastle upon Tyne	Dermatitis [1.43]	Asthma [1.43]	Neonatal [0.84]	Iron [5.02]	Congenital [1.42]	Anxiety [1.03]	Conduct [1.0]	Skin Viral [0.89]	URI [1.26]	Falls [0.91]
N Tyneside	Dermatitis [1.43]	Iron [4.64]	Asthma [1.32]	Neonatal [0.82]	Congenital [1.36]	Anxiety [1.07]	Conduct [0.98]	Skin Viral [0.87]	URI [1.29]	Falls [1.06]
Northumberland	Dermatitis [1.44]	Neonatal [0.85]	Asthma [1.3]	Iron [3.64]	Congenital [1.36]	Anxiety [1.08]	Conduct [0.98]	Skin Viral [0.86]	URI [1.31]	Falls [1.11]
Redcar & Cleveland	Dermatitis [1.45]	Iron [3.71]	Asthma [1.28]	Neonatal [0.8]	Congenital [1.33]	Anxiety [1.1]	Conduct [0.98]	Skin Viral [0.86]	URI [1.33]	Falls [1.18]
S Tyneside	Dermatitis [1.73]	Iron [3.29]	Neonatal [0.84]	Asthma [1.27]	Congenital [1.33]	Anxiety [1.1]	Conduct [0.96]	Skin Viral [0.87]	URI [1.33]	Falls [1.16]
Stockton-on-Tees	Dermatitis [1.44]	Neonatal [1.07]	Iron [4.7]	Asthma [1.34]	Congenital [1.35]	Anxiety [1.08]	Conduct [0.97]	Skin Viral [0.87]	URI [1.29]	Falls [1.05]
Sunderland	Dermatitis [1.71]	Iron [4.99]	Neonatal [0.75]	Asthma [1.15]	Congenital [1.35]	Anxiety [1.08]	Conduct [0.97]	Skin Viral [0.87]	URI [1.3]	Falls [1.08]

eFigure 9d. Leading ten causes of YLDs with the ratio of observed YLDs to YLDs expected on the basis of Socio-Demographic Index alone in 2017, 5-9 years, both sexes combined.
The top ten causes contributing to YLDs are listed globally, by socio-demographic quintile, and then by GBD superregion, region, country, and subnationally where modeled. For each cell, the ratio of observed YLDs to YLDs expected on the basis of socio-demographic index (SDI) alone are listed. Abbreviations: YLD=year of life lived with disability, GBD=Global Burden of Disease.

Values shown in brackets represent the ratio of observed YLDs to predicted YLDs on the basis of Socio-Demographic Index (SDI), rounded to two (2) digits. Color ranges (shown below) were calculated to place a roughly equal number of cells into each bin.

COLOR KEY:	[0.0-0.8]	(0.8-0.89]	(0.89-0.97]	(0.97-1.02]	(1.02-1.1]	(1.1-1.24]	(1.24-1.42]	(1.42-1.91]	1.91+

	1	2	3	4	5	6	7	8	9	10
NW England	Dermatitis [1.44]	Neonatal [1.09]	Asthma [1.34]	Iron [3.3]	Anxiety [1.06]	Congenital [1.29]	Conduct [0.98]	Skin Viral [0.88]	URI [1.28]	Falls [1.02]
Blackburn with Darwen	Dermatitis [1.45]	Neonatal [1.07]	Asthma [1.29]	Iron [2.41]	Anxiety [1.1]	Congenital [1.21]	Conduct [0.97]	Skin Viral [0.87]	URI [1.3]	Falls [1.13]
Blackpool	Dermatitis [1.46]	Neonatal [1.03]	Asthma [1.28]	Iron [2.14]	Anxiety [1.12]	Congenital [1.21]	Conduct [0.97]	Skin Viral [0.87]	URI [1.33]	Falls [1.24]
Bolton	Dermatitis [1.44]	Neonatal [1.02]	Asthma [1.28]	Iron [2.58]	Anxiety [1.09]	Congenital [1.22]	Conduct [0.97]	Skin Viral [0.87]	URI [1.3]	Falls [1.11]
Bury	Dermatitis [1.44]	Neonatal [0.98]	Asthma [1.29]	Iron [3.34]	Anxiety [1.07]	Congenital [1.24]	Conduct [0.98]	Skin Viral [0.87]	URI [1.29]	Falls [1.08]
Cheshire E	Dermatitis [1.43]	Neonatal [1.35]	Asthma [1.41]	Congenital [1.42]	Anxiety [1.03]	Conduct [1.0]	Skin Viral [0.89]	URI [1.27]	Iron [3.15]	Falls [0.93]
Cheshire W & Chester	Dermatitis [1.44]	Neonatal [1.11]	Asthma [1.37]	Congenital [1.41]	Anxiety [1.05]	Iron [3.29]	Conduct [0.99]	Skin Viral [0.89]	URI [1.27]	Falls [0.96]
Cumbria	Dermatitis [1.43]	Neonatal [1.08]	Asthma [1.32]	Iron [3.23]	Anxiety [1.07]	Congenital [1.24]	Conduct [0.97]	Skin Viral [0.87]	URI [1.3]	Falls [1.04]
Halton	Dermatitis [1.44]	Neonatal [1.11]	Asthma [1.33]	Iron [3.56]	Anxiety [1.08]	Congenital [1.23]	Conduct [0.97]	Skin Viral [0.87]	URI [1.28]	Falls [1.04]
Knowsley	Dermatitis [1.44]	Neonatal [1.05]	Asthma [1.31]	Iron [3.6]	Congenital [1.35]	Anxiety [1.08]	Conduct [0.97]	Skin Viral [0.87]	URI [1.29]	Falls [1.08]
Lancashire	Dermatitis [1.43]	Neonatal [1.05]	Asthma [1.32]	Iron [3.21]	Anxiety [1.07]	Congenital [1.24]	Conduct [0.98]	Skin Viral [0.87]	URI [1.29]	Falls [1.04]
Liverpool	Dermatitis [1.44]	Neonatal [1.19]	Asthma [1.36]	Congenital [1.42]	Anxiety [0.97]	Conduct [0.98]	Skin Viral [0.88]	URI [1.27]	Iron [2.39]	Falls [0.98]
Manchester	Dermatitis [1.44]	Neonatal [1.2]	Asthma [1.47]	Anxiety [1.02]	Congenital [1.32]	Conduct [1.0]	Skin Viral [0.91]	URI [1.24]	Iron [3.07]	Falls [0.88]
Oldham	Dermatitis [1.45]	Asthma [1.31]	Neonatal [0.8]	Iron [2.06]	Anxiety [1.12]	Congenital [1.22]	Conduct [0.96]	Skin Viral [0.87]	URI [1.32]	Falls [1.18]
Rochdale	Dermatitis [1.44]	Iron [3.26]	Asthma [1.29]	Neonatal [0.83]	Anxiety [1.1]	Congenital [1.21]	Conduct [0.97]	Skin Viral [0.86]	URI [1.32]	Falls [1.15]
Salford	Dermatitis [1.44]	Neonatal [1.22]	Asthma [1.36]	Anxiety [1.06]	Congenital [1.26]	Conduct [0.98]	Skin Viral [0.88]	URI [1.27]	Iron [2.18]	Falls [1.0]
Sefton	Dermatitis [1.44]	Neonatal [1.08]	Asthma [1.29]	Iron [3.1]	Congenital [1.36]	Anxiety [1.08]	Conduct [0.97]	Skin Viral [0.86]	URI [1.31]	Falls [1.12]
St Helens	Dermatitis [1.44]	Neonatal [1.09]	Asthma [1.28]	Iron [2.86]	Congenital [1.34]	Anxiety [1.09]	Conduct [0.96]	Skin Viral [0.87]	URI [1.31]	Falls [1.12]
Stockport	Dermatitis [1.43]	Neonatal [0.92]	Asthma [1.34]	Iron [3.93]	Anxiety [1.05]	Congenital [1.28]	Conduct [0.99]	Skin Viral [0.88]	URI [1.29]	Falls [1.0]
Tameside	Dermatitis [1.44]	Neonatal [0.89]	Asthma [1.28]	Iron [2.32]	Anxiety [1.1]	Congenital [1.21]	Conduct [0.97]	Skin Viral [0.87]	URI [1.32]	Falls [1.15]
Trafford	Dermatitis [1.44]	Neonatal [1.21]	Asthma [1.42]	Iron [4.83]	Anxiety [1.03]	Congenital [1.29]	Conduct [1.0]	Skin Viral [0.9]	URI [1.25]	Falls [0.9]
Warrington	Dermatitis [1.43]	Neonatal [1.28]	Asthma [1.39]	Iron [5.78]	Anxiety [1.04]	Congenital [1.28]	Conduct [0.99]	Skin Viral [0.89]	URI [1.26]	Falls [0.94]
Wigan	Dermatitis [1.44]	Neonatal [1.08]	Asthma [1.28]	Iron [2.34]	Anxiety [1.09]	Congenital [1.22]	Conduct [0.97]	Skin Viral [0.86]	URI [1.33]	Falls [1.14]
Wirral	Dermatitis [1.44]	Neonatal [1.21]	Asthma [1.28]	Congenital [1.36]	Anxiety [1.09]	Iron [1.98]	Conduct [0.97]	Skin Viral [0.87]	URI [1.31]	Falls [1.11]
SE England	Dermatitis [1.57]	Neonatal [1.02]	Asthma [1.47]	Anxiety [1.05]	Iron [3.57]	Congenital [1.16]	Conduct [0.99]	Skin Viral [0.89]	URI [1.28]	Falls [0.96]
Bracknell Forest	Dermatitis [1.43]	Asthma [1.37]	Neonatal [0.8]	Anxiety [1.04]	Congenital [1.17]	Conduct [1.0]	Skin Viral [0.9]	URI [1.27]	Falls [0.92]	Iron [2.17]
Brighton & Hove	Dermatitis [1.7]	Neonatal [1.12]	Asthma [1.41]	Iron [5.15]	Anxiety [1.03]	Congenital [1.21]	Conduct [1.0]	Skin Viral [0.91]	URI [1.24]	Falls [0.88]
Buckinghamshire	Dermatitis [1.43]	Neonatal [1.15]	Asthma [1.34]	Anxiety [1.04]	Iron [3.68]	Congenital [1.17]	Conduct [0.99]	Skin Viral [0.89]	URI [1.27]	Epilepsy [1.74]
E Sussex	Dermatitis [1.67]	Neonatal [1.06]	Asthma [1.22]	Iron [2.83]	Anxiety [1.08]	Congenital [1.13]	Conduct [0.98]	Skin Viral [0.86]	URI [1.31]	Falls [1.08]
Hampshire	Dermatitis [1.43]	Asthma [2.08]	Neonatal [0.99]	Iron [3.84]	Anxiety [1.05]	Congenital [1.15]	Conduct [0.98]	Skin Viral [0.88]	URI [1.29]	Falls [0.98]
Isle of Wight	Dermatitis [1.41]	Asthma [1.98]	Neonatal [0.92]	Iron [3.33]	Anxiety [1.08]	Congenital [1.1]	Conduct [0.97]	Skin Viral [0.87]	URI [1.3]	Falls [1.08]

eFigure 9d. Leading ten causes of YLDs with the ratio of observed YLDs to YLDs expected on the basis of Socio-Demographic Index alone in 2017, 5-9 years, both sexes combined.
The top ten causes contributing to YLDs are listed globally, by socio-demographic quintile, and then by GBD superregion, region, country, and subnationally where modeled. For each cell, the ratio of observed YLDs to YLDs expected on the basis of socio-demographic index (SDI) alone are listed. Abbreviations: YLD=year of life lived with disability, GBD=Global Burden of Disease.

Values shown in brackets represent the ratio of observed YLDs to predicted YLDs on the basis of Socio-Demographic Index (SDI), rounded to two (2) digits. Color ranges (shown below) were calculated to place a roughly equal number of cells into each bin.

COLOR KEY:	[0.0-0.8]	(0.8-0.89]	(0.89-0.97]	(0.97-1.02]	(1.02-1.1]	(1.1-1.24]	(1.24-1.42]	(1.42-1.91]	1.91+

	1	2	3	4	5	6	7	8	9	10
Kent	Dermatitis [1.67]	Neonatal [0.98]	Asthma [1.26]	Anxiety [1.07]	Iron [2.67]	Congenital [1.13]	Conduct [0.98]	Skin Viral [0.87]	URI [1.3]	ASD [1.55]
Medway	Dermatitis [1.44]	Neonatal [1.03]	Asthma [1.25]	Iron [2.98]	Anxiety [1.09]	Congenital [1.1]	Conduct [0.96]	Skin Viral [0.87]	URI [1.31]	Falls [1.1]
Milton Keynes	Dermatitis [1.43]	Neonatal [0.88]	Asthma [1.37]	Anxiety [1.04]	Congenital [1.16]	Conduct [0.99]	Skin Viral [0.89]	URI [1.26]	Iron [2.87]	Falls [0.96]
Oxfordshire	Dermatitis [1.87]	Neonatal [0.94]	Asthma [1.39]	Iron [5.55]	Anxiety [1.03]	Congenital [1.18]	Conduct [0.99]	Skin Viral [0.9]	URI [1.26]	Falls [0.89]
Portsmouth	Dermatitis [1.44]	Neonatal [0.97]	Asthma [1.34]	Iron [4.95]	Anxiety [1.05]	Conduct [0.99]	Congenital [1.13]	Skin Viral [0.89]	URI [1.26]	Falls [0.95]
Reading	Dermatitis [1.87]	Neonatal [1.22]	Asthma [1.46]	Anxiety [1.02]	Congenital [1.21]	Conduct [1.01]	Skin Viral [0.91]	URI [1.23]	Iron [3.78]	Falls [0.85]
Slough	Dermatitis [1.43]	Neonatal [1.06]	Asthma [1.4]	Anxiety [1.05]	Congenital [1.15]	Conduct [0.99]	Skin Viral [0.89]	URI [1.26]	Iron [2.41]	Falls [0.94]
Southampton	Dermatitis [1.43]	Neonatal [0.96]	Asthma [1.34]	Iron [4.07]	Anxiety [1.04]	Congenital [1.15]	Conduct [0.99]	Skin Viral [0.89]	URI [1.27]	Falls [0.94]
Surrey	Dermatitis [1.66]	Neonatal [1.11]	Asthma [1.4]	Anxiety [1.02]	Congenital [1.2]	Conduct [1.0]	Skin Viral [0.91]	URI [1.26]	Falls [0.88]	Iron [2.81]
West Berkshire	Dermatitis [1.43]	Neonatal [0.93]	Asthma [1.38]	Anxiety [1.04]	Congenital [1.17]	Iron [3.72]	Conduct [1.0]	Skin Viral [0.89]	URI [1.26]	Falls [0.93]
W Sussex	Dermatitis [1.48]	Neonatal [1.02]	Asthma [1.52]	Iron [4.18]	Anxiety [1.05]	Congenital [1.15]	Conduct [0.99]	Skin Viral [0.88]	URI [1.29]	Falls [0.99]
Windsor & Maidenhead	Dermatitis [1.44]	Neonatal [0.97]	Asthma [1.43]	Anxiety [1.02]	Congenital [1.21]	Conduct [1.02]	Skin Viral [0.9]	URI [1.25]	Iron [3.45]	Falls [0.87]
Wokingham	Dermatitis [1.44]	Neonatal [1.0]	Asthma [1.4]	Anxiety [1.02]	Iron [4.25]	Congenital [1.21]	Conduct [1.01]	Skin Viral [0.91]	URI [1.27]	Falls [0.89]
SW England	Dermatitis [1.43]	Neonatal [1.05]	Iron [5.7]	Asthma [1.3]	Anxiety [1.06]	Conduct [0.98]	Skin Viral [0.88]	Congenital [1.03]	URI [1.29]	Falls [1.0]
Bath & NE Somerset	Dermatitis [1.43]	Neonatal [1.41]	Iron [8.02]	Asthma [1.37]	Anxiety [1.03]	Conduct [1.0]	Skin Viral [0.89]	Congenital [1.06]	URI [1.26]	Falls [0.9]
Bournemouth	Dermatitis [1.44]	Iron [7.82]	Neonatal [1.04]	Asthma [1.34]	Anxiety [1.05]	Conduct [0.98]	Skin Viral [0.89]	Congenital [1.05]	URI [1.27]	Falls [0.94]
Bristol	Dermatitis [1.44]	Neonatal [1.22]	Iron [8.25]	Asthma [1.42]	Anxiety [1.03]	Conduct [1.0]	Skin Viral [0.9]	Congenital [1.08]	URI [1.25]	Falls [0.87]
Cornwall	Dermatitis [1.44]	Iron [6.67]	Neonatal [1.03]	Asthma [1.26]	Anxiety [1.08]	Conduct [0.97]	Congenital [1.02]	Skin Viral [0.87]	URI [1.3]	Falls [1.09]
Devon	Dermatitis [1.43]	Neonatal [0.95]	Asthma [1.28]	Iron [4.2]	Anxiety [1.06]	Conduct [0.98]	Skin Viral [0.87]	Congenital [1.03]	URI [1.29]	Falls [1.03]
Dorset	Dermatitis [1.44]	Iron [5.78]	Neonatal [1.01]	Asthma [1.28]	Anxiety [1.07]	Conduct [0.97]	Skin Viral [0.87]	Congenital [1.02]	URI [1.29]	Falls [1.05]
Gloucestershire	Dermatitis [1.43]	Neonatal [0.97]	Asthma [1.33]	Iron [3.94]	Anxiety [1.06]	Conduct [0.98]	Skin Viral [0.88]	Congenital [1.04]	URI [1.28]	Falls [0.97]
N Somerset	Dermatitis [1.43]	Neonatal [1.13]	Iron [6.29]	Asthma [1.29]	Anxiety [1.06]	Conduct [0.98]	Congenital [1.03]	Skin Viral [0.87]	URI [1.29]	Falls [1.02]
Plymouth	Dermatitis [1.44]	Neonatal [1.04]	Asthma [1.29]	Iron [4.17]	Anxiety [1.06]	Conduct [0.97]	Skin Viral [0.88]	Congenital [1.02]	URI [1.28]	Falls [1.01]
Poole	Dermatitis [1.43]	Neonatal [1.03]	Iron [5.41]	Asthma [1.31]	Anxiety [1.05]	Conduct [0.99]	Skin Viral [0.87]	Congenital [1.03]	URI [1.29]	Falls [1.0]
Somerset	Dermatitis [1.44]	Neonatal [1.09]	Asthma [1.26]	Anxiety [1.09]	Iron [2.52]	Conduct [0.97]	Skin Viral [0.87]	Congenital [1.02]	URI [1.31]	Falls [1.09]
S Gloucestershire	Dermatitis [1.44]	Neonatal [1.1]	Asthma [1.36]	Iron [5.57]	Anxiety [1.04]	Conduct [0.99]	Skin Viral [0.89]	Congenital [1.05]	URI [1.27]	Falls [0.92]
Swindon	Dermatitis [1.43]	Iron [8.33]	Asthma [1.34]	Neonatal [0.75]	Anxiety [1.05]	Conduct [0.98]	Skin Viral [0.88]	Congenital [1.02]	URI [1.27]	Falls [0.98]
Torbay	Dermatitis [1.45]	Neonatal [0.85]	Iron [2.96]	Asthma [1.24]	Anxiety [1.11]	Conduct [0.97]	Skin Viral [0.87]	Congenital [1.0]	URI [1.34]	Falls [1.19]
Wiltshire	Dermatitis [1.43]	Iron [7.49]	Neonatal [1.2]	Asthma [1.28]	Anxiety [1.07]	Conduct [0.97]	Skin Viral [0.87]	Congenital [1.02]	URI [1.29]	Falls [1.03]
W Midlands	Dermatitis [1.44]	Neonatal [0.98]	Asthma [1.3]	Iron [3.62]	Anxiety [1.07]	Conduct [0.98]	Congenital [1.1]	Skin Viral [0.87]	URI [1.29]	Falls [1.06]
Birmingham	Dermatitis [1.44]	Neonatal [0.96]	Asthma [1.32]	Iron [3.55]	Anxiety [1.06]	Conduct [0.98]	Congenital [1.12]	Skin Viral [0.88]	URI [1.28]	Falls [1.04]

eFigure 9d. Leading ten causes of YLDs with the ratio of observed YLDs to YLDs expected on the basis of Socio-Demographic Index alone in 2017, 5-9 years, both sexes combined.
The top ten causes contributing to YLDs are listed globally, by socio-demographic quintile, and then by GBD superregion, region, country, and subnationally where modeled. For each cell, the ratio of observed YLDs to YLDs expected on the basis of socio-demographic index (SDI) alone are listed. Abbreviations: YLD=year of life lived with disability, GBD=Global Burden of Disease.

Values shown in brackets represent the ratio of observed YLDs to predicted YLDs on the basis of Socio-Demographic Index (SDI), rounded to two (2) digits. Color ranges (shown below) were calculated to place a roughly equal number of cells into each bin.

COLOR KEY:	[0.0-0.8]	(0.8-0.89]	(0.89-0.97]	(0.97-1.02]	(1.02-1.1]	(1.1-1.24]	(1.24-1.42]	(1.42-1.91]	1.91+

	1	2	3	4	5	6	7	8	9	10
Coventry	Dermatitis [1.44]	Neonatal [0.93]	Asthma [1.35]	Anxiety [1.05]	Iron [3.23]	Conduct [0.98]	Congenital [1.12]	Skin Viral [0.88]	URI [1.26]	Falls [0.97]
Dudley	Dermatitis [1.45]	Neonatal [1.07]	Asthma [1.27]	Iron [2.44]	Anxiety [1.1]	Congenital [1.08]	Conduct [0.96]	Skin Viral [0.87]	URI [1.31]	Falls [1.15]
Herefordshire	Dermatitis [1.43]	Neonatal [1.01]	Asthma [1.3]	Iron [3.93]	Anxiety [1.07]	Conduct [0.98]	Congenital [1.1]	Skin Viral [0.87]	URI [1.29]	Falls [1.03]
Sandwell	Dermatitis [1.45]	Neonatal [1.06]	Asthma [1.28]	Iron [2.69]	Anxiety [1.1]	Conduct [0.97]	Congenital [1.07]	Skin Viral [0.86]	URI [1.31]	Falls [1.18]
Shropshire	Dermatitis [1.44]	Neonatal [0.98]	Asthma [1.29]	Anxiety [1.07]	Iron [2.82]	Conduct [0.97]	Congenital [1.1]	Skin Viral [0.87]	URI [1.29]	Falls [1.03]
Solihull	Dermatitis [1.43]	Asthma [1.35]	Neonatal [0.72]	Iron [3.96]	Anxiety [1.05]	Conduct [0.99]	Congenital [1.12]	Skin Viral [0.89]	URI [1.27]	Falls [0.98]
Staffordshire	Dermatitis [1.44]	Neonatal [0.95]	Iron [4.87]	Asthma [1.28]	Anxiety [1.07]	Conduct [0.98]	Congenital [1.1]	Skin Viral [0.87]	URI [1.3]	Falls [1.09]
Stoke-on-Trent	Dermatitis [1.45]	Neonatal [1.17]	Asthma [1.26]	Iron [2.63]	Anxiety [1.09]	Conduct [0.96]	Congenital [1.08]	Skin Viral [0.87]	URI [1.3]	Falls [1.18]
Telford & Wrekin	Dermatitis [1.44]	Neonatal [0.99]	Asthma [1.29]	Anxiety [1.08]	Iron [2.59]	Conduct [0.96]	Congenital [1.08]	Skin Viral [0.87]	URI [1.29]	Falls [1.07]
Walsall	Dermatitis [1.45]	Neonatal [0.98]	Asthma [1.27]	Iron [2.26]	Anxiety [1.1]	Conduct [0.97]	Congenital [1.07]	Skin Viral [0.86]	URI [1.32]	Falls [1.2]
Warwickshire	Dermatitis [1.44]	Neonatal [1.0]	Asthma [1.35]	Iron [4.97]	Anxiety [1.05]	Conduct [0.99]	Congenital [1.13]	Skin Viral [0.89]	URI [1.28]	Falls [0.97]
Wolverhampton	Dermatitis [1.44]	Asthma [1.27]	Neonatal [0.72]	Iron [2.44]	Anxiety [1.09]	Conduct [0.97]	Congenital [1.08]	Skin Viral [0.87]	URI [1.3]	Falls [1.11]
Worcestershire	Dermatitis [1.44]	Neonatal [1.1]	Asthma [1.3]	Iron [3.6]	Anxiety [1.07]	Conduct [0.98]	Congenital [1.11]	Skin Viral [0.88]	URI [1.29]	Falls [1.04]
Yorkshire & Humber	Dermatitis [1.44]	Neonatal [0.92]	Asthma [1.33]	Iron [3.62]	Anxiety [1.07]	Conduct [0.97]	Congenital [1.1]	Skin Viral [0.87]	URI [1.29]	Falls [1.03]
Barnsley	Dermatitis [1.45]	Neonatal [0.97]	Asthma [1.28]	Iron [2.31]	Anxiety [1.12]	Conduct [0.97]	Congenital [1.06]	Skin Viral [0.87]	URI [1.34]	Falls [1.19]
Bradford	Dermatitis [1.44]	Neonatal [1.02]	Asthma [1.29]	Iron [2.89]	Anxiety [1.1]	Conduct [0.97]	Congenital [1.09]	Skin Viral [0.87]	URI [1.3]	Falls [1.11]
Calderdale	Dermatitis [1.44]	Neonatal [0.89]	Asthma [1.32]	Anxiety [1.08]	Conduct [0.96]	Congenital [1.09]	Skin Viral [0.88]	URI [1.29]	Iron [2.18]	Falls [1.04]
Doncaster	Dermatitis [1.45]	Neonatal [1.19]	Asthma [1.29]	Anxiety [1.11]	Iron [1.8]	Congenital [1.08]	Conduct [0.96]	Skin Viral [0.87]	URI [1.33]	Falls [1.18]
E Riding of Yorkshire	Dermatitis [1.44]	Neonatal [0.91]	Asthma [1.3]	Iron [3.21]	Anxiety [1.08]	Conduct [0.98]	Congenital [1.1]	Skin Viral [0.87]	URI [1.3]	Falls [1.07]
Kingston upon Hull	Dermatitis [1.44]	Asthma [1.33]	Neonatal [0.84]	Anxiety [1.08]	Conduct [0.97]	Congenital [1.09]	Iron [2.16]	Skin Viral [0.87]	URI [1.3]	Falls [1.08]
Kirklees	Dermatitis [1.44]	Neonatal [0.86]	Asthma [1.3]	Iron [3.18]	Anxiety [1.08]	Congenital [1.1]	Conduct [0.97]	Skin Viral [0.87]	URI [1.29]	Falls [1.08]
Leeds	Dermatitis [1.43]	Asthma [1.41]	Neonatal [0.84]	Iron [5.7]	Anxiety [1.03]	Congenital [1.15]	Conduct [0.99]	Skin Viral [0.9]	URI [1.26]	Falls [0.91]
NE Lincolnshire	Dermatitis [1.44]	Neonatal [0.88]	Asthma [1.31]	Iron [3.3]	Anxiety [1.1]	Conduct [0.97]	Congenital [1.08]	Skin Viral [0.87]	URI [1.31]	Falls [1.11]
N Lincolnshire	Dermatitis [1.44]	Asthma [1.31]	Neonatal [0.82]	Iron [3.44]	Anxiety [1.09]	Conduct [0.97]	Congenital [1.08]	Skin Viral [0.87]	URI [1.3]	Falls [1.09]
N Yorkshire	Dermatitis [1.43]	Neonatal [1.05]	Asthma [1.32]	Iron [3.75]	Anxiety [1.06]	Conduct [0.98]	Congenital [1.11]	Skin Viral [0.88]	URI [1.28]	Falls [1.0]
Rotherham	Dermatitis [1.44]	Asthma [1.29]	Neonatal [0.83]	Iron [2.29]	Anxiety [1.1]	Conduct [0.97]	Congenital [1.08]	Skin Viral [0.86]	URI [1.32]	Falls [1.15]
Sheffield	Dermatitis [1.44]	Neonatal [0.97]	Asthma [1.36]	Iron [4.14]	Anxiety [1.05]	Conduct [0.98]	Congenital [1.13]	Skin Viral [0.89]	URI [1.28]	Falls [0.95]
Wakefield	Dermatitis [1.44]	Asthma [1.31]	Iron [3.62]	Neonatal [0.73]	Anxiety [1.09]	Conduct [0.97]	Congenital [1.08]	Skin Viral [0.86]	URI [1.31]	Falls [1.12]
York	Dermatitis [1.44]	Asthma [1.43]	Neonatal [0.86]	Anxiety [1.03]	Conduct [0.99]	Congenital [1.16]	Iron [3.89]	Skin Viral [0.9]	URI [1.26]	Falls [0.89]
N Ireland	Dermatitis [1.19]	Anxiety [1.71]	Neonatal [0.84]	Asthma [1.13]	Skin Viral [0.94]	Congenital [1.02]	Conduct [0.88]	URI [1.27]	Falls [0.98]	Headaches [0.97]
Scotland	Dermatitis [1.29]	Iron [3.4]	Asthma [1.22]	Neonatal [0.79]	Anxiety [1.17]	Congenital [1.19]	Skin Viral [0.93]	Conduct [0.95]	Vit A [1.78]	URI [1.3]

eFigure 9d. Leading ten causes of YLDs with the ratio of observed YLDs to YLDs expected on the basis of Socio-Demographic Index alone in 2017, 5-9 years, both sexes combined.
The top ten causes contributing to YLDs are listed globally, by socio-demographic quintile, and then by GBD superregion, region, country, and subnationally where modeled. For each cell, the ratio of observed YLDs to YLDs expected on the basis of socio-demographic index (SDI) alone are listed. Abbreviations: YLD=year of life lived with disability, GBD=Global Burden of Disease.

Values shown in brackets represent the ratio of observed YLDs to predicted YLDs on the basis of Socio-Demographic Index (SDI), rounded to two (2) digits. Color ranges (shown below) were calculated to place a roughly equal number of cells into each bin.

COLOR KEY:	[0.0-0.8]	(0.8-0.89]	(0.89-0.97]	(0.97-1.02]	(1.02-1.1]	(1.1-1.24]	(1.24-1.42]	(1.42-1.91]	1.91+

	1	2	3	4	5	6	7	8	9	10
Wales	Dermatitis [1.2]	Neonatal [0.81]	Iron [3.27]	Asthma [1.16]	Anxiety [1.19]	Skin Viral [1.01]	Conduct [0.95]	Congenital [1.03]	URI [1.31]	Falls [1.22]
**Latin America and Caribbean**	Neonatal [1.15]	Asthma [1.34]	Dermatitis [0.9]	Vit A [0.94]	Iron [0.72]	Diarrhea [1.37]	Epilepsy [1.23]	Skin Viral [0.82]	Headaches [1.36]	Conduct [0.79]
**Andean Latin America**	Asthma [1.71]	Dermatitis [1.48]	Iron [1.31]	Neonatal [1.09]	Vit A [0.77]	Headaches [1.46]	Diarrhea [1.18]	Skin Viral [0.83]	Conduct [0.78]	Epilepsy [1.08]
Bolivia	Iron [1.64]	Asthma [1.94]	Dermatitis [1.58]	Neonatal [1.07]	Vit A [0.63]	Diarrhea [1.38]	Headaches [1.53]	Skin Viral [0.86]	Conduct [0.8]	Anxiety [0.94]
Ecuador	Asthma [1.57]	Neonatal [1.12]	Dermatitis [1.3]	Headaches [1.73]	Diarrhea [1.46]	Epilepsy [1.3]	Skin Viral [0.83]	Conduct [0.78]	Anxiety [0.88]	Congenital [0.71]
Peru	Iron [1.63]	Dermatitis [1.54]	Asthma [1.7]	Neonatal [1.1]	Vit A [1.0]	Skin Viral [0.82]	Headaches [1.31]	Conduct [0.78]	Anxiety [0.87]	Epilepsy [1.03]
**Caribbean**	Iron [1.84]	Asthma [1.79]	Dermatitis [1.15]	Neonatal [0.93]	Vit A [1.02]	Skin Viral [0.82]	Diarrhea [1.19]	Anxiety [0.96]	Headaches [1.3]	Conduct [0.7]
Antigua	Iron [4.36]	Asthma [2.02]	Dermatitis [1.08]	Neonatal [0.96]	Vit A [0.97]	Skin Viral [0.8]	Anxiety [0.88]	Headaches [1.3]	Diarrhea [1.27]	Conduct [0.68]
Bahamas	Asthma [2.15]	Dermatitis [1.07]	Neonatal [0.96]	Iron [1.35]	Skin Viral [0.8]	Anxiety [0.85]	Headaches [1.32]	Diarrhea [1.48]	Conduct [0.68]	Epilepsy [1.21]
Barbados	Asthma [1.88]	Neonatal [1.04]	Dermatitis [1.05]	Iron [1.04]	Skin Viral [0.8]	Anxiety [0.86]	Headaches [1.31]	Vit A [0.7]	Conduct [0.69]	Diarrhea [1.28]
Belize	Iron [1.58]	Asthma [1.96]	Neonatal [1.02]	Dermatitis [1.21]	Vit A [0.48]	Skin Viral [0.85]	Anxiety [1.03]	Headaches [1.33]	Conduct [0.71]	Diarrhea [0.89]
Bermuda	Asthma [2.58]	Dermatitis [1.03]	Neonatal [0.98]	Skin Viral [0.79]	Anxiety [0.79]	Iron [1.57]	Headaches [1.33]	Diarrhea [1.76]	Conduct [0.68]	Vit A [1.26]
Cuba	Asthma [2.06]	Dermatitis [1.11]	Neonatal [0.92]	Iron [1.31]	Skin Viral [0.81]	Anxiety [0.91]	Headaches [1.3]	Conduct [0.69]	Congenital [0.67]	Urticaria [1.01]
Dominica	Iron [2.17]	Asthma [1.89]	Neonatal [1.1]	Dermatitis [1.11]	Vit A [0.78]	Skin Viral [0.8]	Anxiety [0.92]	Epilepsy [1.26]	Headaches [1.3]	Diarrhea [1.14]
Dominican Rep	Iron [1.37]	Asthma [1.4]	Neonatal [1.03]	Dermatitis [1.21]	Vit A [0.61]	Diarrhea [1.35]	Skin Viral [0.85]	Anxiety [1.03]	Headaches [1.33]	Conduct [0.72]
Grenada	Asthma [1.65]	Neonatal [1.06]	Dermatitis [1.15]	Iron [0.96]	Vit A [0.96]	Diarrhea [1.54]	Skin Viral [0.82]	Anxiety [0.97]	Headaches [1.31]	Conduct [0.7]
Guyana	Iron [1.41]	Asthma [1.81]	Neonatal [1.1]	Dermatitis [1.22]	Vit A [0.69]	Skin Viral [0.86]	Anxiety [1.05]	Headaches [1.35]	Epilepsy [0.93]	Conduct [0.72]
Haiti	Iron [1.22]	Vit A [0.74]	Asthma [1.78]	Dermatitis [1.2]	Neonatal [1.08]	Disaster [963.73]	Diarrhea [1.05]	Skin Viral [0.87]	Anxiety [1.09]	Headaches [1.52]
Jamaica	Asthma [1.97]	Neonatal [1.02]	Dermatitis [1.12]	Iron [0.99]	Vit A [0.89]	Skin Viral [0.81]	Anxiety [0.93]	Headaches [1.3]	Epilepsy [1.17]	Conduct [0.69]
Puerto Rico	Asthma [3.13]	Neonatal [1.04]	Dermatitis [1.03]	Iron [2.02]	Vit A [1.84]	Skin Viral [0.79]	Anxiety [0.78]	Headaches [1.32]	Diarrhea [1.76]	Conduct [0.69]
St Lucia	Asthma [1.91]	Neonatal [1.08]	Dermatitis [1.14]	Iron [0.88]	Diarrhea [1.39]	Vit A [0.61]	Skin Viral [0.81]	Anxiety [0.95]	Headaches [1.31]	Conduct [0.69]
St Vincent	Iron [1.79]	Asthma [1.85]	Neonatal [1.03]	Dermatitis [1.19]	Vit A [0.67]	Skin Viral [0.84]	Anxiety [1.02]	Headaches [1.32]	Diarrhea [0.94]	Epilepsy [0.95]
Suriname	Asthma [2.0]	Iron [1.55]	Neonatal [1.02]	Dermatitis [1.15]	Vit A [0.84]	Skin Viral [0.82]	Anxiety [0.97]	Headaches [1.3]	Epilepsy [1.03]	Conduct [0.7]
Trinidad Tobago	Asthma [2.13]	Neonatal [1.04]	Dermatitis [1.1]	Skin Viral [0.8]	Anxiety [0.9]	Headaches [1.3]	Vit A [0.62]	Iron [0.55]	Conduct [0.69]	Epilepsy [1.06]
Virgin Isl US	Asthma [2.29]	Neonatal [1.0]	Dermatitis [1.03]	Iron [2.2]	Diarrhea [2.15]	Skin Viral [0.79]	Anxiety [0.79]	Headaches [1.33]	Conduct [0.69]	Epilepsy [1.31]
**Central Latin America**	Neonatal [1.37]	Asthma [0.93]	Dermatitis [0.81]	Vit A [0.69]	Diarrhea [1.53]	Epilepsy [1.54]	Conduct [0.85]	Skin Viral [0.83]	Iron [0.38]	Congenital [0.73]
Colombia	Neonatal [1.37]	Dermatitis [0.89]	Asthma [0.95]	Diarrhea [1.81]	Epilepsy [1.57]	Skin Viral [0.83]	Conduct [0.81]	Headaches [1.22]	URI [1.26]	Vit A [0.41]
Costa Rica	Neonatal [1.27]	Asthma [1.36]	Dermatitis [0.83]	Diarrhea [2.07]	Epilepsy [1.45]	Skin Viral [0.82]	Conduct [0.81]	Vit A [0.48]	Headaches [1.15]	Congenital [0.67]
El Salvador	Neonatal [1.39]	Iron [1.16]	Vit A [1.07]	Asthma [1.21]	Dermatitis [0.82]	Diarrhea [1.6]	Epilepsy [1.31]	Skin Viral [0.85]	Conduct [0.84]	Headaches [1.17]
Guatemala	Neonatal [1.46]	Iron [0.71]	Dermatitis [1.07]	Diarrhea [1.79]	Asthma [0.8]	Vit A [0.39]	Skin Viral [0.87]	Conduct [0.86]	Epilepsy [0.97]	Headaches [1.25]
Honduras	Iron [0.88]	Neonatal [1.58]	Asthma [1.18]	Dermatitis [1.06]	Vit A [0.6]	Diarrhea [1.81]	Epilepsy [1.42]	Skin Viral [0.87]	Conduct [0.86]	Headaches [1.26]

eFigure 9d. Leading ten causes of YLDs with the ratio of observed YLDs to YLDs expected on the basis of Socio-Demographic Index alone in 2017, 5-9 years, both sexes combined.
The top ten causes contributing to YLDs are listed globally, by socio-demographic quintile, and then by GBD superregion, region, country, and subnationally where modeled. For each cell, the ratio of observed YLDs to YLDs expected on the basis of socio-demographic index (SDI) alone are listed. Abbreviations: YLD=year of life lived with disability, GBD=Global Burden of Disease.

Values shown in brackets represent the ratio of observed YLDs to predicted YLDs on the basis of Socio-Demographic Index (SDI), rounded to two (2) digits. Color ranges (shown below) were calculated to place a roughly equal number of cells into each bin.

COLOR KEY:	[0.0-0.8]	(0.8-0.89]	(0.89-0.97]	(0.97-1.02]	(1.02-1.1]	(1.1-1.24]	(1.24-1.42]	(1.42-1.91]	1.91+

	1	2	3	4	5	6	7	8	9	10
Mexico	Neonatal [1.38]	Vit A [0.83]	Asthma [0.87]	Dermatitis [0.66]	Epilepsy [1.67]	Conduct [0.87]	Skin Viral [0.82]	Congenital [0.77]	Headaches [1.07]	Urticaria [1.02]
Aguascalientes	Neonatal [1.36]	Asthma [0.96]	Dermatitis [0.65]	Epilepsy [1.78]	Vit A [0.68]	Conduct [0.87]	Skin Viral [0.81]	Congenital [0.83]	Iron [0.4]	Headaches [1.07]
Baja CA	Neonatal [1.34]	Vit A [1.1]	Asthma [0.84]	Dermatitis [0.65]	Epilepsy [1.73]	Conduct [0.87]	Skin Viral [0.81]	Congenital [0.77]	Headaches [1.07]	Urticaria [1.05]
Baja CA Sur	Neonatal [1.42]	Vit A [1.09]	Asthma [0.93]	Dermatitis [0.65]	Epilepsy [1.76]	Conduct [0.87]	Skin Viral [0.81]	Congenital [0.8]	Headaches [1.07]	Urticaria [1.05]
Campeche	Neonatal [1.51]	Vit A [0.97]	Asthma [0.86]	Dermatitis [0.68]	Epilepsy [1.56]	Iron [0.51]	Conduct [0.88]	Skin Viral [0.83]	Congenital [0.76]	Diarrhea [0.89]
Chiapas	Neonatal [1.45]	Vit A [0.62]	Asthma [0.85]	Dermatitis [0.7]	Epilepsy [1.49]	Conduct [0.91]	Skin Viral [0.87]	Diarrhea [0.97]	Congenital [0.7]	Headaches [1.15]
Chihuahua	Neonatal [1.27]	Asthma [0.98]	Epilepsy [1.71]	Dermatitis [0.56]	Vit A [0.61]	Conduct [0.87]	Skin Viral [0.82]	Congenital [0.85]	Diarrhea [1.01]	Headaches [1.07]
Coahuila	Neonatal [1.16]	Asthma [0.97]	Dermatitis [0.66]	Epilepsy [1.71]	Vit A [0.72]	Iron [0.5]	Conduct [0.87]	Diarrhea [1.23]	Skin Viral [0.82]	Congenital [0.75]
Colima	Neonatal [1.45]	Vit A [0.98]	Asthma [0.9]	Dermatitis [0.65]	Epilepsy [1.76]	Conduct [0.87]	Skin Viral [0.81]	Congenital [0.72]	Headaches [1.07]	Urticaria [1.04]
Mexico City	Neonatal [1.45]	Dermatitis [0.81]	Asthma [0.85]	Epilepsy [2.12]	Vit A [0.77]	Skin Viral [0.8]	Conduct [0.81]	Congenital [0.83]	Headaches [1.07]	Urticaria [1.16]
Durango	Neonatal [1.22]	Dermatitis [0.88]	Asthma [0.96]	Vit A [0.79]	Epilepsy [1.65]	Conduct [0.87]	Skin Viral [0.83]	Congenital [0.77]	Diarrhea [0.92]	Headaches [1.08]
Guanajuato	Neonatal [1.25]	Vit A [1.01]	Asthma [0.93]	Dermatitis [0.68]	Epilepsy [1.68]	Conduct [0.88]	Skin Viral [0.83]	Congenital [0.77]	ASD [1.63]	Headaches [1.08]
Guerrero	Neonatal [1.49]	Vit A [0.67]	Asthma [0.86]	Dermatitis [0.71]	Epilepsy [1.52]	Iron [0.31]	Skin Viral [0.92]	Conduct [0.91]	Congenital [0.73]	Headaches [1.13]
Hidalgo	Neonatal [1.72]	Asthma [0.91]	Vit A [0.6]	Dermatitis [0.7]	Epilepsy [1.57]	Conduct [0.9]	Skin Viral [0.86]	Congenital [0.75]	Headaches [1.11]	Urticaria [0.99]
Jalisco	Neonatal [1.31]	Epilepsy [2.02]	Asthma [0.8]	Vit A [0.8]	Dermatitis [0.66]	Conduct [0.87]	Skin Viral [0.81]	Congenital [0.83]	Headaches [1.07]	Urticaria [1.04]
Mexico	Neonatal [1.63]	Vit A [0.79]	Asthma [0.8]	Epilepsy [1.67]	Dermatitis [0.57]	Conduct [0.87]	Skin Viral [0.82]	Congenital [0.7]	Iron [0.33]	Urticaria [1.02]
Michoacan	Neonatal [1.2]	Vit A [0.74]	Asthma [0.9]	Dermatitis [0.7]	Epilepsy [1.51]	Conduct [0.9]	Skin Viral [0.86]	Congenital [0.75]	Headaches [1.11]	Urticaria [0.99]
Morelos	Neonatal [1.34]	Vit A [0.85]	Asthma [0.83]	Epilepsy [1.72]	Dermatitis [0.57]	Conduct [0.87]	Skin Viral [0.82]	Congenital [0.83]	Headaches [1.07]	Urticaria [1.02]
Nayarit	Neonatal [1.47]	Vit A [0.95]	Asthma [0.89]	Dermatitis [0.67]	Epilepsy [1.59]	Conduct [0.88]	Skin Viral [0.83]	Iron [0.38]	Congenital [0.79]	Headaches [1.08]
Nuevo Leon	Neonatal [1.1]	Vit A [1.34]	Asthma [0.83]	Epilepsy [1.84]	Dermatitis [0.55]	Conduct [0.86]	Skin Viral [0.8]	Congenital [0.78]	Diarrhea [1.01]	Headaches [1.07]
Oaxaca	Neonatal [1.42]	Asthma [0.87]	Dermatitis [0.71]	Vit A [0.51]	Epilepsy [1.58]	Conduct [0.91]	Skin Viral [0.87]	Congenital [0.81]	Headaches [1.13]	Urticaria [0.99]
Puebla	Neonatal [1.32]	Asthma [0.9]	Vit A [0.61]	Dermatitis [0.7]	Epilepsy [1.17]	Conduct [0.9]	Skin Viral [0.85]	Congenital [0.76]	Headaches [1.11]	Urticaria [0.99]
Queretaro	Neonatal [1.44]	Asthma [0.93]	Dermatitis [0.66]	Epilepsy [1.7]	Vit A [0.62]	Conduct [0.87]	Skin Viral [0.82]	Congenital [0.76]	Headaches [1.07]	Urticaria [1.03]
Quintana Roo	Neonatal [1.73]	Vit A [1.11]	Iron [0.67]	Asthma [0.86]	Dermatitis [0.67]	Epilepsy [1.69]	Diarrhea [1.22]	Conduct [0.87]	Skin Viral [0.82]	Congenital [0.81]
San Luis Potosi	Neonatal [1.19]	Asthma [0.92]	Vit A [0.83]	Dermatitis [0.68]	Epilepsy [1.64]	Conduct [0.88]	Skin Viral [0.83]	Congenital [0.84]	Headaches [1.08]	Urticaria [1.0]
Sinaloa	Neonatal [1.24]	Vit A [1.05]	Asthma [0.9]	Dermatitis [0.66]	Epilepsy [1.69]	Iron [0.48]	Conduct [0.87]	Skin Viral [0.81]	Congenital [0.74]	Headaches [1.07]
Sonora	Neonatal [1.21]	Vit A [0.97]	Asthma [0.94]	Dermatitis [0.66]	Epilepsy [1.76]	Iron [0.51]	Conduct [0.87]	Skin Viral [0.81]	Congenital [0.83]	Headaches [1.07]
Tabasco	Neonatal [1.49]	Vit A [1.04]	Iron [0.77]	Asthma [0.8]	Dermatitis [0.67]	Epilepsy [1.6]	Conduct [0.88]	Skin Viral [0.83]	Congenital [0.86]	Diarrhea [0.97]
Tamaulipas	Neonatal [1.52]	Asthma [0.9]	Vit A [0.88]	Epilepsy [1.68]	Dermatitis [0.55]	Conduct [0.87]	Skin Viral [0.82]	Diarrhea [1.16]	Congenital [0.79]	Headaches [1.07]
Tlaxcala	Neonatal [1.48]	Asthma [0.93]	Vit A [0.77]	Dermatitis [0.69]	Epilepsy [1.65]	Conduct [0.88]	Skin Viral [0.84]	Congenital [0.84]	Headaches [1.09]	Urticaria [0.99]
Veracruz	Neonatal [1.48]	Vit A [0.74]	Asthma [0.85]	Dermatitis [0.7]	Epilepsy [1.62]	Conduct [0.89]	Skin Viral [0.85]	Congenital [0.81]	Headaches [1.1]	Urticaria [0.99]

eFigure 9d. Leading ten causes of YLDs with the ratio of observed YLDs to YLDs expected on the basis of Socio-Demographic Index alone in 2017, 5-9 years, both sexes combined. The top ten causes contributing to YLDs are listed globally, by socio-demographic quintile, and then by GBD superregion, region, country, and subnationally where modeled. For each cell, the ratio of observed YLDs to YLDs expected on the basis of socio-demographic index (SDI) alone are listed. Abbreviations: YLD=year of life lived with disability, GBD=Global Burden of Disease.

Values shown in brackets represent the ratio of observed YLDs to predicted YLDs on the basis of Socio-Demographic Index (SDI), rounded to two (2) digits. Color ranges (shown below) were calculated to place a roughly equal number of cells into each bin.

COLOR KEY:		[0.0-0.8]	(0.8-0.89]	(0.89-0.97]	(0.97-1.02]	(1.02-1.1]	(1.1-1.24]	(1.24-1.42]	(1.42-1.91]	1.91+
	1	2	3	4	5	6	7	8	9	10
Yucatan	Neonatal [1.27]	Asthma [0.83]	Vit A [0.77]	Epilepsy [1.68]	Dermatitis [0.63]	Iron [0.51]	Conduct [0.87]	Skin Viral [0.82]	Congenital [0.81]	Headaches [1.08]
Zacatecas	Neonatal [0.95]	Asthma [0.94]	Vit A [0.71]	Dermatitis [0.68]	Epilepsy [1.66]	Conduct [0.88]	Skin Viral [0.84]	Iron [0.33]	Congenital [0.71]	Headaches [1.08]
Nicaragua	Neonatal [1.47]	Dermatitis [1.07]	Diarrhea [2.11]	Asthma [0.94]	Epilepsy [1.19]	Iron [0.26]	Skin Viral [0.87]	Conduct [0.86]	Vit A [0.29]	Congenital [0.77]
Panama	Neonatal [1.25]	Dermatitis [1.1]	Asthma [1.28]	Iron [1.17]	Diarrhea [2.78]	Vit A [0.71]	Skin Viral [0.81]	Epilepsy [1.27]	Conduct [0.81]	Headaches [1.15]
Venezuela	Neonatal [1.36]	Diarrhea [3.49]	Dermatitis [0.95]	Asthma [1.01]	Epilepsy [1.5]	Vit A [0.62]	Skin Viral [0.82]	Conduct [0.82]	Headaches [1.14]	Congenital [0.7]
**Tropical Latin America**	Asthma [1.69]	Vit A [1.38]	Neonatal [0.91]	Dermatitis [0.77]	Iron [0.74]	Headaches [1.67]	Anxiety [1.06]	Congenital [0.92]	Skin Viral [0.81]	Back Pain [2.48]
Brazil	Asthma [1.69]	Vit A [1.42]	Neonatal [0.92]	Dermatitis [0.75]	Iron [0.75]	Headaches [1.66]	Anxiety [1.05]	Congenital [0.92]	Skin Viral [0.8]	Back Pain [2.5]
Acre	Iron [1.07]	Asthma [1.47]	Neonatal [0.99]	Vit A [1.07]	Dermatitis [0.8]	Diarrhea [1.43]	Headaches [1.7]	Congenital [1.04]	Anxiety [1.15]	Skin Viral [0.84]
Alagoas	Vit A [1.22]	Asthma [1.38]	Neonatal [0.85]	Dermatitis [0.68]	Iron [0.36]	Headaches [1.77]	Anxiety [1.2]	Diarrhea [1.1]	Congenital [0.93]	Skin Viral [0.86]
Amapa	Asthma [1.6]	Neonatal [0.96]	Iron [1.0]	Vit A [1.04]	Dermatitis [0.76]	Headaches [1.67]	Anxiety [1.06]	Congenital [0.9]	Skin Viral [0.81]	Back Pain [2.41]
Amazonas	Asthma [1.57]	Vit A [1.33]	Iron [1.1]	Neonatal [0.82]	Dermatitis [0.78]	Headaches [1.68]	Anxiety [1.1]	Congenital [0.91]	Skin Viral [0.82]	Back Pain [2.57]
Bahia	Vit A [1.46]	Asthma [1.43]	Iron [0.82]	Neonatal [0.91]	Dermatitis [1.09]	Diarrhea [1.36]	Headaches [1.71]	Anxiety [1.16]	Congenital [0.92]	Skin Viral [0.84]
Ceara	Vit A [1.51]	Asthma [1.51]	Neonatal [0.86]	Dermatitis [0.81]	Headaches [1.7]	Diarrhea [1.26]	Anxiety [1.15]	Scabies [2.67]	Congenital [0.93]	Skin Viral [0.84]
Distrito Federal	Asthma [1.97]	Neonatal [1.01]	Dermatitis [0.69]	Vit A [1.85]	Headaches [1.68]	Anxiety [0.9]	Congenital [0.98]	Skin Viral [0.79]	Back Pain [2.34]	Conduct [0.74]
Espirito Santo	Asthma [1.94]	Vit A [2.01]	Neonatal [0.9]	Iron [1.0]	Dermatitis [0.75]	Headaches [1.66]	Anxiety [1.04]	Congenital [0.9]	Skin Viral [0.8]	Back Pain [2.42]
Goias	Asthma [1.72]	Neonatal [0.83]	Vit A [1.16]	Dermatitis [0.77]	Iron [0.66]	Headaches [1.68]	Anxiety [1.07]	Congenital [0.9]	Skin Viral [0.81]	Back Pain [2.53]
Maranhao	Vit A [1.09]	Asthma [1.37]	Iron [0.55]	Neonatal [0.95]	Dermatitis [0.81]	Headaches [1.82]	Anxiety [1.21]	Diarrhea [1.02]	Skin Viral [0.86]	Congenital [0.87]
Mato Grosso	Asthma [1.81]	Vit A [1.35]	Neonatal [0.87]	Dermatitis [0.76]	Headaches [1.67]	Anxiety [1.06]	Congenital [0.9]	Skin Viral [0.81]	Diarrhea [1.25]	Back Pain [2.53]
Mato Grosso do Sul	Asthma [1.66]	Neonatal [1.0]	Vit A [0.98]	Dermatitis [0.77]	Headaches [1.68]	Congenital [1.01]	Anxiety [1.07]	Diarrhea [1.26]	Skin Viral [0.81]	Back Pain [2.56]
Minas Gerais	Asthma [1.78]	Neonatal [0.96]	Vit A [1.31]	Dermatitis [0.65]	Headaches [1.66]	Iron [0.55]	Anxiety [1.06]	Diarrhea [1.3]	Congenital [0.91]	Skin Viral [0.81]
Para	Asthma [1.54]	Vit A [0.99]	Iron [0.79]	Neonatal [0.88]	Dermatitis [0.83]	Diarrhea [1.67]	Headaches [1.73]	Anxiety [1.18]	Congenital [0.91]	Skin Viral [0.85]
Paraiba	Vit A [1.52]	Asthma [1.44]	Neonatal [0.82]	Dermatitis [0.82]	Diarrhea [1.48]	Headaches [1.74]	Iron [0.35]	Anxiety [1.18]	Congenital [0.93]	Skin Viral [0.86]
Parana	Asthma [1.91]	Neonatal [0.94]	Vit A [1.18]	Dermatitis [0.67]	Headaches [1.67]	Anxiety [1.03]	Congenital [0.97]	Skin Viral [0.8]	Back Pain [2.5]	Diarrhea [1.23]
Pernambuco	Vit A [1.4]	Iron [1.05]	Asthma [1.55]	Neonatal [0.86]	Dermatitis [0.79]	Headaches [1.69]	Anxiety [1.16]	Congenital [0.97]	Skin Viral [0.84]	Back Pain [2.61]
Piaui	Asthma [1.48]	Vit A [0.83]	Neonatal [0.98]	Dermatitis [0.83]	Headaches [1.78]	Diarrhea [1.2]	Anxiety [1.21]	Congenital [0.95]	Skin Viral [0.87]	Back Pain [2.81]
Rio de Janeiro	Asthma [1.74]	Neonatal [1.14]	Dermatitis [0.73]	Headaches [1.67]	Vit A [0.84]	Anxiety [0.99]	Congenital [0.9]	Skin Viral [0.79]	Conduct [0.75]	Epilepsy [1.2]
Rio Grande do Norte	Asthma [1.51]	Vit A [1.12]	Neonatal [0.79]	Iron [0.69]	Dermatitis [0.8]	Diarrhea [1.47]	Headaches [1.69]	Anxiety [1.14]	Skin Viral [0.84]	Congenital [0.89]
Rio Grande do Sul	Asthma [1.95]	Neonatal [0.96]	Dermatitis [0.98]	Congenital [1.57]	Epilepsy [2.11]	Vit A [1.01]	Back Pain [3.24]	Headaches [1.71]	Anxiety [1.01]	Iron [0.66]
Rondonia	Asthma [1.55]	Neonatal [1.01]	Vit A [1.09]	Iron [0.71]	Congenital [1.6]	Dermatitis [0.79]	Headaches [1.68]	Anxiety [1.11]	Skin Viral [0.82]	Hearing [2.16]
Roraima	Asthma [1.58]	Neonatal [1.01]	Vit A [1.38]	Iron [0.83]	Dermatitis [0.77]	Headaches [1.67]	Anxiety [1.07]	Congenital [0.91]	Skin Viral [0.81]	Back Pain [2.55]
Santa Catarina	Asthma [2.07]	Vit A [1.63]	Neonatal [0.86]	Iron [1.1]	Dermatitis [0.74]	Headaches [2.07]	Anxiety [1.01]	Congenital [0.95]	Skin Viral [0.79]	Back Pain [2.4]

eFigure 9d. Leading ten causes of YLDs with the ratio of observed YLDs to YLDs expected on the basis of Socio-Demographic Index alone in 2017, 5-9 years, both sexes combined.
The top ten causes contributing to YLDs are listed globally, by socio-demographic quintile, and then by GBD superregion, region, country, and subnationally where modeled. For each cell, the ratio of observed YLDs to YLDs expected on the basis of socio-demographic index (SDI) alone are listed. Abbreviations: YLD=year of life lived with disability, GBD=Global Burden of Disease.

Values shown in brackets represent the ratio of observed YLDs to predicted YLDs on the basis of Socio-Demographic Index (SDI), rounded to two (2) digits. Color ranges (shown below) were calculated to place a roughly equal number of cells into each bin.

COLOR KEY:	[0.0-0.8]	(0.8-0.89]	(0.89-0.97]	(0.97-1.02]	(1.02-1.1]	(1.1-1.24]	(1.24-1.42]	(1.42-1.91]	1.91+

	1	2	3	4	5	6	7	8	9	10
Sao Paulo	Asthma [1.91]	Neonatal [0.98]	Vit A [1.26]	Dermatitis [0.63]	Headaches [1.6]	Anxiety [0.97]	Iron [0.74]	Skin Viral [0.79]	Back Pain [2.44]	Conduct [0.75]
Sergipe	Vit A [1.59]	Asthma [1.63]	Neonatal [1.0]	Iron [0.59]	Dermatitis [0.66]	Headaches [1.69]	Anxiety [1.13]	Congenital [0.9]	Skin Viral [0.83]	Back Pain [2.56]
Tocantins	Asthma [1.52]	Neonatal [0.98]	Vit A [1.09]	Dermatitis [0.8]	Iron [0.5]	Headaches [1.69]	Anxiety [1.13]	Skin Viral [0.83]	Congenital [0.89]	Back Pain [2.62]
Paraguay	Asthma [1.77]	Dermatitis [1.05]	Iron [0.82]	Neonatal [0.78]	Vit A [0.89]	Diarrhea [2.06]	Headaches [1.72]	Anxiety [1.14]	Congenital [0.97]	Skin Viral [0.83]
**North Africa and Middle East**	Iron [1.81]	Neonatal [0.96]	Vit A [1.04]	Asthma [0.98]	Dermatitis [0.7]	Diarrhea [1.67]	Conduct [1.08]	Anxiety [1.02]	Skin Viral [0.8]	Congenital [0.84]
Afghanistan	Vit A [0.47]	Iron [0.38]	Diarrhea [1.65]	Dermatitis [0.99]	Neonatal [1.35]	Asthma [1.12]	ID [3.72]	Conduct [1.16]	Leish [1508.77]	Conflict Terror [62.7]
Algeria	Neonatal [1.07]	Asthma [1.12]	Iron [1.1]	Dermatitis [0.68]	Vit A [0.98]	Conduct [1.07]	Anxiety [0.97]	Skin Viral [0.78]	Congenital [0.83]	Epilepsy [1.18]
Bahrain	Neonatal [1.03]	Dermatitis [0.9]	Asthma [1.04]	Conduct [1.06]	Diarrhea [1.85]	Vit A [0.8]	Anxiety [0.95]	Skin Viral [0.78]	Epilepsy [1.19]	Congenital [0.77]
Egypt	Iron [1.38]	Neonatal [1.04]	Asthma [1.17]	Conduct [1.1]	Dermatitis [0.57]	Diarrhea [1.18]	Vit A [0.46]	Epilepsy [1.19]	Skin Viral [0.82]	Anxiety [0.98]
Iran	Neonatal [1.24]	Iron [1.1]	Asthma [0.85]	Diarrhea [2.01]	Anxiety [1.26]	Conduct [1.12]	Dermatitis [0.54]	Congenital [0.94]	Epilepsy [1.37]	Skin Viral [0.77]
Iraq	Iron [1.59]	Neonatal [1.12]	Vit A [0.97]	Asthma [1.03]	Dermatitis [0.66]	Conduct [1.12]	Diarrhea [1.22]	Anxiety [1.05]	Conflict Terror [1304.79]	Skin Viral [0.83]
Jordan	Neonatal [1.39]	Asthma [1.32]	Iron [1.23]	Vit A [1.07]	Dermatitis [0.68]	Conduct [1.06]	Anxiety [0.97]	Diarrhea [1.44]	Skin Viral [0.78]	Congenital [0.82]
Kuwait	Neonatal [1.16]	Asthma [1.3]	Iron [2.31]	Dermatitis [0.67]	Conduct [1.06]	Diarrhea [2.33]	Anxiety [0.87]	Skin Viral [0.77]	Congenital [0.85]	Epilepsy [1.29]
Lebanon	Neonatal [1.03]	Asthma [1.45]	Dermatitis [0.8]	Iron [1.26]	Anxiety [1.11]	Conduct [1.02]	Diarrhea [1.88]	Congenital [0.89]	Skin Viral [0.78]	Epilepsy [1.11]
Libya	Iron [2.54]	Neonatal [1.03]	Dermatitis [0.87]	Asthma [1.07]	Diarrhea [3.09]	Conduct [1.06]	Anxiety [0.89]	Skin Viral [0.78]	Congenital [0.86]	Conflict Terror [7017.37]
Morocco	Iron [1.11]	Vit A [1.24]	Neonatal [1.06]	Dermatitis [0.82]	Asthma [0.76]	Conduct [1.11]	Diarrhea [1.35]	Anxiety [1.13]	Skin Viral [0.84]	Congenital [0.86]
Palestine	Neonatal [1.14]	Iron [0.58]	Asthma [1.08]	Dermatitis [0.79]	Vit A [0.47]	Conduct [1.13]	Anxiety [1.15]	Diarrhea [1.07]	Skin Viral [0.85]	Congenital [0.82]
Oman	Iron [2.35]	Neonatal [1.07]	Asthma [0.95]	Diarrhea [2.36]	Dermatitis [0.6]	Conduct [1.06]	Anxiety [0.91]	Skin Viral [0.78]	Congenital [0.86]	Vit A [0.79]
Qatar	Neonatal [0.97]	Dermatitis [0.87]	Asthma [0.95]	Iron [1.36]	Conduct [1.06]	Anxiety [0.89]	Skin Viral [0.78]	Congenital [0.85]	Epilepsy [1.28]	Blindness [1.39]
Saudi Arabia	Dermatitis [0.85]	Neonatal [0.73]	Asthma [0.84]	Conduct [1.07]	Diarrhea [2.27]	Anxiety [0.88]	Congenital [0.92]	Skin Viral [0.78]	Epilepsy [1.38]	Iron [0.92]
Sudan	Iron [1.09]	Vit A [0.7]	Diarrhea [1.93]	Asthma [1.02]	Neonatal [0.86]	Dermatitis [0.72]	Conduct [1.12]	ID [4.23]	Hemog [2.14]	Anxiety [1.16]
Syria	Iron [1.53]	Conflict Terror [6366.83]	Neonatal [0.93]	Asthma [0.91]	Vit A [0.7]	Conduct [1.08]	Dermatitis [0.54]	Anxiety [1.08]	Diarrhea [1.18]	Skin Viral [0.82]
Tunisia	Neonatal [1.03]	Dermatitis [0.79]	Asthma [0.87]	Conduct [1.07]	Anxiety [1.0]	Diarrhea [1.3]	Skin Viral [0.79]	Congenital [0.77]	Urticaria [1.04]	Epilepsy [0.91]
Turkey	Neonatal [0.75]	Iron [1.24]	Asthma [0.92]	Vit A [1.24]	Dermatitis [0.61]	Conduct [1.04]	Diarrhea [1.7]	Skin Viral [0.78]	Epilepsy [1.31]	Congenital [0.78]
UAE	Neonatal [1.02]	Asthma [1.45]	Iron [3.32]	Dermatitis [0.85]	Diarrhea [2.39]	Conduct [1.02]	Anxiety [0.82]	Skin Viral [0.77]	Congenital [0.9]	Epilepsy [1.53]
Yemen	Iron [3.47]	Vit A [1.37]	Neonatal [1.29]	Diarrhea [2.06]	Dermatitis [0.97]	Oth NTD [1.98]	Hemog [2.56]	Asthma [0.82]	Conduct [1.1]	Oth Un Inf [3.63]
**South Asia**	Iron [1.96]	Vit A [1.56]	Neonatal [1.5]	Dermatitis [0.6]	ID [4.93]	Congenital [1.03]	Skin Viral [0.85]	Conduct [0.84]	Hemog [2.19]	Diarrhea [0.82]
Bangladesh	Iron [0.99]	Neonatal [1.56]	Vit A [0.48]	Dermatitis [0.89]	Congenital [0.99]	Nematode [3.53]	Skin Viral [0.84]	Anxiety [1.06]	Conduct [0.81]	Headaches [1.17]
Bhutan	Iron [3.55]	Vit A [1.46]	Neonatal [1.33]	Dermatitis [0.9]	Diarrhea [1.31]	Oth NTD [3.29]	Skin Viral [0.83]	Conduct [0.79]	Epilepsy [0.99]	Congenital [0.82]

eFigure 9d. Leading ten causes of YLDs with the ratio of observed YLDs to YLDs expected on the basis of Socio-Demographic Index alone in 2017, 5-9 years, both sexes combined.
The top ten causes contributing to YLDs are listed globally, by socio-demographic quintile, and then by GBD superregion, region, country, and subnationally where modeled. For each cell, the ratio of observed YLDs to YLDs expected on the basis of socio-demographic index (SDI) alone are listed. Abbreviations: YLD=year of life lived with disability, GBD=Global Burden of Disease.

Values shown in brackets represent the ratio of observed YLDs to predicted YLDs on the basis of Socio-Demographic Index (SDI), rounded to two (2) digits. Color ranges (shown below) were calculated to place a roughly equal number of cells into each bin.

COLOR KEY:	[0.0-0.8]	(0.8-0.89]	(0.89-0.97]	(0.97-1.02]	(1.02-1.1]	(1.1-1.24]	(1.24-1.42]	(1.42-1.91]	1.91+

	1	2	3	4	5	6	7	8	9	10
India	Iron [2.09]	Vit A [1.89]	Neonatal [1.52]	ID [6.37]	Congenital [1.1]	Dermatitis [0.51]	Hemog [2.47]	Skin Viral [0.86]	Conduct [0.85]	Diarrhea [0.79]
Andhra Pradesh	Iron [1.62]	Vit A [1.51]	Neonatal [1.69]	ID [5.32]	Congenital [1.06]	Dermatitis [0.52]	Skin Viral [0.85]	Conduct [0.85]	Hemog [1.93]	Urticaria [0.98]
Arunachal Pradesh	Iron [1.78]	Vit A [1.71]	Neonatal [1.59]	ID [5.2]	Congenital [1.08]	Dermatitis [0.53]	Skin Viral [0.85]	Conduct [0.84]	Hemog [2.18]	Urticaria [0.99]
Assam	Iron [1.47]	Vit A [1.47]	Neonatal [1.47]	ID [6.72]	Congenital [1.12]	Dermatitis [0.52]	Skin Viral [0.85]	Conduct [0.85]	Hemog [1.92]	Skin Fung [2.28]
Bihar	Iron [1.46]	Vit A [1.35]	Neonatal [1.79]	ID [6.89]	Congenital [1.07]	Dermatitis [0.51]	Hemog [1.81]	Skin Viral [0.86]	Conduct [0.85]	Diarrhea [0.85]
Chhattisgarh	Iron [1.33]	Vit A [1.12]	Neonatal [1.59]	ID [5.82]	Congenital [1.08]	Dermatitis [0.52]	Skin Viral [0.85]	Conduct [0.85]	Urticaria [0.98]	Blindness [1.3]
Delhi	Iron [6.17]	Vit A [3.26]	Neonatal [1.46]	Congenital [0.96]	Dermatitis [0.41]	Skin Viral [0.78]	Conduct [0.8]	Hemog [5.26]	Blindness [1.62]	Oth NTD [1899.88]
Goa	Neonatal [1.8]	Vit A [3.35]	Congenital [1.01]	Dermatitis [0.46]	Skin Viral [0.79]	Conduct [0.81]	Iron [0.77]	Urticaria [1.23]	Diarrhea [1.21]	Epilepsy [1.05]
Gujarat	Iron [3.69]	Vit A [2.16]	Neonatal [1.53]	Congenital [1.07]	ID [5.12]	Hemog [3.12]	Dermatitis [0.52]	Skin Viral [0.84]	Conduct [0.84]	Diarrhea [0.81]
Haryana	Iron [4.72]	Vit A [2.53]	Neonatal [1.51]	Oth NTD [6.72]	Hemog [3.72]	Congenital [1.04]	Dermatitis [0.51]	Skin Viral [0.82]	ID [4.52]	Conduct [0.83]
Himachal Pradesh	Iron [5.53]	Vit A [2.49]	Neonatal [1.63]	Congenital [1.01]	Hemog [3.97]	Dermatitis [0.49]	ID [5.11]	Skin Viral [0.8]	Conduct [0.81]	Oth Un Inf [5.37]
Jammu & Kashmir	Iron [1.95]	Vit A [2.1]	Neonatal [1.51]	ID [6.22]	Congenital [1.09]	Dermatitis [0.52]	Skin Viral [0.84]	Conduct [0.84]	Hemog [2.46]	Diarrhea [0.83]
Jharkhand	Iron [1.8]	Vit A [1.55]	Neonatal [1.7]	ID [5.83]	Congenital [1.07]	Conduct [0.98]	Dermatitis [0.51]	Hemog [2.07]	Skin Viral [0.85]	Diarrhea [0.81]
Karnataka	Iron [1.84]	Vit A [1.92]	Neonatal [1.77]	ID [5.35]	Congenital [1.05]	Dermatitis [0.52]	Skin Viral [0.85]	Epilepsy [0.98]	Hemog [2.36]	Conduct [0.7]
Kerala	Neonatal [2.01]	Vit A [2.41]	Iron [1.54]	Congenital [1.03]	Dermatitis [0.48]	Skin Viral [0.8]	Conduct [0.78]	ID [4.9]	Anxiety [0.78]	Urticaria [1.06]
Madhya Pradesh	Iron [2.13]	Vit A [1.67]	Neonatal [1.51]	ID [6.39]	Congenital [1.07]	Hemog [2.31]	Dermatitis [0.51]	Skin Viral [0.85]	Conduct [0.85]	Oth NTD [1.39]
Maharashtra	Iron [3.24]	Vit A [2.29]	Neonatal [1.62]	Congenital [1.04]	ID [5.18]	Conduct [0.86]	Skin Viral [0.81]	Dermatitis [0.42]	Hemog [2.79]	Urticaria [1.0]
Manipur	Neonatal [1.69]	Vit A [1.36]	Iron [0.7]	ID [6.81]	Congenital [1.1]	Dermatitis [0.52]	Skin Viral [0.84]	Conduct [0.84]	Anxiety [0.88]	Diarrhea [0.85]
Meghalaya	Iron [2.3]	Vit A [2.07]	Neonatal [1.63]	ID [6.25]	Congenital [1.1]	Dermatitis [0.52]	Hemog [2.61]	Skin Viral [0.85]	Conduct [0.84]	Oth NTD [2.07]
Mizoram	Neonatal [1.66]	Vit A [1.85]	Iron [0.63]	ID [6.14]	Congenital [1.07]	Dermatitis [0.51]	Skin Viral [0.82]	Conduct [0.82]	Diarrhea [1.05]	Urticaria [1.0]
Nagaland	Iron [2.14]	Neonatal [1.72]	Vit A [1.81]	Congenital [1.05]	Dermatitis [0.49]	ID [5.36]	Skin Viral [0.81]	Conduct [0.81]	Iodine [37.31]	Urticaria [1.02]
Odisha	Vit A [1.7]	Neonatal [1.48]	Iron [0.73]	ID [6.39]	Congenital [1.09]	Dermatitis [0.52]	Skin Viral [0.85]	Conduct [0.85]	Diarrhea [0.89]	Hemog [1.94]
Punjab	Iron [3.8]	Vit A [2.38]	Neonatal [1.54]	Congenital [1.04]	Dermatitis [0.48]	ID [5.05]	Hemog [3.3]	Conduct [0.83]	Skin Viral [0.81]	Oth NTD [10.2]
Rajasthan	Iron [1.8]	Vit A [1.58]	Neonatal [1.48]	ID [6.0]	Congenital [1.07]	Dermatitis [0.52]	Hemog [2.03]	Skin Viral [0.85]	Conduct [0.85]	Oth NTD [1.19]
Sikkim	Iron [3.65]	Vit A [2.31]	Neonatal [1.67]	Congenital [1.02]	Dermatitis [0.49]	Skin Viral [0.81]	Conduct [0.81]	ID [4.49]	Hemog [3.11]	Oth NTD [14.76]
Tamil Nadu	Iron [3.36]	Vit A [2.4]	Neonatal [1.57]	ID [5.27]	Dermatitis [0.5]	Hemog [3.18]	Skin Viral [0.82]	Conduct [0.82]	Congenital [0.86]	Urticaria [0.99]
Telangana	Iron [2.67]	Vit A [1.93]	Neonatal [1.65]	ID [5.31]	Congenital [1.07]	Dermatitis [0.52]	Skin Viral [0.84]	Hemog [2.58]	Conduct [0.84]	Oth NTD [2.6]
Tripura	Iron [1.76]	Vit A [2.04]	Neonatal [1.62]	ID [6.25]	Congenital [1.08]	Dermatitis [0.52]	Hemog [2.43]	Skin Viral [0.85]	Conduct [0.85]	Asthma [0.42]
Uttar Pradesh	Iron [1.66]	Vit A [1.67]	Neonatal [1.46]	ID [6.51]	Congenital [1.17]	Hemog [2.18]	Dermatitis [0.5]	Skin Viral [0.85]	Conduct [0.85]	Diarrhea [0.78]
Uttarakhand	Iron [2.99]	Vit A [1.94]	Neonatal [1.64]	Congenital [1.06]	Dermatitis [0.51]	ID [4.99]	Skin Viral [0.82]	Conduct [0.83]	Hemog [2.67]	Diarrhea [0.91]
W Bengal	Iron [1.61]	Vit A [1.84]	Neonatal [1.5]	ID [6.74]	Congenital [1.09]	Skin Viral [0.96]	Dermatitis [0.52]	Conduct [0.85]	Hemog [2.22]	Anxiety [0.84]

eFigure 9d. Leading ten causes of YLDs with the ratio of observed YLDs to YLDs expected on the basis of Socio-Demographic Index alone in 2017, 5-9 years, both sexes combined. The top ten causes contributing to YLDs are listed globally, by socio-demographic quintile, and then by GBD superregion, region, country, and subnationally where modeled. For each cell, the ratio of observed YLDs to YLDs expected on the basis of socio-demographic index (SDI) alone are listed. Abbreviations: YLD=year of life lived with disability, GBD=Global Burden of Disease.

Values shown in brackets represent the ratio of observed YLDs to predicted YLDs on the basis of Socio-Demographic Index (SDI), rounded to two (2) digits. Color ranges (shown below) were calculated to place a roughly equal number of cells into each bin.

COLOR KEY:	[0.0-0.8]	(0.8-0.89]	(0.89-0.97]	(0.97-1.02]	(1.02-1.1]	(1.1-1.24]	(1.24-1.42]	(1.42-1.91]	1.91+

	1	2	3	4	5	6	7	8	9	10
UTs other than Delhi	Iron [4.77]	Vit A [2.89]	Neonatal [1.75]	Congenital [1.02]	Dermatitis [0.48]	Hemog [3.99]	Skin Viral [0.79]	Conduct [0.81]	ID [4.45]	Urticaria [1.04]
Nepal	Iron [0.81]	Neonatal [1.9]	Vit A [0.65]	Dermatitis [0.89]	Epilepsy [1.03]	Congenital [0.82]	Skin Viral [0.84]	Conduct [0.82]	Headaches [1.46]	Anxiety [0.96]
Pakistan	Iron [1.95]	Neonatal [1.42]	Vit A [0.8]	Dermatitis [0.86]	Diarrhea [1.12]	Epilepsy [0.98]	Skin Viral [0.84]	Hemog [1.88]	Conduct [0.81]	Asthma [0.47]
**Southeast Asia, East Asia, and Oceania**	Neonatal [1.06]	Asthma [0.96]	Dermatitis [0.64]	Skin Viral [1.05]	Vit A [0.78]	Congenital [0.89]	Conduct [0.75]	Anxiety [0.74]	Scabies [3.21]	Iron [0.4]
**East Asia**	Neonatal [1.35]	Asthma [0.84]	Skin Viral [1.11]	Dermatitis [0.52]	Hernia [4.49]	Vit A [0.61]	Anxiety [0.76]	Conduct [0.67]	Congenital [0.72]	Scabies [3.43]
China	Neonatal [1.37]	Asthma [0.83]	Skin Viral [1.11]	Dermatitis [0.51]	Hernia [4.49]	Vit A [0.59]	Anxiety [0.76]	Conduct [0.66]	Congenital [0.71]	Scabies [3.31]
N Korea	Vit A [0.58]	Neonatal [0.72]	Asthma [0.79]	Diarrhea [1.49]	Dermatitis [0.65]	Skin Viral [1.16]	Anxiety [1.0]	Conduct [0.77]	Hernia [4.4]	Congenital [0.75]
Taiwan	Asthma [1.92]	Dermatitis [0.6]	Neonatal [0.57]	Skin Viral [1.04]	Conduct [0.81]	Anxiety [0.72]	Diarrhea [2.07]	Congenital [0.84]	Urticaria [1.35]	Scabies [10.23]
**Oceania**	Iron [1.23]	Asthma [1.92]	Dermatitis [1.02]	Vit A [0.48]	Neonatal [0.79]	Diarrhea [1.4]	Skin Viral [1.07]	Congenital [0.92]	Conduct [0.85]	Blindness [1.52]
Am Samoa	Asthma [2.26]	Iron [2.17]	Dermatitis [0.93]	Diarrhea [2.68]	Vit A [1.25]	Neonatal [0.69]	Skin Viral [0.99]	Conduct [0.8]	Congenital [0.84]	Anxiety [0.77]
Micronesia	Iron [1.64]	Vit A [1.67]	Asthma [1.87]	Dermatitis [1.03]	Neonatal [0.71]	Diarrhea [1.89]	Skin Viral [1.06]	Congenital [0.92]	Conduct [0.83]	Anxiety [0.91]
Fiji	Iron [1.86]	Asthma [1.82]	Dermatitis [0.97]	Neonatal [0.65]	Vit A [0.86]	Diarrhea [1.93]	Skin Viral [1.01]	Conduct [0.8]	Congenital [0.83]	Anxiety [0.84]
Guam	Asthma [2.27]	Diarrhea [6.1]	Iron [3.91]	Dermatitis [0.87]	Neonatal [0.74]	Skin Viral [0.97]	Conduct [0.79]	Congenital [0.81]	Anxiety [0.69]	Vit A [1.07]
Kiribati	Iron [1.28]	Vit A [0.95]	Asthma [1.91]	Dermatitis [1.02]	Neonatal [0.82]	Diarrhea [1.42]	Skin Viral [1.08]	Congenital [0.9]	Conduct [0.85]	Nematode [2.2]
Marshall	Vit A [1.17]	Asthma [1.74]	Dermatitis [1.04]	Iron [0.57]	Neonatal [0.74]	Diarrhea [1.77]	Skin Viral [1.07]	Congenital [0.93]	Conduct [0.84]	Anxiety [0.94]
N Mariana	Asthma [2.35]	Neonatal [0.84]	Dermatitis [0.9]	Diarrhea [3.45]	Vit A [1.94]	Iron [1.24]	Skin Viral [0.98]	Conduct [0.79]	Congenital [0.84]	Anxiety [0.73]
PNG	Iron [1.09]	Asthma [1.99]	Dermatitis [1.02]	Neonatal [0.89]	Vit A [0.34]	Diarrhea [1.29]	Skin Viral [1.08]	Congenital [0.91]	Conduct [0.86]	Blindness [1.69]
Samoa	Iron [1.55]	Vit A [1.34]	Asthma [1.81]	Dermatitis [1.04]	Diarrhea [1.97]	Neonatal [0.73]	Skin Viral [1.06]	Congenital [0.9]	Conduct [0.83]	Anxiety [0.92]
Solomon	Iron [1.62]	Vit A [0.77]	Asthma [1.84]	Dermatitis [1.02]	Neonatal [0.94]	Diarrhea [1.45]	Skin Viral [1.08]	Congenital [0.91]	Conduct [0.85]	Oth NTD [0.91]
Tonga	Asthma [2.42]	Dermatitis [1.0]	Iron [0.79]	Diarrhea [2.17]	Vit A [0.88]	Neonatal [0.69]	Skin Viral [1.02]	Congenital [0.89]	Conduct [0.81]	Anxiety [0.87]
Vanuatu	Iron [1.09]	Vit A [0.98]	Asthma [2.03]	Dermatitis [1.02]	Neonatal [0.83]	Diarrhea [1.3]	Skin Viral [1.07]	Congenital [0.9]	Conduct [0.85]	Anxiety [0.94]
**Southeast Asia**	Asthma [1.08]	Neonatal [0.72]	Dermatitis [0.82]	Vit A [0.89]	Congenital [1.14]	Skin Viral [0.99]	Iron [0.48]	Conduct [0.87]	Scabies [2.77]	Epilepsy [0.96]
Cambodia	Iron [1.59]	Vit A [0.71]	Dermatitis [1.02]	Asthma [0.95]	Neonatal [0.79]	Congenital [1.16]	Skin Viral [1.06]	Conduct [0.88]	Epilepsy [0.97]	Diarrhea [0.85]
Indonesia	Asthma [1.12]	Vit A [1.07]	Neonatal [0.73]	Dermatitis [0.61]	Skin Viral [0.97]	Congenital [1.01]	Conduct [0.9]	Scabies [3.0]	Diarrhea [0.99]	Epilepsy [0.97]
Laos	Vit A [0.99]	Iron [0.53]	Dermatitis [1.03]	Asthma [1.0]	Neonatal [0.58]	Congenital [1.19]	Skin Viral [1.06]	Conduct [0.89]	Anxiety [1.02]	Scabies [1.85]
Malaysia	Dermatitis [0.95]	Neonatal [0.81]	Asthma [0.99]	Diarrhea [2.31]	Congenital [1.12]	Skin Viral [0.98]	Conduct [0.84]	Anxiety [0.79]	Iron [0.81]	Epilepsy [1.17]
Maldives	Dermatitis [0.96]	Asthma [1.01]	Neonatal [0.66]	Iron [0.69]	Congenital [1.1]	Skin Viral [1.0]	Conduct [0.85]	Scabies [3.21]	Vit A [0.42]	Hearing [1.81]
Mauritius	Iron [2.61]	Asthma [1.27]	Dermatitis [0.91]	Neonatal [0.82]	Diarrhea [1.99]	Skin Viral [0.98]	Congenital [1.09]	Conduct [0.83]	Vit A [0.7]	Epilepsy [1.2]
Myanmar	Iron [0.83]	Vit A [0.82]	Dermatitis [1.04]	Congenital [1.71]	Neonatal [0.74]	Asthma [0.89]	Skin Viral [1.06]	Conduct [0.88]	Scabies [2.05]	Blindness [1.56]
Philippines	Asthma [1.4]	Dermatitis [1.05]	Neonatal [0.77]	Vit A [0.8]	Congenital [1.19]	Skin Viral [1.02]	Conduct [0.86]	Scabies [2.42]	Epilepsy [0.98]	Iron [0.32]
Sri Lanka	Dermatitis [0.9]	Neonatal [0.74]	Asthma [1.01]	Vit A [1.15]	Iron [1.02]	Congenital [1.31]	Skin Viral [1.02]	Epilepsy [1.32]	Conduct [0.83]	Hearing [1.96]

eFigure 9d. Leading ten causes of YLDs with the ratio of observed YLDs to YLDs expected on the basis of Socio-Demographic Index alone in 2017, 5-9 years, both sexes combined. The top ten causes contributing to YLDs are listed globally, by socio-demographic quintile, and then by GBD superregion, region, country, and subnationally where modeled. For each cell, the ratio of observed YLDs to YLDs expected on the basis of socio-demographic index (SDI) alone are listed. Abbreviations: YLD=year of life lived with disability, GBD=Global Burden of Disease.

Values shown in brackets represent the ratio of observed YLDs to predicted YLDs on the basis of Socio-Demographic Index (SDI), rounded to two (2) digits. Color ranges (shown below) were calculated to place a roughly equal number of cells into each bin.

COLOR KEY:	[0.0-0.8]	(0.8-0.89]	(0.89-0.97]	(0.97-1.02]	(1.02-1.1]	(1.1-1.24]	(1.24-1.42]	(1.42-1.91]	1.91+

	1	2	3	4	5	6	7	8	9	10
Seychelles	Dermatitis [0.94]	Neonatal [0.81]	Asthma [1.04]	Vit A [0.8]	Skin Viral [0.99]	Congenital [1.1]	Conduct [0.84]	Iron [0.52]	Diarrhea [1.2]	Epilepsy [1.11]
Thailand	Dermatitis [0.98]	Asthma [0.98]	Neonatal [0.67]	Congenital [1.15]	Skin Viral [0.99]	Conduct [0.84]	Scabies [3.92]	Anxiety [0.7]	Hearing [1.91]	Vit A [0.47]
Timor-Leste	Iron [1.05]	Vit A [0.66]	Dermatitis [1.02]	Asthma [0.94]	Neonatal [0.67]	Congenital [1.18]	Skin Viral [1.06]	Conduct [0.89]	Diarrhea [0.95]	Blindness [1.55]
Vietnam	Dermatitis [0.84]	Neonatal [0.61]	Asthma [0.8]	Skin Viral [1.04]	Congenital [1.06]	Vit A [0.5]	Conduct [0.87]	Iron [0.31]	Diarrhea [0.89]	Scabies [2.09]
**Sub-Saharan Africa**	Iron [1.43]	Vit A [1.22]	Dermatitis [1.0]	Neonatal [0.99]	Asthma [0.82]	Congenital [1.17]	Epilepsy [1.19]	Malaria [9.81]	Skin Viral [0.96]	Conduct [0.94]
**Central Sub-Saharan Africa**	Iron [1.51]	Vit A [1.34]	Dermatitis [1.16]	Neonatal [1.04]	Asthma [0.99]	Malaria [17.57]	Oncho [203.01]	Epilepsy [1.59]	Hemog [2.15]	Congenital [1.02]
Angola	Iron [1.97]	Vit A [1.16]	Neonatal [1.21]	Dermatitis [1.16]	Asthma [1.17]	Epilepsy [1.71]	Diarrhea [1.18]	Congenital [1.08]	Hemog [2.09]	Conduct [0.93]
C African Rep	Iron [1.66]	Vit A [1.06]	Malaria [3.33]	Dermatitis [1.16]	Asthma [1.11]	Epilepsy [1.46]	Neonatal [1.05]	Diarrhea [1.13]	Oncho [61.24]	Hemog [1.68]
Congo	Iron [2.16]	Vit A [2.43]	Neonatal [1.13]	Asthma [1.28]	Dermatitis [1.17]	Epilepsy [1.99]	Malaria [118.46]	Diarrhea [1.19]	Congenital [0.98]	Conduct [0.92]
Congo DR	Iron [1.07]	Vit A [1.03]	Oncho [144.34]	Dermatitis [1.16]	Neonatal [1.21]	Malaria [4.25]	Asthma [0.95]	Epilepsy [1.43]	Hemog [1.76]	Congenital [0.98]
Eq Guinea	Iron [1.59]	Vit A [1.3]	Dermatitis [1.12]	Asthma [1.14]	Neonatal [0.73]	Epilepsy [2.15]	Malaria [258.23]	Diarrhea [1.35]	Conduct [0.93]	Skin Viral [0.85]
Gabon	Iron [5.13]	Vit A [2.23]	Neonatal [1.22]	Dermatitis [1.1]	Asthma [1.22]	Epilepsy [2.25]	Malaria [469.95]	Oth NTD [49.58]	Conduct [0.87]	Diarrhea [1.27]
**Eastern Sub-Saharan Africa**	Vit A [0.89]	Iron [0.79]	Neonatal [1.46]	Dermatitis [1.06]	Asthma [1.01]	Congenital [1.19]	Epilepsy [1.13]	Skin Viral [1.07]	Conduct [0.96]	Diarrhea [0.88]
Burundi	Iron [0.61]	Vit A [0.43]	Dermatitis [1.18]	Neonatal [1.17]	Asthma [1.0]	Epilepsy [1.18]	Congenital [1.09]	Skin Viral [1.03]	Diarrhea [0.89]	Conduct [0.94]
Comoros	Iron [1.39]	Vit A [0.87]	Neonatal [1.61]	Dermatitis [1.16]	Asthma [1.09]	Epilepsy [1.17]	Congenital [1.05]	Skin Viral [0.99]	Conduct [0.92]	Diarrhea [0.89]
Djibouti	Iron [0.69]	Neonatal [1.35]	Dermatitis [1.16]	Asthma [0.92]	Epilepsy [1.29]	Vit A [0.32]	Congenital [1.06]	Skin Viral [0.99]	Conduct [0.95]	Anxiety [0.96]
Eritrea	Iron [1.37]	Vit A [0.74]	Neonatal [1.64]	Dermatitis [1.16]	Asthma [0.88]	Epilepsy [1.19]	Congenital [1.06]	Skin Viral [1.0]	Diarrhea [0.97]	Conduct [0.93]
Ethiopia	Vit A [0.97]	Iron [0.6]	Neonatal [1.77]	Dermatitis [1.05]	Asthma [0.94]	Skin Viral [1.28]	Congenital [1.07]	Conduct [1.0]	Diarrhea [0.88]	Epilepsy [0.83]
Kenya	Neonatal [1.73]	Vit A [1.02]	Dermatitis [1.15]	Congenital [1.61]	Asthma [0.76]	Iron [0.3]	Skin Viral [0.98]	Conduct [0.98]	Epilepsy [1.1]	Diarrhea [1.0]
Baringo	Vit A [0.95]	Neonatal [1.88]	Dermatitis [1.12]	Congenital [1.58]	Asthma [0.77]	Diarrhea [1.05]	Skin Viral [0.99]	Conduct [0.99]	Epilepsy [0.95]	Iron [0.17]
Bomet	Neonatal [2.03]	Vit A [0.68]	Dermatitis [1.12]	Congenital [1.58]	Asthma [0.79]	Skin Viral [0.98]	Conduct [0.98]	Diarrhea [0.97]	Epilepsy [0.99]	HIV [103.16]
Bungoma	Vit A [1.31]	Neonatal [1.89]	Iron [0.52]	Dermatitis [1.12]	Congenital [1.58]	Asthma [0.77]	Skin Viral [0.98]	Conduct [0.97]	Epilepsy [0.97]	Diarrhea [0.88]
Busia	Vit A [1.16]	Neonatal [1.89]	Dermatitis [1.12]	Iron [0.39]	Congenital [1.57]	Asthma [0.75]	Malaria [9.7]	Skin Viral [0.99]	Conduct [0.97]	Diarrhea [0.96]
Elgeyo-Marakwet	Neonatal [2.01]	Dermatitis [1.13]	Vit A [0.62]	Congenital [1.59]	Asthma [0.76]	Skin Viral [0.98]	Conduct [0.98]	Epilepsy [1.1]	Diarrhea [0.99]	Anxiety [0.95]
Embu	Vit A [1.51]	Neonatal [1.87]	Iron [0.62]	Dermatitis [1.13]	Congenital [1.59]	Asthma [0.76]	Skin Viral [0.98]	Conduct [0.97]	Epilepsy [1.16]	Diarrhea [1.04]
Garissa	Neonatal [2.59]	Vit A [0.58]	Dermatitis [1.13]	Congenital [1.59]	Asthma [0.84]	Skin Viral [1.02]	Conduct [1.02]	Epilepsy [0.94]	Diarrhea [0.71]	Anxiety [0.99]
HomaBay	Vit A [1.34]	Iron [0.65]	Dermatitis [1.11]	Neonatal [1.14]	Congenital [1.59]	HIV [115.51]	Asthma [0.79]	Diarrhea [1.12]	Epilepsy [1.08]	Skin Viral [0.99]
Isiolo	Vit A [0.73]	Neonatal [1.76]	Dermatitis [1.12]	Congenital [1.58]	Asthma [0.78]	Skin Viral [1.0]	Conduct [1.0]	Diarrhea [0.93]	Epilepsy [0.95]	Anxiety [0.98]
Kajiado	Neonatal [2.03]	Vit A [0.89]	Dermatitis [1.13]	Congenital [1.62]	Asthma [0.77]	Epilepsy [1.23]	Skin Viral [0.98]	Conduct [0.98]	Anxiety [0.95]	Diarrhea [0.87]
Kakamega	Vit A [1.28]	Neonatal [1.61]	Dermatitis [1.12]	Iron [0.41]	Congenital [1.58]	Asthma [0.78]	Diarrhea [1.06]	Skin Viral [0.99]	Conduct [0.98]	Epilepsy [1.01]
Kericho	Neonatal [2.14]	Vit A [0.91]	Dermatitis [1.12]	Congenital [1.61]	Asthma [0.78]	Skin Viral [0.98]	Conduct [0.98]	Diarrhea [1.03]	Epilepsy [1.04]	Anxiety [0.95]

eFigure 9d. Leading ten causes of YLDs with the ratio of observed YLDs to YLDs expected on the basis of Socio-Demographic Index alone in 2017, 5-9 years, both sexes combined. The top ten causes contributing to YLDs are listed globally, by socio-demographic quintile, and then by GBD superregion, region, country, and subnationally where modeled. For each cell, the ratio of observed YLDs to YLDs expected on the basis of socio-demographic index (SDI) alone are listed. Abbreviations: YLD=year of life lived with disability, GBD=Global Burden of Disease.

Values shown in brackets represent the ratio of observed YLDs to predicted YLDs on the basis of Socio-Demographic Index (SDI), rounded to two (2) digits. Color ranges (shown below) were calculated to place a roughly equal number of cells into each bin.

COLOR KEY:	[0.0-0.8]	(0.8-0.89]	(0.89-0.97]	(0.97-1.02]	(1.02-1.1]	(1.1-1.24]	(1.24-1.42]	(1.42-1.91]	1.91+

	1	2	3	4	5	6	7	8	9	10
Kiambu	Neonatal [1.93]	Dermatitis [1.14]	Vit A [0.68]	Congenital [1.62]	Asthma [0.77]	Epilepsy [1.29]	Skin Viral [0.98]	Conduct [0.97]	Diarrhea [0.94]	Anxiety [0.92]
Kilifi	Vit A [0.99]	Neonatal [1.79]	Iron [0.61]	Dermatitis [1.12]	Congenital [1.57]	Asthma [0.72]	Skin Viral [0.98]	Conduct [0.98]	Epilepsy [0.97]	Diarrhea [0.92]
Kirinyaga	Neonatal [1.94]	Dermatitis [1.14]	Congenital [1.62]	Asthma [0.77]	Vit A [0.47]	Diarrhea [1.15]	Skin Viral [0.98]	Epilepsy [1.18]	Conduct [0.98]	Anxiety [0.94]
Kisii	Neonatal [1.56]	Dermatitis [1.13]	Vit A [0.7]	Congenital [1.61]	Asthma [0.75]	Skin Viral [0.98]	Conduct [0.98]	Diarrhea [1.06]	Epilepsy [1.1]	Anxiety [0.95]
Kisumu	Vit A [1.21]	Neonatal [1.1]	Iron [0.5]	Dermatitis [0.94]	Congenital [1.61]	Asthma [0.76]	Epilepsy [1.19]	Skin Viral [0.98]	Conduct [0.97]	Diarrhea [0.99]
Kitui	Neonatal [2.15]	Dermatitis [1.12]	Congenital [1.58]	Vit A [0.39]	Asthma [0.75]	Skin Viral [0.99]	Conduct [0.99]	Diarrhea [1.03]	Epilepsy [1.02]	Anxiety [0.97]
Kwale	Vit A [0.95]	Neonatal [1.84]	Dermatitis [1.12]	Iron [0.36]	Congenital [1.57]	Asthma [0.75]	Skin Viral [0.98]	Conduct [0.98]	Epilepsy [1.05]	Diarrhea [0.89]
Laikipia	Neonatal [2.01]	Vit A [0.94]	Dermatitis [1.14]	Congenital [1.61]	Asthma [0.82]	Skin Viral [0.98]	Conduct [0.98]	Iron [0.29]	Epilepsy [1.16]	Diarrhea [1.02]
Lamu	Neonatal [1.6]	Vit A [0.72]	Dermatitis [1.12]	Congenital [1.57]	Asthma [0.76]	Epilepsy [1.11]	Skin Viral [0.99]	Conduct [0.99]	Diarrhea [0.95]	Conflict Terror [336.32]
Machakos	Neonatal [1.95]	Dermatitis [1.13]	Vit A [0.66]	Congenital [1.59]	Asthma [0.76]	Skin Viral [0.98]	Conduct [0.98]	Epilepsy [1.13]	Diarrhea [1.01]	Anxiety [0.94]
Makueni	Neonatal [2.0]	Dermatitis [1.12]	Vit A [0.57]	Congenital [1.58]	Asthma [0.77]	HIV [115.12]	Skin Viral [0.98]	Conduct [0.98]	Epilepsy [1.02]	Diarrhea [0.87]
Mandera	Vit A [0.87]	Iron [0.55]	Neonatal [2.19]	Dermatitis [1.12]	Congenital [1.57]	Asthma [0.79]	Skin Viral [1.02]	Conduct [1.01]	Epilepsy [0.85]	Diarrhea [0.74]
Marsabit	Neonatal [2.69]	Vit A [0.59]	Dermatitis [1.13]	Congenital [1.58]	Asthma [0.77]	Skin Viral [1.02]	Conduct [1.01]	Diarrhea [0.85]	Epilepsy [0.89]	Anxiety [0.99]
Meru	Neonatal [1.94]	Vit A [0.85]	Dermatitis [1.13]	Congenital [1.61]	Asthma [0.77]	Diarrhea [1.13]	Skin Viral [0.98]	Conduct [0.98]	Epilepsy [1.11]	Anxiety [0.95]
Migori	Vit A [0.98]	Dermatitis [1.12]	Neonatal [1.06]	Iron [0.33]	Congenital [1.58]	Asthma [0.79]	HIV [83.32]	Diarrhea [1.06]	Skin Viral [0.99]	Epilepsy [1.04]
Mombasa	Neonatal [1.57]	Dermatitis [1.14]	Vit A [0.86]	Congenital [1.62]	Asthma [0.74]	Epilepsy [1.29]	Skin Viral [0.98]	Conduct [0.97]	Diarrhea [1.05]	Iron [0.28]
Murang’a	Neonatal [1.95]	Dermatitis [1.13]	Congenital [1.62]	Vit A [0.52]	Asthma [0.77]	Skin Viral [0.98]	Conduct [0.98]	Diarrhea [1.09]	Epilepsy [1.11]	Anxiety [0.94]
Nairobi	Neonatal [1.73]	Dermatitis [1.37]	Vit A [1.24]	Congenital [1.51]	Asthma [0.81]	Epilepsy [1.61]	Skin Viral [0.91]	Conduct [0.92]	Iron [0.5]	Anxiety [0.81]
Nakuru	Neonatal [1.82]	Dermatitis [1.14]	Vit A [0.7]	Congenital [1.64]	Epilepsy [1.71]	Asthma [0.81]	Skin Viral [0.98]	Diarrhea [1.14]	Conduct [0.98]	Anxiety [0.95]
Nandi	Neonatal [2.05]	Vit A [0.88]	Dermatitis [1.12]	Congenital [1.6]	Asthma [0.78]	Iron [0.26]	Skin Viral [0.98]	Conduct [0.98]	Epilepsy [1.07]	Diarrhea [0.94]
Narok	Vit A [0.9]	Neonatal [2.59]	Dermatitis [1.12]	Congenital [1.58]	Asthma [0.83]	Skin Viral [1.0]	Conduct [1.0]	Diarrhea [0.95]	Epilepsy [0.99]	Iron [0.18]
Nyamira	Neonatal [1.77]	Dermatitis [1.14]	Congenital [1.63]	Vit A [0.53]	Asthma [0.77]	Epilepsy [1.31]	Diarrhea [1.23]	Skin Viral [0.98]	Conduct [0.99]	Anxiety [0.94]
Nyandarua	Neonatal [2.06]	Vit A [0.82]	Dermatitis [1.14]	Congenital [1.63]	Asthma [0.78]	Iron [0.33]	Epilepsy [1.31]	Skin Viral [0.98]	Conduct [0.98]	Diarrhea [1.01]
Nyeri	Neonatal [1.92]	Dermatitis [1.14]	Congenital [1.64]	Asthma [0.77]	Vit A [0.48]	Epilepsy [1.3]	Skin Viral [0.98]	Conduct [0.98]	Diarrhea [0.97]	Anxiety [0.94]
Samburu	Neonatal [3.02]	Vit A [0.35]	Dermatitis [1.13]	Congenital [1.59]	Asthma [0.84]	Diarrhea [0.95]	Skin Viral [1.03]	Conduct [1.01]	Epilepsy [0.82]	Anxiety [1.0]
Siaya	Vit A [0.87]	Neonatal [1.22]	Dermatitis [1.12]	Congenital [1.59]	Asthma [0.82]	Skin Viral [0.99]	Conduct [0.98]	Diarrhea [1.01]	Epilepsy [0.97]	Malaria [10.19]
TaitaTaveta	Neonatal [1.79]	Dermatitis [1.14]	Congenital [1.61]	Asthma [0.75]	Epilepsy [1.29]	Skin Viral [0.98]	Conduct [0.98]	Vit A [0.34]	Diarrhea [1.0]	Anxiety [0.94]
TanaRiver	Vit A [1.31]	Iron [1.08]	Neonatal [2.16]	Dermatitis [1.11]	Congenital [1.58]	Asthma [0.75]	Skin Viral [1.0]	Epilepsy [1.0]	Conduct [0.98]	Hemog [1.58]
TharakaNithi	Neonatal [1.52]	Dermatitis [1.13]	Vit A [0.64]	Congenital [1.59]	Asthma [0.76]	Epilepsy [1.22]	Skin Viral [0.98]	Conduct [0.98]	Diarrhea [1.0]	Anxiety [0.95]
TransNzoia	Neonatal [1.95]	Vit A [1.0]	Dermatitis [1.12]	Congenital [1.58]	Asthma [0.77]	Diarrhea [1.16]	Skin Viral [0.98]	Conduct [0.98]	Epilepsy [1.01]	Anxiety [0.94]
Turkana	Vit A [0.7]	Neonatal [2.63]	Dermatitis [1.12]	Congenital [1.6]	Iron [0.25]	Asthma [0.79]	Diarrhea [0.98]	Skin Viral [1.02]	Conduct [1.01]	HIV [28.79]

eFigure 9d. Leading ten causes of YLDs with the ratio of observed YLDs to YLDs expected on the basis of Socio-Demographic Index alone in 2017, 5-9 years, both sexes combined.
The top ten causes contributing to YLDs are listed globally, by socio-demographic quintile, and then by GBD superregion, region, country, and subnationally where modeled. For each cell, the ratio of observed YLDs to YLDs expected on the basis of socio-demographic index (SDI) alone are listed. Abbreviations: YLD=year of life lived with disability, GBD=Global Burden of Disease.

Values shown in brackets represent the ratio of observed YLDs to predicted YLDs on the basis of Socio-Demographic Index (SDI), rounded to two (2) digits. Color ranges (shown below) were calculated to place a roughly equal number of cells into each bin.

COLOR KEY:	[0.0-0.8]	(0.8-0.89]	(0.89-0.97]	(0.97-1.02]	(1.02-1.1]	(1.1-1.24]	(1.24-1.42]	(1.42-1.91]	1.91+

	1	2	3	4	5	6	7	8	9	10
UasinGishu	Neonatal [1.83]	Dermatitis [1.66]	Vit A [0.96]	Congenital [1.6]	Asthma [0.78]	Skin Viral [0.98]	Conduct [0.98]	Diarrhea [1.09]	Epilepsy [1.13]	Anxiety [0.94]
Vihiga	Vit A [1.05]	Neonatal [1.84]	Dermatitis [1.12]	Congenital [1.59]	Iron [0.36]	Asthma [0.77]	Diarrhea [1.09]	Skin Viral [0.98]	Epilepsy [1.09]	Conduct [0.98]
Wajir	Neonatal [2.97]	Vit A [0.41]	Dermatitis [1.12]	Congenital [1.6]	Asthma [0.81]	Skin Viral [1.03]	Conduct [1.02]	Diarrhea [0.76]	Epilepsy [0.76]	Anxiety [0.99]
WestPokot	Neonatal [2.49]	Vit A [0.62]	Dermatitis [1.12]	Congenital [1.58]	Asthma [0.8]	Diarrhea [0.99]	Skin Viral [1.01]	Conduct [1.0]	Epilepsy [0.87]	Anxiety [0.99]
Madagascar	Iron [0.96]	Vit A [0.71]	Congenital [2.69]	Dermatitis [1.17]	Neonatal [1.52]	Asthma [1.11]	Epilepsy [1.23]	Diarrhea [1.0]	Hearing [1.89]	Skin Viral [1.02]
Malawi	Iron [1.16]	Vit A [0.86]	Dermatitis [1.17]	Neonatal [1.33]	HIV [77.0]	Asthma [1.0]	Congenital [1.09]	Epilepsy [1.1]	Malaria [1.86]	Diarrhea [0.96]
Mozambique	Iron [1.06]	Vit A [0.87]	HIV [84.89]	Neonatal [1.27]	Dermatitis [0.93]	Asthma [1.02]	Malaria [2.3]	Epilepsy [1.41]	Congenital [1.06]	Skin Viral [1.02]
Rwanda	Iron [0.78]	Vit A [0.75]	Neonatal [1.34]	Asthma [1.36]	Dermatitis [0.75]	Epilepsy [1.22]	Congenital [1.07]	Skin Viral [1.01]	Conduct [0.93]	Anxiety [1.0]
Somalia	Vit A [0.81]	Iron [0.72]	Dermatitis [1.16]	Neonatal [1.76]	Asthma [0.95]	Congenital [1.06]	Epilepsy [1.0]	Skin Viral [1.04]	Conduct [0.95]	Diarrhea [0.75]
S Sudan	Iron [0.89]	Vit A [0.62]	Oncho [91.03]	Dermatitis [1.16]	Neonatal [1.51]	Asthma [1.12]	Diarrhea [1.15]	Congenital [1.03]	Epilepsy [1.02]	Skin Viral [1.03]
Tanzania	Iron [1.17]	Vit A [0.75]	Neonatal [1.28]	Asthma [1.31]	Dermatitis [0.93]	Epilepsy [1.15]	Skin Viral [0.94]	Conduct [0.93]	Congenital [0.87]	Anxiety [0.99]
Uganda	Vit A [0.73]	Iron [0.67]	Neonatal [1.31]	Dermatitis [1.07]	Asthma [1.12]	Malaria [5.22]	Epilepsy [1.43]	Skin Viral [1.01]	HIV [58.19]	Conduct [0.94]
Zambia	Vit A [1.5]	Iron [0.77]	Dermatitis [1.16]	Neonatal [1.04]	Asthma [0.81]	Epilepsy [1.47]	Congenital [1.06]	Malaria [15.52]	Skin Viral [0.99]	HIV [98.74]
**Southern Sub-Saharan Africa**	Vit A [1.77]	Dermatitis [1.31]	Neonatal [1.02]	Iron [1.16]	Congenital [1.22]	Conduct [0.93]	HIV [334.16]	Skin Viral [0.85]	Anxiety [0.9]	Epilepsy [0.99]
Botswana	Vit A [2.36]	Iron [2.06]	Neonatal [1.26]	Dermatitis [1.3]	Asthma [0.63]	Conduct [0.89]	Skin Viral [0.85]	Congenital [0.83]	Diarrhea [1.15]	Anxiety [0.85]
Lesotho	Vit A [1.5]	Iron [1.06]	Dermatitis [1.39]	Neonatal [0.93]	HIV [194.7]	Conduct [0.94]	Skin Viral [0.9]	Congenital [0.9]	Diarrhea [0.94]	Epilepsy [0.91]
Namibia	Iron [1.71]	Dermatitis [1.36]	Neonatal [1.07]	Vit A [1.09]	Conduct [0.9]	HIV [276.29]	Diarrhea [1.18]	Skin Viral [0.87]	Asthma [0.53]	Congenital [0.85]
S Africa	Vit A [1.89]	Dermatitis [1.27]	Neonatal [1.05]	Iron [0.96]	Congenital [1.45]	Conduct [0.94]	HIV [448.8]	Skin Viral [0.84]	Anxiety [0.91]	Epilepsy [1.06]
Swaziland	Iron [1.65]	Vit A [1.75]	Dermatitis [1.4]	Neonatal [0.84]	HIV [439.91]	Asthma [0.65]	Conduct [0.93]	Diarrhea [1.14]	Skin Viral [0.9]	Congenital [0.88]
Zimbabwe	Iron [0.82]	Vit A [0.96]	Dermatitis [1.39]	Neonatal [1.15]	Conduct [0.94]	Skin Viral [0.91]	Diarrhea [0.96]	HIV [83.37]	Asthma [0.46]	Congenital [0.75]
**Western Sub-Saharan Africa**	Iron [1.97]	Vit A [1.35]	Dermatitis [0.85]	Malaria [12.13]	Hemog [2.51]	Neonatal [0.69]	Congenital [1.17]	Asthma [0.68]	Epilepsy [1.14]	Diarrhea [0.96]
Benin	Vit A [1.13]	Iron [1.03]	Malaria [5.49]	Neonatal [1.18]	Dermatitis [0.95]	Epilepsy [1.24]	Asthma [0.75]	Hemog [1.66]	Congenital [0.97]	Conduct [0.95]
Burkina Faso	Iron [1.6]	Vit A [0.97]	Malaria [1.76]	Dermatitis [0.95]	Asthma [0.94]	Hemog [1.75]	Diarrhea [1.05]	Neonatal [0.96]	Epilepsy [1.02]	Congenital [0.96]
Cameroon	Iron [1.55]	Vit A [1.57]	Dermatitis [0.91]	Malaria [28.93]	Neonatal [0.65]	Epilepsy [1.38]	Diarrhea [1.3]	Asthma [0.68]	Oncho [182.69]	Congenital [1.01]
Cape Verde	Iron [1.64]	Neonatal [1.41]	Dermatitis [0.97]	Asthma [0.73]	Diarrhea [1.29]	Epilepsy [1.34]	Conduct [0.94]	Congenital [0.97]	Skin Viral [0.91]	Anxiety [0.91]
Chad	Iron [1.92]	Vit A [0.88]	Dermatitis [0.94]	Diarrhea [1.16]	Oth NTD [0.79]	Epilepsy [0.98]	Asthma [0.72]	Congenital [0.93]	Malaria [0.66]	Neonatal [0.81]
Cote d'Ivoire	Iron [1.58]	Vit A [1.34]	Dermatitis [0.94]	Neonatal [0.86]	Hemog [2.32]	Asthma [0.81]	Epilepsy [1.27]	Diarrhea [1.16]	Congenital [1.0]	Malaria [5.08]
Gambia	Iron [1.52]	Vit A [1.4]	Neonatal [1.64]	Dermatitis [0.95]	Diarrhea [1.17]	Asthma [0.71]	Hemog [1.72]	Congenital [0.95]	Conduct [0.94]	Skin Viral [0.91]
Ghana	Iron [2.18]	Vit A [2.23]	Neonatal [0.99]	Epilepsy [1.44]	Malaria [67.67]	Diarrhea [1.19]	Dermatitis [0.54]	Congenital [1.01]	Asthma [0.55]	Hemog [2.44]
Guinea	Iron [1.16]	Vit A [0.93]	Malaria [2.44]	Dermatitis [0.95]	Asthma [0.94]	Hemog [1.87]	Neonatal [0.97]	Epilepsy [1.06]	Diarrhea [0.95]	Congenital [0.97]
Guinea-Bissau	Iron [1.33]	Vit A [1.13]	Dermatitis [0.95]	Neonatal [1.21]	Epilepsy [1.2]	Asthma [0.73]	Congenital [1.03]	LF [11.78]	Conduct [0.95]	Skin Viral [0.93]

eFigure 9d. Leading ten causes of YLDs with the ratio of observed YLDs to YLDs expected on the basis of Socio-Demographic Index alone in 2017, 5-9 years, both sexes combined. The top ten causes contributing to YLDs are listed globally, by socio-demographic quintile, and then by GBD superregion, region, country, and subnationally where modeled. For each cell, the ratio of observed YLDs to YLDs expected on the basis of socio-demographic index (SDI) alone are listed. Abbreviations: YLD=year of life lived with disability, GBD=Global Burden of Disease.

Values shown in brackets represent the ratio of observed YLDs to predicted YLDs on the basis of Socio-Demographic Index (SDI), rounded to two (2) digits. Color ranges (shown below) were calculated to place a roughly equal number of cells into each bin.

COLOR KEY:	[0.0-0.8]	(0.8-0.89]	(0.89-0.97]	(0.97-1.02]	(1.02-1.1]	(1.1-1.24]	(1.24-1.42]	(1.42-1.91]	1.91+

	1	2	3	4	5	6	7	8	9	10
Liberia	Iron [1.47]	Vit A [0.84]	Oncho [211.61]	Dermatitis [0.95]	Neonatal [1.22]	Hemog [2.19]	Diarrhea [1.4]	Epilepsy [1.4]	Malaria [1.77]	Asthma [0.78]
Mali	Iron [1.82]	Vit A [1.11]	Malaria [1.3]	Dermatitis [0.79]	Oth NTD [0.76]	Neonatal [0.93]	Diarrhea [0.91]	Congenital [0.94]	Skin Fung [1.44]	Hemog [1.19]
Mauritania	Iron [1.41]	Vit A [1.25]	Neonatal [1.24]	Dermatitis [0.95]	Asthma [0.97]	Diarrhea [1.28]	Epilepsy [1.29]	Congenital [0.97]	Conduct [0.94]	Skin Viral [0.9]
Niger	Iron [1.3]	Vit A [0.7]	Malaria [1.04]	Dermatitis [0.94]	Asthma [0.82]	Hemog [1.13]	Congenital [0.9]	Diarrhea [0.81]	Neonatal [0.98]	Epilepsy [0.83]
Nigeria	Iron [2.58]	Vit A [1.56]	Hemog [3.49]	Dermatitis [0.83]	Congenital [1.41]	Malaria [34.81]	Asthma [0.62]	Epilepsy [1.14]	Neonatal [0.49]	Oth NTD [1.71]
Sao Tome Principe	Vit A [1.53]	Iron [1.23]	Neonatal [1.24]	Dermatitis [0.95]	Diarrhea [1.31]	Asthma [0.7]	Epilepsy [1.3]	Congenital [0.99]	Conduct [0.93]	Skin Viral [0.9]
Senegal	Iron [1.45]	Vit A [0.99]	Neonatal [1.54]	Dermatitis [0.95]	Diarrhea [1.26]	Epilepsy [1.15]	Congenital [0.98]	Asthma [0.63]	Conduct [0.96]	Skin Viral [0.92]
Sierra Leone	Iron [1.68]	Vit A [1.09]	Malaria [5.31]	Hemog [2.75]	Dermatitis [0.95]	Epilepsy [1.21]	Asthma [0.76]	Diarrhea [1.06]	Neonatal [0.76]	Oncho [60.2]
Togo	Vit A [1.15]	Iron [1.02]	Malaria [15.05]	Neonatal [1.12]	Dermatitis [0.95]	Asthma [0.84]	Epilepsy [1.27]	Diarrhea [1.17]	Hemog [1.93]	Congenital [0.99]

eFigure 9e. Leading ten causes of YLDs with the ratio of observed YLDs to YLDs expected on the basis of Socio-Demographic Index alone in 2017, 10-19 years, both sexes combined. The top ten causes contributing to YLDs are listed globally, by socio-demographic quintile, and then by GBD superregion, region, country, and subnationally where modeled. For each cell, the ratio of observed YLDs to YLDs expected on the basis of socio-demographic index (SDI) alone are listed. Abbreviations: YLD=year of life lived with disability, GBD=Global Burden of Disease.

Values shown in brackets represent the ratio of observed YLDs to predicted YLDs on the basis of Socio-Demographic Index (SDI), rounded to two (2) digits. Color ranges (shown below) were calculated to place a roughly equal number of cells into each bin.

COLOR KEY:	[0.0-0.77]	[0.77-0.91]	[0.91-0.97]	[0.97-1.02]	[1.02-1.1]	[1.1-1.25]	[1.25-1.48]	[1.48-1.87]	1.87+

	1	2	3	4	5	6	7	8	9	10
Global	Headaches [0.85]	Iron [1.82]	Neonatal [1.09]	Conduct [0.9]	Anxiety [0.88]	Depression [0.9]	Back Pain [0.82]	Dermatitis [0.82]	Congenital [0.99]	Vit A [2.88]
Low SDI	Iron [1.17]	Headaches [0.92]	Conduct [0.9]	Neonatal [1.43]	Anxiety [0.94]	Depression [0.92]	Back Pain [0.77]	Vit A [1.33]	Dermatitis [0.9]	Congenital [1.04]
Low-middle SDI	Iron [1.53]	Headaches [0.95]	Neonatal [1.15]	Conduct [0.89]	Anxiety [0.92]	Back Pain [0.91]	Depression [0.9]	Dermatitis [0.85]	Congenital [1.05]	Vit A [1.92]
Middle SDI	Headaches [0.81]	Neonatal [1.21]	Conduct [0.88]	Anxiety [0.78]	Back Pain [0.75]	Iron [1.1]	Depression [0.73]	Congenital [0.93]	Dermatitis [0.7]	Asthma [0.71]
High-middle SDI	Headaches [0.79]	Neonatal [1.25]	Back Pain [0.82]	Anxiety [0.76]	Conduct [0.95]	Depression [0.87]	Iron [2.32]	Dermatitis [0.63]	Congenital [0.91]	Asthma [0.65]
High SDI	Headaches [0.93]	Anxiety [1.16]	Depression [1.33]	Back Pain [1.05]	Conduct [0.98]	Drugs [3.14]	Neonatal [0.89]	Acne [1.63]	Dermatitis [1.12]	Asthma [0.97]
Central Europe, Eastern Europe, and Central Asia	Headaches [0.9]	Neonatal [1.38]	Conduct [1.03]	Back Pain [0.8]	Falls [1.9]	Anxiety [0.62]	Depression [0.71]	Iron [2.19]	Upper Digest [3.35]	Congenital [1.11]
Central Asia	Headaches [0.88]	Iron [2.39]	Conduct [0.98]	Neonatal [0.93]	Back Pain [0.74]	Depression [0.79]	Anxiety [0.59]	Falls [1.64]	Epilepsy [1.45]	Congenital [1.1]
Armenia	Headaches [0.86]	Conduct [1.0]	Neonatal [0.98]	Iron [1.82]	Back Pain [0.74]	Depression [0.71]	Anxiety [0.57]	Falls [1.47]	Congenital [1.11]	Epilepsy [1.32]
Azerbaijan	Headaches [0.87]	Conduct [1.0]	Neonatal [0.89]	Iron [1.74]	Back Pain [0.72]	Depression [0.66]	Anxiety [0.57]	Falls [1.48]	Congenital [1.07]	Epilepsy [1.43]
Georgia	Headaches [0.87]	Conduct [1.0]	Neonatal [0.97]	Depression [0.72]	Back Pain [0.64]	Anxiety [0.58]	Iron [1.27]	Congenital [1.13]	Epilepsy [1.36]	Falls [1.23]
Kazakhstan	Headaches [0.86]	Conduct [1.01]	Neonatal [1.01]	Depression [0.91]	Back Pain [0.75]	Iron [2.14]	Anxiety [0.56]	Falls [1.3]	Congenital [1.12]	Epilepsy [1.54]
Kyrgyzstan	Headaches [0.9]	Iron [1.86]	Conduct [0.97]	Neonatal [1.03]	Depression [0.84]	Back Pain [0.78]	Anxiety [0.64]	Falls [2.16]	Congenital [1.12]	Epilepsy [1.15]
Mongolia	Headaches [0.88]	Conduct [0.98]	Neonatal [0.98]	Depression [0.96]	Iron [1.27]	Back Pain [0.74]	Anxiety [0.61]	Falls [2.08]	Vit A [4.49]	Congenital [1.12]
Tajikistan	Headaches [0.93]	Conduct [0.97]	Iron [0.94]	Neonatal [1.04]	Back Pain [0.8]	Depression [0.71]	Anxiety [0.67]	Falls [2.82]	Epilepsy [1.26]	Congenital [1.12]
Turkmenistan	Headaches [0.87]	Conduct [0.99]	Iron [1.83]	Neonatal [0.83]	Back Pain [0.71]	Depression [0.76]	Anxiety [0.58]	Falls [1.56]	Congenital [1.1]	Epilepsy [1.45]
Uzbekistan	Iron [2.73]	Headaches [0.89]	Conduct [0.97]	Neonatal [0.91]	Back Pain [0.76]	Depression [0.81]	Anxiety [0.63]	Falls [1.92]	Epilepsy [1.41]	Congenital [1.08]
Central Europe	Headaches [0.87]	Neonatal [1.43]	Falls [2.28]	Back Pain [0.91]	Conduct [1.03]	Anxiety [0.69]	Depression [0.59]	Congenital [1.28]	Upper Digest [3.4]	Dermatitis [0.7]
Albania	Headaches [0.9]	Back Pain [1.13]	Neonatal [1.19]	Falls [3.35]	Conduct [0.97]	Anxiety [0.79]	Depression [0.65]	Congenital [1.23]	Iron [0.8]	Upper Digest [2.93]
Bosnia	Headaches [0.89]	Neonatal [1.42]	Falls [3.19]	Conduct [0.98]	Back Pain [0.92]	Anxiety [0.77]	Depression [0.66]	Congenital [1.24]	Upper Digest [2.57]	Iron [0.84]
Bulgaria	Headaches [0.87]	Neonatal [1.26]	Conduct [1.02]	Falls [2.35]	Back Pain [0.86]	Anxiety [0.74]	Depression [0.61]	Congenital [1.33]	Upper Digest [2.65]	Dermatitis [0.56]
Croatia	Headaches [0.88]	Neonatal [1.55]	Conduct [1.03]	Back Pain [0.89]	Anxiety [0.69]	Depression [0.71]	Falls [1.39]	Congenital [1.27]	Acne [0.69]	Dermatitis [0.6]
Czech	Headaches [0.87]	Falls [2.55]	Back Pain [1.01]	Conduct [1.06]	Neonatal [0.93]	Anxiety [0.67]	Depression [0.65]	Congenital [1.16]	Dermatitis [0.71]	Drugs [1.21]
Hungary	Headaches [0.88]	Neonatal [1.37]	Back Pain [1.01]	Falls [2.33]	Conduct [1.03]	Anxiety [0.69]	Depression [0.62]	Congenital [1.28]	Dermatitis [0.83]	Diarrhea [3.64]
Macedonia	Headaches [0.88]	Neonatal [1.38]	Falls [2.66]	Conduct [1.0]	Anxiety [0.73]	Back Pain [0.69]	Depression [0.61]	Iron [1.74]	Congenital [1.22]	Upper Digest [2.57]
Montenegro	Headaches [0.88]	Neonatal [1.35]	Back Pain [0.96]	Falls [2.44]	Conduct [1.02]	Anxiety [0.71]	Depression [0.64]	Congenital [1.24]	Upper Digest [2.71]	Dermatitis [0.61]
Poland	Headaches [0.88]	Neonatal [1.59]	Falls [2.11]	Conduct [1.04]	Back Pain [0.74]	Anxiety [0.68]	Depression [0.52]	Upper Digest [3.99]	Congenital [1.28]	Dermatitis [0.8]
Romania	Headaches [0.87]	Neonatal [1.47]	Back Pain [1.06]	Conduct [1.01]	Falls [2.37]	Iron [3.64]	Anxiety [0.67]	Upper Digest [4.92]	Depression [0.61]	Congenital [1.26]
Serbia	Neonatal [1.82]	Headaches [0.87]	Back Pain [1.0]	Falls [2.76]	Conduct [1.01]	Anxiety [0.73]	Depression [0.6]	Congenital [1.34]	Dermatitis [0.61]	Bipolar [0.78]
Slovakia	Headaches [0.88]	Back Pain [1.01]	Falls [2.13]	Conduct [1.04]	Neonatal [1.03]	Anxiety [0.68]	Depression [0.58]	Congenital [1.35]	Upper Digest [3.42]	Dermatitis [0.7]
Slovenia	Headaches [0.88]	Falls [2.44]	Back Pain [1.08]	Conduct [1.06]	Neonatal [1.09]	Anxiety [0.67]	Depression [0.68]	Congenital [1.32]	Upper Digest [3.41]	Acne [0.65]

eFigure 9e. Leading ten causes of YLDs with the ratio of observed YLDs to YLDs expected on the basis of Socio-Demographic Index alone in 2017, 10-19 years, both sexes combined. The top ten causes contributing to YLDs are listed globally, by socio-demographic quintile, and then by GBD superregion, region, country, and subnationally where modeled. For each cell, the ratio of observed YLDs to YLDs expected on the basis of socio-demographic index (SDI) alone are listed. Abbreviations: YLD=year of life lived with disability, GBD=Global Burden of Disease.

Values shown in brackets represent the ratio of observed YLDs to predicted YLDs on the basis of Socio-Demographic Index (SDI), rounded to two (2) digits. Color ranges (shown below) were calculated to place a roughly equal number of cells into each bin.

COLOR KEY:	[0.0-0.77]	(0.77-0.91]	(0.91-0.97]	(0.97-1.02]	(1.02-1.1]	(1.1-1.25]	(1.25-1.48]	(1.48-1.87]	1.87+

	1	2	3	4	5	6	7	8	9	10
**Eastern Europe**	Headaches [0.95]	Neonatal [1.65]	Conduct [1.08]	Back Pain [0.79]	Falls [1.82]	Depression [0.74]	Anxiety [0.6]	Upper Digest [5.16]	Congenital [1.04]	Dermatitis [0.68]
Belarus	Headaches [0.96]	Neonatal [1.66]	Conduct [1.01]	Back Pain [0.82]	Depression [0.82]	Falls [1.85]	Anxiety [0.62]	Upper Digest [4.97]	Dermatitis [0.77]	Congenital [1.03]
Estonia	Headaches [0.97]	Neonatal [1.72]	Conduct [1.07]	Back Pain [0.73]	Depression [0.83]	Falls [1.47]	Anxiety [0.57]	Upper Digest [5.65]	Dermatitis [1.03]	Congenital [1.06]
Latvia	Headaches [0.96]	Neonatal [1.57]	Conduct [1.04]	Back Pain [0.81]	Upper Digest [6.95]	Falls [1.6]	Anxiety [0.59]	Depression [0.68]	Dermatitis [0.72]	Congenital [0.98]
Lithuania	Headaches [1.05]	Neonatal [1.79]	Conduct [1.03]	Back Pain [0.78]	Depression [0.88]	Falls [1.57]	Anxiety [0.59]	Upper Digest [5.59]	Congenital [1.17]	Acne [0.65]
Moldova	Headaches [0.98]	Neonatal [1.79]	Conduct [0.98]	Upper Digest [6.7]	Back Pain [0.89]	Falls [2.65]	Depression [0.76]	Anxiety [0.69]	Iron [1.21]	Dermatitis [0.81]
Russian Federation	Headaches [0.94]	Neonatal [1.65]	Conduct [1.09]	Back Pain [0.73]	Falls [1.81]	Anxiety [0.6]	Depression [0.7]	Upper Digest [5.06]	Congenital [1.03]	Iron [1.7]
Ukraine	Headaches [0.97]	Neonatal [1.65]	Conduct [1.06]	Back Pain [0.98]	Depression [0.85]	Falls [2.06]	Upper Digest [5.21]	Anxiety [0.63]	Congenital [1.09]	Dermatitis [0.69]
**High-income**	Headaches [0.94]	Anxiety [1.1]	Depression [1.38]	Back Pain [1.03]	Conduct [1.03]	Drugs [2.89]	Neonatal [0.89]	Acne [1.26]	Dermatitis [1.14]	Asthma [0.98]
**Australasia**	Depression [1.62]	Anxiety [1.26]	Headaches [0.88]	Back Pain [1.22]	Falls [1.94]	Conduct [1.02]	Asthma [1.87]	Neonatal [0.89]	Acne [1.09]	Bipolar [1.48]
Australia	Depression [1.65]	Anxiety [1.21]	Headaches [0.88]	Back Pain [1.17]	Falls [1.87]	Asthma [1.95]	Conduct [1.0]	Neonatal [0.87]	Drugs [2.07]	Acne [1.08]
New Zealand	Anxiety [1.55]	Back Pain [1.51]	Headaches [0.89]	Depression [1.49]	Falls [2.34]	Conduct [1.09]	Neonatal [1.02]	Asthma [1.5]	Dermatitis [1.25]	Bipolar [1.48]
**High-income Asia Pacific**	Back Pain [1.19]	Headaches [0.75]	Neonatal [1.12]	Anxiety [0.78]	Conduct [0.99]	Depression [0.94]	Acne [1.26]	Falls [1.18]	Dermatitis [1.15]	Iron [2.79]
Brunei	Headaches [0.74]	Back Pain [0.96]	Neonatal [1.13]	Anxiety [0.8]	Conduct [0.93]	Depression [0.86]	Dermatitis [1.07]	Acne [1.08]	Falls [1.07]	Iron [2.28]
Japan	Back Pain [1.27]	Headaches [0.7]	Neonatal [1.11]	Conduct [1.03]	Anxiety [0.77]	Depression [0.92]	Falls [1.31]	Acne [1.27]	Dermatitis [1.21]	Iron [3.33]
Aichi	Back Pain [1.26]	Neonatal [1.13]	Conduct [1.04]	Anxiety [0.76]	Headaches [0.56]	Depression [0.92]	Falls [1.27]	Acne [1.24]	Dermatitis [1.22]	Iron [3.11]
Akita	Back Pain [1.29]	Headaches [0.71]	Neonatal [1.22]	Conduct [1.01]	Depression [0.98]	Anxiety [0.79]	Falls [1.42]	Dermatitis [1.22]	Acne [1.36]	Iron [2.56]
Aomori	Back Pain [1.29]	Headaches [0.71]	Neonatal [1.14]	Conduct [1.0]	Anxiety [0.79]	Depression [0.95]	Falls [1.44]	Dermatitis [1.22]	Acne [1.35]	Iron [3.03]
Chiba	Back Pain [1.26]	Headaches [0.71]	Conduct [1.03]	Neonatal [1.1]	Anxiety [0.77]	Depression [0.92]	Falls [1.32]	Acne [1.28]	Dermatitis [1.22]	Iron [3.33]
Ehime	Back Pain [1.25]	Headaches [0.71]	Conduct [1.02]	Anxiety [0.78]	Depression [0.91]	Neonatal [0.93]	Falls [1.37]	Dermatitis [1.22]	Acne [1.31]	Iron [3.01]
Fukui	Back Pain [1.26]	Headaches [0.71]	Conduct [1.02]	Neonatal [1.1]	Anxiety [0.77]	Depression [0.89]	Falls [1.34]	Acne [1.31]	Dermatitis [1.22]	Iron [3.31]
Fukuoka	Back Pain [1.26]	Headaches [0.71]	Conduct [1.03]	Neonatal [1.09]	Anxiety [0.77]	Depression [0.91]	Falls [1.34]	Acne [1.3]	Dermatitis [1.21]	Iron [3.75]
Fukushima	Back Pain [1.28]	Headaches [0.7]	Conduct [1.01]	Neonatal [1.05]	Anxiety [0.79]	Depression [0.92]	Falls [1.42]	Dermatitis [1.22]	Acne [1.34]	Iron [2.82]
Gifu	Back Pain [1.29]	Headaches [0.71]	Neonatal [1.14]	Conduct [1.02]	Anxiety [0.78]	Depression [0.78]	Falls [1.35]	Dermatitis [1.22]	Acne [1.3]	Iron [3.48]
Gunma	Back Pain [1.28]	Headaches [0.71]	Conduct [1.02]	Neonatal [1.08]	Anxiety [0.78]	Depression [0.95]	Falls [1.36]	Acne [1.3]	Dermatitis [1.21]	Iron [3.95]
Hiroshima	Back Pain [1.24]	Headaches [0.71]	Neonatal [1.15]	Conduct [1.04]	Anxiety [0.76]	Depression [0.88]	Falls [1.29]	Acne [1.26]	Dermatitis [1.22]	Iron [3.68]
Hokkaido	Back Pain [1.28]	Headaches [0.71]	Conduct [1.01]	Neonatal [1.08]	Anxiety [0.78]	Depression [0.93]	Falls [1.39]	Dermatitis [1.21]	Acne [1.33]	Iron [3.66]
Hyogo	Back Pain [1.26]	Headaches [0.72]	Neonatal [1.12]	Conduct [1.03]	Anxiety [0.77]	Depression [0.94]	Falls [1.31]	Acne [1.29]	Dermatitis [1.22]	Iron [3.08]
Ibaraki	Back Pain [1.27]	Headaches [0.62]	Neonatal [1.16]	Conduct [1.03]	Anxiety [0.77]	Depression [0.93]	Falls [1.34]	Acne [1.31]	Dermatitis [1.21]	Iron [2.52]
Ishikawa	Back Pain [1.26]	Headaches [0.72]	Conduct [1.02]	Neonatal [1.08]	Anxiety [0.77]	Depression [0.9]	Falls [1.34]	Acne [1.31]	Dermatitis [1.21]	Iron [3.22]
Iwate	Back Pain [1.29]	Headaches [0.71]	Neonatal [1.17]	Conduct [1.01]	Anxiety [0.79]	Depression [0.96]	Falls [1.43]	Dermatitis [1.22]	Acne [1.35]	Iron [2.93]

eFigure 9e. Leading ten causes of YLDs with the ratio of observed YLDs to YLDs expected on the basis of Socio-Demographic Index alone in 2017, 10-19 years, both sexes combined. The top ten causes contributing to YLDs are listed globally, by socio-demographic quintile, and then by GBD superregion, region, country, and subnationally where modeled. For each cell, the ratio of observed YLDs to YLDs expected on the basis of socio-demographic index (SDI) alone are listed. Abbreviations: YLD=year of life lived with disability, GBD=Global Burden of Disease.

Values shown in brackets represent the ratio of observed YLDs to predicted YLDs on the basis of Socio-Demographic Index (SDI), rounded to two (2) digits. Color ranges (shown below) were calculated to place a roughly equal number of cells into each bin.

COLOR KEY:		[0.0-0.77]	(0.77-0.91]	(0.91-0.97]	(0.97-1.02]	(1.02-1.1]	(1.1-1.25]	(1.25-1.48]	(1.48-1.87]	1.87+
	1	2	3	4	5	6	7	8	9	10
Kagawa	Back Pain [1.24]	Headaches [0.71]	Conduct [1.02]	Neonatal [1.06]	Anxiety [0.78]	Depression [0.88]	Falls [1.33]	Dermatitis [1.22]	Acne [1.3]	Iron [3.37]
Kagoshima	Back Pain [1.25]	Headaches [0.7]	Conduct [1.02]	Anxiety [0.79]	Neonatal [0.97]	Depression [0.78]	Falls [1.41]	Dermatitis [1.23]	Acne [1.31]	Iron [2.56]
Kanagawa	Back Pain [1.26]	Headaches [0.72]	Neonatal [1.15]	Conduct [1.04]	Anxiety [0.76]	Depression [0.95]	Falls [1.3]	Acne [1.26]	Dermatitis [1.21]	Iron [3.01]
Kochi	Back Pain [1.27]	Headaches [0.71]	Conduct [1.01]	Neonatal [1.04]	Anxiety [0.79]	Depression [0.9]	Iron [3.9]	Dermatitis [1.22]	Falls [1.41]	Acne [1.35]
Kumamoto	Back Pain [1.25]	Headaches [0.7]	Conduct [1.02]	Anxiety [0.79]	Neonatal [1.02]	Depression [0.89]	Falls [1.42]	Dermatitis [1.22]	Acne [1.34]	Iron [3.14]
Kyoto	Back Pain [1.29]	Headaches [0.73]	Neonatal [1.15]	Conduct [1.03]	Anxiety [0.76]	Depression [0.94]	Falls [1.29]	Acne [1.27]	Dermatitis [0.91]	Iron [3.35]
Mie	Back Pain [1.25]	Headaches [0.71]	Neonatal [1.14]	Conduct [1.03]	Anxiety [0.77]	Depression [0.89]	Falls [1.32]	Acne [1.29]	Dermatitis [1.22]	Iron [4.11]
Miyagi	Back Pain [1.29]	Headaches [0.72]	Conduct [1.02]	Neonatal [1.06]	Anxiety [0.78]	Depression [0.97]	Falls [1.37]	Acne [1.34]	Dermatitis [1.21]	Iron [3.2]
Miyazaki	Back Pain [1.26]	Headaches [0.7]	Conduct [1.01]	Anxiety [0.79]	Neonatal [1.02]	Depression [0.91]	Iron [4.08]	Falls [1.43]	Dermatitis [1.22]	Acne [1.34]
Nagano	Back Pain [1.26]	Headaches [0.71]	Neonatal [1.17]	Conduct [1.03]	Anxiety [0.77]	Depression [0.9]	Falls [1.35]	Acne [1.31]	Dermatitis [1.22]	Iron [3.8]
Nagasaki	Back Pain [1.25]	Headaches [0.71]	Conduct [1.01]	Anxiety [0.79]	Neonatal [1.02]	Depression [0.89]	Falls [1.44]	Dermatitis [1.22]	Acne [1.35]	Iron [3.42]
Nara	Back Pain [1.31]	Headaches [0.72]	Conduct [1.02]	Anxiety [0.78]	Neonatal [1.0]	Depression [0.91]	Falls [1.37]	Acne [1.31]	Dermatitis [1.21]	Iron [3.1]
Niigata	Back Pain [1.43]	Headaches [0.71]	Neonatal [1.13]	Conduct [1.02]	Anxiety [0.78]	Depression [0.97]	Falls [1.37]	Acne [1.33]	Dermatitis [1.22]	Iron [2.89]
Oita	Back Pain [1.25]	Headaches [0.71]	Neonatal [1.17]	Conduct [1.02]	Anxiety [0.78]	Depression [0.91]	Falls [1.36]	Dermatitis [1.22]	Acne [1.32]	Iron [3.49]
Okayama	Back Pain [1.27]	Headaches [0.72]	Neonatal [1.18]	Conduct [1.03]	Anxiety [0.77]	Depression [0.9]	Falls [1.33]	Acne [1.3]	Dermatitis [1.21]	Asthma [0.68]
Okinawa	Back Pain [1.25]	Headaches [0.7]	Conduct [1.01]	Neonatal [1.08]	Anxiety [0.79]	Depression [0.88]	Falls [1.47]	Dermatitis [1.22]	Acne [1.34]	Iron [3.46]
Osaka	Back Pain [1.28]	Headaches [0.72]	Conduct [1.03]	Anxiety [0.76]	Neonatal [1.05]	Depression [0.96]	Falls [1.31]	Acne [1.26]	Dermatitis [1.21]	Congenital [1.07]
Saga	Back Pain [1.26]	Headaches [0.71]	Conduct [1.01]	Neonatal [1.06]	Anxiety [0.79]	Depression [0.88]	Falls [1.39]	Dermatitis [1.22]	Acne [1.33]	Iron [3.3]
Saitama	Back Pain [1.31]	Headaches [0.71]	Conduct [1.02]	Neonatal [1.09]	Anxiety [0.78]	Depression [0.95]	Falls [1.38]	Acne [1.31]	Dermatitis [1.21]	Iron [3.41]
Shiga	Back Pain [1.26]	Headaches [0.71]	Conduct [1.04]	Anxiety [0.76]	Neonatal [1.04]	Depression [0.89]	Dermatitis [1.5]	Falls [1.29]	Acne [1.26]	Iron [3.47]
Shimane	Back Pain [1.18]	Headaches [0.7]	Conduct [1.02]	Neonatal [1.05]	Anxiety [0.79]	Depression [0.92]	Falls [1.41]	Dermatitis [1.21]	Acne [1.33]	Iron [3.08]
Shizuoka	Back Pain [1.24]	Headaches [0.71]	Neonatal [1.22]	Conduct [1.04]	Anxiety [0.77]	Depression [0.9]	Falls [1.3]	Acne [1.28]	Dermatitis [1.22]	Iron [3.65]
Tochigi	Back Pain [1.27]	Headaches [0.71]	Neonatal [1.17]	Conduct [1.03]	Anxiety [0.77]	Depression [0.9]	Falls [1.32]	Dermatitis [1.22]	Acne [1.29]	Congenital [1.36]
Tokushima	Back Pain [1.26]	Headaches [0.72]	Neonatal [1.11]	Conduct [1.01]	Anxiety [0.78]	Depression [0.91]	Falls [1.35]	Dermatitis [1.22]	Acne [1.3]	Iron [2.88]
Tokyo	Back Pain [1.24]	Headaches [0.73]	Neonatal [1.15]	Conduct [1.08]	Anxiety [0.73]	Depression [0.88]	Acne [1.16]	Falls [1.15]	Dermatitis [1.23]	Congenital [1.19]
Tottori	Back Pain [1.26]	Headaches [0.66]	Neonatal [1.13]	Conduct [1.01]	Anxiety [0.79]	Depression [0.91]	Falls [1.39]	Dermatitis [1.22]	Acne [1.33]	Iron [2.68]
Toyama	Back Pain [1.25]	Headaches [0.71]	Conduct [1.04]	Neonatal [1.07]	Anxiety [0.77]	Depression [0.91]	Falls [1.3]	Acne [1.28]	Dermatitis [1.22]	Iron [4.23]
Wakayama	Back Pain [1.44]	Headaches [0.71]	Neonatal [1.17]	Conduct [1.02]	Anxiety [0.78]	Depression [0.92]	Falls [1.38]	Dermatitis [1.22]	Acne [1.31]	Iron [3.13]
Yamagata	Back Pain [1.28]	Headaches [0.71]	Conduct [1.01]	Anxiety [0.79]	Neonatal [0.96]	Depression [0.88]	Falls [1.44]	Dermatitis [1.22]	Acne [1.35]	Iron [3.56]
Yamaguchi	Back Pain [1.25]	Headaches [0.71]	Conduct [1.02]	Anxiety [0.78]	Neonatal [0.98]	Depression [0.91]	Falls [1.33]	Acne [1.31]	Dermatitis [1.22]	Iron [3.22]
Yamanashi	Back Pain [1.28]	Headaches [0.71]	Neonatal [1.23]	Conduct [1.03]	Anxiety [0.77]	Depression [0.91]	Falls [1.33]	Acne [1.29]	Dermatitis [1.2]	Iron [3.84]

eFigure 9e. Leading ten causes of YLDs with the ratio of observed YLDs to YLDs expected on the basis of Socio-Demographic Index alone in 2017, 10-19 years, both sexes combined. The top ten causes contributing to YLDs are listed globally, by socio-demographic quintile, and then by GBD superregion, region, country, and subnationally where modeled. For each cell, the ratio of observed YLDs to YLDs expected on the basis of socio-demographic index (SDI) alone are listed. Abbreviations: YLD=year of life lived with disability, GBD=Global Burden of Disease.

Values shown in brackets represent the ratio of observed YLDs to predicted YLDs on the basis of Socio-Demographic Index (SDI), rounded to two (2) digits. Color ranges (shown below) were calculated to place a roughly equal number of cells into each bin.

COLOR KEY:	[0.0-0.77]	(0.77-0.91]	(0.91-0.97]	(0.97-1.02]	(1.02-1.1]	(1.1-1.25]	(1.25-1.48]	(1.48-1.87]	1.87+

	1	2	3	4	5	6	7	8	9	10
S Korea	Headaches [0.86]	Back Pain [1.06]	Neonatal [1.14]	Anxiety [0.83]	Depression [0.97]	Conduct [0.89]	Acne [1.29]	Dermatitis [1.03]	Falls [0.97]	Drugs [1.22]
Singapore	Headaches [0.72]	Depression [1.24]	Back Pain [0.91]	Neonatal [1.14]	Anxiety [0.82]	Conduct [0.89]	Acne [1.23]	Falls [1.03]	Dermatitis [0.95]	Drugs [1.25]
**High-income North America**	Depression [1.83]	Headaches [1.02]	Drugs [5.6]	Anxiety [1.02]	Conduct [1.2]	Back Pain [0.83]	Neonatal [0.86]	Acne [1.21]	Dermatitis [1.18]	Oth MSK [5.79]
Canada	Headaches [1.04]	Drugs [5.09]	Depression [1.36]	Back Pain [0.93]	Anxiety [0.83]	Conduct [1.02]	Acne [1.25]	Dermatitis [1.22]	Neonatal [0.6]	Asthma [0.99]
Greenland	Depression [2.51]	Headaches [1.01]	Drugs [4.51]	Anxiety [1.04]	Conduct [0.98]	Back Pain [0.79]	Dermatitis [1.17]	Neonatal [0.59]	Asthma [1.1]	Acne [1.28]
USA	Depression [1.87]	Drugs [5.66]	Headaches [1.01]	Anxiety [1.05]	Conduct [1.21]	Back Pain [0.82]	Neonatal [0.88]	Oth MSK [5.98]	Acne [1.21]	Dermatitis [1.17]
Alabama	Drugs [6.86]	Depression [1.84]	Headaches [1.02]	Anxiety [1.08]	Back Pain [1.05]	Conduct [1.17]	Neonatal [0.87]	Oth MSK [6.4]	Dermatitis [1.02]	Acne [1.08]
Alaska	Depression [1.87]	Drugs [5.81]	Headaches [1.01]	Anxiety [1.05]	Conduct [1.21]	Back Pain [0.93]	Neonatal [0.84]	Oth MSK [6.43]	Dermatitis [1.0]	Acne [0.85]
Arizona	Drugs [7.34]	Depression [2.13]	Headaches [1.01]	Anxiety [1.07]	Conduct [1.18]	Back Pain [0.9]	Neonatal [0.88]	Dermatitis [1.19]	Oth MSK [6.22]	Acne [1.23]
Arkansas	Depression [2.15]	Drugs [6.93]	Headaches [1.02]	Anxiety [1.09]	Conduct [1.17]	Back Pain [0.82]	Neonatal [0.77]	Dermatitis [0.94]	Oth MSK [4.93]	Acne [0.98]
California	Depression [1.83]	Headaches [1.02]	Drugs [4.25]	Anxiety [1.02]	Conduct [1.21]	Back Pain [0.7]	Dermatitis [1.31]	Acne [1.3]	Neonatal [0.73]	Oth MSK [5.39]
Colorado	Depression [2.07]	Headaches [1.02]	Drugs [4.84]	Anxiety [1.04]	Conduct [1.22]	Back Pain [0.83]	Neonatal [0.89]	Oth MSK [6.29]	Acne [1.13]	Dermatitis [1.03]
Connecticut	Drugs [7.08]	Headaches [1.04]	Depression [1.67]	Anxiety [1.02]	Conduct [1.23]	Neonatal [1.03]	Back Pain [0.7]	Dermatitis [1.37]	Acne [1.27]	Oth MSK [6.07]
Delaware	Drugs [7.67]	Depression [1.97]	Headaches [1.03]	Anxiety [1.05]	Conduct [1.2]	Back Pain [0.72]	Neonatal [0.85]	Oth MSK [5.99]	Dermatitis [1.06]	Acne [1.04]
DC	Drugs [5.92]	Headaches [1.06]	Depression [1.63]	Anxiety [1.06]	Conduct [1.2]	Dermatitis [1.53]	Acne [1.45]	Neonatal [0.89]	Back Pain [0.52]	Oth MSK [4.76]
Florida	Drugs [5.83]	Depression [1.83]	Headaches [1.03]	Anxiety [1.06]	Conduct [1.2]	Neonatal [0.98]	Back Pain [0.73]	Acne [1.24]	Dermatitis [1.14]	Oth MSK [4.74]
Georgia	Headaches [1.02]	Depression [1.77]	Drugs [5.37]	Anxiety [1.07]	Conduct [1.19]	Back Pain [0.8]	Neonatal [0.81]	Dermatitis [1.26]	Acne [1.28]	Oth MSK [5.7]
Hawaii	Depression [1.85]	Headaches [1.02]	Anxiety [1.04]	Conduct [1.21]	Drugs [2.92]	Dermatitis [1.41]	Neonatal [0.81]	Acne [1.32]	Back Pain [0.58]	Oth MSK [4.42]
Idaho	Depression [2.07]	Headaches [1.01]	Drugs [5.5]	Anxiety [1.07]	Conduct [1.19]	Back Pain [0.84]	Oth MSK [6.93]	Neonatal [0.73]	Acne [1.14]	Dermatitis [0.95]
Illinois	Headaches [1.03]	Depression [1.7]	Drugs [4.84]	Anxiety [1.04]	Conduct [1.21]	Back Pain [0.9]	Neonatal [1.03]	Oth MSK [6.49]	Acne [1.2]	Dermatitis [1.03]
Indiana	Drugs [6.67]	Depression [1.98]	Headaches [1.02]	Anxiety [1.07]	Conduct [1.18]	Back Pain [0.96]	Neonatal [0.84]	Oth MSK [6.68]	Dermatitis [1.04]	Acne [1.11]
Iowa	Headaches [1.02]	Depression [1.78]	Back Pain [1.43]	Anxiety [1.05]	Conduct [1.2]	Oth MSK [7.81]	Drugs [2.84]	Neonatal [0.85]	Acne [1.09]	Dermatitis [0.95]
Kansas	Depression [1.89]	Headaches [1.02]	Back Pain [1.13]	Anxiety [1.05]	Conduct [1.2]	Drugs [3.56]	Neonatal [0.9]	Oth MSK [6.45]	Acne [1.06]	Dermatitis [0.99]
Kentucky	Drugs [10.19]	Depression [1.99]	Headaches [1.01]	Anxiety [1.08]	Back Pain [1.09]	Conduct [1.17]	Neonatal [0.86]	Oth MSK [7.19]	Acne [1.33]	Dermatitis [1.05]
Louisiana	Drugs [7.58]	Headaches [1.02]	Depression [1.78]	Anxiety [1.08]	Conduct [1.18]	Back Pain [0.87]	Neonatal [0.79]	Acne [1.39]	Dermatitis [1.11]	Oth MSK [5.93]
Maine	Drugs [7.37]	Depression [1.95]	Headaches [1.02]	Anxiety [1.05]	Conduct [1.2]	Back Pain [0.89]	Oth MSK [8.35]	Neonatal [0.88]	Asthma [1.03]	Acne [0.91]
Maryland	Depression [1.78]	Headaches [1.03]	Drugs [4.57]	Anxiety [1.03]	Conduct [1.23]	Neonatal [1.02]	Back Pain [0.66]	Dermatitis [1.33]	Acne [1.16]	Oth MSK [5.14]
Massachusetts	Drugs [7.04]	Depression [1.81]	Headaches [1.05]	Anxiety [1.02]	Conduct [1.24]	Neonatal [0.98]	Back Pain [0.68]	Oth MSK [5.94]	Acne [1.15]	Dermatitis [1.23]
Michigan	Drugs [6.32]	Depression [1.93]	Headaches [0.83]	Anxiety [1.05]	Conduct [1.2]	Back Pain [0.79]	Neonatal [0.87]	Oth MSK [6.61]	Acne [1.23]	Dermatitis [1.08]
Minnesota	Depression [1.83]	Headaches [1.03]	Anxiety [1.03]	Conduct [1.23]	Drugs [2.85]	Back Pain [0.72]	Neonatal [0.92]	Oth MSK [6.05]	Acne [1.07]	Dermatitis [0.92]
Mississippi	Drugs [6.4]	Headaches [1.02]	Depression [1.8]	Anxiety [1.1]	Conduct [1.17]	Back Pain [0.74]	Neonatal [0.84]	Dermatitis [0.93]	Oth MSK [4.75]	Acne [0.93]

eFigure 9e. Leading ten causes of YLDs with the ratio of observed YLDs to YLDs expected on the basis of Socio-Demographic Index alone in 2017, 10-19 years, both sexes combined. The top ten causes contributing to YLDs are listed globally, by socio-demographic quintile, and then by GBD superregion, region, country, and subnationally where modeled. For each cell, the ratio of observed YLDs to YLDs expected on the basis of socio-demographic index (SDI) alone are listed. Abbreviations: YLD=year of life lived with disability, GBD=Global Burden of Disease.

Values shown in brackets represent the ratio of observed YLDs to predicted YLDs on the basis of Socio-Demographic Index (SDI), rounded to two (2) digits. Color ranges (shown below) were calculated to place a roughly equal number of cells into each bin.

COLOR KEY:	[0.0-0.77]	[0.77-0.91]	[0.91-0.97]	[0.97-1.02]	[1.02-1.1]	[1.1-1.25]	[1.25-1.48]	[1.48-1.87]	1.87+

	1	2	3	4	5	6	7	8	9	10
Missouri	Depression [2.01]	Drugs [5.75]	Headaches [1.02]	Anxiety [1.06]	Conduct [1.19]	Back Pain [0.9]	Neonatal [0.9]	Oth MSK [6.69]	Acne [1.14]	Dermatitis [0.98]
Montana	Depression [1.97]	Headaches [1.01]	Drugs [5.31]	Anxiety [1.05]	Conduct [1.21]	Back Pain [0.94]	Oth MSK [7.42]	Neonatal [0.85]	Acne [1.0]	Dermatitis [0.9]
Nebraska	Headaches [1.02]	Depression [1.76]	Back Pain [1.22]	Anxiety [1.04]	Conduct [1.22]	Drugs [3.0]	Neonatal [0.85]	Oth MSK [6.02]	Acne [1.08]	Dermatitis [0.94]
Nevada	Drugs [7.34]	Depression [1.96]	Headaches [1.01]	Anxiety [1.07]	Conduct [1.19]	Back Pain [0.71]	Neonatal [0.83]	Dermatitis [1.11]	Oth MSK [5.5]	Acne [1.03]
New Hampshire	Drugs [7.97]	Depression [2.03]	Headaches [1.04]	Anxiety [1.02]	Conduct [1.23]	Back Pain [0.83]	Oth MSK [6.5]	Neonatal [0.83]	Dermatitis [0.97]	Acne [0.91]
New Jersey	Drugs [5.52]	Headaches [1.03]	Depression [1.57]	Anxiety [1.02]	Conduct [1.23]	Back Pain [0.77]	Neonatal [0.93]	Dermatitis [1.43]	Acne [1.3]	Oth MSK [5.66]
New Mexico	Depression [2.14]	Drugs [6.46]	Headaches [1.01]	Anxiety [1.08]	Conduct [1.18]	Back Pain [0.96]	Neonatal [0.93]	Oth MSK [6.61]	Acne [1.14]	Dermatitis [1.01]
New York	Depression [1.94]	Headaches [1.03]	Drugs [5.1]	Anxiety [1.03]	Conduct [1.22]	Neonatal [0.97]	Acne [1.48]	Dermatitis [1.5]	Back Pain [0.69]	Oth MSK [6.16]
N Carolina	Depression [1.83]	Headaches [1.02]	Drugs [5.72]	Anxiety [1.0]	Conduct [1.11]	Back Pain [0.72]	Neonatal [0.89]	Acne [1.22]	Dermatitis [1.09]	Oth MSK [5.4]
N Dakota	Headaches [1.02]	Depression [1.7]	Anxiety [1.04]	Back Pain [1.02]	Conduct [1.22]	Oth MSK [9.65]	Drugs [2.79]	Neonatal [0.92]	Acne [1.01]	Dermatitis [0.86]
Ohio	Drugs [8.17]	Depression [1.9]	Headaches [1.02]	Anxiety [1.06]	Conduct [1.19]	Back Pain [0.87]	Neonatal [0.83]	Oth MSK [6.53]	Dermatitis [1.13]	Acne [1.14]
Oklahoma	Drugs [7.79]	Depression [1.96]	Headaches [1.01]	Anxiety [1.07]	Conduct [1.18]	Back Pain [0.98]	Oth MSK [7.18]	Neonatal [0.79]	Dermatitis [1.17]	Acne [1.09]
Oregon	Drugs [7.22]	Depression [2.05]	Headaches [1.02]	Anxiety [0.97]	Conduct [1.1]	Back Pain [0.76]	Oth MSK [6.67]	Dermatitis [1.22]	Neonatal [0.76]	Acne [1.08]
Pennsylvania	Drugs [7.93]	Depression [1.84]	Headaches [0.93]	Anxiety [1.04]	Back Pain [1.04]	Conduct [1.21]	Oth MSK [7.09]	Neonatal [0.87]	Acne [1.2]	Dermatitis [1.19]
Rhode Island	Drugs [8.95]	Depression [2.02]	Headaches [1.04]	Anxiety [1.04]	Conduct [1.21]	Back Pain [0.68]	Neonatal [0.84]	Oth MSK [5.74]	Acne [1.13]	Dermatitis [1.08]
S Carolina	Drugs [6.93]	Depression [1.91]	Headaches [1.02]	Anxiety [1.07]	Conduct [1.18]	Back Pain [0.86]	Neonatal [0.83]	Dermatitis [1.26]	Oth MSK [5.96]	Acne [1.21]
S Dakota	Headaches [1.01]	Depression [1.65]	Back Pain [1.23]	Anxiety [1.05]	Conduct [1.21]	Oth MSK [8.25]	Drugs [2.82]	Neonatal [0.75]	Acne [1.12]	Dermatitis [0.88]
Tennessee	Drugs [8.23]	Depression [1.95]	Headaches [1.02]	Anxiety [1.08]	Conduct [1.17]	Back Pain [0.85]	Neonatal [0.83]	Oth MSK [6.0]	Dermatitis [1.07]	Acne [1.12]
Texas	Depression [1.81]	Headaches [1.01]	Anxiety [1.07]	Conduct [1.33]	Drugs [3.63]	Back Pain [0.8]	Neonatal [0.96]	Dermatitis [1.19]	Acne [1.27]	Oth MSK [5.46]
Utah	Depression [2.24]	Drugs [6.51]	Headaches [1.01]	Anxiety [1.06]	Conduct [1.19]	Back Pain [0.83]	Neonatal [0.81]	Acne [1.18]	Oth MSK [5.72]	Dermatitis [0.95]
Vermont	Drugs [5.98]	Depression [1.89]	Headaches [1.04]	Anxiety [1.04]	Conduct [1.23]	Back Pain [0.77]	Neonatal [0.93]	Oth MSK [5.92]	Dermatitis [0.92]	Acne [0.83]
Virginia	Depression [1.98]	Headaches [1.03]	Drugs [4.7]	Anxiety [1.04]	Conduct [1.22]	Back Pain [0.75]	Neonatal [0.95]	Dermatitis [1.33]	Acne [1.11]	Oth MSK [5.4]
Washington	Depression [2.03]	Drugs [5.64]	Headaches [1.02]	Anxiety [1.03]	Conduct [1.22]	Back Pain [0.83]	Oth MSK [7.33]	Neonatal [0.81]	Dermatitis [1.18]	Acne [1.12]
W Virginia	Drugs [12.25]	Depression [2.0]	Headaches [1.01]	Anxiety [1.09]	Conduct [1.16]	Back Pain [0.92]	Neonatal [0.83]	Oth MSK [6.1]	Dermatitis [1.05]	Acne [1.18]
Wisconsin	Depression [1.97]	Drugs [5.96]	Headaches [1.02]	Anxiety [1.04]	Conduct [1.21]	Back Pain [0.94]	Neonatal [0.93]	Oth MSK [6.17]	Acne [1.06]	Dermatitis [0.92]
Wyoming	Depression [2.08]	Headaches [1.01]	Back Pain [1.15]	Anxiety [1.04]	Conduct [1.22]	Drugs [3.5]	Oth MSK [6.89]	Neonatal [0.71]	Dermatitis [0.92]	Acne [0.75]
**Southern Latin America**	Anxiety [1.36]	Back Pain [1.36]	Depression [1.38]	Headaches [0.66]	Conduct [0.93]	Neonatal [0.99]	Dermatitis [1.02]	Drugs [1.82]	Acne [1.31]	Asthma [0.9]
Argentina	Anxiety [1.38]	Back Pain [1.23]	Depression [1.35]	Headaches [0.63]	Conduct [0.93]	Neonatal [0.97]	Dermatitis [0.95]	Drugs [1.87]	Acne [1.3]	Asthma [0.89]
Chile	Back Pain [1.8]	Anxiety [1.32]	Depression [1.51]	Headaches [0.71]	Conduct [0.92]	Neonatal [1.01]	Dermatitis [1.22]	Acne [1.33]	Drugs [1.73]	Asthma [0.89]
Uruguay	Anxiety [1.39]	Headaches [0.67]	Back Pain [1.08]	Depression [1.12]	Conduct [0.93]	Neonatal [1.03]	Dermatitis [0.93]	Asthma [1.05]	Iron [1.1]	Acne [1.37]
**Western Europe**	Headaches [1.0]	Anxiety [1.24]	Back Pain [1.1]	Depression [1.09]	Conduct [0.91]	Neonatal [0.81]	Acne [1.34]	Dermatitis [1.13]	Asthma [1.11]	Falls [0.94]

eFigure 9e. Leading ten causes of YLDs with the ratio of observed YLDs to YLDs expected on the basis of Socio-Demographic Index alone in 2017, 10-19 years, both sexes combined. The top ten causes contributing to YLDs are listed globally, by socio-demographic quintile, and then by GBD superregion, region, country, and subnationally where modeled. For each cell, the ratio of observed YLDs to YLDs expected on the basis of socio-demographic index (SDI) alone are listed. Abbreviations: YLD=year of life lived with disability, GBD=Global Burden of Disease.

Values shown in brackets represent the ratio of observed YLDs to predicted YLDs on the basis of Socio-Demographic Index (SDI), rounded to two (2) digits. Color ranges (shown below) were calculated to place a roughly equal number of cells into each bin.

COLOR KEY:	[0.0-0.77]	(0.77-0.91]	(0.91-0.97]	(0.97-1.02]	(1.02-1.1]	(1.1-1.25]	(1.25-1.48]	(1.48-1.87]	1.87+

	1	2	3	4	5	6	7	8	9	10
Andorra	Headaches [1.02]	Anxiety [1.14]	Back Pain [1.06]	Depression [1.05]	Conduct [0.92]	Dermatitis [1.4]	Acne [1.27]	Neonatal [0.84]	Asthma [1.19]	Falls [0.86]
Austria	Headaches [1.04]	Anxiety [1.17]	Neonatal [1.36]	Back Pain [0.86]	Conduct [0.89]	Depression [0.77]	Acne [1.35]	Falls [0.95]	Asthma [0.96]	Bipolar [1.11]
Belgium	Headaches [1.25]	Anxiety [1.13]	Back Pain [0.94]	Depression [1.05]	Conduct [0.91]	Acne [1.14]	Falls [1.02]	Dermatitis [0.95]	Neonatal [0.55]	Bipolar [1.09]
Cyprus	Headaches [1.02]	Anxiety [1.18]	Back Pain [1.13]	Neonatal [1.09]	Depression [1.0]	Conduct [0.89]	Acne [1.29]	Falls [0.92]	Asthma [0.98]	Bipolar [1.12]
Denmark	Back Pain [1.38]	Anxiety [1.13]	Headaches [0.85]	Conduct [0.91]	Dermatitis [1.45]	Depression [0.79]	Acne [1.2]	Neonatal [0.66]	Asthma [1.05]	Falls [0.82]
Finland	Headaches [1.01]	Neonatal [1.7]	Depression [1.37]	Anxiety [0.83]	Conduct [0.9]	Dermatitis [1.48]	Back Pain [0.62]	Acne [1.27]	Falls [0.98]	Asthma [1.1]
France	Anxiety [1.41]	Headaches [0.83]	Back Pain [1.08]	Depression [1.12]	Conduct [0.9]	Acne [1.48]	Dermatitis [1.31]	Neonatal [0.64]	Asthma [1.09]	Falls [0.94]
Germany	Anxiety [1.4]	Back Pain [1.38]	Headaches [0.87]	Depression [1.04]	Conduct [0.9]	Neonatal [0.92]	Acne [1.16]	Dermatitis [0.91]	Falls [0.93]	Asthma [0.8]
Greece	Headaches [1.19]	Anxiety [1.33]	Depression [1.35]	Back Pain [0.95]	Conduct [0.87]	Neonatal [0.86]	Acne [1.49]	Falls [1.03]	Asthma [0.95]	Bipolar [1.11]
Iceland	Headaches [1.02]	Anxiety [1.14]	Back Pain [1.01]	Dermatitis [1.55]	Conduct [0.92]	Depression [0.88]	Asthma [1.65]	Acne [1.3]	Neonatal [0.85]	Falls [0.84]
Ireland	Headaches [1.01]	Anxiety [1.27]	Depression [1.21]	Back Pain [0.81]	Conduct [0.9]	Asthma [1.45]	Acne [1.27]	Dermatitis [1.22]	Neonatal [0.72]	Falls [0.91]
Israel	Headaches [0.99]	Back Pain [1.17]	Depression [1.14]	Anxiety [0.74]	Neonatal [0.95]	Conduct [0.87]	Acne [1.29]	Blindness [3.01]	Dermatitis [1.03]	Falls [1.07]
Italy	Headaches [1.24]	Anxiety [1.26]	Back Pain [1.01]	Depression [1.08]	Conduct [0.89]	Acne [1.39]	Neonatal [0.64]	Dermatitis [0.94]	Bipolar [1.1]	Falls [0.82]
Luxembourg	Headaches [1.17]	Anxiety [1.13]	Back Pain [0.99]	Neonatal [1.1]	Depression [0.96]	Conduct [0.93]	Dermatitis [1.41]	Acne [1.27]	Asthma [1.29]	Falls [0.81]
Malta	Headaches [1.02]	Anxiety [1.21]	Back Pain [1.12]	Depression [1.02]	Conduct [0.87]	Acne [1.37]	Neonatal [0.71]	Falls [1.18]	Asthma [1.11]	Congenital [1.21]
Netherlands	Anxiety [1.39]	Headaches [1.06]	Depression [1.08]	Back Pain [0.8]	Conduct [1.01]	Acne [1.27]	Neonatal [0.85]	Dermatitis [1.25]	Asthma [1.19]	Bipolar [1.06]
Norway	Anxiety [1.58]	Headaches [1.07]	Back Pain [0.95]	Depression [1.02]	Conduct [1.0]	Neonatal [1.0]	Dermatitis [1.44]	Acne [1.29]	Asthma [1.49]	Falls [1.1]
Portugal	Headaches [1.0]	Anxiety [1.27]	Depression [1.28]	Back Pain [1.11]	Conduct [0.85]	Asthma [1.65]	Acne [1.49]	Neonatal [0.6]	Dermatitis [0.86]	Bipolar [1.14]
Spain	Headaches [1.18]	Anxiety [1.15]	Back Pain [1.19]	Depression [1.03]	Conduct [0.88]	Acne [1.36]	Neonatal [0.7]	Drugs [1.57]	Falls [0.96]	Asthma [0.92]
Sweden	Headaches [1.06]	Anxiety [1.14]	Depression [1.22]	Back Pain [0.92]	Dermatitis [1.85]	Conduct [1.01]	Acne [1.51]	Asthma [1.65]	Neonatal [0.75]	Falls [1.15]
Stockholm	Headaches [1.06]	Anxiety [1.12]	Conduct [1.04]	Depression [0.99]	Acne [1.43]	Dermatitis [1.46]	Back Pain [0.61]	Asthma [1.42]	Falls [1.07]	Neonatal [0.73]
Sweden w/o Stockholm	Headaches [1.06]	Anxiety [1.15]	Depression [1.28]	Back Pain [1.01]	Dermatitis [1.96]	Conduct [1.0]	Asthma [1.72]	Acne [1.53]	Neonatal [0.76]	Falls [1.18]
Switzerland	Back Pain [2.12]	Anxiety [1.15]	Headaches [0.86]	Depression [0.95]	Conduct [0.91]	Dermatitis [1.4]	Neonatal [0.83]	Acne [1.28]	Asthma [1.1]	Bipolar [1.11]
UK	Headaches [0.93]	Anxiety [1.04]	Depression [1.11]	Back Pain [0.92]	Conduct [0.98]	Neonatal [0.98]	Dermatitis [1.56]	Asthma [1.73]	Acne [1.33]	Drugs [2.14]
England	Headaches [0.92]	Anxiety [1.0]	Back Pain [0.92]	Depression [1.1]	Conduct [1.0]	Neonatal [1.01]	Dermatitis [1.59]	Asthma [1.75]	Acne [1.32]	Drugs [2.22]
E Midlands	Headaches [0.93]	Anxiety [1.02]	Depression [1.1]	Back Pain [0.93]	Conduct [0.99]	Neonatal [0.96]	Dermatitis [1.55]	Asthma [1.68]	Acne [1.37]	Drugs [2.26]
Derby	Headaches [0.92]	Anxiety [1.01]	Back Pain [0.94]	Depression [1.09]	Conduct [1.0]	Neonatal [0.96]	Dermatitis [1.55]	Asthma [1.68]	Acne [1.3]	Drugs [2.24]
Derbyshire	Headaches [0.92]	Anxiety [1.04]	Depression [1.1]	Conduct [0.98]	Back Pain [0.83]	Dermatitis [1.56]	Asthma [1.68]	Neonatal [0.89]	Acne [1.41]	Drugs [2.31]
Leicester	Headaches [0.93]	Anxiety [1.01]	Back Pain [0.95]	Depression [1.11]	Conduct [0.99]	Dermatitis [1.55]	Asthma [1.69]	Neonatal [0.82]	Drugs [2.28]	Acne [1.32]
Leicestershire	Headaches [0.93]	Anxiety [1.01]	Back Pain [0.95]	Depression [1.1]	Conduct [1.0]	Dermatitis [1.55]	Asthma [1.66]	Neonatal [0.8]	Acne [1.39]	Drugs [2.26]
Lincolnshire	Headaches [0.93]	Anxiety [1.05]	Back Pain [0.96]	Depression [1.11]	Conduct [0.97]	Neonatal [0.96]	Dermatitis [1.55]	Asthma [1.68]	Acne [1.44]	Drugs [2.31]

eFigure 9e. Leading ten causes of YLDs with the ratio of observed YLDs to YLDs expected on the basis of Socio-Demographic Index alone in 2017, 10-19 years, both sexes combined. The top ten causes contributing to YLDs are listed globally, by socio-demographic quintile, and then by GBD superregion, region, country, and subnationally where modeled. For each cell, the ratio of observed YLDs to YLDs expected on the basis of socio-demographic index (SDI) alone are listed. Abbreviations: YLD=year of life lived with disability, GBD=Global Burden of Disease.

Values shown in brackets represent the ratio of observed YLDs to predicted YLDs on the basis of Socio-Demographic Index (SDI), rounded to two (2) digits. Color ranges (shown below) were calculated to place a roughly equal number of cells into each bin.

COLOR KEY:	[0.0-0.77]	[0.77-0.91]	(0.91-0.97]	(0.97-1.02]	(1.02-1.1]	(1.1-1.25]	(1.25-1.48]	(1.48-1.87]	1.87+

	1	2	3	4	5	6	7	8	9	10
Northamptonshire	Headaches [0.91]	Anxiety [1.02]	Back Pain [0.94]	Depression [1.08]	Conduct [0.99]	Neonatal [1.06]	Dermatitis [1.57]	Asthma [1.68]	Acne [1.35]	Drugs [2.16]
Nottingham	Headaches [0.96]	Anxiety [1.0]	Back Pain [0.97]	Depression [1.13]	Neonatal [1.16]	Conduct [1.0]	Dermatitis [1.54]	Asthma [1.71]	Drugs [2.36]	Acne [1.3]
Nottinghamshire	Headaches [0.91]	Anxiety [1.03]	Back Pain [0.95]	Depression [1.09]	Conduct [0.98]	Neonatal [1.07]	Dermatitis [1.55]	Asthma [1.68]	Acne [1.39]	Drugs [2.23]
Rutland	Headaches [0.93]	Anxiety [1.01]	Back Pain [0.96]	Depression [1.12]	Conduct [0.99]	Dermatitis [1.53]	Asthma [1.67]	Acne [1.42]	Drugs [2.39]	Neonatal [0.67]
E England	Headaches [0.92]	Anxiety [1.01]	Depression [1.09]	Back Pain [0.92]	Conduct [1.0]	Dermatitis [1.55]	Asthma [1.67]	Neonatal [0.92]	Acne [1.37]	Drugs [2.21]
Bedford	Headaches [0.92]	Anxiety [1.01]	Back Pain [0.94]	Depression [1.09]	Neonatal [1.13]	Conduct [0.99]	Dermatitis [1.55]	Asthma [1.66]	Acne [1.35]	Drugs [2.26]
Cambridgeshire	Headaches [0.93]	Anxiety [0.99]	Depression [1.1]	Back Pain [0.86]	Conduct [1.02]	Dermatitis [1.55]	Asthma [1.67]	Neonatal [0.92]	Acne [1.35]	Drugs [2.21]
Cen Bedfordshire	Headaches [0.91]	Anxiety [1.01]	Back Pain [0.92]	Depression [1.08]	Conduct [1.0]	Neonatal [1.0]	Dermatitis [1.56]	Asthma [1.66]	Acne [1.37]	Drugs [2.16]
Essex	Headaches [0.92]	Anxiety [1.02]	Back Pain [0.93]	Depression [1.09]	Conduct [0.99]	Dermatitis [1.55]	Neonatal [0.91]	Asthma [1.67]	Acne [1.4]	Drugs [2.25]
Hertfordshire	Headaches [0.92]	Anxiety [0.99]	Back Pain [0.91]	Depression [1.08]	Conduct [1.02]	Dermatitis [1.56]	Asthma [1.66]	Neonatal [0.9]	Acne [1.3]	Drugs [2.09]
Luton	Headaches [0.91]	Anxiety [1.01]	Back Pain [0.92]	Depression [1.07]	Conduct [1.0]	Neonatal [1.04]	Dermatitis [1.57]	Asthma [1.7]	Acne [1.28]	Drugs [2.07]
Norfolk	Headaches [0.93]	Anxiety [1.03]	Back Pain [0.95]	Depression [1.1]	Conduct [0.98]	Dermatitis [1.55]	Asthma [1.68]	Neonatal [0.78]	Acne [1.43]	Drugs [2.36]
Peterborough	Headaches [0.91]	Anxiety [1.03]	Back Pain [0.93]	Depression [1.07]	Conduct [0.99]	Dermatitis [1.56]	Asthma [1.73]	Neonatal [0.73]	Acne [1.35]	Drugs [2.21]
Southend-on-Sea	Headaches [0.91]	Anxiety [1.04]	Depression [1.09]	Back Pain [0.92]	Neonatal [1.11]	Conduct [0.98]	Dermatitis [1.56]	Asthma [1.68]	Drugs [2.35]	Acne [1.4]
Suffolk	Headaches [0.91]	Anxiety [1.03]	Depression [1.09]	Back Pain [0.93]	Conduct [0.98]	Neonatal [0.97]	Dermatitis [1.55]	Asthma [1.67]	Acne [1.42]	Drugs [2.24]
Thurrock	Headaches [0.91]	Anxiety [1.04]	Depression [1.08]	Back Pain [0.91]	Conduct [0.98]	Neonatal [1.03]	Dermatitis [1.57]	Asthma [1.71]	Acne [1.39]	Drugs [2.15]
Greater London	Headaches [0.93]	Anxiety [0.97]	Back Pain [0.91]	Depression [1.08]	Asthma [1.96]	Conduct [1.04]	Neonatal [1.04]	Dermatitis [1.58]	Acne [1.2]	Drugs [2.07]
Barking & Dagenham	Headaches [0.9]	Anxiety [1.04]	Dermatitis [2.06]	Back Pain [0.94]	Depression [1.07]	Conduct [0.98]	Asthma [1.97]	Neonatal [1.02]	Drugs [2.14]	Acne [1.34]
Barnet	Headaches [0.91]	Anxiety [0.99]	Back Pain [0.91]	Depression [1.07]	Conduct [1.02]	Asthma [1.95]	Neonatal [0.98]	Dermatitis [1.56]	Acne [1.3]	Drugs [2.08]
Bexley	Headaches [0.92]	Anxiety [1.03]	Back Pain [0.95]	Depression [1.1]	Asthma [1.98]	Conduct [0.98]	Dermatitis [1.56]	Neonatal [0.95]	Acne [1.41]	Drugs [2.22]
Brent	Headaches [0.91]	Anxiety [1.0]	Back Pain [0.92]	Depression [1.07]	Conduct [1.01]	Asthma [1.97]	Neonatal [1.05]	Dermatitis [1.56]	Acne [1.32]	Drugs [2.11]
Bromley	Headaches [0.92]	Anxiety [1.0]	Back Pain [0.93]	Depression [1.08]	Neonatal [1.12]	Conduct [1.0]	Asthma [1.95]	Dermatitis [1.56]	Acne [1.35]	Drugs [2.14]
Camden	Headaches [0.97]	Anxiety [0.96]	Back Pain [0.94]	Depression [1.13]	Asthma [1.99]	Conduct [1.05]	Dermatitis [1.57]	Neonatal [0.88]	Acne [1.24]	Drugs [2.25]
Croydon	Headaches [0.92]	Anxiety [1.02]	Back Pain [0.93]	Depression [1.08]	Asthma [1.96]	Conduct [0.99]	Neonatal [1.01]	Dermatitis [1.57]	Acne [1.34]	Drugs [2.1]
Ealing	Headaches [0.91]	Anxiety [0.99]	Back Pain [0.91]	Depression [1.07]	Neonatal [1.13]	Conduct [1.02]	Asthma [1.96]	Dermatitis [1.57]	Acne [1.27]	Drugs [2.05]
Enfield	Headaches [0.91]	Anxiety [1.01]	Back Pain [0.93]	Depression [1.07]	Conduct [1.0]	Asthma [1.96]	Neonatal [1.05]	Dermatitis [1.56]	Acne [1.34]	Drugs [2.11]
Greenwich	Headaches [0.92]	Anxiety [1.02]	Back Pain [0.94]	Depression [1.09]	Conduct [0.99]	Asthma [1.95]	Neonatal [0.97]	Dermatitis [1.56]	Acne [1.34]	Drugs [2.16]
Hackney	Headaches [0.93]	Anxiety [0.98]	Back Pain [0.92]	Depression [1.08]	Asthma [2.07]	Conduct [1.03]	Neonatal [1.01]	Dermatitis [1.57]	Acne [1.21]	Drugs [2.11]
Hammersmith & Fulham	Headaches [0.95]	Anxiety [0.95]	Back Pain [0.91]	Depression [1.1]	Asthma [1.99]	Conduct [1.06]	Neonatal [0.99]	Dermatitis [1.58]	Acne [1.19]	Drugs [2.11]
Haringey	Headaches [0.91]	Anxiety [1.0]	Back Pain [0.92]	Depression [1.07]	Neonatal [1.1]	Conduct [1.01]	Asthma [1.96]	Dermatitis [1.56]	Acne [1.29]	Drugs [2.1]
Harrow	Headaches [0.91]	Anxiety [1.0]	Back Pain [0.92]	Depression [1.08]	Neonatal [1.15]	Conduct [1.01]	Asthma [1.95]	Dermatitis [1.56]	Acne [1.34]	Drugs [2.14]

eFigure 9e. Leading ten causes of YLDs with the ratio of observed YLDs to YLDs expected on the basis of Socio-Demographic Index alone in 2017, 10-19 years, both sexes combined. The top ten causes contributing to YLDs are listed globally, by socio-demographic quintile, and then by GBD superregion, region, country, and subnationally where modeled. For each cell, the ratio of observed YLDs to YLDs expected on the basis of socio-demographic index (SDI) alone are listed. Abbreviations: YLD=year of life lived with disability, GBD=Global Burden of Disease.

Values shown in brackets represent the ratio of observed YLDs to predicted YLDs on the basis of Socio-Demographic Index (SDI), rounded to two (2) digits. Color ranges (shown below) were calculated to place a roughly equal number of cells into each bin.

COLOR KEY:	[0.0-0.77]	(0.77-0.91]	(0.91-0.97]	(0.97-1.02]	(1.02-1.1]	(1.1-1.25]	(1.25-1.48]	(1.48-1.87]	1.87+

	1	2	3	4	5	6	7	8	9	10
Havering	Headaches [0.93]	Anxiety [1.03]	Back Pain [0.96]	Depression [1.1]	Asthma [1.98]	Conduct [0.98]	Neonatal [1.0]	Dermatitis [1.55]	Acne [1.4]	Drugs [2.23]
Hillingdon	Headaches [0.93]	Anxiety [0.98]	Back Pain [0.93]	Depression [1.1]	Neonatal [1.15]	Asthma [1.99]	Conduct [1.02]	Dermatitis [1.56]	Acne [1.29]	Drugs [2.16]
Hounslow	Headaches [0.92]	Anxiety [0.98]	Back Pain [0.91]	Depression [1.08]	Asthma [1.97]	Conduct [1.03]	Neonatal [1.09]	Dermatitis [1.57]	Acne [1.24]	Drugs [2.03]
Islington	Headaches [0.96]	Anxiety [0.96]	Back Pain [0.93]	Depression [1.11]	Asthma [2.01]	Conduct [1.04]	Dermatitis [1.58]	Neonatal [0.86]	Acne [1.2]	Drugs [2.19]
Kensington & Chelsea	Headaches [0.96]	Anxiety [0.95]	Back Pain [0.92]	Depression [1.12]	Asthma [1.97]	Conduct [1.05]	Dermatitis [1.58]	Acne [1.25]	Neonatal [0.85]	Drugs [2.24]
Kingston upon Thames	Headaches [0.95]	Anxiety [0.98]	Back Pain [0.93]	Depression [1.11]	Asthma [1.96]	Neonatal [1.08]	Conduct [1.02]	Dermatitis [1.57]	Acne [1.29]	Drugs [2.12]
Lambeth	Headaches [0.94]	Anxiety [0.97]	Back Pain [0.91]	Depression [1.08]	Asthma [1.96]	Conduct [1.04]	Dermatitis [1.58]	Acne [1.19]	Neonatal [0.73]	Drugs [2.06]
Lewisham	Headaches [0.92]	Anxiety [1.01]	Neonatal [1.21]	Back Pain [0.93]	Depression [1.08]	Conduct [1.0]	Asthma [1.96]	Dermatitis [1.56]	Acne [1.29]	Drugs [2.11]
Merton	Headaches [0.91]	Anxiety [0.98]	Back Pain [0.9]	Depression [1.07]	Conduct [1.03]	Asthma [1.95]	Neonatal [1.04]	Dermatitis [1.56]	Acne [1.26]	Drugs [2.04]
Newham	Headaches [0.93]	Anxiety [1.02]	Back Pain [0.95]	Depression [1.1]	Neonatal [1.08]	Asthma [1.96]	Conduct [0.99]	Dermatitis [1.56]	Acne [1.32]	Drugs [2.17]
Redbridge	Headaches [0.9]	Anxiety [1.02]	Back Pain [0.93]	Depression [1.07]	Conduct [1.0]	Asthma [1.96]	Neonatal [1.01]	Dermatitis [1.56]	Acne [1.34]	Drugs [2.1]
Richmond upon Thames	Headaches [0.93]	Anxiety [0.97]	Back Pain [0.9]	Depression [1.07]	Neonatal [1.14]	Conduct [1.04]	Asthma [1.94]	Dermatitis [1.58]	Acne [1.23]	Drugs [1.98]
Southwark	Headaches [0.94]	Anxiety [0.96]	Back Pain [0.91]	Depression [1.1]	Asthma [1.98]	Conduct [1.05]	Dermatitis [1.58]	Acne [1.19]	Neonatal [0.75]	Drugs [2.07]
Sutton	Headaches [0.92]	Anxiety [1.01]	Neonatal [1.29]	Back Pain [0.93]	Depression [1.09]	Asthma [1.96]	Conduct [1.0]	Dermatitis [1.56]	Acne [1.35]	Drugs [2.14]
Tower Hamlets	Headaches [0.94]	Anxiety [0.96]	Back Pain [0.92]	Depression [1.09]	Asthma [2.02]	Neonatal [1.11]	Conduct [1.04]	Dermatitis [1.57]	Acne [1.19]	Drugs [2.09]
Waltham Forest	Headaches [0.91]	Anxiety [1.03]	Back Pain [0.93]	Depression [1.08]	Conduct [0.99]	Asthma [1.95]	Neonatal [1.02]	Dermatitis [1.57]	Acne [1.35]	Drugs [2.15]
Wandsworth	Headaches [0.94]	Anxiety [0.96]	Back Pain [0.9]	Depression [1.09]	Asthma [1.95]	Conduct [1.04]	Dermatitis [1.59]	Neonatal [0.9]	Acne [1.19]	Drugs [1.99]
Westminster	Headaches [0.95]	Anxiety [0.95]	Back Pain [0.93]	Depression [1.11]	Asthma [2.04]	Conduct [1.06]	Dermatitis [1.56]	Acne [1.23]	Drugs [2.31]	Neonatal [0.8]
NE England	Headaches [0.92]	Anxiety [1.03]	Depression [1.1]	Back Pain [0.94]	Conduct [0.98]	Dermatitis [1.68]	Asthma [1.7]	Neonatal [0.91]	Drugs [2.38]	Acne [1.38]
County Durham	Headaches [0.93]	Anxiety [1.04]	Depression [1.11]	Dermatitis [1.87]	Back Pain [0.89]	Conduct [0.97]	Neonatal [0.98]	Asthma [1.73]	Drugs [2.41]	Acne [1.41]
Darlington	Headaches [0.92]	Anxiety [1.03]	Back Pain [0.95]	Depression [1.09]	Conduct [0.99]	Dermatitis [1.56]	Asthma [1.73]	Neonatal [0.88]	Acne [1.34]	Drugs [2.21]
Gateshead	Headaches [0.92]	Anxiety [1.03]	Back Pain [0.96]	Depression [1.1]	Dermatitis [1.85]	Conduct [0.98]	Neonatal [1.01]	Asthma [1.73]	Drugs [2.4]	Acne [1.35]
Hartlepool	Headaches [0.92]	Anxiety [1.05]	Back Pain [0.96]	Depression [1.11]	Conduct [0.96]	Neonatal [1.05]	Dermatitis [1.55]	Asthma [1.75]	Drugs [2.55]	Acne [1.41]
Middlesbrough	Headaches [0.92]	Anxiety [1.04]	Back Pain [0.95]	Depression [1.1]	Conduct [0.97]	Dermatitis [1.55]	Asthma [1.75]	Neonatal [0.8]	Drugs [2.42]	Acne [1.37]
Newcastle upon Tyne	Headaches [0.96]	Anxiety [0.99]	Back Pain [0.97]	Depression [1.14]	Conduct [1.0]	Asthma [1.72]	Dermatitis [1.53]	Drugs [2.48]	Neonatal [0.84]	Acne [1.35]
N Tyneside	Headaches [0.91]	Anxiety [1.02]	Back Pain [0.93]	Depression [1.08]	Conduct [0.99]	Dermatitis [1.56]	Asthma [1.7]	Neonatal [0.84]	Drugs [2.24]	Acne [1.34]
Northumberland	Headaches [0.91]	Anxiety [1.03]	Back Pain [0.94]	Depression [1.09]	Conduct [0.98]	Dermatitis [1.55]	Asthma [1.71]	Neonatal [0.88]	Drugs [2.38]	Acne [1.41]
Redcar & Cleveland	Headaches [0.92]	Anxiety [1.05]	Depression [1.1]	Back Pain [0.95]	Conduct [0.97]	Dermatitis [1.55]	Asthma [1.72]	Neonatal [0.83]	Drugs [2.38]	Acne [1.45]
S Tyneside	Headaches [0.93]	Anxiety [1.06]	Back Pain [0.96]	Depression [1.11]	Dermatitis [1.87]	Conduct [0.96]	Asthma [1.72]	Neonatal [0.88]	Drugs [2.39]	Acne [1.45]
Stockton-on-Tees	Headaches [0.91]	Anxiety [1.02]	Back Pain [0.94]	Depression [1.09]	Neonatal [1.12]	Conduct [0.99]	Dermatitis [1.55]	Asthma [1.72]	Drugs [2.34]	Acne [1.36]
Sunderland	Headaches [0.92]	Anxiety [1.03]	Back Pain [0.95]	Depression [1.1]	Dermatitis [1.87]	Conduct [0.98]	Asthma [1.51]	Neonatal [0.77]	Drugs [2.38]	Acne [1.39]

eFigure 9e. Leading ten causes of YLDs with the ratio of observed YLDs to YLDs expected on the basis of Socio-Demographic Index alone in 2017, 10-19 years, both sexes combined. The top ten causes contributing to YLDs are listed globally, by socio-demographic quintile, and then by GBD superregion, region, country, and subnationally where modeled. For each cell, the ratio of observed YLDs to YLDs expected on the basis of socio-demographic index (SDI) alone are listed. Abbreviations: YLD=year of life lived with disability, GBD=Global Burden of Disease.

Values shown in brackets represent the ratio of observed YLDs to predicted YLDs on the basis of Socio-Demographic Index (SDI), rounded to two (2) digits. Color ranges (shown below) were calculated to place a roughly equal number of cells into each bin.

COLOR KEY:		[0.0-0.77]	[0.77-0.91]	[0.91-0.97]	[0.97-1.02]	[1.02-1.1]	[1.1-1.25]	[1.25-1.48]	[1.48-1.87]	1.87+
	1	2	3	4	5	6	7	8	9	10
NW England	Headaches [0.92]	Anxiety [1.01]	Depression [1.11]	Back Pain [0.93]	Neonatal [1.12]	Conduct [0.99]	Dermatitis [1.55]	Asthma [1.71]	Drugs [2.3]	Acne [1.33]
Blackburn with Darwen	Headaches [0.91]	Anxiety [1.05]	Back Pain [0.95]	Depression [1.08]	Neonatal [1.11]	Conduct [0.98]	Dermatitis [1.56]	Asthma [1.73]	Drugs [2.3]	Acne [1.35]
Blackpool	Headaches [0.92]	Anxiety [1.07]	Back Pain [0.97]	Depression [1.1]	Neonatal [1.08]	Conduct [0.96]	Asthma [1.79]	Dermatitis [1.57]	Drugs [2.62]	Acne [1.39]
Bolton	Headaches [0.91]	Anxiety [1.04]	Back Pain [0.96]	Depression [1.09]	Conduct [0.98]	Neonatal [1.06]	Dermatitis [1.55]	Asthma [1.7]	Drugs [2.41]	Acne [1.37]
Bury	Headaches [0.9]	Anxiety [1.02]	Back Pain [0.95]	Depression [1.08]	Conduct [0.99]	Neonatal [1.01]	Dermatitis [1.55]	Asthma [1.69]	Drugs [2.24]	Acne [1.34]
Cheshire E	Headaches [0.93]	Neonatal [1.38]	Anxiety [0.99]	Back Pain [0.94]	Depression [1.09]	Conduct [1.01]	Dermatitis [1.56]	Asthma [1.7]	Acne [1.3]	Drugs [2.18]
Cheshire W & Chester	Headaches [0.93]	Anxiety [1.0]	Back Pain [0.94]	Depression [1.1]	Neonatal [1.12]	Conduct [1.0]	Dermatitis [1.55]	Asthma [1.69]	Acne [1.33]	Drugs [2.27]
Cumbria	Headaches [0.92]	Anxiety [1.02]	Back Pain [0.94]	Depression [1.1]	Neonatal [1.11]	Conduct [0.99]	Dermatitis [1.55]	Asthma [1.71]	Drugs [2.34]	Acne [1.39]
Halton	Headaches [0.92]	Anxiety [1.03]	Back Pain [0.93]	Depression [1.09]	Neonatal [1.14]	Conduct [0.98]	Dermatitis [1.56]	Asthma [1.72]	Drugs [2.24]	Acne [1.34]
Knowsley	Headaches [0.92]	Anxiety [1.03]	Back Pain [0.95]	Depression [1.1]	Neonatal [1.09]	Conduct [0.98]	Asthma [1.76]	Dermatitis [1.55]	Drugs [2.36]	Acne [1.37]
Lancashire	Headaches [0.93]	Anxiety [1.02]	Depression [1.1]	Back Pain [0.88]	Neonatal [1.08]	Conduct [0.99]	Dermatitis [1.55]	Asthma [1.71]	Drugs [2.34]	Acne [1.37]
Liverpool	Headaches [0.96]	Depression [1.31]	Anxiety [0.93]	Back Pain [0.96]	Neonatal [1.21]	Conduct [0.98]	Dermatitis [1.54]	Asthma [1.7]	Drugs [2.48]	Acne [1.35]
Manchester	Headaches [0.95]	Anxiety [0.98]	Back Pain [0.98]	Neonatal [1.2]	Depression [1.11]	Conduct [1.02]	Asthma [1.73]	Dermatitis [1.55]	Acne [1.22]	Drugs [2.22]
Oldham	Headaches [0.91]	Anxiety [1.05]	Back Pain [0.96]	Depression [1.09]	Conduct [0.97]	Dermatitis [1.56]	Asthma [1.77]	Neonatal [0.83]	Drugs [2.32]	Acne [1.37]
Rochdale	Headaches [0.91]	Anxiety [1.05]	Back Pain [0.96]	Depression [1.09]	Conduct [0.97]	Dermatitis [1.56]	Asthma [1.74]	Neonatal [0.86]	Drugs [2.37]	Acne [1.39]
Salford	Headaches [0.92]	Anxiety [1.02]	Neonatal [1.25]	Back Pain [0.95]	Depression [1.09]	Conduct [0.99]	Dermatitis [1.56]	Asthma [1.72]	Drugs [2.23]	Acne [1.29]
Sefton	Headaches [0.92]	Anxiety [1.04]	Back Pain [0.94]	Depression [1.09]	Neonatal [1.12]	Conduct [0.98]	Dermatitis [1.55]	Asthma [1.71]	Drugs [2.42]	Acne [1.39]
St Helens	Headaches [0.91]	Anxiety [1.04]	Back Pain [0.95]	Depression [1.09]	Neonatal [1.13]	Conduct [0.98]	Dermatitis [1.55]	Asthma [1.71]	Drugs [2.39]	Acne [1.37]
Stockport	Headaches [0.92]	Anxiety [1.01]	Back Pain [0.93]	Depression [1.09]	Conduct [1.0]	Dermatitis [1.56]	Neonatal [0.94]	Asthma [1.68]	Acne [1.31]	Drugs [2.24]
Tameside	Headaches [0.91]	Anxiety [1.05]	Depression [1.09]	Conduct [0.97]	Dermatitis [1.56]	Back Pain [0.77]	Asthma [1.74]	Neonatal [0.92]	Drugs [2.31]	Acne [1.39]
Trafford	Headaches [0.91]	Anxiety [0.98]	Neonatal [1.22]	Back Pain [0.91]	Depression [1.07]	Conduct [1.03]	Dermatitis [1.57]	Asthma [1.7]	Acne [1.25]	Drugs [2.04]
Warrington	Headaches [0.92]	Anxiety [0.99]	Neonatal [1.3]	Back Pain [0.93]	Depression [1.09]	Conduct [1.01]	Dermatitis [1.55]	Asthma [1.72]	Acne [1.29]	Drugs [2.25]
Wigan	Headaches [0.91]	Anxiety [1.04]	Back Pain [0.95]	Depression [1.08]	Neonatal [1.13]	Conduct [0.98]	Dermatitis [1.55]	Asthma [1.71]	Drugs [2.34]	Acne [1.38]
Wirral	Headaches [0.91]	Anxiety [1.04]	Neonatal [1.27]	Back Pain [0.94]	Depression [1.09]	Conduct [0.98]	Dermatitis [1.55]	Asthma [1.7]	Drugs [2.36]	Acne [1.38]
SE England	Headaches [0.92]	Anxiety [1.0]	Depression [1.09]	Back Pain [0.91]	Conduct [1.0]	Dermatitis [1.71]	Neonatal [1.03]	Asthma [1.81]	Acne [1.34]	Drugs [2.19]
Bracknell Forest	Headaches [0.92]	Anxiety [0.99]	Back Pain [0.91]	Depression [1.08]	Conduct [1.02]	Dermatitis [1.56]	Asthma [1.65]	Acne [1.32]	Neonatal [0.8]	Drugs [2.13]
Brighton & Hove	Headaches [0.96]	Anxiety [0.98]	Depression [1.13]	Back Pain [0.93]	Dermatitis [1.85]	Neonatal [1.12]	Conduct [1.01]	Asthma [1.65]	Acne [1.32]	Drugs [2.39]
Buckinghamshire	Headaches [0.92]	Anxiety [0.99]	Back Pain [0.92]	Depression [1.09]	Neonatal [1.15]	Conduct [1.01]	Dermatitis [1.56]	Asthma [1.63]	Acne [1.31]	Drugs [2.12]
E Sussex	Headaches [0.92]	Anxiety [1.04]	Depression [1.1]	Back Pain [0.94]	Dermatitis [1.83]	Neonatal [1.1]	Conduct [0.97]	Asthma [1.59]	Drugs [2.33]	Acne [1.42]
Hampshire	Headaches [0.92]	Asthma [2.51]	Anxiety [1.0]	Back Pain [0.92]	Depression [1.09]	Conduct [1.0]	Neonatal [1.01]	Dermatitis [1.56]	Acne [1.38]	Drugs [2.18]
Isle of Wight	Headaches [0.92]	Asthma [2.54]	Anxiety [1.03]	Depression [1.1]	Back Pain [0.94]	Conduct [0.98]	Neonatal [0.95]	Dermatitis [1.5]	Acne [1.46]	Drugs [2.37]

eFigure 9e. Leading ten causes of YLDs with the ratio of observed YLDs to YLDs expected on the basis of Socio-Demographic Index alone in 2017, 10-19 years, both sexes combined. The top ten causes contributing to YLDs are listed globally, by socio-demographic quintile, and then by GBD superregion, region, country, and subnationally where modeled. For each cell, the ratio of observed YLDs to YLDs expected on the basis of socio-demographic index (SDI) alone are listed. Abbreviations: YLD=year of life lived with disability, GBD=Global Burden of Disease.

Values shown in brackets represent the ratio of observed YLDs to predicted YLDs on the basis of Socio-Demographic Index (SDI), rounded to two (2) digits. Color ranges (shown below) were calculated to place a roughly equal number of cells into each bin.

COLOR KEY:	[0.0-0.77]	(0.77-0.91]	(0.91-0.97]	(0.97-1.02]	(1.02-1.1]	(1.1-1.25]	(1.25-1.48]	(1.48-1.87]	1.87+

	1	2	3	4	5	6	7	8	9	10
Kent	Headaches [0.92]	Anxiety [1.02]	Back Pain [0.93]	Depression [1.1]	Dermatitis [1.83]	Conduct [0.99]	Neonatal [1.0]	Asthma [1.63]	Acne [1.39]	Drugs [2.26]
Medway	Headaches [0.92]	Anxiety [1.04]	Depression [1.1]	Back Pain [0.93]	Neonatal [1.07]	Conduct [0.97]	Dermatitis [1.55]	Asthma [1.65]	Drugs [2.31]	Acne [1.4]
Milton Keynes	Headaches [0.91]	Anxiety [1.0]	Back Pain [0.9]	Depression [1.06]	Conduct [1.02]	Dermatitis [1.58]	Asthma [1.68]	Neonatal [0.88]	Acne [1.25]	Drugs [1.99]
Oxfordshire	Headaches [0.94]	Anxiety [0.98]	Dermatitis [2.04]	Back Pain [0.92]	Depression [1.1]	Conduct [1.02]	Neonatal [0.93]	Asthma [1.65]	Acne [1.32]	Drugs [2.17]
Portsmouth	Headaches [0.94]	Anxiety [1.0]	Depression [1.12]	Back Pain [0.93]	Conduct [1.0]	Neonatal [0.98]	Dermatitis [1.54]	Asthma [1.65]	Acne [1.35]	Drugs [2.36]
Reading	Headaches [0.94]	Anxiety [0.97]	Dermatitis [2.06]	Neonatal [1.21]	Back Pain [0.91]	Depression [1.1]	Conduct [1.03]	Asthma [1.68]	Acne [1.23]	Drugs [2.14]
Slough	Headaches [0.9]	Anxiety [0.99]	Back Pain [0.89]	Depression [1.05]	Conduct [1.02]	Neonatal [1.07]	Dermatitis [1.58]	Asthma [1.72]	Acne [1.22]	Drugs [1.96]
Southampton	Headaches [0.94]	Anxiety [1.0]	Depression [1.12]	Back Pain [0.93]	Conduct [1.0]	Neonatal [0.97]	Dermatitis [1.54]	Asthma [1.65]	Acne [1.36]	Drugs [2.38]
Surrey	Headaches [0.93]	Anxiety [0.98]	Depression [1.09]	Dermatitis [1.83]	Neonatal [1.12]	Back Pain [0.84]	Conduct [1.03]	Asthma [1.64]	Acne [1.29]	Drugs [2.08]
West Berkshire	Headaches [0.92]	Anxiety [0.99]	Back Pain [0.92]	Depression [1.09]	Conduct [1.02]	Dermatitis [1.56]	Asthma [1.67]	Neonatal [0.93]	Acne [1.32]	Drugs [2.11]
W Sussex	Headaches [0.91]	Anxiety [1.0]	Back Pain [0.92]	Depression [1.08]	Conduct [1.0]	Neonatal [1.05]	Asthma [1.9]	Dermatitis [1.62]	Acne [1.35]	Drugs [2.19]
Windsor & Maidenhead	Headaches [0.92]	Anxiety [0.96]	Back Pain [0.9]	Depression [1.07]	Conduct [1.04]	Dermatitis [1.56]	Neonatal [0.96]	Asthma [1.65]	Acne [1.24]	Drugs [2.07]
Wokingham	Headaches [0.92]	Anxiety [0.97]	Back Pain [0.9]	Depression [1.08]	Conduct [1.03]	Neonatal [0.99]	Dermatitis [1.57]	Asthma [1.64]	Acne [1.27]	Drugs [2.02]
SW England	Headaches [0.93]	Anxiety [1.01]	Depression [1.1]	Back Pain [0.93]	Neonatal [1.08]	Conduct [0.99]	Dermatitis [1.55]	Asthma [1.66]	Acne [1.39]	Drugs [2.25]
Bath & NE Somerset	Headaches [0.96]	Neonatal [1.43]	Anxiety [0.99]	Back Pain [0.95]	Depression [1.14]	Conduct [1.0]	Dermatitis [1.54]	Asthma [1.66]	Acne [1.4]	Drugs [2.36]
Bournemouth	Headaches [0.95]	Anxiety [1.0]	Depression [1.12]	Back Pain [0.94]	Conduct [1.0]	Neonatal [1.06]	Dermatitis [1.54]	Asthma [1.65]	Acne [1.38]	Drugs [2.37]
Bristol	Headaches [0.95]	Anxiety [0.99]	Neonatal [1.22]	Back Pain [0.92]	Depression [1.12]	Conduct [1.01]	Dermatitis [1.56]	Asthma [1.68]	Acne [1.28]	Drugs [2.17]
Cornwall	Headaches [0.93]	Anxiety [1.04]	Depression [1.11]	Back Pain [0.89]	Neonatal [1.07]	Conduct [0.98]	Dermatitis [1.55]	Asthma [1.65]	Acne [1.44]	Drugs [2.33]
Devon	Headaches [0.92]	Anxiety [1.01]	Back Pain [0.94]	Depression [1.11]	Conduct [0.99]	Neonatal [0.98]	Dermatitis [1.55]	Asthma [1.65]	Acne [1.42]	Drugs [2.28]
Dorset	Headaches [0.92]	Anxiety [1.02]	Back Pain [0.93]	Depression [1.09]	Conduct [0.99]	Neonatal [1.05]	Dermatitis [1.55]	Asthma [1.66]	Acne [1.43]	Drugs [2.27]
Gloucestershire	Headaches [0.93]	Anxiety [1.01]	Back Pain [0.93]	Depression [1.1]	Conduct [1.0]	Neonatal [0.99]	Dermatitis [1.55]	Asthma [1.66]	Acne [1.39]	Drugs [2.21]
N Somerset	Headaches [0.92]	Anxiety [1.02]	Neonatal [1.17]	Depression [1.09]	Back Pain [0.92]	Conduct [0.99]	Dermatitis [1.56]	Asthma [1.66]	Acne [1.35]	Drugs [2.26]
Plymouth	Headaches [0.93]	Anxiety [1.02]	Depression [1.11]	Back Pain [0.93]	Neonatal [1.07]	Conduct [0.98]	Dermatitis [1.55]	Asthma [1.65]	Drugs [2.38]	Acne [1.38]
Poole	Headaches [0.92]	Anxiety [1.01]	Depression [1.09]	Back Pain [0.92]	Conduct [1.0]	Neonatal [1.05]	Dermatitis [1.56]	Asthma [1.66]	Acne [1.37]	Drugs [2.13]
Somerset	Headaches [0.92]	Anxiety [1.03]	Back Pain [0.95]	Depression [1.11]	Neonatal [1.13]	Conduct [0.98]	Dermatitis [1.55]	Asthma [1.66]	Acne [1.45]	Drugs [2.3]
S Gloucestershire	Headaches [0.93]	Anxiety [0.99]	Back Pain [0.92]	Depression [1.09]	Neonatal [1.12]	Conduct [1.01]	Dermatitis [1.55]	Asthma [1.66]	Acne [1.35]	Drugs [2.15]
Swindon	Headaches [0.92]	Anxiety [1.01]	Back Pain [0.93]	Depression [1.08]	Conduct [1.0]	Dermatitis [1.56]	Asthma [1.68]	Neonatal [0.77]	Acne [1.32]	Drugs [2.12]
Torbay	Headaches [0.91]	Anxiety [1.05]	Depression [1.1]	Back Pain [0.94]	Conduct [0.97]	Dermatitis [1.56]	Asthma [1.67]	Neonatal [0.88]	Drugs [2.33]	Acne [1.46]
Wiltshire	Headaches [0.92]	Anxiety [1.02]	Neonatal [1.25]	Back Pain [0.94]	Depression [1.09]	Conduct [0.99]	Dermatitis [1.55]	Asthma [1.66]	Acne [1.41]	Drugs [2.19]
W Midlands	Headaches [0.92]	Anxiety [1.02]	Back Pain [0.93]	Depression [1.09]	Conduct [0.99]	Neonatal [1.01]	Dermatitis [1.55]	Asthma [1.68]	Drugs [2.27]	Acne [1.34]
Birmingham	Headaches [0.92]	Anxiety [1.01]	Back Pain [0.94]	Depression [1.09]	Conduct [0.99]	Neonatal [0.98]	Dermatitis [1.56]	Asthma [1.68]	Drugs [2.24]	Acne [1.3]

eFigure 9e. Leading ten causes of YLDs with the ratio of observed YLDs to YLDs expected on the basis of Socio-Demographic Index alone in 2017, 10-19 years, both sexes combined. The top ten causes contributing to YLDs are listed globally, by socio-demographic quintile, and then by GBD superregion, region, country, and subnationally where modeled. For each cell, the ratio of observed YLDs to YLDs expected on the basis of socio-demographic index (SDI) alone are listed. Abbreviations: YLD=year of life lived with disability, GBD=Global Burden of Disease.

Values shown in brackets represent the ratio of observed YLDs to predicted YLDs on the basis of Socio-Demographic Index (SDI), rounded to two (2) digits. Color ranges (shown below) were calculated to place a roughly equal number of cells into each bin.

COLOR KEY:	[0.0-0.77]	[0.77-0.91]	[0.91-0.97]	[0.97-1.02]	[1.02-1.1]	[1.1-1.25]	[1.25-1.48]	[1.48-1.87]	1.87+

	1	2	3	4	5	6	7	8	9	10
Coventry	Headaches [0.94]	Anxiety [1.01]	Back Pain [0.95]	Depression [1.12]	Conduct [0.99]	Dermatitis [1.55]	Neonatal [0.95]	Asthma [1.7]	Acne [1.32]	Drugs [2.28]
Dudley	Headaches [0.91]	Anxiety [1.05]	Back Pain [0.96]	Depression [1.09]	Neonatal [1.12]	Conduct [0.97]	Dermatitis [1.56]	Asthma [1.69]	Drugs [2.27]	Acne [1.38]
Herefordshire	Headaches [0.93]	Anxiety [1.02]	Back Pain [0.95]	Depression [1.1]	Conduct [0.98]	Neonatal [1.03]	Dermatitis [1.56]	Asthma [1.68]	Acne [1.4]	Drugs [2.31]
Sandwell	Headaches [0.9]	Anxiety [1.04]	Back Pain [0.95]	Depression [1.08]	Neonatal [1.1]	Conduct [0.98]	Dermatitis [1.56]	Asthma [1.73]	Drugs [2.22]	Acne [1.34]
Shropshire	Headaches [0.93]	Anxiety [1.02]	Back Pain [0.96]	Depression [1.11]	Conduct [0.98]	Neonatal [1.0]	Dermatitis [1.55]	Asthma [1.67]	Acne [1.41]	Drugs [2.32]
Solihull	Headaches [0.92]	Anxiety [1.0]	Back Pain [0.93]	Depression [1.09]	Conduct [1.01]	Dermatitis [1.55]	Asthma [1.67]	Acne [1.33]	Drugs [2.22]	Neonatal [0.72]
Staffordshire	Headaches [0.92]	Anxiety [1.02]	Depression [1.1]	Conduct [0.99]	Back Pain [0.85]	Neonatal [0.99]	Dermatitis [1.55]	Asthma [1.67]	Acne [1.4]	Drugs [2.32]
Stoke-on-Trent	Headaches [0.91]	Anxiety [1.04]	Neonatal [1.21]	Depression [1.09]	Conduct [0.98]	Back Pain [0.85]	Dermatitis [1.56]	Asthma [1.7]	Drugs [2.34]	Acne [1.34]
Telford & Wrekin	Headaches [0.93]	Anxiety [1.04]	Back Pain [0.95]	Depression [1.1]	Conduct [0.97]	Neonatal [1.01]	Dermatitis [1.56]	Asthma [1.69]	Drugs [2.3]	Acne [1.37]
Walsall	Headaches [0.91]	Anxiety [1.05]	Back Pain [0.97]	Depression [1.09]	Conduct [0.97]	Neonatal [1.02]	Dermatitis [1.56]	Asthma [1.72]	Drugs [2.33]	Acne [1.38]
Warwickshire	Headaches [0.92]	Anxiety [1.0]	Back Pain [0.93]	Depression [1.09]	Conduct [1.01]	Neonatal [1.02]	Dermatitis [1.56]	Asthma [1.69]	Acne [1.32]	Drugs [2.24]
Wolverhampton	Headaches [0.92]	Anxiety [1.04]	Back Pain [0.95]	Depression [1.1]	Conduct [0.98]	Dermatitis [1.55]	Asthma [1.69]	Neonatal [0.74]	Drugs [2.26]	Acne [1.35]
Worcestershire	Headaches [0.92]	Anxiety [1.02]	Back Pain [0.94]	Depression [1.1]	Neonatal [1.14]	Conduct [0.99]	Dermatitis [1.55]	Asthma [1.67]	Acne [1.36]	Drugs [2.27]
Yorkshire & Humber	Headaches [0.92]	Anxiety [1.02]	Back Pain [0.95]	Depression [1.1]	Conduct [0.99]	Dermatitis [1.55]	Neonatal [0.95]	Asthma [1.71]	Acne [1.36]	Drugs [2.26]
Barnsley	Headaches [0.92]	Anxiety [1.06]	Back Pain [0.97]	Depression [1.1]	Conduct [0.96]	Neonatal [1.01]	Dermatitis [1.56]	Asthma [1.75]	Drugs [2.36]	Acne [1.41]
Bradford	Headaches [0.91]	Anxiety [1.04]	Back Pain [0.95]	Depression [1.08]	Conduct [0.98]	Neonatal [1.07]	Dermatitis [1.56]	Asthma [1.71]	Acne [1.35]	Drugs [2.17]
Calderdale	Headaches [0.92]	Anxiety [1.03]	Back Pain [0.95]	Depression [1.09]	Conduct [0.98]	Dermatitis [1.56]	Asthma [1.71]	Neonatal [0.91]	Acne [1.36]	Drugs [2.23]
Doncaster	Headaches [0.92]	Anxiety [1.06]	Neonatal [1.24]	Back Pain [0.96]	Depression [1.09]	Conduct [0.96]	Dermatitis [1.57]	Asthma [1.74]	Drugs [2.29]	Acne [1.41]
E Riding of Yorkshire	Headaches [0.93]	Anxiety [1.03]	Back Pain [0.95]	Depression [1.11]	Conduct [0.98]	Dermatitis [1.55]	Neonatal [0.94]	Asthma [1.71]	Acne [1.43]	Drugs [2.3]
Kingston upon Hull	Headaches [0.91]	Anxiety [1.03]	Back Pain [0.94]	Depression [1.09]	Conduct [0.98]	Dermatitis [1.55]	Asthma [1.75]	Neonatal [0.87]	Drugs [2.33]	Acne [1.36]
Kirklees	Headaches [0.92]	Anxiety [1.04]	Back Pain [0.95]	Depression [1.1]	Conduct [0.98]	Dermatitis [1.56]	Asthma [1.7]	Neonatal [0.88]	Acne [1.38]	Drugs [2.23]
Leeds	Headaches [0.94]	Anxiety [0.99]	Back Pain [0.94]	Depression [1.11]	Conduct [1.01]	Dermatitis [1.55]	Asthma [1.7]	Neonatal [0.84]	Acne [1.29]	Drugs [2.2]
NE Lincolnshire	Headaches [0.92]	Anxiety [1.04]	Back Pain [0.94]	Depression [1.09]	Conduct [0.97]	Dermatitis [1.56]	Asthma [1.75]	Neonatal [0.91]	Drugs [2.3]	Acne [1.4]
N Lincolnshire	Headaches [0.91]	Anxiety [1.04]	Back Pain [0.94]	Depression [1.08]	Conduct [0.98]	Dermatitis [1.56]	Asthma [1.74]	Neonatal [0.84]	Acne [1.38]	Drugs [2.2]
N Yorkshire	Headaches [0.91]	Anxiety [1.01]	Back Pain [0.94]	Depression [1.09]	Conduct [1.0]	Neonatal [1.08]	Dermatitis [1.55]	Asthma [1.68]	Acne [1.38]	Drugs [2.21]
Rotherham	Headaches [0.92]	Anxiety [1.05]	Back Pain [0.97]	Depression [1.1]	Conduct [0.97]	Dermatitis [1.56]	Asthma [1.75]	Neonatal [0.86]	Drugs [2.34]	Acne [1.41]
Sheffield	Headaches [0.95]	Anxiety [1.0]	Back Pain [0.96]	Depression [1.12]	Conduct [0.99]	Neonatal [0.98]	Dermatitis [1.54]	Asthma [1.69]	Acne [1.35]	Drugs [2.34]
Wakefield	Headaches [0.92]	Anxiety [1.04]	Back Pain [0.96]	Depression [1.09]	Conduct [0.97]	Dermatitis [1.56]	Asthma [1.76]	Neonatal [0.75]	Drugs [2.36]	Acne [1.4]
York	Headaches [0.96]	Anxiety [0.99]	Back Pain [0.96]	Depression [1.14]	Conduct [1.01]	Dermatitis [1.54]	Asthma [1.7]	Neonatal [0.85]	Acne [1.35]	Drugs [2.35]
N Ireland	Anxiety [1.67]	Headaches [1.0]	Depression [1.21]	Back Pain [0.98]	Conduct [0.83]	Neonatal [0.84]	Asthma [1.51]	Dermatitis [1.3]	Acne [1.36]	Bipolar [1.39]
Scotland	Headaches [0.98]	Anxiety [1.12]	Depression [1.14]	Back Pain [0.92]	Conduct [0.91]	Asthma [1.77]	Dermatitis [1.44]	Neonatal [0.81]	Acne [1.4]	Drugs [2.13]

eFigure 9e. Leading ten causes of YLDs with the ratio of observed YLDs to YLDs expected on the basis of Socio-Demographic Index alone in 2017, 10-19 years, both sexes combined. The top ten causes contributing to YLDs are listed globally, by socio-demographic quintile, and then by GBD superregion, region, country, and subnationally where modeled. For each cell, the ratio of observed YLDs to YLDs expected on the basis of socio-demographic index (SDI) alone are listed. Abbreviations: YLD=year of life lived with disability, GBD=Global Burden of Disease.

Values shown in brackets represent the ratio of observed YLDs to predicted YLDs on the basis of Socio-Demographic Index (SDI), rounded to two (2) digits. Color ranges (shown below) were calculated to place a roughly equal number of cells into each bin.

COLOR KEY:	[0.0-0.77]	(0.77-0.91]	(0.91-0.97]	(0.97-1.02]	(1.02-1.1]	(1.1-1.25]	(1.25-1.48]	(1.48-1.87]	1.87+

	1	2	3	4	5	6	7	8	9	10
Wales	Headaches [1.0]	Anxiety [1.12]	Depression [1.2]	Conduct [0.9]	Back Pain [0.77]	Neonatal [0.83]	Asthma [1.49]	Dermatitis [1.31]	Acne [1.42]	Falls [1.29]
**Latin America and Caribbean**	Headaches [1.17]	Back Pain [1.28]	Neonatal [1.18]	Conduct [0.95]	Anxiety [0.94]	Depression [0.93]	Iron [0.88]	Asthma [1.18]	Bipolar [1.36]	Dermatitis [0.89]
**Andean Latin America**	Headaches [1.12]	Back Pain [1.08]	Neonatal [1.12]	Conduct [0.94]	Anxiety [0.97]	Dermatitis [1.4]	Depression [0.77]	Asthma [1.33]	Iron [0.8]	Bipolar [1.29]
Bolivia	Headaches [1.16]	Back Pain [1.21]	Iron [1.2]	Conduct [0.93]	Neonatal [1.08]	Anxiety [1.02]	Dermatitis [1.46]	Depression [0.8]	Asthma [1.59]	Bipolar [1.33]
Ecuador	Headaches [1.33]	Neonatal [1.13]	Back Pain [1.0]	Conduct [0.93]	Anxiety [0.97]	Depression [0.98]	Dermatitis [1.23]	Asthma [1.21]	Epilepsy [1.51]	Bipolar [1.3]
Peru	Headaches [0.99]	Back Pain [1.08]	Neonatal [1.13]	Conduct [0.94]	Anxiety [0.96]	Dermatitis [1.48]	Iron [0.9]	Asthma [1.3]	Depression [0.64]	Bipolar [1.28]
**Caribbean**	Headaches [1.15]	Iron [1.79]	Disaster [1042.81]	Anxiety [1.05]	Conduct [0.86]	Depression [0.95]	Neonatal [0.95]	Back Pain [0.79]	Asthma [1.55]	Dermatitis [1.05]
Antigua	Iron [5.53]	Headaches [1.13]	Anxiety [0.97]	Conduct [0.87]	Neonatal [0.98]	Depression [0.83]	Back Pain [0.74]	Asthma [1.57]	Dermatitis [0.98]	Bipolar [1.28]
Bahamas	Headaches [1.13]	Anxiety [0.94]	Conduct [0.89]	Neonatal [0.97]	Asthma [1.61]	Depression [0.82]	Back Pain [0.71]	Dermatitis [0.99]	Bipolar [1.2]	Acne [1.09]
Barbados	Headaches [1.13]	Anxiety [0.95]	Neonatal [1.05]	Conduct [0.88]	Depression [0.87]	Back Pain [0.73]	Asthma [1.44]	Dermatitis [0.96]	Acne [1.22]	Bipolar [1.23]
Belize	Headaches [1.16]	Iron [1.57]	Anxiety [1.1]	Neonatal [1.05]	Conduct [0.86]	Depression [0.94]	Back Pain [0.83]	Asthma [1.75]	Dermatitis [1.1]	Bipolar [1.33]
Bermuda	Headaches [1.13]	Anxiety [0.89]	Conduct [0.91]	Neonatal [0.98]	Asthma [1.77]	Depression [0.87]	Back Pain [0.7]	Acne [1.21]	Dermatitis [0.98]	Bipolar [1.22]
Cuba	Headaches [1.14]	Anxiety [1.01]	Conduct [0.87]	Neonatal [0.96]	Depression [0.93]	Asthma [1.63]	Iron [1.37]	Back Pain [0.64]	Dermatitis [1.0]	Acne [1.52]
Dominica	Headaches [1.13]	Iron [2.3]	Anxiety [1.0]	Neonatal [1.11]	Conduct [0.87]	Back Pain [0.77]	Depression [0.84]	Asthma [1.61]	Dermatitis [1.02]	Epilepsy [1.54]
Dominican Rep	Headaches [1.17]	Anxiety [1.11]	Neonatal [1.05]	Conduct [0.86]	Depression [1.01]	Iron [1.03]	Back Pain [0.85]	Asthma [1.3]	Dermatitis [1.09]	Bipolar [1.37]
Grenada	Headaches [1.15]	Anxiety [1.05]	Neonatal [1.09]	Conduct [0.87]	Depression [0.92]	Back Pain [0.8]	Asthma [1.7]	Dermatitis [1.05]	Iron [0.77]	Bipolar [1.33]
Guyana	Headaches [1.18]	Depression [1.41]	Anxiety [1.12]	Neonatal [1.11]	Conduct [0.85]	Iron [0.99]	Back Pain [0.85]	Asthma [1.65]	Dermatitis [1.09]	Bipolar [1.39]
Haiti	Disaster [1113.3]	Iron [1.4]	Headaches [1.23]	Anxiety [1.14]	Conduct [0.85]	Depression [0.94]	Back Pain [0.94]	Neonatal [1.04]	Asthma [1.65]	Dermatitis [1.07]
Jamaica	Headaches [1.14]	Anxiety [1.01]	Iron [1.91]	Neonatal [1.04]	Conduct [0.86]	Back Pain [0.79]	Depression [0.86]	Asthma [1.62]	Dermatitis [1.01]	Bipolar [1.34]
Puerto Rico	Headaches [1.13]	Anxiety [0.89]	Asthma [2.12]	Neonatal [1.05]	Conduct [0.91]	Back Pain [0.68]	Depression [0.77]	Acne [1.21]	Dermatitis [0.96]	Bipolar [1.26]
St Lucia	Headaches [1.15]	Anxiety [1.04]	Neonatal [1.11]	Conduct [0.86]	Depression [0.94]	Back Pain [0.8]	Asthma [1.64]	Iron [1.1]	Dermatitis [1.04]	Bipolar [1.33]
St Vincent	Headaches [1.16]	Anxiety [1.09]	Neonatal [1.07]	Conduct [0.86]	Depression [0.96]	Back Pain [0.83]	Asthma [1.67]	Iron [0.88]	Dermatitis [1.08]	Bipolar [1.33]
Suriname	Headaches [1.14]	Depression [1.29]	Anxiety [1.05]	Neonatal [1.04]	Conduct [0.87]	Asthma [1.73]	Back Pain [0.77]	Iron [0.98]	Dermatitis [1.05]	Bipolar [1.29]
Trinidad Tobago	Headaches [1.12]	Anxiety [0.98]	Depression [1.05]	Neonatal [1.06]	Conduct [0.88]	Asthma [1.74]	Back Pain [0.75]	Dermatitis [1.01]	Iron [1.14]	Bipolar [1.23]
Virgin Isl US	Headaches [1.12]	Anxiety [0.89]	Neonatal [1.01]	Conduct [0.92]	Depression [0.87]	Asthma [1.59]	Back Pain [0.69]	Dermatitis [0.98]	Acne [1.08]	Bipolar [1.18]
**Central Latin America**	Headaches [1.0]	Neonatal [1.41]	Conduct [0.98]	Back Pain [0.81]	Depression [0.74]	Anxiety [0.69]	Epilepsy [1.69]	Asthma [1.0]	Dermatitis [0.89]	Bipolar [1.11]
Colombia	Headaches [1.05]	Neonatal [1.39]	Back Pain [1.11]	Conduct [0.95]	Anxiety [0.6]	Depression [0.63]	Epilepsy [1.73]	Dermatitis [1.02]	Asthma [1.01]	Drugs [1.8]
Costa Rica	Headaches [1.0]	Neonatal [1.31]	Conduct [0.95]	Back Pain [0.85]	Depression [0.82]	Anxiety [0.67]	Asthma [1.38]	Iron [1.0]	Epilepsy [1.58]	Dermatitis [0.96]
El Salvador	Headaches [1.02]	Neonatal [1.41]	Conduct [0.94]	Back Pain [0.92]	Depression [0.95]	Anxiety [0.72]	Asthma [1.32]	Epilepsy [1.43]	Dermatitis [0.91]	Iron [0.5]
Guatemala	Headaches [1.04]	Neonatal [1.48]	Conduct [0.94]	Back Pain [1.06]	Depression [0.94]	Anxiety [0.73]	Dermatitis [1.09]	Iron [0.4]	Epilepsy [1.08]	Asthma [0.92]
Honduras	Headaches [1.05]	Neonatal [1.62]	Conduct [0.94]	Back Pain [0.97]	Anxiety [0.73]	Iron [0.58]	Depression [0.74]	Epilepsy [1.59]	Asthma [1.29]	Dermatitis [1.09]

eFigure 9e. Leading ten causes of YLDs with the ratio of observed YLDs to YLDs expected on the basis of Socio-Demographic Index alone in 2017, 10-19 years, both sexes combined. The top ten causes contributing to YLDs are listed globally, by socio-demographic quintile, and then by GBD superregion, region, country, and subnationally where modeled. For each cell, the ratio of observed YLDs to YLDs expected on the basis of socio-demographic index (SDI) alone are listed. Abbreviations: YLD=year of life lived with disability, GBD=Global Burden of Disease.

Values shown in brackets represent the ratio of observed YLDs to predicted YLDs on the basis of Socio-Demographic Index (SDI), rounded to two (2) digits. Color ranges (shown below) were calculated to place a roughly equal number of cells into each bin.

COLOR KEY:	[0.0-0.77]	(0.77-0.91]	(0.91-0.97]	(0.97-1.02]	(1.02-1.1]	(1.1-1.25]	(1.25-1.48]	(1.48-1.87]	1.87+

	1	2	3	4	5	6	7	8	9	10
Mexico	Headaches [0.97]	Neonatal [1.42]	Conduct [1.01]	Anxiety [0.72]	Depression [0.73]	Back Pain [0.65]	Epilepsy [1.83]	Asthma [0.93]	Bipolar [1.12]	Acne [1.38]
Aguascalientes	Headaches [0.97]	Neonatal [1.4]	Conduct [1.01]	Anxiety [0.71]	Back Pain [0.67]	Depression [0.72]	Epilepsy [1.94]	Asthma [1.0]	Bipolar [1.09]	Acne [1.3]
Baja CA	Headaches [0.97]	Neonatal [1.4]	Conduct [1.01]	Anxiety [0.71]	Back Pain [0.66]	Depression [0.67]	Epilepsy [1.87]	Drugs [2.07]	Acne [1.37]	Bipolar [1.11]
Baja CA Sur	Headaches [0.96]	Neonatal [1.47]	Conduct [1.01]	Anxiety [0.7]	Depression [0.73]	Back Pain [0.64]	Epilepsy [1.91]	Asthma [0.97]	Drugs [1.66]	Acne [1.33]
Campeche	Headaches [0.98]	Neonatal [1.57]	Conduct [1.01]	Depression [0.86]	Anxiety [0.74]	Back Pain [0.68]	Epilepsy [1.7]	Iron [0.64]	Asthma [0.94]	Bipolar [1.13]
Chiapas	Headaches [1.01]	Neonatal [1.48]	Conduct [1.01]	Anxiety [0.77]	Back Pain [0.72]	Depression [0.72]	Epilepsy [1.61]	Asthma [0.96]	Bipolar [1.15]	Dermatitis [0.74]
Chihuahua	Headaches [0.97]	Neonatal [1.3]	Conduct [1.01]	Anxiety [0.72]	Depression [0.76]	Back Pain [0.68]	Epilepsy [1.85]	Asthma [1.04]	Drugs [1.88]	Acne [1.37]
Coahuila	Headaches [0.97]	Neonatal [1.19]	Conduct [1.01]	Anxiety [0.71]	Back Pain [0.68]	Depression [0.72]	Epilepsy [1.87]	Asthma [1.02]	Bipolar [1.12]	Acne [1.36]
Colima	Headaches [0.97]	Neonatal [1.5]	Conduct [1.01]	Anxiety [0.71]	Back Pain [0.66]	Depression [0.71]	Epilepsy [1.92]	Asthma [0.94]	Bipolar [1.1]	Acne [1.31]
Mexico City	Headaches [0.96]	Neonatal [1.48]	Conduct [0.98]	Depression [0.74]	Back Pain [0.64]	Anxiety [0.63]	Epilepsy [2.28]	Dermatitis [0.86]	Acne [1.21]	Bipolar [1.09]
Durango	Headaches [0.98]	Neonatal [1.26]	Conduct [1.01]	Anxiety [0.73]	Back Pain [0.69]	Depression [0.72]	Epilepsy [1.79]	Dermatitis [0.94]	Asthma [1.02]	Bipolar [1.12]
Guanajuato	Headaches [0.98]	Neonatal [1.29]	Conduct [1.01]	Anxiety [0.74]	Depression [0.77]	Back Pain [0.69]	Epilepsy [1.83]	Asthma [1.01]	Bipolar [1.12]	Acne [1.41]
Guerrero	Headaches [1.0]	Neonatal [1.53]	Conduct [1.01]	Anxiety [0.77]	Back Pain [0.71]	Depression [0.71]	Epilepsy [1.61]	Asthma [0.96]	Drugs [1.86]	Bipolar [1.16]
Hidalgo	Headaches [0.99]	Neonatal [1.78]	Conduct [1.01]	Anxiety [0.76]	Back Pain [0.7]	Depression [0.7]	Epilepsy [1.7]	Asthma [1.01]	Drugs [1.8]	Bipolar [1.14]
Jalisco	Headaches [0.97]	Neonatal [1.35]	Conduct [1.01]	Anxiety [0.71]	Epilepsy [2.19]	Depression [0.76]	Back Pain [0.67]	Asthma [0.85]	Bipolar [1.09]	Acne [1.32]
Mexico	Headaches [0.93]	Neonatal [1.7]	Conduct [1.01]	Anxiety [0.72]	Depression [0.71]	Epilepsy [1.8]	Back Pain [0.51]	Bipolar [1.11]	Acne [1.39]	Asthma [0.84]
Michoacan	Headaches [1.0]	Neonatal [1.23]	Conduct [1.01]	Anxiety [0.76]	Back Pain [0.71]	Depression [0.73]	Epilepsy [1.62]	Asthma [1.0]	Bipolar [1.14]	Drugs [1.67]
Morelos	Headaches [0.98]	Neonatal [1.39]	Conduct [1.01]	Anxiety [0.72]	Depression [0.7]	Epilepsy [1.88]	Back Pain [0.58]	Asthma [0.88]	Bipolar [1.11]	Acne [1.38]
Nayarit	Headaches [0.98]	Neonatal [1.52]	Conduct [1.01]	Anxiety [0.73]	Back Pain [0.68]	Depression [0.7]	Epilepsy [1.74]	Asthma [0.97]	Bipolar [1.12]	Acne [1.43]
Nuevo Leon	Headaches [0.96]	Conduct [1.02]	Neonatal [1.14]	Anxiety [0.69]	Back Pain [0.66]	Depression [0.71]	Epilepsy [2.0]	Acne [1.33]	Bipolar [1.11]	Asthma [0.83]
Oaxaca	Headaches [1.01]	Neonatal [1.45]	Conduct [1.01]	Anxiety [0.77]	Back Pain [0.71]	Epilepsy [1.68]	Depression [0.7]	Asthma [0.97]	Bipolar [1.15]	Dermatitis [0.74]
Puebla	Headaches [1.0]	Neonatal [1.35]	Conduct [1.01]	Anxiety [0.76]	Back Pain [0.71]	Depression [0.72]	Epilepsy [1.31]	Asthma [1.0]	Bipolar [1.16]	Dermatitis [0.74]
Queretaro	Headaches [0.97]	Neonatal [1.49]	Conduct [1.01]	Anxiety [0.72]	Back Pain [0.67]	Depression [0.73]	Epilepsy [1.85]	Asthma [0.99]	Bipolar [1.1]	Acne [1.35]
Quintana Roo	Neonatal [1.81]	Headaches [0.98]	Conduct [1.01]	Depression [0.84]	Anxiety [0.73]	Back Pain [0.66]	Epilepsy [1.83]	Asthma [0.93]	Bipolar [1.13]	Acne [1.42]
San Luis Potosi	Headaches [0.98]	Neonatal [1.23]	Conduct [1.01]	Anxiety [0.74]	Depression [0.74]	Back Pain [0.69]	Epilepsy [1.78]	Asthma [1.0]	Bipolar [1.11]	Acne [1.42]
Sinaloa	Headaches [0.97]	Neonatal [1.28]	Conduct [1.01]	Anxiety [0.71]	Back Pain [0.66]	Depression [0.68]	Epilepsy [1.83]	Asthma [0.95]	Acne [1.4]	Bipolar [1.11]
Sonora	Headaches [0.97]	Neonatal [1.24]	Conduct [1.01]	Anxiety [0.71]	Back Pain [0.67]	Depression [0.72]	Epilepsy [1.93]	Asthma [0.99]	Acne [1.35]	Bipolar [1.11]
Tabasco	Headaches [0.98]	Neonatal [1.55]	Conduct [1.01]	Depression [0.81]	Anxiety [0.74]	Back Pain [0.69]	Epilepsy [1.74]	Iron [0.59]	Asthma [0.88]	Bipolar [1.11]
Tamaulipas	Headaches [0.97]	Neonatal [1.59]	Conduct [1.01]	Anxiety [0.71]	Back Pain [0.67]	Depression [0.68]	Epilepsy [1.82]	Asthma [0.94]	Drugs [1.71]	Acne [1.42]
Tlaxcala	Headaches [0.99]	Neonatal [1.53]	Conduct [1.01]	Anxiety [0.75]	Back Pain [0.7]	Epilepsy [1.8]	Depression [0.7]	Asthma [1.01]	Bipolar [1.13]	Acne [1.49]
Veracruz	Headaches [0.99]	Neonatal [1.53]	Conduct [1.01]	Anxiety [0.76]	Depression [0.75]	Back Pain [0.7]	Epilepsy [1.78]	Asthma [0.96]	Bipolar [1.15]	Dermatitis [0.74]

eFigure 9e. Leading ten causes of YLDs with the ratio of observed YLDs to YLDs expected on the basis of Socio-Demographic Index alone in 2017, 10-19 years, both sexes combined. The top ten causes contributing to YLDs are listed globally, by socio-demographic quintile, and then by GBD superregion, region, country, and subnationally where modeled. For each cell, the ratio of observed YLDs to YLDs expected on the basis of socio-demographic index (SDI) alone are listed. Abbreviations: YLD=year of life lived with disability, GBD=Global Burden of Disease.

Values shown in brackets represent the ratio of observed YLDs to predicted YLDs on the basis of Socio-Demographic Index (SDI), rounded to two (2) digits. Color ranges (shown below) were calculated to place a roughly equal number of cells into each bin.

COLOR KEY:	[0.0-0.77]	(0.77-0.91]	(0.91-0.97]	(0.97-1.02]	(1.02-1.1]	(1.1-1.25]	(1.25-1.48]	(1.48-1.87]	1.87+

	1	2	3	4	5	6	7	8	9	10
Yucatan	Headaches [0.98]	Neonatal [1.32]	Conduct [1.01]	Depression [0.82]	Anxiety [0.73]	Epilepsy [1.84]	Asthma [0.89]	Bipolar [1.15]	Acne [1.39]	Dermatitis [0.67]
Zacatecas	Headaches [0.98]	Conduct [1.01]	Neonatal [0.98]	Anxiety [0.74]	Back Pain [0.7]	Epilepsy [1.82]	Depression [0.69]	Asthma [1.03]	Bipolar [1.12]	Dermatitis [0.73]
Nicaragua	Headaches [1.04]	Neonatal [1.51]	Conduct [0.95]	Back Pain [0.95]	Depression [0.88]	Anxiety [0.73]	Dermatitis [1.1]	Epilepsy [1.27]	Asthma [1.06]	Diarrhea [1.63]
Panama	Headaches [0.99]	Neonatal [1.28]	Conduct [0.96]	Back Pain [0.82]	Dermatitis [1.31]	Depression [0.74]	Anxiety [0.65]	Iron [1.15]	Asthma [1.27]	Diarrhea [2.46]
Venezuela	Headaches [0.98]	Neonatal [1.4]	Conduct [0.96]	Back Pain [0.75]	Depression [0.79]	Anxiety [0.66]	Diarrhea [3.0]	Dermatitis [1.1]	Epilepsy [1.65]	Asthma [1.06]
**Tropical Latin America**	Headaches [1.43]	Back Pain [2.06]	Anxiety [1.22]	Depression [1.23]	Conduct [0.94]	Iron [1.63]	Neonatal [0.93]	Bipolar [1.73]	Asthma [1.28]	Acne [1.41]
Brazil	Headaches [1.43]	Back Pain [2.08]	Anxiety [1.21]	Depression [1.23]	Conduct [0.94]	Iron [1.69]	Neonatal [0.94]	Bipolar [1.72]	Asthma [1.27]	Acne [1.39]
Acre	Headaches [1.45]	Back Pain [2.2]	Iron [1.75]	Anxiety [1.29]	Depression [1.23]	Conduct [0.95]	Neonatal [1.01]	Bipolar [1.75]	Asthma [1.18]	Falls [1.91]
Alagoas	Headaches [1.49]	Back Pain [2.22]	Iron [1.33]	Depression [1.4]	Anxiety [1.33]	Conduct [0.95]	Neonatal [0.86]	Bipolar [1.82]	Asthma [1.17]	Congenital [0.96]
Amapa	Headaches [1.43]	Back Pain [2.01]	Anxiety [1.22]	Iron [2.0]	Depression [1.23]	Conduct [0.95]	Neonatal [0.99]	Bipolar [1.7]	Asthma [1.27]	Acne [1.36]
Amazonas	Headaches [1.44]	Back Pain [2.12]	Anxiety [1.25]	Iron [1.74]	Depression [1.13]	Conduct [0.95]	Neonatal [0.83]	Bipolar [1.73]	Asthma [1.25]	Acne [1.46]
Bahia	Headaches [1.46]	Back Pain [2.18]	Iron [1.63]	Anxiety [1.31]	Depression [1.11]	Conduct [0.86]	Neonatal [0.93]	Bipolar [1.8]	Asthma [1.14]	Dermatitis [0.99]
Ceara	Headaches [1.46]	Back Pain [2.17]	Iron [1.63]	Anxiety [1.3]	Depression [1.3]	Conduct [0.94]	Neonatal [0.88]	Bipolar [1.81]	Asthma [1.22]	Vit A [2.97]
Distrito Federal	Headaches [1.42]	Back Pain [1.9]	Anxiety [1.07]	Depression [1.17]	Conduct [0.99]	Neonatal [1.02]	Asthma [1.29]	Bipolar [1.6]	Acne [1.17]	Iron [2.22]
Espirito Santo	Headaches [1.43]	Back Pain [2.0]	Iron [2.54]	Anxiety [1.2]	Depression [1.26]	Conduct [0.95]	Neonatal [0.91]	Asthma [1.52]	Bipolar [1.69]	Acne [1.37]
Goias	Headaches [1.44]	Back Pain [2.11]	Anxiety [1.24]	Depression [1.28]	Conduct [0.95]	Iron [1.35]	Neonatal [0.85]	Bipolar [1.73]	Asthma [1.33]	Acne [1.44]
Maranhao	Headaches [1.5]	Back Pain [2.25]	Iron [1.55]	Anxiety [1.33]	Depression [1.19]	Conduct [0.95]	Neonatal [0.97]	Bipolar [1.85]	Asthma [1.18]	Vit A [1.98]
Mato Grosso	Headaches [1.43]	Back Pain [2.08]	Anxiety [1.22]	Depression [1.18]	Conduct [0.95]	Iron [1.38]	Neonatal [0.89]	Asthma [1.4]	Bipolar [1.69]	Acne [1.39]
Mato Grosso do Sul	Headaches [1.44]	Back Pain [2.12]	Anxiety [1.23]	Depression [1.32]	Conduct [0.95]	Neonatal [1.03]	Iron [1.26]	Bipolar [1.71]	Asthma [1.28]	Falls [1.61]
Minas Gerais	Headaches [1.42]	Back Pain [2.1]	Anxiety [1.23]	Depression [1.21]	Conduct [0.95]	Neonatal [0.99]	Iron [1.46]	Bipolar [1.74]	Asthma [1.31]	Acne [1.45]
Para	Headaches [1.47]	Back Pain [2.17]	Anxiety [1.32]	Conduct [0.95]	Depression [1.04]	Iron [1.03]	Neonatal [0.9]	Bipolar [1.8]	Asthma [1.28]	Diarrhea [1.5]
Paraiba	Headaches [1.47]	Back Pain [2.2]	Anxiety [1.32]	Iron [1.34]	Depression [1.24]	Conduct [0.95]	Neonatal [0.83]	Bipolar [1.81]	Asthma [1.24]	Vit A [2.4]
Parana	Headaches [1.43]	Back Pain [2.08]	Anxiety [1.2]	Depression [1.24]	Conduct [0.95]	Neonatal [0.96]	Iron [1.63]	Asthma [1.35]	Bipolar [1.72]	Acne [1.38]
Pernambuco	Headaches [1.46]	Back Pain [2.17]	Depression [1.41]	Iron [1.54]	Anxiety [1.31]	Conduct [0.95]	Neonatal [0.87]	Bipolar [1.79]	Asthma [1.28]	Congenital [1.01]
Piaui	Headaches [1.49]	Back Pain [2.26]	Anxiety [1.34]	Depression [1.17]	Conduct [0.95]	Neonatal [1.0]	Bipolar [1.85]	Asthma [1.26]	Iron [0.46]	Congenital [0.98]
Rio de Janeiro	Headaches [1.43]	Back Pain [1.77]	Anxiety [1.16]	Depression [1.14]	Neonatal [1.15]	Conduct [0.96]	Iron [1.47]	Bipolar [1.68]	Asthma [1.23]	Acne [1.27]
Rio Grande do Norte	Headaches [1.46]	Back Pain [2.16]	Anxiety [1.3]	Iron [1.58]	Depression [1.28]	Conduct [0.95]	Neonatal [0.8]	Bipolar [1.8]	Asthma [1.23]	Acne [1.64]
Rio Grande do Sul	Back Pain [2.7]	Headaches [1.43]	Anxiety [1.17]	Depression [1.29]	Conduct [0.88]	Neonatal [0.96]	Iron [1.51]	Epilepsy [2.18]	Congenital [1.61]	Asthma [1.34]
Rondonia	Headaches [1.45]	Back Pain [2.16]	Anxiety [1.27]	Depression [1.25]	Conduct [0.95]	Neonatal [1.05]	Iron [1.23]	Congenital [1.66]	Bipolar [1.75]	Asthma [1.27]
Roraima	Headaches [1.43]	Back Pain [2.08]	Iron [2.08]	Depression [1.41]	Anxiety [1.23]	Conduct [0.95]	Neonatal [1.04]	Bipolar [1.68]	Asthma [1.26]	Acne [1.41]
Santa Catarina	Headaches [1.7]	Back Pain [2.0]	Depression [1.37]	Anxiety [1.17]	Conduct [0.96]	Iron [1.74]	Neonatal [0.87]	Asthma [1.49]	Bipolar [1.71]	Acne [1.37]

eFigure 9e. Leading ten causes of YLDs with the ratio of observed YLDs to YLDs expected on the basis of Socio-Demographic Index alone in 2017, 10-19 years, both sexes combined. The top ten causes contributing to YLDs are listed globally, by socio-demographic quintile, and then by GBD superregion, region, country, and subnationally where modeled. For each cell, the ratio of observed YLDs to YLDs expected on the basis of socio-demographic index (SDI) alone are listed. Abbreviations: YLD=year of life lived with disability, GBD=Global Burden of Disease.

Values shown in brackets represent the ratio of observed YLDs to predicted YLDs on the basis of Socio-Demographic Index (SDI), rounded to two (2) digits. Color ranges (shown below) were calculated to place a roughly equal number of cells into each bin.

COLOR KEY:	[0.0-0.77]	(0.77-0.91]	(0.91-0.97]	(0.97-1.02]	(1.02-1.1]	(1.1-1.25]	(1.25-1.48]	(1.48-1.87]	1.87+

	1	2	3	4	5	6	7	8	9	10
Sao Paulo	Headaches [1.36]	Back Pain [2.0]	Depression [1.3]	Anxiety [1.13]	Conduct [0.97]	Neonatal [0.97]	Iron [1.8]	Bipolar [1.71]	Asthma [1.27]	Acne [1.29]
Sergipe	Headaches [1.45]	Back Pain [2.12]	Iron [1.86]	Anxiety [1.28]	Depression [1.23]	Conduct [0.95]	Neonatal [1.03]	Bipolar [1.77]	Asthma [1.34]	Acne [1.53]
Tocantins	Headaches [1.45]	Back Pain [2.17]	Anxiety [1.28]	Iron [1.58]	Depression [1.3]	Conduct [0.95]	Neonatal [1.01]	Bipolar [1.77]	Asthma [1.3]	Acne [1.57]
Paraguay	Headaches [1.44]	Back Pain [1.41]	Anxiety [1.3]	Depression [1.06]	Conduct [0.9]	Iron [1.09]	Asthma [1.63]	Neonatal [0.79]	Bipolar [1.7]	Dermatitis [1.0]
**North Africa and Middle East**	Headaches [1.02]	Back Pain [1.24]	Anxiety [1.15]	Depression [1.17]	Conduct [0.94]	Iron [1.42]	Neonatal [0.96]	Dermatitis [0.85]	Asthma [0.86]	Diarrhea [1.73]
Afghanistan	Headaches [1.19]	Back Pain [1.56]	Anxiety [1.31]	Depression [1.17]	Conduct [0.95]	Leish [1535.56]	Conflict Terror [27.09]	Neonatal [1.34]	Iron [0.3]	Dermatitis [1.08]
Algeria	Headaches [0.99]	Back Pain [1.13]	Anxiety [1.1]	Depression [1.11]	Conduct [0.95]	Neonatal [1.07]	Dermatitis [0.87]	Asthma [0.9]	Bipolar [0.99]	Congenital [0.89]
Bahrain	Headaches [0.98]	Back Pain [1.11]	Anxiety [1.08]	Depression [1.18]	Conduct [0.95]	Neonatal [1.05]	Dermatitis [1.01]	Iron [1.06]	Asthma [0.82]	Bipolar [1.02]
Egypt	Headaches [1.08]	Back Pain [1.4]	Iron [1.39]	Anxiety [1.08]	Conduct [0.94]	Neonatal [1.04]	Depression [1.0]	Asthma [1.04]	Bipolar [1.03]	Congenital [0.87]
Iran	Headaches [1.38]	Anxiety [1.45]	Depression [1.42]	Back Pain [1.09]	Neonatal [1.21]	Conduct [1.02]	Congenital [1.02]	Drugs [1.56]	Diarrhea [2.28]	Dermatitis [0.66]
Iraq	Headaches [1.01]	Back Pain [1.24]	Anxiety [1.16]	Neonatal [1.11]	Conduct [0.92]	Depression [1.06]	Iron [0.82]	Conflict Terror [552.98]	Asthma [0.92]	Dermatitis [0.79]
Jordan	Headaches [0.97]	Neonatal [1.38]	Back Pain [1.13]	Anxiety [1.09]	Depression [1.1]	Conduct [0.96]	Iron [1.55]	Asthma [1.04]	Dermatitis [0.88]	Bipolar [0.98]
Kuwait	Headaches [0.81]	Back Pain [1.05]	Anxiety [0.99]	Neonatal [1.15]	Depression [1.06]	Conduct [0.99]	Dermatitis [0.88]	Asthma [0.93]	Bipolar [0.92]	Congenital [0.91]
Lebanon	Headaches [0.98]	Anxiety [1.28]	Depression [1.05]	Neonatal [1.04]	Conduct [0.92]	Back Pain [0.84]	Dermatitis [1.05]	Asthma [1.09]	Bipolar [1.24]	Drugs [1.54]
Libya	Headaches [0.98]	Back Pain [1.1]	Anxiety [1.02]	Depression [1.11]	Conduct [0.97]	Neonatal [1.01]	Conflict Terror [2634.12]	Dermatitis [0.99]	Diarrhea [3.67]	Drugs [1.69]
Morocco	Headaches [1.02]	Depression [1.72]	Back Pain [1.46]	Anxiety [1.24]	Conduct [0.94]	Neonatal [1.06]	Iron [0.84]	Dermatitis [1.05]	Bipolar [1.06]	Diarrhea [1.4]
Palestine	Headaches [1.03]	Depression [1.66]	Back Pain [1.27]	Anxiety [1.25]	Conduct [0.94]	Neonatal [1.16]	Iron [0.65]	Conflict Terror [312.6]	Dermatitis [0.99]	Asthma [0.97]
Oman	Headaches [0.97]	Back Pain [1.08]	Anxiety [1.04]	Depression [1.09]	Conduct [0.97]	Neonatal [1.07]	Dermatitis [0.77]	Iron [1.24]	Diarrhea [2.64]	Blindness [1.72]
Qatar	Headaches [0.95]	Back Pain [1.12]	Anxiety [1.0]	Depression [1.07]	Conduct [1.0]	Neonatal [0.95]	Dermatitis [0.98]	Iron [1.38]	Bipolar [0.94]	Congenital [0.92]
Saudi Arabia	Headaches [1.03]	Back Pain [1.11]	Anxiety [1.01]	Depression [1.11]	Conduct [0.98]	Neonatal [0.72]	Dermatitis [0.97]	Bipolar [1.0]	Congenital [0.98]	Diarrhea [2.54]
Sudan	Iron [1.32]	Headaches [1.04]	Back Pain [1.39]	Anxiety [1.25]	Depression [1.13]	Conduct [0.94]	Neonatal [0.85]	Diarrhea [1.64]	Dermatitis [0.88]	Asthma [0.94]
Syria	Conflict Terror [2029.97]	Headaches [0.98]	Back Pain [1.2]	Anxiety [1.18]	Depression [1.11]	Conduct [0.94]	Iron [1.06]	Neonatal [0.9]	Asthma [0.83]	Congenital [0.89]
Tunisia	Headaches [0.99]	Anxiety [1.13]	Depression [1.15]	Back Pain [1.04]	Conduct [0.95]	Neonatal [1.04]	Dermatitis [1.03]	Bipolar [1.02]	Asthma [0.73]	Congenital [0.83]
Turkey	Headaches [0.8]	Back Pain [1.26]	Depression [1.11]	Conduct [0.94]	Anxiety [0.83]	Neonatal [0.74]	Dermatitis [0.79]	Bipolar [1.02]	Iron [0.97]	Asthma [0.74]
UAE	Headaches [0.97]	Back Pain [1.02]	Anxiety [0.96]	Conduct [0.96]	Neonatal [1.01]	Depression [0.85]	Dermatitis [0.97]	Asthma [1.04]	Drugs [1.56]	Bipolar [0.97]
Yemen	Iron [2.95]	Headaches [1.05]	Back Pain [1.3]	Anxiety [1.24]	Depression [1.16]	Conduct [0.92]	Neonatal [1.28]	Vit A [1.77]	Diarrhea [1.68]	Hemog [2.71]
**South Asia**	Iron [1.94]	Headaches [0.97]	Neonatal [1.51]	Conduct [0.86]	Anxiety [0.84]	Depression [0.75]	Back Pain [0.62]	Vit A [2.48]	Congenital [1.03]	Diarrhea [1.44]
Bangladesh	Headaches [1.07]	Iron [0.85]	Neonatal [1.56]	Anxiety [1.05]	Depression [1.0]	Conduct [0.8]	Back Pain [0.94]	Dermatitis [1.0]	Congenital [1.0]	Oth MSK [11.38]
Bhutan	Iron [2.02]	Headaches [1.01]	Neonatal [1.31]	Conduct [0.81]	Anxiety [0.92]	Back Pain [0.83]	Depression [0.77]	Diarrhea [2.06]	Dermatitis [1.02]	Epilepsy [1.0]

eFigure 9e. Leading ten causes of YLDs with the ratio of observed YLDs to YLDs expected on the basis of Socio-Demographic Index alone in 2017, 10-19 years, both sexes combined. The top ten causes contributing to YLDs are listed globally, by socio-demographic quintile, and then by GBD superregion, region, country, and subnationally where modeled. For each cell, the ratio of observed YLDs to YLDs expected on the basis of socio-demographic index (SDI) alone are listed. Abbreviations: YLD=year of life lived with disability, GBD=Global Burden of Disease.

Values shown in brackets represent the ratio of observed YLDs to predicted YLDs on the basis of Socio-Demographic Index (SDI), rounded to two (2) digits. Color ranges (shown below) were calculated to place a roughly equal number of cells into each bin.

COLOR KEY:	[0.0-0.77]	(0.77-0.91]	(0.91-0.97]	(0.97-1.02]	(1.02-1.1]	(1.1-1.25]	(1.25-1.48]	(1.48-1.87]	1.87+

	1	2	3	4	5	6	7	8	9	10
India	Iron [2.34]	Headaches [0.97]	Neonatal [1.54]	Conduct [0.87]	Anxiety [0.8]	Vit A [3.28]	Depression [0.72]	Back Pain [0.56]	ID [6.21]	Congenital [1.1]
Andhra Pradesh	Iron [1.84]	Headaches [0.99]	Neonatal [1.72]	Conduct [0.86]	Depression [0.94]	Anxiety [0.82]	Back Pain [0.6]	Vit A [2.52]	ID [5.27]	Congenital [1.07]
Arunachal Pradesh	Iron [2.19]	Headaches [0.98]	Neonatal [1.62]	Conduct [0.87]	Depression [0.88]	Anxiety [0.81]	Back Pain [0.58]	Vit A [2.28]	Congenital [1.09]	ID [5.13]
Assam	Iron [2.93]	Headaches [0.98]	Neonatal [1.45]	Conduct [0.86]	Anxiety [0.81]	Vit A [3.26]	Depression [0.75]	ID [6.6]	Congenital [1.1]	Hemog [2.57]
Bihar	Iron [1.62]	Headaches [1.01]	Neonatal [1.82]	Conduct [0.88]	Vit A [2.06]	Anxiety [0.81]	ID [6.78]	Depression [0.64]	Back Pain [0.61]	Diarrhea [1.34]
Chhattisgarh	Iron [2.61]	Headaches [0.99]	Neonatal [1.61]	Conduct [0.86]	Vit A [3.22]	Anxiety [0.75]	Depression [0.62]	Back Pain [0.6]	ID [5.7]	Congenital [1.07]
Delhi	Iron [7.64]	Headaches [0.92]	Neonatal [1.47]	Conduct [0.89]	Anxiety [0.68]	Depression [0.62]	Vit A [6.41]	Back Pain [0.39]	Diarrhea [2.4]	Congenital [1.01]
Goa	Neonatal [1.82]	Headaches [0.93]	Conduct [0.9]	Anxiety [0.63]	Depression [0.68]	Back Pain [0.49]	Iron [1.47]	Congenital [1.04]	Diarrhea [2.61]	Dermatitis [0.6]
Gujarat	Iron [4.26]	Headaches [0.95]	Neonatal [1.55]	Conduct [0.87]	Anxiety [0.76]	Vit A [3.97]	Depression [0.67]	Back Pain [0.57]	Congenital [1.09]	Hemog [3.46]
Haryana	Iron [4.26]	Headaches [0.94]	Neonatal [1.55]	Conduct [0.88]	Depression [0.84]	Anxiety [0.78]	Vit A [3.74]	Back Pain [0.58]	Congenital [1.07]	Hemog [3.56]
Himachal Pradesh	Iron [4.26]	Headaches [0.94]	Neonatal [1.69]	Conduct [0.87]	Anxiety [0.75]	Depression [0.71]	Back Pain [0.56]	Vit A [3.8]	Congenital [1.05]	Diarrhea [1.7]
Jammu & Kashmir	Headaches [0.96]	Neonatal [1.52]	Iron [1.43]	Conduct [0.88]	Anxiety [0.79]	Depression [0.67]	Vit A [3.79]	Back Pain [0.59]	ID [6.24]	Congenital [1.11]
Jharkhand	Iron [1.89]	Headaches [1.0]	Neonatal [1.74]	Conduct [0.95]	Anxiety [0.85]	Vit A [2.38]	Depression [0.68]	Back Pain [0.6]	ID [5.72]	Congenital [1.08]
Karnataka	Neonatal [1.82]	Headaches [0.95]	Iron [1.35]	Conduct [0.74]	Anxiety [0.81]	Depression [0.74]	Back Pain [0.58]	Vit A [3.16]	Congenital [1.06]	ID [5.33]
Kerala	Neonatal [2.08]	Headaches [0.95]	Conduct [0.85]	Anxiety [0.82]	Depression [0.71]	Iron [1.0]	Back Pain [0.53]	Congenital [1.06]	Diarrhea [1.84]	Dermatitis [0.63]
Madhya Pradesh	Iron [2.54]	Headaches [1.0]	Neonatal [1.54]	Conduct [0.86]	Vit A [2.52]	Anxiety [0.74]	Depression [0.67]	ID [6.24]	Back Pain [0.6]	Congenital [1.07]
Maharashtra	Iron [3.1]	Headaches [0.95]	Neonatal [1.66]	Conduct [0.92]	Anxiety [0.77]	Depression [0.76]	Vit A [4.16]	Back Pain [0.52]	Congenital [1.07]	Diarrhea [1.75]
Manipur	Headaches [0.97]	Neonatal [1.73]	Conduct [0.87]	Anxiety [0.89]	Depression [0.84]	Back Pain [0.58]	ID [6.85]	Vit A [3.08]	Congenital [1.11]	Diarrhea [1.56]
Meghalaya	Iron [2.47]	Headaches [0.97]	Neonatal [1.68]	Conduct [0.87]	Depression [0.87]	Anxiety [0.8]	Back Pain [0.58]	Vit A [2.93]	ID [6.19]	Congenital [1.1]
Mizoram	Headaches [0.96]	Neonatal [1.7]	Conduct [0.88]	Anxiety [0.78]	Iron [0.87]	Depression [0.66]	Back Pain [0.57]	Diarrhea [2.19]	ID [6.2]	Congenital [1.09]
Nagaland	Iron [3.29]	Neonatal [1.76]	Headaches [0.94]	Conduct [0.87]	Anxiety [0.76]	Depression [0.71]	Back Pain [0.54]	Vit A [4.29]	Congenital [1.07]	ID [5.54]
Odisha	Headaches [0.99]	Iron [1.23]	Neonatal [1.51]	Conduct [0.86]	Depression [0.89]	Anxiety [0.82]	Vit A [2.9]	Back Pain [0.6]	ID [6.26]	Diarrhea [1.62]
Punjab	Iron [3.72]	Headaches [0.93]	Neonatal [1.6]	Conduct [0.88]	Anxiety [0.73]	Back Pain [0.57]	Depression [0.61]	Vit A [3.9]	Congenital [1.08]	Diarrhea [1.6]
Rajasthan	Iron [1.59]	Headaches [0.98]	Neonatal [1.5]	Conduct [0.87]	Anxiety [0.84]	Vit A [2.3]	Depression [0.66]	ID [5.92]	Congenital [1.08]	Diarrhea [1.28]
Sikkim	Iron [2.85]	Headaches [0.96]	Neonatal [1.73]	Conduct [0.86]	Anxiety [0.77]	Depression [0.72]	Back Pain [0.57]	Diarrhea [1.99]	Congenital [1.05]	Vit A [3.23]
Tamil Nadu	Headaches [0.96]	Neonatal [1.62]	Iron [1.69]	Conduct [0.87]	Depression [0.95]	Anxiety [0.77]	Vit A [5.21]	Back Pain [0.57]	ID [5.3]	Diarrhea [1.6]
Telangana	Iron [2.8]	Headaches [0.98]	Neonatal [1.68]	Conduct [0.86]	Depression [0.92]	Anxiety [0.81]	Back Pain [0.59]	Vit A [3.03]	Congenital [1.08]	ID [5.29]
Tripura	Iron [1.61]	Headaches [0.98]	Neonatal [1.65]	Conduct [0.87]	Anxiety [0.81]	Depression [0.68]	Vit A [2.68]	Back Pain [0.59]	ID [6.21]	Congenital [1.09]
Uttar Pradesh	Iron [2.12]	Headaches [1.0]	Neonatal [1.46]	Conduct [0.87]	Anxiety [0.8]	Vit A [2.37]	Depression [0.66]	ID [6.43]	Back Pain [0.61]	Congenital [1.17]
Uttarakhand	Iron [3.4]	Headaches [0.96]	Neonatal [1.69]	Conduct [0.87]	Anxiety [0.78]	Depression [0.67]	Back Pain [0.59]	Vit A [3.23]	Diarrhea [1.83]	Congenital [1.08]
W Bengal	Headaches [0.99]	Iron [1.33]	Neonatal [1.5]	Conduct [0.86]	Anxiety [0.83]	Vit A [2.96]	Depression [0.69]	Back Pain [0.61]	ID [6.61]	Congenital [1.09]

eFigure 9e. Leading ten causes of YLDs with the ratio of observed YLDs to YLDs expected on the basis of Socio-Demographic Index alone in 2017, 10-19 years, both sexes combined. The top ten causes contributing to YLDs are listed globally, by socio-demographic quintile, and then by GBD superregion, region, country, and subnationally where modeled. For each cell, the ratio of observed YLDs to YLDs expected on the basis of socio-demographic index (SDI) alone are listed. Abbreviations: YLD=year of life lived with disability, GBD=Global Burden of Disease.

Values shown in brackets represent the ratio of observed YLDs to predicted YLDs on the basis of Socio-Demographic Index (SDI), rounded to two (2) digits. Color ranges (shown below) were calculated to place a roughly equal number of cells into each bin.

COLOR KEY:	[0.0-0.77]	(0.77-0.91]	(0.91-0.97]	(0.97-1.02]	(1.02-1.1]	(1.1-1.25]	(1.25-1.48]	(1.48-1.87]	1.87+

	1	2	3	4	5	6	7	8	9	10
UTs other than Delhi	Iron [3.18]	Neonatal [1.81]	Headaches [0.94]	Conduct [0.88]	Anxiety [0.74]	Depression [0.77]	Back Pain [0.54]	Vit A [4.13]	Congenital [1.06]	Dermatitis [0.63]
Nepal	Headaches [1.34]	Neonatal [1.87]	Back Pain [1.1]	Iron [0.61]	Anxiety [0.95]	Conduct [0.81]	Depression [0.8]	Dermatitis [0.99]	Epilepsy [1.03]	Congenital [0.83]
Pakistan	Headaches [0.89]	Neonatal [1.38]	Iron [0.84]	Conduct [0.82]	Anxiety [0.93]	Depression [0.74]	Back Pain [0.71]	Dermatitis [1.15]	Diarrhea [1.57]	Epilepsy [0.99]
**Southeast Asia, East Asia, and Oceania**	Headaches [0.61]	Neonatal [1.08]	Conduct [0.82]	Anxiety [0.72]	Back Pain [0.67]	Depression [0.65]	Congenital [0.89]	Hearing [1.57]	Scabies [3.38]	Asthma [0.67]
**East Asia**	Neonatal [1.36]	Anxiety [0.73]	Headaches [0.45]	Conduct [0.73]	Back Pain [0.63]	Depression [0.59]	Drugs [1.22]	Scabies [3.66]	Hearing [1.44]	Congenital [0.71]
China	Neonatal [1.39]	Anxiety [0.73]	Conduct [0.73]	Headaches [0.44]	Back Pain [0.62]	Depression [0.59]	Drugs [1.23]	Scabies [3.54]	Hearing [1.43]	Congenital [0.71]
N Korea	Headaches [0.58]	Back Pain [0.94]	Anxiety [0.92]	Iron [0.74]	Conduct [0.72]	Neonatal [0.72]	Depression [0.65]	Scabies [1.61]	Hearing [1.09]	Drugs [1.49]
Taiwan	Headaches [0.7]	Back Pain [0.85]	Anxiety [0.71]	Conduct [0.86]	Depression [0.53]	Neonatal [0.55]	Drugs [1.06]	Congenital [0.83]	Hearing [2.06]	Acne [0.54]
**Oceania**	Iron [1.02]	Headaches [0.73]	Conduct [0.87]	Anxiety [0.9]	Depression [0.87]	Back Pain [0.87]	Asthma [1.56]	Neonatal [0.79]	Dermatitis [0.83]	Diarrhea [1.24]
Am Samoa	Headaches [0.68]	Conduct [0.88]	Iron [1.79]	Anxiety [0.78]	Back Pain [0.75]	Asthma [1.56]	Depression [0.76]	Neonatal [0.72]	Diarrhea [2.97]	Dermatitis [0.78]
Micronesia	Iron [1.44]	Headaches [0.7]	Conduct [0.87]	Anxiety [0.88]	Depression [0.87]	Back Pain [0.82]	Asthma [1.45]	Neonatal [0.72]	Diarrhea [1.9]	Dermatitis [0.86]
Fiji	Headaches [0.69]	Iron [1.45]	Conduct [0.87]	Anxiety [0.83]	Depression [0.85]	Back Pain [0.72]	Asthma [1.37]	Neonatal [0.65]	Diarrhea [2.08]	Dermatitis [0.82]
Guam	Headaches [0.68]	Depression [0.97]	Conduct [0.91]	Diarrhea [6.97]	Anxiety [0.71]	Back Pain [0.68]	Asthma [1.39]	Neonatal [0.74]	Iron [2.64]	Dermatitis [0.75]
Kiribati	Iron [1.08]	Headaches [0.76]	Conduct [0.86]	Anxiety [0.91]	Depression [0.85]	Back Pain [0.85]	Asthma [1.56]	Neonatal [0.82]	Diarrhea [1.25]	Dermatitis [0.84]
Marshall	Iron [1.2]	Headaches [0.71]	Conduct [0.87]	Anxiety [0.89]	Depression [0.85]	Back Pain [0.79]	Neonatal [0.75]	Asthma [1.37]	Diarrhea [1.73]	Dermatitis [0.86]
N Mariana	Headaches [0.71]	Conduct [0.87]	Anxiety [0.74]	Depression [0.87]	Back Pain [0.73]	Neonatal [0.87]	Asthma [1.47]	Iron [2.08]	Diarrhea [3.71]	Dermatitis [0.73]
PNG	Iron [0.88]	Headaches [0.76]	Conduct [0.87]	Anxiety [0.9]	Depression [0.87]	Back Pain [0.9]	Asthma [1.61]	Neonatal [0.87]	Dermatitis [0.83]	Blindness [1.58]
Samoa	Headaches [0.7]	Iron [1.13]	Conduct [0.87]	Anxiety [0.88]	Back Pain [0.81]	Depression [0.84]	Neonatal [0.76]	Asthma [1.42]	Diarrhea [2.02]	Dermatitis [0.86]
Solomon	Iron [1.23]	Headaches [0.75]	Conduct [0.87]	Anxiety [0.9]	Depression [0.84]	Back Pain [0.84]	Neonatal [0.96]	Asthma [1.47]	Diarrhea [1.24]	Vit A [0.99]
Tonga	Headaches [0.69]	Conduct [0.87]	Asthma [1.84]	Anxiety [0.85]	Back Pain [0.81]	Depression [0.78]	Neonatal [0.71]	Iron [0.85]	Diarrhea [2.37]	Dermatitis [0.84]
Vanuatu	Iron [1.41]	Headaches [0.72]	Conduct [0.87]	Anxiety [0.9]	Back Pain [0.91]	Depression [0.85]	Asthma [1.62]	Neonatal [0.83]	Vit A [1.55]	Dermatitis [0.84]
**Southeast Asia**	Headaches [0.83]	Conduct [0.93]	Back Pain [0.74]	Anxiety [0.72]	Depression [0.73]	Neonatal [0.72]	Iron [0.72]	Congenital [1.15]	Hearing [1.71]	Asthma [0.88]
Cambodia	Iron [1.08]	Headaches [0.88]	Conduct [0.89]	Back Pain [1.05]	Depression [0.87]	Anxiety [0.81]	Neonatal [0.79]	Congenital [1.16]	Dermatitis [0.91]	Hearing [1.44]
Indonesia	Headaches [0.83]	Conduct [0.98]	Back Pain [0.74]	Anxiety [0.72]	Neonatal [0.74]	Depression [0.64]	Iron [0.83]	Asthma [0.93]	Congenital [1.02]	Hearing [1.67]
Laos	Headaches [0.86]	Iron [0.91]	Conduct [0.89]	Anxiety [1.01]	Back Pain [0.89]	Depression [0.79]	Congenital [1.18]	Dermatitis [0.92]	Neonatal [0.55]	Hearing [1.5]
Malaysia	Depression [1.38]	Headaches [0.76]	Conduct [0.92]	Anxiety [0.82]	Neonatal [0.8]	Back Pain [0.64]	Dermatitis [0.91]	Congenital [1.13]	Hearing [2.14]	Scabies [5.74]
Maldives	Headaches [0.82]	Conduct [0.91]	Iron [1.4]	Depression [0.85]	Anxiety [0.72]	Back Pain [0.7]	Neonatal [0.66]	Dermatitis [0.87]	Congenital [1.13]	Hearing [1.79]
Mauritius	Headaches [0.81]	Iron [3.15]	Depression [1.15]	Conduct [0.9]	Neonatal [0.83]	Back Pain [0.72]	Anxiety [0.68]	Dermatitis [0.83]	Asthma [0.92]	Congenital [1.11]
Myanmar	Headaches [0.85]	Conduct [0.89]	Iron [0.81]	Anxiety [0.8]	Congenital [1.7]	Neonatal [0.73]	Back Pain [0.63]	Hearing [1.71]	Dermatitis [0.93]	Depression [0.5]
Philippines	Headaches [0.83]	Conduct [0.9]	Back Pain [0.88]	Anxiety [0.76]	Depression [0.77]	Neonatal [0.76]	Dermatitis [1.0]	Asthma [1.06]	Congenital [1.2]	Hearing [1.67]
Sri Lanka	Headaches [0.81]	Conduct [0.91]	Depression [0.9]	Anxiety [0.71]	Neonatal [0.75]	Back Pain [0.63]	Congenital [1.33]	Iron [0.86]	Dermatitis [0.85]	Hearing [1.9]

eFigure 9e. Leading ten causes of YLDs with the ratio of observed YLDs to YLDs expected on the basis of Socio-Demographic Index alone in 2017, 10-19 years, both sexes combined. The top ten causes contributing to YLDs are listed globally, by socio-demographic quintile, and then by GBD superregion, region, country, and subnationally where modeled. For each cell, the ratio of observed YLDs to YLDs expected on the basis of socio-demographic index (SDI) alone are listed. Abbreviations: YLD=year of life lived with disability, GBD=Global Burden of Disease.

Values shown in brackets represent the ratio of observed YLDs to predicted YLDs on the basis of Socio-Demographic Index (SDI), rounded to two (2) digits. Color ranges (shown below) were calculated to place a roughly equal number of cells into each bin.

COLOR KEY:	[0.0-0.77]	(0.77-0.91]	(0.91-0.97]	(0.97-1.02]	(1.02-1.1]	(1.1-1.25]	(1.25-1.48]	(1.48-1.87]	1.87+

	1	2	3	4	5	6	7	8	9	10
Seychelles	Headaches [0.82]	Conduct [0.91]	Neonatal [0.82]	Anxiety [0.7]	Depression [0.77]	Back Pain [0.69]	Dermatitis [0.86]	Congenital [1.12]	Hearing [1.88]	Asthma [0.81]
Thailand	Headaches [0.92]	Conduct [0.9]	Anxiety [0.71]	Depression [0.79]	Back Pain [0.7]	Neonatal [0.67]	Dermatitis [0.93]	Drugs [1.83]	Congenital [1.16]	Scabies [4.09]
Timor-Leste	Iron [1.32]	Headaches [0.86]	Conduct [0.89]	Depression [0.94]	Anxiety [0.8]	Back Pain [0.77]	Neonatal [0.66]	LF [23.66]	Congenital [1.18]	Dermatitis [0.92]
Vietnam	Headaches [0.84]	Conduct [0.9]	Depression [0.75]	Back Pain [0.66]	Neonatal [0.62]	Anxiety [0.5]	Congenital [1.06]	Hearing [1.57]	Dermatitis [0.77]	Scabies [2.2]
**Sub-Saharan Africa**	Iron [1.14]	Headaches [0.8]	Conduct [0.94]	Depression [1.05]	Anxiety [0.96]	Back Pain [0.85]	Neonatal [0.95]	Vit A [1.57]	Dermatitis [1.01]	Epilepsy [1.2]
**Central Sub-Saharan Africa**	Iron [0.88]	Oncho [244.55]	Headaches [0.79]	Depression [1.21]	Conduct [0.93]	Anxiety [0.95]	Vit A [2.31]	Back Pain [0.9]	Neonatal [1.0]	Epilepsy [1.63]
Angola	Iron [1.62]	Headaches [0.78]	Depression [1.21]	Conduct [0.93]	Neonatal [1.19]	Anxiety [0.95]	Back Pain [0.88]	Epilepsy [1.76]	Dermatitis [1.08]	Diarrhea [1.52]
C African Rep	Iron [0.99]	Headaches [0.86]	Depression [1.25]	Conduct [0.94]	Anxiety [0.98]	Back Pain [0.98]	Vit A [1.15]	Epilepsy [1.49]	Oncho [44.14]	Malaria [2.36]
Congo	Iron [1.78]	Headaches [0.75]	Depression [1.21]	Conduct [0.94]	Neonatal [1.06]	Anxiety [0.93]	Back Pain [0.89]	Epilepsy [2.09]	Vit A [4.41]	Dermatitis [1.1]
Congo DR	Oncho [150.6]	Headaches [0.83]	Depression [1.19]	Conduct [0.94]	Vit A [1.71]	Anxiety [0.96]	Iron [0.47]	Back Pain [0.95]	Neonatal [1.16]	Epilepsy [1.46]
Eq Guinea	Headaches [0.72]	Iron [1.62]	Depression [1.22]	Conduct [0.98]	Anxiety [0.86]	Back Pain [0.82]	Epilepsy [2.27]	Neonatal [0.67]	Dermatitis [1.04]	Malaria [434.52]
Gabon	Iron [2.79]	Headaches [0.75]	Depression [1.22]	Neonatal [1.2]	Conduct [0.93]	Anxiety [0.87]	Back Pain [0.79]	Epilepsy [2.42]	Dermatitis [1.04]	Asthma [1.01]
**Eastern Sub-Saharan Africa**	Iron [0.78]	Depression [1.12]	Conduct [0.96]	Anxiety [1.02]	Headaches [0.61]	Neonatal [1.4]	Dermatitis [1.11]	Vit A [1.03]	Epilepsy [1.19]	Back Pain [0.6]
Burundi	Depression [1.13]	Conduct [0.94]	Anxiety [1.05]	Headaches [0.66]	Iron [0.36]	Dermatitis [1.17]	Conflict Terror [27.99]	Epilepsy [1.25]	Back Pain [0.67]	Neonatal [1.08]
Comoros	Neonatal [1.54]	Iron [0.67]	Conduct [0.92]	Headaches [0.61]	Anxiety [1.01]	Depression [0.99]	Dermatitis [1.16]	Back Pain [0.67]	Epilepsy [1.29]	Asthma [1.14]
Djibouti	Depression [1.14]	Conduct [0.95]	Neonatal [1.28]	Headaches [0.58]	Iron [0.7]	Anxiety [0.98]	Dermatitis [1.16]	Epilepsy [1.4]	Back Pain [0.59]	Congenital [1.06]
Eritrea	Iron [1.28]	Depression [1.15]	Neonatal [1.56]	Conduct [0.93]	Headaches [0.61]	Anxiety [1.01]	Dermatitis [1.15]	Epilepsy [1.31]	Back Pain [0.55]	Diarrhea [1.19]
Ethiopia	Conduct [1.01]	Depression [1.04]	Anxiety [1.04]	Neonatal [1.72]	Headaches [0.62]	Vit A [1.04]	Iron [0.35]	Dermatitis [1.12]	Diarrhea [1.12]	Back Pain [0.55]
Kenya	Neonatal [1.71]	Conduct [1.0]	Headaches [0.59]	Anxiety [0.99]	Depression [0.95]	Congenital [1.59]	Dermatitis [1.24]	Iron [0.49]	Back Pain [0.61]	Epilepsy [1.21]
Baringo	Neonatal [1.83]	Conduct [1.0]	Headaches [0.61]	Anxiety [0.99]	Depression [0.94]	Congenital [1.56]	Dermatitis [1.2]	Back Pain [0.63]	Diarrhea [1.34]	Epilepsy [1.06]
Bomet	Neonatal [2.08]	Conduct [1.0]	Headaches [0.6]	Anxiety [1.0]	Depression [0.94]	Congenital [1.58]	Dermatitis [1.22]	Back Pain [0.6]	Epilepsy [1.05]	HIV [81.58]
Bungoma	Neonatal [1.88]	Conduct [1.0]	Headaches [0.6]	Anxiety [0.99]	Depression [0.92]	Congenital [1.57]	Dermatitis [1.22]	Back Pain [0.6]	Iron [0.35]	Epilepsy [1.06]
Busia	Neonatal [1.82]	Iron [0.77]	Conduct [0.99]	Headaches [0.61]	Anxiety [0.99]	Depression [0.94]	Schisto [72.91]	Congenital [1.55]	Dermatitis [1.2]	Back Pain [0.61]
Elgeyo-Marakwet	Neonatal [2.03]	Conduct [1.0]	Headaches [0.59]	Anxiety [0.99]	Depression [0.95]	Congenital [1.58]	Dermatitis [1.22]	Back Pain [0.6]	Epilepsy [1.23]	Diarrhea [1.34]
Embu	Neonatal [1.9]	Conduct [1.0]	Headaches [0.59]	Anxiety [0.98]	Depression [0.96]	Congenital [1.59]	Dermatitis [1.23]	Back Pain [0.59]	Schisto [102.34]	Epilepsy [1.26]
Garissa	Iron [0.82]	Neonatal [2.54]	Conduct [1.03]	Headaches [0.64]	Anxiety [0.99]	Depression [0.9]	Congenital [1.6]	Dermatitis [1.19]	Back Pain [0.72]	Epilepsy [1.01]
HomaBay	Conduct [0.99]	HIV [130.62]	Headaches [0.62]	Anxiety [0.99]	Depression [0.99]	Iron [0.53]	Neonatal [1.04]	Congenital [1.55]	Schisto [45.19]	Dermatitis [1.2]
Isiolo	Conduct [1.01]	Neonatal [1.67]	Headaches [0.63]	Anxiety [1.0]	Depression [0.94]	Congenital [1.57]	Iron [0.37]	Dermatitis [1.19]	Back Pain [0.64]	Epilepsy [1.04]
Kajiado	Neonatal [2.08]	Conduct [1.0]	Headaches [0.59]	Anxiety [0.99]	Depression [0.96]	Congenital [1.59]	Dermatitis [1.23]	Back Pain [0.6]	Epilepsy [1.36]	Hearing [1.37]
Kakamega	Iron [0.81]	Neonatal [1.53]	Conduct [0.99]	Headaches [0.61]	Anxiety [1.0]	Depression [0.93]	Congenital [1.55]	Dermatitis [1.21]	Vit A [1.55]	Back Pain [0.61]
Kericho	Neonatal [2.21]	Conduct [1.0]	Headaches [0.59]	Anxiety [0.99]	Depression [0.95]	Congenital [1.6]	Dermatitis [1.23]	Back Pain [0.6]	Iron [0.38]	Epilepsy [1.13]

eFigure 9e. Leading ten causes of YLDs with the ratio of observed YLDs to YLDs expected on the basis of Socio-Demographic Index alone in 2017, 10-19 years, both sexes combined. The top ten causes contributing to YLDs are listed globally, by socio-demographic quintile, and then by GBD superregion, region, country, and subnationally where modeled. For each cell, the ratio of observed YLDs to YLDs expected on the basis of socio-demographic index (SDI) alone are listed. Abbreviations: YLD=year of life lived with disability, GBD=Global Burden of Disease.

Values shown in brackets represent the ratio of observed YLDs to predicted YLDs on the basis of Socio-Demographic Index (SDI), rounded to two (2) digits. Color ranges (shown below) were calculated to place a roughly equal number of cells into each bin.

COLOR KEY:	[0.0-0.77]	(0.77-0.91]	(0.91-0.97]	(0.97-1.02]	(1.02-1.1]	(1.1-1.25]	(1.25-1.48]	(1.48-1.87]	1.87+

	1	2	3	4	5	6	7	8	9	10
Kiambu	Neonatal [1.97]	Conduct [1.0]	Headaches [0.58]	Anxiety [0.98]	Depression [0.96]	Congenital [1.6]	Dermatitis [1.24]	Back Pain [0.57]	Epilepsy [1.44]	Hearing [1.48]
Kilifi	Neonatal [1.77]	Conduct [1.0]	Headaches [0.6]	Anxiety [0.99]	Depression [0.93]	Iron [0.54]	Congenital [1.56]	Dermatitis [1.21]	Back Pain [0.62]	Epilepsy [1.06]
Kirinyaga	Neonatal [1.95]	Conduct [0.99]	Headaches [0.59]	Anxiety [0.99]	Depression [0.96]	Congenital [1.6]	Dermatitis [1.23]	Back Pain [0.59]	Epilepsy [1.31]	Diarrhea [1.47]
Kisii	Neonatal [1.48]	Conduct [1.0]	Headaches [0.59]	Anxiety [0.99]	Depression [0.95]	Congenital [1.57]	Dermatitis [1.23]	Back Pain [0.58]	Epilepsy [1.23]	Iron [0.41]
Kisumu	Conduct [0.98]	Headaches [0.6]	Anxiety [1.0]	Iron [0.77]	Neonatal [1.03]	Depression [0.84]	Congenital [1.56]	HIV [129.8]	Back Pain [0.6]	Epilepsy [1.31]
Kitui	Neonatal [2.16]	Conduct [1.0]	Headaches [0.61]	Anxiety [1.0]	Depression [0.94]	Congenital [1.57]	Dermatitis [1.21]	Back Pain [0.62]	Epilepsy [1.11]	Diarrhea [1.27]
Kwale	Iron [0.98]	Neonatal [1.77]	Conduct [0.99]	Headaches [0.6]	Anxiety [0.99]	Depression [0.94]	Schisto [83.36]	Congenital [1.54]	Dermatitis [1.21]	Back Pain [0.62]
Laikipia	Neonatal [2.08]	Conduct [1.01]	Headaches [0.58]	Anxiety [0.97]	Depression [0.96]	Congenital [1.6]	Dermatitis [1.24]	Back Pain [0.6]	Epilepsy [1.28]	Hearing [1.44]
Lamu	Neonatal [1.52]	Conduct [1.0]	Headaches [0.61]	Anxiety [1.0]	Depression [0.95]	Congenital [1.56]	Dermatitis [1.2]	Conflict Terror [122.44]	Back Pain [0.61]	Epilepsy [1.22]
Machakos	Neonatal [1.97]	Conduct [1.0]	Headaches [0.59]	Anxiety [0.98]	Depression [0.95]	Congenital [1.58]	Dermatitis [1.22]	Back Pain [0.6]	Epilepsy [1.22]	Hearing [1.37]
Makueni	Neonatal [2.0]	Conduct [0.99]	Headaches [0.61]	Anxiety [0.99]	Depression [0.95]	Congenital [1.57]	Dermatitis [1.2]	Back Pain [0.63]	HIV [85.31]	Epilepsy [1.09]
Mandera	Conduct [1.04]	Neonatal [2.1]	Iron [0.49]	Headaches [0.66]	Anxiety [0.99]	Depression [0.93]	Congenital [1.61]	Dermatitis [1.18]	Back Pain [0.73]	Epilepsy [0.95]
Marsabit	Neonatal [2.69]	Conduct [1.02]	Headaches [0.65]	Anxiety [1.01]	Depression [0.94]	Iron [0.37]	Congenital [1.58]	Dermatitis [1.19]	Back Pain [0.68]	Epilepsy [0.97]
Meru	Neonatal [1.94]	Conduct [0.99]	Headaches [0.6]	Anxiety [0.99]	Depression [0.96]	Congenital [1.59]	Dermatitis [1.22]	Back Pain [0.6]	Epilepsy [1.21]	Diarrhea [1.38]
Migori	Conduct [1.0]	Iron [0.61]	Headaches [0.62]	Anxiety [1.0]	Depression [0.97]	HIV [84.91]	Neonatal [0.98]	Congenital [1.55]	Vit A [1.43]	Dermatitis [1.2]
Mombasa	Neonatal [1.52]	Iron [1.29]	Conduct [0.98]	Headaches [0.59]	Anxiety [0.99]	Depression [0.97]	Congenital [1.58]	Dermatitis [1.23]	Back Pain [0.57]	Epilepsy [1.39]
Murang’a	Neonatal [1.98]	Conduct [1.0]	Headaches [0.59]	Anxiety [0.98]	Depression [0.95]	Congenital [1.61]	Dermatitis [1.22]	Back Pain [0.6]	Epilepsy [1.21]	Hearing [1.38]
Nairobi	Neonatal [1.72]	Conduct [0.99]	Headaches [0.58]	Anxiety [0.9]	Depression [0.95]	Dermatitis [1.51]	Congenital [1.5]	Epilepsy [1.87]	Back Pain [0.52]	Hearing [1.69]
Nakuru	Neonatal [1.74]	Conduct [1.0]	Headaches [0.58]	Anxiety [0.98]	Depression [0.95]	Epilepsy [1.78]	Congenital [1.61]	Dermatitis [1.23]	Back Pain [0.6]	Diarrhea [1.44]
Nandi	Neonatal [2.11]	Conduct [1.0]	Headaches [0.6]	Anxiety [0.99]	Depression [0.95]	Congenital [1.58]	Dermatitis [1.22]	Back Pain [0.6]	Epilepsy [1.17]	Hearing [1.34]
Narok	Neonatal [2.64]	Conduct [1.0]	Headaches [0.63]	Anxiety [1.0]	Depression [0.94]	Congenital [1.56]	Dermatitis [1.2]	Back Pain [0.65]	Epilepsy [1.11]	Diarrhea [1.18]
Nyamira	Neonatal [1.7]	Conduct [1.0]	Headaches [0.59]	Anxiety [0.99]	Depression [0.97]	Congenital [1.59]	Dermatitis [1.24]	Epilepsy [1.51]	Back Pain [0.58]	Diarrhea [1.74]
Nyandarua	Neonatal [2.06]	Conduct [1.0]	Headaches [0.59]	Anxiety [0.98]	Depression [0.99]	Congenital [1.61]	Dermatitis [1.23]	Epilepsy [1.51]	Back Pain [0.61]	Hearing [1.39]
Nyeri	Neonatal [1.92]	Conduct [1.0]	Headaches [0.59]	Anxiety [0.98]	Depression [0.96]	Congenital [1.61]	Dermatitis [1.23]	Back Pain [0.59]	Epilepsy [1.45]	Hearing [1.42]
Samburu	Neonatal [3.13]	Conduct [1.03]	Headaches [0.66]	Anxiety [1.01]	Depression [0.93]	Iron [0.44]	Congenital [1.6]	Dermatitis [1.2]	Back Pain [0.7]	Diarrhea [1.05]
Siaya	Conduct [0.99]	Headaches [0.61]	Anxiety [1.0]	Depression [0.95]	Neonatal [1.13]	Iron [0.52]	Congenital [1.55]	Dermatitis [1.21]	Back Pain [0.62]	HIV [85.06]
TaitaTaveta	Neonatal [1.76]	Conduct [1.0]	Headaches [0.58]	Anxiety [0.98]	Depression [0.95]	Congenital [1.58]	Dermatitis [1.22]	Back Pain [0.63]	Epilepsy [1.43]	Schisto [83.81]
TanaRiver	Iron [0.77]	Neonatal [2.1]	Conduct [1.0]	Headaches [0.63]	Anxiety [1.0]	Depression [0.94]	Schisto [35.45]	Congenital [1.56]	Vit A [1.18]	Dermatitis [1.2]
TharakaNithi	Neonatal [1.48]	Conduct [0.99]	Headaches [0.59]	Anxiety [0.99]	Depression [0.97]	Congenital [1.57]	Dermatitis [1.23]	Back Pain [0.59]	Epilepsy [1.38]	Hearing [1.39]
TransNzoia	Neonatal [1.99]	Conduct [1.0]	Headaches [0.6]	Anxiety [0.99]	Depression [0.96]	Congenital [1.57]	Dermatitis [1.22]	Back Pain [0.61]	Diarrhea [1.43]	Epilepsy [1.08]
Turkana	Neonatal [2.63]	Iron [0.61]	Conduct [1.03]	Headaches [0.66]	Anxiety [1.0]	Depression [0.92]	Congenital [1.62]	Dermatitis [1.19]	Back Pain [0.72]	Vit A [0.75]

eFigure 9e. Leading ten causes of YLDs with the ratio of observed YLDs to YLDs expected on the basis of Socio-Demographic Index alone in 2017, 10-19 years, both sexes combined. The top ten causes contributing to YLDs are listed globally, by socio-demographic quintile, and then by GBD superregion, region, country, and subnationally where modeled. For each cell, the ratio of observed YLDs to YLDs expected on the basis of socio-demographic index (SDI) alone are listed. Abbreviations: YLD=year of life lived with disability, GBD=Global Burden of Disease.

Values shown in brackets represent the ratio of observed YLDs to predicted YLDs on the basis of Socio-Demographic Index (SDI), rounded to two (2) digits. Color ranges (shown below) were calculated to place a roughly equal number of cells into each bin.

COLOR KEY:									
[0.0-0.77]	(0.77-0.91]	(0.91-0.97]	(0.97-1.02]	(1.02-1.1]	(1.1-1.25]	(1.25-1.48]	(1.48-1.87]	1.87+	

	1	2	3	4	5	6	7	8	9	10
UasinGishu	Neonatal [1.83]	Conduct [0.99]	Headaches [0.59]	Anxiety [0.99]	Dermatitis [1.8]	Depression [0.95]	Congenital [1.58]	Back Pain [0.59]	Epilepsy [1.22]	Iron [0.43]
Vihiga	Neonatal [1.77]	Conduct [0.99]	Headaches [0.61]	Anxiety [0.99]	Depression [0.95]	Iron [0.48]	Congenital [1.56]	Dermatitis [1.21]	Back Pain [0.61]	Epilepsy [1.2]
Wajir	Neonatal [2.99]	Conduct [1.06]	Headaches [0.67]	Anxiety [0.99]	Depression [0.9]	Congenital [1.66]	Dermatitis [1.19]	Iron [0.28]	Back Pain [0.76]	Vit A [0.55]
WestPokot	Neonatal [2.51]	Conduct [1.01]	Headaches [0.63]	Anxiety [1.01]	Depression [0.94]	Congenital [1.56]	Dermatitis [1.2]	Back Pain [0.65]	Iron [0.3]	Diarrhea [1.17]
Madagascar	Iron [0.91]	Congenital [2.74]	Depression [1.13]	Conduct [0.93]	Headaches [0.64]	Anxiety [1.03]	Neonatal [1.43]	Epilepsy [1.3]	Hearing [1.78]	Dermatitis [1.16]
Malawi	Iron [1.3]	Conduct [0.93]	Headaches [0.64]	Anxiety [1.03]	Depression [0.99]	HIV [70.75]	Neonatal [1.22]	Vit A [0.98]	Dermatitis [1.16]	Back Pain [0.7]
Mozambique	Iron [0.91]	Depression [1.14]	Conduct [0.93]	Anxiety [1.03]	Headaches [0.64]	Vit A [1.25]	HIV [59.94]	Epilepsy [1.47]	Neonatal [1.18]	Back Pain [0.65]
Rwanda	Depression [1.24]	Iron [0.6]	Conduct [0.93]	Headaches [0.62]	Anxiety [1.03]	Neonatal [1.3]	Asthma [1.39]	Epilepsy [1.28]	Back Pain [0.63]	Congenital [1.05]
Somalia	Iron [1.03]	Depression [1.11]	Conduct [0.96]	Headaches [0.68]	Anxiety [1.03]	Neonatal [1.75]	Dermatitis [1.17]	Vit A [0.65]	Epilepsy [1.11]	Back Pain [0.7]
S Sudan	Iron [0.9]	Oncho [79.0]	Depression [1.1]	Conduct [0.96]	Headaches [0.65]	Anxiety [1.02]	Neonatal [1.45]	Vit A [0.76]	Dermatitis [1.16]	Diarrhea [1.26]
Tanzania	Iron [1.34]	Depression [1.14]	Conduct [0.92]	Anxiety [1.02]	Neonatal [1.21]	Headaches [0.5]	Asthma [1.34]	Epilepsy [1.24]	Dermatitis [0.99]	Back Pain [0.56]
Uganda	Iron [1.11]	Depression [1.59]	Conduct [0.93]	Headaches [0.61]	Anxiety [0.98]	Neonatal [1.22]	Epilepsy [1.47]	Dermatitis [1.16]	Vit A [1.1]	Back Pain [0.66]
Zambia	Headaches [0.77]	Conduct [0.92]	Iron [0.69]	Depression [1.04]	Anxiety [1.01]	Neonatal [0.95]	Epilepsy [1.6]	Dermatitis [1.17]	HIV [92.44]	Vit A [1.41]
**Southern Sub-Saharan Africa**	Headaches [0.75]	Conduct [0.99]	Anxiety [0.95]	Depression [0.98]	Neonatal [0.91]	HIV [297.35]	Dermatitis [1.31]	Iron [0.95]	Back Pain [0.53]	Congenital [1.23]
Botswana	Iron [2.73]	Headaches [0.74]	Neonatal [1.23]	Conduct [0.95]	Depression [1.07]	Anxiety [0.89]	Dermatitis [1.32]	Back Pain [0.58]	Diarrhea [1.95]	Asthma [0.79]
Lesotho	Depression [1.76]	Headaches [0.79]	Conduct [0.93]	Iron [0.77]	Anxiety [0.99]	HIV [171.15]	Dermatitis [1.38]	Neonatal [0.81]	Back Pain [0.63]	Diarrhea [1.55]
Namibia	Headaches [0.75]	Conduct [0.94]	Iron [1.32]	Anxiety [0.94]	Neonatal [1.0]	Depression [0.93]	Dermatitis [1.38]	Back Pain [0.62]	HIV [210.06]	Diarrhea [2.06]
S Africa	Headaches [0.75]	Conduct [1.02]	Anxiety [0.97]	Depression [1.0]	Neonatal [0.92]	HIV [396.28]	Dermatitis [1.27]	Congenital [1.48]	Back Pain [0.49]	Hearing [1.79]
Swaziland	Headaches [0.76]	Depression [1.14]	Conduct [0.94]	Anxiety [0.97]	HIV [246.08]	Iron [0.83]	Dermatitis [1.4]	Neonatal [0.76]	Back Pain [0.58]	Diarrhea [2.04]
Zimbabwe	Headaches [0.8]	Iron [0.73]	Conduct [0.93]	Anxiety [0.9]	Neonatal [1.05]	Dermatitis [1.38]	Depression [0.76]	Back Pain [0.67]	HIV [80.62]	Diarrhea [1.37]
**Western Sub-Saharan Africa**	Iron [1.53]	Headaches [1.02]	Conduct [0.93]	Back Pain [1.12]	Depression [0.93]	Anxiety [0.9]	Vit A [1.61]	Congenital [1.16]	Epilepsy [1.09]	Neonatal [0.66]
Benin	Iron [1.34]	Headaches [1.0]	Conduct [0.93]	Back Pain [1.11]	Anxiety [0.91]	Depression [0.86]	Vit A [1.42]	Neonatal [1.14]	Malaria [4.47]	Epilepsy [1.17]
Burkina Faso	Iron [1.22]	Headaches [1.1]	Conduct [0.96]	Vit A [1.19]	Anxiety [0.93]	Depression [0.86]	Back Pain [1.0]	Malaria [1.36]	Diarrhea [1.13]	Hemog [1.6]
Cameroon	Headaches [0.97]	Iron [0.8]	Conduct [0.94]	Back Pain [1.04]	Anxiety [0.9]	Depression [0.9]	Oncho [182.57]	Vit A [1.91]	Epilepsy [1.33]	Diarrhea [1.56]
Cape Verde	Headaches [0.97]	Neonatal [1.39]	Iron [1.18]	Conduct [0.93]	Back Pain [0.96]	Depression [1.0]	Anxiety [0.9]	Epilepsy [1.31]	Diarrhea [1.72]	Dermatitis [0.95]
Chad	Iron [1.44]	Headaches [1.12]	Back Pain [1.38]	Conduct [0.96]	Depression [0.97]	Anxiety [0.94]	Vit A [0.79]	Diarrhea [1.23]	Dermatitis [0.94]	Epilepsy [0.94]
Cote d'Ivoire	Iron [1.13]	Headaches [1.01]	Conduct [0.95]	Back Pain [0.96]	Anxiety [0.9]	Depression [0.77]	Vit A [1.25]	Epilepsy [1.2]	Neonatal [0.81]	Dermatitis [0.93]
Gambia	Iron [1.86]	Headaches [1.02]	Vit A [2.15]	Depression [1.13]	Neonatal [1.59]	Conduct [0.92]	Back Pain [1.04]	Anxiety [0.91]	Diarrhea [1.37]	Dermatitis [0.93]
Ghana	Iron [2.26]	Headaches [0.96]	Conduct [0.93]	Anxiety [0.89]	Neonatal [0.94]	Depression [0.87]	Back Pain [0.83]	Vit A [3.41]	Epilepsy [1.38]	Diarrhea [1.55]
Guinea	Iron [0.93]	Headaches [1.08]	Conduct [0.95]	Back Pain [1.11]	Anxiety [0.93]	Depression [0.86]	Vit A [0.88]	Malaria [1.61]	Neonatal [0.95]	Dermatitis [0.94]
Guinea-Bissau	Iron [1.29]	Headaches [1.06]	Conduct [0.94]	Back Pain [1.09]	Anxiety [0.92]	Depression [0.87]	Vit A [1.13]	Neonatal [1.15]	LF [8.03]	Epilepsy [1.14]

eFigure 9e. Leading ten causes of YLDs with the ratio of observed YLDs to YLDs expected on the basis of Socio-Demographic Index alone in 2017, 10-19 years, both sexes combined. The top ten causes contributing to YLDs are listed globally, by socio-demographic quintile, and then by GBD superregion, region, country, and subnationally where modeled. For each cell, the ratio of observed YLDs to YLDs expected on the basis of socio-demographic index (SDI) alone are listed. Abbreviations: YLD=year of life lived with disability, GBD=Global Burden of Disease.

Values shown in brackets represent the ratio of observed YLDs to predicted YLDs on the basis of Socio-Demographic Index (SDI), rounded to two (2) digits. Color ranges (shown below) were calculated to place a roughly equal number of cells into each bin.

COLOR KEY:	[0.0-0.77]	(0.77-0.91]	(0.91-0.97]	(0.97-1.02]	(1.02-1.1]	(1.1-1.25]	(1.25-1.48]	(1.48-1.87]	1.87+

	1	2	3	4	5	6	7	8	9	10
Liberia	Oncho [194.57]	Iron [0.93]	Headaches [1.06]	Conduct [0.94]	Back Pain [1.09]	Depression [0.95]	Anxiety [0.92]	LF [7.85]	Neonatal [1.21]	Epilepsy [1.33]
Mali	Iron [1.62]	Headaches [1.12]	Vit A [1.36]	Conduct [0.95]	Anxiety [0.94]	Back Pain [0.94]	Depression [0.69]	LF [4.91]	Malaria [0.97]	Diarrhea [1.1]
Mauritania	Iron [1.19]	Headaches [0.98]	Conduct [0.93]	Neonatal [1.22]	Back Pain [0.96]	Anxiety [0.91]	Depression [0.74]	Epilepsy [1.25]	Diarrhea [1.55]	Dermatitis [0.95]
Niger	Iron [0.94]	Headaches [1.17]	Conduct [0.97]	Back Pain [1.24]	Anxiety [0.95]	Depression [0.83]	Vit A [0.74]	Diarrhea [0.97]	Malaria [0.77]	Dermatitis [0.95]
Nigeria	Iron [1.99]	Headaches [1.01]	Back Pain [1.24]	Conduct [0.92]	Depression [1.03]	Anxiety [0.9]	Congenital [1.41]	Vit A [1.96]	Hemog [3.02]	Hearing [1.54]
Sao Tome Principe	Headaches [0.97]	Iron [0.91]	Conduct [0.94]	Neonatal [1.23]	Back Pain [0.92]	Anxiety [0.9]	Depression [0.85]	Epilepsy [1.27]	Vit A [1.73]	Dermatitis [0.95]
Senegal	Iron [1.62]	Headaches [1.04]	Conduct [0.95]	Neonatal [1.5]	Anxiety [0.91]	Back Pain [0.88]	Depression [0.78]	Vit A [1.14]	Diarrhea [1.4]	Epilepsy [1.09]
Sierra Leone	Iron [0.97]	Headaches [1.07]	Conduct [0.93]	Back Pain [1.1]	Anxiety [0.92]	Depression [0.89]	Malaria [3.31]	Vit A [1.01]	Oncho [40.94]	Epilepsy [1.14]
Togo	Iron [1.04]	Headaches [1.0]	Conduct [0.94]	Back Pain [1.04]	Anxiety [0.9]	Depression [0.87]	Malaria [11.94]	Vit A [1.62]	Neonatal [1.07]	Epilepsy [1.21]

eFigure 10: Percent Change in Maternal Mortality Rate vs. Percent Change All−cause Child DALYs Rate, 1990 to 2017

© 2019 GBD 2017 Child and Adolescent Health Collaborators. JAMA Pediatrics.

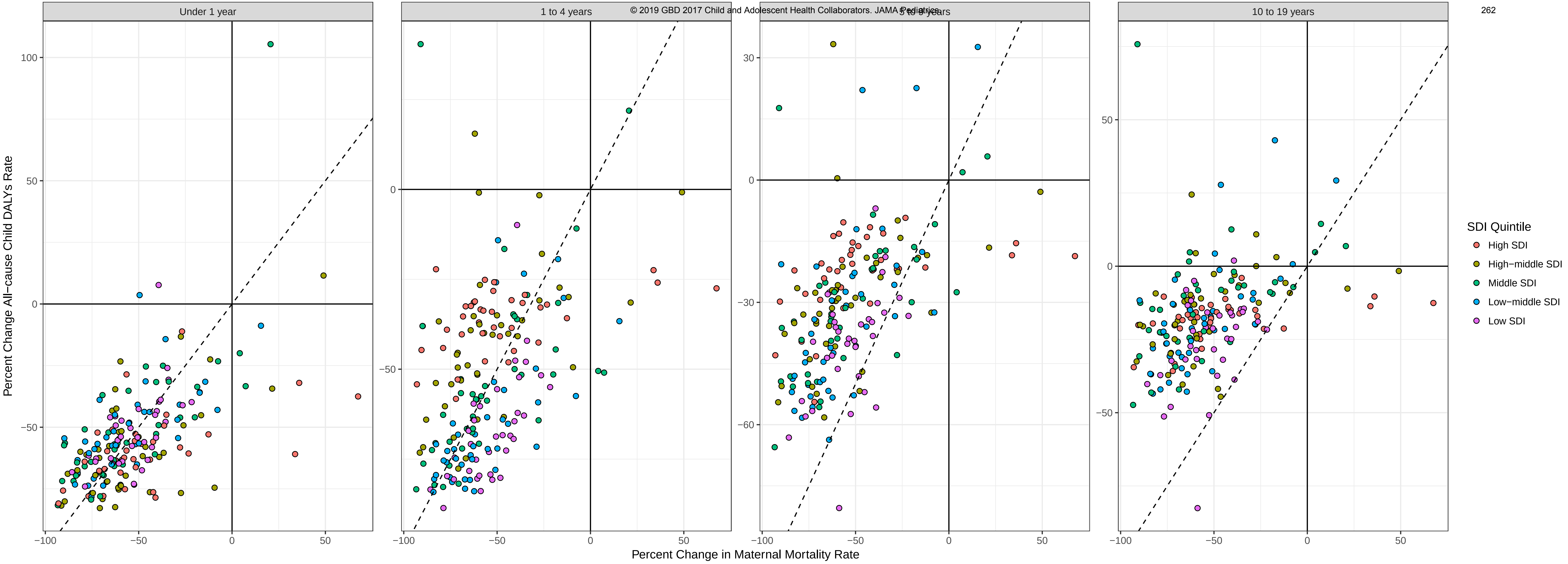

The percent change in maternal mortality rate (MMR) is plotted against the percent change in all−cause child disability−adjusted life year (DALY) rate by country. Panel a) displays the scatter plot of percent change in MMR from 1990 to 2017 against childhood all−cause DALY rate from 1990 to 2017; panel b) displays the scatter plot of percent change in MMR from 1990 to 2017 against childhood all−cause DALY rate from 1990 to 2000; and panel c) displays the scatter plot of percent change in MMR from 1990 to 2017 against childhood all−cause DALY rate from 2000 to 2017. Within each panel, patterns for all children and adolescents less than 20 are in the left sub−panel, under 1 in the second sub−panel, 1 to 4 in the third sub−panel, 5 to 9 in the fourth sub−panel and 10 to 19 in the right sub−panel. In each, every country is colored by its socio−demographic index (SDI) quintile.

eFigure 10: Percent Change in Maternal Mortality Rate vs. Percent Change All−cause Child DALYs Rate, 1990 to 2000

© 2019 GBD 2017 Child and Adolescent Health Collaborators. JAMA Pediatrics.

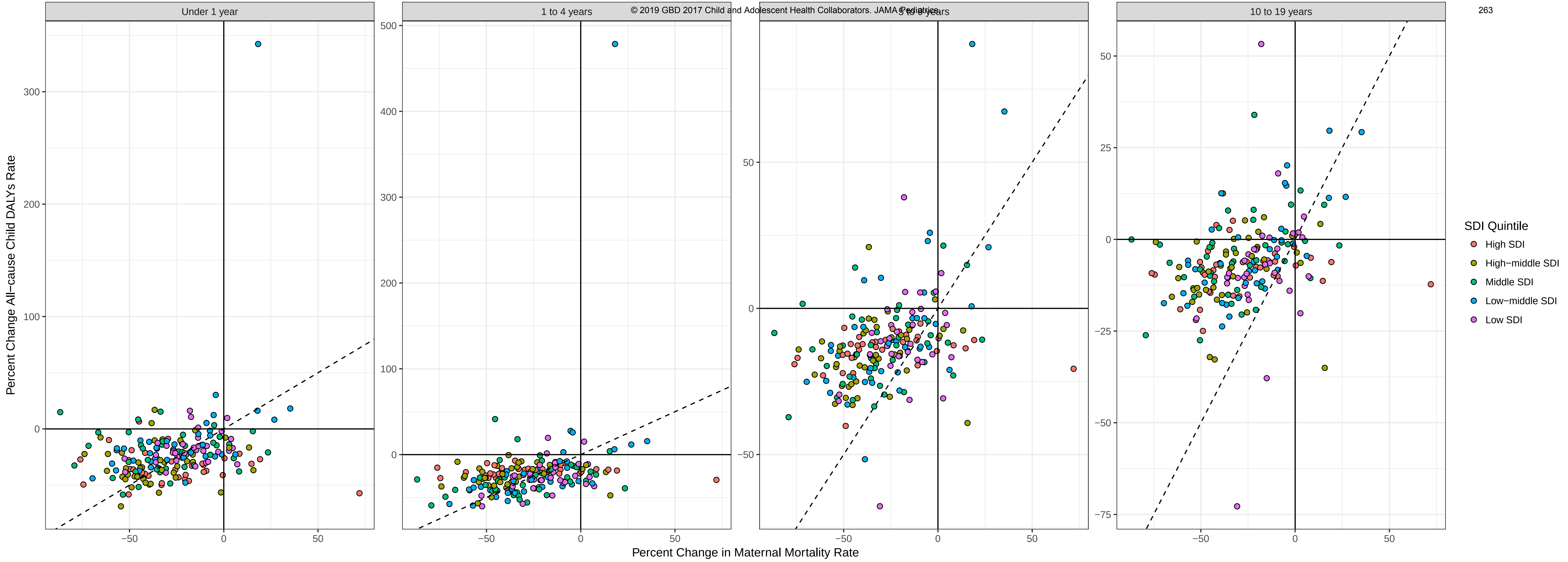

The percent change in maternal mortality rate (MMR) is plotted against the percent change in all−cause child disability−adjusted life year (DALY) rate by country. Panel a) displays the scatter plot of percent change in MMR from 1990 to 2017 against childhood all−cause DALY rate from 1990 to 2017; panel b) displays the scatter plot of percent change in MMR from 1990 to 2017 against childhood all−cause DALY rate from 1990 to 2000; and panel c) displays the scatter plot of percent change in MMR from 1990 to 2017 against childhood all−cause DALY rate from 2000 to 2017. Within each panel, patterns for all children and adolescents less than 20 are in the left sub−panel, under 1 in the second sub−panel, 1 to 4 in the third sub−panel, 5 to 9 in the fourth sub−panel and 10 to 19 in the right sub−panel. In each, every country is colored by its socio−demographic index (SDI) quintile.

eFigure 10: Percent Change in Maternal Mortality Rate vs. Percent Change All−cause Child DALYs Rate, 2000 to 2017

© 2019 GBD 2017 Child and Adolescent Health Collaborators. JAMA Pediatrics.

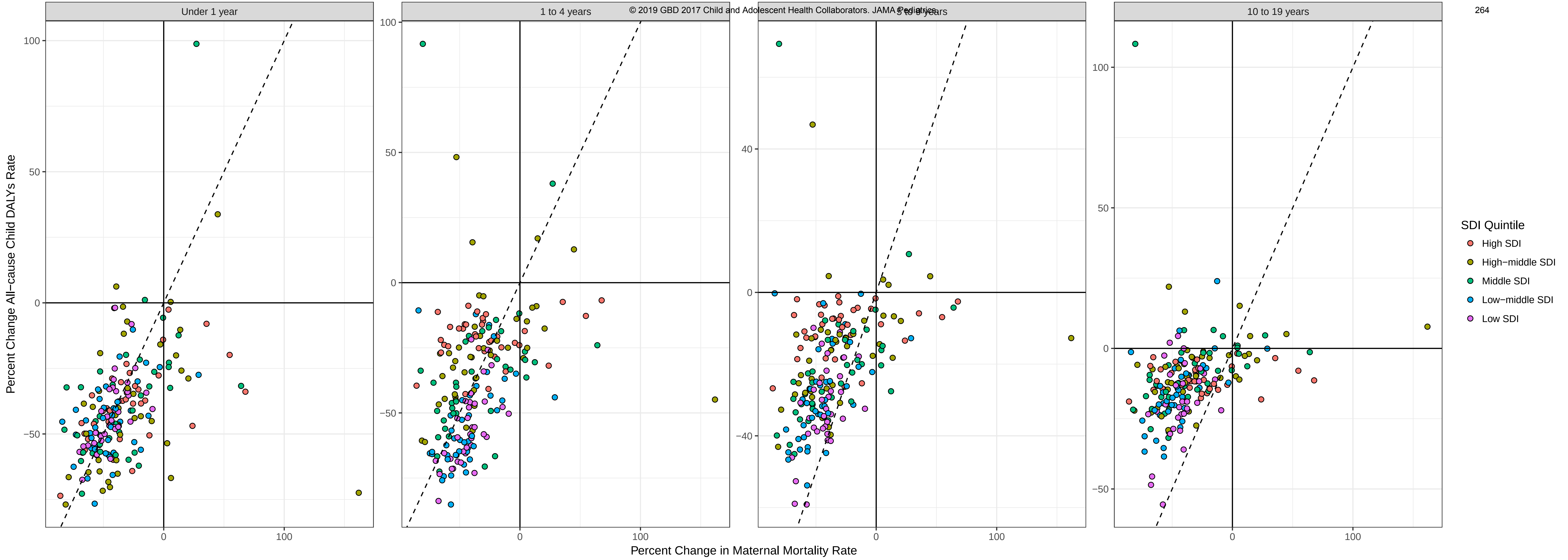

The percent change in maternal mortality rate (MMR) is plotted against the percent change in all−cause child disability−adjusted life year (DALY) rate by country. Panel a) displays the scatter plot of percent change in MMR from 1990 to 2017 against childhood all−cause DALY rate from 1990 to 2017; panel b) displays the scatter plot of percent change in MMR from 1990 to 2017 against childhood all−cause DALY rate from 1990 to 2000; and panel c) displays the scatter plot of percent change in MMR from 1990 to 2017 against childhood all−cause DALY rate from 2000 to 2017. Within each panel, patterns for all children and adolescents less than 20 are in the left sub−panel, under 1 in the second sub−panel, 1 to 4 in the third sub−panel, 5 to 9 in the fourth sub−panel and 10 to 19 in the right sub−panel. In each, every country is colored by its socio−demographic index (SDI) quintile.

eTable 1: Socio-Demographic Index values for all estimated GBD 2017 locations, 1990-2017

Location	1990	1991	1992	1993	1994	1995	1996	1997	1998	1999	2000	2001	2002	2003	2004	2005	2006	2007	2008	2009	2010	2011	2012	2013	2014	2015	2016	2017
Global	0.52	0.53	0.53	0.54	0.54	0.55	0.55	0.56	0.56	0.57	0.57	0.58	0.58	0.59	0.59	0.6	0.6	0.61	0.61	0.62	0.62	0.62	0.63	0.63	0.64	0.64	0.65	0.65
Central Europe, Eastern Europe, and Central Asia	0.66	0.66	0.67	0.67	0.68	0.68	0.69	0.69	0.69	0.69	0.7	0.7	0.71	0.71	0.72	0.72	0.73	0.73	0.74	0.74	0.74	0.75	0.75	0.75	0.76	0.76	0.76	0.77
Central Asia	0.56	0.57	0.57	0.57	0.58	0.58	0.58	0.58	0.58	0.58	0.59	0.59	0.59	0.6	0.6	0.61	0.62	0.62	0.63	0.63	0.64	0.64	0.65	0.65	0.66	0.66	0.67	0.67
Armenia	0.56	0.56	0.56	0.56	0.57	0.57	0.57	0.57	0.58	0.58	0.59	0.59	0.6	0.61	0.62	0.63	0.64	0.65	0.66	0.67	0.67	0.68	0.68	0.69	0.69	0.7	0.7	0.7
Azerbaijan	0.61	0.61	0.62	0.62	0.62	0.61	0.61	0.61	0.6	0.6	0.6	0.6	0.6	0.61	0.61	0.62	0.63	0.64	0.65	0.65	0.66	0.67	0.68	0.68	0.69	0.69	0.7	0.7
Georgia	0.65	0.66	0.66	0.66	0.65	0.65	0.64	0.63	0.63	0.63	0.62	0.62	0.62	0.62	0.63	0.63	0.64	0.64	0.65	0.65	0.66	0.67	0.67	0.68	0.68	0.69	0.7	0.7
Kazakhstan	0.61	0.62	0.62	0.63	0.63	0.64	0.64	0.65	0.65	0.65	0.65	0.66	0.66	0.67	0.67	0.68	0.68	0.69	0.7	0.7	0.71	0.71	0.71	0.71	0.72	0.72	0.73	0.74
Kyrgyzstan	0.57	0.57	0.58	0.58	0.58	0.57	0.57	0.57	0.56	0.56	0.56	0.56	0.56	0.56	0.57	0.57	0.57	0.57	0.57	0.58	0.58	0.58	0.58	0.59	0.59	0.6	0.6	0.61
Mongolia	0.54	0.55	0.55	0.56	0.56	0.56	0.57	0.57	0.58	0.58	0.59	0.59	0.59	0.6	0.6	0.61	0.61	0.62	0.62	0.63	0.63	0.64	0.64	0.65	0.65	0.65	0.66	0.66
Tajikistan	0.47	0.48	0.49	0.49	0.49	0.48	0.47	0.47	0.46	0.46	0.46	0.45	0.46	0.46	0.47	0.47	0.47	0.48	0.48	0.49	0.49	0.5	0.51	0.51	0.51	0.52	0.52	0.52
Turkmenistan	0.59	0.59	0.59	0.6	0.6	0.6	0.61	0.61	0.61	0.61	0.61	0.61	0.62	0.62	0.63	0.64	0.64	0.64	0.64	0.65	0.65	0.66	0.66	0.67	0.68	0.69	0.69	0.7
Uzbekistan	0.48	0.48	0.49	0.49	0.5	0.5	0.51	0.51	0.52	0.53	0.53	0.54	0.54	0.55	0.56	0.56	0.57	0.57	0.58	0.58	0.59	0.59	0.6	0.6	0.61	0.62	0.62	0.63
Central Europe	0.67	0.67	0.68	0.68	0.69	0.7	0.71	0.71	0.72	0.72	0.73	0.74	0.75	0.75	0.76	0.76	0.77	0.77	0.78	0.78	0.79	0.79	0.8	0.8	0.81	0.81	0.81	0.81
Albania	0.55	0.55	0.54	0.54	0.54	0.55	0.55	0.56	0.57	0.58	0.58	0.59	0.6	0.61	0.62	0.63	0.64	0.64	0.65	0.65	0.66	0.66	0.67	0.67	0.67	0.68	0.68	0.69
Bosnia and Herzegovina	0.5	0.5	0.5	0.5	0.5	0.51	0.53	0.55	0.57	0.59	0.61	0.62	0.63	0.64	0.65	0.65	0.66	0.67	0.67	0.68	0.69	0.69	0.69	0.7	0.7	0.71	0.71	0.71
Bulgaria	0.66	0.67	0.68	0.68	0.69	0.7	0.71	0.71	0.7	0.7	0.71	0.72	0.72	0.73	0.73	0.74	0.74	0.75	0.75	0.76	0.77	0.77	0.78	0.78	0.78	0.78	0.79	0.79
Croatia	0.73	0.73	0.73	0.73	0.73	0.73	0.73	0.74	0.74	0.75	0.76	0.76	0.77	0.77	0.78	0.78	0.79	0.79	0.8	0.8	0.81	0.81	0.81	0.82	0.82	0.82	0.82	0.83
Czech Republic	0.71	0.72	0.73	0.74	0.76	0.77	0.78	0.78	0.79	0.79	0.8	0.8	0.81	0.81	0.82	0.82	0.83	0.83	0.83	0.84	0.84	0.84	0.85	0.85	0.85	0.85	0.85	0.85
Hungary	0.68	0.68	0.69	0.7	0.71	0.72	0.72	0.73	0.74	0.75	0.75	0.76	0.76	0.77	0.78	0.78	0.79	0.79	0.8	0.8	0.8	0.81	0.81	0.81	0.81	0.81	0.81	0.82
Macedonia	0.63	0.63	0.63	0.63	0.63	0.64	0.64	0.65	0.65	0.66	0.67	0.67	0.68	0.69	0.69	0.7	0.7	0.71	0.72	0.72	0.72	0.73	0.73	0.74	0.74	0.75	0.75	0.75
Montenegro	0.71	0.71	0.71	0.7	0.7	0.7	0.7	0.7	0.7	0.7	0.71	0.71	0.72	0.72	0.73	0.73	0.74	0.74	0.75	0.76	0.76	0.77	0.77	0.78	0.78	0.78	0.79	0.79
Poland	0.66	0.67	0.68	0.69	0.7	0.71	0.71	0.72	0.73	0.74	0.75	0.76	0.77	0.77	0.78	0.78	0.79	0.79	0.8	0.8	0.81	0.82	0.82	0.83	0.83	0.84	0.84	0.84
Romania	0.65	0.66	0.66	0.67	0.67	0.68	0.68	0.69	0.69	0.69	0.7	0.71	0.71	0.72	0.72	0.73	0.73	0.74	0.75	0.75	0.76	0.76	0.77	0.77	0.77	0.78	0.78	0.78
Serbia	0.63	0.64	0.64	0.64	0.64	0.64	0.64	0.65	0.65	0.66	0.66	0.67	0.67	0.68	0.68	0.69	0.7	0.71	0.71	0.71	0.72	0.72	0.73	0.74	0.74	0.75	0.75	0.75
Slovakia	0.68	0.69	0.7	0.71	0.72	0.73	0.74	0.75	0.76	0.76	0.77	0.78	0.78	0.79	0.79	0.8	0.8	0.81	0.81	0.82	0.82	0.83	0.83	0.83	0.84	0.84	0.84	0.84
Slovenia	0.74	0.75	0.75	0.76	0.76	0.77	0.78	0.78	0.79	0.79	0.8	0.81	0.81	0.82	0.82	0.83	0.83	0.84	0.84	0.84	0.85	0.85	0.85	0.85	0.85	0.86	0.86	0.86
Eastern Europe	0.68	0.69	0.69	0.7	0.7	0.7	0.71	0.71	0.71	0.71	0.71	0.71	0.72	0.72	0.73	0.73	0.74	0.75	0.75	0.76	0.76	0.76	0.77	0.77	0.78	0.78	0.78	0.79
Belarus	0.63	0.63	0.64	0.64	0.65	0.65	0.65	0.65	0.66	0.66	0.67	0.67	0.68	0.68	0.69	0.7	0.7	0.71	0.72	0.73	0.73	0.74	0.75	0.75	0.76	0.76	0.77	0.77
Estonia	0.71	0.72	0.73	0.74	0.74	0.75	0.75	0.76	0.76	0.77	0.77	0.78	0.78	0.79	0.79	0.8	0.81	0.81	0.82	0.83	0.83	0.84	0.84	0.85	0.85	0.85	0.86	0.86
Latvia	0.7	0.7	0.71	0.72	0.73	0.73	0.73	0.73	0.74	0.74	0.74	0.75	0.75	0.76	0.76	0.77	0.78	0.78	0.79	0.8	0.81	0.81	0.81	0.82	0.82	0.82	0.82	0.83
Lithuania	0.71	0.71	0.72	0.73	0.73	0.73	0.73	0.74	0.74	0.75	0.75	0.76	0.77	0.77	0.78	0.79	0.79	0.8	0.8	0.81	0.82	0.82	0.83	0.83	0.84	0.84	0.84	0.84
Moldova	0.58	0.58	0.58	0.58	0.58	0.58	0.58	0.58	0.58	0.58	0.57	0.57	0.58	0.58	0.59	0.6	0.6	0.61	0.62	0.62	0.63	0.64	0.65	0.65	0.66	0.67	0.67	0.68
Russian Federation	0.68	0.69	0.7	0.71	0.71	0.71	0.72	0.72	0.72	0.72	0.72	0.72	0.72	0.73	0.73	0.74	0.75	0.75	0.76	0.76	0.77	0.77	0.77	0.78	0.78	0.79	0.79	0.79
Ukraine	0.66	0.67	0.67	0.67	0.68	0.68	0.68	0.68	0.67	0.67	0.67	0.67	0.68	0.68	0.69	0.69	0.7	0.71	0.71	0.72	0.72	0.73	0.73	0.73	0.74	0.74	0.74	0.74
High-income	0.77	0.77	0.78	0.78	0.79	0.79	0.8	0.8	0.8	0.8	0.81	0.81	0.81	0.82	0.82	0.82	0.82	0.83	0.83	0.83	0.84	0.84	0.84	0.85	0.85	0.85	0.85	0.85
Australasia	0.78	0.79	0.79	0.79	0.8	0.8	0.81	0.81	0.81	0.82	0.82	0.83	0.83	0.83	0.84	0.84	0.84	0.84	0.84	0.85	0.85	0.85	0.86	0.86	0.86	0.86	0.87	0.87
Australia	0.79	0.79	0.79	0.8	0.8	0.81	0.81	0.81	0.82	0.82	0.83	0.83	0.83	0.84	0.84	0.84	0.84	0.85	0.85	0.85	0.85	0.86	0.86	0.86	0.87	0.87	0.87	0.87

eTable 1: Socio-Demographic Index values for all estimated GBD 2017 locations, 1990-2017

Location	1990	1991	1992	1993	1994	1995	1996	1997	1998	1999	2000	2001	2002	2003	2004	2005	2006	2007	2008	2009	2010	2011	2012	2013	2014	2015	2016	2017
New Zealand	0.77	0.77	0.77	0.77	0.78	0.78	0.78	0.79	0.79	0.79	0.8	0.8	0.81	0.81	0.81	0.81	0.81	0.81	0.81	0.82	0.82	0.82	0.83	0.83	0.84	0.84	0.84	0.84
High-income Asia-Pacific	0.78	0.79	0.79	0.8	0.8	0.81	0.81	0.82	0.82	0.82	0.83	0.83	0.83	0.84	0.84	0.84	0.84	0.85	0.85	0.85	0.85	0.86	0.86	0.86	0.86	0.87	0.87	0.87
Brunei	0.73	0.73	0.74	0.75	0.75	0.76	0.76	0.77	0.77	0.78	0.78	0.79	0.8	0.8	0.81	0.81	0.82	0.82	0.83	0.83	0.84	0.84	0.84	0.85	0.85	0.85	0.85	0.86
Japan	0.8	0.81	0.81	0.82	0.82	0.82	0.83	0.83	0.83	0.83	0.83	0.84	0.84	0.84	0.84	0.84	0.85	0.85	0.85	0.85	0.85	0.86	0.86	0.86	0.86	0.86	0.86	0.87
Aichi	0.81	0.82	0.82	0.83	0.83	0.83	0.84	0.84	0.84	0.84	0.84	0.85	0.85	0.85	0.85	0.85	0.86	0.86	0.86	0.86	0.86	0.86	0.87	0.87	0.87	0.87	0.87	0.88
Akita	0.77	0.77	0.78	0.78	0.78	0.79	0.79	0.79	0.79	0.8	0.8	0.8	0.8	0.8	0.81	0.81	0.81	0.81	0.81	0.81	0.82	0.82	0.82	0.82	0.83	0.83	0.83	0.83
Aomori	0.76	0.77	0.77	0.77	0.78	0.78	0.78	0.79	0.79	0.79	0.79	0.79	0.8	0.8	0.8	0.8	0.81	0.81	0.81	0.81	0.81	0.82	0.82	0.82	0.82	0.82	0.82	0.83
Chiba	0.8	0.81	0.81	0.82	0.82	0.82	0.83	0.83	0.83	0.83	0.83	0.83	0.84	0.84	0.84	0.84	0.84	0.84	0.85	0.85	0.85	0.85	0.85	0.85	0.86	0.86	0.86	0.86
Ehime	0.78	0.78	0.79	0.79	0.79	0.8	0.8	0.8	0.8	0.81	0.81	0.81	0.81	0.81	0.82	0.82	0.82	0.82	0.82	0.82	0.83	0.83	0.83	0.83	0.83	0.84	0.84	0.84
Fukui	0.78	0.79	0.79	0.8	0.8	0.81	0.81	0.81	0.82	0.82	0.82	0.82	0.82	0.83	0.83	0.83	0.83	0.83	0.84	0.84	0.84	0.84	0.84	0.85	0.85	0.85	0.85	0.85
Fukuoka	0.8	0.8	0.81	0.81	0.81	0.82	0.82	0.82	0.82	0.83	0.83	0.83	0.83	0.83	0.84	0.84	0.84	0.84	0.84	0.84	0.84	0.85	0.85	0.85	0.85	0.85	0.85	0.86
Fukushima	0.77	0.77	0.78	0.78	0.78	0.79	0.79	0.79	0.79	0.8	0.8	0.8	0.8	0.8	0.81	0.81	0.81	0.81	0.81	0.82	0.82	0.82	0.82	0.83	0.83	0.83	0.83	0.83
Gifu	0.79	0.79	0.8	0.8	0.8	0.81	0.81	0.81	0.82	0.82	0.82	0.82	0.82	0.82	0.83	0.83	0.83	0.83	0.83	0.84	0.84	0.84	0.84	0.84	0.85	0.85	0.85	0.85
Gunma	0.79	0.79	0.8	0.8	0.8	0.81	0.81	0.81	0.82	0.82	0.82	0.82	0.82	0.82	0.83	0.83	0.83	0.83	0.84	0.84	0.84	0.84	0.84	0.85	0.85	0.85	0.85	0.85
Hiroshima	0.8	0.81	0.81	0.81	0.82	0.82	0.83	0.83	0.83	0.83	0.83	0.84	0.84	0.84	0.84	0.84	0.84	0.84	0.85	0.85	0.85	0.85	0.85	0.86	0.86	0.86	0.86	0.86
Hokkaidō	0.78	0.79	0.79	0.8	0.8	0.8	0.81	0.81	0.81	0.81	0.81	0.81	0.82	0.82	0.82	0.82	0.82	0.83	0.83	0.83	0.83	0.83	0.83	0.84	0.84	0.84	0.84	0.84
Hyōgo	0.8	0.8	0.81	0.81	0.82	0.82	0.82	0.83	0.83	0.83	0.83	0.83	0.84	0.84	0.84	0.84	0.84	0.84	0.85	0.85	0.85	0.85	0.85	0.85	0.86	0.86	0.86	0.86
Ibaraki	0.79	0.79	0.8	0.8	0.81	0.81	0.81	0.82	0.82	0.82	0.82	0.82	0.82	0.83	0.83	0.83	0.83	0.83	0.84	0.84	0.84	0.84	0.84	0.85	0.85	0.85	0.85	0.85
Ishikawa	0.79	0.8	0.8	0.8	0.81	0.81	0.82	0.82	0.82	0.82	0.82	0.83	0.83	0.83	0.83	0.84	0.84	0.84	0.84	0.84	0.84	0.85	0.85	0.85	0.85	0.85	0.85	0.86
Iwate	0.76	0.76	0.77	0.77	0.78	0.78	0.78	0.79	0.79	0.79	0.79	0.79	0.79	0.8	0.8	0.8	0.8	0.81	0.81	0.81	0.81	0.82	0.82	0.82	0.82	0.82	0.82	0.83
Kagawa	0.79	0.79	0.8	0.8	0.8	0.81	0.81	0.81	0.82	0.82	0.82	0.82	0.82	0.83	0.83	0.83	0.83	0.83	0.83	0.84	0.84	0.84	0.84	0.84	0.85	0.85	0.85	0.85
Kagoshima	0.77	0.77	0.78	0.78	0.78	0.79	0.79	0.79	0.8	0.8	0.8	0.8	0.8	0.8	0.81	0.81	0.81	0.81	0.81	0.81	0.82	0.82	0.82	0.82	0.83	0.83	0.83	0.83
Kanagawa	0.82	0.82	0.83	0.83	0.84	0.84	0.84	0.84	0.85	0.85	0.85	0.85	0.85	0.85	0.86	0.86	0.86	0.86	0.86	0.86	0.87	0.87	0.87	0.87	0.87	0.87	0.87	0.88
Kōchi	0.76	0.76	0.77	0.77	0.78	0.78	0.78	0.79	0.79	0.79	0.79	0.8	0.8	0.8	0.8	0.81	0.81	0.81	0.81	0.81	0.81	0.82	0.82	0.82	0.82	0.82	0.82	0.83
Kumamoto	0.77	0.77	0.78	0.78	0.79	0.79	0.79	0.79	0.8	0.8	0.8	0.8	0.8	0.81	0.81	0.81	0.81	0.81	0.81	0.82	0.82	0.82	0.82	0.83	0.83	0.83	0.83	0.83
Kyōto	0.81	0.82	0.82	0.83	0.83	0.83	0.84	0.84	0.84	0.84	0.85	0.85	0.85	0.85	0.85	0.85	0.86	0.86	0.86	0.86	0.86	0.86	0.87	0.87	0.87	0.87	0.87	0.87
Mie	0.79	0.79	0.8	0.8	0.81	0.81	0.81	0.82	0.82	0.82	0.82	0.82	0.83	0.83	0.83	0.83	0.83	0.84	0.84	0.84	0.84	0.84	0.85	0.85	0.85	0.85	0.85	0.85
Miyagi	0.79	0.79	0.8	0.8	0.81	0.81	0.81	0.81	0.82	0.82	0.82	0.82	0.82	0.82	0.83	0.83	0.83	0.83	0.84	0.84	0.84	0.84	0.84	0.85	0.85	0.85	0.85	0.85
Miyazaki	0.76	0.77	0.77	0.78	0.78	0.78	0.78	0.79	0.79	0.79	0.79	0.79	0.8	0.8	0.8	0.8	0.8	0.8	0.81	0.81	0.81	0.81	0.81	0.82	0.82	0.82	0.82	0.82
Nagano	0.79	0.8	0.8	0.81	0.81	0.81	0.81	0.82	0.82	0.82	0.82	0.82	0.82	0.83	0.83	0.83	0.83	0.83	0.84	0.84	0.84	0.84	0.84	0.85	0.85	0.85	0.85	0.85
Nagasaki	0.77	0.77	0.77	0.78	0.78	0.78	0.79	0.79	0.79	0.79	0.8	0.8	0.8	0.8	0.8	0.81	0.81	0.81	0.81	0.81	0.81	0.82	0.82	0.82	0.82	0.82	0.82	0.83
Nara	0.79	0.79	0.8	0.8	0.81	0.81	0.81	0.82	0.82	0.82	0.82	0.82	0.83	0.83	0.83	0.83	0.83	0.83	0.84	0.84	0.84	0.84	0.84	0.84	0.84	0.85	0.85	0.85
Niigata	0.78	0.78	0.78	0.79	0.79	0.8	0.8	0.8	0.8	0.81	0.81	0.81	0.81	0.82	0.82	0.82	0.82	0.82	0.83	0.83	0.83	0.83	0.84	0.84	0.84	0.84	0.84	0.84
Ōita	0.79	0.79	0.8	0.8	0.81	0.81	0.81	0.81	0.81	0.81	0.82	0.82	0.82	0.82	0.82	0.83	0.83	0.83	0.83	0.83	0.83	0.84	0.84	0.84	0.84	0.84	0.84	0.85
Okayama	0.79	0.79	0.8	0.8	0.81	0.81	0.81	0.82	0.82	0.82	0.82	0.82	0.83	0.83	0.83	0.83	0.84	0.84	0.84	0.84	0.84	0.85	0.85	0.85	0.85	0.85	0.85	0.86
Okinawa	0.75	0.76	0.76	0.77	0.78	0.78	0.78	0.78	0.78	0.78	0.78	0.78	0.79	0.79	0.79	0.8	0.8	0.8	0.8	0.8	0.8	0.81	0.81	0.81	0.81	0.82	0.82	0.82
Ōsaka	0.82	0.82	0.82	0.83	0.83	0.83	0.84	0.84	0.84	0.84	0.85	0.85	0.85	0.85	0.85	0.85	0.86	0.86	0.86	0.86	0.86	0.86	0.87	0.87	0.87	0.87	0.87	0.87
Saga	0.77	0.78	0.78	0.79	0.79	0.79	0.8	0.8	0.8	0.8	0.8	0.8	0.81	0.81	0.81	0.81	0.81	0.82	0.82	0.82	0.82	0.82	0.83	0.83	0.83	0.83	0.83	0.83

eTable 1: Socio-Demographic Index values for all estimated GBD 2017 locations, 1990-2017

Location	1990	1991	1992	1993	1994	1995	1996	1997	1998	1999	2000	2001	2002	2003	2004	2005	2006	2007	2008	2009	2010	2011	2012	2013	2014	2015	2016	2017
Saitama	0.79	0.8	0.8	0.81	0.81	0.81	0.82	0.82	0.82	0.82	0.83	0.83	0.83	0.83	0.83	0.83	0.84	0.84	0.84	0.84	0.84	0.84	0.85	0.85	0.85	0.85	0.85	0.85
Shiga	0.8	0.81	0.81	0.82	0.82	0.83	0.83	0.83	0.84	0.84	0.84	0.84	0.84	0.85	0.85	0.85	0.85	0.85	0.86	0.86	0.86	0.86	0.86	0.87	0.87	0.87	0.87	0.87
Shimane	0.76	0.77	0.77	0.78	0.78	0.78	0.79	0.79	0.79	0.8	0.8	0.8	0.8	0.8	0.81	0.81	0.81	0.81	0.81	0.82	0.82	0.82	0.82	0.83	0.83	0.83	0.83	0.83
Shizuoka	0.8	0.8	0.81	0.81	0.81	0.82	0.82	0.82	0.83	0.83	0.83	0.83	0.83	0.83	0.84	0.84	0.84	0.84	0.84	0.84	0.85	0.85	0.85	0.85	0.86	0.86	0.86	0.86
Tochigi	0.79	0.79	0.8	0.8	0.81	0.81	0.81	0.82	0.82	0.82	0.82	0.82	0.82	0.83	0.83	0.83	0.83	0.83	0.84	0.84	0.84	0.84	0.85	0.85	0.85	0.85	0.85	0.85
Tokushima	0.78	0.78	0.79	0.79	0.79	0.8	0.8	0.81	0.81	0.81	0.81	0.81	0.82	0.82	0.82	0.82	0.83	0.83	0.83	0.83	0.83	0.84	0.84	0.84	0.84	0.84	0.84	0.85
Tōkyō	0.87	0.88	0.88	0.88	0.89	0.89	0.9	0.9	0.9	0.9	0.91	0.91	0.91	0.91	0.91	0.91	0.91	0.92	0.92	0.92	0.92	0.92	0.92	0.92	0.92	0.92	0.92	0.92
Tottori	0.77	0.78	0.78	0.78	0.79	0.79	0.79	0.8	0.8	0.8	0.8	0.8	0.81	0.81	0.81	0.81	0.81	0.82	0.82	0.82	0.82	0.82	0.83	0.83	0.83	0.83	0.83	0.83
Toyama	0.79	0.8	0.8	0.81	0.81	0.81	0.82	0.82	0.82	0.82	0.83	0.83	0.83	0.83	0.84	0.84	0.84	0.84	0.84	0.85	0.85	0.85	0.85	0.85	0.86	0.86	0.86	0.86
Wakayama	0.78	0.78	0.79	0.79	0.79	0.8	0.8	0.8	0.81	0.81	0.81	0.81	0.81	0.82	0.82	0.82	0.82	0.82	0.82	0.83	0.83	0.83	0.83	0.83	0.84	0.84	0.84	0.84
Yamagata	0.77	0.77	0.78	0.78	0.78	0.79	0.79	0.79	0.79	0.79	0.8	0.8	0.8	0.8	0.81	0.81	0.81	0.81	0.81	0.82	0.82	0.82	0.82	0.83	0.83	0.83	0.83	0.83
Yamaguchi	0.79	0.79	0.8	0.8	0.81	0.81	0.81	0.82	0.82	0.82	0.82	0.82	0.82	0.83	0.83	0.83	0.83	0.83	0.83	0.84	0.84	0.84	0.84	0.84	0.85	0.85	0.85	0.85
Yamanashi	0.79	0.8	0.8	0.8	0.81	0.81	0.82	0.82	0.82	0.82	0.82	0.83	0.83	0.83	0.83	0.83	0.84	0.84	0.84	0.84	0.84	0.84	0.85	0.85	0.85	0.85	0.85	0.85
South Korea	0.71	0.72	0.73	0.74	0.75	0.76	0.77	0.78	0.78	0.79	0.8	0.81	0.81	0.82	0.83	0.83	0.83	0.84	0.84	0.85	0.85	0.85	0.86	0.86	0.86	0.87	0.87	0.87
Singapore	0.74	0.74	0.75	0.76	0.77	0.77	0.78	0.79	0.79	0.79	0.8	0.81	0.82	0.82	0.82	0.83	0.83	0.84	0.84	0.85	0.85	0.86	0.86	0.86	0.87	0.87	0.87	0.87
High-income North America	0.78	0.79	0.79	0.79	0.8	0.8	0.81	0.81	0.81	0.81	0.82	0.82	0.82	0.83	0.83	0.83	0.83	0.83	0.84	0.84	0.85	0.85	0.86	0.86	0.86	0.87	0.87	0.87
Canada	0.8	0.81	0.81	0.81	0.81	0.82	0.82	0.83	0.83	0.84	0.84	0.85	0.85	0.85	0.86	0.86	0.86	0.86	0.86	0.87	0.87	0.87	0.88	0.88	0.88	0.88	0.88	0.88
Greenland	0.67	0.67	0.67	0.67	0.67	0.68	0.68	0.68	0.69	0.69	0.7	0.7	0.7	0.71	0.71	0.72	0.72	0.73	0.73	0.74	0.74	0.75	0.75	0.75	0.76	0.76	0.76	0.76
USA	0.78	0.78	0.79	0.79	0.79	0.8	0.8	0.8	0.81	0.81	0.81	0.82	0.82	0.82	0.83	0.83	0.83	0.83	0.83	0.84	0.85	0.85	0.85	0.86	0.86	0.86	0.87	0.87
Alabama	0.75	0.75	0.75	0.76	0.76	0.77	0.77	0.77	0.77	0.77	0.78	0.78	0.79	0.79	0.79	0.79	0.79	0.8	0.8	0.81	0.82	0.82	0.82	0.83	0.83	0.83	0.84	0.84
Alaska	0.76	0.76	0.76	0.77	0.77	0.78	0.79	0.79	0.79	0.8	0.8	0.8	0.81	0.81	0.81	0.81	0.81	0.81	0.82	0.83	0.83	0.84	0.84	0.85	0.85	0.86	0.86	0.86
Arizona	0.75	0.75	0.76	0.76	0.76	0.76	0.77	0.77	0.77	0.77	0.78	0.78	0.78	0.79	0.79	0.79	0.79	0.8	0.81	0.82	0.82	0.83	0.83	0.84	0.84	0.84	0.85	0.85
Arkansas	0.72	0.73	0.73	0.74	0.74	0.75	0.75	0.75	0.75	0.75	0.76	0.76	0.77	0.77	0.77	0.77	0.77	0.77	0.78	0.79	0.8	0.8	0.81	0.81	0.82	0.82	0.83	0.83
California	0.77	0.77	0.77	0.78	0.78	0.79	0.79	0.8	0.8	0.81	0.81	0.82	0.82	0.83	0.83	0.83	0.83	0.84	0.84	0.85	0.85	0.86	0.86	0.86	0.87	0.87	0.87	0.87
Colorado	0.8	0.8	0.8	0.81	0.81	0.82	0.82	0.82	0.82	0.82	0.82	0.83	0.83	0.84	0.84	0.84	0.84	0.84	0.85	0.86	0.86	0.87	0.87	0.87	0.88	0.88	0.88	0.88
Connecticut	0.84	0.84	0.85	0.85	0.85	0.86	0.86	0.86	0.86	0.87	0.87	0.87	0.88	0.88	0.88	0.88	0.88	0.88	0.89	0.89	0.89	0.9	0.9	0.9	0.9	0.91	0.91	0.91
Delaware	0.8	0.8	0.81	0.81	0.81	0.82	0.82	0.82	0.82	0.82	0.83	0.83	0.83	0.84	0.84	0.84	0.84	0.84	0.85	0.85	0.86	0.86	0.86	0.87	0.87	0.87	0.87	0.87
Washington, DC	0.8	0.8	0.81	0.81	0.82	0.83	0.83	0.84	0.85	0.85	0.86	0.86	0.87	0.87	0.87	0.87	0.87	0.87	0.87	0.87	0.87	0.88	0.88	0.88	0.88	0.89	0.89	0.89
Florida	0.77	0.78	0.78	0.79	0.79	0.79	0.8	0.8	0.8	0.8	0.81	0.81	0.82	0.82	0.82	0.82	0.83	0.83	0.83	0.84	0.85	0.85	0.85	0.86	0.86	0.86	0.86	0.86
Georgia	0.76	0.76	0.76	0.77	0.77	0.78	0.78	0.78	0.78	0.79	0.79	0.79	0.8	0.8	0.8	0.8	0.8	0.8	0.81	0.82	0.83	0.83	0.83	0.84	0.84	0.85	0.85	0.85
Hawaii	0.78	0.78	0.79	0.79	0.8	0.81	0.81	0.81	0.82	0.82	0.82	0.82	0.83	0.83	0.83	0.83	0.83	0.83	0.84	0.85	0.85	0.85	0.86	0.86	0.86	0.87	0.87	0.87
Idaho	0.76	0.76	0.76	0.77	0.77	0.78	0.78	0.78	0.78	0.79	0.79	0.79	0.79	0.8	0.8	0.8	0.8	0.8	0.81	0.81	0.82	0.82	0.83	0.83	0.83	0.84	0.84	0.84
Illinois	0.79	0.79	0.79	0.8	0.8	0.81	0.81	0.81	0.82	0.82	0.82	0.83	0.83	0.84	0.84	0.84	0.84	0.85	0.85	0.86	0.86	0.86	0.87	0.87	0.87	0.88	0.88	0.88
Indiana	0.77	0.77	0.78	0.78	0.78	0.79	0.79	0.79	0.79	0.79	0.8	0.8	0.81	0.81	0.81	0.81	0.81	0.81	0.81	0.82	0.83	0.83	0.83	0.84	0.84	0.84	0.85	0.85
Iowa	0.79	0.8	0.8	0.8	0.81	0.81	0.81	0.81	0.82	0.82	0.82	0.83	0.83	0.83	0.83	0.83	0.83	0.83	0.84	0.84	0.85	0.85	0.86	0.86	0.86	0.87	0.87	0.87
Kansas	0.78	0.79	0.79	0.79	0.79	0.8	0.8	0.8	0.8	0.81	0.81	0.81	0.82	0.82	0.82	0.82	0.82	0.82	0.82	0.83	0.84	0.84	0.85	0.85	0.86	0.86	0.86	0.86
Kentucky	0.74	0.75	0.75	0.76	0.76	0.76	0.77	0.77	0.77	0.77	0.78	0.78	0.79	0.79	0.79	0.79	0.79	0.79	0.79	0.8	0.81	0.81	0.81	0.82	0.82	0.83	0.83	0.83
Louisiana	0.74	0.74	0.74	0.75	0.75	0.76	0.76	0.76	0.77	0.77	0.77	0.78	0.78	0.79	0.79	0.79	0.79	0.79	0.8	0.8	0.81	0.82	0.82	0.82	0.83	0.83	0.84	0.84

eTable 1: Socio-Demographic Index values for all estimated GBD 2017 locations, 1990-2017

Location	1990	1991	1992	1993	1994	1995	1996	1997	1998	1999	2000	2001	2002	2003	2004	2005	2006	2007	2008	2009	2010	2011	2012	2013	2014	2015	2016	2017
Maine	0.79	0.8	0.8	0.81	0.81	0.81	0.82	0.82	0.82	0.82	0.83	0.83	0.83	0.84	0.84	0.84	0.84	0.84	0.85	0.85	0.86	0.86	0.86	0.86	0.87	0.87	0.87	0.87
Maryland	0.81	0.82	0.82	0.83	0.83	0.83	0.84	0.84	0.84	0.84	0.85	0.85	0.85	0.86	0.86	0.86	0.86	0.86	0.87	0.87	0.88	0.88	0.89	0.89	0.89	0.89	0.9	0.9
Massachusetts	0.84	0.85	0.85	0.85	0.86	0.86	0.86	0.87	0.87	0.87	0.88	0.88	0.88	0.89	0.89	0.89	0.89	0.89	0.9	0.9	0.9	0.9	0.91	0.91	0.91	0.91	0.91	0.91
Michigan	0.79	0.79	0.8	0.8	0.8	0.81	0.81	0.82	0.82	0.82	0.82	0.83	0.83	0.84	0.84	0.84	0.84	0.84	0.84	0.85	0.85	0.85	0.85	0.86	0.86	0.86	0.87	0.87
Minnesota	0.82	0.82	0.83	0.83	0.83	0.83	0.84	0.84	0.84	0.84	0.85	0.85	0.85	0.86	0.86	0.86	0.86	0.86	0.87	0.87	0.87	0.88	0.88	0.88	0.89	0.89	0.89	0.89
Mississippi	0.72	0.72	0.73	0.73	0.74	0.74	0.75	0.75	0.75	0.75	0.75	0.76	0.76	0.76	0.77	0.76	0.76	0.76	0.77	0.78	0.79	0.79	0.8	0.8	0.81	0.81	0.82	0.82
Missouri	0.77	0.77	0.78	0.78	0.78	0.79	0.79	0.8	0.8	0.8	0.8	0.81	0.81	0.81	0.81	0.81	0.81	0.81	0.82	0.83	0.83	0.84	0.84	0.84	0.85	0.85	0.85	0.85
Montana	0.78	0.78	0.78	0.79	0.79	0.8	0.8	0.81	0.81	0.81	0.81	0.81	0.82	0.82	0.82	0.82	0.82	0.82	0.83	0.83	0.84	0.84	0.85	0.85	0.86	0.86	0.86	0.86
Nebraska	0.8	0.8	0.8	0.81	0.81	0.81	0.82	0.82	0.82	0.82	0.82	0.82	0.83	0.83	0.83	0.83	0.83	0.83	0.84	0.84	0.85	0.85	0.86	0.86	0.87	0.87	0.87	0.87
Nevada	0.76	0.76	0.76	0.77	0.77	0.77	0.78	0.78	0.78	0.79	0.79	0.8	0.8	0.81	0.81	0.81	0.81	0.81	0.82	0.83	0.83	0.84	0.84	0.84	0.84	0.85	0.85	0.85
New Hampshire	0.83	0.83	0.84	0.84	0.84	0.84	0.85	0.85	0.85	0.86	0.86	0.87	0.87	0.87	0.87	0.88	0.88	0.88	0.88	0.89	0.89	0.89	0.89	0.9	0.9	0.9	0.9	0.9
New Jersey	0.83	0.83	0.83	0.84	0.84	0.85	0.85	0.85	0.85	0.85	0.86	0.86	0.86	0.87	0.87	0.87	0.87	0.87	0.88	0.88	0.89	0.89	0.89	0.89	0.9	0.9	0.9	0.9
New Mexico	0.73	0.74	0.74	0.74	0.75	0.76	0.76	0.76	0.76	0.77	0.77	0.77	0.78	0.78	0.78	0.78	0.78	0.78	0.79	0.8	0.81	0.81	0.82	0.82	0.83	0.83	0.84	0.84
New York	0.82	0.82	0.82	0.83	0.83	0.83	0.84	0.84	0.84	0.84	0.85	0.85	0.86	0.86	0.86	0.86	0.86	0.86	0.87	0.87	0.88	0.88	0.88	0.89	0.89	0.89	0.89	0.89
North Carolina	0.76	0.77	0.77	0.77	0.78	0.78	0.79	0.79	0.79	0.79	0.79	0.8	0.8	0.8	0.81	0.81	0.81	0.81	0.82	0.82	0.83	0.83	0.84	0.84	0.84	0.85	0.85	0.85
North Dakota	0.79	0.79	0.8	0.8	0.8	0.81	0.81	0.82	0.82	0.82	0.83	0.83	0.83	0.83	0.84	0.84	0.83	0.84	0.84	0.84	0.85	0.85	0.86	0.86	0.87	0.88	0.88	0.88
Ohio	0.78	0.78	0.79	0.79	0.79	0.8	0.8	0.8	0.8	0.81	0.81	0.81	0.82	0.82	0.82	0.82	0.82	0.82	0.83	0.83	0.84	0.84	0.84	0.85	0.85	0.85	0.86	0.86
Oklahoma	0.75	0.75	0.76	0.76	0.76	0.76	0.77	0.77	0.77	0.77	0.77	0.78	0.78	0.78	0.79	0.79	0.78	0.79	0.79	0.8	0.81	0.81	0.82	0.82	0.83	0.84	0.84	0.84
Oregon	0.79	0.79	0.79	0.79	0.8	0.8	0.81	0.81	0.81	0.81	0.82	0.82	0.83	0.83	0.83	0.83	0.83	0.84	0.84	0.85	0.85	0.86	0.86	0.86	0.86	0.87	0.87	0.87
Pennsylvania	0.8	0.8	0.81	0.81	0.82	0.82	0.82	0.83	0.83	0.83	0.83	0.84	0.84	0.84	0.85	0.85	0.85	0.85	0.85	0.86	0.86	0.86	0.87	0.87	0.87	0.88	0.88	0.88
Rhode Island	0.82	0.82	0.82	0.83	0.83	0.83	0.83	0.84	0.84	0.84	0.84	0.85	0.85	0.85	0.86	0.86	0.86	0.86	0.87	0.87	0.88	0.88	0.88	0.88	0.89	0.89	0.89	0.89
South Carolina	0.75	0.76	0.76	0.77	0.77	0.78	0.78	0.78	0.78	0.79	0.79	0.79	0.8	0.8	0.8	0.8	0.8	0.8	0.81	0.82	0.82	0.83	0.83	0.83	0.84	0.84	0.85	0.85
South Dakota	0.77	0.77	0.78	0.78	0.79	0.79	0.8	0.8	0.8	0.81	0.81	0.81	0.81	0.81	0.82	0.81	0.81	0.81	0.82	0.83	0.83	0.84	0.84	0.85	0.85	0.86	0.86	0.86
Tennessee	0.75	0.75	0.76	0.76	0.77	0.77	0.77	0.78	0.78	0.78	0.78	0.79	0.79	0.79	0.79	0.79	0.79	0.79	0.8	0.8	0.81	0.82	0.82	0.82	0.83	0.83	0.84	0.84
Texas	0.74	0.75	0.75	0.75	0.75	0.76	0.76	0.76	0.76	0.77	0.77	0.78	0.78	0.78	0.78	0.78	0.78	0.79	0.79	0.8	0.81	0.82	0.82	0.82	0.83	0.83	0.84	0.84
Utah	0.76	0.77	0.77	0.77	0.78	0.78	0.79	0.79	0.79	0.79	0.8	0.8	0.8	0.81	0.81	0.81	0.81	0.81	0.82	0.83	0.83	0.84	0.84	0.85	0.85	0.85	0.86	0.86
Vermont	0.82	0.82	0.82	0.83	0.83	0.83	0.84	0.84	0.84	0.84	0.85	0.85	0.86	0.86	0.86	0.87	0.87	0.87	0.87	0.88	0.88	0.88	0.89	0.89	0.89	0.89	0.9	0.9
Virginia	0.8	0.8	0.81	0.81	0.81	0.82	0.82	0.82	0.82	0.83	0.83	0.83	0.84	0.84	0.85	0.85	0.85	0.85	0.86	0.86	0.87	0.87	0.87	0.88	0.88	0.88	0.89	0.89
Washington	0.8	0.8	0.8	0.81	0.81	0.82	0.82	0.82	0.83	0.83	0.83	0.84	0.84	0.85	0.85	0.85	0.85	0.85	0.86	0.86	0.87	0.87	0.87	0.87	0.88	0.88	0.88	0.88
West Virginia	0.75	0.75	0.76	0.76	0.76	0.77	0.77	0.77	0.78	0.78	0.78	0.78	0.79	0.79	0.79	0.79	0.78	0.78	0.79	0.79	0.8	0.8	0.81	0.81	0.81	0.82	0.82	0.83
Wisconsin	0.8	0.8	0.81	0.81	0.82	0.82	0.82	0.83	0.83	0.83	0.83	0.84	0.84	0.84	0.84	0.84	0.84	0.85	0.85	0.85	0.86	0.86	0.87	0.87	0.87	0.87	0.88	0.88
Wyoming	0.77	0.77	0.78	0.78	0.79	0.79	0.8	0.8	0.8	0.8	0.8	0.81	0.81	0.82	0.82	0.82	0.82	0.82	0.83	0.84	0.85	0.85	0.86	0.86	0.86	0.87	0.87	0.87
Southern Latin America	0.59	0.6	0.61	0.61	0.62	0.63	0.63	0.64	0.64	0.65	0.65	0.66	0.66	0.66	0.67	0.67	0.68	0.68	0.68	0.69	0.69	0.7	0.7	0.7	0.71	0.71	0.72	0.72
Argentina	0.59	0.6	0.6	0.61	0.62	0.62	0.63	0.64	0.64	0.64	0.65	0.65	0.65	0.65	0.66	0.67	0.67	0.67	0.68	0.68	0.68	0.69	0.69	0.69	0.7	0.7	0.71	0.71
Chile	0.6	0.61	0.62	0.62	0.63	0.63	0.64	0.65	0.65	0.66	0.67	0.67	0.68	0.69	0.69	0.7	0.7	0.7	0.7	0.71	0.71	0.72	0.73	0.73	0.74	0.74	0.75	0.75
Uruguay	0.59	0.6	0.6	0.6	0.61	0.61	0.61	0.62	0.63	0.63	0.64	0.64	0.64	0.65	0.65	0.66	0.66	0.66	0.66	0.67	0.67	0.68	0.68	0.69	0.69	0.7	0.7	0.71
Western Europe	0.76	0.77	0.78	0.78	0.79	0.79	0.8	0.8	0.8	0.81	0.81	0.81	0.82	0.82	0.82	0.83	0.83	0.83	0.83	0.84	0.84	0.84	0.85	0.85	0.85	0.85	0.86	0.86
Andorra	0.85	0.85	0.86	0.86	0.86	0.86	0.86	0.86	0.87	0.87	0.87	0.87	0.88	0.88	0.88	0.88	0.89	0.89	0.89	0.89	0.89	0.89	0.9	0.9	0.9	0.9	0.9	0.9

eTable 1: Socio-Demographic Index values for all estimated GBD 2017 locations, 1990-2017

Location	1990	1991	1992	1993	1994	1995	1996	1997	1998	1999	2000	2001	2002	2003	2004	2005	2006	2007	2008	2009	2010	2011	2012	2013	2014	2015	2016	2017
Austria	0.78	0.78	0.78	0.79	0.79	0.8	0.8	0.81	0.81	0.81	0.82	0.82	0.83	0.83	0.83	0.83	0.84	0.84	0.85	0.85	0.85	0.85	0.86	0.86	0.86	0.86	0.87	0.87
Belgium	0.8	0.81	0.81	0.82	0.82	0.83	0.83	0.83	0.84	0.84	0.84	0.84	0.85	0.85	0.85	0.85	0.86	0.86	0.86	0.86	0.87	0.87	0.88	0.88	0.88	0.88	0.89	0.89
Cyprus	0.72	0.73	0.74	0.75	0.76	0.77	0.77	0.78	0.78	0.79	0.8	0.8	0.81	0.82	0.82	0.83	0.84	0.84	0.85	0.85	0.85	0.86	0.86	0.86	0.86	0.86	0.86	0.87
Denmark	0.85	0.85	0.85	0.86	0.86	0.86	0.87	0.87	0.87	0.88	0.88	0.88	0.89	0.89	0.89	0.9	0.9	0.9	0.9	0.9	0.9	0.91	0.91	0.91	0.91	0.92	0.92	0.92
Finland	0.81	0.81	0.81	0.82	0.82	0.83	0.83	0.83	0.84	0.84	0.84	0.85	0.85	0.85	0.86	0.86	0.86	0.87	0.87	0.87	0.88	0.88	0.88	0.88	0.89	0.89	0.89	0.89
France	0.77	0.78	0.78	0.79	0.79	0.8	0.8	0.81	0.81	0.81	0.82	0.82	0.82	0.83	0.83	0.83	0.84	0.84	0.84	0.84	0.85	0.85	0.85	0.85	0.86	0.86	0.86	0.87
Germany	0.79	0.8	0.8	0.81	0.81	0.81	0.81	0.81	0.81	0.81	0.82	0.82	0.83	0.83	0.83	0.84	0.84	0.84	0.85	0.85	0.85	0.86	0.86	0.86	0.86	0.87	0.87	0.87
Greece	0.72	0.72	0.73	0.74	0.74	0.75	0.76	0.76	0.77	0.77	0.78	0.78	0.79	0.79	0.8	0.8	0.8	0.81	0.81	0.81	0.82	0.82	0.82	0.82	0.82	0.82	0.82	0.82
Iceland	0.81	0.82	0.82	0.83	0.83	0.83	0.83	0.84	0.84	0.84	0.85	0.85	0.86	0.86	0.87	0.87	0.87	0.88	0.88	0.88	0.89	0.89	0.89	0.9	0.9	0.9	0.91	0.91
Ireland	0.76	0.76	0.77	0.77	0.78	0.79	0.79	0.8	0.8	0.81	0.81	0.82	0.83	0.83	0.84	0.84	0.85	0.85	0.85	0.86	0.86	0.86	0.87	0.87	0.87	0.87	0.88	0.88
Israel	0.73	0.74	0.74	0.75	0.75	0.76	0.76	0.76	0.77	0.77	0.78	0.78	0.78	0.79	0.79	0.79	0.8	0.8	0.8	0.8	0.8	0.8	0.81	0.81	0.81	0.81	0.81	0.82
Italy	0.77	0.77	0.78	0.78	0.79	0.79	0.8	0.8	0.8	0.81	0.81	0.81	0.82	0.82	0.82	0.82	0.83	0.83	0.83	0.83	0.83	0.83	0.84	0.84	0.84	0.84	0.84	0.84
Luxembourg	0.85	0.85	0.85	0.85	0.86	0.86	0.87	0.87	0.87	0.88	0.88	0.88	0.88	0.88	0.89	0.89	0.89	0.89	0.9	0.9	0.9	0.9	0.91	0.91	0.91	0.91	0.92	0.92
Malta	0.73	0.73	0.74	0.74	0.75	0.75	0.76	0.76	0.77	0.77	0.78	0.78	0.79	0.79	0.8	0.8	0.8	0.81	0.81	0.81	0.81	0.82	0.82	0.82	0.83	0.83	0.83	0.84
Netherlands	0.83	0.83	0.84	0.84	0.85	0.85	0.85	0.86	0.86	0.86	0.87	0.87	0.87	0.88	0.88	0.88	0.89	0.89	0.89	0.89	0.9	0.9	0.9	0.9	0.91	0.91	0.91	0.91
Norway	0.81	0.82	0.82	0.83	0.83	0.84	0.84	0.85	0.85	0.86	0.86	0.87	0.87	0.87	0.88	0.88	0.88	0.88	0.89	0.89	0.89	0.9	0.9	0.9	0.91	0.91	0.91	0.91
Portugal	0.64	0.65	0.66	0.67	0.68	0.68	0.69	0.69	0.7	0.71	0.71	0.72	0.72	0.73	0.73	0.74	0.74	0.74	0.75	0.75	0.76	0.76	0.76	0.77	0.77	0.77	0.78	0.78
Spain	0.72	0.72	0.73	0.74	0.75	0.75	0.76	0.76	0.77	0.77	0.78	0.78	0.79	0.79	0.79	0.8	0.8	0.8	0.81	0.81	0.81	0.82	0.82	0.82	0.82	0.82	0.82	0.83
Sweden	0.78	0.79	0.8	0.8	0.81	0.82	0.82	0.83	0.83	0.84	0.84	0.84	0.84	0.85	0.85	0.85	0.86	0.86	0.86	0.86	0.87	0.87	0.87	0.87	0.88	0.88	0.88	0.88
Stockholm	0.83	0.83	0.84	0.84	0.85	0.85	0.85	0.86	0.86	0.87	0.87	0.87	0.88	0.88	0.88	0.89	0.89	0.89	0.89	0.9	0.9	0.9	0.9	0.91	0.91	0.91	0.91	0.91
Sweden except Stockholm	0.77	0.78	0.79	0.79	0.8	0.81	0.81	0.82	0.82	0.83	0.83	0.83	0.83	0.84	0.84	0.84	0.85	0.85	0.85	0.85	0.85	0.86	0.86	0.86	0.87	0.87	0.87	0.87
Switzerland	0.84	0.84	0.84	0.85	0.85	0.85	0.85	0.85	0.85	0.85	0.85	0.86	0.86	0.86	0.86	0.86	0.87	0.87	0.87	0.87	0.88	0.88	0.88	0.88	0.88	0.89	0.89	0.89
United Kingdom	0.72	0.73	0.74	0.74	0.75	0.75	0.76	0.76	0.77	0.77	0.78	0.79	0.79	0.79	0.8	0.8	0.8	0.81	0.81	0.81	0.82	0.82	0.83	0.83	0.84	0.84	0.84	0.84
England	0.73	0.74	0.75	0.75	0.76	0.76	0.77	0.77	0.78	0.78	0.79	0.79	0.8	0.8	0.8	0.81	0.81	0.81	0.82	0.82	0.82	0.83	0.83	0.84	0.84	0.85	0.85	0.85
East Midlands	0.71	0.72	0.72	0.73	0.74	0.74	0.75	0.75	0.76	0.76	0.77	0.77	0.78	0.78	0.79	0.79	0.79	0.79	0.8	0.8	0.8	0.81	0.81	0.82	0.82	0.83	0.83	0.83
Derby	0.72	0.72	0.73	0.74	0.75	0.75	0.75	0.76	0.76	0.77	0.78	0.79	0.79	0.79	0.8	0.8	0.8	0.81	0.81	0.81	0.82	0.82	0.83	0.83	0.84	0.84	0.84	0.85
Derbyshire	0.7	0.7	0.71	0.72	0.72	0.73	0.73	0.74	0.74	0.75	0.75	0.76	0.76	0.77	0.77	0.77	0.77	0.78	0.78	0.78	0.79	0.79	0.8	0.8	0.81	0.81	0.82	0.82
Leicester	0.71	0.72	0.72	0.73	0.74	0.74	0.75	0.75	0.76	0.76	0.77	0.78	0.78	0.79	0.79	0.79	0.8	0.8	0.81	0.81	0.81	0.82	0.82	0.83	0.83	0.84	0.84	0.84
Leicestershire	0.73	0.73	0.74	0.75	0.75	0.76	0.76	0.77	0.77	0.78	0.78	0.79	0.79	0.8	0.8	0.81	0.81	0.81	0.82	0.82	0.82	0.83	0.83	0.84	0.84	0.84	0.84	0.85
Lincolnshire	0.7	0.71	0.71	0.72	0.72	0.73	0.73	0.74	0.74	0.75	0.75	0.76	0.76	0.77	0.77	0.77	0.77	0.78	0.78	0.78	0.78	0.79	0.79	0.8	0.8	0.81	0.81	0.81
Northamptonshire	0.71	0.72	0.73	0.74	0.74	0.75	0.75	0.75	0.76	0.76	0.77	0.78	0.78	0.78	0.78	0.79	0.79	0.79	0.8	0.8	0.8	0.81	0.81	0.82	0.82	0.83	0.83	0.83
Nottingham	0.74	0.74	0.75	0.76	0.76	0.77	0.77	0.78	0.79	0.79	0.8	0.81	0.81	0.82	0.82	0.83	0.83	0.83	0.84	0.84	0.84	0.85	0.85	0.86	0.86	0.86	0.86	0.86
Nottinghamshire	0.7	0.7	0.71	0.72	0.72	0.73	0.73	0.73	0.74	0.75	0.75	0.76	0.77	0.77	0.77	0.78	0.78	0.78	0.78	0.79	0.79	0.79	0.8	0.8	0.81	0.81	0.81	0.81
Rutland	0.73	0.74	0.74	0.75	0.75	0.76	0.76	0.76	0.77	0.77	0.78	0.78	0.78	0.79	0.79	0.79	0.8	0.8	0.8	0.81	0.81	0.81	0.82	0.82	0.83	0.83	0.83	0.83
East of England	0.72	0.73	0.74	0.74	0.75	0.76	0.76	0.76	0.77	0.77	0.78	0.79	0.79	0.79	0.8	0.8	0.8	0.81	0.81	0.81	0.81	0.82	0.82	0.83	0.83	0.84	0.84	0.84
Bedford	0.73	0.74	0.74	0.75	0.76	0.76	0.76	0.77	0.77	0.78	0.79	0.79	0.79	0.8	0.8	0.8	0.81	0.81	0.81	0.81	0.82	0.82	0.83	0.83	0.83	0.84	0.84	0.84
Cambridgeshire	0.75	0.76	0.76	0.77	0.78	0.78	0.79	0.79	0.8	0.8	0.81	0.82	0.82	0.82	0.83	0.83	0.83	0.84	0.84	0.84	0.85	0.85	0.86	0.86	0.86	0.87	0.87	0.87

eTable 1: Socio-Demographic Index values for all estimated GBD 2017 locations, 1990-2017

Location	1990	1991	1992	1993	1994	1995	1996	1997	1998	1999	2000	2001	2002	2003	2004	2005	2006	2007	2008	2009	2010	2011	2012	2013	2014	2015	2016	2017
Central Bedfordshire	0.72	0.73	0.74	0.74	0.75	0.75	0.76	0.76	0.77	0.77	0.78	0.78	0.79	0.79	0.79	0.79	0.8	0.8	0.8	0.8	0.81	0.81	0.82	0.82	0.83	0.83	0.83	0.83
Essex	0.71	0.72	0.73	0.73	0.74	0.75	0.75	0.75	0.76	0.76	0.77	0.78	0.78	0.78	0.79	0.79	0.79	0.8	0.8	0.8	0.81	0.81	0.82	0.82	0.83	0.83	0.83	0.83
Hertfordshire	0.75	0.76	0.76	0.77	0.78	0.78	0.78	0.79	0.8	0.8	0.81	0.82	0.82	0.83	0.83	0.83	0.84	0.84	0.84	0.84	0.85	0.85	0.86	0.86	0.86	0.87	0.87	0.87
Luton	0.71	0.72	0.72	0.73	0.74	0.74	0.75	0.75	0.75	0.76	0.77	0.77	0.78	0.78	0.78	0.79	0.79	0.8	0.8	0.81	0.81	0.81	0.82	0.83	0.83	0.83	0.83	0.83
Norfolk	0.71	0.72	0.72	0.73	0.74	0.74	0.75	0.75	0.75	0.76	0.77	0.77	0.78	0.78	0.78	0.78	0.79	0.79	0.79	0.8	0.8	0.8	0.81	0.81	0.82	0.82	0.82	0.83
Peterborough	0.71	0.72	0.72	0.73	0.74	0.74	0.74	0.75	0.75	0.76	0.76	0.77	0.77	0.77	0.77	0.77	0.78	0.78	0.78	0.78	0.78	0.79	0.8	0.8	0.81	0.81	0.82	0.82
Southend-on-Sea	0.7	0.7	0.71	0.71	0.72	0.72	0.73	0.73	0.74	0.74	0.75	0.76	0.76	0.77	0.77	0.77	0.77	0.78	0.78	0.78	0.79	0.79	0.8	0.8	0.81	0.81	0.81	0.81
Suffolk	0.71	0.72	0.73	0.73	0.74	0.74	0.75	0.75	0.75	0.76	0.76	0.77	0.77	0.77	0.78	0.78	0.78	0.78	0.79	0.79	0.79	0.8	0.8	0.81	0.81	0.82	0.82	0.82
Thurrock	0.71	0.72	0.72	0.73	0.74	0.74	0.75	0.75	0.75	0.76	0.76	0.77	0.77	0.77	0.77	0.77	0.78	0.78	0.78	0.78	0.78	0.79	0.79	0.8	0.8	0.8	0.81	0.81
Greater London	0.78	0.79	0.79	0.8	0.81	0.81	0.81	0.82	0.82	0.83	0.84	0.84	0.85	0.85	0.85	0.86	0.86	0.86	0.86	0.87	0.87	0.88	0.88	0.89	0.89	0.89	0.89	0.89
Barking and Dagenham	0.7	0.71	0.72	0.72	0.73	0.73	0.74	0.74	0.75	0.75	0.76	0.76	0.76	0.76	0.76	0.76	0.76	0.76	0.76	0.77	0.77	0.77	0.78	0.79	0.79	0.8	0.8	0.8
Barnet	0.76	0.77	0.77	0.78	0.78	0.79	0.79	0.8	0.8	0.81	0.81	0.82	0.82	0.83	0.83	0.83	0.83	0.84	0.84	0.84	0.84	0.85	0.85	0.86	0.86	0.86	0.86	0.87
Bexley	0.72	0.73	0.73	0.74	0.75	0.75	0.75	0.76	0.76	0.77	0.77	0.78	0.78	0.78	0.79	0.79	0.79	0.79	0.8	0.8	0.8	0.8	0.81	0.82	0.82	0.82	0.82	0.83
Brent	0.75	0.76	0.76	0.77	0.78	0.78	0.79	0.79	0.8	0.8	0.81	0.81	0.81	0.82	0.82	0.82	0.82	0.82	0.82	0.82	0.83	0.83	0.83	0.84	0.84	0.85	0.85	0.85
Bromley	0.75	0.76	0.76	0.77	0.77	0.78	0.78	0.79	0.79	0.8	0.8	0.81	0.81	0.82	0.82	0.82	0.83	0.83	0.83	0.83	0.83	0.83	0.84	0.84	0.84	0.85	0.85	0.85
Camden	0.84	0.84	0.85	0.85	0.86	0.86	0.87	0.87	0.87	0.88	0.89	0.89	0.89	0.9	0.9	0.9	0.9	0.91	0.91	0.91	0.91	0.92	0.92	0.92	0.93	0.93	0.93	0.93
Croydon	0.74	0.75	0.75	0.76	0.77	0.77	0.77	0.78	0.78	0.79	0.79	0.8	0.8	0.81	0.81	0.81	0.81	0.81	0.82	0.82	0.82	0.82	0.82	0.83	0.83	0.83	0.83	0.83
Ealing	0.76	0.77	0.78	0.78	0.79	0.79	0.8	0.8	0.81	0.81	0.82	0.83	0.83	0.83	0.83	0.83	0.84	0.84	0.84	0.84	0.84	0.84	0.85	0.85	0.86	0.86	0.86	0.87
Enfield	0.74	0.74	0.75	0.76	0.76	0.77	0.77	0.77	0.78	0.78	0.79	0.79	0.8	0.8	0.8	0.8	0.8	0.81	0.81	0.81	0.81	0.82	0.82	0.83	0.83	0.84	0.84	0.84
Greenwich	0.73	0.73	0.74	0.75	0.75	0.76	0.76	0.77	0.77	0.78	0.78	0.79	0.79	0.79	0.8	0.8	0.8	0.8	0.8	0.81	0.81	0.81	0.82	0.82	0.83	0.83	0.83	0.83
Hackney	0.77	0.77	0.78	0.79	0.8	0.8	0.8	0.8	0.81	0.81	0.82	0.82	0.83	0.83	0.83	0.83	0.84	0.84	0.85	0.85	0.86	0.86	0.87	0.88	0.88	0.88	0.89	0.89
Hammersmith and Fulham	0.83	0.83	0.84	0.85	0.85	0.86	0.86	0.86	0.87	0.88	0.88	0.88	0.89	0.89	0.89	0.9	0.9	0.9	0.9	0.91	0.91	0.91	0.92	0.92	0.92	0.92	0.93	0.93
Haringey	0.76	0.76	0.77	0.77	0.78	0.78	0.79	0.79	0.79	0.8	0.8	0.81	0.81	0.82	0.82	0.82	0.82	0.83	0.83	0.83	0.83	0.84	0.84	0.85	0.85	0.85	0.85	0.85
Harrow	0.75	0.75	0.76	0.77	0.77	0.78	0.78	0.78	0.79	0.8	0.8	0.81	0.81	0.82	0.82	0.82	0.82	0.83	0.83	0.83	0.83	0.83	0.84	0.84	0.84	0.84	0.85	0.85
Havering	0.72	0.73	0.73	0.74	0.74	0.75	0.75	0.75	0.76	0.77	0.77	0.78	0.78	0.78	0.79	0.79	0.79	0.79	0.8	0.8	0.8	0.81	0.81	0.81	0.82	0.82	0.82	0.82
Hillingdon	0.78	0.79	0.79	0.8	0.81	0.81	0.82	0.82	0.82	0.83	0.83	0.84	0.84	0.84	0.85	0.85	0.85	0.85	0.85	0.85	0.86	0.86	0.87	0.87	0.88	0.88	0.88	0.88
Hounslow	0.77	0.78	0.78	0.79	0.79	0.8	0.8	0.81	0.81	0.82	0.82	0.83	0.83	0.84	0.84	0.84	0.84	0.85	0.85	0.85	0.85	0.86	0.86	0.87	0.87	0.88	0.88	0.88
Islington	0.81	0.82	0.83	0.83	0.84	0.84	0.85	0.85	0.86	0.86	0.87	0.87	0.88	0.88	0.88	0.89	0.89	0.89	0.9	0.9	0.9	0.91	0.91	0.91	0.92	0.92	0.92	0.92
Kensington and Chelsea	0.84	0.85	0.85	0.86	0.86	0.87	0.87	0.87	0.88	0.88	0.89	0.89	0.9	0.9	0.9	0.91	0.91	0.91	0.91	0.92	0.92	0.92	0.92	0.93	0.93	0.93	0.93	0.93
Kingston upon Thames	0.79	0.79	0.8	0.81	0.81	0.82	0.82	0.83	0.83	0.84	0.84	0.85	0.85	0.86	0.86	0.86	0.87	0.87	0.87	0.87	0.88	0.88	0.88	0.89	0.89	0.89	0.89	0.89
Lambeth	0.78	0.78	0.79	0.8	0.8	0.81	0.81	0.82	0.82	0.83	0.84	0.84	0.85	0.85	0.85	0.86	0.86	0.86	0.87	0.87	0.88	0.88	0.89	0.89	0.9	0.9	0.9	0.9
Lewisham	0.73	0.74	0.75	0.75	0.76	0.76	0.77	0.77	0.78	0.78	0.79	0.79	0.8	0.8	0.8	0.8	0.81	0.81	0.81	0.81	0.82	0.82	0.83	0.83	0.84	0.84	0.84	0.84
Merton	0.76	0.77	0.77	0.78	0.79	0.79	0.8	0.8	0.81	0.81	0.82	0.82	0.83	0.83	0.83	0.84	0.84	0.84	0.84	0.84	0.85	0.85	0.86	0.86	0.87	0.87	0.87	0.87
Newham	0.72	0.72	0.73	0.73	0.74	0.74	0.75	0.75	0.75	0.76	0.77	0.77	0.78	0.78	0.78	0.78	0.78	0.79	0.79	0.79	0.8	0.81	0.82	0.82	0.83	0.83	0.84	0.84
Redbridge	0.73	0.73	0.74	0.75	0.75	0.76	0.76	0.76	0.77	0.77	0.78	0.79	0.79	0.79	0.79	0.8	0.8	0.8	0.8	0.81	0.81	0.81	0.82	0.82	0.83	0.83	0.83	0.83

eTable 1: Socio-Demographic Index values for all estimated GBD 2017 locations, 1990-2017

Location	1990	1991	1992	1993	1994	1995	1996	1997	1998	1999	2000	2001	2002	2003	2004	2005	2006	2007	2008	2009	2010	2011	2012	2013	2014	2015	2016	2017
Richmond upon Thames	0.79	0.8	0.81	0.81	0.82	0.82	0.83	0.83	0.84	0.84	0.85	0.86	0.86	0.86	0.87	0.87	0.87	0.87	0.88	0.88	0.88	0.88	0.89	0.89	0.9	0.9	0.9	0.9
Southwark	0.79	0.8	0.81	0.82	0.82	0.83	0.83	0.84	0.84	0.85	0.85	0.86	0.86	0.86	0.87	0.87	0.87	0.88	0.88	0.88	0.89	0.89	0.9	0.9	0.91	0.91	0.91	0.91
Sutton	0.74	0.74	0.75	0.75	0.76	0.77	0.77	0.77	0.78	0.79	0.79	0.8	0.8	0.81	0.81	0.81	0.81	0.81	0.82	0.82	0.82	0.82	0.83	0.83	0.84	0.84	0.84	0.84
Tower Hamlets	0.77	0.77	0.78	0.79	0.8	0.8	0.81	0.81	0.82	0.83	0.83	0.84	0.84	0.85	0.85	0.86	0.86	0.87	0.87	0.88	0.88	0.89	0.89	0.9	0.9	0.9	0.9	0.91
Waltham Forest	0.71	0.72	0.72	0.73	0.74	0.74	0.75	0.75	0.75	0.76	0.77	0.77	0.77	0.78	0.78	0.78	0.78	0.78	0.78	0.78	0.79	0.79	0.8	0.81	0.81	0.81	0.82	0.82
Wandsworth	0.79	0.8	0.81	0.81	0.82	0.83	0.83	0.84	0.84	0.85	0.86	0.86	0.87	0.87	0.87	0.88	0.88	0.88	0.89	0.89	0.89	0.89	0.9	0.9	0.91	0.91	0.91	0.91
Westminster	0.83	0.84	0.84	0.85	0.85	0.86	0.86	0.87	0.87	0.88	0.88	0.89	0.89	0.89	0.9	0.9	0.9	0.9	0.91	0.91	0.91	0.91	0.92	0.92	0.92	0.92	0.93	0.93
North East England	0.7	0.7	0.71	0.72	0.73	0.73	0.73	0.74	0.74	0.75	0.76	0.76	0.77	0.77	0.77	0.78	0.78	0.78	0.79	0.79	0.79	0.8	0.8	0.81	0.81	0.82	0.82	0.82
County Durham	0.69	0.7	0.7	0.71	0.72	0.72	0.73	0.73	0.74	0.74	0.75	0.75	0.76	0.76	0.77	0.77	0.77	0.77	0.78	0.78	0.78	0.79	0.79	0.8	0.8	0.81	0.81	0.81
Darlington	0.7	0.71	0.72	0.72	0.73	0.73	0.74	0.74	0.75	0.75	0.76	0.76	0.77	0.77	0.78	0.78	0.78	0.79	0.79	0.79	0.8	0.8	0.81	0.81	0.82	0.82	0.82	0.83
Gateshead	0.7	0.71	0.71	0.72	0.73	0.73	0.74	0.74	0.75	0.75	0.76	0.76	0.77	0.77	0.78	0.78	0.78	0.79	0.79	0.79	0.8	0.8	0.81	0.81	0.82	0.82	0.82	0.83
Hartlepool	0.68	0.68	0.69	0.7	0.7	0.71	0.71	0.71	0.72	0.73	0.73	0.74	0.74	0.74	0.74	0.75	0.75	0.75	0.76	0.76	0.76	0.77	0.77	0.78	0.79	0.79	0.79	0.79
Middlesbrough	0.69	0.7	0.7	0.71	0.72	0.72	0.72	0.73	0.73	0.74	0.75	0.75	0.76	0.76	0.76	0.77	0.77	0.77	0.78	0.78	0.78	0.79	0.79	0.8	0.8	0.8	0.81	0.81
Newcastle upon Tyne	0.74	0.74	0.75	0.76	0.77	0.77	0.78	0.78	0.79	0.79	0.8	0.81	0.81	0.82	0.83	0.83	0.84	0.84	0.84	0.85	0.85	0.85	0.86	0.86	0.87	0.87	0.87	0.87
North Tyneside	0.7	0.7	0.71	0.72	0.72	0.73	0.73	0.74	0.74	0.75	0.76	0.76	0.77	0.77	0.78	0.78	0.78	0.79	0.79	0.79	0.8	0.8	0.81	0.81	0.82	0.82	0.82	0.83
Northumberland	0.7	0.7	0.71	0.72	0.72	0.73	0.73	0.74	0.74	0.75	0.75	0.76	0.76	0.76	0.77	0.77	0.77	0.78	0.78	0.78	0.78	0.79	0.79	0.8	0.8	0.81	0.81	0.81
Redcar and Cleveland	0.67	0.68	0.68	0.69	0.7	0.7	0.7	0.71	0.71	0.72	0.73	0.73	0.74	0.74	0.74	0.74	0.75	0.75	0.75	0.75	0.76	0.76	0.77	0.78	0.78	0.78	0.79	0.79
South Tyneside	0.66	0.67	0.68	0.69	0.69	0.7	0.7	0.71	0.71	0.72	0.73	0.73	0.74	0.74	0.74	0.75	0.75	0.75	0.76	0.76	0.76	0.77	0.77	0.78	0.79	0.79	0.79	0.79
Stockton-on-Tees	0.71	0.71	0.72	0.73	0.74	0.74	0.74	0.75	0.75	0.76	0.77	0.77	0.78	0.78	0.78	0.78	0.78	0.79	0.79	0.79	0.79	0.8	0.8	0.81	0.82	0.82	0.82	0.82
Sunderland	0.69	0.7	0.7	0.71	0.72	0.73	0.73	0.73	0.74	0.75	0.75	0.76	0.76	0.77	0.77	0.77	0.78	0.78	0.78	0.78	0.79	0.79	0.8	0.8	0.81	0.81	0.81	0.82
North West England	0.72	0.72	0.73	0.74	0.74	0.75	0.75	0.76	0.76	0.77	0.77	0.78	0.78	0.79	0.79	0.79	0.8	0.8	0.8	0.8	0.81	0.81	0.82	0.82	0.83	0.83	0.83	0.83
Blackburn with Darwen	0.69	0.7	0.7	0.71	0.71	0.72	0.72	0.72	0.73	0.73	0.74	0.75	0.75	0.75	0.75	0.76	0.76	0.76	0.76	0.77	0.77	0.77	0.78	0.79	0.79	0.8	0.8	0.8
Blackpool	0.68	0.69	0.69	0.7	0.7	0.71	0.71	0.71	0.72	0.72	0.73	0.73	0.74	0.74	0.74	0.74	0.75	0.75	0.75	0.75	0.75	0.76	0.76	0.77	0.77	0.78	0.78	0.78
Bolton	0.7	0.71	0.71	0.72	0.73	0.73	0.73	0.74	0.74	0.75	0.75	0.76	0.76	0.76	0.76	0.77	0.77	0.77	0.77	0.77	0.78	0.78	0.79	0.79	0.8	0.8	0.8	0.81
Bury	0.7	0.71	0.72	0.72	0.73	0.73	0.74	0.74	0.75	0.75	0.76	0.76	0.77	0.77	0.77	0.77	0.78	0.78	0.78	0.78	0.79	0.79	0.8	0.8	0.81	0.81	0.81	0.82
Cheshire East	0.75	0.75	0.76	0.77	0.77	0.78	0.78	0.79	0.79	0.8	0.8	0.81	0.81	0.82	0.82	0.82	0.83	0.83	0.83	0.83	0.84	0.84	0.85	0.85	0.86	0.86	0.86	0.86
Cheshire West and Chester	0.74	0.75	0.75	0.76	0.77	0.77	0.78	0.78	0.79	0.79	0.8	0.8	0.81	0.81	0.82	0.82	0.82	0.83	0.83	0.83	0.83	0.84	0.84	0.85	0.85	0.85	0.85	0.86
Cumbria	0.72	0.72	0.73	0.74	0.74	0.75	0.75	0.76	0.76	0.77	0.77	0.77	0.78	0.78	0.78	0.78	0.79	0.79	0.79	0.79	0.8	0.8	0.81	0.82	0.82	0.82	0.83	0.83
Halton	0.7	0.71	0.72	0.72	0.73	0.73	0.74	0.74	0.75	0.75	0.76	0.76	0.77	0.77	0.77	0.78	0.78	0.78	0.78	0.79	0.79	0.8	0.8	0.81	0.82	0.82	0.82	0.82
Knowsley	0.69	0.7	0.7	0.71	0.71	0.72	0.72	0.73	0.73	0.74	0.74	0.75	0.75	0.76	0.76	0.76	0.77	0.77	0.77	0.78	0.78	0.79	0.79	0.8	0.81	0.81	0.81	0.82
Lancashire	0.72	0.73	0.73	0.74	0.75	0.75	0.76	0.76	0.77	0.77	0.78	0.78	0.78	0.79	0.79	0.79	0.79	0.8	0.8	0.8	0.81	0.81	0.82	0.82	0.82	0.83	0.83	0.83
Liverpool	0.72	0.73	0.74	0.74	0.75	0.75	0.76	0.76	0.77	0.78	0.78	0.79	0.8	0.8	0.81	0.81	0.82	0.82	0.82	0.83	0.83	0.84	0.84	0.84	0.85	0.85	0.85	0.85
Manchester	0.75	0.75	0.76	0.77	0.78	0.78	0.79	0.79	0.8	0.8	0.81	0.82	0.83	0.83	0.84	0.84	0.84	0.85	0.85	0.86	0.86	0.86	0.87	0.87	0.88	0.88	0.88	0.89
Oldham	0.69	0.69	0.7	0.7	0.71	0.71	0.71	0.72	0.72	0.72	0.73	0.73	0.74	0.74	0.74	0.74	0.75	0.75	0.75	0.75	0.76	0.76	0.77	0.78	0.78	0.79	0.79	0.79
Rochdale	0.69	0.69	0.7	0.71	0.71	0.72	0.72	0.72	0.73	0.73	0.74	0.74	0.75	0.75	0.75	0.75	0.76	0.76	0.76	0.76	0.77	0.77	0.78	0.78	0.79	0.79	0.79	0.8
Salford	0.71	0.71	0.72	0.73	0.74	0.74	0.74	0.75	0.75	0.76	0.77	0.78	0.78	0.79	0.79	0.79	0.8	0.8	0.8	0.81	0.81	0.81	0.82	0.83	0.83	0.83	0.84	0.84

eTable 1: Socio-Demographic Index values for all estimated GBD 2017 locations, 1990-2017

Location	1990	1991	1992	1993	1994	1995	1996	1997	1998	1999	2000	2001	2002	2003	2004	2005	2006	2007	2008	2009	2010	2011	2012	2013	2014	2015	2016	2017
Sefton	0.71	0.71	0.72	0.73	0.73	0.74	0.74	0.75	0.75	0.76	0.76	0.77	0.77	0.78	0.78	0.78	0.79	0.79	0.79	0.79	0.79	0.8	0.8	0.8	0.81	0.81	0.81	0.81
St Helens	0.68	0.69	0.7	0.7	0.71	0.71	0.72	0.72	0.73	0.73	0.74	0.74	0.75	0.75	0.76	0.76	0.76	0.77	0.77	0.77	0.77	0.78	0.79	0.79	0.8	0.8	0.8	0.8
Stockport	0.73	0.73	0.74	0.75	0.76	0.76	0.76	0.77	0.77	0.78	0.79	0.79	0.8	0.8	0.8	0.81	0.81	0.81	0.81	0.82	0.82	0.82	0.83	0.83	0.84	0.84	0.84	0.84
Tameside	0.69	0.7	0.7	0.71	0.72	0.72	0.72	0.73	0.73	0.74	0.75	0.75	0.76	0.76	0.76	0.76	0.76	0.76	0.77	0.77	0.77	0.77	0.78	0.79	0.79	0.79	0.8	0.8
Trafford	0.75	0.76	0.76	0.77	0.78	0.78	0.79	0.79	0.8	0.8	0.81	0.82	0.82	0.82	0.83	0.83	0.84	0.84	0.84	0.84	0.85	0.85	0.86	0.86	0.87	0.87	0.87	0.87
Warrington	0.74	0.75	0.75	0.76	0.77	0.77	0.77	0.78	0.78	0.79	0.8	0.8	0.81	0.81	0.81	0.82	0.82	0.83	0.83	0.83	0.83	0.84	0.84	0.85	0.85	0.86	0.86	0.86
Wigan	0.69	0.7	0.7	0.71	0.72	0.72	0.72	0.73	0.73	0.74	0.74	0.75	0.75	0.75	0.76	0.76	0.76	0.76	0.76	0.77	0.77	0.77	0.78	0.79	0.79	0.79	0.8	0.8
Wirral	0.7	0.7	0.71	0.71	0.72	0.73	0.73	0.73	0.74	0.74	0.75	0.75	0.76	0.76	0.76	0.77	0.77	0.77	0.77	0.78	0.78	0.78	0.79	0.79	0.8	0.8	0.8	0.8
South East England	0.75	0.76	0.76	0.77	0.77	0.78	0.78	0.78	0.79	0.8	0.8	0.81	0.81	0.81	0.82	0.82	0.82	0.82	0.83	0.83	0.83	0.84	0.84	0.85	0.85	0.85	0.86	0.86
Bracknell Forest	0.76	0.76	0.77	0.78	0.78	0.79	0.79	0.79	0.8	0.8	0.81	0.82	0.82	0.83	0.83	0.83	0.84	0.84	0.84	0.84	0.85	0.85	0.85	0.86	0.86	0.87	0.87	0.87
Brighton and Hove	0.77	0.77	0.78	0.79	0.79	0.8	0.8	0.81	0.81	0.82	0.82	0.83	0.83	0.84	0.84	0.85	0.85	0.86	0.86	0.86	0.87	0.87	0.87	0.88	0.88	0.88	0.88	0.89
Buckinghamshire	0.76	0.77	0.78	0.78	0.79	0.79	0.8	0.8	0.8	0.81	0.82	0.82	0.82	0.83	0.83	0.83	0.83	0.84	0.84	0.84	0.84	0.85	0.85	0.86	0.86	0.86	0.86	0.87
East Sussex	0.71	0.72	0.72	0.73	0.74	0.74	0.74	0.75	0.75	0.75	0.76	0.76	0.77	0.77	0.77	0.77	0.78	0.78	0.78	0.78	0.79	0.79	0.8	0.8	0.81	0.81	0.81	0.81
Hampshire	0.74	0.75	0.76	0.76	0.77	0.77	0.78	0.78	0.78	0.79	0.79	0.8	0.8	0.8	0.81	0.81	0.81	0.82	0.82	0.82	0.82	0.83	0.83	0.84	0.84	0.85	0.85	0.85
Isle of Wight	0.7	0.71	0.72	0.72	0.73	0.73	0.74	0.74	0.74	0.75	0.76	0.76	0.77	0.77	0.77	0.77	0.77	0.78	0.78	0.78	0.78	0.79	0.79	0.8	0.81	0.81	0.81	0.81
Kent	0.72	0.73	0.73	0.74	0.75	0.75	0.75	0.76	0.76	0.77	0.77	0.77	0.78	0.78	0.78	0.79	0.79	0.79	0.79	0.8	0.8	0.81	0.81	0.82	0.82	0.82	0.83	0.83
Medway	0.7	0.71	0.72	0.72	0.73	0.73	0.73	0.74	0.74	0.75	0.75	0.76	0.76	0.76	0.77	0.77	0.77	0.77	0.78	0.78	0.78	0.79	0.79	0.8	0.8	0.81	0.81	0.81
Milton Keynes	0.75	0.76	0.77	0.77	0.78	0.78	0.79	0.79	0.79	0.8	0.8	0.81	0.81	0.81	0.82	0.82	0.82	0.82	0.82	0.83	0.83	0.83	0.84	0.85	0.85	0.86	0.86	0.86
Oxfordshire	0.77	0.78	0.78	0.79	0.79	0.8	0.8	0.81	0.81	0.82	0.82	0.83	0.83	0.84	0.84	0.84	0.84	0.85	0.85	0.85	0.86	0.86	0.86	0.87	0.87	0.88	0.88	0.88
Portsmouth	0.75	0.76	0.76	0.77	0.78	0.78	0.79	0.79	0.8	0.8	0.81	0.81	0.82	0.82	0.82	0.82	0.83	0.83	0.83	0.84	0.84	0.84	0.85	0.85	0.85	0.86	0.86	0.86
Reading	0.79	0.79	0.8	0.8	0.81	0.81	0.82	0.82	0.83	0.83	0.84	0.85	0.85	0.86	0.86	0.86	0.87	0.87	0.87	0.87	0.87	0.88	0.88	0.89	0.89	0.89	0.89	0.9
Slough	0.76	0.77	0.78	0.78	0.79	0.79	0.8	0.8	0.8	0.81	0.81	0.81	0.82	0.82	0.82	0.82	0.82	0.82	0.82	0.83	0.83	0.83	0.84	0.85	0.85	0.86	0.86	0.86
Southampton	0.75	0.76	0.77	0.77	0.78	0.78	0.79	0.79	0.8	0.81	0.81	0.82	0.82	0.82	0.83	0.83	0.83	0.83	0.84	0.84	0.84	0.84	0.85	0.85	0.85	0.86	0.86	0.86
Surrey	0.77	0.78	0.79	0.79	0.8	0.8	0.81	0.81	0.81	0.82	0.83	0.83	0.84	0.84	0.85	0.85	0.85	0.85	0.86	0.86	0.86	0.86	0.87	0.87	0.88	0.88	0.88	0.88
West Berkshire	0.77	0.78	0.79	0.79	0.8	0.8	0.81	0.81	0.81	0.82	0.82	0.83	0.83	0.84	0.84	0.84	0.84	0.84	0.84	0.84	0.85	0.85	0.86	0.86	0.87	0.87	0.87	0.87
West Sussex	0.74	0.75	0.75	0.76	0.76	0.77	0.77	0.77	0.78	0.78	0.79	0.79	0.8	0.8	0.8	0.8	0.81	0.81	0.81	0.81	0.82	0.82	0.83	0.83	0.84	0.84	0.84	0.84
Windsor and Maidenhead	0.78	0.78	0.79	0.8	0.8	0.81	0.81	0.81	0.82	0.82	0.83	0.84	0.84	0.84	0.85	0.85	0.85	0.86	0.86	0.86	0.87	0.87	0.87	0.88	0.88	0.89	0.89	0.89
Wokingham	0.78	0.78	0.79	0.8	0.8	0.81	0.81	0.81	0.82	0.83	0.83	0.84	0.84	0.85	0.85	0.85	0.86	0.86	0.86	0.86	0.87	0.87	0.87	0.88	0.88	0.88	0.88	0.89
South West England	0.73	0.74	0.74	0.75	0.75	0.76	0.76	0.77	0.77	0.78	0.78	0.79	0.79	0.8	0.8	0.8	0.81	0.81	0.81	0.81	0.82	0.82	0.83	0.83	0.84	0.84	0.84	0.84
Bath and North East Somerset	0.75	0.76	0.76	0.77	0.78	0.78	0.79	0.79	0.8	0.8	0.81	0.82	0.82	0.83	0.83	0.84	0.84	0.85	0.85	0.85	0.86	0.86	0.86	0.87	0.87	0.87	0.87	0.88
Bournemouth	0.74	0.74	0.75	0.76	0.76	0.77	0.77	0.78	0.78	0.79	0.8	0.8	0.81	0.81	0.82	0.82	0.82	0.83	0.83	0.83	0.84	0.84	0.84	0.85	0.85	0.85	0.86	0.86
Bristol, City of	0.76	0.77	0.78	0.78	0.79	0.8	0.8	0.81	0.81	0.82	0.82	0.83	0.83	0.84	0.84	0.84	0.85	0.85	0.85	0.86	0.86	0.86	0.87	0.87	0.88	0.88	0.88	0.88
Cornwall	0.7	0.71	0.71	0.72	0.73	0.73	0.73	0.74	0.74	0.75	0.76	0.76	0.76	0.77	0.77	0.77	0.78	0.78	0.78	0.79	0.79	0.79	0.8	0.81	0.81	0.81	0.82	0.82
Devon	0.72	0.73	0.73	0.74	0.75	0.75	0.75	0.76	0.76	0.77	0.78	0.78	0.79	0.79	0.79	0.8	0.8	0.8	0.81	0.81	0.81	0.82	0.82	0.83	0.83	0.83	0.84	0.84
Dorset	0.72	0.72	0.73	0.73	0.74	0.74	0.75	0.75	0.76	0.76	0.77	0.77	0.78	0.78	0.78	0.78	0.79	0.79	0.79	0.79	0.8	0.8	0.81	0.81	0.82	0.82	0.82	0.83
Gloucestershire	0.74	0.74	0.75	0.75	0.76	0.77	0.77	0.77	0.78	0.78	0.79	0.8	0.8	0.8	0.81	0.81	0.81	0.82	0.82	0.82	0.82	0.83	0.83	0.84	0.84	0.85	0.85	0.85
North Somerset	0.71	0.72	0.73	0.73	0.74	0.74	0.75	0.75	0.76	0.76	0.77	0.77	0.78	0.78	0.78	0.79	0.79	0.79	0.8	0.8	0.8	0.81	0.81	0.82	0.82	0.83	0.83	0.83

eTable 1: Socio-Demographic Index values for all estimated GBD 2017 locations, 1990-2017

Location	1990	1991	1992	1993	1994	1995	1996	1997	1998	1999	2000	2001	2002	2003	2004	2005	2006	2007	2008	2009	2010	2011	2012	2013	2014	2015	2016	2017
Plymouth	0.72	0.73	0.74	0.74	0.75	0.75	0.76	0.76	0.77	0.77	0.78	0.78	0.79	0.79	0.79	0.8	0.8	0.8	0.81	0.81	0.81	0.81	0.82	0.83	0.83	0.83	0.83	0.84
Poole	0.73	0.73	0.74	0.75	0.75	0.76	0.76	0.77	0.77	0.78	0.78	0.79	0.79	0.8	0.8	0.8	0.8	0.81	0.81	0.81	0.81	0.82	0.82	0.83	0.84	0.84	0.84	0.84
Somerset	0.71	0.72	0.72	0.73	0.74	0.74	0.74	0.75	0.75	0.76	0.76	0.77	0.77	0.77	0.78	0.78	0.78	0.78	0.79	0.79	0.79	0.79	0.8	0.81	0.81	0.81	0.81	0.82
South Gloucestershire	0.75	0.75	0.76	0.77	0.77	0.78	0.78	0.78	0.79	0.8	0.8	0.81	0.81	0.82	0.82	0.82	0.83	0.83	0.83	0.84	0.84	0.84	0.85	0.86	0.86	0.86	0.87	0.87
Swindon	0.75	0.75	0.76	0.77	0.77	0.78	0.78	0.78	0.79	0.79	0.8	0.8	0.81	0.81	0.81	0.81	0.81	0.81	0.82	0.82	0.82	0.83	0.83	0.84	0.84	0.84	0.85	0.85
Torbay	0.7	0.71	0.71	0.72	0.72	0.73	0.73	0.73	0.74	0.74	0.75	0.75	0.75	0.75	0.75	0.76	0.76	0.76	0.76	0.76	0.76	0.77	0.77	0.78	0.78	0.79	0.79	0.79
Wiltshire	0.73	0.73	0.74	0.74	0.75	0.75	0.76	0.76	0.76	0.77	0.78	0.78	0.78	0.79	0.79	0.79	0.79	0.8	0.8	0.8	0.8	0.81	0.81	0.82	0.82	0.83	0.83	0.83
West Midlands	0.71	0.71	0.72	0.73	0.73	0.74	0.74	0.75	0.75	0.76	0.77	0.77	0.78	0.78	0.78	0.79	0.79	0.79	0.8	0.8	0.8	0.81	0.81	0.82	0.82	0.83	0.83	0.83
Birmingham	0.71	0.71	0.72	0.73	0.74	0.74	0.74	0.75	0.75	0.76	0.77	0.77	0.78	0.78	0.79	0.79	0.79	0.8	0.8	0.81	0.81	0.82	0.82	0.83	0.83	0.84	0.84	0.84
Coventry	0.72	0.73	0.74	0.75	0.75	0.76	0.76	0.77	0.77	0.78	0.79	0.79	0.8	0.8	0.8	0.81	0.81	0.81	0.82	0.82	0.82	0.83	0.83	0.84	0.84	0.84	0.85	0.85
Dudley	0.69	0.7	0.7	0.71	0.72	0.72	0.72	0.73	0.73	0.74	0.75	0.75	0.76	0.76	0.76	0.77	0.77	0.77	0.77	0.77	0.78	0.78	0.78	0.79	0.79	0.8	0.8	0.8
Herefordshire, County of	0.71	0.71	0.72	0.73	0.73	0.74	0.74	0.75	0.75	0.76	0.77	0.77	0.78	0.78	0.78	0.79	0.79	0.79	0.8	0.8	0.8	0.81	0.81	0.82	0.82	0.83	0.83	0.83
Sandwell	0.68	0.69	0.7	0.7	0.71	0.71	0.72	0.72	0.72	0.73	0.74	0.74	0.74	0.75	0.75	0.75	0.75	0.76	0.76	0.76	0.77	0.77	0.78	0.78	0.79	0.79	0.8	0.8
Shropshire	0.71	0.71	0.72	0.73	0.73	0.74	0.74	0.75	0.75	0.76	0.77	0.77	0.78	0.78	0.79	0.79	0.79	0.8	0.8	0.8	0.81	0.81	0.82	0.82	0.83	0.83	0.83	0.83
Solihull	0.73	0.74	0.75	0.76	0.76	0.77	0.77	0.78	0.78	0.79	0.8	0.8	0.81	0.81	0.82	0.82	0.82	0.83	0.83	0.83	0.83	0.84	0.84	0.85	0.85	0.85	0.85	0.86
Staffordshire	0.71	0.71	0.72	0.73	0.74	0.74	0.74	0.75	0.76	0.76	0.77	0.77	0.78	0.78	0.78	0.79	0.79	0.79	0.8	0.8	0.8	0.81	0.81	0.82	0.82	0.82	0.82	0.83
Stoke-on-Trent	0.69	0.7	0.7	0.71	0.72	0.72	0.72	0.73	0.73	0.74	0.74	0.75	0.75	0.75	0.75	0.75	0.76	0.76	0.76	0.76	0.77	0.77	0.78	0.79	0.79	0.8	0.8	0.8
Telford and Wrekin	0.71	0.72	0.73	0.73	0.74	0.75	0.75	0.75	0.76	0.76	0.77	0.77	0.78	0.78	0.78	0.78	0.78	0.78	0.79	0.79	0.79	0.8	0.8	0.81	0.81	0.82	0.82	0.82
Walsall	0.68	0.69	0.69	0.7	0.7	0.71	0.71	0.71	0.72	0.72	0.73	0.73	0.74	0.74	0.74	0.75	0.75	0.75	0.76	0.76	0.76	0.77	0.77	0.78	0.78	0.79	0.79	0.79
Warwickshire	0.73	0.74	0.75	0.75	0.76	0.77	0.77	0.77	0.78	0.79	0.79	0.8	0.8	0.81	0.81	0.81	0.82	0.82	0.82	0.83	0.83	0.83	0.84	0.85	0.85	0.85	0.86	0.86
Wolverhampton	0.69	0.7	0.7	0.71	0.72	0.72	0.73	0.73	0.74	0.74	0.75	0.76	0.76	0.76	0.77	0.77	0.77	0.78	0.78	0.78	0.78	0.79	0.79	0.8	0.8	0.81	0.81	0.81
Worcestershire	0.71	0.71	0.72	0.73	0.73	0.74	0.74	0.75	0.75	0.76	0.77	0.78	0.78	0.79	0.79	0.79	0.8	0.8	0.8	0.81	0.81	0.81	0.82	0.82	0.83	0.83	0.83	0.83
Yorkshire and the Humber	0.71	0.72	0.72	0.73	0.74	0.74	0.75	0.75	0.76	0.76	0.77	0.77	0.78	0.78	0.79	0.79	0.79	0.79	0.8	0.8	0.8	0.81	0.81	0.82	0.82	0.83	0.83	0.83
Barnsley	0.68	0.68	0.69	0.69	0.7	0.7	0.71	0.71	0.72	0.72	0.73	0.73	0.74	0.74	0.74	0.74	0.75	0.75	0.75	0.75	0.76	0.76	0.77	0.77	0.78	0.78	0.79	0.79
Bradford	0.69	0.7	0.71	0.71	0.72	0.72	0.72	0.73	0.73	0.74	0.74	0.75	0.75	0.75	0.76	0.76	0.76	0.77	0.77	0.77	0.78	0.78	0.79	0.79	0.8	0.8	0.81	0.81
Calderdale	0.71	0.71	0.72	0.73	0.74	0.74	0.74	0.75	0.75	0.76	0.76	0.77	0.77	0.77	0.78	0.78	0.78	0.79	0.79	0.79	0.8	0.8	0.81	0.81	0.82	0.82	0.83	0.83
Doncaster	0.67	0.68	0.69	0.69	0.7	0.7	0.7	0.71	0.71	0.72	0.72	0.73	0.73	0.74	0.74	0.74	0.75	0.75	0.75	0.76	0.76	0.76	0.77	0.78	0.78	0.79	0.79	0.79
East Riding of Yorkshire	0.71	0.72	0.73	0.73	0.74	0.75	0.75	0.75	0.76	0.76	0.77	0.77	0.78	0.78	0.78	0.79	0.79	0.79	0.79	0.8	0.8	0.8	0.81	0.81	0.82	0.82	0.82	0.82
Kingston upon Hull, City of	0.69	0.7	0.7	0.71	0.72	0.72	0.73	0.73	0.74	0.74	0.75	0.76	0.76	0.76	0.77	0.77	0.77	0.78	0.78	0.78	0.79	0.79	0.8	0.8	0.81	0.81	0.81	0.81
Kirklees	0.7	0.71	0.72	0.72	0.73	0.73	0.74	0.74	0.75	0.75	0.76	0.76	0.77	0.77	0.77	0.78	0.78	0.78	0.78	0.79	0.79	0.79	0.8	0.81	0.81	0.81	0.81	0.82
Leeds	0.74	0.75	0.75	0.76	0.77	0.77	0.78	0.78	0.79	0.8	0.8	0.81	0.82	0.82	0.83	0.83	0.83	0.84	0.84	0.84	0.85	0.85	0.85	0.86	0.86	0.86	0.87	0.87
North East Lincolnshire	0.7	0.71	0.71	0.72	0.73	0.73	0.73	0.74	0.74	0.75	0.75	0.75	0.76	0.76	0.76	0.76	0.76	0.77	0.77	0.78	0.78	0.78	0.79	0.8	0.8	0.8	0.8	0.8
North Lincolnshire	0.7	0.71	0.71	0.72	0.73	0.73	0.73	0.74	0.74	0.75	0.75	0.76	0.76	0.76	0.76	0.77	0.77	0.77	0.77	0.78	0.78	0.79	0.79	0.8	0.8	0.81	0.81	0.81
North Yorkshire	0.72	0.73	0.74	0.74	0.75	0.75	0.76	0.76	0.77	0.77	0.78	0.79	0.79	0.79	0.8	0.8	0.8	0.81	0.81	0.81	0.81	0.82	0.82	0.83	0.83	0.83	0.84	0.84
Rotherham	0.68	0.69	0.69	0.7	0.71	0.71	0.71	0.72	0.72	0.73	0.73	0.74	0.74	0.75	0.75	0.75	0.76	0.76	0.76	0.76	0.77	0.77	0.78	0.78	0.79	0.79	0.79	0.8

eTable 1: Socio-Demographic Index values for all estimated GBD 2017 locations, 1990-2017

Location	1990	1991	1992	1993	1994	1995	1996	1997	1998	1999	2000	2001	2002	2003	2004	2005	2006	2007	2008	2009	2010	2011	2012	2013	2014	2015	2016	2017
Sheffield	0.73	0.73	0.74	0.75	0.75	0.76	0.76	0.77	0.77	0.78	0.79	0.79	0.8	0.8	0.81	0.81	0.82	0.82	0.82	0.83	0.83	0.83	0.84	0.84	0.85	0.85	0.85	0.85
Wakefield	0.69	0.7	0.71	0.71	0.72	0.72	0.73	0.73	0.73	0.74	0.75	0.75	0.76	0.76	0.76	0.76	0.77	0.77	0.77	0.77	0.77	0.78	0.78	0.79	0.8	0.8	0.8	0.81
York	0.76	0.77	0.78	0.78	0.79	0.8	0.8	0.8	0.81	0.82	0.82	0.83	0.83	0.84	0.84	0.85	0.85	0.85	0.86	0.86	0.86	0.87	0.87	0.87	0.87	0.88	0.88	0.88
Northern Ireland	0.71	0.72	0.73	0.74	0.74	0.75	0.75	0.76	0.76	0.77	0.77	0.78	0.79	0.79	0.8	0.8	0.8	0.81	0.81	0.81	0.82	0.82	0.82	0.83	0.83	0.83	0.83	0.84
Scotland	0.67	0.68	0.69	0.69	0.7	0.71	0.71	0.72	0.72	0.73	0.74	0.74	0.75	0.75	0.76	0.76	0.76	0.77	0.77	0.78	0.78	0.79	0.79	0.79	0.8	0.8	0.8	0.81
Wales	0.64	0.65	0.66	0.67	0.68	0.68	0.69	0.69	0.7	0.71	0.72	0.73	0.73	0.74	0.75	0.75	0.76	0.76	0.77	0.77	0.77	0.78	0.79	0.79	0.8	0.8	0.8	0.81
Latin America and Caribbean	0.5	0.5	0.51	0.51	0.52	0.53	0.53	0.54	0.55	0.55	0.56	0.56	0.57	0.57	0.58	0.58	0.59	0.59	0.6	0.6	0.61	0.61	0.62	0.62	0.63	0.63	0.64	0.64
Andean Latin America	0.48	0.49	0.49	0.5	0.5	0.51	0.51	0.52	0.53	0.53	0.54	0.55	0.55	0.56	0.56	0.57	0.57	0.58	0.58	0.59	0.59	0.6	0.6	0.61	0.61	0.62	0.62	0.63
Bolivia	0.4	0.41	0.42	0.42	0.43	0.44	0.45	0.46	0.47	0.48	0.49	0.49	0.5	0.51	0.52	0.53	0.53	0.54	0.54	0.54	0.55	0.55	0.56	0.56	0.57	0.57	0.58	0.59
Ecuador	0.51	0.51	0.52	0.52	0.52	0.52	0.52	0.52	0.53	0.53	0.54	0.55	0.56	0.56	0.57	0.58	0.58	0.59	0.59	0.59	0.59	0.6	0.61	0.62	0.62	0.63	0.63	0.64
Peru	0.49	0.5	0.5	0.5	0.51	0.52	0.53	0.53	0.54	0.55	0.56	0.56	0.56	0.57	0.57	0.58	0.58	0.58	0.59	0.59	0.6	0.6	0.61	0.61	0.62	0.63	0.63	0.64
Caribbean	0.53	0.54	0.54	0.54	0.55	0.55	0.55	0.56	0.56	0.56	0.57	0.57	0.58	0.58	0.59	0.6	0.6	0.61	0.61	0.61	0.62	0.62	0.62	0.63	0.63	0.63	0.64	0.64
Antigua and Barbuda	0.61	0.62	0.62	0.63	0.63	0.63	0.64	0.64	0.64	0.65	0.65	0.66	0.66	0.67	0.67	0.68	0.68	0.69	0.69	0.69	0.7	0.7	0.7	0.7	0.71	0.71	0.71	0.72
The Bahamas	0.67	0.67	0.67	0.67	0.67	0.68	0.68	0.69	0.7	0.7	0.71	0.72	0.72	0.72	0.72	0.73	0.73	0.74	0.74	0.75	0.75	0.75	0.76	0.76	0.76	0.76	0.76	0.76
Barbados	0.65	0.66	0.66	0.67	0.67	0.68	0.68	0.68	0.68	0.68	0.69	0.69	0.69	0.7	0.7	0.71	0.71	0.71	0.72	0.72	0.72	0.73	0.73	0.73	0.73	0.74	0.74	0.74
Belize	0.4	0.41	0.43	0.45	0.46	0.47	0.48	0.49	0.5	0.5	0.51	0.51	0.52	0.53	0.53	0.54	0.55	0.56	0.56	0.57	0.58	0.58	0.59	0.59	0.59	0.6	0.6	0.6
Bermuda	0.73	0.73	0.73	0.74	0.74	0.74	0.74	0.74	0.75	0.75	0.75	0.75	0.76	0.76	0.77	0.77	0.77	0.78	0.78	0.79	0.79	0.79	0.8	0.8	0.8	0.8	0.81	0.81
Cuba	0.59	0.6	0.6	0.61	0.6	0.6	0.6	0.6	0.6	0.6	0.61	0.61	0.62	0.62	0.63	0.64	0.64	0.65	0.65	0.65	0.65	0.66	0.66	0.67	0.67	0.68	0.68	0.69
Dominica	0.53	0.54	0.54	0.55	0.56	0.56	0.57	0.58	0.58	0.59	0.6	0.61	0.61	0.62	0.63	0.64	0.64	0.65	0.65	0.66	0.66	0.67	0.67	0.68	0.68	0.68	0.68	0.69
Dominican Republic	0.44	0.44	0.45	0.45	0.45	0.46	0.46	0.47	0.48	0.48	0.48	0.49	0.5	0.5	0.51	0.53	0.54	0.55	0.56	0.56	0.57	0.57	0.58	0.58	0.58	0.58	0.59	0.59
Grenada	0.46	0.47	0.49	0.5	0.51	0.52	0.53	0.54	0.55	0.56	0.57	0.57	0.58	0.58	0.59	0.59	0.6	0.6	0.61	0.61	0.62	0.62	0.62	0.62	0.63	0.63	0.64	0.64
Guyana	0.44	0.45	0.45	0.45	0.46	0.47	0.47	0.48	0.49	0.5	0.5	0.51	0.52	0.52	0.53	0.53	0.53	0.54	0.54	0.55	0.55	0.56	0.56	0.57	0.57	0.58	0.58	0.58
Haiti	0.33	0.33	0.34	0.34	0.34	0.35	0.35	0.36	0.36	0.37	0.37	0.38	0.38	0.39	0.39	0.4	0.4	0.41	0.41	0.41	0.42	0.42	0.42	0.43	0.43	0.44	0.44	0.44
Jamaica	0.55	0.55	0.56	0.57	0.57	0.58	0.59	0.59	0.6	0.6	0.61	0.62	0.62	0.63	0.63	0.64	0.64	0.64	0.65	0.65	0.66	0.66	0.67	0.67	0.67	0.68	0.68	0.68
Puerto Rico	0.68	0.69	0.69	0.7	0.7	0.71	0.71	0.72	0.72	0.73	0.74	0.75	0.75	0.76	0.76	0.76	0.77	0.77	0.77	0.78	0.78	0.79	0.79	0.8	0.81	0.81	0.81	0.81
Saint Lucia	0.51	0.52	0.52	0.53	0.54	0.55	0.56	0.57	0.57	0.58	0.59	0.59	0.59	0.6	0.6	0.61	0.61	0.61	0.62	0.62	0.63	0.63	0.64	0.64	0.64	0.65	0.65	0.65
Saint Vincent and the Grenadines	0.47	0.47	0.48	0.49	0.49	0.5	0.51	0.51	0.52	0.52	0.53	0.54	0.54	0.55	0.55	0.56	0.56	0.57	0.57	0.58	0.58	0.58	0.59	0.59	0.6	0.6	0.61	0.61
Suriname	0.53	0.53	0.54	0.54	0.54	0.54	0.54	0.55	0.55	0.56	0.56	0.57	0.58	0.59	0.59	0.6	0.6	0.6	0.61	0.61	0.62	0.62	0.63	0.63	0.63	0.64	0.64	0.64
Trinidad and Tobago	0.6	0.6	0.61	0.61	0.62	0.62	0.62	0.63	0.63	0.64	0.65	0.65	0.66	0.66	0.67	0.68	0.68	0.69	0.69	0.69	0.69	0.69	0.7	0.7	0.7	0.7	0.7	0.7
Virgin Islands	0.67	0.68	0.69	0.7	0.71	0.71	0.72	0.73	0.73	0.73	0.74	0.74	0.75	0.76	0.76	0.77	0.77	0.78	0.79	0.79	0.8	0.8	0.8	0.8	0.81	0.81	0.81	0.81
Central Latin America	0.49	0.5	0.5	0.51	0.52	0.52	0.53	0.53	0.54	0.55	0.55	0.56	0.56	0.56	0.57	0.57	0.58	0.58	0.59	0.59	0.6	0.6	0.61	0.61	0.61	0.62	0.62	0.62
Colombia	0.48	0.48	0.49	0.49	0.5	0.5	0.51	0.52	0.52	0.53	0.53	0.54	0.54	0.54	0.55	0.55	0.56	0.57	0.57	0.58	0.59	0.6	0.6	0.61	0.62	0.62	0.63	0.63
Costa Rica	0.52	0.53	0.54	0.54	0.55	0.55	0.56	0.56	0.57	0.57	0.58	0.59	0.59	0.6	0.6	0.61	0.61	0.61	0.62	0.62	0.63	0.63	0.64	0.65	0.65	0.65	0.66	0.66
El Salvador	0.41	0.41	0.41	0.42	0.42	0.43	0.44	0.45	0.46	0.47	0.48	0.49	0.5	0.51	0.52	0.52	0.53	0.54	0.55	0.55	0.56	0.56	0.57	0.57	0.58	0.58	0.59	0.59
Guatemala	0.31	0.32	0.33	0.34	0.35	0.36	0.37	0.38	0.4	0.41	0.42	0.43	0.43	0.44	0.45	0.46	0.47	0.47	0.48	0.48	0.49	0.5	0.5	0.51	0.51	0.52	0.52	0.52
Honduras	0.34	0.34	0.35	0.35	0.36	0.37	0.37	0.38	0.39	0.4	0.41	0.42	0.43	0.44	0.45	0.45	0.46	0.47	0.47	0.48	0.48	0.49	0.49	0.5	0.5	0.5	0.51	0.51
Mexico	0.51	0.52	0.52	0.53	0.54	0.54	0.55	0.56	0.56	0.57	0.57	0.58	0.58	0.59	0.59	0.6	0.6	0.6	0.61	0.61	0.61	0.61	0.62	0.62	0.62	0.62	0.63	0.63

eTable 1: Socio-Demographic Index values for all estimated GBD 2017 locations, 1990-2017

Location	1990	1991	1992	1993	1994	1995	1996	1997	1998	1999	2000	2001	2002	2003	2004	2005	2006	2007	2008	2009	2010	2011	2012	2013	2014	2015	2016	2017
Aguascalientes	0.56	0.56	0.57	0.57	0.58	0.58	0.59	0.6	0.6	0.61	0.61	0.61	0.62	0.62	0.63	0.63	0.64	0.64	0.64	0.64	0.65	0.65	0.65	0.65	0.65	0.66	0.66	0.66
Baja California	0.57	0.57	0.58	0.58	0.58	0.59	0.59	0.59	0.6	0.6	0.61	0.61	0.62	0.62	0.62	0.63	0.63	0.63	0.64	0.64	0.64	0.65	0.65	0.65	0.65	0.65	0.66	0.66
Baja California Sur	0.57	0.57	0.58	0.58	0.59	0.59	0.59	0.6	0.6	0.61	0.61	0.61	0.61	0.62	0.62	0.62	0.63	0.63	0.64	0.64	0.64	0.65	0.65	0.65	0.65	0.66	0.66	0.66
Campeche	0.49	0.49	0.5	0.5	0.51	0.52	0.53	0.53	0.54	0.55	0.56	0.56	0.57	0.57	0.58	0.58	0.59	0.59	0.59	0.6	0.6	0.6	0.6	0.61	0.61	0.61	0.61	0.62
Chiapas	0.4	0.41	0.41	0.42	0.43	0.44	0.44	0.45	0.46	0.47	0.47	0.48	0.48	0.48	0.49	0.49	0.5	0.5	0.51	0.51	0.51	0.52	0.52	0.52	0.53	0.53	0.53	0.53
Chihuahua	0.55	0.55	0.55	0.56	0.56	0.56	0.56	0.57	0.57	0.57	0.58	0.58	0.59	0.59	0.59	0.6	0.6	0.61	0.62	0.62	0.62	0.63	0.63	0.63	0.63	0.64	0.64	0.64
Coahuila	0.55	0.56	0.56	0.57	0.57	0.58	0.58	0.59	0.59	0.6	0.6	0.6	0.61	0.61	0.62	0.62	0.62	0.63	0.63	0.63	0.63	0.64	0.64	0.64	0.64	0.64	0.64	0.65
Colima	0.54	0.54	0.55	0.56	0.56	0.57	0.57	0.58	0.58	0.59	0.59	0.6	0.6	0.61	0.61	0.62	0.62	0.63	0.63	0.63	0.64	0.64	0.64	0.65	0.65	0.65	0.65	0.65
Mexico City	0.6	0.61	0.61	0.62	0.63	0.64	0.65	0.66	0.66	0.67	0.67	0.68	0.68	0.69	0.69	0.69	0.7	0.7	0.7	0.7	0.7	0.7	0.71	0.71	0.71	0.71	0.71	0.72
Durango	0.51	0.51	0.51	0.52	0.52	0.52	0.53	0.54	0.54	0.55	0.56	0.56	0.57	0.57	0.58	0.59	0.59	0.6	0.6	0.61	0.61	0.61	0.61	0.61	0.62	0.62	0.62	0.62
Guanajuato	0.49	0.5	0.5	0.51	0.52	0.53	0.53	0.54	0.55	0.55	0.56	0.57	0.57	0.58	0.58	0.58	0.59	0.59	0.6	0.6	0.6	0.6	0.61	0.61	0.61	0.62	0.62	0.62
Guerrero	0.43	0.43	0.43	0.44	0.45	0.45	0.46	0.47	0.47	0.48	0.49	0.49	0.5	0.51	0.51	0.52	0.52	0.53	0.54	0.54	0.54	0.55	0.55	0.55	0.56	0.56	0.56	0.56
Hidalgo	0.42	0.42	0.43	0.44	0.45	0.46	0.47	0.48	0.49	0.5	0.51	0.52	0.53	0.53	0.54	0.54	0.54	0.55	0.55	0.56	0.56	0.57	0.57	0.58	0.58	0.58	0.59	0.59
Jalisco	0.53	0.54	0.55	0.55	0.56	0.56	0.57	0.58	0.58	0.59	0.59	0.6	0.6	0.61	0.61	0.62	0.62	0.62	0.63	0.63	0.63	0.64	0.64	0.64	0.64	0.65	0.65	0.65
México	0.53	0.52	0.53	0.54	0.56	0.57	0.57	0.58	0.58	0.59	0.59	0.6	0.6	0.6	0.61	0.61	0.61	0.61	0.61	0.61	0.62	0.62	0.62	0.63	0.63	0.63	0.63	0.64
Michoacán de Ocampo	0.46	0.47	0.48	0.48	0.49	0.49	0.5	0.51	0.52	0.52	0.53	0.53	0.54	0.54	0.55	0.55	0.56	0.56	0.56	0.57	0.57	0.57	0.57	0.58	0.58	0.58	0.58	0.59
Morelos	0.53	0.53	0.54	0.55	0.55	0.56	0.56	0.57	0.58	0.58	0.59	0.59	0.6	0.6	0.6	0.61	0.61	0.61	0.62	0.62	0.62	0.62	0.62	0.63	0.63	0.63	0.63	0.64
Nayarit	0.5	0.5	0.51	0.51	0.52	0.52	0.53	0.54	0.55	0.55	0.56	0.57	0.57	0.58	0.58	0.59	0.59	0.59	0.6	0.6	0.6	0.6	0.61	0.61	0.61	0.62	0.62	0.62
Nuevo León	0.6	0.6	0.61	0.61	0.62	0.62	0.62	0.63	0.63	0.64	0.64	0.64	0.65	0.65	0.65	0.66	0.66	0.66	0.67	0.67	0.67	0.67	0.67	0.67	0.67	0.68	0.68	0.68
Oaxaca	0.43	0.44	0.44	0.45	0.45	0.46	0.46	0.47	0.47	0.48	0.49	0.49	0.5	0.5	0.51	0.51	0.52	0.52	0.53	0.53	0.54	0.54	0.55	0.55	0.55	0.56	0.56	0.56
Puebla	0.46	0.46	0.47	0.48	0.49	0.49	0.5	0.5	0.5	0.5	0.5	0.51	0.52	0.52	0.53	0.54	0.54	0.55	0.55	0.56	0.56	0.57	0.57	0.58	0.58	0.58	0.58	0.58
Querétaro	0.5	0.5	0.51	0.52	0.53	0.54	0.56	0.57	0.58	0.58	0.59	0.6	0.6	0.61	0.61	0.61	0.62	0.62	0.62	0.62	0.63	0.63	0.63	0.63	0.64	0.64	0.64	0.64
Quintana Roo	0.52	0.53	0.53	0.54	0.55	0.56	0.56	0.57	0.58	0.58	0.59	0.59	0.59	0.6	0.6	0.6	0.61	0.61	0.61	0.61	0.62	0.62	0.62	0.62	0.62	0.62	0.63	0.63
San Luis Potosí	0.48	0.49	0.49	0.5	0.51	0.51	0.52	0.53	0.54	0.55	0.55	0.56	0.56	0.57	0.57	0.58	0.58	0.59	0.59	0.6	0.6	0.6	0.61	0.61	0.61	0.62	0.62	0.62
Sinaloa	0.52	0.53	0.53	0.54	0.54	0.55	0.56	0.56	0.57	0.58	0.58	0.59	0.59	0.6	0.6	0.61	0.61	0.62	0.62	0.63	0.63	0.63	0.64	0.64	0.64	0.64	0.65	0.65
Sonora	0.55	0.56	0.56	0.57	0.57	0.57	0.58	0.58	0.59	0.59	0.6	0.6	0.61	0.61	0.61	0.62	0.62	0.63	0.63	0.63	0.64	0.64	0.64	0.64	0.65	0.65	0.65	0.65
Tabasco	0.47	0.48	0.49	0.49	0.5	0.51	0.52	0.52	0.53	0.54	0.55	0.55	0.56	0.56	0.57	0.57	0.58	0.58	0.59	0.59	0.59	0.6	0.6	0.6	0.6	0.61	0.61	0.61
Tamaulipas	0.55	0.55	0.56	0.56	0.57	0.57	0.57	0.58	0.59	0.59	0.6	0.6	0.61	0.61	0.61	0.62	0.62	0.62	0.63	0.63	0.63	0.64	0.64	0.64	0.64	0.64	0.65	0.65
Tlaxcala	0.48	0.48	0.49	0.5	0.51	0.52	0.52	0.53	0.54	0.54	0.55	0.55	0.56	0.56	0.57	0.57	0.58	0.58	0.59	0.59	0.59	0.59	0.6	0.6	0.6	0.6	0.6	0.6
Veracruz de Ignacio de la Llave	0.46	0.46	0.47	0.47	0.48	0.48	0.49	0.49	0.5	0.51	0.52	0.52	0.53	0.53	0.54	0.55	0.55	0.56	0.56	0.57	0.57	0.57	0.58	0.58	0.58	0.59	0.59	0.59
Yucatán	0.5	0.5	0.51	0.52	0.52	0.53	0.54	0.55	0.56	0.56	0.57	0.58	0.58	0.59	0.59	0.6	0.6	0.6	0.61	0.61	0.61	0.61	0.62	0.62	0.62	0.63	0.63	0.63
Zacatecas	0.48	0.49	0.5	0.5	0.51	0.51	0.52	0.53	0.53	0.54	0.55	0.55	0.56	0.56	0.56	0.57	0.57	0.58	0.58	0.58	0.59	0.59	0.59	0.6	0.6	0.6	0.61	0.61
Nicaragua	0.36	0.36	0.37	0.37	0.38	0.39	0.4	0.41	0.42	0.42	0.43	0.44	0.45	0.45	0.46	0.47	0.47	0.48	0.48	0.49	0.49	0.5	0.5	0.51	0.51	0.52	0.53	0.53
Panama	0.54	0.55	0.55	0.56	0.56	0.57	0.57	0.57	0.58	0.58	0.59	0.6	0.6	0.6	0.61	0.61	0.61	0.62	0.62	0.63	0.63	0.64	0.64	0.65	0.66	0.66	0.67	0.68
Venezuela	0.53	0.54	0.55	0.56	0.57	0.58	0.59	0.59	0.59	0.59	0.59	0.6	0.6	0.59	0.58	0.58	0.59	0.61	0.62	0.63	0.63	0.63	0.64	0.65	0.65	0.65	0.65	0.66
Tropical Latin America	0.49	0.5	0.51	0.51	0.52	0.53	0.54	0.54	0.55	0.56	0.56	0.57	0.57	0.58	0.58	0.59	0.59	0.6	0.61	0.61	0.62	0.63	0.64	0.64	0.65	0.65	0.66	0.66
Brazil	0.49	0.5	0.51	0.52	0.52	0.53	0.54	0.55	0.55	0.56	0.56	0.57	0.57	0.58	0.58	0.59	0.6	0.6	0.61	0.62	0.62	0.63	0.64	0.64	0.65	0.66	0.66	0.66

eTable 1: Socio-Demographic Index values for all estimated GBD 2017 locations, 1990-2017

Location	1990	1991	1992	1993	1994	1995	1996	1997	1998	1999	2000	2001	2002	2003	2004	2005	2006	2007	2008	2009	2010	2011	2012	2013	2014	2015	2016	2017
Acre	0.38	0.39	0.4	0.41	0.42	0.42	0.44	0.45	0.45	0.46	0.47	0.47	0.48	0.49	0.49	0.5	0.51	0.52	0.53	0.54	0.55	0.56	0.57	0.58	0.58	0.59	0.6	0.6
Alagoas	0.36	0.36	0.37	0.38	0.39	0.4	0.4	0.41	0.42	0.43	0.43	0.44	0.44	0.45	0.46	0.46	0.47	0.48	0.49	0.5	0.51	0.51	0.52	0.53	0.54	0.55	0.55	0.56
Amapá	0.47	0.48	0.48	0.49	0.5	0.51	0.52	0.53	0.53	0.54	0.55	0.55	0.56	0.56	0.57	0.58	0.58	0.59	0.6	0.61	0.61	0.62	0.63	0.64	0.64	0.65	0.66	0.66
Amazonas	0.44	0.45	0.46	0.47	0.48	0.48	0.49	0.5	0.51	0.51	0.51	0.52	0.52	0.53	0.53	0.54	0.55	0.55	0.56	0.57	0.58	0.59	0.59	0.6	0.61	0.62	0.63	0.63
Bahia	0.4	0.41	0.42	0.43	0.44	0.44	0.45	0.46	0.47	0.47	0.48	0.48	0.49	0.49	0.5	0.5	0.51	0.52	0.53	0.53	0.54	0.55	0.56	0.57	0.58	0.58	0.59	0.59
Ceará	0.41	0.42	0.43	0.43	0.44	0.45	0.46	0.46	0.47	0.48	0.48	0.49	0.49	0.5	0.51	0.51	0.52	0.53	0.54	0.54	0.55	0.56	0.57	0.58	0.58	0.59	0.6	0.6
Distrito Federal	0.63	0.64	0.64	0.65	0.66	0.66	0.67	0.68	0.69	0.69	0.7	0.7	0.71	0.71	0.72	0.73	0.73	0.74	0.74	0.75	0.76	0.76	0.77	0.78	0.78	0.79	0.79	0.79
Espírito Santo	0.5	0.51	0.52	0.52	0.53	0.54	0.55	0.56	0.56	0.57	0.58	0.58	0.59	0.59	0.6	0.61	0.61	0.62	0.63	0.63	0.64	0.64	0.65	0.66	0.66	0.67	0.67	0.68
Goiás	0.46	0.47	0.48	0.48	0.49	0.5	0.51	0.52	0.53	0.53	0.54	0.55	0.55	0.56	0.56	0.57	0.58	0.59	0.59	0.6	0.61	0.62	0.62	0.63	0.64	0.64	0.65	0.65
Maranhão	0.31	0.32	0.33	0.34	0.35	0.36	0.36	0.37	0.38	0.38	0.38	0.39	0.39	0.39	0.4	0.4	0.41	0.42	0.43	0.44	0.45	0.46	0.47	0.48	0.49	0.5	0.5	0.51
Mato Grosso	0.48	0.48	0.49	0.5	0.51	0.52	0.53	0.54	0.54	0.55	0.55	0.56	0.56	0.57	0.58	0.58	0.59	0.6	0.6	0.61	0.62	0.63	0.63	0.64	0.65	0.65	0.66	0.66
Mato Grosso do Sul	0.47	0.47	0.48	0.49	0.5	0.51	0.52	0.52	0.53	0.54	0.54	0.55	0.56	0.56	0.57	0.57	0.58	0.59	0.59	0.6	0.61	0.61	0.62	0.63	0.64	0.64	0.65	0.65
Minas Gerais	0.49	0.5	0.51	0.51	0.52	0.53	0.54	0.55	0.55	0.56	0.56	0.57	0.57	0.58	0.59	0.59	0.6	0.6	0.61	0.62	0.62	0.63	0.64	0.64	0.65	0.65	0.66	0.66
Pará	0.41	0.42	0.43	0.43	0.44	0.45	0.45	0.46	0.47	0.47	0.47	0.48	0.48	0.48	0.49	0.49	0.5	0.51	0.51	0.52	0.53	0.54	0.55	0.55	0.56	0.57	0.58	0.58
Paraíba	0.4	0.41	0.41	0.42	0.43	0.43	0.44	0.45	0.45	0.46	0.46	0.47	0.47	0.47	0.48	0.49	0.49	0.5	0.51	0.52	0.53	0.54	0.54	0.55	0.56	0.57	0.57	0.57
Paraná	0.51	0.52	0.53	0.53	0.54	0.55	0.56	0.56	0.57	0.58	0.59	0.59	0.6	0.6	0.61	0.62	0.62	0.63	0.63	0.64	0.65	0.65	0.66	0.66	0.67	0.68	0.68	0.68
Pernambuco	0.42	0.42	0.43	0.44	0.44	0.45	0.46	0.47	0.47	0.48	0.48	0.49	0.49	0.5	0.5	0.51	0.52	0.53	0.53	0.54	0.55	0.56	0.56	0.57	0.58	0.59	0.59	0.59
Piauí	0.37	0.37	0.38	0.39	0.39	0.4	0.41	0.42	0.42	0.43	0.43	0.43	0.44	0.44	0.45	0.46	0.47	0.47	0.48	0.49	0.5	0.51	0.52	0.53	0.54	0.54	0.55	0.55
Rio de Janeiro	0.58	0.58	0.59	0.59	0.6	0.6	0.61	0.61	0.62	0.62	0.63	0.63	0.64	0.64	0.65	0.65	0.66	0.66	0.67	0.67	0.68	0.68	0.69	0.69	0.7	0.7	0.71	0.71
Rio Grande do Norte	0.42	0.42	0.43	0.44	0.44	0.45	0.46	0.47	0.47	0.48	0.49	0.49	0.5	0.5	0.51	0.52	0.52	0.53	0.54	0.55	0.56	0.57	0.58	0.58	0.59	0.6	0.6	0.61
Rio Grande do Sul	0.54	0.55	0.56	0.56	0.57	0.57	0.58	0.59	0.59	0.6	0.6	0.61	0.61	0.62	0.62	0.63	0.64	0.64	0.65	0.65	0.66	0.67	0.67	0.68	0.68	0.69	0.69	0.69
Rondônia	0.42	0.43	0.44	0.45	0.46	0.47	0.48	0.48	0.49	0.5	0.5	0.51	0.52	0.52	0.53	0.54	0.54	0.55	0.56	0.57	0.58	0.58	0.59	0.6	0.61	0.61	0.62	0.62
Roraima	0.43	0.44	0.45	0.46	0.47	0.47	0.48	0.49	0.5	0.5	0.51	0.51	0.52	0.53	0.53	0.54	0.55	0.56	0.57	0.58	0.59	0.6	0.61	0.62	0.63	0.64	0.64	0.65
Santa Catarina	0.54	0.55	0.55	0.56	0.57	0.57	0.58	0.59	0.6	0.6	0.61	0.61	0.62	0.62	0.63	0.64	0.64	0.65	0.65	0.66	0.67	0.67	0.68	0.68	0.69	0.7	0.7	0.7
São Paulo	0.56	0.57	0.57	0.58	0.59	0.6	0.6	0.61	0.62	0.62	0.63	0.64	0.64	0.65	0.65	0.66	0.66	0.67	0.67	0.68	0.69	0.69	0.7	0.7	0.71	0.71	0.72	0.72
Sergipe	0.43	0.43	0.44	0.45	0.46	0.46	0.47	0.48	0.49	0.49	0.5	0.51	0.51	0.52	0.52	0.53	0.54	0.55	0.55	0.56	0.57	0.58	0.59	0.59	0.6	0.61	0.61	0.62
Tocantins	0.4	0.4	0.41	0.42	0.43	0.44	0.45	0.45	0.46	0.47	0.47	0.48	0.48	0.49	0.5	0.51	0.52	0.53	0.54	0.55	0.56	0.57	0.58	0.59	0.59	0.6	0.61	0.61
Paraguay	0.47	0.47	0.48	0.48	0.49	0.49	0.5	0.5	0.51	0.52	0.53	0.53	0.54	0.54	0.55	0.55	0.56	0.56	0.57	0.57	0.57	0.58	0.58	0.59	0.6	0.61	0.61	0.62
North Africa and Middle East	0.46	0.46	0.47	0.48	0.49	0.49	0.5	0.51	0.51	0.52	0.53	0.54	0.55	0.55	0.56	0.57	0.57	0.58	0.59	0.6	0.6	0.61	0.61	0.62	0.62	0.63	0.64	0.64
North Africa and Middle East	0.46	0.46	0.47	0.48	0.49	0.49	0.5	0.51	0.51	0.52	0.53	0.54	0.55	0.55	0.56	0.57	0.57	0.58	0.59	0.6	0.6	0.61	0.61	0.62	0.62	0.63	0.64	0.64
Afghanistan	0.15	0.15	0.15	0.15	0.15	0.15	0.15	0.15	0.15	0.16	0.16	0.16	0.16	0.17	0.18	0.18	0.19	0.2	0.21	0.22	0.23	0.24	0.25	0.26	0.27	0.28	0.28	0.29
Algeria	0.5	0.5	0.51	0.52	0.53	0.54	0.55	0.56	0.57	0.57	0.58	0.59	0.6	0.61	0.62	0.63	0.63	0.64	0.65	0.65	0.66	0.67	0.67	0.68	0.68	0.69	0.69	0.7
Bahrain	0.61	0.62	0.62	0.63	0.64	0.64	0.64	0.65	0.65	0.66	0.66	0.67	0.67	0.68	0.68	0.68	0.69	0.69	0.69	0.7	0.7	0.7	0.7	0.7	0.7	0.71	0.71	0.71
Egypt	0.44	0.45	0.46	0.47	0.48	0.49	0.49	0.5	0.51	0.51	0.52	0.53	0.53	0.54	0.54	0.55	0.55	0.55	0.56	0.56	0.56	0.57	0.56	0.57	0.57	0.58	0.6	0.6
Iran	0.5	0.51	0.52	0.53	0.54	0.55	0.55	0.56	0.56	0.57	0.59	0.6	0.62	0.63	0.63	0.64	0.65	0.66	0.66	0.67	0.67	0.68	0.68	0.68	0.69	0.69	0.7	0.7
Iraq	0.43	0.43	0.44	0.44	0.44	0.45	0.45	0.46	0.46	0.47	0.48	0.49	0.49	0.5	0.5	0.51	0.51	0.52	0.52	0.53	0.54	0.54	0.55	0.56	0.57	0.57	0.58	0.59
Jordan	0.55	0.56	0.56	0.57	0.57	0.57	0.58	0.58	0.59	0.59	0.6	0.6	0.6	0.61	0.61	0.62	0.62	0.63	0.63	0.64	0.65	0.66	0.67	0.68	0.69	0.69	0.69	0.7

eTable 1: Socio-Demographic Index values for all estimated GBD 2017 locations, 1990-2017

Location	1990	1991	1992	1993	1994	1995	1996	1997	1998	1999	2000	2001	2002	2003	2004	2005	2006	2007	2008	2009	2010	2011	2012	2013	2014	2015	2016	2017
Kuwait	0.66	0.65	0.64	0.64	0.63	0.63	0.64	0.65	0.67	0.67	0.68	0.68	0.69	0.69	0.69	0.7	0.71	0.72	0.73	0.74	0.74	0.75	0.75	0.76	0.77	0.78	0.78	0.79
Lebanon	0.52	0.53	0.54	0.54	0.55	0.56	0.57	0.58	0.59	0.6	0.6	0.61	0.62	0.63	0.64	0.64	0.65	0.66	0.67	0.68	0.69	0.69	0.7	0.71	0.72	0.72	0.73	0.73
Libya	0.65	0.66	0.67	0.68	0.69	0.7	0.71	0.71	0.72	0.73	0.73	0.73	0.74	0.74	0.75	0.75	0.76	0.77	0.77	0.78	0.78	0.78	0.78	0.78	0.78	0.77	0.77	0.76
Morocco	0.39	0.4	0.4	0.41	0.42	0.43	0.43	0.44	0.45	0.46	0.46	0.47	0.48	0.48	0.49	0.5	0.5	0.51	0.52	0.53	0.53	0.54	0.55	0.55	0.56	0.57	0.57	0.58
Palestine	0.36	0.37	0.37	0.38	0.38	0.39	0.4	0.41	0.41	0.42	0.43	0.43	0.43	0.44	0.44	0.44	0.45	0.45	0.46	0.47	0.48	0.49	0.5	0.51	0.52	0.53	0.54	0.54
Oman	0.47	0.49	0.5	0.51	0.52	0.53	0.55	0.56	0.57	0.58	0.59	0.61	0.62	0.63	0.64	0.65	0.66	0.67	0.68	0.69	0.7	0.7	0.71	0.72	0.73	0.73	0.74	0.74
Qatar	0.6	0.62	0.63	0.64	0.65	0.66	0.67	0.67	0.68	0.69	0.7	0.7	0.71	0.71	0.72	0.72	0.73	0.73	0.73	0.74	0.74	0.74	0.75	0.75	0.76	0.76	0.76	0.77
Saudi Arabia	0.44	0.46	0.48	0.5	0.51	0.53	0.55	0.56	0.57	0.59	0.6	0.61	0.62	0.63	0.65	0.66	0.67	0.68	0.7	0.71	0.72	0.73	0.74	0.75	0.76	0.77	0.77	0.78
Sudan	0.22	0.23	0.24	0.24	0.25	0.26	0.27	0.28	0.28	0.29	0.3	0.31	0.32	0.33	0.34	0.35	0.36	0.37	0.38	0.39	0.4	0.41	0.43	0.44	0.45	0.46	0.47	0.48
Syria	0.39	0.39	0.4	0.41	0.42	0.43	0.44	0.45	0.46	0.47	0.47	0.48	0.49	0.5	0.51	0.52	0.53	0.54	0.55	0.56	0.57	0.58	0.58	0.59	0.6	0.6	0.61	0.61
Tunisia	0.45	0.46	0.47	0.48	0.49	0.5	0.51	0.52	0.53	0.54	0.55	0.56	0.57	0.58	0.59	0.6	0.61	0.62	0.63	0.63	0.64	0.65	0.65	0.66	0.66	0.67	0.67	0.68
Turkey	0.51	0.52	0.52	0.53	0.54	0.55	0.56	0.56	0.57	0.58	0.59	0.59	0.6	0.61	0.62	0.63	0.63	0.64	0.65	0.66	0.67	0.68	0.69	0.7	0.71	0.72	0.72	0.73
United Arab Emirates	0.62	0.64	0.65	0.66	0.68	0.69	0.7	0.71	0.72	0.73	0.73	0.74	0.75	0.76	0.76	0.77	0.77	0.78	0.78	0.78	0.79	0.79	0.79	0.79	0.79	0.79	0.79	0.8
Yemen	0.2	0.21	0.21	0.22	0.23	0.24	0.25	0.26	0.27	0.28	0.28	0.29	0.3	0.31	0.32	0.34	0.35	0.36	0.37	0.38	0.39	0.4	0.41	0.42	0.42	0.43	0.43	0.43
South Asia	0.31	0.32	0.32	0.33	0.34	0.35	0.35	0.36	0.36	0.37	0.38	0.39	0.4	0.4	0.41	0.42	0.43	0.44	0.45	0.46	0.46	0.47	0.48	0.49	0.5	0.52	0.53	0.53
South Asia	0.31	0.32	0.32	0.33	0.34	0.35	0.35	0.36	0.36	0.37	0.38	0.39	0.4	0.4	0.41	0.42	0.43	0.44	0.45	0.46	0.46	0.47	0.48	0.49	0.5	0.52	0.53	0.53
Bangladesh	0.26	0.26	0.27	0.28	0.29	0.3	0.31	0.31	0.32	0.33	0.34	0.34	0.35	0.35	0.36	0.36	0.37	0.38	0.39	0.39	0.4	0.41	0.42	0.43	0.44	0.44	0.45	0.46
Bhutan	0.32	0.33	0.34	0.35	0.36	0.37	0.38	0.39	0.4	0.41	0.42	0.43	0.44	0.45	0.46	0.47	0.48	0.49	0.5	0.51	0.52	0.53	0.53	0.54	0.55	0.56	0.56	0.57
India	0.32	0.32	0.33	0.34	0.35	0.35	0.36	0.37	0.37	0.38	0.39	0.4	0.4	0.41	0.42	0.43	0.44	0.45	0.46	0.47	0.47	0.48	0.49	0.5	0.52	0.53	0.54	0.55
Andhra Pradesh	0.28	0.29	0.29	0.3	0.31	0.32	0.33	0.34	0.34	0.35	0.36	0.37	0.38	0.4	0.41	0.42	0.42	0.43	0.44	0.45	0.46	0.47	0.47	0.49	0.5	0.52	0.53	0.54
Arunachal Pradesh	0.31	0.32	0.32	0.33	0.34	0.35	0.35	0.36	0.37	0.37	0.38	0.39	0.4	0.41	0.42	0.42	0.43	0.44	0.45	0.46	0.47	0.47	0.48	0.5	0.52	0.54	0.55	0.56
Assam	0.33	0.33	0.34	0.35	0.35	0.36	0.36	0.37	0.37	0.38	0.39	0.4	0.41	0.42	0.42	0.43	0.44	0.45	0.45	0.46	0.46	0.47	0.47	0.49	0.5	0.51	0.52	0.53
Bihar	0.29	0.29	0.3	0.3	0.3	0.3	0.3	0.3	0.29	0.29	0.29	0.29	0.3	0.3	0.3	0.31	0.32	0.32	0.33	0.34	0.35	0.36	0.37	0.38	0.4	0.41	0.43	0.43
Chhattisgarh	0.29	0.29	0.3	0.3	0.31	0.31	0.32	0.32	0.32	0.33	0.34	0.34	0.35	0.36	0.37	0.38	0.39	0.4	0.41	0.42	0.43	0.43	0.44	0.46	0.47	0.49	0.5	0.51
Delhi	0.49	0.49	0.5	0.52	0.52	0.53	0.53	0.54	0.54	0.55	0.56	0.57	0.58	0.58	0.59	0.6	0.61	0.62	0.63	0.64	0.64	0.65	0.66	0.67	0.69	0.7	0.71	0.72
Goa	0.5	0.51	0.51	0.52	0.53	0.53	0.54	0.55	0.56	0.57	0.58	0.59	0.6	0.61	0.62	0.63	0.63	0.64	0.66	0.67	0.67	0.68	0.69	0.7	0.71	0.72	0.73	0.74
Gujarat	0.35	0.35	0.36	0.37	0.38	0.39	0.4	0.4	0.41	0.42	0.43	0.43	0.44	0.45	0.46	0.47	0.48	0.49	0.5	0.51	0.52	0.52	0.53	0.54	0.56	0.57	0.58	0.58
Haryana	0.34	0.35	0.36	0.36	0.37	0.38	0.39	0.39	0.4	0.41	0.41	0.42	0.43	0.44	0.45	0.46	0.47	0.48	0.49	0.5	0.51	0.51	0.52	0.54	0.56	0.58	0.59	0.6
Himachal Pradesh	0.34	0.35	0.36	0.37	0.38	0.39	0.4	0.41	0.42	0.43	0.45	0.46	0.47	0.48	0.49	0.5	0.51	0.52	0.53	0.54	0.55	0.56	0.57	0.59	0.6	0.61	0.63	0.63
Jammu and Kashmir	0.33	0.34	0.34	0.35	0.36	0.36	0.37	0.38	0.39	0.4	0.42	0.43	0.44	0.45	0.47	0.48	0.49	0.49	0.5	0.51	0.52	0.53	0.54	0.55	0.56	0.57	0.58	0.59
Jharkhand	0.28	0.28	0.29	0.29	0.29	0.3	0.3	0.3	0.31	0.31	0.32	0.33	0.33	0.34	0.35	0.36	0.37	0.38	0.39	0.4	0.41	0.41	0.42	0.43	0.45	0.47	0.48	0.49
Karnataka	0.32	0.33	0.34	0.35	0.36	0.37	0.38	0.38	0.39	0.4	0.41	0.42	0.42	0.43	0.44	0.45	0.46	0.47	0.48	0.49	0.5	0.5	0.51	0.53	0.54	0.56	0.57	0.57
Kerala	0.4	0.41	0.42	0.43	0.44	0.45	0.46	0.46	0.47	0.48	0.5	0.51	0.52	0.53	0.54	0.55	0.56	0.57	0.57	0.58	0.59	0.59	0.6	0.61	0.63	0.64	0.65	0.66
Madhya Pradesh	0.28	0.29	0.29	0.3	0.3	0.31	0.32	0.32	0.32	0.33	0.33	0.34	0.35	0.36	0.36	0.37	0.37	0.38	0.38	0.39	0.39	0.4	0.41	0.43	0.45	0.47	0.48	0.49
Maharashtra	0.36	0.37	0.38	0.39	0.4	0.41	0.42	0.43	0.43	0.44	0.45	0.46	0.47	0.48	0.49	0.5	0.51	0.52	0.53	0.54	0.55	0.55	0.56	0.57	0.59	0.6	0.61	0.62
Manipur	0.38	0.39	0.39	0.4	0.41	0.41	0.42	0.42	0.43	0.44	0.45	0.45	0.46	0.47	0.48	0.49	0.5	0.51	0.51	0.52	0.53	0.53	0.54	0.55	0.56	0.57	0.58	0.59
Meghalaya	0.33	0.34	0.35	0.35	0.36	0.36	0.37	0.38	0.38	0.39	0.4	0.41	0.42	0.43	0.44	0.45	0.46	0.47	0.48	0.49	0.5	0.5	0.51	0.52	0.53	0.55	0.56	0.57
Mizoram	0.39	0.4	0.41	0.42	0.42	0.43	0.44	0.45	0.46	0.47	0.48	0.48	0.49	0.5	0.51	0.52	0.53	0.53	0.54	0.55	0.55	0.55	0.56	0.57	0.59	0.6	0.61	0.62

eTable 1: Socio-Demographic Index values for all estimated GBD 2017 locations, 1990-2017

Location	1990	1991	1992	1993	1994	1995	1996	1997	1998	1999	2000	2001	2002	2003	2004	2005	2006	2007	2008	2009	2010	2011	2012	2013	2014	2015	2016	2017
Nagaland	0.4	0.4	0.41	0.41	0.42	0.43	0.44	0.44	0.45	0.45	0.46	0.47	0.48	0.49	0.5	0.51	0.52	0.53	0.54	0.55	0.56	0.57	0.57	0.59	0.6	0.61	0.63	0.63
Odisha	0.28	0.29	0.29	0.3	0.31	0.31	0.32	0.33	0.33	0.34	0.35	0.36	0.36	0.37	0.38	0.39	0.4	0.42	0.43	0.43	0.44	0.45	0.46	0.47	0.49	0.5	0.52	0.52
Punjab	0.38	0.39	0.4	0.4	0.41	0.42	0.43	0.44	0.44	0.45	0.46	0.47	0.48	0.48	0.49	0.5	0.51	0.52	0.53	0.54	0.55	0.55	0.56	0.58	0.59	0.61	0.61	0.62
Rajasthan	0.26	0.27	0.27	0.28	0.29	0.3	0.3	0.31	0.31	0.32	0.33	0.34	0.35	0.36	0.37	0.38	0.39	0.39	0.4	0.4	0.41	0.42	0.42	0.44	0.46	0.47	0.48	0.49
Sikkim	0.34	0.35	0.36	0.36	0.37	0.38	0.38	0.39	0.4	0.4	0.41	0.42	0.43	0.44	0.45	0.46	0.47	0.48	0.49	0.51	0.53	0.54	0.56	0.57	0.59	0.61	0.62	0.63
Tamil Nadu	0.35	0.35	0.36	0.37	0.38	0.39	0.4	0.41	0.41	0.42	0.44	0.45	0.46	0.47	0.48	0.49	0.5	0.51	0.51	0.52	0.53	0.54	0.54	0.56	0.58	0.6	0.61	0.62
Telangana	0.29	0.29	0.3	0.31	0.32	0.32	0.33	0.34	0.34	0.35	0.36	0.37	0.38	0.4	0.41	0.42	0.43	0.44	0.45	0.47	0.48	0.49	0.5	0.52	0.54	0.55	0.57	0.58
Tripura	0.33	0.34	0.34	0.35	0.35	0.36	0.36	0.37	0.37	0.39	0.4	0.41	0.42	0.43	0.44	0.45	0.46	0.46	0.47	0.48	0.48	0.48	0.49	0.5	0.52	0.53	0.54	0.54
Uttar Pradesh	0.27	0.28	0.28	0.29	0.29	0.3	0.3	0.31	0.31	0.32	0.33	0.33	0.34	0.35	0.36	0.37	0.37	0.38	0.39	0.4	0.4	0.41	0.42	0.44	0.45	0.47	0.48	0.49
Uttarakhand	0.3	0.31	0.31	0.32	0.32	0.33	0.34	0.34	0.35	0.36	0.37	0.38	0.39	0.41	0.42	0.43	0.45	0.46	0.48	0.49	0.51	0.52	0.54	0.55	0.57	0.59	0.6	0.61
West Bengal	0.32	0.32	0.33	0.34	0.35	0.35	0.36	0.37	0.37	0.38	0.39	0.4	0.41	0.42	0.43	0.43	0.44	0.45	0.46	0.46	0.47	0.47	0.48	0.49	0.51	0.52	0.53	0.54
Union Territories other than Delhi	0.41	0.41	0.41	0.42	0.42	0.43	0.44	0.45	0.45	0.46	0.47	0.49	0.5	0.51	0.52	0.54	0.55	0.56	0.57	0.58	0.59	0.59	0.6	0.61	0.62	0.64	0.65	0.65
Nepal	0.22	0.22	0.23	0.24	0.24	0.25	0.26	0.27	0.27	0.28	0.29	0.3	0.31	0.32	0.32	0.33	0.34	0.35	0.36	0.36	0.37	0.38	0.39	0.4	0.41	0.42	0.42	0.43
Pakistan	0.31	0.31	0.31	0.32	0.32	0.33	0.34	0.34	0.35	0.36	0.37	0.38	0.38	0.39	0.4	0.4	0.41	0.42	0.43	0.44	0.44	0.45	0.46	0.47	0.47	0.48	0.49	0.49
Southeast Asia, East Asia, and Oceania	0.47	0.48	0.49	0.5	0.51	0.52	0.53	0.54	0.55	0.55	0.56	0.57	0.57	0.58	0.59	0.6	0.61	0.62	0.62	0.63	0.64	0.65	0.66	0.66	0.67	0.67	0.68	0.69
East Asia	0.46	0.48	0.49	0.5	0.5	0.52	0.53	0.54	0.55	0.56	0.57	0.58	0.58	0.59	0.6	0.61	0.62	0.63	0.64	0.65	0.66	0.67	0.68	0.69	0.69	0.7	0.7	0.71
China	0.46	0.47	0.48	0.49	0.5	0.51	0.52	0.54	0.55	0.55	0.56	0.57	0.58	0.59	0.6	0.61	0.62	0.63	0.64	0.65	0.66	0.67	0.68	0.68	0.69	0.7	0.69	0.71
North Korea	0.51	0.52	0.52	0.52	0.52	0.52	0.52	0.52	0.51	0.51	0.5	0.5	0.5	0.5	0.5	0.5	0.5	0.51	0.51	0.51	0.51	0.52	0.52	0.52	0.53	0.53	0.54	0.54
Taiwan (Province of China)	0.69	0.7	0.71	0.72	0.72	0.73	0.74	0.75	0.76	0.76	0.77	0.78	0.79	0.79	0.8	0.81	0.81	0.82	0.83	0.83	0.84	0.84	0.85	0.85	0.85	0.86	0.86	0.86
Oceania	0.41	0.41	0.41	0.41	0.42	0.42	0.42	0.42	0.43	0.43	0.43	0.43	0.43	0.43	0.44	0.44	0.44	0.44	0.44	0.44	0.45	0.45	0.45	0.46	0.46	0.46	0.47	0.47
American Samoa	0.61	0.61	0.62	0.62	0.62	0.63	0.63	0.64	0.64	0.64	0.65	0.65	0.66	0.66	0.66	0.67	0.67	0.67	0.67	0.68	0.68	0.68	0.69	0.69	0.69	0.7	0.7	0.7
Federated States of Micronesia	0.46	0.47	0.48	0.48	0.49	0.5	0.5	0.51	0.51	0.51	0.52	0.52	0.53	0.53	0.54	0.54	0.54	0.55	0.55	0.55	0.56	0.56	0.56	0.57	0.57	0.57	0.57	0.58
Fiji	0.53	0.54	0.54	0.55	0.55	0.56	0.56	0.57	0.57	0.58	0.59	0.59	0.59	0.6	0.6	0.6	0.61	0.61	0.61	0.61	0.62	0.62	0.62	0.62	0.63	0.63	0.64	0.64
Guam	0.7	0.7	0.69	0.69	0.7	0.7	0.71	0.72	0.73	0.74	0.75	0.76	0.77	0.77	0.77	0.77	0.77	0.77	0.77	0.77	0.78	0.78	0.78	0.79	0.79	0.79	0.79	0.79
Kiribati	0.36	0.36	0.36	0.36	0.36	0.37	0.37	0.37	0.37	0.38	0.38	0.38	0.39	0.39	0.39	0.39	0.4	0.4	0.4	0.4	0.4	0.4	0.41	0.41	0.41	0.42	0.42	0.43
Marshall Islands	0.41	0.42	0.43	0.44	0.44	0.45	0.46	0.46	0.47	0.47	0.47	0.48	0.48	0.49	0.49	0.49	0.5	0.5	0.51	0.51	0.52	0.52	0.53	0.53	0.54	0.54	0.55	0.55
Northern Mariana Islands	0.74	0.74	0.75	0.75	0.75	0.75	0.76	0.76	0.76	0.76	0.76	0.77	0.77	0.77	0.77	0.77	0.77	0.76	0.76	0.76	0.76	0.76	0.76	0.76	0.76	0.76	0.76	0.76
Papua New Guinea	0.32	0.32	0.32	0.33	0.34	0.34	0.34	0.35	0.35	0.35	0.36	0.36	0.36	0.36	0.36	0.37	0.37	0.37	0.38	0.38	0.38	0.39	0.39	0.4	0.4	0.41	0.42	0.42
Samoa	0.54	0.54	0.54	0.54	0.54	0.54	0.54	0.54	0.54	0.54	0.54	0.54	0.55	0.55	0.55	0.55	0.55	0.56	0.56	0.56	0.56	0.56	0.56	0.56	0.56	0.57	0.57	0.58
Solomon Islands	0.32	0.32	0.33	0.33	0.34	0.34	0.35	0.36	0.36	0.36	0.37	0.37	0.37	0.37	0.37	0.37	0.37	0.38	0.38	0.38	0.39	0.39	0.4	0.41	0.41	0.42	0.42	0.43
Tonga	0.52	0.53	0.53	0.54	0.54	0.55	0.55	0.56	0.56	0.56	0.57	0.57	0.57	0.58	0.58	0.58	0.59	0.59	0.59	0.6	0.6	0.6	0.61	0.61	0.61	0.62	0.62	0.63
Vanuatu	0.38	0.38	0.39	0.39	0.4	0.4	0.4	0.41	0.41	0.41	0.42	0.42	0.42	0.42	0.43	0.43	0.43	0.44	0.44	0.45	0.45	0.45	0.46	0.46	0.47	0.47	0.47	0.48
Southeast Asia	0.47	0.48	0.49	0.5	0.5	0.51	0.52	0.53	0.53	0.54	0.54	0.55	0.55	0.56	0.56	0.57	0.57	0.58	0.58	0.59	0.6	0.6	0.61	0.62	0.62	0.63	0.64	0.64
Cambodia	0.26	0.27	0.27	0.28	0.29	0.29	0.3	0.31	0.32	0.33	0.34	0.34	0.35	0.36	0.37	0.38	0.39	0.4	0.41	0.42	0.43	0.44	0.44	0.45	0.46	0.47	0.48	0.48
Indonesia	0.47	0.48	0.49	0.5	0.51	0.52	0.53	0.54	0.54	0.55	0.55	0.55	0.56	0.56	0.57	0.57	0.58	0.58	0.59	0.59	0.6	0.61	0.62	0.62	0.63	0.64	0.64	0.65
Laos	0.31	0.31	0.32	0.33	0.33	0.34	0.35	0.35	0.36	0.36	0.37	0.38	0.39	0.39	0.4	0.41	0.42	0.43	0.43	0.44	0.45	0.46	0.47	0.48	0.49	0.5	0.51	0.52

eTable 1: Socio-Demographic Index values for all estimated GBD 2017 locations, 1990-2017

Location	1990	1991	1992	1993	1994	1995	1996	1997	1998	1999	2000	2001	2002	2003	2004	2005	2006	2007	2008	2009	2010	2011	2012	2013	2014	2015	2016	2017
Malaysia	0.57	0.58	0.58	0.59	0.6	0.61	0.62	0.63	0.64	0.65	0.66	0.66	0.67	0.68	0.68	0.69	0.7	0.7	0.71	0.71	0.72	0.73	0.73	0.74	0.74	0.75	0.75	0.76
Maldives	0.39	0.4	0.41	0.42	0.44	0.45	0.46	0.48	0.49	0.51	0.52	0.53	0.54	0.55	0.55	0.56	0.57	0.58	0.58	0.59	0.6	0.61	0.62	0.63	0.64	0.64	0.65	0.66
Mauritius	0.55	0.56	0.57	0.58	0.59	0.6	0.6	0.61	0.61	0.62	0.62	0.63	0.63	0.64	0.65	0.65	0.66	0.66	0.67	0.68	0.68	0.69	0.69	0.7	0.71	0.71	0.72	0.72
Myanmar	0.33	0.33	0.34	0.34	0.35	0.35	0.36	0.37	0.38	0.38	0.39	0.4	0.41	0.42	0.43	0.45	0.46	0.47	0.48	0.49	0.5	0.51	0.52	0.53	0.54	0.54	0.55	0.56
Philippines	0.51	0.52	0.52	0.53	0.53	0.53	0.54	0.54	0.55	0.55	0.55	0.55	0.56	0.56	0.56	0.56	0.56	0.57	0.57	0.57	0.57	0.58	0.59	0.59	0.6	0.61	0.61	0.62
Sri Lanka	0.49	0.5	0.5	0.51	0.52	0.52	0.53	0.54	0.55	0.55	0.56	0.57	0.57	0.58	0.58	0.59	0.6	0.6	0.61	0.62	0.63	0.63	0.64	0.65	0.66	0.67	0.67	0.68
Seychelles	0.55	0.56	0.57	0.57	0.58	0.59	0.6	0.61	0.61	0.62	0.63	0.63	0.64	0.64	0.64	0.65	0.65	0.65	0.66	0.66	0.66	0.66	0.67	0.67	0.68	0.68	0.69	0.69
Thailand	0.5	0.51	0.53	0.53	0.54	0.55	0.56	0.57	0.57	0.57	0.58	0.59	0.59	0.6	0.61	0.61	0.62	0.62	0.63	0.64	0.64	0.65	0.65	0.66	0.67	0.67	0.68	0.68
Timor-Leste	0.28	0.28	0.29	0.3	0.3	0.31	0.31	0.32	0.33	0.32	0.32	0.33	0.33	0.35	0.36	0.38	0.4	0.42	0.44	0.45	0.46	0.47	0.48	0.49	0.5	0.5	0.5	0.51
Vietnam	0.41	0.41	0.42	0.43	0.44	0.44	0.45	0.46	0.47	0.48	0.48	0.49	0.5	0.5	0.51	0.52	0.53	0.53	0.54	0.55	0.55	0.56	0.57	0.58	0.59	0.59	0.6	0.61
Sub-Saharan Africa	0.3	0.31	0.31	0.31	0.32	0.32	0.32	0.33	0.33	0.34	0.34	0.34	0.35	0.35	0.36	0.37	0.37	0.38	0.39	0.39	0.4	0.41	0.41	0.42	0.43	0.44	0.44	0.45
Central sub-Saharan Africa	0.3	0.3	0.31	0.31	0.31	0.31	0.32	0.32	0.32	0.32	0.33	0.33	0.33	0.34	0.34	0.35	0.36	0.36	0.37	0.38	0.39	0.4	0.41	0.42	0.43	0.44	0.45	0.46
Angola	0.24	0.24	0.25	0.25	0.25	0.26	0.26	0.27	0.28	0.28	0.29	0.29	0.3	0.31	0.31	0.32	0.33	0.34	0.35	0.36	0.38	0.39	0.4	0.41	0.43	0.44	0.45	0.46
Central African Republic	0.22	0.23	0.23	0.23	0.24	0.24	0.24	0.25	0.25	0.25	0.26	0.26	0.27	0.27	0.27	0.28	0.28	0.29	0.29	0.3	0.3	0.31	0.32	0.33	0.33	0.33	0.33	0.33
Congo (Brazzaville)	0.38	0.39	0.4	0.41	0.41	0.42	0.42	0.43	0.43	0.43	0.44	0.44	0.45	0.46	0.46	0.47	0.48	0.48	0.49	0.5	0.51	0.52	0.53	0.54	0.55	0.56	0.57	0.57
DR Congo	0.29	0.3	0.3	0.3	0.3	0.29	0.29	0.29	0.28	0.28	0.27	0.27	0.27	0.26	0.26	0.26	0.26	0.27	0.27	0.27	0.28	0.29	0.3	0.32	0.33	0.34	0.36	0.36
Equatorial Guinea	0.2	0.2	0.21	0.22	0.23	0.24	0.26	0.29	0.32	0.34	0.36	0.39	0.41	0.43	0.45	0.47	0.48	0.5	0.52	0.53	0.54	0.56	0.57	0.59	0.6	0.61	0.62	0.63
Gabon	0.43	0.44	0.45	0.46	0.47	0.48	0.49	0.5	0.51	0.51	0.52	0.53	0.54	0.54	0.55	0.56	0.56	0.57	0.58	0.58	0.59	0.6	0.61	0.62	0.63	0.63	0.64	0.65
Eastern sub-Saharan Africa	0.23	0.23	0.24	0.24	0.24	0.25	0.25	0.25	0.26	0.26	0.27	0.27	0.28	0.28	0.29	0.29	0.3	0.31	0.32	0.32	0.33	0.34	0.35	0.36	0.37	0.37	0.38	0.39
Burundi	0.25	0.25	0.26	0.26	0.26	0.27	0.27	0.27	0.27	0.27	0.27	0.27	0.27	0.27	0.27	0.27	0.27	0.28	0.28	0.28	0.29	0.29	0.3	0.3	0.3	0.31	0.31	0.31
Comoros	0.27	0.28	0.29	0.29	0.3	0.3	0.31	0.31	0.31	0.32	0.33	0.33	0.34	0.34	0.35	0.36	0.37	0.37	0.38	0.38	0.39	0.4	0.4	0.41	0.42	0.42	0.43	0.43
Djibouti	0.31	0.32	0.32	0.32	0.33	0.33	0.33	0.34	0.34	0.34	0.34	0.35	0.36	0.37	0.39	0.4	0.41	0.41	0.42	0.43	0.43	0.44	0.45	0.45	0.46	0.47	0.48	0.49
Eritrea	0.2	0.21	0.22	0.23	0.25	0.26	0.27	0.29	0.3	0.31	0.32	0.32	0.33	0.34	0.34	0.35	0.35	0.36	0.36	0.36	0.37	0.37	0.38	0.38	0.39	0.4	0.4	0.41
Ethiopia	0.14	0.14	0.14	0.15	0.15	0.15	0.16	0.16	0.17	0.17	0.17	0.18	0.18	0.19	0.2	0.2	0.21	0.22	0.23	0.25	0.26	0.27	0.28	0.29	0.3	0.31	0.33	0.33
Kenya	0.34	0.35	0.36	0.36	0.37	0.38	0.38	0.39	0.39	0.4	0.4	0.4	0.41	0.41	0.42	0.42	0.43	0.43	0.44	0.45	0.45	0.46	0.47	0.47	0.48	0.49	0.49	0.5
Baringo	0.25	0.27	0.28	0.29	0.3	0.31	0.31	0.32	0.33	0.33	0.34	0.34	0.35	0.35	0.36	0.36	0.37	0.38	0.38	0.39	0.4	0.41	0.41	0.42	0.43	0.43	0.44	0.44
Bomet	0.31	0.32	0.33	0.33	0.34	0.35	0.35	0.36	0.36	0.37	0.37	0.37	0.38	0.39	0.39	0.4	0.41	0.41	0.42	0.43	0.44	0.45	0.46	0.47	0.48	0.48	0.49	0.5
Bungoma	0.32	0.33	0.33	0.34	0.35	0.35	0.36	0.36	0.37	0.37	0.37	0.37	0.38	0.38	0.38	0.39	0.39	0.4	0.4	0.41	0.42	0.42	0.43	0.44	0.45	0.45	0.46	0.46
Busia	0.3	0.3	0.31	0.32	0.33	0.33	0.34	0.34	0.34	0.35	0.35	0.35	0.36	0.36	0.37	0.37	0.38	0.38	0.39	0.39	0.4	0.4	0.41	0.42	0.42	0.43	0.43	0.44
Elgeyo Marakwet	0.29	0.3	0.31	0.32	0.33	0.34	0.34	0.35	0.36	0.36	0.37	0.37	0.38	0.39	0.39	0.4	0.41	0.42	0.43	0.44	0.44	0.45	0.46	0.47	0.48	0.48	0.49	0.5
Embu	0.38	0.38	0.39	0.4	0.41	0.41	0.42	0.42	0.43	0.43	0.43	0.44	0.44	0.44	0.45	0.45	0.46	0.46	0.47	0.48	0.49	0.49	0.5	0.51	0.51	0.52	0.53	0.53
Garissa	0.15	0.16	0.17	0.18	0.18	0.19	0.2	0.2	0.21	0.21	0.22	0.22	0.22	0.23	0.23	0.24	0.24	0.25	0.26	0.26	0.27	0.28	0.29	0.3	0.31	0.32	0.33	0.33
Homa Bay	0.21	0.22	0.23	0.24	0.25	0.26	0.27	0.27	0.28	0.29	0.29	0.29	0.3	0.31	0.31	0.32	0.33	0.34	0.35	0.36	0.37	0.37	0.38	0.39	0.4	0.41	0.42	0.43
Isiolo	0.26	0.27	0.28	0.28	0.29	0.29	0.3	0.3	0.3	0.31	0.31	0.31	0.31	0.31	0.32	0.32	0.33	0.33	0.34	0.34	0.35	0.36	0.36	0.37	0.37	0.38	0.38	0.39
Kajiado	0.38	0.39	0.4	0.41	0.41	0.42	0.43	0.43	0.43	0.44	0.44	0.45	0.45	0.45	0.46	0.46	0.46	0.47	0.48	0.48	0.49	0.49	0.5	0.51	0.51	0.52	0.53	0.53
Kakamega	0.3	0.3	0.31	0.32	0.33	0.33	0.34	0.34	0.35	0.36	0.36	0.36	0.37	0.37	0.38	0.38	0.38	0.39	0.39	0.4	0.41	0.41	0.42	0.43	0.43	0.44	0.45	0.45
Kericho	0.27	0.28	0.29	0.3	0.31	0.32	0.32	0.33	0.34	0.35	0.35	0.36	0.36	0.37	0.38	0.39	0.39	0.4	0.41	0.43	0.44	0.45	0.45	0.46	0.48	0.49	0.49	0.5
Kiambu	0.44	0.44	0.45	0.46	0.46	0.47	0.47	0.48	0.48	0.48	0.49	0.49	0.49	0.5	0.5	0.5	0.51	0.52	0.52	0.53	0.54	0.54	0.55	0.56	0.56	0.57	0.58	0.58

eTable 1: Socio-Demographic Index values for all estimated GBD 2017 locations, 1990-2017

Location	1990	1991	1992	1993	1994	1995	1996	1997	1998	1999	2000	2001	2002	2003	2004	2005	2006	2007	2008	2009	2010	2011	2012	2013	2014	2015	2016	2017
Kilifi	0.29	0.3	0.31	0.31	0.32	0.33	0.33	0.34	0.34	0.35	0.35	0.35	0.35	0.36	0.36	0.37	0.37	0.38	0.39	0.39	0.4	0.41	0.42	0.42	0.43	0.44	0.45	0.46
Kirinyaga	0.39	0.4	0.4	0.41	0.41	0.42	0.42	0.42	0.43	0.43	0.43	0.43	0.44	0.44	0.45	0.45	0.46	0.46	0.47	0.48	0.49	0.49	0.5	0.51	0.51	0.52	0.53	0.53
Kisii	0.34	0.35	0.36	0.37	0.38	0.38	0.39	0.4	0.4	0.41	0.41	0.41	0.42	0.42	0.43	0.43	0.44	0.45	0.45	0.46	0.47	0.48	0.49	0.5	0.5	0.51	0.52	0.52
Kisumu	0.32	0.33	0.33	0.34	0.35	0.36	0.36	0.36	0.37	0.38	0.38	0.38	0.39	0.4	0.4	0.41	0.42	0.42	0.43	0.44	0.45	0.46	0.47	0.48	0.48	0.49	0.5	0.5
Kitui	0.28	0.29	0.3	0.3	0.31	0.32	0.32	0.33	0.33	0.34	0.34	0.35	0.35	0.36	0.36	0.37	0.38	0.38	0.39	0.4	0.41	0.42	0.42	0.43	0.44	0.45	0.46	0.46
Kwale	0.29	0.3	0.31	0.31	0.32	0.33	0.33	0.33	0.34	0.34	0.34	0.35	0.35	0.35	0.36	0.36	0.37	0.37	0.38	0.39	0.4	0.41	0.41	0.42	0.43	0.44	0.45	0.46
Laikipia	0.35	0.35	0.36	0.37	0.38	0.38	0.39	0.39	0.4	0.4	0.41	0.41	0.42	0.42	0.43	0.44	0.45	0.46	0.47	0.48	0.49	0.5	0.51	0.52	0.53	0.54	0.55	0.56
Lamu	0.3	0.3	0.31	0.32	0.33	0.33	0.34	0.34	0.35	0.35	0.36	0.36	0.36	0.36	0.37	0.37	0.38	0.38	0.39	0.4	0.41	0.41	0.42	0.43	0.43	0.44	0.45	0.45
Machakos	0.35	0.36	0.37	0.38	0.39	0.4	0.4	0.41	0.42	0.43	0.43	0.44	0.44	0.44	0.45	0.45	0.46	0.46	0.47	0.47	0.48	0.48	0.49	0.49	0.5	0.51	0.51	0.52
Makueni	0.3	0.31	0.31	0.32	0.33	0.33	0.34	0.34	0.34	0.35	0.35	0.35	0.35	0.36	0.37	0.37	0.38	0.39	0.4	0.41	0.42	0.43	0.44	0.45	0.45	0.46	0.47	0.47
Mandera	0.1	0.11	0.12	0.13	0.14	0.14	0.15	0.16	0.16	0.17	0.18	0.18	0.19	0.19	0.2	0.21	0.21	0.22	0.22	0.23	0.24	0.24	0.25	0.26	0.27	0.28	0.29	0.3
Marsabit	0.22	0.22	0.23	0.23	0.24	0.24	0.24	0.24	0.25	0.25	0.26	0.26	0.26	0.26	0.27	0.27	0.28	0.28	0.29	0.29	0.3	0.3	0.31	0.32	0.32	0.33	0.34	0.34
Meru	0.34	0.35	0.35	0.36	0.37	0.38	0.38	0.39	0.39	0.4	0.4	0.41	0.41	0.42	0.42	0.43	0.43	0.44	0.45	0.45	0.46	0.47	0.48	0.48	0.49	0.5	0.5	0.51
Migori	0.23	0.24	0.25	0.26	0.26	0.27	0.27	0.27	0.28	0.29	0.29	0.29	0.29	0.3	0.3	0.31	0.32	0.33	0.33	0.34	0.36	0.36	0.37	0.38	0.4	0.4	0.41	0.42
Mombasa	0.4	0.41	0.41	0.42	0.43	0.44	0.44	0.45	0.46	0.46	0.47	0.47	0.47	0.48	0.48	0.48	0.49	0.49	0.5	0.5	0.51	0.52	0.53	0.53	0.54	0.55	0.56	0.57
Murang’a	0.38	0.39	0.4	0.4	0.41	0.41	0.42	0.42	0.43	0.44	0.44	0.44	0.45	0.45	0.46	0.47	0.47	0.48	0.48	0.49	0.49	0.5	0.5	0.51	0.52	0.52	0.52	0.53
Nairobi	0.5	0.51	0.51	0.52	0.53	0.53	0.53	0.54	0.54	0.55	0.55	0.56	0.56	0.57	0.57	0.58	0.59	0.59	0.6	0.61	0.62	0.63	0.63	0.64	0.65	0.66	0.67	0.67
Nakuru	0.34	0.35	0.36	0.37	0.38	0.38	0.39	0.39	0.4	0.41	0.41	0.41	0.42	0.42	0.43	0.44	0.45	0.45	0.46	0.47	0.48	0.49	0.5	0.51	0.52	0.53	0.54	0.55
Nandi	0.32	0.33	0.34	0.35	0.36	0.37	0.38	0.38	0.39	0.39	0.4	0.4	0.41	0.41	0.42	0.42	0.43	0.44	0.44	0.45	0.46	0.46	0.47	0.48	0.48	0.49	0.5	0.5
Narok	0.22	0.23	0.23	0.24	0.25	0.26	0.26	0.26	0.27	0.28	0.28	0.29	0.29	0.3	0.31	0.31	0.32	0.33	0.33	0.34	0.35	0.36	0.36	0.37	0.38	0.39	0.4	0.4
Nyamira	0.36	0.37	0.38	0.39	0.4	0.4	0.41	0.41	0.41	0.42	0.42	0.42	0.43	0.43	0.44	0.44	0.45	0.46	0.47	0.48	0.49	0.5	0.5	0.51	0.52	0.53	0.54	0.54
Nyandarua	0.35	0.36	0.37	0.38	0.38	0.39	0.39	0.4	0.4	0.41	0.41	0.42	0.42	0.43	0.44	0.44	0.45	0.46	0.47	0.48	0.48	0.49	0.5	0.51	0.51	0.52	0.53	0.53
Nyeri	0.41	0.41	0.42	0.43	0.43	0.44	0.44	0.45	0.45	0.46	0.46	0.47	0.47	0.48	0.48	0.48	0.49	0.5	0.5	0.51	0.52	0.52	0.53	0.53	0.54	0.54	0.55	0.55
Samburu	0.21	0.21	0.22	0.22	0.23	0.23	0.23	0.23	0.24	0.24	0.24	0.24	0.24	0.24	0.25	0.25	0.25	0.26	0.26	0.27	0.27	0.28	0.28	0.29	0.29	0.3	0.3	0.31
Siaya	0.23	0.24	0.25	0.26	0.27	0.27	0.28	0.29	0.29	0.3	0.31	0.31	0.32	0.33	0.33	0.34	0.35	0.36	0.37	0.38	0.39	0.4	0.41	0.42	0.43	0.44	0.45	0.46
Taita Taveta	0.35	0.36	0.37	0.38	0.39	0.4	0.4	0.41	0.42	0.42	0.43	0.43	0.43	0.44	0.44	0.45	0.45	0.46	0.46	0.47	0.48	0.49	0.49	0.5	0.51	0.52	0.52	0.53
Tana River	0.23	0.24	0.24	0.25	0.26	0.26	0.26	0.27	0.27	0.27	0.28	0.28	0.28	0.28	0.29	0.29	0.29	0.3	0.3	0.31	0.32	0.33	0.33	0.34	0.35	0.36	0.37	0.38
Tharaka Nithi	0.34	0.35	0.36	0.36	0.37	0.38	0.38	0.39	0.39	0.4	0.4	0.41	0.41	0.42	0.42	0.43	0.44	0.45	0.45	0.46	0.47	0.48	0.49	0.5	0.51	0.51	0.52	0.53
Trans Nzoia	0.32	0.33	0.34	0.35	0.35	0.36	0.36	0.37	0.37	0.38	0.38	0.39	0.39	0.4	0.41	0.41	0.42	0.43	0.43	0.44	0.45	0.45	0.46	0.47	0.48	0.48	0.49	0.5
Turkana	0.21	0.22	0.22	0.23	0.23	0.23	0.24	0.24	0.24	0.25	0.25	0.25	0.25	0.25	0.25	0.25	0.25	0.26	0.26	0.26	0.26	0.27	0.27	0.27	0.28	0.29	0.29	0.3
Uasin Gishu	0.36	0.37	0.38	0.39	0.4	0.41	0.41	0.42	0.42	0.43	0.43	0.44	0.44	0.45	0.45	0.46	0.47	0.47	0.48	0.49	0.5	0.5	0.51	0.52	0.53	0.53	0.54	0.55
Vihiga	0.33	0.34	0.35	0.36	0.36	0.37	0.37	0.37	0.38	0.38	0.38	0.38	0.38	0.38	0.39	0.39	0.4	0.41	0.42	0.43	0.44	0.44	0.45	0.46	0.46	0.47	0.47	0.48
Wajir	0.1	0.11	0.12	0.13	0.14	0.14	0.15	0.15	0.16	0.16	0.17	0.17	0.17	0.17	0.18	0.18	0.18	0.19	0.19	0.2	0.2	0.21	0.21	0.22	0.23	0.23	0.24	0.24
West Pokot	0.21	0.22	0.23	0.23	0.24	0.25	0.25	0.25	0.26	0.27	0.27	0.27	0.27	0.28	0.29	0.29	0.3	0.31	0.31	0.32	0.33	0.33	0.34	0.35	0.36	0.37	0.38	0.38
Madagascar	0.26	0.26	0.27	0.27	0.27	0.27	0.27	0.27	0.27	0.27	0.27	0.28	0.28	0.29	0.29	0.29	0.3	0.3	0.3	0.3	0.3	0.3	0.31	0.31	0.32	0.32	0.33	0.33
Malawi	0.2	0.2	0.2	0.21	0.21	0.21	0.22	0.22	0.23	0.23	0.23	0.24	0.24	0.25	0.26	0.26	0.27	0.28	0.29	0.3	0.31	0.31	0.32	0.33	0.33	0.34	0.35	0.35
Mozambique	0.13	0.14	0.14	0.14	0.14	0.14	0.15	0.15	0.16	0.17	0.18	0.18	0.19	0.2	0.21	0.22	0.22	0.24	0.25	0.26	0.27	0.28	0.29	0.3	0.31	0.32	0.33	0.34
Rwanda	0.27	0.27	0.27	0.27	0.27	0.27	0.27	0.27	0.28	0.28	0.28	0.29	0.3	0.3	0.31	0.32	0.33	0.33	0.34	0.35	0.36	0.36	0.37	0.38	0.39	0.39	0.4	0.41

eTable 1: Socio-Demographic Index values for all estimated GBD 2017 locations, 1990-2017

Location	1990	1991	1992	1993	1994	1995	1996	1997	1998	1999	2000	2001	2002	2003	2004	2005	2006	2007	2008	2009	2010	2011	2012	2013	2014	2015	2016	2017
Somalia	0.15	0.16	0.16	0.16	0.16	0.16	0.16	0.16	0.16	0.16	0.17	0.17	0.17	0.17	0.18	0.18	0.18	0.19	0.19	0.2	0.2	0.21	0.21	0.22	0.22	0.23	0.23	0.24
South Sudan	0.18	0.18	0.18	0.18	0.18	0.19	0.19	0.19	0.19	0.19	0.2	0.2	0.2	0.21	0.21	0.22	0.22	0.23	0.23	0.23	0.24	0.24	0.25	0.25	0.26	0.27	0.27	0.28
Tanzania	0.28	0.28	0.29	0.29	0.29	0.29	0.3	0.3	0.3	0.31	0.31	0.32	0.32	0.33	0.33	0.33	0.34	0.34	0.35	0.36	0.36	0.37	0.37	0.38	0.39	0.4	0.41	0.41
Uganda	0.16	0.16	0.17	0.17	0.17	0.18	0.19	0.19	0.2	0.21	0.22	0.23	0.24	0.25	0.27	0.28	0.29	0.3	0.31	0.32	0.33	0.34	0.35	0.36	0.37	0.37	0.38	0.39
Zambia	0.31	0.31	0.32	0.32	0.32	0.32	0.33	0.33	0.33	0.34	0.34	0.34	0.35	0.35	0.36	0.37	0.37	0.38	0.39	0.4	0.41	0.42	0.43	0.44	0.45	0.46	0.47	0.47
Southern sub-Saharan Africa	0.52	0.53	0.53	0.54	0.54	0.54	0.55	0.55	0.56	0.56	0.57	0.57	0.57	0.58	0.58	0.59	0.59	0.6	0.6	0.61	0.61	0.62	0.62	0.62	0.63	0.63	0.64	0.64
Botswana	0.46	0.47	0.48	0.49	0.5	0.51	0.52	0.52	0.53	0.54	0.55	0.55	0.56	0.57	0.57	0.58	0.59	0.6	0.61	0.61	0.62	0.63	0.63	0.64	0.65	0.65	0.66	0.66
Lesotho	0.33	0.34	0.35	0.35	0.36	0.37	0.38	0.38	0.39	0.4	0.4	0.41	0.41	0.42	0.42	0.43	0.43	0.44	0.44	0.45	0.45	0.46	0.46	0.47	0.48	0.48	0.49	0.49
Namibia	0.45	0.46	0.47	0.47	0.48	0.48	0.49	0.5	0.5	0.51	0.51	0.52	0.52	0.53	0.53	0.53	0.54	0.55	0.55	0.56	0.57	0.57	0.58	0.59	0.6	0.6	0.61	0.62
South Africa	0.56	0.56	0.57	0.57	0.57	0.58	0.58	0.58	0.59	0.59	0.6	0.6	0.61	0.61	0.62	0.62	0.62	0.63	0.64	0.64	0.65	0.65	0.66	0.66	0.66	0.67	0.67	0.68
Swaziland	0.43	0.44	0.44	0.45	0.46	0.47	0.47	0.48	0.48	0.49	0.49	0.5	0.5	0.51	0.51	0.52	0.52	0.53	0.53	0.54	0.54	0.55	0.55	0.56	0.56	0.57	0.57	0.58
Zimbabwe	0.41	0.42	0.42	0.43	0.44	0.44	0.45	0.45	0.45	0.46	0.46	0.46	0.46	0.46	0.45	0.45	0.44	0.44	0.43	0.43	0.42	0.43	0.43	0.44	0.44	0.45	0.46	0.46
Western sub-Saharan Africa	0.29	0.3	0.3	0.3	0.31	0.31	0.31	0.32	0.32	0.32	0.33	0.33	0.34	0.34	0.35	0.36	0.37	0.37	0.38	0.39	0.4	0.4	0.41	0.42	0.42	0.43	0.44	0.44
Benin	0.22	0.22	0.23	0.23	0.24	0.24	0.25	0.25	0.26	0.26	0.27	0.27	0.28	0.28	0.29	0.3	0.3	0.31	0.31	0.32	0.33	0.33	0.34	0.35	0.35	0.36	0.37	0.37
Burkina Faso	0.14	0.14	0.15	0.15	0.15	0.16	0.16	0.17	0.17	0.18	0.18	0.19	0.2	0.2	0.21	0.21	0.22	0.23	0.23	0.24	0.24	0.25	0.25	0.26	0.27	0.27	0.28	0.28
Cameroon	0.33	0.34	0.34	0.35	0.35	0.35	0.36	0.36	0.36	0.36	0.36	0.36	0.36	0.36	0.37	0.37	0.38	0.39	0.4	0.41	0.41	0.42	0.43	0.44	0.45	0.46	0.47	0.48
Cape Verde	0.31	0.32	0.32	0.33	0.34	0.35	0.36	0.37	0.38	0.39	0.4	0.41	0.42	0.43	0.44	0.45	0.46	0.47	0.48	0.49	0.5	0.51	0.52	0.52	0.53	0.54	0.54	0.55
Chad	0.12	0.12	0.13	0.13	0.14	0.14	0.14	0.14	0.15	0.15	0.15	0.15	0.15	0.16	0.16	0.17	0.17	0.18	0.19	0.19	0.2	0.21	0.22	0.22	0.23	0.24	0.25	0.25
Cote d'Ivoire	0.27	0.28	0.29	0.29	0.3	0.3	0.31	0.31	0.32	0.32	0.33	0.33	0.33	0.34	0.34	0.35	0.35	0.35	0.36	0.36	0.37	0.37	0.38	0.38	0.39	0.4	0.41	0.41
The Gambia	0.25	0.25	0.26	0.26	0.27	0.28	0.28	0.29	0.29	0.3	0.31	0.31	0.32	0.33	0.33	0.34	0.34	0.35	0.36	0.36	0.37	0.37	0.38	0.38	0.39	0.4	0.4	0.41
Ghana	0.38	0.39	0.39	0.4	0.4	0.4	0.41	0.41	0.42	0.42	0.42	0.43	0.43	0.44	0.44	0.44	0.45	0.46	0.47	0.47	0.48	0.49	0.5	0.51	0.51	0.52	0.53	0.54
Guinea	0.16	0.17	0.17	0.18	0.18	0.18	0.19	0.19	0.2	0.21	0.21	0.22	0.22	0.23	0.23	0.24	0.25	0.25	0.26	0.27	0.27	0.28	0.29	0.3	0.3	0.31	0.32	0.33
Guinea-Bissau	0.18	0.19	0.19	0.2	0.21	0.21	0.22	0.22	0.23	0.24	0.24	0.25	0.25	0.26	0.26	0.27	0.27	0.28	0.29	0.29	0.3	0.31	0.32	0.32	0.33	0.34	0.34	0.35
Liberia	0.21	0.21	0.21	0.21	0.2	0.19	0.18	0.17	0.18	0.18	0.2	0.21	0.23	0.23	0.24	0.25	0.25	0.26	0.26	0.27	0.28	0.28	0.29	0.3	0.31	0.32	0.32	0.33
Mali	0.13	0.13	0.14	0.14	0.15	0.15	0.15	0.16	0.16	0.17	0.17	0.18	0.18	0.19	0.19	0.2	0.2	0.21	0.22	0.22	0.23	0.23	0.24	0.24	0.25	0.26	0.26	0.27
Mauritania	0.3	0.31	0.31	0.32	0.33	0.34	0.34	0.35	0.35	0.36	0.36	0.36	0.37	0.37	0.38	0.38	0.39	0.4	0.41	0.41	0.42	0.43	0.44	0.44	0.45	0.46	0.47	0.47
Niger	0.09	0.1	0.1	0.11	0.11	0.11	0.11	0.12	0.12	0.12	0.12	0.13	0.13	0.13	0.13	0.14	0.14	0.14	0.15	0.15	0.16	0.16	0.17	0.17	0.18	0.18	0.19	0.19
Nigeria	0.34	0.35	0.35	0.35	0.35	0.36	0.36	0.36	0.37	0.37	0.37	0.38	0.39	0.39	0.4	0.41	0.42	0.43	0.44	0.45	0.45	0.46	0.47	0.47	0.48	0.48	0.49	0.49
Sao Tome and Principe	0.29	0.29	0.3	0.3	0.31	0.31	0.31	0.32	0.33	0.33	0.34	0.35	0.35	0.36	0.37	0.38	0.39	0.4	0.41	0.42	0.43	0.44	0.44	0.45	0.46	0.47	0.48	0.49
Senegal	0.24	0.25	0.26	0.26	0.27	0.27	0.28	0.28	0.29	0.29	0.3	0.3	0.3	0.31	0.31	0.32	0.32	0.32	0.33	0.33	0.34	0.34	0.35	0.35	0.36	0.36	0.37	0.37
Sierra Leone	0.2	0.21	0.21	0.21	0.22	0.22	0.22	0.23	0.23	0.23	0.23	0.23	0.24	0.24	0.25	0.26	0.26	0.27	0.28	0.29	0.29	0.3	0.31	0.33	0.34	0.34	0.35	0.36
Togo	0.26	0.27	0.28	0.28	0.29	0.29	0.3	0.3	0.31	0.31	0.32	0.32	0.32	0.33	0.33	0.33	0.34	0.34	0.35	0.35	0.36	0.37	0.37	0.38	0.39	0.4	0.41	0.41

eTable 2. ENN, LNN, PNN, 1-4, 5-9, 10-14, 15-19, <1, <5, 10-19, and <20 years mortality in 1990, 2000 and 2017 with mean percentage change for all GBD causes.

	Metric	Early Neonatal	Late Neonatal	Post Neonatal	1 to 4	5 to 9	10 to 14	15 to 19	<1 year	Under 5	<20 years	10 to 19
**Global**	Death #, 1990	3,037,044 (2,966,409 to 3,104,943)	1,263,098 (1,220,453 to 1,308,864)	3,854,632 (3,771,297 to 3,942,560)	3,615,809 (3,519,278 to 3,715,515)	853,941 (844,956 to 862,845)	466,727 (462,844 to 470,900)	674,033 (666,858 to 681,883)	8,154,774 (8,024,357 to 8,285,234)	11,770,583 (11,618,335 to 11,926,603)	13,765,285 (13,601,506 to 13,934,158)	1,140,760 (1,130,272 to 1,151,953)
	Death #, 2000	2,743,663 (2,683,053 to 2,807,354)	918,145 (887,698 to 954,872)	3,092,086 (3,030,247 to 3,163,815)	2,926,972 (2,853,818 to 3,007,898)	703,302 (696,132 to 711,149)	483,735 (479,703 to 487,837)	678,423 (670,973 to 686,282)	6,753,895 (6,641,564 to 6,883,034)	9,680,867 (9,545,127 to 9,822,519)	11,546,328 (11,402,470 to 11,696,143)	1,162,159 (1,151,054 to 1,173,659)
	Death #, 2017	1,859,529 (1,793,656 to 1,933,838)	507,698 (489,055 to 527,088)	1,621,135 (1,558,657 to 1,693,323)	1,403,200 (1,340,221 to 1,475,816)	412,113 (404,482 to 420,010)	319,630 (314,713 to 325,138)	516,509 (506,942 to 527,177)	3,988,362 (3,847,837 to 4,140,124)	5,391,562 (5,195,363 to 5,612,906)	6,639,815 (6,437,215 to 6,870,058)	836,139 (822,746 to 851,197)
	% change 1990-2017	**-38.8% (-41.5% to -35.9%)#**	**-59.8% (-61.8% to -57.6%)#**	**-57.9% (-59.9% to -55.8%)#**	**-61.2% (-63.3% to -59.0%)#**	**-51.7% (-52.8% to -50.7%)#**	**-31.5% (-32.6% to -30.3%)#**	**-23.4% (-24.9% to -21.7%)#**	**-51.1% (-53.1% to -49.1%)#**	**-54.2% (-56.0% to -52.3%)#**	**-51.8% (-53.4% to -50.0%)#**	**-26.7% (-28.0% to -25.3%)#**
	% change 2000-2017	**-32.2% (-35.1% to -29.1%)#**	**-44.7% (-47.5% to -41.7%)#**	**-47.6% (-49.9% to -45.0%)#**	**-52.1% (-54.5% to -49.4%)#**	**-41.4% (-42.7% to -40.1%)#**	**-33.9% (-35.0% to -32.9%)#**	**-23.9% (-25.2% to -22.5%)#**	**-41.0% (-43.2% to -38.3%)#**	**-44.3% (-46.4% to -41.9%)#**	**-42.5% (-44.4% to -40.3%)#**	**-28.1% (-29.2% to -26.9%)#**
**Low SDI**	Death #, 1990	1,048,756 (1,000,660 to 1,099,967)	499,125 (462,152 to 541,079)	1,407,561 (1,351,942 to 1,473,057)	1,649,664 (1,571,877 to 1,744,345)	294,379 (288,394 to 300,299)	134,914 (132,405 to 137,132)	173,480 (168,538 to 178,248)	2,955,442 (2,859,649 to 3,044,743)	4,605,107 (4,498,615 to 4,697,610)	5,207,880 (5,094,452 to 5,309,147)	308,395 (301,354 to 315,308)
	% of global total, 1990	34.5% (33.4% to 35.8%)	39.5% (37.6% to 41.4%)	36.5% (35.5% to 37.7%)	45.6% (44.3% to 47.2%)	34.5% (34.0% to 35.0%)	28.9% (28.5% to 29.3%)	25.7% (25.1% to 26.3%)	36.2% (35.4% to 37.0%)	39.1% (38.5% to 39.7%)	37.8% (37.2% to 38.4%)	27.0% (26.5% to 27.5%)
	Death #, 2000	1,047,243 (1,006,123 to 1,091,383)	377,909 (352,083 to 410,913)	1,312,429 (1,273,115 to 1,357,139)	1,433,329 (1,378,745 to 1,493,988)	269,317 (263,349 to 274,995)	152,533 (150,081 to 154,909)	192,073 (188,328 to 195,751)	2,737,581 (2,658,104 to 2,824,273)	4,170,910 (4,078,681 to 4,267,250)	4,784,833 (4,684,232 to 4,887,154)	344,605 (338,629 to 350,377)
	% of global total, 2000	38.2% (37.0% to 39.3%)	41.2% (39.3% to 43.4%)	42.4% (41.4% to 43.6%)	49.0% (47.7% to 50.2%)	38.3% (37.7% to 38.9%)	31.5% (31.1% to 31.9%)	28.3% (27.9% to 28.8%)	40.5% (39.6% to 41.4%)	43.1% (42.4% to 43.8%)	41.4% (40.8% to 42.1%)	29.6% (29.2% to 30.1%)
	Death #, 2017	799,558 (756,228 to 844,647)	216,363 (204,706 to 228,297)	764,529 (731,080 to 800,527)	697,767 (666,056 to 730,782)	170,474 (165,388 to 175,721)	116,484 (113,419 to 119,513)	164,477 (160,277 to 168,964)	1,780,450 (1,700,258 to 1,869,233)	2,478,217 (2,374,041 to 2,592,363)	2,929,653 (2,817,698 to 3,050,894)	280,962 (274,764 to 287,360)
	% of global total, 2017	43.0% (41.2% to 44.9%)	42.6% (40.9% to 44.5%)	47.2% (45.1% to 49.2%)	49.7% (47.4% to 52.1%)	41.4% (40.5% to 42.4%)	36.4% (35.7% to 37.2%)	31.8% (31.1% to 32.6%)	44.6% (42.8% to 46.4%)	46.0% (44.1% to 47.7%)	44.1% (42.5% to 45.6%)	33.6% (32.9% to 34.3%)
	% change 1990-2017	**-23.8% (-29.0% to -17.6%)#**	**-56.6% (-60.4% to -52.0%)#**	**-45.7% (-49.1% to -42.2%)#**	**-57.7% (-60.8% to -54.4%)#**	**-42.1% (-44.1% to -39.9%)#**	**-13.7% (-16.3% to -10.7%)#**	**-5.2% (-8.6% to -1.6%)#**	**-39.8% (-43.0% to -36.1%)#**	**-46.2% (-48.6% to -43.4%)#**	**-43.8% (-46.1% to -41.2%)#**	**-8.9% (-11.8% to -6.0%)#**
	% change 2000-2017	**-23.6% (-28.8% to -17.7%)#**	**-42.8% (-48.2% to -37.3%)#**	**-41.8% (-44.8% to -38.6%)#**	**-51.3% (-54.2% to -48.2%)#**	**-36.7% (-39.2% to -34.3%)#**	**-23.6% (-26.0% to -21.3%)#**	**-14.4% (-16.9% to -12.0%)#**	**-35.0% (-38.5% to -31.2%)#**	**-40.6% (-43.4% to -37.4%)#**	**-38.8% (-41.5% to -35.7%)#**	**-18.5% (-20.6% to -16.4%)#**
**Low-middle SDI**	Death #, 1990	1,050,591 (1,005,227 to 1,101,086)	420,295 (402,095 to 440,764)	1,231,685 (1,187,111 to 1,281,266)	1,241,777 (1,188,516 to 1,291,581)	252,941 (248,961 to 256,718)	135,510 (133,125 to 137,988)	191,154 (186,315 to 196,539)	2,702,571 (2,635,701 to 2,772,458)	3,944,348 (3,854,061 to 4,037,247)	4,523,953 (4,433,296 to 4,620,989)	326,664 (319,584 to 334,446)
	% of global total, 1990	34.6% (33.4% to 35.9%)	33.3% (31.9% to 34.8%)	31.9% (31.0% to 33.0%)	34.3% (33.0% to 35.6%)	29.6% (29.2% to 30.0%)	29.0% (28.6% to 29.5%)	28.4% (27.8% to 29.0%)	33.1% (32.5% to 33.9%)	33.5% (33.0% to 34.1%)	32.9% (32.4% to 33.5%)	28.6% (28.1% to 29.2%)
	Death #, 2000	1,027,894 (985,060 to 1,072,304)	337,892 (322,334 to 353,887)	1,103,094 (1,057,458 to 1,152,018)	1,111,181 (1,061,360 to 1,162,163)	226,250 (222,741 to 229,883)	145,940 (143,053 to 148,953)	207,612 (201,623 to 214,285)	2,468,880 (2,386,134 to 2,550,868)	3,580,061 (3,482,534 to 3,681,176)	4,159,863 (4,056,664 to 4,267,343)	353,552 (344,879 to 363,321)
	% of global total, 2000	37.5% (36.2% to 38.7%)	36.8% (35.0% to 38.4%)	35.7% (34.6% to 36.8%)	38.0% (36.6% to 39.3%)	32.2% (31.7% to 32.6%)	30.2% (29.7% to 30.6%)	30.6% (30.0% to 31.3%)	36.5% (35.6% to 37.5%)	37.0% (36.2% to 37.7%)	36.0% (35.4% to 36.7%)	30.4% (29.9% to 31.0%)
	Death #, 2017	741,928 (687,548 to 799,669)	201,579 (187,087 to 217,116)	598,744 (546,451 to 659,900)	556,133 (501,195 to 617,348)	143,064 (137,522 to 148,843)	109,895 (106,336 to 114,277)	171,571 (163,916 to 180,817)	1,542,251 (1,428,045 to 1,667,161)	2,098,384 (1,931,802 to 2,278,025)	2,522,915 (2,349,624 to 2,716,084)	281,467 (270,507 to 294,587)
	% of global total, 2017	39.9% (37.9% to 42.0%)	39.7% (37.7% to 41.8%)	36.9% (34.6% to 39.4%)	39.6% (36.9% to 42.2%)	34.7% (33.7% to 35.7%)	34.4% (33.6% to 35.3%)	33.2% (32.1% to 34.4%)	38.7% (36.7% to 40.7%)	38.9% (36.9% to 41.0%)	38.0% (36.2% to 39.8%)	33.7% (32.7% to 34.7%)
	% change 1990-2017	**-29.4% (-35.7% to -22.9%)#**	**-52.0% (-56.3% to -47.8%)#**	**-51.4% (-56.1% to -45.6%)#**	**-55.2% (-59.9% to -50.0%)#**	**-43.4% (-45.8% to -41.0%)#**	**-18.9% (-21.6% to -15.7%)#**	**-10.2% (-14.5% to -5.3%)#**	**-42.9% (-47.3% to -38.1%)#**	**-46.8% (-50.9% to -42.1%)#**	**-44.2% (-48.0% to -39.8%)#**	**-13.8% (-17.3% to -9.6%)#**
	% change 2000-2017	**-27.8% (-34.1% to -21.1%)#**	**-40.3% (-45.4% to -34.9%)#**	**-45.7% (-51.3% to -39.6%)#**	**-50.0% (-55.1% to -44.0%)#**	**-36.8% (-39.5% to -34.0%)#**	**-24.7% (-27.0% to -22.1%)#**	**-17.4% (-20.8% to -13.5%)#**	**-37.5% (-42.4% to -32.3%)#**	**-41.4% (-46.1% to -36.1%)#**	**-39.4% (-43.7% to -34.5%)#**	**-20.4% (-23.3% to -17.1%)#**
**Middle SDI**	Death #, 1990	628,643 (610,187 to 646,947)	238,799 (230,555 to 247,253)	868,238 (837,395 to 901,662)	537,299 (517,549 to 556,637)	202,488 (198,125 to 206,891)	121,109 (119,519 to 122,793)	173,289 (170,735 to 175,772)	1,735,679 (1,683,546 to 1,790,834)	2,272,978 (2,208,184 to 2,340,940)	2,769,865 (2,698,798 to 2,842,310)	294,398 (290,461 to 298,249)
	% of global total, 1990	20.7% (20.0% to 21.4%)	18.9% (18.1% to 19.8%)	22.5% (21.8% to 23.3%)	14.9% (14.2% to 15.4%)	23.7% (23.3% to 24.1%)	25.9% (25.6% to 26.3%)	25.7% (25.3% to 26.1%)	21.3% (20.7% to 21.9%)	19.3% (18.8% to 19.8%)	20.1% (19.6% to 20.6%)	25.8% (25.4% to 26.2%)
	Death #, 2000	462,225 (451,844 to 472,926)	139,884 (135,990 to 143,999)	493,464 (480,843 to 506,778)	278,288 (269,324 to 287,279)	141,272 (138,903 to 143,669)	117,620 (116,463 to 118,791)	157,626 (155,839 to 159,372)	1,095,573 (1,073,483 to 1,120,170)	1,373,862 (1,345,819 to 1,401,556)	1,790,380 (1,759,629 to 1,820,258)	275,246 (272,638 to 277,738)
	% of global total, 2000	16.9% (16.4% to 17.3%)	15.2% (14.6% to 15.9%)	16.0% (15.5% to 16.4%)	9.5% (9.1% to 9.9%)	20.1% (19.7% to 20.4%)	24.3% (24.0% to 24.6%)	23.2% (22.9% to 23.6%)	16.2% (15.8% to 16.6%)	14.2% (13.9% to 14.5%)	15.5% (15.2% to 15.8%)	23.7% (23.4% to 23.9%)
	Death #, 2017	222,226 (214,317 to 230,498)	60,514 (58,427 to 62,842)	183,100 (174,148 to 193,196)	104,767 (100,420 to 109,319)	68,791 (67,224 to 70,555)	64,108 (63,168 to 65,105)	116,098 (113,774 to 118,242)	465,841 (449,615 to 483,118)	570,608 (551,051 to 591,996)	819,604 (797,304 to 842,529)	180,206 (177,373 to 182,959)
	% of global total, 2017	11.9% (11.4% to 12.6%)	11.9% (11.3% to 12.5%)	11.3% (10.7% to 12.1%)	7.5% (7.0% to 7.9%)	16.7% (16.2% to 17.1%)	20.1% (19.6% to 20.5%)	22.5% (21.9% to 23.0%)	11.7% (11.2% to 12.2%)	10.6% (10.1% to 11.1%)	12.3% (11.9% to 12.8%)	21.6% (21.1% to 22.0%)
	% change 1990-2017	**-64.7% (-66.2% to -62.9%)#**	**-74.7% (-75.8% to -73.3%)#**	**-78.9% (-80.3% to -77.4%)#**	**-80.5% (-81.5% to -79.3%)#**	**-66.0% (-67.2% to -64.7%)#**	**-47.1% (-48.2% to -45.9%)#**	**-33.0% (-34.6% to -31.4%)#**	**-73.2% (-74.4% to -71.8%)#**	**-74.9% (-76.0% to -73.7%)#**	**-70.4% (-71.5% to -69.2%)#**	**-38.8% (-40.1% to -37.5%)#**
	% change 2000-2017	**-51.9% (-53.9% to -49.8%)#**	**-56.7% (-58.6% to -54.6%)#**	**-62.9% (-64.9% to -60.5%)#**	**-62.4% (-64.2% to -60.4%)#**	**-51.3% (-52.7% to -49.8%)#**	**-45.5% (-46.5% to -44.5%)#**	**-26.4% (-27.9% to -24.7%)#**	**-57.5% (-59.0% to -55.6%)#**	**-58.5% (-60.0% to -56.7%)#**	**-54.2% (-55.6% to -52.7%)#**	**-34.5% (-35.7% to -33.3%)#**
**High-middle SDI**	Death #, 1990	251,380 (243,625 to 259,229)	88,124 (85,426 to 91,122)	294,385 (285,064 to 304,510)	156,447 (149,526 to 163,923)	85,156 (83,615 to 86,820)	57,768 (57,072 to 58,454)	86,847 (85,614 to 88,149)	633,890 (616,360 to 653,955)	790,337 (768,924 to 813,197)	1,020,107 (996,550 to 1,045,005)	144,615 (142,872 to 146,484)
	% of global total, 1990	8.3% (8.0% to 8.6%)	7.0% (6.7% to 7.3%)	7.6% (7.3% to 7.9%)	4.3% (4.1% to 4.5%)	10.0% (9.8% to 10.2%)	12.4% (12.2% to 12.5%)	12.9% (12.7% to 13.1%)	7.8% (7.5% to 8.1%)	6.7% (6.5% to 6.9%)	7.4% (7.2% to 7.6%)	12.7% (12.5% to 12.9%)
	Death #, 2000	168,748 (163,067 to 174,696)	50,722 (48,938 to 52,476)	152,970 (147,699 to 158,189)	85,695 (81,187 to 90,292)	54,832 (53,762 to 55,920)	54,631 (53,990 to 55,338)	84,218 (83,489 to 85,016)	372,439 (361,974 to 383,119)	458,135 (445,459 to 471,978)	651,816 (637,814 to 666,613)	138,849 (137,574 to 140,224)
	% of global total, 2000	6.2% (5.9% to 6.4%)	5.5% (5.2% to 5.8%)	5.0% (4.8% to 5.1%)	2.9% (2.8% to 3.1%)	7.8% (7.6% to 8.0%)	11.3% (11.1% to 11.4%)	12.4% (12.2% to 12.6%)	5.5% (5.3% to 5.7%)	4.7% (4.6% to 4.9%)	5.7% (5.5% to 5.8%)	11.9% (11.8% to 12.1%)
	Death #, 2017	69,837 (66,696 to 73,005)	21,430 (20,560 to 22,308)	56,477 (53,589 to 59,480)	33,866 (32,037 to 35,979)	23,303 (22,679 to 23,920)	21,805 (21,438 to 22,160)	42,409 (41,619 to 43,208)	147,743 (141,663 to 153,908)	181,609 (174,033 to 188,764)	269,126 (260,627 to 277,283)	64,214 (63,108 to 65,265)
	% of global total, 2017	3.8% (3.5% to 4.0%)	4.2% (4.0% to 4.5%)	3.5% (3.3% to 3.7%)	2.4% (2.2% to 2.6%)	5.7% (5.5% to 5.8%)	6.8% (6.7% to 7.0%)	8.2% (8.0% to 8.4%)	3.7% (3.5% to 3.9%)	3.4% (3.2% to 3.5%)	4.0% (3.9% to 4.2%)	7.7% (7.5% to 7.8%)
	% change 1990-2017	**-72.2% (-73.7% to -70.7%)#**	**-75.7% (-76.9% to -74.4%)#**	**-80.8% (-82.0% to -79.6%)#**	**-78.3% (-79.8% to -76.8%)#**	**-72.6% (-73.4% to -71.9%)#**	**-62.2% (-63.0% to -61.5%)#**	**-51.2% (-52.3% to -50.0%)#**	**-76.7% (-77.8% to -75.5%)#**	**-77.0% (-78.1% to -76.0%)#**	**-73.6% (-74.6% to -72.7%)#**	**-55.6% (-56.5% to -54.7%)#**
	% change 2000-2017	**-58.6% (-60.7% to -56.2%)#**	**-57.8% (-59.9% to -55.5%)#**	**-63.1% (-65.3% to -60.6%)#**	**-60.5% (-63.4% to -57.4%)#**	**-57.5% (-58.8% to -56.2%)#**	**-60.1% (-60.9% to -59.3%)#**	**-49.6% (-50.8% to -48.6%)#**	**-60.3% (-62.2% to -58.4%)#**	**-60.4% (-62.2% to -58.5%)#**	**-58.7% (-60.2% to -57.2%)#**	**-53.8% (-54.7% to -52.8%)#**
**High SDI**	Death #, 1990	51,138 (50,076 to 52,156)	14,066 (13,636 to 14,520)	40,672 (39,568 to 41,787)	23,862 (23,087 to 24,712)	16,274 (15,946 to 16,603)	15,870 (15,745 to 15,997)	47,148 (47,037 to 47,267)	105,875 (104,730 to 107,028)	129,737 (128,508 to 131,047)	209,029 (207,361 to 210,885)	63,018 (62,791 to 63,243)
	% of global total, 1990	1.7% (1.6% to 1.7%)	1.1% (1.1% to 1.2%)	1.1% (1.0% to 1.1%)	0.7% (0.6% to 0.7%)	1.9% (1.9% to 1.9%)	3.4% (3.4% to 3.4%)	7.0% (6.9% to 7.1%)	1.3% (1.3% to 1.3%)	1.1% (1.1% to 1.1%)	1.5% (1.5% to 1.5%)	5.5% (5.5% to 5.6%)
	Death #, 2000	33,111 (32,535 to 33,688)	10,224 (9,938 to 10,536)	23,583 (22,957 to 24,177)	14,817 (14,389 to 15,295)	9,693 (9,641 to 9,746)	11,387 (11,347 to 11,430)	35,359 (35,294 to 35,431)	66,918 (66,183 to 67,627)	81,735 (81,099 to 82,476)	138,175 (137,405 to 138,996)	46,747 (46,647 to 46,849)
	% of global total, 2000	1.2% (1.2% to 1.2%)	1.1% (1.1% to 1.2%)	0.8% (0.7% to 0.8%)	0.5% (0.5% to 0.5%)	1.4% (1.4% to 1.4%)	2.4% (2.3% to 2.4%)	5.2% (5.2% to 5.3%)	1.0% (1.0% to 1.0%)	0.8% (0.8% to 0.9%)	1.2% (1.2% to 1.2%)	4.0% (4.0% to 4.1%)
	Death #, 2017	24,050 (23,335 to 24,802)	7,181 (6,937 to 7,450)	16,108 (15,624 to 16,662)	9,390 (9,153 to 9,642)	5,757 (5,681 to 5,836)	6,702 (6,640 to 6,765)	21,083 (20,783 to 21,392)	47,340 (45,953 to 48,790)	56,729 (55,145 to 58,397)	90,272 (88,442 to 92,159)	27,786 (27,447 to 28,137)
	% of global total, 2017	1.3% (1.2% to 1.4%)	1.4% (1.3% to 1.5%)	1.0% (0.9% to 1.1%)	0.7% (0.6% to 0.7%)	1.4% (1.4% to 1.4%)	2.1% (2.1% to 2.1%)	4.1% (4.0% to 4.2%)	1.2% (1.1% to 1.2%)	1.1% (1.0% to 1.1%)	1.4% (1.3% to 1.4%)	3.3% (3.2% to 3.4%)
	% change 1990-2017	**-53.0% (-54.6% to -51.1%)#**	**-48.9% (-51.3% to -46.3%)#**	**-60.4% (-62.1% to -58.8%)#**	**-60.6% (-62.1% to -58.9%)#**	**-64.6% (-65.4% to -63.8%)#**	**-57.8% (-58.3% to -57.3%)#**	**-55.3% (-55.9% to -54.6%)#**	**-55.3% (-56.6% to -53.9%)#**	**-56.3% (-57.5% to -54.9%)#**	**-56.8% (-57.7% to -55.9%)#**	**-55.9% (-56.5% to -55.3%)#**

eTable 2. ENN, LNN, PNN, 1-4, 5-9, 10-14, 15-19, <1, <5, 10-19, and <20 years mortality in 1990, 2000 and 2017 with mean percentage change for all GBD causes.

	Metric	Early Neonatal	Late Neonatal	Post Neonatal	1 to 4	5 to 9	10 to 14	15 to 19	<1 year	Under 5	<20 years	10 to 19
	% change 2000-2017	-27.4% (-29.9% to -24.9%)#	-29.8% (-33.1% to -26.3%)#	-31.7% (-34.3% to -28.8%)#	-36.6% (-39.1% to -34.0%)#	-40.6% (-41.5% to -39.8%)#	-41.1% (-41.8% to -40.5%)#	-40.4% (-41.2% to -39.5%)#	-29.3% (-31.5% to -27.0%)#	-30.6% (-32.6% to -28.5%)#	-34.7% (-36.0% to -33.3%)#	-40.6% (-41.3% to -39.8%)#
Central Europe, Eastern Europe, and Central Asia	Death #, 1990	73,320 (70,988 to 76,027)	22,842 (21,899 to 23,766)	68,305 (65,811 to 70,674)	48,601 (46,244 to 51,144)	18,852 (18,564 to 19,150)	14,373 (14,260 to 14,490)	27,884 (27,808 to 27,961)	164,467 (160,702 to 168,521)	213,068 (208,102 to 218,152)	274,177 (268,776 to 279,621)	42,257 (42,077 to 42,444)
	% of global total, 1990	2.4% (2.3% to 2.5%)	1.8% (1.7% to 1.9%)	1.8% (1.7% to 1.8%)	1.3% (1.3% to 1.4%)	2.2% (2.2% to 2.2%)	3.1% (3.0% to 3.1%)	4.1% (4.1% to 4.2%)	2.0% (2.0% to 2.1%)	1.8% (1.8% to 1.9%)	2.0% (1.9% to 2.0%)	3.7% (3.7% to 3.7%)
	Death #, 2000	44,106 (42,514 to 45,682)	14,478 (13,823 to 15,186)	40,174 (38,550 to 41,775)	23,548 (22,314 to 24,859)	13,552 (13,299 to 13,814)	14,810 (14,695 to 14,929)	34,676 (34,578 to 34,777)	98,759 (95,336 to 101,977)	122,307 (118,279 to 126,282)	185,345 (180,920 to 189,738)	49,487 (49,287 to 49,689)
	% of global total, 2000	1.6% (1.5% to 1.7%)	1.6% (1.5% to 1.7%)	1.3% (1.2% to 1.4%)	0.8% (0.8% to 0.9%)	1.9% (1.9% to 2.0%)	3.1% (3.0% to 3.1%)	5.1% (5.0% to 5.2%)	1.5% (1.4% to 1.5%)	1.3% (1.2% to 1.3%)	1.6% (1.6% to 1.6%)	4.3% (4.2% to 4.3%)
	Death #, 2017	27,240 (25,556 to 29,234)	9,246 (8,595 to 10,039)	20,751 (19,368 to 22,432)	12,737 (11,939 to 13,688)	7,006 (6,766 to 7,284)	6,472 (6,349 to 6,612)	13,553 (13,327 to 13,797)	57,237 (53,584 to 61,476)	69,973 (65,596 to 75,050)	97,005 (92,307 to 102,471)	20,025 (19,710 to 20,372)
	% of global total, 2017	1.5% (1.4% to 1.6%)	1.8% (1.7% to 2.0%)	1.3% (1.2% to 1.4%)	0.9% (0.8% to 1.0%)	1.7% (1.6% to 1.8%)	2.0% (2.0% to 2.1%)	2.6% (2.6% to 2.7%)	1.4% (1.3% to 1.6%)	1.3% (1.2% to 1.4%)	1.5% (1.4% to 1.6%)	2.4% (2.3% to 2.5%)
	% change 1990-2017	-62.9% (-65.5% to -59.7%)#	-59.5% (-62.9% to -55.7%)#	-69.6% (-72.0% to -66.9%)#	-73.8% (-75.8% to -71.5%)#	-62.8% (-64.2% to -61.2%)#	-55.0% (-55.9% to -53.9%)#	-51.4% (-52.2% to -50.5%)#	-65.2% (-67.6% to -62.4%)#	-67.2% (-69.4% to -64.7%)#	-64.6% (-66.5% to -62.5%)#	-52.6% (-53.4% to -51.7%)#
	% change 2000-2017	-38.2% (-42.7% to -33.4%)#	-36.1% (-41.4% to -30.6%)#	-48.4% (-52.2% to -43.8%)#	-45.9% (-50.1% to -41.0%)#	-48.3% (-50.1% to -46.1%)#	-56.3% (-57.1% to -55.3%)#	-60.9% (-61.5% to -60.2%)#	-42.0% (-46.0% to -37.3%)#	-42.8% (-46.6% to -38.4%)#	-47.7% (-50.4% to -44.6%)#	-59.5% (-60.2% to -58.8%)#
Central Asia	Death #, 1990	33,981 (31,831 to 36,270)	10,759 (10,082 to 11,455)	39,803 (37,413 to 42,143)	28,368 (26,122 to 30,688)	5,928 (5,742 to 6,126)	3,828 (3,759 to 3,900)	5,430 (5,388 to 5,471)	84,543 (81,146 to 88,042)	112,911 (108,542 to 117,665)	128,097 (123,446 to 133,135)	9,258 (9,150 to 9,361)
	% of global total, 1990	1.1% (1.1% to 1.2%)	0.8% (0.8% to 0.9%)	1.0% (1.0% to 1.1%)	0.8% (0.7% to 0.8%)	0.7% (0.7% to 0.7%)	0.8% (0.8% to 0.8%)	0.8% (0.8% to 0.8%)	1.0% (1.0% to 1.1%)	1.0% (0.9% to 1.0%)	0.9% (0.9% to 1.0%)	0.8% (0.8% to 0.8%)
	Death #, 2000	23,081 (21,548 to 24,597)	7,851 (7,246 to 8,536)	25,837 (24,269 to 27,418)	14,223 (13,046 to 15,470)	6,032 (5,781 to 6,281)	4,728 (4,615 to 4,840)	6,412 (6,323 to 6,505)	56,769 (53,492 to 59,968)	70,992 (67,035 to 74,911)	88,165 (83,812 to 92,378)	11,140 (10,945 to 11,329)
	% of global total, 2000	0.8% (0.8% to 0.9%)	0.9% (0.8% to 0.9%)	0.8% (0.8% to 0.9%)	0.5% (0.4% to 0.5%)	0.9% (0.8% to 0.9%)	1.0% (0.9% to 1.0%)	0.9% (0.9% to 1.0%)	0.8% (0.8% to 0.9%)	0.7% (0.7% to 0.8%)	0.8% (0.7% to 0.8%)	1.0% (0.9% to 1.0%)
	Death #, 2017	17,873 (16,199 to 19,838)	5,966 (5,350 to 6,701)	14,331 (12,948 to 15,974)	8,013 (7,227 to 8,961)	3,714 (3,480 to 3,994)	2,823 (2,704 to 2,954)	4,614 (4,404 to 4,855)	38,171 (34,528 to 42,400)	46,184 (41,881 to 51,176)	57,335 (52,672 to 62,768)	7,437 (7,138 to 7,773)
	% of global total, 2017	1.0% (0.9% to 1.1%)	1.2% (1.1% to 1.3%)	0.9% (0.8% to 1.0%)	0.6% (0.5% to 0.6%)	0.9% (0.8% to 1.0%)	0.9% (0.8% to 0.9%)	0.9% (0.8% to 0.9%)	1.0% (0.9% to 1.1%)	0.9% (0.8% to 0.9%)	0.9% (0.8% to 0.9%)	0.9% (0.8% to 0.9%)
	% change 1990-2017	-47.4% (-53.4% to -40.3%)#	-44.5% (-51.1% to -37.2%)#	-64.0% (-67.9% to -59.3%)#	-71.8% (-75.1% to -68.0%)#	-37.4% (-41.8% to -32.1%)#	-26.2% (-29.7% to -22.6%)#	-15.0% (-19.0% to -10.6%)#	-54.9% (-59.5% to -49.5%)#	-59.1% (-63.2% to -54.5%)#	-55.2% (-59.2% to -50.7%)#	-19.7% (-23.0% to -15.8%)#
	% change 2000-2017	-22.6% (-31.2% to -12.2%)#	-24.0% (-33.2% to -14.0%)#	-44.5% (-50.7% to -37.4%)#	-43.7% (-50.6% to -35.6%)#	-38.4% (-42.7% to -33.4%)#	-40.3% (-43.0% to -37.1%)#	-28.0% (-31.4% to -24.3%)#	-32.8% (-40.0% to -24.4%)#	-34.9% (-41.7% to -27.3%)#	-35.0% (-40.9% to -28.2%)#	-33.2% (-36.1% to -30.2%)#
Armenia	Death #, 1990	1,229 (934 to 1,580)	381 (351 to 413)	1,390 (1,239 to 1,561)	374 (275 to 485)	185 (172 to 200)	112 (107 to 117)	164 (159 to 169)	3,001 (2,635 to 3,390)	3,375 (3,036 to 3,754)	3,836 (3,476 to 4,239)	276 (267 to 285)
	Death #, 2000	479 (380 to 575)	116 (99 to 135)	422 (361 to 485)	132 (92 to 183)	77 (70 to 84)	79 (75 to 83)	141 (139 to 144)	1,018 (921 to 1,121)	1,150 (1,022 to 1,281)	1,447 (1,307 to 1,591)	220 (215 to 226)
	Death #, 2017	160 (143 to 178)	48 (42 to 55)	101 (89 to 114)	59 (43 to 78)	45 (42 to 49)	34 (33 to 36)	67 (65 to 69)	308 (277 to 342)	367 (331 to 408)	514 (472 to 560)	101 (98 to 105)
	% change 1990-2017	-87.0% (-90.1% to -82.6%)#	-87.5% (-89.4% to -85.2%)#	-92.8% (-94.0% to -91.2%)#	-84.3% (-90.8% to -72.7%)#	-75.5% (-78.0% to -73.0%)#	-69.1% (-70.9% to -67.2%)#	-59.3% (-61.2% to -57.4%)#	-89.7% (-91.4% to -87.8%)#	-89.1% (-90.6% to -87.4%)#	-86.6% (-88.2% to -84.8%)#	-63.3% (-64.9% to -61.5%)#
	% change 2000-2017	-66.6% (-73.0% to -56.9%)#	-59.0% (-66.3% to -49.2%)#	-76.2% (-80.0% to -71.2%)#	-55.5% (-71.7% to -26.7%)#	-41.1% (-47.6% to -33.8%)#	-56.4% (-59.2% to -53.4%)#	-52.7% (-54.6% to -50.9%)#	-69.7% (-73.7% to -65.2%)#	-68.1% (-72.6% to -62.8%)#	-64.5% (-68.8% to -59.6%)#	-54.0% (-56.1% to -52.0%)#
Azerbaijan	Death #, 1990	4,200 (3,543 to 4,951)	1,691 (1,439 to 1,989)	6,250 (5,310 to 7,289)	2,600 (2,203 to 3,018)	701 (647 to 761)	351 (335 to 368)	578 (554 to 602)	12,141 (10,939 to 13,639)	14,740 (13,236 to 16,563)	16,370 (14,784 to 18,261)	928 (892 to 965)
	Death #, 2000	3,056 (2,567 to 3,589)	1,016 (835 to 1,217)	3,216 (2,767 to 3,737)	1,337 (1,048 to 1,694)	1,092 (1,005 to 1,185)	515 (492 to 540)	560 (539 to 585)	7,288 (6,418 to 8,285)	8,625 (7,621 to 9,755)	10,793 (9,683 to 12,019)	1,076 (1,035 to 1,120)
	Death #, 2017	2,668 (2,027 to 3,477)	742 (570 to 959)	1,824 (1,409 to 2,339)	749 (591 to 939)	487 (430 to 551)	265 (244 to 286)	429 (398 to 462)	5,233 (4,008 to 6,769)	5,982 (4,599 to 7,713)	7,163 (5,692 to 8,980)	694 (643 to 743)
	% change 1990-2017	-36.5% (-54.5% to -11.9%)#	-56.1% (-67.6% to -40.4%)#	-70.8% (-78.5% to -60.4%)#	-71.2% (-78.3% to -61.9%)#	-30.6% (-40.4% to -19.6%)#	-24.5% (-31.1% to -17.6%)#	-25.8% (-31.8% to -18.8%)#	-56.9% (-68.1% to -43.0%)#	-59.4% (-69.8% to -46.9%)#	-56.2% (-66.4% to -44.4%)#	-25.3% (-31.2% to -19.0%)#
	% change 2000-2017	-12.7% (-38.5% to 18.4%)	-27.0% (-47.1% to -0.9%)#	-43.3% (-57.5% to -23.4%)#	-44.0% (-59.1% to -22.5%)#	-55.5% (-61.6% to -48.1%)#	-48.6% (-53.3% to -43.9%)#	-23.5% (-29.8% to -17.1%)#	-28.2% (-47.2% to -4.6%)#	-30.6% (-48.3% to -8.2%)#	-33.6% (-48.3% to -14.8%)#	-35.5% (-40.8% to -30.5%)#
Georgia	Death #, 1990	1,623 (1,390 to 1,874)	254 (208 to 310)	1,329 (1,113 to 1,585)	406 (293 to 534)	250 (231 to 270)	178 (169 to 186)	278 (266 to 291)	3,207 (2,855 to 3,617)	3,612 (3,205 to 4,084)	4,318 (3,895 to 4,816)	456 (437 to 475)
	Death #, 2000	927 (784 to 1,097)	190 (155 to 228)	575 (490 to 680)	208 (165 to 257)	106 (98 to 115)	92 (88 to 96)	165 (159 to 172)	1,691 (1,481 to 1,950)	1,900 (1,667 to 2,169)	2,263 (2,017 to 2,549)	257 (247 to 267)
	Death #, 2017	220 (177 to 276)	98 (78 to 123)	150 (120 to 188)	82 (58 to 116)	57 (50 to 66)	55 (51 to 60)	119 (114 to 123)	467 (375 to 587)	549 (441 to 689)	780 (658 to 935)	174 (165 to 182)
	% change 1990-2017	-86.5% (-90.0% to -81.5%)#	-61.5% (-70.5% to -51.0%)#	-88.7% (-91.3% to -85.4%)#	-79.8% (-87.3% to -68.4%)#	-77.0% (-80.6% to -72.8%)#	-68.9% (-71.9% to -65.9%)#	-57.3% (-59.8% to -54.9%)#	-85.4% (-88.8% to -81.2%)#	-84.8% (-88.0% to -80.6%)#	-81.9% (-85.1% to -78.0%)#	-61.9% (-64.2% to -59.4%)#
	% change 2000-2017	-76.3% (-82.4% to -68.7%)#	-48.4% (-60.2% to -31.2%)#	-74.0% (-79.5% to -66.1%)#	-60.7% (-74.8% to -38.0%)#	-45.7% (-54.1% to -36.6%)#	-40.0% (-45.4% to -34.2%)#	-28.1% (-31.9% to -24.2%)#	-72.4% (-78.6% to -64.5%)#	-71.1% (-77.4% to -63.1%)#	-65.5% (-71.5% to -58.0%)#	-32.4% (-36.3% to -28.2%)#
Kazakhstan	Death #, 1990	4,905 (4,321 to 5,497)	1,617 (1,363 to 1,923)	4,673 (4,085 to 5,380)	3,424 (2,715 to 4,219)	1,162 (1,081 to 1,252)	868 (834 to 903)	1,421 (1,404 to 1,439)	11,196 (10,275 to 12,209)	14,620 (13,347 to 16,031)	18,071 (16,661 to 19,629)	2,289 (2,239 to 2,343)
	Death #, 2000	2,987 (2,623 to 3,407)	880 (757 to 1,011)	2,473 (2,173 to 2,807)	1,529 (1,282 to 1,808)	889 (810 to 979)	856 (815 to 902)	1,562 (1,540 to 1,587)	6,341 (5,623 to 7,128)	7,869 (7,034 to 8,823)	11,176 (10,213 to 12,290)	2,418 (2,356 to 2,488)
	Death #, 2017	1,702 (1,203 to 2,320)	764 (595 to 987)	1,604 (1,212 to 2,085)	1,087 (754 to 1,520)	528 (456 to 619)	411 (380 to 448)	720 (687 to 759)	4,070 (3,195 to 5,225)	5,157 (4,108 to 6,558)	6,816 (5,656 to 8,363)	1,132 (1,073 to 1,197)
	% change 1990-2017	-65.3% (-76.0% to -51.1%)#	-52.7% (-64.9% to -37.2%)#	-65.7% (-74.9% to -52.9%)#	-68.2% (-79.4% to -52.7%)#	-54.6% (-61.5% to -46.3%)#	-52.6% (-56.7% to -48.0%)#	-49.3% (-51.8% to -46.6%)#	-63.6% (-72.0% to -52.9%)#	-64.7% (-72.3% to -54.6%)#	-62.3% (-69.2% to -53.2%)#	-50.6% (-53.3% to -47.5%)#
	% change 2000-2017	-43.0% (-60.1% to -19.6%)#	-13.2% (-33.0% to 13.0%)	-35.2% (-52.6% to -12.4%)#	-28.9% (-50.7% to 1.4%)	-40.6% (-49.2% to -29.8%)#	-52.0% (-55.9% to -47.2%)#	-53.9% (-56.0% to -51.4%)#	-35.8% (-50.2% to -16.3%)#	-34.5% (-47.6% to -15.7%)#	-39.0% (-49.2% to -24.6%)#	-53.2% (-55.7% to -50.1%)#
Kyrgyzstan	Death #, 1990	3,071 (2,598 to 3,592)	887 (714 to 1,076)	2,487 (2,019 to 3,037)	1,709 (1,324 to 2,172)	428 (400 to 459)	290 (278 to 303)	403 (385 to 421)	6,445 (5,731 to 7,255)	8,154 (7,455 to 8,960)	9,275 (8,527 to 10,131)	693 (665 to 721)
	Death #, 2000	1,735 (1,426 to 2,114)	535 (420 to 688)	1,964 (1,655 to 2,275)	849 (644 to 1,070)	363 (335 to 394)	308 (294 to 324)	418 (401 to 438)	4,234 (3,655 to 4,870)	5,083 (4,559 to 5,665)	6,172 (5,610 to 6,810)	726 (699 to 760)
	Death #, 2017	1,837 (1,660 to 2,028)	264 (210 to 322)	654 (593 to 718)	495 (361 to 675)	193 (183 to 204)	198 (192 to 204)	278 (271 to 285)	2,754 (2,482 to 3,048)	3,250 (2,968 to 3,565)	3,918 (3,626 to 4,249)	476 (464 to 487)
	% change 1990-2017	-40.2% (-50.4% to -26.9%)#	-70.3% (-78.7% to -59.3%)#	-73.7% (-79.2% to -66.8%)#	-71.0% (-80.9% to -55.8%)#	-55.0% (-58.9% to -50.6%)#	-31.7% (-35.1% to -28.1%)#	-31.0% (-34.5% to -27.4%)#	-57.3% (-64.1% to -49.5%)#	-60.1% (-65.0% to -54.0%)#	-57.8% (-62.5% to -52.1%)#	-31.3% (-34.5% to -27.9%)#
	% change 2000-2017	5.9% (-16.1% to 33.1%)	-50.7% (-66.4% to -28.7%)#	-66.7% (-72.5% to -59.7%)#	-41.6% (-63.6% to -7.6%)#	-46.9% (-52.0% to -41.6%)#	-35.6% (-39.4% to -32.0%)#	-33.6% (-37.1% to -30.2%)#	-35.0% (-46.4% to -21.2%)#	-36.1% (-44.8% to -26.3%)#	-36.5% (-44.4% to -28.1%)#	-34.5% (-37.8% to -31.3%)#
	Death #, 1990	1,635 (1,308 to 2,005)	395 (329 to 465)	2,552 (2,147 to 2,948)	2,121 (1,742 to 2,570)	264 (247 to 282)	152 (146 to 158)	245 (236 to 255)	4,582 (3,955 to 5,218)	6,703 (6,150 to 7,346)	7,364 (6,785 to 8,034)	397 (382 to 413)
	Death #, 2000	1,089 (969 to 1,233)	240 (209 to 274)	1,465 (1,317 to 1,619)	779 (621 to 948)	189 (177 to 203)	188 (181 to 196)	232 (224 to 239)	2,794 (2,539 to 3,075)	3,573 (3,260 to 3,897)	4,182 (3,846 to 4,529)	420 (406 to 433)

eTable 2. ENN, LNN, PNN, 1-4, 5-9, 10-14, 15-19, <1, <5, 10-19, and <20 years mortality in 1990, 2000 and 2017 with mean percentage change for all GBD causes.

	Metric	Early Neonatal	Late Neonatal	Post Neonatal	1 to 4	5 to 9	10 to 14	15 to 19	<1 year	Under 5	<20 years	10 to 19
Mongolia	Death #, 2017	909 (743 to 1,083)	210 (168 to 261)	599 (494 to 727)	327 (191 to 537)	143 (125 to 165)	112 (102 to 122)	161 (147 to 175)	1,719 (1,463 to 2,003)	2,046 (1,685 to 2,476)	2,461 (2,067 to 2,919)	272 (250 to 296)
	% change 1990-2017	-44.4% (-60.2% to -21.8%)#	-46.8% (-61.9% to -27.3%)#	-76.5% (-82.3% to -68.7%)#	-84.6% (-91.3% to -74.0%)#	-45.9% (-53.6% to -36.7%)#	-26.4% (-33.1% to -18.8%)#	-34.4% (-40.7% to -28.0%)#	-62.5% (-69.3% to -53.2%)#	-69.5% (-75.1% to -62.5%)#	-66.6% (-72.2% to -59.5%)#	-31.4% (-37.5% to -25.0%)#
	% change 2000-2017	-16.5% (-37.6% to 6.8%)	-12.5% (-34.0% to 14.5%)	-59.1% (-67.2% to -47.9%)#	-58.0% (-76.2% to -30.7%)#	-24.7% (-35.5% to -12.0%)#	-40.7% (-46.1% to -34.7%)#	-30.6% (-37.0% to -24.0%)#	-38.5% (-48.8% to -25.6%)#	-42.8% (-54.0% to -29.6%)#	-41.2% (-51.5% to -29.3%)#	-35.1% (-40.8% to -29.3%)#
Tajikistan	Death #, 1990	3,998 (2,901 to 5,321)	1,249 (1,016 to 1,497)	6,203 (4,976 to 7,337)	5,508 (4,724 to 6,394)	655 (609 to 705)	347 (334 to 363)	418 (408 to 429)	11,450 (10,202 to 12,787)	16,958 (15,413 to 18,681)	18,378 (16,768 to 20,176)	766 (744 to 791)
	Death #, 2000	3,351 (2,851 to 3,953)	975 (826 to 1,144)	4,914 (4,287 to 5,606)	2,969 (2,419 to 3,572)	711 (658 to 764)	482 (462 to 504)	559 (537 to 580)	9,241 (8,194 to 10,470)	12,210 (10,948 to 13,597)	13,961 (12,632 to 15,429)	1,041 (1,002 to 1,082)
	Death #, 2017	4,041 (3,277 to 5,037)	1,092 (882 to 1,365)	4,140 (3,350 to 5,140)	2,070 (1,687 to 2,545)	630 (553 to 725)	431 (393 to 468)	666 (636 to 706)	9,273 (7,516 to 11,535)	11,343 (9,181 to 14,078)	13,070 (10,812 to 15,912)	1,098 (1,037 to 1,165)
	% change 1990-2017	1.1% (-29.1% to 50.0%)	-12.6% (-33.5% to 16.6%)	-33.3% (-49.3% to -11.7%)#	-62.4% (-70.3% to -51.9%)#	-3.8% (-17.7% to 12.7%)	24.2% (12.3% to 35.9%)#	59.2% (50.5% to 68.7%)#	-19.0% (-35.6% to 3.4%)	-33.1% (-46.3% to -15.7%)#	-28.9% (-41.6% to -12.0%)#	43.3% (34.1% to 53.0%)#
	% change 2000-2017	20.6% (-7.0% to 57.2%)	12.0% (-12.8% to 43.5%)	-15.8% (-34.1% to 7.3%)	-30.3% (-46.3% to -8.6%)#	-11.4% (-24.0% to 3.7%)	-10.4% (-19.2% to -2.0%)#	19.1% (12.0% to 26.9%)#	0.3% (-21.1% to 27.0%)	-7.1% (-25.6% to 16.7%)	-6.4% (-23.6% to 15.5%)	5.4% (-1.5% to 12.7%)
Turkmenistan	Death #, 1990	2,879 (2,553 to 3,197)	815 (690 to 951)	2,263 (1,904 to 2,636)	2,746 (2,127 to 3,437)	367 (334 to 402)	228 (217 to 239)	341 (335 to 346)	5,957 (5,286 to 6,648)	8,703 (7,730 to 9,726)	9,639 (8,618 to 10,716)	569 (552 to 585)
	Death #, 2000	2,222 (1,878 to 2,615)	654 (535 to 777)	1,998 (1,668 to 2,391)	1,461 (1,133 to 1,825)	326 (299 to 357)	292 (278 to 307)	427 (420 to 434)	4,874 (4,148 to 5,694)	6,334 (5,532 to 7,239)	7,380 (6,532 to 8,323)	720 (698 to 741)
	Death #, 2017	1,169 (885 to 1,518)	332 (246 to 440)	809 (606 to 1,061)	524 (392 to 687)	177 (157 to 201)	150 (140 to 163)	295 (274 to 318)	2,310 (1,736 to 3,019)	2,834 (2,129 to 3,716)	3,457 (2,729 to 4,388)	445 (417 to 478)
	% change 1990-2017	-59.4% (-69.5% to -46.7%)#	-59.2% (-71.0% to -43.3%)#	-64.2% (-74.1% to -51.2%)#	-80.9% (-86.9% to -72.6%)#	-51.7% (-58.7% to -43.3%)#	-34.2% (-40.0% to -28.1%)#	-13.3% (-19.3% to -6.7%)#	-61.2% (-71.2% to -48.2%)#	-67.4% (-76.0% to -56.1%)#	-64.1% (-72.4% to -53.3%)#	-21.7% (-27.2% to -15.6%)#
	% change 2000-2017	-47.4% (-61.6% to -28.3%)#	-49.2% (-63.6% to -30.6%)#	-59.5% (-71.1% to -44.4%)#	-64.1% (-74.9% to -49.6%)#	-45.7% (-53.0% to -36.5%)#	-48.7% (-53.0% to -43.3%)#	-30.9% (-35.8% to -25.3%)#	-52.6% (-65.0% to -35.2%)#	-55.2% (-67.0% to -39.9%)#	-53.1% (-63.7% to -39.4%)#	-38.1% (-42.4% to -33.0%)#
Uzbekistan	Death #, 1990	10,441 (9,133 to 11,928)	3,470 (3,056 to 3,931)	12,655 (11,262 to 14,092)	9,479 (7,821 to 11,366)	1,917 (1,781 to 2,075)	1,303 (1,253 to 1,359)	1,581 (1,561 to 1,602)	26,566 (24,470 to 28,790)	36,045 (32,910 to 39,663)	40,847 (37,500 to 44,692)	2,884 (2,814 to 2,963)
	Death #, 2000	7,236 (6,166 to 8,455)	3,244 (2,742 to 3,868)	8,809 (7,616 to 10,191)	4,960 (4,068 to 5,924)	2,279 (2,084 to 2,498)	1,916 (1,823 to 2,023)	2,347 (2,268 to 2,429)	19,289 (16,652 to 22,264)	24,249 (21,211 to 27,849)	30,791 (27,426 to 34,777)	4,262 (4,107 to 4,429)
	Death #, 2017	5,168 (4,192 to 6,493)	2,416 (1,974 to 3,020)	4,452 (3,650 to 5,554)	2,619 (2,153 to 3,245)	1,454 (1,266 to 1,701)	1,166 (1,057 to 1,289)	1,879 (1,683 to 2,096)	12,036 (9,817 to 15,067)	14,656 (11,978 to 18,328)	19,156 (16,110 to 23,155)	3,046 (2,772 to 3,353)
	% change 1990-2017	-50.5% (-61.8% to -34.6%)#	-30.4% (-45.0% to -10.7%)#	-64.8% (-72.2% to -55.2%)#	-72.4% (-78.9% to -63.6%)#	-24.1% (-35.3% to -10.4%)#	-10.5% (-19.5% to -0.9%)#	18.9% (6.1% to 32.8%)#	-54.7% (-63.7% to -41.5%)#	-59.3% (-67.6% to -48.1%)#	-53.1% (-61.6% to -42.4%)#	5.6% (-4.6% to 16.6%)
	% change 2000-2017	-28.6% (-43.9% to -4.8%)#	-25.5% (-41.8% to -3.6%)#	-49.5% (-60.3% to -35.6%)#	-47.2% (-59.2% to -32.5%)#	-36.2% (-45.0% to -25.1%)#	-39.1% (-44.9% to -32.5%)#	-19.9% (-28.4% to -10.3%)#	-37.6% (-50.8% to -19.9%)#	-39.6% (-51.9% to -22.8%)#	-37.8% (-48.5% to -23.4%)#	-28.6% (-35.7% to -20.9%)#
Central Europe	Death #, 1990	13,840 (13,233 to 14,499)	4,577 (4,293 to 4,848)	12,817 (12,332 to 13,288)	6,746 (6,355 to 7,152)	3,453 (3,421 to 3,483)	3,267 (3,253 to 3,283)	6,226 (6,211 to 6,240)	31,234 (30,709 to 31,724)	37,980 (37,505 to 38,461)	50,925 (50,405 to 51,453)	9,493 (9,467 to 9,519)
	% of global total, 1990	0.5% (0.4% to 0.5%)	0.4% (0.3% to 0.4%)	0.3% (0.3% to 0.3%)	0.2% (0.2% to 0.2%)	0.4% (0.4% to 0.4%)	0.7% (0.7% to 0.7%)	0.9% (0.9% to 0.9%)	0.4% (0.4% to 0.4%)	0.3% (0.3% to 0.3%)	0.4% (0.4% to 0.4%)	0.8% (0.8% to 0.8%)
	Death #, 2000	6,097 (5,824 to 6,386)	2,303 (2,154 to 2,462)	5,437 (5,135 to 5,715)	2,603 (2,455 to 2,755)	1,951 (1,919 to 1,983)	2,515 (2,496 to 2,536)	4,963 (4,941 to 4,984)	13,836 (13,491 to 14,181)	16,440 (16,041 to 16,850)	25,869 (25,417 to 26,348)	7,478 (7,441 to 7,518)
	% of global total, 2000	0.2% (0.2% to 0.2%)	0.2% (0.2% to 0.3%)	0.2% (0.2% to 0.2%)	0.1% (0.1% to 0.1%)	0.3% (0.3% to 0.3%)	0.5% (0.5% to 0.5%)	0.7% (0.7% to 0.7%)	0.2% (0.2% to 0.2%)	0.2% (0.2% to 0.2%)	0.2% (0.2% to 0.2%)	0.6% (0.6% to 0.7%)
	Death #, 2017	2,304 (2,087 to 2,539)	1,058 (970 to 1,154)	1,763 (1,616 to 1,913)	964 (906 to 1,029)	694 (670 to 719)	815 (796 to 832)	2,022 (1,975 to 2,069)	5,124 (4,709 to 5,591)	6,088 (5,627 to 6,597)	9,618 (9,100 to 10,182)	2,837 (2,774 to 2,897)
	% of global total, 2017	0.1% (0.1% to 0.1%)	0.2% (0.2% to 0.2%)	0.1% (0.1% to 0.1%)	0.1% (0.1% to 0.1%)	0.2% (0.2% to 0.2%)	0.2% (0.2% to 0.3%)	0.4% (0.4% to 0.4%)	0.1% (0.1% to 0.1%)	0.1% (0.1% to 0.1%)	0.1% (0.1% to 0.1%)	0.3% (0.3% to 0.3%)
	% change 1990-2017	-83.4% (-85.2% to -81.4%)#	-76.9% (-79.3% to -74.4%)#	-86.2% (-87.5% to -84.9%)#	-85.7% (-86.9% to -84.5%)#	-79.9% (-80.6% to -79.2%)#	-75.1% (-75.6% to -74.5%)#	-67.5% (-68.3% to -66.8%)#	-83.6% (-85.0% to -82.1%)#	-84.0% (-85.3% to -82.7%)#	-81.1% (-82.2% to -80.0%)#	-70.1% (-70.8% to -69.5%)#
	% change 2000-2017	-62.2% (-66.2% to -57.9%)#	-54.1% (-58.6% to -49.0%)#	-67.6% (-70.8% to -64.2%)#	-63.0% (-65.9% to -59.8%)#	-64.4% (-65.7% to -63.0%)#	-67.6% (-68.4% to -66.9%)#	-59.2% (-60.2% to -58.3%)#	-63.0% (-66.0% to -59.5%)#	-63.0% (-65.8% to -59.8%)#	-62.8% (-64.8% to -60.6%)#	-62.1% (-63.0% to -61.2%)#
Albania	Death #, 1990	1,021 (808 to 1,246)	197 (159 to 241)	1,395 (1,181 to 1,636)	855 (753 to 968)	250 (232 to 268)	157 (150 to 163)	196 (193 to 199)	2,614 (2,367 to 2,868)	3,469 (3,157 to 3,806)	4,070 (3,731 to 4,432)	352 (343 to 360)
	Death #, 2000	623 (495 to 754)	129 (99 to 166)	685 (548 to 848)	278 (220 to 346)	226 (207 to 246)	144 (136 to 151)	187 (178 to 197)	1,437 (1,289 to 1,598)	1,715 (1,511 to 1,922)	2,272 (2,041 to 2,507)	331 (316 to 347)
	Death #, 2017	192 (129 to 279)	59 (38 to 88)	141 (92 to 209)	73 (52 to 99)	49 (42 to 57)	44 (39 to 50)	92 (77 to 110)	392 (277 to 546)	465 (331 to 644)	651 (502 to 839)	137 (117 to 160)
	% change 1990-2017	-81.2% (-88.2% to -69.6%)#	-70.0% (-82.1% to -51.0%)#	-89.9% (-94.0% to -83.9%)#	-91.5% (-94.0% to -88.5%)#	-80.3% (-83.3% to -76.8%)#	-71.7% (-74.8% to -68.0%)#	-52.8% (-60.7% to -43.8%)#	-85.0% (-89.5% to -79.1%)#	-86.6% (-90.5% to -81.4%)#	-84.0% (-87.8% to -79.0%)#	-61.2% (-66.8% to -54.9%)#
	% change 2000-2017	-69.2% (-80.5% to -52.1%)#	-54.2% (-71.9% to -28.0%)#	-79.4% (-87.2% to -68.6%)#	-73.9% (-82.1% to -62.2%)#	-78.2% (-81.7% to -74.2%)#	-69.1% (-73.0% to -65.3%)#	-50.7% (-59.4% to -40.6%)#	-72.7% (-81.2% to -62.1%)#	-72.9% (-81.2% to -62.1%)#	-71.4% (-78.3% to -62.8%)#	-58.7% (-64.9% to -51.3%)#
Bosnia	Death #, 1990	683 (646 to 723)	143 (127 to 160)	158 (137 to 181)	121 (101 to 145)	91 (87 to 94)	78 (77 to 80)	140 (138 to 141)	985 (934 to 1,038)	1,106 (1,050 to 1,164)	1,415 (1,354 to 1,479)	218 (215 to 221)
	Death #, 2000	223 (198 to 248)	96 (83 to 109)	64 (55 to 74)	71 (50 to 97)	44 (42 to 46)	56 (53 to 59)	123 (112 to 134)	383 (350 to 417)	454 (425 to 483)	677 (641 to 713)	179 (166 to 193)
	Death #, 2017	46 (39 to 54)	95 (81 to 111)	18 (15 to 21)	24 (21 to 28)	23 (21 to 26)	25 (24 to 27)	72 (68 to 77)	159 (135 to 186)	183 (156 to 213)	304 (273 to 340)	98 (92 to 103)
	% change 1990-2017	-93.3% (-94.3% to -92.1%)#	-33.8% (-44.5% to -19.5%)#	-88.5% (-90.5% to -86.0%)#	-80.4% (-84.1% to -75.6%)#	-74.1% (-76.8% to -70.8%)#	-67.7% (-69.6% to -65.4%)#	-48.1% (-51.3% to -44.9%)#	-83.9% (-86.1% to -81.0%)#	-83.5% (-85.8% to -80.8%)#	-78.5% (-80.8% to -75.8%)#	-55.2% (-57.6% to -52.5%)#
	% change 2000-2017	-79.4% (-83.1% to -74.9%)#	-0.9% (-19.4% to 22.0%)	-71.8% (-77.4% to -64.5%)#	-66.4% (-75.5% to -51.4%)#	-46.7% (-52.6% to -39.5%)#	-54.9% (-58.2% to -50.8%)#	-41.2% (-47.5% to -34.0%)#	-58.5% (-65.3% to -50.1%)#	-59.8% (-65.9% to -52.3%)#	-55.1% (-60.1% to -49.0%)#	-45.5% (-50.8% to -39.8%)#
Bulgaria	Death #, 1990	548 (475 to 620)	269 (234 to 310)	689 (623 to 758)	438 (377 to 501)	220 (214 to 227)	251 (247 to 255)	452 (448 to 456)	1506 (1428 to 1585)	1944 (1871 to 2018)	2867 (2781 to 2954)	703 (695 to 710)
	Death #, 2000	351 (310 to 397)	183 (158 to 210)	445 (405 to 487)	206 (181 to 231)	131 (127 to 136)	145 (142 to 148)	336 (333 to 339)	980 (922 to 1036)	1186 (1130 to 1245)	1799 (1733 to 1869)	481 (476 to 487)
	Death #, 2017	161 (124 to 207)	70 (54 to 90)	148 (117 to 188)	76 (63 to 91)	56 (51 to 61)	58 (55 to 61)	142 (134 to 150)	379 (295 to 487)	456 (358 to 579)	711 (603 to 842)	200 (189 to 211)
	% change 1990-2017	-70.6% (-78.1% to -60.9%)#	-74.0% (-80.8% to -65.1%)#	-78.5% (-83.3% to -72.3%)#	-82.6% (-86.0% to -78.1%)#	-74.7% (-77.1% to -72.0%)#	-76.9% (-78.3% to -75.4%)#	-68.6% (-70.4% to -66.8%)#	-74.8% (-80.7% to -67.7%)#	-76.6% (-81.7% to -70.3%)#	-75.2% (-79.0% to -70.4%)#	-71.6% (-73.1% to -70.0%)#
	% change 2000-2017	-54.2% (-65.9% to -39.2%)#	-61.9% (-72.0% to -48.7%)#	-66.7% (-74.0% to -57.3%)#	-63.0% (-70.0% to -53.6%)#	-57.5% (-61.8% to -53.0%)#	-60.0% (-62.5% to -57.4%)#	-57.8% (-60.3% to -55.5%)#	-61.3% (-70.0% to -50.2%)#	-61.6% (-70.0% to -51.3%)#	-60.5% (-66.7% to -53.0%)#	-58.5% (-60.8% to -56.2%)#
Croatia	Death #, 1990	324 (298 to 355)	89 (77 to 102)	157 (136 to 179)	94 (79 to 110)	103 (98 to 108)	73 (71 to 75)	193 (191 to 196)	570 (533 to 608)	664 (623 to 708)	1,033 (986 to 1,085)	267 (263 to 271)
	Death #, 2000	187 (166 to 208)	53 (44 to 63)	71 (62 to 82)	52 (45 to 60)	46 (44 to 48)	53 (51 to 54)	165 (163 to 167)	311 (288 to 335)	364 (336 to 390)	627 (594 to 659)	218 (214 to 221)
	Death #, 2017	73 (64 to 85)	28 (24 to 32)	32 (28 to 37)	25 (21 to 29)	21 (19 to 24)	20 (19 to 22)	65 (60 to 69)	133 (116 to 153)	158 (137 to 183)	264 (239 to 293)	85 (80 to 90)

eTable 2. ENN, LNN, PNN, 1-4, 5-9, 10-14, 15-19, <1, <5, 10-19, and <20 years mortality in 1990, 2000 and 2017 with mean percentage change for all GBD causes.

	Metric	Early Neonatal	Late Neonatal	Post Neonatal	1 to 4	5 to 9	10 to 14	15 to 19	<1 year	Under 5	<20 years	10 to 19
	% change 1990-2017	**-77.4% (-80.9% to -73.3%)#**	**-68.5% (-74.1% to -61.5%)#**	**-79.7% (-83.0% to -75.4%)#**	**-73.9% (-79.7% to -66.9%)#**	**-79.1% (-81.5% to -76.5%)#**	**-72.5% (-74.5% to -70.3%)#**	**-66.7% (-68.8% to -64.5%)#**	**-76.6% (-79.8% to -72.8%)#**	**-76.3% (-79.7% to -72.2%)#**	**-74.5% (-77.3% to -71.3%)#**	**-68.2% (-70.2% to -66.2%)#**
	% change 2000-2017	**-60.8% (-67.1% to -53.0%)#**	**-47.4% (-58.2% to -33.7%)#**	**-55.4% (-63.3% to -46.0%)#**	**-53.2% (-62.7% to -40.8%)#**	**-53.3% (-59.1% to -46.9%)#**	**-61.7% (-64.5% to -58.6%)#**	**-60.9% (-63.4% to -58.2%)#**	**-57.2% (-63.6% to -49.9%)#**	**-56.7% (-63.2% to -49.0%)#**	**-58.0% (-62.5% to -52.6%)#**	**-61.1% (-63.4% to -58.6%)#**
Czech	Death #, 1990	676 (602 to 747)	219 (183 to 257)	436 (384 to 494)	233 (201 to 269)	174 (168 to 180)	198 (194 to 201)	488 (484 to 492)	1331 (1268 to 1400)	1564 (1498 to 1631)	2424 (2348 to 2502)	686 (679 to 693)
	Death #, 2000	153 (134 to 174)	89 (77 to 102)	140 (127 to 155)	95 (83 to 109)	98 (93 to 103)	120 (117 to 124)	343 (339 to 347)	382 (354 to 411)	476 (445 to 509)	1038 (995 to 1081)	463 (457 to 470)
	Death #, 2017	90 (70 to 115)	47 (37 to 60)	93 (74 to 115)	60 (50 to 71)	39 (35 to 43)	49 (46 to 52)	128 (121 to 136)	230 (181 to 290)	289 (231 to 362)	505 (438 to 585)	177 (168 to 187)
	% change 1990-2017	**-86.7% (-89.9% to -82.6%)#**	**-78.4% (-83.8% to -70.6%)#**	**-78.8% (-83.4% to -72.9%)#**	**-74.5% (-79.2% to -68.2%)#**	**-77.8% (-79.9% to -75.3%)#**	**-75.1% (-76.6% to -73.6%)#**	**-73.7% (-75.2% to -72.2%)#**	**-82.7% (-86.5% to -78.3%)#**	**-81.5% (-85.2% to -76.9%)#**	**-79.2% (-81.9% to -75.7%)#**	**-74.1% (-75.6% to -72.7%)#**
	% change 2000-2017	**-41.1% (-56.2% to -22.4%)#**	**-46.8% (-60.2% to -30.2%)#**	**-33.8% (-47.8% to -16.3%)#**	**-37.2% (-48.9% to -21.1%)#**	**-60.6% (-64.6% to -55.7%)#**	**-59.2% (-61.7% to -56.5%)#**	**-62.6% (-64.7% to -60.4%)#**	**-39.8% (-53.1% to -23.2%)#**	**-39.2% (-51.7% to -23.4%)#**	**-51.3% (-58.1% to -43.1%)#**	**-61.7% (-63.8% to -59.5%)#**
Hungary	Death #, 1990	1105 (1033 to 1180)	288 (244 to 338)	499 (438 to 568)	251 (211 to 294)	183 (178 to 188)	218 (215 to 222)	545 (541 to 549)	1891 (1822 to 1962)	2142 (2058 to 2230)	3088 (2994 to 3186)	763 (756 to 770)
	Death #, 2000	431 (396 to 465)	149 (131 to 170)	270 (236 to 308)	156 (138 to 176)	100 (96 to 104)	133 (130 to 135)	279 (277 to 282)	850 (804 to 894)	1007 (958 to 1055)	1518 (1461 to 1574)	412 (407 to 417)
	Death #, 2017	142 (100 to 199)	75 (56 to 100)	128 (98 to 167)	64 (53 to 77)	37 (34 to 41)	59 (55 to 63)	137 (130 to 145)	346 (266 to 445)	410 (320 to 524)	643 (545 to 762)	196 (186 to 206)
	% change 1990-2017	**-87.1% (-91.1% to -81.8%)#**	**-73.9% (-81.8% to -63.6%)#**	**-74.3% (-81.2% to -65.8%)#**	**-74.4% (-79.9% to -67.3%)#**	**-79.6% (-81.5% to -77.3%)#**	**-73.0% (-74.6% to -71.2%)#**	**-74.9% (-76.2% to -73.4%)#**	**-81.7% (-85.9% to -76.3%)#**	**-80.9% (-85.0% to -75.6%)#**	**-79.2% (-82.3% to -75.2%)#**	**-74.3% (-75.6% to -72.9%)#**
	% change 2000-2017	**-67.0% (-77.2% to -53.2%)#**	**-49.6% (-63.6% to -31.9%)#**	**-52.6% (-64.9% to -37.3%)#**	**-59.0% (-66.8% to -48.3%)#**	**-62.5% (-66.2% to -58.5%)#**	**-55.6% (-58.2% to -52.6%)#**	**-51.0% (-53.5% to -48.0%)#**	**-59.4% (-68.9% to -48.0%)#**	**-59.3% (-68.2% to -48.2%)#**	**-57.6% (-64.1% to -49.7%)#**	**-52.5% (-54.9% to -49.8%)#**
Macedonia	Death #, 1990	477 (377 to 586)	152 (127 to 176)	525 (441 to 605)	124 (106 to 146)	66 (63 to 68)	52 (51 to 53)	88 (87 to 90)	1155 (1098 to 1212)	1279 (1221 to 1342)	1486 (1423 to 1550)	141 (138 to 143)
	Death #, 2000	189 (166 to 212)	52 (44 to 60)	124 (108 to 141)	55 (47 to 64)	35 (33 to 36)	37 (36 to 38)	72 (71 to 73)	365 (342 to 389)	421 (395 to 448)	564 (535 to 594)	109 (107 to 111)
	Death #, 2017	105 (76 to 136)	35 (27 to 45)	50 (39 to 64)	24 (20 to 28)	19 (17 to 21)	17 (16 to 18)	50 (46 to 55)	190 (166 to 217)	214 (186 to 245)	300 (270 to 336)	67 (62 to 72)
	% change 1990-2017	**-78.1% (-84.9% to -69.0%)#**	**-76.7% (-82.9% to -68.2%)#**	**-90.4% (-93.0% to -87.0%)#**	**-80.8% (-84.7% to -76.1%)#**	**-71.2% (-74.0% to -68.0%)#**	**-67.8% (-70.4% to -65.5%)#**	**-43.4% (-47.7% to -38.0%)#**	**-83.5% (-85.8% to -80.9%)#**	**-83.2% (-85.5% to -80.6%)#**	**-79.8% (-82.1% to -77.2%)#**	**-52.5% (-56.0% to -48.4%)#**
	% change 2000-2017	**-44.7% (-60.8% to -26.5%)#**	**-31.6% (-49.5% to -8.9%)#**	**-59.5% (-69.8% to -46.3%)#**	**-56.9% (-65.3% to -45.4%)#**	**-45.3% (-50.9% to -38.7%)#**	**-54.1% (-57.7% to -50.5%)#**	**-30.7% (-36.1% to -24.4%)#**	**-47.9% (-55.4% to -39.2%)#**	**-49.1% (-56.3% to -40.6%)#**	**-46.8% (-52.8% to -39.7%)#**	**-38.6% (-43.1% to -33.6%)#**
Montenegro	Death #, 1990	77 (65 to 92)	21 (18 to 25)	43 (36 to 52)	22 (17 to 28)	15 (14 to 16)	13 (12 to 13)	21 (20 to 21)	142 (124 to 161)	164 (143 to 187)	212 (190 to 237)	34 (33 to 35)
	Death #, 2000	56 (47 to 65)	16 (12 to 19)	34 (28 to 39)	12 (10 to 14)	10 (9 to 11)	11 (11 to 12)	27 (26 to 28)	105 (94 to 116)	117 (105 to 130)	166 (152 to 180)	39 (38 to 40)
	Death #, 2017	13 (11 to 15)	4 (4 to 5)	6 (5 to 7)	5 (4 to 6)	5 (4 to 5)	7 (6 to 7)	11 (11 to 12)	23 (20 to 27)	28 (24 to 33)	51 (46 to 58)	18 (17 to 20)
	% change 1990-2017	**-83.0% (-86.1% to -78.9%)#**	**-79.7% (-83.7% to -74.9%)#**	**-86.9% (-89.5% to -83.8%)#**	**-76.6% (-82.3% to -68.7%)#**	**-68.0% (-72.2% to -63.1%)#**	**-47.1% (-51.7% to -42.1%)#**	**-45.2% (-49.4% to -40.2%)#**	**-83.7% (-86.2% to -80.3%)#**	**-82.8% (-85.5% to -79.0%)#**	**-75.9% (-79.1% to -71.8%)#**	**-45.9% (-50.0% to -41.4%)#**
	% change 2000-2017	**-76.4% (-80.4% to -71.4%)#**	**-72.4% (-78.4% to -64.1%)#**	**-83.1% (-86.3% to -79.0%)#**	**-57.7% (-66.8% to -46.1%)#**	**-52.4% (-58.2% to -45.5%)#**	**-41.5% (-46.4% to -36.5%)#**	**-57.8% (-61.0% to -54.2%)#**	**-78.0% (-81.2% to -74.2%)#**	**-75.8% (-79.5% to -71.5%)#**	**-69.1% (-72.7% to -65.0%)#**	**-53.0% (-56.4% to -49.2%)#**
Poland	Death #, 1990	4,987 (4,447 to 5,523)	1,403 (1,190 to 1,616)	2,288 (2,019 to 2,570)	1,345 (1,160 to 1,543)	957 (943 to 971)	922 (914 to 929)	1,965 (1,957 to 1,972)	8,678 (8,438 to 8,928)	10,024 (9,835 to 10,223)	13,867 (13,650 to 14,087)	2,886 (2,872 to 2,901)
	Death #, 2000	1,597 (1,455 to 1,746)	565 (472 to 668)	831 (729 to 937)	509 (434 to 588)	437 (427 to 448)	566 (560 to 574)	1,763 (1,754 to 1,771)	2,993 (2,871 to 3,109)	3,502 (3,389 to 3,621)	6,268 (6,135 to 6,412)	2,329 (2,314 to 2,345)
	Death #, 2017	682 (532 to 864)	266 (207 to 339)	375 (299 to 469)	235 (206 to 271)	182 (168 to 198)	202 (192 to 213)	652 (619 to 687)	1,323 (1,041 to 1,669)	1,559 (1,258 to 1,930)	2,594 (2,265 to 3,003)	854 (813 to 898)
	% change 1990-2017	**-86.3% (-89.5% to -81.8%)#**	**-81.0% (-86.0% to -74.4%)#**	**-83.6% (-87.3% to -78.6%)#**	**-82.5% (-85.7% to -78.8%)#**	**-81.0% (-82.5% to -79.2%)#**	**-78.1% (-79.2% to -76.8%)#**	**-66.8% (-68.5% to -65.0%)#**	**-84.8% (-88.1% to -80.7%)#**	**-84.5% (-87.5% to -80.8%)#**	**-81.3% (-83.8% to -78.3%)#**	**-70.4% (-71.9% to -68.9%)#**
	% change 2000-2017	**-57.3% (-67.1% to -44.6%)#**	**-52.9% (-65.0% to -36.1%)#**	**-54.9% (-65.3% to -41.8%)#**	**-53.8% (-62.0% to -43.4%)#**	**-58.3% (-61.7% to -54.6%)#**	**-64.3% (-66.1% to -62.3%)#**	**-63.0% (-64.9% to -61.0%)#**	**-55.8% (-65.4% to -44.0%)#**	**-55.5% (-63.9% to -45.3%)#**	**-58.6% (-63.7% to -52.3%)#**	**-63.3% (-65.1% to -61.4%)#**
Romania	Death #, 1990	1,690 (1,505 to 1,870)	1,117 (971 to 1,275)	4,975 (4,698 to 5,271)	2,505 (2,187 to 2,829)	1,043 (1,029 to 1,058)	922 (915 to 930)	1,355 (1,349 to 1,361)	7,782 (7,476 to 8,071)	10,287 (10,082 to 10,507)	13,607 (13,379 to 13,852)	2,277 (2,265 to 2,290)
	Death #, 2000	1,375 (1,220 to 1,536)	661 (577 to 762)	2,103 (1,897 to 2,313)	828 (743 to 918)	579 (568 to 591)	991 (981 to 1,002)	1,007 (1,001 to 1,013)	4,138 (4,013 to 4,279)	4,966 (4,835 to 5,113)	7,544 (7,393 to 7,715)	1,999 (1,982 to 2,014)
	Death #, 2017	501 (425 to 594)	238 (203 to 282)	554 (478 to 650)	264 (230 to 307)	180 (166 to 195)	236 (225 to 248)	437 (415 to 459)	1,294 (1,102 to 1,523)	1,558 (1,348 to 1,828)	2,411 (2,170 to 2,709)	673 (641 to 705)
	% change 1990-2017	**-70.4% (-75.6% to -63.9%)#**	**-78.7% (-82.7% to -73.4%)#**	**-88.9% (-90.5% to -86.8%)#**	**-89.5% (-91.2% to -87.2%)#**	**-82.7% (-84.1% to -81.2%)#**	**-74.4% (-75.7% to -73.1%)#**	**-67.7% (-69.4% to -66.1%)#**	**-83.4% (-85.8% to -80.2%)#**	**-84.9% (-86.9% to -82.2%)#**	**-82.3% (-84.0% to -80.0%)#**	**-70.4% (-71.8% to -69.1%)#**
	% change 2000-2017	**-63.6% (-70.4% to -54.8%)#**	**-63.9% (-70.6% to -55.1%)#**	**-73.6% (-78.0% to -68.1%)#**	**-68.1% (-73.0% to -62.0%)#**	**-68.9% (-71.4% to -66.2%)#**	**-76.2% (-77.3% to -74.9%)#**	**-56.6% (-58.8% to -54.4%)#**	**-68.7% (-73.4% to -63.0%)#**	**-68.6% (-73.1% to -63.2%)#**	**-68.0% (-71.3% to -64.0%)#**	**-66.3% (-67.9% to -64.7%)#**
Serbia	Death #, 1990	1,645 (1,579 to 1,705)	500 (481 to 519)	1,276 (1,225 to 1,322)	549 (454 to 654)	200 (194 to 205)	230 (226 to 233)	438 (435 to 442)	3,422 (3,288 to 3,545)	3,971 (3,842 to 4,102)	4,839 (4,700 to 4,976)	668 (663 to 675)
	Death #, 2000	670 (560 to 786)	218 (182 to 255)	495 (413 to 579)	229 (183 to 284)	150 (132 to 170)	148 (138 to 162)	384 (374 to 393)	1,382 (1,155 to 1,618)	1,611 (1,355 to 1,885)	2,294 (2,001 to 2,610)	532 (513 to 554)
	Death #, 2017	178 (149 to 214)	81 (67 to 97)	107 (91 to 127)	51 (42 to 61)	42 (39 to 46)	54 (51 to 56)	122 (115 to 129)	366 (307 to 438)	417 (353 to 495)	636 (564 to 718)	176 (166 to 185)
	% change 1990-2017	**-89.2% (-90.9% to -87.0%)#**	**-83.9% (-86.5% to -80.7%)#**	**-91.6% (-92.9% to -90.0%)#**	**-90.7% (-92.9% to -87.6%)#**	**-78.8% (-80.6% to -76.7%)#**	**-76.7% (-78.0% to -75.3%)#**	**-72.1% (-73.8% to -70.5%)#**	**-89.3% (-91.0% to -87.2%)#**	**-89.5% (-91.1% to -87.4%)#**	**-86.9% (-88.3% to -85.1%)#**	**-73.7% (-75.1% to -72.3%)#**
	% change 2000-2017	**-73.4% (-77.8% to -68.0%)#**	**-62.9% (-69.1% to -55.3%)#**	**-78.3% (-82.0% to -74.1%)#**	**-77.6% (-82.3% to -71.8%)#**	**-71.8% (-75.9% to -67.2%)#**	**-63.9% (-67.4% to -60.3%)#**	**-68.1% (-70.2% to -66.1%)#**	**-73.5% (-78.0% to -68.1%)#**	**-74.1% (-78.2% to -69.2%)#**	**-72.3% (-75.8% to -68.3%)#**	**-66.9% (-69.2% to -64.7%)#**
Slovakia	Death #, 1990	506 (448 to 560)	153 (119 to 195)	315 (288 to 345)	167 (144 to 191)	119 (114 to 123)	119 (116 to 121)	258 (256 to 261)	974 (918 to 1,028)	1,141 (1,088 to 1,196)	1,637 (1,576 to 1,700)	377 (372 to 382)
	Death #, 2000	195 (172 to 218)	82 (71 to 95)	151 (136 to 165)	96 (83 to 110)	78 (75 to 82)	93 (91 to 95)	198 (196 to 200)	428 (402 to 454)	524 (492 to 556)	894 (855 to 933)	291 (286 to 295)
	Death #, 2017	106 (72 to 146)	47 (33 to 66)	102 (76 to 137)	54 (44 to 66)	33 (30 to 37)	32 (30 to 34)	85 (80 to 90)	255 (197 to 330)	309 (242 to 396)	460 (387 to 551)	117 (111 to 123)
	% change 1990-2017	**-79.1% (-85.9% to -70.6%)#**	**-69.1% (-79.8% to -54.9%)#**	**-67.5% (-76.3% to -56.1%)#**	**-67.8% (-74.3% to -58.8%)#**	**-71.8% (-74.7% to -68.6%)#**	**-72.9% (-74.7% to -71.2%)#**	**-67.1% (-69.0% to -65.3%)#**	**-73.8% (-80.0% to -66.0%)#**	**-72.9% (-79.0% to -65.3%)#**	**-71.9% (-76.6% to -66.0%)#**	**-69.0% (-70.6% to -67.2%)#**
	% change 2000-2017	**-45.8% (-63.3% to -23.6%)#**	**-42.5% (-61.3% to -17.2%)#**	**-32.2% (-50.5% to -8.8%)#**	**-43.8% (-55.3% to -27.9%)#**	**-57.4% (-62.0% to -52.6%)#**	**-65.4% (-67.7% to -63.0%)#**	**-57.1% (-59.5% to -54.6%)#**	**-40.4% (-54.5% to -23.3%)#**	**-41.0% (-54.5% to -24.4%)#**	**-48.5% (-57.3% to -38.1%)#**	**-59.8% (-61.9% to -57.5%)#**
	Death #, 1990	100 (91 to 110)	25 (22 to 29)	59 (52 to 67)	40 (36 to 45)	35 (33 to 36)	34 (33 to 35)	86 (85 to 88)	185 (172 to 199)	225 (210 to 242)	380 (362 to 401)	120 (118 to 123)
	Death #, 2000	48 (43 to 52)	11 (10 to 12)	22 (20 to 25)	16 (14 to 19)	16 (15 to 17)	17 (17 to 18)	78 (77 to 79)	81 (74 to 88)	97 (88 to 106)	209 (198 to 220)	95 (94 to 97)

eTable 2. ENN, LNN, PNN, 1-4, 5-9, 10-14, 15-19, <1, <5, 10-19, and <20 years mortality in 1990, 2000 and 2017 with mean percentage change for all GBD causes.

	Metric	Early Neonatal	Late Neonatal	Post Neonatal	1 to 4	5 to 9	10 to 14	15 to 19	<1 year	Under 5	<20 years	10 to 19
Slovenia	Death #, 2017	15 (13 to 18)	11 (9 to 13)	7 (6 to 8)	9 (8 to 11)	6 (6 to 7)	11 (10 to 12)	28 (26 to 30)	33 (27 to 39)	42 (36 to 50)	88 (80 to 98)	40 (37 to 42)
	% change 1990-2017	-85.0% (-87.8% to -81.7%)#	-55.9% (-64.3% to -44.9%)#	-89.0% (-91.0% to -86.6%)#	-76.5% (-81.4% to -70.9%)#	-81.9% (-83.9% to -79.7%)#	-66.9% (-69.4% to -64.2%)#	-67.1% (-69.5% to -64.7%)#	-82.3% (-85.4% to -78.6%)#	-81.2% (-84.5% to -77.3%)#	-76.8% (-79.3% to -74.0%)#	-67.1% (-69.2% to -64.8%)#
	% change 2000-2017	-68.2% (-74.5% to -61.3%)#	1.5% (-17.5% to 23.6%)	-70.7% (-76.0% to -64.0%)#	-41.3% (-54.2% to -25.1%)#	-61.4% (-65.8% to -56.2%)#	-35.5% (-40.8% to -29.7%)#	-63.6% (-66.3% to -60.8%)#	-59.5% (-66.5% to -51.1%)#	-56.4% (-64.1% to -47.7%)#	-57.7% (-62.5% to -52.6%)#	-58.4% (-61.4% to -55.5%)#
Eastern Europe	Death #, 1990	25,498 (24,609 to 26,395)	7,506 (7,008 to 8,083)	15,686 (15,259 to 16,130)	13,487 (12,904 to 14,149)	9,471 (9,287 to 9,670)	7,278 (7,199 to 7,360)	16,228 (16,168 to 16,288)	48,690 (47,370 to 50,033)	62,177 (60,618 to 63,831)	95,154 (93,297 to 97,077)	23,506 (23,369 to 23,644)
	% of global total, 1990	0.8% (0.8% to 0.9%)	0.6% (0.6% to 0.6%)	0.4% (0.4% to 0.4%)	0.4% (0.3% to 0.4%)	1.1% (1.1% to 1.1%)	1.6% (1.5% to 1.6%)	2.4% (2.4% to 2.4%)	0.6% (0.6% to 0.6%)	0.5% (0.5% to 0.5%)	0.7% (0.7% to 0.7%)	2.1% (2.0% to 2.1%)
	Death #, 2000	14,928 (14,694 to 15,166)	4,324 (4,112 to 4,577)	8,901 (8,736 to 9,080)	6,721 (6,478 to 6,987)	5,568 (5,527 to 5,607)	7,567 (7,541 to 7,591)	23,302 (23,273 to 23,330)	28,153 (27,805 to 28,482)	34,875 (34,467 to 35,272)	71,311 (70,836 to 71,776)	30,868 (30,816 to 30,918)
	% of global total, 2000	0.5% (0.5% to 0.6%)	0.5% (0.4% to 0.5%)	0.3% (0.3% to 0.3%)	0.2% (0.2% to 0.2%)	0.8% (0.8% to 0.8%)	1.6% (1.6% to 1.6%)	3.4% (3.4% to 3.5%)	0.4% (0.4% to 0.4%)	0.4% (0.3% to 0.4%)	0.6% (0.6% to 0.6%)	2.7% (2.6% to 2.7%)
	Death #, 2017	7,064 (6,811 to 7,327)	2,222 (2,042 to 2,483)	4,657 (4,445 to 4,878)	3,759 (3,548 to 3,996)	2,599 (2,565 to 2,635)	2,834 (2,807 to 2,863)	6,917 (6,816 to 7,018)	13,942 (13,384 to 14,555)	17,701 (17,090 to 18,393)	30,051 (29,372 to 30,813)	9,751 (9,629 to 9,875)
	% of global total, 2017	0.4% (0.4% to 0.4%)	0.4% (0.4% to 0.5%)	0.3% (0.3% to 0.3%)	0.3% (0.2% to 0.3%)	0.6% (0.6% to 0.7%)	0.9% (0.9% to 0.9%)	1.3% (1.3% to 1.4%)	0.3% (0.3% to 0.4%)	0.3% (0.3% to 0.3%)	0.5% (0.4% to 0.5%)	1.2% (1.1% to 1.2%)
	% change 1990-2017	-72.3% (-73.8% to -70.7%)#	-70.4% (-73.8% to -66.3%)#	-70.3% (-71.5% to -69.0%)#	-72.1% (-74.2% to -70.0%)#	-72.6% (-73.2% to -71.9%)#	-61.1% (-61.6% to -60.5%)#	-57.4% (-58.0% to -56.7%)#	-71.4% (-72.6% to -69.9%)#	-71.5% (-72.7% to -70.2%)#	-68.4% (-69.3% to -67.4%)#	-58.5% (-59.1% to -57.9%)#
	% change 2000-2017	-52.7% (-54.6% to -50.7%)#	-48.6% (-53.4% to -42.3%)#	-47.7% (-50.3% to -45.1%)#	-44.1% (-47.9% to -39.9%)#	-53.3% (-54.0% to -52.6%)#	-62.5% (-62.9% to -62.2%)#	-70.3% (-70.8% to -69.9%)#	-50.5% (-52.5% to -48.2%)#	-49.2% (-51.1% to -47.2%)#	-57.9% (-58.9% to -56.7%)#	-68.4% (-68.8% to -68.0%)#
Belarus	Death #, 1990	1,167 (999 to 1,344)	322 (275 to 375)	800 (683 to 923)	658 (522 to 811)	418 (376 to 464)	302 (286 to 320)	605 (594 to 616)	2,289 (1,955 to 2,632)	2,947 (2,571 to 3,373)	4,271 (3,824 to 4,774)	907 (880 to 935)
	Death #, 2000	662 (570 to 751)	185 (159 to 211)	463 (402 to 525)	344 (283 to 414)	230 (206 to 255)	258 (243 to 273)	663 (651 to 674)	1,309 (1,136 to 1,480)	1,654 (1,431 to 1,875)	2,804 (2,533 to 3,069)	920 (894 to 947)
	Death #, 2017	266 (201 to 359)	85 (64 to 115)	161 (123 to 215)	151 (119 to 196)	103 (90 to 119)	71 (66 to 76)	173 (164 to 182)	512 (388 to 689)	663 (507 to 886)	1,009 (830 to 1,250)	243 (230 to 257)
	% change 1990-2017	-77.2% (-83.2% to -68.6%)#	-73.6% (-80.8% to -63.4%)#	-79.9% (-85.2% to -72.7%)#	-77.0% (-82.8% to -67.5%)#	-75.5% (-79.0% to -70.9%)#	-76.6% (-78.6% to -74.3%)#	-71.5% (-73.0% to -69.7%)#	-77.6% (-83.6% to -69.4%)#	-77.5% (-83.2% to -69.9%)#	-76.4% (-81.0% to -70.4%)#	-73.2% (-74.8% to -71.3%)#
	% change 2000-2017	-59.8% (-70.0% to -44.6%)#	-53.9% (-65.8% to -37.0%)#	-65.3% (-74.0% to -53.2%)#	-56.1% (-67.1% to -40.5%)#	-55.4% (-62.0% to -47.3%)#	-72.5% (-74.9% to -69.9%)#	-74.0% (-75.5% to -72.3%)#	-60.9% (-70.8% to -46.7%)#	-59.9% (-69.9% to -46.3%)#	-64.0% (-70.8% to -55.2%)#	-73.6% (-75.2% to -71.8%)#
Estonia	Death #, 1990	140 (130 to 149)	45 (42 to 48)	97 (88 to 106)	85 (74 to 98)	63 (61 to 66)	51 (50 to 53)	129 (127 to 131)	281 (263 to 300)	367 (347 to 386)	611 (587 to 634)	181 (178 to 184)
	Death #, 2000	44 (40 to 48)	23 (20 to 26)	45 (40 to 51)	30 (25 to 34)	28 (26 to 29)	29 (28 to 30)	78 (77 to 79)	112 (103 to 123)	142 (131 to 155)	276 (262 to 292)	107 (105 to 109)
	Death #, 2017	12 (10 to 15)	5 (4 to 6)	12 (10 to 15)	11 (8 to 17)	9 (8 to 11)	9 (8 to 10)	23 (21 to 26)	30 (24 to 36)	41 (34 to 50)	82 (73 to 94)	32 (29 to 36)
	% change 1990-2017	-91.1% (-92.8% to -88.9%)#	-89.0% (-91.0% to -86.3%)#	-87.5% (-89.8% to -84.5%)#	-86.8% (-91.3% to -80.2%)#	-85.5% (-87.5% to -83.3%)#	-82.2% (-83.8% to -80.5%)#	-82.0% (-84.1% to -79.9%)#	-89.5% (-91.5% to -87.0%)#	-88.9% (-90.8% to -86.2%)#	-86.5% (-88.2% to -84.4%)#	-82.1% (-83.9% to -80.1%)#
	% change 2000-2017	-71.8% (-77.4% to -64.5%)#	-78.3% (-82.7% to -72.4%)#	-73.4% (-78.5% to -66.9%)#	-61.9% (-74.7% to -42.7%)#	-66.8% (-71.2% to -61.6%)#	-68.5% (-71.2% to -65.4%)#	-70.1% (-73.5% to -66.4%)#	-73.8% (-78.7% to -67.4%)#	-71.3% (-76.8% to -65.0%)#	-70.2% (-74.0% to -65.9%)#	-69.7% (-72.8% to -66.4%)#
Latvia	Death #, 1990	243 (223 to 262)	76 (70 to 82)	151 (138 to 164)	149 (126 to 173)	126 (121 to 130)	94 (92 to 96)	228 (225 to 230)	469 (439 to 502)	618 (585 to 648)	1,066 (1,027 to 1,103)	322 (318 to 326)
	Death #, 2000	94 (87 to 103)	42 (38 to 45)	82 (73 to 91)	51 (45 to 57)	57 (54 to 60)	59 (57 to 60)	151 (149 to 153)	218 (201 to 235)	269 (251 to 287)	535 (513 to 559)	210 (207 to 213)
	Death #, 2017	37 (28 to 48)	11 (8 to 14)	28 (22 to 36)	18 (14 to 23)	17 (15 to 18)	16 (15 to 17)	39 (34 to 43)	76 (58 to 98)	94 (76 to 116)	165 (143 to 191)	54 (49 to 60)
	% change 1990-2017	-84.8% (-88.6% to -80.1%)#	-85.8% (-89.3% to -81.4%)#	-81.3% (-85.9% to -75.8%)#	-87.7% (-90.7% to -83.6%)#	-86.7% (-87.9% to -85.2%)#	-83.2% (-84.5% to -81.8%)#	-83.1% (-84.9% to -81.1%)#	-83.8% (-87.8% to -79.2%)#	-84.7% (-87.7% to -81.1%)#	-84.5% (-86.5% to -82.0%)#	-83.1% (-84.7% to -81.4%)#
	% change 2000-2017	-60.8% (-71.0% to -48.7%)#	-74.2% (-80.9% to -66.3%)#	-65.4% (-74.2% to -54.7%)#	-64.0% (-72.0% to -53.2%)#	-70.5% (-73.7% to -67.2%)#	-73.0% (-75.1% to -70.7%)#	-74.5% (-77.2% to -71.6%)#	-65.1% (-73.9% to -54.4%)#	-64.9% (-71.8% to -56.2%)#	-69.1% (-73.5% to -64.1%)#	-74.1% (-76.6% to -71.5%)#
Lithuania	Death #, 1990	333 (310 to 356)	99 (92 to 106)	210 (192 to 228)	171 (151 to 192)	133 (129 to 138)	101 (99 to 103)	273 (270 to 276)	642 (606 to 675)	814 (774 to 851)	1,320 (1,273 to 1,365)	373 (369 to 378)
	Death #, 2000	116 (108 to 124)	43 (40 to 46)	126 (117 to 135)	85 (73 to 100)	71 (68 to 74)	77 (75 to 79)	221 (218 to 223)	285 (268 to 302)	370 (347 to 395)	738 (710 to 769)	297 (293 to 301)
	Death #, 2017	44 (37 to 54)	21 (17 to 25)	49 (41 to 58)	28 (24 to 32)	14 (12 to 15)	25 (23 to 26)	64 (60 to 68)	114 (96 to 137)	142 (120 to 169)	244 (219 to 273)	88 (84 to 94)
	% change 1990-2017	-86.7% (-89.0% to -83.7%)#	-79.0% (-82.6% to -74.2%)#	-76.6% (-80.5% to -71.5%)#	-84.0% (-86.7% to -80.5%)#	-89.8% (-90.6% to -88.7%)#	-75.5% (-76.8% to -74.0%)#	-76.7% (-78.0% to -75.2%)#	-82.2% (-85.0% to -78.5%)#	-82.6% (-85.2% to -79.1%)#	-81.5% (-83.4% to -79.2%)#	-76.3% (-77.6% to -75.0%)#
	% change 2000-2017	-61.6% (-68.5% to -53.5%)#	-52.0% (-60.4% to -41.9%)#	-61.1% (-67.4% to -53.5%)#	-67.8% (-73.8% to -59.2%)#	-80.6% (-82.5% to -78.7%)#	-67.8% (-69.8% to -65.7%)#	-71.1% (-73.0% to -69.3%)#	-59.9% (-66.7% to -51.9%)#	-61.7% (-67.8% to -54.3%)#	-67.0% (-70.5% to -62.8%)#	-70.3% (-72.0% to -68.5%)#
Moldova	Death #, 1990	1,078 (947 to 1,210)	382 (277 to 511)	852 (746 to 969)	672 (575 to 780)	310 (284 to 336)	188 (180 to 196)	307 (302 to 311)	2,313 (2,035 to 2,598)	2,985 (2,647 to 3,342)	3,790 (3,410 to 4,184)	495 (482 to 507)
	Death #, 2000	667 (597 to 745)	256 (211 to 307)	350 (291 to 417)	184 (145 to 230)	169 (156 to 184)	172 (164 to 180)	278 (274 to 282)	1,273 (1,131 to 1,433)	1,457 (1,289 to 1,642)	2,076 (1,888 to 2,283)	450 (438 to 462)
	Death #, 2017	314 (225 to 424)	107 (86 to 134)	89 (64 to 119)	73 (55 to 95)	57 (50 to 65)	50 (47 to 54)	97 (94 to 101)	509 (381 to 668)	582 (436 to 763)	786 (629 to 983)	147 (141 to 154)
	% change 1990-2017	-70.9% (-80.0% to -58.9%)#	-72.1% (-80.9% to -58.4%)#	-89.6% (-92.8% to -85.5%)#	-89.2% (-92.1% to -85.5%)#	-81.6% (-84.3% to -78.5%)#	-73.4% (-75.6% to -71.0%)#	-68.3% (-69.4% to -67.1%)#	-78.0% (-84.0% to -69.8%)#	-80.5% (-85.8% to -73.5%)#	-79.3% (-83.8% to -73.2%)#	-70.2% (-71.7% to -68.7%)#
	% change 2000-2017	-53.0% (-66.7% to -35.3%)#	-58.3% (-68.7% to -45.1%)#	-74.7% (-82.6% to -64.5%)#	-60.5% (-71.4% to -44.4%)#	-66.3% (-71.2% to -60.9%)#	-70.9% (-73.3% to -68.3%)#	-64.9% (-66.2% to -63.7%)#	-60.0% (-70.9% to -46.3%)#	-60.1% (-70.9% to -46.2%)#	-62.1% (-70.5% to -51.8%)#	-67.2% (-68.9% to -65.6%)#
Russian Federation	Death #, 1990	16,760 (16,620 to 16,909)	4,667 (4,627 to 4,708)	10,712 (10,600 to 10,827)	8,970 (8,805 to 9,155)	6,360 (6,332 to 6,389)	4,952 (4,939 to 4,965)	11,481 (11,460 to 11,501)	32,139 (31,899 to 32,371)	41,109 (40,852 to 41,398)	63,901 (63,601 to 64,235)	16,433 (16,402 to 16,463)
	Death #, 2000	10,717 (10,620 to 10,811)	2,987 (2,963 to 3,011)	6,284 (6,222 to 6,349)	4,905 (4,803 to 5,016)	3,942 (3,925 to 3,960)	5,587 (5,574 to 5,600)	18,449 (18,429 to 18,470)	19,988 (19,831 to 20,138)	24,893 (24,743 to 25,045)	52,871 (52,687 to 53,061)	24,037 (24,005 to 24,067)
	Death #, 2017	4,952 (4,844 to 5,065)	1,508 (1,476 to 1,542)	3,111 (3,047 to 3,180)	2,588 (2,527 to 2,654)	1,797 (1,784 to 1,810)	1,990 (1,979 to 2,001)	4,560 (4,526 to 4,593)	9,572 (9,368 to 9,781)	12,160 (11,902 to 12,437)	20,507 (20,236 to 20,796)	6,550 (6,506 to 6,591)
	% change 1990-2017	-70.5% (-71.2% to -69.7%)#	-67.7% (-68.5% to -66.9%)#	-71.0% (-71.6% to -70.2%)#	-71.2% (-72.0% to -70.2%)#	-71.7% (-72.0% to -71.5%)#	-59.8% (-60.0% to -59.6%)#	-60.3% (-60.6% to -60.0%)#	-70.2% (-70.9% to -69.5%)#	-70.4% (-71.0% to -69.7%)#	-67.9% (-68.3% to -67.4%)#	-60.1% (-60.4% to -59.9%)#
	% change 2000-2017	-53.8% (-54.8% to -52.7%)#	-49.5% (-50.6% to -48.3%)#	-50.5% (-51.6% to -49.3%)#	-47.2% (-48.8% to -45.6%)#	-54.4% (-54.8% to -54.0%)#	-64.4% (-64.6% to -64.2%)#	-75.3% (-75.5% to -75.1%)#	-52.1% (-53.1% to -51.0%)#	-51.1% (-52.2% to -50.0%)#	-61.2% (-61.7% to -60.7%)#	-72.8% (-72.9% to -72.6%)#
Ukraine	Death #, 1990	5,778 (4,906 to 6,704)	1,915 (1,400 to 2,460)	2,864 (2,478 to 3,276)	2,782 (2,242 to 3,373)	2,061 (1,877 to 2,262)	1,589 (1,510 to 1,671)	3,206 (3,150 to 3,262)	10,557 (9,333 to 11,891)	13,339 (11,897 to 14,916)	20,195 (18,453 to 22,106)	4,796 (4,661 to 4,921)
	Death #, 2000	2,629 (2,443 to 2,806)	789 (590 to 1,031)	1,551 (1,420 to 1,676)	1,122 (928 to 1,325)	1,072 (1,053 to 1,091)	1,386 (1,372 to 1,399)	3,462 (3,444 to 3,479)	4,968 (4,761 to 5,176)	6,090 (5,880 to 6,320)	12,010 (11,772 to 12,259)	4,848 (4,819 to 4,877)
	Death #, 2017	1,438 (1,260 to 1,645)	485 (313 to 743)	1,207 (1,013 to 1,419)	890 (699 to 1,115)	602 (575 to 632)	674 (649 to 700)	1,962 (1,866 to 2,059)	3,130 (2,676 to 3,681)	4,020 (3,492 to 4,635)	7,258 (6,691 to 7,904)	2,635 (2,518 to 2,750)

eTable 2. ENN, LNN, PNN, 1-4, 5-9, 10-14, 15-19, <1, <5, 10-19, and <20 years mortality in 1990, 2000 and 2017 with mean percentage change for all GBD causes.

	Metric	Early Neonatal	Late Neonatal	Post Neonatal	1 to 4	5 to 9	10 to 14	15 to 19	<1 year	Under 5	<20 years	10 to 19
	% change 1990-2017	-75.1% (-80.0% to -68.7%)#	-74.7% (-85.3% to -56.1%)#	-57.9% (-63.9% to -51.0%)#	-68.0% (-76.8% to -56.6%)#	-70.8% (-73.6% to -67.4%)#	-57.6% (-60.0% to -54.9%)#	-38.8% (-41.9% to -35.6%)#	-70.3% (-75.6% to -64.2%)#	-69.9% (-74.8% to -64.3%)#	-64.1% (-68.2% to -59.6%)#	-45.0% (-47.7% to -42.1%)#
	% change 2000-2017	-45.3% (-53.0% to -36.7%)#	-38.5% (-61.8% to 1.7%)	-22.2% (-35.8% to -7.0%)#	-20.7% (-42.5% to 8.7%)	-43.8% (-46.6% to -40.8%)#	-51.4% (-53.2% to -49.5%)#	-43.3% (-46.1% to -40.4%)#	-37.0% (-46.5% to -26.0%)#	-34.0% (-42.6% to -24.1%)#	-39.6% (-44.5% to -34.2%)#	-45.6% (-48.0% to -43.2%)#
High-income	Death #, 1990	54,254 (53,284 to 55,355)	14,096 (13,676 to 14,512)	43,048 (41,942 to 44,200)	23,591 (22,804 to 24,395)	15,090 (14,787 to 15,413)	15,085 (14,967 to 15,204)	45,005 (44,916 to 45,089)	111,397 (110,350 to 112,445)	134,989 (133,878 to 136,154)	210,168 (208,570 to 211,818)	60,090 (59,889 to 60,296)
	% of global total, 1990	1.8% (1.7% to 1.8%)	1.1% (1.1% to 1.2%)	1.1% (1.1% to 1.2%)	0.7% (0.6% to 0.7%)	1.8% (1.7% to 1.8%)	3.2% (3.2% to 3.3%)	6.7% (6.6% to 6.8%)	1.4% (1.3% to 1.4%)	1.1% (1.1% to 1.2%)	1.5% (1.5% to 1.6%)	5.3% (5.2% to 5.3%)
	Death #, 2000	36,774 (36,181 to 37,352)	10,965 (10,666 to 11,271)	26,016 (25,403 to 26,609)	15,376 (14,915 to 15,882)	9,638 (9,597 to 9,680)	11,068 (11,041 to 11,095)	34,283 (34,246 to 34,320)	73,755 (73,130 to 74,394)	89,131 (88,610 to 89,619)	144,120 (143,521 to 144,703)	45,351 (45,294 to 45,408)
	% of global total, 2000	1.3% (1.3% to 1.4%)	1.2% (1.1% to 1.2%)	0.8% (0.8% to 0.9%)	0.5% (0.5% to 0.6%)	1.4% (1.4% to 1.4%)	2.3% (2.3% to 2.3%)	5.0% (5.0% to 5.1%)	1.1% (1.1% to 1.1%)	0.9% (0.9% to 0.9%)	1.2% (1.2% to 1.3%)	3.9% (3.9% to 3.9%)
	Death #, 2017	26,022 (25,166 to 26,962)	7,917 (7,611 to 8,244)	16,626 (16,071 to 17,214)	9,636 (9,381 to 9,899)	6,016 (5,917 to 6,122)	7,120 (7,037 to 7,208)	22,797 (22,403 to 23,232)	50,565 (48,939 to 52,356)	60,201 (58,363 to 62,241)	96,134 (94,086 to 98,387)	29,917 (29,454 to 30,417)
	% of global total, 2017	1.4% (1.3% to 1.5%)	1.6% (1.5% to 1.6%)	1.0% (1.0% to 1.1%)	0.7% (0.7% to 0.7%)	1.5% (1.4% to 1.5%)	2.2% (2.2% to 2.3%)	4.4% (4.3% to 4.5%)	1.3% (1.2% to 1.3%)	1.1% (1.1% to 1.2%)	1.4% (1.4% to 1.5%)	3.6% (3.5% to 3.7%)
	% change 1990-2017	-52.0% (-53.9% to -50.0%)#	-43.8% (-46.7% to -40.7%)#	-61.4% (-63.0% to -59.7%)#	-59.1% (-60.7% to -57.3%)#	-60.1% (-61.2% to -59.0%)#	-52.8% (-53.5% to -52.1%)#	-49.4% (-50.2% to -48.4%)#	-54.6% (-56.1% to -53.0%)#	-55.4% (-56.8% to -53.8%)#	-54.3% (-55.3% to -53.0%)#	-50.2% (-51.0% to -49.4%)#
	% change 2000-2017	-29.2% (-32.0% to -26.6%)#	-27.8% (-31.4% to -24.1%)#	-36.1% (-38.6% to -33.3%)#	-37.3% (-39.8% to -34.5%)#	-37.6% (-38.6% to -36.5%)#	-35.7% (-36.4% to -34.9%)#	-33.5% (-34.7% to -32.2%)#	-31.4% (-33.7% to -29.0%)#	-32.5% (-34.5% to -30.1%)#	-33.3% (-34.7% to -31.7%)#	-34.0% (-35.1% to -32.9%)#
Australasia	Death #, 1990	1,194 (1,116 to 1,273)	280 (247 to 316)	1,122 (1,020 to 1,223)	499 (445 to 559)	293 (286 to 300)	300 (296 to 304)	1,213 (1,204 to 1,221)	2,595 (2,503 to 2,689)	3,094 (3,003 to 3,192)	4,900 (4,796 to 5,015)	1,513 (1,502 to 1,524)
	% of global total, 1990	0.0% (0.0% to 0.0%)	0.0% (0.0% to 0.0%)	0.0% (0.0% to 0.0%)	0.0% (0.0% to 0.0%)	0.0% (0.0% to 0.0%)	0.1% (0.1% to 0.1%)	0.2% (0.2% to 0.2%)	0.0% (0.0% to 0.0%)	0.0% (0.0% to 0.0%)	0.0% (0.0% to 0.0%)	0.1% (0.1% to 0.1%)
	Death #, 2000	855 (795 to 916)	206 (185 to 230)	565 (513 to 613)	345 (309 to 383)	220 (214 to 226)	261 (257 to 264)	886 (880 to 893)	1,626 (1,567 to 1,694)	1,972 (1,903 to 2,041)	3,338 (3,259 to 3,418)	1,147 (1,137 to 1,156)
	% of global total, 2000	0.0% (0.0% to 0.0%)	0.0% (0.0% to 0.0%)	0.0% (0.0% to 0.0%)	0.0% (0.0% to 0.0%)	0.0% (0.0% to 0.0%)	0.1% (0.1% to 0.1%)	0.1% (0.1% to 0.1%)	0.0% (0.0% to 0.0%)	0.0% (0.0% to 0.0%)	0.0% (0.0% to 0.0%)	0.1% (0.1% to 0.1%)
	Death #, 2017	705 (566 to 880)	157 (124 to 198)	393 (326 to 475)	239 (202 to 285)	173 (158 to 190)	172 (161 to 183)	583 (535 to 633)	1,255 (1,016 to 1,551)	1,494 (1,216 to 1,834)	2,422 (2,107 to 2,797)	756 (697 to 815)
	% of global total, 2017	0.0% (0.0% to 0.1%)	0.0% (0.0% to 0.0%)	0.0% (0.0% to 0.0%)	0.0% (0.0% to 0.0%)	0.0% (0.0% to 0.1%)	0.1% (0.1% to 0.1%)	0.1% (0.1% to 0.1%)	0.0% (0.0% to 0.0%)	0.0% (0.0% to 0.0%)	0.0% (0.0% to 0.0%)	0.1% (0.1% to 0.1%)
	% change 1990-2017	-40.9% (-53.4% to -25.6%)#	-44.0% (-56.8% to -26.9%)#	-65.0% (-71.2% to -57.1%)#	-52.1% (-61.1% to -41.0%)#	-41.0% (-46.2% to -35.1%)#	-42.6% (-46.4% to -38.7%)#	-51.9% (-55.9% to -47.8%)#	-51.6% (-60.9% to -40.4%)#	-51.7% (-60.8% to -41.0%)#	-50.6% (-57.1% to -42.7%)#	-50.0% (-54.0% to -46.1%)#
	% change 2000-2017	-17.5% (-33.7% to 2.6%)	-24.0% (-40.8% to -1.3%)#	-30.4% (-43.4% to -13.7%)#	-30.9% (-43.0% to -14.8%)#	-21.4% (-28.3% to -13.8%)#	-33.9% (-38.1% to -29.7%)#	-34.2% (-39.8% to -28.3%)#	-22.8% (-37.6% to -4.7%)#	-24.2% (-37.9% to -7.0%)#	-27.4% (-36.8% to -16.3%)#	-34.1% (-39.2% to -29.0%)#
Australia	Death #, 1990	997 (923 to 1,081)	222 (190 to 258)	783 (686 to 878)	395 (341 to 449)	234 (228 to 241)	236 (232 to 240)	918 (911 to 925)	2,002 (1,912 to 2,090)	2,396 (2,313 to 2,486)	3,785 (3,690 to 3,887)	1,154 (1,144 to 1,164)
	Death #, 2000	692 (632 to 752)	171 (150 to 195)	414 (364 to 462)	260 (225 to 296)	170 (165 to 175)	197 (193 to 200)	703 (697 to 708)	1,277 (1,216 to 1,343)	1,537 (1,474 to 1,606)	2,606 (2,532 to 2,687)	899 (891 to 908)
	Death #, 2017	583 (447 to 757)	130 (98 to 172)	312 (246 to 394)	196 (158 to 242)	131 (117 to 148)	140 (129 to 151)	461 (413 to 512)	1,025 (790 to 1,321)	1,222 (948 to 1,564)	1,954 (1,641 to 2,317)	601 (543 to 661)
	% change 1990-2017	-41.5% (-56.2% to -22.9%)#	-41.4% (-57.1% to -19.7%)#	-60.2% (-69.1% to -47.8%)#	-50.3% (-61.6% to -35.8%)#	-43.9% (-50.2% to -36.7%)#	-40.5% (-45.4% to -35.7%)#	-49.8% (-55.0% to -44.2%)#	-48.8% (-60.2% to -33.9%)#	-49.0% (-60.4% to -35.0%)#	-48.4% (-56.6% to -38.5%)#	-47.9% (-53.0% to -42.9%)#
	% change 2000-2017	-15.7% (-36.1% to 10.3%)	-23.9% (-44.2% to 3.5%)	-24.7% (-42.5% to -1.0%)#	-24.6% (-41.4% to -2.2%)#	-22.8% (-31.4% to -13.0%)#	-28.6% (-34.0% to -22.9%)#	-34.4% (-41.2% to -27.0%)#	-19.7% (-38.6% to 4.6%)	-20.5% (-38.7% to 1.6%)	-25.0% (-37.1% to -10.5%)#	-33.1% (-39.5% to -26.5%)#
New Zealand	Death #, 1990	197 (181 to 213)	58 (51 to 65)	339 (317 to 361)	105 (93 to 118)	59 (57 to 61)	64 (63 to 65)	294 (291 to 298)	593 (558 to 629)	698 (662 to 736)	1,115 (1,075 to 1,159)	358 (354 to 363)
	Death #, 2000	164 (152 to 175)	35 (30 to 41)	151 (142 to 159)	85 (76 to 95)	50 (48 to 52)	64 (63 to 65)	184 (181 to 186)	349 (330 to 367)	435 (413 to 456)	732 (707 to 758)	248 (244 to 251)
	Death #, 2017	122 (110 to 134)	26 (24 to 29)	81 (74 to 89)	43 (38 to 49)	42 (38 to 45)	32 (30 to 33)	122 (116 to 129)	229 (208 to 252)	272 (245 to 300)	468 (436 to 502)	154 (147 to 162)
	% change 1990-2017	-38.1% (-45.3% to -29.7%)#	-54.2% (-60.9% to -46.6%)#	-76.1% (-78.6% to -73.2%)#	-59.2% (-66.0% to -51.8%)#	-29.2% (-35.3% to -22.8%)#	-50.2% (-52.7% to -47.5%)#	-58.5% (-60.5% to -56.2%)#	-61.3% (-65.4% to -57.0%)#	-61.0% (-65.2% to -56.6%)#	-58.0% (-61.3% to -54.7%)#	-57.0% (-59.0% to -54.8%)#
	% change 2000-2017	-25.5% (-33.8% to -16.2%)#	-24.6% (-36.5% to -9.8%)#	-46.1% (-51.1% to -40.6%)#	-50.0% (-57.6% to -40.7%)#	-16.8% (-23.5% to -9.3%)#	-50.2% (-52.6% to -47.6%)#	-33.3% (-36.7% to -29.8%)#	-34.3% (-40.9% to -27.6%)#	-37.4% (-43.5% to -30.6%)#	-36.1% (-40.7% to -31.1%)#	-37.7% (-40.6% to -34.7%)#
High-income Asia Pacific	Death #, 1990	4,105 (3,756 to 4,480)	1,487 (1,342 to 1,660)	4,307 (3,891 to 4,767)	4,677 (4,297 to 5,108)	4,176 (3,882 to 4,502)	3,247 (3,135 to 3,369)	8,445 (8,370 to 8,522)	9,898 (9,368 to 10,443)	14,575 (13,708 to 15,538)	30,443 (29,095 to 31,933)	11,692 (11,505 to 11,890)
	% of global total, 1990	0.1% (0.1% to 0.1%)	0.1% (0.1% to 0.1%)	0.1% (0.1% to 0.1%)	0.1% (0.1% to 0.1%)	0.5% (0.5% to 0.5%)	0.7% (0.7% to 0.7%)	1.2% (1.2% to 1.3%)	0.1% (0.1% to 0.1%)	0.1% (0.1% to 0.1%)	0.2% (0.2% to 0.2%)	1.0% (1.0% to 1.1%)
	Death #, 2000	2,752 (2,627 to 2,876)	1,134 (1,045 to 1,229)	3,077 (2,878 to 3,270)	2,893 (2,705 to 3,101)	1,716 (1,698 to 1,734)	1,436 (1,428 to 1,446)	4,352 (4,341 to 4,362)	6,963 (6,749 to 7,162)	9,856 (9,721 to 10,006)	17,360 (17,203 to 17,537)	5,788 (5,769 to 5,805)
	% of global total, 2000	0.1% (0.1% to 0.1%)	0.1% (0.1% to 0.1%)	0.1% (0.1% to 0.1%)	0.1% (0.1% to 0.1%)	0.2% (0.2% to 0.2%)	0.3% (0.3% to 0.3%)	0.6% (0.6% to 0.7%)	0.1% (0.1% to 0.1%)	0.1% (0.1% to 0.1%)	0.1% (0.1% to 0.1%)	0.5% (0.5% to 0.5%)
	Death #, 2017	1,194 (1,102 to 1,275)	484 (441 to 524)	1,320 (1,187 to 1,458)	1,004 (962 to 1,049)	675 (657 to 692)	668 (657 to 679)	1,992 (1,941 to 2,046)	2,998 (2,838 to 3,166)	4,002 (3,809 to 4,209)	7,337 (7,102 to 7,579)	2,660 (2,601 to 2,723)
	% of global total, 2017	0.1% (0.1% to 0.1%)	0.1% (0.1% to 0.1%)	0.1% (0.1% to 0.1%)	0.1% (0.1% to 0.1%)	0.2% (0.2% to 0.2%)	0.2% (0.2% to 0.2%)	0.4% (0.4% to 0.4%)	0.1% (0.1% to 0.1%)	0.1% (0.1% to 0.1%)	0.1% (0.1% to 0.1%)	0.3% (0.3% to 0.3%)
	% change 1990-2017	-70.9% (-74.3% to -67.1%)#	-67.4% (-72.0% to -62.3%)#	-69.4% (-74.0% to -63.9%)#	-78.5% (-80.5% to -76.5%)#	-83.8% (-85.0% to -82.5%)#	-79.4% (-80.2% to -78.6%)#	-76.4% (-77.1% to -75.8%)#	-69.7% (-71.8% to -67.4%)#	-72.5% (-74.5% to -70.4%)#	-75.9% (-77.2% to -74.6%)#	-77.2% (-77.9% to -76.6%)#
	% change 2000-2017	-56.6% (-60.8% to -52.6%)#	-57.3% (-62.6% to -51.4%)#	-57.1% (-62.4% to -51.4%)#	-65.3% (-68.1% to -62.4%)#	-60.7% (-61.7% to -59.5%)#	-53.5% (-54.3% to -52.7%)#	-54.2% (-55.4% to -53.0%)#	-57.0% (-59.3% to -54.2%)#	-59.4% (-61.4% to -57.3%)#	-57.7% (-59.1% to -56.3%)#	-54.0% (-55.1% to -52.9%)#
Brunei	Death #, 1990	29 (25 to 33)	9 (7 to 11)	23 (20 to 26)	18 (15 to 22)	12 (11 to 13)	14 (14 to 15)	21 (20 to 22)	61 (54 to 68)	79 (72 to 86)	127 (119 to 136)	36 (34 to 37)
	Death #, 2000	27 (24 to 31)	9 (7 to 10)	21 (18 to 24)	14 (12 to 17)	10 (9 to 10)	12 (11 to 12)	20 (19 to 21)	56 (49 to 65)	70 (61 to 81)	112 (102 to 124)	32 (31 to 33)
	Death #, 2017	21 (17 to 24)	8 (7 to 10)	23 (19 to 27)	8 (7 to 10)	7 (6 to 9)	10 (9 to 11)	24 (22 to 26)	52 (43 to 61)	60 (50 to 71)	101 (89 to 116)	34 (30 to 36)
	% change 1990-2017	-29.1% (-44.7% to -10.0%)#	-3.3% (-23.2% to 20.4%)	-0.5% (-22.2% to 25.6%)	-53.7% (-63.1% to -42.9%)#	-40.4% (-49.1% to -26.6%)#	-32.2% (-40.2% to -21.3%)#	11.8% (1.7% to 22.7%)#	-14.6% (-31.6% to 6.2%)	-23.7% (-36.8% to -8.4%)#	-20.3% (-31.2% to -8.0%)#	-6.0% (-14.8% to 2.9%)
	% change 2000-2017	-24.3% (-38.9% to -4.7%)#	-2.3% (-21.6% to 20.9%)	11.4% (-10.5% to 40.5%)	-39.9% (-52.2% to -24.5%)#	-26.0% (-37.3% to -8.1%)#	-17.7% (-27.9% to -3.8%)#	19.4% (7.6% to 31.8%)#	-8.0% (-25.5% to 15.5%)	-14.4% (-30.7% to 6.5%)	-9.8% (-23.1% to 6.1%)	5.5% (-5.1% to 16.4%)
Japan	Death #, 1990	2,394 (2,322 to 2,472)	871 (825 to 917)	2,399 (2,345 to 2,456)	2,276 (2,197 to 2,356)	1,410 (1,400 to 1,420)	1,250 (1,245 to 1,255)	4,366 (4,356 to 4,376)	5,665 (5,567 to 5,761)	7,941 (7,853 to 8,030)	14,967 (14,856 to 15,072)	5,616 (5,602 to 5,630)
	Death #, 2000	1,502 (1,445 to 1,555)	619 (584 to 655)	1,727 (1,679 to 1,773)	1,442 (1,390 to 1,497)	758 (751 to 764)	759 (755 to 762)	2,490 (2,484 to 2,496)	3,848 (3,761 to 3,937)	5,290 (5,213 to 5,369)	9,297 (9,208 to 9,384)	3,249 (3,240 to 3,258)
	Death #, 2017	716 (663 to 772)	250 (232 to 271)	755 (707 to 806)	688 (655 to 726)	427 (415 to 438)	438 (430 to 446)	1,218 (1,189 to 1,248)	1,720 (1,604 to 1,847)	2,409 (2,265 to 2,567)	4,492 (4,327 to 4,673)	1,656 (1,621 to 1,693)

eTable 2. ENN, LNN, PNN, 1-4, 5-9, 10-14, 15-19, <1, <5, 10-19, and <20 years mortality in 1990, 2000 and 2017 with mean percentage change for all GBD causes.

	Metric	Early Neonatal	Late Neonatal	Post Neonatal	1 to 4	5 to 9	10 to 14	15 to 19	<1 year	Under 5	<20 years	10 to 19
	% change 1990-2017	**-70.1% (-72.5% to -67.4%)#**	**-71.3% (-73.8% to -68.4%)#**	**-68.5% (-70.6% to -66.4%)#**	**-69.8% (-71.6% to -67.9%)#**	**-69.7% (-70.6% to -68.9%)#**	**-64.9% (-65.6% to -64.3%)#**	**-72.1% (-72.8% to -71.4%)#**	**-69.6% (-71.8% to -67.3%)#**	**-69.7% (-71.5% to -67.7%)#**	**-70.0% (-71.1% to -68.8%)#**	**-70.5% (-71.1% to -69.8%)#**
	% change 2000-2017	**-52.4% (-56.1% to -48.2%)#**	**-59.6% (-63.3% to -55.4%)#**	**-56.3% (-59.1% to -53.1%)#**	**-52.3% (-55.0% to -49.0%)#**	**-43.7% (-45.2% to -42.1%)#**	**-42.2% (-43.3% to -41.2%)#**	**-51.1% (-52.2% to -49.9%)#**	**-55.3% (-58.5% to -52.0%)#**	**-54.5% (-57.3% to -51.6%)#**	**-51.7% (-53.5% to -49.8%)#**	**-49.0% (-50.1% to -47.8%)#**
	Death #, 1990	121 (106 to 140)	50 (40 to 61)	133 (119 to 147)	112 (93 to 132)	75 (72 to 78)	59 (58 to 61)	258 (255 to 261)	304 (283 to 325)	416 (395 to 439)	807 (781 to 835)	317 (313 to 321)
	Death #, 2000	77 (66 to 89)	34 (27 to 42)	99 (89 to 110)	82 (69 to 94)	41 (40 to 43)	48 (47 to 49)	164 (162 to 166)	209 (190 to 230)	291 (276 to 308)	544 (525 to 564)	212 (209 to 214)
Aichi	Death #, 2017	43 (31 to 59)	16 (12 to 23)	50 (37 to 65)	42 (35 to 50)	27 (24 to 31)	28 (25 to 30)	74 (66 to 84)	109 (80 to 146)	151 (115 to 196)	280 (236 to 332)	102 (92 to 113)
	% change 1990-2017	**-64.6% (-75.8% to -48.6%)#**	**-67.3% (-78.1% to -51.1%)#**	**-62.5% (-72.4% to -49.2%)#**	**-62.8% (-71.1% to -52.8%)#**	**-63.1% (-67.5% to -58.0%)#**	**-53.4% (-57.2% to -49.0%)#**	**-71.3% (-74.6% to -67.4%)#**	**-64.1% (-73.8% to -50.5%)#**	**-63.8% (-72.5% to -52.4%)#**	**-65.3% (-70.8% to -58.6%)#**	**-67.9% (-71.2% to -64.2%)#**
	% change 2000-2017	**-44.3% (-60.9% to -19.9%)#**	**-51.6% (-67.9% to -28.1%)#**	**-49.6% (-63.0% to -32.6%)#**	**-49.0% (-59.2% to -35.5%)#**	**-33.7% (-41.5% to -24.6%)#**	**-41.7% (-46.6% to -36.2%)#**	**-54.8% (-60.0% to -48.8%)#**	**-48.0% (-62.3% to -29.3%)#**	**-48.3% (-60.7% to -32.3%)#**	**-48.6% (-56.5% to -38.6%)#**	**-51.9% (-56.8% to -46.4%)#**
	Death #, 1990	28 (23 to 34)	7 (5 to 10)	20 (17 to 23)	24 (21 to 29)	14 (13 to 15)	13 (12 to 13)	35 (34 to 35)	55 (49 to 62)	79 (73 to 86)	141 (134 to 149)	48 (47 to 49)
	Death #, 2000	12 (10 to 14)	4 (3 to 5)	10 (9 to 12)	12 (10 to 15)	9 (8 to 10)	7 (7 to 7)	26 (25 to 26)	26 (24 to 29)	38 (35 to 42)	80 (76 to 84)	33 (32 to 33)
Akita	Death #, 2017	3 (2 to 5)	2 (1 to 2)	5 (3 to 6)	5 (4 to 6)	4 (4 to 5)	4 (4 to 5)	9 (8 to 10)	9 (7 to 13)	14 (10 to 19)	32 (27 to 38)	14 (12 to 15)
	% change 1990-2017	**-88.0% (-92.4% to -82.1%)#**	**-78.8% (-87.3% to -65.6%)#**	**-77.2% (-84.2% to -68.2%)#**	**-80.5% (-84.8% to -75.3%)#**	**-68.3% (-73.2% to -63.1%)#**	**-65.9% (-69.2% to -62.3%)#**	**-73.6% (-76.8% to -70.0%)#**	**-82.9% (-88.5% to -76.1%)#**	**-82.2% (-87.1% to -76.2%)#**	**-77.2% (-81.0% to -72.8%)#**	**-71.5% (-74.5% to -68.0%)#**
	% change 2000-2017	**-71.7% (-81.4% to -57.8%)#**	**-63.9% (-77.3% to -44.7%)#**	**-55.0% (-68.2% to -36.5%)#**	**-60.6% (-71.0% to -46.1%)#**	**-50.1% (-57.8% to -41.3%)#**	**-36.2% (-43.0% to -29.2%)#**	**-64.5% (-68.9% to -59.8%)#**	**-64.0% (-75.0% to -49.4%)#**	**-63.0% (-73.0% to -50.1%)#**	**-59.7% (-66.5% to -51.9%)#**	**-58.6% (-63.1% to -53.5%)#**
	Death #, 1990	41 (34 to 49)	10 (7 to 13)	32 (28 to 36)	32 (27 to 38)	23 (22 to 24)	12 (12 to 13)	42 (42 to 43)	83 (74 to 93)	115 (106 to 124)	193 (183 to 204)	55 (54 to 56)
	Death #, 2000	23 (20 to 27)	7 (5 to 8)	18 (15 to 20)	17 (13 to 20)	7 (7 to 7)	9 (8 to 9)	28 (28 to 29)	48 (44 to 52)	64 (59 to 69)	108 (102 to 114)	37 (36 to 37)
Aomori	Death #, 2017	7 (5 to 10)	2 (2 to 3)	6 (5 to 8)	8 (6 to 10)	5 (4 to 6)	4 (4 to 5)	13 (12 to 15)	16 (11 to 22)	23 (17 to 31)	46 (39 to 56)	18 (16 to 20)
	% change 1990-2017	**-82.2% (-88.4% to -73.9%)#**	**-76.4% (-85.1% to -63.4%)#**	**-80.7% (-86.0% to -73.9%)#**	**-76.0% (-82.6% to -68.2%)#**	**-78.9% (-82.2% to -75.5%)#**	**-64.4% (-67.9% to -60.6%)#**	**-68.1% (-71.9% to -63.8%)#**	**-80.9% (-86.7% to -73.9%)#**	**-79.6% (-85.1% to -73.0%)#**	**-76.0% (-80.3% to -71.2%)#**	**-67.2% (-70.7% to -63.5%)#**
	% change 2000-2017	**-68.8% (-78.7% to -55.9%)#**	**-65.8% (-77.6% to -48.3%)#**	**-64.8% (-74.7% to -50.7%)#**	**-53.9% (-67.0% to -36.8%)#**	**-29.4% (-40.5% to -17.0%)#**	**-48.4% (-53.7% to -42.7%)#**	**-52.1% (-57.7% to -45.7%)#**	**-67.0% (-76.7% to -54.5%)#**	**-63.6% (-73.4% to -50.9%)#**	**-57.2% (-64.8% to -47.8%)#**	**-51.2% (-56.5% to -45.5%)#**
	Death #, 1990	90 (78 to 106)	36 (29 to 45)	98 (88 to 109)	89 (75 to 103)	60 (58 to 63)	51 (50 to 51)	220 (217 to 222)	225 (208 to 244)	314 (299 to 329)	645 (626 to 663)	270 (267 to 274)
	Death #, 2000	64 (55 to 74)	28 (22 to 35)	82 (74 to 92)	72 (61 to 83)	29 (28 to 30)	34 (33 to 35)	119 (118 to 120)	174 (158 to 192)	246 (233 to 261)	429 (413 to 445)	153 (151 to 155)
Chiba	Death #, 2017	39 (28 to 55)	14 (10 to 19)	41 (31 to 54)	38 (31 to 45)	19 (17 to 21)	22 (20 to 24)	62 (55 to 70)	94 (70 to 127)	132 (101 to 172)	235 (197 to 280)	84 (76 to 93)
	% change 1990-2017	**-56.3% (-70.5% to -35.9%)#**	**-62.1% (-75.0% to -43.4%)#**	**-58.2% (-69.3% to -43.9%)#**	**-57.8% (-66.4% to -47.4%)#**	**-68.9% (-72.6% to -64.7%)#**	**-56.9% (-60.5% to -53.0%)#**	**-71.6% (-74.8% to -68.0%)#**	**-58.1% (-70.0% to -43.3%)#**	**-58.0% (-68.2% to -45.2%)#**	**-63.6% (-69.3% to -56.5%)#**	**-68.9% (-72.0% to -65.3%)#**
	% change 2000-2017	**-38.8% (-57.4% to -11.6%)#**	**-50.0% (-67.1% to -25.1%)#**	**-50.1% (-63.2% to -33.6%)#**	**-47.7% (-58.7% to -33.9%)#**	**-35.9% (-43.5% to -26.9%)#**	**-36.3% (-42.0% to -30.3%)#**	**-47.5% (-53.5% to -41.0%)#**	**-45.9% (-61.0% to -25.7%)#**	**-46.4% (-59.1% to -29.8%)#**	**-45.2% (-54.2% to -34.3%)#**	**-45.0% (-50.5% to -39.1%)#**
	Death #, 1990	30 (25 to 36)	10 (8 to 13)	29 (26 to 32)	28 (24 to 33)	16 (16 to 17)	15 (15 to 16)	46 (46 to 47)	69 (62 to 76)	97 (90 to 105)	175 (166 to 184)	62 (61 to 63)
	Death #, 2000	14 (12 to 16)	6 (4 to 7)	19 (17 to 20)	15 (12 to 18)	9 (8 to 10)	8 (8 to 9)	31 (30 to 31)	38 (35 to 42)	54 (50 to 58)	102 (97 to 107)	39 (39 to 40)
Ehime	Death #, 2017	6 (4 to 8)	2 (2 to 3)	6 (5 to 8)	6 (5 to 8)	7 (6 to 8)	5 (5 to 6)	16 (14 to 18)	14 (10 to 20)	20 (15 to 27)	48 (41 to 58)	21 (19 to 24)
	% change 1990-2017	**-81.4% (-88.1% to -72.3%)#**	**-76.0% (-85.3% to -62.8%)#**	**-78.2% (-84.0% to -70.8%)#**	**-77.9% (-83.8% to -70.8%)#**	**-59.1% (-65.4% to -52.5%)#**	**-64.4% (-67.9% to -60.5%)#**	**-66.0% (-70.2% to -61.1%)#**	**-79.3% (-85.7% to -71.5%)#**	**-78.9% (-84.9% to -72.0%)#**	**-72.3% (-77.0% to -67.0%)#**	**-65.6% (-69.5% to -61.3%)#**
	% change 2000-2017	**-61.0% (-73.5% to -44.4%)#**	**-57.5% (-72.6% to -36.0%)#**	**-66.3% (-75.5% to -53.9%)#**	**-58.9% (-70.7% to -43.2%)#**	**-25.7% (-37.3% to -13.0%)#**	**-35.9% (-42.4% to -28.6%)#**	**-48.9% (-55.2% to -41.7%)#**	**-63.0% (-73.8% to -49.1%)#**	**-61.9% (-72.4% to -48.7%)#**	**-52.6% (-60.4% to -43.4%)#**	**-46.1% (-52.2% to -39.1%)#**
	Death #, 1990	16 (13 to 20)	6 (5 to 8)	14 (13 to 16)	15 (12 to 17)	10 (10 to 11)	9 (9 to 10)	26 (25 to 26)	37 (33 to 42)	52 (48 to 57)	98 (92 to 104)	35 (35 to 36)
	Death #, 2000	10 (9 to 12)	5 (4 to 6)	11 (10 to 12)	8 (6 to 10)	5 (5 to 6)	8 (7 to 8)	16 (16 to 17)	26 (23 to 28)	33 (31 to 36)	63 (59 to 66)	24 (23 to 24)
Fukui	Death #, 2017	4 (2 to 5)	1 (1 to 2)	4 (3 to 5)	4 (3 to 5)	4 (3 to 4)	3 (2 to 3)	6 (6 to 7)	9 (6 to 12)	13 (9 to 17)	25 (21 to 31)	9 (8 to 10)
	% change 1990-2017	**-77.7% (-85.4% to -66.6%)#**	**-77.9% (-86.3% to -64.9%)#**	**-73.1% (-80.8% to -62.8%)#**	**-75.3% (-82.2% to -67.1%)#**	**-64.8% (-70.1% to -58.4%)#**	**-72.2% (-74.8% to -69.2%)#**	**-75.3% (-77.8% to -72.5%)#**	**-76.0% (-83.4% to -66.2%)#**	**-75.8% (-82.5% to -67.2%)#**	**-74.1% (-78.7% to -68.6%)#**	**-74.4% (-76.8% to -71.7%)#**
	% change 2000-2017	**-64.8% (-75.7% to -49.1%)#**	**-69.3% (-80.5% to -54.4%)#**	**-63.8% (-74.1% to -50.3%)#**	**-53.0% (-66.9% to -33.9%)#**	**-31.6% (-42.3% to -19.2%)#**	**-66.1% (-69.5% to -62.5%)#**	**-60.5% (-64.6% to -55.8%)#**	**-65.2% (-75.5% to -50.9%)#**	**-62.4% (-72.8% to -48.7%)#**	**-59.7% (-67.2% to -50.9%)#**	**-62.3% (-65.9% to -58.2%)#**
	Death #, 1990	93 (81 to 107)	39 (31 to 49)	101 (90 to 111)	99 (84 to 116)	55 (53 to 57)	49 (49 to 50)	167 (165 to 169)	232 (215 to 250)	332 (316 to 348)	603 (584 to 622)	216 (214 to 219)
	Death #, 2000	64 (54 to 74)	23 (19 to 29)	70 (62 to 79)	59 (50 to 68)	36 (35 to 38)	32 (31 to 33)	110 (108 to 111)	157 (140 to 174)	217 (203 to 229)	395 (379 to 411)	142 (140 to 144)
Fukuoka	Death #, 2017	34 (24 to 46)	10 (8 to 14)	34 (26 to 45)	33 (27 to 39)	20 (18 to 23)	16 (15 to 17)	43 (38 to 49)	78 (58 to 105)	111 (86 to 144)	190 (160 to 227)	59 (53 to 66)
	% change 1990-2017	**-63.7% (-75.1% to -47.3%)#**	**-73.1% (-82.3% to -59.2%)#**	**-65.9% (-74.9% to -54.0%)#**	**-67.1% (-73.8% to -58.3%)#**	**-62.7% (-67.0% to -57.4%)#**	**-68.0% (-70.7% to -65.0%)#**	**-74.2% (-77.4% to -70.7%)#**	**-66.2% (-75.5% to -54.2%)#**	**-66.5% (-74.5% to -55.8%)#**	**-68.4% (-73.5% to -61.8%)#**	**-72.8% (-75.7% to -69.6%)#**
	% change 2000-2017	**-47.2% (-63.1% to -23.7%)#**	**-55.1% (-70.0% to -33.4%)#**	**-51.2% (-64.3% to -35.3%)#**	**-44.6% (-56.0% to -30.4%)#**	**-43.7% (-50.7% to -35.3%)#**	**-50.8% (-55.0% to -46.0%)#**	**-60.9% (-65.6% to -55.5%)#**	**-50.2% (-64.3% to -32.0%)#**	**-48.6% (-61.0% to -33.1%)#**	**-51.8% (-59.6% to -42.0%)#**	**-58.6% (-63.1% to -53.7%)#**
	Death #, 1990	42 (36 to 49)	16 (13 to 21)	50 (45 to 57)	45 (38 to 52)	31 (30 to 33)	25 (25 to 26)	69 (68 to 70)	108 (100 to 117)	153 (145 to 161)	279 (270 to 289)	94 (93 to 96)
	Death #, 2000	20 (17 to 24)	8 (6 to 11)	30 (26 to 34)	25 (20 to 29)	11 (10 to 11)	18 (17 to 18)	53 (52 to 54)	59 (53 to 65)	84 (77 to 90)	165 (157 to 173)	71 (69 to 72)
Fukushima	Death #, 2017	9 (6 to 13)	3 (2 to 4)	11 (9 to 15)	10 (8 to 13)	6 (5 to 7)	6 (5 to 6)	24 (21 to 27)	24 (17 to 32)	34 (25 to 45)	69 (58 to 82)	29 (26 to 33)
	% change 1990-2017	**-78.2% (-85.6% to -68.4%)#**	**-81.7% (-88.5% to -71.7%)#**	**-77.1% (-83.4% to -68.9%)#**	**-77.7% (-83.6% to -70.4%)#**	**-80.9% (-83.7% to -77.9%)#**	**-78.2% (-80.3% to -75.9%)#**	**-65.8% (-70.0% to -61.3%)#**	**-78.2% (-84.7% to -70.0%)#**	**-78.0% (-84.0% to -70.7%)#**	**-75.4% (-79.4% to -70.4%)#**	**-69.2% (-72.7% to -65.4%)#**
	% change 2000-2017	**-54.8% (-69.1% to -35.1%)#**	**-64.2% (-76.9% to -44.5%)#**	**-61.9% (-72.5% to -47.6%)#**	**-59.9% (-70.9% to -46.2%)#**	**-44.8% (-53.2% to -35.9%)#**	**-68.5% (-71.7% to -65.3%)#**	**-55.4% (-60.9% to -49.4%)#**	**-59.8% (-71.5% to -44.6%)#**	**-59.8% (-70.5% to -46.6%)#**	**-58.4% (-65.3% to -50.1%)#**	**-58.7% (-63.3% to -53.5%)#**
	Death #, 1990	35 (29 to 43)	15 (12 to 19)	34 (31 to 37)	37 (32 to 44)	27 (26 to 29)	23 (22 to 23)	70 (69 to 71)	85 (77 to 93)	122 (114 to 131)	242 (231 to 253)	93 (91 to 94)
	Death #, 2000	24 (20 to 27)	10 (8 to 12)	26 (23 to 29)	24 (20 to 29)	21 (20 to 22)	18 (18 to 19)	40 (39 to 41)	59 (53 to 65)	83 (77 to 90)	162 (154 to 171)	58 (57 to 59)

eTable 2. ENN, LNN, PNN, 1-4, 5-9, 10-14, 15-19, <1, <5, 10-19, and <20 years mortality in 1990, 2000 and 2017 with mean percentage change for all GBD causes.

	Metric	Early Neonatal	Late Neonatal	Post Neonatal	1 to 4	5 to 9	10 to 14	15 to 19	<1 year	Under 5	<20 years	10 to 19
Gifu	Death #, 2017	12 (8 to 17)	4 (3 to 6)	12 (9 to 15)	12 (9 to 15)	8 (7 to 10)	7 (6 to 8)	24 (21 to 27)	28 (20 to 38)	40 (30 to 53)	79 (66 to 95)	31 (28 to 35)
	% change 1990-2017	**-65.7% (-77.9% to -48.9%)#**	**-73.9% (-83.5% to -60.6%)#**	**-66.0% (-75.0% to -54.1%)#**	**-67.5% (-76.0% to -57.2%)#**	**-69.3% (-73.7% to -64.4%)#**	**-70.1% (-72.9% to -67.1%)#**	**-65.6% (-69.8% to -61.2%)#**	**-67.3% (-77.3% to -54.9%)#**	**-67.3% (-76.5% to -56.5%)#**	**-67.3% (-73.1% to -60.9%)#**	**-66.7% (-70.3% to -62.7%)#**
	% change 2000-2017	**-48.3% (-64.5% to -26.0%)#**	**-58.8% (-73.2% to -38.5%)#**	**-55.0% (-67.4% to -38.7%)#**	**-50.4% (-63.8% to -33.9%)#**	**-60.2% (-66.2% to -53.4%)#**	**-62.5% (-66.3% to -58.7%)#**	**-39.8% (-46.8% to -31.6%)#**	**-52.9% (-66.4% to -34.7%)#**	**-52.2% (-64.8% to -35.2%)#**	**-51.4% (-59.8% to -41.2%)#**	**-46.9% (-52.6% to -40.2%)#**
Gunma	Death #, 1990	38 (32 to 46)	12 (10 to 15)	35 (32 to 38)	33 (28 to 39)	18 (18 to 19)	21 (21 to 22)	74 (72 to 75)	85 (78 to 94)	119 (111 to 127)	232 (222 to 242)	95 (93 to 97)
	Death #, 2000	25 (22 to 30)	8 (6 to 9)	27 (24 to 30)	21 (17 to 25)	10 (9 to 11)	11 (11 to 12)	47 (46 to 48)	60 (54 to 67)	81 (74 to 89)	150 (142 to 159)	59 (57 to 60)
	Death #, 2017	11 (7 to 15)	3 (2 to 4)	10 (7 to 13)	10 (8 to 12)	6 (5 to 7)	8 (8 to 9)	24 (21 to 27)	23 (17 to 32)	33 (24 to 45)	72 (60 to 85)	32 (29 to 37)
	% change 1990-2017	**-71.5% (-81.7% to -57.8%)#**	**-76.5% (-85.3% to -64.3%)#**	**-72.6% (-80.0% to -62.9%)#**	**-70.5% (-78.3% to -61.2%)#**	**-66.8% (-71.8% to -61.5%)#**	**-60.2% (-64.2% to -56.0%)#**	**-67.6% (-72.0% to -62.8%)#**	**-72.7% (-81.2% to -62.4%)#**	**-72.0% (-79.9% to -62.8%)#**	**-69.1% (-74.2% to -63.2%)#**	**-65.9% (-69.9% to -61.4%)#**
	% change 2000-2017	**-57.2% (-71.1% to -37.9%)#**	**-62.9% (-75.2% to -45.5%)#**	**-64.6% (-74.4% to -51.9%)#**	**-53.5% (-66.5% to -37.2%)#**	**-39.5% (-48.3% to -29.5%)#**	**-24.4% (-32.1% to -16.8%)#**	**-49.5% (-56.2% to -42.2%)#**	**-61.3% (-72.4% to -46.1%)#**	**-59.3% (-70.0% to -44.8%)#**	**-52.3% (-60.2% to -42.8%)#**	**-44.7% (-51.0% to -37.7%)#**
Hiroshima	Death #, 1990	49 (42 to 59)	18 (14 to 22)	59 (53 to 65)	53 (45 to 62)	33 (32 to 35)	29 (28 to 30)	102 (100 to 103)	127 (116 to 138)	179 (168 to 191)	343 (329 to 358)	131 (129 to 133)
	Death #, 2000	26 (22 to 31)	10 (8 to 13)	38 (34 to 42)	31 (26 to 36)	20 (19 to 21)	19 (19 to 20)	58 (57 to 59)	75 (67 to 83)	105 (98 to 113)	203 (193 to 212)	77 (76 to 79)
	Death #, 2017	15 (10 to 21)	5 (4 to 7)	17 (12 to 22)	15 (12 to 19)	7 (6 to 8)	9 (8 to 10)	28 (25 to 32)	37 (26 to 50)	52 (38 to 69)	97 (81 to 116)	38 (34 to 42)
	% change 1990-2017	**-70.2% (-81.0% to -56.0%)#**	**-71.6% (-82.3% to -57.4%)#**	**-71.6% (-79.1% to -61.5%)#**	**-71.2% (-78.8% to -62.1%)#**	**-77.8% (-80.8% to -74.2%)#**	**-68.2% (-71.2% to -64.9%)#**	**-72.2% (-75.4% to -68.7%)#**	**-71.0% (-79.9% to -60.2%)#**	**-71.1% (-79.0% to -61.3%)#**	**-71.8% (-76.7% to -65.9%)#**	**-71.3% (-74.2% to -68.0%)#**
	% change 2000-2017	**-44.0% (-62.3% to -18.1%)#**	**-50.5% (-67.6% to -27.3%)#**	**-55.9% (-68.4% to -39.9%)#**	**-50.4% (-63.3% to -34.5%)#**	**-63.2% (-68.4% to -57.3%)#**	**-52.4% (-57.0% to -47.4%)#**	**-51.1% (-56.8% to -45.0%)#**	**-51.0% (-65.5% to -31.8%)#**	**-50.8% (-64.1% to -34.2%)#**	**-52.3% (-60.7% to -42.3%)#**	**-51.5% (-56.5% to -45.8%)#**
Hokkaido	Death #, 1990	113 (97 to 130)	34 (27 to 43)	111 (98 to 123)	121 (101 to 141)	81 (78 to 84)	61 (59 to 62)	212 (210 to 214)	258 (240 to 276)	378 (360 to 398)	731 (709 to 756)	273 (269 to 276)
	Death #, 2000	58 (50 to 67)	22 (17 to 27)	67 (59 to 75)	68 (56 to 79)	39 (37 to 41)	36 (35 to 37)	135 (133 to 136)	146 (133 to 161)	214 (201 to 226)	424 (408 to 438)	171 (169 to 173)
	Death #, 2017	29 (21 to 39)	9 (6 to 12)	27 (20 to 35)	26 (21 to 30)	15 (13 to 17)	17 (16 to 19)	51 (45 to 57)	65 (48 to 87)	90 (69 to 118)	173 (147 to 205)	68 (61 to 75)
	% change 1990-2017	**-74.6% (-82.5% to -63.2%)#**	**-74.0% (-82.9% to -60.2%)#**	**-75.5% (-82.0% to -66.9%)#**	**-78.8% (-83.4% to -73.4%)#**	**-81.6% (-83.8% to -78.9%)#**	**-71.7% (-74.0% to -69.0%)#**	**-76.0% (-78.8% to -73.1%)#**	**-74.9% (-81.8% to -65.5%)#**	**-76.2% (-81.8% to -68.7%)#**	**-76.3% (-79.8% to -72.0%)#**	**-75.0% (-77.6% to -72.2%)#**
	% change 2000-2017	**-50.5% (-64.8% to -29.4%)#**	**-58.8% (-72.6% to -38.4%)#**	**-59.3% (-70.1% to -45.4%)#**	**-62.3% (-70.2% to -52.1%)#**	**-62.0% (-66.4% to -56.9%)#**	**-52.6% (-56.7% to -48.4%)#**	**-62.2% (-66.6% to -57.5%)#**	**-55.7% (-67.7% to -39.3%)#**	**-57.8% (-67.6% to -45.2%)#**	**-59.1% (-65.1% to -51.9%)#**	**-60.2% (-64.3% to -55.8%)#**
Hyogo	Death #, 1990	104 (89 to 120)	41 (31 to 54)	99 (85 to 115)	98 (79 to 119)	61 (59 to 63)	58 (57 to 59)	188 (186 to 190)	245 (223 to 268)	342 (327 to 360)	649 (630 to 671)	246 (243 to 249)
	Death #, 2000	63 (53 to 75)	27 (22 to 32)	73 (64 to 84)	62 (51 to 74)	36 (34 to 38)	35 (34 to 36)	101 (100 to 102)	164 (147 to 180)	226 (212 to 240)	398 (381 to 416)	136 (134 to 138)
	Death #, 2017	24 (17 to 33)	10 (7 to 13)	31 (23 to 40)	30 (25 to 35)	19 (17 to 22)	20 (18 to 22)	53 (46 to 60)	64 (48 to 87)	94 (72 to 122)	185 (158 to 219)	72 (65 to 81)
	% change 1990-2017	**-76.9% (-84.2% to -66.9%)#**	**-76.8% (-85.0% to -64.0%)#**	**-69.0% (-77.3% to -57.2%)#**	**-69.8% (-76.9% to -60.3%)#**	**-68.8% (-72.5% to -64.2%)#**	**-66.0% (-69.1% to -62.8%)#**	**-72.0% (-75.3% to -68.3%)#**	**-73.7% (-80.9% to -63.6%)#**	**-72.5% (-79.0% to -64.4%)#**	**-71.5% (-75.8% to -66.1%)#**	**-70.6% (-73.8% to -67.2%)#**
	% change 2000-2017	**-62.1% (-73.5% to -45.0%)#**	**-64.4% (-76.2% to -46.3%)#**	**-57.9% (-69.5% to -42.3%)#**	**-52.5% (-63.3% to -37.8%)#**	**-47.3% (-54.0% to -39.3%)#**	**-43.8% (-48.7% to -38.2%)#**	**-47.9% (-54.0% to -41.0%)#**	**-60.6% (-71.2% to -46.0%)#**	**-58.4% (-68.2% to -45.2%)#**	**-53.4% (-60.3% to -44.5%)#**	**-46.9% (-52.4% to -40.7%)#**
Ibaraki	Death #, 1990	53 (46 to 61)	17 (14 to 21)	55 (49 to 62)	55 (45 to 65)	36 (34 to 38)	39 (38 to 40)	171 (169 to 174)	125 (116 to 135)	180 (169 to 192)	426 (412 to 441)	210 (207 to 213)
	Death #, 2000	30 (26 to 36)	11 (9 to 13)	40 (36 to 46)	37 (32 to 43)	21 (20 to 22)	20 (19 to 20)	77 (76 to 78)	81 (73 to 91)	119 (111 to 127)	237 (227 to 247)	97 (95 to 98)
	Death #, 2017	19 (13 to 27)	6 (4 to 8)	19 (13 to 25)	15 (12 to 19)	11 (9 to 13)	13 (12 to 15)	37 (32 to 41)	44 (31 to 61)	59 (43 to 78)	120 (99 to 144)	50 (45 to 56)
	% change 1990-2017	**-63.5% (-76.1% to -46.6%)#**	**-64.9% (-78.1% to -45.6%)#**	**-65.8% (-75.7% to -53.0%)#**	**-73.0% (-80.4% to -63.6%)#**	**-70.3% (-75.2% to -64.8%)#**	**-65.3% (-69.0% to -61.4%)#**	**-78.6% (-81.2% to -76.0%)#**	**-64.7% (-75.5% to -50.8%)#**	**-67.2% (-76.3% to -55.3%)#**	**-71.9% (-76.9% to -65.8%)#**	**-76.2% (-78.7% to -73.5%)#**
	% change 2000-2017	**-36.0% (-56.7% to -7.1%)#**	**-43.4% (-63.5% to -16.5%)#**	**-53.5% (-67.5% to -35.9%)#**	**-60.8% (-70.9% to -48.5%)#**	**-49.5% (-58.0% to -40.1%)#**	**-32.4% (-39.8% to -24.6%)#**	**-52.4% (-58.0% to -46.4%)#**	**-45.7% (-62.5% to -23.7%)#**	**-50.4% (-64.5% to -33.5%)#**	**-49.5% (-58.6% to -38.8%)#**	**-48.3% (-53.9% to -42.4%)#**
Ishikawa	Death #, 1990	22 (18 to 26)	7 (5 to 9)	22 (19 to 25)	20 (17 to 23)	13 (12 to 14)	14 (13 to 14)	35 (35 to 36)	51 (45 to 57)	71 (65 to 76)	133 (126 to 140)	49 (48 to 50)
	Death #, 2000	14 (13 to 17)	6 (4 to 8)	17 (15 to 19)	12 (9 to 14)	9 (9 to 10)	7 (7 to 7)	19 (19 to 20)	37 (34 to 41)	49 (45 to 53)	85 (80 to 90)	26 (26 to 27)
	Death #, 2017	6 (4 to 8)	2 (2 to 3)	6 (4 to 7)	6 (4 to 7)	5 (5 to 6)	6 (5 to 6)	11 (10 to 12)	14 (10 to 19)	20 (14 to 26)	42 (35 to 50)	17 (15 to 18)
	% change 1990-2017	**-73.4% (-82.8% to -60.8%)#**	**-65.8% (-78.9% to -46.4%)#**	**-74.8% (-81.8% to -65.0%)#**	**-70.0% (-78.1% to -60.1%)#**	**-59.9% (-66.1% to -52.9%)#**	**-58.8% (-62.9% to -54.5%)#**	**-69.0% (-72.6% to -65.2%)#**	**-72.9% (-81.2% to -62.1%)#**	**-72.1% (-79.8% to -62.3%)#**	**-68.7% (-74.1% to -62.1%)#**	**-66.2% (-69.6% to -62.4%)#**
	% change 2000-2017	**-60.3% (-73.1% to -42.6%)#**	**-58.2% (-73.6% to -35.9%)#**	**-67.5% (-76.7% to -55.0%)#**	**-49.0% (-63.2% to -28.8%)#**	**-43.8% (-52.2% to -34.3%)#**	**-20.8% (-28.7% to -12.6%)#**	**-43.3% (-50.2% to -36.3%)#**	**-63.2% (-74.2% to -48.3%)#**	**-59.9% (-71.1% to -46.1%)#**	**-51.1% (-59.6% to -41.1%)#**	**-37.3% (-43.6% to -30.5%)#**
Iwate	Death #, 1990	22 (19 to 26)	8 (6 to 10)	25 (22 to 28)	29 (23 to 36)	20 (19 to 21)	13 (13 to 14)	46 (46 to 47)	55 (50 to 61)	84 (78 to 92)	164 (155 to 174)	60 (59 to 61)
	Death #, 2000	11 (10 to 13)	4 (3 to 5)	17 (15 to 19)	16 (12 to 20)	9 (8 to 9)	8 (8 to 8)	25 (25 to 26)	33 (29 to 36)	48 (44 to 52)	90 (85 to 95)	33 (33 to 34)
	Death #, 2017	7 (5 to 10)	3 (2 to 4)	7 (5 to 9)	8 (6 to 10)	5 (5 to 6)	4 (3 to 4)	13 (11 to 15)	16 (12 to 22)	24 (18 to 32)	46 (39 to 56)	17 (15 to 19)
	% change 1990-2017	**-68.5% (-79.0% to -53.8%)#**	**-68.7% (-79.7% to -51.0%)#**	**-73.1% (-80.3% to -63.3%)#**	**-72.7% (-80.7% to -61.9%)#**	**-73.0% (-77.1% to -68.1%)#**	**-71.6% (-74.5% to -68.5%)#**	**-71.9% (-75.2% to -68.2%)#**	**-70.6% (-79.0% to -59.6%)#**	**-71.3% (-79.4% to -61.4%)#**	**-71.7% (-76.8% to -65.6%)#**	**-71.8% (-74.9% to -68.5%)#**
	% change 2000-2017	**-38.1% (-58.3% to -11.0%)#**	**-40.6% (-62.5% to -6.7%)#**	**-60.2% (-71.2% to -44.7%)#**	**-49.4% (-64.9% to -27.1%)#**	**-38.8% (-48.2% to -28.2%)#**	**-52.0% (-56.8% to -46.9%)#**	**-48.7% (-54.8% to -41.7%)#**	**-49.9% (-64.8% to -30.2%)#**	**-49.8% (-63.1% to -32.5%)#**	**-48.6% (-57.8% to -37.7%)#**	**-49.5% (-54.9% to -43.1%)#**
Kagawa	Death #, 1990	19 (16 to 23)	7 (5 to 9)	17 (15 to 20)	17 (14 to 20)	16 (15 to 17)	11 (11 to 12)	37 (36 to 38)	43 (39 to 49)	60 (55 to 66)	124 (118 to 132)	48 (47 to 49)
	Death #, 2000	10 (9 to 11)	4 (3 to 5)	11 (10 to 12)	10 (8 to 13)	5 (4 to 5)	8 (8 to 8)	26 (26 to 27)	25 (23 to 27)	35 (32 to 38)	74 (70 to 78)	34 (33 to 35)
	Death #, 2017	4 (3 to 6)	2 (1 to 2)	5 (4 to 7)	5 (4 to 6)	3 (3 to 3)	5 (4 to 5)	11 (10 to 13)	12 (8 to 16)	17 (12 to 22)	36 (30 to 43)	16 (14 to 18)
	% change 1990-2017	**-76.7% (-84.9% to -65.6%)#**	**-74.7% (-84.5% to -59.2%)#**	**-68.4% (-77.7% to -56.0%)#**	**-70.3% (-78.5% to -60.4%)#**	**-81.5% (-84.3% to -77.9%)#**	**-58.1% (-62.4% to -53.6%)#**	**-69.1% (-72.9% to -64.9%)#**	**-73.1% (-81.3% to -62.4%)#**	**-72.3% (-79.8% to -62.1%)#**	**-71.3% (-76.1% to -65.4%)#**	**-66.6% (-70.2% to -62.6%)#**
	% change 2000-2017	**-54.2% (-68.5% to -34.7%)#**	**-53.7% (-70.9% to -27.5%)#**	**-51.2% (-64.4% to -33.4%)#**	**-51.1% (-65.7% to -30.9%)#**	**-34.6% (-44.7% to -22.9%)#**	**-42.0% (-47.7% to -35.8%)#**	**-56.4% (-61.7% to -50.7%)#**	**-52.8% (-66.7% to -34.3%)#**	**-52.3% (-65.6% to -35.5%)#**	**-51.5% (-59.7% to -41.9%)#**	**-53.0% (-58.0% to -47.6%)#**
	Death #, 1990	32 (27 to 38)	12 (10 to 15)	37 (34 to 41)	44 (37 to 52)	25 (24 to 26)	23 (22 to 24)	47 (46 to 48)	81 (74 to 89)	125 (117 to 133)	220 (211 to 231)	70 (69 to 71)

eTable 2. ENN, LNN, PNN, 1-4, 5-9, 10-14, 15-19, <1, <5, 10-19, and <20 years mortality in 1990, 2000 and 2017 with mean percentage change for all GBD causes.

	Metric	Early Neonatal	Late Neonatal	Post Neonatal	1 to 4	5 to 9	10 to 14	15 to 19	<1 year	Under 5	<20 years	10 to 19
Kagoshima	Death #, 2000	14 (12 to 16)	7 (5 to 8)	28 (25 to 31)	24 (19 to 29)	11 (11 to 12)	10 (10 to 10)	39 (38 to 39)	48 (44 to 53)	72 (66 to 78)	133 (125 to 140)	49 (48 to 50)
	Death #, 2017	9 (7 to 13)	3 (2 to 4)	12 (9 to 15)	9 (7 to 12)	7 (6 to 8)	6 (5 to 6)	19 (16 to 21)	24 (17 to 33)	33 (25 to 45)	64 (53 to 78)	24 (21 to 27)
	% change 1990-2017	**-70.6% (-80.6% to -57.1%)#**	**-74.9% (-84.3% to -62.3%)#**	**-68.6% (-77.2% to -58.3%)#**	**-78.7% (-84.3% to -72.0%)#**	**-73.7% (-77.5% to -69.6%)#**	**-75.5% (-77.8% to -72.9%)#**	**-60.6% (-65.7% to -55.1%)#**	**-70.3% (-79.0% to -59.5%)#**	**-73.2% (-80.4% to -64.3%)#**	**-70.8% (-76.0% to -64.8%)#**	**-65.5% (-69.5% to -61.2%)#**
	% change 2000-2017	**-32.9% (-53.6% to -4.7%)#**	**-53.8% (-69.2% to -32.9%)#**	**-57.9% (-69.7% to -43.6%)#**	**-61.2% (-71.8% to -47.1%)#**	**-42.1% (-50.2% to -32.6%)#**	**-44.4% (-49.6% to -38.3%)#**	**-52.1% (-58.3% to -45.4%)#**	**-50.1% (-64.7% to -31.6%)#**	**-53.8% (-65.8% to -38.3%)#**	**-51.6% (-60.0% to -41.4%)#**	**-50.5% (-56.0% to -44.4%)#**
Kanagawa	Death #, 1990	161 (139 to 187)	58 (46 to 71)	148 (133 to 164)	127 (107 to 148)	76 (73 to 78)	74 (73 to 75)	302 (300 to 305)	366 (341 to 393)	493 (472 to 514)	945 (921 to 970)	376 (372 to 380)
	Death #, 2000	113 (95 to 131)	45 (36 to 56)	122 (108 to 137)	94 (81 to 108)	35 (34 to 37)	46 (45 to 47)	122 (120 to 123)	280 (252 to 310)	374 (352 to 397)	577 (552 to 603)	168 (165 to 170)
	Death #, 2017	58 (42 to 80)	19 (14 to 27)	54 (40 to 71)	50 (41 to 59)	26 (23 to 30)	30 (27 to 32)	79 (70 to 89)	131 (96 to 177)	181 (138 to 236)	316 (266 to 377)	109 (98 to 120)
	% change 1990-2017	**-63.9% (-75.4% to -47.3%)#**	**-66.7% (-77.7% to -49.6%)#**	**-63.4% (-73.0% to -50.7%)#**	**-60.9% (-68.8% to -50.7%)#**	**-65.5% (-69.4% to -60.8%)#**	**-60.0% (-63.3% to -56.5%)#**	**-73.8% (-76.8% to -70.7%)#**	**-64.1% (-74.1% to -51.0%)#**	**-63.3% (-72.3% to -51.9%)#**	**-66.6% (-71.9% to -59.9%)#**	**-71.1% (-74.0% to -67.9%)#**
	% change 2000-2017	**-48.5% (-64.2% to -25.6%)#**	**-57.5% (-71.8% to -37.1%)#**	**-55.6% (-67.9% to -40.7%)#**	**-47.5% (-58.1% to -34.2%)#**	**-25.8% (-35.1% to -15.1%)#**	**-35.5% (-40.8% to -29.5%)#**	**-35.0% (-42.5% to -26.9%)#**	**-53.1% (-66.3% to -35.3%)#**	**-51.7% (-63.6% to -36.0%)#**	**-45.3% (-54.2% to -34.0%)#**	**-35.1% (-41.6% to -28.1%)#**
Kochi	Death #, 1990	18 (15 to 22)	6 (5 to 8)	19 (17 to 22)	13 (11 to 15)	11 (11 to 12)	9 (8 to 9)	27 (27 to 28)	43 (38 to 49)	56 (52 to 61)	103 (98 to 110)	36 (35 to 37)
	Death #, 2000	9 (8 to 10)	4 (3 to 5)	11 (10 to 13)	7 (5 to 8)	7 (6 to 7)	7 (7 to 8)	18 (18 to 19)	24 (22 to 27)	31 (29 to 34)	63 (60 to 67)	26 (25 to 26)
	Death #, 2017	4 (3 to 5)	2 (1 to 2)	4 (3 to 5)	5 (4 to 6)	3 (2 to 3)	3 (3 to 3)	7 (6 to 8)	9 (7 to 13)	14 (10 to 19)	27 (23 to 33)	10 (9 to 11)
	% change 1990-2017	**-78.2% (-85.8% to -67.3%)#**	**-74.4% (-84.6% to -59.8%)#**	**-79.2% (-85.1% to -71.7%)#**	**-62.4% (-72.3% to -50.3%)#**	**-74.6% (-78.5% to -70.4%)#**	**-66.1% (-69.6% to -62.7%)#**	**-73.3% (-76.4% to -69.9%)#**	**-78.1% (-85.0% to -69.5%)#**	**-74.6% (-81.5% to -66.0%)#**	**-73.5% (-78.3% to -68.0%)#**	**-71.6% (-74.5% to -68.3%)#**
	% change 2000-2017	**-57.4% (-70.5% to -39.3%)#**	**-59.3% (-73.7% to -39.0%)#**	**-64.7% (-74.7% to -52.1%)#**	**-30.9% (-51.2% to -3.2%)#**	**-55.8% (-62.6% to -48.0%)#**	**-60.6% (-64.6% to -56.5%)#**	**-59.8% (-64.5% to -54.9%)#**	**-61.1% (-72.8% to -45.6%)#**	**-54.5% (-66.8% to -38.5%)#**	**-56.8% (-64.5% to -47.7%)#**	**-60.0% (-64.2% to -55.5%)#**
Kumamoto	Death #, 1990	36 (32 to 41)	12 (10 to 15)	37 (32 to 42)	41 (33 to 49)	26 (25 to 27)	21 (20 to 21)	50 (49 to 51)	85 (79 to 90)	126 (117 to 135)	222 (211 to 233)	71 (69 to 72)
	Death #, 2000	20 (18 to 24)	8 (6 to 10)	22 (20 to 25)	19 (16 to 23)	12 (11 to 12)	13 (13 to 14)	35 (35 to 36)	51 (46 to 55)	70 (65 to 75)	130 (124 to 136)	48 (48 to 49)
	Death #, 2017	8 (5 to 11)	3 (2 to 5)	9 (7 to 12)	11 (8 to 13)	6 (6 to 7)	7 (7 to 8)	16 (15 to 18)	20 (15 to 27)	31 (23 to 41)	61 (51 to 73)	24 (21 to 26)
	% change 1990-2017	**-78.6% (-85.9% to -69.2%)#**	**-73.0% (-82.4% to -60.5%)#**	**-74.5% (-81.3% to -64.6%)#**	**-74.3% (-81.2% to -65.7%)#**	**-75.5% (-78.8% to -71.7%)#**	**-65.5% (-68.8% to -62.0%)#**	**-67.2% (-70.8% to -63.1%)#**	**-76.0% (-82.9% to -67.4%)#**	**-75.5% (-81.7% to -67.2%)#**	**-72.7% (-77.2% to -67.1%)#**	**-66.7% (-70.0% to -62.9%)#**
	% change 2000-2017	**-61.9% (-73.9% to -45.8%)#**	**-58.3% (-72.7% to -36.9%)#**	**-58.5% (-69.7% to -43.3%)#**	**-46.1% (-60.9% to -27.3%)#**	**-45.1% (-52.8% to -36.5%)#**	**-45.1% (-50.2% to -39.5%)#**	**-53.8% (-59.0% to -47.9%)#**	**-59.8% (-71.0% to -44.9%)#**	**-56.0% (-67.2% to -41.8%)#**	**-53.4% (-61.2% to -44.0%)#**	**-51.5% (-56.3% to -46.0%)#**
Kyoto	Death #, 1990	55 (48 to 64)	17 (14 to 21)	48 (42 to 54)	41 (34 to 49)	31 (30 to 32)	22 (21 to 23)	100 (99 to 102)	120 (112 to 129)	161 (153 to 172)	314 (303 to 327)	122 (120 to 124)
	Death #, 2000	31 (27 to 36)	13 (10 to 16)	34 (31 to 38)	28 (23 to 32)	16 (16 to 17)	13 (13 to 14)	50 (49 to 51)	78 (70 to 87)	106 (98 to 115)	186 (176 to 196)	63 (62 to 64)
	Death #, 2017	17 (11 to 23)	5 (4 to 8)	15 (11 to 20)	15 (12 to 19)	9 (8 to 11)	8 (7 to 9)	20 (18 to 23)	37 (27 to 51)	52 (39 to 70)	90 (74 to 110)	28 (25 to 31)
	% change 1990-2017	**-70.0% (-80.3% to -56.7%)#**	**-67.8% (-79.3% to -51.4%)#**	**-68.5% (-76.9% to -57.0%)#**	**-62.7% (-72.7% to -49.8%)#**	**-70.2% (-74.5% to -65.5%)#**	**-63.0% (-66.6% to -59.1%)#**	**-80.0% (-82.4% to -77.2%)#**	**-69.1% (-78.2% to -57.2%)#**	**-67.5% (-76.4% to -56.3%)#**	**-71.4% (-76.7% to -64.7%)#**	**-76.9% (-79.3% to -74.2%)#**
	% change 2000-2017	**-47.2% (-64.2% to -23.9%)#**	**-56.5% (-72.3% to -34.0%)#**	**-56.1% (-68.5% to -40.2%)#**	**-44.5% (-59.6% to -26.1%)#**	**-44.2% (-52.3% to -34.9%)#**	**-38.1% (-44.4% to -32.0%)#**	**-59.8% (-64.9% to -54.3%)#**	**-52.6% (-66.6% to -33.6%)#**	**-50.5% (-64.0% to -33.4%)#**	**-51.6% (-60.9% to -40.6%)#**	**-55.3% (-60.1% to -50.2%)#**
Mie	Death #, 1990	42 (36 to 47)	13 (10 to 16)	32 (28 to 36)	29 (24 to 36)	22 (20 to 23)	18 (17 to 18)	78 (76 to 79)	86 (81 to 92)	115 (107 to 123)	232 (222 to 242)	95 (94 to 97)
	Death #, 2000	22 (19 to 26)	8 (7 to 10)	27 (24 to 29)	20 (16 to 24)	11 (10 to 12)	10 (10 to 10)	46 (45 to 47)	57 (53 to 62)	77 (71 to 83)	145 (137 to 152)	56 (55 to 58)
	Death #, 2017	11 (7 to 15)	4 (3 to 5)	12 (9 to 16)	11 (8 to 13)	6 (5 to 7)	6 (5 to 7)	20 (17 to 22)	27 (19 to 36)	37 (27 to 50)	69 (56 to 83)	26 (23 to 29)
	% change 1990-2017	**-74.5% (-83.2% to -63.3%)#**	**-70.4% (-81.2% to -55.6%)#**	**-61.9% (-72.2% to -46.7%)#**	**-63.9% (-74.1% to -51.1%)#**	**-73.2% (-77.1% to -68.7%)#**	**-66.0% (-69.4% to -62.5%)#**	**-74.6% (-77.6% to -71.2%)#**	**-69.2% (-78.1% to -58.0%)#**	**-67.9% (-76.3% to -56.5%)#**	**-70.5% (-75.6% to -63.9%)#**	**-73.0% (-75.9% to -69.9%)#**
	% change 2000-2017	**-52.9% (-67.9% to -31.6%)#**	**-55.1% (-70.2% to -33.9%)#**	**-54.4% (-66.6% to -38.3%)#**	**-46.4% (-60.9% to -26.2%)#**	**-48.0% (-55.6% to -39.4%)#**	**-41.3% (-46.8% to -35.2%)#**	**-57.3% (-62.3% to -51.6%)#**	**-53.9% (-67.0% to -36.4%)#**	**-52.0% (-64.6% to -35.0%)#**	**-52.6% (-61.0% to -42.3%)#**	**-54.4% (-59.3% to -49.2%)#**
Miyagi	Death #, 1990	45 (38 to 53)	14 (11 to 18)	41 (36 to 46)	47 (39 to 57)	25 (24 to 27)	24 (24 to 25)	77 (76 to 79)	101 (91 to 111)	148 (139 to 157)	275 (264 to 287)	102 (100 to 103)
	Death #, 2000	25 (21 to 30)	9 (7 to 12)	29 (24 to 33)	26 (20 to 33)	17 (16 to 19)	19 (18 to 19)	56 (55 to 57)	63 (56 to 70)	88 (81 to 97)	180 (171 to 191)	75 (73 to 76)
	Death #, 2017	14 (10 to 19)	5 (3 to 7)	14 (11 to 19)	16 (12 to 20)	9 (7 to 10)	8 (7 to 9)	22 (19 to 25)	33 (24 to 44)	49 (36 to 64)	87 (72 to 105)	30 (27 to 33)
	% change 1990-2017	**-69.9% (-80.2% to -56.1%)#**	**-66.9% (-78.7% to -48.7%)#**	**-65.4% (-74.4% to -53.6%)#**	**-65.7% (-75.5% to -53.2%)#**	**-66.3% (-71.0% to -61.0%)#**	**-67.0% (-70.2% to -63.7%)#**	**-71.7% (-74.9% to -68.2%)#**	**-67.6% (-77.1% to -55.6%)#**	**-67.0% (-76.0% to -56.5%)#**	**-68.3% (-74.1% to -61.7%)#**	**-70.6% (-73.5% to -67.3%)#**
	% change 2000-2017	**-45.1% (-62.1% to -20.9%)#**	**-47.3% (-65.9% to -18.4%)#**	**-50.5% (-64.5% to -31.5%)#**	**-37.5% (-55.7% to -12.5%)#**	**-50.7% (-58.1% to -43.1%)#**	**-57.0% (-61.1% to -52.7%)#**	**-61.0% (-65.4% to -56.0%)#**	**-47.9% (-63.0% to -27.2%)#**	**-44.9% (-59.2% to -27.0%)#**	**-51.7% (-60.3% to -41.9%)#**	**-60.0% (-64.1% to -55.6%)#**
Miyazaki	Death #, 1990	27 (22 to 33)	8 (7 to 11)	26 (23 to 28)	25 (21 to 30)	18 (17 to 19)	11 (11 to 11)	37 (36 to 38)	61 (55 to 68)	86 (80 to 93)	152 (144 to 161)	48 (47 to 49)
	Death #, 2000	12 (11 to 14)	5 (4 to 6)	16 (14 to 18)	12 (9 to 14)	8 (7 to 8)	9 (8 to 9)	29 (29 to 30)	34 (30 to 37)	45 (41 to 50)	91 (86 to 96)	38 (37 to 39)
	Death #, 2017	5 (4 to 8)	2 (2 to 3)	8 (6 to 10)	5 (4 to 6)	4 (4 to 5)	7 (6 to 8)	11 (10 to 13)	15 (11 to 21)	20 (15 to 27)	43 (36 to 51)	18 (16 to 20)
	% change 1990-2017	**-80.2% (-87.3% to -70.5%)#**	**-71.4% (-82.1% to -56.5%)#**	**-69.9% (-78.3% to -58.9%)#**	**-81.0% (-86.2% to -74.6%)#**	**-75.8% (-79.5% to -71.8%)#**	**-37.4% (-43.7% to -30.9%)#**	**-69.4% (-73.4% to -65.1%)#**	**-74.7% (-82.5% to -65.3%)#**	**-76.5% (-83.4% to -68.4%)#**	**-71.9% (-76.9% to -66.2%)#**	**-62.0% (-66.2% to -57.5%)#**
	% change 2000-2017	**-57.4% (-70.9% to -38.4%)#**	**-51.6% (-68.8% to -27.2%)#**	**-52.9% (-66.5% to -34.7%)#**	**-59.0% (-71.0% to -42.2%)#**	**-41.7% (-51.3% to -31.4%)#**	**-19.6% (-27.9% to -10.6%)#**	**-61.5% (-66.5% to -55.8%)#**	**-54.4% (-68.2% to -36.1%)#**	**-55.5% (-68.2% to -39.4%)#**	**-52.9% (-61.2% to -42.7%)#**	**-51.9% (-57.0% to -45.9%)#**
Nagano	Death #, 1990	39 (34 to 44)	13 (10 to 16)	35 (31 to 40)	33 (27 to 40)	22 (21 to 23)	20 (19 to 20)	60 (59 to 61)	87 (81 to 92)	120 (111 to 128)	221 (211 to 231)	79 (78 to 81)
	Death #, 2000	16 (14 to 19)	6 (5 to 8)	22 (20 to 24)	22 (17 to 26)	18 (17 to 19)	15 (14 to 15)	44 (43 to 45)	45 (41 to 48)	66 (62 to 71)	143 (136 to 150)	59 (58 to 60)
	Death #, 2017	8 (6 to 12)	3 (2 to 4)	9 (6 to 11)	10 (8 to 13)	8 (7 to 9)	10 (9 to 10)	24 (21 to 27)	20 (14 to 27)	30 (22 to 40)	71 (60 to 84)	33 (30 to 37)
	% change 1990-2017	**-78.0% (-85.2% to -68.4%)#**	**-79.7% (-87.1% to -69.3%)#**	**-75.6% (-82.1% to -66.0%)#**	**-69.2% (-77.6% to -58.6%)#**	**-64.5% (-69.8% to -58.5%)#**	**-51.9% (-56.6% to -47.0%)#**	**-60.0% (-64.5% to -54.9%)#**	**-77.3% (-83.7% to -69.0%)#**	**-75.1% (-81.5% to -66.6%)#**	**-67.9% (-73.0% to -61.9%)#**	**-58.0% (-62.3% to -53.3%)#**
	% change 2000-2017	**-48.6% (-64.8% to -26.7%)#**	**-59.9% (-73.2% to -40.4%)#**	**-60.2% (-71.1% to -46.1%)#**	**-53.2% (-65.8% to -36.6%)#**	**-56.6% (-63.1% to -49.4%)#**	**-36.0% (-42.4% to -29.4%)#**	**-45.5% (-51.8% to -38.4%)#**	**-55.9% (-68.6% to -39.4%)#**	**-55.0% (-67.0% to -39.8%)#**	**-50.3% (-58.1% to -41.6%)#**	**-43.1% (-48.9% to -36.6%)#**

eTable 2. ENN, LNN, PNN, 1-4, 5-9, 10-14, 15-19, <1, <5, 10-19, and <20 years mortality in 1990, 2000 and 2017 with mean percentage change for all GBD causes.

	Metric	Early Neonatal	Late Neonatal	Post Neonatal	1 to 4	5 to 9	10 to 14	15 to 19	<1 year	Under 5	<20 years	10 to 19
Nagasaki	Death #, 1990	27 (22 to 33)	9 (7 to 12)	30 (26 to 33)	34 (29 to 41)	23 (22 to 24)	18 (17 to 18)	48 (47 to 49)	66 (60 to 73)	100 (93 to 108)	190 (181 to 199)	66 (65 to 67)
	Death #, 2000	16 (14 to 18)	7 (5 to 9)	19 (16 to 21)	18 (15 to 22)	11 (11 to 12)	11 (10 to 11)	31 (30 to 31)	41 (38 to 45)	59 (55 to 64)	112 (106 to 118)	41 (41 to 42)
	Death #, 2017	7 (5 to 10)	3 (2 to 4)	7 (5 to 9)	8 (6 to 10)	4 (4 to 5)	6 (5 to 6)	13 (11 to 14)	17 (12 to 23)	25 (18 to 33)	47 (39 to 57)	18 (17 to 20)
	% change 1990-2017	-74.0% (-83.2% to -61.1%)#	-67.1% (-78.9% to -49.8%)#	-76.5% (-83.1% to -68.2%)#	-77.7% (-83.5% to -70.7%)#	-82.4% (-85.0% to -79.6%)#	-68.6% (-71.5% to -65.4%)#	-73.4% (-76.4% to -70.0%)#	-74.2% (-82.2% to -64.4%)#	-75.3% (-82.3% to -67.4%)#	-75.1% (-79.5% to -69.9%)#	-72.1% (-74.9% to -68.9%)#
	% change 2000-2017	-54.9% (-69.1% to -36.1%)#	-56.7% (-71.9% to -33.2%)#	-62.9% (-73.2% to -48.9%)#	-57.3% (-69.1% to -41.5%)#	-63.5% (-69.1% to -57.5%)#	-47.6% (-52.8% to -42.1%)#	-58.3% (-63.1% to -52.9%)#	-58.8% (-70.7% to -42.8%)#	-58.4% (-69.9% to -44.3%)#	-57.8% (-65.4% to -49.2%)#	-55.5% (-60.1% to -50.4%)#
Nara	Death #, 1990	27 (23 to 32)	8 (6 to 11)	27 (24 to 31)	23 (19 to 28)	13 (12 to 14)	9 (9 to 9)	48 (47 to 49)	63 (57 to 69)	86 (79 to 93)	157 (148 to 165)	57 (56 to 58)
	Death #, 2000	13 (11 to 17)	7 (5 to 8)	19 (16 to 22)	16 (13 to 19)	9 (9 to 10)	9 (8 to 9)	32 (31 to 32)	39 (35 to 43)	55 (50 to 61)	104 (98 to 112)	40 (39 to 41)
	Death #, 2017	6 (4 to 9)	3 (2 to 4)	8 (6 to 11)	8 (6 to 10)	4 (4 to 5)	6 (5 to 6)	13 (12 to 15)	17 (12 to 23)	25 (18 to 33)	48 (40 to 58)	19 (17 to 21)
	% change 1990-2017	-76.4% (-84.4% to -65.0%)#	-69.5% (-81.3% to -51.6%)#	-71.0% (-79.2% to -59.9%)#	-66.5% (-75.8% to -55.1%)#	-66.4% (-71.5% to -60.6%)#	-37.1% (-43.2% to -30.6%)#	-72.2% (-75.4% to -68.4%)#	-73.1% (-81.2% to -62.4%)#	-71.3% (-79.1% to -61.5%)#	-69.2% (-74.5% to -62.9%)#	-66.7% (-70.2% to -62.6%)#
	% change 2000-2017	-52.5% (-68.7% to -28.4%)#	-61.2% (-74.7% to -42.6%)#	-57.8% (-69.9% to -41.4%)#	-51.3% (-65.3% to -32.4%)#	-52.1% (-59.6% to -43.9%)#	-34.4% (-41.0% to -27.4%)#	-57.7% (-62.9% to -51.8%)#	-56.5% (-69.6% to -40.0%)#	-55.0% (-67.5% to -39.5%)#	-53.8% (-62.3% to -44.1%)#	-52.7% (-57.7% to -46.9%)#
Niigata	Death #, 1990	38 (32 to 45)	15 (12 to 18)	38 (34 to 42)	41 (34 to 48)	31 (30 to 33)	28 (27 to 29)	76 (74 to 77)	90 (82 to 99)	131 (122 to 140)	266 (254 to 278)	103 (102 to 105)
	Death #, 2000	24 (20 to 28)	11 (9 to 14)	27 (24 to 31)	25 (21 to 30)	14 (14 to 15)	14 (14 to 15)	47 (46 to 47)	63 (55 to 71)	88 (80 to 97)	163 (154 to 174)	61 (60 to 62)
	Death #, 2017	11 (8 to 16)	3 (2 to 5)	11 (8 to 14)	9 (7 to 11)	6 (6 to 7)	8 (7 to 8)	22 (20 to 25)	25 (18 to 35)	34 (25 to 46)	71 (58 to 84)	30 (27 to 33)
	% change 1990-2017	-70.4% (-80.9% to -56.2%)#	-77.3% (-85.7% to -65.8%)#	-71.2% (-79.2% to -61.2%)#	-77.7% (-83.6% to -70.6%)#	-79.8% (-82.8% to -76.4%)#	-72.7% (-75.5% to -69.9%)#	-70.5% (-74.0% to -66.8%)#	-71.8% (-80.7% to -61.0%)#	-73.7% (-81.2% to -64.6%)#	-73.4% (-78.3% to -68.1%)#	-71.1% (-74.2% to -68.0%)#
	% change 2000-2017	-53.1% (-68.0% to -32.4%)#	-71.1% (-81.2% to -57.6%)#	-60.6% (-71.8% to -46.1%)#	-64.2% (-73.6% to -51.8%)#	-56.0% (-62.5% to -48.7%)#	-45.9% (-51.3% to -40.3%)#	-52.3% (-57.8% to -46.2%)#	-59.7% (-71.7% to -43.3%)#	-61.0% (-71.9% to -47.7%)#	-56.8% (-64.6% to -48.3%)#	-50.8% (-55.9% to -45.1%)#
Oita	Death #, 1990	24 (21 to 29)	7 (5 to 9)	20 (17 to 22)	25 (21 to 29)	19 (18 to 20)	15 (14 to 15)	37 (36 to 37)	51 (46 to 56)	75 (70 to 81)	146 (139 to 154)	51 (50 to 52)
	Death #, 2000	13 (11 to 15)	5 (4 to 6)	12 (11 to 14)	12 (9 to 15)	6 (5 to 6)	7 (6 to 7)	28 (28 to 29)	30 (28 to 33)	42 (39 to 46)	83 (79 to 87)	35 (34 to 36)
	Death #, 2017	7 (5 to 9)	2 (2 to 3)	6 (5 to 8)	6 (4 to 7)	6 (5 to 7)	4 (4 to 5)	12 (11 to 14)	15 (11 to 21)	21 (15 to 28)	43 (36 to 52)	17 (15 to 18)
	% change 1990-2017	-72.8% (-82.4% to -60.4%)#	-65.6% (-79.0% to -46.0%)#	-67.8% (-76.8% to -55.6%)#	-76.5% (-82.9% to -68.9%)#	-69.7% (-74.4% to -64.7%)#	-70.3% (-73.3% to -67.2%)#	-66.7% (-70.9% to -62.4%)#	-69.9% (-79.3% to -58.6%)#	-72.1% (-79.9% to -62.6%)#	-70.2% (-75.7% to -64.3%)#	-67.7% (-71.3% to -63.9%)#
	% change 2000-2017	-47.9% (-64.8% to -24.2%)#	-52.5% (-69.0% to -30.3%)#	-49.4% (-63.4% to -30.8%)#	-51.9% (-65.9% to -32.1%)#	0.7% (-14.8% to 17.6%)	-35.5% (-42.2% to -28.7%)#	-57.0% (-62.3% to -51.2%)#	-49.3% (-63.8% to -29.4%)#	-50.0% (-63.7% to -31.9%)#	-47.7% (-57.0% to -37.0%)#	-52.9% (-58.0% to -47.3%)#
Okayama	Death #, 1990	31 (26 to 37)	10 (7 to 13)	38 (35 to 42)	40 (34 to 47)	28 (26 to 29)	22 (21 to 22)	70 (69 to 72)	79 (72 to 86)	119 (111 to 127)	239 (229 to 249)	92 (91 to 94)
	Death #, 2000	17 (15 to 20)	7 (6 to 9)	29 (27 to 31)	24 (20 to 29)	13 (12 to 13)	12 (12 to 13)	42 (41 to 42)	53 (49 to 58)	78 (72 to 84)	144 (137 to 152)	54 (53 to 55)
	Death #, 2017	8 (6 to 12)	3 (2 to 4)	11 (8 to 14)	10 (8 to 13)	6 (5 to 7)	6 (6 to 7)	16 (14 to 19)	22 (16 to 31)	33 (24 to 44)	62 (51 to 74)	22 (20 to 25)
	% change 1990-2017	-72.6% (-82.1% to -59.7%)#	-66.6% (-79.3% to -47.8%)#	-71.7% (-79.1% to -61.9%)#	-74.0% (-80.8% to -65.8%)#	-77.6% (-80.9% to -74.0%)#	-72.3% (-74.9% to -69.6%)#	-76.6% (-79.4% to -73.3%)#	-71.4% (-79.8% to -60.9%)#	-72.3% (-79.7% to -63.4%)#	-74.2% (-78.6% to -69.1%)#	-75.6% (-78.1% to -72.8%)#
	% change 2000-2017	-50.4% (-65.9% to -28.5%)#	-55.5% (-70.7% to -32.7%)#	-62.7% (-72.9% to -50.1%)#	-56.7% (-68.2% to -41.1%)#	-51.0% (-58.2% to -42.9%)#	-50.8% (-55.3% to -45.8%)#	-60.4% (-65.1% to -55.0%)#	-57.8% (-69.7% to -41.8%)#	-57.5% (-68.4% to -43.0%)#	-57.2% (-64.2% to -48.4%)#	-58.2% (-62.5% to -53.4%)#
Okinawa	Death #, 1990	33 (29 to 38)	14 (11 to 17)	31 (27 to 36)	25 (21 to 31)	21 (20 to 22)	18 (17 to 19)	64 (62 to 65)	78 (73 to 83)	104 (97 to 110)	206 (197 to 215)	82 (80 to 83)
	Death #, 2000	19 (16 to 22)	8 (7 to 10)	24 (20 to 28)	15 (12 to 18)	10 (10 to 11)	7 (7 to 8)	38 (38 to 39)	51 (47 to 56)	66 (61 to 71)	122 (116 to 129)	46 (45 to 47)
	Death #, 2017	10 (7 to 15)	4 (3 to 6)	11 (8 to 14)	8 (6 to 10)	7 (6 to 8)	5 (5 to 6)	19 (17 to 21)	25 (18 to 35)	33 (24 to 44)	65 (53 to 78)	24 (22 to 27)
	% change 1990-2017	-68.8% (-79.1% to -55.2%)#	-69.7% (-80.5% to -54.0%)#	-65.3% (-75.0% to -52.1%)#	-70.2% (-78.6% to -59.5%)#	-65.0% (-70.0% to -59.3%)#	-70.0% (-72.9% to -66.8%)#	-70.0% (-73.6% to -66.1%)#	-67.5% (-77.1% to -55.4%)#	-68.2% (-76.8% to -56.7%)#	-68.6% (-74.0% to -61.9%)#	-70.0% (-73.2% to -66.5%)#
	% change 2000-2017	-45.3% (-62.6% to -20.6%)#	-51.1% (-67.8% to -27.2%)#	-54.2% (-67.9% to -36.1%)#	-48.2% (-62.6% to -27.9%)#	-30.8% (-41.1% to -19.3%)#	-26.9% (-34.1% to -19.2%)#	-50.5% (-56.3% to -44.1%)#	-50.4% (-65.4% to -30.2%)#	-49.9% (-64.2% to -32.2%)#	-47.1% (-56.9% to -35.4%)#	-46.7% (-52.4% to -40.6%)#
Osaka	Death #, 1990	175 (150 to 206)	66 (52 to 80)	185 (165 to 205)	171 (145 to 201)	86 (83 to 89)	83 (82 to 85)	337 (333 to 340)	426 (393 to 461)	597 (565 to 631)	1103 (1064 to 1144)	420 (415 to 425)
	Death #, 2000	99 (84 to 114)	44 (35 to 55)	128 (114 to 142)	107 (90 to 125)	43 (41 to 45)	42 (41 to 43)	153 (152 to 155)	272 (245 to 298)	379 (358 to 398)	617 (593 to 639)	195 (193 to 197)
	Death #, 2017	46 (33 to 64)	16 (11 to 22)	51 (38 to 67)	43 (36 to 51)	28 (25 to 32)	27 (25 to 30)	78 (69 to 88)	113 (83 to 153)	156 (119 to 204)	290 (246 to 345)	105 (95 to 117)
	% change 1990-2017	-73.6% (-82.1% to -61.8%)#	-75.9% (-83.9% to -63.2%)#	-72.3% (-79.4% to -62.5%)#	-74.9% (-80.2% to -68.3%)#	-67.0% (-70.9% to -62.1%)#	-67.2% (-69.9% to -64.2%)#	-76.9% (-79.4% to -73.9%)#	-73.4% (-80.8% to -63.6%)#	-73.8% (-80.2% to -65.6%)#	-73.7% (-77.8% to -68.5%)#	-75.0% (-77.3% to -72.2%)#
	% change 2000-2017	-53.4% (-67.4% to -32.6%)#	-64.2% (-76.3% to -46.7%)#	-60.1% (-71.1% to -46.9%)#	-59.8% (-68.5% to -49.8%)#	-34.0% (-42.0% to -24.6%)#	-34.5% (-40.0% to -28.3%)#	-49.2% (-54.8% to -42.8%)#	-58.3% (-69.6% to -42.6%)#	-58.7% (-68.7% to -46.1%)#	-53.0% (-60.2% to -43.7%)#	-46.0% (-51.4% to -40.1%)#
Saga	Death #, 1990	18 (15 to 22)	7 (5 to 9)	17 (15 to 19)	21 (18 to 24)	11 (11 to 12)	11 (10 to 11)	31 (30 to 31)	41 (37 to 47)	62 (58 to 67)	115 (109 to 121)	41 (41 to 42)
	Death #, 2000	9 (8 to 11)	4 (3 to 5)	11 (10 to 12)	12 (9 to 14)	5 (5 to 5)	5 (5 to 6)	20 (20 to 20)	24 (22 to 26)	36 (32 to 39)	66 (62 to 70)	25 (25 to 26)
	Death #, 2017	3 (2 to 5)	1 (1 to 2)	4 (3 to 6)	4 (3 to 5)	4 (3 to 5)	3 (3 to 4)	9 (8 to 10)	9 (6 to 12)	13 (10 to 18)	29 (24 to 35)	12 (11 to 13)
	% change 1990-2017	-80.7% (-87.6% to -71.3%)#	-81.6% (-88.8% to -71.3%)#	-74.3% (-81.4% to -64.8%)#	-79.9% (-85.0% to -74.0%)#	-65.4% (-71.0% to -59.5%)#	-68.7% (-71.9% to -65.5%)#	-72.1% (-75.5% to -68.2%)#	-78.3% (-85.1% to -69.6%)#	-78.8% (-84.6% to -71.5%)#	-74.7% (-78.9% to -69.6%)#	-71.2% (-74.2% to -67.8%)#
	% change 2000-2017	-61.5% (-73.6% to -44.4%)#	-66.8% (-79.2% to -48.3%)#	-61.3% (-72.0% to -46.8%)#	-64.5% (-74.8% to -50.6%)#	-20.7% (-33.0% to -6.2%)#	-37.3% (-43.4% to -30.4%)#	-57.0% (-62.1% to -51.0%)#	-62.2% (-73.2% to -47.9%)#	-63.0% (-72.8% to -50.1%)#	-55.9% (-63.4% to -47.0%)#	-52.8% (-57.8% to -47.2%)#
Saitama	Death #, 1990	124 (108 to 144)	45 (36 to 55)	122 (109 to 135)	108 (90 to 126)	66 (63 to 68)	66 (65 to 67)	239 (237 to 242)	291 (272 to 313)	399 (381 to 418)	770 (749 to 793)	305 (302 to 309)
	Death #, 2000	77 (66 to 89)	34 (27 to 43)	101 (90 to 113)	82 (69 to 95)	41 (40 to 43)	41 (40 to 42)	124 (123 to 126)	212 (192 to 234)	294 (278 to 312)	501 (482 to 522)	165 (163 to 167)
	Death #, 2017	40 (29 to 55)	13 (10 to 19)	44 (33 to 58)	42 (35 to 50)	24 (21 to 27)	21 (20 to 23)	75 (67 to 85)	97 (72 to 131)	139 (107 to 181)	260 (219 to 308)	97 (87 to 108)
	% change 1990-2017	-68.0% (-78.1% to -53.1%)#	-70.1% (-80.2% to -54.8%)#	-64.0% (-73.5% to -51.6%)#	-61.1% (-69.2% to -51.0%)#	-63.4% (-67.6% to -58.3%)#	-67.7% (-70.4% to -64.6%)#	-68.6% (-72.1% to -64.5%)#	-66.6% (-76.0% to -54.4%)#	-65.1% (-73.5% to -54.5%)#	-66.3% (-71.7% to -60.0%)#	-68.4% (-71.6% to -64.8%)#

eTable 2. ENN, LNN, PNN, 1-4, 5-9, 10-14, 15-19, <1, <5, 10-19, and <20 years mortality in 1990, 2000 and 2017 with mean percentage change for all GBD causes.

	Metric	Early Neonatal	Late Neonatal	Post Neonatal	1 to 4	5 to 9	10 to 14	15 to 19	<1 year	Under 5	<20 years	10 to 19
	% change 2000-2017	**-48.5% (-63.9% to -25.7%)#**	**-60.5% (-73.8% to -41.0%)#**	**-56.4% (-68.0% to -41.6%)#**	**-49.1% (-59.5% to -35.8%)#**	**-41.6% (-48.6% to -33.6%)#**	**-47.9% (-52.5% to -42.9%)#**	**-39.4% (-46.1% to -31.5%)#**	**-54.2% (-67.0% to -37.3%)#**	**-52.8% (-63.9% to -38.1%)#**	**-48.1% (-56.1% to -38.5%)#**	**-41.5% (-47.4% to -34.9%)#**
Shiga	Death #, 1990	26 (23 to 30)	8 (6 to 11)	23 (19 to 27)	20 (17 to 24)	13 (12 to 14)	14 (14 to 15)	50 (50 to 51)	57 (52 to 63)	77 (72 to 84)	155 (148 to 163)	65 (64 to 66)
	Death #, 2000	19 (16 to 21)	7 (5 to 8)	19 (17 to 21)	13 (11 to 16)	9 (9 to 10)	7 (7 to 7)	27 (27 to 28)	45 (40 to 49)	58 (54 to 63)	102 (96 to 107)	34 (34 to 35)
	Death #, 2017	9 (6 to 13)	3 (2 to 4)	8 (6 to 11)	8 (6 to 11)	5 (4 to 6)	5 (4 to 5)	13 (12 to 15)	21 (15 to 29)	29 (21 to 39)	52 (42 to 63)	18 (16 to 20)
	% change 1990-2017	**-64.2% (-76.2% to -48.1%)#**	**-63.5% (-77.9% to -40.6%)#**	**-62.8% (-73.7% to -46.9%)#**	**-59.4% (-70.2% to -46.5%)#**	**-63.0% (-68.7% to -56.7%)#**	**-68.2% (-71.4% to -65.0%)#**	**-73.5% (-76.6% to -70.0%)#**	**-63.5% (-74.4% to -49.5%)#**	**-62.4% (-72.7% to -49.2%)#**	**-66.6% (-72.8% to -59.0%)#**	**-72.3% (-75.2% to -69.0%)#**
	% change 2000-2017	**-49.8% (-65.1% to -27.6%)#**	**-55.8% (-71.0% to -33.6%)#**	**-56.1% (-68.4% to -40.3%)#**	**-38.1% (-55.4% to -14.0%)#**	**-47.2% (-55.0% to -38.1%)#**	**-36.2% (-42.4% to -29.7%)#**	**-51.0% (-56.8% to -44.3%)#**	**-53.4% (-67.2% to -35.3%)#**	**-49.9% (-63.7% to -32.7%)#**	**-49.0% (-58.7% to -37.6%)#**	**-47.9% (-53.4% to -41.6%)#**
Shimane	Death #, 1990	12 (10 to 15)	5 (4 to 7)	15 (13 to 17)	12 (10 to 13)	10 (9 to 11)	11 (11 to 12)	21 (21 to 21)	32 (28 to 36)	44 (40 to 48)	86 (81 to 91)	32 (31 to 33)
	Death #, 2000	6 (6 to 7)	3 (3 to 4)	9 (8 to 9)	7 (5 to 9)	5 (5 to 5)	5 (5 to 5)	15 (15 to 16)	18 (17 to 20)	25 (23 to 28)	51 (48 to 54)	21 (20 to 21)
	Death #, 2017	3 (2 to 4)	1 (1 to 2)	3 (2 to 4)	3 (2 to 4)	3 (2 to 3)	3 (2 to 3)	8 (7 to 9)	7 (5 to 10)	10 (8 to 14)	23 (20 to 28)	10 (9 to 11)
	% change 1990-2017	**-76.1% (-85.0% to -63.5%)#**	**-74.8% (-84.4% to -60.0%)#**	**-77.7% (-84.2% to -69.1%)#**	**-73.8% (-81.0% to -65.3%)#**	**-73.2% (-77.3% to -68.4%)#**	**-77.4% (-79.7% to -75.0%)#**	**-63.8% (-68.2% to -59.2%)#**	**-76.6% (-83.8% to -67.4%)#**	**-75.9% (-82.6% to -67.3%)#**	**-72.8% (-77.4% to -67.4%)#**	**-68.5% (-71.9% to -64.9%)#**
	% change 2000-2017	**-53.9% (-68.4% to -34.2%)#**	**-63.7% (-77.1% to -43.4%)#**	**-62.0% (-72.4% to -48.0%)#**	**-56.2% (-69.7% to -37.2%)#**	**-46.5% (-54.8% to -36.8%)#**	**-50.3% (-55.5% to -45.0%)#**	**-50.8% (-56.6% to -44.2%)#**	**-59.5% (-71.3% to -43.1%)#**	**-58.6% (-70.4% to -43.6%)#**	**-54.2% (-62.3% to -45.0%)#**	**-50.6% (-55.8% to -44.8%)#**
Shizuoka	Death #, 1990	63 (54 to 73)	25 (20 to 31)	70 (63 to 77)	70 (59 to 82)	45 (43 to 47)	39 (38 to 40)	118 (117 to 120)	158 (146 to 171)	228 (216 to 240)	430 (415 to 445)	157 (155 to 160)
	Death #, 2000	37 (32 to 43)	16 (13 to 20)	44 (39 to 49)	41 (34 to 48)	23 (22 to 24)	21 (21 to 22)	73 (73 to 75)	97 (88 to 107)	139 (130 to 148)	256 (246 to 267)	95 (93 to 96)
	Death #, 2017	19 (14 to 26)	7 (5 to 9)	19 (14 to 25)	18 (15 to 22)	12 (10 to 13)	13 (12 to 15)	32 (28 to 36)	45 (33 to 61)	64 (49 to 83)	120 (102 to 143)	45 (40 to 50)
	% change 1990-2017	**-69.6% (-79.3% to -55.6%)#**	**-73.5% (-82.5% to -59.6%)#**	**-72.3% (-79.6% to -63.0%)#**	**-73.7% (-79.1% to -66.9%)#**	**-74.2% (-77.2% to -70.6%)#**	**-65.5% (-68.5% to -62.2%)#**	**-73.2% (-76.5% to -69.6%)#**	**-71.4% (-79.2% to -60.7%)#**	**-72.1% (-78.8% to -63.3%)#**	**-72.0% (-76.4% to -66.5%)#**	**-71.2% (-74.3% to -67.9%)#**
	% change 2000-2017	**-48.5% (-64.1% to -26.1%)#**	**-59.1% (-72.8% to -39.4%)#**	**-55.9% (-67.8% to -41.2%)#**	**-55.4% (-64.9% to -43.6%)#**	**-49.5% (-55.5% to -42.5%)#**	**-36.1% (-41.6% to -29.9%)#**	**-56.7% (-62.0% to -50.8%)#**	**-53.6% (-66.3% to -36.6%)#**	**-54.1% (-64.8% to -40.3%)#**	**-53.0% (-60.0% to -44.0%)#**	**-52.1% (-57.2% to -46.5%)#**
Tochigi	Death #, 1990	44 (37 to 53)	14 (11 to 17)	41 (37 to 44)	40 (34 to 48)	24 (23 to 25)	19 (19 to 20)	88 (87 to 90)	98 (90 to 108)	139 (130 to 149)	271 (260 to 282)	108 (106 to 109)
	Death #, 2000	30 (25 to 35)	8 (6 to 10)	29 (25 to 32)	27 (22 to 32)	15 (14 to 16)	14 (14 to 15)	47 (46 to 48)	66 (59 to 74)	93 (86 to 101)	170 (161 to 179)	62 (61 to 63)
	Death #, 2017	12 (8 to 16)	4 (3 to 5)	12 (9 to 16)	14 (11 to 17)	5 (5 to 6)	7 (6 to 7)	24 (21 to 28)	28 (20 to 38)	42 (31 to 55)	78 (65 to 94)	31 (28 to 35)
	% change 1990-2017	**-73.2% (-82.5% to -60.7%)#**	**-72.8% (-82.7% to -59.4%)#**	**-69.5% (-77.5% to -59.2%)#**	**-65.6% (-74.7% to -55.1%)#**	**-77.7% (-80.9% to -74.3%)#**	**-65.2% (-68.6% to -61.6%)#**	**-72.3% (-75.7% to -68.5%)#**	**-71.6% (-80.1% to -61.5%)#**	**-69.9% (-78.1% to -60.2%)#**	**-71.0% (-76.1% to -65.2%)#**	**-71.0% (-74.2% to -67.5%)#**
	% change 2000-2017	**-60.5% (-73.2% to -42.8%)#**	**-52.5% (-69.8% to -29.0%)#**	**-56.8% (-68.6% to -41.7%)#**	**-48.5% (-62.4% to -31.5%)#**	**-63.4% (-68.7% to -57.4%)#**	**-53.5% (-58.0% to -48.9%)#**	**-48.2% (-54.5% to -41.1%)#**	**-58.0% (-70.2% to -42.3%)#**	**-55.2% (-66.8% to -40.6%)#**	**-53.8% (-62.1% to -44.7%)#**	**-49.4% (-54.8% to -43.4%)#**
Tokushima	Death #, 1990	17 (14 to 20)	5 (4 to 7)	16 (14 to 18)	15 (13 to 17)	12 (11 to 13)	10 (9 to 10)	26 (25 to 26)	39 (34 to 43)	54 (49 to 59)	101 (96 to 108)	36 (35 to 36)
	Death #, 2000	10 (8 to 11)	5 (4 to 6)	12 (11 to 13)	9 (7 to 11)	4 (4 to 4)	6 (5 to 6)	16 (15 to 16)	26 (24 to 28)	35 (32 to 38)	60 (57 to 65)	21 (21 to 22)
	Death #, 2017	6 (4 to 8)	2 (2 to 3)	6 (4 to 8)	3 (2 to 4)	4 (4 to 5)	4 (3 to 4)	8 (7 to 9)	14 (10 to 20)	17 (12 to 24)	33 (27 to 41)	11 (10 to 13)
	% change 1990-2017	**-65.8% (-78.3% to -48.5%)#**	**-56.9% (-73.3% to -30.7%)#**	**-63.0% (-73.7% to -48.5%)#**	**-77.5% (-84.2% to -69.6%)#**	**-66.1% (-71.3% to -59.9%)#**	**-63.1% (-66.8% to -59.0%)#**	**-70.4% (-73.9% to -66.6%)#**	**-63.4% (-75.0% to -48.0%)#**	**-67.3% (-76.7% to -54.6%)#**	**-67.6% (-73.9% to -59.8%)#**	**-68.4% (-71.7% to -64.7%)#**
	% change 2000-2017	**-39.6% (-59.1% to -11.8%)#**	**-50.1% (-68.1% to -25.2%)#**	**-48.9% (-63.2% to -29.8%)#**	**-62.5% (-74.3% to -45.2%)#**	-1.1% (-16.0% to 16.6%)	**-35.6% (-42.5% to -28.4%)#**	**-51.5% (-57.4% to -45.0%)#**	**-45.6% (-62.1% to -23.3%)#**	**-50.0% (-64.5% to -31.1%)#**	**-45.7% (-56.5% to -32.8%)#**	**-47.4% (-52.6% to -41.1%)#**
Tokyo	Death #, 1990	236 (195 to 282)	100 (70 to 136)	256 (224 to 291)	212 (166 to 268)	88 (84 to 91)	98 (96 to 101)	330 (327 to 333)	592 (530 to 655)	805 (764 to 847)	1,321 (1,274 to 1,368)	429 (424 to 434)
	Death #, 2000	234 (198 to 272)	102 (80 to 128)	202 (172 to 231)	147 (118 to 184)	56 (53 to 58)	52 (51 to 53)	158 (156 to 160)	538 (479 to 596)	685 (635 to 735)	950 (898 to 1,005)	210 (207 to 213)
	Death #, 2017	90 (64 to 123)	31 (22 to 43)	94 (71 to 122)	74 (54 to 103)	37 (33 to 42)	38 (35 to 41)	96 (85 to 107)	214 (157 to 288)	288 (220 to 378)	459 (381 to 556)	133 (120 to 148)
	% change 1990-2017	**-62.0% (-74.5% to -44.2%)#**	**-69.0% (-80.7% to -49.9%)#**	**-63.5% (-72.9% to -50.3%)#**	**-65.1% (-76.2% to -49.6%)#**	**-57.7% (-62.7% to -51.5%)#**	**-61.8% (-65.0% to -58.1%)#**	**-71.0% (-74.3% to -67.5%)#**	**-63.8% (-73.9% to -50.9%)#**	**-64.2% (-73.0% to -53.0%)#**	**-65.3% (-71.3% to -57.5%)#**	**-68.9% (-72.0% to -65.6%)#**
	% change 2000-2017	**-61.7% (-73.1% to -45.6%)#**	**-69.7% (-80.3% to -54.0%)#**	**-53.6% (-66.5% to -37.1%)#**	**-49.6% (-66.1% to -23.9%)#**	**-33.0% (-41.7% to -23.0%)#**	**-27.9% (-33.9% to -20.9%)#**	**-39.4% (-46.4% to -32.2%)#**	**-60.2% (-71.4% to -45.4%)#**	**-57.9% (-68.3% to -44.5%)#**	**-51.7% (-60.3% to -40.9%)#**	**-36.5% (-42.8% to -29.6%)#**
Tottori	Death #, 1990	10 (8 to 14)	4 (3 to 5)	12 (10 to 14)	10 (8 to 12)	11 (10 to 12)	7 (7 to 7)	16 (16 to 17)	27 (23 to 30)	37 (33 to 41)	71 (66 to 76)	23 (23 to 24)
	Death #, 2000	6 (5 to 7)	3 (2 to 3)	6 (5 to 7)	6 (4 to 7)	5 (5 to 5)	4 (4 to 4)	10 (10 to 11)	14 (13 to 16)	20 (18 to 22)	40 (37 to 43)	15 (14 to 15)
	Death #, 2017	4 (3 to 5)	2 (1 to 2)	3 (2 to 4)	3 (2 to 4)	3 (3 to 3)	3 (3 to 3)	6 (6 to 7)	8 (6 to 12)	12 (9 to 16)	24 (20 to 29)	9 (8 to 10)
	% change 1990-2017	**-64.7% (-77.7% to -44.1%)#**	**-62.7% (-76.2% to -42.9%)#**	**-73.5% (-81.4% to -63.0%)#**	**-67.2% (-76.8% to -54.2%)#**	**-72.2% (-76.9% to -66.8%)#**	**-55.6% (-60.4% to -50.8%)#**	**-61.9% (-66.0% to -57.5%)#**	**-68.3% (-78.2% to -55.6%)#**	**-68.0% (-77.0% to -56.3%)#**	**-66.0% (-72.5% to -58.6%)#**	**-60.0% (-64.0% to -55.8%)#**
	% change 2000-2017	**-38.5% (-59.1% to -10.4%)#**	**-38.4% (-60.1% to -6.5%)#**	**-44.1% (-60.8% to -21.0%)#**	**-42.2% (-60.0% to -18.3%)#**	**-40.5% (-50.1% to -29.4%)#**	**-25.8% (-33.8% to -17.5%)#**	**-40.1% (-46.6% to -33.1%)#**	**-40.7% (-59.2% to -16.4%)#**	**-41.1% (-58.0% to -20.2%)#**	**-39.1% (-50.5% to -26.1%)#**	**-36.0% (-42.2% to -29.0%)#**
Toyama	Death #, 1990	25 (20 to 30)	10 (7 to 13)	21 (19 to 23)	21 (18 to 24)	11 (10 to 12)	9 (9 to 9)	37 (36 to 38)	56 (50 to 63)	76 (70 to 83)	134 (126 to 141)	46 (45 to 47)
	Death #, 2000	14 (13 to 16)	7 (5 to 9)	14 (12 to 15)	11 (9 to 14)	7 (6 to 7)	8 (8 to 9)	20 (20 to 20)	35 (32 to 38)	46 (42 to 50)	81 (77 to 86)	28 (28 to 29)
	Death #, 2017	5 (3 to 7)	2 (2 to 3)	6 (4 to 8)	5 (4 to 7)	4 (3 to 5)	6 (5 to 6)	13 (11 to 15)	13 (9 to 18)	18 (13 to 25)	41 (34 to 49)	19 (17 to 20)
	% change 1990-2017	**-80.4% (-87.6% to -70.5%)#**	**-75.5% (-85.4% to -62.2%)#**	**-73.1% (-80.4% to -63.7%)#**	**-74.2% (-81.2% to -65.9%)#**	**-64.7% (-70.3% to -59.1%)#**	**-38.8% (-45.0% to -32.8%)#**	**-65.0% (-69.0% to -60.7%)#**	**-76.8% (-84.2% to -67.4%)#**	**-76.1% (-82.9% to -68.0%)#**	**-69.5% (-74.8% to -63.5%)#**	**-59.8% (-64.0% to -55.5%)#**
	% change 2000-2017	**-66.7% (-77.1% to -52.6%)#**	**-65.3% (-77.6% to -47.4%)#**	**-58.7% (-69.9% to -43.5%)#**	**-52.0% (-66.1% to -32.5%)#**	**-43.5% (-52.2% to -33.6%)#**	**-31.8% (-38.8% to -24.8%)#**	**-34.9% (-42.4% to -26.9%)#**	**-63.3% (-74.2% to -48.4%)#**	**-60.5% (-71.6% to -46.8%)#**	**-49.9% (-58.4% to -40.2%)#**	**-34.0% (-40.6% to -26.9%)#**
Wakayama	Death #, 1990	22 (18 to 26)	8 (6 to 10)	19 (16 to 21)	18 (16 to 21)	13 (13 to 14)	10 (9 to 10)	41 (41 to 42)	48 (42 to 55)	66 (61 to 72)	131 (125 to 139)	51 (50 to 52)
	Death #, 2000	12 (10 to 13)	4 (3 to 5)	12 (11 to 14)	11 (9 to 14)	6 (6 to 6)	5 (5 to 5)	20 (20 to 21)	28 (26 to 31)	39 (36 to 43)	70 (66 to 75)	25 (25 to 26)
	Death #, 2017	5 (3 to 7)	2 (1 to 3)	5 (3 to 6)	4 (3 to 5)	4 (3 to 4)	3 (3 to 4)	8 (7 to 9)	12 (8 to 16)	16 (11 to 21)	31 (25 to 37)	11 (10 to 13)

eTable 2. ENN, LNN, PNN, 1-4, 5-9, 10-14, 15-19, <1, <5, 10-19, and <20 years mortality in 1990, 2000 and 2017 with mean percentage change for all GBD causes.

	Metric	Early Neonatal	Late Neonatal	Post Neonatal	1 to 4	5 to 9	10 to 14	15 to 19	<1 year	Under 5	<20 years	10 to 19
	% change 1990-2017	-77.4% (-85.4% to -66.3%)#	-74.4% (-84.2% to -59.1%)#	-74.5% (-81.8% to -64.8%)#	-78.7% (-84.7% to -71.4%)#	-73.6% (-77.7% to -69.1%)#	-66.7% (-70.0% to -63.3%)#	-80.3% (-82.6% to -77.9%)#	-75.8% (-83.4% to -66.1%)#	-76.6% (-83.3% to -68.1%)#	-76.7% (-81.0% to -71.5%)#	-77.7% (-80.0% to -75.3%)#
	% change 2000-2017	-57.6% (-71.6% to -38.5%)#	-51.7% (-69.7% to -24.0%)#	-61.6% (-72.2% to -47.5%)#	-64.4% (-75.1% to -49.9%)#	-39.9% (-49.1% to -29.3%)#	-38.9% (-44.9% to -32.1%)#	-59.5% (-64.3% to -54.3%)#	-58.6% (-70.8% to -41.3%)#	-60.2% (-71.5% to -45.3%)#	-56.7% (-64.5% to -46.9%)#	-55.2% (-60.0% to -50.1%)#
Yamagata	Death #, 1990	21 (18 to 26)	6 (5 to 7)	19 (17 to 21)	21 (18 to 25)	12 (11 to 12)	11 (11 to 12)	27 (27 to 28)	47 (42 to 53)	67 (63 to 73)	118 (112 to 124)	39 (38 to 39)
	Death #, 2000	12 (10 to 14)	5 (4 to 7)	13 (11 to 14)	11 (9 to 14)	7 (7 to 8)	7 (6 to 7)	24 (24 to 25)	29 (27 to 32)	41 (38 to 44)	79 (74 to 83)	31 (30 to 31)
	Death #, 2017	7 (5 to 10)	3 (2 to 4)	6 (5 to 8)	6 (4 to 7)	4 (4 to 5)	4 (4 to 4)	12 (10 to 13)	16 (11 to 22)	22 (16 to 29)	41 (34 to 50)	16 (14 to 18)
	% change 1990-2017	-66.7% (-78.7% to -50.2%)#	-55.4% (-71.8% to -32.1%)#	-67.2% (-76.1% to -55.8%)#	-73.6% (-80.9% to -64.6%)#	-65.6% (-70.9% to -60.2%)#	-65.2% (-68.7% to -61.7%)#	-57.4% (-62.8% to -51.5%)#	-65.5% (-76.0% to -52.7%)#	-68.0% (-77.2% to -56.9%)#	-65.0% (-71.7% to -57.5%)#	-59.7% (-64.1% to -54.5%)#
	% change 2000-2017	-40.1% (-58.7% to -15.2%)#	-47.0% (-67.1% to -16.3%)#	-50.0% (-63.5% to -31.2%)#	-51.8% (-65.9% to -32.9%)#	-43.4% (-51.8% to -33.9%)#	-40.5% (-46.5% to -34.3%)#	-51.5% (-57.6% to -44.6%)#	-45.5% (-61.8% to -24.0%)#	-47.2% (-61.9% to -28.2%)#	-47.6% (-57.5% to -36.6%)#	-49.2% (-55.1% to -42.8%)#
Yamaguchi	Death #, 1990	31 (25 to 38)	12 (9 to 15)	29 (26 to 32)	27 (22 to 31)	17 (16 to 18)	19 (19 to 20)	49 (48 to 50)	71 (64 to 80)	98 (91 to 106)	183 (174 to 193)	68 (67 to 70)
	Death #, 2000	14 (12 to 17)	7 (5 to 9)	19 (17 to 22)	17 (13 to 20)	8 (8 to 9)	8 (8 to 8)	28 (28 to 29)	41 (37 to 44)	57 (53 to 62)	102 (97 to 108)	36 (36 to 37)
	Death #, 2017	7 (5 to 10)	2 (2 to 3)	8 (6 to 10)	7 (6 to 9)	3 (3 to 4)	4 (4 to 5)	15 (13 to 16)	17 (12 to 24)	25 (18 to 33)	47 (39 to 56)	19 (17 to 21)
	% change 1990-2017	-76.6% (-84.9% to -65.5%)#	-79.7% (-87.2% to -68.7%)#	-72.8% (-80.4% to -63.1%)#	-72.2% (-79.4% to -63.4%)#	-80.3% (-83.2% to -77.2%)#	-77.8% (-79.9% to -75.5%)#	-70.3% (-73.9% to -66.4%)#	-75.6% (-83.2% to -66.3%)#	-74.7% (-81.9% to -66.4%)#	-74.3% (-79.1% to -68.9%)#	-72.4% (-75.4% to -69.0%)#
	% change 2000-2017	-49.5% (-65.5% to -27.4%)#	-65.8% (-78.0% to -47.4%)#	-60.1% (-71.8% to -44.7%)#	-55.5% (-67.9% to -39.1%)#	-60.4% (-66.2% to -53.6%)#	-47.3% (-52.7% to -41.4%)#	-48.6% (-54.8% to -41.8%)#	-57.4% (-69.9% to -40.3%)#	-56.8% (-68.8% to -41.1%)#	-54.1% (-62.3% to -44.2%)#	-48.3% (-53.8% to -42.1%)#
Yamanashi	Death #, 1990	17 (14 to 20)	6 (4 to 7)	16 (14 to 18)	17 (14 to 20)	10 (9 to 10)	8 (7 to 8)	35 (35 to 36)	39 (34 to 43)	55 (51 to 60)	108 (102 to 114)	43 (42 to 44)
	Death #, 2000	10 (8 to 12)	5 (3 to 6)	12 (10 to 15)	9 (7 to 12)	7 (6 to 7)	5 (5 to 5)	20 (20 to 21)	27 (24 to 30)	36 (33 to 40)	68 (64 to 73)	25 (25 to 26)
	Death #, 2017	4 (3 to 5)	1 (1 to 2)	4 (3 to 5)	4 (3 to 5)	3 (3 to 3)	2 (2 to 2)	8 (7 to 9)	9 (7 to 13)	13 (9 to 18)	27 (22 to 32)	11 (9 to 12)
	% change 1990-2017	-77.3% (-85.3% to -66.2%)#	-74.6% (-84.0% to -60.1%)#	-75.3% (-82.2% to -65.9%)#	-77.6% (-84.0% to -69.8%)#	-68.9% (-73.9% to -63.0%)#	-70.2% (-73.1% to -67.1%)#	-76.3% (-79.0% to -73.3%)#	-76.0% (-83.4% to -66.3%)#	-76.5% (-83.2% to -68.0%)#	-75.3% (-79.7% to -70.1%)#	-75.3% (-77.9% to -72.3%)#
	% change 2000-2017	-61.9% (-74.8% to -44.1%)#	-67.6% (-79.9% to -47.5%)#	-67.9% (-77.7% to -54.3%)#	-60.6% (-72.8% to -45.0%)#	-57.1% (-64.1% to -49.3%)#	-53.4% (-58.0% to -48.4%)#	-59.1% (-63.6% to -53.9%)#	-65.6% (-76.3% to -51.6%)#	-64.3% (-74.2% to -51.6%)#	-61.3% (-68.0% to -53.0%)#	-58.0% (-62.2% to -53.0%)#
S Korea	Death #, 1990	1,538 (1,199 to 1,897)	557 (414 to 727)	1,753 (1,337 to 2,234)	2,312 (1,946 to 2,711)	2,710 (2,422 to 3,034)	1,935 (1,823 to 2,054)	3,945 (3,871 to 4,020)	3,847 (3,347 to 4,402)	6,160 (5,326 to 7,123)	14,748 (13,443 to 16,216)	5,879 (5,695 to 6,076)
	Death #, 2000	1,141 (1,030 to 1,248)	472 (387 to 561)	1,248 (1,064 to 1,432)	1,400 (1,221 to 1,598)	920 (903 to 937)	627 (620 to 636)	1,768 (1,760 to 1,776)	2,860 (2,672 to 3,029)	4,260 (4,144 to 4,377)	7,575 (7,434 to 7,717)	2,395 (2,380 to 2,409)
	Death #, 2017	425 (357 to 492)	212 (175 to 247)	509 (387 to 635)	290 (267 to 313)	221 (208 to 233)	200 (192 to 209)	688 (644 to 734)	1,146 (1,050 to 1,237)	1,436 (1,316 to 1,550)	2,545 (2,399 to 2,691)	888 (838 to 941)
	% change 1990-2017	-72.3% (-79.0% to -62.1%)#	-62.0% (-73.7% to -44.8%)#	-71.0% (-80.6% to -56.0%)#	-87.4% (-89.5% to -84.8%)#	-91.8% (-92.8% to -90.7%)#	-89.6% (-90.4% to -88.8%)#	-82.6% (-83.8% to -81.3%)#	-70.2% (-74.3% to -65.3%)#	-76.7% (-80.1% to -72.7%)#	-82.7% (-84.5% to -80.8%)#	-84.9% (-85.9% to -83.9%)#
	% change 2000-2017	-62.7% (-69.7% to -54.7%)#	-55.2% (-66.3% to -40.5%)#	-59.2% (-70.3% to -45.0%)#	-79.3% (-82.2% to -75.6%)#	-76.0% (-77.4% to -74.5%)#	-68.0% (-69.5% to -66.7%)#	-61.1% (-63.6% to -58.5%)#	-60.0% (-63.8% to -55.6%)#	-66.3% (-69.2% to -63.5%)#	-66.4% (-68.5% to -64.4%)#	-62.9% (-65.0% to -60.8%)#
Singapore	Death #, 1990	144 (131 to 155)	50 (45 to 54)	132 (121 to 141)	70 (59 to 82)	44 (42 to 46)	48 (47 to 49)	113 (112 to 115)	325 (299 to 350)	395 (369 to 419)	601 (571 to 628)	162 (159 to 164)
	Death #, 2000	82 (73 to 92)	34 (31 to 38)	83 (74 to 91)	37 (31 to 45)	28 (27 to 30)	39 (37 to 40)	74 (69 to 79)	199 (179 to 220)	236 (215 to 259)	376 (352 to 403)	112 (106 to 119)
	Death #, 2017	32 (24 to 44)	14 (10 to 19)	33 (26 to 43)	17 (14 to 20)	20 (18 to 21)	20 (19 to 21)	62 (59 to 66)	80 (60 to 104)	97 (75 to 124)	198 (172 to 229)	82 (77 to 87)
	% change 1990-2017	-77.6% (-83.8% to -69.5%)#	-71.7% (-79.4% to -61.7%)#	-74.5% (-80.2% to -67.4%)#	-76.0% (-80.8% to -70.2%)#	-54.7% (-59.0% to -50.2%)#	-59.2% (-61.8% to -56.6%)#	-45.1% (-48.3% to -41.4%)#	-75.5% (-81.6% to -67.5%)#	-75.5% (-81.3% to -68.6%)#	-67.0% (-71.7% to -61.5%)#	-49.3% (-52.3% to -46.2%)#
	% change 2000-2017	-60.8% (-72.1% to -46.5%)#	-58.7% (-70.3% to -44.0%)#	-59.4% (-68.7% to -47.2%)#	-54.3% (-63.3% to -41.9%)#	-29.2% (-35.9% to -22.0%)#	-48.9% (-52.5% to -45.2%)#	-15.6% (-22.7% to -7.3%)#	-59.9% (-70.5% to -46.5%)#	-59.0% (-68.7% to -47.0%)#	-47.2% (-55.0% to -38.1%)#	-27.1% (-32.6% to -20.9%)#
**High-income North America**	Death #, 1990	20,966 (20,560 to 21,390)	4,530 (4,374 to 4,675)	15,231 (14,818 to 15,627)	7,689 (7,445 to 7,928)	4,428 (4,403 to 4,456)	4,912 (4,896 to 4,928)	16,791 (16,762 to 16,820)	40,727 (40,344 to 41,108)	48,416 (48,056 to 48,799)	74,547 (74,121 to 74,991)	21,703 (21,662 to 21,746)
	% of global total, 1990	0.7% (0.7% to 0.7%)	0.4% (0.3% to 0.4%)	0.4% (0.4% to 0.4%)	0.2% (0.2% to 0.2%)	0.5% (0.5% to 0.5%)	1.1% (1.0% to 1.1%)	2.5% (2.5% to 2.5%)	0.5% (0.5% to 0.5%)	0.4% (0.4% to 0.4%)	0.5% (0.5% to 0.6%)	1.9% (1.9% to 1.9%)
	Death #, 2000	15,806 (15,543 to 16,076)	4,011 (3,871 to 4,153)	10,112 (9,862 to 10,371)	5,429 (5,257 to 5,614)	3,545 (3,522 to 3,567)	4,442 (4,427 to 4,458)	14,690 (14,666 to 14,716)	29,928 (29,600 to 30,225)	35,357 (35,056 to 35,653)	58,034 (57,694 to 58,375)	19,132 (19,097 to 19,169)
	% of global total, 2000	0.6% (0.6% to 0.6%)	0.4% (0.4% to 0.5%)	0.3% (0.3% to 0.3%)	0.2% (0.2% to 0.2%)	0.5% (0.5% to 0.5%)	0.9% (0.9% to 0.9%)	2.2% (2.1% to 2.2%)	0.4% (0.4% to 0.5%)	0.4% (0.4% to 0.4%)	0.5% (0.5% to 0.5%)	1.6% (1.6% to 1.7%)
	Death #, 2017	12,803 (12,330 to 13,265)	3,219 (3,070 to 3,364)	7,899 (7,613 to 8,175)	4,194 (4,060 to 4,326)	2,573 (2,528 to 2,614)	3,305 (3,258 to 3,348)	11,758 (11,511 to 12,028)	23,921 (23,011 to 24,781)	28,115 (27,078 to 29,090)	45,750 (44,558 to 46,850)	15,062 (14,778 to 15,363)
	% of global total, 2017	0.7% (0.7% to 0.7%)	0.6% (0.6% to 0.7%)	0.5% (0.5% to 0.5%)	0.3% (0.3% to 0.3%)	0.6% (0.6% to 0.6%)	1.0% (1.0% to 1.1%)	2.3% (2.2% to 2.3%)	0.6% (0.6% to 0.6%)	0.5% (0.5% to 0.6%)	0.7% (0.7% to 0.7%)	1.8% (1.8% to 1.9%)
	% change 1990-2017	-38.9% (-41.6% to -36.2%)#	-29.0% (-32.9% to -24.7%)#	-48.1% (-50.4% to -45.9%)#	-45.5% (-47.9% to -42.9%)#	-41.9% (-42.9% to -40.8%)#	-32.7% (-33.7% to -31.8%)#	-30.0% (-31.5% to -28.4%)#	-41.3% (-43.5% to -39.0%)#	-41.9% (-44.1% to -39.8%)#	-38.6% (-40.3% to -37.1%)#	-30.6% (-31.9% to -29.2%)#
	% change 2000-2017	-19.0% (-22.2% to -15.8%)#	-19.8% (-24.4% to -14.9%)#	-21.9% (-25.3% to -18.4%)#	-22.8% (-26.3% to -19.2%)#	-27.4% (-28.8% to -26.1%)#	-25.6% (-26.7% to -24.6%)#	-20.0% (-21.7% to -18.1%)#	-20.1% (-23.1% to -17.1%)#	-20.5% (-23.5% to -17.6%)#	-21.2% (-23.3% to -19.2%)#	-21.3% (-22.8% to -19.7%)#
Canada	Death #, 1990	1,482 (1,369 to 1,602)	312 (261 to 369)	939 (840 to 1,046)	543 (468 to 632)	378 (368 to 387)	418 (412 to 424)	1,261 (1,253 to 1,269)	2,733 (2,610 to 2,850)	3,276 (3,176 to 3,379)	5,333 (5,211 to 5,456)	1,679 (1,666 to 1,692)
	Death #, 2000	925 (865 to 988)	232 (197 to 270)	535 (486 to 585)	317 (266 to 371)	256 (249 to 264)	318 (313 to 323)	1,043 (1,036 to 1,050)	1,692 (1,626 to 1,755)	2,009 (1,936 to 2,088)	3,627 (3,538 to 3,722)	1,361 (1,350 to 1,373)
	Death #, 2017	890 (681 to 1,157)	287 (218 to 376)	641 (496 to 823)	273 (226 to 329)	181 (167 to 197)	249 (237 to 262)	804 (761 to 849)	1,819 (1,393 to 2,353)	2,091 (1,621 to 2,679)	3,326 (2,812 to 3,971)	1,053 (999 to 1,108)
	% change 1990-2017	-39.9% (-54.5% to -21.7%)#	-8.1% (-34.2% to 26.5%)	-31.7% (-49.0% to -10.1%)#	-49.8% (-59.9% to -36.3%)#	-52.0% (-55.8% to -47.7%)#	-40.4% (-43.5% to -37.2%)#	-36.3% (-39.8% to -32.6%)#	-33.5% (-49.0% to -13.4%)#	-36.2% (-50.5% to -18.2%)#	-37.6% (-47.3% to -25.8%)#	-37.3% (-40.4% to -33.9%)#
	% change 2000-2017	-3.7% (-27.4% to 26.1%)	23.5% (-10.4% to 67.0%)	19.8% (-9.1% to 56.1%)	-14.1% (-34.2% to 8.8%)	-29.3% (-35.3% to -23.1%)#	-21.6% (-25.7% to -17.2%)#	-22.9% (-27.1% to -18.6%)#	7.5% (-18.3% to 38.8%)	4.1% (-20.3% to 33.7%)	-8.3% (-22.9% to 9.2%)	-22.6% (-26.6% to -18.4%)#
Greenland	Death #, 1990	16 (14 to 18)	4 (4 to 5)	13 (10 to 15)	6 (5 to 8)	5 (4 to 5)	4 (4 to 4)	11 (10 to 11)	33 (29 to 37)	40 (34 to 45)	59 (53 to 65)	14 (14 to 15)
	Death #, 2000	7 (6 to 8)	2 (2 to 2)	5 (4 to 6)	5 (4 to 6)	3 (3 to 3)	4 (3 to 4)	8 (8 to 9)	14 (12 to 17)	19 (16 to 23)	34 (30 to 39)	12 (12 to 13)
	Death #, 2017	4 (3 to 6)	1 (1 to 1)	3 (2 to 4)	3 (2 to 3)	1 (1 to 1)	2 (1 to 2)	4 (4 to 4)	9 (7 to 11)	11 (9 to 14)	18 (15 to 21)	5 (5 to 6)
	% change 1990-2017	-72.5% (-79.1% to -64.4%)#	-73.4% (-80.0% to -64.8%)#	-74.9% (-81.4% to -65.6%)#	-60.9% (-72.4% to -45.4%)#	-78.2% (-81.9% to -74.6%)#	-59.4% (-64.5% to -54.7%)#	-63.6% (-66.7% to -60.9%)#	-73.5% (-79.7% to -65.7%)#	-71.4% (-78.4% to -62.5%)#	-69.8% (-75.7% to -62.9%)#	-62.5% (-66.1% to -59.5%)#

eTable 2. ENN, LNN, PNN, 1-4, 5-9, 10-14, 15-19, <1, <5, 10-19, and <20 years mortality in 1990, 2000 and 2017 with mean percentage change for all GBD causes.

	Metric	Early Neonatal	Late Neonatal	Post Neonatal	1 to 4	5 to 9	10 to 14	15 to 19	<1 year	Under 5	<20 years	10 to 19
	% change 2000-2017	-35.8% (-50.5% to -16.1%)#	-41.0% (-55.3% to -20.3%)#	-40.3% (-52.8% to -22.4%)#	-47.3% (-61.5% to -25.3%)#	-66.2% (-71.8% to -60.2%)#	-57.9% (-63.2% to -52.5%)#	-53.6% (-57.4% to -50.1%)#	-38.2% (-51.6% to -20.8%)#	-40.5% (-53.6% to -22.1%)#	-47.9% (-56.9% to -36.2%)#	-54.9% (-59.1% to -50.8%)#
USA	Death #, 1990	19,467 (19,088 to 19,867)	4,214 (4,061 to 4,357)	14,279 (13,861 to 14,692)	7,140 (6,914 to 7,363)	4,046 (4,023 to 4,071)	4,490 (4,476 to 4,505)	15,519 (15,494 to 15,546)	37,960 (37,579 to 38,339)	45,100 (44,766 to 45,461)	69,154 (68,771 to 69,567)	20,009 (19,972 to 20,048)
	Death #, 2000	14,874 (14,612 to 15,136)	3,776 (3,642 to 3,909)	9,571 (9,326 to 9,827)	5,107 (4,946 to 5,280)	3,285 (3,264 to 3,307)	4,120 (4,106 to 4,135)	13,638 (13,614 to 13,662)	28,221 (27,905 to 28,518)	33,328 (33,035 to 33,613)	54,372 (54,034 to 54,701)	17,759 (17,724 to 17,793)
	Death #, 2017	11,908 (11,509 to 12,293)	2,930 (2,809 to 3,051)	7,254 (7,022 to 7,476)	3,919 (3,795 to 4,042)	2,391 (2,350 to 2,431)	3,054 (3,009 to 3,096)	10,950 (10,698 to 11,209)	22,093 (21,359 to 22,809)	26,012 (25,175 to 26,833)	42,406 (41,395 to 43,345)	14,003 (13,720 to 14,296)
	% change 1990-2017	-38.8% (-41.3% to -36.4%)#	-30.5% (-34.1% to -26.5%)#	-49.2% (-51.2% to -47.0%)#	-45.1% (-47.6% to -42.5%)#	-40.9% (-42.0% to -39.8%)#	-32.0% (-33.0% to -31.0%)#	-29.4% (-31.1% to -27.8%)#	-41.8% (-43.8% to -39.7%)#	-42.3% (-44.2% to -40.3%)#	-38.7% (-40.2% to -37.2%)#	-30.0% (-31.4% to -28.6%)#
	% change 2000-2017	-19.9% (-23.0% to -16.9%)#	-22.4% (-26.5% to -18.2%)#	-24.2% (-27.2% to -21.1%)#	-23.3% (-27.1% to -19.7%)#	-27.2% (-28.6% to -25.9%)#	-25.9% (-27.0% to -24.8%)#	-19.7% (-21.6% to -17.8%)#	-21.7% (-24.4% to -18.9%)#	-21.9% (-24.6% to -19.2%)#	-22.0% (-23.9% to -20.1%)#	-21.1% (-22.8% to -19.5%)#
Alabama	Death #, 1990	395 (350 to 441)	90 (73 to 110)	251 (212 to 293)	136 (114 to 163)	82 (79 to 86)	102 (100 to 105)	360 (355 to 364)	736 (692 to 782)	872 (828 to 917)	1,416 (1,363 to 1,470)	462 (456 to 468)
	Death #, 2000	300 (261 to 337)	89 (71 to 109)	229 (195 to 270)	113 (90 to 140)	66 (63 to 69)	88 (86 to 90)	306 (302 to 310)	618 (575 to 660)	731 (692 to 774)	1,190 (1,145 to 1,239)	394 (388 to 399)
	Death #, 2017	222 (188 to 261)	87 (72 to 105)	185 (161 to 213)	83 (72 to 96)	53 (49 to 57)	65 (60 to 70)	239 (213 to 268)	493 (421 to 575)	577 (495 to 671)	933 (837 to 1,046)	304 (274 to 337)
	% change 1990-2017	-43.8% (-54.2% to -30.4%)#	-3.5% (-26.1% to 29.0%)	-26.4% (-40.6% to -8.5%)#	-39.0% (-51.5% to -23.0%)#	-35.9% (-41.1% to -29.7%)#	-36.7% (-41.0% to -31.7%)#	-33.5% (-41.0% to -25.4%)#	-32.9% (-43.2% to -20.6%)#	-33.9% (-43.5% to -21.9%)#	-34.1% (-41.2% to -25.2%)#	-34.2% (-40.7% to -27.0%)#
	% change 2000-2017	-26.0% (-40.0% to -7.2%)#	-2.3% (-26.1% to 31.9%)	-19.4% (-35.0% to -0.8%)#	-26.7% (-43.0% to -4.2%)#	-19.6% (-26.4% to -11.7%)#	-26.1% (-31.5% to -20.3%)#	-21.9% (-30.4% to -12.5%)#	-20.1% (-33.0% to -4.8%)#	-21.1% (-32.6% to -7.1%)#	-21.6% (-30.1% to -11.4%)#	-22.8% (-30.3% to -14.4%)#
Alaska	Death #, 1990	34 (29 to 38)	10 (7 to 14)	54 (46 to 63)	22 (18 to 27)	16 (15 to 17)	15 (15 to 16)	50 (49 to 51)	98 (90 to 107)	120 (112 to 129)	202 (192 to 212)	66 (64 to 67)
	Death #, 2000	22 (19 to 25)	7 (5 to 10)	36 (31 to 42)	17 (13 to 22)	9 (9 to 10)	17 (16 to 17)	57 (56 to 58)	65 (59 to 71)	81 (75 to 88)	164 (156 to 172)	73 (72 to 75)
	Death #, 2017	20 (16 to 26)	9 (5 to 13)	28 (22 to 35)	14 (10 to 20)	10 (8 to 11)	12 (11 to 13)	36 (33 to 39)	57 (46 to 69)	71 (57 to 87)	129 (112 to 149)	48 (45 to 52)
	% change 1990-2017	-39.5% (-54.2% to -23.0%)#	-17.3% (-54.5% to 46.4%)	-49.2% (-62.1% to -31.6%)#	-35.8% (-56.4% to -7.3%)#	-39.7% (-48.1% to -29.2%)#	-20.6% (-27.6% to -12.8%)#	-28.1% (-34.0% to -21.7%)#	-42.5% (-53.5% to -29.1%)#	-41.3% (-53.2% to -26.2%)#	-36.3% (-45.4% to -25.5%)#	-26.3% (-32.1% to -20.1%)#
	% change 2000-2017	-5.6% (-26.6% to 20.3%)	18.8% (-35.2% to 105.7%)	-22.7% (-42.3% to 0.7%)	-16.2% (-46.0% to 25.0%)	5.1% (-10.2% to 23.3%)	-26.0% (-32.4% to -18.9%)#	-36.4% (-41.5% to -30.7%)#	-12.4% (-29.9% to 9.8%)	-13.2% (-31.6% to 9.5%)	-21.5% (-32.6% to -8.2%)#	-34.1% (-39.1% to -28.6%)#
Arizona	Death #, 1990	249 (223 to 277)	64 (54 to 74)	247 (215 to 281)	124 (106 to 144)	65 (62 to 68)	74 (73 to 76)	248 (244 to 251)	560 (525 to 598)	684 (650 to 719)	1071 (1032 to 1113)	322 (317 to 326)
	Death #, 2000	253 (229 to 279)	75 (63 to 89)	202 (177 to 228)	110 (91 to 133)	72 (69 to 75)	86 (84 to 88)	295 (292 to 299)	530 (495 to 565)	640 (605 to 674)	1093 (1050 to 1134)	381 (376 to 386)
	Death #, 2017	215 (183 to 251)	59 (49 to 72)	159 (139 to 182)	101 (88 to 116)	52 (48 to 56)	69 (64 to 74)	257 (227 to 290)	432 (371 to 504)	534 (459 to 621)	912 (821 to 1015)	326 (292 to 363)
	% change 1990-2017	-13.6% (-28.8% to 4.9%)	-8.5% (-27.8% to 19.1%)	-35.8% (-46.6% to -22.4%)#	-18.0% (-34.6% to 1.9%)	-20.4% (-27.1% to -12.9%)#	-7.2% (-14.1% to 0.1%)	3.9% (-8.2% to 17.4%)	-22.8% (-34.1% to -9.0%)#	-21.9% (-33.3% to -8.7%)#	-14.8% (-23.7% to -4.4%)#	1.4% (-9.4% to 12.7%)
	% change 2000-2017	-15.2% (-29.7% to 1.9%)	-21.6% (-39.2% to 2.2%)	-21.3% (-34.7% to -6.2%)#	-8.2% (-26.9% to 16.2%)	-28.1% (-33.7% to -21.6%)#	-19.8% (-25.3% to -13.7%)#	-12.8% (-22.8% to -1.4%)#	-18.4% (-31.1% to -4.1%)#	-16.7% (-28.8% to -2.1%)#	-16.6% (-25.2% to -6.6%)#	-14.4% (-22.9% to -4.6%)#
Arkansas	Death #, 1990	159 (135 to 184)	46 (31 to 64)	164 (140 to 190)	79 (66 to 91)	55 (52 to 58)	62 (61 to 64)	203 (199 to 206)	369 (345 to 396)	447 (420 to 477)	767 (734 to 804)	265 (260 to 270)
	Death #, 2000	145 (125 to 167)	41 (32 to 53)	133 (110 to 158)	69 (60 to 80)	39 (38 to 42)	59 (57 to 61)	184 (181 to 186)	319 (296 to 345)	389 (365 to 415)	671 (642 to 702)	243 (239 to 247)
	Death #, 2017	124 (105 to 146)	30 (25 to 37)	108 (94 to 125)	46 (40 to 53)	35 (33 to 38)	40 (37 to 44)	150 (133 to 171)	263 (226 to 306)	309 (265 to 359)	534 (480 to 596)	190 (171 to 213)
	% change 1990-2017	-21.6% (-37.2% to -0.8%)#	-34.4% (-55.2% to 1.4%)	-34.2% (-45.9% to -18.9%)#	-41.5% (-53.1% to -26.3%)#	-35.4% (-41.3% to -28.8%)#	-35.5% (-40.3% to -29.7%)#	-26.1% (-34.2% to -15.9%)#	-28.8% (-40.1% to -15.5%)#	-31.0% (-41.8% to -18.7%)#	-30.4% (-38.3% to -21.2%)#	-28.3% (-35.4% to -19.2%)#
	% change 2000-2017	-14.4% (-31.4% to 6.5%)	-27.4% (-47.9% to 1.1%)	-18.4% (-35.8% to 3.4%)	-33.6% (-44.9% to -19.4%)#	-10.1% (-18.3% to -0.9%)#	-31.9% (-36.9% to -25.7%)#	-18.4% (-27.6% to -6.8%)#	-17.8% (-30.9% to -2.3%)#	-20.6% (-33.0% to -6.0%)#	-20.4% (-29.3% to -9.6%)#	-21.7% (-29.7% to -12.0%)#
California	Death #, 1990	2,053 (1,863 to 2,263)	502 (420 to 582)	2,063 (1,819 to 2,315)	904 (777 to 1,047)	454 (445 to 463)	496 (491 to 502)	1,852 (1,842 to 1,862)	4,618 (4,412 to 4,828)	5,522 (5,370 to 5,683)	8,324 (8,152 to 8,505)	2,348 (2,334 to 2,362)
	Death #, 2000	1,518 (1,397 to 1,652)	394 (329 to 467)	1,094 (971 to 1,225)	579 (478 to 698)	375 (367 to 383)	458 (453 to 464)	1,356 (1,349 to 1,365)	3,006 (2,859 to 3,147)	3,585 (3,484 to 3,681)	5,774 (5,656 to 5,893)	1,815 (1,802 to 1,827)
	Death #, 2017	1,189 (1,018 to 1,388)	283 (235 to 342)	654 (571 to 753)	370 (322 to 425)	222 (206 to 240)	292 (275 to 312)	1,067 (957 to 1,184)	2,126 (1,828 to 2,476)	2,496 (2,151 to 2,897)	4,078 (3,674 to 4,556)	1,360 (1,237 to 1,495)
	% change 1990-2017	-42.1% (-52.4% to -30.5%)#	-43.6% (-55.4% to -27.5%)#	-68.3% (-73.7% to -62.0%)#	-59.1% (-66.8% to -49.8%)#	-51.1% (-54.7% to -47.0%)#	-41.0% (-44.7% to -37.0%)#	-42.4% (-48.3% to -36.1%)#	-54.0% (-61.0% to -46.0%)#	-54.8% (-61.0% to -47.4%)#	-51.0% (-55.9% to -45.1%)#	-42.1% (-47.4% to -36.5%)#
	% change 2000-2017	-21.7% (-34.4% to -5.6%)#	-28.2% (-44.1% to -6.8%)#	-40.2% (-50.3% to -28.8%)#	-36.1% (-49.1% to -18.5%)#	-40.9% (-45.4% to -35.8%)#	-36.2% (-40.2% to -31.6%)#	-21.3% (-29.6% to -12.7%)#	-29.3% (-40.0% to -16.6%)#	-30.4% (-40.1% to -18.7%)#	-29.4% (-36.3% to -20.8%)#	-25.1% (-32.0% to -17.6%)#
Colorado	Death #, 1990	216 (190 to 243)	48 (39 to 58)	198 (168 to 235)	84 (73 to 97)	50 (47 to 52)	57 (56 to 59)	191 (188 to 193)	462 (432 to 497)	546 (514 to 582)	844 (807 to 885)	248 (244 to 252)
	Death #, 2000	223 (200 to 247)	50 (40 to 62)	132 (112 to 154)	72 (62 to 83)	43 (41 to 45)	70 (68 to 72)	200 (198 to 203)	405 (379 to 430)	476 (448 to 505)	790 (756 to 823)	271 (267 to 275)
	Death #, 2017	196 (167 to 229)	42 (35 to 51)	84 (73 to 97)	63 (54 to 72)	33 (30 to 35)	56 (52 to 60)	182 (161 to 204)	323 (276 to 378)	386 (331 to 450)	657 (591 to 731)	239 (214 to 263)
	% change 1990-2017	-9.3% (-26.6% to 10.8%)	-12.0% (-32.6% to 14.7%)	-57.4% (-65.2% to -47.6%)#	-25.1% (-39.1% to -7.4%)#	-34.0% (-39.5% to -27.8%)#	-2.1% (-8.9% to 5.0%)	-4.3% (-15.3% to 6.6%)	-30.2% (-40.6% to -17.1%)#	-29.4% (-39.7% to -17.0%)#	-22.1% (-30.3% to -12.6%)#	-3.8% (-13.6% to 5.8%)
	% change 2000-2017	-12.3% (-26.8% to 6.7%)	-14.6% (-35.3% to 13.6%)	-36.0% (-49.0% to -20.6%)#	-12.1% (-27.9% to 7.9%)	-23.4% (-29.8% to -16.0%)#	-20.1% (-25.9% to -14.3%)#	-9.0% (-19.6% to 1.6%)	-20.3% (-32.5% to -6.1%)#	-19.0% (-31.3% to -5.0%)#	-16.8% (-25.8% to -6.2%)#	-11.8% (-20.9% to -3.1%)#
Connecticut	Death #, 1990	255 (226 to 288)	38 (29 to 49)	107 (88 to 127)	65 (52 to 83)	37 (36 to 39)	34 (33 to 34)	143 (141 to 145)	400 (372 to 430)	465 (439 to 494)	679 (650 to 712)	177 (174 to 179)
	Death #, 2000	169 (148 to 190)	33 (25 to 43)	63 (54 to 74)	36 (29 to 45)	29 (27 to 30)	36 (35 to 36)	107 (105 to 108)	265 (244 to 289)	301 (283 to 321)	471 (451 to 495)	142 (140 to 145)
	Death #, 2017	114 (92 to 141)	18 (13 to 25)	53 (42 to 66)	22 (13 to 33)	16 (13 to 18)	23 (20 to 25)	86 (76 to 98)	185 (152 to 225)	206 (166 to 257)	331 (285 to 387)	109 (97 to 122)
	% change 1990-2017	-55.3% (-64.9% to -43.1%)#	-51.6% (-67.7% to -27.8%)#	-50.7% (-63.1% to -34.1%)#	-67.2% (-80.3% to -46.5%)#	-58.3% (-64.0% to -51.4%)#	-33.0% (-39.5% to -25.9%)#	-39.7% (-47.0% to -31.4%)#	-53.7% (-62.2% to -43.5%)#	-55.6% (-64.3% to -44.6%)#	-51.3% (-58.4% to -42.8%)#	-38.5% (-45.3% to -30.7%)#
	% change 2000-2017	-32.6% (-48.2% to -12.6%)#	-43.3% (-60.1% to -20.4%)#	-16.9% (-35.4% to 7.8%)	-40.1% (-64.8% to -2.7%)#	-45.5% (-53.1% to -36.4%)#	-36.7% (-43.0% to -29.9%)#	-19.1% (-28.9% to -7.9%)#	-30.2% (-44.1% to -12.7%)#	-31.3% (-45.6% to -13.3%)#	-29.8% (-40.9% to -17.0%)#	-23.5% (-32.1% to -14.0%)#
Delaware	Death #, 1990	72 (64 to 79)	11 (8 to 14)	38 (33 to 44)	18 (14 to 22)	9 (9 to 10)	11 (11 to 12)	29 (28 to 29)	120 (112 to 128)	138 (129 to 148)	187 (177 to 198)	40 (39 to 41)
	Death #, 2000	56 (49 to 63)	12 (9 to 15)	32 (27 to 37)	14 (11 to 18)	9 (8 to 9)	11 (11 to 12)	35 (35 to 36)	99 (92 to 108)	113 (105 to 122)	169 (159 to 179)	47 (46 to 47)
	Death #, 2017	52 (41 to 65)	10 (6 to 15)	23 (18 to 29)	11 (8 to 15)	6 (5 to 7)	8 (8 to 9)	31 (29 to 35)	84 (68 to 103)	95 (77 to 117)	140 (119 to 166)	40 (37 to 43)

eTable 2. ENN, LNN, PNN, 1-4, 5-9, 10-14, 15-19, <1, <5, 10-19, and <20 years mortality in 1990, 2000 and 2017 with mean percentage change for all GBD causes.

	Metric	Early Neonatal	Late Neonatal	Post Neonatal	1 to 4	5 to 9	10 to 14	15 to 19	<1 year	Under 5	<20 years	10 to 19
	% change 1990-2017	**-28.0% (-43.9% to -7.6%)#**	-10.4% (-50.5% to 55.5%)	**-40.1% (-55.4% to -18.4%)#**	**-39.2% (-57.9% to -11.4%)#**	**-38.0% (-46.9% to -27.5%)#**	**-25.0% (-31.6% to -17.6%)#**	**10.1% (0.1% to 21.3%)#**	**-30.3% (-43.5% to -13.9%)#**	**-31.4% (-44.5% to -14.7%)#**	**-25.1% (-36.5% to -11.4%)#**	0.2% (-8.3% to 9.4%)
	% change 2000-2017	-7.6% (-29.1% to 21.7%)	-19.1% (-53.5% to 35.1%)	**-28.4% (-46.1% to -3.9%)#**	-21.4% (-48.2% to 17.2%)	**-35.9% (-45.5% to -23.8%)#**	**-26.3% (-33.0% to -18.9%)#**	**-10.6% (-18.6% to -1.9%)#**	-15.6% (-32.4% to 6.1%)	-16.3% (-33.6% to 5.4%)	**-16.8% (-30.1% to -0.8%)#**	**-14.4% (-21.6% to -7.0%)#**
DC	Death #, 1990	153 (137 to 170)	23 (17 to 31)	137 (119 to 158)	36 (29 to 44)	11 (10 to 11)	13 (12 to 13)	95 (93 to 97)	313 (295 to 333)	349 (331 to 369)	467 (447 to 489)	107 (105 to 110)
	Death #, 2000	67 (59 to 75)	14 (10 to 18)	58 (49 to 68)	18 (14 to 23)	9 (9 to 10)	7 (7 to 8)	48 (47 to 49)	138 (129 to 149)	156 (146 to 168)	221 (210 to 234)	56 (54 to 57)
	Death #, 2017	52 (41 to 65)	13 (7 to 20)	43 (33 to 55)	15 (10 to 21)	6 (5 to 7)	4 (4 to 5)	19 (17 to 22)	107 (88 to 131)	122 (99 to 151)	151 (126 to 182)	23 (21 to 26)
	% change 1990-2017	**-66.2% (-73.8% to -56.6%)#**	**-46.0% (-70.5% to -5.4%)#**	**-68.8% (-76.3% to -58.6%)#**	**-58.3% (-72.0% to -39.8%)#**	**-42.0% (-49.9% to -32.4%)#**	**-66.5% (-69.6% to -63.2%)#**	**-79.9% (-82.4% to -77.1%)#**	**-65.8% (-72.2% to -57.8%)#**	**-65.1% (-72.0% to -56.5%)#**	**-67.6% (-73.2% to -60.8%)#**	**-78.3% (-80.8% to -75.6%)#**
	% change 2000-2017	**-22.3% (-40.1% to -0.5%)#**	-9.1% (-50.4% to 55.9%)	**-25.8% (-44.8% to -2.9%)#**	-19.1% (-48.1% to 21.3%)	**-33.0% (-42.3% to -21.2%)#**	**-42.6% (-47.9% to -36.8%)#**	**-60.5% (-65.4% to -54.8%)#**	**-22.4% (-37.8% to -4.3%)#**	**-22.0% (-37.9% to -1.8%)#**	**-31.6% (-43.6% to -16.9%)#**	**-58.1% (-62.9% to -52.9%)#**
Florida	Death #, 1990	996 (905 to 1,101)	217 (179 to 250)	817 (717 to 923)	425 (368 to 488)	208 (202 to 213)	221 (218 to 224)	738 (732 to 744)	2,030 (1,942 to 2,119)	2,455 (2,374 to 2,539)	3,622 (3,531 to 3,717)	959 (950 to 967)
	Death #, 2000	774 (706 to 842)	211 (174 to 253)	597 (536 to 663)	313 (274 to 360)	156 (150 to 161)	228 (224 to 232)	727 (721 to 733)	1,582 (1,495 to 1,665)	1,895 (1,811 to 1,979)	3,007 (2,907 to 3,103)	955 (946 to 965)
	Death #, 2017	762 (651 to 888)	182 (151 to 221)	477 (417 to 549)	279 (242 to 321)	143 (132 to 155)	169 (157 to 181)	653 (579 to 723)	1,422 (1,221 to 1,656)	1,701 (1,465 to 1,976)	2,665 (2,389 to 2,989)	821 (740 to 904)
	% change 1990-2017	**-23.5% (-37.1% to -8.2%)#**	-15.8% (-32.8% to 9.1%)	**-41.6% (-51.6% to -30.1%)#**	**-34.4% (-46.5% to -18.9%)#**	**-31.2% (-36.9% to -25.3%)#**	**-23.6% (-28.8% to -18.0%)#**	**-11.6% (-21.6% to -1.9%)#**	**-30.0% (-40.3% to -17.9%)#**	**-30.7% (-40.7% to -19.2%)#**	**-26.4% (-34.6% to -17.5%)#**	**-14.3% (-23.0% to -5.8%)#**
	% change 2000-2017	-1.6% (-17.4% to 17.4%)	-13.4% (-33.9% to 14.3%)	**-20.1% (-33.4% to -5.3%)#**	-11.0% (-26.3% to 8.2%)	-8.3% (-15.7% to 0.2%)	**-26.0% (-31.0% to -20.8%)#**	**-10.3% (-20.4% to -0.2%)#**	-10.1% (-23.2% to 5.3%)	-10.3% (-22.7% to 5.2%)	**-11.4% (-20.8% to -0.3%)#**	**-14.0% (-22.8% to -5.2%)#**
Georgia	Death #, 1990	743 (664 to 829)	149 (125 to 171)	473 (418 to 528)	229 (196 to 268)	136 (131 to 140)	133 (131 to 135)	476 (472 to 481)	1,365 (1,298 to 1,435)	1,594 (1,525 to 1,662)	2,339 (2,262 to 2,417)	609 (603 to 615)
	Death #, 2000	566 (517 to 615)	154 (129 to 183)	377 (332 to 423)	183 (149 to 223)	112 (108 to 116)	145 (143 to 148)	466 (461 to 471)	1,096 (1,036 to 1,162)	1,279 (1,227 to 1,335)	2,002 (1,941 to 2,069)	611 (604 to 618)
	Death #, 2017	471 (401 to 549)	128 (106 to 155)	307 (268 to 354)	155 (134 to 178)	96 (89 to 104)	124 (115 to 133)	417 (369 to 469)	906 (777 to 1,057)	1,060 (912 to 1,234)	1,697 (1,511 to 1,899)	540 (486 to 601)
	% change 1990-2017	**-36.7% (-48.5% to -22.8%)#**	-13.9% (-31.7% to 11.8%)	**-35.0% (-45.4% to -21.6%)#**	**-32.4% (-45.8% to -16.4%)#**	**-29.2% (-34.9% to -22.7%)#**	-7.0% (-13.4% to 0.2%)	**-12.5% (-22.4% to -1.5%)#**	**-33.6% (-43.4% to -21.5%)#**	**-33.5% (-43.2% to -21.5%)#**	**-27.4% (-35.3% to -18.1%)#**	**-11.3% (-20.2% to -1.4%)#**
	% change 2000-2017	**-16.8% (-30.6% to -0.5%)#**	-16.9% (-35.5% to 8.2%)	**-18.4% (-32.5% to -2.2%)#**	-15.3% (-33.6% to 8.5%)	**-14.1% (-21.0% to -6.4%)#**	**-14.8% (-20.6% to -8.1%)#**	-10.5% (-20.8% to 0.9%)	**-17.4% (-30.0% to -2.6%)#**	**-17.1% (-28.8% to -2.7%)#**	**-15.2% (-24.5% to -4.5%)#**	**-11.6% (-20.5% to -1.7%)#**
Hawaii	Death #, 1990	66 (58 to 74)	15 (10 to 21)	55 (47 to 65)	24 (20 to 29)	13 (12 to 14)	16 (16 to 17)	45 (44 to 46)	136 (126 to 147)	160 (150 to 171)	234 (223 to 246)	61 (60 to 62)
	Death #, 2000	65 (58 to 74)	15 (11 to 19)	42 (36 to 48)	17 (14 to 22)	8 (7 to 8)	12 (11 to 12)	40 (39 to 40)	121 (113 to 131)	139 (130 to 148)	197 (188 to 207)	51 (50 to 52)
	Death #, 2017	56 (45 to 71)	9 (6 to 15)	29 (23 to 38)	14 (10 to 19)	10 (8 to 11)	10 (9 to 11)	33 (30 to 38)	95 (78 to 116)	109 (88 to 135)	162 (138 to 191)	43 (39 to 48)
	% change 1990-2017	-15.1% (-34.4% to 9.8%)	-37.0% (-66.5% to 13.8%)	**-46.6% (-60.4% to -29.1%)#**	**-41.0% (-59.5% to -15.3%)#**	**-25.4% (-35.9% to -12.5%)#**	**-39.8% (-45.9% to -33.2%)#**	**-25.5% (-34.3% to -15.8%)#**	**-30.3% (-44.0% to -12.8%)#**	**-31.9% (-45.3% to -13.7%)#**	**-30.8% (-41.7% to -17.1%)#**	**-29.3% (-36.8% to -21.0%)#**
	% change 2000-2017	-13.4% (-33.3% to 11.7%)	-35.0% (-62.8% to 14.1%)	**-29.8% (-48.3% to -6.0%)#**	-18.8% (-47.5% to 19.0%)	**27.5% (9.4% to 48.0%)#**	**-15.4% (-24.0% to -6.3%)#**	**-15.3% (-25.6% to -4.2%)#**	**-21.6% (-36.8% to -3.8%)#**	**-21.3% (-37.0% to -2.6%)#**	**-17.9% (-30.6% to -2.5%)#**	**-15.3% (-24.7% to -5.5%)#**
Idaho	Death #, 1990	65 (57 to 73)	13 (9 to 17)	55 (47 to 65)	29 (24 to 36)	19 (19 to 20)	33 (32 to 34)	74 (73 to 76)	133 (123 to 142)	162 (153 to 172)	289 (278 to 302)	108 (105 to 110)
	Death #, 2000	71 (62 to 80)	12 (9 to 15)	41 (35 to 49)	25 (19 to 32)	21 (20 to 22)	26 (25 to 26)	80 (78 to 82)	124 (116 to 133)	149 (141 to 158)	275 (265 to 287)	106 (104 to 108)
	Death #, 2017	55 (44 to 69)	9 (5 to 14)	29 (23 to 38)	17 (12 to 23)	15 (13 to 17)	25 (23 to 28)	69 (61 to 78)	94 (77 to 114)	110 (90 to 136)	220 (191 to 253)	94 (84 to 105)
	% change 1990-2017	-14.7% (-33.7% to 10.6%)	-27.1% (-60.1% to 29.6%)	**-46.9% (-60.1% to -30.0%)#**	**-43.6% (-62.0% to -18.0%)#**	**-24.6% (-34.6% to -12.4%)#**	**-24.9% (-32.2% to -16.6%)#**	-6.6% (-17.9% to 5.2%)	**-29.3% (-42.7% to -12.7%)#**	**-31.9% (-45.0% to -15.1%)#**	**-24.1% (-34.2% to -12.1%)#**	**-12.2% (-21.8% to -2.0%)#**
	% change 2000-2017	-21.9% (-40.0% to 0.6%)	-21.6% (-56.6% to 29.3%)	**-29.0% (-46.0% to -7.2%)#**	-34.0% (-58.0% to 0.7%)	**-28.6% (-38.5% to -17.1%)#**	-2.5% (-12.1% to 8.4%)	**-13.3% (-23.8% to -2.0%)#**	**-24.3% (-38.8% to -6.6%)#**	**-25.9% (-40.7% to -8.2%)#**	**-20.3% (-31.2% to -8.1%)#**	**-10.7% (-20.6% to -0.4%)#**
Illinois	Death #, 1990	1,015 (918 to 1,119)	216 (181 to 250)	673 (590 to 768)	329 (284 to 376)	170 (165 to 175)	206 (203 to 210)	735 (729 to 741)	1,904 (1,822 to 1,989)	2,233 (2,156 to 2,310)	3,343 (3,256 to 3,434)	941 (932 to 950)
	Death #, 2000	787 (719 to 853)	187 (157 to 221)	452 (400 to 507)	220 (192 to 252)	134 (130 to 139)	171 (169 to 174)	621 (615 to 626)	1,426 (1,363 to 1,493)	1,646 (1,582 to 1,716)	2,573 (2,498 to 2,653)	792 (784 to 800)
	Death #, 2017	520 (444 to 609)	116 (96 to 141)	264 (229 to 305)	131 (113 to 151)	87 (81 to 94)	115 (107 to 123)	445 (393 to 501)	900 (772 to 1,053)	1,031 (884 to 1,202)	1,678 (1,512 to 1,886)	560 (500 to 624)
	% change 1990-2017	**-48.7% (-57.5% to -38.6%)#**	**-46.2% (-56.9% to -31.1%)#**	**-60.8% (-67.4% to -52.8%)#**	**-60.2% (-67.5% to -51.2%)#**	**-48.4% (-52.3% to -44.3%)#**	**-44.4% (-48.1% to -40.3%)#**	**-39.4% (-46.4% to -31.6%)#**	**-52.7% (-59.7% to -44.3%)#**	**-53.8% (-60.3% to -46.2%)#**	**-49.8% (-54.9% to -43.6%)#**	**-40.5% (-46.9% to -33.6%)#**
	% change 2000-2017	**-33.9% (-44.8% to -20.9%)#**	**-37.9% (-51.5% to -19.2%)#**	**-41.6% (-51.7% to -29.4%)#**	**-40.4% (-50.6% to -27.4%)#**	**-34.9% (-39.9% to -28.9%)#**	**-33.1% (-37.5% to -27.9%)#**	**-28.3% (-36.8% to -19.1%)#**	**-36.9% (-45.8% to -25.2%)#**	**-37.4% (-46.3% to -26.6%)#**	**-34.8% (-41.5% to -26.3%)#**	**-29.3% (-36.8% to -21.2%)#**
Indiana	Death #, 1990	465 (417 to 512)	92 (77 to 107)	295 (258 to 334)	160 (138 to 183)	92 (89 to 95)	106 (104 to 109)	354 (349 to 358)	851 (804 to 897)	1,011 (965 to 1,058)	1,563 (1,510 to 1,618)	460 (454 to 466)
	Death #, 2000	363 (329 to 395)	99 (83 to 117)	220 (195 to 248)	135 (118 to 155)	77 (74 to 81)	91 (89 to 93)	340 (336 to 343)	682 (642 to 724)	817 (778 to 858)	1,325 (1,279 to 1,374)	431 (425 to 436)
	Death #, 2017	287 (245 to 335)	75 (62 to 91)	177 (154 to 205)	86 (75 to 99)	57 (53 to 62)	78 (73 to 84)	291 (259 to 327)	539 (461 to 630)	625 (536 to 727)	1,051 (941 to 1,175)	369 (332 to 410)
	% change 1990-2017	**-38.3% (-49.5% to -25.1%)#**	-18.5% (-36.0% to 5.6%)	**-39.9% (-50.0% to -27.3%)#**	**-46.0% (-56.2% to -33.9%)#**	**-37.9% (-43.3% to -32.1%)#**	**-26.8% (-32.0% to -20.9%)#**	**-17.8% (-27.3% to -7.5%)#**	**-36.7% (-46.6% to -25.2%)#**	**-38.2% (-47.9% to -27.1%)#**	**-32.8% (-40.0% to -24.5%)#**	**-19.9% (-28.1% to -10.9%)#**
	% change 2000-2017	**-20.9% (-34.3% to -4.5%)#**	**-24.7% (-41.8% to -1.6%)#**	**-19.5% (-34.5% to -3.1%)#**	**-36.1% (-47.5% to -21.1%)#**	**-26.2% (-32.4% to -19.2%)#**	**-14.2% (-20.4% to -7.5%)#**	**-14.5% (-24.1% to -3.5%)#**	**-21.0% (-33.1% to -6.2%)#**	**-23.5% (-35.0% to -10.3%)#**	**-20.7% (-29.3% to -10.5%)#**	**-14.4% (-23.0% to -4.5%)#**
Iowa	Death #, 1990	158 (135 to 184)	36 (26 to 45)	113 (98 to 129)	57 (46 to 72)	44 (42 to 46)	46 (45 to 47)	146 (143 to 148)	306 (285 to 330)	363 (344 to 385)	599 (576 to 624)	192 (189 to 195)
	Death #, 2000	116 (102 to 132)	28 (22 to 35)	76 (65 to 89)	46 (37 to 57)	35 (34 to 37)	38 (37 to 39)	156 (154 to 159)	221 (202 to 241)	266 (251 to 283)	495 (476 to 516)	193 (190 to 197)
	Death #, 2017	73 (60 to 88)	20 (14 to 27)	57 (46 to 72)	37 (24 to 56)	23 (20 to 27)	32 (29 to 35)	97 (86 to 109)	151 (125 to 181)	187 (151 to 233)	340 (293 to 391)	129 (116 to 144)
	% change 1990-2017	**-53.7% (-64.1% to -40.8%)#**	**-43.5% (-62.2% to -16.0%)#**	**-49.1% (-60.9% to -33.9%)#**	-35.7% (-60.4% to 3.1%)	**-46.8% (-54.7% to -37.8%)#**	**-30.8% (-37.8% to -23.5%)#**	**-33.2% (-41.3% to -24.9%)#**	**-50.8% (-60.4% to -40.6%)#**	**-48.4% (-58.9% to -35.4%)#**	**-43.2% (-51.5% to -33.9%)#**	**-32.6% (-39.9% to -25.0%)#**
	% change 2000-2017	**-37.0% (-51.2% to -18.9%)#**	**-28.8% (-50.0% to -0.6%)#**	**-24.9% (-41.3% to -2.5%)#**	-19.4% (-51.3% to 27.9%)	**-34.4% (-44.1% to -22.9%)#**	**-15.0% (-23.3% to -6.0%)#**	**-37.6% (-44.8% to -29.5%)#**	**-31.8% (-44.0% to -16.3%)#**	**-29.6% (-43.8% to -12.5%)#**	**-31.4% (-41.0% to -20.2%)#**	**-33.2% (-40.1% to -25.2%)#**
	Death #, 1990	151 (132 to 173)	33 (25 to 42)	116 (96 to 139)	65 (51 to 84)	43 (42 to 45)	45 (44 to 46)	153 (151 to 156)	301 (284 to 320)	366 (347 to 386)	607 (585 to 631)	198 (195 to 202)
	Death #, 2000	136 (118 to 154)	31 (24 to 40)	83 (71 to 98)	56 (44 to 69)	27 (26 to 28)	48 (46 to 49)	158 (156 to 161)	250 (229 to 273)	305 (290 to 323)	538 (520 to 560)	206 (203 to 210)

eTable 2. ENN, LNN, PNN, 1-4, 5-9, 10-14, 15-19, <1, <5, 10-19, and <20 years mortality in 1990, 2000 and 2017 with mean percentage change for all GBD causes.

	Metric	Early Neonatal	Late Neonatal	Post Neonatal	1 to 4	5 to 9	10 to 14	15 to 19	<1 year	Under 5	<20 years	10 to 19
Kansas	Death #, 2017	110 (91 to 134)	26 (18 to 35)	59 (48 to 73)	39 (24 to 59)	29 (25 to 33)	33 (30 to 36)	111 (98 to 125)	195 (162 to 235)	234 (189 to 291)	406 (351 to 477)	144 (129 to 161)
	% change 1990-2017	**-26.9% (-42.7% to -8.1%)#**	-22.6% (-48.6% to 18.1%)	**-49.1% (-61.4% to -32.6%)#**	**-40.8% (-64.4% to -5.6%)#**	**-33.8% (-42.8% to -22.8%)#**	**-26.9% (-34.2% to -19.3%)#**	**-27.6% (-36.1% to -18.4%)#**	**-35.0% (-46.9% to -21.4%)#**	**-36.0% (-48.9% to -20.0%)#**	**-33.1% (-42.5% to -21.2%)#**	**-27.5% (-35.3% to -18.7%)#**
	% change 2000-2017	-18.6% (-35.9% to 4.6%)	-16.6% (-41.6% to 18.8%)	**-29.1% (-45.3% to -7.9%)#**	-30.6% (-58.7% to 10.5%)	6.8% (-7.6% to 23.9%)	**-30.7% (-37.6% to -23.4%)#**	**-30.0% (-38.2% to -21.1%)#**	**-21.8% (-36.5% to -3.6%)#**	**-23.4% (-39.3% to -3.7%)#**	**-24.5% (-35.2% to -11.1%)#**	**-30.1% (-37.5% to -22.3%)#**
Kentucky	Death #, 1990	244 (218 to 273)	65 (53 to 77)	226 (195 to 262)	112 (93 to 134)	66 (63 to 69)	70 (68 to 72)	263 (260 to 267)	535 (510 to 563)	648 (611 to 687)	1,047 (1,004 to 1,093)	333 (328 to 338)
	Death #, 2000	204 (180 to 231)	63 (51 to 76)	156 (135 to 179)	82 (71 to 94)	50 (48 to 53)	63 (61 to 65)	228 (225 to 231)	423 (393 to 453)	504 (474 to 537)	846 (808 to 884)	291 (287 to 295)
	Death #, 2017	174 (147 to 205)	45 (37 to 57)	130 (113 to 150)	64 (55 to 74)	41 (38 to 44)	53 (49 to 57)	184 (163 to 207)	349 (300 to 408)	413 (355 to 482)	691 (619 to 775)	237 (213 to 262)
	% change 1990-2017	**-28.8% (-42.0% to -12.2%)#**	**-30.1% (-46.7% to -7.8%)#**	**-42.5% (-53.0% to -29.9%)#**	**-42.9% (-55.0% to -27.8%)#**	**-38.2% (-43.6% to -32.2%)#**	**-24.0% (-30.1% to -18.3%)#**	**-30.0% (-38.0% to -21.6%)#**	**-34.8% (-44.3% to -23.5%)#**	**-36.2% (-45.9% to -24.8%)#**	**-34.0% (-41.6% to -25.7%)#**	**-28.8% (-36.2% to -21.2%)#**
	% change 2000-2017	-14.8% (-30.0% to 4.7%)	**-28.1% (-45.9% to -5.5%)#**	-16.2% (-31.5% to 1.3%)	**-21.7% (-35.1% to -3.3%)#**	**-18.8% (-25.6% to -11.1%)#**	**-16.0% (-22.5% to -9.5%)#**	**-19.3% (-28.3% to -9.7%)#**	**-17.3% (-29.8% to -1.9%)#**	**-18.0% (-30.3% to -3.7%)#**	**-18.3% (-27.1% to -7.4%)#**	**-18.6% (-26.7% to -10.1%)#**
Louisiana	Death #, 1990	386 (349 to 429)	90 (73 to 108)	289 (253 to 328)	163 (140 to 188)	87 (83 to 90)	122 (119 to 124)	391 (386 to 395)	765 (723 to 806)	928 (881 to 972)	1,528 (1,473 to 1,581)	513 (506 to 519)
	Death #, 2000	309 (279 to 346)	87 (70 to 109)	223 (192 to 257)	131 (114 to 150)	78 (74 to 81)	93 (91 to 96)	327 (323 to 331)	619 (585 to 656)	749 (713 to 789)	1,248 (1,204 to 1,296)	421 (415 to 426)
	Death #, 2017	218 (185 to 258)	64 (53 to 79)	197 (165 to 236)	107 (93 to 124)	52 (48 to 56)	67 (63 to 72)	230 (203 to 261)	480 (411 to 558)	587 (505 to 682)	936 (838 to 1,049)	298 (266 to 332)
	% change 1990-2017	**-43.6% (-53.9% to -31.1%)#**	**-28.3% (-45.1% to -6.3%)#**	**-31.8% (-44.5% to -15.0%)#**	**-34.1% (-46.5% to -19.2%)#**	**-40.4% (-45.6% to -34.6%)#**	**-44.8% (-48.9% to -40.5%)#**	**-41.1% (-48.3% to -33.2%)#**	**-37.3% (-47.1% to -26.0%)#**	**-36.7% (-46.3% to -25.6%)#**	**-38.7% (-46.0% to -31.1%)#**	**-42.0% (-48.1% to -35.1%)#**
	% change 2000-2017	**-29.5% (-42.0% to -13.8%)#**	**-26.1% (-44.4% to -1.6%)#**	-11.4% (-29.4% to 12.6%)	-17.7% (-32.1% to 0.7%)	**-33.4% (-39.1% to -27.0%)#**	**-28.1% (-33.0% to -22.5%)#**	**-29.6% (-38.1% to -20.3%)#**	**-22.5% (-34.0% to -8.7%)#**	**-21.6% (-33.1% to -8.0%)#**	**-24.9% (-33.3% to -15.3%)#**	**-29.3% (-36.6% to -21.1%)#**
Maine	Death #, 1990	75 (66 to 85)	13 (9 to 18)	39 (33 to 47)	26 (21 to 32)	19 (18 to 20)	18 (17 to 18)	64 (62 to 65)	128 (119 to 138)	154 (144 to 164)	255 (242 to 267)	81 (80 to 83)
	Death #, 2000	52 (46 to 58)	11 (8 to 14)	22 (19 to 26)	18 (14 to 23)	13 (13 to 14)	17 (17 to 18)	57 (56 to 58)	85 (79 to 92)	103 (96 to 111)	191 (182 to 200)	74 (73 to 76)
	Death #, 2017	47 (38 to 59)	10 (6 to 16)	29 (23 to 37)	11 (8 to 16)	9 (8 to 10)	11 (10 to 12)	46 (41 to 53)	87 (71 to 106)	98 (79 to 122)	165 (142 to 191)	58 (51 to 65)
	% change 1990-2017	**-37.2% (-51.7% to -19.0%)#**	-21.8% (-57.6% to 42.0%)	-26.5% (-45.9% to 1.0%)	**-56.2% (-70.5% to -36.4%)#**	**-54.3% (-60.6% to -46.4%)#**	**-36.2% (-42.4% to -28.9%)#**	**-27.1% (-36.0% to -16.3%)#**	**-32.4% (-45.6% to -15.6%)#**	**-36.4% (-49.4% to -20.6%)#**	**-35.4% (-44.9% to -23.8%)#**	**-29.1% (-37.0% to -19.2%)#**
	% change 2000-2017	-9.3% (-30.4% to 18.0%)	-5.7% (-45.9% to 59.2%)	30.9% (-3.8% to 77.1%)	**-36.4% (-59.7% to -2.5%)#**	**-33.8% (-43.1% to -21.7%)#**	**-35.4% (-41.8% to -28.3%)#**	**-18.6% (-28.4% to -6.2%)#**	1.5% (-18.9% to 27.1%)	-5.0% (-24.7% to 19.8%)	-13.8% (-26.8% to 1.6%)	**-22.5% (-31.1% to -11.9%)#**
Maryland	Death #, 1990	410 (370 to 450)	85 (71 to 100)	247 (216 to 281)	133 (113 to 156)	64 (62 to 67)	74 (72 to 76)	289 (286 to 293)	743 (700 to 787)	876 (831 to 920)	1,303 (1,252 to 1,355)	363 (358 to 368)
	Death #, 2000	330 (303 to 360)	78 (64 to 93)	155 (137 to 175)	83 (71 to 97)	56 (54 to 59)	78 (77 to 80)	266 (262 to 269)	564 (529 to 599)	647 (611 to 683)	1,048 (1,006 to 1,089)	344 (339 to 349)
	Death #, 2017	292 (249 to 341)	59 (48 to 71)	127 (110 to 147)	79 (68 to 90)	47 (43 to 51)	51 (47 to 54)	192 (170 to 217)	477 (410 to 558)	556 (477 to 648)	845 (752 to 954)	243 (217 to 270)
	% change 1990-2017	**-28.9% (-41.1% to -12.7%)#**	**-31.3% (-46.2% to -10.7%)#**	**-48.6% (-57.2% to -38.0%)#**	**-40.9% (-52.5% to -26.4%)#**	**-27.0% (-33.2% to -20.0%)#**	**-31.6% (-36.4% to -26.0%)#**	**-33.6% (-41.3% to -24.9%)#**	**-35.7% (-45.6% to -23.5%)#**	**-36.5% (-46.1% to -25.1%)#**	**-35.1% (-42.6% to -26.3%)#**	**-33.2% (-40.0% to -25.5%)#**
	% change 2000-2017	-11.7% (-25.9% to 5.9%)	**-24.9% (-42.6% to -2.2%)#**	**-18.2% (-32.5% to -0.9%)#**	-5.7% (-23.2% to 15.8%)	**-16.9% (-24.0% to -9.0%)#**	**-35.5% (-40.3% to -30.3%)#**	**-27.7% (-36.2% to -18.3%)#**	-15.3% (-28.6% to 0.1%)	-14.1% (-27.6% to 0.9%)	**-19.3% (-28.6% to -8.4%)#**	**-29.5% (-37.0% to -21.3%)#**
Massachusetts	Death #, 1990	398 (358 to 439)	80 (66 to 94)	191 (167 to 216)	107 (92 to 122)	57 (54 to 59)	63 (62 to 65)	240 (237 to 243)	669 (627 to 713)	776 (730 to 822)	1,136 (1,084 to 1,187)	303 (299 to 307)
	Death #, 2000	268 (245 to 293)	49 (41 to 59)	95 (84 to 106)	68 (57 to 81)	47 (45 to 49)	60 (59 to 62)	173 (171 to 175)	412 (388 to 437)	480 (451 to 513)	760 (727 to 799)	233 (230 to 237)
	Death #, 2017	213 (181 to 249)	39 (32 to 48)	84 (73 to 97)	53 (46 to 61)	31 (29 to 34)	39 (36 to 41)	150 (134 to 171)	336 (288 to 394)	389 (334 to 455)	609 (542 to 686)	189 (170 to 211)
	% change 1990-2017	**-46.5% (-56.2% to -35.0%)#**	**-51.2% (-61.5% to -36.9%)#**	**-55.8% (-63.6% to -46.1%)#**	**-50.5% (-60.1% to -38.3%)#**	**-44.7% (-49.6% to -39.1%)#**	**-38.9% (-43.2% to -34.2%)#**	**-37.5% (-44.5% to -29.0%)#**	**-49.8% (-57.9% to -40.1%)#**	**-49.9% (-57.9% to -40.5%)#**	**-46.4% (-52.8% to -39.0%)#**	**-37.8% (-44.0% to -30.3%)#**
	% change 2000-2017	**-20.7% (-34.3% to -4.7%)#**	-20.2% (-38.7% to 3.6%)	-11.0% (-26.5% to 6.7%)	**-21.6% (-36.9% to -2.0%)#**	**-32.6% (-38.8% to -26.4%)#**	**-36.0% (-40.5% to -31.1%)#**	**-13.2% (-22.8% to -1.4%)#**	**-18.4% (-31.3% to -4.1%)#**	**-18.9% (-31.8% to -4.4%)#**	**-19.8% (-29.2% to -9.4%)#**	**-19.1% (-27.1% to -9.2%)#**
Michigan	Death #, 1990	885 (803 to 975)	167 (139 to 196)	499 (435 to 561)	267 (229 to 307)	153 (149 to 158)	177 (174 to 180)	642 (636 to 648)	1,551 (1,479 to 1,623)	1,818 (1,749 to 1,889)	2,791 (2,712 to 2,873)	820 (811 to 828)
	Death #, 2000	620 (573 to 672)	147 (123 to 175)	325 (290 to 363)	177 (151 to 209)	127 (123 to 131)	143 (140 to 145)	458 (454 to 463)	1,091 (1,047 to 1,136)	1,269 (1,212 to 1,325)	1,997 (1,931 to 2,062)	601 (595 to 608)
	Death #, 2017	386 (330 to 451)	97 (81 to 118)	229 (199 to 265)	111 (96 to 127)	69 (63 to 74)	102 (95 to 109)	353 (312 to 399)	713 (610 to 834)	824 (706 to 959)	1,347 (1,209 to 1,509)	455 (410 to 508)
	% change 1990-2017	**-56.4% (-64.1% to -47.7%)#**	**-41.6% (-54.0% to -24.1%)#**	**-54.0% (-61.9% to -44.6%)#**	**-58.6% (-66.5% to -48.7%)#**	**-55.3% (-59.0% to -51.3%)#**	**-42.5% (-46.4% to -38.0%)#**	**-45.0% (-51.5% to -37.6%)#**	**-54.0% (-61.1% to -45.8%)#**	**-54.7% (-61.7% to -47.0%)#**	**-51.7% (-56.9% to -45.6%)#**	**-44.5% (-50.1% to -37.9%)#**
	% change 2000-2017	**-37.7% (-48.0% to -25.4%)#**	**-33.5% (-49.0% to -13.4%)#**	**-29.4% (-41.6% to -15.9%)#**	**-37.6% (-49.2% to -22.4%)#**	**-46.0% (-50.5% to -41.2%)#**	**-28.5% (-33.5% to -23.0%)#**	**-23.0% (-32.1% to -12.9%)#**	**-34.7% (-44.3% to -23.5%)#**	**-35.1% (-44.7% to -24.4%)#**	**-32.5% (-40.0% to -24.0%)#**	**-24.3% (-31.9% to -15.3%)#**
Minnesota	Death #, 1990	233 (209 to 258)	50 (40 to 60)	165 (145 to 186)	83 (71 to 97)	65 (62 to 68)	60 (59 to 62)	211 (208 to 214)	448 (420 to 476)	531 (502 to 561)	867 (833 to 903)	271 (267 to 275)
	Death #, 2000	188 (169 to 206)	47 (37 to 61)	116 (99 to 137)	64 (52 to 78)	55 (53 to 58)	65 (63 to 66)	198 (195 to 201)	351 (327 to 375)	416 (391 to 440)	734 (703 to 763)	263 (258 to 267)
	Death #, 2017	175 (147 to 208)	41 (34 to 51)	87 (72 to 105)	51 (44 to 58)	40 (37 to 44)	47 (44 to 50)	150 (133 to 171)	304 (259 to 356)	354 (304 to 413)	591 (529 to 665)	197 (177 to 220)
	% change 1990-2017	**-25.0% (-38.8% to -7.8%)#**	-16.3% (-37.0% to 10.5%)	**-47.0% (-58.1% to -32.7%)#**	**-39.2% (-51.0% to -24.2%)#**	**-37.8% (-43.5% to -31.7%)#**	**-22.5% (-28.1% to -15.7%)#**	**-28.9% (-36.9% to -18.9%)#**	**-32.1% (-43.0% to -19.6%)#**	**-33.2% (-43.6% to -21.4%)#**	**-31.8% (-39.7% to -22.9%)#**	**-27.4% (-34.5% to -18.5%)#**
	% change 2000-2017	-6.7% (-22.8% to 13.2%)	-12.2% (-34.9% to 19.4%)	**-25.1% (-42.6% to -2.6%)#**	-21.5% (-38.8% to 0.7%)	**-27.2% (-33.8% to -20.1%)#**	**-28.0% (-33.2% to -21.9%)#**	**-24.2% (-32.8% to -14.1%)#**	-13.5% (-27.3% to 2.5%)	-14.8% (-27.9% to 0.8%)	**-19.4% (-28.7% to -8.8%)#**	**-25.2% (-32.5% to -16.1%)#**
Mississippi	Death #, 1990	271 (243 to 301)	53 (44 to 62)	207 (182 to 235)	104 (89 to 120)	72 (69 to 75)	88 (86 to 90)	250 (246 to 253)	531 (501 to 562)	635 (603 to 668)	1,045 (1,007 to 1,084)	338 (333 to 343)
	Death #, 2000	225 (204 to 246)	61 (51 to 72)	180 (159 to 203)	95 (83 to 109)	50 (48 to 53)	71 (69 to 72)	232 (229 to 236)	466 (435 to 498)	561 (528 to 596)	914 (875 to 954)	303 (298 to 308)
	Death #, 2017	152 (129 to 179)	48 (40 to 58)	131 (114 to 151)	64 (55 to 74)	35 (33 to 38)	48 (45 to 52)	165 (147 to 183)	331 (283 to 386)	395 (339 to 460)	643 (577 to 720)	213 (192 to 234)
	% change 1990-2017	**-44.0% (-54.1% to -31.7%)#**	-9.1% (-28.9% to 18.7%)	**-36.9% (-47.6% to -23.8%)#**	**-38.4% (-50.3% to -24.1%)#**	**-50.5% (-54.7% to -45.9%)#**	**-45.4% (-49.4% to -41.0%)#**	**-34.1% (-41.2% to -26.5%)#**	**-37.8% (-47.6% to -26.4%)#**	**-37.9% (-47.5% to -26.7%)#**	**-38.5% (-45.6% to -30.6%)#**	**-37.1% (-43.2% to -30.5%)#**
	% change 2000-2017	**-32.3% (-43.7% to -18.1%)#**	-21.8% (-39.9% to 2.7%)	**-27.2% (-39.3% to -12.8%)#**	**-32.8% (-44.3% to -17.6%)#**	**-29.4% (-35.5% to -22.5%)#**	**-31.6% (-36.7% to -26.4%)#**	**-29.2% (-37.0% to -20.8%)#**	**-28.9% (-39.8% to -16.1%)#**	**-29.6% (-40.2% to -17.0%)#**	**-29.6% (-37.8% to -20.6%)#**	**-29.8% (-36.8% to -22.6%)#**
	Death #, 1990	373 (336 to 412)	85 (69 to 102)	279 (244 to 315)	150 (129 to 173)	94 (91 to 98)	96 (93 to 98)	369 (365 to 373)	737 (696 to 780)	886 (844 to 930)	1445 (1394 to 1498)	465 (459 to 470)

eTable 2. ENN, LNN, PNN, 1-4, 5-9, 10-14, 15-19, <1, <5, 10-19, and <20 years mortality in 1990, 2000 and 2017 with mean percentage change for all GBD causes.

	Metric	Early Neonatal	Late Neonatal	Post Neonatal	1 to 4	5 to 9	10 to 14	15 to 19	<1 year	Under 5	<20 years	10 to 19
Missouri	Death #, 2000	303 (271 to 337)	72 (56 to 95)	185 (160 to 212)	101 (87 to 117)	80 (77 to 84)	96 (93 to 98)	367 (363 to 372)	560 (523 to 599)	661 (622 to 701)	1205 (1155 to 1254)	463 (456 to 470)
	Death #, 2017	245 (207 to 290)	48 (39 to 59)	150 (125 to 181)	83 (72 to 95)	56 (52 to 61)	68 (63 to 73)	267 (237 to 301)	443 (379 to 517)	526 (451 to 611)	917 (826 to 1024)	335 (302 to 373)
	% change 1990-2017	-34.4% (-46.3% to -19.7%)#	-43.3% (-56.7% to -25.5%)#	-46.1% (-56.6% to -32.0%)#	-44.6% (-55.1% to -31.7%)#	-40.5% (-45.5% to -34.9%)#	-28.9% (-33.9% to -23.4%)#	-27.7% (-35.8% to -18.3%)#	-39.9% (-49.1% to -29.0%)#	-40.7% (-49.6% to -30.2%)#	-36.6% (-43.1% to -28.7%)#	-28.0% (-35.1% to -19.7%)#
	% change 2000-2017	-19.3% (-32.8% to -0.7%)#	-33.6% (-51.7% to -7.6%)#	-18.7% (-35.8% to 1.9%)	-18.1% (-32.7% to 0.1%)	-30.3% (-36.3% to -23.6%)#	-28.9% (-34.0% to -23.3%)#	-27.4% (-35.6% to -18.2%)#	-21.0% (-32.9% to -6.4%)#	-20.5% (-32.1% to -6.0%)#	-23.9% (-31.8% to -14.3%)#	-27.7% (-35.1% to -19.5%)#
Montana	Death #, 1990	40 (35 to 46)	9 (6 to 12)	47 (40 to 54)	21 (17 to 26)	16 (15 to 17)	18 (17 to 18)	61 (60 to 62)	96 (89 to 103)	117 (110 to 125)	212 (203 to 223)	79 (77 to 81)
	Death #, 2000	37 (32 to 43)	8 (6 to 11)	31 (26 to 36)	17 (13 to 21)	16 (15 to 16)	19 (18 to 19)	61 (60 to 63)	76 (70 to 82)	92 (86 to 99)	188 (179 to 197)	80 (78 to 82)
	Death #, 2017	32 (25 to 40)	10 (6 to 15)	24 (18 to 31)	15 (11 to 21)	10 (8 to 11)	13 (12 to 15)	50 (45 to 57)	65 (54 to 79)	80 (65 to 99)	154 (133 to 178)	64 (57 to 71)
	% change 1990-2017	-20.5% (-39.2% to 4.4%)	5.7% (-41.0% to 83.8%)	-49.3% (-62.5% to -30.9%)#	-30.1% (-52.7% to 2.1%)	-40.5% (-49.1% to -29.3%)#	-25.4% (-32.8% to -17.3%)#	-17.6% (-27.8% to -6.5%)#	-32.0% (-45.6% to -15.4%)#	-31.7% (-45.6% to -13.6%)#	-27.7% (-38.0% to -15.1%)#	-19.3% (-28.1% to -9.3%)#
	% change 2000-2017	-13.3% (-34.0% to 14.4%)	19.4% (-32.6% to 101.1%)	-22.8% (-42.6% to 3.2%)	-11.6% (-43.3% to 33.4%)	-38.2% (-47.5% to -26.7%)#	-29.1% (-35.7% to -21.4%)#	-17.8% (-27.3% to -7.0%)#	-13.7% (-30.8% to 6.7%)	-13.3% (-31.3% to 8.9%)	-18.4% (-30.0% to -5.2%)#	-20.4% (-28.9% to -11.2%)#
Nebraska	Death #, 1990	82 (71 to 93)	24 (18 to 31)	71 (58 to 85)	37 (29 to 46)	37 (35 to 38)	30 (29 to 31)	97 (95 to 98)	176 (162 to 192)	213 (201 to 227)	376 (360 to 394)	127 (124 to 129)
	Death #, 2000	88 (77 to 99)	19 (14 to 25)	50 (42 to 59)	29 (22 to 36)	23 (21 to 24)	27 (26 to 27)	96 (94 to 97)	157 (144 to 169)	185 (173 to 198)	330 (315 to 346)	122 (120 to 124)
	Death #, 2017	68 (55 to 83)	13 (9 to 18)	34 (28 to 43)	23 (14 to 35)	16 (14 to 19)	17 (15 to 19)	71 (63 to 80)	115 (96 to 139)	138 (111 to 173)	242 (208 to 283)	88 (79 to 99)
	% change 1990-2017	-16.8% (-34.4% to 6.2%)	-44.9% (-64.2% to -16.2%)#	-51.6% (-63.9% to -35.1%)#	-38.4% (-62.5% to -2.5%)#	-55.0% (-61.6% to -47.3%)#	-43.8% (-49.2% to -37.8%)#	-26.8% (-35.0% to -17.0%)#	-34.6% (-46.9% to -19.9%)#	-35.2% (-48.6% to -18.9%)#	-35.7% (-45.3% to -24.4%)#	-30.9% (-38.3% to -22.6%)#
	% change 2000-2017	-22.5% (-39.5% to -0.1%)#	-30.8% (-51.6% to 1.2%)	-31.5% (-47.4% to -10.6%)#	-21.2% (-54.0% to 29.1%)	-27.4% (-38.1% to -14.4%)#	-35.8% (-42.1% to -29.2%)#	-26.1% (-34.4% to -16.1%)#	-26.4% (-40.3% to -8.9%)#	-25.6% (-40.8% to -4.6%)#	-26.7% (-37.2% to -12.8%)#	-28.2% (-35.8% to -19.3%)#
Nevada	Death #, 1990	64 (56 to 72)	18 (13 to 24)	88 (76 to 103)	36 (29 to 44)	19 (18 to 20)	22 (22 to 23)	78 (77 to 79)	170 (159 to 182)	206 (194 to 219)	326 (312 to 341)	100 (98 to 102)
	Death #, 2000	83 (73 to 94)	29 (21 to 38)	81 (69 to 94)	39 (31 to 50)	26 (24 to 27)	27 (27 to 28)	94 (93 to 96)	193 (180 to 208)	233 (218 to 247)	380 (364 to 396)	122 (120 to 124)
	Death #, 2017	98 (79 to 122)	20 (12 to 32)	61 (48 to 77)	41 (29 to 56)	23 (20 to 27)	32 (30 to 35)	114 (101 to 128)	180 (149 to 216)	220 (180 to 271)	390 (339 to 450)	146 (131 to 162)
	% change 1990-2017	54.8% (20.4% to 98.9%)#	12.2% (-39.2% to 101.7%)	-31.2% (-47.1% to -9.8%)#	13.3% (-22.6% to 63.3%)	20.3% (3.9% to 40.1%)#	45.6% (32.6% to 60.0%)#	45.6% (29.0% to 64.2%)#	5.6% (-13.5% to 28.8%)	7.0% (-13.6% to 32.9%)	19.7% (3.0% to 39.6%)#	45.6% (30.3% to 61.5%)#
	% change 2000-2017	18.7% (-7.9% to 52.3%)	-29.1% (-59.6% to 19.7%)	-25.4% (-44.0% to -1.6%)#	3.1% (-32.7% to 53.6%)	-9.6% (-22.0% to 5.1%)	18.7% (8.0% to 30.7%)#	20.7% (6.7% to 35.4%)#	-7.0% (-24.6% to 13.8%)	-5.3% (-23.9% to 18.6%)	2.6% (-11.7% to 20.1%)	20.2% (7.7% to 33.1%)#
New Hampshire	Death #, 1990	68 (59 to 77)	14 (10 to 18)	44 (37 to 52)	18 (15 to 23)	14 (13 to 15)	12 (12 to 13)	50 (49 to 51)	126 (116 to 136)	144 (135 to 154)	221 (210 to 232)	63 (61 to 64)
	Death #, 2000	48 (42 to 55)	10 (7 to 13)	21 (17 to 25)	13 (10 to 16)	9 (9 to 10)	15 (15 to 16)	47 (46 to 48)	79 (72 to 86)	92 (85 to 99)	164 (155 to 172)	62 (61 to 64)
	Death #, 2017	34 (26 to 43)	8 (5 to 13)	18 (14 to 23)	9 (6 to 12)	7 (6 to 8)	10 (9 to 11)	33 (29 to 37)	60 (48 to 74)	69 (55 to 86)	118 (101 to 137)	42 (38 to 47)
	% change 1990-2017	-50.5% (-62.7% to -35.6%)#	-40.9% (-66.6% to 3.0%)	-60.0% (-70.9% to -45.3%)#	-50.9% (-66.2% to -29.1%)#	-52.3% (-59.5% to -43.5%)#	-22.5% (-29.9% to -14.8%)#	-34.9% (-42.6% to -26.4%)#	-52.8% (-62.2% to -40.8%)#	-52.5% (-62.4% to -40.8%)#	-46.8% (-54.6% to -37.4%)#	-32.5% (-39.4% to -24.8%)#
	% change 2000-2017	-30.4% (-46.9% to -8.8%)#	-18.3% (-55.6% to 41.1%)	-15.1% (-37.5% to 13.5%)	-29.9% (-54.0% to 6.0%)	-27.4% (-38.3% to -14.4%)#	-37.8% (-44.0% to -31.6%)#	-30.1% (-38.3% to -21.1%)#	-24.8% (-40.5% to -5.3%)#	-25.5% (-41.5% to -5.8%)#	-28.1% (-38.9% to -15.5%)#	-32.0% (-38.9% to -24.1%)#
New Jersey	Death #, 1990	605 (544 to 666)	115 (96 to 134)	372 (323 to 421)	194 (166 to 225)	97 (93 to 100)	101 (99 to 103)	306 (303 to 309)	1,092 (1,030 to 1,156)	1,286 (1,230 to 1,340)	1,790 (1,728 to 1,850)	407 (403 to 412)
	Death #, 2000	392 (355 to 428)	93 (77 to 109)	213 (187 to 239)	105 (86 to 128)	71 (68 to 74)	84 (82 to 86)	250 (247 to 253)	697 (653 to 741)	802 (760 to 844)	1,207 (1,159 to 1,255)	334 (330 to 338)
	Death #, 2017	250 (213 to 293)	59 (49 to 71)	135 (117 to 156)	76 (66 to 88)	49 (45 to 52)	62 (57 to 66)	205 (180 to 229)	444 (380 to 520)	520 (445 to 607)	835 (746 to 936)	266 (239 to 294)
	% change 1990-2017	-58.6% (-66.1% to -49.8%)#	-48.7% (-59.8% to -33.4%)#	-63.7% (-70.1% to -55.9%)#	-60.7% (-68.1% to -51.8%)#	-49.8% (-54.0% to -45.3%)#	-38.9% (-43.2% to -34.2%)#	-33.2% (-41.2% to -25.2%)#	-59.3% (-65.7% to -52.0%)#	-59.5% (-65.8% to -52.6%)#	-53.3% (-58.7% to -47.8%)#	-34.6% (-41.5% to -27.8%)#
	% change 2000-2017	-36.1% (-47.2% to -23.0%)#	-36.3% (-50.2% to -17.9%)#	-36.6% (-47.7% to -22.9%)#	-27.3% (-43.1% to -6.8%)#	-31.4% (-36.9% to -24.9%)#	-26.7% (-31.8% to -21.1%)#	-18.1% (-27.9% to -8.1%)#	-36.3% (-45.9% to -24.2%)#	-35.1% (-44.6% to -23.2%)#	-30.8% (-38.5% to -21.9%)#	-20.3% (-28.5% to -11.7%)#
New Mexico	Death #, 1990	100 (86 to 115)	27 (20 to 35)	93 (78 to 113)	53 (40 to 69)	37 (36 to 39)	39 (38 to 40)	148 (146 to 151)	221 (208 to 234)	273 (257 to 292)	498 (478 to 520)	187 (184 to 190)
	Death #, 2000	76 (67 to 86)	27 (21 to 33)	69 (58 to 82)	38 (29 to 48)	22 (21 to 23)	35 (34 to 36)	137 (135 to 140)	172 (159 to 186)	210 (196 to 226)	404 (387 to 422)	172 (169 to 175)
	Death #, 2017	64 (52 to 77)	21 (15 to 29)	43 (35 to 54)	32 (21 to 49)	20 (17 to 23)	29 (26 to 32)	100 (88 to 112)	128 (106 to 154)	160 (130 to 200)	308 (266 to 359)	128 (115 to 143)
	% change 1990-2017	-36.1% (-50.0% to -18.7%)#	-22.4% (-47.5% to 17.2%)	-54.0% (-65.0% to -38.5%)#	-38.5% (-63.0% to -0.9%)#	-47.4% (-54.9% to -38.5%)#	-26.6% (-34.1% to -18.1%)#	-32.8% (-40.5% to -24.4%)#	-42.0% (-52.5% to -29.9%)#	-41.3% (-53.1% to -26.3%)#	-38.1% (-46.8% to -27.8%)#	-31.5% (-38.8% to -23.5%)#
	% change 2000-2017	-16.8% (-33.3% to 5.3%)	-20.3% (-44.9% to 14.2%)	-37.7% (-53.0% to -18.9%)#	-13.5% (-48.9% to 39.3%)	-10.8% (-24.4% to 4.2%)	-17.7% (-26.1% to -8.6%)#	-27.3% (-35.9% to -18.5%)#	-25.7% (-39.2% to -8.9%)#	-23.5% (-39.7% to -3.7%)#	-23.6% (-34.6% to -11.0%)#	-25.4% (-33.5% to -17.0%)#
New York	Death #, 1990	1,565 (1,421 to 1,726)	304 (253 to 355)	1,020 (895 to 1,148)	484 (416 to 564)	253 (247 to 259)	266 (263 to 270)	944 (938 to 951)	2,889 (2,765 to 3,015)	3,372 (3,266 to 3,482)	4,836 (4,718 to 4,961)	1,211 (1,201 to 1,221)
	Death #, 2000	950 (870 to 1,026)	231 (194 to 273)	531 (467 to 600)	289 (239 to 352)	177 (171 to 182)	205 (201 to 208)	631 (626 to 636)	1,712 (1,623 to 1,801)	2,001 (1,927 to 2,078)	3,014 (2,930 to 3,101)	836 (828 to 844)
	Death #, 2017	593 (507 to 692)	156 (130 to 188)	359 (312 to 413)	197 (172 to 227)	108 (100 to 117)	130 (121 to 140)	418 (370 to 470)	1,107 (952 to 1,292)	1,305 (1,123 to 1,515)	1,961 (1,747 to 2,205)	548 (495 to 610)
	% change 1990-2017	-62.1% (-68.7% to -53.9%)#	-48.7% (-59.2% to -33.9%)#	-64.8% (-70.8% to -57.7%)#	-59.2% (-66.9% to -50.0%)#	-57.2% (-60.5% to -53.5%)#	-51.1% (-54.5% to -47.3%)#	-55.7% (-60.8% to -50.2%)#	-61.7% (-67.4% to -54.9%)#	-61.3% (-66.8% to -54.6%)#	-59.4% (-63.8% to -54.2%)#	-54.7% (-59.2% to -49.5%)#
	% change 2000-2017	-37.6% (-48.0% to -25.4%)#	-32.5% (-47.1% to -12.8%)#	-32.5% (-44.7% to -18.9%)#	-31.8% (-46.6% to -13.1%)#	-38.7% (-43.6% to -33.4%)#	-36.4% (-40.9% to -31.6%)#	-33.8% (-41.5% to -25.2%)#	-35.3% (-45.0% to -24.3%)#	-34.8% (-44.1% to -23.9%)#	-34.9% (-42.3% to -26.5%)#	-34.4% (-41.0% to -26.8%)#
N Carolina	Death #, 1990	680 (616 to 751)	122 (101 to 143)	388 (340 to 439)	206 (177 to 237)	109 (105 to 113)	128 (125 to 130)	440 (435 to 444)	1,190 (1,125 to 1,254)	1,396 (1,332 to 1,460)	2,073 (2,000 to 2,144)	568 (561 to 574)
	Death #, 2000	612 (563 to 664)	139 (114 to 166)	319 (284 to 358)	154 (135 to 177)	99 (96 to 103)	127 (124 to 129)	402 (398 to 406)	1,070 (1,014 to 1,125)	1,224 (1,169 to 1,281)	1,852 (1,789 to 1,915)	528 (523 to 534)
	Death #, 2017	455 (389 to 532)	109 (91 to 132)	261 (227 to 301)	139 (120 to 160)	82 (76 to 89)	104 (97 to 111)	370 (330 to 416)	826 (708 to 964)	965 (828 to 1,123)	1,521 (1,363 to 1,702)	474 (428 to 525)
	% change 1990-2017	-33.1% (-44.6% to -18.9%)#	-10.7% (-29.5% to 16.0%)	-32.7% (-44.0% to -19.1%)#	-32.5% (-45.3% to -16.5%)#	-24.7% (-30.5% to -17.8%)#	-18.8% (-24.4% to -12.6%)#	-15.8% (-25.2% to -5.4%)#	-30.6% (-40.8% to -17.6%)#	-30.9% (-40.7% to -18.4%)#	-26.6% (-34.4% to -16.9%)#	-16.4% (-24.5% to -7.5%)#
	% change 2000-2017	-25.6% (-37.4% to -11.0%)#	-21.3% (-40.1% to 2.7%)	-18.2% (-32.2% to -1.6%)#	-10.0% (-25.8% to 9.9%)	-17.0% (-23.6% to -9.5%)#	-17.9% (-23.3% to -11.8%)#	-7.8% (-17.9% to 3.6%)	-22.8% (-34.5% to -9.1%)#	-21.2% (-33.4% to -7.5%)#	-17.9% (-26.6% to -7.6%)#	-10.2% (-18.9% to -0.6%)#

eTable 2. ENN, LNN, PNN, 1-4, 5-9, 10-14, 15-19, <1, <5, 10-19, and <20 years mortality in 1990, 2000 and 2017 with mean percentage change for all GBD causes.

	Metric	Early Neonatal	Late Neonatal	Post Neonatal	1 to 4	5 to 9	10 to 14	15 to 19	<1 year	Under 5	<20 years	10 to 19
N Dakota	Death #, 1990	36 (31 to 40)	10 (7 to 13)	27 (23 to 31)	14 (12 to 18)	13 (13 to 14)	12 (12 to 13)	33 (32 to 34)	72 (66 to 78)	86 (80 to 93)	145 (137 to 153)	45 (44 to 46)
	Death #, 2000	30 (26 to 35)	7 (5 to 9)	20 (17 to 24)	9 (7 to 11)	5 (5 to 6)	11 (11 to 12)	34 (33 to 35)	57 (51 to 63)	65 (60 to 71)	116 (110 to 122)	45 (44 to 46)
	Death #, 2017	29 (23 to 37)	5 (3 to 8)	22 (17 to 28)	9 (6 to 13)	5 (4 to 6)	6 (5 to 6)	29 (27 to 32)	56 (46 to 69)	65 (53 to 81)	105 (90 to 123)	35 (32 to 38)
	% change 1990-2017	-17.5% (-36.9% to 7.0%)	-44.1% (-69.7% to -0.7%)#	-18.7% (-39.5% to 9.9%)	-37.5% (-58.0% to -7.8%)#	-64.5% (-69.8% to -58.1%)#	-52.5% (-56.9% to -47.9%)#	-11.5% (-19.7% to -2.8%)#	-21.5% (-36.8% to -1.2%)#	-24.2% (-39.6% to -4.3%)#	-27.4% (-38.6% to -13.5%)#	-22.6% (-29.6% to -15.3%)#
	% change 2000-2017	-2.0% (-26.4% to 31.1%)	-20.3% (-56.5% to 40.5%)	8.1% (-17.7% to 41.8%)	5.2% (-32.5% to 57.6%)	-8.1% (-22.6% to 8.3%)	-48.6% (-53.3% to -43.4%)#	-13.4% (-21.3% to -4.8%)#	-0.6% (-21.6% to 25.1%)	0.1% (-20.8% to 26.4%)	-8.9% (-23.0% to 7.7%)	-22.2% (-29.1% to -14.8%)#
Ohio	Death #, 1990	870 (786 to 960)	178 (147 to 208)	560 (491 to 629)	282 (244 to 323)	177 (172 to 182)	194 (191 to 197)	585 (580 to 591)	1,609 (1,540 to 1,685)	1,891 (1,824 to 1,959)	2,847 (2,768 to 2,925)	779 (771 to 787)
	Death #, 2000	665 (609 to 721)	162 (136 to 193)	369 (329 to 411)	193 (168 to 221)	129 (125 to 133)	164 (162 to 167)	484 (479 to 488)	1,196 (1,141 to 1,249)	1,389 (1,335 to 1,440)	2,165 (2,099 to 2,226)	648 (641 to 655)
	Death #, 2017	526 (449 to 616)	111 (92 to 135)	284 (247 to 328)	138 (120 to 159)	84 (77 to 90)	119 (110 to 127)	413 (365 to 464)	922 (792 to 1,078)	1,061 (910 to 1,235)	1,676 (1,500 to 1,873)	532 (478 to 590)
	% change 1990-2017	-39.5% (-50.4% to -27.1%)#	-37.5% (-50.7% to -19.0%)#	-49.2% (-58.2% to -38.9%)#	-51.0% (-60.2% to -39.6%)#	-52.8% (-56.7% to -48.7%)#	-38.7% (-43.0% to -34.3%)#	-29.4% (-37.4% to -20.6%)#	-42.7% (-51.7% to -32.5%)#	-43.9% (-52.2% to -34.2%)#	-41.1% (-47.7% to -33.8%)#	-31.7% (-38.6% to -24.3%)#
	% change 2000-2017	-20.8% (-33.7% to -5.0%)#	-31.3% (-47.0% to -10.8%)#	-22.9% (-36.2% to -8.3%)#	-28.1% (-40.1% to -12.2%)#	-35.1% (-40.2% to -29.1%)#	-27.8% (-32.9% to -22.5%)#	-14.5% (-24.5% to -3.8%)#	-22.9% (-34.6% to -9.2%)#	-23.6% (-34.7% to -10.3%)#	-22.6% (-30.9% to -12.9%)#	-17.9% (-26.3% to -9.1%)#
Oklahoma	Death #, 1990	191 (165 to 221)	56 (43 to 70)	193 (166 to 222)	106 (92 to 123)	62 (60 to 66)	66 (65 to 68)	239 (235 to 242)	439 (410 to 470)	545 (515 to 580)	913 (876 to 954)	305 (300 to 310)
	Death #, 2000	181 (158 to 205)	62 (49 to 77)	161 (134 to 191)	80 (69 to 92)	48 (46 to 51)	65 (63 to 67)	217 (214 to 220)	404 (378 to 434)	484 (454 to 516)	813 (778 to 851)	281 (277 to 286)
	Death #, 2017	182 (154 to 213)	46 (38 to 56)	135 (118 to 156)	79 (68 to 91)	49 (45 to 53)	55 (51 to 59)	194 (172 to 218)	363 (311 to 424)	442 (380 to 514)	741 (666 to 826)	250 (224 to 276)
	% change 1990-2017	-4.8% (-23.2% to 18.9%)	-16.3% (-38.8% to 16.7%)	-29.9% (-42.6% to -14.2%)#	-25.8% (-40.3% to -8.1%)#	-21.3% (-28.3% to -13.8%)#	-16.6% (-22.8% to -10.1%)#	-18.7% (-28.4% to -8.3%)#	-17.2% (-29.8% to -2.2%)#	-18.9% (-31.0% to -4.8%)#	-18.9% (-27.8% to -8.8%)#	-18.2% (-26.9% to -9.2%)#
	% change 2000-2017	0.2% (-17.7% to 22.5%)	-24.6% (-43.4% to 2.9%)	-16.2% (-32.5% to 4.5%)	-1.2% (-18.7% to 21.0%)	2.1% (-6.6% to 12.3%)	-14.6% (-20.8% to -8.0%)#	-10.3% (-20.5% to 0.8%)	-10.1% (-23.5% to 6.2%)	-8.7% (-21.8% to 7.6%)	-8.9% (-18.5% to 2.2%)	-11.3% (-20.3% to -1.7%)#
Oregon	Death #, 1990	152 (133 to 173)	35 (26 to 44)	153 (129 to 181)	71 (55 to 91)	41 (40 to 43)	49 (48 to 51)	171 (168 to 173)	340 (320 to 363)	411 (390 to 435)	672 (648 to 700)	220 (217 to 224)
	Death #, 2000	139 (122 to 157)	38 (30 to 47)	89 (75 to 105)	54 (43 to 68)	37 (36 to 39)	43 (42 to 44)	147 (145 to 149)	266 (245 to 287)	320 (302 to 339)	547 (525 to 570)	190 (187 to 193)
	Death #, 2017	126 (103 to 155)	28 (20 to 39)	76 (61 to 94)	36 (23 to 55)	22 (19 to 25)	34 (31 to 38)	113 (100 to 128)	231 (191 to 278)	266 (215 to 332)	436 (374 to 511)	148 (133 to 164)
	% change 1990-2017	-16.8% (-35.1% to 5.5%)	-18.3% (-45.0% to 20.6%)	-50.6% (-62.0% to -34.2%)#	-49.2% (-69.5% to -18.1%)#	-48.0% (-55.2% to -39.2%)#	-30.5% (-36.9% to -23.2%)#	-33.7% (-41.2% to -25.1%)#	-32.2% (-45.0% to -18.0%)#	-35.1% (-48.0% to -18.9%)#	-35.2% (-44.8% to -23.9%)#	-33.0% (-39.9% to -25.1%)#
	% change 2000-2017	-9.1% (-27.9% to 16.0%)	-25.3% (-47.1% to 4.5%)	-14.8% (-36.5% to 12.2%)	-33.6% (-60.7% to 8.5%)	-42.2% (-50.3% to -32.3%)#	-20.0% (-27.4% to -11.4%)#	-22.9% (-31.7% to -12.6%)#	-13.3% (-28.9% to 7.0%)	-16.8% (-33.0% to 3.7%)	-20.4% (-31.8% to -6.7%)#	-22.2% (-30.3% to -12.9%)#
Pennsylvania	Death #, 1990	965 (874 to 1,064)	189 (155 to 220)	523 (458 to 587)	293 (253 to 336)	165 (160 to 170)	173 (170 to 175)	557 (552 to 562)	1,677 (1,600 to 1,758)	1,970 (1,895 to 2,041)	2,865 (2,782 to 2,945)	729 (723 to 737)
	Death #, 2000	652 (597 to 703)	135 (113 to 160)	310 (273 to 349)	182 (160 to 209)	136 (132 to 140)	148 (146 to 151)	532 (527 to 536)	1,096 (1,043 to 1,155)	1,278 (1,228 to 1,335)	2,094 (2,035 to 2,160)	680 (673 to 686)
	Death #, 2017	515 (440 to 602)	103 (85 to 125)	242 (211 to 280)	131 (114 to 151)	81 (75 to 88)	100 (94 to 108)	418 (369 to 470)	861 (740 to 1,007)	992 (851 to 1,156)	1,592 (1,435 to 1,786)	519 (464 to 576)
	% change 1990-2017	-46.6% (-56.1% to -35.8%)#	-45.3% (-56.8% to -29.4%)#	-53.6% (-61.8% to -44.1%)#	-55.2% (-63.7% to -44.8%)#	-50.8% (-54.8% to -46.5%)#	-41.8% (-45.8% to -37.4%)#	-24.9% (-33.6% to -15.5%)#	-48.6% (-56.8% to -39.4%)#	-49.6% (-57.0% to -40.9%)#	-44.4% (-50.4% to -37.0%)#	-28.9% (-36.5% to -21.0%)#
	% change 2000-2017	-20.9% (-33.7% to -5.8%)#	-23.8% (-41.5% to -0.4%)#	-21.7% (-35.5% to -6.1%)#	-27.9% (-40.4% to -12.1%)#	-40.1% (-45.0% to -34.8%)#	-32.3% (-37.1% to -27.6%)#	-21.3% (-30.6% to -11.6%)#	-21.5% (-33.5% to -7.8%)#	-22.4% (-34.2% to -9.3%)#	-24.0% (-32.0% to -14.9%)#	-23.7% (-31.7% to -15.3%)#
Rhode Island	Death #, 1990	84 (74 to 95)	15 (10 to 20)	38 (33 to 44)	18 (15 to 23)	11 (10 to 11)	13 (13 to 14)	35 (34 to 36)	137 (127 to 148)	155 (145 to 167)	214 (203 to 227)	48 (47 to 49)
	Death #, 2000	63 (55 to 71)	8 (6 to 10)	23 (20 to 27)	11 (9 to 15)	12 (11 to 13)	11 (10 to 11)	36 (35 to 37)	94 (86 to 102)	105 (97 to 114)	164 (155 to 174)	47 (46 to 48)
	Death #, 2017	45 (35 to 56)	7 (4 to 10)	17 (13 to 22)	9 (6 to 12)	4 (4 to 5)	7 (6 to 7)	24 (21 to 27)	68 (56 to 84)	77 (62 to 95)	112 (94 to 133)	31 (27 to 34)
	% change 1990-2017	-47.1% (-58.9% to -32.8%)#	-54.9% (-75.6% to -18.4%)#	-55.5% (-66.3% to -40.5%)#	-53.5% (-68.9% to -31.5%)#	-58.2% (-64.2% to -51.3%)#	-50.4% (-55.1% to -45.0%)#	-31.3% (-39.1% to -21.8%)#	-50.2% (-60.1% to -38.7%)#	-50.6% (-60.8% to -38.6%)#	-47.8% (-56.2% to -38.0%)#	-36.6% (-43.2% to -28.9%)#
	% change 2000-2017	-28.7% (-45.4% to -7.9%)#	-16.9% (-54.8% to 47.5%)	-27.2% (-45.3% to -4.0%)#	-24.2% (-53.0% to 14.8%)	-63.8% (-69.1% to -57.6%)#	-37.3% (-43.2% to -30.3%)#	-33.8% (-41.3% to -24.5%)#	-27.4% (-42.7% to -8.8%)#	-27.0% (-42.6% to -7.8%)#	-31.9% (-42.8% to -18.3%)#	-34.6% (-41.6% to -26.7%)#
S Carolina	Death #, 1990	386 (345 to 433)	78 (64 to 91)	248 (216 to 280)	129 (111 to 149)	69 (66 to 72)	81 (80 to 83)	264 (261 to 268)	712 (671 to 756)	840 (799 to 885)	1,255 (1,206 to 1,306)	346 (341 to 351)
	Death #, 2000	301 (275 to 327)	67 (56 to 79)	159 (139 to 179)	81 (70 to 94)	61 (58 to 64)	66 (64 to 67)	251 (248 to 254)	526 (495 to 557)	607 (574 to 642)	985 (945 to 1,025)	317 (312 to 321)
	Death #, 2017	221 (189 to 258)	51 (42 to 62)	128 (111 to 147)	75 (65 to 87)	46 (42 to 49)	50 (47 to 54)	220 (194 to 247)	400 (342 to 468)	475 (408 to 553)	790 (707 to 882)	270 (243 to 300)
	% change 1990-2017	-42.7% (-53.1% to -30.3%)#	-34.9% (-48.8% to -15.6%)#	-48.4% (-57.1% to -37.6%)#	-41.6% (-52.7% to -27.8%)#	-33.3% (-38.9% to -27.1%)#	-38.5% (-42.9% to -33.7%)#	-16.9% (-26.7% to -6.6%)#	-43.8% (-52.7% to -33.5%)#	-43.5% (-52.2% to -33.3%)#	-37.0% (-44.1% to -29.6%)#	-22.0% (-30.2% to -13.2%)#
	% change 2000-2017	-26.4% (-38.7% to -12.1%)#	-24.4% (-41.1% to -0.1%)#	-19.4% (-33.4% to -1.7%)#	-7.3% (-24.6% to 14.4%)	-25.1% (-31.5% to -17.9%)#	-23.7% (-29.3% to -17.6%)#	-12.5% (-22.4% to -1.6%)#	-24.1% (-35.5% to -10.1%)#	-21.8% (-33.3% to -7.6%)#	-19.8% (-28.8% to -9.4%)#	-14.8% (-23.6% to -5.3%)#
S Dakota	Death #, 1990	42 (36 to 47)	9 (6 to 11)	43 (37 to 50)	20 (17 to 25)	18 (17 to 19)	16 (16 to 16)	52 (51 to 53)	93 (86 to 100)	114 (106 to 122)	199 (190 to 209)	68 (66 to 69)
	Death #, 2000	32 (28 to 36)	6 (5 to 8)	33 (28 to 38)	15 (12 to 19)	13 (12 to 13)	21 (20 to 21)	51 (50 to 53)	71 (66 to 76)	86 (79 to 92)	170 (162 to 179)	72 (70 to 74)
	Death #, 2017	33 (26 to 42)	7 (4 to 10)	25 (19 to 32)	12 (9 to 17)	12 (10 to 13)	14 (13 to 15)	43 (39 to 47)	65 (53 to 79)	77 (62 to 96)	145 (127 to 168)	57 (52 to 61)
	% change 1990-2017	-20.0% (-38.6% to 2.7%)	-22.8% (-57.8% to 38.1%)	-42.4% (-57.0% to -22.7%)#	-39.9% (-58.6% to -13.1%)#	-33.9% (-43.1% to -22.3%)#	-14.2% (-21.6% to -5.8%)#	-17.1% (-24.1% to -10.0%)#	-30.6% (-44.3% to -13.7%)#	-32.2% (-46.1% to -15.0%)#	-27.0% (-36.9% to -15.0%)#	-16.4% (-23.2% to -9.2%)#
	% change 2000-2017	4.6% (-19.9% to 37.0%)	5.4% (-39.0% to 72.5%)	-23.9% (-43.5% to 1.4%)	-17.8% (-47.3% to 22.7%)	-6.9% (-20.2% to 9.7%)	-33.8% (-39.8% to -27.2%)#	-16.6% (-23.9% to -9.2%)#	-8.5% (-25.8% to 13.1%)	-10.1% (-28.6% to 12.5%)	-14.7% (-26.6% to -0.7%)#	-21.6% (-27.9% to -14.9%)#
Tennessee	Death #, 1990	424 (382 to 466)	105 (88 to 123)	326 (285 to 371)	154 (132 to 179)	84 (81 to 87)	100 (98 to 102)	356 (351 to 360)	855 (806 to 902)	1,009 (958 to 1,060)	1,549 (1,489 to 1,607)	456 (450 to 461)
	Death #, 2000	373 (340 to 409)	100 (82 to 119)	274 (243 to 307)	127 (110 to 146)	74 (71 to 77)	109 (107 to 112)	353 (349 to 357)	748 (704 to 792)	875 (828 to 921)	1,411 (1,356 to 1,466)	462 (456 to 468)
	Death #, 2017	246 (210 to 289)	75 (62 to 91)	214 (187 to 247)	87 (75 to 101)	57 (53 to 62)	77 (71 to 83)	273 (242 to 308)	536 (459 to 625)	623 (536 to 725)	1,030 (924 to 1,155)	350 (315 to 389)
	% change 1990-2017	-41.8% (-52.2% to -28.9%)#	-28.8% (-44.4% to -7.2%)#	-34.3% (-45.3% to -20.8%)#	-43.4% (-54.3% to -30.2%)#	-32.1% (-37.9% to -25.7%)#	-23.2% (-28.9% to -17.2%)#	-23.3% (-32.1% to -13.4%)#	-37.4% (-46.9% to -26.0%)#	-38.3% (-47.6% to -27.2%)#	-33.5% (-40.8% to -25.2%)#	-23.3% (-31.0% to -14.5%)#

eTable 2. ENN, LNN, PNN, 1-4, 5-9, 10-14, 15-19, <1, <5, 10-19, and <20 years mortality in 1990, 2000 and 2017 with mean percentage change for all GBD causes.

	Metric	Early Neonatal	Late Neonatal	Post Neonatal	1 to 4	5 to 9	10 to 14	15 to 19	<1 year	Under 5	<20 years	10 to 19
	% change 2000-2017	**-34.0% (-44.9% to -21.0%)#**	**-25.4% (-43.0% to -2.6%)#**	**-21.9% (-35.3% to -7.3%)#**	**-31.2% (-43.1% to -15.9%)#**	**-23.1% (-29.3% to -16.3%)#**	**-29.8% (-34.9% to -24.5%)#**	**-22.6% (-31.2% to -12.3%)#**	**-28.4% (-39.3% to -16.0%)#**	**-28.8% (-39.5% to -16.6%)#**	**-27.0% (-34.5% to -18.0%)#**	**-24.3% (-31.9% to -15.4%)#**
Texas	Death #, 1990	1,182 (1,069 to 1,299)	300 (252 to 351)	1,012 (886 to 1,147)	563 (480 to 664)	318 (310 to 326)	365 (360 to 370)	1,266 (1,257 to 1,273)	2,495 (2,377 to 2,615)	3,059 (2,965 to 3,160)	5,007 (4,897 to 5,126)	1,631 (1,618 to 1,642)
	Death #, 2000	920 (838 to 999)	288 (240 to 341)	798 (707 to 892)	453 (389 to 530)	291 (283 to 298)	338 (333 to 343)	1,202 (1,194 to 1,210)	2,006 (1,907 to 2,104)	2,459 (2,373 to 2,543)	4,290 (4,188 to 4,392)	1,540 (1,529 to 1,552)
	Death #, 2017	1,018 (871 to 1,189)	293 (243 to 354)	717 (628 to 825)	402 (350 to 463)	252 (234 to 273)	304 (285 to 324)	1,042 (932 to 1,163)	2,029 (1,743 to 2,361)	2,431 (2,097 to 2,829)	4,029 (3,627 to 4,488)	1,346 (1,220 to 1,481)
	% change 1990-2017	-13.9% (-29.0% to 4.1%)	-2.5% (-22.9% to 26.8%)	**-29.2% (-40.9% to -14.4%)#**	**-28.6% (-42.6% to -11.3%)#**	**-20.7% (-26.7% to -13.9%)#**	**-16.8% (-22.0% to -10.9%)#**	**-17.6% (-26.3% to -8.2%)#**	**-18.7% (-30.5% to -4.6%)#**	**-20.5% (-31.7% to -7.0%)#**	**-19.5% (-27.8% to -9.9%)#**	**-17.4% (-25.1% to -9.2%)#**
	% change 2000-2017	10.7% (-7.4% to 31.4%)	1.7% (-21.4% to 31.0%)	-10.2% (-25.8% to 8.0%)	-11.1% (-28.2% to 11.0%)	**-13.2% (-19.8% to -5.8%)#**	**-10.2% (-15.7% to -4.2%)#**	**-13.3% (-22.5% to -3.3%)#**	1.1% (-13.9% to 18.5%)	-1.1% (-15.4% to 15.5%)	-6.1% (-15.6% to 4.8%)	**-12.6% (-20.9% to -3.7%)#**
Utah	Death #, 1990	81 (68 to 96)	24 (18 to 30)	87 (75 to 101)	46 (36 to 58)	37 (35 to 39)	43 (41 to 44)	113 (111 to 115)	193 (178 to 207)	238 (225 to 252)	431 (414 to 449)	156 (153 to 159)
	Death #, 2000	98 (85 to 112)	26 (21 to 31)	66 (56 to 76)	43 (34 to 55)	26 (24 to 27)	38 (37 to 39)	127 (125 to 130)	190 (176 to 204)	233 (220 to 247)	425 (408 to 442)	166 (163 to 169)
	Death #, 2017	105 (87 to 127)	25 (18 to 34)	58 (47 to 70)	37 (28 to 48)	26 (24 to 28)	41 (38 to 43)	135 (121 to 151)	187 (159 to 221)	224 (193 to 260)	426 (384 to 472)	176 (159 to 194)
	% change 1990-2017	28.3% (-0.8% to 65.3%)	3.1% (-30.6% to 50.1%)	**-33.4% (-47.9% to -14.5%)#**	-18.5% (-43.1% to 14.8%)	**-30.2% (-36.3% to -23.6%)#**	-4.3% (-11.0% to 2.6%)	**19.7% (6.6% to 34.2%)#**	-2.8% (-19.8% to 17.3%)	-5.8% (-20.2% to 10.8%)	-1.0% (-11.5% to 10.4%)	**13.1% (2.1% to 25.0%)#**
	% change 2000-2017	6.5% (-14.5% to 36.4%)	-4.7% (-31.7% to 28.5%)	-11.9% (-32.0% to 12.1%)	-14.4% (-42.1% to 23.8%)	0.5% (-7.8% to 9.8%)	6.3% (-0.8% to 13.9%)	6.2% (-5.0% to 19.2%)	-1.4% (-18.2% to 17.4%)	-3.8% (-17.9% to 12.8%)	0.4% (-9.7% to 11.4%)	6.2% (-3.7% to 17.4%)
Vermont	Death #, 1990	33 (29 to 38)	7 (5 to 9)	20 (17 to 23)	11 (9 to 14)	9 (8 to 9)	10 (10 to 11)	30 (29 to 30)	60 (55 to 66)	72 (66 to 78)	120 (113 to 127)	40 (39 to 41)
	Death #, 2000	25 (22 to 29)	6 (4 to 7)	13 (11 to 15)	7 (5 to 9)	7 (6 to 7)	7 (7 to 7)	28 (27 to 29)	44 (40 to 48)	50 (46 to 55)	92 (86 to 98)	35 (34 to 36)
	Death #, 2017	16 (12 to 20)	5 (3 to 8)	8 (6 to 11)	5 (3 to 6)	3 (3 to 4)	4 (3 to 4)	17 (16 to 19)	29 (23 to 36)	34 (27 to 42)	58 (50 to 68)	21 (19 to 23)
	% change 1990-2017	**-52.8% (-64.1% to -38.3%)#**	-24.8% (-57.3% to 31.9%)	**-59.2% (-70.6% to -44.1%)#**	**-59.6% (-73.4% to -40.6%)#**	**-59.7% (-66.1% to -52.8%)#**	**-64.2% (-67.6% to -60.6%)#**	**-41.3% (-46.0% to -36.2%)#**	**-51.6% (-62.2% to -39.5%)#**	**-52.9% (-63.6% to -40.4%)#**	**-51.5% (-59.2% to -42.6%)#**	**-47.2% (-51.4% to -42.9%)#**
	% change 2000-2017	**-37.4% (-52.8% to -17.1%)#**	-6.3% (-48.6% to 64.8%)	**-36.5% (-53.0% to -14.4%)#**	-31.4% (-57.5% to 6.4%)	**-48.5% (-56.5% to -38.5%)#**	**-48.5% (-53.4% to -43.3%)#**	**-38.2% (-43.3% to -32.8%)#**	**-33.1% (-47.9% to -14.2%)#**	**-32.9% (-48.3% to -14.0%)#**	**-36.9% (-46.6% to -25.1%)#**	**-40.3% (-45.1% to -35.2%)#**
Virginia	Death #, 1990	584 (529 to 644)	114 (94 to 133)	316 (278 to 358)	161 (138 to 185)	96 (93 to 99)	99 (97 to 101)	363 (359 to 367)	1014 (958 to 1072)	1175 (1122 to 1232)	1733 (1673 to 1798)	462 (457 to 468)
	Death #, 2000	388 (352 to 424)	99 (80 to 119)	221 (198 to 246)	111 (97 to 129)	64 (61 to 67)	89 (87 to 91)	315 (311 to 318)	708 (661 to 754)	819 (772 to 866)	1287 (1233 to 1340)	404 (398 to 409)
	Death #, 2017	324 (276 to 378)	85 (70 to 103)	198 (172 to 228)	99 (86 to 114)	60 (55 to 64)	69 (64 to 74)	256 (227 to 290)	606 (518 to 708)	705 (605 to 821)	1090 (975 to 1218)	325 (292 to 362)
	% change 1990-2017	**-44.6% (-54.6% to -32.5%)#**	**-25.5% (-41.9% to -3.4%)#**	**-37.4% (-47.9% to -24.8%)#**	**-38.4% (-49.8% to -23.9%)#**	**-37.9% (-42.9% to -32.1%)#**	**-30.4% (-35.3% to -25.3%)#**	**-29.4% (-37.5% to -20.3%)#**	**-40.2% (-49.2% to -29.4%)#**	**-40.0% (-49.2% to -29.0%)#**	**-37.1% (-44.0% to -28.9%)#**	**-29.6% (-36.8% to -21.8%)#**
	% change 2000-2017	**-16.7% (-30.3% to -0.0%)#**	-14.1% (-34.9% to 14.2%)	-10.4% (-25.4% to 6.6%)	-11.0% (-26.7% to 8.9%)	-7.2% (-15.1% to 1.6%)	**-22.7% (-28.1% to -16.9%)#**	**-18.5% (-27.9% to -8.1%)#**	-14.3% (-27.1% to 0.9%)	-13.9% (-26.3% to 1.7%)	**-15.3% (-24.2% to -3.8%)#**	**-19.4% (-27.9% to -10.3%)#**
Washington	Death #, 1990	261 (232 to 291)	69 (56 to 84)	264 (224 to 307)	114 (98 to 132)	77 (74 to 80)	77 (75 to 79)	266 (262 to 269)	594 (559 to 633)	707 (670 to 751)	1127 (1082 to 1178)	343 (338 to 348)
	Death #, 2000	219 (193 to 246)	60 (50 to 72)	162 (139 to 187)	88 (72 to 107)	63 (59 to 66)	76 (74 to 79)	251 (248 to 255)	440 (408 to 474)	528 (496 to 563)	919 (879 to 960)	328 (322 to 333)
	Death #, 2017	211 (179 to 247)	53 (42 to 68)	130 (113 to 149)	72 (62 to 83)	43 (40 to 47)	55 (51 to 59)	201 (177 to 226)	394 (337 to 459)	465 (400 to 542)	764 (685 to 853)	256 (230 to 285)
	% change 1990-2017	**-19.2% (-33.2% to -1.4%)#**	-23.1% (-42.0% to 3.0%)	**-50.8% (-60.1% to -39.6%)#**	**-37.0% (-49.1% to -22.0%)#**	**-43.7% (-48.4% to -38.1%)#**	**-28.6% (-33.6% to -23.2%)#**	**-24.5% (-33.4% to -14.8%)#**	**-33.7% (-43.7% to -21.3%)#**	**-34.2% (-43.9% to -21.9%)#**	**-32.2% (-39.3% to -23.3%)#**	**-25.4% (-33.3% to -16.8%)#**
	% change 2000-2017	-3.7% (-21.3% to 19.4%)	-11.4% (-31.6% to 17.0%)	**-19.7% (-35.0% to -1.8%)#**	-18.7% (-36.1% to 4.2%)	**-31.1% (-37.2% to -24.1%)#**	**-28.0% (-33.1% to -22.4%)#**	**-20.0% (-29.4% to -9.8%)#**	-10.6% (-24.9% to 6.5%)	-12.0% (-25.2% to 4.6%)	**-16.8% (-25.9% to -5.5%)#**	**-21.9% (-30.0% to -12.9%)#**
W Virginia	Death #, 1990	132 (114 to 151)	32 (23 to 42)	98 (77 to 122)	51 (39 to 66)	24 (23 to 25)	36 (35 to 37)	134 (131 to 136)	262 (246 to 280)	313 (295 to 333)	506 (484 to 529)	169 (167 to 172)
	Death #, 2000	94 (83 to 107)	28 (20 to 37)	72 (62 to 86)	31 (24 to 40)	25 (24 to 26)	33 (32 to 34)	106 (105 to 108)	194 (180 to 208)	225 (210 to 240)	390 (371 to 408)	140 (137 to 142)
	Death #, 2017	68 (55 to 83)	20 (14 to 28)	52 (42 to 66)	25 (16 to 38)	17 (15 to 20)	23 (21 to 26)	80 (70 to 90)	141 (116 to 170)	165 (133 to 206)	286 (245 to 336)	103 (92 to 115)
	% change 1990-2017	**-48.5% (-59.4% to -34.1%)#**	**-37.4% (-58.6% to -4.8%)#**	**-46.3% (-61.4% to -26.4%)#**	**-51.5% (-70.9% to -22.1%)#**	**-26.3% (-37.1% to -12.6%)#**	**-35.4% (-42.2% to -28.6%)#**	**-40.2% (-47.4% to -32.4%)#**	**-46.3% (-56.6% to -34.6%)#**	**-47.1% (-58.1% to -33.9%)#**	**-43.5% (-51.8% to -33.4%)#**	**-39.2% (-45.9% to -32.0%)#**
	% change 2000-2017	**-27.8% (-43.6% to -8.2%)#**	-26.7% (-50.9% to 10.6%)	**-27.5% (-45.8% to -5.3%)#**	-20.9% (-53.1% to 31.3%)	**-30.2% (-41.0% to -17.4%)#**	**-30.3% (-37.0% to -23.1%)#**	**-24.9% (-33.8% to -15.0%)#**	**-27.5% (-41.4% to -11.5%)#**	**-26.6% (-42.5% to -7.7%)#**	**-26.7% (-37.9% to -14.1%)#**	**-26.2% (-34.4% to -17.4%)#**
Wisconsin	Death #, 1990	295 (266 to 327)	66 (55 to 77)	219 (192 to 249)	113 (97 to 131)	73 (69 to 76)	85 (83 to 87)	285 (281 to 288)	580 (544 to 615)	693 (657 to 729)	1,135 (1,092 to 1,179)	370 (365 to 375)
	Death #, 2000	257 (234 to 280)	58 (49 to 68)	148 (130 to 168)	86 (75 to 99)	65 (62 to 68)	74 (73 to 76)	265 (261 to 269)	463 (437 to 493)	549 (522 to 580)	954 (920 to 992)	339 (335 to 344)
	Death #, 2017	206 (176 to 242)	45 (37 to 55)	101 (87 to 117)	57 (49 to 65)	37 (34 to 40)	54 (50 to 58)	178 (155 to 200)	352 (300 to 413)	409 (350 to 477)	678 (605 to 763)	231 (206 to 258)
	% change 1990-2017	**-30.1% (-42.3% to -14.8%)#**	**-31.4% (-45.9% to -10.9%)#**	**-53.9% (-61.8% to -44.2%)#**	**-49.7% (-59.6% to -37.6%)#**	**-48.6% (-52.9% to -43.5%)#**	**-36.8% (-41.7% to -31.6%)#**	**-37.7% (-45.5% to -29.7%)#**	**-39.2% (-48.3% to -28.1%)#**	**-41.0% (-49.5% to -30.1%)#**	**-40.3% (-46.8% to -32.1%)#**	**-37.5% (-44.2% to -30.6%)#**
	% change 2000-2017	**-19.8% (-32.9% to -4.6%)#**	-22.4% (-40.6% to 1.5%)	**-31.6% (-43.9% to -18.0%)#**	**-34.3% (-46.0% to -19.7%)#**	**-42.5% (-47.4% to -37.0%)#**	**-27.8% (-33.4% to -22.2%)#**	**-33.0% (-41.6% to -24.5%)#**	**-23.9% (-36.0% to -10.1%)#**	**-25.6% (-37.6% to -12.5%)#**	**-28.9% (-37.0% to -20.0%)#**	**-31.8% (-39.5% to -24.2%)#**
Wyoming	Death #, 1990	26 (23 to 30)	5 (4 to 7)	30 (25 to 35)	13 (10 to 16)	10 (9 to 11)	13 (12 to 13)	37 (36 to 38)	61 (57 to 66)	74 (69 to 80)	134 (127 to 141)	50 (49 to 51)
	Death #, 2000	23 (20 to 27)	5 (4 to 7)	16 (14 to 20)	9 (7 to 12)	11 (10 to 12)	11 (11 to 12)	38 (38 to 39)	45 (41 to 49)	54 (49 to 59)	114 (108 to 121)	49 (48 to 51)
	Death #, 2017	23 (18 to 29)	5 (3 to 8)	11 (8 to 14)	9 (6 to 12)	6 (5 to 7)	6 (5 to 6)	29 (27 to 32)	39 (32 to 48)	48 (39 to 60)	89 (78 to 103)	35 (32 to 38)
	% change 1990-2017	-10.9% (-32.5% to 17.4%)	-7.0% (-49.0% to 66.2%)	**-64.0% (-73.9% to -51.1%)#**	-30.8% (-52.9% to 1.2%)	**-38.2% (-47.4% to -27.1%)#**	**-54.3% (-58.4% to -49.7%)#**	**-20.9% (-27.7% to -14.0%)#**	**-36.4% (-48.8% to -20.7%)#**	**-35.4% (-49.0% to -17.9%)#**	**-33.4% (-42.6% to -22.1%)#**	**-29.4% (-35.0% to -23.7%)#**
	% change 2000-2017	0.1% (-24.4% to 34.0%)	-2.4% (-43.0% to 62.9%)	**-35.1% (-51.0% to -11.8%)#**	-1.9% (-37.6% to 50.2%)	**-43.4% (-51.9% to -32.3%)#**	**-47.4% (-52.4% to -41.7%)#**	**-24.0% (-30.1% to -17.4%)#**	-13.1% (-30.9% to 9.1%)	-11.2% (-30.2% to 13.8%)	**-22.1% (-33.0% to -8.2%)#**	**-29.2% (-34.7% to -23.3%)#**
	Death #, 1990	11384 (10860 to 11896)	2729 (2548 to 2920)	8811 (8344 to 9301)	3628 (3339 to 3955)	1601 (1587 to 1616)	1625 (1617 to 1633)	3068 (3057 to 3079)	22923 (22498 to 23337)	26551 (26257 to 26868)	32845 (32523 to 33190)	4693 (4675 to 4711)
	% of global total, 1990	0.4% (0.4% to 0.4%)	0.2% (0.2% to 0.2%)	0.2% (0.2% to 0.2%)	0.1% (0.1% to 0.1%)	0.2% (0.2% to 0.2%)	0.3% (0.3% to 0.3%)	0.5% (0.5% to 0.5%)	0.3% (0.3% to 0.3%)	0.2% (0.2% to 0.2%)	0.2% (0.2% to 0.2%)	0.4% (0.4% to 0.4%)
	Death #, 2000	7224 (6973 to 7474)	2072 (1955 to 2202)	5489 (5214 to 5767)	2399 (2208 to 2616)	1304 (1289 to 1319)	1392 (1383 to 1402)	3376 (3364 to 3388)	14786 (14491 to 15065)	17185 (16909 to 17457)	23257 (22953 to 23549)	4768 (4749 to 4788)

eTable 2. ENN, LNN, PNN, 1-4, 5-9, 10-14, 15-19, <1, <5, 10-19, and <20 years mortality in 1990, 2000 and 2017 with mean percentage change for all GBD causes.

	Metric	Early Neonatal	Late Neonatal	Post Neonatal	1 to 4	5 to 9	10 to 14	15 to 19	<1 year	Under 5	<20 years	10 to 19
Southern Latin America	% of global total, 2000	0.3% (0.2% to 0.3%)	0.2% (0.2% to 0.2%)	0.2% (0.2% to 0.2%)	0.1% (0.1% to 0.1%)	0.2% (0.2% to 0.2%)	0.3% (0.3% to 0.3%)	0.5% (0.5% to 0.5%)	0.2% (0.2% to 0.2%)	0.2% (0.2% to 0.2%)	0.2% (0.2% to 0.2%)	0.4% (0.4% to 0.4%)
	Death #, 2017	4509 (3944 to 5131)	1624 (1425 to 1846)	2788 (2436 to 3184)	1520 (1350 to 1716)	846 (774 to 925)	1027 (966 to 1098)	3284 (3003 to 3600)	8920 (7847 to 10151)	10441 (9201 to 11840)	15597 (14197 to 17303)	4311 (3990 to 4689)
	% of global total, 2017	0.2% (0.2% to 0.3%)	0.3% (0.3% to 0.4%)	0.2% (0.1% to 0.2%)	0.1% (0.1% to 0.1%)	0.2% (0.2% to 0.2%)	0.3% (0.3% to 0.3%)	0.6% (0.6% to 0.7%)	0.2% (0.2% to 0.3%)	0.2% (0.2% to 0.2%)	0.2% (0.2% to 0.3%)	0.5% (0.5% to 0.6%)
	% change 1990-2017	-60.4% (-65.5% to -54.5%)#	-40.5% (-48.6% to -31.0%)#	-68.4% (-72.4% to -63.9%)#	-58.1% (-63.3% to -51.5%)#	-47.2% (-51.5% to -42.2%)#	-36.8% (-40.6% to -32.4%)#	7.0% (-2.0% to 17.5%)	-61.1% (-65.8% to -55.8%)#	-60.7% (-65.3% to -55.3%)#	-52.5% (-56.8% to -47.3%)#	-8.2% (-15.0% to -0.1%)#
	% change 2000-2017	-37.6% (-45.6% to -28.2%)#	-21.6% (-32.5% to -10.0%)#	-49.2% (-55.9% to -42.0%)#	-36.6% (-45.6% to -26.4%)#	-35.1% (-40.6% to -29.0%)#	-26.2% (-30.7% to -21.2%)#	-2.7% (-10.9% to 6.7%)	-39.7% (-46.9% to -31.5%)#	-39.2% (-46.6% to -31.1%)#	-32.9% (-39.0% to -25.5%)#	-9.6% (-16.3% to -1.7%)#
Argentina	Death #, 1990	8820 (8341 to 9284)	2051 (1870 to 2243)	6266 (5821 to 6721)	2626 (2338 to 2933)	1094 (1083 to 1106)	1144 (1137 to 1151)	2015 (2006 to 2025)	17138 (16757 to 17517)	19764 (19521 to 20032)	24016 (23751 to 24306)	3159 (3145 to 3174)
	Death #, 2000	5844 (5612 to 6087)	1598 (1476 to 1724)	4224 (3963 to 4493)	1814 (1632 to 2023)	939 (925 to 952)	1016 (1007 to 1025)	2516 (2505 to 2527)	11666 (11395 to 11933)	13481 (13229 to 13726)	17951 (17675 to 18224)	3532 (3514 to 3550)
	Death #, 2017	3459 (2952 to 4035)	1317 (1125 to 1536)	2254 (1940 to 2618)	1218 (1056 to 1404)	643 (574 to 721)	782 (723 to 853)	2554 (2284 to 2861)	7030 (6015 to 8196)	8248 (7091 to 9607)	12227 (10878 to 13785)	3336 (3015 to 3707)
	% change 1990-2017	-60.8% (-66.8% to -53.6%)#	-35.8% (-46.4% to -23.5%)#	-64.0% (-69.6% to -57.8%)#	-53.6% (-60.8% to -44.5%)#	-41.2% (-47.4% to -34.2%)#	-31.6% (-37.0% to -25.3%)#	26.8% (12.9% to 42.1%)#	-59.0% (-64.8% to -52.0%)#	-58.3% (-64.2% to -51.4%)#	-49.1% (-54.7% to -42.2%)#	5.6% (-4.6% to 17.4%)
	% change 2000-2017	-40.8% (-49.9% to -30.5%)#	-17.6% (-30.9% to -2.6%)#	-46.6% (-54.9% to -37.9%)#	-32.9% (-44.8% to -19.7%)#	-31.5% (-38.9% to -23.2%)#	-23.0% (-29.1% to -16.0%)#	1.5% (-9.4% to 13.8%)	-39.7% (-48.7% to -30.2%)#	-38.8% (-47.8% to -28.8%)#	-31.9% (-39.4% to -23.1%)#	-5.5% (-14.7% to 5.1%)
Chile	Death #, 1990	2,042 (1,893 to 2,202)	530 (494 to 566)	2,099 (1,963 to 2,243)	855 (770 to 949)	432 (424 to 441)	392 (388 to 396)	856 (850 to 862)	4,670 (4,528 to 4,813)	5,525 (5,390 to 5,668)	7,205 (7,055 to 7,363)	1,248 (1,239 to 1,257)
	Death #, 2000	1,090 (1,008 to 1,167)	337 (321 to 354)	950 (876 to 1,031)	461 (415 to 513)	303 (296 to 312)	311 (306 to 315)	675 (670 to 680)	2,377 (2,294 to 2,469)	2,838 (2,747 to 2,937)	4,127 (4,022 to 4,240)	986 (977 to 995)
	Death #, 2017	882 (660 to 1,160)	236 (177 to 309)	401 (278 to 564)	241 (198 to 294)	168 (149 to 188)	196 (180 to 212)	558 (498 to 624)	1,519 (1,155 to 1,982)	1,760 (1,352 to 2,276)	2,681 (2,225 to 3,244)	753 (682 to 833)
	% change 1990-2017	-56.8% (-68.2% to -43.1%)#	-55.5% (-66.8% to -41.4%)#	-80.9% (-86.9% to -72.8%)#	-71.8% (-77.1% to -64.6%)#	-61.2% (-65.5% to -56.3%)#	-50.1% (-54.1% to -46.0%)#	-34.9% (-41.8% to -27.1%)#	-67.5% (-75.3% to -57.9%)#	-68.1% (-75.5% to -58.9%)#	-62.8% (-69.1% to -55.0%)#	-39.7% (-45.5% to -33.2%)#
	% change 2000-2017	-19.1% (-39.8% to 8.7%)	-30.0% (-47.6% to -7.4%)#	-57.8% (-71.5% to -41.1%)#	-47.6% (-58.4% to -35.0%)#	-44.8% (-50.8% to -38.0%)#	-37.0% (-42.1% to -31.9%)#	-17.4% (-26.3% to -7.7%)#	-36.1% (-51.2% to -17.1%)#	-38.0% (-52.0% to -20.3%)#	-35.0% (-46.1% to -21.2%)#	-23.6% (-30.8% to -15.7%)#
Uruguay	Death #, 1990	521 (488 to 557)	147 (132 to 162)	445 (410 to 483)	147 (129 to 167)	75 (73 to 78)	89 (88 to 91)	197 (194 to 199)	1,114 (1,069 to 1,160)	1,261 (1,205 to 1,322)	1,622 (1,562 to 1,687)	286 (282 to 290)
	Death #, 2000	290 (264 to 315)	137 (124 to 150)	314 (286 to 345)	124 (111 to 137)	62 (60 to 64)	66 (65 to 68)	184 (182 to 187)	741 (698 to 786)	865 (820 to 914)	1,178 (1,127 to 1,233)	251 (247 to 254)
	Death #, 2017	167 (129 to 213)	71 (55 to 90)	133 (98 to 177)	61 (49 to 74)	36 (31 to 40)	49 (45 to 53)	172 (153 to 191)	371 (299 to 457)	432 (349 to 533)	688 (595 to 799)	221 (200 to 244)
	% change 1990-2017	-68.0% (-75.7% to -58.5%)#	-51.9% (-63.7% to -37.1%)#	-70.1% (-78.5% to -59.9%)#	-58.6% (-67.2% to -47.7%)#	-52.9% (-58.7% to -45.9%)#	-45.0% (-49.6% to -40.2%)#	-12.6% (-22.1% to -2.5%)#	-66.7% (-73.3% to -58.8%)#	-65.8% (-72.6% to -57.6%)#	-57.6% (-63.8% to -50.3%)#	-22.8% (-30.3% to -14.7%)#
	% change 2000-2017	-42.3% (-55.9% to -25.9%)#	-48.4% (-61.0% to -33.7%)#	-57.7% (-69.5% to -43.2%)#	-50.9% (-60.7% to -39.0%)#	-42.6% (-49.5% to -34.8%)#	-25.7% (-31.9% to -19.0%)#	-6.9% (-17.2% to 3.9%)	-50.0% (-59.5% to -38.1%)#	-50.1% (-59.7% to -38.5%)#	-41.6% (-49.6% to -31.7%)#	-11.9% (-20.3% to -2.5%)#
Western Europe	Death #, 1990	16,606 (15,953 to 17,233)	5,070 (4,768 to 5,425)	13,578 (12,756 to 14,402)	7,098 (6,592 to 7,638)	4,591 (4,562 to 4,623)	5,001 (4,981 to 5,020)	15,488 (15,462 to 15,515)	35,255 (34,620 to 35,871)	42,353 (42,010 to 42,721)	67,433 (67,027 to 67,867)	20,489 (20,446 to 20,533)
	% of global total, 1990	0.6% (0.5% to 0.6%)	0.4% (0.4% to 0.4%)	0.3% (0.3% to 0.4%)	0.2% (0.2% to 0.2%)	0.5% (0.5% to 0.5%)	1.1% (1.1% to 1.1%)	2.3% (2.3% to 2.3%)	0.4% (0.4% to 0.4%)	0.4% (0.3% to 0.4%)	0.5% (0.5% to 0.5%)	1.8% (1.8% to 1.8%)
	Death #, 2000	10,136 (9,687 to 10,581)	3,542 (3,325 to 3,763)	6,773 (6,337 to 7,195)	4,309 (3,976 to 4,659)	2,853 (2,830 to 2,876)	3,537 (3,520 to 3,554)	10,979 (10,958 to 11,001)	20,452 (20,014 to 20,835)	24,761 (24,513 to 25,018)	42,130 (41,833 to 42,437)	14,516 (14,480 to 14,552)
	% of global total, 2000	0.4% (0.3% to 0.4%)	0.4% (0.4% to 0.4%)	0.2% (0.2% to 0.2%)	0.1% (0.1% to 0.2%)	0.4% (0.4% to 0.4%)	0.7% (0.7% to 0.7%)	1.6% (1.6% to 1.6%)	0.3% (0.3% to 0.3%)	0.3% (0.2% to 0.3%)	0.4% (0.4% to 0.4%)	1.2% (1.2% to 1.3%)
	Death #, 2017	6,811 (6,375 to 7,278)	2,433 (2,263 to 2,617)	4,227 (3,920 to 4,596)	2,679 (2,562 to 2,802)	1,750 (1,702 to 1,798)	1,948 (1,914 to 1,985)	5,180 (5,058 to 5,310)	13,471 (12,670 to 14,322)	16,150 (15,240 to 17,108)	25,028 (24,009 to 26,097)	7,129 (6,983 to 7,279)
	% of global total, 2017	0.4% (0.3% to 0.4%)	0.5% (0.4% to 0.5%)	0.3% (0.2% to 0.3%)	0.2% (0.2% to 0.2%)	0.4% (0.4% to 0.4%)	0.6% (0.6% to 0.6%)	1.0% (1.0% to 1.0%)	0.3% (0.3% to 0.4%)	0.3% (0.3% to 0.3%)	0.4% (0.4% to 0.4%)	0.8% (0.8% to 0.9%)
	% change 1990-2017	-59.0% (-62.1% to -55.4%)#	-52.0% (-56.6% to -46.9%)#	-68.9% (-71.9% to -65.6%)#	-62.3% (-65.4% to -58.9%)#	-61.9% (-63.0% to -60.8%)#	-61.0% (-61.7% to -60.3%)#	-66.5% (-67.4% to -65.7%)#	-61.8% (-64.1% to -59.2%)#	-61.9% (-63.9% to -59.5%)#	-62.9% (-64.4% to -61.2%)#	-65.2% (-65.9% to -64.5%)#
	% change 2000-2017	-32.8% (-38.0% to -27.0%)#	-31.3% (-37.8% to -24.2%)#	-37.6% (-43.1% to -30.6%)#	-37.8% (-43.0% to -31.5%)#	-38.7% (-40.5% to -36.9%)#	-44.9% (-45.9% to -43.9%)#	-52.8% (-53.9% to -51.6%)#	-34.1% (-38.4% to -29.8%)#	-34.8% (-38.5% to -30.8%)#	-40.6% (-43.0% to -37.9%)#	-50.9% (-51.9% to -49.8%)#
Andorra	Death #, 1990	1 (1 to 2)	0 (0 to 1)	1 (1 to 1)	2 (1 to 2)	0 (0 to 1)	1 (0 to 1)	2 (1 to 2)	3 (2 to 3)	4 (3 to 5)	7 (5 to 8)	2 (2 to 2)
	Death #, 2000	1 (1 to 1)	0 (0 to 0)	0 (0 to 1)	1 (1 to 2)	0 (0 to 0)	0 (0 to 0)	1 (1 to 1)	2 (1 to 2)	3 (2 to 4)	4 (4 to 5)	1 (1 to 1)
	Death #, 2017	0 (0 to 0)	0 (0 to 0)	0 (0 to 0)	1 (0 to 1)	0 (0 to 0)	0 (0 to 0)	1 (1 to 1)	1 (1 to 1)	1 (1 to 2)	3 (2 to 3)	1 (1 to 1)
	% change 1990-2017	-72.8% (-78.8% to -65.4%)#	-68.4% (-75.3% to -59.7%)#	-65.5% (-73.0% to -56.2%)#	-66.5% (-82.4% to -41.5%)#	-35.7% (-48.1% to -19.7%)#	-36.9% (-45.9% to -25.4%)#	-45.2% (-54.5% to -33.5%)#	-70.0% (-76.5% to -61.8%)#	-68.7% (-78.3% to -57.0%)#	-58.2% (-67.3% to -47.8%)#	-43.2% (-52.1% to -32.2%)#
	% change 2000-2017	-52.5% (-60.6% to -42.9%)#	-50.7% (-59.1% to -40.6%)#	-48.4% (-57.3% to -37.8%)#	-54.1% (-72.2% to -22.7%)#	-21.9% (-33.7% to -8.2%)#	-3.1% (-14.6% to 9.1%)	-8.3% (-21.9% to 6.9%)	-50.9% (-59.3% to -41.0%)#	-52.2% (-64.9% to -35.7%)#	-36.0% (-47.5% to -21.9%)#	-7.0% (-19.4% to 6.1%)
Austria	Death #, 1990	330 (285 to 375)	108 (92 to 125)	291 (236 to 346)	141 (107 to 182)	86 (83 to 90)	71 (69 to 72)	354 (350 to 358)	728 (674 to 779)	869 (824 to 918)	1,380 (1,327 to 1,437)	425 (419 to 430)
	Death #, 2000	183 (160 to 207)	61 (53 to 69)	116 (93 to 141)	84 (63 to 109)	56 (53 to 58)	69 (68 to 71)	279 (276 to 282)	360 (330 to 389)	443 (415 to 471)	847 (813 to 881)	348 (344 to 353)
	Death #, 2017	144 (115 to 179)	34 (27 to 42)	69 (57 to 84)	47 (40 to 55)	32 (29 to 36)	33 (31 to 35)	133 (125 to 142)	247 (199 to 305)	293 (239 to 361)	493 (431 to 564)	167 (157 to 176)
	% change 1990-2017	-56.3% (-65.9% to -43.9%)#	-68.7% (-75.7% to -59.2%)#	-76.3% (-81.9% to -68.2%)#	-66.7% (-75.3% to -54.5%)#	-62.6% (-66.5% to -58.2%)#	-52.6% (-55.6% to -49.4%)#	-62.3% (-64.5% to -60.0%)#	-66.2% (-72.9% to -57.4%)#	-66.2% (-72.7% to -58.2%)#	-64.3% (-69.1% to -58.6%)#	-60.7% (-63.0% to -58.3%)#
	% change 2000-2017	-21.5% (-40.0% to 1.4%)	-44.3% (-57.9% to -28.4%)#	-40.5% (-56.0% to -19.1%)#	-43.8% (-58.2% to -23.2%)#	-42.1% (-48.1% to -35.5%)#	-51.7% (-55.0% to -48.5%)#	-52.2% (-55.1% to -49.3%)#	-31.4% (-46.3% to -12.8%)#	-33.8% (-46.7% to -18.2%)#	-41.9% (-49.8% to -32.8%)#	-52.1% (-54.9% to -49.3%)#
Belgium	Death #, 1990	459 (382 to 536)	128 (117 to 141)	434 (353 to 516)	183 (139 to 237)	125 (121 to 130)	123 (120 to 125)	405 (401 to 409)	1,020 (955 to 1,087)	1,203 (1,147 to 1,261)	1,856 (1,790 to 1,923)	528 (522 to 534)
	Death #, 2000	271 (229 to 312)	86 (70 to 103)	207 (162 to 252)	128 (96 to 164)	80 (76 to 83)	98 (95 to 100)	319 (316 to 322)	564 (518 to 615)	692 (653 to 734)	1,188 (1,143 to 1,238)	416 (411 to 422)
	Death #, 2017	177 (139 to 226)	59 (46 to 75)	125 (100 to 157)	79 (67 to 93)	61 (56 to 68)	45 (43 to 48)	148 (140 to 157)	361 (284 to 457)	440 (351 to 551)	695 (591 to 815)	193 (183 to 205)
	% change 1990-2017	-61.3% (-71.5% to -47.0%)#	-54.0% (-64.9% to -40.5%)#	-71.2% (-78.3% to -61.4%)#	-56.8% (-68.3% to -41.1%)#	-51.0% (-55.9% to -45.4%)#	-63.1% (-65.5% to -60.6%)#	-63.5% (-65.6% to -61.3%)#	-64.6% (-72.4% to -54.6%)#	-63.4% (-70.8% to -54.1%)#	-62.6% (-68.2% to -55.9%)#	-63.4% (-65.4% to -61.3%)#
	% change 2000-2017	-34.5% (-50.8% to -12.4%)#	-31.9% (-50.4% to -8.1%)#	-39.6% (-56.6% to -15.1%)#	-38.1% (-52.7% to -15.3%)#	-23.0% (-31.1% to -14.3%)#	-53.5% (-56.7% to -50.3%)#	-53.6% (-56.3% to -50.8%)#	-36.0% (-50.6% to -17.9%)#	-36.4% (-49.6% to -20.3%)#	-41.5% (-50.5% to -31.1%)#	-53.6% (-56.2% to -50.8%)#
	Death #, 1990	73 (68 to 79)	19 (18 to 21)	55 (51 to 59)	18 (14 to 22)	16 (15 to 17)	9 (9 to 9)	33 (33 to 34)	147 (136 to 159)	165 (153 to 178)	224 (210 to 238)	43 (42 to 43)

eTable 2. ENN, LNN, PNN, 1-4, 5-9, 10-14, 15-19, <1, <5, 10-19, and <20 years mortality in 1990, 2000 and 2017 with mean percentage change for all GBD causes.

	Metric	Early Neonatal	Late Neonatal	Post Neonatal	1 to 4	5 to 9	10 to 14	15 to 19	<1 year	Under 5	<20 years	10 to 19
Cyprus	Death #, 2000	35<br>(31 to 39)	10<br>(9 to 12)	21<br>(18 to 23)	11<br>(9 to 13)	13<br>(12 to 14)	12<br>(11 to 12)	48<br>(46 to 51)	66<br>(59 to 74)	77<br>(69 to 85)	150<br>(140 to 161)	60<br>(57 to 64)
	Death #, 2017	14<br>(12 to 17)	5<br>(4 to 6)	9<br>(8 to 11)	5<br>(4 to 6)	5<br>(5 to 6)	5<br>(5 to 5)	22<br>(20 to 24)	28<br>(24 to 34)	33<br>(28 to 39)	66<br>(59 to 73)	27<br>(25 to 30)
	% change 1990-2017	**-80.2%<br>(-83.4% to -76.5%)#**	**-74.2%<br>(-78.3% to -69.2%)#**	**-83.7%<br>(-86.2% to -80.6%)#**	**-73.2%<br>(-79.6% to -64.8%)#**	**-67.2%<br>(-70.4% to -63.8%)#**	**-45.1%<br>(-49.0% to -40.8%)#**	**-33.6%<br>(-39.8% to -26.9%)#**	**-80.7%<br>(-83.8% to -77.1%)#**	**-79.9%<br>(-83.1% to -76.2%)#**	**-70.7%<br>(-73.7% to -67.1%)#**	**-36.1%<br>(-41.7% to -30.1%)#**
	% change 2000-2017	**-58.4%<br>(-65.2% to -50.9%)#**	**-52.7%<br>(-60.5% to -44.2%)#**	**-56.3%<br>(-63.2% to -48.6%)#**	**-55.8%<br>(-64.7% to -44.5%)#**	**-59.9%<br>(-64.2% to -55.1%)#**	**-57.8%<br>(-61.0% to -54.4%)#**	**-54.0%<br>(-59.0% to -48.7%)#**	**-56.8%<br>(-63.9% to -49.1%)#**	**-56.7%<br>(-63.7% to -49.0%)#**	**-56.2%<br>(-60.6% to -50.9%)#**	**-54.8%<br>(-59.2% to -49.9%)#**
Denmark	Death #, 1990	232<br>(205 to 259)	61<br>(53 to 68)	185<br>(155 to 217)	95<br>(69 to 124)	55<br>(52 to 58)	62<br>(60 to 63)	153<br>(151 to 155)	478<br>(443 to 511)	572<br>(534 to 610)	843<br>(799 to 885)	215<br>(211 to 218)
	Death #, 2000	166<br>(143 to 190)	46<br>(37 to 56)	91<br>(77 to 106)	66<br>(50 to 85)	40<br>(38 to 42)	46<br>(45 to 47)	121<br>(120 to 123)	302<br>(273 to 333)	369<br>(344 to 397)	576<br>(546 to 608)	167<br>(164 to 170)
	Death #, 2017	100<br>(77 to 127)	31<br>(24 to 40)	64<br>(50 to 81)	28<br>(23 to 33)	27<br>(24 to 30)	20<br>(18 to 21)	59<br>(56 to 63)	194<br>(151 to 248)	222<br>(175 to 281)	327<br>(277 to 391)	79<br>(74 to 83)
	% change 1990-2017	**-57.2%<br>(-67.8% to -43.9%)#**	**-49.0%<br>(-62.0% to -32.9%)#**	**-65.5%<br>(-74.1% to -52.9%)#**	**-70.6%<br>(-79.0% to -59.0%)#**	**-51.7%<br>(-56.8% to -45.9%)#**	**-68.3%<br>(-70.5% to -65.8%)#**	**-61.4%<br>(-63.7% to -58.9%)#**	**-59.4%<br>(-68.6% to -47.1%)#**	**-61.2%<br>(-69.9% to -50.9%)#**	**-61.1%<br>(-67.6% to -53.2%)#**	**-63.4%<br>(-65.5% to -61.1%)#**
	% change 2000-2017	**-40.0%<br>(-55.7% to -20.4%)#**	**-32.2%<br>(-52.1% to -5.9%)#**	**-29.7%<br>(-46.9% to -7.1%)#**	**-58.1%<br>(-68.8% to -42.3%)#**	**-33.1%<br>(-40.3% to -24.6%)#**	**-57.4%<br>(-60.2% to -54.1%)#**	**-51.3%<br>(-54.1% to -48.2%)#**	**-35.7%<br>(-51.3% to -16.8%)#**	**-39.8%<br>(-53.2% to -22.5%)#**	**-43.1%<br>(-52.6% to -31.1%)#**	**-52.9%<br>(-55.6% to -50.1%)#**
Finland	Death #, 1990	213<br>(182 to 240)	50<br>(41 to 58)	114<br>(92 to 140)	70<br>(53 to 91)	61<br>(58 to 64)	61<br>(60 to 63)	212<br>(209 to 214)	377<br>(345 to 408)	447<br>(418 to 476)	782<br>(746 to 816)	273<br>(269 to 277)
	Death #, 2000	109<br>(96 to 123)	30<br>(24 to 36)	59<br>(48 to 70)	46<br>(34 to 59)	43<br>(40 to 46)	45<br>(44 to 47)	164<br>(162 to 167)	198<br>(179 to 217)	243<br>(226 to 262)	496<br>(473 to 520)	210<br>(206 to 213)
	Death #, 2017	54<br>(44 to 64)	14<br>(11 to 19)	32<br>(19 to 51)	24<br>(20 to 30)	28<br>(25 to 31)	30<br>(28 to 32)	82<br>(77 to 87)	100<br>(76 to 131)	124<br>(96 to 160)	263<br>(228 to 305)	111<br>(105 to 118)
	% change 1990-2017	**-74.8%<br>(-80.0% to -68.2%)#**	**-70.8%<br>(-79.0% to -59.2%)#**	**-72.2%<br>(-83.9% to -52.4%)#**	**-65.4%<br>(-75.1% to -52.2%)#**	**-54.5%<br>(-59.5% to -48.3%)#**	**-51.6%<br>(-54.9% to -48.0%)#**	**-61.5%<br>(-63.8% to -59.0%)#**	**-73.5%<br>(-80.0% to -64.8%)#**	**-72.2%<br>(-78.7% to -64.0%)#**	**-66.3%<br>(-70.8% to -60.6%)#**	**-59.3%<br>(-61.6% to -56.7%)#**
	% change 2000-2017	**-50.8%<br>(-61.8% to -38.2%)#**	**-51.4%<br>(-66.7% to -31.2%)#**	**-46.0%<br>(-69.0% to -10.3%)#**	**-46.8%<br>(-60.2% to -24.2%)#**	**-35.2%<br>(-43.0% to -25.8%)#**	**-34.3%<br>(-38.9% to -29.0%)#**	**-50.4%<br>(-53.5% to -47.1%)#**	**-49.5%<br>(-62.6% to -33.1%)#**	**-49.0%<br>(-61.3% to -33.5%)#**	**-46.9%<br>(-54.2% to -37.6%)#**	**-46.9%<br>(-50.1% to -43.5%)#**
France	Death #, 1990	2,076<br>(1,847 to 2,302)	814<br>(715 to 917)	2,651<br>(2,315 to 2,992)	1,163<br>(899 to 1,489)	679<br>(667 to 692)	731<br>(724 to 738)	2,373<br>(2,362 to 2,382)	5,541<br>(5,162 to 5,889)	6,704<br>(6,543 to 6,878)	10,487<br>(10,302 to 10,687)	3,104<br>(3,087 to 3,119)
	Death #, 2000	1,549<br>(1,295 to 1,819)	634<br>(521 to 752)	1,268<br>(1,050 to 1,488)	738<br>(548 to 946)	462<br>(451 to 474)	603<br>(596 to 612)	1,900<br>(1,890 to 1,909)	3,451<br>(3,222 to 3,668)	4,189<br>(4,069 to 4,322)	7,154<br>(7,011 to 7,312)	2,503<br>(2,487 to 2,519)
	Death #, 2017	1,054<br>(849 to 1,306)	475<br>(383 to 588)	768<br>(632 to 932)	570<br>(499 to 652)	314<br>(290 to 341)	319<br>(302 to 334)	847<br>(800 to 894)	2,297<br>(1,863 to 2,827)	2,867<br>(2,362 to 3,478)	4,347<br>(3,782 to 5,012)	1,165<br>(1,108 to 1,225)
	% change 1990-2017	**-49.2%<br>(-59.9% to -35.4%)#**	**-41.6%<br>(-55.4% to -25.2%)#**	**-71.0%<br>(-77.0% to -63.0%)#**	**-51.0%<br>(-62.9% to -34.7%)#**	**-53.8%<br>(-57.4% to -49.7%)#**	**-56.4%<br>(-58.8% to -54.2%)#**	**-64.3%<br>(-66.3% to -62.3%)#**	**-58.5%<br>(-66.4% to -48.4%)#**	**-57.2%<br>(-64.8% to -48.0%)#**	**-58.5%<br>(-63.9% to -52.3%)#**	**-62.5%<br>(-64.3% to -60.5%)#**
	% change 2000-2017	**-32.0%<br>(-48.9% to -9.2%)#**	**-25.0%<br>(-44.4% to -0.5%)#**	**-39.4%<br>(-52.9% to -22.6%)#**	-22.8%<br>(-41.2% to 5.6%)	**-32.0%<br>(-37.4% to -26.2%)#**	**-47.2%<br>(-49.9% to -44.4%)#**	**-55.4%<br>(-57.8% to -52.9%)#**	**-33.4%<br>(-46.7% to -17.0%)#**	**-31.6%<br>(-43.5% to -17.2%)#**	**-39.2%<br>(-46.9% to -30.1%)#**	**-53.4%<br>(-55.8% to -51.0%)#**
Germany	Death #, 1990	2,524<br>(2,050 to 3,008)	862<br>(638 to 1,123)	2,844<br>(2,235 to 3,484)	1,402<br>(1,066 to 1,774)	910<br>(895 to 925)	788<br>(781 to 795)	2,591<br>(2,581 to 2,601)	6,230<br>(5,855 to 6,580)	7,632<br>(7,463 to 7,795)	11,922<br>(11,724 to 12,108)	3,380<br>(3,363 to 3,396)
	Death #, 2000	1,590<br>(1,351 to 1,844)	500<br>(388 to 642)	1,223<br>(948 to 1,487)	765<br>(580 to 983)	484<br>(472 to 496)	634<br>(625 to 644)	2,099<br>(2,088 to 2,109)	3,313<br>(3,094 to 3,521)	4,077<br>(3,955 to 4,195)	7,294<br>(7,143 to 7,437)	2,733<br>(2,714 to 2,752)
	Death #, 2017	1,243<br>(970 to 1,555)	346<br>(244 to 462)	758<br>(533 to 1,043)	428<br>(364 to 504)	261<br>(233 to 291)	319<br>(294 to 346)	1,043<br>(935 to 1,158)	2,346<br>(1,888 to 2,859)	2,774<br>(2,266 to 3,365)	4,398<br>(3,829 to 5,073)	1,363<br>(1,235 to 1,497)
	% change 1990-2017	**-50.8%<br>(-64.1% to -32.4%)#**	**-59.9%<br>(-75.5% to -35.3%)#**	**-73.4%<br>(-81.8% to -60.0%)#**	**-69.5%<br>(-76.7% to -58.1%)#**	**-71.3%<br>(-74.5% to -67.8%)#**	**-59.5%<br>(-62.7% to -56.0%)#**	**-59.7%<br>(-63.9% to -55.3%)#**	**-62.4%<br>(-70.0% to -53.8%)#**	**-63.6%<br>(-70.5% to -56.2%)#**	**-63.1%<br>(-68.0% to -57.5%)#**	**-59.7%<br>(-63.5% to -55.7%)#**
	% change 2000-2017	-21.9%<br>(-40.9% to 3.6%)	**-30.8%<br>(-54.5% to -0.0%)#**	**-38.0%<br>(-58.8% to -8.3%)#**	**-44.0%<br>(-58.6% to -23.7%)#**	**-46.1%<br>(-51.9% to -39.3%)#**	**-49.7%<br>(-53.7% to -45.4%)#**	**-50.3%<br>(-55.5% to -44.7%)#**	**-29.2%<br>(-43.5% to -11.5%)#**	**-32.0%<br>(-44.8% to -17.7%)#**	**-39.7%<br>(-47.8% to -30.4%)#**	**-50.1%<br>(-54.8% to -45.2%)#**
Greece	Death #, 1990	497<br>(436 to 564)	189<br>(153 to 226)	277<br>(230 to 328)	136<br>(102 to 180)	127<br>(123 to 132)	144<br>(141 to 148)	392<br>(388 to 396)	963<br>(899 to 1,028)	1,100<br>(1,050 to 1,153)	1,763<br>(1,703 to 1,825)	536<br>(529 to 543)
	Death #, 2000	267<br>(231 to 306)	127<br>(102 to 156)	160<br>(133 to 191)	89<br>(68 to 114)	71<br>(68 to 74)	86<br>(84 to 88)	354<br>(350 to 357)	554<br>(511 to 600)	644<br>(607 to 682)	1,154<br>(1,111 to 1,199)	439<br>(434 to 445)
	Death #, 2017	140<br>(105 to 184)	73<br>(55 to 96)	104<br>(80 to 134)	64<br>(55 to 75)	51<br>(46 to 56)	46<br>(44 to 49)	131<br>(124 to 138)	318<br>(245 to 410)	382<br>(299 to 484)	610<br>(519 to 723)	177<br>(168 to 187)
	% change 1990-2017	**-71.8%<br>(-79.5% to -61.5%)#**	**-61.1%<br>(-71.5% to -46.5%)#**	**-62.4%<br>(-72.7% to -49.0%)#**	**-53.2%<br>(-66.0% to -33.7%)#**	**-59.9%<br>(-63.7% to -55.5%)#**	**-68.0%<br>(-69.8% to -66.0%)#**	**-66.6%<br>(-68.5% to -64.7%)#**	**-67.0%<br>(-74.4% to -56.8%)#**	**-65.3%<br>(-72.7% to -56.0%)#**	**-65.4%<br>(-70.6% to -59.1%)#**	**-67.0%<br>(-68.7% to -65.2%)#**
	% change 2000-2017	**-47.5%<br>(-62.4% to -28.2%)#**	**-42.2%<br>(-59.9% to -17.6%)#**	**-35.0%<br>(-52.7% to -9.3%)#**	**-28.6%<br>(-45.9% to -2.5%)#**	**-27.8%<br>(-34.7% to -19.7%)#**	**-45.9%<br>(-49.2% to -42.5%)#**	**-63.0%<br>(-65.1% to -60.9%)#**	**-42.7%<br>(-56.2% to -25.2%)#**	**-40.7%<br>(-54.2% to -24.1%)#**	**-47.2%<br>(-55.3% to -37.2%)#**	**-59.7%<br>(-61.8% to -57.5%)#**
Iceland	Death #, 1990	14<br>(12 to 16)	3<br>(3 to 4)	7<br>(6 to 8)	5<br>(4 to 7)	4<br>(4 to 5)	5<br>(5 to 5)	13<br>(13 to 14)	24<br>(21 to 27)	29<br>(26 to 33)	52<br>(48 to 56)	18<br>(18 to 19)
	Death #, 2000	6<br>(5 to 7)	2<br>(2 to 3)	4<br>(3 to 5)	4<br>(4 to 5)	3<br>(2 to 3)	3<br>(3 to 3)	8<br>(7 to 8)	12<br>(11 to 14)	17<br>(14 to 19)	30<br>(27 to 33)	10<br>(10 to 11)
	Death #, 2017	3<br>(2 to 3)	1<br>(1 to 2)	2<br>(2 to 3)	3<br>(2 to 4)	2<br>(2 to 2)	2<br>(2 to 2)	6<br>(6 to 6)	6<br>(5 to 8)	9<br>(7 to 12)	20<br>(17 to 23)	8<br>(8 to 8)
	% change 1990-2017	**-81.4%<br>(-86.6% to -74.3%)#**	**-53.6%<br>(-64.9% to -40.0%)#**	**-67.8%<br>(-77.6% to -54.7%)#**	**-39.3%<br>(-56.7% to -11.9%)#**	**-54.2%<br>(-60.5% to -46.9%)#**	**-53.4%<br>(-56.8% to -49.3%)#**	**-56.5%<br>(-58.8% to -54.1%)#**	**-73.7%<br>(-80.5% to -65.3%)#**	**-67.6%<br>(-75.6% to -57.3%)#**	**-62.3%<br>(-67.9% to -55.3%)#**	**-55.7%<br>(-58.1% to -53.0%)#**
	% change 2000-2017	**-57.6%<br>(-70.1% to -39.9%)#**	**-33.1%<br>(-44.0% to -19.9%)#**	**-44.7%<br>(-61.8% to -22.3%)#**	**-26.0%<br>(-43.2% to -3.4%)#**	**-23.9%<br>(-34.7% to -11.2%)#**	**-15.3%<br>(-21.9% to -8.0%)#**	**-25.2%<br>(-29.1% to -21.2%)#**	**-48.9%<br>(-62.4% to -31.7%)#**	**-43.0%<br>(-57.6% to -24.3%)#**	**-34.2%<br>(-44.6% to -21.4%)#**	**-22.6%<br>(-26.7% to -18.1%)#**
Ireland	Death #, 1990	215<br>(191 to 240)	46<br>(39 to 53)	150<br>(119 to 183)	87<br>(67 to 111)	60<br>(58 to 63)	66<br>(64 to 68)	169<br>(167 to 172)	411<br>(375 to 447)	498<br>(468 to 532)	794<br>(758 to 834)	235<br>(232 to 239)
	Death #, 2000	179<br>(156 to 204)	44<br>(35 to 53)	106<br>(94 to 117)	59<br>(44 to 77)	37<br>(35 to 39)	46<br>(45 to 47)	197<br>(194 to 200)	329<br>(299 to 359)	388<br>(361 to 416)	667<br>(636 to 701)	243<br>(239 to 247)
	Death #, 2017	108<br>(89 to 130)	30<br>(25 to 36)	61<br>(51 to 72)	26<br>(23 to 30)	24<br>(21 to 27)	28<br>(27 to 30)	71<br>(67 to 76)	199<br>(165 to 238)	225<br>(188 to 267)	349<br>(308 to 396)	100<br>(94 to 106)
	% change 1990-2017	**-49.8%<br>(-59.7% to -38.2%)#**	**-34.7%<br>(-49.6% to -15.2%)#**	**-59.3%<br>(-68.8% to -45.7%)#**	**-69.9%<br>(-77.6% to -60.0%)#**	**-60.6%<br>(-64.9% to -55.8%)#**	**-57.0%<br>(-59.7% to -54.0%)#**	**-58.0%<br>(-60.8% to -55.2%)#**	**-51.5%<br>(-60.4% to -40.7%)#**	**-54.8%<br>(-62.6% to -45.2%)#**	**-56.1%<br>(-61.5% to -49.6%)#**	**-57.7%<br>(-60.3% to -55.1%)#**
	% change 2000-2017	**-39.7%<br>(-51.7% to -24.9%)#**	**-31.5%<br>(-48.0% to -8.7%)#**	**-42.3%<br>(-53.1% to -29.8%)#**	**-55.5%<br>(-66.5% to -38.6%)#**	**-35.5%<br>(-42.3% to -27.6%)#**	**-38.5%<br>(-42.3% to -34.3%)#**	**-63.8%<br>(-66.2% to -61.3%)#**	**-39.4%<br>(-50.5% to -26.4%)#**	**-41.9%<br>(-51.9% to -30.4%)#**	**-47.8%<br>(-54.1% to -40.4%)#**	**-59.0%<br>(-61.5% to -56.5%)#**
Israel	Death #, 1990	489<br>(420 to 562)	148<br>(123 to 174)	374<br>(300 to 461)	200<br>(154 to 254)	110<br>(106 to 113)	82<br>(81 to 84)	206<br>(203 to 209)	1,011<br>(940 to 1,086)	1,211<br>(1,160 to 1,268)	1,609<br>(1,551 to 1,672)	288<br>(284 to 292)
	Death #, 2000	345<br>(292 to 401)	126<br>(107 to 147)	254<br>(197 to 320)	172<br>(132 to 218)	88<br>(85 to 91)	94<br>(92 to 96)	244<br>(241 to 247)	725<br>(664 to 782)	897<br>(853 to 943)	1,323<br>(1,271 to 1,376)	337<br>(333 to 342)
	Death #, 2017	233<br>(199 to 270)	105<br>(90 to 122)	172<br>(149 to 198)	122<br>(107 to 140)	79<br>(72 to 87)	78<br>(74 to 82)	169<br>(160 to 179)	510<br>(438 to 589)	633<br>(548 to 728)	958<br>(864 to 1,064)	247<br>(234 to 260)
	% change 1990-2017	**-52.3%<br>(-61.6% to -40.7%)#**	**-29.1%<br>(-44.3% to -9.1%)#**	**-54.0%<br>(-63.9% to -39.6%)#**	**-38.9%<br>(-53.9% to -18.8%)#**	**-28.1%<br>(-34.7% to -20.9%)#**	-5.3%<br>(-10.4% to 0.4%)	**-17.9%<br>(-22.8% to -12.7%)#**	**-49.5%<br>(-57.3% to -40.7%)#**	**-47.8%<br>(-55.1% to -39.6%)#**	**-40.4%<br>(-46.8% to -33.6%)#**	**-14.3%<br>(-19.0% to -9.5%)#**
	% change 2000-2017	**-32.5%<br>(-45.8% to -15.2%)#**	-16.9%<br>(-33.8% to 4.1%)	**-32.0%<br>(-48.5% to -10.2%)#**	**-28.7%<br>(-45.3% to -4.4%)#**	**-10.6%<br>(-19.1% to -0.8%)#**	**-17.0%<br>(-21.9% to -12.1%)#**	**-30.6%<br>(-34.7% to -26.1%)#**	**-29.6%<br>(-41.2% to -16.9%)#**	**-29.5%<br>(-39.3% to -17.9%)#**	**-27.5%<br>(-35.3% to -18.6%)#**	**-26.9%<br>(-30.7% to -22.6%)#**

eTable 2. ENN, LNN, PNN, 1-4, 5-9, 10-14, 15-19, <1, <5, 10-19, and <20 years mortality in 1990, 2000 and 2017 with mean percentage change for all GBD causes.

	Metric	Early Neonatal	Late Neonatal	Post Neonatal	1 to 4	5 to 9	10 to 14	15 to 19	<1 year	Under 5	<20 years	10 to 19
Italy	Death #, 1990	2815 (2556 to 3087)	704 (584 to 843)	1133 (961 to 1312)	715 (554 to 902)	512 (501 to 522)	699 (692 to 707)	2405 (2393 to 2417)	4653 (4422 to 4872)	5368 (5228 to 5516)	8984 (8820 to 9156)	3104 (3087 to 3123)
	Death #, 2000	1313 (1098 to 1533)	488 (388 to 595)	661 (510 to 818)	426 (320 to 550)	323 (315 to 332)	419 (412 to 425)	1265 (1258 to 1273)	2462 (2302 to 2600)	2889 (2796 to 2989)	4895 (4781 to 5016)	1684 (1671 to 1697)
	Death #, 2017	614 (480 to 781)	261 (205 to 332)	367 (295 to 456)	248 (218 to 283)	221 (204 to 240)	263 (250 to 276)	579 (547 to 615)	1243 (978 to 1571)	1491 (1197 to 1853)	2555 (2220 to 2957)	842 (800 to 886)
	% change 1990-2017	-78.2% (-83.3% to -71.5%)#	-62.9% (-72.2% to -49.2%)#	-67.6% (-74.9% to -58.1%)#	-65.2% (-73.2% to -53.5%)#	-56.8% (-60.2% to -52.9%)#	-62.4% (-64.3% to -60.4%)#	-75.9% (-77.2% to -74.5%)#	-73.3% (-79.1% to -66.4%)#	-72.2% (-77.6% to -65.5%)#	-71.6% (-75.2% to -67.1%)#	-72.9% (-74.2% to -71.5%)#
	% change 2000-2017	-53.2% (-65.2% to -36.9%)#	-46.4% (-61.0% to -25.9%)#	-44.4% (-59.6% to -21.9%)#	-41.8% (-56.1% to -18.5%)#	-31.5% (-37.1% to -25.4%)#	-37.2% (-40.4% to -33.7%)#	-54.2% (-56.8% to -51.4%)#	-49.5% (-60.5% to -35.5%)#	-48.4% (-58.6% to -36.0%)#	-47.8% (-54.7% to -39.6%)#	-50.0% (-52.5% to -47.2%)#
Luxembourg	Death #, 1990	16 (15 to 17)	5 (5 to 7)	15 (12 to 17)	6 (5 to 7)	4 (4 to 5)	3 (3 to 4)	21 (20 to 21)	36 (33 to 40)	42 (38 to 47)	71 (66 to 76)	24 (23 to 25)
	Death #, 2000	10 (9 to 12)	4 (3 to 4)	7 (6 to 8)	5 (4 to 6)	3 (3 to 3)	4 (4 to 5)	12 (12 to 12)	20 (18 to 23)	26 (23 to 29)	45 (41 to 49)	16 (16 to 17)
	Death #, 2017	5 (4 to 7)	2 (2 to 3)	3 (2 to 5)	3 (2 to 3)	2 (2 to 2)	3 (3 to 3)	9 (8 to 9)	11 (8 to 14)	13 (10 to 18)	27 (23 to 32)	12 (11 to 12)
	% change 1990-2017	-67.9% (-77.5% to -54.5%)#	-62.5% (-73.6% to -47.1%)#	-76.1% (-83.6% to -65.4%)#	-56.5% (-67.3% to -42.3%)#	-50.6% (-57.3% to -42.8%)#	-17.1% (-24.2% to -9.7%)#	-57.5% (-60.9% to -53.7%)#	-70.4% (-78.1% to -60.4%)#	-68.3% (-76.2% to -58.4%)#	-61.6% (-67.5% to -54.5%)#	-51.7% (-55.4% to -47.8%)#
	% change 2000-2017	-49.4% (-65.3% to -28.4%)#	-43.4% (-59.0% to -22.9%)#	-48.2% (-64.0% to -25.9%)#	-46.3% (-59.8% to -29.6%)#	-27.4% (-37.5% to -15.4%)#	-33.6% (-39.4% to -27.5%)#	-26.5% (-32.4% to -20.2%)#	-47.9% (-62.2% to -29.9%)#	-47.6% (-61.4% to -30.7%)#	-39.3% (-49.0% to -27.4%)#	-28.4% (-34.0% to -22.6%)#
Malta	Death #, 1990	32 (29 to 35)	9 (8 to 10)	11 (9 to 14)	8 (6 to 10)	5 (5 to 6)	3 (3 to 4)	10 (10 to 11)	52 (48 to 57)	60 (55 to 65)	79 (73 to 85)	14 (13 to 14)
	Death #, 2000	17 (15 to 19)	4 (4 to 5)	5 (4 to 6)	6 (5 to 6)	3 (3 to 4)	4 (4 to 4)	11 (11 to 11)	26 (23 to 29)	31 (28 to 35)	50 (46 to 54)	15 (15 to 16)
	Death #, 2017	14 (11 to 16)	4 (2 to 5)	5 (3 to 7)	3 (2 to 3)	2 (2 to 3)	3 (2 to 3)	4 (4 to 5)	22 (18 to 27)	25 (21 to 30)	35 (30 to 40)	7 (7 to 7)
	% change 1990-2017	-57.0% (-65.2% to -47.7%)#	-60.0% (-73.8% to -40.0%)#	-56.0% (-71.8% to -33.1%)#	-64.4% (-73.3% to -51.5%)#	-54.3% (-59.8% to -48.2%)#	-24.0% (-29.4% to -18.0%)#	-57.1% (-59.3% to -54.8%)#	-57.3% (-65.7% to -46.8%)#	-58.2% (-66.4% to -47.6%)#	-56.3% (-63.2% to -47.8%)#	-48.9% (-51.5% to -46.1%)#
	% change 2000-2017	-16.9% (-29.1% to -2.1%)#	-17.1% (-44.6% to 19.8%)	1.6% (-30.9% to 47.5%)	-51.9% (-61.0% to -41.1%)#	-29.8% (-38.1% to -20.7%)#	-34.2% (-39.1% to -29.0%)#	-60.2% (-62.3% to -58.2%)#	-13.4% (-29.6% to 4.9%)	-20.3% (-34.5% to -4.3%)#	-31.0% (-40.7% to -20.3%)#	-53.4% (-56.0% to -50.8%)#
Netherlands	Death #, 1990	733 (642 to 818)	190 (158 to 224)	472 (386 to 569)	307 (235 to 392)	161 (156 to 166)	185 (182 to 188)	439 (435 to 443)	1,394 (1,291 to 1,488)	1,702 (1,634 to 1,771)	2,487 (2,409 to 2,568)	624 (618 to 631)
	Death #, 2000	612 (534 to 690)	171 (138 to 209)	285 (235 to 339)	222 (164 to 286)	129 (124 to 133)	144 (142 to 147)	347 (343 to 350)	1,067 (988 to 1,141)	1,289 (1,230 to 1,349)	1,908 (1,843 to 1,979)	491 (485 to 497)
	Death #, 2017	317 (250 to 399)	110 (87 to 139)	143 (114 to 179)	98 (83 to 114)	57 (53 to 63)	79 (74 to 83)	189 (179 to 201)	571 (452 to 717)	668 (535 to 831)	993 (848 to 1,170)	267 (253 to 283)
	% change 1990-2017	-56.8% (-66.5% to -44.0%)#	-41.8% (-56.9% to -21.5%)#	-69.6% (-77.3% to -59.2%)#	-68.2% (-76.4% to -57.1%)#	-64.3% (-67.6% to -60.7%)#	-57.5% (-59.9% to -54.8%)#	-57.0% (-59.4% to -54.2%)#	-59.1% (-67.7% to -48.0%)#	-60.7% (-68.7% to -50.8%)#	-60.1% (-66.1% to -52.8%)#	-57.1% (-59.5% to -54.6%)#
	% change 2000-2017	-48.2% (-60.9% to -32.6%)#	-35.3% (-52.8% to -11.0%)#	-49.6% (-61.9% to -34.0%)#	-56.0% (-66.9% to -39.3%)#	-55.3% (-59.3% to -50.8%)#	-45.5% (-48.8% to -42.2%)#	-45.5% (-48.5% to -42.1%)#	-46.5% (-58.3% to -32.2%)#	-48.1% (-58.4% to -35.5%)#	-48.0% (-55.8% to -38.6%)#	-45.5% (-48.5% to -42.5%)#
Norway	Death #, 1990	218 (208 to 228)	67 (64 to 70)	140 (133 to 149)	97 (91 to 104)	52 (51 to 53)	53 (53 to 54)	185 (184 to 187)	425 (411 to 440)	523 (508 to 538)	813 (796 to 831)	239 (237 to 240)
	Death #, 2000	117 (111 to 124)	40 (37 to 43)	65 (61 to 68)	63 (58 to 68)	39 (38 to 40)	34 (33 to 34)	144 (143 to 145)	222 (212 to 231)	285 (276 to 294)	501 (491 to 513)	177 (176 to 179)
	Death #, 2017	65 (60 to 71)	23 (21 to 25)	40 (37 to 43)	26 (24 to 28)	13 (13 to 14)	29 (29 to 30)	83 (82 to 85)	128 (119 to 138)	154 (144 to 165)	280 (268 to 293)	113 (111 to 115)
	% change 1990-2017	-70.1% (-72.8% to -67.2%)#	-65.7% (-68.8% to -62.4%)#	-71.5% (-74.0% to -69.0%)#	-73.4% (-75.9% to -70.7%)#	-74.1% (-75.3% to -72.9%)#	-44.6% (-46.1% to -43.1%)#	-55.1% (-56.0% to -54.1%)#	-69.9% (-72.3% to -67.3%)#	-70.5% (-72.9% to -68.2%)#	-65.5% (-67.3% to -63.8%)#	-52.7% (-53.7% to -51.8%)#
	% change 2000-2017	-44.3% (-49.4% to -38.4%)#	-42.1% (-47.8% to -35.2%)#	-38.3% (-43.2% to -32.9%)#	-58.9% (-62.8% to -54.5%)#	-65.9% (-67.4% to -64.3%)#	-12.8% (-15.0% to -10.4%)#	-42.0% (-43.2% to -40.8%)#	-42.2% (-46.7% to -37.1%)#	-45.9% (-49.8% to -41.6%)#	-44.1% (-46.7% to -41.3%)#	-36.5% (-37.8% to -35.1%)#
Portugal	Death #, 1990	650 (580 to 719)	159 (139 to 179)	471 (391 to 553)	368 (283 to 461)	251 (243 to 260)	314 (308 to 320)	800 (793 to 807)	1,280 (1,174 to 1,382)	1,648 (1,581 to 1,719)	3,013 (2,927 to 3,104)	1,113 (1,102 to 1,125)
	Death #, 2000	312 (260 to 368)	105 (88 to 122)	238 (196 to 282)	191 (156 to 230)	127 (122 to 132)	142 (138 to 145)	439 (435 to 444)	655 (605 to 705)	847 (798 to 895)	1,554 (1,492 to 1,614)	581 (574 to 588)
	Death #, 2017	115 (87 to 151)	37 (28 to 49)	79 (61 to 101)	58 (49 to 69)	31 (29 to 34)	49 (46 to 52)	130 (122 to 137)	231 (177 to 301)	289 (226 to 369)	499 (428 to 586)	178 (169 to 188)
	% change 1990-2017	-82.3% (-86.8% to -76.4%)#	-76.5% (-82.7% to -68.0%)#	-83.3% (-87.6% to -77.4%)#	-84.3% (-88.4% to -78.3%)#	-87.6% (-88.7% to -86.2%)#	-84.5% (-85.5% to -83.5%)#	-83.8% (-84.7% to -82.8%)#	-81.9% (-86.2% to -76.5%)#	-82.5% (-86.3% to -77.6%)#	-83.5% (-85.8% to -80.5%)#	-84.0% (-84.9% to -83.1%)#
	% change 2000-2017	-63.1% (-73.7% to -49.2%)#	-64.3% (-74.5% to -51.0%)#	-67.0% (-75.3% to -55.2%)#	-69.8% (-76.6% to -60.7%)#	-75.3% (-77.6% to -72.6%)#	-65.7% (-67.7% to -63.3%)#	-70.5% (-72.2% to -68.7%)#	-64.7% (-73.4% to -53.4%)#	-65.8% (-73.3% to -55.9%)#	-67.9% (-72.5% to -61.9%)#	-69.3% (-71.0% to -67.6%)#
Spain	Death #, 1990	1,452 (1,265 to 1,648)	531 (452 to 617)	1,006 (808 to 1,236)	699 (539 to 888)	571 (559 to 584)	761 (752 to 770)	2,136 (2,125 to 2,148)	2,990 (2,789 to 3,172)	3,689 (3,584 to 3,798)	7,157 (7,028 to 7,297)	2,897 (2,878 to 2,918)
	Death #, 2000	747 (635 to 860)	381 (328 to 434)	598 (454 to 757)	388 (293 to 496)	292 (283 to 301)	362 (357 to 368)	1,168 (1,160 to 1,175)	1,726 (1,593 to 1,859)	2,115 (2,033 to 2,203)	3,936 (3,838 to 4,046)	1,530 (1,518 to 1,542)
	Death #, 2017	475 (387 to 582)	243 (199 to 297)	363 (305 to 430)	236 (206 to 269)	177 (163 to 193)	189 (179 to 199)	391 (371 to 411)	1,080 (891 to 1,309)	1,316 (1,097 to 1,579)	2,073 (1,828 to 2,357)	580 (552 to 607)
	% change 1990-2017	-67.3% (-74.3% to -58.6%)#	-54.3% (-64.1% to -41.2%)#	-64.0% (-72.0% to -51.8%)#	-66.3% (-74.4% to -55.3%)#	-69.0% (-71.5% to -66.0%)#	-75.2% (-76.5% to -73.9%)#	-81.7% (-82.6% to -80.7%)#	-63.9% (-70.3% to -55.8%)#	-64.3% (-70.2% to -57.3%)#	-71.0% (-74.4% to -66.9%)#	-80.0% (-81.0% to -79.0%)#
	% change 2000-2017	-36.5% (-51.6% to -18.2%)#	-36.2% (-50.4% to -18.5%)#	-39.4% (-54.8% to -17.3%)#	-39.3% (-53.3% to -18.2%)#	-39.2% (-44.3% to -33.1%)#	-47.9% (-50.7% to -45.0%)#	-66.5% (-68.2% to -64.8%)#	-37.4% (-49.4% to -22.9%)#	-37.8% (-48.2% to -24.9%)#	-47.3% (-53.7% to -39.8%)#	-62.1% (-63.9% to -60.3%)#
Sweden	Death #, 1990	355 (322 to 389)	91 (82 to 100)	263 (226 to 308)	121 (101 to 144)	69 (67 to 72)	79 (77 to 80)	260 (257 to 263)	709 (669 to 748)	830 (795 to 868)	1238 (1197 to 1282)	339 (335 to 343)
	Death #, 2000	150 (141 to 159)	47 (43 to 51)	98 (77 to 124)	62 (50 to 76)	60 (57 to 62)	73 (72 to 75)	166 (165 to 168)	295 (273 to 317)	357 (338 to 376)	656 (632 to 681)	240 (236 to 243)
	Death #, 2017	132 (113 to 155)	44 (38 to 52)	93 (80 to 108)	54 (49 to 61)	46 (43 to 50)	44 (42 to 46)	107 (101 to 113)	269 (231 to 315)	324 (279 to 376)	521 (469 to 580)	151 (144 to 159)
	% change 1990-2017	-62.7% (-69.3% to -54.8%)#	-51.4% (-59.9% to -41.0%)#	-64.8% (-72.1% to -56.0%)#	-54.9% (-62.9% to -44.0%)#	-32.9% (-38.1% to -27.1%)#	-44.1% (-46.8% to -41.1%)#	-59.0% (-61.2% to -56.7%)#	-62.0% (-68.0% to -55.0%)#	-61.0% (-66.7% to -54.1%)#	-57.9% (-62.4% to -52.9%)#	-55.6% (-57.6% to -53.3%)#
	% change 2000-2017	-11.6% (-26.5% to 4.6%)	-5.6% (-21.8% to 12.8%)	-5.8% (-27.0% to 25.4%)	-12.2% (-30.9% to 13.2%)	-22.1% (-28.6% to -15.5%)#	-40.0% (-43.0% to -36.8%)#	-36.0% (-39.4% to -32.4%)#	-8.7% (-23.1% to 8.3%)	-9.3% (-22.8% to 6.0%)	-20.7% (-29.2% to -11.3%)#	-37.2% (-40.2% to -34.0%)#
Stockholm	Death #, 1990	72 (62 to 84)	18 (16 to 21)	58 (47 to 72)	26 (20 to 33)	13 (12 to 15)	14 (13 to 15)	49 (48 to 51)	149 (133 to 166)	175 (157 to 194)	252 (232 to 273)	63 (62 to 65)
	Death #, 2000	33 (29 to 37)	12 (10 to 13)	24 (17 to 33)	15 (11 to 19)	15 (14 to 16)	13 (13 to 14)	32 (31 to 32)	69 (61 to 76)	84 (76 to 91)	144 (135 to 154)	45 (44 to 46)
	Death #, 2017	27 (23 to 33)	10 (9 to 13)	21 (18 to 26)	13 (12 to 16)	17 (15 to 19)	10 (9 to 11)	23 (21 to 24)	59 (49 to 72)	73 (60 to 87)	122 (108 to 140)	33 (30 to 35)
	% change 1990-2017	-62.1% (-70.4% to -51.2%)#	-43.3% (-55.4% to -26.8%)#	-63.6% (-73.1% to -51.6%)#	-48.1% (-59.6% to -31.2%)#	27.2% (12.2% to 45.0%)#	-27.9% (-33.1% to -22.1%)#	-54.4% (-57.8% to -50.9%)#	-60.4% (-68.5% to -50.1%)#	-58.5% (-66.4% to -48.5%)#	-51.5% (-58.2% to -43.1%)#	-48.5% (-51.9% to -44.9%)#

eTable 2. ENN, LNN, PNN, 1-4, 5-9, 10-14, 15-19, <1, <5, 10-19, and <20 years mortality in 1990, 2000 and 2017 with mean percentage change for all GBD causes.

	Metric	Early Neonatal	Late Neonatal	Post Neonatal	1 to 4	5 to 9	10 to 14	15 to 19	<1 year	Under 5	<20 years	10 to 19
	% change 2000-2017	-17.2% (-34.0% to 4.2%)	-9.8% (-28.7% to 15.2%)	-11.5% (-37.3% to 31.4%)	-9.9% (-33.3% to 24.0%)	10.3% (-1.5% to 24.1%)	-24.7% (-29.6% to -18.9%)#	-29.1% (-34.2% to -23.7%)#	-14.0% (-30.5% to 7.0%)	-13.2% (-28.5% to 5.8%)	-15.3% (-26.0% to -2.1%)#	-27.8% (-32.6% to -22.6%)#
Sweden w/o Stockholm	Death #, 1990	282 (249 to 314)	73 (64 to 81)	205 (168 to 247)	95 (76 to 115)	56 (54 to 58)	65 (64 to 66)	211 (208 to 214)	560 (524 to 596)	655 (622 to 687)	986 (949 to 1,023)	276 (272 to 280)
	Death #, 2000	116 (108 to 125)	35 (32 to 39)	75 (53 to 98)	47 (36 to 61)	44 (42 to 47)	60 (58 to 62)	135 (133 to 136)	226 (205 to 247)	273 (254 to 292)	512 (488 to 537)	195 (192 to 198)
	Death #, 2017	105 (86 to 128)	34 (28 to 41)	72 (59 to 86)	41 (35 to 47)	29 (27 to 32)	34 (32 to 36)	84 (79 to 90)	210 (173 to 255)	251 (208 to 303)	399 (350 to 456)	118 (112 to 125)
	% change 1990-2017	-62.9% (-70.5% to -53.0%)#	-53.5% (-63.1% to -41.0%)#	-65.1% (-73.9% to -54.0%)#	-56.8% (-66.2% to -43.1%)#	-47.3% (-52.7% to -41.4%)#	-47.6% (-50.6% to -44.1%)#	-60.1% (-62.6% to -57.4%)#	-62.5% (-69.7% to -53.8%)#	-61.6% (-68.4% to -53.6%)#	-59.6% (-64.9% to -53.5%)#	-57.2% (-59.6% to -54.5%)#
	% change 2000-2017	-10.1% (-28.3% to 11.0%)	-4.2% (-24.1% to 20.4%)	-3.9% (-30.7% to 39.5%)	-12.9% (-35.8% to 18.6%)	-33.5% (-40.6% to -25.3%)#	-43.4% (-46.9% to -40.0%)#	-37.6% (-41.7% to -33.4%)#	-7.1% (-25.2% to 14.7%)	-8.1% (-24.6% to 12.2%)	-22.2% (-32.3% to -10.3%)#	-39.4% (-42.9% to -35.6%)#
Switzerland	Death #, 1990	294 (256 to 332)	63 (52 to 76)	225 (187 to 264)	120 (92 to 153)	71 (68 to 74)	72 (70 to 74)	283 (279 to 286)	582 (536 to 627)	703 (665 to 744)	1,129 (1,085 to 1,176)	355 (350 to 360)
	Death #, 2000	230 (199 to 265)	42 (34 to 51)	99 (76 to 122)	81 (60 to 105)	47 (44 to 49)	67 (65 to 69)	173 (171 to 176)	371 (338 to 406)	452 (424 to 483)	740 (706 to 775)	241 (237 to 244)
	Death #, 2017	193 (148 to 249)	29 (22 to 38)	61 (48 to 77)	45 (38 to 53)	25 (22 to 27)	32 (31 to 34)	91 (85 to 96)	283 (218 to 365)	328 (256 to 418)	475 (398 to 571)	123 (116 to 130)
	% change 1990-2017	-34.5% (-50.9% to -12.1%)#	-53.9% (-66.5% to -37.0%)#	-73.0% (-80.0% to -63.5%)#	-62.9% (-72.5% to -49.8%)#	-65.3% (-68.9% to -61.2%)#	-55.1% (-57.8% to -52.1%)#	-67.9% (-69.8% to -65.9%)#	-51.4% (-62.8% to -36.2%)#	-53.4% (-63.7% to -41.1%)#	-57.9% (-64.8% to -49.3%)#	-65.3% (-67.2% to -63.3%)#
	% change 2000-2017	-16.2% (-38.7% to 13.7%)	-30.4% (-50.8% to -1.2%)#	-38.5% (-55.1% to -14.9%)#	-45.1% (-58.7% to -22.0%)#	-47.3% (-52.8% to -40.7%)#	-51.7% (-54.7% to -48.3%)#	-47.6% (-51.0% to -44.4%)#	-23.8% (-42.1% to 0.2%)	-27.6% (-43.7% to -7.2%)#	-35.7% (-46.3% to -22.5%)#	-48.8% (-51.6% to -45.8%)#
UK	Death #, 1990	2,899 (2,825 to 2,978)	818 (796 to 843)	2,446 (2,384 to 2,514)	1,148 (1,092 to 1,212)	655 (650 to 660)	683 (680 to 686)	2,030 (2,025 to 2,035)	6,164 (6,079 to 6,248)	7,312 (7,235 to 7,383)	10,681 (10,597 to 10,758)	2,714 (2,706 to 2,721)
	Death #, 2000	1,915 (1,877 to 1,956)	593 (576 to 611)	1,203 (1,167 to 1,249)	697 (667 to 732)	452 (448 to 457)	548 (544 to 551)	1,509 (1,502 to 1,516)	3,711 (3,654 to 3,774)	4,409 (4,349 to 4,472)	6,918 (6,846 to 6,990)	2,057 (2,047 to 2,066)
	Death #, 2017	1,604 (1,548 to 1,664)	503 (485 to 521)	905 (871 to 943)	509 (495 to 523)	287 (281 to 294)	330 (327 to 334)	880 (869 to 890)	3,011 (2,910 to 3,114)	3,520 (3,408 to 3,636)	5,017 (4,890 to 5,146)	1,210 (1,197 to 1,223)
	% change 1990-2017	-44.7% (-47.1% to -42.0%)#	-38.5% (-41.2% to -35.6%)#	-63.0% (-64.8% to -61.2%)#	-55.7% (-58.2% to -53.0%)#	-56.1% (-57.1% to -55.1%)#	-51.7% (-52.2% to -51.0%)#	-56.7% (-57.2% to -56.1%)#	-51.1% (-52.9% to -49.4%)#	-51.9% (-53.5% to -50.2%)#	-53.0% (-54.2% to -51.7%)#	-55.4% (-55.9% to -54.9%)#
	% change 2000-2017	-16.3% (-19.5% to -12.6%)#	-15.2% (-19.1% to -10.9%)#	-24.8% (-28.6% to -20.8%)#	-27.0% (-30.9% to -23.1%)#	-36.5% (-38.0% to -34.9%)#	-39.7% (-40.5% to -39.0%)#	-41.7% (-42.5% to -41.0%)#	-18.9% (-21.8% to -15.8%)#	-20.1% (-22.9% to -17.3%)#	-27.5% (-29.4% to -25.5%)#	-41.2% (-41.9% to -40.5%)#
England	Death #, 1990	2,431 (2,390 to 2,477)	688 (673 to 701)	2,075 (2,032 to 2,117)	941 (915 to 968)	537 (533 to 541)	538 (536 to 540)	1,651 (1,648 to 1,654)	5,194 (5,135 to 5,252)	6,135 (6,077 to 6,191)	8,862 (8,796 to 8,922)	2,189 (2,184 to 2,194)
	Death #, 2000	1,618 (1,592 to 1,644)	498 (488 to 509)	1,018 (998 to 1,038)	586 (570 to 602)	372 (369 to 375)	444 (442 to 446)	1,186 (1,182 to 1,190)	3,134 (3,093 to 3,176)	3,720 (3,675 to 3,765)	5,722 (5,671 to 5,772)	1,630 (1,625 to 1,635)
	Death #, 2017	1,374 (1,337 to 1,416)	433 (421 to 445)	783 (765 to 802)	439 (431 to 447)	235 (232 to 237)	278 (277 to 280)	695 (690 to 701)	2,589 (2,525 to 2,654)	3,027 (2,959 to 3,099)	4,235 (4,163 to 4,314)	974 (967 to 980)
	% change 1990-2017	-43.5% (-45.4% to -41.5%)#	-37.1% (-39.2% to -34.9%)#	-62.3% (-63.5% to -61.1%)#	-53.4% (-55.1% to -51.8%)#	-56.3% (-56.9% to -55.8%)#	-48.3% (-48.7% to -47.9%)#	-57.9% (-58.2% to -57.6%)#	-50.2% (-51.5% to -48.8%)#	-50.7% (-51.9% to -49.4%)#	-52.2% (-53.1% to -51.3%)#	-55.5% (-55.8% to -55.2%)#
	% change 2000-2017	-15.1% (-17.9% to -12.1%)#	-13.2% (-16.0% to -10.2%)#	-23.1% (-25.3% to -20.6%)#	-25.1% (-27.4% to -22.6%)#	-36.9% (-37.8% to -36.1%)#	-37.4% (-37.8% to -36.8%)#	-41.4% (-41.8% to -40.9%)#	-17.4% (-19.6% to -14.9%)#	-18.6% (-20.6% to -16.4%)#	-26.0% (-27.4% to -24.5%)#	-40.3% (-40.7% to -39.8%)#
E Midlands	Death #, 1990	214 (199 to 229)	56 (52 to 60)	170 (157 to 182)	77 (69 to 86)	45 (44 to 46)	44 (43 to 44)	171 (169 to 172)	439 (422 to 457)	516 (500 to 533)	776 (758 to 794)	214 (213 to 216)
	Death #, 2000	137 (130 to 146)	42 (39 to 46)	79 (74 to 85)	48 (43 to 54)	34 (33 to 35)	34 (34 to 35)	121 (120 to 123)	259 (247 to 272)	307 (294 to 320)	496 (482 to 511)	156 (154 to 157)
	Death #, 2017	124 (113 to 136)	36 (33 to 40)	62 (56 to 68)	33 (31 to 36)	16 (15 to 17)	24 (23 to 24)	66 (64 to 68)	222 (204 to 243)	255 (236 to 277)	361 (340 to 384)	89 (87 to 92)
	% change 1990-2017	-42.1% (-48.7% to -34.6%)#	-35.0% (-42.7% to -26.1%)#	-63.6% (-67.8% to -59.0%)#	-56.8% (-62.4% to -50.8%)#	-64.4% (-66.2% to -62.4%)#	-46.2% (-47.7% to -44.5%)#	-61.4% (-62.5% to -60.3%)#	-49.5% (-53.9% to -44.2%)#	-50.6% (-54.6% to -45.6%)#	-53.5% (-56.5% to -50.1%)#	-58.3% (-59.4% to -57.2%)#
	% change 2000-2017	-9.9% (-18.8% to 1.0%)	-14.3% (-23.6% to -3.0%)#	-21.7% (-30.6% to -10.8%)#	-31.1% (-38.7% to -23.3%)#	-52.6% (-55.0% to -49.7%)#	-31.5% (-33.6% to -29.2%)#	-45.7% (-47.2% to -44.1%)#	-14.2% (-21.8% to -4.4%)#	-16.9% (-24.0% to -8.2%)#	-27.4% (-31.9% to -21.7%)#	-42.6% (-44.1% to -41.0%)#
E England	Death #, 1990	207 (193 to 222)	59 (55 to 64)	167 (155 to 179)	83 (74 to 92)	49 (48 to 50)	66 (65 to 67)	174 (172 to 175)	433 (416 to 452)	517 (500 to 535)	805 (786 to 826)	240 (238 to 242)
	Death #, 2000	143 (135 to 152)	42 (39 to 46)	86 (80 to 93)	55 (49 to 61)	35 (34 to 36)	38 (37 to 38)	128 (127 to 130)	272 (259 to 284)	327 (314 to 341)	528 (512 to 543)	166 (164 to 168)
	Death #, 2017	130 (119 to 143)	36 (33 to 40)	66 (61 to 72)	42 (39 to 45)	25 (24 to 26)	25 (25 to 26)	66 (65 to 68)	233 (215 to 252)	275 (255 to 296)	391 (369 to 414)	91 (89 to 94)
	% change 1990-2017	-37.3% (-44.4% to -29.4%)#	-38.4% (-45.5% to -30.1%)#	-60.2% (-64.4% to -55.6%)#	-49.7% (-55.6% to -41.9%)#	-49.3% (-51.5% to -47.0%)#	-61.8% (-62.8% to -60.8%)#	-61.9% (-62.9% to -60.8%)#	-46.3% (-51.1% to -41.3%)#	-46.9% (-51.0% to -42.6%)#	-51.5% (-54.3% to -48.5%)#	-61.9% (-62.8% to -60.8%)#
	% change 2000-2017	-9.2% (-17.8% to 1.2%)	-14.0% (-24.0% to -3.5%)#	-23.1% (-32.1% to -13.5%)#	-23.9% (-31.1% to -15.1%)#	-29.4% (-32.8% to -25.7%)#	-32.9% (-35.0% to -30.8%)#	-48.5% (-49.8% to -46.8%)#	-14.4% (-21.5% to -6.3%)#	-16.0% (-22.7% to -8.5%)#	-26.0% (-30.4% to -21.0%)#	-44.9% (-46.3% to -43.3%)#
Greater London	Death #, 1990	384 (371 to 396)	122 (117 to 127)	367 (353 to 382)	157 (148 to 167)	76 (75 to 77)	54 (53 to 54)	192 (191 to 193)	873 (854 to 892)	1,031 (1,010 to 1,051)	1,352 (1,330 to 1,374)	246 (245 to 247)
	Death #, 2000	296 (288 to 305)	96 (93 to 100)	215 (208 to 223)	106 (100 to 111)	55 (54 to 56)	70 (69 to 70)	148 (147 to 149)	608 (593 to 622)	714 (698 to 730)	987 (969 to 1,004)	218 (216 to 219)
	Death #, 2017	224 (214 to 234)	74 (71 to 78)	153 (147 to 159)	87 (84 to 89)	44 (43 to 45)	43 (43 to 44)	90 (89 to 91)	451 (433 to 471)	538 (519 to 559)	715 (694 to 739)	133 (132 to 135)
	% change 1990-2017	-41.6% (-44.8% to -38.2%)#	-39.2% (-42.6% to -35.1%)#	-58.4% (-60.7% to -56.1%)#	-44.9% (-48.4% to -41.1%)#	-41.6% (-42.7% to -40.3%)#	-19.5% (-20.5% to -18.5%)#	-53.0% (-53.8% to -52.3%)#	-48.4% (-50.6% to -45.9%)#	-47.8% (-49.9% to -45.7%)#	-47.1% (-48.8% to -45.3%)#	-45.7% (-46.4% to -45.0%)#
	% change 2000-2017	-24.5% (-28.4% to -20.1%)#	-22.8% (-27.1% to -18.2%)#	-29.1% (-32.5% to -25.1%)#	-17.9% (-23.0% to -12.5%)#	-19.9% (-21.7% to -18.1%)#	-38.0% (-38.9% to -37.1%)#	-39.1% (-40.0% to -38.1%)#	-25.8% (-29.2% to -22.2%)#	-24.7% (-27.7% to -21.4%)#	-27.5% (-29.8% to -25.0%)#	-38.7% (-39.6% to -37.9%)#
NE England	Death #, 1990	131 (123 to 141)	40 (37 to 43)	123 (116 to 131)	54 (49 to 60)	35 (35 to 36)	33 (33 to 34)	83 (82 to 83)	295 (283 to 306)	349 (338 to 360)	500 (487 to 513)	116 (115 to 117)
	Death #, 2000	77 (73 to 82)	26 (24 to 28)	56 (52 to 60)	30 (27 to 33)	21 (20 to 22)	25 (24 to 25)	59 (58 to 60)	159 (152 to 166)	189 (182 to 197)	294 (286 to 303)	84 (83 to 85)
	Death #, 2017	51 (46 to 55)	20 (18 to 22)	35 (32 to 38)	19 (18 to 21)	9 (8 to 9)	13 (13 to 13)	36 (35 to 37)	106 (98 to 113)	125 (117 to 134)	183 (174 to 192)	49 (48 to 50)
	% change 1990-2017	-61.6% (-65.7% to -57.1%)#	-50.4% (-56.0% to -44.2%)#	-71.5% (-74.4% to -68.5%)#	-63.9% (-68.2% to -58.8%)#	-75.8% (-76.8% to -74.9%)#	-61.2% (-62.1% to -60.2%)#	-56.0% (-57.2% to -54.8%)#	-64.2% (-67.0% to -61.2%)#	-64.2% (-66.8% to -61.4%)#	-63.4% (-65.5% to -61.3%)#	-57.5% (-58.5% to -56.4%)#
	% change 2000-2017	-34.6% (-41.4% to -27.2%)#	-23.6% (-32.2% to -14.1%)#	-37.3% (-43.8% to -30.4%)#	-35.4% (-41.1% to -28.8%)#	-59.1% (-60.8% to -57.5%)#	-47.8% (-49.1% to -46.5%)#	-38.5% (-40.2% to -36.7%)#	-33.8% (-39.2% to -28.0%)#	-34.0% (-38.9% to -28.8%)#	-37.9% (-41.2% to -34.2%)#	-41.3% (-42.8% to -39.7%)#
NW England	Death #, 1990	343 (328 to 357)	93 (88 to 98)	315 (300 to 331)	144 (134 to 155)	88 (86 to 90)	87 (86 to 88)	230 (229 to 231)	751 (729 to 772)	895 (873 to 917)	1,300 (1,276 to 1,325)	317 (315 to 319)
	Death #, 2000	228 (219 to 238)	74 (70 to 77)	154 (147 to 163)	86 (81 to 92)	58 (57 to 59)	66 (65 to 67)	193 (191 to 195)	456 (440 to 474)	542 (525 to 561)	859 (839 to 880)	259 (257 to 261)
	Death #, 2017	193 (181 to 206)	67 (63 to 72)	126 (119 to 134)	67 (64 to 70)	34 (33 to 35)	46 (45 to 47)	112 (110 to 114)	386 (363 to 412)	453 (428 to 480)	644 (618 to 674)	158 (155 to 160)

eTable 2. ENN, LNN, PNN, 1-4, 5-9, 10-14, 15-19, <1, <5, 10-19, and <20 years mortality in 1990, 2000 and 2017 with mean percentage change for all GBD causes.

	Metric	Early Neonatal	Late Neonatal	Post Neonatal	1 to 4	5 to 9	10 to 14	15 to 19	<1 year	Under 5	<20 years	10 to 19
	% change 1990-2017	-43.8% (-48.0% to -39.3%)#	-27.6% (-33.3% to -21.2%)#	-60.1% (-63.1% to -56.8%)#	-53.7% (-57.5% to -49.2%)#	-61.3% (-62.5% to -59.9%)#	-47.1% (-48.1% to -46.0%)#	-51.5% (-52.4% to -50.5%)#	-48.6% (-51.8% to -45.0%)#	-49.5% (-52.4% to -46.2%)#	-50.5% (-52.6% to -48.1%)#	-50.3% (-51.1% to -49.4%)#
	% change 2000-2017	-15.5% (-22.1% to -8.2%)#	-8.6% (-16.3% to -0.2%)#	-18.6% (-24.4% to -12.4%)#	-22.7% (-29.0% to -15.4%)#	-41.2% (-43.2% to -39.0%)#	-30.4% (-31.9% to -28.8%)#	-42.2% (-43.3% to -40.9%)#	-15.4% (-21.4% to -9.4%)#	-16.6% (-22.0% to -10.9%)#	-25.0% (-28.6% to -21.4%)#	-39.2% (-40.3% to -37.9%)#
SE England	Death #, 1990	327 (312 to 343)	96 (90 to 101)	270 (254 to 286)	126 (115 to 137)	76 (74 to 77)	82 (81 to 83)	259 (258 to 261)	693 (673 to 713)	819 (797 to 841)	1,236 (1,211 to 1,261)	341 (339 to 344)
	Death #, 2000	197 (188 to 206)	64 (60 to 68)	117 (110 to 123)	74 (69 to 79)	55 (54 to 56)	66 (65 to 67)	169 (167 to 170)	378 (363 to 392)	451 (435 to 467)	741 (723 to 760)	234 (232 to 237)
	Death #, 2017	181 (168 to 194)	53 (49 to 56)	91 (85 to 98)	57 (54 to 59)	29 (28 to 30)	36 (36 to 37)	99 (97 to 101)	325 (303 to 347)	381 (357 to 405)	546 (520 to 571)	135 (133 to 138)
	% change 1990-2017	-44.8% (-49.6% to -40.2%)#	-44.8% (-50.0% to -39.4%)#	-66.2% (-69.1% to -63.1%)#	-55.1% (-59.3% to -50.2%)#	-61.3% (-62.7% to -59.9%)#	-55.6% (-56.6% to -54.7%)#	-61.8% (-62.6% to -60.9%)#	-53.2% (-56.6% to -49.7%)#	-53.5% (-56.7% to -50.4%)#	-55.8% (-58.1% to -53.6%)#	-60.3% (-61.1% to -59.4%)#
	% change 2000-2017	-8.2% (-15.6% to -0.1%)#	-17.6% (-24.8% to -9.6%)#	-21.9% (-27.8% to -14.9%)#	-23.3% (-30.4% to -16.1%)#	-46.7% (-48.9% to -44.5%)#	-44.8% (-46.2% to -43.5%)#	-41.2% (-42.5% to -39.7%)#	-14.0% (-20.4% to -7.2%)#	-15.6% (-21.4% to -9.4%)#	-26.3% (-30.1% to -22.2%)#	-42.2% (-43.5% to -40.9%)#
SW England	Death #, 1990	189 (178 to 200)	55 (51 to 58)	167 (158 to 176)	72 (66 to 79)	51 (50 to 52)	43 (43 to 43)	181 (180 to 182)	411 (397 to 424)	483 (470 to 495)	758 (744 to 772)	224 (223 to 226)
	Death #, 2000	126 (120 to 133)	38 (36 to 40)	76 (72 to 81)	47 (43 to 51)	31 (30 to 32)	41 (40 to 41)	109 (108 to 110)	240 (231 to 249)	287 (277 to 297)	468 (457 to 480)	150 (148 to 151)
	Death #, 2017	103 (94 to 112)	33 (30 to 35)	58 (54 to 63)	34 (32 to 36)	20 (19 to 21)	24 (24 to 25)	69 (68 to 71)	194 (179 to 208)	227 (212 to 243)	341 (324 to 358)	94 (92 to 96)
	% change 1990-2017	-45.7% (-51.4% to -39.7%)#	-40.5% (-46.9% to -33.6%)#	-64.9% (-68.2% to -61.6%)#	-53.5% (-58.3% to -48.3%)#	-61.1% (-62.7% to -59.5%)#	-43.4% (-44.8% to -42.0%)#	-61.7% (-62.6% to -60.9%)#	-52.8% (-56.7% to -48.8%)#	-52.9% (-56.4% to -49.2%)#	-55.0% (-57.4% to -52.5%)#	-58.2% (-59.1% to -57.3%)#
	% change 2000-2017	-18.8% (-26.9% to -10.4%)#	-14.1% (-22.9% to -4.7%)#	-23.0% (-30.4% to -15.4%)#	-28.5% (-34.9% to -21.8%)#	-36.3% (-39.2% to -33.2%)#	-40.1% (-41.7% to -38.4%)#	-36.3% (-37.8% to -34.8%)#	-19.4% (-26.2% to -12.4%)#	-20.9% (-27.0% to -14.6%)#	-27.2% (-31.3% to -23.0%)#	-37.4% (-38.8% to -35.9%)#
W Midlands	Death #, 1990	379 (361 to 398)	86 (81 to 92)	253 (237 to 271)	122 (111 to 135)	59 (58 to 60)	68 (67 to 69)	195 (193 to 196)	719 (693 to 745)	841 (816 to 865)	1,162 (1,135 to 1,189)	262 (261 to 264)
	Death #, 2000	234 (222 to 247)	57 (53 to 61)	109 (101 to 117)	65 (60 to 72)	40 (39 to 42)	54 (53 to 55)	131 (129 to 132)	400 (382 to 419)	466 (445 to 486)	691 (668 to 714)	185 (183 to 187)
	Death #, 2017	246 (224 to 271)	65 (59 to 71)	106 (98 to 115)	53 (50 to 57)	32 (31 to 33)	35 (35 to 36)	78 (77 to 80)	417 (382 to 457)	471 (434 to 512)	616 (578 to 660)	114 (112 to 116)
	% change 1990-2017	-35.1% (-41.4% to -27.3%)#	-24.6% (-32.3% to -15.0%)#	-58.0% (-62.6% to -53.2%)#	-56.2% (-61.0% to -50.9%)#	-46.5% (-48.4% to -44.5%)#	-47.8% (-49.0% to -46.6%)#	-59.7% (-60.6% to -58.7%)#	-41.9% (-47.4% to -35.8%)#	-44.0% (-48.7% to -38.6%)#	-47.0% (-50.6% to -43.0%)#	-56.6% (-57.5% to -55.6%)#
	% change 2000-2017	5.0% (-6.2% to 18.9%)	14.0% (1.3% to 29.1%)#	-2.3% (-11.8% to 9.5%)	-18.3% (-27.9% to -8.3%)#	-21.8% (-24.8% to -18.6%)#	-34.4% (-36.0% to -32.8%)#	-39.9% (-41.4% to -38.4%)#	4.3% (-5.8% to 16.6%)	1.1% (-8.2% to 11.8%)	-10.8% (-17.2% to -3.5%)#	-38.3% (-39.7% to -36.9%)#
Yorkshire & Humber	Death #, 1990	256 (245 to 269)	81 (77 to 86)	243 (229 to 258)	105 (97 to 114)	58 (57 to 59)	62 (61 to 62)	167 (166 to 168)	581 (563 to 599)	686 (667 to 706)	973 (952 to 995)	229 (227 to 231)
	Death #, 2000	179 (171 to 187)	59 (56 to 62)	125 (118 to 133)	74 (68 to 80)	42 (41 to 43)	51 (51 to 52)	128 (127 to 129)	363 (349 to 377)	437 (421 to 452)	658 (640 to 675)	179 (177 to 181)
	Death #, 2017	124 (114 to 133)	48 (45 to 52)	85 (79 to 91)	47 (44 to 49)	26 (25 to 27)	31 (31 to 32)	78 (76 to 80)	257 (239 to 275)	303 (284 to 324)	439 (418 to 461)	109 (107 to 112)
	% change 1990-2017	-51.8% (-55.9% to -47.1%)#	-40.9% (-46.4% to -35.2%)#	-65.2% (-68.2% to -61.6%)#	-55.5% (-60.0% to -50.7%)#	-55.2% (-56.9% to -53.5%)#	-49.2% (-50.3% to -48.1%)#	-53.3% (-54.3% to -52.2%)#	-55.8% (-59.2% to -52.3%)#	-55.8% (-58.9% to -52.5%)#	-54.9% (-57.3% to -52.4%)#	-52.2% (-53.2% to -51.1%)#
	% change 2000-2017	-30.7% (-36.9% to -23.9%)#	-18.2% (-25.3% to -10.1%)#	-32.4% (-38.1% to -26.1%)#	-36.9% (-43.2% to -30.2%)#	-38.3% (-40.8% to -35.7%)#	-39.0% (-40.4% to -37.5%)#	-38.8% (-40.3% to -37.4%)#	-29.3% (-34.8% to -23.4%)#	-30.6% (-35.7% to -25.0%)#	-33.3% (-36.9% to -29.3%)#	-38.9% (-40.2% to -37.5%)#
N Ireland	Death #, 1990	102 (82 to 128)	25 (20 to 31)	79 (66 to 92)	47 (33 to 67)	29 (28 to 30)	32 (31 to 33)	84 (82 to 85)	206 (184 to 234)	253 (237 to 273)	398 (378 to 421)	116 (113 to 118)
	Death #, 2000	74 (64 to 86)	16 (13 to 19)	31 (24 to 39)	22 (15 to 33)	19 (18 to 21)	21 (20 to 22)	69 (67 to 71)	121 (108 to 137)	143 (130 to 157)	252 (235 to 270)	90 (87 to 93)
	Death #, 2017	77 (61 to 98)	14 (11 to 17)	23 (16 to 32)	15 (12 to 19)	10 (8 to 11)	11 (10 to 12)	37 (34 to 41)	114 (92 to 142)	129 (103 to 160)	187 (157 to 221)	48 (44 to 52)
	% change 1990-2017	-24.6% (-44.8% to 3.7%)	-45.2% (-60.3% to -23.9%)#	-70.7% (-80.5% to -58.0%)#	-68.0% (-78.9% to -50.1%)#	-66.7% (-71.8% to -60.7%)#	-67.2% (-70.4% to -63.7%)#	-55.1% (-59.0% to -51.0%)#	-44.7% (-56.8% to -29.9%)#	-49.0% (-59.5% to -36.0%)#	-53.1% (-60.7% to -43.9%)#	-58.5% (-61.8% to -54.9%)#
	% change 2000-2017	3.9% (-18.3% to 33.0%)	-12.3% (-38.0% to 21.7%)	-26.1% (-53.1% to 15.5%)	-32.7% (-57.4% to 10.0%)	-49.9% (-58.1% to -38.9%)#	-50.1% (-55.1% to -43.9%)#	-45.4% (-50.3% to -40.1%)#	-5.9% (-26.4% to 21.3%)	-10.1% (-28.9% to 15.2%)	-26.1% (-37.6% to -10.8%)#	-46.5% (-51.2% to -41.7%)#
Scotland	Death #, 1990	240 (193 to 299)	69 (54 to 87)	194 (150 to 240)	104 (66 to 150)	54 (51 to 56)	78 (76 to 80)	196 (194 to 199)	503 (450 to 560)	607 (569 to 646)	935 (891 to 979)	274 (270 to 279)
	Death #, 2000	140 (117 to 169)	51 (39 to 65)	97 (71 to 134)	56 (37 to 82)	40 (37 to 43)	52 (50 to 54)	168 (164 to 173)	288 (258 to 322)	344 (311 to 383)	604 (564 to 650)	220 (214 to 226)
	Death #, 2017	96 (64 to 139)	36 (24 to 52)	63 (39 to 93)	36 (27 to 47)	28 (23 to 34)	27 (24 to 30)	96 (88 to 104)	194 (134 to 274)	231 (161 to 321)	382 (304 to 482)	123 (113 to 133)
	% change 1990-2017	-60.0% (-74.6% to -35.6%)#	-48.3% (-67.5% to -18.4%)#	-67.7% (-81.0% to -48.4%)#	-65.0% (-78.2% to -40.3%)#	-47.3% (-56.3% to -36.2%)#	-65.4% (-68.8% to -61.5%)#	-51.2% (-55.1% to -47.1%)#	-61.4% (-73.2% to -44.0%)#	-62.0% (-73.3% to -46.2%)#	-59.2% (-67.7% to -47.8%)#	-55.3% (-58.7% to -51.5%)#
	% change 2000-2017	-31.5% (-54.2% to 3.6%)	-29.4% (-55.4% to 12.5%)	-35.5% (-63.5% to 8.5%)	-35.8% (-59.6% to 8.3%)	-28.6% (-41.6% to -12.6%)#	-47.7% (-53.1% to -41.6%)#	-43.1% (-47.6% to -38.1%)#	-32.5% (-54.4% to -1.7%)#	-33.0% (-53.1% to -5.2%)#	-36.8% (-50.3% to -19.2%)#	-44.2% (-48.5% to -39.4%)#
Wales	Death #, 1990	126 (101 to 157)	36 (30 to 43)	98 (77 to 121)	57 (37 to 80)	35 (34 to 37)	35 (34 to 36)	99 (98 to 101)	260 (238 to 287)	317 (299 to 338)	486 (465 to 510)	134 (132 to 136)
	Death #, 2000	83 (73 to 95)	28 (24 to 34)	57 (44 to 73)	33 (22 to 48)	22 (20 to 23)	31 (29 to 32)	87 (84 to 89)	168 (151 to 187)	201 (183 to 220)	340 (319 to 363)	117 (114 to 121)
	Death #, 2017	57 (44 to 74)	21 (16 to 27)	36 (28 to 46)	19 (14 to 25)	15 (13 to 17)	14 (13 to 15)	51 (48 to 55)	114 (88 to 148)	133 (105 to 168)	214 (183 to 253)	65 (62 to 70)
	% change 1990-2017	-54.7% (-67.6% to -36.4%)#	-42.2% (-57.8% to -20.8%)#	-62.9% (-73.0% to -48.4%)#	-66.5% (-79.0% to -45.4%)#	-57.4% (-61.8% to -51.9%)#	-59.1% (-61.8% to -56.0%)#	-48.4% (-51.8% to -44.8%)#	-56.1% (-66.1% to -42.5%)#	-57.9% (-66.7% to -46.9%)#	-56.0% (-62.4% to -47.9%)#	-51.2% (-54.2% to -47.9%)#
	% change 2000-2017	-31.4% (-48.1% to -10.1%)#	-26.2% (-46.2% to 1.4%)	-35.6% (-55.0% to -6.8%)#	-41.9% (-60.3% to -14.0%)#	-30.9% (-39.3% to -21.4%)#	-53.4% (-56.6% to -49.5%)#	-41.0% (-45.1% to -36.7%)#	-31.9% (-48.1% to -9.6%)#	-33.6% (-47.6% to -14.3%)#	-37.1% (-46.5% to -24.6%)#	-44.2% (-47.8% to -40.3%)#
**Latin America and Caribbean**	Death #, 1990	167,686 (162,841 to 173,331)	59,535 (57,355 to 61,765)	240,213 (234,191 to 246,872)	122,916 (117,229 to 128,598)	36,251 (35,764 to 36,672)	27,248 (27,034 to 27,447)	48,168 (47,912 to 48,419)	467,434 (458,948 to 475,710)	590,350 (580,106 to 599,761)	702,017 (691,154 to 711,859)	75,415 (75,002 to 75,828)
	% of global total, 1990	5.5% (5.3% to 5.8%)	4.7% (4.5% to 5.0%)	6.2% (6.0% to 6.4%)	3.4% (3.2% to 3.6%)	4.2% (4.2% to 4.3%)	5.8% (5.8% to 5.9%)	7.2% (7.1% to 7.2%)	5.7% (5.6% to 5.9%)	5.0% (4.9% to 5.1%)	5.1% (5.0% to 5.2%)	6.6% (6.5% to 6.7%)
	Death #, 2000	123,160 (119,683 to 126,987)	36,480 (35,346 to 37,726)	141,458 (137,963 to 145,260)	66,995 (64,343 to 69,596)	25,051 (24,691 to 25,421)	22,359 (22,161 to 22,556)	50,919 (50,599 to 51,223)	301,098 (294,715 to 307,502)	368,093 (360,680 to 375,865)	466,422 (458,278 to 474,745)	73,278 (72,793 to 73,747)
	% of global total, 2000	4.5% (4.3% to 4.6%)	4.0% (3.8% to 4.2%)	4.6% (4.4% to 4.7%)	2.3% (2.2% to 2.4%)	3.6% (3.5% to 3.6%)	4.6% (4.6% to 4.7%)	7.5% (7.4% to 7.6%)	4.5% (4.3% to 4.6%)	3.8% (3.7% to 3.9%)	4.0% (4.0% to 4.1%)	6.3% (6.2% to 6.4%)
	Death #, 2017	64,395 (61,275 to 68,162)	20,011 (18,874 to 21,350)	65,768 (61,832 to 69,879)	29,853 (28,343 to 31,577)	15,167 (14,812 to 15,588)	16,965 (16,720 to 17,241)	49,767 (48,771 to 50,751)	150,174 (142,977 to 158,312)	180,026 (171,436 to 189,920)	261,925 (252,797 to 272,512)	66,732 (65,610 to 67,918)
	% of global total, 2017	3.5% (3.2% to 3.7%)	3.9% (3.7% to 4.2%)	4.1% (3.8% to 4.4%)	2.1% (2.0% to 2.3%)	3.7% (3.6% to 3.8%)	5.3% (5.2% to 5.4%)	9.6% (9.4% to 9.9%)	3.8% (3.5% to 4.0%)	3.3% (3.1% to 3.6%)	3.9% (3.8% to 4.2%)	8.0% (7.8% to 8.2%)
	% change 1990-2017	-61.6% (-63.9% to -59.1%)#	-66.4% (-68.6% to -63.7%)#	-72.6% (-74.3% to -70.7%)#	-75.7% (-77.3% to -74.0%)#	-58.2% (-59.2% to -56.9%)#	-37.7% (-38.8% to -36.7%)#	3.3% (1.2% to 5.5%)#	-67.9% (-69.4% to -66.0%)#	-69.5% (-71.0% to -67.8%)#	-62.7% (-64.0% to -61.1%)#	-11.5% (-13.1% to -9.8%)#

eTable 2. ENN, LNN, PNN, 1-4, 5-9, 10-14, 15-19, <1, <5, 10-19, and <20 years mortality in 1990, 2000 and 2017 with mean percentage change for all GBD causes.

	Metric	Early Neonatal	Late Neonatal	Post Neonatal	1 to 4	5 to 9	10 to 14	15 to 19	<1 year	Under 5	<20 years	10 to 19
	% change 2000-2017	-47.7% (-50.5% to -44.5%)#	-45.1% (-48.7% to -41.1%)#	-53.5% (-56.4% to -50.4%)#	-55.4% (-58.2% to -52.1%)#	-39.5% (-41.1% to -37.7%)#	-24.1% (-25.3% to -22.9%)#	-2.3% (-4.3% to -0.3%)#	-50.1% (-52.6% to -47.2%)#	-51.1% (-53.5% to -48.3%)#	-43.8% (-45.9% to -41.5%)#	-8.9% (-10.6% to -7.3%)#
Andean Latin America	Death #, 1990	22,212 (19,482 to 25,282)	10,691 (9,454 to 12,108)	33,994 (30,393 to 37,731)	28,614 (25,847 to 31,541)	5,392 (5,179 to 5,616)	3,767 (3,666 to 3,869)	5,107 (4,937 to 5,290)	66,897 (62,370 to 71,462)	95,512 (90,174 to 100,529)	109,777 (104,168 to 115,157)	8,874 (8,620 to 9,137)
	% of global total, 1990	0.7% (0.6% to 0.8%)	0.8% (0.8% to 1.0%)	0.9% (0.8% to 1.0%)	0.8% (0.7% to 0.9%)	0.6% (0.6% to 0.7%)	0.8% (0.8% to 0.8%)	0.8% (0.7% to 0.8%)	0.8% (0.8% to 0.9%)	0.8% (0.8% to 0.9%)	0.8% (0.8% to 0.8%)	0.8% (0.8% to 0.8%)
	Death #, 2000	15,450 (14,108 to 16,850)	6,183 (5,627 to 6,763)	18,706 (17,146 to 20,414)	13,513 (12,072 to 15,016)	4,060 (3,872 to 4,245)	3,015 (2,921 to 3,108)	4,474 (4,321 to 4,625)	40,339 (37,834 to 43,012)	53,852 (50,622 to 57,492)	65,402 (61,951 to 69,222)	7,489 (7,255 to 7,718)
	% of global total, 2000	0.6% (0.5% to 0.6%)	0.7% (0.6% to 0.7%)	0.6% (0.6% to 0.7%)	0.5% (0.4% to 0.5%)	0.6% (0.6% to 0.6%)	0.6% (0.6% to 0.6%)	0.7% (0.6% to 0.7%)	0.6% (0.6% to 0.6%)	0.6% (0.5% to 0.6%)	0.6% (0.5% to 0.6%)	0.6% (0.6% to 0.7%)
	Death #, 2017	8,577 (7,043 to 10,299)	2,677 (2,120 to 3,330)	8,313 (6,886 to 9,925)	5,056 (4,279 to 5,925)	2,438 (2,237 to 2,640)	2,050 (1,935 to 2,165)	3,789 (3,528 to 4,064)	19,567 (16,227 to 23,208)	24,623 (20,514 to 29,113)	32,900 (28,460 to 37,739)	5,840 (5,490 to 6,206)
	% of global total, 2017	0.5% (0.4% to 0.6%)	0.5% (0.4% to 0.7%)	0.5% (0.4% to 0.6%)	0.4% (0.3% to 0.4%)	0.6% (0.5% to 0.6%)	0.6% (0.6% to 0.7%)	0.7% (0.7% to 0.8%)	0.5% (0.4% to 0.6%)	0.5% (0.4% to 0.5%)	0.5% (0.4% to 0.6%)	0.7% (0.7% to 0.7%)
	% change 1990-2017	-61.4% (-69.9% to -51.9%)#	-75.0% (-80.9% to -68.2%)#	-75.5% (-80.1% to -70.0%)#	-82.3% (-85.6% to -78.7%)#	-54.8% (-58.9% to -50.3%)#	-45.6% (-49.0% to -41.9%)#	-25.8% (-31.7% to -19.7%)#	-70.8% (-76.0% to -64.5%)#	-74.2% (-78.9% to -69.0%)#	-70.0% (-74.4% to -65.0%)#	-34.2% (-38.7% to -29.5%)#
	% change 2000-2017	-44.5% (-55.0% to -32.6%)#	-56.7% (-66.9% to -44.8%)#	-55.6% (-63.6% to -46.1%)#	-62.6% (-69.5% to -54.5%)#	-40.0% (-45.6% to -33.8%)#	-32.0% (-36.3% to -27.5%)#	-15.3% (-21.8% to -8.7%)#	-51.5% (-60.4% to -42.2%)#	-54.3% (-62.5% to -45.3%)#	-49.7% (-57.2% to -41.8%)#	-22.0% (-27.1% to -16.5%)#
Bolivia	Death #, 1990	5,626 (4,959 to 6,437)	3,037 (2,474 to 3,648)	9,850 (8,619 to 11,066)	9,181 (7,996 to 10,480)	1,198 (1,120 to 1,282)	715 (678 to 752)	965 (895 to 1,038)	18,512 (16,971 to 20,104)	27,693 (25,718 to 29,959)	30,572 (28,499 to 32,930)	1,680 (1,576 to 1,784)
	Death #, 2000	4,686 (4,094 to 5,312)	2,053 (1,745 to 2,393)	6,160 (5,191 to 7,288)	5,596 (4,753 to 6,462)	971 (891 to 1,045)	648 (609 to 685)	946 (863 to 1,019)	12,899 (11,451 to 14,515)	18,495 (16,549 to 20,590)	21,061 (19,042 to 23,314)	1,594 (1,478 to 1,697)
	Death #, 2017	2,732 (1,931 to 3,792)	1,004 (712 to 1,390)	2,518 (1,792 to 3,467)	1,573 (1,143 to 2,135)	588 (518 to 653)	422 (383 to 459)	787 (669 to 878)	6,255 (4,440 to 8,642)	7,828 (5,580 to 10,750)	9,624 (7,311 to 12,651)	1,208 (1,069 to 1,323)
	% change 1990-2017	-51.4% (-66.9% to -30.8%)#	-66.9% (-77.8% to -51.3%)#	-74.4% (-82.4% to -63.8%)#	-82.9% (-87.9% to -75.8%)#	-50.9% (-57.8% to -44.4%)#	-41.0% (-47.3% to -34.9%)#	-18.5% (-32.7% to -5.6%)#	-66.2% (-76.7% to -52.6%)#	-71.7% (-80.4% to -60.9%)#	-68.5% (-76.5% to -58.2%)#	-28.1% (-37.3% to -19.1%)#
	% change 2000-2017	-41.7% (-59.5% to -20.1%)#	-51.1% (-66.2% to -32.3%)#	-59.1% (-71.8% to -42.3%)#	-71.9% (-80.1% to -61.0%)#	-39.4% (-47.3% to -30.8%)#	-35.0% (-41.5% to -27.5%)#	-16.9% (-29.5% to -4.7%)#	-51.5% (-65.7% to -32.8%)#	-57.7% (-69.8% to -42.4%)#	-54.3% (-65.6% to -40.0%)#	-24.2% (-33.3% to -15.0%)#
Ecuador	Death #, 1990	3,321 (2,741 to 3,909)	1,222 (999 to 1,476)	4,407 (3,768 to 5,075)	3,394 (2,835 to 4,033)	1,019 (963 to 1,078)	855 (830 to 881)	1,212 (1,197 to 1,225)	8,950 (8,047 to 9,858)	12,343 (11,413 to 13,430)	15,429 (14,407 to 16,616)	2,067 (2,028 to 2,105)
	Death #, 2000	3,798 (3,063 to 4,593)	1,208 (934 to 1,540)	4,008 (3,352 to 4,749)	1,621 (1,211 to 2,075)	1,000 (896 to 1,106)	864 (816 to 914)	1,430 (1,404 to 1,456)	9,013 (7,661 to 10,465)	10,634 (9,011 to 12,263)	13,928 (12,153 to 15,711)	2,295 (2,224 to 2,369)
	Death #, 2017	1,741 (1,202 to 2,484)	660 (321 to 1,206)	1,707 (1,141 to 2,377)	1,184 (893 to 1,572)	680 (586 to 790)	690 (631 to 751)	1,467 (1,342 to 1,616)	4,109 (2,897 to 5,720)	5,293 (3,799 to 7,289)	8,130 (6,483 to 10,312)	2,157 (1,987 to 2,356)
	% change 1990-2017	-47.6% (-65.7% to -21.6%)#	-45.9% (-74.2% to 2.8%)	-61.2% (-75.0% to -43.5%)#	-65.1% (-75.1% to -52.4%)#	-33.2% (-42.5% to -21.7%)#	-19.4% (-26.3% to -11.8%)#	21.1% (10.7% to 33.5%)#	-54.1% (-67.9% to -35.0%)#	-57.1% (-69.5% to -40.1%)#	-47.3% (-58.4% to -32.6%)#	4.4% (-3.6% to 14.1%)
	% change 2000-2017	-54.2% (-69.5% to -34.3%)#	-45.3% (-76.3% to 14.2%)	-57.4% (-72.9% to -35.9%)#	-26.9% (-51.4% to 11.2%)	-31.9% (-43.6% to -18.0%)#	-20.2% (-28.1% to -11.1%)#	2.6% (-6.5% to 13.3%)	-54.4% (-69.5% to -34.3%)#	-50.2% (-66.2% to -29.2%)#	-41.6% (-55.6% to -23.7%)#	-6.0% (-14.0% to 3.1%)
Peru	Death #, 1990	13,265 (10,614 to 16,110)	6,433 (5,348 to 7,781)	19,737 (16,378 to 23,129)	16,040 (13,655 to 18,778)	3,175 (2,983 to 3,371)	2,196 (2,102 to 2,289)	2,930 (2,771 to 3,078)	39,435 (35,550 to 43,330)	55,475 (51,013 to 60,542)	63,777 (58,972 to 69,256)	5,127 (4,893 to 5,352)
	Death #, 2000	6,967 (6,055 to 7,971)	2,922 (2,544 to 3,306)	8,538 (7,490 to 9,839)	6,297 (5,131 to 7,599)	2,090 (1,954 to 2,233)	1,503 (1,430 to 1,576)	2,097 (1,961 to 2,228)	18,427 (16,800 to 20,460)	24,723 (22,388 to 27,310)	30,413 (27,882 to 33,162)	3,600 (3,410 to 3,797)
	Death #, 2017	4,104 (3,085 to 5,397)	1,012 (767 to 1,323)	4,088 (3,098 to 5,363)	2,299 (1,794 to 2,961)	1,169 (1,010 to 1,357)	939 (849 to 1,040)	1,535 (1,335 to 1,770)	9,203 (6,949 to 12,079)	11,502 (8,742 to 15,044)	15,146 (12,145 to 18,804)	2,474 (2,184 to 2,767)
	% change 1990-2017	-69.1% (-78.5% to -56.5%)#	-84.3% (-88.8% to -77.9%)#	-79.3% (-85.0% to -71.4%)#	-85.7% (-89.5% to -80.7%)#	-63.2% (-68.6% to -56.6%)#	-57.3% (-61.8% to -52.3%)#	-47.6% (-54.8% to -39.4%)#	-76.7% (-82.8% to -69.1%)#	-79.3% (-84.5% to -72.4%)#	-76.2% (-81.4% to -69.8%)#	-51.7% (-57.5% to -45.2%)#
	% change 2000-2017	-41.1% (-57.0% to -20.2%)#	-65.4% (-74.5% to -52.2%)#	-52.1% (-65.2% to -35.7%)#	-63.5% (-72.9% to -50.4%)#	-44.0% (-52.3% to -34.0%)#	-37.5% (-43.9% to -30.2%)#	-26.8% (-37.5% to -15.2%)#	-50.0% (-63.0% to -33.0%)#	-53.5% (-64.8% to -38.4%)#	-50.2% (-60.5% to -37.0%)#	-31.3% (-39.6% to -22.4%)#
Caribbean	Death #, 1990	14,363 (12,619 to 16,229)	6,916 (5,717 to 8,309)	22,048 (20,217 to 23,950)	17,389 (14,726 to 20,118)	3,165 (3,051 to 3,276)	2,134 (2,074 to 2,190)	3,787 (3,683 to 3,887)	43,326 (40,614 to 46,157)	60,715 (57,368 to 64,110)	69,800 (66,212 to 73,305)	5,920 (5,764 to 6,075)
	% of global total, 1990	0.5% (0.4% to 0.5%)	0.6% (0.5% to 0.7%)	0.6% (0.5% to 0.6%)	0.5% (0.4% to 0.6%)	0.4% (0.4% to 0.4%)	0.5% (0.4% to 0.5%)	0.6% (0.5% to 0.6%)	0.5% (0.5% to 0.6%)	0.5% (0.5% to 0.6%)	0.5% (0.5% to 0.5%)	0.5% (0.5% to 0.5%)
	Death #, 2000	12,481 (11,165 to 13,830)	4,913 (4,331 to 5,539)	15,071 (13,569 to 16,810)	10,830 (9,394 to 12,424)	2,715 (2,579 to 2,866)	2,009 (1,926 to 2,115)	3,249 (3,097 to 3,406)	32,466 (30,213 to 35,014)	43,296 (40,192 to 46,794)	51,268 (47,909 to 55,094)	5,258 (5,045 to 5,495)
	% of global total, 2000	0.5% (0.4% to 0.5%)	0.5% (0.5% to 0.6%)	0.5% (0.4% to 0.5%)	0.4% (0.3% to 0.4%)	0.4% (0.4% to 0.4%)	0.4% (0.4% to 0.4%)	0.5% (0.5% to 0.5%)	0.5% (0.5% to 0.5%)	0.5% (0.4% to 0.5%)	0.4% (0.4% to 0.5%)	0.5% (0.4% to 0.5%)
	Death #, 2017	10,539 (8,742 to 12,551)	3,531 (2,920 to 4,208)	9,510 (7,018 to 12,722)	5,505 (4,628 to 6,560)	1,902 (1,755 to 2,067)	1,720 (1,625 to 1,844)	3,307 (3,058 to 3,598)	23,580 (19,877 to 27,773)	29,085 (24,537 to 34,308)	36,014 (31,219 to 41,385)	5,026 (4,703 to 5,423)
	% of global total, 2017	0.6% (0.5% to 0.7%)	0.7% (0.6% to 0.8%)	0.6% (0.4% to 0.8%)	0.4% (0.3% to 0.5%)	0.5% (0.4% to 0.5%)	0.5% (0.5% to 0.6%)	0.6% (0.6% to 0.7%)	0.6% (0.5% to 0.7%)	0.5% (0.5% to 0.6%)	0.5% (0.5% to 0.6%)	0.6% (0.6% to 0.7%)
	% change 1990-2017	-26.6% (-41.5% to -7.2%)#	-49.0% (-60.8% to -33.9%)#	-56.9% (-69.1% to -41.4%)#	-68.3% (-75.0% to -60.3%)#	-39.9% (-45.0% to -34.0%)#	-19.4% (-24.3% to -13.1%)#	-12.7% (-19.5% to -4.3%)#	-45.6% (-55.0% to -35.6%)#	-52.1% (-59.9% to -43.3%)#	-48.4% (-55.6% to -40.6%)#	-15.1% (-20.8% to -7.8%)#
	% change 2000-2017	-15.6% (-31.2% to 3.9%)	-28.1% (-42.0% to -11.0%)#	-36.9% (-53.8% to -16.3%)#	-49.2% (-59.2% to -37.2%)#	-29.9% (-36.7% to -22.9%)#	-14.4% (-20.9% to -7.5%)#	1.8% (-5.9% to 10.9%)	-27.4% (-39.9% to -13.1%)#	-32.8% (-44.2% to -19.6%)#	-29.8% (-39.8% to -18.2%)#	-4.4% (-11.0% to 3.1%)
Antigua	Death #, 1990	7 (6 to 9)	2 (2 to 2)	3 (2 to 4)	4 (3 to 5)	2 (2 to 2)	2 (2 to 2)	3 (3 to 4)	12 (10 to 14)	16 (13 to 19)	23 (20 to 27)	5 (5 to 6)
	Death #, 2000	10 (8 to 12)	3 (2 to 4)	4 (3 to 5)	4 (3 to 5)	2 (2 to 3)	2 (2 to 2)	4 (4 to 4)	17 (13 to 20)	20 (16 to 25)	29 (24 to 34)	6 (6 to 7)
	Death #, 2017	4 (3 to 6)	2 (1 to 3)	3 (2 to 4)	4 (2 to 5)	2 (1 to 2)	2 (2 to 2)	5 (5 to 5)	9 (7 to 13)	13 (9 to 19)	22 (17 to 28)	7 (6 to 7)
	% change 1990-2017	-38.2% (-59.0% to -9.7%)#	8.8% (-30.4% to 64.7%)	-2.8% (-40.9% to 63.0%)	-2.6% (-37.6% to 55.5%)	-9.5% (-25.6% to 10.4%)	-16.0% (-24.9% to -6.2%)#	45.1% (34.3% to 57.1%)#	-22.2% (-48.2% to 13.9%)	-17.6% (-45.2% to 21.7%)	-7.5% (-30.0% to 21.5%)	22.4% (12.9% to 32.9%)#
	% change 2000-2017	-55.1% (-69.8% to -34.3%)#	-25.9% (-48.5% to 10.2%)	-24.1% (-48.3% to 14.2%)	3.1% (-31.1% to 55.0%)	-28.9% (-40.8% to -14.4%)#	-16.2% (-25.3% to -6.0%)#	20.8% (10.9% to 31.7%)#	-43.1% (-60.5% to -20.2%)#	-34.9% (-55.0% to -9.1%)#	-25.1% (-41.6% to -4.4%)#	8.5% (-0.3% to 18.2%)
Bahamas	Death #, 1990	54 (44 to 65)	14 (10 to 19)	34 (26 to 43)	19 (14 to 24)	13 (12 to 15)	10 (9 to 11)	19 (19 to 20)	103 (91 to 117)	121 (105 to 140)	163 (145 to 185)	29 (28 to 30)
	Death #, 2000	27 (24 to 31)	11 (7 to 17)	27 (22 to 31)	16 (13 to 19)	16 (14 to 17)	11 (10 to 11)	24 (23 to 25)	65 (56 to 76)	81 (70 to 93)	132 (117 to 146)	35 (33 to 36)
	Death #, 2017	23 (17 to 29)	9 (5 to 16)	19 (14 to 24)	11 (8 to 14)	8 (7 to 10)	10 (10 to 11)	26 (24 to 28)	50 (37 to 68)	60 (45 to 82)	105 (87 to 130)	36 (34 to 39)
	% change 1990-2017	-58.5% (-70.2% to -41.6%)#	-37.2% (-69.2% to 27.4%)	-45.7% (-61.7% to -22.1%)#	-43.2% (-61.4% to -17.3%)#	-37.4% (-47.4% to -25.0%)#	3.4% (-7.0% to 14.9%)	35.2% (23.6% to 47.2%)#	-51.3% (-64.4% to -32.9%)#	-50.1% (-63.5% to -31.0%)#	-35.8% (-48.2% to -20.0%)#	24.2% (14.1% to 34.8%)#
	% change 2000-2017	-17.6% (-38.0% to 10.4%)	-22.3% (-60.6% to 57.9%)	-30.2% (-48.1% to -4.5%)#	-34.9% (-53.4% to -7.1%)#	-47.0% (-55.2% to -36.1%)#	-1.4% (-10.9% to 9.5%)	7.2% (-1.6% to 16.7%)	-23.5% (-44.0% to 7.2%)	-25.8% (-45.6% to 3.9%)	-20.3% (-35.0% to 1.0%)	4.6% (-4.1% to 13.4%)
	Death #, 1990	38 (31 to 45)	8 (6 to 9)	14 (12 to 18)	9 (7 to 11)	7 (6 to 8)	9 (9 to 10)	17 (17 to 18)	60 (50 to 70)	68 (57 to 81)	102 (90 to 117)	27 (26 to 28)
	Death #, 2000	24 (19 to 29)	7 (5 to 9)	12 (8 to 16)	6 (4 to 8)	3 (3 to 4)	8 (8 to 9)	17 (17 to 18)	43 (34 to 54)	48 (38 to 62)	77 (66 to 91)	26 (25 to 27)

eTable 2. ENN, LNN, PNN, 1-4, 5-9, 10-14, 15-19, <1, <5, 10-19, and <20 years mortality in 1990, 2000 and 2017 with mean percentage change for all GBD causes.

	Metric	Early Neonatal	Late Neonatal	Post Neonatal	1 to 4	5 to 9	10 to 14	15 to 19	<1 year	Under 5	<20 years	10 to 19
Barbados	Death #, 2017	19 (13 to 27)	5 (4 to 8)	8 (5 to 11)	3 (2 to 4)	3 (3 to 4)	8 (7 to 9)	12 (11 to 13)	32 (22 to 46)	35 (24 to 50)	58 (46 to 74)	20 (19 to 21)
	% change 1990-2017	-50.1% (-67.9% to -24.4%)#	-30.6% (-54.8% to 5.6%)	-46.9% (-65.9% to -20.5%)#	-65.0% (-77.7% to -45.1%)#	-56.8% (-63.9% to -48.1%)#	-15.7% (-23.7% to -6.3%)#	-30.8% (-36.5% to -24.7%)#	-46.8% (-65.3% to -19.4%)#	-49.1% (-66.6% to -22.7%)#	-43.5% (-57.0% to -24.1%)#	-25.5% (-31.2% to -19.1%)#
	% change 2000-2017	-21.3% (-47.1% to 20.2%)	-21.9% (-51.2% to 28.0%)	-35.6% (-58.5% to 4.1%)	-47.5% (-65.4% to -20.0%)#	-7.5% (-22.3% to 9.6%)	-2.6% (-11.1% to 7.5%)	-31.1% (-36.8% to -24.6%)#	-25.4% (-50.1% to 14.1%)	-28.0% (-52.1% to 9.6%)	-25.1% (-42.3% to -0.8%)#	-22.0% (-28.0% to -15.5%)#
Belize	Death #, 1990	79 (64 to 96)	18 (15 to 21)	95 (81 to 109)	56 (46 to 67)	20 (18 to 22)	13 (12 to 13)	16 (15 to 17)	192 (169 to 216)	248 (222 to 274)	297 (268 to 326)	29 (28 to 30)
	Death #, 2000	71 (58 to 85)	17 (14 to 19)	59 (50 to 69)	34 (29 to 41)	19 (17 to 20)	17 (16 to 17)	34 (33 to 34)	147 (125 to 170)	181 (159 to 205)	250 (226 to 276)	50 (49 to 51)
	Death #, 2017	60 (48 to 76)	25 (18 to 37)	40 (28 to 59)	22 (17 to 29)	12 (11 to 14)	15 (14 to 16)	42 (40 to 44)	126 (94 to 171)	148 (112 to 200)	217 (177 to 274)	56 (54 to 59)
	% change 1990-2017	-23.9% (-43.8% to 3.0%)	41.8% (-4.9% to 107.2%)	-57.4% (-71.6% to -37.5%)#	-60.2% (-71.3% to -44.8%)#	-39.0% (-47.0% to -29.3%)#	13.0% (4.4% to 23.2%)#	162.3% (147.6% to 179.3%)#	-34.3% (-52.0% to -9.6%)#	-40.1% (-56.1% to -20.0%)#	-26.8% (-41.4% to -8.0%)#	95.6% (84.8% to 107.7%)#
	% change 2000-2017	-14.8% (-35.9% to 16.0%)	53.7% (5.2% to 123.3%)#	-31.6% (-53.9% to -0.0%)#	-35.1% (-53.4% to -10.3%)#	-34.7% (-43.3% to -24.5%)#	-12.6% (-18.9% to -5.1%)#	24.8% (19.5% to 30.6%)#	-13.9% (-36.5% to 18.2%)	-17.9% (-39.1% to 10.9%)	-13.1% (-30.0% to 9.3%)	12.4% (7.2% to 18.4%)#
Bermuda	Death #, 1990	3 (3 to 4)	1 (1 to 1)	2 (2 to 3)	2 (2 to 3)	1 (1 to 2)	1 (1 to 2)	3 (3 to 3)	7 (6 to 8)	9 (8 to 10)	15 (13 to 17)	4 (4 to 5)
	Death #, 2000	2 (1 to 2)	0 (0 to 1)	1 (1 to 1)	1 (1 to 2)	1 (1 to 1)	1 (1 to 1)	2 (2 to 2)	3 (3 to 4)	4 (4 to 5)	8 (7 to 9)	3 (2 to 3)
	Death #, 2017	1 (1 to 1)	0 (0 to 0)	1 (0 to 1)	1 (1 to 1)	0 (0 to 0)	0 (0 to 0)	1 (1 to 1)	2 (2 to 3)	3 (2 to 4)	5 (4 to 6)	2 (1 to 2)
	% change 1990-2017	-65.1% (-73.6% to -54.2%)#	-66.8% (-75.6% to -54.5%)#	-75.1% (-81.9% to -64.7%)#	-60.3% (-69.7% to -46.0%)#	-72.2% (-76.2% to -66.7%)#	-73.2% (-76.1% to -69.4%)#	-60.8% (-65.3% to -55.0%)#	-68.9% (-76.0% to -60.0%)#	-66.7% (-73.6% to -58.1%)#	-66.8% (-72.2% to -60.0%)#	-65.0% (-69.0% to -59.9%)#
	% change 2000-2017	-29.1% (-48.4% to -0.9%)#	-33.7% (-51.0% to -12.5%)#	-37.7% (-54.1% to -16.3%)#	-36.3% (-43.9% to -27.4%)#	-49.8% (-57.0% to -40.2%)#	-54.7% (-60.1% to -48.2%)#	-33.5% (-41.8% to -23.5%)#	-32.4% (-47.1% to -12.8%)#	-33.6% (-44.5% to -21.0%)#	-37.6% (-46.0% to -26.8%)#	-40.8% (-48.1% to -32.2%)#
Cuba	Death #, 1990	872 (753 to 985)	313 (263 to 369)	712 (620 to 801)	491 (399 to 598)	274 (267 to 280)	330 (325 to 335)	1005 (997 to 1013)	1897 (1773 to 2010)	2388 (2308 to 2467)	3997 (3903 to 4086)	1335 (1323 to 1347)
	Death #, 2000	413 (351 to 473)	198 (166 to 233)	366 (307 to 432)	277 (225 to 334)	211 (203 to 218)	263 (258 to 268)	423 (419 to 427)	977 (905 to 1052)	1254 (1196 to 1319)	2151 (2078 to 2234)	686 (678 to 694)
	Death #, 2017	166 (139 to 196)	87 (72 to 103)	179 (151 to 210)	149 (126 to 176)	100 (89 to 112)	120 (111 to 131)	277 (251 to 307)	432 (362 to 510)	581 (490 to 684)	1079 (960 to 1219)	397 (363 to 436)
	% change 1990-2017	-80.9% (-84.6% to -76.2%)#	-72.2% (-78.2% to -64.2%)#	-74.9% (-79.9% to -69.1%)#	-69.6% (-76.8% to -61.2%)#	-63.5% (-67.7% to -58.9%)#	-63.7% (-66.5% to -60.4%)#	-72.4% (-75.1% to -69.3%)#	-77.2% (-81.0% to -72.8%)#	-75.7% (-79.5% to -71.2%)#	-73.0% (-76.0% to -69.4%)#	-70.2% (-72.8% to -67.2%)#
	% change 2000-2017	-59.7% (-67.6% to -50.1%)#	-56.1% (-64.9% to -44.0%)#	-51.1% (-60.5% to -38.2%)#	-46.1% (-58.2% to -28.5%)#	-52.6% (-58.0% to -46.6%)#	-54.4% (-57.9% to -50.3%)#	-34.5% (-40.7% to -27.3%)#	-55.8% (-62.9% to -46.7%)#	-53.6% (-61.0% to -45.1%)#	-49.9% (-55.6% to -43.1%)#	-42.1% (-47.2% to -36.4%)#
Dominica	Death #, 1990	13 (11 to 16)	3 (3 to 4)	8 (6 to 9)	5 (4 to 6)	2 (2 to 3)	4 (4 to 4)	7 (7 to 8)	24 (20 to 29)	29 (24 to 35)	43 (37 to 49)	11 (11 to 12)
	Death #, 2000	11 (8 to 13)	3 (2 to 4)	6 (5 to 7)	4 (3 to 4)	2 (2 to 2)	4 (4 to 4)	7 (6 to 7)	19 (15 to 23)	23 (18 to 28)	35 (30 to 41)	10 (10 to 11)
	Death #, 2017	13 (9 to 18)	4 (3 to 5)	8 (5 to 11)	4 (3 to 5)	2 (2 to 2)	4 (4 to 4)	6 (5 to 6)	24 (17 to 34)	28 (19 to 39)	39 (30 to 51)	10 (9 to 10)
	% change 1990-2017	-3.8% (-35.6% to 42.4%)	5.5% (-29.2% to 55.9%)	1.4% (-31.4% to 50.0%)	-28.6% (-51.1% to 5.5%)	-21.2% (-33.7% to -5.7%)#	-4.7% (-13.8% to 5.8%)	-20.5% (-26.0% to -14.7%)#	-0.9% (-33.4% to 46.5%)	-5.7% (-36.2% to 39.5%)	-8.9% (-31.9% to 23.5%)	-14.8% (-20.8% to -8.3%)#
	% change 2000-2017	21.5% (-14.5% to 70.6%)	26.0% (-11.3% to 76.4%)	31.8% (-7.3% to 83.7%)	-1.4% (-30.8% to 38.7%)	-11.4% (-25.3% to 5.7%)	2.9% (-7.0% to 13.5%)	-11.1% (-17.2% to -4.6%)#	25.3% (-11.7% to 75.3%)	21.0% (-14.6% to 68.3%)	11.3% (-14.9% to 44.6%)	-5.9% (-12.9% to 1.1%)
Dominican Rep	Death #, 1990	4,620 (3,644 to 5,636)	1,514 (1,220 to 1,822)	5,802 (4,904 to 6,779)	3,325 (2,607 to 4,111)	741 (691 to 796)	442 (421 to 464)	658 (614 to 705)	11,936 (10,613 to 13,356)	15,261 (13,740 to 16,940)	17,102 (15,482 to 18,836)	1,099 (1,037 to 1,166)
	Death #, 2000	4,107 (3,291 to 5,005)	1,149 (946 to 1,374)	2,541 (2,022 to 3,086)	1,671 (1,303 to 2,159)	568 (524 to 612)	401 (378 to 424)	617 (566 to 668)	7,797 (6,658 to 9,014)	9,469 (8,239 to 10,772)	11,054 (9,775 to 12,443)	1,018 (949 to 1,086)
	Death #, 2017	3,054 (2,171 to 4,230)	738 (525 to 1,019)	1,240 (884 to 1,694)	705 (519 to 940)	370 (319 to 431)	372 (334 to 411)	784 (693 to 880)	5,033 (3,583 to 6,945)	5,738 (4,096 to 7,883)	7,264 (5,508 to 9,459)	1,156 (1,034 to 1,284)
	% change 1990-2017	-33.9% (-56.7% to -2.1%)#	-51.2% (-66.7% to -29.7%)#	-78.6% (-85.0% to -69.4%)#	-78.8% (-85.5% to -68.8%)#	-50.0% (-57.7% to -41.1%)#	-15.8% (-25.3% to -5.5%)#	19.3% (2.8% to 37.7%)#	-57.8% (-71.0% to -40.9%)#	-62.4% (-73.9% to -47.1%)#	-57.5% (-68.8% to -43.1%)#	5.2% (-7.6% to 18.9%)
	% change 2000-2017	-25.6% (-49.0% to 9.6%)	-35.8% (-56.4% to -6.5%)#	-51.2% (-67.0% to -28.4%)#	-57.8% (-72.3% to -39.1%)#	-34.8% (-44.8% to -22.8%)#	-7.3% (-18.4% to 3.9%)	27.2% (9.7% to 46.6%)#	-35.5% (-54.7% to -7.8%)#	-39.4% (-57.3% to -14.6%)#	-34.3% (-50.7% to -11.4%)#	13.6% (-0.2% to 28.0%)
Grenada	Death #, 1990	19 (14 to 24)	5 (4 to 6)	13 (10 to 18)	10 (7 to 14)	5 (4 to 5)	4 (4 to 5)	7 (7 to 7)	37 (28 to 48)	47 (36 to 61)	63 (51 to 78)	11 (11 to 12)
	Death #, 2000	17 (14 to 21)	4 (3 to 4)	8 (6 to 11)	6 (4 to 7)	3 (3 to 4)	4 (3 to 4)	7 (6 to 7)	29 (23 to 36)	35 (28 to 43)	48 (41 to 57)	10 (10 to 11)
	Death #, 2017	10 (7 to 14)	2 (1 to 3)	4 (2 to 6)	3 (1 to 5)	2 (2 to 3)	3 (3 to 3)	5 (5 to 5)	16 (11 to 22)	19 (13 to 27)	29 (22 to 38)	8 (8 to 9)
	% change 1990-2017	-46.0% (-63.7% to -19.2%)#	-61.0% (-74.2% to -40.6%)#	-71.9% (-83.6% to -53.5%)#	-72.7% (-87.7% to -39.1%)#	-49.4% (-59.1% to -38.4%)#	-27.5% (-35.2% to -19.3%)#	-29.5% (-34.0% to -25.0%)#	-57.3% (-71.2% to -36.9%)#	-60.6% (-74.7% to -38.6%)#	-54.1% (-66.7% to -35.7%)#	-28.7% (-34.3% to -23.2%)#
	% change 2000-2017	-41.1% (-58.3% to -17.5%)#	-47.1% (-64.0% to -23.7%)#	-54.6% (-72.5% to -28.3%)#	-49.5% (-75.5% to -2.3%)#	-30.8% (-43.3% to -17.0%)#	-13.2% (-21.9% to -3.7%)#	-26.1% (-30.8% to -21.2%)#	-45.7% (-62.2% to -23.9%)#	-46.3% (-63.9% to -21.1%)#	-39.9% (-55.0% to -19.8%)#	-21.4% (-27.2% to -15.5%)#
Guyana	Death #, 1990	577 (459 to 710)	81 (65 to 98)	281 (209 to 362)	157 (131 to 186)	62 (58 to 67)	57 (55 to 60)	97 (95 to 99)	940 (810 to 1,084)	1,096 (958 to 1,245)	1,313 (1,171 to 1,469)	154 (151 to 158)
	Death #, 2000	359 (300 to 425)	45 (36 to 54)	178 (140 to 222)	84 (68 to 103)	52 (48 to 55)	44 (43 to 46)	80 (79 to 81)	582 (508 to 664)	666 (590 to 749)	842 (760 to 931)	125 (122 to 127)
	Death #, 2017	198 (143 to 269)	27 (20 to 37)	80 (58 to 109)	43 (32 to 57)	29 (25 to 33)	31 (28 to 34)	75 (68 to 83)	306 (222 to 415)	349 (254 to 472)	484 (381 to 615)	106 (97 to 116)
	% change 1990-2017	-65.7% (-76.6% to -50.8%)#	-66.3% (-77.2% to -52.2%)#	-71.4% (-80.9% to -56.4%)#	-72.6% (-80.7% to -61.6%)#	-53.3% (-59.8% to -46.3%)#	-45.8% (-51.0% to -40.3%)#	-22.5% (-30.4% to -14.7%)#	-67.5% (-77.3% to -55.4%)#	-68.2% (-77.8% to -56.6%)#	-63.1% (-72.1% to -52.8%)#	-31.1% (-37.6% to -24.6%)#
	% change 2000-2017	-45.0% (-62.0% to -21.1%)#	-38.4% (-57.8% to -9.3%)#	-54.8% (-70.2% to -33.8%)#	-48.9% (-63.5% to -28.5%)#	-44.1% (-51.8% to -35.5%)#	-30.2% (-36.5% to -23.0%)#	-6.1% (-15.3% to 3.5%)	-47.5% (-63.1% to -26.4%)#	-47.6% (-62.7% to -27.4%)#	-42.5% (-55.9% to -25.4%)#	-14.7% (-22.5% to -6.2%)#
Haiti	Death #, 1990	5868 (4399 to 7478)	4269 (3123 to 5595)	13242 (11669 to 14821)	12093 (9853 to 14584)	1597 (1499 to 1688)	906 (854 to 957)	1287 (1192 to 1372)	23379 (21034 to 25697)	35472 (32608 to 38397)	39262 (36205 to 42336)	2194 (2050 to 2325)
	Death #, 2000	5680 (4782 to 6673)	2913 (2393 to 3490)	10684 (9299 to 12147)	7988 (6679 to 9408)	1498 (1370 to 1631)	959 (883 to 1062)	1353 (1216 to 1499)	19277 (17405 to 21409)	27265 (24531 to 30335)	31076 (28129 to 34293)	2312 (2118 to 2535)
	Death #, 2017	5782 (4384 to 7394)	2247 (1709 to 2868)	7229 (4854 to 10380)	4125 (3281 to 5074)	1114 (978 to 1274)	843 (756 to 957)	1391 (1177 to 1670)	15258 (12125 to 18926)	19383 (15398 to 23946)	22730 (18731 to 27560)	2233 (1956 to 2616)
	% change 1990-2017	-1.5% (-32.1% to 42.3%)	-47.4% (-64.3% to -23.1%)#	-45.4% (-65.0% to -20.2%)#	-65.9% (-74.4% to -54.7%)#	-30.2% (-39.4% to -19.4%)#	-7.0% (-17.6% to 7.2%)	8.0% (-9.5% to 31.5%)	-34.7% (-49.4% to -17.4%)#	-45.4% (-57.1% to -31.2%)#	-42.1% (-53.0% to -28.8%)#	1.8% (-11.4% to 20.2%)
	% change 2000-2017	1.8% (-24.7% to 35.0%)	-22.9% (-42.4% to 3.4%)	-32.3% (-55.5% to -5.1%)#	-48.4% (-60.7% to -32.5%)#	-25.7% (-36.7% to -12.9%)#	-12.2% (-23.6% to 0.8%)	2.8% (-12.3% to 22.3%)	-20.9% (-38.0% to -0.3%)#	-28.9% (-44.1% to -11.2%)#	-26.9% (-41.0% to -10.4%)#	-3.4% (-15.5% to 12.2%)
	Death #, 1990	577 (463 to 707)	144 (114 to 182)	517 (422 to 634)	320 (263 to 385)	131 (117 to 146)	96 (90 to 102)	91 (87 to 95)	1,238 (1,023 to 1,472)	1,558 (1,298 to 1,830)	1,876 (1,598 to 2,173)	187 (178 to 196)

eTable 2. ENN, LNN, PNN, 1-4, 5-9, 10-14, 15-19, <1, <5, 10-19, and <20 years mortality in 1990, 2000 and 2017 with mean percentage change for all GBD causes.

	Metric	Early Neonatal	Late Neonatal	Post Neonatal	1 to 4	5 to 9	10 to 14	15 to 19	<1 year	Under 5	<20 years	10 to 19
Jamaica	Death #, 2000	552 (448 to 671)	130 (105 to 160)	221 (168 to 280)	156 (125 to 190)	115 (102 to 129)	78 (74 to 83)	128 (125 to 130)	903 (741 to 1,086)	1,059 (872 to 1,270)	1,380 (1,171 to 1,610)	206 (199 to 213)
	Death #, 2017	409 (286 to 574)	95 (67 to 133)	121 (69 to 197)	78 (55 to 109)	46 (40 to 54)	58 (52 to 65)	165 (142 to 189)	625 (436 to 878)	703 (492 to 989)	972 (744 to 1,268)	223 (196 to 252)
	% change 1990-2017	-29.0% (-53.9% to 5.9%)	**-34.2% (-55.9% to -1.2%)#**	**-76.7% (-87.5% to -58.6%)#**	**-75.6% (-83.2% to -65.1%)#**	**-64.8% (-70.8% to -57.7%)#**	**-39.1% (-46.1% to -31.5%)#**	**81.5% (56.0% to 108.9%)#**	**-49.5% (-65.9% to -25.3%)#**	**-54.9% (-69.2% to -33.4%)#**	**-48.2% (-61.4% to -28.8%)#**	**19.6% (4.0% to 36.2%)#**
	% change 2000-2017	-25.9% (-48.9% to 9.2%)	-27.0% (-50.0% to 7.4%)	**-45.4% (-68.2% to -6.7%)#**	**-50.0% (-65.1% to -28.7%)#**	**-59.8% (-66.2% to -51.6%)#**	**-25.4% (-33.7% to -16.5%)#**	**28.7% (10.6% to 47.0%)#**	-30.8% (-52.2% to 1.4%)	**-33.6% (-54.0% to -3.3%)#**	**-29.6% (-47.2% to -4.2%)#**	8.2% (-5.3% to 22.5%)
Puerto Rico	Death #, 1990	543 (506 to 578)	141 (123 to 160)	204 (177 to 231)	118 (97 to 144)	73 (70 to 76)	81 (79 to 82)	269 (266 to 273)	888 (836 to 941)	1,006 (953 to 1,062)	1,429 (1,370 to 1,490)	350 (345 to 355)
	Death #, 2000	298 (258 to 341)	124 (109 to 142)	144 (126 to 165)	68 (53 to 88)	53 (50 to 55)	58 (56 to 59)	267 (264 to 271)	567 (528 to 608)	635 (595 to 673)	1,013 (968 to 1,055)	325 (320 to 330)
	Death #, 2017	111 (92 to 132)	51 (43 to 60)	67 (58 to 77)	80 (76 to 84)	93 (91 to 95)	128 (126 to 129)	256 (247 to 264)	229 (193 to 269)	309 (270 to 353)	785 (741 to 837)	383 (374 to 393)
	% change 1990-2017	**-79.5% (-83.2% to -75.4%)#**	**-64.0% (-71.2% to -54.9%)#**	**-67.0% (-72.7% to -59.4%)#**	**-32.7% (-44.8% to -17.9%)#**	**27.8% (22.3% to 34.1%)#**	**58.5% (54.6% to 62.7%)#**	**-5.0% (-8.5% to -1.6%)#**	**-74.2% (-78.5% to -69.2%)#**	**-69.3% (-73.5% to -64.6%)#**	**-45.0% (-48.9% to -40.6%)#**	**9.6% (6.4% to 12.7%)#**
	% change 2000-2017	**-62.8% (-70.5% to -54.1%)#**	**-59.0% (-67.2% to -49.2%)#**	**-53.4% (-61.7% to -43.9%)#**	16.3% (-8.9% to 53.5%)	**77.1% (68.4% to 86.2%)#**	**121.9% (115.8% to 128.1%)#**	**-4.4% (-8.1% to -1.0%)#**	**-59.5% (-66.1% to -51.6%)#**	**-51.4% (-57.8% to -43.7%)#**	**-22.4% (-28.0% to -16.3%)#**	**17.9% (14.5% to 21.2%)#**
St Lucia	Death #, 1990	40 (34 to 47)	5 (5 to 6)	17 (14 to 21)	16 (13 to 20)	6 (5 to 6)	6 (6 to 7)	12 (12 to 12)	63 (53 to 73)	78 (66 to 92)	102 (89 to 118)	18 (18 to 19)
	Death #, 2000	26 (22 to 31)	6 (4 to 7)	11 (8 to 13)	8 (6 to 11)	8 (7 to 9)	6 (6 to 7)	12 (12 to 13)	42 (36 to 50)	51 (42 to 61)	78 (68 to 89)	19 (18 to 19)
	Death #, 2017	18 (13 to 26)	4 (3 to 5)	6 (4 to 8)	3 (2 to 4)	3 (2 to 3)	4 (4 to 4)	9 (9 to 10)	28 (19 to 39)	31 (21 to 44)	47 (36 to 61)	13 (12 to 14)
	% change 1990-2017	**-54.3% (-70.4% to -32.8%)#**	**-33.4% (-56.1% to -2.1%)#**	**-66.4% (-78.7% to -49.0%)#**	**-81.4% (-88.2% to -71.0%)#**	**-51.1% (-59.5% to -41.1%)#**	**-35.8% (-42.4% to -28.4%)#**	**-23.9% (-29.0% to -18.4%)#**	**-55.8% (-71.4% to -35.1%)#**	**-61.0% (-74.9% to -42.3%)#**	**-54.5% (-66.5% to -38.4%)#**	**-28.0% (-33.1% to -22.0%)#**
	% change 2000-2017	**-30.9% (-52.7% to -1.1%)#**	**-33.8% (-55.4% to -4.0%)#**	**-45.4% (-64.4% to -18.1%)#**	**-64.5% (-77.6% to -45.8%)#**	**-67.3% (-72.8% to -60.6%)#**	**-36.0% (-42.5% to -28.7%)#**	**-26.3% (-31.5% to -21.0%)#**	**-34.9% (-55.4% to -7.2%)#**	**-39.7% (-59.3% to -12.9%)#**	**-40.2% (-54.7% to -21.3%)#**	**-29.6% (-34.9% to -24.3%)#**
St Vincent	Death #, 1990	33 (28 to 39)	8 (6 to 9)	22 (19 to 26)	15 (12 to 18)	4 (4 to 5)	5 (5 to 6)	8 (8 to 9)	63 (54 to 74)	78 (66 to 90)	95 (82 to 109)	14 (13 to 14)
	Death #, 2000	24 (20 to 28)	7 (6 to 9)	13 (11 to 16)	8 (6 to 10)	6 (6 to 7)	6 (6 to 6)	10 (10 to 10)	44 (37 to 52)	52 (43 to 62)	74 (64 to 85)	16 (15 to 17)
	Death #, 2017	13 (9 to 19)	4 (3 to 6)	7 (5 to 10)	3 (2 to 4)	3 (2 to 3)	6 (5 to 6)	8 (8 to 9)	24 (17 to 34)	28 (20 to 38)	44 (35 to 56)	14 (13 to 15)
	% change 1990-2017	**-60.2% (-72.5% to -41.5%)#**	**-44.4% (-61.4% to -19.8%)#**	**-68.7% (-78.3% to -54.7%)#**	**-78.7% (-86.2% to -67.7%)#**	**-27.7% (-40.1% to -11.6%)#**	7.5% (-2.9% to 19.2%)	-2.7% (-8.7% to 3.5%)	**-61.3% (-73.0% to -43.7%)#**	**-64.5% (-75.7% to -48.7%)#**	**-53.6% (-64.2% to -38.9%)#**	1.2% (-5.9% to 8.8%)
	% change 2000-2017	**-44.4% (-61.0% to -21.5%)#**	**-37.9% (-57.7% to -9.5%)#**	**-47.7% (-63.3% to -24.3%)#**	**-59.8% (-72.7% to -41.4%)#**	**-53.6% (-60.9% to -44.9%)#**	-7.4% (-15.8% to 2.1%)	**-16.9% (-21.5% to -12.0%)#**	**-44.4% (-61.2% to -21.0%)#**	**-46.7% (-62.6% to -24.4%)#**	**-40.1% (-53.1% to -22.7%)#**	**-13.3% (-19.1% to -7.2%)#**
Suriname	Death #, 1990	217 (187 to 252)	59 (51 to 69)	174 (151 to 203)	66 (54 to 83)	34 (31 to 37)	25 (23 to 26)	49 (46 to 51)	450 (389 to 524)	517 (447 to 595)	624 (549 to 707)	73 (70 to 77)
	Death #, 2000	186 (161 to 214)	54 (46 to 62)	136 (118 to 156)	47 (41 to 55)	21 (19 to 24)	26 (25 to 28)	41 (39 to 43)	375 (326 to 431)	423 (366 to 484)	511 (451 to 578)	67 (64 to 71)
	Death #, 2017	134 (109 to 162)	38 (31 to 46)	77 (63 to 93)	29 (23 to 35)	17 (15 to 20)	24 (22 to 26)	49 (44 to 54)	250 (204 to 302)	278 (228 to 336)	369 (313 to 432)	73 (67 to 79)
	% change 1990-2017	**-38.1% (-51.9% to -22.2%)#**	**-35.6% (-49.8% to -19.2%)#**	**-55.6% (-65.4% to -44.6%)#**	**-57.0% (-67.0% to -42.6%)#**	**-48.5% (-56.7% to -39.4%)#**	-2.5% (-12.6% to 8.6%)	0.6% (-10.0% to 12.5%)	**-44.5% (-56.8% to -30.4%)#**	**-46.1% (-57.7% to -32.7%)#**	**-40.9% (-51.9% to -28.4%)#**	-0.4% (-10.3% to 10.6%)
	% change 2000-2017	**-27.9% (-42.8% to -10.2%)#**	**-28.5% (-43.3% to -11.2%)#**	**-43.0% (-54.8% to -29.4%)#**	**-39.8% (-52.3% to -24.7%)#**	**-19.1% (-30.9% to -4.7%)#**	-8.4% (-17.1% to 1.4%)	**19.6% (6.7% to 32.7%)#**	**-33.4% (-47.1% to -17.3%)#**	**-34.1% (-47.5% to -18.4%)#**	**-27.9% (-40.4% to -13.5%)#**	8.6% (-1.8% to 19.7%)
Trinidad Tobago	Death #, 1990	287 (248 to 328)	87 (75 to 99)	144 (124 to 163)	85 (74 to 98)	81 (73 to 90)	64 (61 to 68)	99 (97 to 102)	518 (447 to 590)	603 (523 to 687)	848 (754 to 944)	164 (158 to 169)
	Death #, 2000	231 (192 to 274)	70 (57 to 84)	136 (107 to 169)	74 (55 to 95)	40 (35 to 44)	49 (46 to 52)	105 (102 to 107)	437 (375 to 504)	510 (435 to 593)	704 (620 to 795)	154 (148 to 159)
	Death #, 2017	138 (97 to 193)	62 (43 to 87)	79 (56 to 110)	44 (31 to 60)	27 (23 to 31)	29 (26 to 32)	73 (60 to 86)	278 (196 to 390)	322 (227 to 452)	450 (348 to 591)	102 (86 to 118)
	% change 1990-2017	**-52.1% (-67.3% to -30.6%)#**	-29.1% (-51.7% to 2.6%)	**-45.2% (-62.5% to -21.0%)#**	**-48.5% (-65.0% to -25.7%)#**	**-67.2% (-72.6% to -59.9%)#**	**-55.2% (-60.8% to -48.8%)#**	**-26.9% (-39.5% to -12.9%)#**	**-46.3% (-63.4% to -22.3%)#**	**-46.6% (-63.5% to -22.8%)#**	**-46.9% (-60.1% to -28.8%)#**	**-38.0% (-47.2% to -27.7%)#**
	% change 2000-2017	**-40.3% (-59.2% to -13.7%)#**	-11.6% (-41.0% to 29.5%)	**-42.1% (-61.9% to -13.6%)#**	**-40.7% (-61.1% to -9.7%)#**	**-32.8% (-44.1% to -18.5%)#**	**-41.1% (-48.3% to -32.8%)#**	**-30.6% (-42.8% to -17.1%)#**	**-36.3% (-56.1% to -8.6%)#**	**-36.9% (-56.5% to -9.2%)#**	**-36.0% (-51.8% to -15.3%)#**	**-34.0% (-44.2% to -23.3%)#**
Virgin Isl US	Death #, 1990	23 (20 to 26)	5 (4 to 6)	8 (6 to 10)	5 (4 to 6)	4 (3 to 4)	4 (3 to 4)	9 (9 to 10)	36 (31 to 41)	41 (35 to 47)	57 (51 to 64)	13 (12 to 14)
	Death #, 2000	11 (9 to 14)	4 (3 to 5)	4 (3 to 6)	4 (3 to 5)	2 (2 to 3)	3 (2 to 3)	7 (6 to 8)	19 (15 to 24)	23 (18 to 28)	34 (29 to 42)	9 (8 to 11)
	Death #, 2017	6 (4 to 8)	2 (1 to 3)	2 (2 to 3)	2 (1 to 3)	2 (1 to 2)	2 (2 to 2)	4 (4 to 5)	10 (7 to 14)	12 (8 to 16)	20 (16 to 25)	6 (6 to 7)
	% change 1990-2017	**-74.5% (-82.8% to -63.6%)#**	**-63.0% (-75.1% to -45.4%)#**	**-72.8% (-81.6% to -59.7%)#**	**-59.0% (-71.9% to -37.6%)#**	**-56.1% (-64.1% to -47.4%)#**	**-51.0% (-56.3% to -45.0%)#**	**-53.2% (-57.2% to -48.0%)#**	**-72.5% (-81.2% to -61.4%)#**	**-70.9% (-79.9% to -58.9%)#**	**-65.8% (-73.4% to -56.2%)#**	**-52.6% (-56.6% to -47.5%)#**
	% change 2000-2017	**-47.6% (-65.1% to -21.5%)#**	**-45.8% (-63.6% to -18.1%)#**	**-50.3% (-66.8% to -24.6%)#**	**-46.9% (-63.0% to -23.2%)#**	**-31.7% (-44.6% to -17.0%)#**	**-29.7% (-39.4% to -19.2%)#**	**-33.6% (-43.8% to -22.1%)#**	**-47.9% (-64.8% to -21.4%)#**	**-47.7% (-64.3% to -22.1%)#**	**-42.5% (-56.3% to -23.4%)#**	**-32.5% (-42.3% to -21.7%)#**
**Central Latin America**	Death #, 1990	62,409 (59,531 to 65,161)	19,413 (18,511 to 20,267)	76,920 (73,892 to 80,039)	41,661 (39,261 to 44,204)	15,752 (15,456 to 16,047)	11,922 (11,801 to 12,044)	21,773 (21,660 to 21,896)	158,742 (154,678 to 162,836)	200,404 (195,692 to 204,980)	249,851 (244,736 to 254,871)	33,695 (33,476 to 33,919)
	% of global total, 1990	2.0% (1.9% to 2.2%)	1.5% (1.4% to 1.6%)	2.0% (1.9% to 2.1%)	1.1% (1.1% to 1.2%)	1.8% (1.8% to 1.9%)	2.5% (2.5% to 2.6%)	3.2% (3.2% to 3.3%)	1.9% (1.9% to 2.0%)	1.7% (1.7% to 1.8%)	1.8% (1.8% to 1.9%)	3.0% (2.9% to 3.0%)
	Death #, 2000	45,951 (44,014 to 48,075)	14,848 (14,198 to 15,549)	47,860 (45,942 to 49,922)	25,640 (24,563 to 26,833)	10,401 (10,192 to 10,630)	9,557 (9,441 to 9,670)	22,664 (22,506 to 22,830)	108,658 (105,377 to 111,963)	134,298 (130,209 to 138,509)	176,921 (172,550 to 181,582)	32,221 (31,963 to 32,473)
[truncated: 647,475 more chars]
